# Strain-Dependent Transcriptome Signatures for Robustness in *Lactococcus lactis*

**DOI:** 10.1371/journal.pone.0167944

**Published:** 2016-12-14

**Authors:** Annereinou R. Dijkstra, Wynand Alkema, Marjo J. C. Starrenburg, Jeroen Hugenholtz, Sacha A. F. T. van Hijum, Peter A. Bron

**Affiliations:** 1 Kluyver Centre for Genomics of Industrial Fermentation, GA Delft, The Netherlands; 2 NIZO food research, BA Ede, The Netherlands; 3 Universiteit van Amsterdam, Swammerdam Institute for Life Sciences, Science Park 904, XH Amsterdam, The Netherlands; 4 Centre for Molecular and Biomolecular Informatics, Radboudumc, HB Nijmegen, the Netherlands; 5 TI Food & Nutrition, Nieuwe Kanaal 9A, PA Wageningen, The Netherlands; National Renewable Energy Laboratory, UNITED STATES

## Abstract

Recently, we demonstrated that fermentation conditions have a strong impact on subsequent survival of *Lactococcus lactis* strain MG1363 during heat and oxidative stress, two important parameters during spray drying. Moreover, employment of a transcriptome-phenotype matching approach revealed groups of genes associated with robustness towards heat and/or oxidative stress. To investigate if other strains have similar or distinct transcriptome signatures for robustness, we applied an identical transcriptome-robustness phenotype matching approach on the *L*. *lactis* strains IL1403, KF147 and SK11, which have previously been demonstrated to display highly diverse robustness phenotypes. These strains were subjected to an identical fermentation regime as was performed earlier for strain MG1363 and consisted of twelve conditions, varying in the level of salt and/or oxygen, as well as fermentation temperature and pH. In the exponential phase of growth, cells were harvested for transcriptome analysis and assessment of heat and oxidative stress survival phenotypes. The variation in fermentation conditions resulted in differences in heat and oxidative stress survival of up to five 10-log units. Effects of the fermentation conditions on stress survival of the *L*. *lactis* strains were typically strain-dependent, although the fermentation conditions had mainly similar effects on the growth characteristics of the different strains. By association of the transcriptomes and robustness phenotypes highly strain-specific transcriptome signatures for robustness towards heat and oxidative stress were identified, indicating that multiple mechanisms exist to increase robustness and, as a consequence, robustness of each strain requires individual optimization. However, a relatively small overlap in the transcriptome responses of the strains was also identified and this generic transcriptome signature included genes previously associated with stress (*ctsR* and *lplL*) and novel genes, including *nanE* and genes encoding transport proteins. The transcript levels of these genes can function as indicators of robustness and could aid in selection of fermentation parameters, potentially resulting in more optimal robustness during spray drying.

## Introduction

Owing to their spoilage-preventing, texture-improving and flavor-enhancing properties, lactic acid bacteria have a long history of application in food fermentations [1, 2]. One of the most widely used lactic acid bacteria in the food industry is *Lactococcus lactis*, notably for the production of cheese and butter(milk) [2]. These milk fermentation processes are typically initiated with the addition of starter cultures containing high concentrations of one or multiple *L*. *lactis* strains. During the production of these starter cultures prior to application in the food industry, *L*. *lactis* strains encounter severe stresses, for example heat and oxidative stress during spray drying [3–5]. Although spray drying is a cost-effective and energy-efficient method for the preservation of starter cultures, it generally results in a relatively large decrease in viability as compared with other preservation methods such as freezing and freeze drying [6]. Viability of starter cultures is essential for an adequate contribution to the fermentation end-product, justifying the industrial interest to better understand and improve robustness [1].

Genes involved in stress responses appear highly conserved among bacteria, nevertheless regulation of these stress genes can differ between organisms [7, 8]. Recently, we demonstrated a large diversity in heat and oxidative stress survival among *L*. *lactis* strains, suggesting differential regulation of stress responses [5]. Furthermore, strains with an *L*. *lactis* subsp. *cremoris* phenotype appeared to have a less efficient response as compared with strains with an *L*. *lactis* subsp. *lactis* phenotype when these strains were pre-adapted to a minor dose of acid, bile or freezing stress, prior to exposure to a lethal dose of the same stress [9].

Previously, we demonstrated that for the *L*. *lactis* subsp. *cremoris* strain MG1363 [10], oxygen level and fermentation temperature strongly affect subsequent survival during heat and oxidative stress assays, respectively [11]. Furthermore, by applying a transcriptome-phenotype matching approach, we revealed transcriptome signatures associated with robustness towards heat and oxidative stress, which could function as indicators for robustness. These transcriptome signatures included the *metC*-*cysK* operon, of which the transcript levels positively correlated with robustness. The role of this operon was confirmed by demonstrating an increase in robustness towards oxidative stress of MG1363 after growth in medium lacking cysteine, which has been demonstrated to induce the *metC-cysK* operon [11, 12].

*L*. *lactis* strains that are applied in food industry are diverse in subspecies and isolation source. It remains unclear if the correlation of gene expression levels and robustness as found in strain MG1363 are generic and, therefore, can also be employed for other *L*. *lactis* strains to predict their robustness. Specific individual gene transcripts that associated with robustness in MG1363 [11] were previously established to be important during heat, acid and osmotic stress in *L*. *lactis* subsp. *lactis* strain IL1403 [13], suggesting at least partially overlapping stress responses in these two *L*. *lactis* strains.

To investigate if other strains have transcriptome signatures for robustness towards heat and oxidative stress similar to or distinct from those of strain MG1363, we applied an identical transcriptome-phenotype matching strategy [11] on three other strains. These three strains were the dairy *L*. *lactis* subsp. *lactis* strain IL1403 [14], the non-dairy *L*. *lactis* subsp. *lactis* strain KF147 [15] and the dairy *L*. *lactis* subsp. *cremoris* strain SK11 [16]. Besides the differences in subspecies and origin, we previously revealed highly diverse robustness phenotypes of these strains[5]. The strains were individually grown under the twelve conditions that were previously applied to MG1363 [11] and the effect of these conditions on heat and oxidative stress survival was assessed. Moreover, we determined full genome transcriptome profiles, allowing association of gene expression and stress survival to identify transcriptome signatures for robustness towards heat and oxidative stress in the individual strains.

## Materials and Methods

### Strains and fermentations

*L*. *lactis* strains IL1403 [14], KF147 [15] and SK11 [16] were cultivated in chemically defined medium (CDM) as described previously [11]. Briefly, the strains were fermented under twelve different conditions varying in sodium chloride concentration (0 or 100 mM), initial pH (6.0 or 6.5), temperature (27, 30 or 35°C) and level of oxygen (static in 50 ml Falcon tube or shaken at 100 rpm in 500 ml shake flask with a cotton plug) (Table 1). Fermentations were performed on two separate days (fermentation number 1–6 on day 1, 7–12 on day 2) and, therefore, a replicate of fermentation 6 was added on day 2 (fermentation 13). Biomass formation was determined by measurement of the optical density (OD) at 600 nm. In the exponential phase of growth (OD_600_ between 0.5 and 0.7), cells were harvested for heat and oxidative stress survival assays and RNA isolation.

**Table 1:** Fermentation conditions, growth characteristics and stress survival.

					μ (h^-1^)			OD_final_			heat stress survival(%)			oxidative stress survival (%)		
fermentation number	salt (mM)	initial pH	temperature (°C)	level of oxygen	IL1403	KF147	SK11	IL1403	KF147	SK11	IL1403	KF147	SK11	IL1403	KF147	SK11
1	0	6.0	27	high	0.37	0.87	0.52	1.59	2.12	1.34	0.23	0.38	1.2	6.5	0.045	0.051
2	100	6.5	27	high	0.43	0.77	0.57	2.43	2.56	1.81	0.75	1.1	0.36	12	0.10	0.55
3	0	6.5	27	low	0.59	0.79	0.67	2.42	2.37	2.43	0.000092	0.012	0.63	0.0011	0.10	0.13
4	100	6.0	27	low	0.30	0.87	0.49	1.48	1.50	1.30	4.5	0.021	0.35	0.75	0.12	0.11
5	0	6.0	30	low	0.59	1.16	0.66	1.56	1.83	1.46	0.000044	0.0077	2.5	0.00046	0.27	0.074
6	100	6.5	30	low	0.73	1.09	0.69	2.27	2.23	2.08	0.0075	0.12	4.0	0.032	0.042	0.047
7	0	6.5	30	high	0.74	0.94	0.63	2.62	2.73	1.98	0.0052	1.7	6.8	0.0065	0.071	0.037
8	100	6.0	30	high	0.39	0.85	0.57	1.53	1.80	1.50	3.2	18	9.4	53	0.46	0.038
9	0	6.0	35	high	0.80	1.12	0.26	2.18	1.99	1.16	5.3	25	13	0.0029	1.7	58
10	100	6.5	35	high	0.77	1.09	0.25	2.42	2.75	1.32	2.7	53	6.4	1.2	0.18	20
11	0	6.5	35	low	1.00	1.22	0.61	2.90	2.58	1.89	0.095	0.79	34	0.040	0.012	0.0084
12	100	6.0	35	low	0.92	1.18	0.50	1.77	1.59	1.26	0.45	0.93	7.4	0.0042	0.00070	0.010
13	100	6.5	30	low	0.76	1.06	0.74	2.34	2.21	2.04	0.011	0.033	3.1	0.0066	0.0023	0.036

Fermentation parameters of the various fermentations and resulting maximum growth rates (μ) and optical densities at the end of fermentation (OD_final_) and survival after 60 minutes (IL1403) or 10 minutes (KF147 and SK11) of heat stress and after 30 minutes of oxidative stress of strains IL1403, KF147 and SK11. Survival at the other time point of the stress assays can be found in S1 Table. Survival data represent averages of technical duplicates. Shaken and static fermentations are indicated as a relatively high level of oxygen (“high”) and a relatively low level of oxygen (“low”), respectively.

### Heat and oxidative stress survival assays

Stress survival was determined as described previously [11]. Cells were harvested from 5 ml of culture by centrifugation at 1865 × g for 10 minutes and resuspended in 2.5 ml sterile 50 mM sodium phosphate (Merck) buffer pH 7.2. For assessment of heat stress survival, 0.5 ml of the cell suspensions was diluted by adding 0.5 ml of phosphate buffer and were incubated in duplicate in a volume of 0.1 ml at 50°C for 10 and 30 minutes (KF147, SK11) or 30 and 60 minutes (IL1403) in 0.2 ml PCR tubes (Bioplastics BV, Landgraaf, The Netherlands) in a Gene-Amp PCR system 9600 (Applied BioSystems, Foster City, California, USA). For assessment of oxidative stress survival, hydrogen peroxide (Merck) in phosphate buffer was added to 0.25 ml of the cell suspensions in duplicate to a final concentration of 5 mM and an end volume of 0.5 ml, followed by incubation for 30 and 60 minutes at 30°C in a water bath. After incubation, samples were centrifuged at 15,000 × g for 3 minutes and cell pellets were resuspended in 0.5 ml of phosphate buffer. Survival was assessed by spotting serial dilutions in triplicate on M17 agar plates supplemented with 0.5% glucose[17]. Colony forming units (CFU) were determined after incubation of the plates for 72 hours at 30°C.

### RNA isolation and DNA microarrays

RNA isolation, subsequent cDNA synthesis and labeling, as well as DNA microarray hybridizations were performed using routine procedures, as described previously for MG1363 [11]. Briefly, aliquots of 5 ml of culture were centrifuged at 4000 × *g* for 3 minutes at 2°C and cells were resuspended in 0.5 ml cold TE buffer. To this suspension, 500 μl 1:1 phenol/chloroform, 30 μl 10% SDS, 30 μl 3M sodium acetate pH 5.2 and 500 mg 0.1 mm zirconia beads (Biospec Products, Inc., Bartlesville, USA) was added in a 2 ml screw-cap tube and samples were frozen in liquid nitrogen and stored at -80°C. The DNA microarray hybridization scheme contained two connected loops, both containing samples derived on a single day (S1 Fig). A two-dye microarray-based gene expression analysis was performed on a custom-made 60-mer oligonucleotide array (Agilent Technologies, Santa Clara, California, USA, submitted in Gene Expression Omnibus under GEO Series accession number GSE72045) to determine genome-wide gene transcription levels. Co-hybridization of Cy5- and Cy3-labeled cDNA probes was performed on these oligonucleotide arrays at 65°C and 10 rpm for 17 h using GEX HI-RPM buffer (Agilent Technologies). After hybridization, slides were washed and scanned.

### Data analysis

Data analysis was performed as previously described for strain MG1363 [11]. The raw expression data were Lowess normalized and scaled to normalized probe expression levels using MicroPreP [18]. Multiple probes were designed for each ORF and the ORF expression level was calculated from the median of its probe signals. Normalized gene expression levels were further analyzed using the R BioConductor packages Biobase and limma (www.bioconductor.org). After 2-log transformation, gene expression levels were plotted against robustness levels and significance of the correlation was assessed by a linear model. We selected the genes with a significant correlation (*P* < 0.05) at both time points of the stress assay and further analyzed the genes with the most significant correlation by calculating the product of both *P*-values. To identify a generic transcriptome signature, we used survival at the time point at which the dynamic range of robustness was the largest. As a consequence, the selected time points for heat stress were 60, 10 and 10 minutes for IL1403, KF147 and SK11, respectively, whereas for oxidative stress the selected time point was 30 minutes for all strains. These data were compared with the survival of MG1363 after 30 minutes of heat stress or oxidative stress [11]. Correlation of survival and growth rate or optical density was determined by calculating the Pearson correlation coefficient. Differences in the effect of individual fermentation parameters on growth characteristics and robustness were assessed with a *t*-test in R (version 3.0.1, www.R-project.org) and differences were considered significant if the *P*-value was smaller than 0.05.

## Results and Discussion

### Variations in fermentation conditions impose largely similar effects on the growth characteristics of different *L*. *lactis* strains

To compare the effect of fermentation conditions on the growth characteristics, *L*. *lactis* strains IL1403, KF147 and SK11 were grown under the twelve different conditions that were previously applied to strain MG1363 [11]. These conditions varied in the level of salt and/or oxygen, as well as fermentation pH and temperature and resulted in variation of growth characteristics (Table 1, S2 Fig). Strain KF147 displayed maximum growth rates (μ_max_) in the same range (0.7 h^-1^ to 1.2 h^-1^) as we previously established for strain MG1363 [11], whereas SK11 had lower growth rates (0.5 h^-1^ to 0.7 h^-1^ [Table 1]). Strain IL1403 displayed the largest variation in maximum growth rate, ranging from 0.3 h^-1^ to 1.0 h^-1^ (Table 1).

**Fig 1:**
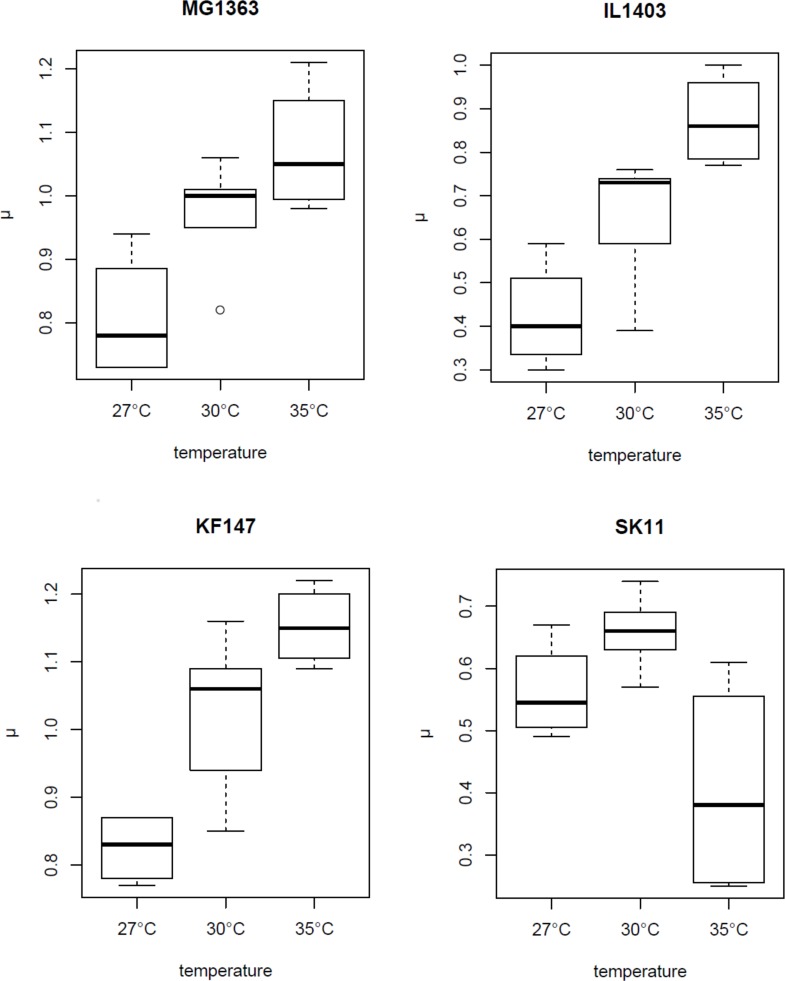
Effect of temperature on growth rate. Boxplots of maximum growth rate (μ_max_ in h^-1^) of strains MG1363, IL1403, KF147 and SK11 in fermentations at 27, 30 and 35°C.

The effect of fermentation temperature on maximum growth rate of the strains KF147 and IL1403 was similar to what we previously observed for MG1363 [11] (Fig 1). In contrast to the other strains, the maximum growth rate of SK11 was significant lower in fermentations at 35°C as compared with 30°C (Fig 1), which is in line with the fact that SK11 has an *L*. *lactis* subsp. *cremoris* phenotype in contrast to MG1363, IL1403 and KF147, which have an *L*. *lactis* subsp. *lactis* phenotype [19]. One of the characteristics that discriminates these phenotypes is that strains with an *L*. *lactis* subsp. *cremoris* phenotype are incapable of growing at high temperature in contrast to strains with an *L*. *lactis* subsp. *lactis* phenotype [20].

**Fig 2:**
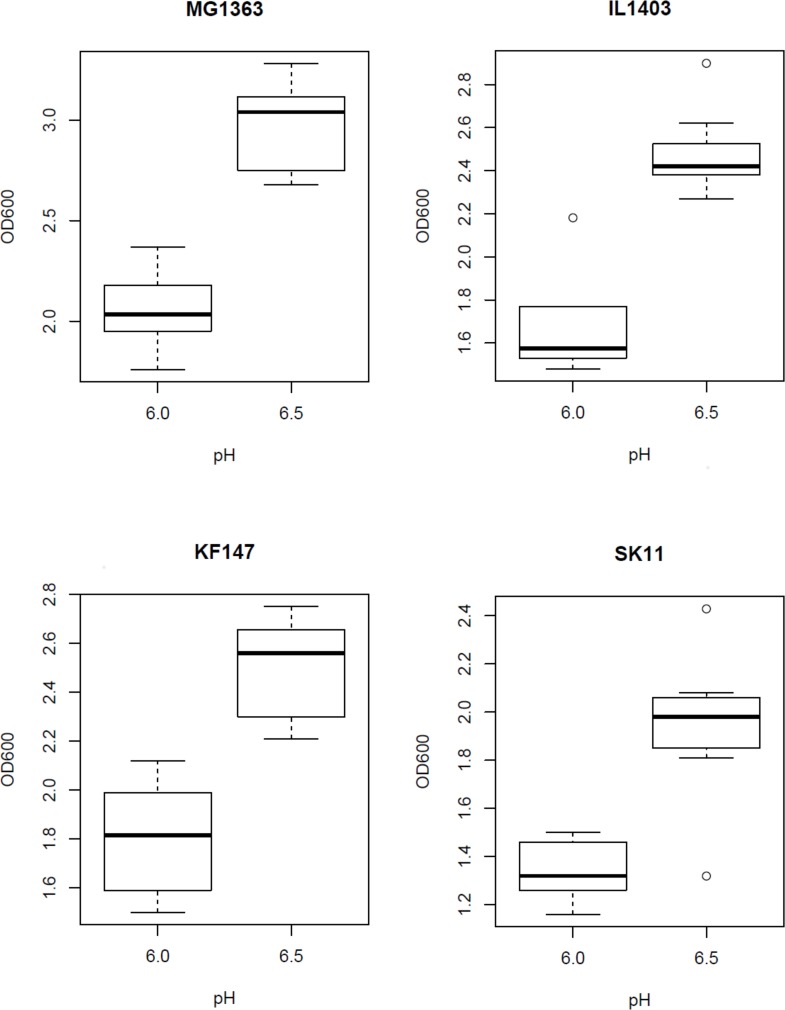
Effect of pH on final OD. Boxplots of final optical density (OD_final_) of strains MG1363, IL1403, KF147 and SK11 in fermentations with an initial pH of 6.0 or 6.5.

Both biomass formation (OD_final_) and final pH at the end of fermentation were strongly affected by the fermentation conditions and the observed effects were similar for all strains (Table 1). The initial pH of fermentation had the most significant effect on biomass formation. In fermentations with an initial pH of 6.5 a significantly higher biomass formation was reached for all strains as compared to fermentations with an initial pH of 6.0 (Fig 2). The final pH at the end of fermentation was mostly affected by the oxygen level and was significantly lower in fermentations with a relatively low level of oxygen as compared with fermentations with a relatively high level (data not shown). This is in line with an earlier study, which demonstrated that the acidifying ability of *L*. *lactis* strain CNRZ 483 decreased as initial oxygen concentration increased [21].

With the notable exception of the effect of fermentation temperature on growth rate of SK11, all other applied fermentation parameters had similar effects on the growth characteristics of the *L*. *lactis* strains, revealing an overlap in responses towards the applied fermentation conditions.

### The effect of fermentation conditions on robustness is strain-dependent

To study the effect of the fermentation conditions on robustness phenotypes, cells were harvested in exponential phase of growth for assessment of heat and oxidative stress survival phenotypes, representing robustness during spray drying [5]. During the stress assays, survival was determined at two time points, similar as for MG1363 [11]. For KF147 and SK11 the time points for heat stress survival measurement were adjusted because these strains displayed a higher sensitivity towards heat stress as compared with MG1363 and IL1403 (see Materials and Methods). Variation in fermentation conditions resulted in differences in both heat and oxidative stress survival of up to five log units (Table 1, S1 Table). Moreover, the various fermentation conditions had a different impact on the stress survival of the various strains. Strain IL1403 displayed the largest variation in robustness towards both heat and oxidative stress, which is in line with our observation that differences in fermentation conditions imposed the largest variation on growth characteristics of this strain as well. The observed differences in robustness towards both heat and oxidative stress of strain SK11 in the various fermentations demonstrate that contrary to earlier observations by Kim *et al*. [9] also strains with an *L*. *lactis* subsp. *cremoris* phenotype can have an adaptive response to stress.

As was observed before for strain MG1363 [11], no correlation of growth rate and survival towards heat stress was observed for the three strains. Only strain SK11 displayed a correlation of growth rate and oxidative stress survival (Pearson correlation coefficient = 0.79). Overall, this appears to support the study of Dressaire *et al*., which demonstrated that downregulation of stress genes at increasing growth rates, as observed in yeast [22], does not occur in *L*. *lactis* [23]. This implies that fermentation conditions resulting in improved robustness are not necessarily more time-consuming. Moreover, neither for heat stress nor oxidative stress, correlation of final biomass formation and survival was found, indicating that increased robustness can be achieved without the necessity to reduce yield.

**Fig 3:**
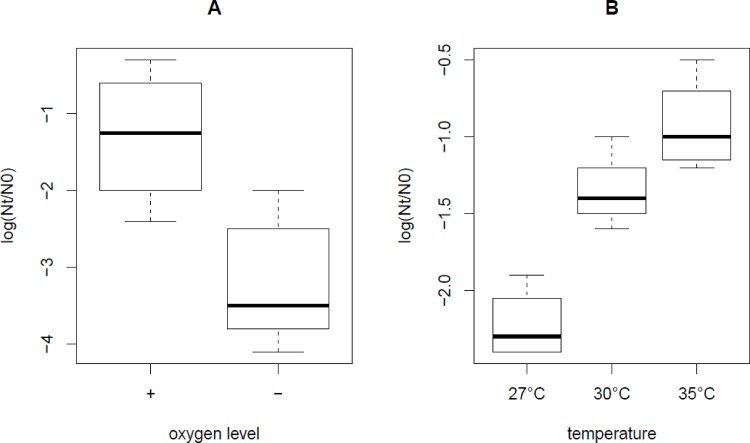
Heat stress survival of KF147 and SK11. Boxplots of robustness phenotypes towards 10 minutes of heat stress at relatively low and high oxygen levels for strain KF147 (A) and at various fermentation temperatures for strain SK11 (B). Robustness is expressed as the difference of log CFU/ml after stress (N_t_) and before stress (N_0_).

To identify the individual fermentation parameters with the most pronounced effect on heat or oxidative stress survival, we compared survival phenotypes in fermentations with one variant of this parameter with survival phenotypes in fermentations with the other variant of this parameter. Similar to what was previously observed for MG1363 [11], survival of KF147 during heat stress significantly increased during fermentation with a high level of oxygen (Fig 3A), whereas for SK11 robustness towards heat stress significantly increased with increasing fermentation temperature (Fig 3B). Contrasting our earlier observations in MG1363 [11], oxidative stress survival of strains IL1403, KF147 and SK11 was not significantly higher in fermentation at 35°C as compared with fermentations at 27°C. Survival of IL1403, which displayed a large variation in robustness phenotypes in the various fermentations, was not significantly altered by any of the specific individual fermentation parameters (S2 Table).

These experiments demonstrate that fermentation parameters have a substantial impact on subsequent stress survival of *L*. *lactis* strains. Irrespective of the strain’s general robustness level [5], survival can be dramatically altered by varying fermentation conditions. Although the fermentation parameters had similar effects on growth characteristics, the effect of specific fermentation parameters on survival is strain-dependent. This indicates that a general fermentation strategy to optimize robustness is difficult to achieve and to accomplish optimal robustness, fermentation conditions should be individually optimized for each *L*. *lactis* strain.

### Transcriptome-phenotype matching reveals strain-specific associations of gene expression with robustness

We determined the effect of the fermentation parameters on gene expression. As previously demonstrated for strain MG1363 [11], the oxygen level and the fermentation temperature also had the most pronounced effect on gene expression in IL1403, KF147 and SK11 (S3 Fig), which appears to be in line with the observed effect of oxygen level and fermentation temperature on robustness phenotypes of several strains.

Subsequently, we calculated the correlation (according to a linear model) of gene expression levels in the various fermentations with the corresponding robustness phenotypes (S1–S6 Files). Similarly as for MG1363 [11], we selected the genes displaying a significant correlation (*P* < 0.05) with robustness at both time points of the stress assay. The genes with the most significant correlation at both time points of the stress assay (product of *P*-values < 5×10^−5^) were further analyzed (Table 2). For IL1403, 54 and 32 genes met these criteria for heat and oxidative stress survival, respectively. Only two genes displayed a significant correlation with oxidative stress survival in KF147, whereas 174 genes correlated with heat stress survival in this strain. In SK11, 124 and 63 genes displayed a significant correlation with heat and oxidative stress survival, respectively.

**Table 2:** Individual correlating gene expressions with robustness towards heat stress (A) or oxidative stress (B).

A					
Strain	Locus tag	Gene	Function	Correlation	Slope
IL1403	*L133770*	*rpmH*	50S_ribosomal_protein_L34	negative	3.1
	*L127611*	*yveD*	hypothetical protein	negative	0.6
	*L36850*	*ps104*	prophage_ps1_protein_04	negative	0.1
	*L52686*	*ycfD*	hypothetical_protein	negative	1.1
	***L52019***	***gntK***	**gluconate_kinase**	positive	0.4
	*L18206*	*ysdB*	ABC transporter ATP binding protein	negative	1.8
	*L167426*	*zitS*	zinc ABC transporter substrate binding protein	negative	1.9
	*L94973*	*ycjG*	hypothetical protein	negative	3.4
	*L14408*	*nagB*	glucosamine-6-P isomerase	negative	3.9
	*L72115*	*yohD*	hypothetical protein	negative	2.9
	*L154225*	*ylfD*	hypothetical protein	negative	2.4
	*L0163*	*ribG*	riboflavin-specific deaminase	positive	0.1
	*L145739*	*floL*	flotillin-like protein	negative	5.2
	*L39365*	*yqdA*	hypothetical protein	negative	2.8
	*L11493*	*arsC*	arsenate reductase	negative	1.7
	*L175712*	*ynhD*	hypothetical protein	negative	3.2
	*L196779*	*yfjD*	tRNA/rRNA methyltransferase	negative	1.5
	*L113377*	*ps221*	prophage ps2 protein 21	negative	0.4
	*L77017*	*ykhJ*	hypothetical protein	negative	0.3
	*L0397*	*rpsT*	30S ribosomal protein S20	negative	17.7
	*L0275*	*dnaN*	DNA polymerase III subunit beta	positive	6.1
	*L0063*	*aroF*	phospho-2-dehydro-3-deoxyheptonate aldolase	negative	5.2
	*L193734*	*pdc*	phenolic acid decarboxylase	negative	0.3
	*L156445*	*ylfH*	N-acetylglucosamine catabolic protein	positive	1.8
	*L126998*	*yveC*	hypothetical protein	negative	2.4
	*L158972*	*yjfJ*	hypothetical protein	negative	5.7
	*L189881*	*rluC*	pseudouridine synthase	negative	1.5
	*L109379*	*yjaJ*	transcription regulator	negative	4.8
	*L198904*	*ps304*	prophage ps3 protein 04	negative	0.3
	*L16848*	*ysdA*	ABC transporter permease protein	negative	2.7
	*L193031*	*yhjA*	hypothetical protein	negative	11.4
	*L0064*	*aroH*	phospho-2-dehydro-3-deoxyheptonate aldolase	negative	17.4
	*L30663*	*ycdA*	hypothetical protein	negative	0.8
	*L102317*	*hslA*	HU like DNA-binding protein	negative	11.1
	*L0285*	*dnaD*	hypothetical protein	positive	2.7
	*L0151*	*rgrB*	GntR family transcription regulator	negative	4.1
	*L188392*	*ybiH*	hypothetical protein	positive	0.2
	*L192589*	*pydA*	dihydroorotate dehydrogenase 1A	negative	4.8
	*L19745*	*bar*	acyltransferase	negative	2.4
	*L117821*	*yxdC*	cation-transporting ATPase	negative	0.5
	*L67463*	*yuiB*	hypothetical protein	negative	7.9
	*L199277*	*ps305*	prophage ps3 protein 05	negative	0.7
	*L71486*	*yohC*	transcription regulator	negative	2.0
	*L140714*	*adk*	adenylate kinase	negative	2.7
	*L43222*	*recX*	recombination regulator RecX	negative	5.3
	*L72684*	*ykhE*	hypothetical protein	negative	0.3
	*L00096*	*rpmF*	50S ribosomal protein L32	negative	13.5
	*L155044*	*dcdA*	dCMP deaminase	negative	1.5
	*L122849*	*ybcG*	hypothetical protein	negative	7.5
	*L3272*	*yiaD*	putative NADH-flavin reductase	negative	3.9
	*L0416*	*rplT*	50S ribosomal protein L20	negative	10.5
	*L0217*	*rlrD*	LysR family transcription regulator	negative	1.7
	*L148007*	*ybeM*	hypothetical protein	negative	0.9
	*L162840*	*yhgC*	transcription regulator	negative	0.1
KF147	*LLKF_1804*	*trxB*	thioredoxin reductase	positive	12.6
	***LLKF_1758***	***rarA***	**ArsR family transcriptional regulator**	positive	0.7
	*LLKF_0447*	*yeaA*	beta-lactamase superfamily Zn-dependent hydrolase	positive	6.0
	*LLKF_2085*	*ytgB*	hypothetical protein	positive	17.7
	*LLKF_1563*	*bglH*	beta-glucosidase/ 6-phospho-beta-glucosidase	positive	0.4
	*LLKF_1820*	*yrbB*	transglycosylase	positive	26.3
	*LLKF_2083*		hypothetical protein	positive	15.2
	*LLKF_2084*	*ytgA*	hypothetical protein	positive	14.1
	*LLKF_1723*		excisionase	positive	0.1
	*LLKF_2082*	*ytgH*	Gls24 family general stress protein	positive	16.5
	*LLKF_0716*	*glgD*	glucose-1-phosphate adenylyltransferase regulatory subunit	negative	2.9
	*LLKF_0747*	*menC*	O-succinylbenzoate synthase	positive	5.1
	*LLKF_0746*	*yhdA*	1,4-dihydroxy-2-naphthoyl-CoA thioesterase	positive	2.2
	*LLKF_0965*	*yjgC*	amino acid ABC transporter substrate-binding protein	positive	7.0
	*LLKF_0036*	*pdhC*	pyruvate dehydrogenase complex dihydrolipoamide acetyltransferase	positive	23.9
	*LLKF_1210*		hypothetical protein	positive	1.5
	*LLKF_0039*	*lplL*	lipoate-protein ligase	positive	17.5
	*LLKF_1293*		AMP-dependent synthetase and ligase family protein	negative	0.6
	*LLKF_0381*	*ydcG*	Cro/CI family transcriptional regulator	positive	5.6
	*LLKF_1201*	*nanE*	N-acetylmannosamine-6-phosphate 2-epimerase	positive	0.8
	*LLKF_0715*	*glgC*	glucose-1-phosphate adenylyltransferase catalytic subunit	negative	1.6
	***LLKF_0967***	***yjgE***	**amino acid transport, ATP-binding protein**	positive	4.9
	*LLKF_1852*	*yrfB*	NADH-dependent oxidoreductase	positive	5.8
	*LLKF_0684*		CHW repeat-/cell adhesion domain-containing transglutaminase-like protease	negative	21.7
	*LLKF_1259*	*ymdE*	hypothetical protein	positive	16.9
	*LLKF_0384*	*fhuG*	ferrichrome ABC transporter permease FhuG	positive	2.9
	*LLKF_1275*	*trmFO*	tRNA (uracil-5-)-methyltransferase Gid	positive	11.6
	*LLKF_0110*	*pmrB*	MF superfamily multidrug resistance efflux pump protein	positive	0.9
	*LLKF_1417*	*yngB*	fibronectin-binding protein A	positive	1.9
	*LLKF_1270*	*ilvA*	threonine dehydratase	negative	2.9
	*LLKF_1118*	*ykjI*	hypothetical protein	positive	0.7
	*LLKF_1265*	*ymeB*	ABC transporter ATP-binding protein	negative	0.3
	*LLKF_0493*	*pyrG*	CTP synthase	positive	7.4
	*LLKF_0849*	*trmU*	tRNA (5-methylaminomethyl-2-thiouridylate)-methyltransferase	positive	10.2
	*LLKF_0664*	*scrK*	fructokinase	positive	0.7
	*LLKF_0555*	*yfhA*	GNAT family acetyltransferase	positive	0.5
	*LLKF_1344*	*xerD*	site-specific tyrosine recombinase XerD	positive	1.5
	*LLKF_2234*		hypothetical protein	negative	1.5
	*LLKF_2318*		family 2 glycosyltransferase	negative	0.3
	*LLKF_0959*	*yjfG*	hypothetical protein	positive	3.5
	*LLKF_0901*	*hslB*	DNA-binding protein HU	positive	1.1
	*LLKF_0500*	*dnaE*	DNA polymerase III subunit alpha	positive	2.0
	*LLKF_2242*		hypothetical protein	negative	1.9
	*LLKF_1294*		acyl carrier protein	negative	0.3
	*LLKF_1209*		hypothetical protein	positive	0.8
	***LLKF_0382***	***fhuC***	**ferrichrome ABC transporter ATP-binding protein FhuC**	positive	5.8
	*LLKF_0518*	*cysK*	cysteine synthase	positive	1.0
	*LLKF_2139*	*yudI*	tRNA-dihydrouridine synthase	positive	8.4
	*LLKF_1001*	*ftsE*	cell division ATP-binding protein FtsE	positive	10.8
	*LLKF_2231*	*ardA*	conjugative transposon antirestriction protein	negative	0.5
	*LLKF_1299*	*nisK*	nisin biosynthesis two-component system, sensor histidine kinase NisK	positive	0.9
	*LLKF_0094*		ABC transporter ATPase protein	negative	8.1
	***LLKF_0853***	***uvrC***	**excinuclease ABC subunit C**	positive	4.1
	*LLKF_0037*	*pdhB*	pyruvate dehydrogenase E1 component subunit beta	positive	16.0
	*LLKF_1962*	*nifU*	SUF system FeS assembly protein	positive	11.1
	*LLKF_0964*	*yjgB*	gamma-D-glutamyl-meso-diaminopimelate peptidase I, NlpC/P60 family	positive	6.7
	*LLKF_2244*		FtsK/SpoIIIE family DNA segregation ATPase	negative	2.2
	*LLKF_0038*	*pdhA*	pyruvate dehydrogenase E1 component subunit alpha	positive	13.4
	*LLKF_1851*	*yrfA*	ArsR family transcriptional regulator	positive	3.1
	*LLKF_0577*	*yfiL*	GNAT family acetyltransferase	positive	2.0
	*LLKF_1710*	*uxaC*	uronate isomerase	negative	0.1
	*LLKF_0098*		hypothetical protein	negative	2.1
	*LLKF_0441*	*trxH*	thioredoxin	positive	3.0
	*LLKF_0047*	*yahA*	HAD superfamily hydrolase	positive	5.6
	*LLKF_1812*	*yraD*	hypothetical protein	positive	1.1
	*LLKF_1857*		ABC transporter ATP-binding protein	negative	0.3
	*LLKF_0540*	*uvrB*	excinuclease ABC subunit B	positive	2.4
	*LLKF_1295*		hypothetical protein	negative	0.5
	*LLKF_1966*	*sufC*	SUF system FeS cluster assembly protein ATP-dependent transporter SufC	positive	11.5
	***LLKF_0052***	***cysD***	**O-acetyl-L-homoserine sulfhydrolase/O-acetyl-L-serine sulfhydrolase**	positive	1.8
	*LLKF_0904*	*yjaF*	hypothetical protein	positive	5.6
	*LLKF_0162*	*ybhA*	5-formyltetrahydrofolate cyclo-ligase	negative	0.7
	*LLKF_0035*	*pdhD*	pyruvate dehydrogenase complex dihydrolipoamide acetyltransferase	positive	21.6
	*LLKF_1720*		hypothetical protein	negative	0.1
	*LLKF_1579*	*ypaE*	hypothetical protein	negative	4.6
	*LLKF_2241*		hypothetical protein	negative	1.9
	*LLKF_2238*		hypothetical protein	negative	1.7
	*LLKF_1856*		transcriptional regulator	negative	0.7
	*LLKF_2243*		replication initiation factor	negative	1.5
	*LLKF_2233*		CHAP domain family N-acetylmuramoyl-L-alanine amidase	negative	0.5
	*LLKF_2236*		hypothetical protein	negative	1.1
	*LLKF_2246*		hypothetical protein	negative	2.7
	*LLKF_1948*	*ysdC*	hypothetical protein	negative	0.3
	*LLKF_1167*	*ylfFG*	acyl-[acyl-carrier-protein] hydrolase	positive	2.5
	*LLKF_1550*	*coaA*	pantothenate kinase	positive	2.4
	*LLKF_0668*		GFO/IDH/MOCA family oxidoreductase	negative	0.2
	*LLKF_0861*	*choS*	glycine betaine ABC transporter permease/substrate-binding protein	positive	2.7
	*LLKF_0999*	*yjjH*	calcineurin-like phosphoesterase	positive	1.0
	*LLKF_1961*	*sufB*	cysteine desulfurase activator complex subunit SufB	positive	13.9
	*LLKF_0443*	*noxE*	NADH oxidase	positive	29.5
	*LLKF_0020*	*tilS*	tRNA(Ile)-lysidine synthetase	positive	2.3
	***LLKF_0802***	***cysK***	**cysteine synthase**	positive	2.2
	*LLKF_0898*	*pnuC*	nicotinamide mononucleotide transporter/n-ribosylnicotinamide transporter	positive	4.0
	*LLKF_1536*	*pp270*	phage protein	positive	0.6
	*LLKF_0661*	*scrR*	LacI family sucrose operon repressor	positive	0.8
	*LLKF_1521*	*pp255*	phage protein	negative	0.3
	*LLKF_0284*		transcriptional regulator	positive	2.5
	*LLKF_0982*	*grpE*	molecular chaperone GrpE	negative	6.4
	*LLKF_1261*	*leuB*	3-isopropylmalate dehydrogenase	negative	0.5
	*LLKF_2093*	*ytgF*	2,3-cyclic-nucleotide 2-phosphodiesterase	positive	10.6
	*LLKF_0100*		short chain dehydrogenase	negative	5.8
	*LLKF_1331*	*ymjE*	family 2 glycosyltransferase	positive	2.3
	*LLKF_0093*		ABC transporter permease	negative	8.2
	*LLKF_1359*	*rnhB*	ribonuclease HII	positive	0.9
	*LLKF_0165*	*ybhD*	GNAT family acetyltransferase	positive	0.5
	*LLKF_1075*	*pp146*	phage protein	positive	2.7
	*LLKF_0310*		hypothetical protein	negative	0.6
	*LLKF_0981*	*hrcA*	Heat-inducible transcription repressor HrcA	negative	5.7
	*LLKF_0695*		hypothetical protein	positive	7.5
	*LLKF_1578*	*ypaD*	hypothetical protein	negative	4.5
	*LLKF_1799*	*aroD*	3-dehydroquinate dehydratase	negative	1.9
	*LLKF_2229*		conjugative transposon Tn5276 integrase	negative	0.8
	*LLKF_1872*	*yrgF*	hypothetical protein	negative	0.5
	*LLKF_1527*	*pp261*	phage protein	negative	0.1
	*LLKF_0029*	*yafF*	hypothetical protein	positive	0.8
	*LLKF_2431*	*gntR*	RpiR family transcriptional regulator	negative	2.1
	*LLKF_0983*	*dnaK*	chaperone protein DnaK	negative	12.8
	*LLKF_1695*	*thiL*	acetyl-CoA acetyltransferase	positive	4.6
	*LLKF_0551*	*dfpA*	phosphopantothenoylcysteine decarboxylase	positive	1.5
	*LLKF_0663*	*scrA*	PTS system sucrose-specific transporter subunit IIABC	positive	0.5
	*LLKF_2232*		hypothetical protein	negative	0.9
	*LLKF_1965*	*sufD*	SUF system FeS cluster assembly protein SufD	positive	11.3
	*LLKF_0510*	*adaA*	methylphosphotriester-DNA alkyltransferase	positive	0.1
	*LLKF_1352*	*gltB*	glutamate synthase large subunit	negative	5.1
	*LLKF_1018*	*ribH*	riboflavin synthase subunit beta	positive	0.2
	*LLKF_0570*	*yfiE*	organic hydroperoxide resistance family protein	positive	24.3
	*LLKF_0647*	*citB*	aconitate hydratase	negative	0.3
	*LLKF_0471*	*ligA*	NAD-dependent DNA ligase	positive	2.9
	*LLKF_0215*	*yqeL*	GTP-binding protein	positive	2.0
	*LLKF_0151*	*ybgA*	hypothetical protein	negative	0.3
	*LLKF_2444*	*pp401*	phage integrase	positive	2.4
	*LLKF_1853*		hypothetical protein	positive	7.3
	*LLKF_1066*	*pp137*	phage HNH endonuclease	positive	0.7
	*LLKF_2398*	*adhE*	alcohol dehydrogenase/ acetaldehyde dehydrogenase	negative	6.5
	*LLKF_1858*		ABC transporter permease	negative	0.4
	*LLKF_1656*	*yphI*	hypothetical protein	positive	0.3
	*LLKF_1324*	*dltC*	D-alanine—poly(phosphoribitol) ligase subunit 2	negative	3.2
	*LLKF_1284*	*recA*	recombinase recA, C-terminal fragement	negative	0.2
	*LLKF_1644*	*clpB*	ATP-dependent Clp protease chaperonin ATPase ClpB	negative	3.1
	*LLKF_0873*	*xseA*	exodeoxyribonuclease VII large subunit	positive	2.5
	*LLKF_0520*	*yfcI*	metallo-beta-lactamase family protein	positive	0.9
	*LLKF_1071*	*pp142*	phage major head protein	positive	1.6
	***LLKF_1566***	***trpA***	**tryptophan synthase subunit alpha**	positive	0.8
	*LLKF_1269*	*ilvC*	ketol-acid reductoisomerase	negative	1.8
	*LLKF_0822*	*rnc*	ribonuclease III	positive	1.5
	*LLKF_1132*	*cobQ*	cobB/cobQ-like glutamine amidotransferase	positive	3.0
	*LLKF_1501*	*pp235*	phage terminase large subunit	negative	0.1
	*LLKF_1887*	*pstA*	phosphate ABC transporter ATP-binding protein	positive	4.0
	*LLKF_1424*	*pfkA*	6-phosphofructokinase	negative	14.2
	*LLKF_0854*	*mutY*	A/G-specific adenine DNA glycosylase	positive	1.5
	*LLKF_0889*	*yijB*	hypothetical protein	negative	0.2
	*LLKF_0505*	*yfaA*	hypothetical protein	positive	0.8
	*LLKF_0918*	*tcsR*	Two-component response regulator	positive	1.7
	*LLKF_0390*	*yddD*	glyoxalase family protein	positive	0.2
	*LLKF_1805*	*ccpA*	catabolite control protein A	positive	4.6
	*LLKF_2245*		hypothetical protein	negative	2.2
	*LLKF_1546*	*deoC*	deoxyribose-phosphate aldolase	positive	3.2
	*LLKF_1589*		putrescine/ornithine aminotransferase	negative	0.1
	*LLKF_0270*	*nrdD*	anaerobic ribonucleoside-triphosphate reductase	negative	11.7
	*LLKF_0313*		hypothetical protein	negative	0.1
	*LLKF_1486*	*pp220*	phage protein	positive	0.5
	*LLKF_0104*		hypothetical protein	negative	0.2
	*LLKF_2239*		hypothetical protein	negative	3.0
	*LLKF_1351*	*gltD*	glutamate synthase small subunit	negative	3.3
	*LLKF_1728*	*csc2A*	c-terminal membrane anchored cell surface protein	negative	0.1
	*LLKF_2066*	*yteB*	glycine/D-amino acid oxidase family protein	positive	0.2
	*LLKF_0915*	*rpsN*	50S ribosomal protein S14P	negative	0.1
	*LLKF_0385*	*fhuD*	ferrichrome ABC transporter substrate-binding protein FhuD	positive	8.9
	*LLKF_0640*	*pfl*	formate acetyltransferase	negative	11.3
	*LLKF_1348*	*murI*	glutamate racemase	positive	2.2
	*LLKF_2368*	*comGE*	competence protein ComGE	negative	0.1
	*LLKF_0222*	*yccJ*	hypothetical protein	positive	4.8
SK11	***LACR_2496***		**gluconate kinase**	positive	
	*LACR_2183*		manganese transporter NRAMP	positive	1.5
	*LACR_2273*		hypothetical protein	positive	25.8
	*LACR_2219*		hypothetical protein	positive	1.1
	*LACR_1490*		hypothetical protein	positive	0.2
	*LACR_C29*		hypothetical protein	positive	15.9
	***LACR_1011***		**ABC-type polar amino acid transport system, ATPase component**	positive	12.9
	*LACR_1370*		cation-transporting P-ATPase	positive	20.0
	*LACR_1188*		hypothetical protein	positive	3.8
	*LACR_2217*		hypothetical protein	positive	1.0
	*LACR_1428*		hypothetical protein	positive	14.1
	*LACR_1467*		hypothetical protein	positive	8.6
	*LACR_0359*		hypothetical protein	positive	5.3
	*LACR_2213*		hypothetical protein	positive	3.3
	*LACR_2358*		integral membrane protein	negative	6.2
	*LACR_1427*		DeoR family transcriptional regulator	positive	9.4
	*LACR_1389*		hypothetical protein	positive	7.1
	*LACR_0544*		hypothetical protein	positive	0.7
	*LACR_1168*		hypothetical protein	positive	0.5
	*LACR_1369*		Mn-dependent transcriptional regulator	positive	6.5
	*LACR_0743*		flavodoxin	positive	2.0
	*LACR_1502*		hypothetical protein	positive	1.9
	*LACR_A11*		relaxase/mobilization nuclease domain-containing protein	positive	39.9
	*LACR_0543*	*recU*	Holliday junction-specific endonuclease	positive	3.2
	*LACR_0274*		hypothetical protein	positive	2.4
	*LACR_2272*		hypothetical protein	positive	5.3
	*LACR_1231*		hypothetical protein	negative	1.2
	*LACR_0805*		hypothetical protein	positive	1.3
	*LACR_2216*		hypothetical protein	positive	2.0
	*LACR_1390*		transcriptional regulator	positive	19.2
	*LACR_2499*		hypothetical protein	positive	2.3
	*LACR_0927*		acetyltransferase	positive	5.2
	*LACR_1715*		cation transport protein	positive	3.4
	*LACR_0774*		menaquinone-specific isochorismate synthase	positive	4.0
	*LACR_1524*		Signal transduction histidine kinase	positive	7.3
	*LACR_2012*		gamma-aminobutyrate permease related permease	negative	4.4
	*LACR_1302*	*xerS*	site-specific tyrosine recombinase XerS	positive	16.5
	*LACR_C54*		hypothetical protein	positive	4.8
	*LACR_0329*		acetyltransferase	positive	3.0
	*LACR_0302*		transcriptional regulator	positive	2.0
	*LACR_0398*	*asnB*	asparagine synthetase B	negative	17.5
	*LACR_A05*		hypothetical protein	positive	3.0
	*LACR_2026*		ABC-type oligopeptide transport system, periplasmic component	negative	4.4
	*LACR_2220*		hypothetical protein	positive	1.4
	*LACR_2522*		hypothetical protein	positive	4.4
	*LACR_1437*		transposase	positive	9.2
	***LACR_1714***		**ArsR family transcriptional regulator**	positive	3.1
	*LACR_0904*		transcriptional regulator	positive	0.6
	*LACR_2151*		hypothetical protein	positive	3.1
	*LACR_1052*		putative exporter of polyketide antibiotics	positive	3.3
	*LACR_2126*		hypothetical protein	negative	6.1
	*LACR_1379*		hypothetical protein	positive	1.2
	*LACR_1525*		hypothetical protein	positive	2.3
	*LACR_0781*		hypothetical protein	positive	2.5
	*LACR_1237*	*truB*	tRNA pseudouridine synthase B	positive	2.0
	*LACR_1261*		hypothetical protein	positive	1.5
	*LACR_C27*		pyrrolidone-carboxylate peptidase	positive	6.5
	*LACR_1505*		transposase	positive	9.0
	*LACR_0803*		hypothetical protein	positive	1.3
	*LACR_2218*		hypothetical protein	positive	2.1
	*LACR_2270*		hypothetical protein	positive	18.7
	*LACR_1987*	*murE*	UDP-N-acetylmuramoylalanyl-D-glutamate—2,6-diaminopimelate ligase	positive	3.6
	*LACR_1104*		hypothetical protein	negative	5.0
	*LACR_0812*		putative effector of murein hydrolase LrgA	positive	4.1
	*LACR_1019*		hypothetical protein	negative	4.4
	*LACR_1523*		DNA-binding response regulator	positive	5.0
	*LACR_0804*		hypothetical protein	positive	2.2
	*LACR_0140*		hypothetical protein	positive	0.1
	*LACR_0505*		hypothetical protein	negative	0.4
	*LACR_1362*		transcriptional regulator	positive	1.8
	*LACR_C28*		dienelactone hydrolase family protein	positive	14.6
	*LACR_2274*		hypothetical protein	positive	12.9
	*LACR_1031*		lactose transport regulator	positive	2.6
	*LACR_1067*		amidase	positive	0.5
	*LACR_2592*		hypothetical protein	positive	0.2
	*LACR_1032*		tagatose-6-phosphate kinase	positive	4.9
	*LACR_0422*		transcriptional regulator	positive	0.4
	*LACR_0450*		hypothetical protein	positive	0.4
	*LACR_1982*		pleiotropic transcriptional repressor	positive	0.1
	*LACR_0809*		hypothetical protein	positive	2.3
	*LACR_2381*	*secY*	preprotein translocase subunit SecY	negative	21.0
	*LACR_2340*		hypothetical protein	positive	1.7
	*LACR_D08*		site-specific recombinase, DNA invertase Pin related protein	negative	11.5
	*LACR_1260*		hypothetical protein	positive	1.4
	*LACR_1122*		deoxyuridine 5'-triphosphate nucleotidohydrolase	negative	6.4
	*LACR_1079*		hypothetical protein	positive	1.5
	*LACR_2118*		deoxyuridine 5'-triphosphate nucleotidohydrolase	negative	4.6
	*LACR_0432*		membrane carboxypeptidase (penicillin-binding protein)	positive	2.6
	*LACR_0807*		sortase (surface protein transpeptidase)	positive	1.2
	*LACR_1020*		hypothetical protein	negative	4.4
	*LACR_1164*		hypothetical protein	positive	0.3
	*LACR_0301*		integrase	positive	2.2
	*LACR_2515*	*ruvB*	Holliday junction DNA helicase RuvB	positive	2.8
	*LACR_2119*		hypothetical protein	negative	1.8
	*LACR_0582*		dinucleoside polyphosphate hydrolase	positive	1.9
	*LACR_0511*		hypothetical protein	positive	4.8
	*LACR_0775*		SSU ribosomal protein S5P alanine acetyltransferase	positive	1.0
	*LACR_2134*		hypothetical protein	negative	1.9
	*LACR_2116*		hypothetical protein	negative	1.5
	*LACR_2357*		hypothetical protein	negative	1.3
	*LACR_2558*		transcriptional regulator	positive	0.5
	*LACR_0956*		transcriptional regulator	positive	1.7
	*LACR_1891*		competence protein	negative	0.2
	*LACR_0094*		D-tyrosyl-tRNA(Tyr) deacylase	positive	0.7
	*LACR_0201*		hypothetical protein	negative	5.9
	*LACR_2462*		transposase	positive	12.1
	*LACR_1458*		N-acetylglucosamine 6-phosphate deacetylase	positive	3.5
	*LACR_C08*		acetyltransferase	negative	0.6
	*LACR_1266*		xanthine/uracil permease	negative	0.9
	*LACR_0870*		HAD superfamily hydrolase	positive	2.3
	*LACR_D23*		replication initiator protein	positive	2.5
	*LACR_1635*		transposase	positive	9.3
	*LACR_0715*		Mg-dependent DNase	positive	1.4
	*LACR_1856*		hypothetical protein	positive	1.4
	*LACR_0652*		XRE family transcriptional regulator	positive	1.5
	*LACR_1631*	*thyA*	thymidylate synthase	positive	2.0
	*LACR_0249*		HAD superfamily hydrolase	negative	1.1
	*LACR_0680*		transposase	positive	12.4
	*LACR_1099*		XRE family transcriptional regulator	positive	7.6
	*LACR_2061*		TIM-barrel fold family protein	negative	11.5
	*LACR_1423*		hypothetical protein	positive	4.0
	*LACR_1063*		ribonucleoside-diphosphate reductase class Ib glutaredoxin subunit	positive	10.8
	*LACR_0066*		transcriptional regulator	positive	2.2
	*LACR_C32*		transposase	negative	20.6
B					
Strain	Locus tag	Gene	Function	Correlation	Slope
IL1403	***L162840***	***yhgC***	**transcription regulator**	negative	0.1
	***L79507***	***yahD***	**hypothetical protein**	positive	2.8
	*L0275*	*dnaN*	DNA polymerase III subunit beta	positive	7.1
	*L104969*	*napC*	multidrug-efflux transporter	positive	0.2
	*L189822*	*ybiK*	hypothetical protein	positive	8.4
	***L109527***	***rsuA***	**ribosomal small subunit pseudouridine synthase A**	negative	0.8
	*L84992*	*ytaB*	YtaB	positive	2.9
	*L0165*	*ribA*	3,4-dihydroxy-2-butanone 4-phosphate synthase	positive	0.2
	*L4822*	*ptsK*	HPr kinase/phosphorylase	positive	5.9
	*L196779*	*yfjD*	tRNA/rRNA methyltransferase	negative	1.7
	*L180241*	*mycA*	myosin-cross-reactive antigen	positive	5.4
	*L7798*	*ps316*	integrase	negative	1.6
	*L30663*	*ycdA*	hypothetical protein	negative	0.9
	*L20937*	*ywdF*	hypothetical protein	negative	3.3
	*L190009*	*feoB*	ferrous ion transport protein B	positive	8.1
	*L0016*	*gpsA*	NAD(P)H-dependent glycerol-3-phosphate dehydrogenase	positive	4.8
	***L193030***	***yjjD***	**ABC transporter permease protein**	positive	0.9
	*L136552*	*ybdJ*	hypothetical protein	positive	0.2
	*L179531*	*ispB*	heptaprenyl diphosphate synthase component II	positive	4.3
	*L0241*	*uxuB*	fructuronate reductase	positive	0.1
	*L177590*	*hasC*	UTP-glucose-1-phosphate uridylyltransferase	positive	5.7
	*L0274*	*dnaA*	chromosomal replication initiation protein	positive	7.2
	*L114325*	*ybbE*	hypothetical protein	negative	0.8
	*L0298*	*topA*	DNA topoisomerase I	negative	4.3
	*L32731*	*ykdB*	hypothetical protein	positive	1.0
	*L17893*	*yebF*	transcription regulator	positive	1.2
	*L180104*	*umuC*	UmuC	positive	0.3
	*L0101*	*metA*	homoserine O-succinyltransferase	positive	1.7
	*L197697*	*yfjE*	flavodoxin	negative	1.5
	*L200024*		hypothetical protein	positive	0.4
	*L5776*	*lgt*	prolipoprotein diacylglyceryl transferase	positive	2.0
	*L135900*	*ybdI*	hypothetical protein	positive	0.2
KF147	*LLKF_2311*		family 2 glycosyltransferase	negative	0.3
	*LLKF_0448*	*tcsK*	Two-component sensor histidine kinase	negative	5.5
SK11	*LACR_0741*		hypothetical protein	positive	0.8
	*LACR_0891*		copper/potassium-transporting ATPase	positive	4.4
	*LACR_E7*		hypothetical protein	positive	4.1
	*LACR_1450*		fibronectin-binding protein	positive	1.1
	***LACR_0073***		**esterase**	positive	10.0
	*LACR_0714*		hypothetical protein	positive	3.7
	*LACR_C16*		replication initiator protein	positive	3.3
	*LACR_0074*		lactoylglutathione lyase related lyase	positive	7.0
	*LACR_1221*		hypothetical protein	positive	2.1
	*LACR_0072*		hypothetical protein	positive	8.5
	*LACR_0920*		copper-potassium transporting ATPase B	positive	5.0
	*LACR_0959*		hypothetical protein	positive	1.3
	*LACR_0242*		saccharopine dehydrogenase related protein	positive	10.1
	*LACR_0451*		ABC-type multidrug transport system, permease component	positive	3.0
	*LACR_0713*		acetyltransferase	positive	2.9
	*LACR_0452*		ABC-type multidrug transport system, ATPase component	positive	5.2
	*LACR_0381*		hypothetical protein	positive	0.5
	*LACR_1506*		hypothetical protein	positive	0.3
	*LACR_0744*		lysophospholipase L1 related esterase	positive	1.5
	*LACR_2167*		N-acetylmuramoyl-L-alanine amidase	positive	4.5
	*LACR_0347*		ABC-type multidrug transport system, ATPase and permease component	positive	4.4
	*LACR_1291*		Beta-xylosidase	positive	0.5
	*LACR_1468*		orotidine 5'-phosphate decarboxylase	positive	5.4
	*LACR_0240*		NADPH:quinone reductase related Zn-dependent oxidoreductase	positive	11.5
	*LACR_1051*		ABC-type multidrug transport system, ATPase component	positive	3.0
	*LACR_0075*		hypothetical protein	positive	6.7
	*LACR_0241*		nucleoside-diphosphate sugar epimerase	positive	11.2
	*LACR_0105*		hypothetical protein	positive	3.3
	*LACR_0629*		major facilitator superfamily permease	positive	0.3
	*LACR_0164*		hypothetical protein	positive	3.8
	*LACR_2411*		hypothetical protein	negative	0.9
	*LACR_1362*		transcriptional regulator	positive	1.1
	*LACR_0982*		ring-cleavage extradiol dioxygenase	positive	3.6
	*LACR_0742*		transcriptional regulator	positive	2.0
	*LACR_0537*		cysteine synthase	positive	0.4
	*LACR_0743*		flavodoxin	positive	1.2
	*LACR_D16*		oligopeptidase O1	negative	11.3
	*LACR_2476*		transcriptional regulator	positive	5.7
	*LACR_0839*		sugar metabolism transcriptional regulator	positive	1.7
	*LACR_1302*	*xerS*	site-specific tyrosine recombinase XerS	positive	9.7
	*LACR_1290*		endoglucanase	positive	0.2
	*LACR_2355*		hypothetical protein	positive	0.8
	*LACR_1976*		negative regulator of genetic competence, sporulation and motility	positive	2.4
	*LACR_1629*		transcriptional regulator	positive	2.1
	*LACR_1395*		hypothetical protein	positive	3.7
	*LACR_1922*		hypothetical protein	negative	1.1
	*LACR_1267*		hypothetical protein	positive	0.4
	*LACR_2497*		6-phosphogluconate dehydrogenase-like protein	positive	0.9
	*LACR_0431*		tyrosyl-tRNA synthetase	negative	10.0
	*LACR_0570*	*dnaG*	DNA primase	positive	2.4
	*LACR_0657*		adenine phosphoribosyltransferase	negative	5.6
	*LACR_2490*	*recX*	recombination regulator RecX	positive	14.2
	*LACR_1728*		Mg2+ transporter	positive	1.6
	*LACR_1751*		transposase	positive	3.3
	*LACR_0206*		glycosyltransferase	negative	1.0
	***LACR_1052***		**putative exporter of polyketide antibiotics**	positive	2.0
	*LACR_0642*		6-phosphogluconate dehydrogenase	negative	5.1
	*LACR_0800*		XRE family transcriptional regulator	positive	1.2
	*LACR_1078*		transcriptional regulator	negative	0.1
	***LACR_2545***		**ribosomal small subunit pseudouridine synthase A**	negative	1.6
	*LACR_2184*		oxidoreductase	positive	9.7
	*LACR_0212*		lipopolysaccharide biosynthesis protein	negative	1.8
	*LACR_1105*		hypothetical protein	positive	4.3

Correlating gene expressions with robustness towards heat stress (A) or oxidative stress (B) as assessed by a linear model of the strains IL1403, KF147 and SK11. Genes of which expression correlated with survival in more than one strain (including MG1363 [11]) are indicated in bold. Genes are ranked based on the significance of correlation (lowest *P*-value on top). Slope represents the average slope of the linear models fitting the data of both time points of the stress assay.

In KF147, the operon encoding the pyruvate dehydrogenase complex (*pdhABCD)* and a lipoate-protein ligase (*lplL*) as well as an operon encoding a ferrichrome ABC transporter (*fhuCDG*) and an operon encoding hypothetical proteins and a Gls24 family general stress protein (*ytgH*) displayed a positive correlation with heat stress survival. Surprisingly, the heat shock genes *grpE* and *dnaK* anti-correlated with robustness towards heat stress of KF147 and also their repressor *hrcA* displayed anti-correlation [24]. The gene *fhuC* was previously associated with heat stress survival in MG1363 [11], as well as four other genes: *uvrC*, *cysD*, *cysK* and *trpA*. In contrast to KF147 and MG1363, these transcripts did not show a significant correlation with heat stress survival in IL1403 nor in SK11, although a previous study by Xie *et al*. did suggest a role of *cysK* in heat stress survival of IL1403 [13]. Two other genes were found to associate with heat stress survival in both KF147 and SK11 (*rarA* and *yjgE*/ *LACR_1011*) and one in both IL1403 and SK11 (*gntK*). However, the majority of the correlating genes were shown to associate with stress survival in only one of the strains. In IL1403 the genes *aroF* and *aroH* encoding a phospho-2-dehydro-3-deoxyheptonate aldolase anti-correlated with heat stress survival. The gene *aroF* was previously shown to be upregulated in this strain during osmotic stress [13], suggesting this gene could be involved in a general stress mechanism. In SK11, multiple genes encoding hypothetical proteins were found to correlate with heat stress survival and also a gene encoding a manganese transporter (*LACR_2183*). Manganese transport was also associated with heat stress survival in an earlier study, where *mtsC*, encoding part of a manganese ABC transporter was shown to be present in robust strains and absent in sensitive strains within an *L*. *lactis* strain collection [5]. Metal ions have several functions in the cell and can be involved in stabilizing proteins, ribosomes and the cell membrane [25, 26]. Because these cellular components are affected during heat stress [8], manganese might have a role in the prevention of damage caused by heat stress.

Similar as for heat stress, the transcriptome signature associated with oxidative stress survival was highly strain-specific, which is exemplified by the fact that only three genes associated with oxidative stress survival in more than one strain. In both IL1403 and SK11 the gene expressions *yahD*/ *LACR_0073*, *yjjD*/ *LACR_1052* and *rsuA*/ *LACR_2545* were found to correlate with oxidative stress survival. In IL1403, 32 genes displayed correlation of expression with survival, among which was the gene *feoB*, which is involved in iron transport and was previously associated with heat stress survival in MG1363 [11]. In SK11, a gene encoding cysteine synthase positively correlated with oxidative stress survival. In MG1363 we previously demonstrated a link between cysteine metabolism and oxidative stress survival [11]. Sulfur-containing amino acids are readily oxidized and, therefore, cysteine metabolism could be involved in oxidative stress survival by affecting the redox balance in the cell. Furthermore, genes associated with oxidative stress survival in SK11 included genes encoding membrane proteins and regulators. For application as indicators for robustness, the genes with a high variation in gene expression (indicated by the slope in Table 2) appear to be most suitable, because they can be detected with methods such as quantitative PCR. For both heat and oxidative stress, none of the genes were associated with survival in more than two strains, although the majority of the genes that displayed correlation with survival are present in all four strains. This lack in overlap demonstrates that the transcriptome signature associated with stress survival is largely strain-dependent, and the complete transcriptome signature associated with robustness in one strain cannot be extrapolated fully to other strains. This indicates that the mechanisms aiming to improve robustness vary among the strains and, therefore, strategies resulting in improved robustness of one strain do not necessarily increase robustness of other strains. To acquire optimal robustness, the fermentation conditions of each strain require individual optimization.

### Generic *L*. *lactis* genes associated with robustness towards heat or oxidative stress

To establish whether a generic transcriptome signature for *L*. *lactis* exists, we searched for single genes with the most significant correlation with robustness towards heat and oxidative stress in all strains. For this, we chose one time point of the stress assay, in which the range between the extreme values of survival in all fermentations was the largest (see Materials and methods). We selected the orthologous groups (OGs) in which the genes of all four strains displayed either a positive or a negative correlation (*P* < 0.2, assessed with a linear model) of expression level with robustness phenotype and ranked these on average *P*-value per OG (Table 3). Notably, the top 10 genes included *ctsR*, encoding a class three stress genes transcriptional repressor, which displayed negative correlation of expression with oxidative stress survival in all four strains. This gene was previously demonstrated to be a key regulator of heat-shock induced gene expression in MG1363 [27]. The observation that the transcript level of this gene appeared in the top 10 list of most significant correlating genes with oxidative stress survival suggests that CtsR is also involved in oxidative stress regulation in *L*. *lactis*. Involvement of CtsR in other stress responses besides heat stress response was already suggested by Frees *et al*., who demonstrated that the CtsR regulon was induced at low pH [28]. Furthermore, in *Bacillus subtilis* involvement of CtsR in oxidative stress survival has been previously suggested as transcription of the CtsR regulon was increased during oxidative stress [29]. Besides the significant correlation with heat stress survival of KF147, as mentioned in the previous paragraph, the gene *lplL* also displayed a positive correlation of expression and heat stress survival in the other three strains. This gene was previously demonstrated to be involved in heat shock response in strain IL1403 [13], which further supports the role of this gene in heat stress survival in *L*. *lactis* strains in general. Furthermore, the list contained multiple genes encoding for proteins involved in iron(complex) transport (*feoA*, *fhuD*, *fhuG* and *fhuB*). The *fhu* operon may be involved in haem uptake, enabling respiration metabolism in *L*. *lactis* [30, 31] and was recently demonstrated to be induced in strain MG1363 during the early phase of growth at high oxygen levels [32]. Furthermore, it has been demonstrated that free intracellular iron increases oxidative stress through generation of ROS from hydrogen peroxide by the Fenton reaction, which causes cellular damage and mortality in stationary phase cells of *L*. *lactis* [33]. A link between iron metabolism and heat stress survival has been demonstrated in *Bacillus licheniformis*, where an overlap in response to heat shock and iron limitation was revealed [34]. Taken together, a link between iron metabolism and stress survival in *L*. *lactis* appears likely.

**Table 3:** Generic correlating gene expressions with robustness towards heat stress (A) or oxidative stress (B).

A								
locustag IL1403	locustag SK11	locustag KF147	locustag MG1363	gene	function	correlation	average *P*-value	maximum *P*-value
L191486	LACR_1356	LLKF_1201	llmg_1317	*yljB/nanE*	N-acetylmannosamine-6-phosphate 2-epimerase	positive	0.025	0.039
L101688	LACR_1561	LLKF_1575	llmg_1029	*ypaA*	hypothetical protein	negative	0.026	0.062
L195318	LACR_1054	LLKF_0997	llmg_1551	*yjjF/fdhC*	formate/nitrite transporter	negative	0.031	0.059
L89001	LACR_1179	LLKF_1106	llmg_1494	*ykiI*	ABC transporter permease	positive	0.035	0.095
L64373	LACR_0052	LLKF_0039	llmg_0075	*lplL*	lipoate-protein ligase	positive	0.044	0.116
L143312	LACR_0389	LLKF_0398	llmg_0362	*dppA/optS*	oligopeptide ABC transporter substrate binding protein	negative	0.046	0.138
L72684	LACR_1157	LLKF_1090	llmg_1513	*ykhE*	arsenate reductase	negative	0.048	0.103
L18206	LACR_1946	LLKF_1947	llmg_1957	*ysdB*	sodium ABC transporter ATP-binding protein	negative	0.053	0.106
L192240	LACR_0194	LLKF_0183	llmg_0200	*feoA*	ferrous iron transport protein A	positive	0.054	0.113
L148945	LACR_1868	LLKF_1872	llmg_0725	*yrgF*	hypothetical protein	negative	0.057	0.184
B								
locustag IL1403	locustag SK11	locustag KF147	locustag MG1363	gene	function	correlation	average *P*-value	maximum *P*-value
L0223	LACR_0665	LLKF_0631	llmg_0614	*ctsR*	class III stress genes transcriptional repressor	negative	0.025	0.055
L128386	LACR_0373	LLKF_0384	llmg_0348	*fhuG*	ferrichrome ABC transporter permease FhuG	positive	0.030	0.051
L100027	LACR_2040	LLKF_2041	llmg_2036	*ytbC*	hypothetical protein	negative	0.039	0.127
L0046	LACR_0642	LLKF_0600	llmg_0586	*gnd*	6-phosphogluconate dehydrogenase	negative	0.044	0.115
L127476	LACR_0372	LLKF_0383	llmg_0347	*fhuB*	ferrichrome ABC transporter permease protein	positive	0.059	0.109
L117074	LACR_2341	LLKF_2294	llmg_2327	*yvdD*	glycerol uptake facilitator protein	negative	0.066	0.134
L103246	LACR_1565	LLKF_1577	llmg_1026	*ypaC*	methylase for ubiquinone/menaquinone biosynthesis	negative	0.077	0.102
L104745	LACR_1567	LLKF_1579	llmg_1024	*ypaE*	hypothetical protein	negative	0.081	0.118
L162870	LACR_1609	LLKF_1641	llmg_0989	*ypgD*	ABC transporter ATP binding and permease protein	positive	0.098	0.183
L129403	LACR_0374	LLKF_0385	llmg_0349	*fhuD*	ferrichrome ABC transporter substrate binding protein	positive	0.099	0.157

Top 10 highest correlating transcript levels with robustness towards heat stress (A) or oxidative stress (B). Average *P*-value is the average of the *P*-values of the correlation as assessed by a linear model of the strains MG1363, IL1403, KF147 and SK11 and was used to rank the genes. Maximum *P*-value indicates the largest *P*-value of the correlation among the four strains.

Besides the genes that have previously been demonstrated to be involved in stress, the top 10 lists also included genes which to the best of our knowledge have not been associated with stress before. The transcript levels of *yljB*/*nanE*, encoding an *N*-acetylmannosamine-6-phosphate 2-epimerase involved in amino sugar metabolism, displayed the highest correlation in all four strains with robustness towards heat stress. Furthermore, genes encoding transport proteins or hypothetical proteins were among the genes with the most significant correlation of expression and heat or oxidative stress survival in all strains. Revealing the exact mechanism via which the functions encoded by these genes impact on robustness requires additional work.

The strains included in this study varied in type of subspecies, isolation source and general robustness [5] and therefore appear to represent a major part of the *L*. *lactis* species. Therefore, it is tempting to suggest that the generic gene expressions associated with robustness in this study can be applied as indicators of robustness for *L*. *lactis* strains in general, although individual transcriptome signatures are expected to predict robustness of specific strains more accurately.

## Conclusions

In this study we demonstrated that fermentation conditions (e.g. temperature and level of oxygen) have a large impact on heat and oxidative stress survival of *L*. *lactis* strains. Therefore, fermentation conditions prior to industrial processing of starter cultures should be carefully selected, and this is true for both intrinsically robust and sensitive strains [5]. The development of a general fermentation strategy for improved robustness of *L*. *lactis* starter cultures appears complicated as the effect of fermentation conditions on robustness towards heat and oxidative stress is strain-dependent, even though fermentation conditions have largely similar effects on growth characteristics. The larger part of the transcriptome signatures associated with robustness also appeared strain-specific, indicating that different mechanisms exist to improve robustness. Hence, to obtain optimal robustness in each individual strain tailor-made optimization of fermentation parameters is required. Furthermore, we explored the most significant associations of transcript levels and robustness that overlapped in all four strains, resulting in a generic transcriptome signature associated with robustness in these *L*. *lactis* strains, which included both known genes encoding stress related functions and novel genes. This generic transcriptome signature could function as an indicator for robustness and aid the selection of optimal fermentation conditions for optimal robustness during spray drying.

## Supporting Information

S1 Fig DNA microarray hybridization scheme. Numbers indicate fermentations as presented in Table 1. Samples connected with arrows were hybridized together, the arrow head represents Cy5-labeling, the back end Cy3-labeling.
(TIFF)

S2 Fig Growth curves during various fermentations Growth curves of strains IL1403, KF147 and SK11 in fermentations as presented in Table 1. The data points between the dotted lines indicate the moment of harvesting cells for RNA isolation and stress survival assays.
(TIF)

S3 Fig Genes expressed by individual fermentation parameters. Numbers indicate the amount of genes that are differently expressed (*P* < 0.05) by both the individual fermentation parameter (salt, oxygen, pH and temperature) specified in the top row and in the left column. Bars indicate percentages of overlap of differently expressed genes by both fermentation parameters (full bar = 100%).
(TIF)

S1 Table Heat and oxidative stress survival at the additional time point. Survival after 30 minutes of heat stress and after 60 minutes of oxidative stress in the various fermentations of strains IL1403, KF147 and SK11. Survival data represent averages of technical duplicates.
(DOCX)

S2 Table Correlation fermentation parameters and robustness. T-test-based correlation of individual fermentation parameters and robustness. Significant differences (*P* < 0.05) are underlined.
(DOCX)

S1 File Plots of gene expression and robustness levels in IL1403 (part 1). Expression levels of genes *L0001* –*L75633* plotted against survival after 60 minutes heat and 30 min oxidative stress. Survival is expressed as the difference of log CFU/ml after stress and before stress. Numbers indicate fermentations as presented in Table 1. *P*-values above the plots indicate significance of correlation (assessed by a linear model).
(ZIP)

S2 File Plots of gene expression and robustness levels in IL1403 (part 2). Expression levels of genes *L75676* –*L1889726* plotted against survival after 60 minutes heat and 30 min oxidative stress. Survival is expressed as the difference of log CFU/ml after stress and before stress. Numbers indicate fermentations as presented in Table 1. *P*-values above the plots indicate significance of correlation (assessed by a linear model).
(ZIP)

S3 File Plots of gene expression and robustness levels in KF147 (part 1). Expression levels of genes *LLKF_0001 –LLKF_1273* plotted against survival after 10 minutes heat and 30 minutes oxidative stress. Survival is expressed as the difference of log CFU/ml after stress and before stress. Numbers indicate fermentations as presented in Table 1. *P*-values above the plots indicate significance of correlation (assessed by a linear model).
(ZIP)

S4 File Plots of gene expression and robustness levels in KF147 (part 2). Expression levels of genes *LLKF_1274 –LLKF_2533* and *LLKF_p0001 –LLKF_p0036* plotted against survival after 10 minutes heat and 30 minutes oxidative stress. Survival is expressed as the difference of log CFU/ml after stress and before stress. Numbers indicate fermentations as presented in Table 1. *P*-values above the plots indicate significance of correlation (assessed by a linear model).
(ZIP)

S5 File Plots of gene expression and robustness levels in SK11 (part 1). Expression levels of genes *LACR_0001* –*LACR_1382* plotted against survival after 10 minutes heat and 30 minutes oxidative stress. Survival is expressed as the difference of log CFU/ml after stress and before stress. Numbers indicate fermentations as presented in Table 1. *P*-values above the plots indicate significance of correlation (assessed by a linear model).
(ZIP)

S6 File Plots of gene expression and robustness levels in SK11 (part 2). Expression levels of genes *LACR_1383* –*LACR_2610* and *LACR_A01* –*LACR_E8* plotted against survival after 10 minutes heat and 30 minutes oxidative stress. Survival is expressed as the difference of log CFU/ml after stress and before stress. Numbers indicate fermentations as presented in Table 1. *P*-values above the plots indicate significance of correlation (assessed by a linear model).
(ZIP)
