# Supplementary figures and images for: Strain-Dependent Transcriptome Signatures for Robustness in Lactococcus lactis (part 1 of 13)
Source: PLoS One. 2016 Dec 14;11(12):e0167944. doi: 10.1371/journal.pone.0167944 (PMC5156439; doi:10.1371/journal.pone.0167944)

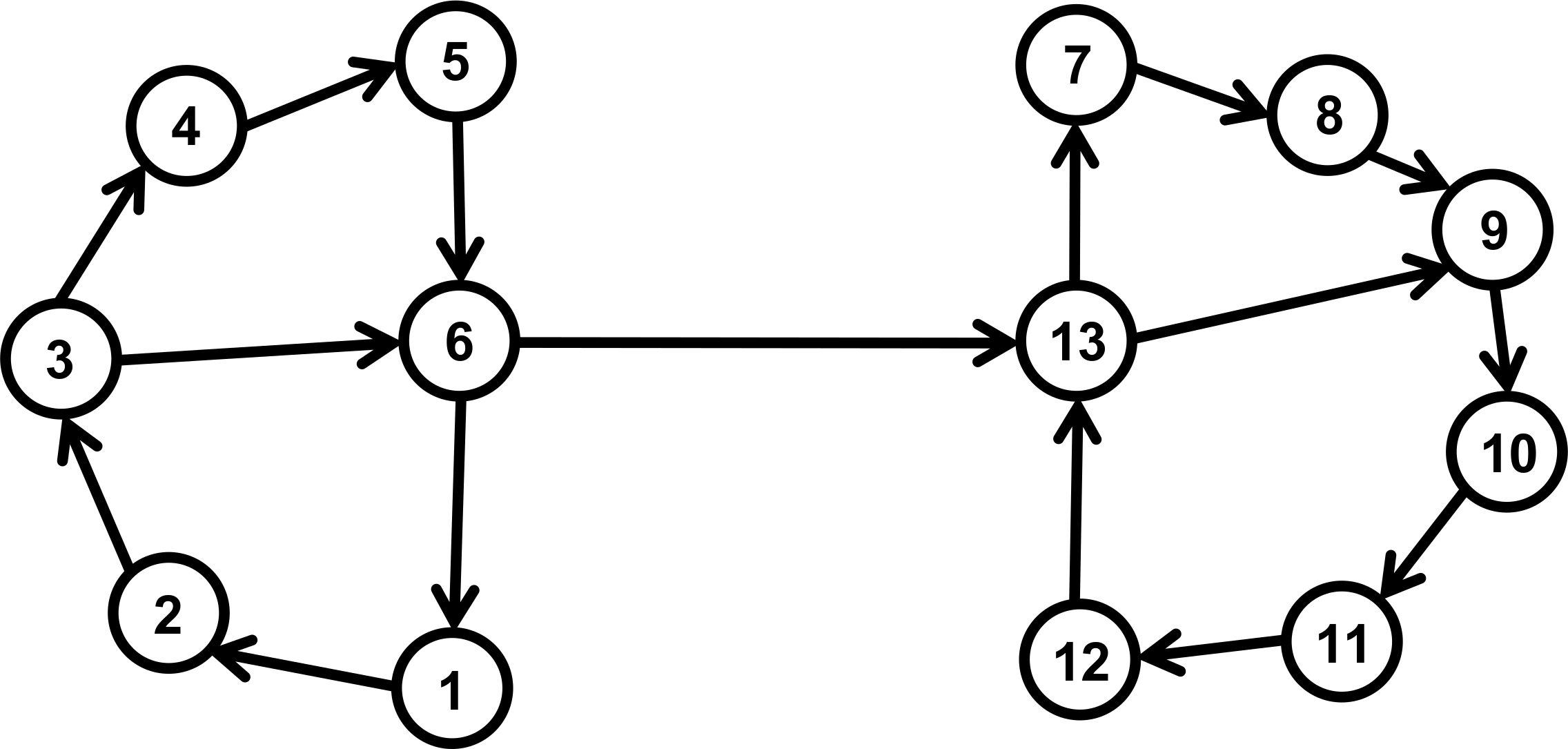

Supplement: S1 Fig — Numbers indicate fermentations as presented in Table 1. Samples connected with arrows were hybridized together, the arrow head represents Cy5-labeling, the back end Cy3-labeling. (TIFF) [file pone.0167944.s001.tiff]

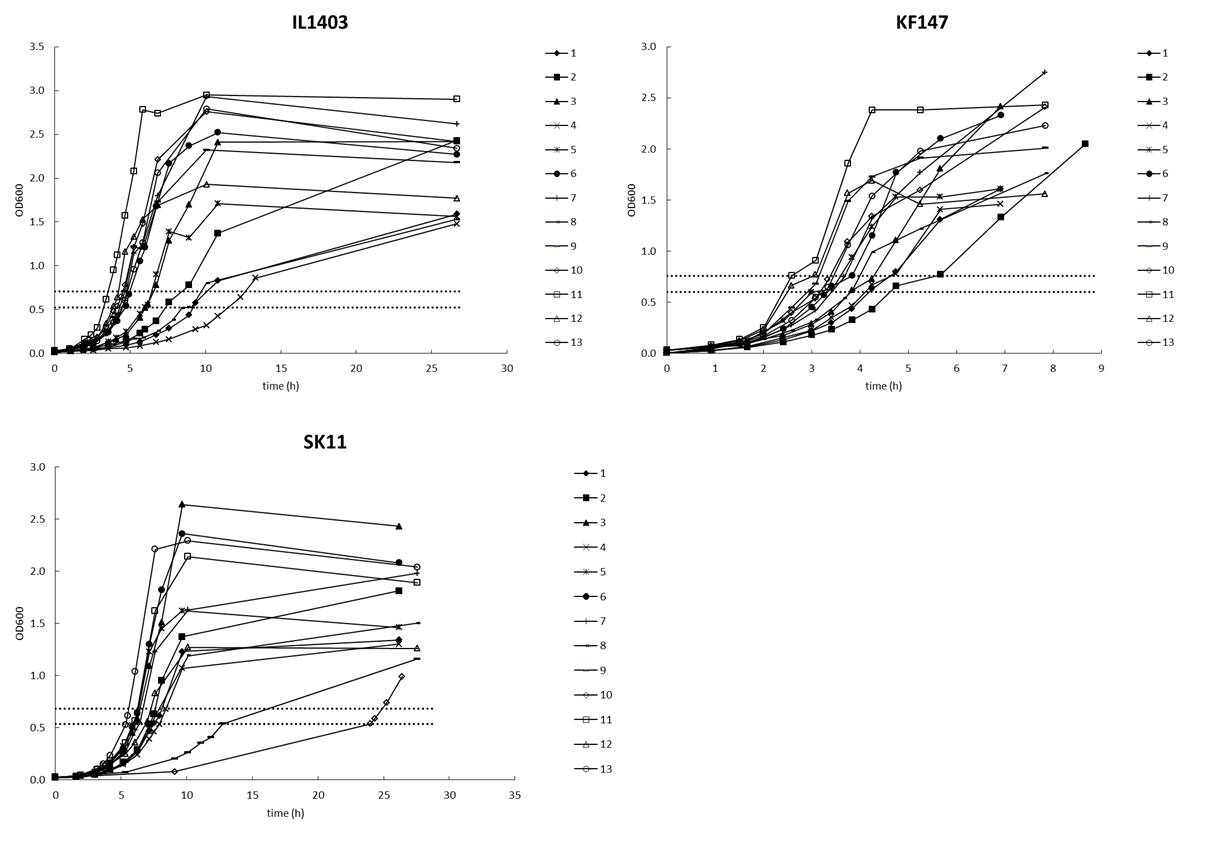

Supplement: S2 Fig — Growth curves of strains IL1403, KF147 and SK11 in fermentations as presented in Table 1. The data points between the dotted lines indicate the moment of harvesting cells for RNA isolation and stress survival assays. (TIF) [file pone.0167944.s002.tif]

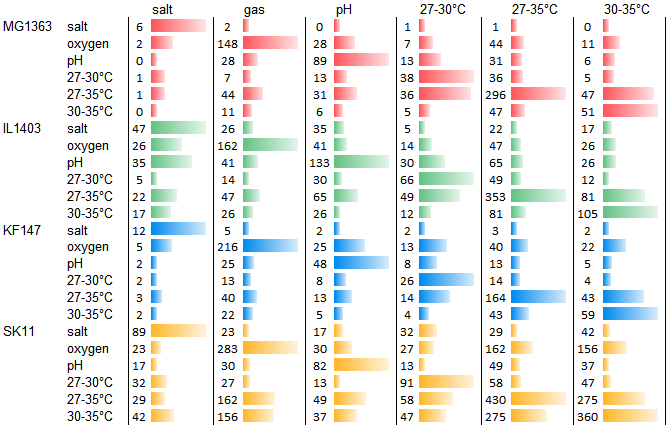

Supplement: S3 Fig — Numbers indicate the amount of genes that are differently expressed (P < 0.05) by both the individual fermentation parameter (salt, oxygen, pH and temperature) specified in the top row and in the left column. Bars indicate percentages of overlap of differently expressed genes by both fermentation parameters (full bar = 100%). (TIF) [file pone.0167944.s003.tif]

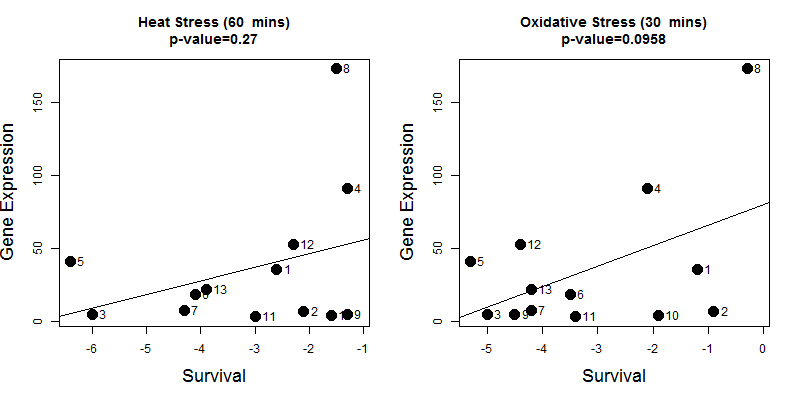

Supplement: S1 File — Expression levels of genes L0001 –L75633 plotted against survival after 60 minutes heat and 30 min oxidative stress. Survival is expressed as the difference of log CFU/ml after stress and before stress. Numbers indicate fermentations as presented in Table 1. P-values above the plots indicate significance of correlation (assessed by a linear model). (ZIP) [file pone.0167944.s006.zip › S1_File/L0001_real_dat.png]

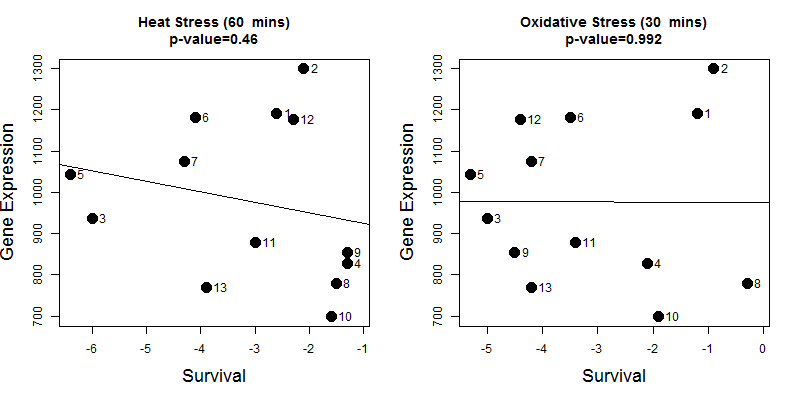

Supplement: S1 File — Expression levels of genes L0001 –L75633 plotted against survival after 60 minutes heat and 30 min oxidative stress. Survival is expressed as the difference of log CFU/ml after stress and before stress. Numbers indicate fermentations as presented in Table 1. P-values above the plots indicate significance of correlation (assessed by a linear model). (ZIP) [file pone.0167944.s006.zip › S1_File/L0002_real_dat.png]

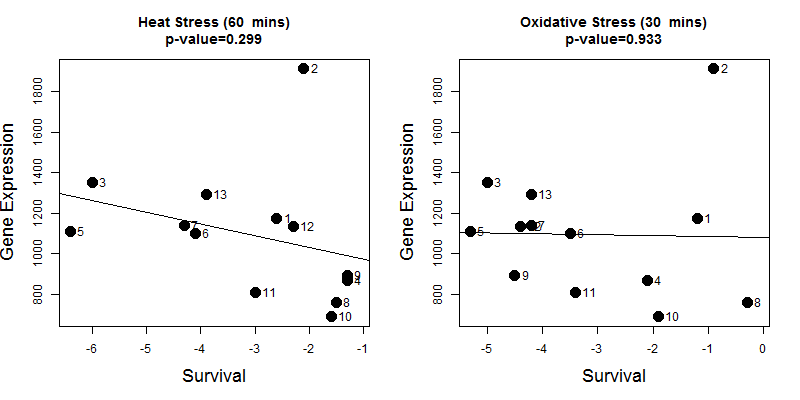

Supplement: S1 File — Expression levels of genes L0001 –L75633 plotted against survival after 60 minutes heat and 30 min oxidative stress. Survival is expressed as the difference of log CFU/ml after stress and before stress. Numbers indicate fermentations as presented in Table 1. P-values above the plots indicate significance of correlation (assessed by a linear model). (ZIP) [file pone.0167944.s006.zip › S1_File/L0003_real_dat.png]

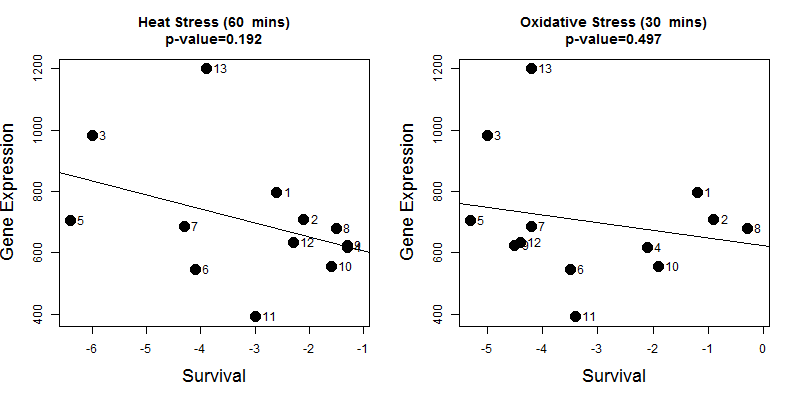

Supplement: S1 File — Expression levels of genes L0001 –L75633 plotted against survival after 60 minutes heat and 30 min oxidative stress. Survival is expressed as the difference of log CFU/ml after stress and before stress. Numbers indicate fermentations as presented in Table 1. P-values above the plots indicate significance of correlation (assessed by a linear model). (ZIP) [file pone.0167944.s006.zip › S1_File/L0004_real_dat.png]

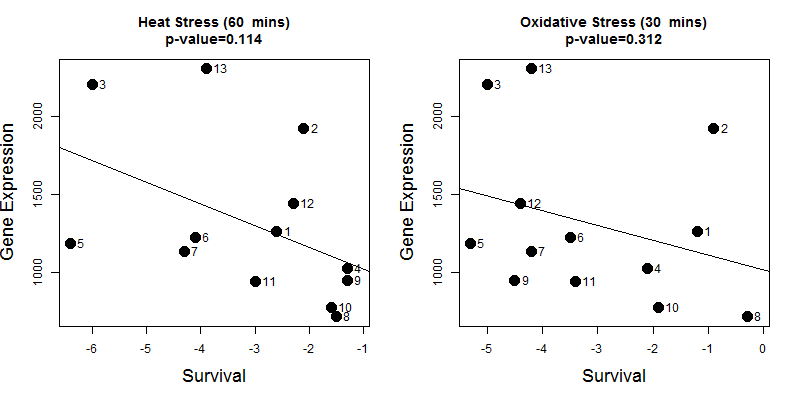

Supplement: S1 File — Expression levels of genes L0001 –L75633 plotted against survival after 60 minutes heat and 30 min oxidative stress. Survival is expressed as the difference of log CFU/ml after stress and before stress. Numbers indicate fermentations as presented in Table 1. P-values above the plots indicate significance of correlation (assessed by a linear model). (ZIP) [file pone.0167944.s006.zip › S1_File/L0005_real_dat.png]

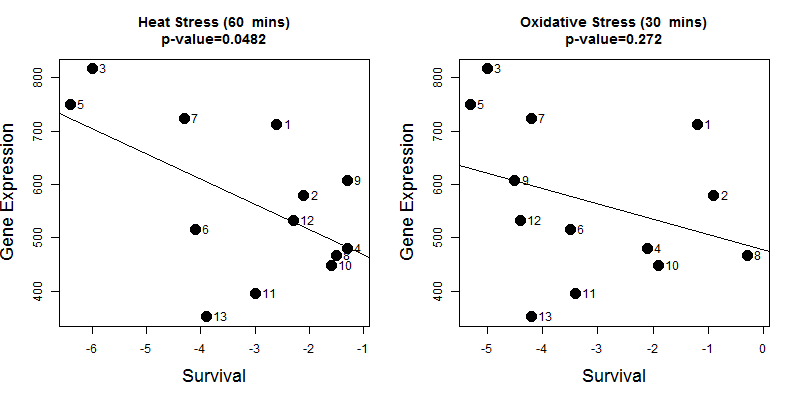

Supplement: S1 File — Expression levels of genes L0001 –L75633 plotted against survival after 60 minutes heat and 30 min oxidative stress. Survival is expressed as the difference of log CFU/ml after stress and before stress. Numbers indicate fermentations as presented in Table 1. P-values above the plots indicate significance of correlation (assessed by a linear model). (ZIP) [file pone.0167944.s006.zip › S1_File/L0006_real_dat.png]

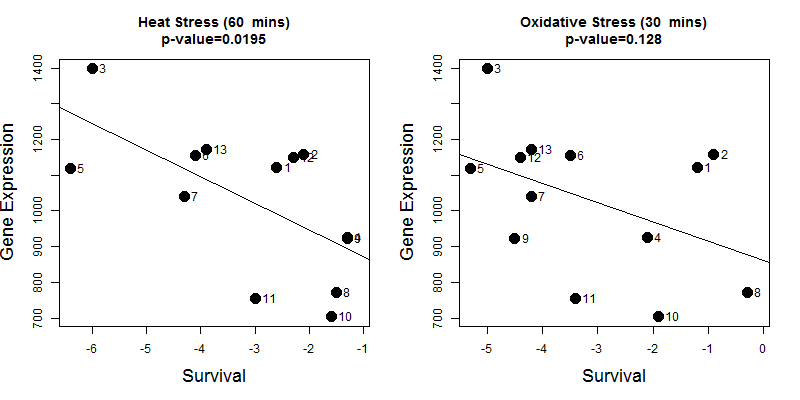

Supplement: S1 File — Expression levels of genes L0001 –L75633 plotted against survival after 60 minutes heat and 30 min oxidative stress. Survival is expressed as the difference of log CFU/ml after stress and before stress. Numbers indicate fermentations as presented in Table 1. P-values above the plots indicate significance of correlation (assessed by a linear model). (ZIP) [file pone.0167944.s006.zip › S1_File/L0007_real_dat.png]

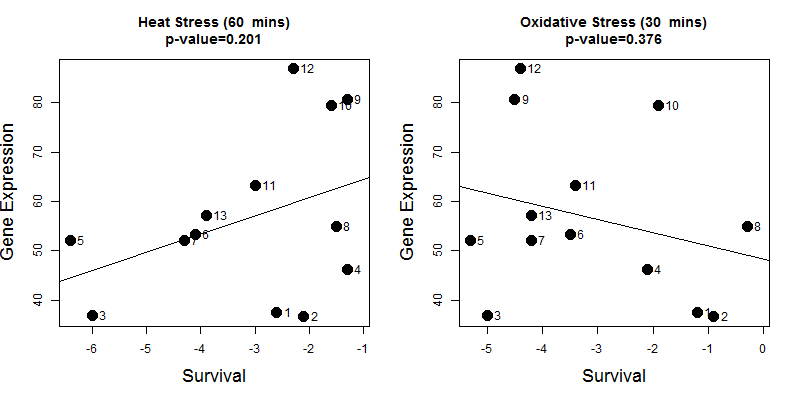

Supplement: S1 File — Expression levels of genes L0001 –L75633 plotted against survival after 60 minutes heat and 30 min oxidative stress. Survival is expressed as the difference of log CFU/ml after stress and before stress. Numbers indicate fermentations as presented in Table 1. P-values above the plots indicate significance of correlation (assessed by a linear model). (ZIP) [file pone.0167944.s006.zip › S1_File/L0008_real_dat.png]

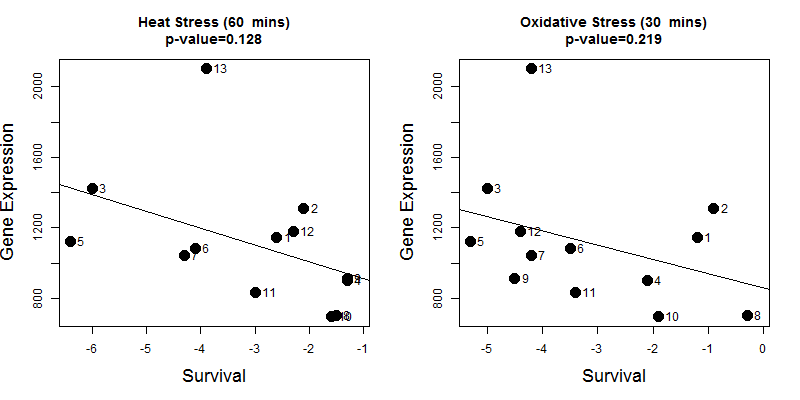

Supplement: S1 File — Expression levels of genes L0001 –L75633 plotted against survival after 60 minutes heat and 30 min oxidative stress. Survival is expressed as the difference of log CFU/ml after stress and before stress. Numbers indicate fermentations as presented in Table 1. P-values above the plots indicate significance of correlation (assessed by a linear model). (ZIP) [file pone.0167944.s006.zip › S1_File/L0009_real_dat.png]

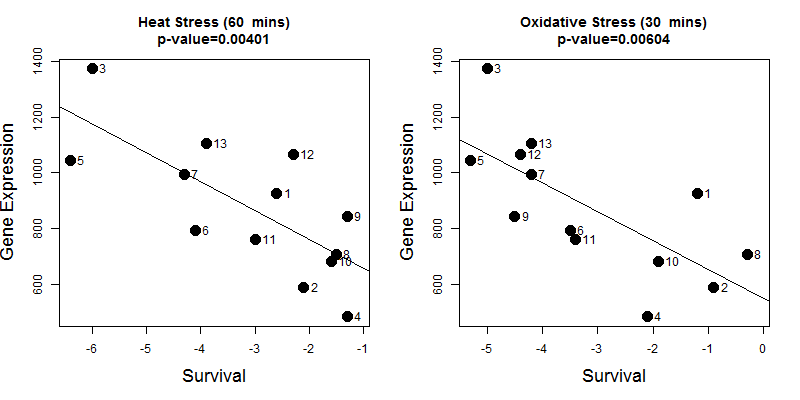

Supplement: S1 File — Expression levels of genes L0001 –L75633 plotted against survival after 60 minutes heat and 30 min oxidative stress. Survival is expressed as the difference of log CFU/ml after stress and before stress. Numbers indicate fermentations as presented in Table 1. P-values above the plots indicate significance of correlation (assessed by a linear model). (ZIP) [file pone.0167944.s006.zip › S1_File/L00096_real_dat.png]

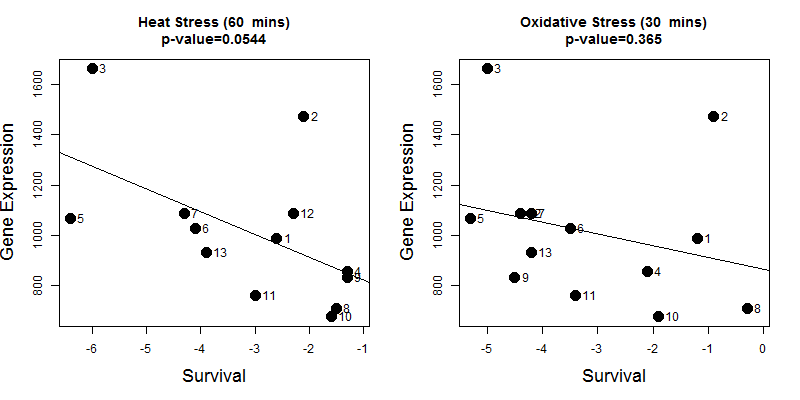

Supplement: S1 File — Expression levels of genes L0001 –L75633 plotted against survival after 60 minutes heat and 30 min oxidative stress. Survival is expressed as the difference of log CFU/ml after stress and before stress. Numbers indicate fermentations as presented in Table 1. P-values above the plots indicate significance of correlation (assessed by a linear model). (ZIP) [file pone.0167944.s006.zip › S1_File/L0010_real_dat.png]

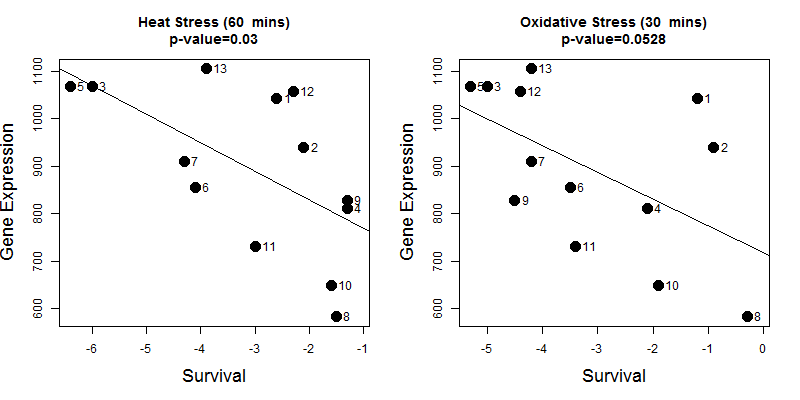

Supplement: S1 File — Expression levels of genes L0001 –L75633 plotted against survival after 60 minutes heat and 30 min oxidative stress. Survival is expressed as the difference of log CFU/ml after stress and before stress. Numbers indicate fermentations as presented in Table 1. P-values above the plots indicate significance of correlation (assessed by a linear model). (ZIP) [file pone.0167944.s006.zip › S1_File/L0011_real_dat.png]

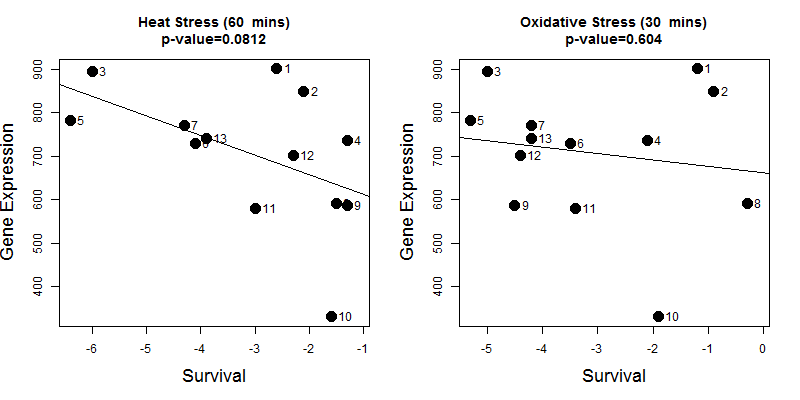

Supplement: S1 File — Expression levels of genes L0001 –L75633 plotted against survival after 60 minutes heat and 30 min oxidative stress. Survival is expressed as the difference of log CFU/ml after stress and before stress. Numbers indicate fermentations as presented in Table 1. P-values above the plots indicate significance of correlation (assessed by a linear model). (ZIP) [file pone.0167944.s006.zip › S1_File/L0012_real_dat.png]

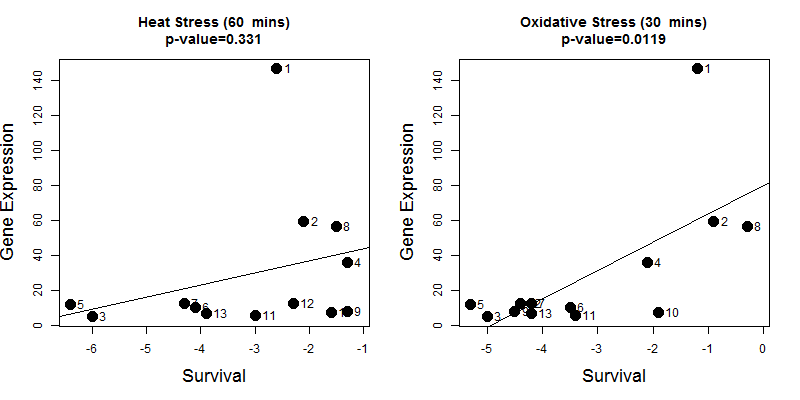

Supplement: S1 File — Expression levels of genes L0001 –L75633 plotted against survival after 60 minutes heat and 30 min oxidative stress. Survival is expressed as the difference of log CFU/ml after stress and before stress. Numbers indicate fermentations as presented in Table 1. P-values above the plots indicate significance of correlation (assessed by a linear model). (ZIP) [file pone.0167944.s006.zip › S1_File/L0013_real_dat.png]

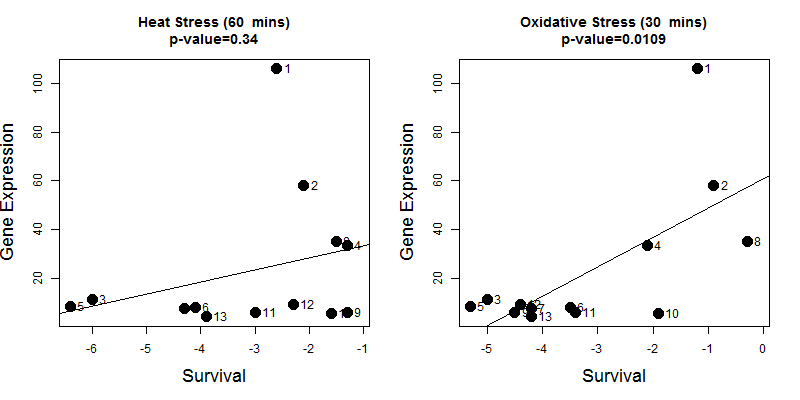

Supplement: S1 File — Expression levels of genes L0001 –L75633 plotted against survival after 60 minutes heat and 30 min oxidative stress. Survival is expressed as the difference of log CFU/ml after stress and before stress. Numbers indicate fermentations as presented in Table 1. P-values above the plots indicate significance of correlation (assessed by a linear model). (ZIP) [file pone.0167944.s006.zip › S1_File/L0014_real_dat.png]

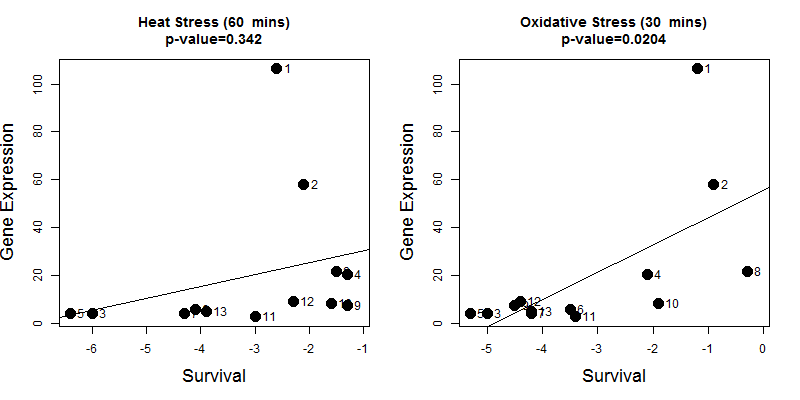

Supplement: S1 File — Expression levels of genes L0001 –L75633 plotted against survival after 60 minutes heat and 30 min oxidative stress. Survival is expressed as the difference of log CFU/ml after stress and before stress. Numbers indicate fermentations as presented in Table 1. P-values above the plots indicate significance of correlation (assessed by a linear model). (ZIP) [file pone.0167944.s006.zip › S1_File/L0015_real_dat.png]

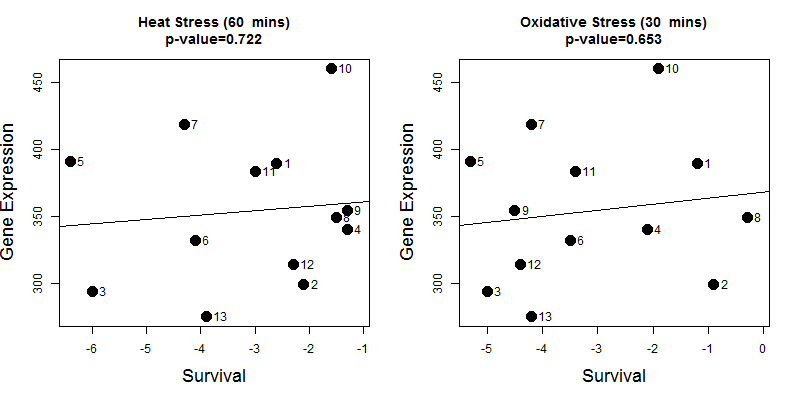

Supplement: S1 File — Expression levels of genes L0001 –L75633 plotted against survival after 60 minutes heat and 30 min oxidative stress. Survival is expressed as the difference of log CFU/ml after stress and before stress. Numbers indicate fermentations as presented in Table 1. P-values above the plots indicate significance of correlation (assessed by a linear model). (ZIP) [file pone.0167944.s006.zip › S1_File/L00157_real_dat.png]

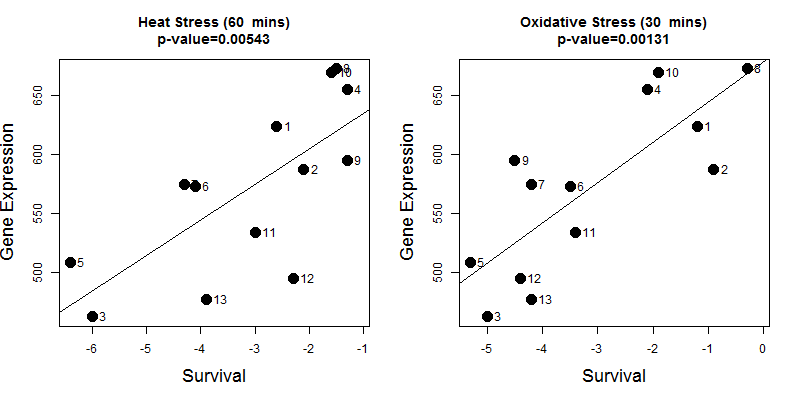

Supplement: S1 File — Expression levels of genes L0001 –L75633 plotted against survival after 60 minutes heat and 30 min oxidative stress. Survival is expressed as the difference of log CFU/ml after stress and before stress. Numbers indicate fermentations as presented in Table 1. P-values above the plots indicate significance of correlation (assessed by a linear model). (ZIP) [file pone.0167944.s006.zip › S1_File/L0016_real_dat.png]

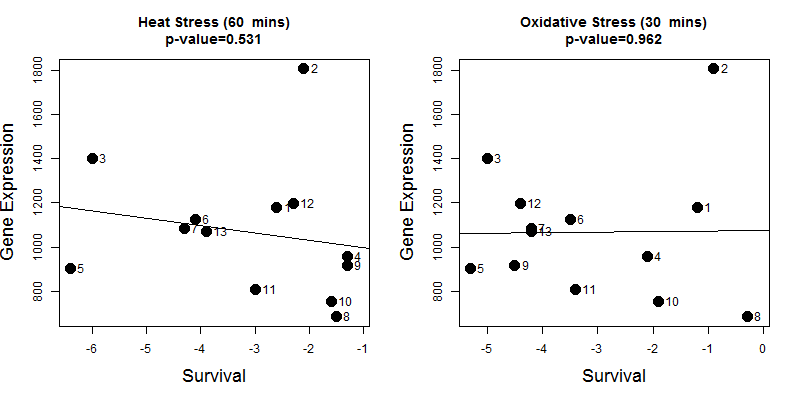

Supplement: S1 File — Expression levels of genes L0001 –L75633 plotted against survival after 60 minutes heat and 30 min oxidative stress. Survival is expressed as the difference of log CFU/ml after stress and before stress. Numbers indicate fermentations as presented in Table 1. P-values above the plots indicate significance of correlation (assessed by a linear model). (ZIP) [file pone.0167944.s006.zip › S1_File/L0017_real_dat.png]

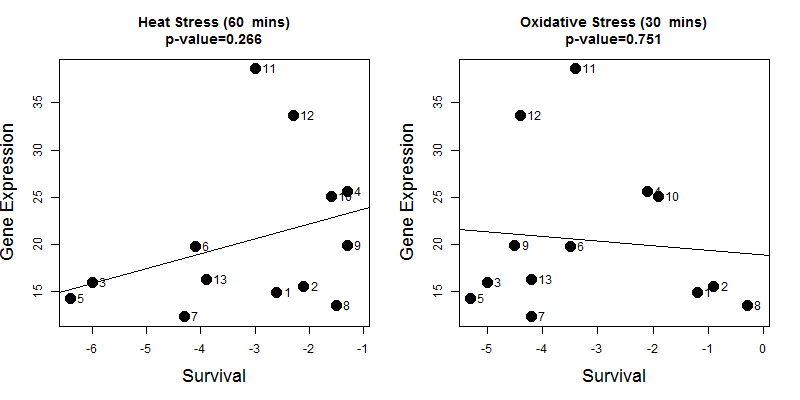

Supplement: S1 File — Expression levels of genes L0001 –L75633 plotted against survival after 60 minutes heat and 30 min oxidative stress. Survival is expressed as the difference of log CFU/ml after stress and before stress. Numbers indicate fermentations as presented in Table 1. P-values above the plots indicate significance of correlation (assessed by a linear model). (ZIP) [file pone.0167944.s006.zip › S1_File/L0018_real_dat.png]

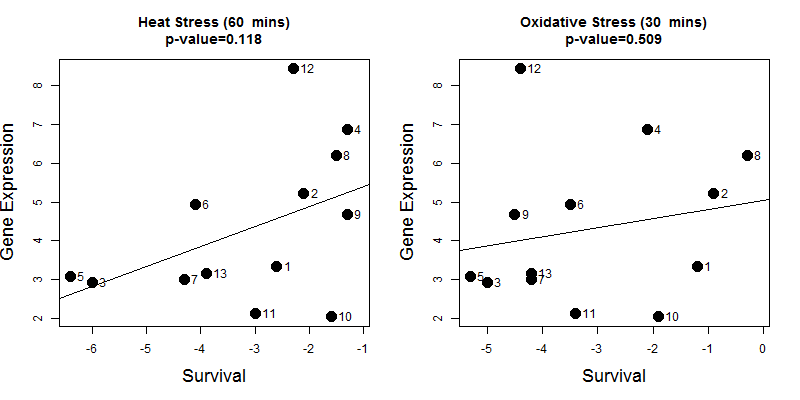

Supplement: S1 File — Expression levels of genes L0001 –L75633 plotted against survival after 60 minutes heat and 30 min oxidative stress. Survival is expressed as the difference of log CFU/ml after stress and before stress. Numbers indicate fermentations as presented in Table 1. P-values above the plots indicate significance of correlation (assessed by a linear model). (ZIP) [file pone.0167944.s006.zip › S1_File/L0019_real_dat.png]

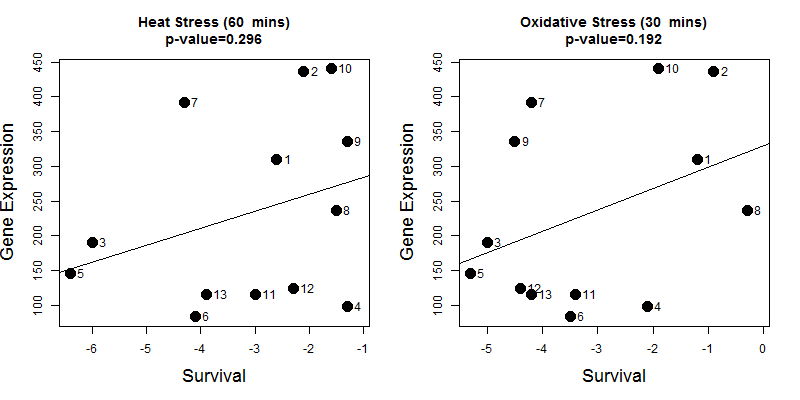

Supplement: S1 File — Expression levels of genes L0001 –L75633 plotted against survival after 60 minutes heat and 30 min oxidative stress. Survival is expressed as the difference of log CFU/ml after stress and before stress. Numbers indicate fermentations as presented in Table 1. P-values above the plots indicate significance of correlation (assessed by a linear model). (ZIP) [file pone.0167944.s006.zip › S1_File/L00196_real_dat.png]

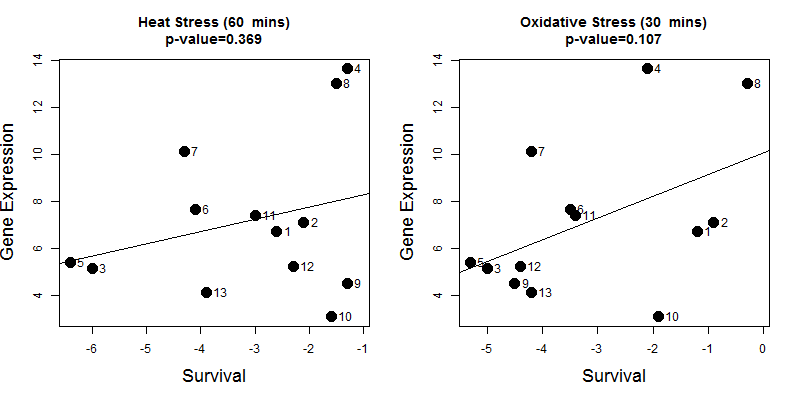

Supplement: S1 File — Expression levels of genes L0001 –L75633 plotted against survival after 60 minutes heat and 30 min oxidative stress. Survival is expressed as the difference of log CFU/ml after stress and before stress. Numbers indicate fermentations as presented in Table 1. P-values above the plots indicate significance of correlation (assessed by a linear model). (ZIP) [file pone.0167944.s006.zip › S1_File/L0020_real_dat.png]

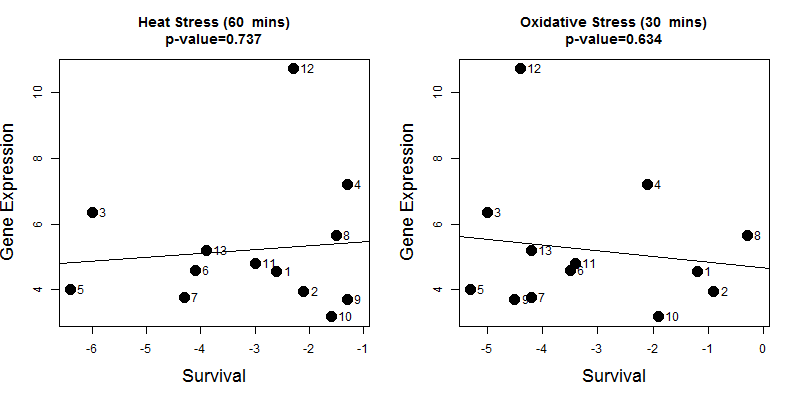

Supplement: S1 File — Expression levels of genes L0001 –L75633 plotted against survival after 60 minutes heat and 30 min oxidative stress. Survival is expressed as the difference of log CFU/ml after stress and before stress. Numbers indicate fermentations as presented in Table 1. P-values above the plots indicate significance of correlation (assessed by a linear model). (ZIP) [file pone.0167944.s006.zip › S1_File/L0021_real_dat.png]

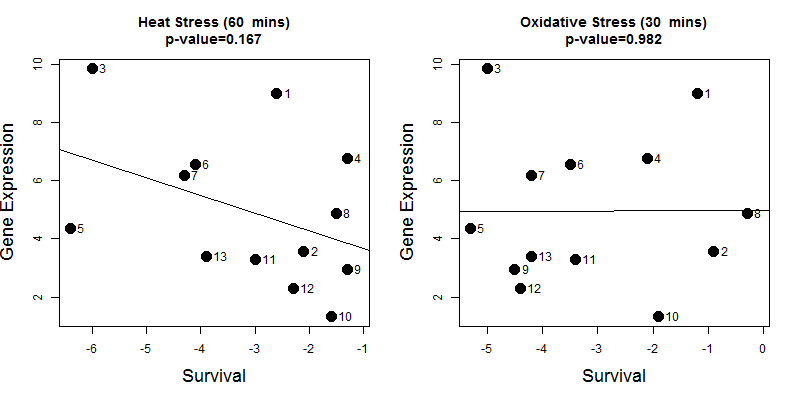

Supplement: S1 File — Expression levels of genes L0001 –L75633 plotted against survival after 60 minutes heat and 30 min oxidative stress. Survival is expressed as the difference of log CFU/ml after stress and before stress. Numbers indicate fermentations as presented in Table 1. P-values above the plots indicate significance of correlation (assessed by a linear model). (ZIP) [file pone.0167944.s006.zip › S1_File/L0022_real_dat.png]

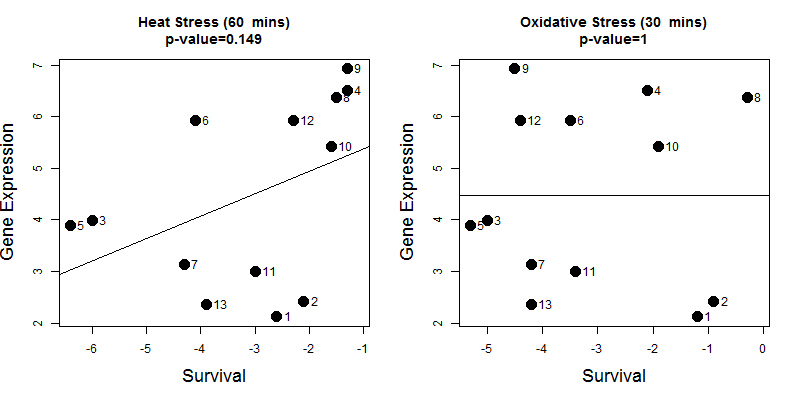

Supplement: S1 File — Expression levels of genes L0001 –L75633 plotted against survival after 60 minutes heat and 30 min oxidative stress. Survival is expressed as the difference of log CFU/ml after stress and before stress. Numbers indicate fermentations as presented in Table 1. P-values above the plots indicate significance of correlation (assessed by a linear model). (ZIP) [file pone.0167944.s006.zip › S1_File/L0023_real_dat.png]

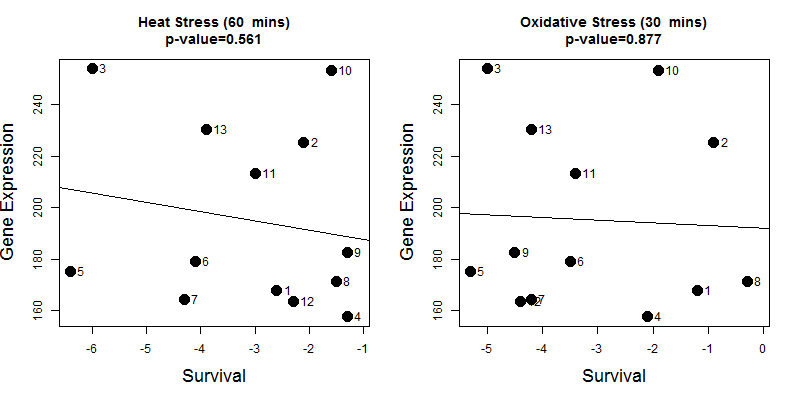

Supplement: S1 File — Expression levels of genes L0001 –L75633 plotted against survival after 60 minutes heat and 30 min oxidative stress. Survival is expressed as the difference of log CFU/ml after stress and before stress. Numbers indicate fermentations as presented in Table 1. P-values above the plots indicate significance of correlation (assessed by a linear model). (ZIP) [file pone.0167944.s006.zip › S1_File/L0024_real_dat.png]

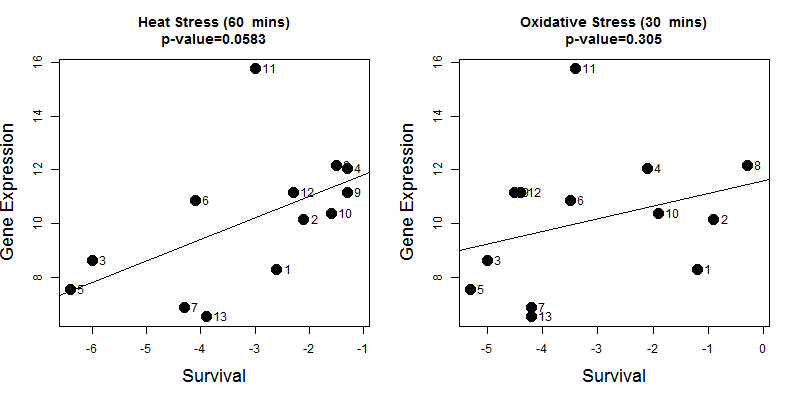

Supplement: S1 File — Expression levels of genes L0001 –L75633 plotted against survival after 60 minutes heat and 30 min oxidative stress. Survival is expressed as the difference of log CFU/ml after stress and before stress. Numbers indicate fermentations as presented in Table 1. P-values above the plots indicate significance of correlation (assessed by a linear model). (ZIP) [file pone.0167944.s006.zip › S1_File/L0025_real_dat.png]

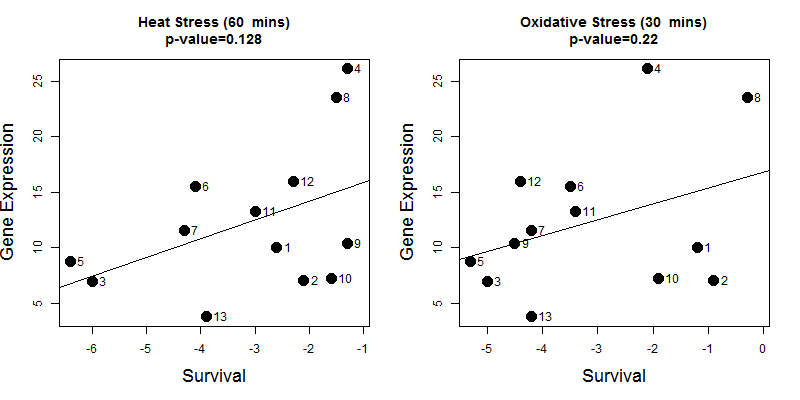

Supplement: S1 File — Expression levels of genes L0001 –L75633 plotted against survival after 60 minutes heat and 30 min oxidative stress. Survival is expressed as the difference of log CFU/ml after stress and before stress. Numbers indicate fermentations as presented in Table 1. P-values above the plots indicate significance of correlation (assessed by a linear model). (ZIP) [file pone.0167944.s006.zip › S1_File/L0026_real_dat.png]

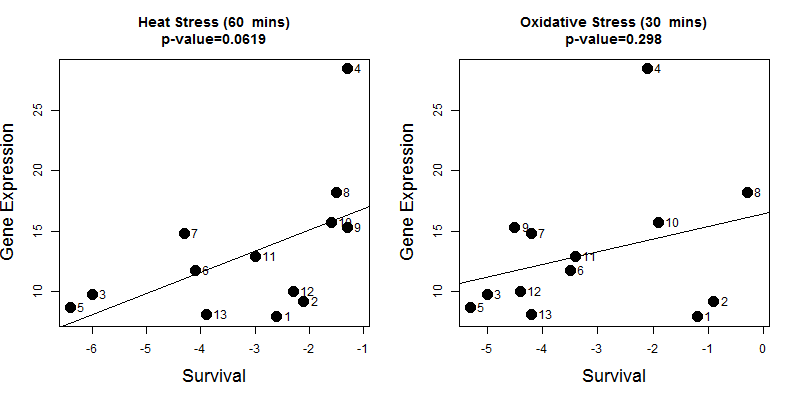

Supplement: S1 File — Expression levels of genes L0001 –L75633 plotted against survival after 60 minutes heat and 30 min oxidative stress. Survival is expressed as the difference of log CFU/ml after stress and before stress. Numbers indicate fermentations as presented in Table 1. P-values above the plots indicate significance of correlation (assessed by a linear model). (ZIP) [file pone.0167944.s006.zip › S1_File/L0027_real_dat.png]

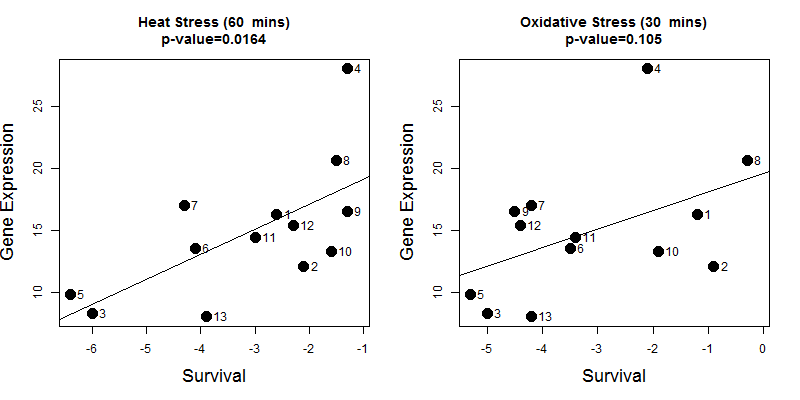

Supplement: S1 File — Expression levels of genes L0001 –L75633 plotted against survival after 60 minutes heat and 30 min oxidative stress. Survival is expressed as the difference of log CFU/ml after stress and before stress. Numbers indicate fermentations as presented in Table 1. P-values above the plots indicate significance of correlation (assessed by a linear model). (ZIP) [file pone.0167944.s006.zip › S1_File/L0028_real_dat.png]

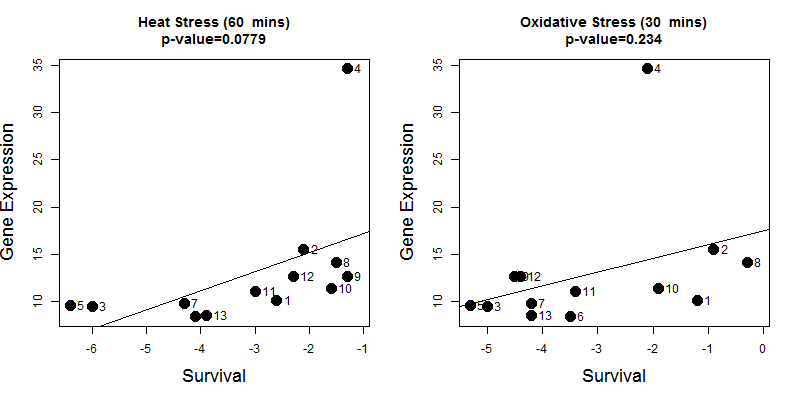

Supplement: S1 File — Expression levels of genes L0001 –L75633 plotted against survival after 60 minutes heat and 30 min oxidative stress. Survival is expressed as the difference of log CFU/ml after stress and before stress. Numbers indicate fermentations as presented in Table 1. P-values above the plots indicate significance of correlation (assessed by a linear model). (ZIP) [file pone.0167944.s006.zip › S1_File/L0029_real_dat.png]

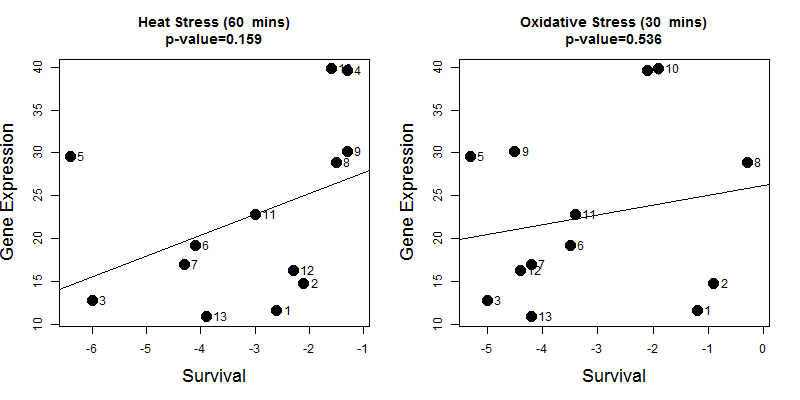

Supplement: S1 File — Expression levels of genes L0001 –L75633 plotted against survival after 60 minutes heat and 30 min oxidative stress. Survival is expressed as the difference of log CFU/ml after stress and before stress. Numbers indicate fermentations as presented in Table 1. P-values above the plots indicate significance of correlation (assessed by a linear model). (ZIP) [file pone.0167944.s006.zip › S1_File/L0031_real_dat.png]

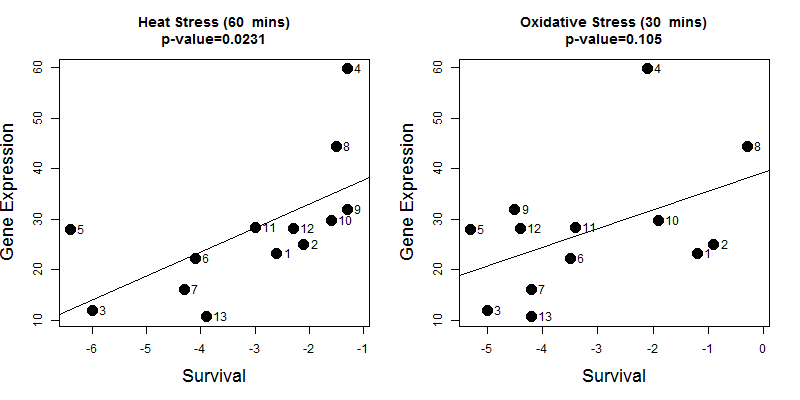

Supplement: S1 File — Expression levels of genes L0001 –L75633 plotted against survival after 60 minutes heat and 30 min oxidative stress. Survival is expressed as the difference of log CFU/ml after stress and before stress. Numbers indicate fermentations as presented in Table 1. P-values above the plots indicate significance of correlation (assessed by a linear model). (ZIP) [file pone.0167944.s006.zip › S1_File/L0032_real_dat.png]

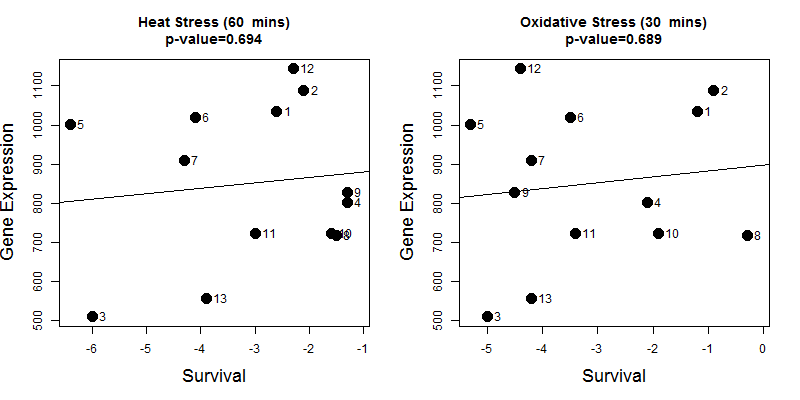

Supplement: S1 File — Expression levels of genes L0001 –L75633 plotted against survival after 60 minutes heat and 30 min oxidative stress. Survival is expressed as the difference of log CFU/ml after stress and before stress. Numbers indicate fermentations as presented in Table 1. P-values above the plots indicate significance of correlation (assessed by a linear model). (ZIP) [file pone.0167944.s006.zip › S1_File/L0033_real_dat.png]

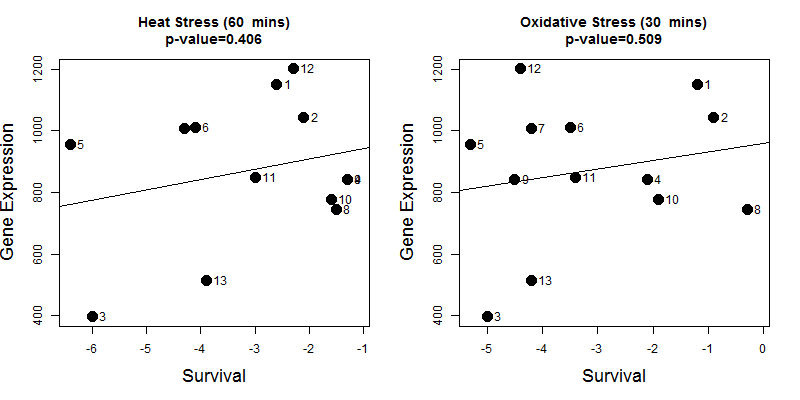

Supplement: S1 File — Expression levels of genes L0001 –L75633 plotted against survival after 60 minutes heat and 30 min oxidative stress. Survival is expressed as the difference of log CFU/ml after stress and before stress. Numbers indicate fermentations as presented in Table 1. P-values above the plots indicate significance of correlation (assessed by a linear model). (ZIP) [file pone.0167944.s006.zip › S1_File/L0034_real_dat.png]

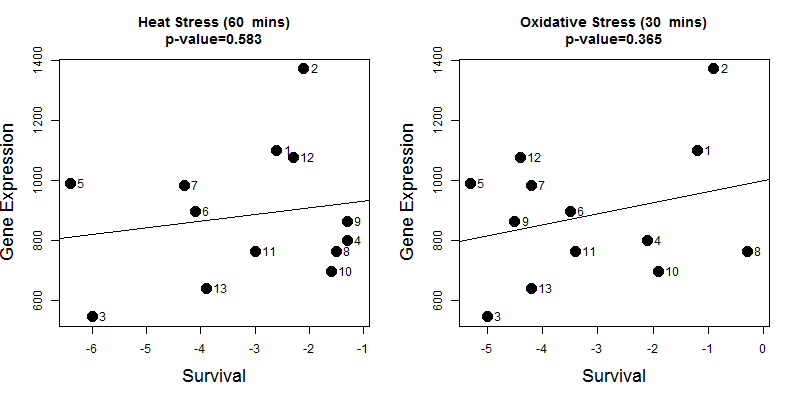

Supplement: S1 File — Expression levels of genes L0001 –L75633 plotted against survival after 60 minutes heat and 30 min oxidative stress. Survival is expressed as the difference of log CFU/ml after stress and before stress. Numbers indicate fermentations as presented in Table 1. P-values above the plots indicate significance of correlation (assessed by a linear model). (ZIP) [file pone.0167944.s006.zip › S1_File/L0035_real_dat.png]

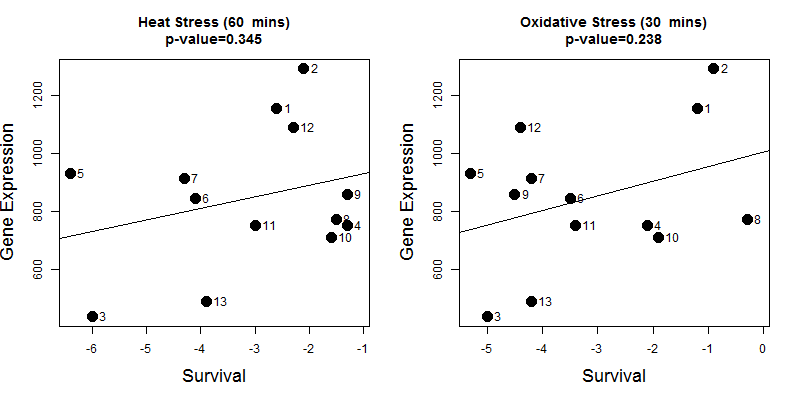

Supplement: S1 File — Expression levels of genes L0001 –L75633 plotted against survival after 60 minutes heat and 30 min oxidative stress. Survival is expressed as the difference of log CFU/ml after stress and before stress. Numbers indicate fermentations as presented in Table 1. P-values above the plots indicate significance of correlation (assessed by a linear model). (ZIP) [file pone.0167944.s006.zip › S1_File/L0036_real_dat.png]

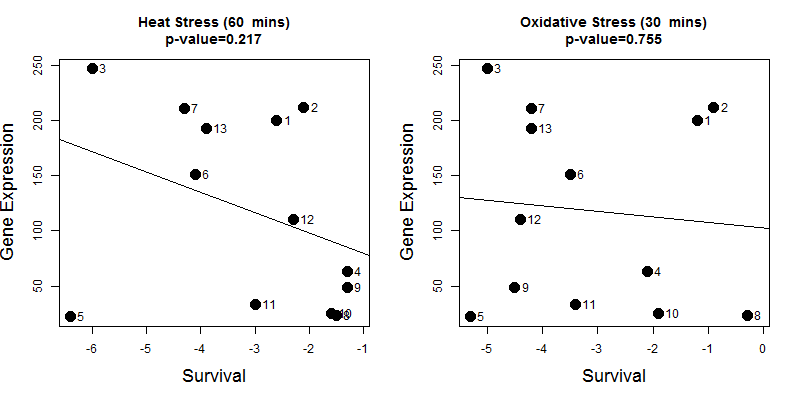

Supplement: S1 File — Expression levels of genes L0001 –L75633 plotted against survival after 60 minutes heat and 30 min oxidative stress. Survival is expressed as the difference of log CFU/ml after stress and before stress. Numbers indicate fermentations as presented in Table 1. P-values above the plots indicate significance of correlation (assessed by a linear model). (ZIP) [file pone.0167944.s006.zip › S1_File/L0037_real_dat.png]

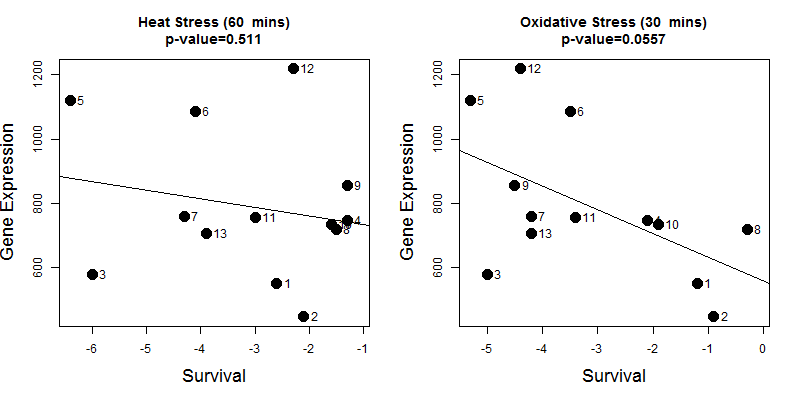

Supplement: S1 File — Expression levels of genes L0001 –L75633 plotted against survival after 60 minutes heat and 30 min oxidative stress. Survival is expressed as the difference of log CFU/ml after stress and before stress. Numbers indicate fermentations as presented in Table 1. P-values above the plots indicate significance of correlation (assessed by a linear model). (ZIP) [file pone.0167944.s006.zip › S1_File/L0038_real_dat.png]

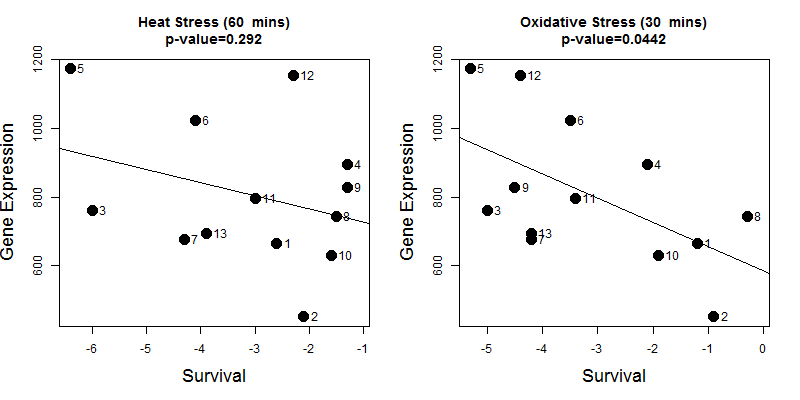

Supplement: S1 File — Expression levels of genes L0001 –L75633 plotted against survival after 60 minutes heat and 30 min oxidative stress. Survival is expressed as the difference of log CFU/ml after stress and before stress. Numbers indicate fermentations as presented in Table 1. P-values above the plots indicate significance of correlation (assessed by a linear model). (ZIP) [file pone.0167944.s006.zip › S1_File/L0039_real_dat.png]

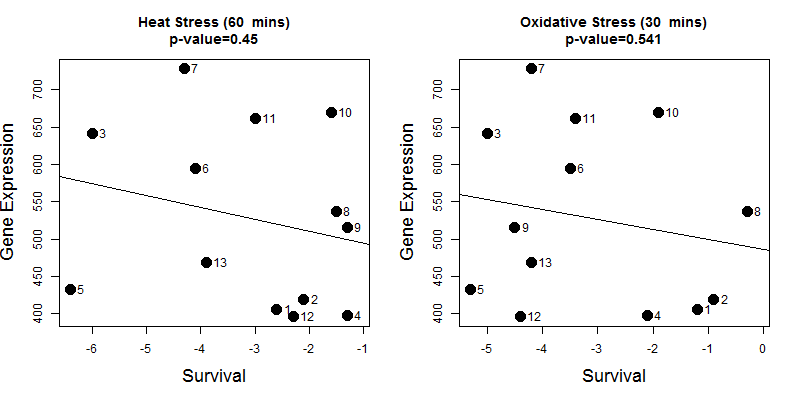

Supplement: S1 File — Expression levels of genes L0001 –L75633 plotted against survival after 60 minutes heat and 30 min oxidative stress. Survival is expressed as the difference of log CFU/ml after stress and before stress. Numbers indicate fermentations as presented in Table 1. P-values above the plots indicate significance of correlation (assessed by a linear model). (ZIP) [file pone.0167944.s006.zip › S1_File/L00396_real_dat.png]

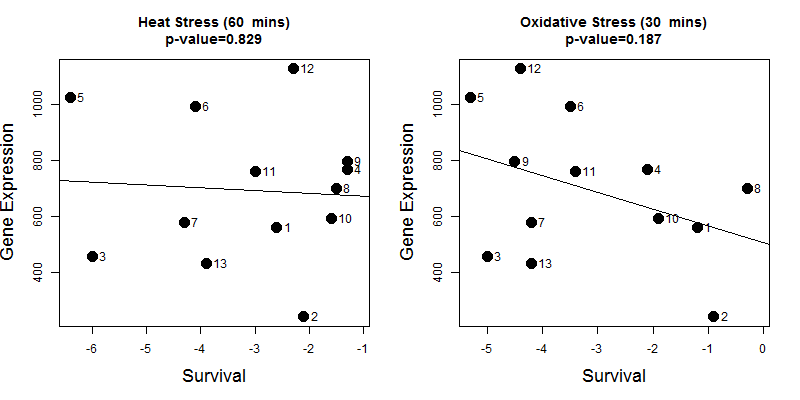

Supplement: S1 File — Expression levels of genes L0001 –L75633 plotted against survival after 60 minutes heat and 30 min oxidative stress. Survival is expressed as the difference of log CFU/ml after stress and before stress. Numbers indicate fermentations as presented in Table 1. P-values above the plots indicate significance of correlation (assessed by a linear model). (ZIP) [file pone.0167944.s006.zip › S1_File/L0040_real_dat.png]

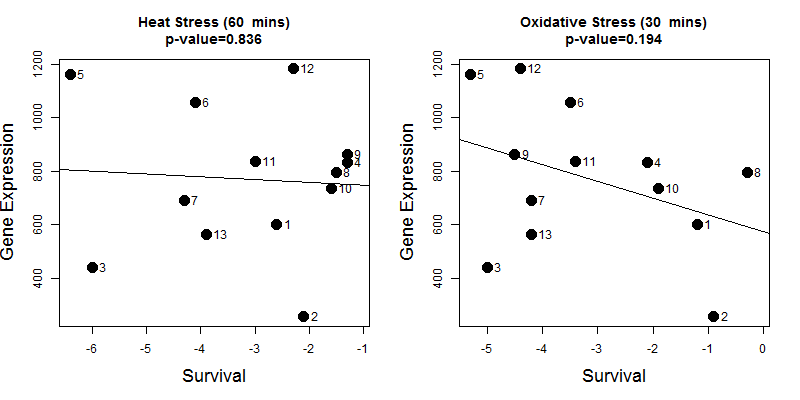

Supplement: S1 File — Expression levels of genes L0001 –L75633 plotted against survival after 60 minutes heat and 30 min oxidative stress. Survival is expressed as the difference of log CFU/ml after stress and before stress. Numbers indicate fermentations as presented in Table 1. P-values above the plots indicate significance of correlation (assessed by a linear model). (ZIP) [file pone.0167944.s006.zip › S1_File/L0041_real_dat.png]

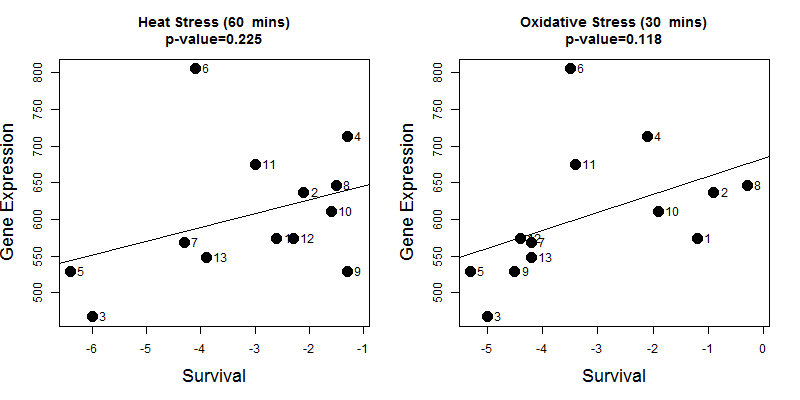

Supplement: S1 File — Expression levels of genes L0001 –L75633 plotted against survival after 60 minutes heat and 30 min oxidative stress. Survival is expressed as the difference of log CFU/ml after stress and before stress. Numbers indicate fermentations as presented in Table 1. P-values above the plots indicate significance of correlation (assessed by a linear model). (ZIP) [file pone.0167944.s006.zip › S1_File/L0043_real_dat.png]

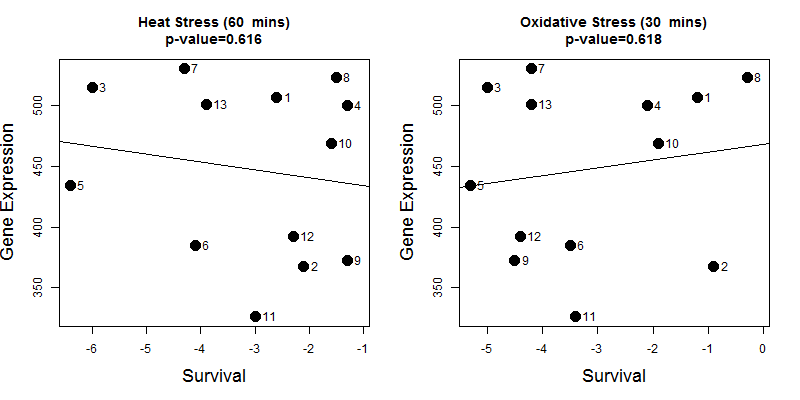

Supplement: S1 File — Expression levels of genes L0001 –L75633 plotted against survival after 60 minutes heat and 30 min oxidative stress. Survival is expressed as the difference of log CFU/ml after stress and before stress. Numbers indicate fermentations as presented in Table 1. P-values above the plots indicate significance of correlation (assessed by a linear model). (ZIP) [file pone.0167944.s006.zip › S1_File/L0044_real_dat.png]

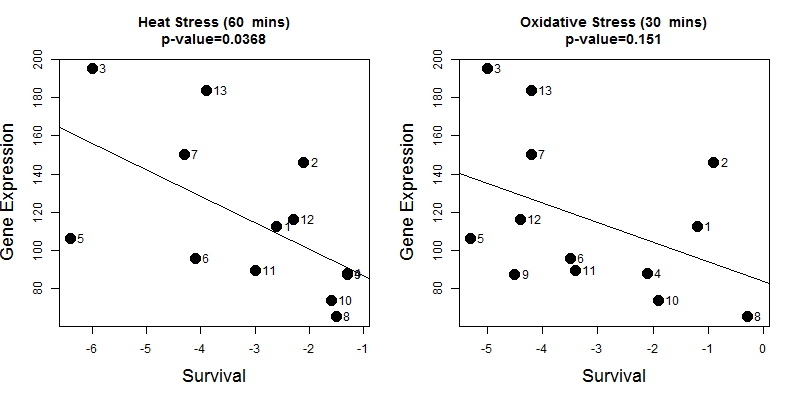

Supplement: S1 File — Expression levels of genes L0001 –L75633 plotted against survival after 60 minutes heat and 30 min oxidative stress. Survival is expressed as the difference of log CFU/ml after stress and before stress. Numbers indicate fermentations as presented in Table 1. P-values above the plots indicate significance of correlation (assessed by a linear model). (ZIP) [file pone.0167944.s006.zip › S1_File/L0045_real_dat.png]

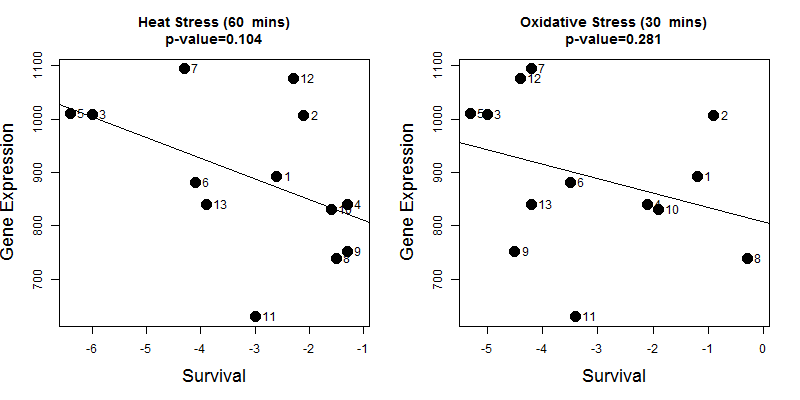

Supplement: S1 File — Expression levels of genes L0001 –L75633 plotted against survival after 60 minutes heat and 30 min oxidative stress. Survival is expressed as the difference of log CFU/ml after stress and before stress. Numbers indicate fermentations as presented in Table 1. P-values above the plots indicate significance of correlation (assessed by a linear model). (ZIP) [file pone.0167944.s006.zip › S1_File/L00457_real_dat.png]

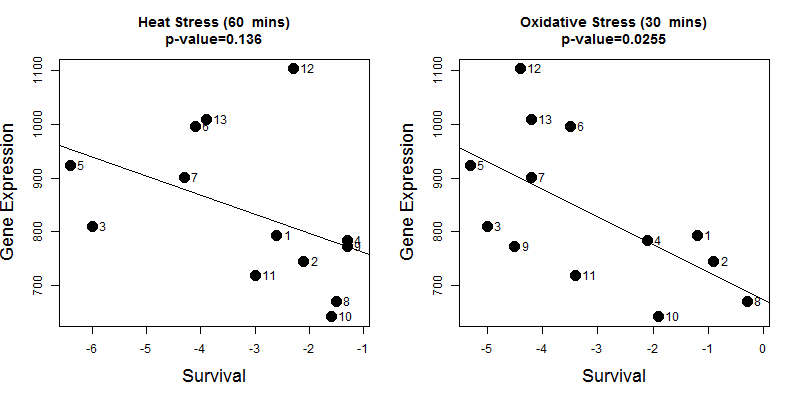

Supplement: S1 File — Expression levels of genes L0001 –L75633 plotted against survival after 60 minutes heat and 30 min oxidative stress. Survival is expressed as the difference of log CFU/ml after stress and before stress. Numbers indicate fermentations as presented in Table 1. P-values above the plots indicate significance of correlation (assessed by a linear model). (ZIP) [file pone.0167944.s006.zip › S1_File/L0046_real_dat.png]

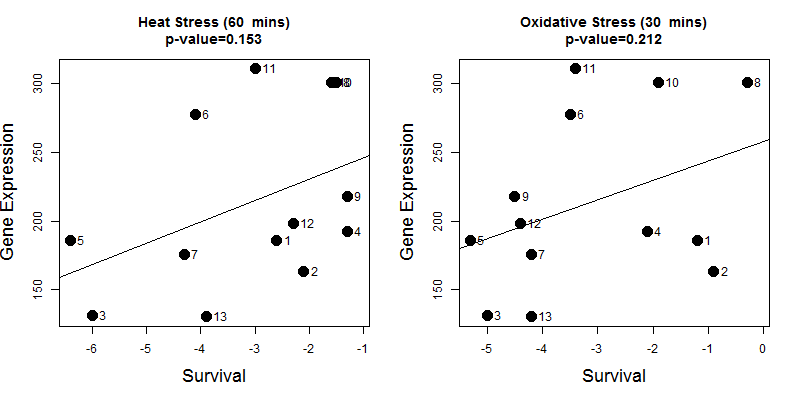

Supplement: S1 File — Expression levels of genes L0001 –L75633 plotted against survival after 60 minutes heat and 30 min oxidative stress. Survival is expressed as the difference of log CFU/ml after stress and before stress. Numbers indicate fermentations as presented in Table 1. P-values above the plots indicate significance of correlation (assessed by a linear model). (ZIP) [file pone.0167944.s006.zip › S1_File/L0047_real_dat.png]

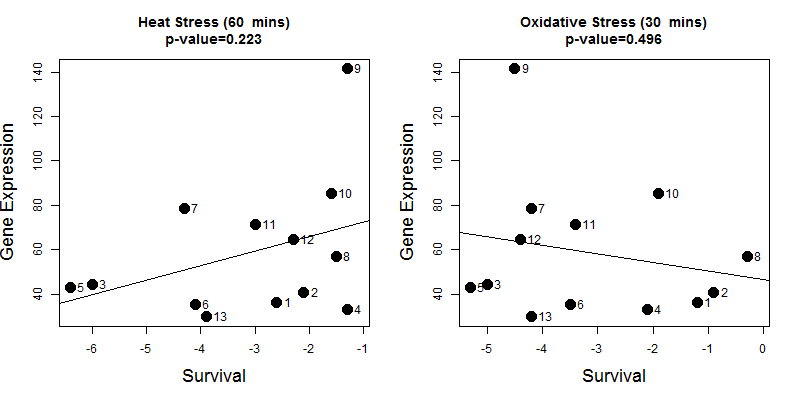

Supplement: S1 File — Expression levels of genes L0001 –L75633 plotted against survival after 60 minutes heat and 30 min oxidative stress. Survival is expressed as the difference of log CFU/ml after stress and before stress. Numbers indicate fermentations as presented in Table 1. P-values above the plots indicate significance of correlation (assessed by a linear model). (ZIP) [file pone.0167944.s006.zip › S1_File/L0048_real_dat.png]

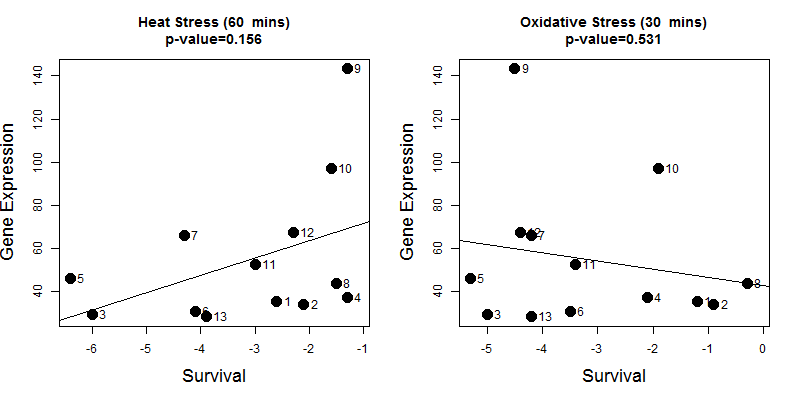

Supplement: S1 File — Expression levels of genes L0001 –L75633 plotted against survival after 60 minutes heat and 30 min oxidative stress. Survival is expressed as the difference of log CFU/ml after stress and before stress. Numbers indicate fermentations as presented in Table 1. P-values above the plots indicate significance of correlation (assessed by a linear model). (ZIP) [file pone.0167944.s006.zip › S1_File/L0049_real_dat.png]

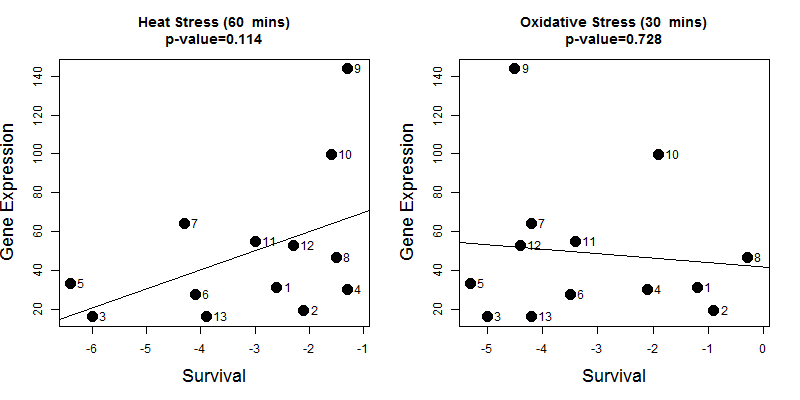

Supplement: S1 File — Expression levels of genes L0001 –L75633 plotted against survival after 60 minutes heat and 30 min oxidative stress. Survival is expressed as the difference of log CFU/ml after stress and before stress. Numbers indicate fermentations as presented in Table 1. P-values above the plots indicate significance of correlation (assessed by a linear model). (ZIP) [file pone.0167944.s006.zip › S1_File/L0050_real_dat.png]

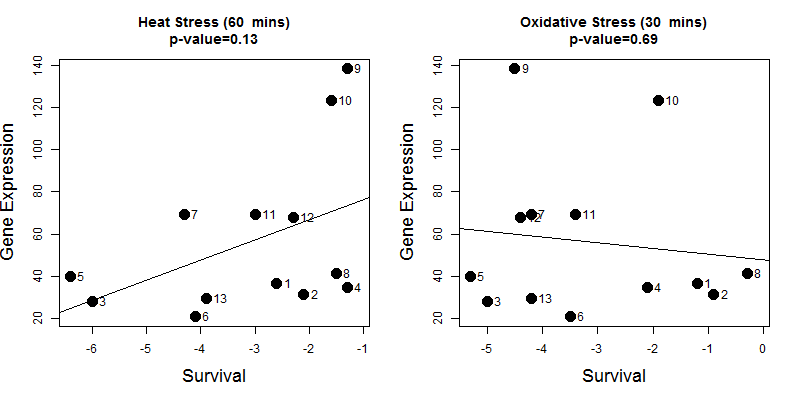

Supplement: S1 File — Expression levels of genes L0001 –L75633 plotted against survival after 60 minutes heat and 30 min oxidative stress. Survival is expressed as the difference of log CFU/ml after stress and before stress. Numbers indicate fermentations as presented in Table 1. P-values above the plots indicate significance of correlation (assessed by a linear model). (ZIP) [file pone.0167944.s006.zip › S1_File/L0051_real_dat.png]

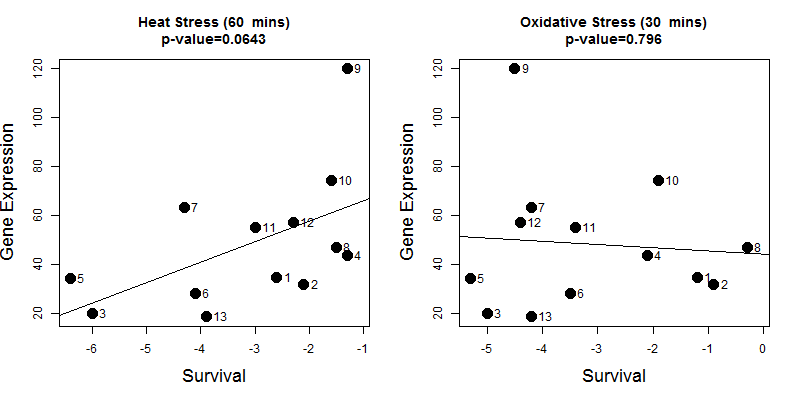

Supplement: S1 File — Expression levels of genes L0001 –L75633 plotted against survival after 60 minutes heat and 30 min oxidative stress. Survival is expressed as the difference of log CFU/ml after stress and before stress. Numbers indicate fermentations as presented in Table 1. P-values above the plots indicate significance of correlation (assessed by a linear model). (ZIP) [file pone.0167944.s006.zip › S1_File/L0052_real_dat.png]

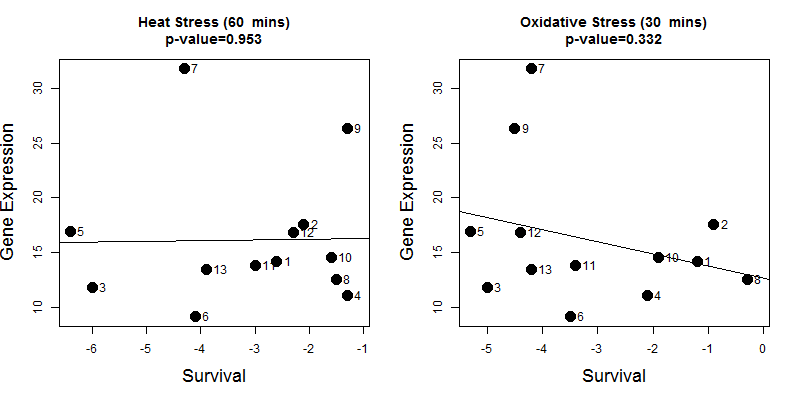

Supplement: S1 File — Expression levels of genes L0001 –L75633 plotted against survival after 60 minutes heat and 30 min oxidative stress. Survival is expressed as the difference of log CFU/ml after stress and before stress. Numbers indicate fermentations as presented in Table 1. P-values above the plots indicate significance of correlation (assessed by a linear model). (ZIP) [file pone.0167944.s006.zip › S1_File/L0053_real_dat.png]

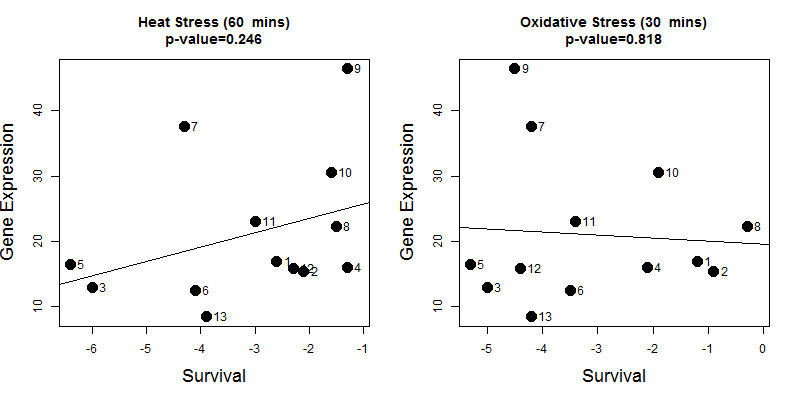

Supplement: S1 File — Expression levels of genes L0001 –L75633 plotted against survival after 60 minutes heat and 30 min oxidative stress. Survival is expressed as the difference of log CFU/ml after stress and before stress. Numbers indicate fermentations as presented in Table 1. P-values above the plots indicate significance of correlation (assessed by a linear model). (ZIP) [file pone.0167944.s006.zip › S1_File/L0054_real_dat.png]

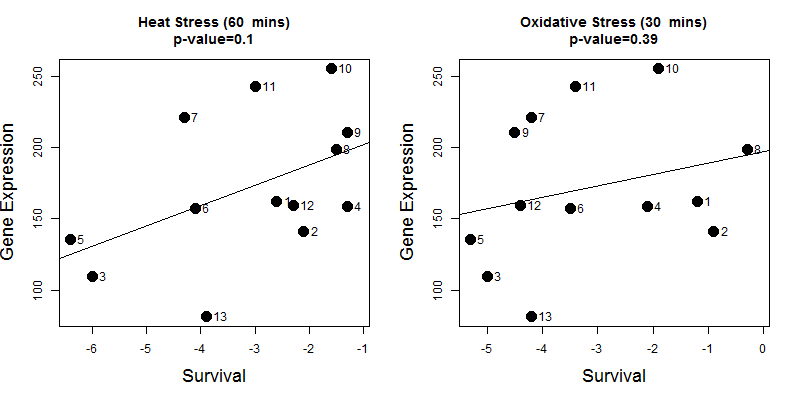

Supplement: S1 File — Expression levels of genes L0001 –L75633 plotted against survival after 60 minutes heat and 30 min oxidative stress. Survival is expressed as the difference of log CFU/ml after stress and before stress. Numbers indicate fermentations as presented in Table 1. P-values above the plots indicate significance of correlation (assessed by a linear model). (ZIP) [file pone.0167944.s006.zip › S1_File/L0055_real_dat.png]

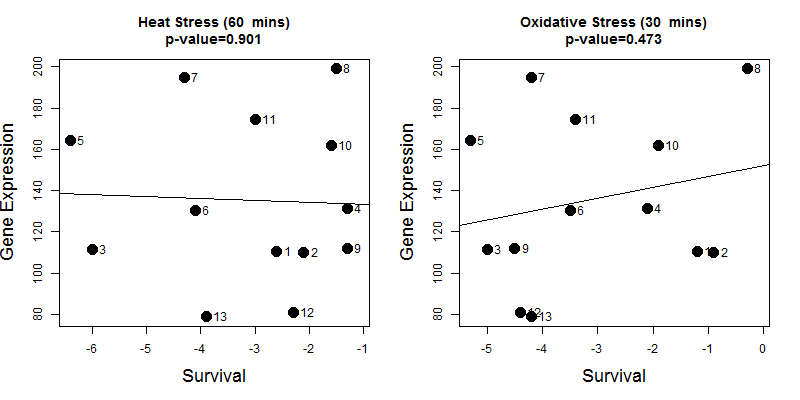

Supplement: S1 File — Expression levels of genes L0001 –L75633 plotted against survival after 60 minutes heat and 30 min oxidative stress. Survival is expressed as the difference of log CFU/ml after stress and before stress. Numbers indicate fermentations as presented in Table 1. P-values above the plots indicate significance of correlation (assessed by a linear model). (ZIP) [file pone.0167944.s006.zip › S1_File/L0056_real_dat.png]

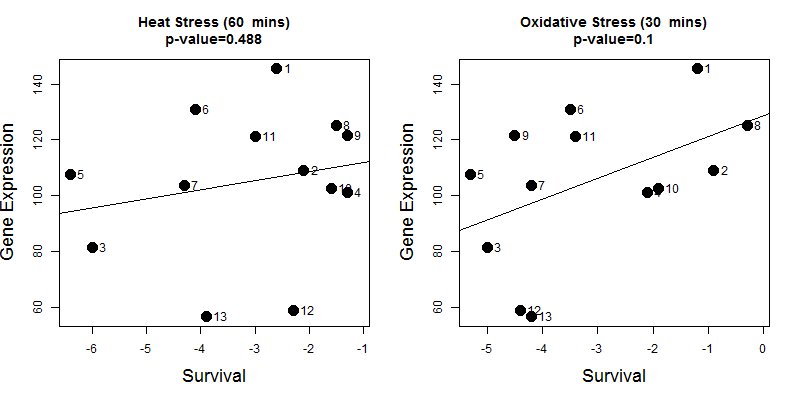

Supplement: S1 File — Expression levels of genes L0001 –L75633 plotted against survival after 60 minutes heat and 30 min oxidative stress. Survival is expressed as the difference of log CFU/ml after stress and before stress. Numbers indicate fermentations as presented in Table 1. P-values above the plots indicate significance of correlation (assessed by a linear model). (ZIP) [file pone.0167944.s006.zip › S1_File/L0057_real_dat.png]

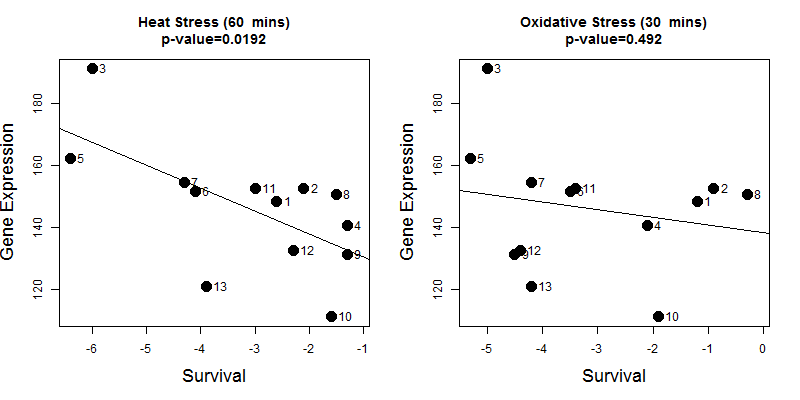

Supplement: S1 File — Expression levels of genes L0001 –L75633 plotted against survival after 60 minutes heat and 30 min oxidative stress. Survival is expressed as the difference of log CFU/ml after stress and before stress. Numbers indicate fermentations as presented in Table 1. P-values above the plots indicate significance of correlation (assessed by a linear model). (ZIP) [file pone.0167944.s006.zip › S1_File/L0058_real_dat.png]

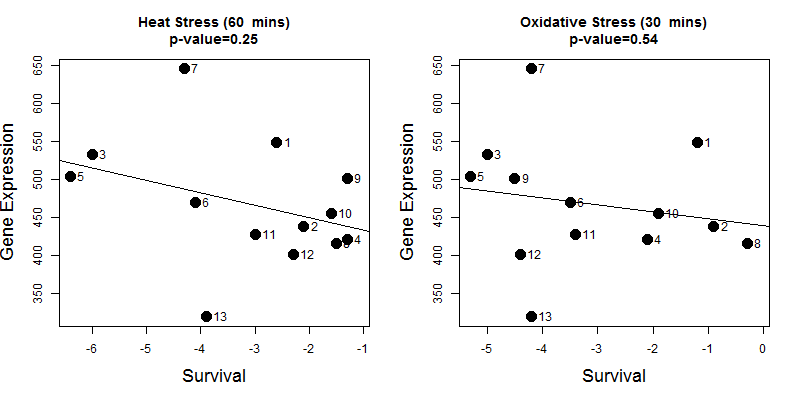

Supplement: S1 File — Expression levels of genes L0001 –L75633 plotted against survival after 60 minutes heat and 30 min oxidative stress. Survival is expressed as the difference of log CFU/ml after stress and before stress. Numbers indicate fermentations as presented in Table 1. P-values above the plots indicate significance of correlation (assessed by a linear model). (ZIP) [file pone.0167944.s006.zip › S1_File/L0059_real_dat.png]

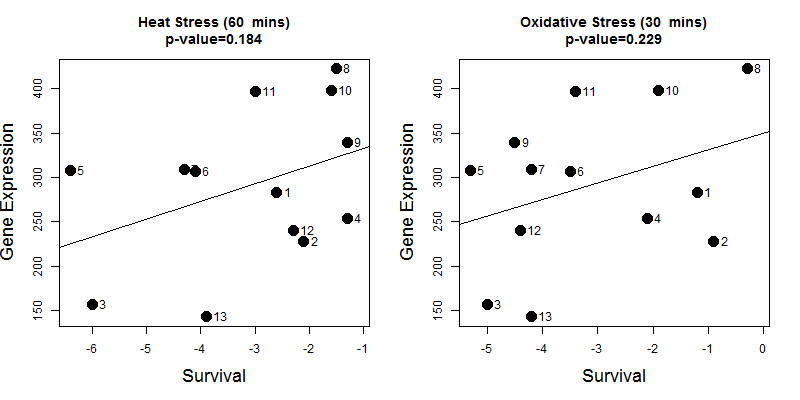

Supplement: S1 File — Expression levels of genes L0001 –L75633 plotted against survival after 60 minutes heat and 30 min oxidative stress. Survival is expressed as the difference of log CFU/ml after stress and before stress. Numbers indicate fermentations as presented in Table 1. P-values above the plots indicate significance of correlation (assessed by a linear model). (ZIP) [file pone.0167944.s006.zip › S1_File/L0060_real_dat.png]

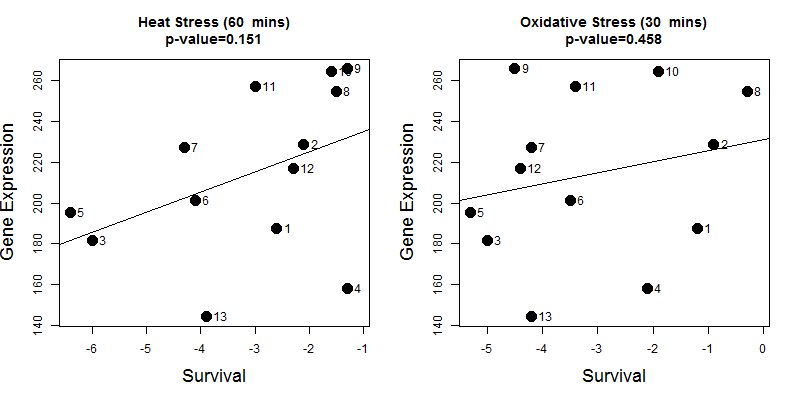

Supplement: S1 File — Expression levels of genes L0001 –L75633 plotted against survival after 60 minutes heat and 30 min oxidative stress. Survival is expressed as the difference of log CFU/ml after stress and before stress. Numbers indicate fermentations as presented in Table 1. P-values above the plots indicate significance of correlation (assessed by a linear model). (ZIP) [file pone.0167944.s006.zip › S1_File/L0061_real_dat.png]

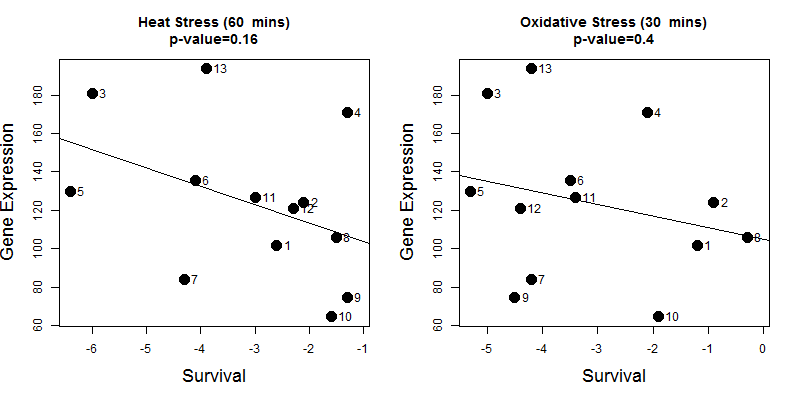

Supplement: S1 File — Expression levels of genes L0001 –L75633 plotted against survival after 60 minutes heat and 30 min oxidative stress. Survival is expressed as the difference of log CFU/ml after stress and before stress. Numbers indicate fermentations as presented in Table 1. P-values above the plots indicate significance of correlation (assessed by a linear model). (ZIP) [file pone.0167944.s006.zip › S1_File/L0062_real_dat.png]

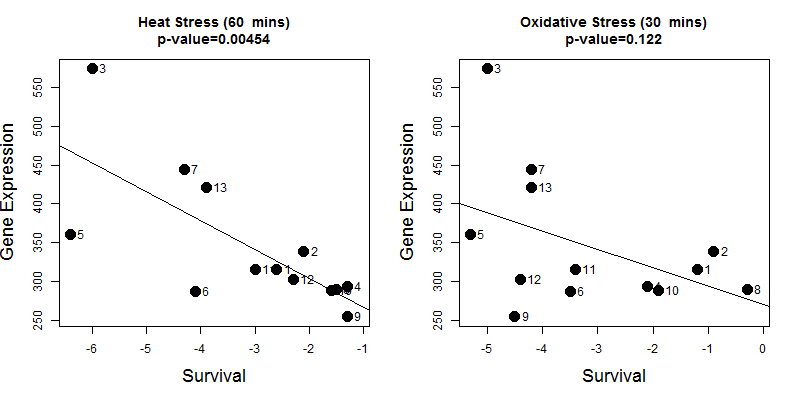

Supplement: S1 File — Expression levels of genes L0001 –L75633 plotted against survival after 60 minutes heat and 30 min oxidative stress. Survival is expressed as the difference of log CFU/ml after stress and before stress. Numbers indicate fermentations as presented in Table 1. P-values above the plots indicate significance of correlation (assessed by a linear model). (ZIP) [file pone.0167944.s006.zip › S1_File/L0063_real_dat.png]

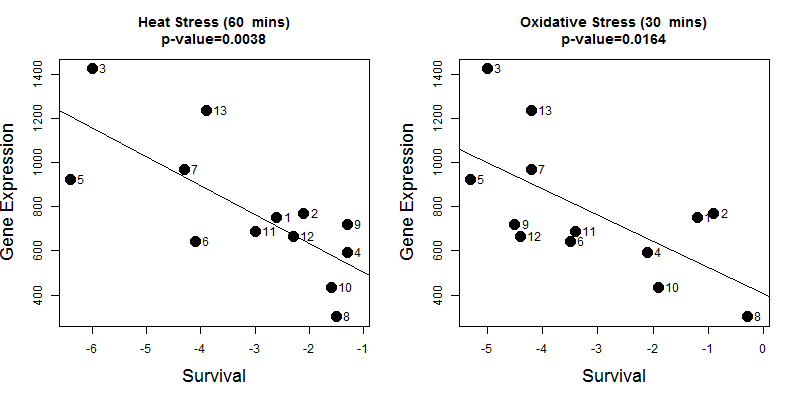

Supplement: S1 File — Expression levels of genes L0001 –L75633 plotted against survival after 60 minutes heat and 30 min oxidative stress. Survival is expressed as the difference of log CFU/ml after stress and before stress. Numbers indicate fermentations as presented in Table 1. P-values above the plots indicate significance of correlation (assessed by a linear model). (ZIP) [file pone.0167944.s006.zip › S1_File/L0064_real_dat.png]

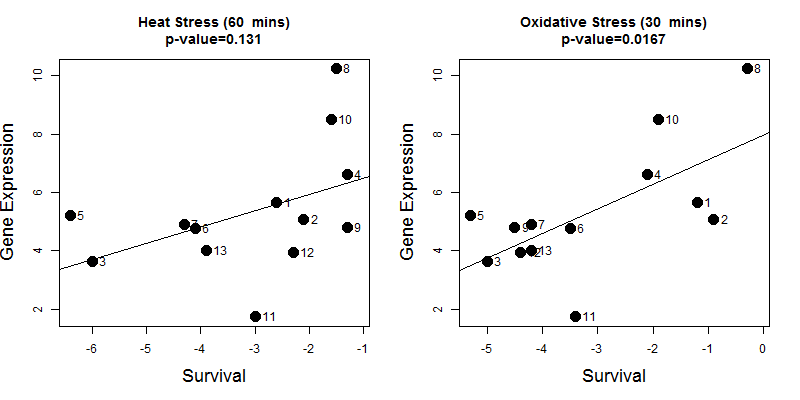

Supplement: S1 File — Expression levels of genes L0001 –L75633 plotted against survival after 60 minutes heat and 30 min oxidative stress. Survival is expressed as the difference of log CFU/ml after stress and before stress. Numbers indicate fermentations as presented in Table 1. P-values above the plots indicate significance of correlation (assessed by a linear model). (ZIP) [file pone.0167944.s006.zip › S1_File/L0065_real_dat.png]

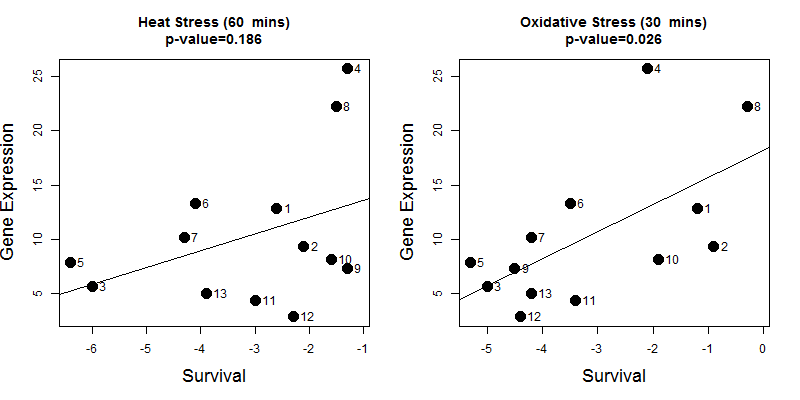

Supplement: S1 File — Expression levels of genes L0001 –L75633 plotted against survival after 60 minutes heat and 30 min oxidative stress. Survival is expressed as the difference of log CFU/ml after stress and before stress. Numbers indicate fermentations as presented in Table 1. P-values above the plots indicate significance of correlation (assessed by a linear model). (ZIP) [file pone.0167944.s006.zip › S1_File/L0066_real_dat.png]

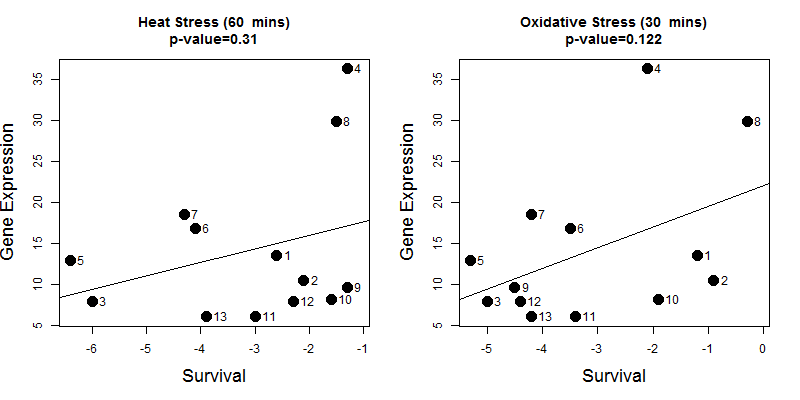

Supplement: S1 File — Expression levels of genes L0001 –L75633 plotted against survival after 60 minutes heat and 30 min oxidative stress. Survival is expressed as the difference of log CFU/ml after stress and before stress. Numbers indicate fermentations as presented in Table 1. P-values above the plots indicate significance of correlation (assessed by a linear model). (ZIP) [file pone.0167944.s006.zip › S1_File/L0067_real_dat.png]

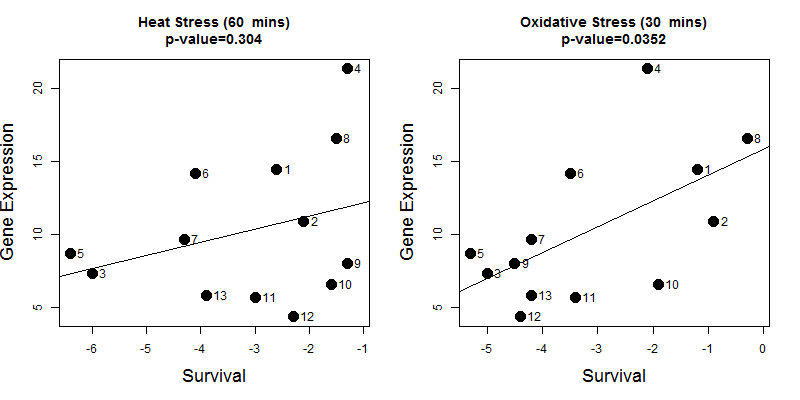

Supplement: S1 File — Expression levels of genes L0001 –L75633 plotted against survival after 60 minutes heat and 30 min oxidative stress. Survival is expressed as the difference of log CFU/ml after stress and before stress. Numbers indicate fermentations as presented in Table 1. P-values above the plots indicate significance of correlation (assessed by a linear model). (ZIP) [file pone.0167944.s006.zip › S1_File/L0068_real_dat.png]

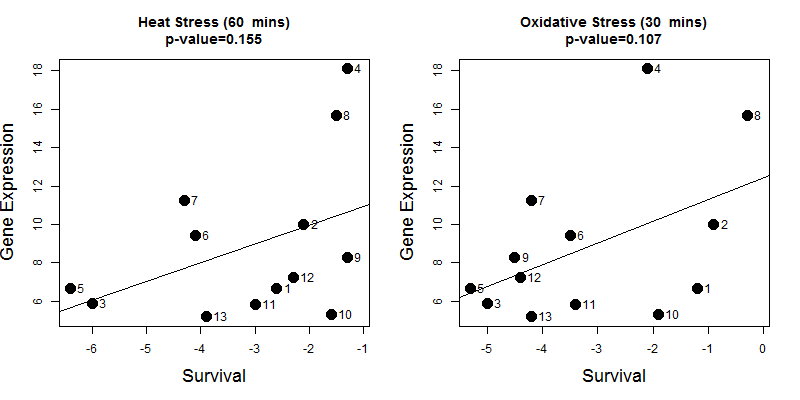

Supplement: S1 File — Expression levels of genes L0001 –L75633 plotted against survival after 60 minutes heat and 30 min oxidative stress. Survival is expressed as the difference of log CFU/ml after stress and before stress. Numbers indicate fermentations as presented in Table 1. P-values above the plots indicate significance of correlation (assessed by a linear model). (ZIP) [file pone.0167944.s006.zip › S1_File/L0069_real_dat.png]

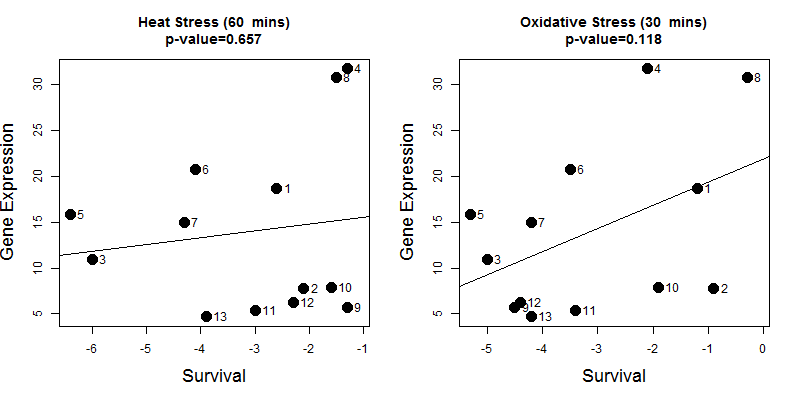

Supplement: S1 File — Expression levels of genes L0001 –L75633 plotted against survival after 60 minutes heat and 30 min oxidative stress. Survival is expressed as the difference of log CFU/ml after stress and before stress. Numbers indicate fermentations as presented in Table 1. P-values above the plots indicate significance of correlation (assessed by a linear model). (ZIP) [file pone.0167944.s006.zip › S1_File/L0070_real_dat.png]

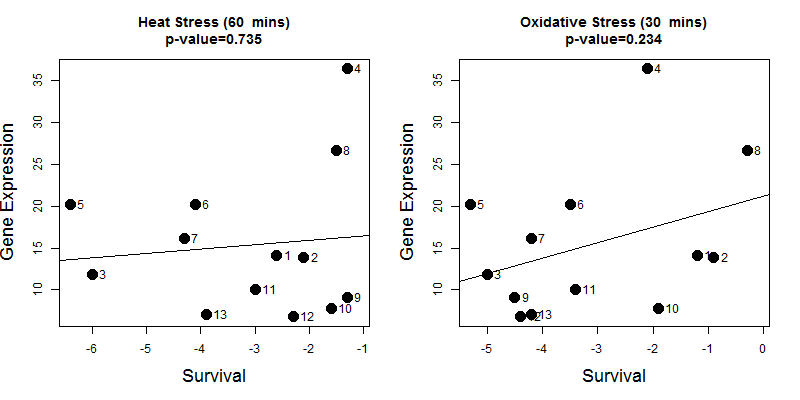

Supplement: S1 File — Expression levels of genes L0001 –L75633 plotted against survival after 60 minutes heat and 30 min oxidative stress. Survival is expressed as the difference of log CFU/ml after stress and before stress. Numbers indicate fermentations as presented in Table 1. P-values above the plots indicate significance of correlation (assessed by a linear model). (ZIP) [file pone.0167944.s006.zip › S1_File/L0071_real_dat.png]

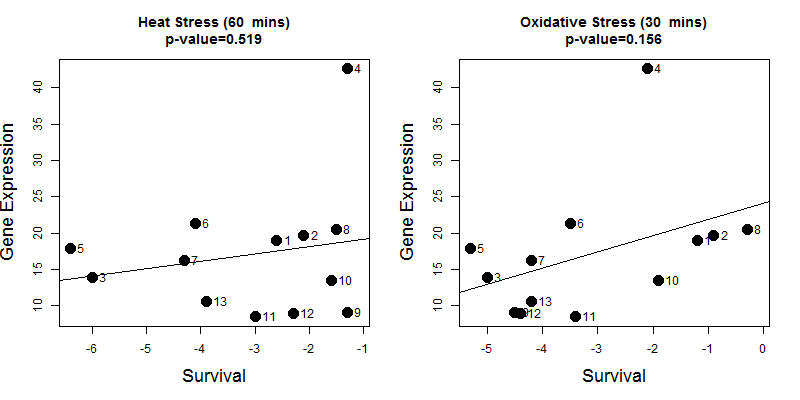

Supplement: S1 File — Expression levels of genes L0001 –L75633 plotted against survival after 60 minutes heat and 30 min oxidative stress. Survival is expressed as the difference of log CFU/ml after stress and before stress. Numbers indicate fermentations as presented in Table 1. P-values above the plots indicate significance of correlation (assessed by a linear model). (ZIP) [file pone.0167944.s006.zip › S1_File/L0072_real_dat.png]

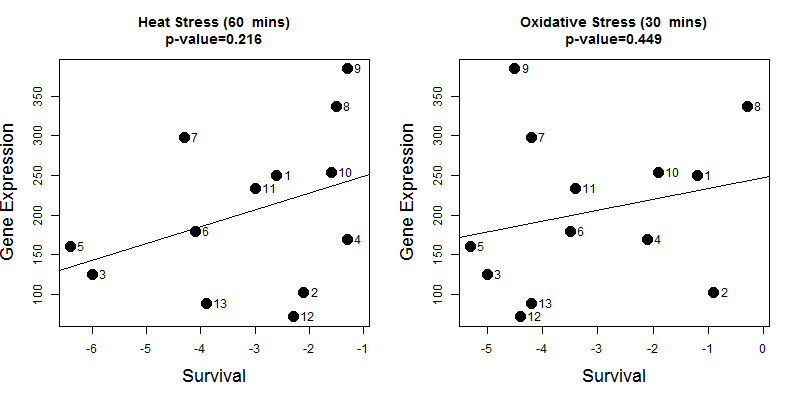

Supplement: S1 File — Expression levels of genes L0001 –L75633 plotted against survival after 60 minutes heat and 30 min oxidative stress. Survival is expressed as the difference of log CFU/ml after stress and before stress. Numbers indicate fermentations as presented in Table 1. P-values above the plots indicate significance of correlation (assessed by a linear model). (ZIP) [file pone.0167944.s006.zip › S1_File/L0074_real_dat.png]

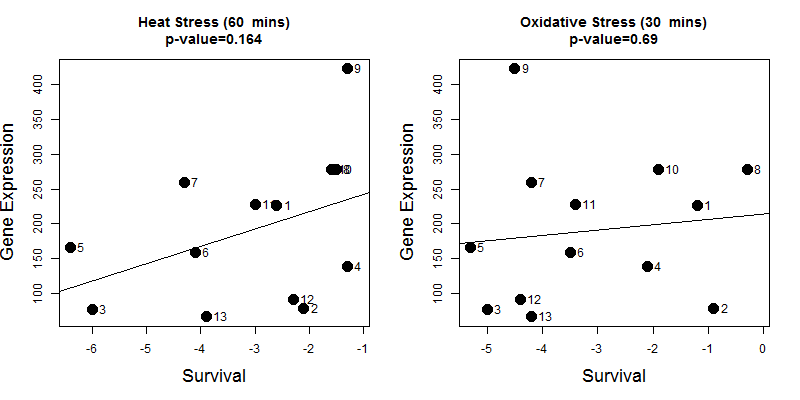

Supplement: S1 File — Expression levels of genes L0001 –L75633 plotted against survival after 60 minutes heat and 30 min oxidative stress. Survival is expressed as the difference of log CFU/ml after stress and before stress. Numbers indicate fermentations as presented in Table 1. P-values above the plots indicate significance of correlation (assessed by a linear model). (ZIP) [file pone.0167944.s006.zip › S1_File/L0075_real_dat.png]

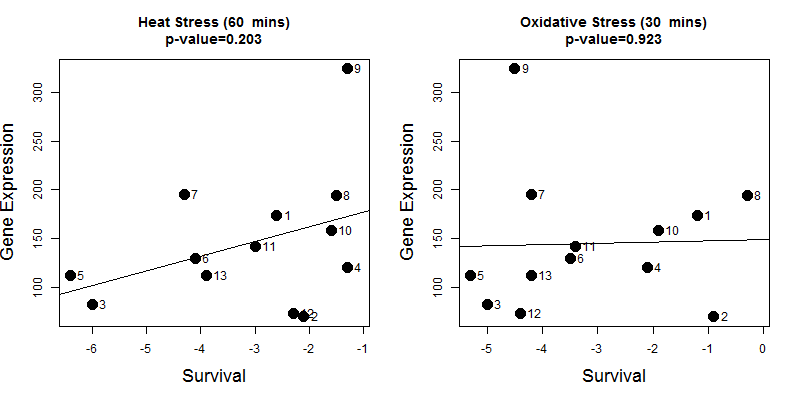

Supplement: S1 File — Expression levels of genes L0001 –L75633 plotted against survival after 60 minutes heat and 30 min oxidative stress. Survival is expressed as the difference of log CFU/ml after stress and before stress. Numbers indicate fermentations as presented in Table 1. P-values above the plots indicate significance of correlation (assessed by a linear model). (ZIP) [file pone.0167944.s006.zip › S1_File/L0076_real_dat.png]

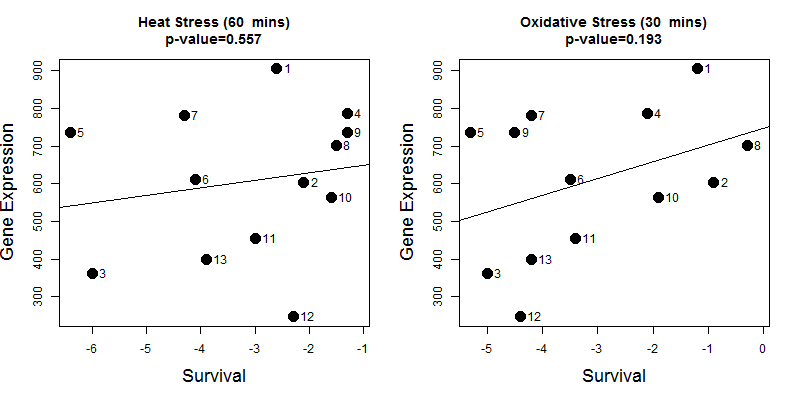

Supplement: S1 File — Expression levels of genes L0001 –L75633 plotted against survival after 60 minutes heat and 30 min oxidative stress. Survival is expressed as the difference of log CFU/ml after stress and before stress. Numbers indicate fermentations as presented in Table 1. P-values above the plots indicate significance of correlation (assessed by a linear model). (ZIP) [file pone.0167944.s006.zip › S1_File/L0077_real_dat.png]

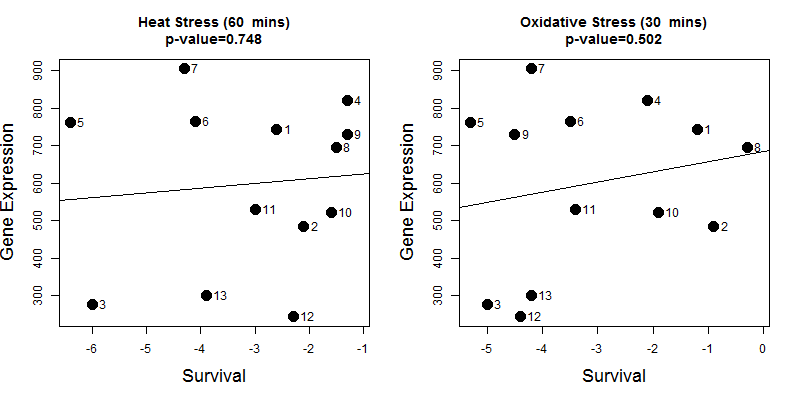

Supplement: S1 File — Expression levels of genes L0001 –L75633 plotted against survival after 60 minutes heat and 30 min oxidative stress. Survival is expressed as the difference of log CFU/ml after stress and before stress. Numbers indicate fermentations as presented in Table 1. P-values above the plots indicate significance of correlation (assessed by a linear model). (ZIP) [file pone.0167944.s006.zip › S1_File/L0078_real_dat.png]

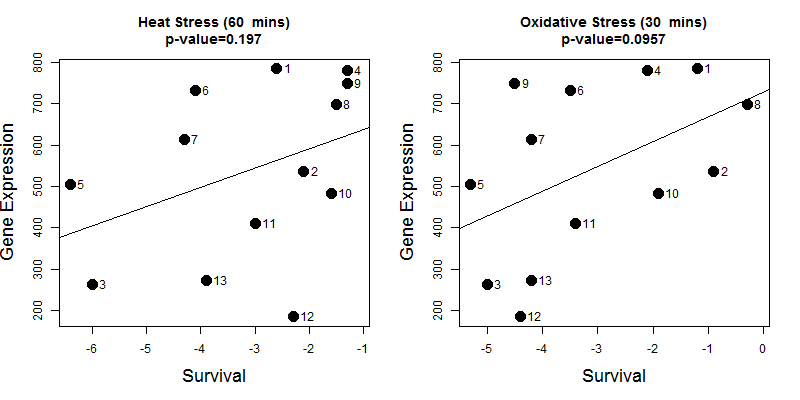

Supplement: S1 File — Expression levels of genes L0001 –L75633 plotted against survival after 60 minutes heat and 30 min oxidative stress. Survival is expressed as the difference of log CFU/ml after stress and before stress. Numbers indicate fermentations as presented in Table 1. P-values above the plots indicate significance of correlation (assessed by a linear model). (ZIP) [file pone.0167944.s006.zip › S1_File/L0079_real_dat.png]

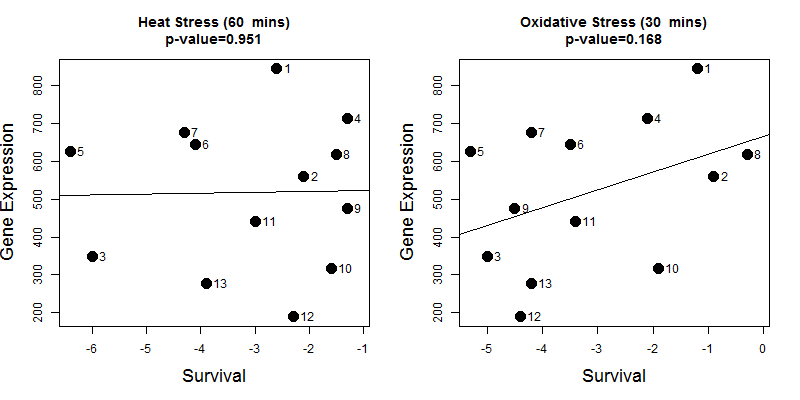

Supplement: S1 File — Expression levels of genes L0001 –L75633 plotted against survival after 60 minutes heat and 30 min oxidative stress. Survival is expressed as the difference of log CFU/ml after stress and before stress. Numbers indicate fermentations as presented in Table 1. P-values above the plots indicate significance of correlation (assessed by a linear model). (ZIP) [file pone.0167944.s006.zip › S1_File/L0080_real_dat.png]

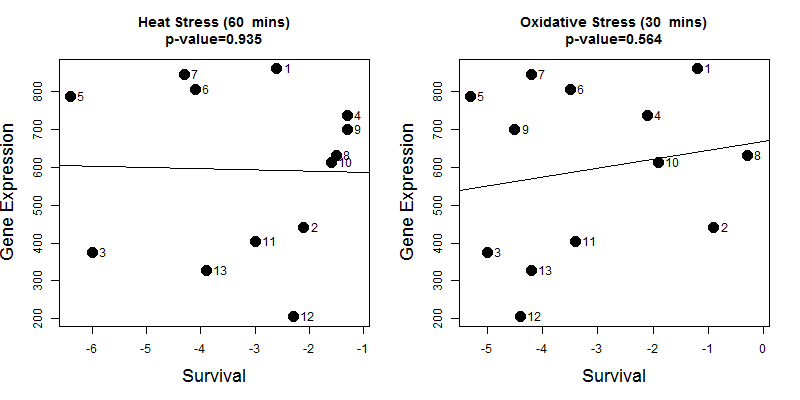

Supplement: S1 File — Expression levels of genes L0001 –L75633 plotted against survival after 60 minutes heat and 30 min oxidative stress. Survival is expressed as the difference of log CFU/ml after stress and before stress. Numbers indicate fermentations as presented in Table 1. P-values above the plots indicate significance of correlation (assessed by a linear model). (ZIP) [file pone.0167944.s006.zip › S1_File/L0081_real_dat.png]

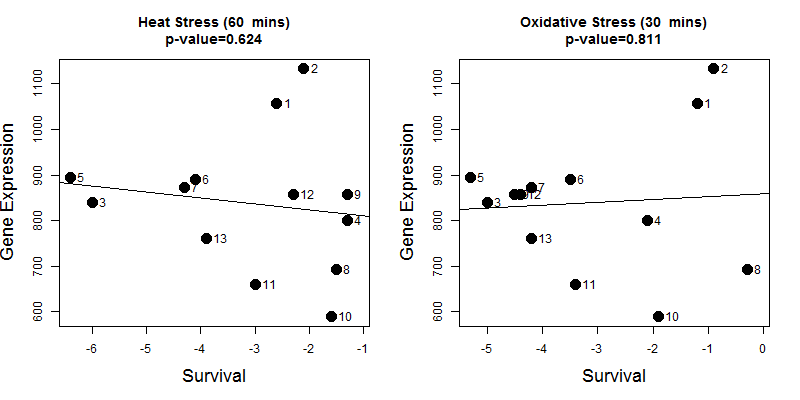

Supplement: S1 File — Expression levels of genes L0001 –L75633 plotted against survival after 60 minutes heat and 30 min oxidative stress. Survival is expressed as the difference of log CFU/ml after stress and before stress. Numbers indicate fermentations as presented in Table 1. P-values above the plots indicate significance of correlation (assessed by a linear model). (ZIP) [file pone.0167944.s006.zip › S1_File/L0082_real_dat.png]

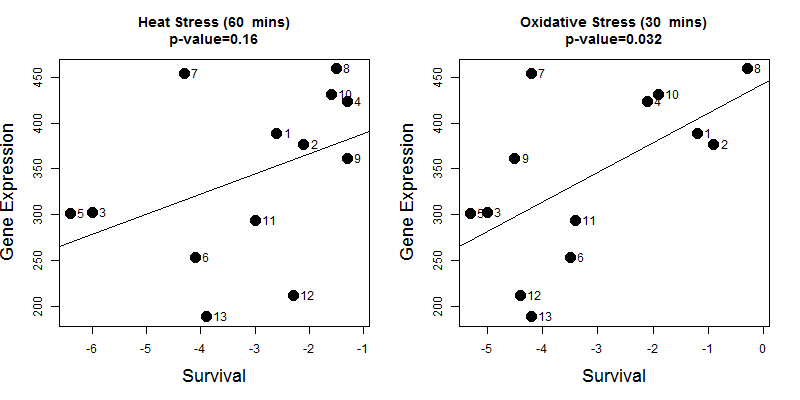

Supplement: S1 File — Expression levels of genes L0001 –L75633 plotted against survival after 60 minutes heat and 30 min oxidative stress. Survival is expressed as the difference of log CFU/ml after stress and before stress. Numbers indicate fermentations as presented in Table 1. P-values above the plots indicate significance of correlation (assessed by a linear model). (ZIP) [file pone.0167944.s006.zip › S1_File/L0083_real_dat.png]

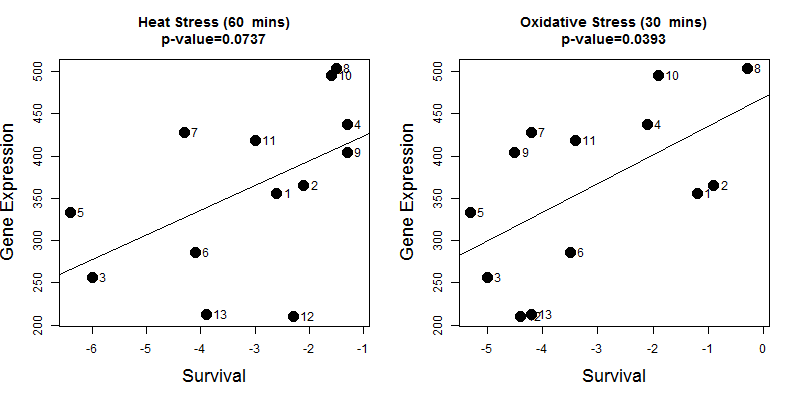

Supplement: S1 File — Expression levels of genes L0001 –L75633 plotted against survival after 60 minutes heat and 30 min oxidative stress. Survival is expressed as the difference of log CFU/ml after stress and before stress. Numbers indicate fermentations as presented in Table 1. P-values above the plots indicate significance of correlation (assessed by a linear model). (ZIP) [file pone.0167944.s006.zip › S1_File/L0084_real_dat.png]

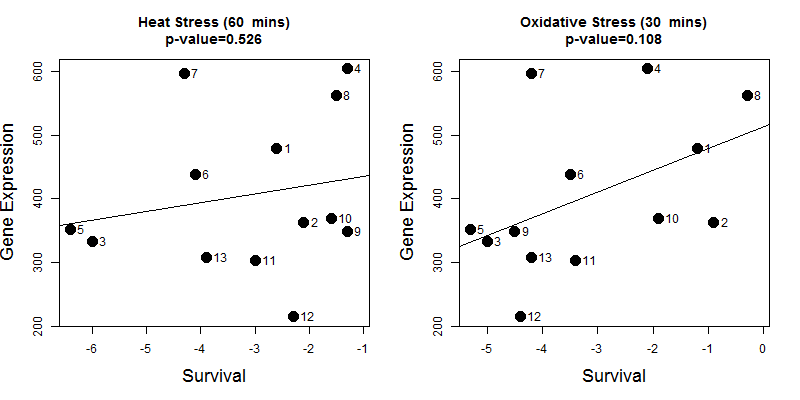

Supplement: S1 File — Expression levels of genes L0001 –L75633 plotted against survival after 60 minutes heat and 30 min oxidative stress. Survival is expressed as the difference of log CFU/ml after stress and before stress. Numbers indicate fermentations as presented in Table 1. P-values above the plots indicate significance of correlation (assessed by a linear model). (ZIP) [file pone.0167944.s006.zip › S1_File/L0085_real_dat.png]

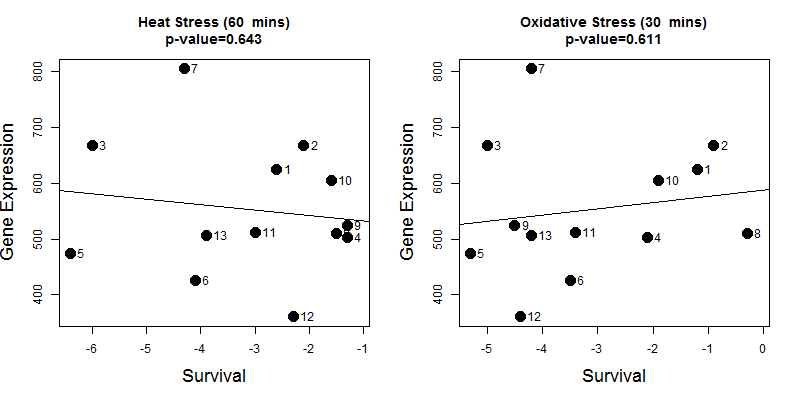

Supplement: S1 File — Expression levels of genes L0001 –L75633 plotted against survival after 60 minutes heat and 30 min oxidative stress. Survival is expressed as the difference of log CFU/ml after stress and before stress. Numbers indicate fermentations as presented in Table 1. P-values above the plots indicate significance of correlation (assessed by a linear model). (ZIP) [file pone.0167944.s006.zip › S1_File/L0086_real_dat.png]

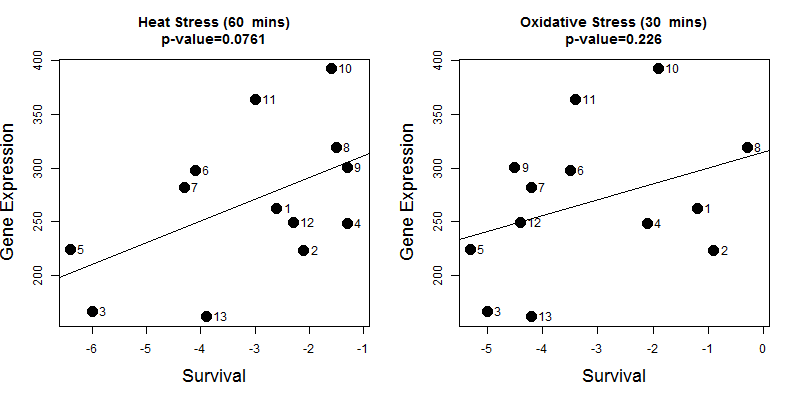

Supplement: S1 File — Expression levels of genes L0001 –L75633 plotted against survival after 60 minutes heat and 30 min oxidative stress. Survival is expressed as the difference of log CFU/ml after stress and before stress. Numbers indicate fermentations as presented in Table 1. P-values above the plots indicate significance of correlation (assessed by a linear model). (ZIP) [file pone.0167944.s006.zip › S1_File/L0087_real_dat.png]

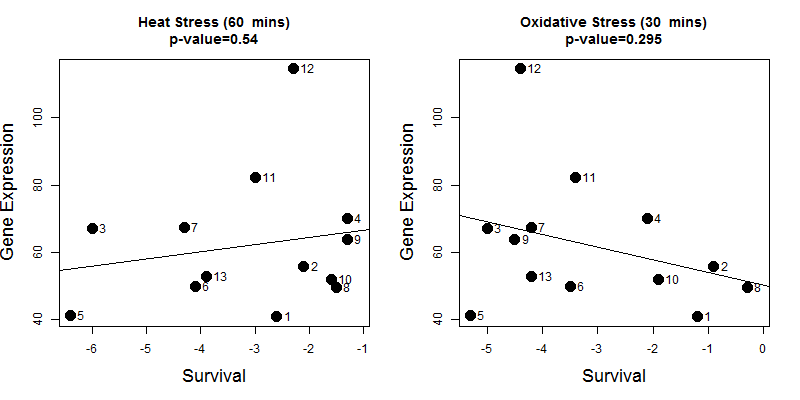

Supplement: S1 File — Expression levels of genes L0001 –L75633 plotted against survival after 60 minutes heat and 30 min oxidative stress. Survival is expressed as the difference of log CFU/ml after stress and before stress. Numbers indicate fermentations as presented in Table 1. P-values above the plots indicate significance of correlation (assessed by a linear model). (ZIP) [file pone.0167944.s006.zip › S1_File/L0088_real_dat.png]

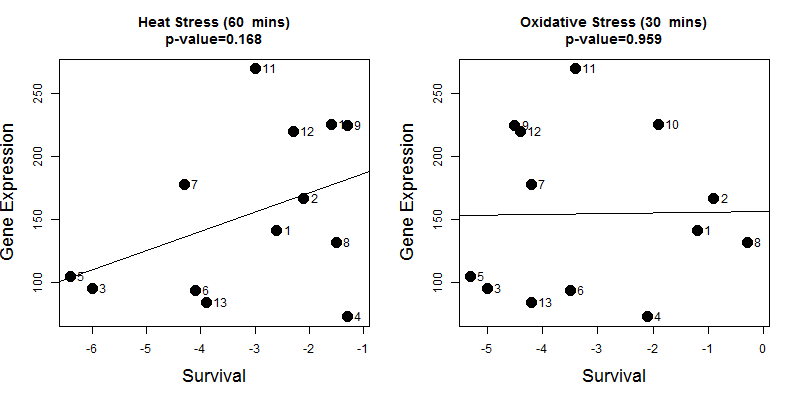

Supplement: S1 File — Expression levels of genes L0001 –L75633 plotted against survival after 60 minutes heat and 30 min oxidative stress. Survival is expressed as the difference of log CFU/ml after stress and before stress. Numbers indicate fermentations as presented in Table 1. P-values above the plots indicate significance of correlation (assessed by a linear model). (ZIP) [file pone.0167944.s006.zip › S1_File/L0089_real_dat.png]

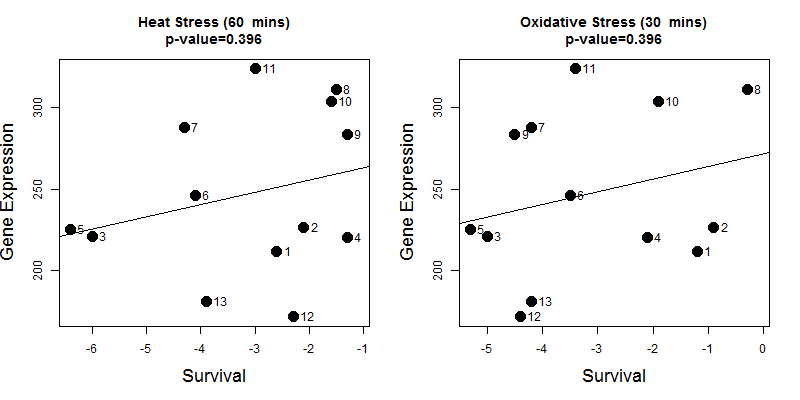

Supplement: S1 File — Expression levels of genes L0001 –L75633 plotted against survival after 60 minutes heat and 30 min oxidative stress. Survival is expressed as the difference of log CFU/ml after stress and before stress. Numbers indicate fermentations as presented in Table 1. P-values above the plots indicate significance of correlation (assessed by a linear model). (ZIP) [file pone.0167944.s006.zip › S1_File/L0090_real_dat.png]

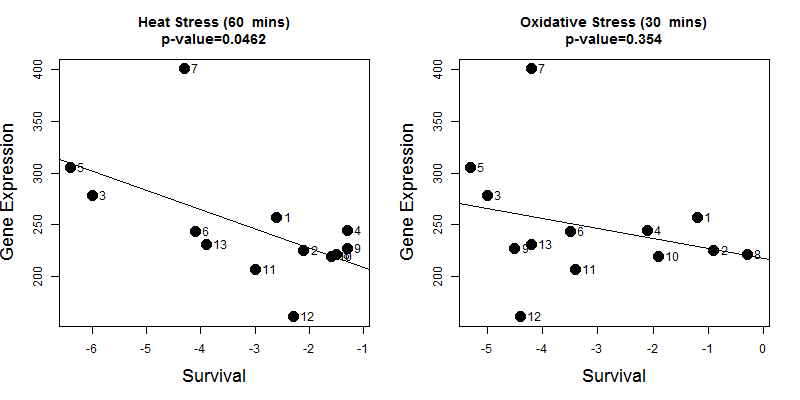

Supplement: S1 File — Expression levels of genes L0001 –L75633 plotted against survival after 60 minutes heat and 30 min oxidative stress. Survival is expressed as the difference of log CFU/ml after stress and before stress. Numbers indicate fermentations as presented in Table 1. P-values above the plots indicate significance of correlation (assessed by a linear model). (ZIP) [file pone.0167944.s006.zip › S1_File/L0091_real_dat.png]

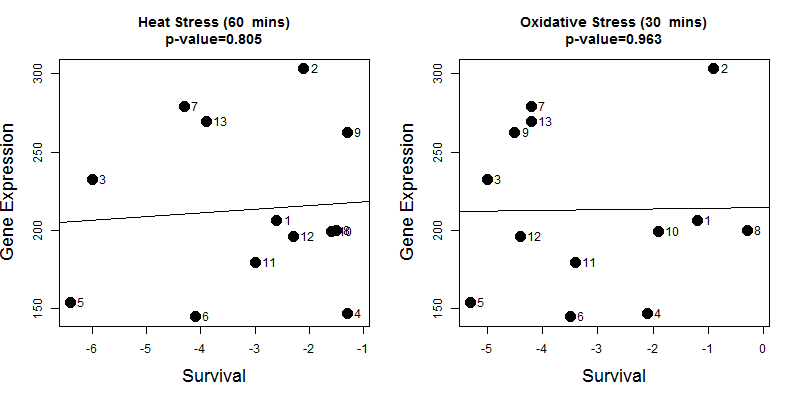

Supplement: S1 File — Expression levels of genes L0001 –L75633 plotted against survival after 60 minutes heat and 30 min oxidative stress. Survival is expressed as the difference of log CFU/ml after stress and before stress. Numbers indicate fermentations as presented in Table 1. P-values above the plots indicate significance of correlation (assessed by a linear model). (ZIP) [file pone.0167944.s006.zip › S1_File/L0092_real_dat.png]

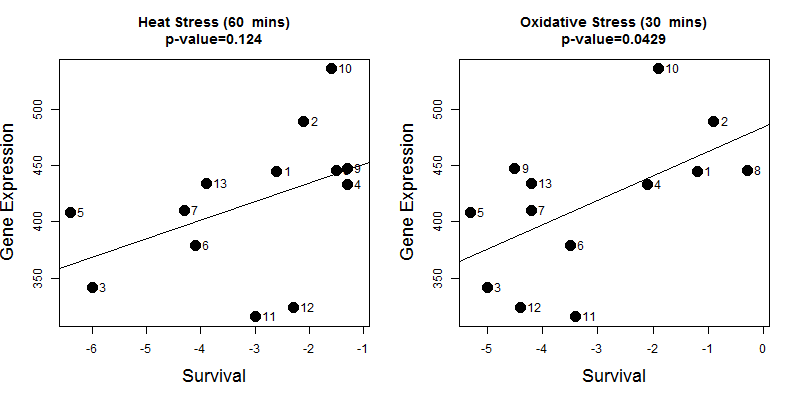

Supplement: S1 File — Expression levels of genes L0001 –L75633 plotted against survival after 60 minutes heat and 30 min oxidative stress. Survival is expressed as the difference of log CFU/ml after stress and before stress. Numbers indicate fermentations as presented in Table 1. P-values above the plots indicate significance of correlation (assessed by a linear model). (ZIP) [file pone.0167944.s006.zip › S1_File/L0093_real_dat.png]

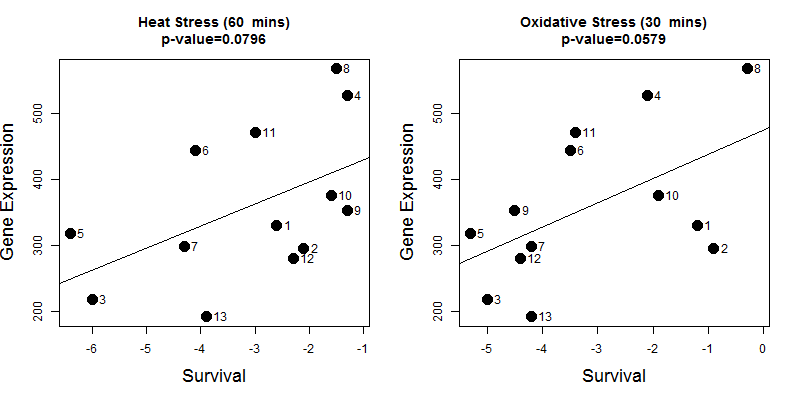

Supplement: S1 File — Expression levels of genes L0001 –L75633 plotted against survival after 60 minutes heat and 30 min oxidative stress. Survival is expressed as the difference of log CFU/ml after stress and before stress. Numbers indicate fermentations as presented in Table 1. P-values above the plots indicate significance of correlation (assessed by a linear model). (ZIP) [file pone.0167944.s006.zip › S1_File/L0094_real_dat.png]

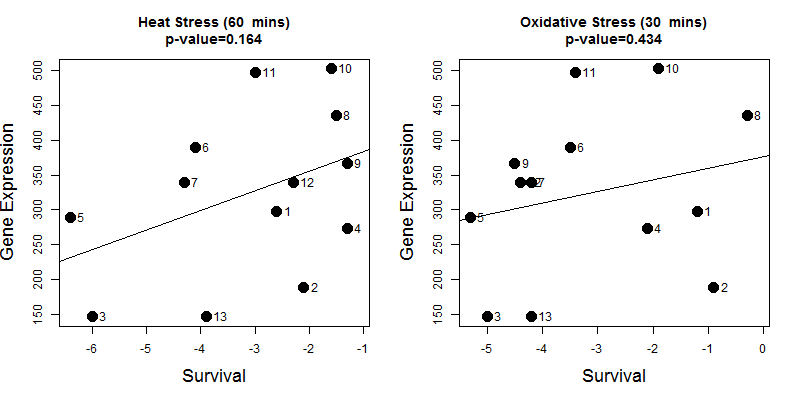

Supplement: S1 File — Expression levels of genes L0001 –L75633 plotted against survival after 60 minutes heat and 30 min oxidative stress. Survival is expressed as the difference of log CFU/ml after stress and before stress. Numbers indicate fermentations as presented in Table 1. P-values above the plots indicate significance of correlation (assessed by a linear model). (ZIP) [file pone.0167944.s006.zip › S1_File/L0095_real_dat.png]
