# Supplementary material for: Strain-Dependent Transcriptome Signatures for Robustness in Lactococcus lactis
Source: PLoS One. 2016 Dec 14;11(12):e0167944. doi: 10.1371/journal.pone.0167944 (PMC5156439; doi:10.1371/journal.pone.0167944)
Supplement: S1 Table — Survival after 30 minutes of heat stress and after 60 minutes of oxidative stress in the various fermentations of strains IL1403, KF147 and SK11. Survival data represent averages of technical duplicates. (DOCX) [file pone.0167944.s004.docx]

**S1 Table. Heat and oxidative stress survival at additional time point.**

|  |  |  |  |  | **heat stress survival (%)** | | | **oxidative stress survival (%)** | | |
| --- | --- | --- | --- | --- | --- | --- | --- | --- | --- | --- |
| **fermentation number** | **salt (mM)** | **starting pH** | **temperature (°C)** | **level of oxygen** | **IL1403 30 min** | **KF147 30 min** | **SK11 30 min** | **IL1403 60 min** | **KF147 60 min** | **SK11 60 min** |
| 1 | 0 | 6.0 | 27 | + | 62 | 0.0018 | 0.0001 | 0.0045 | 0.00028 | 0.0011 |
| 2 | 100 | 6.5 | 27 | + | 43 | 0.0016 | 0.0002 | 0.00051 | 0.0043 | 0.0021 |
| 3 | 0 | 6.5 | 27 | - | 0.048 | 0.00011 | 0.0001 | 0.00004 | 0.00013 | 0.0049 |
| 4 | 100 | 6.0 | 27 | - | 58 | 0.00017 | 0.0001 | 0.0013 | 0.0045 | 0.0008 |
| 5 | 0 | 6.0 | 30 | - | 0.36 | 0.00013 | 0.0001 | 0.00004 | 0.00015 | 0.0029 |
| 6 | 100 | 6.5 | 30 | - | 4.1 | 0.00010 | 0.0001 | 0.00081 | 0.00036 | 0.0011 |
| 7 | 0 | 6.5 | 30 | + | 9.7 | 0.0086 | 0.0003 | 0.00038 | 0.00011 | 0.0016 |
| 8 | 100 | 6.0 | 30 | + | 74 | 0.013 | 0.0003 | 0.093 | 0.014 | 0.0022 |
| 9 | 0 | 6.0 | 35 | + | 126 | 0.27 | 0.0098 | 0.00053 | 0.00078 | 1.9 |
| 10 | 100 | 6.5 | 35 | + | 65 | 0.42 | 0.0043 | 0.0014 | 0.00095 | 0.093 |
| 11 | 0 | 6.5 | 35 | - | 9.6 | 0.0054 | 0.0059 | 0.00053 | 0.00023 | 0.0002 |
| 12 | 100 | 6.0 | 35 | - | 46 | 0.0018 | 0.0007 | 0.00042 | 0.00008 | 0.0009 |
| 13 | 100 | 6.5 | 30 | - | 12 | 0.00013 | 0.0002 | 0.00074 | 0.00019 | 0.0002 |
