# Supplementary material for: Strain-Dependent Transcriptome Signatures for Robustness in Lactococcus lactis
Source: PLoS One. 2016 Dec 14;11(12):e0167944. doi: 10.1371/journal.pone.0167944 (PMC5156439; doi:10.1371/journal.pone.0167944)
Supplement: S2 Table — T-test-based correlation of individual fermentation parameters and robustness. Significant differences (P < 0.05) are underlined. (DOCX) [file pone.0167944.s005.docx]

**S2 Table. Correlation fermentation parameters and robustness.**

| **IL1403** |  | oxygen | salt | pH | T 27 vs. 30°C | T 27 vs. 35°C | T 30 vs. 35°C |
| --- | --- | --- | --- | --- | --- | --- | --- |
| heat stress | 30 min | 0.050 | 0.205 | 0.253 | 0.847 | 0.371 | 0.264 |
|  | 60 min | 0.082 | 0.138 | 0.329 | 0.365 | 0.430 | 0.100 |
| oxidative stress | 30 min | 0.060 | 0.117 | 0.745 | 0.321 | 0.326 | 0.966 |
|  | 60 min | 0.103 | 0.146 | 0.382 | 0.726 | 1.000 | 0.726 |
| **KF147** |  |  |  |  |  |  |  |
| heat stress | 10 min | 0.003 | 0.666 | 0.890 | 0.608 | 0.053 | 0.104 |
|  | 30 min | 0.007 | 0.765 | 0.831 | 0.840 | 0.031 | 0.036 |
| oxidative stress | 30 min | 0.050 | 0.341 | 0.518 | 0.826 | 0.628 | 0.770 |
|  | 60 min | 0.169 | 0.074 | 0.514 | 0.573 | 0.464 | 0.830 |
| **SK11** |  |  |  |  |  |  |  |
| heat stress | 10 min | 0.786 | 0.595 | 0.995 | 0.001 | 0.000 | 0.040 |
|  | 30 min | 0.426 | 0.768 | 0.755 | 0.526 | 0.000 | 0.000 |
| oxidative stress | 30 min | 0.103 | 0.957 | 0.957 | 0.583 | 0.467 | 0.203 |
|  | 60 min | 0.115 | 0.544 | 0.570 | 0.845 | 0.264 | 0.178 |
