# Supplementary figures and images for: Strain-Dependent Transcriptome Signatures for Robustness in Lactococcus lactis (part 3 of 13)
Source: PLoS One. 2016 Dec 14;11(12):e0167944. doi: 10.1371/journal.pone.0167944 (PMC5156439; doi:10.1371/journal.pone.0167944)

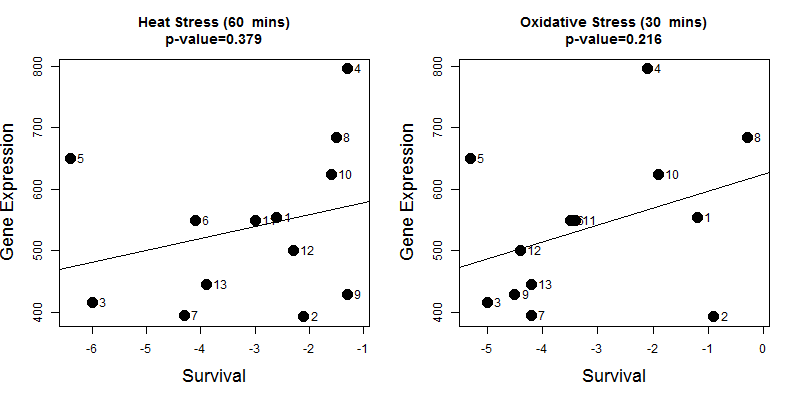

Supplement: S1 File — Expression levels of genes L0001 –L75633 plotted against survival after 60 minutes heat and 30 min oxidative stress. Survival is expressed as the difference of log CFU/ml after stress and before stress. Numbers indicate fermentations as presented in Table 1. P-values above the plots indicate significance of correlation (assessed by a linear model). (ZIP) [file pone.0167944.s006.zip › S1_File/L0197_real_dat.png]

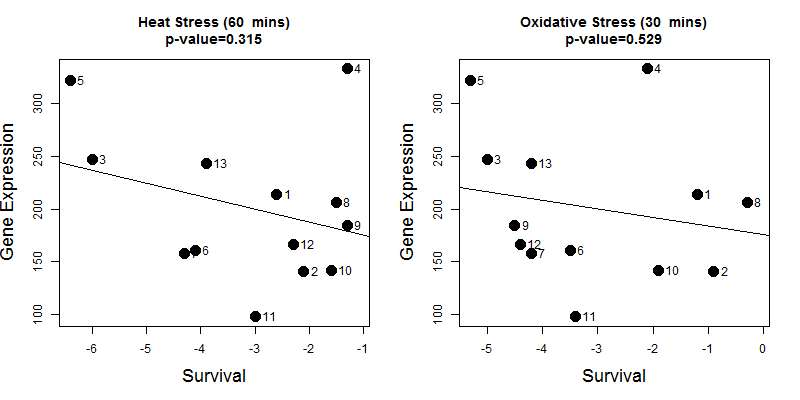

Supplement: S1 File — Expression levels of genes L0001 –L75633 plotted against survival after 60 minutes heat and 30 min oxidative stress. Survival is expressed as the difference of log CFU/ml after stress and before stress. Numbers indicate fermentations as presented in Table 1. P-values above the plots indicate significance of correlation (assessed by a linear model). (ZIP) [file pone.0167944.s006.zip › S1_File/L0198_real_dat.png]

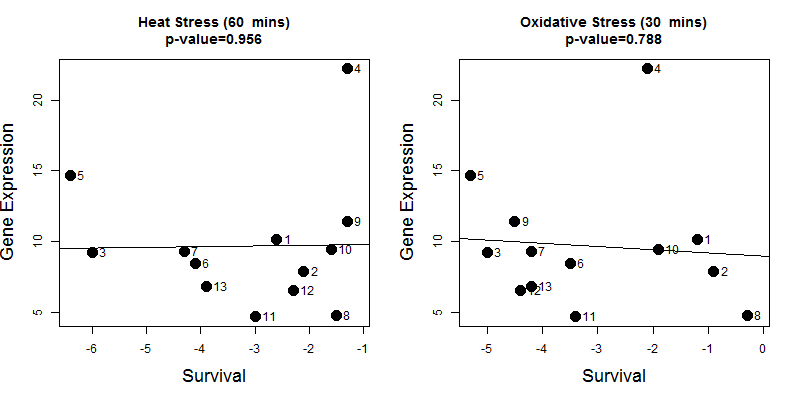

Supplement: S1 File — Expression levels of genes L0001 –L75633 plotted against survival after 60 minutes heat and 30 min oxidative stress. Survival is expressed as the difference of log CFU/ml after stress and before stress. Numbers indicate fermentations as presented in Table 1. P-values above the plots indicate significance of correlation (assessed by a linear model). (ZIP) [file pone.0167944.s006.zip › S1_File/L0199_real_dat.png]

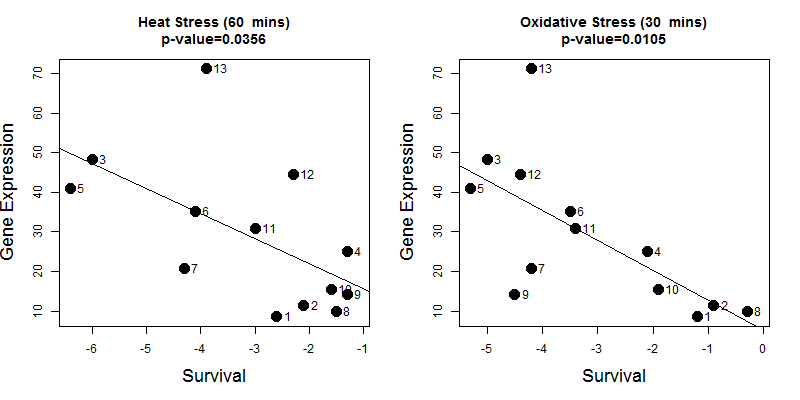

Supplement: S2 File — Expression levels of genes L75676 –L1889726 plotted against survival after 60 minutes heat and 30 min oxidative stress. Survival is expressed as the difference of log CFU/ml after stress and before stress. Numbers indicate fermentations as presented in Table 1. P-values above the plots indicate significance of correlation (assessed by a linear model). (ZIP) [file pone.0167944.s007.zip › S2_File/L100027_real_dat.png]

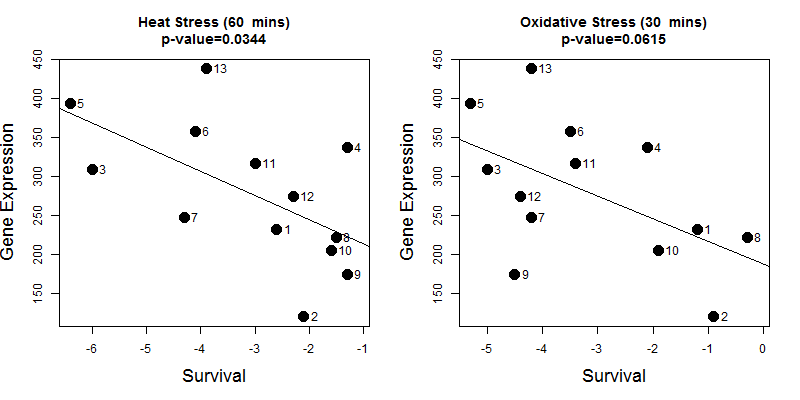

Supplement: S2 File — Expression levels of genes L75676 –L1889726 plotted against survival after 60 minutes heat and 30 min oxidative stress. Survival is expressed as the difference of log CFU/ml after stress and before stress. Numbers indicate fermentations as presented in Table 1. P-values above the plots indicate significance of correlation (assessed by a linear model). (ZIP) [file pone.0167944.s007.zip › S2_File/L100099_real_dat.png]

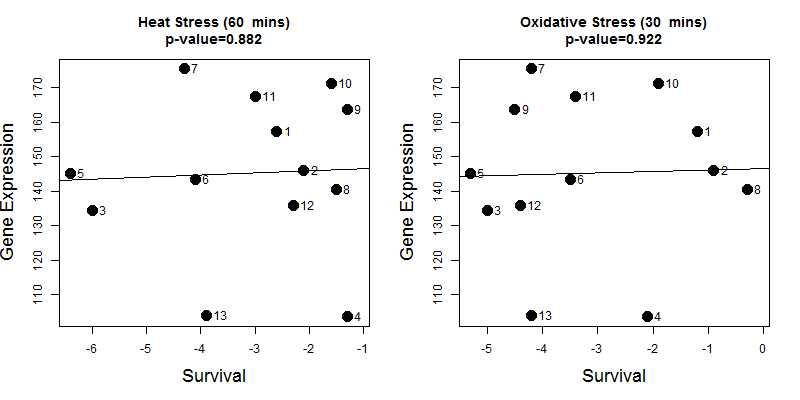

Supplement: S2 File — Expression levels of genes L75676 –L1889726 plotted against survival after 60 minutes heat and 30 min oxidative stress. Survival is expressed as the difference of log CFU/ml after stress and before stress. Numbers indicate fermentations as presented in Table 1. P-values above the plots indicate significance of correlation (assessed by a linear model). (ZIP) [file pone.0167944.s007.zip › S2_File/L100263_real_dat.png]

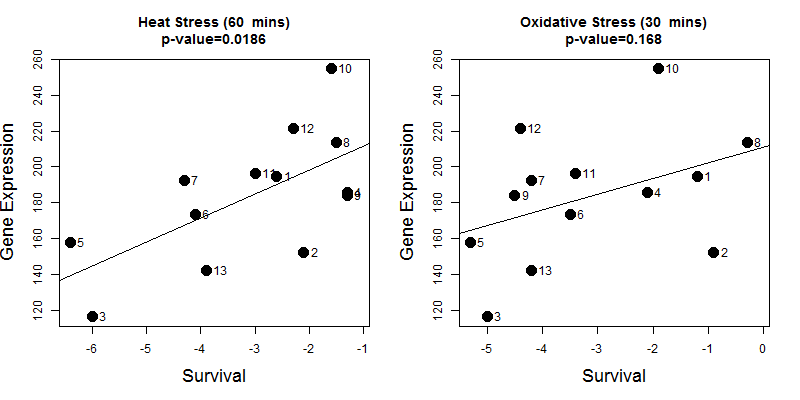

Supplement: S2 File — Expression levels of genes L75676 –L1889726 plotted against survival after 60 minutes heat and 30 min oxidative stress. Survival is expressed as the difference of log CFU/ml after stress and before stress. Numbers indicate fermentations as presented in Table 1. P-values above the plots indicate significance of correlation (assessed by a linear model). (ZIP) [file pone.0167944.s007.zip › S2_File/L100350_real_dat.png]

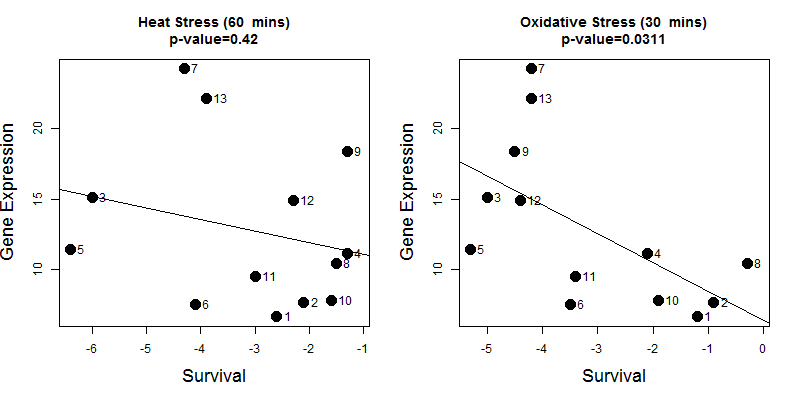

Supplement: S2 File — Expression levels of genes L75676 –L1889726 plotted against survival after 60 minutes heat and 30 min oxidative stress. Survival is expressed as the difference of log CFU/ml after stress and before stress. Numbers indicate fermentations as presented in Table 1. P-values above the plots indicate significance of correlation (assessed by a linear model). (ZIP) [file pone.0167944.s007.zip › S2_File/L100541_real_dat.png]

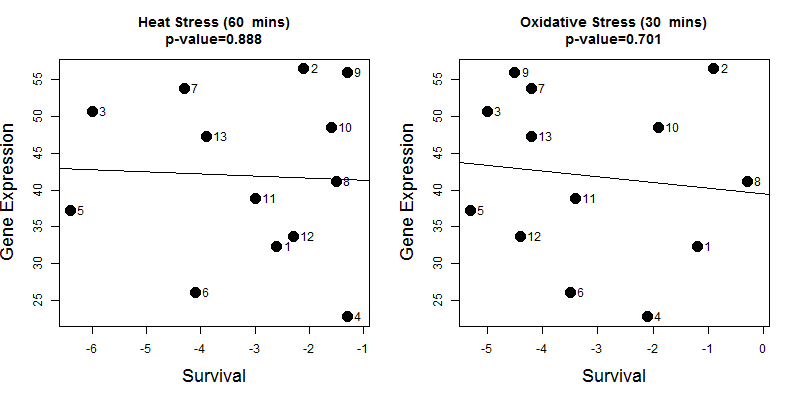

Supplement: S2 File — Expression levels of genes L75676 –L1889726 plotted against survival after 60 minutes heat and 30 min oxidative stress. Survival is expressed as the difference of log CFU/ml after stress and before stress. Numbers indicate fermentations as presented in Table 1. P-values above the plots indicate significance of correlation (assessed by a linear model). (ZIP) [file pone.0167944.s007.zip › S2_File/L100575_real_dat.png]

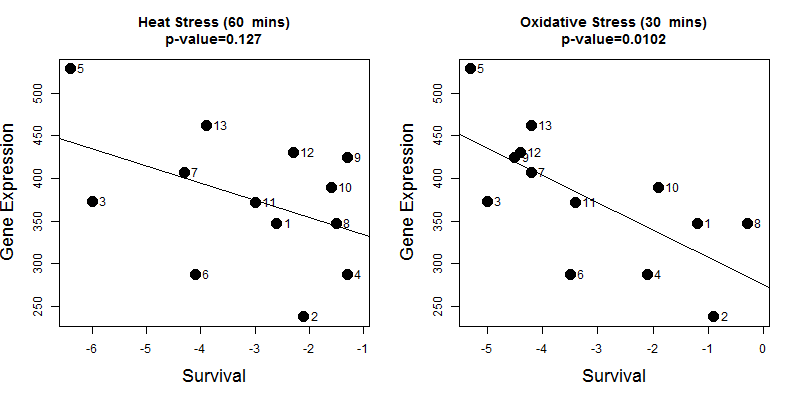

Supplement: S2 File — Expression levels of genes L75676 –L1889726 plotted against survival after 60 minutes heat and 30 min oxidative stress. Survival is expressed as the difference of log CFU/ml after stress and before stress. Numbers indicate fermentations as presented in Table 1. P-values above the plots indicate significance of correlation (assessed by a linear model). (ZIP) [file pone.0167944.s007.zip › S2_File/L100735_real_dat.png]

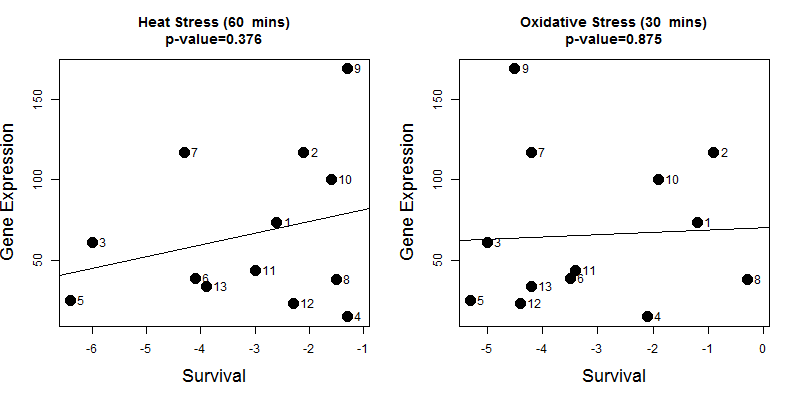

Supplement: S2 File — Expression levels of genes L75676 –L1889726 plotted against survival after 60 minutes heat and 30 min oxidative stress. Survival is expressed as the difference of log CFU/ml after stress and before stress. Numbers indicate fermentations as presented in Table 1. P-values above the plots indicate significance of correlation (assessed by a linear model). (ZIP) [file pone.0167944.s007.zip › S2_File/L100822_real_dat.png]

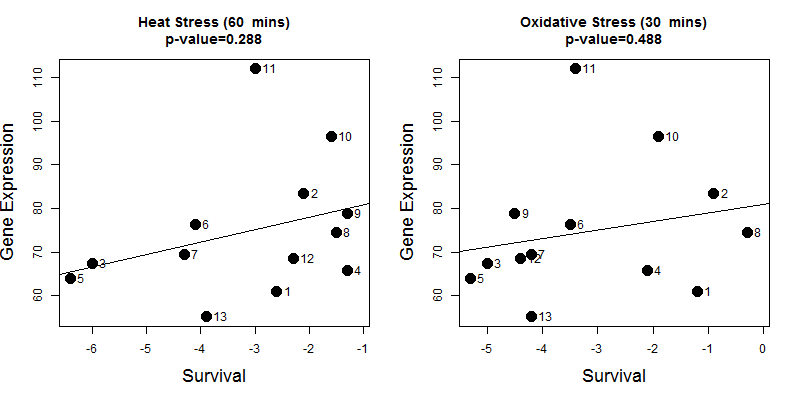

Supplement: S2 File — Expression levels of genes L75676 –L1889726 plotted against survival after 60 minutes heat and 30 min oxidative stress. Survival is expressed as the difference of log CFU/ml after stress and before stress. Numbers indicate fermentations as presented in Table 1. P-values above the plots indicate significance of correlation (assessed by a linear model). (ZIP) [file pone.0167944.s007.zip › S2_File/L101209_real_dat.png]

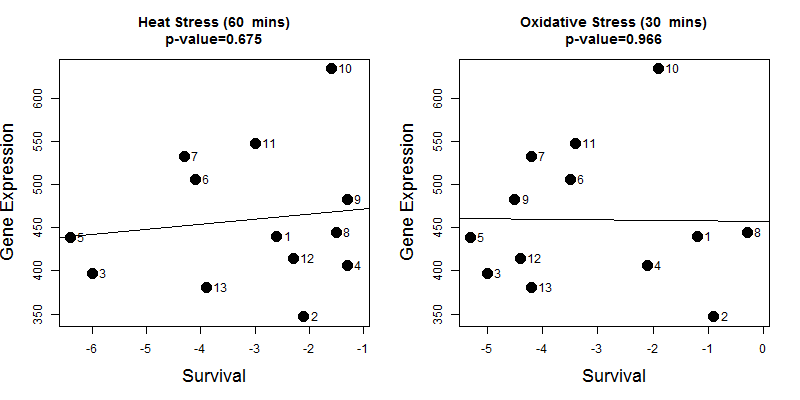

Supplement: S2 File — Expression levels of genes L75676 –L1889726 plotted against survival after 60 minutes heat and 30 min oxidative stress. Survival is expressed as the difference of log CFU/ml after stress and before stress. Numbers indicate fermentations as presented in Table 1. P-values above the plots indicate significance of correlation (assessed by a linear model). (ZIP) [file pone.0167944.s007.zip › S2_File/L101219_real_dat.png]

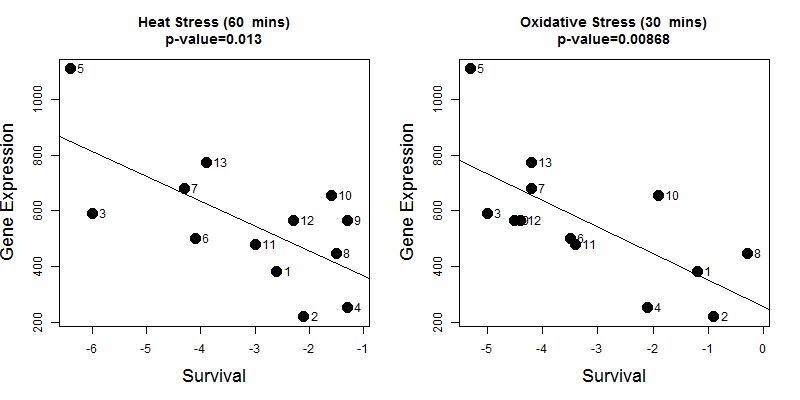

Supplement: S2 File — Expression levels of genes L75676 –L1889726 plotted against survival after 60 minutes heat and 30 min oxidative stress. Survival is expressed as the difference of log CFU/ml after stress and before stress. Numbers indicate fermentations as presented in Table 1. P-values above the plots indicate significance of correlation (assessed by a linear model). (ZIP) [file pone.0167944.s007.zip › S2_File/L101488_real_dat.png]

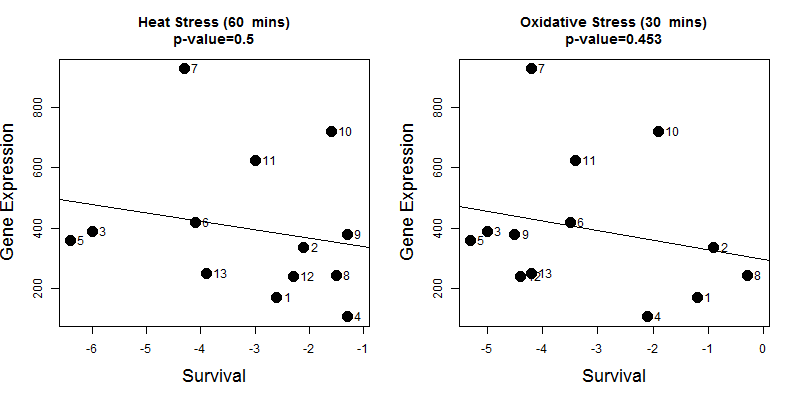

Supplement: S2 File — Expression levels of genes L75676 –L1889726 plotted against survival after 60 minutes heat and 30 min oxidative stress. Survival is expressed as the difference of log CFU/ml after stress and before stress. Numbers indicate fermentations as presented in Table 1. P-values above the plots indicate significance of correlation (assessed by a linear model). (ZIP) [file pone.0167944.s007.zip › S2_File/L101560_real_dat.png]

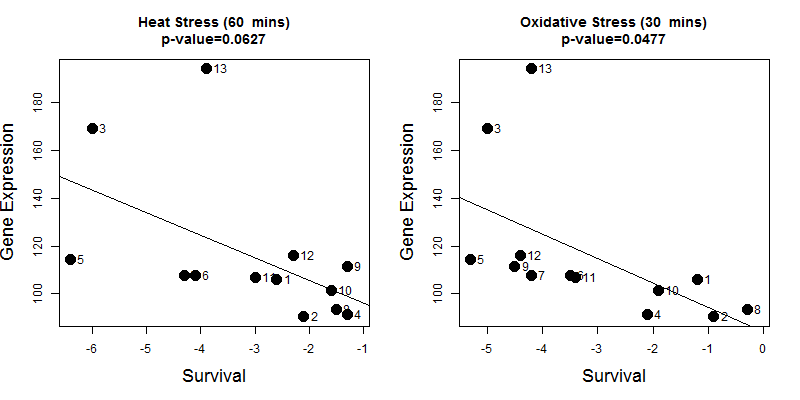

Supplement: S2 File — Expression levels of genes L75676 –L1889726 plotted against survival after 60 minutes heat and 30 min oxidative stress. Survival is expressed as the difference of log CFU/ml after stress and before stress. Numbers indicate fermentations as presented in Table 1. P-values above the plots indicate significance of correlation (assessed by a linear model). (ZIP) [file pone.0167944.s007.zip › S2_File/L101577_real_dat.png]

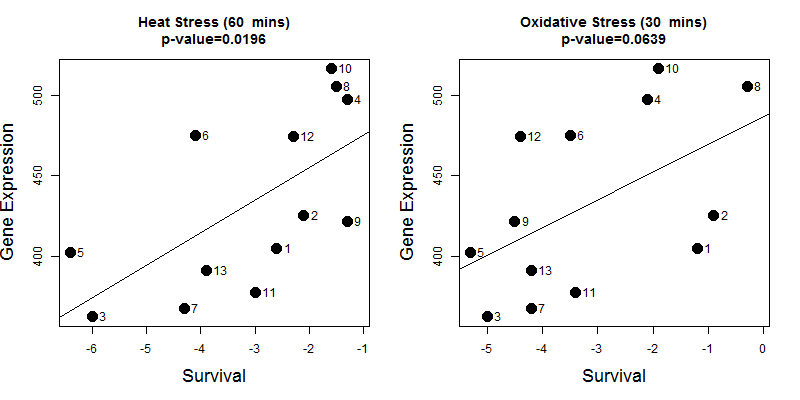

Supplement: S2 File — Expression levels of genes L75676 –L1889726 plotted against survival after 60 minutes heat and 30 min oxidative stress. Survival is expressed as the difference of log CFU/ml after stress and before stress. Numbers indicate fermentations as presented in Table 1. P-values above the plots indicate significance of correlation (assessed by a linear model). (ZIP) [file pone.0167944.s007.zip › S2_File/L101678_real_dat.png]

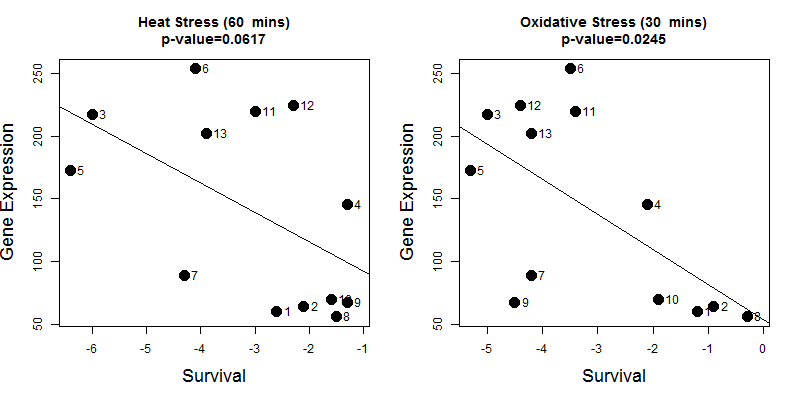

Supplement: S2 File — Expression levels of genes L75676 –L1889726 plotted against survival after 60 minutes heat and 30 min oxidative stress. Survival is expressed as the difference of log CFU/ml after stress and before stress. Numbers indicate fermentations as presented in Table 1. P-values above the plots indicate significance of correlation (assessed by a linear model). (ZIP) [file pone.0167944.s007.zip › S2_File/L101688_real_dat.png]

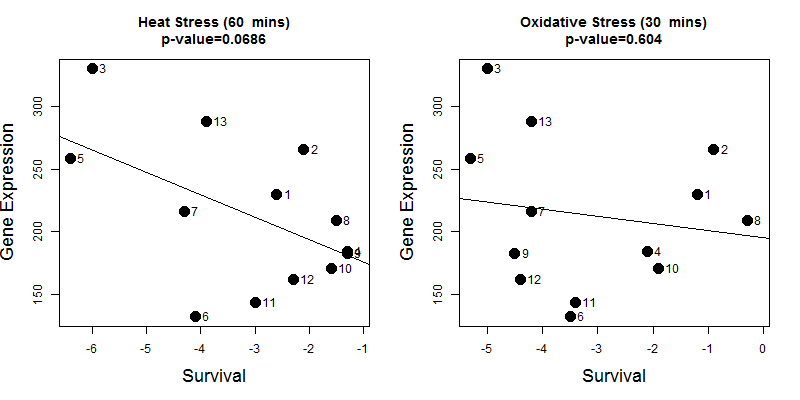

Supplement: S2 File — Expression levels of genes L75676 –L1889726 plotted against survival after 60 minutes heat and 30 min oxidative stress. Survival is expressed as the difference of log CFU/ml after stress and before stress. Numbers indicate fermentations as presented in Table 1. P-values above the plots indicate significance of correlation (assessed by a linear model). (ZIP) [file pone.0167944.s007.zip › S2_File/L101699_real_dat.png]

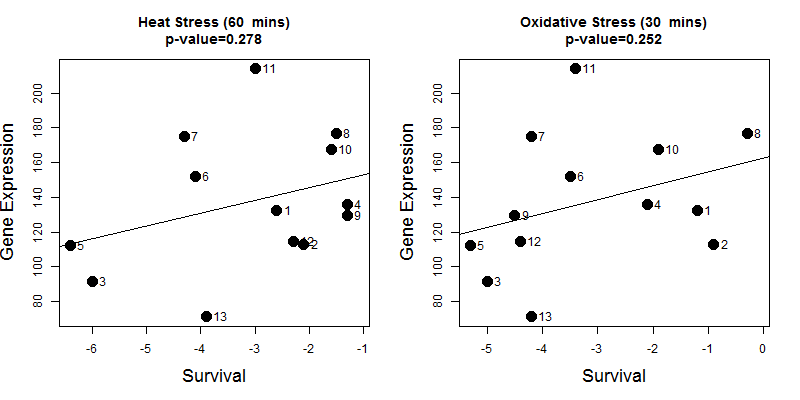

Supplement: S2 File — Expression levels of genes L75676 –L1889726 plotted against survival after 60 minutes heat and 30 min oxidative stress. Survival is expressed as the difference of log CFU/ml after stress and before stress. Numbers indicate fermentations as presented in Table 1. P-values above the plots indicate significance of correlation (assessed by a linear model). (ZIP) [file pone.0167944.s007.zip › S2_File/L101706_real_dat.png]

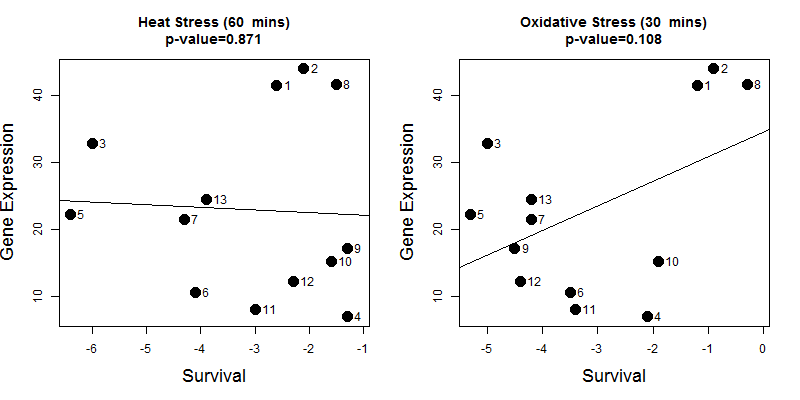

Supplement: S2 File — Expression levels of genes L75676 –L1889726 plotted against survival after 60 minutes heat and 30 min oxidative stress. Survival is expressed as the difference of log CFU/ml after stress and before stress. Numbers indicate fermentations as presented in Table 1. P-values above the plots indicate significance of correlation (assessed by a linear model). (ZIP) [file pone.0167944.s007.zip › S2_File/L101896_real_dat.png]

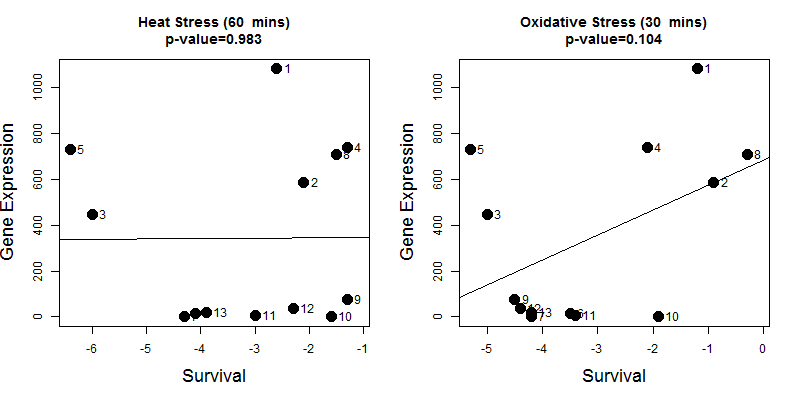

Supplement: S2 File — Expression levels of genes L75676 –L1889726 plotted against survival after 60 minutes heat and 30 min oxidative stress. Survival is expressed as the difference of log CFU/ml after stress and before stress. Numbers indicate fermentations as presented in Table 1. P-values above the plots indicate significance of correlation (assessed by a linear model). (ZIP) [file pone.0167944.s007.zip › S2_File/L101912_real_dat.png]

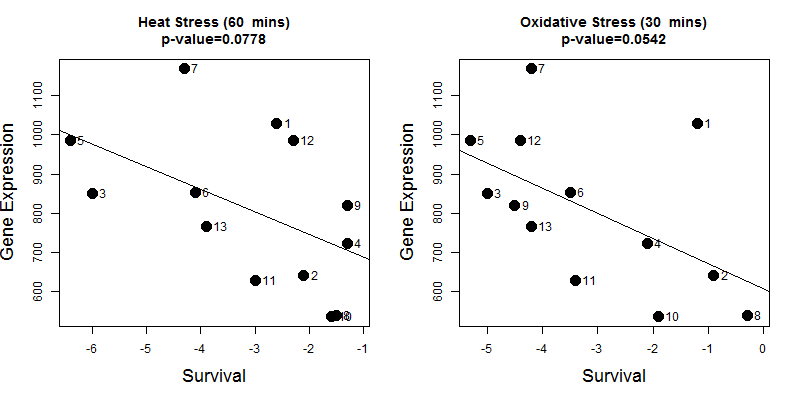

Supplement: S2 File — Expression levels of genes L75676 –L1889726 plotted against survival after 60 minutes heat and 30 min oxidative stress. Survival is expressed as the difference of log CFU/ml after stress and before stress. Numbers indicate fermentations as presented in Table 1. P-values above the plots indicate significance of correlation (assessed by a linear model). (ZIP) [file pone.0167944.s007.zip › S2_File/L102051_real_dat.png]

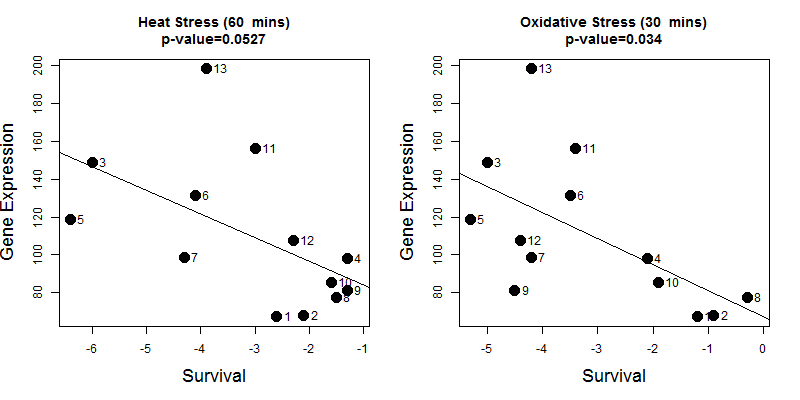

Supplement: S2 File — Expression levels of genes L75676 –L1889726 plotted against survival after 60 minutes heat and 30 min oxidative stress. Survival is expressed as the difference of log CFU/ml after stress and before stress. Numbers indicate fermentations as presented in Table 1. P-values above the plots indicate significance of correlation (assessed by a linear model). (ZIP) [file pone.0167944.s007.zip › S2_File/L102062_real_dat.png]

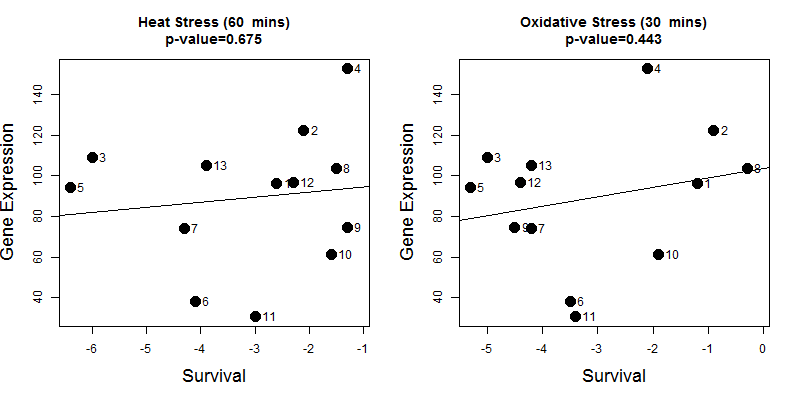

Supplement: S2 File — Expression levels of genes L75676 –L1889726 plotted against survival after 60 minutes heat and 30 min oxidative stress. Survival is expressed as the difference of log CFU/ml after stress and before stress. Numbers indicate fermentations as presented in Table 1. P-values above the plots indicate significance of correlation (assessed by a linear model). (ZIP) [file pone.0167944.s007.zip › S2_File/L102093_real_dat.png]

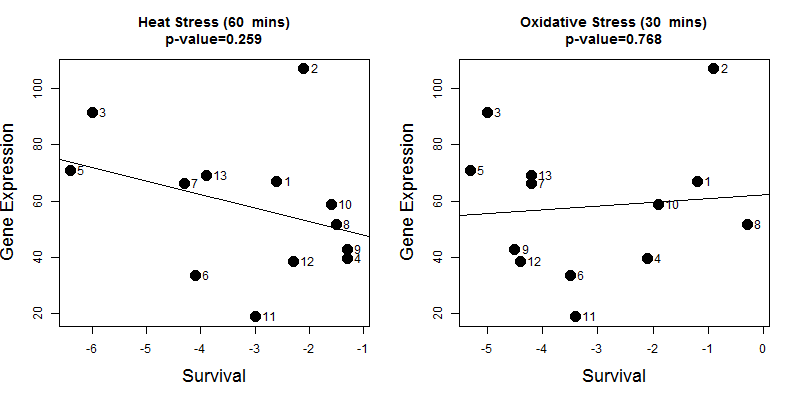

Supplement: S2 File — Expression levels of genes L75676 –L1889726 plotted against survival after 60 minutes heat and 30 min oxidative stress. Survival is expressed as the difference of log CFU/ml after stress and before stress. Numbers indicate fermentations as presented in Table 1. P-values above the plots indicate significance of correlation (assessed by a linear model). (ZIP) [file pone.0167944.s007.zip › S2_File/L102100_real_dat.png]

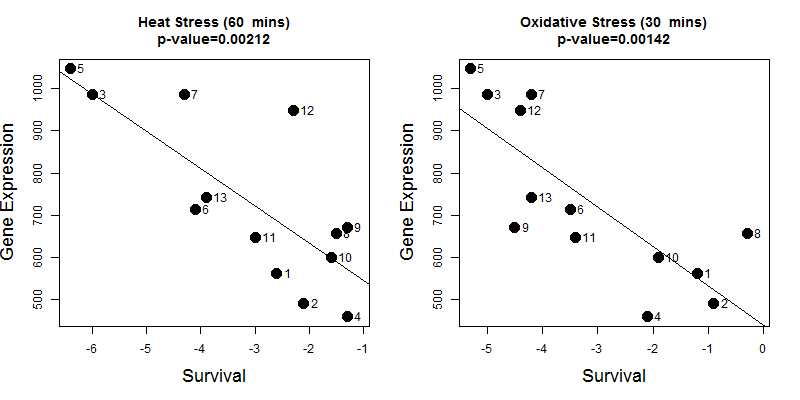

Supplement: S2 File — Expression levels of genes L75676 –L1889726 plotted against survival after 60 minutes heat and 30 min oxidative stress. Survival is expressed as the difference of log CFU/ml after stress and before stress. Numbers indicate fermentations as presented in Table 1. P-values above the plots indicate significance of correlation (assessed by a linear model). (ZIP) [file pone.0167944.s007.zip › S2_File/L102317_real_dat.png]

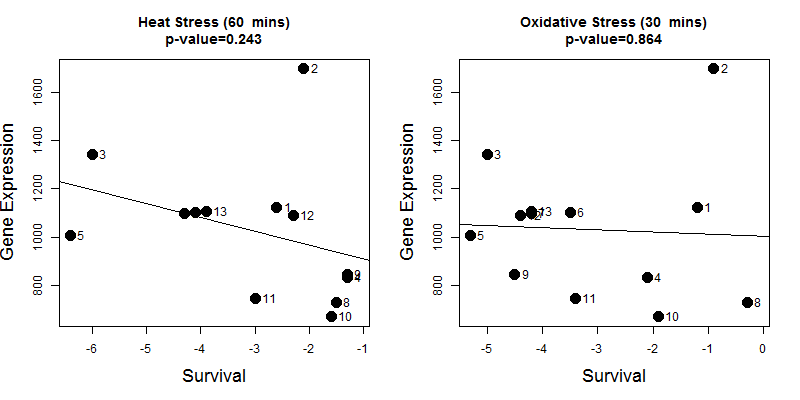

Supplement: S2 File — Expression levels of genes L75676 –L1889726 plotted against survival after 60 minutes heat and 30 min oxidative stress. Survival is expressed as the difference of log CFU/ml after stress and before stress. Numbers indicate fermentations as presented in Table 1. P-values above the plots indicate significance of correlation (assessed by a linear model). (ZIP) [file pone.0167944.s007.zip › S2_File/L102360_real_dat.png]

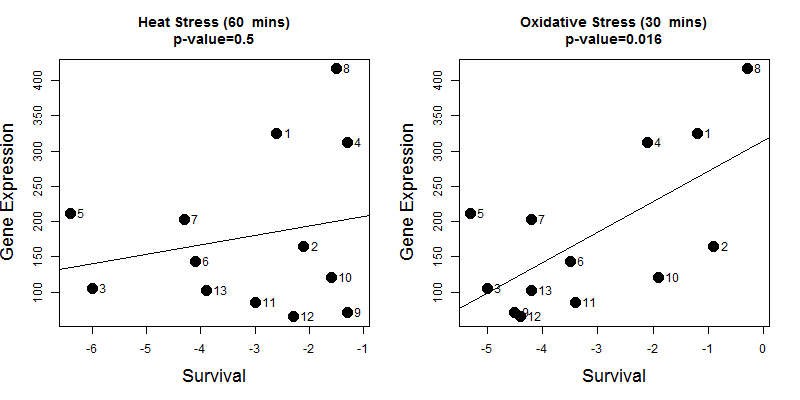

Supplement: S2 File — Expression levels of genes L75676 –L1889726 plotted against survival after 60 minutes heat and 30 min oxidative stress. Survival is expressed as the difference of log CFU/ml after stress and before stress. Numbers indicate fermentations as presented in Table 1. P-values above the plots indicate significance of correlation (assessed by a linear model). (ZIP) [file pone.0167944.s007.zip › S2_File/L102412_real_dat.png]

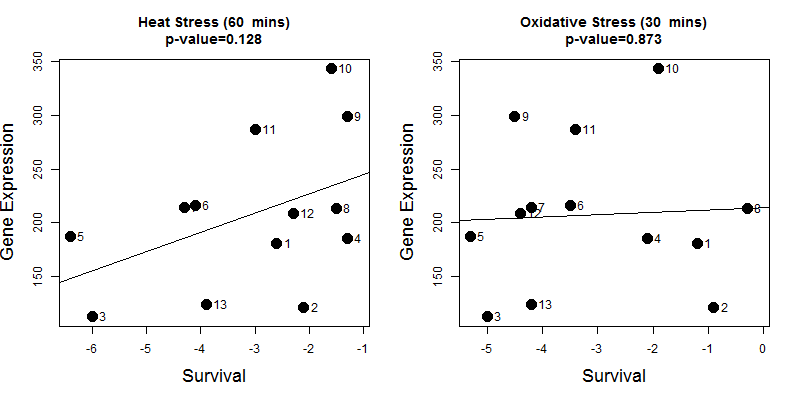

Supplement: S2 File — Expression levels of genes L75676 –L1889726 plotted against survival after 60 minutes heat and 30 min oxidative stress. Survival is expressed as the difference of log CFU/ml after stress and before stress. Numbers indicate fermentations as presented in Table 1. P-values above the plots indicate significance of correlation (assessed by a linear model). (ZIP) [file pone.0167944.s007.zip › S2_File/L102491_real_dat.png]

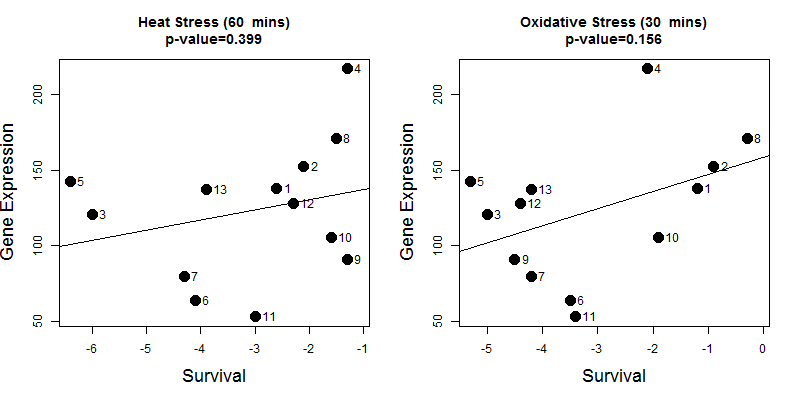

Supplement: S2 File — Expression levels of genes L75676 –L1889726 plotted against survival after 60 minutes heat and 30 min oxidative stress. Survival is expressed as the difference of log CFU/ml after stress and before stress. Numbers indicate fermentations as presented in Table 1. P-values above the plots indicate significance of correlation (assessed by a linear model). (ZIP) [file pone.0167944.s007.zip › S2_File/L102634_real_dat.png]

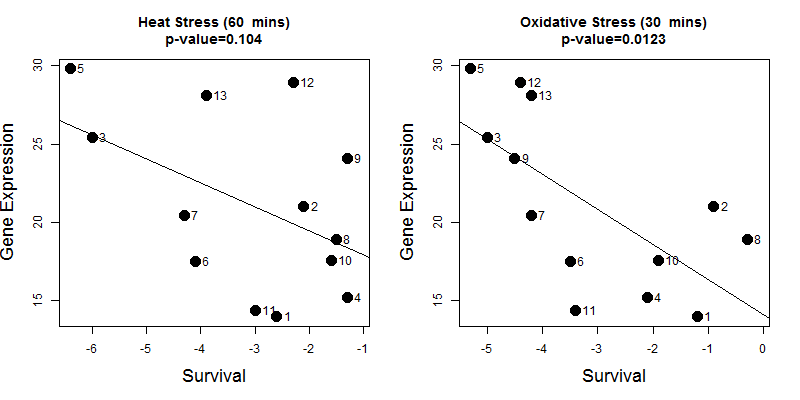

Supplement: S2 File — Expression levels of genes L75676 –L1889726 plotted against survival after 60 minutes heat and 30 min oxidative stress. Survival is expressed as the difference of log CFU/ml after stress and before stress. Numbers indicate fermentations as presented in Table 1. P-values above the plots indicate significance of correlation (assessed by a linear model). (ZIP) [file pone.0167944.s007.zip › S2_File/L102735_real_dat.png]

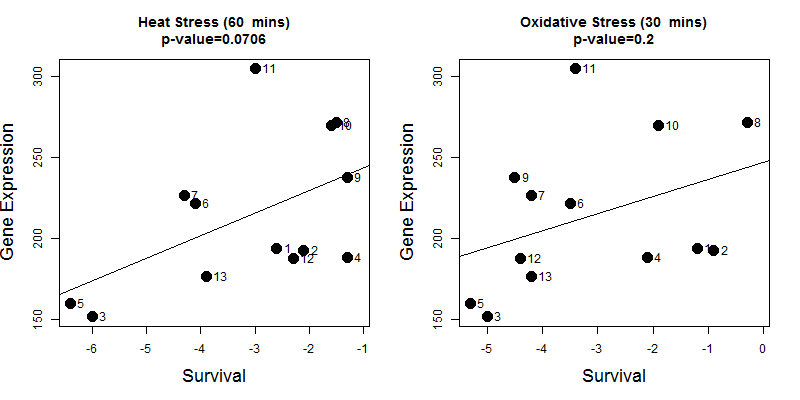

Supplement: S2 File — Expression levels of genes L75676 –L1889726 plotted against survival after 60 minutes heat and 30 min oxidative stress. Survival is expressed as the difference of log CFU/ml after stress and before stress. Numbers indicate fermentations as presented in Table 1. P-values above the plots indicate significance of correlation (assessed by a linear model). (ZIP) [file pone.0167944.s007.zip › S2_File/L102972_real_dat.png]

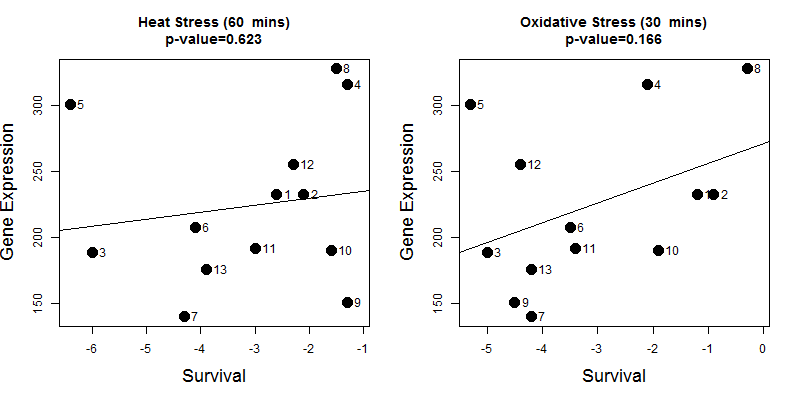

Supplement: S2 File — Expression levels of genes L75676 –L1889726 plotted against survival after 60 minutes heat and 30 min oxidative stress. Survival is expressed as the difference of log CFU/ml after stress and before stress. Numbers indicate fermentations as presented in Table 1. P-values above the plots indicate significance of correlation (assessed by a linear model). (ZIP) [file pone.0167944.s007.zip › S2_File/L102979_real_dat.png]

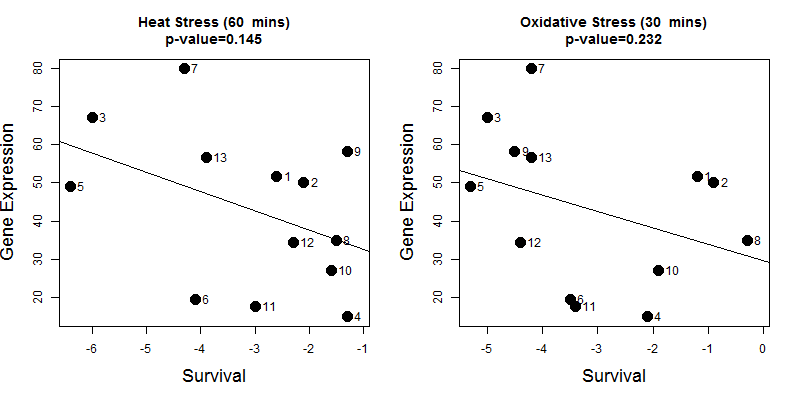

Supplement: S2 File — Expression levels of genes L75676 –L1889726 plotted against survival after 60 minutes heat and 30 min oxidative stress. Survival is expressed as the difference of log CFU/ml after stress and before stress. Numbers indicate fermentations as presented in Table 1. P-values above the plots indicate significance of correlation (assessed by a linear model). (ZIP) [file pone.0167944.s007.zip › S2_File/L103086_real_dat.png]

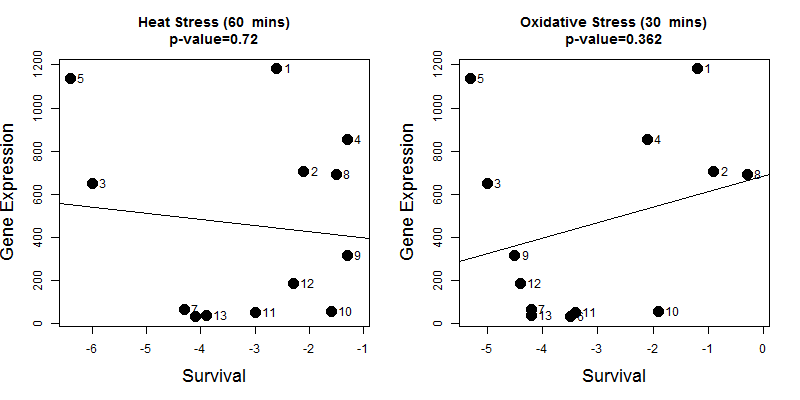

Supplement: S2 File — Expression levels of genes L75676 –L1889726 plotted against survival after 60 minutes heat and 30 min oxidative stress. Survival is expressed as the difference of log CFU/ml after stress and before stress. Numbers indicate fermentations as presented in Table 1. P-values above the plots indicate significance of correlation (assessed by a linear model). (ZIP) [file pone.0167944.s007.zip › S2_File/L103195_real_dat.png]

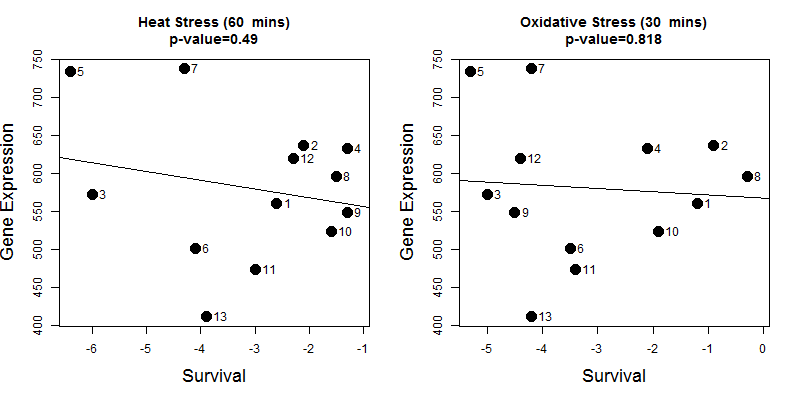

Supplement: S2 File — Expression levels of genes L75676 –L1889726 plotted against survival after 60 minutes heat and 30 min oxidative stress. Survival is expressed as the difference of log CFU/ml after stress and before stress. Numbers indicate fermentations as presented in Table 1. P-values above the plots indicate significance of correlation (assessed by a linear model). (ZIP) [file pone.0167944.s007.zip › S2_File/L103212_real_dat.png]

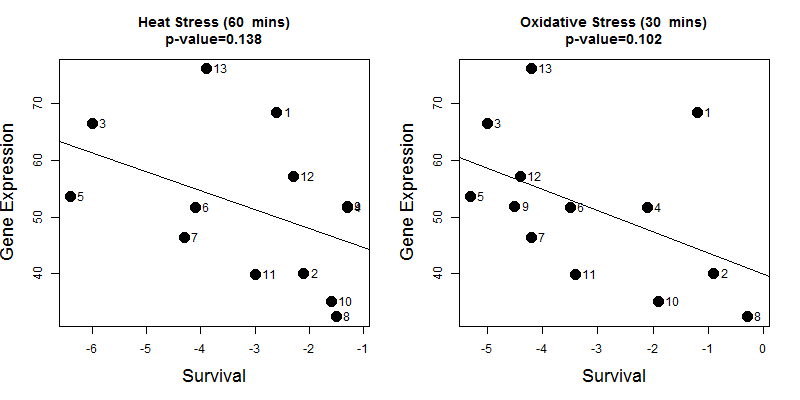

Supplement: S2 File — Expression levels of genes L75676 –L1889726 plotted against survival after 60 minutes heat and 30 min oxidative stress. Survival is expressed as the difference of log CFU/ml after stress and before stress. Numbers indicate fermentations as presented in Table 1. P-values above the plots indicate significance of correlation (assessed by a linear model). (ZIP) [file pone.0167944.s007.zip › S2_File/L103246_real_dat.png]

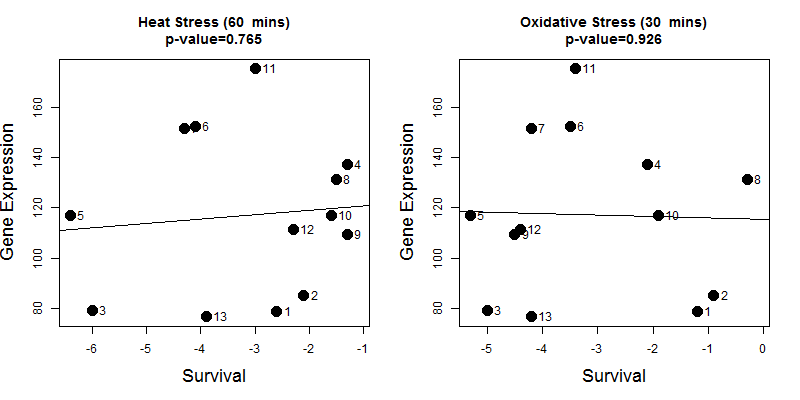

Supplement: S2 File — Expression levels of genes L75676 –L1889726 plotted against survival after 60 minutes heat and 30 min oxidative stress. Survival is expressed as the difference of log CFU/ml after stress and before stress. Numbers indicate fermentations as presented in Table 1. P-values above the plots indicate significance of correlation (assessed by a linear model). (ZIP) [file pone.0167944.s007.zip › S2_File/L103321_real_dat.png]

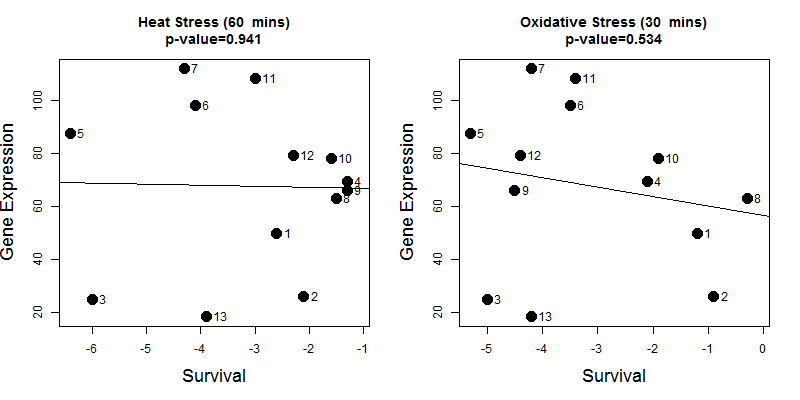

Supplement: S2 File — Expression levels of genes L75676 –L1889726 plotted against survival after 60 minutes heat and 30 min oxidative stress. Survival is expressed as the difference of log CFU/ml after stress and before stress. Numbers indicate fermentations as presented in Table 1. P-values above the plots indicate significance of correlation (assessed by a linear model). (ZIP) [file pone.0167944.s007.zip › S2_File/L103502_real_dat.png]

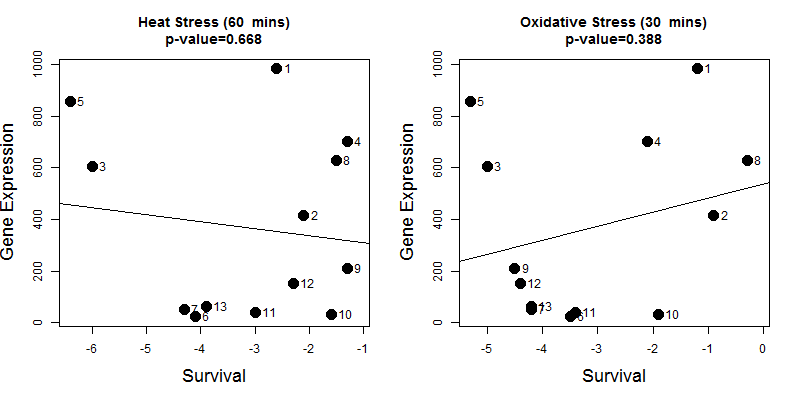

Supplement: S2 File — Expression levels of genes L75676 –L1889726 plotted against survival after 60 minutes heat and 30 min oxidative stress. Survival is expressed as the difference of log CFU/ml after stress and before stress. Numbers indicate fermentations as presented in Table 1. P-values above the plots indicate significance of correlation (assessed by a linear model). (ZIP) [file pone.0167944.s007.zip › S2_File/L103652_real_dat.png]

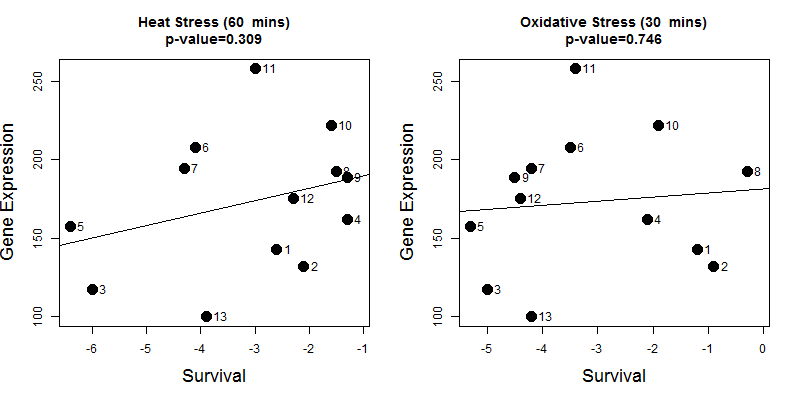

Supplement: S2 File — Expression levels of genes L75676 –L1889726 plotted against survival after 60 minutes heat and 30 min oxidative stress. Survival is expressed as the difference of log CFU/ml after stress and before stress. Numbers indicate fermentations as presented in Table 1. P-values above the plots indicate significance of correlation (assessed by a linear model). (ZIP) [file pone.0167944.s007.zip › S2_File/L103661_real_dat.png]

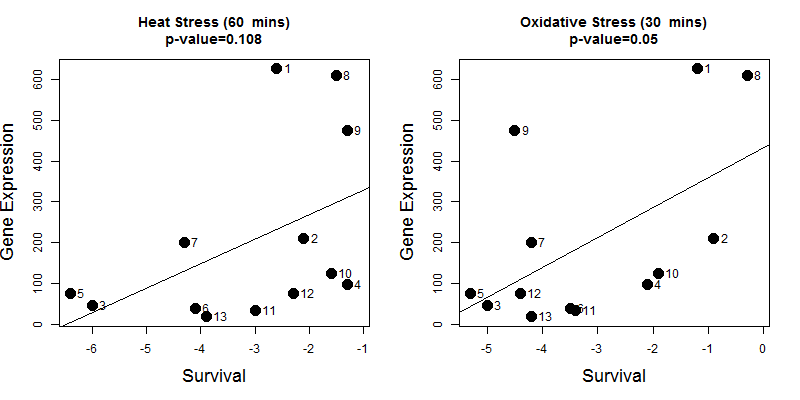

Supplement: S2 File — Expression levels of genes L75676 –L1889726 plotted against survival after 60 minutes heat and 30 min oxidative stress. Survival is expressed as the difference of log CFU/ml after stress and before stress. Numbers indicate fermentations as presented in Table 1. P-values above the plots indicate significance of correlation (assessed by a linear model). (ZIP) [file pone.0167944.s007.zip › S2_File/L103741_real_dat.png]

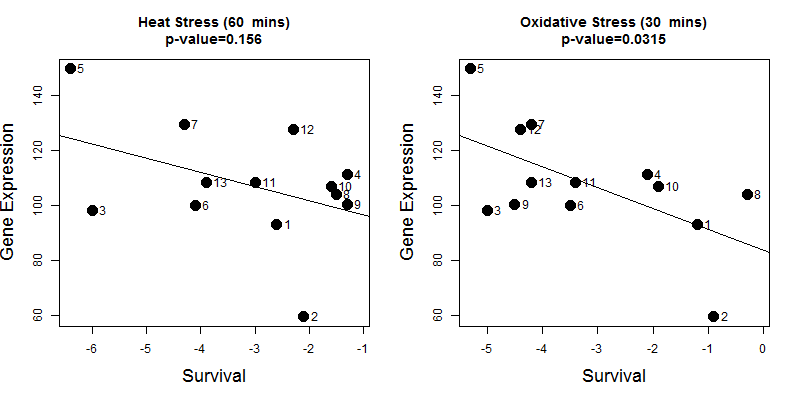

Supplement: S2 File — Expression levels of genes L75676 –L1889726 plotted against survival after 60 minutes heat and 30 min oxidative stress. Survival is expressed as the difference of log CFU/ml after stress and before stress. Numbers indicate fermentations as presented in Table 1. P-values above the plots indicate significance of correlation (assessed by a linear model). (ZIP) [file pone.0167944.s007.zip › S2_File/L104007_real_dat.png]

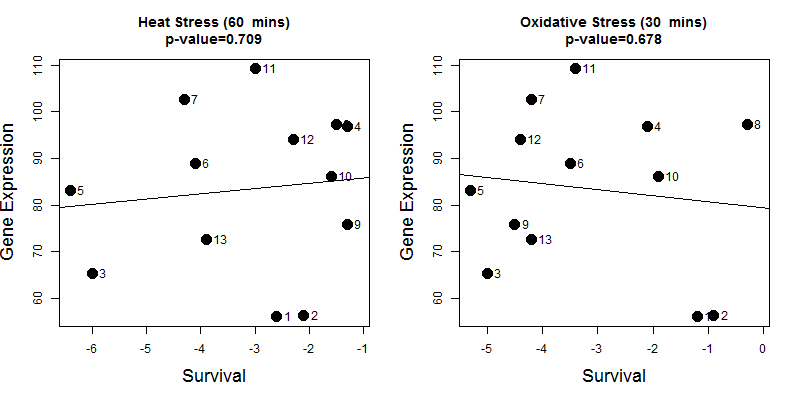

Supplement: S2 File — Expression levels of genes L75676 –L1889726 plotted against survival after 60 minutes heat and 30 min oxidative stress. Survival is expressed as the difference of log CFU/ml after stress and before stress. Numbers indicate fermentations as presented in Table 1. P-values above the plots indicate significance of correlation (assessed by a linear model). (ZIP) [file pone.0167944.s007.zip › S2_File/L104021_real_dat.png]

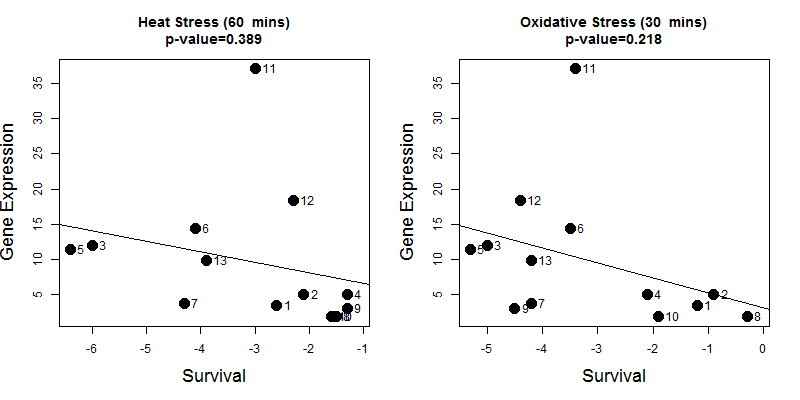

Supplement: S2 File — Expression levels of genes L75676 –L1889726 plotted against survival after 60 minutes heat and 30 min oxidative stress. Survival is expressed as the difference of log CFU/ml after stress and before stress. Numbers indicate fermentations as presented in Table 1. P-values above the plots indicate significance of correlation (assessed by a linear model). (ZIP) [file pone.0167944.s007.zip › S2_File/L104065_real_dat.png]

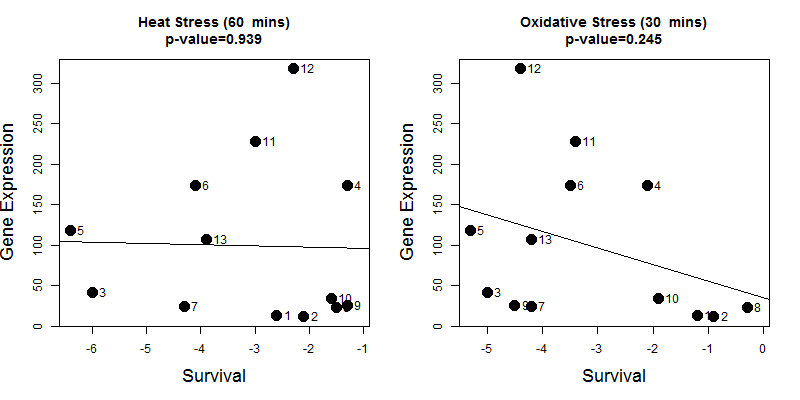

Supplement: S2 File — Expression levels of genes L75676 –L1889726 plotted against survival after 60 minutes heat and 30 min oxidative stress. Survival is expressed as the difference of log CFU/ml after stress and before stress. Numbers indicate fermentations as presented in Table 1. P-values above the plots indicate significance of correlation (assessed by a linear model). (ZIP) [file pone.0167944.s007.zip › S2_File/L104115_real_dat.png]

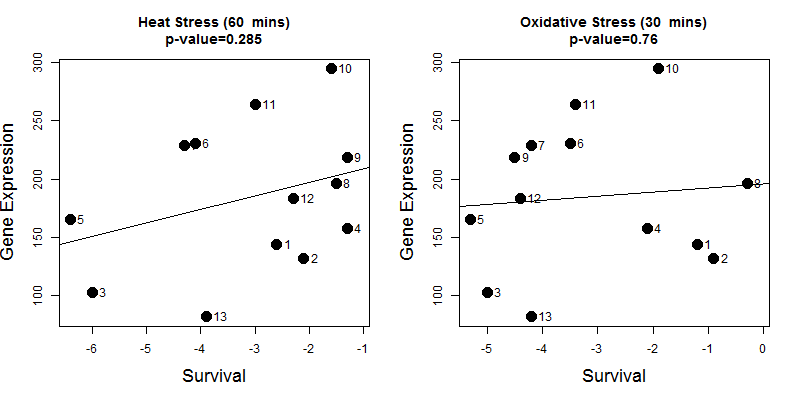

Supplement: S2 File — Expression levels of genes L75676 –L1889726 plotted against survival after 60 minutes heat and 30 min oxidative stress. Survival is expressed as the difference of log CFU/ml after stress and before stress. Numbers indicate fermentations as presented in Table 1. P-values above the plots indicate significance of correlation (assessed by a linear model). (ZIP) [file pone.0167944.s007.zip › S2_File/L104221_real_dat.png]

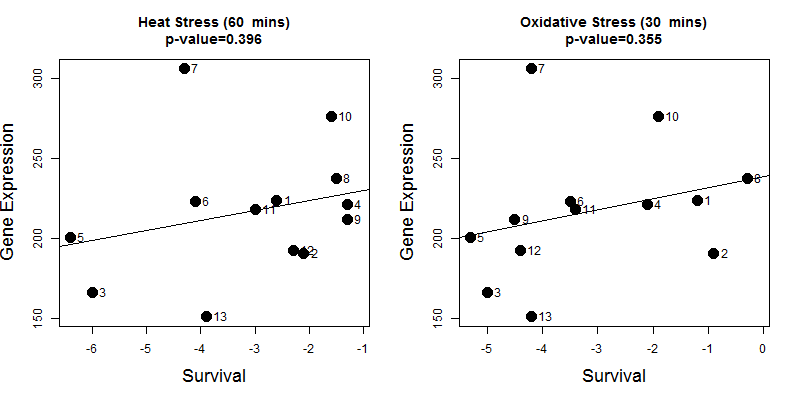

Supplement: S2 File — Expression levels of genes L75676 –L1889726 plotted against survival after 60 minutes heat and 30 min oxidative stress. Survival is expressed as the difference of log CFU/ml after stress and before stress. Numbers indicate fermentations as presented in Table 1. P-values above the plots indicate significance of correlation (assessed by a linear model). (ZIP) [file pone.0167944.s007.zip › S2_File/L104285_real_dat.png]

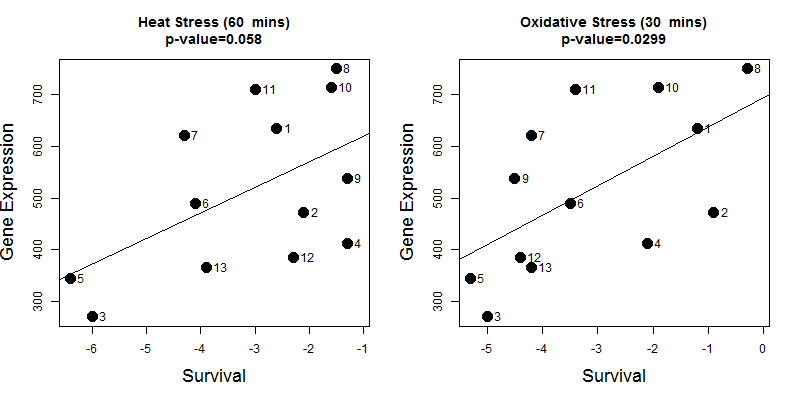

Supplement: S2 File — Expression levels of genes L75676 –L1889726 plotted against survival after 60 minutes heat and 30 min oxidative stress. Survival is expressed as the difference of log CFU/ml after stress and before stress. Numbers indicate fermentations as presented in Table 1. P-values above the plots indicate significance of correlation (assessed by a linear model). (ZIP) [file pone.0167944.s007.zip › S2_File/L104437_real_dat.png]

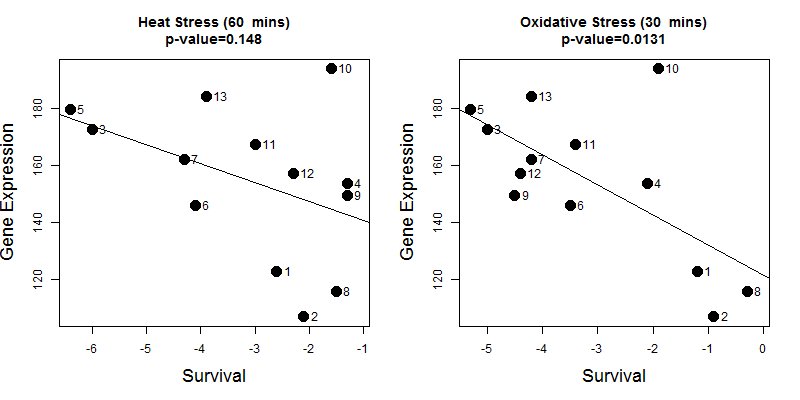

Supplement: S2 File — Expression levels of genes L75676 –L1889726 plotted against survival after 60 minutes heat and 30 min oxidative stress. Survival is expressed as the difference of log CFU/ml after stress and before stress. Numbers indicate fermentations as presented in Table 1. P-values above the plots indicate significance of correlation (assessed by a linear model). (ZIP) [file pone.0167944.s007.zip › S2_File/L104552_real_dat.png]

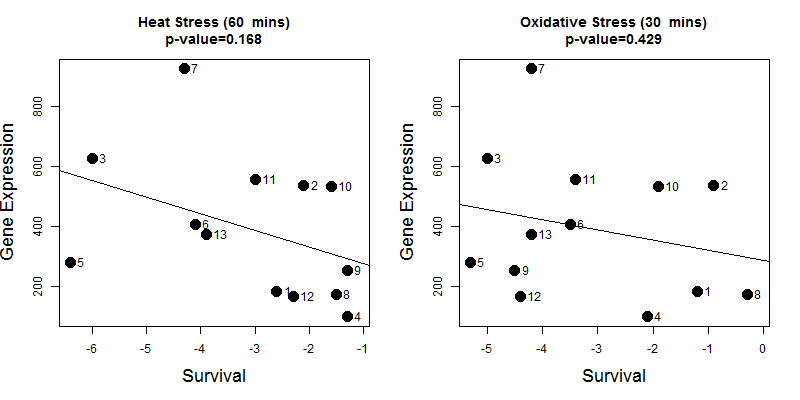

Supplement: S2 File — Expression levels of genes L75676 –L1889726 plotted against survival after 60 minutes heat and 30 min oxidative stress. Survival is expressed as the difference of log CFU/ml after stress and before stress. Numbers indicate fermentations as presented in Table 1. P-values above the plots indicate significance of correlation (assessed by a linear model). (ZIP) [file pone.0167944.s007.zip › S2_File/L104681_real_dat.png]

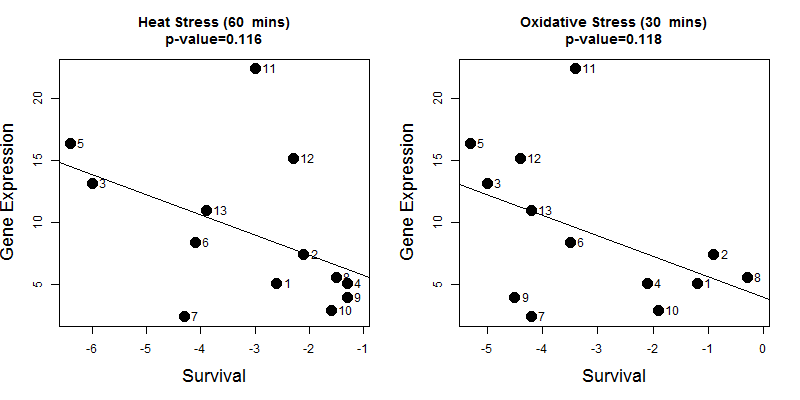

Supplement: S2 File — Expression levels of genes L75676 –L1889726 plotted against survival after 60 minutes heat and 30 min oxidative stress. Survival is expressed as the difference of log CFU/ml after stress and before stress. Numbers indicate fermentations as presented in Table 1. P-values above the plots indicate significance of correlation (assessed by a linear model). (ZIP) [file pone.0167944.s007.zip › S2_File/L104745_real_dat.png]

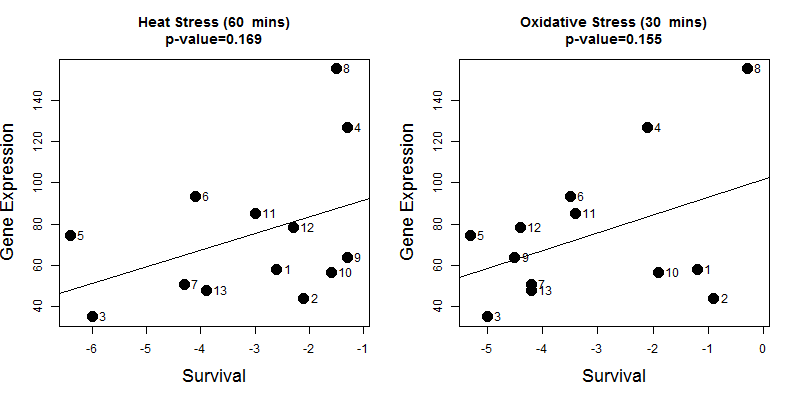

Supplement: S2 File — Expression levels of genes L75676 –L1889726 plotted against survival after 60 minutes heat and 30 min oxidative stress. Survival is expressed as the difference of log CFU/ml after stress and before stress. Numbers indicate fermentations as presented in Table 1. P-values above the plots indicate significance of correlation (assessed by a linear model). (ZIP) [file pone.0167944.s007.zip › S2_File/L104789_real_dat.png]

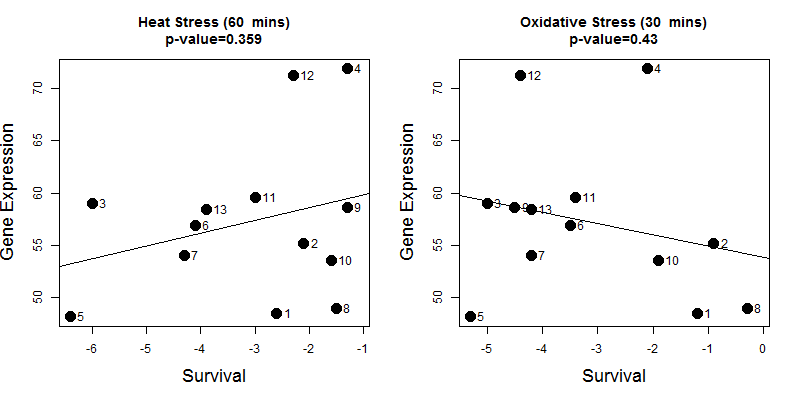

Supplement: S2 File — Expression levels of genes L75676 –L1889726 plotted against survival after 60 minutes heat and 30 min oxidative stress. Survival is expressed as the difference of log CFU/ml after stress and before stress. Numbers indicate fermentations as presented in Table 1. P-values above the plots indicate significance of correlation (assessed by a linear model). (ZIP) [file pone.0167944.s007.zip › S2_File/L104895_real_dat.png]

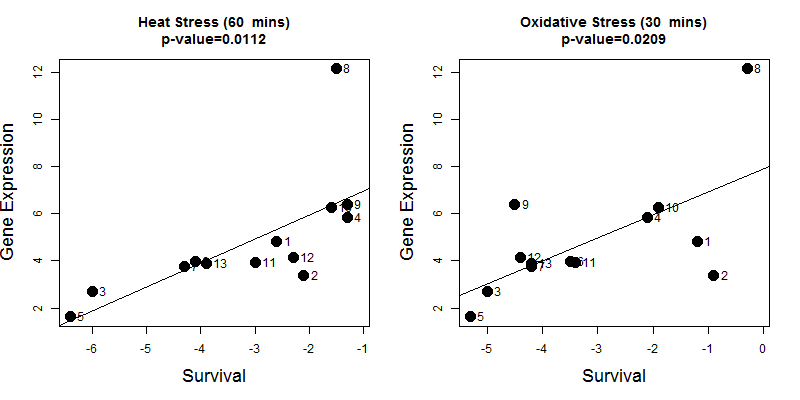

Supplement: S2 File — Expression levels of genes L75676 –L1889726 plotted against survival after 60 minutes heat and 30 min oxidative stress. Survival is expressed as the difference of log CFU/ml after stress and before stress. Numbers indicate fermentations as presented in Table 1. P-values above the plots indicate significance of correlation (assessed by a linear model). (ZIP) [file pone.0167944.s007.zip › S2_File/L104969_real_dat.png]

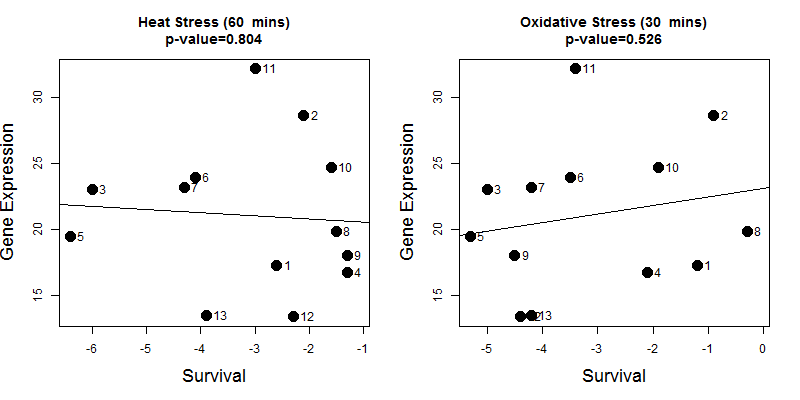

Supplement: S2 File — Expression levels of genes L75676 –L1889726 plotted against survival after 60 minutes heat and 30 min oxidative stress. Survival is expressed as the difference of log CFU/ml after stress and before stress. Numbers indicate fermentations as presented in Table 1. P-values above the plots indicate significance of correlation (assessed by a linear model). (ZIP) [file pone.0167944.s007.zip › S2_File/L105113_real_dat.png]

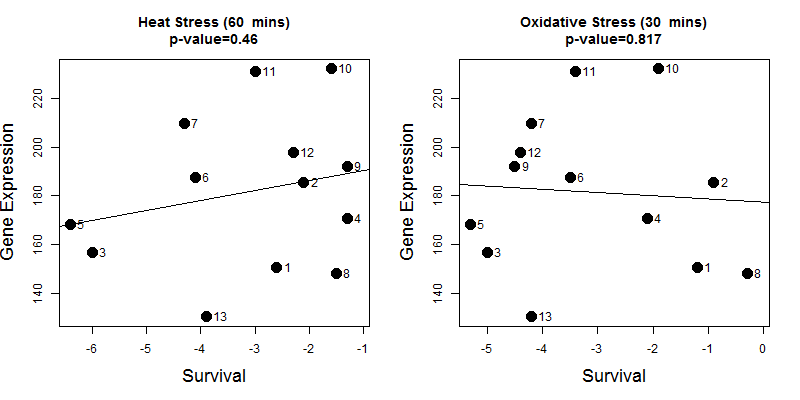

Supplement: S2 File — Expression levels of genes L75676 –L1889726 plotted against survival after 60 minutes heat and 30 min oxidative stress. Survival is expressed as the difference of log CFU/ml after stress and before stress. Numbers indicate fermentations as presented in Table 1. P-values above the plots indicate significance of correlation (assessed by a linear model). (ZIP) [file pone.0167944.s007.zip › S2_File/L105256_real_dat.png]

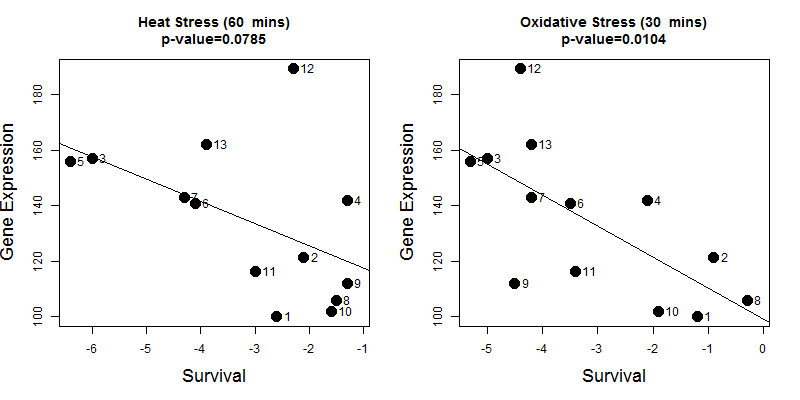

Supplement: S2 File — Expression levels of genes L75676 –L1889726 plotted against survival after 60 minutes heat and 30 min oxidative stress. Survival is expressed as the difference of log CFU/ml after stress and before stress. Numbers indicate fermentations as presented in Table 1. P-values above the plots indicate significance of correlation (assessed by a linear model). (ZIP) [file pone.0167944.s007.zip › S2_File/L105494_real_dat.png]

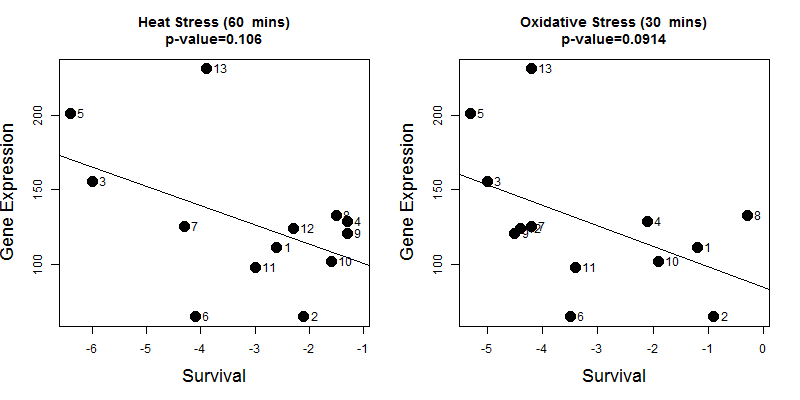

Supplement: S2 File — Expression levels of genes L75676 –L1889726 plotted against survival after 60 minutes heat and 30 min oxidative stress. Survival is expressed as the difference of log CFU/ml after stress and before stress. Numbers indicate fermentations as presented in Table 1. P-values above the plots indicate significance of correlation (assessed by a linear model). (ZIP) [file pone.0167944.s007.zip › S2_File/L105533_real_dat.png]

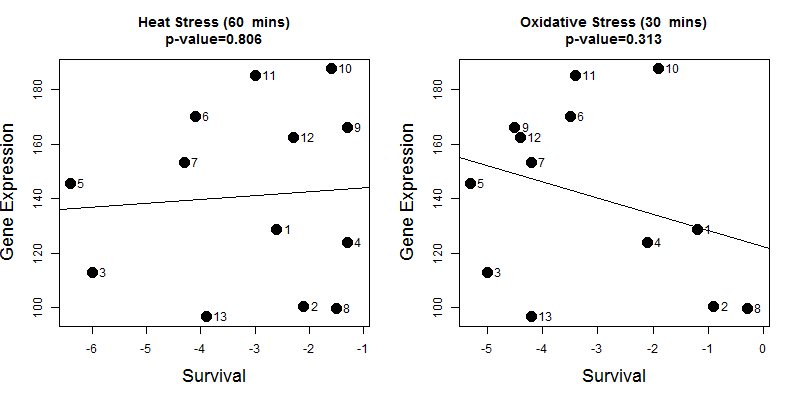

Supplement: S2 File — Expression levels of genes L75676 –L1889726 plotted against survival after 60 minutes heat and 30 min oxidative stress. Survival is expressed as the difference of log CFU/ml after stress and before stress. Numbers indicate fermentations as presented in Table 1. P-values above the plots indicate significance of correlation (assessed by a linear model). (ZIP) [file pone.0167944.s007.zip › S2_File/L106031_real_dat.png]

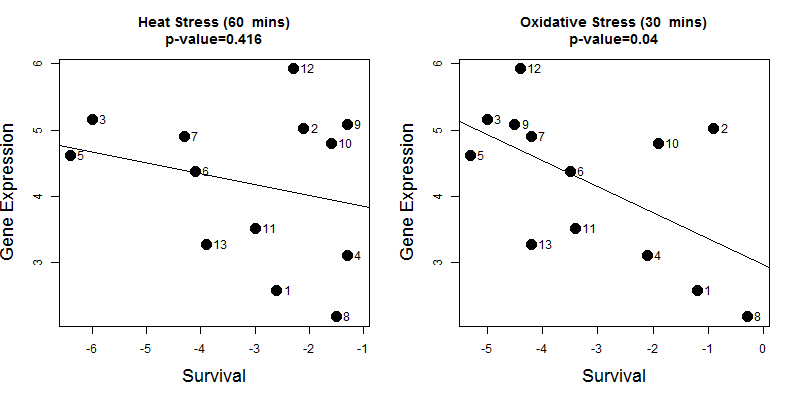

Supplement: S2 File — Expression levels of genes L75676 –L1889726 plotted against survival after 60 minutes heat and 30 min oxidative stress. Survival is expressed as the difference of log CFU/ml after stress and before stress. Numbers indicate fermentations as presented in Table 1. P-values above the plots indicate significance of correlation (assessed by a linear model). (ZIP) [file pone.0167944.s007.zip › S2_File/L106083_real_dat.png]

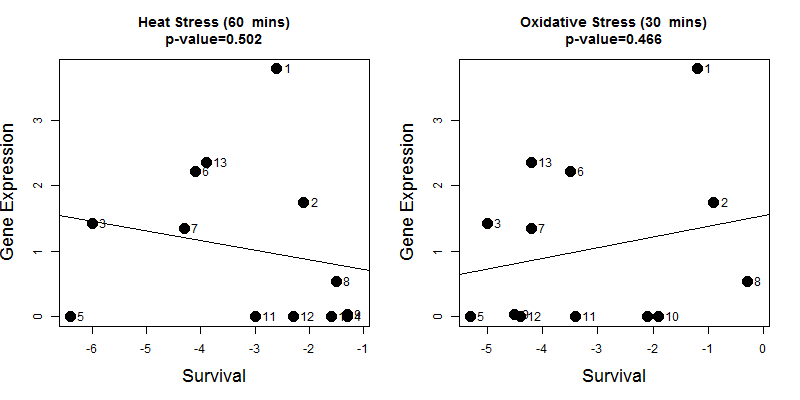

Supplement: S2 File — Expression levels of genes L75676 –L1889726 plotted against survival after 60 minutes heat and 30 min oxidative stress. Survival is expressed as the difference of log CFU/ml after stress and before stress. Numbers indicate fermentations as presented in Table 1. P-values above the plots indicate significance of correlation (assessed by a linear model). (ZIP) [file pone.0167944.s007.zip › S2_File/L106117_real_dat.png]

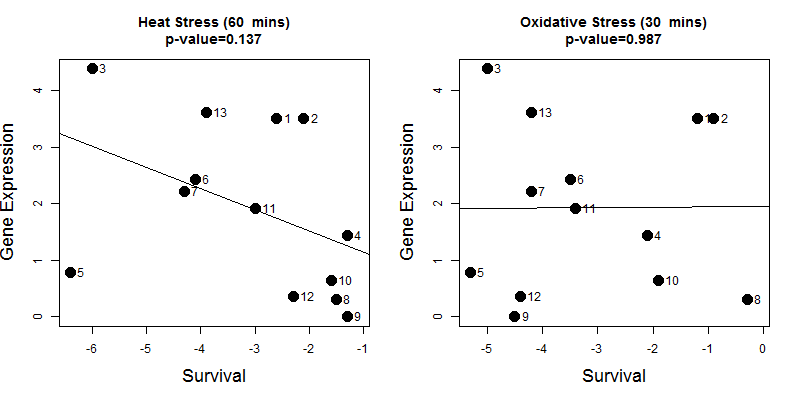

Supplement: S2 File — Expression levels of genes L75676 –L1889726 plotted against survival after 60 minutes heat and 30 min oxidative stress. Survival is expressed as the difference of log CFU/ml after stress and before stress. Numbers indicate fermentations as presented in Table 1. P-values above the plots indicate significance of correlation (assessed by a linear model). (ZIP) [file pone.0167944.s007.zip › S2_File/L106345_real_dat.png]

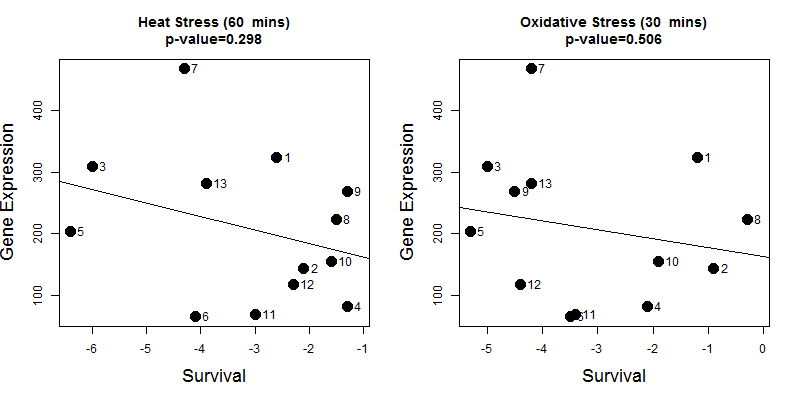

Supplement: S2 File — Expression levels of genes L75676 –L1889726 plotted against survival after 60 minutes heat and 30 min oxidative stress. Survival is expressed as the difference of log CFU/ml after stress and before stress. Numbers indicate fermentations as presented in Table 1. P-values above the plots indicate significance of correlation (assessed by a linear model). (ZIP) [file pone.0167944.s007.zip › S2_File/L106356_real_dat.png]

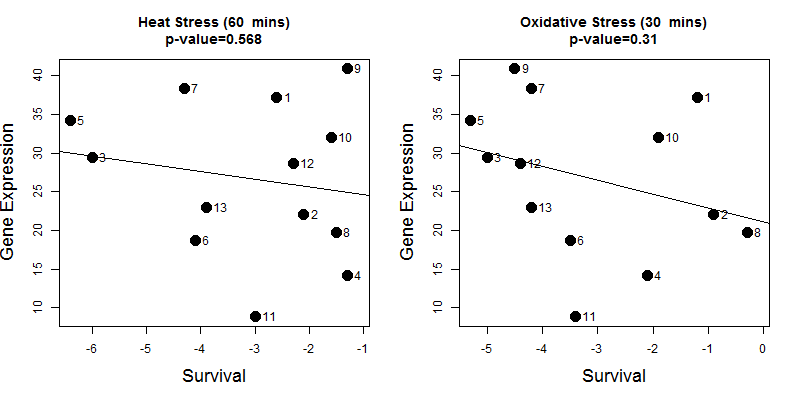

Supplement: S2 File — Expression levels of genes L75676 –L1889726 plotted against survival after 60 minutes heat and 30 min oxidative stress. Survival is expressed as the difference of log CFU/ml after stress and before stress. Numbers indicate fermentations as presented in Table 1. P-values above the plots indicate significance of correlation (assessed by a linear model). (ZIP) [file pone.0167944.s007.zip › S2_File/L106374_real_dat.png]

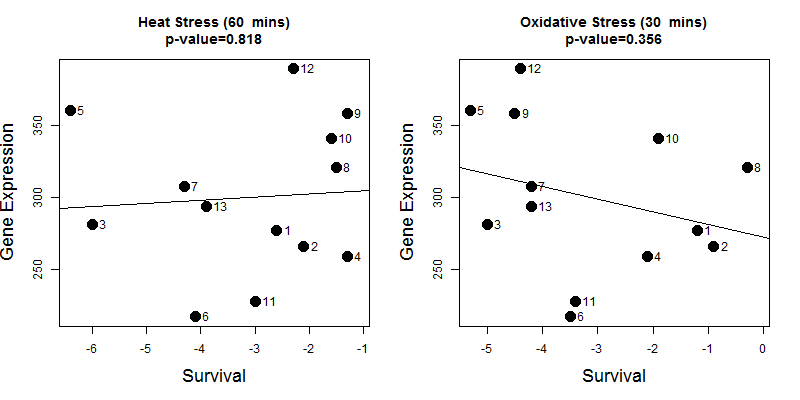

Supplement: S2 File — Expression levels of genes L75676 –L1889726 plotted against survival after 60 minutes heat and 30 min oxidative stress. Survival is expressed as the difference of log CFU/ml after stress and before stress. Numbers indicate fermentations as presented in Table 1. P-values above the plots indicate significance of correlation (assessed by a linear model). (ZIP) [file pone.0167944.s007.zip › S2_File/L106425_real_dat.png]

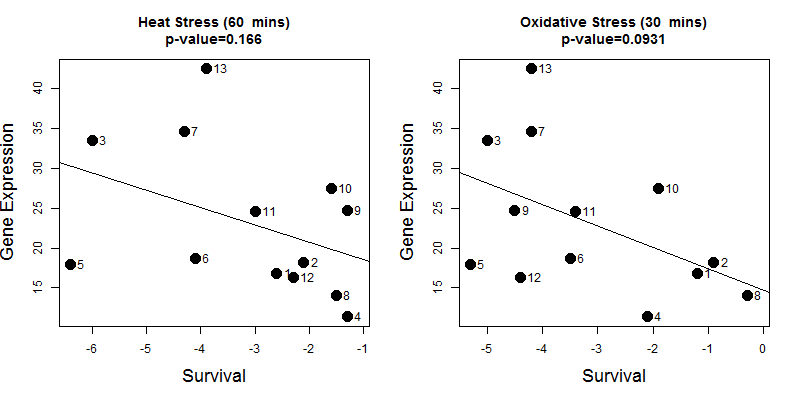

Supplement: S2 File — Expression levels of genes L75676 –L1889726 plotted against survival after 60 minutes heat and 30 min oxidative stress. Survival is expressed as the difference of log CFU/ml after stress and before stress. Numbers indicate fermentations as presented in Table 1. P-values above the plots indicate significance of correlation (assessed by a linear model). (ZIP) [file pone.0167944.s007.zip › S2_File/L106489_real_dat.png]

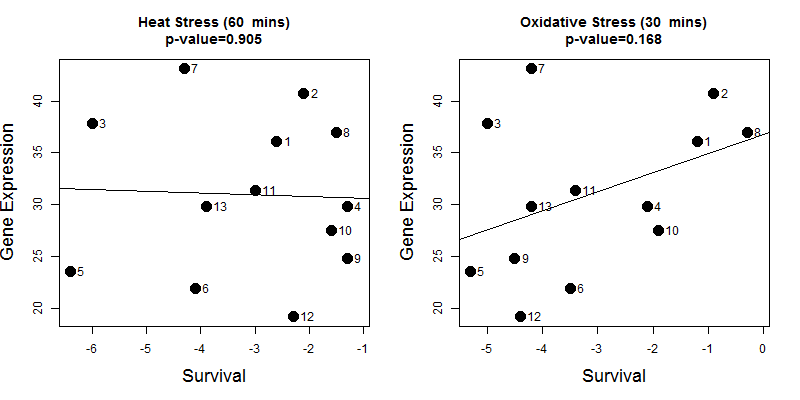

Supplement: S2 File — Expression levels of genes L75676 –L1889726 plotted against survival after 60 minutes heat and 30 min oxidative stress. Survival is expressed as the difference of log CFU/ml after stress and before stress. Numbers indicate fermentations as presented in Table 1. P-values above the plots indicate significance of correlation (assessed by a linear model). (ZIP) [file pone.0167944.s007.zip › S2_File/L106608_real_dat.png]

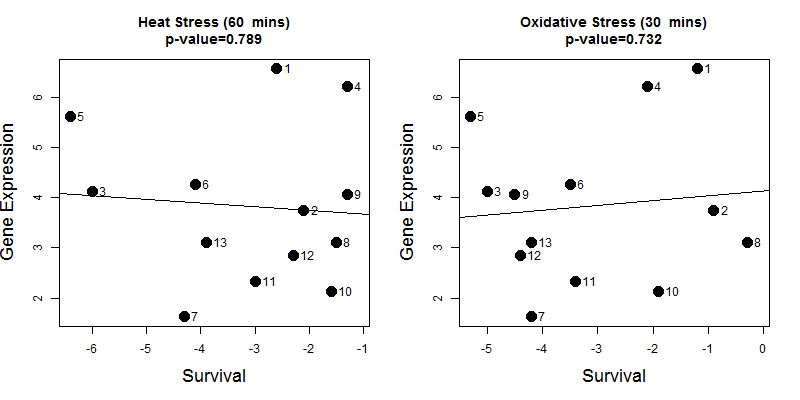

Supplement: S2 File — Expression levels of genes L75676 –L1889726 plotted against survival after 60 minutes heat and 30 min oxidative stress. Survival is expressed as the difference of log CFU/ml after stress and before stress. Numbers indicate fermentations as presented in Table 1. P-values above the plots indicate significance of correlation (assessed by a linear model). (ZIP) [file pone.0167944.s007.zip › S2_File/L106731_real_dat.png]

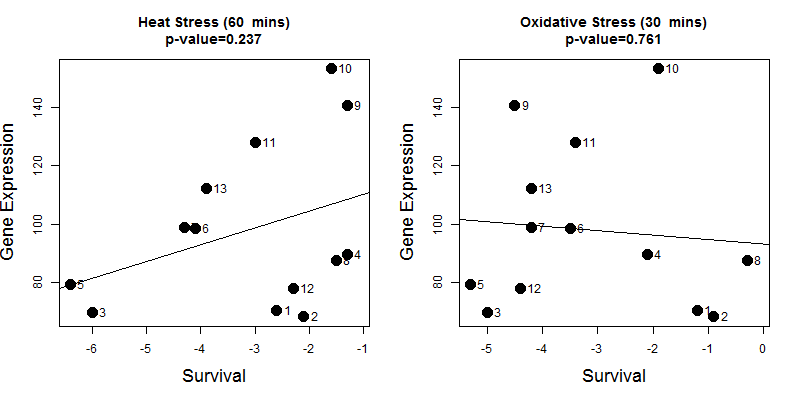

Supplement: S2 File — Expression levels of genes L75676 –L1889726 plotted against survival after 60 minutes heat and 30 min oxidative stress. Survival is expressed as the difference of log CFU/ml after stress and before stress. Numbers indicate fermentations as presented in Table 1. P-values above the plots indicate significance of correlation (assessed by a linear model). (ZIP) [file pone.0167944.s007.zip › S2_File/L106755_real_dat.png]

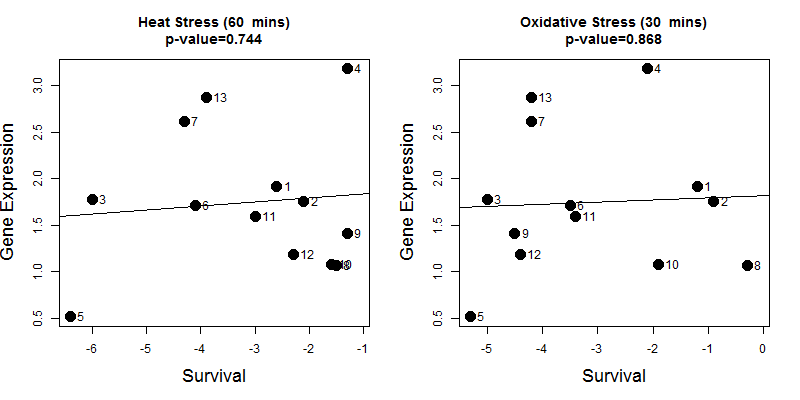

Supplement: S2 File — Expression levels of genes L75676 –L1889726 plotted against survival after 60 minutes heat and 30 min oxidative stress. Survival is expressed as the difference of log CFU/ml after stress and before stress. Numbers indicate fermentations as presented in Table 1. P-values above the plots indicate significance of correlation (assessed by a linear model). (ZIP) [file pone.0167944.s007.zip › S2_File/L106963_real_dat.png]

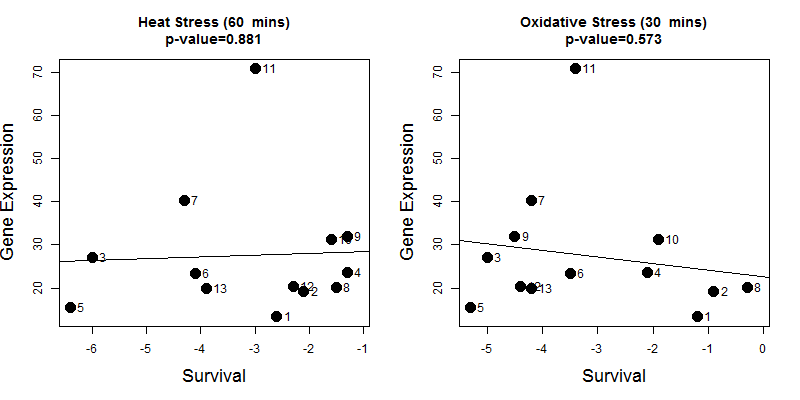

Supplement: S2 File — Expression levels of genes L75676 –L1889726 plotted against survival after 60 minutes heat and 30 min oxidative stress. Survival is expressed as the difference of log CFU/ml after stress and before stress. Numbers indicate fermentations as presented in Table 1. P-values above the plots indicate significance of correlation (assessed by a linear model). (ZIP) [file pone.0167944.s007.zip › S2_File/L106965_real_dat.png]

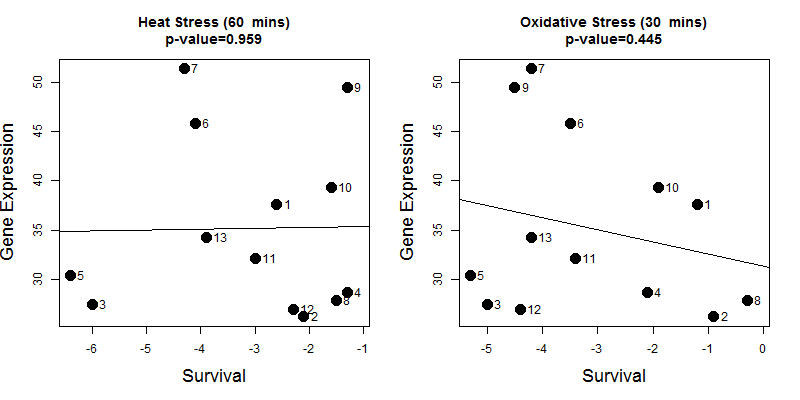

Supplement: S2 File — Expression levels of genes L75676 –L1889726 plotted against survival after 60 minutes heat and 30 min oxidative stress. Survival is expressed as the difference of log CFU/ml after stress and before stress. Numbers indicate fermentations as presented in Table 1. P-values above the plots indicate significance of correlation (assessed by a linear model). (ZIP) [file pone.0167944.s007.zip › S2_File/L107270_real_dat.png]

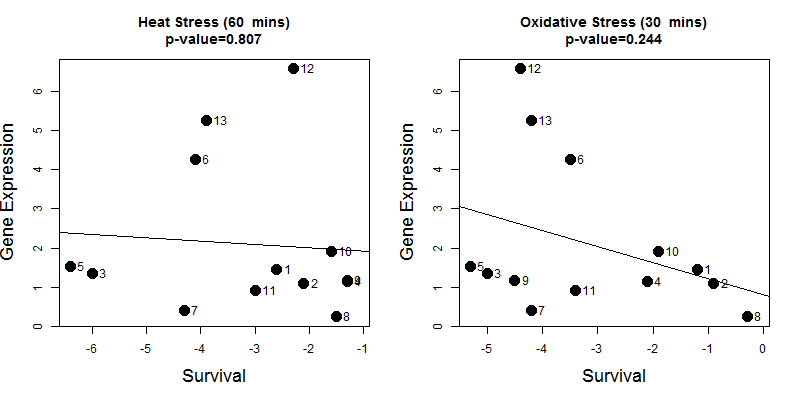

Supplement: S2 File — Expression levels of genes L75676 –L1889726 plotted against survival after 60 minutes heat and 30 min oxidative stress. Survival is expressed as the difference of log CFU/ml after stress and before stress. Numbers indicate fermentations as presented in Table 1. P-values above the plots indicate significance of correlation (assessed by a linear model). (ZIP) [file pone.0167944.s007.zip › S2_File/L107339_real_dat.png]

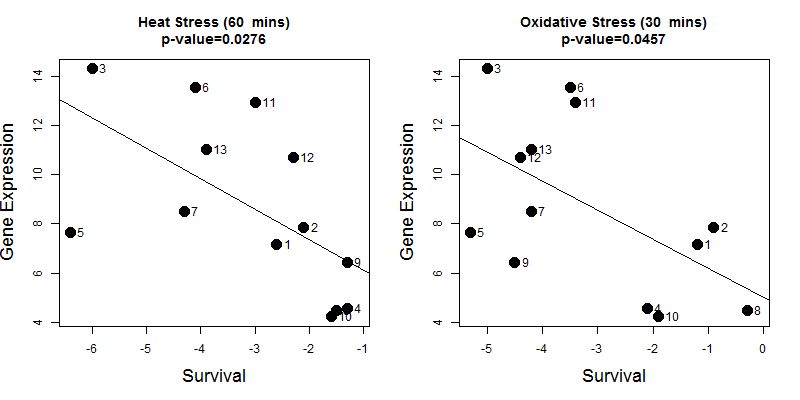

Supplement: S2 File — Expression levels of genes L75676 –L1889726 plotted against survival after 60 minutes heat and 30 min oxidative stress. Survival is expressed as the difference of log CFU/ml after stress and before stress. Numbers indicate fermentations as presented in Table 1. P-values above the plots indicate significance of correlation (assessed by a linear model). (ZIP) [file pone.0167944.s007.zip › S2_File/L107379_real_dat.png]

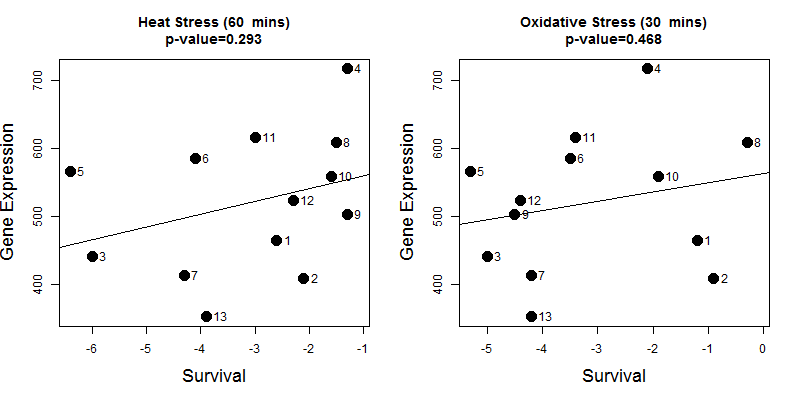

Supplement: S2 File — Expression levels of genes L75676 –L1889726 plotted against survival after 60 minutes heat and 30 min oxidative stress. Survival is expressed as the difference of log CFU/ml after stress and before stress. Numbers indicate fermentations as presented in Table 1. P-values above the plots indicate significance of correlation (assessed by a linear model). (ZIP) [file pone.0167944.s007.zip › S2_File/L107468_real_dat.png]

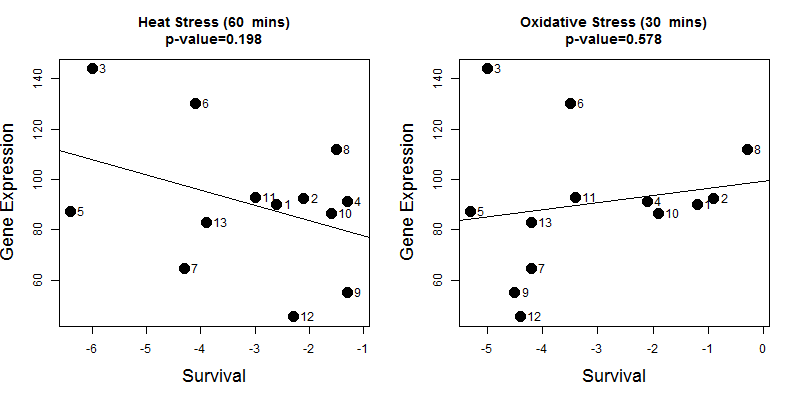

Supplement: S2 File — Expression levels of genes L75676 –L1889726 plotted against survival after 60 minutes heat and 30 min oxidative stress. Survival is expressed as the difference of log CFU/ml after stress and before stress. Numbers indicate fermentations as presented in Table 1. P-values above the plots indicate significance of correlation (assessed by a linear model). (ZIP) [file pone.0167944.s007.zip › S2_File/L107499_real_dat.png]

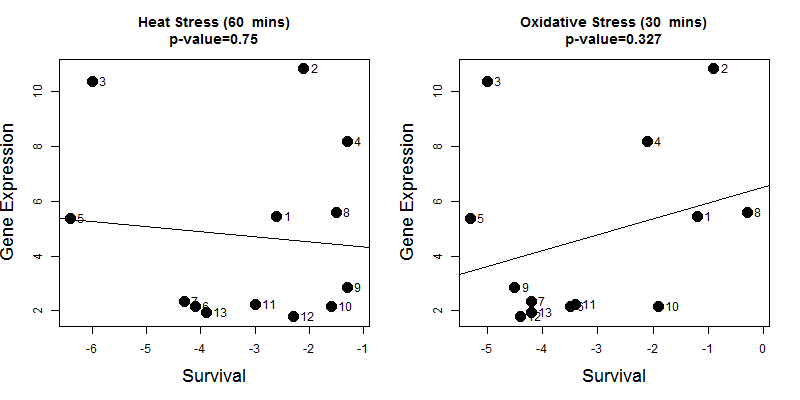

Supplement: S2 File — Expression levels of genes L75676 –L1889726 plotted against survival after 60 minutes heat and 30 min oxidative stress. Survival is expressed as the difference of log CFU/ml after stress and before stress. Numbers indicate fermentations as presented in Table 1. P-values above the plots indicate significance of correlation (assessed by a linear model). (ZIP) [file pone.0167944.s007.zip › S2_File/L107665_real_dat.png]

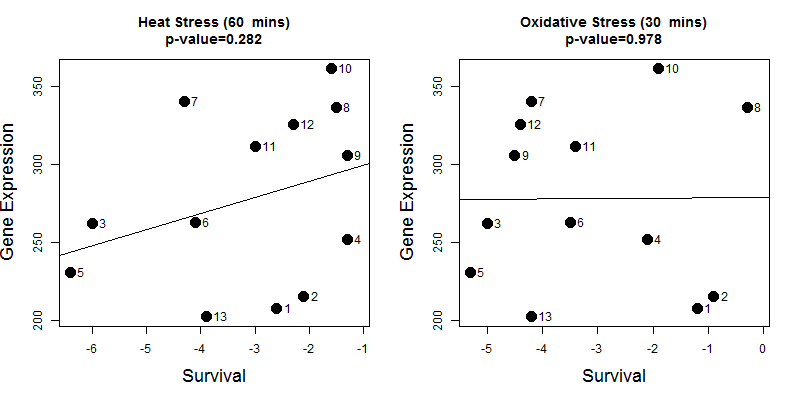

Supplement: S2 File — Expression levels of genes L75676 –L1889726 plotted against survival after 60 minutes heat and 30 min oxidative stress. Survival is expressed as the difference of log CFU/ml after stress and before stress. Numbers indicate fermentations as presented in Table 1. P-values above the plots indicate significance of correlation (assessed by a linear model). (ZIP) [file pone.0167944.s007.zip › S2_File/L107724_real_dat.png]

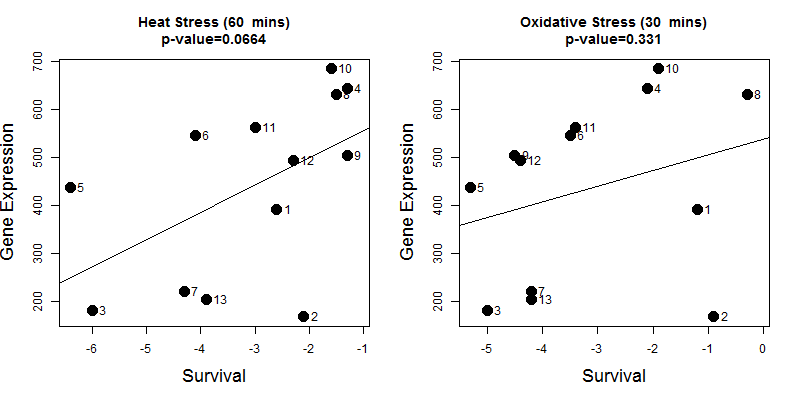

Supplement: S2 File — Expression levels of genes L75676 –L1889726 plotted against survival after 60 minutes heat and 30 min oxidative stress. Survival is expressed as the difference of log CFU/ml after stress and before stress. Numbers indicate fermentations as presented in Table 1. P-values above the plots indicate significance of correlation (assessed by a linear model). (ZIP) [file pone.0167944.s007.zip › S2_File/L107726_real_dat.png]

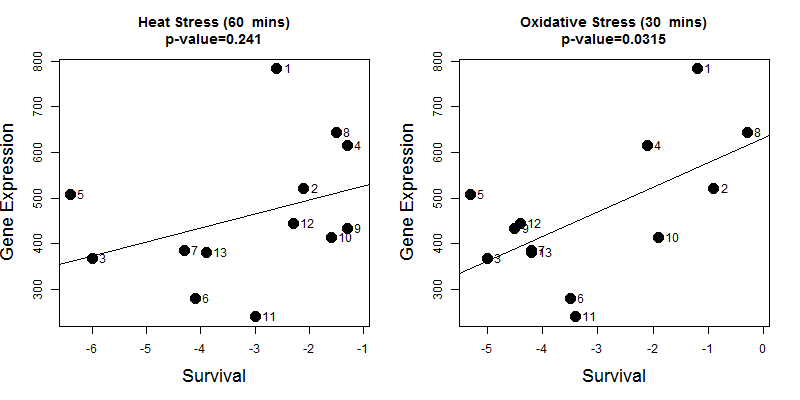

Supplement: S2 File — Expression levels of genes L75676 –L1889726 plotted against survival after 60 minutes heat and 30 min oxidative stress. Survival is expressed as the difference of log CFU/ml after stress and before stress. Numbers indicate fermentations as presented in Table 1. P-values above the plots indicate significance of correlation (assessed by a linear model). (ZIP) [file pone.0167944.s007.zip › S2_File/L107797_real_dat.png]

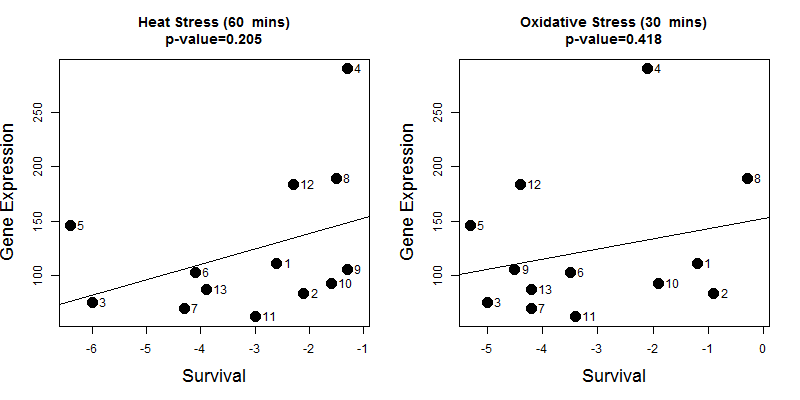

Supplement: S2 File — Expression levels of genes L75676 –L1889726 plotted against survival after 60 minutes heat and 30 min oxidative stress. Survival is expressed as the difference of log CFU/ml after stress and before stress. Numbers indicate fermentations as presented in Table 1. P-values above the plots indicate significance of correlation (assessed by a linear model). (ZIP) [file pone.0167944.s007.zip › S2_File/L107870_real_dat.png]

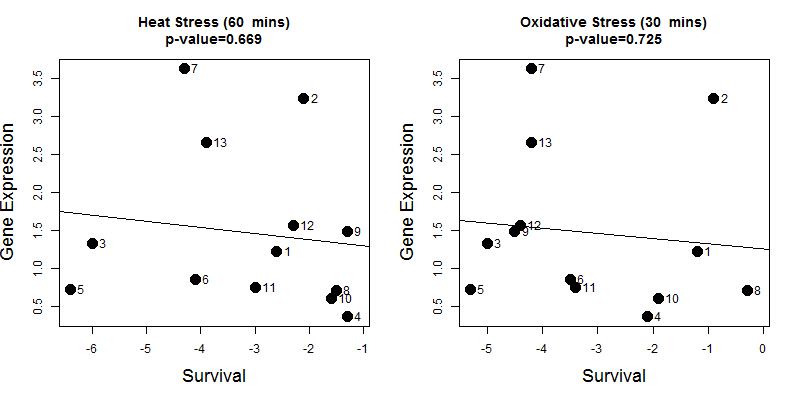

Supplement: S2 File — Expression levels of genes L75676 –L1889726 plotted against survival after 60 minutes heat and 30 min oxidative stress. Survival is expressed as the difference of log CFU/ml after stress and before stress. Numbers indicate fermentations as presented in Table 1. P-values above the plots indicate significance of correlation (assessed by a linear model). (ZIP) [file pone.0167944.s007.zip › S2_File/L107922_real_dat.png]

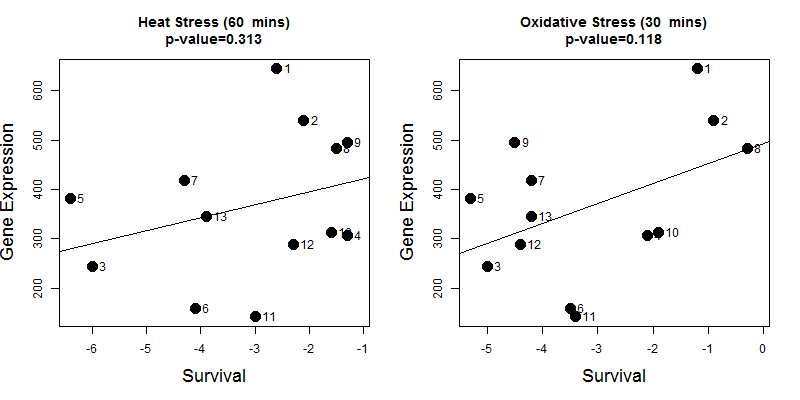

Supplement: S2 File — Expression levels of genes L75676 –L1889726 plotted against survival after 60 minutes heat and 30 min oxidative stress. Survival is expressed as the difference of log CFU/ml after stress and before stress. Numbers indicate fermentations as presented in Table 1. P-values above the plots indicate significance of correlation (assessed by a linear model). (ZIP) [file pone.0167944.s007.zip › S2_File/L108008_real_dat.png]

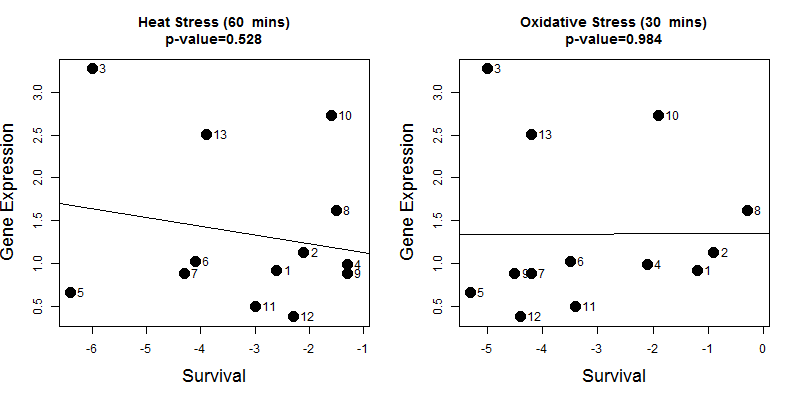

Supplement: S2 File — Expression levels of genes L75676 –L1889726 plotted against survival after 60 minutes heat and 30 min oxidative stress. Survival is expressed as the difference of log CFU/ml after stress and before stress. Numbers indicate fermentations as presented in Table 1. P-values above the plots indicate significance of correlation (assessed by a linear model). (ZIP) [file pone.0167944.s007.zip › S2_File/L108106_real_dat.png]

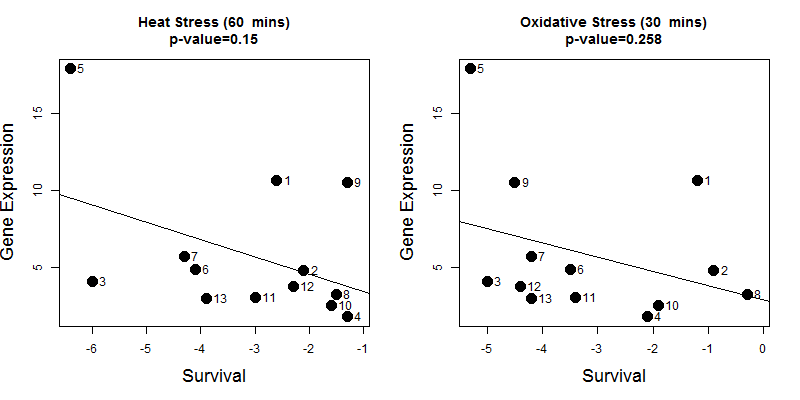

Supplement: S2 File — Expression levels of genes L75676 –L1889726 plotted against survival after 60 minutes heat and 30 min oxidative stress. Survival is expressed as the difference of log CFU/ml after stress and before stress. Numbers indicate fermentations as presented in Table 1. P-values above the plots indicate significance of correlation (assessed by a linear model). (ZIP) [file pone.0167944.s007.zip › S2_File/L108425_real_dat.png]

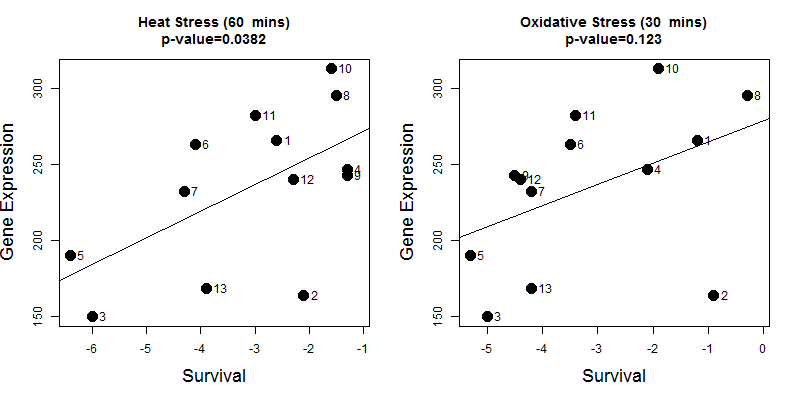

Supplement: S2 File — Expression levels of genes L75676 –L1889726 plotted against survival after 60 minutes heat and 30 min oxidative stress. Survival is expressed as the difference of log CFU/ml after stress and before stress. Numbers indicate fermentations as presented in Table 1. P-values above the plots indicate significance of correlation (assessed by a linear model). (ZIP) [file pone.0167944.s007.zip › S2_File/L108430_real_dat.png]

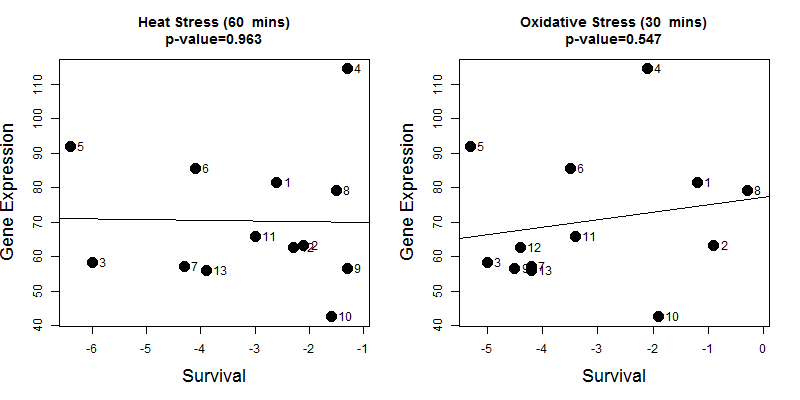

Supplement: S2 File — Expression levels of genes L75676 –L1889726 plotted against survival after 60 minutes heat and 30 min oxidative stress. Survival is expressed as the difference of log CFU/ml after stress and before stress. Numbers indicate fermentations as presented in Table 1. P-values above the plots indicate significance of correlation (assessed by a linear model). (ZIP) [file pone.0167944.s007.zip › S2_File/L108503_real_dat.png]

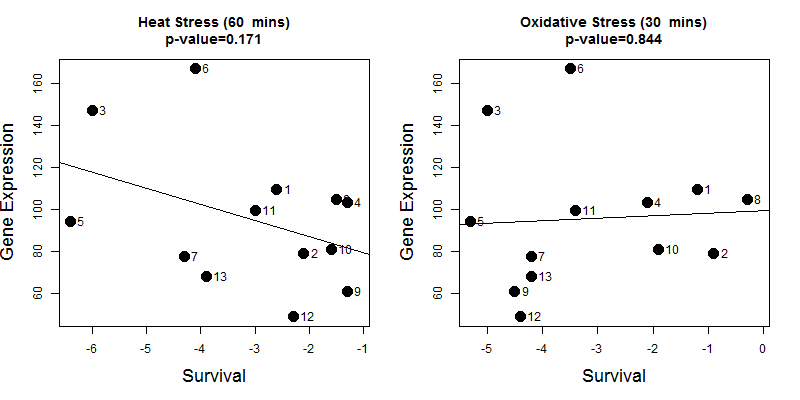

Supplement: S2 File — Expression levels of genes L75676 –L1889726 plotted against survival after 60 minutes heat and 30 min oxidative stress. Survival is expressed as the difference of log CFU/ml after stress and before stress. Numbers indicate fermentations as presented in Table 1. P-values above the plots indicate significance of correlation (assessed by a linear model). (ZIP) [file pone.0167944.s007.zip › S2_File/L108747_real_dat.png]

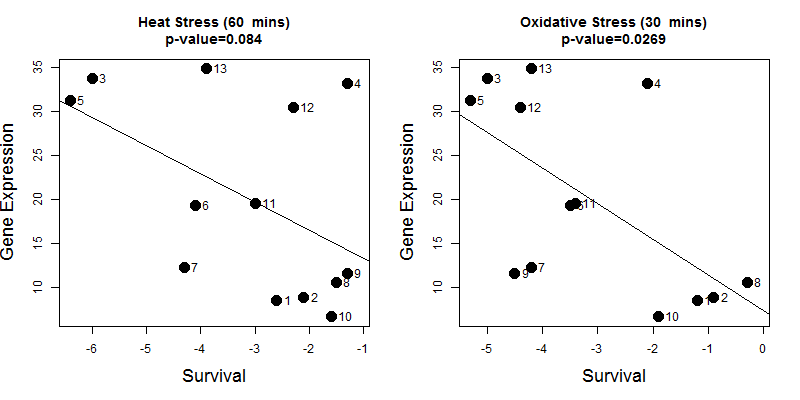

Supplement: S2 File — Expression levels of genes L75676 –L1889726 plotted against survival after 60 minutes heat and 30 min oxidative stress. Survival is expressed as the difference of log CFU/ml after stress and before stress. Numbers indicate fermentations as presented in Table 1. P-values above the plots indicate significance of correlation (assessed by a linear model). (ZIP) [file pone.0167944.s007.zip › S2_File/L108801_real_dat.png]

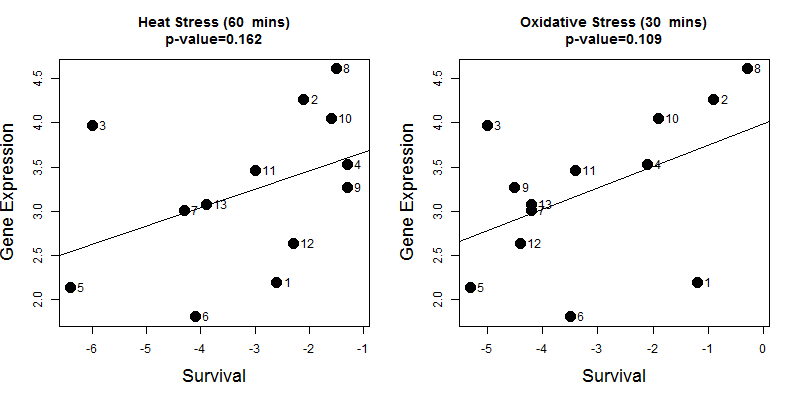

Supplement: S2 File — Expression levels of genes L75676 –L1889726 plotted against survival after 60 minutes heat and 30 min oxidative stress. Survival is expressed as the difference of log CFU/ml after stress and before stress. Numbers indicate fermentations as presented in Table 1. P-values above the plots indicate significance of correlation (assessed by a linear model). (ZIP) [file pone.0167944.s007.zip › S2_File/L108911_real_dat.png]

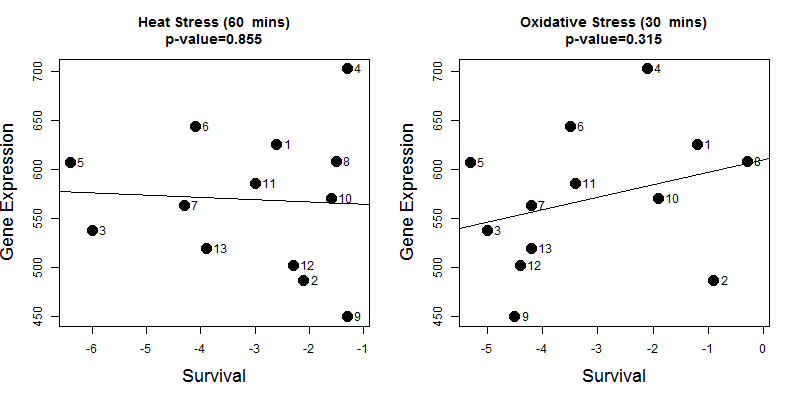

Supplement: S2 File — Expression levels of genes L75676 –L1889726 plotted against survival after 60 minutes heat and 30 min oxidative stress. Survival is expressed as the difference of log CFU/ml after stress and before stress. Numbers indicate fermentations as presented in Table 1. P-values above the plots indicate significance of correlation (assessed by a linear model). (ZIP) [file pone.0167944.s007.zip › S2_File/L108967_real_dat.png]

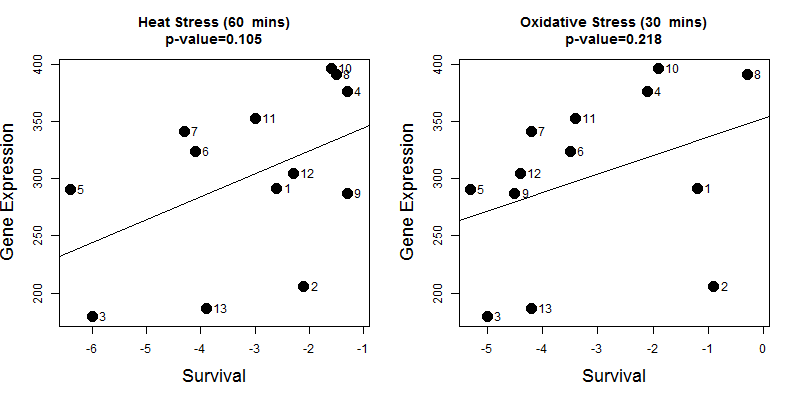

Supplement: S2 File — Expression levels of genes L75676 –L1889726 plotted against survival after 60 minutes heat and 30 min oxidative stress. Survival is expressed as the difference of log CFU/ml after stress and before stress. Numbers indicate fermentations as presented in Table 1. P-values above the plots indicate significance of correlation (assessed by a linear model). (ZIP) [file pone.0167944.s007.zip › S2_File/L108989_real_dat.png]

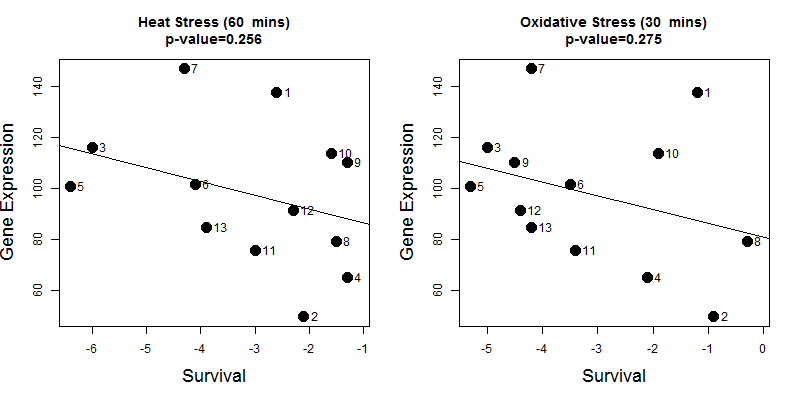

Supplement: S2 File — Expression levels of genes L75676 –L1889726 plotted against survival after 60 minutes heat and 30 min oxidative stress. Survival is expressed as the difference of log CFU/ml after stress and before stress. Numbers indicate fermentations as presented in Table 1. P-values above the plots indicate significance of correlation (assessed by a linear model). (ZIP) [file pone.0167944.s007.zip › S2_File/L108994_real_dat.png]

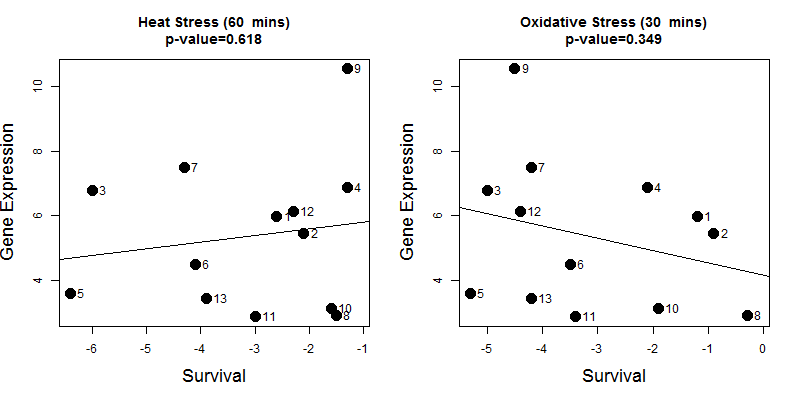

Supplement: S2 File — Expression levels of genes L75676 –L1889726 plotted against survival after 60 minutes heat and 30 min oxidative stress. Survival is expressed as the difference of log CFU/ml after stress and before stress. Numbers indicate fermentations as presented in Table 1. P-values above the plots indicate significance of correlation (assessed by a linear model). (ZIP) [file pone.0167944.s007.zip › S2_File/L109011_real_dat.png]

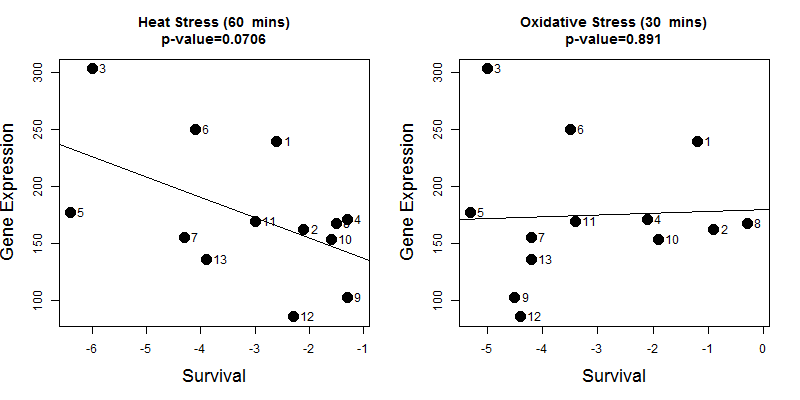

Supplement: S2 File — Expression levels of genes L75676 –L1889726 plotted against survival after 60 minutes heat and 30 min oxidative stress. Survival is expressed as the difference of log CFU/ml after stress and before stress. Numbers indicate fermentations as presented in Table 1. P-values above the plots indicate significance of correlation (assessed by a linear model). (ZIP) [file pone.0167944.s007.zip › S2_File/L109013_real_dat.png]

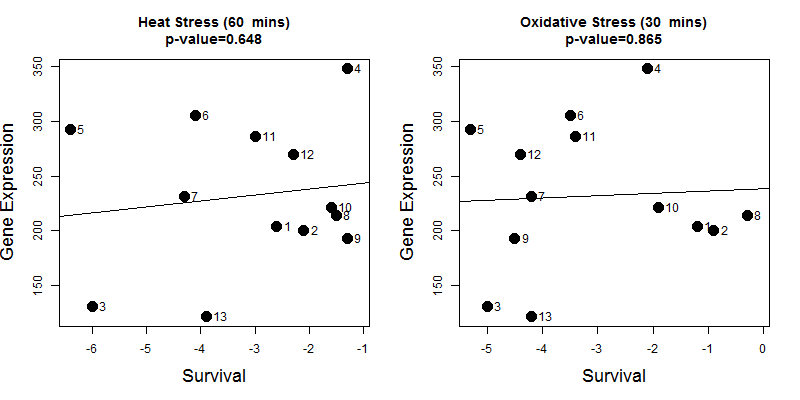

Supplement: S2 File — Expression levels of genes L75676 –L1889726 plotted against survival after 60 minutes heat and 30 min oxidative stress. Survival is expressed as the difference of log CFU/ml after stress and before stress. Numbers indicate fermentations as presented in Table 1. P-values above the plots indicate significance of correlation (assessed by a linear model). (ZIP) [file pone.0167944.s007.zip › S2_File/L109162_real_dat.png]

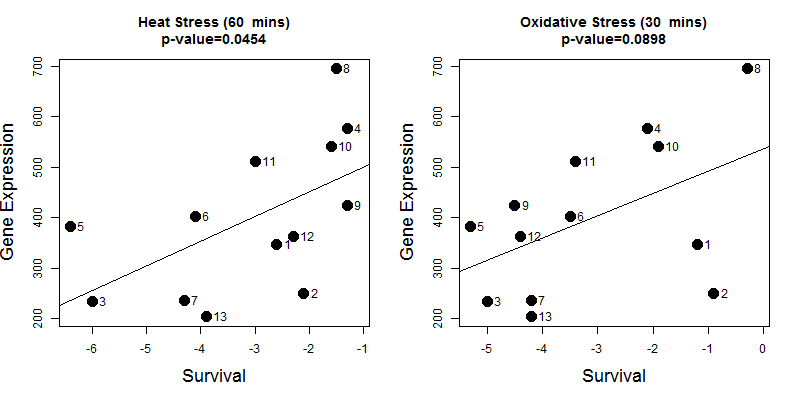

Supplement: S2 File — Expression levels of genes L75676 –L1889726 plotted against survival after 60 minutes heat and 30 min oxidative stress. Survival is expressed as the difference of log CFU/ml after stress and before stress. Numbers indicate fermentations as presented in Table 1. P-values above the plots indicate significance of correlation (assessed by a linear model). (ZIP) [file pone.0167944.s007.zip › S2_File/L109201_real_dat.png]

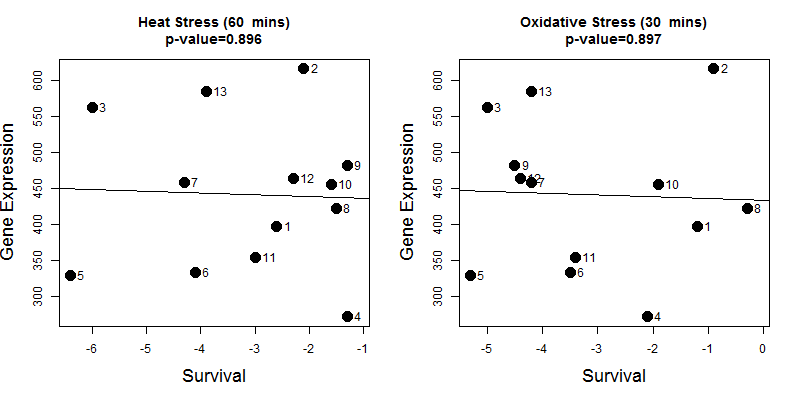

Supplement: S2 File — Expression levels of genes L75676 –L1889726 plotted against survival after 60 minutes heat and 30 min oxidative stress. Survival is expressed as the difference of log CFU/ml after stress and before stress. Numbers indicate fermentations as presented in Table 1. P-values above the plots indicate significance of correlation (assessed by a linear model). (ZIP) [file pone.0167944.s007.zip › S2_File/L109335_real_dat.png]
