# Supplementary figures and images for: Strain-Dependent Transcriptome Signatures for Robustness in Lactococcus lactis (part 2 of 13)
Source: PLoS One. 2016 Dec 14;11(12):e0167944. doi: 10.1371/journal.pone.0167944 (PMC5156439; doi:10.1371/journal.pone.0167944)

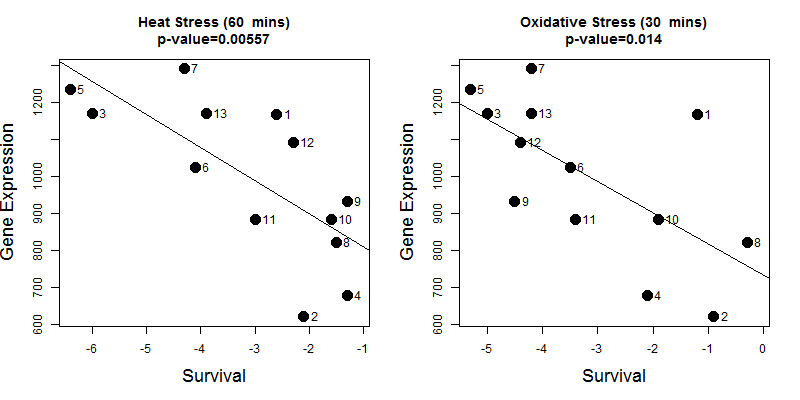

Supplement: S1 File — Expression levels of genes L0001 –L75633 plotted against survival after 60 minutes heat and 30 min oxidative stress. Survival is expressed as the difference of log CFU/ml after stress and before stress. Numbers indicate fermentations as presented in Table 1. P-values above the plots indicate significance of correlation (assessed by a linear model). (ZIP) [file pone.0167944.s006.zip › S1_File/L0096_real_dat.png]

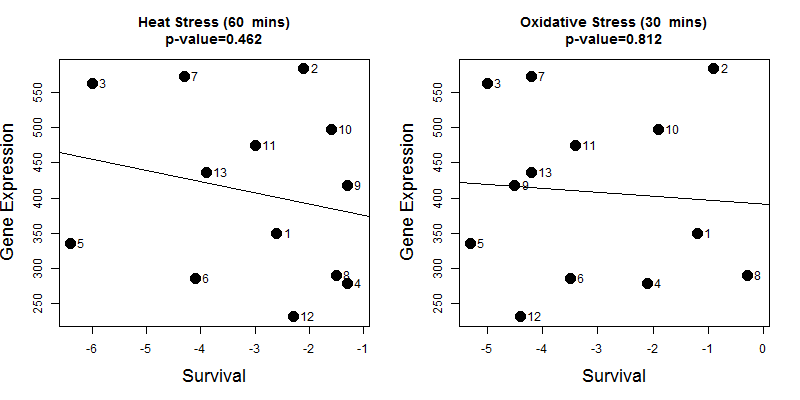

Supplement: S1 File — Expression levels of genes L0001 –L75633 plotted against survival after 60 minutes heat and 30 min oxidative stress. Survival is expressed as the difference of log CFU/ml after stress and before stress. Numbers indicate fermentations as presented in Table 1. P-values above the plots indicate significance of correlation (assessed by a linear model). (ZIP) [file pone.0167944.s006.zip › S1_File/L0097_real_dat.png]

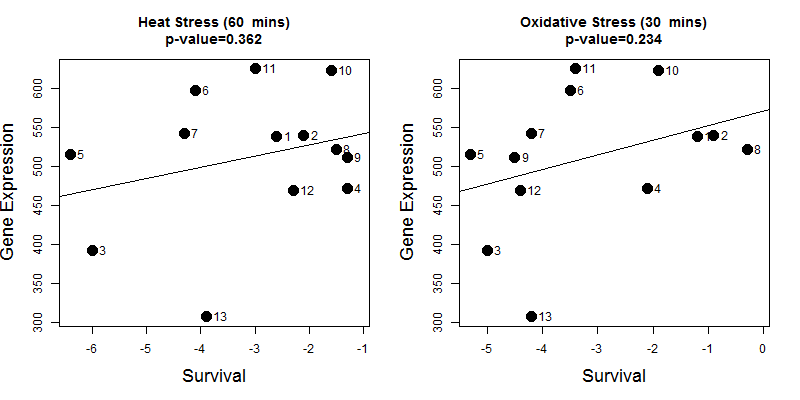

Supplement: S1 File — Expression levels of genes L0001 –L75633 plotted against survival after 60 minutes heat and 30 min oxidative stress. Survival is expressed as the difference of log CFU/ml after stress and before stress. Numbers indicate fermentations as presented in Table 1. P-values above the plots indicate significance of correlation (assessed by a linear model). (ZIP) [file pone.0167944.s006.zip › S1_File/L0098_real_dat.png]

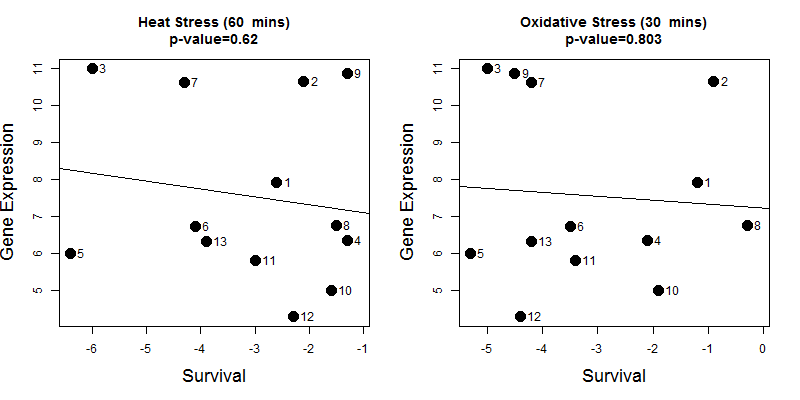

Supplement: S1 File — Expression levels of genes L0001 –L75633 plotted against survival after 60 minutes heat and 30 min oxidative stress. Survival is expressed as the difference of log CFU/ml after stress and before stress. Numbers indicate fermentations as presented in Table 1. P-values above the plots indicate significance of correlation (assessed by a linear model). (ZIP) [file pone.0167944.s006.zip › S1_File/L0099_real_dat.png]

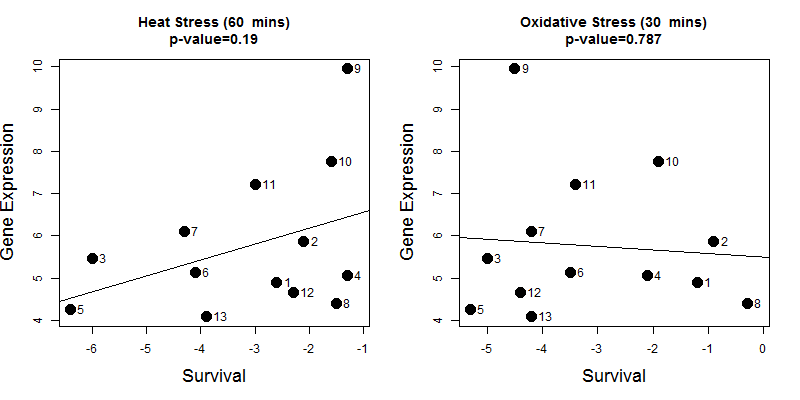

Supplement: S1 File — Expression levels of genes L0001 –L75633 plotted against survival after 60 minutes heat and 30 min oxidative stress. Survival is expressed as the difference of log CFU/ml after stress and before stress. Numbers indicate fermentations as presented in Table 1. P-values above the plots indicate significance of correlation (assessed by a linear model). (ZIP) [file pone.0167944.s006.zip › S1_File/L0100_real_dat.png]

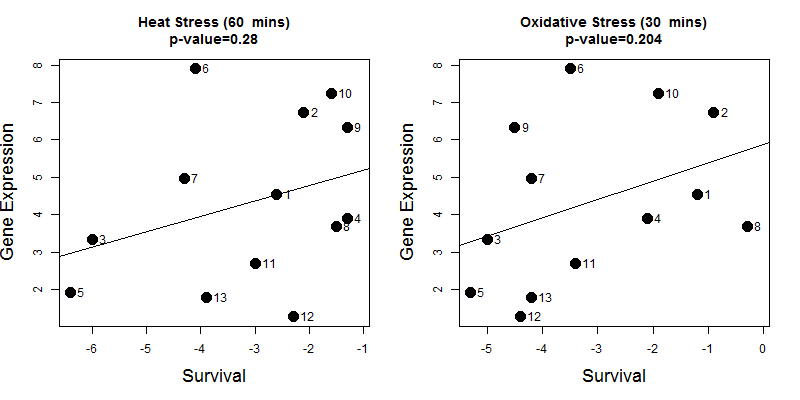

Supplement: S1 File — Expression levels of genes L0001 –L75633 plotted against survival after 60 minutes heat and 30 min oxidative stress. Survival is expressed as the difference of log CFU/ml after stress and before stress. Numbers indicate fermentations as presented in Table 1. P-values above the plots indicate significance of correlation (assessed by a linear model). (ZIP) [file pone.0167944.s006.zip › S1_File/L01009_real_dat.png]

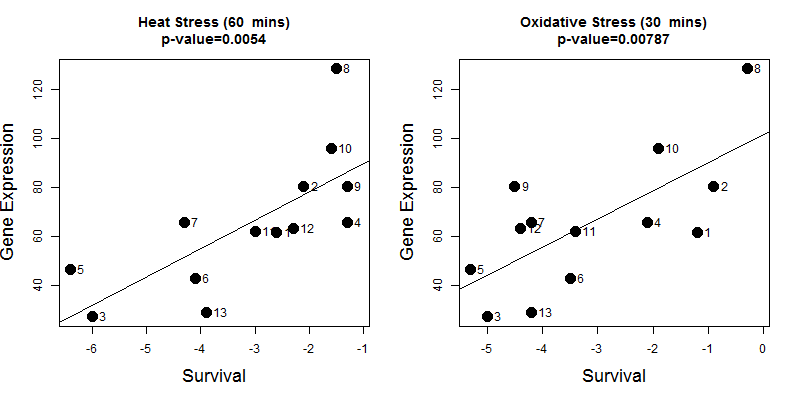

Supplement: S1 File — Expression levels of genes L0001 –L75633 plotted against survival after 60 minutes heat and 30 min oxidative stress. Survival is expressed as the difference of log CFU/ml after stress and before stress. Numbers indicate fermentations as presented in Table 1. P-values above the plots indicate significance of correlation (assessed by a linear model). (ZIP) [file pone.0167944.s006.zip › S1_File/L0101_real_dat.png]

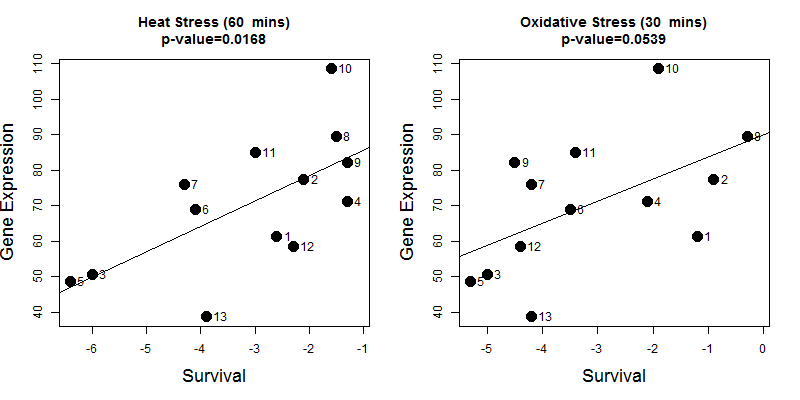

Supplement: S1 File — Expression levels of genes L0001 –L75633 plotted against survival after 60 minutes heat and 30 min oxidative stress. Survival is expressed as the difference of log CFU/ml after stress and before stress. Numbers indicate fermentations as presented in Table 1. P-values above the plots indicate significance of correlation (assessed by a linear model). (ZIP) [file pone.0167944.s006.zip › S1_File/L0102_real_dat.png]

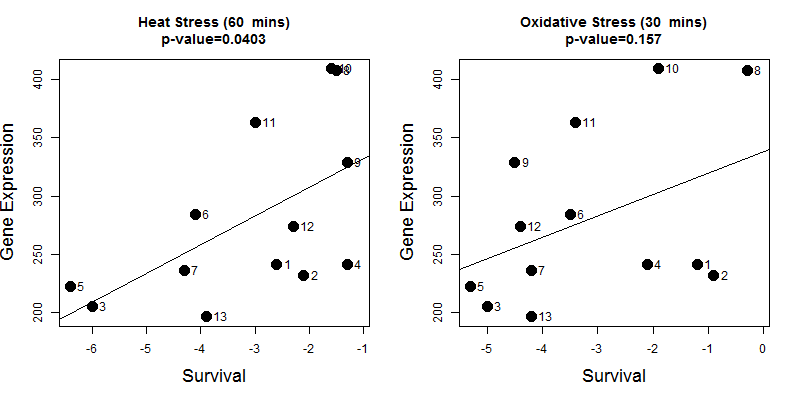

Supplement: S1 File — Expression levels of genes L0001 –L75633 plotted against survival after 60 minutes heat and 30 min oxidative stress. Survival is expressed as the difference of log CFU/ml after stress and before stress. Numbers indicate fermentations as presented in Table 1. P-values above the plots indicate significance of correlation (assessed by a linear model). (ZIP) [file pone.0167944.s006.zip › S1_File/L0103_real_dat.png]

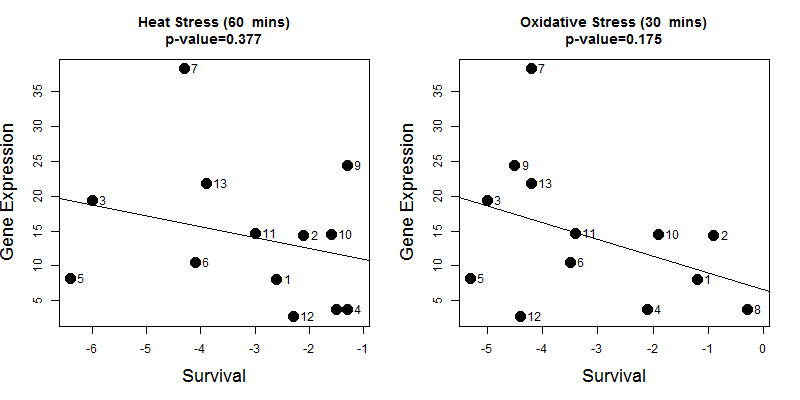

Supplement: S1 File — Expression levels of genes L0001 –L75633 plotted against survival after 60 minutes heat and 30 min oxidative stress. Survival is expressed as the difference of log CFU/ml after stress and before stress. Numbers indicate fermentations as presented in Table 1. P-values above the plots indicate significance of correlation (assessed by a linear model). (ZIP) [file pone.0167944.s006.zip › S1_File/L0104_real_dat.png]

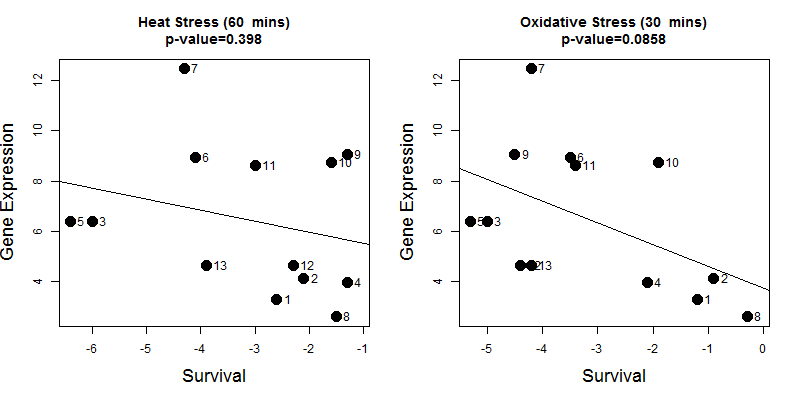

Supplement: S1 File — Expression levels of genes L0001 –L75633 plotted against survival after 60 minutes heat and 30 min oxidative stress. Survival is expressed as the difference of log CFU/ml after stress and before stress. Numbers indicate fermentations as presented in Table 1. P-values above the plots indicate significance of correlation (assessed by a linear model). (ZIP) [file pone.0167944.s006.zip › S1_File/L0105_real_dat.png]

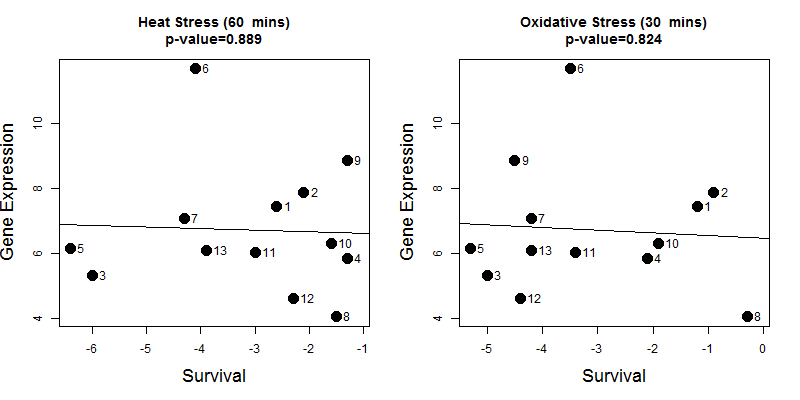

Supplement: S1 File — Expression levels of genes L0001 –L75633 plotted against survival after 60 minutes heat and 30 min oxidative stress. Survival is expressed as the difference of log CFU/ml after stress and before stress. Numbers indicate fermentations as presented in Table 1. P-values above the plots indicate significance of correlation (assessed by a linear model). (ZIP) [file pone.0167944.s006.zip › S1_File/L0106_real_dat.png]

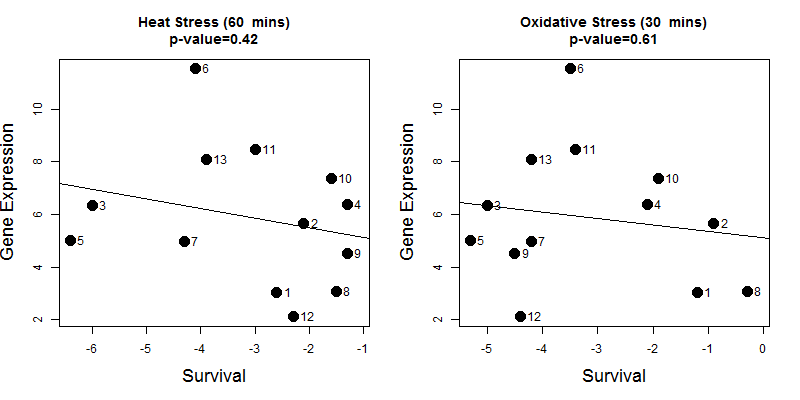

Supplement: S1 File — Expression levels of genes L0001 –L75633 plotted against survival after 60 minutes heat and 30 min oxidative stress. Survival is expressed as the difference of log CFU/ml after stress and before stress. Numbers indicate fermentations as presented in Table 1. P-values above the plots indicate significance of correlation (assessed by a linear model). (ZIP) [file pone.0167944.s006.zip › S1_File/L0107_real_dat.png]

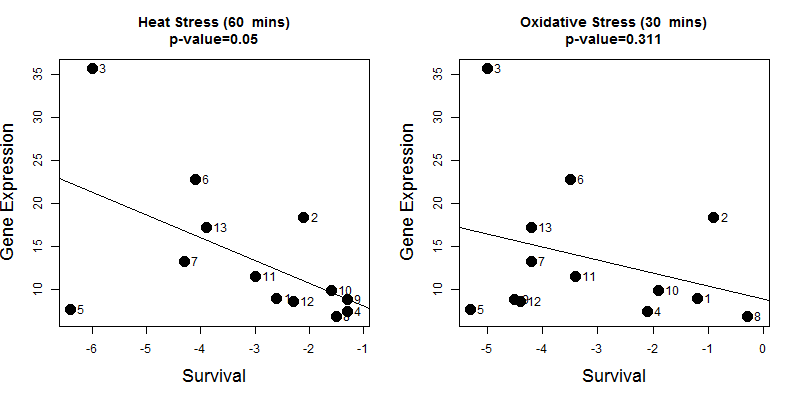

Supplement: S1 File — Expression levels of genes L0001 –L75633 plotted against survival after 60 minutes heat and 30 min oxidative stress. Survival is expressed as the difference of log CFU/ml after stress and before stress. Numbers indicate fermentations as presented in Table 1. P-values above the plots indicate significance of correlation (assessed by a linear model). (ZIP) [file pone.0167944.s006.zip › S1_File/L0108_real_dat.png]

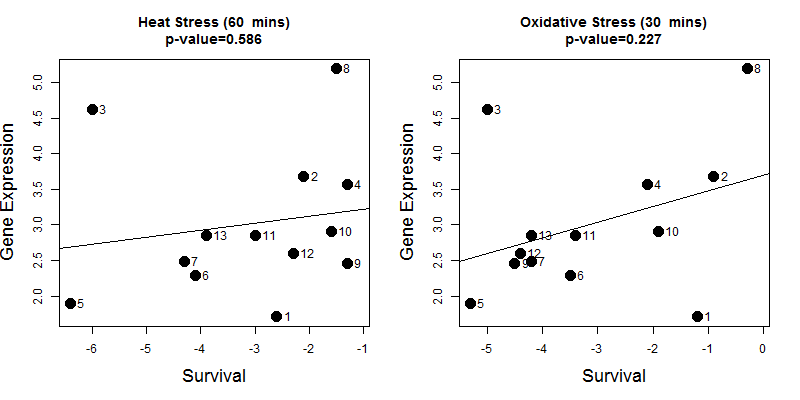

Supplement: S1 File — Expression levels of genes L0001 –L75633 plotted against survival after 60 minutes heat and 30 min oxidative stress. Survival is expressed as the difference of log CFU/ml after stress and before stress. Numbers indicate fermentations as presented in Table 1. P-values above the plots indicate significance of correlation (assessed by a linear model). (ZIP) [file pone.0167944.s006.zip › S1_File/L0109_real_dat.png]

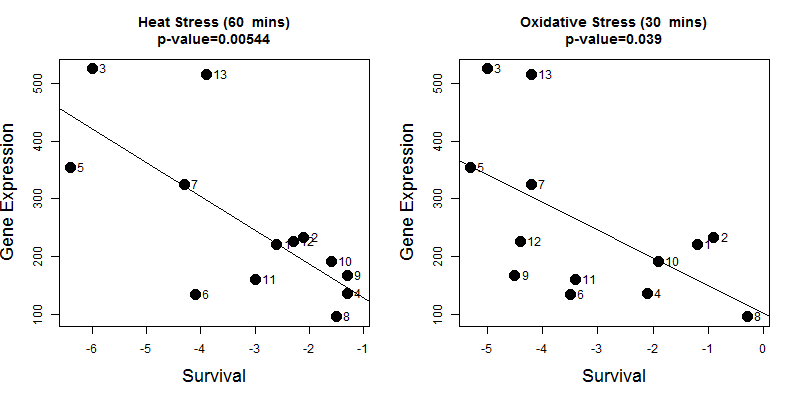

Supplement: S1 File — Expression levels of genes L0001 –L75633 plotted against survival after 60 minutes heat and 30 min oxidative stress. Survival is expressed as the difference of log CFU/ml after stress and before stress. Numbers indicate fermentations as presented in Table 1. P-values above the plots indicate significance of correlation (assessed by a linear model). (ZIP) [file pone.0167944.s006.zip › S1_File/L0110_real_dat.png]

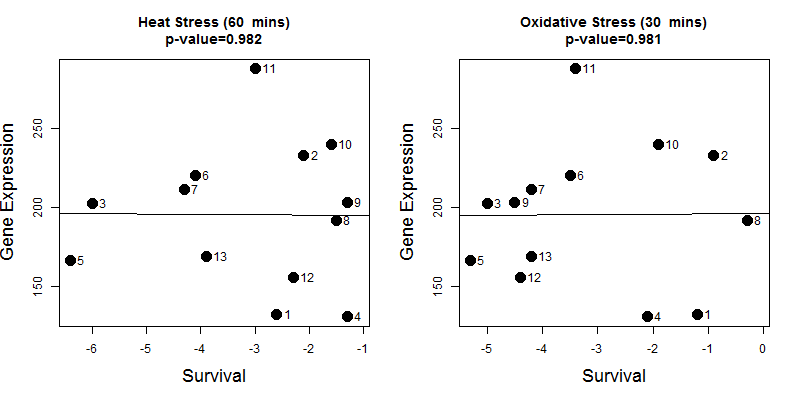

Supplement: S1 File — Expression levels of genes L0001 –L75633 plotted against survival after 60 minutes heat and 30 min oxidative stress. Survival is expressed as the difference of log CFU/ml after stress and before stress. Numbers indicate fermentations as presented in Table 1. P-values above the plots indicate significance of correlation (assessed by a linear model). (ZIP) [file pone.0167944.s006.zip › S1_File/L0111_real_dat.png]

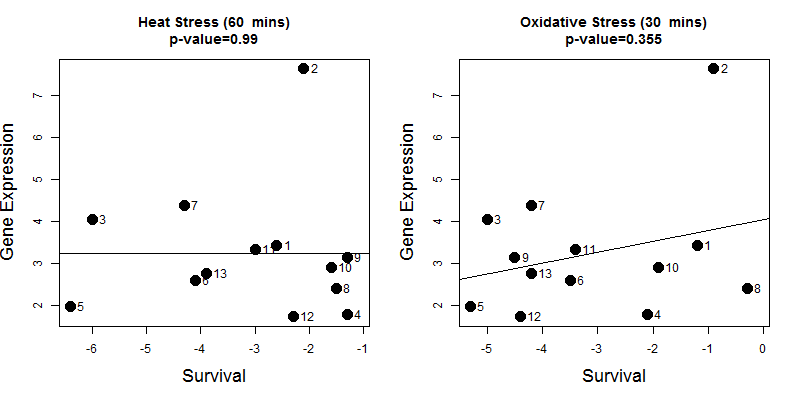

Supplement: S1 File — Expression levels of genes L0001 –L75633 plotted against survival after 60 minutes heat and 30 min oxidative stress. Survival is expressed as the difference of log CFU/ml after stress and before stress. Numbers indicate fermentations as presented in Table 1. P-values above the plots indicate significance of correlation (assessed by a linear model). (ZIP) [file pone.0167944.s006.zip › S1_File/L0112_real_dat.png]

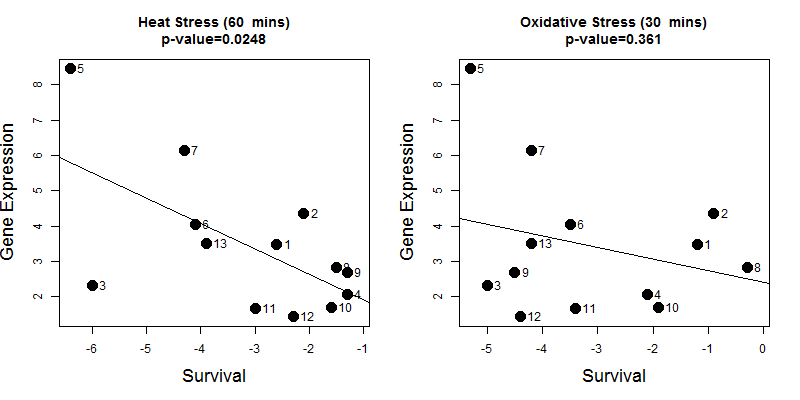

Supplement: S1 File — Expression levels of genes L0001 –L75633 plotted against survival after 60 minutes heat and 30 min oxidative stress. Survival is expressed as the difference of log CFU/ml after stress and before stress. Numbers indicate fermentations as presented in Table 1. P-values above the plots indicate significance of correlation (assessed by a linear model). (ZIP) [file pone.0167944.s006.zip › S1_File/L0113_real_dat.png]

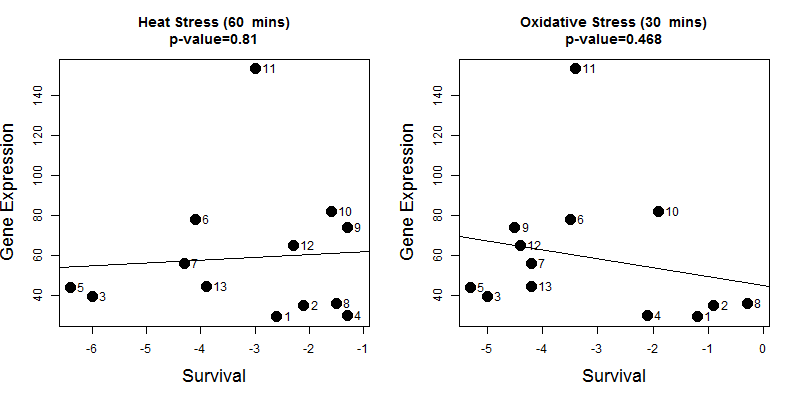

Supplement: S1 File — Expression levels of genes L0001 –L75633 plotted against survival after 60 minutes heat and 30 min oxidative stress. Survival is expressed as the difference of log CFU/ml after stress and before stress. Numbers indicate fermentations as presented in Table 1. P-values above the plots indicate significance of correlation (assessed by a linear model). (ZIP) [file pone.0167944.s006.zip › S1_File/L0114_real_dat.png]

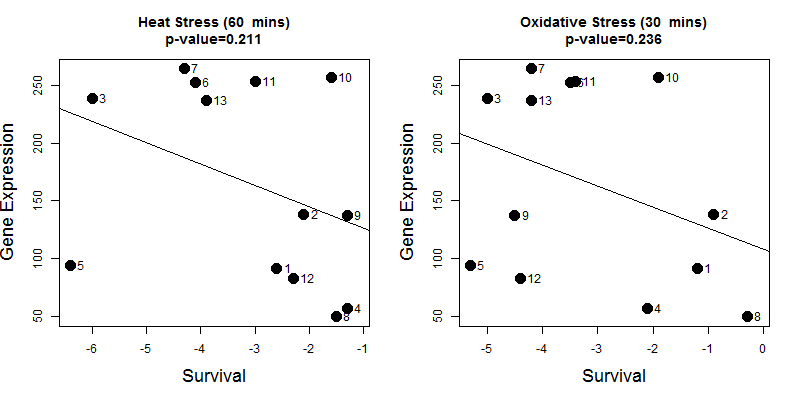

Supplement: S1 File — Expression levels of genes L0001 –L75633 plotted against survival after 60 minutes heat and 30 min oxidative stress. Survival is expressed as the difference of log CFU/ml after stress and before stress. Numbers indicate fermentations as presented in Table 1. P-values above the plots indicate significance of correlation (assessed by a linear model). (ZIP) [file pone.0167944.s006.zip › S1_File/L0115_real_dat.png]

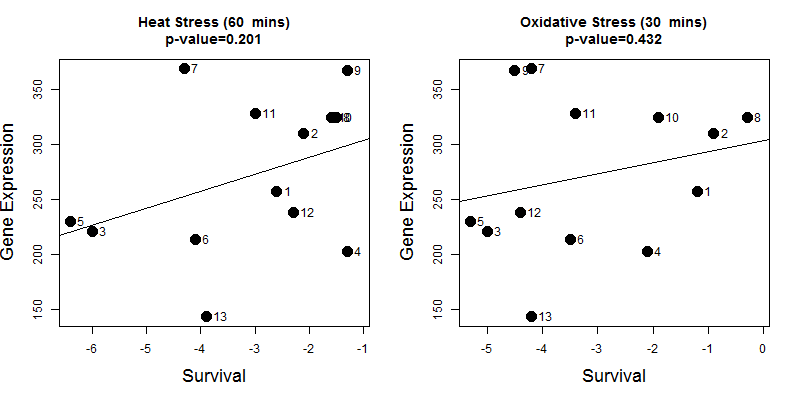

Supplement: S1 File — Expression levels of genes L0001 –L75633 plotted against survival after 60 minutes heat and 30 min oxidative stress. Survival is expressed as the difference of log CFU/ml after stress and before stress. Numbers indicate fermentations as presented in Table 1. P-values above the plots indicate significance of correlation (assessed by a linear model). (ZIP) [file pone.0167944.s006.zip › S1_File/L0116_real_dat.png]

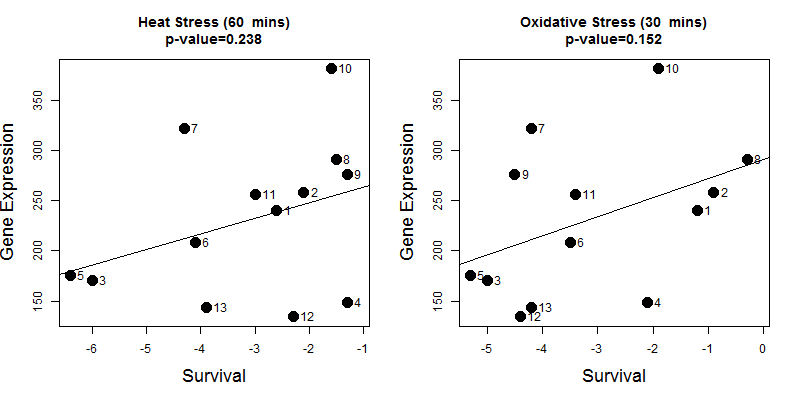

Supplement: S1 File — Expression levels of genes L0001 –L75633 plotted against survival after 60 minutes heat and 30 min oxidative stress. Survival is expressed as the difference of log CFU/ml after stress and before stress. Numbers indicate fermentations as presented in Table 1. P-values above the plots indicate significance of correlation (assessed by a linear model). (ZIP) [file pone.0167944.s006.zip › S1_File/L0117_real_dat.png]

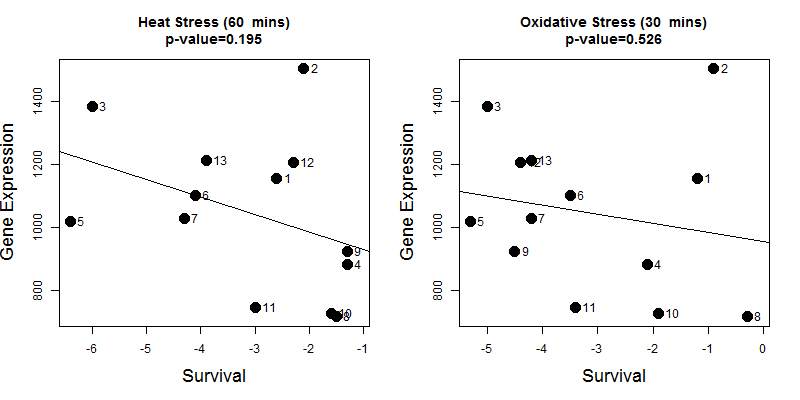

Supplement: S1 File — Expression levels of genes L0001 –L75633 plotted against survival after 60 minutes heat and 30 min oxidative stress. Survival is expressed as the difference of log CFU/ml after stress and before stress. Numbers indicate fermentations as presented in Table 1. P-values above the plots indicate significance of correlation (assessed by a linear model). (ZIP) [file pone.0167944.s006.zip › S1_File/L0118_real_dat.png]

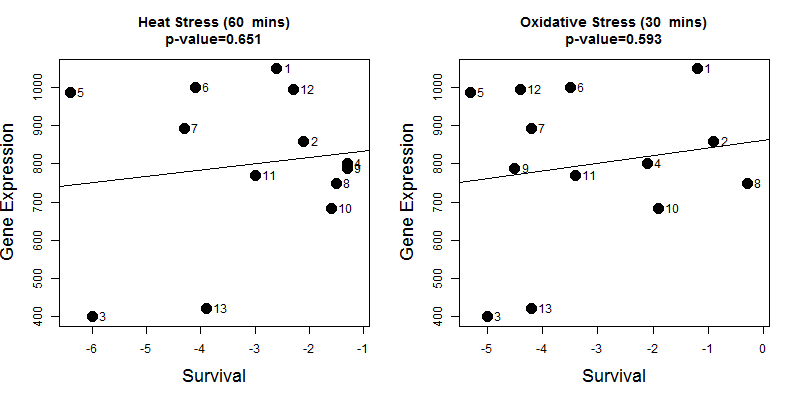

Supplement: S1 File — Expression levels of genes L0001 –L75633 plotted against survival after 60 minutes heat and 30 min oxidative stress. Survival is expressed as the difference of log CFU/ml after stress and before stress. Numbers indicate fermentations as presented in Table 1. P-values above the plots indicate significance of correlation (assessed by a linear model). (ZIP) [file pone.0167944.s006.zip › S1_File/L0119_real_dat.png]

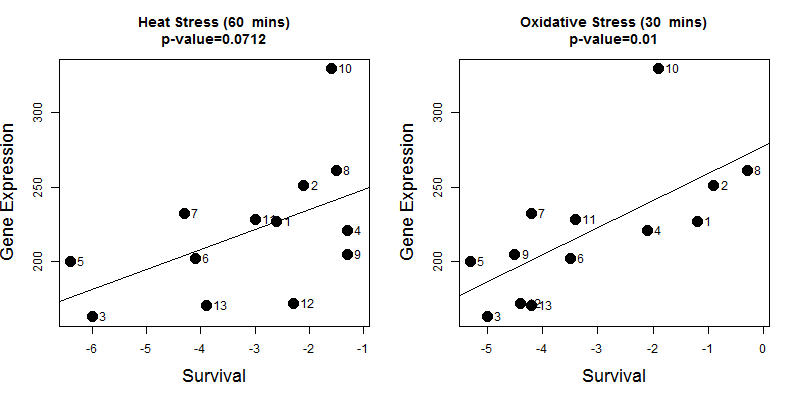

Supplement: S1 File — Expression levels of genes L0001 –L75633 plotted against survival after 60 minutes heat and 30 min oxidative stress. Survival is expressed as the difference of log CFU/ml after stress and before stress. Numbers indicate fermentations as presented in Table 1. P-values above the plots indicate significance of correlation (assessed by a linear model). (ZIP) [file pone.0167944.s006.zip › S1_File/L0120_real_dat.png]

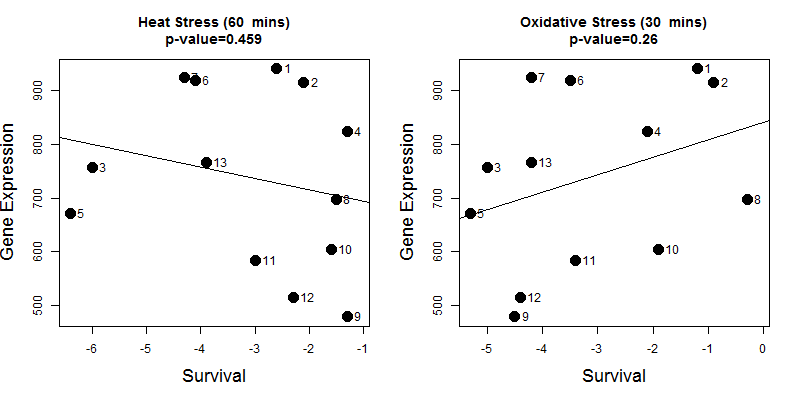

Supplement: S1 File — Expression levels of genes L0001 –L75633 plotted against survival after 60 minutes heat and 30 min oxidative stress. Survival is expressed as the difference of log CFU/ml after stress and before stress. Numbers indicate fermentations as presented in Table 1. P-values above the plots indicate significance of correlation (assessed by a linear model). (ZIP) [file pone.0167944.s006.zip › S1_File/L0121_real_dat.png]

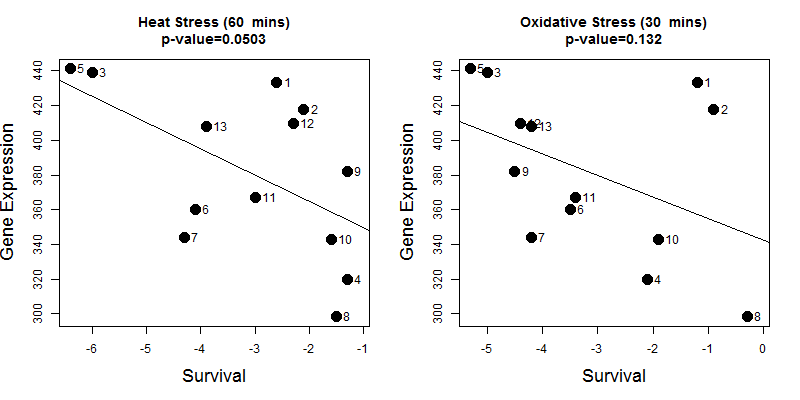

Supplement: S1 File — Expression levels of genes L0001 –L75633 plotted against survival after 60 minutes heat and 30 min oxidative stress. Survival is expressed as the difference of log CFU/ml after stress and before stress. Numbers indicate fermentations as presented in Table 1. P-values above the plots indicate significance of correlation (assessed by a linear model). (ZIP) [file pone.0167944.s006.zip › S1_File/L0122_real_dat.png]

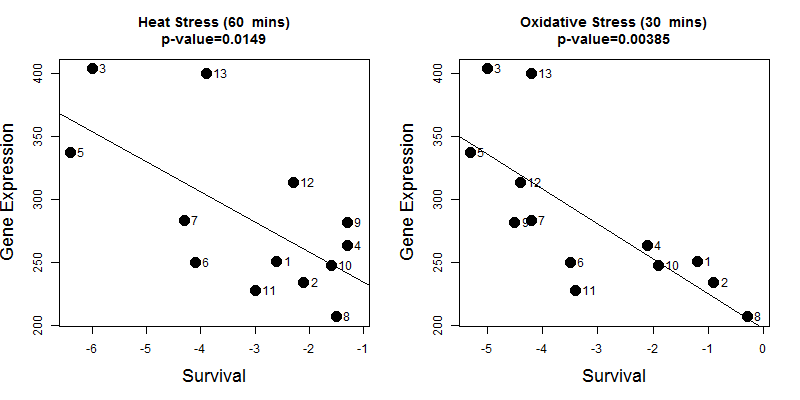

Supplement: S1 File — Expression levels of genes L0001 –L75633 plotted against survival after 60 minutes heat and 30 min oxidative stress. Survival is expressed as the difference of log CFU/ml after stress and before stress. Numbers indicate fermentations as presented in Table 1. P-values above the plots indicate significance of correlation (assessed by a linear model). (ZIP) [file pone.0167944.s006.zip › S1_File/L0123_real_dat.png]

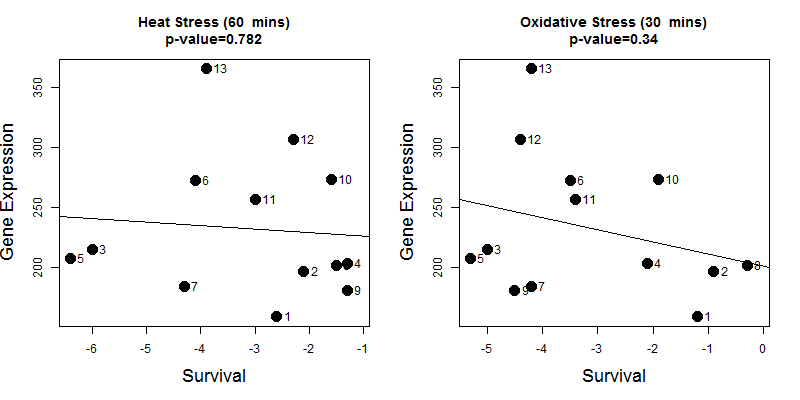

Supplement: S1 File — Expression levels of genes L0001 –L75633 plotted against survival after 60 minutes heat and 30 min oxidative stress. Survival is expressed as the difference of log CFU/ml after stress and before stress. Numbers indicate fermentations as presented in Table 1. P-values above the plots indicate significance of correlation (assessed by a linear model). (ZIP) [file pone.0167944.s006.zip › S1_File/L0124_real_dat.png]

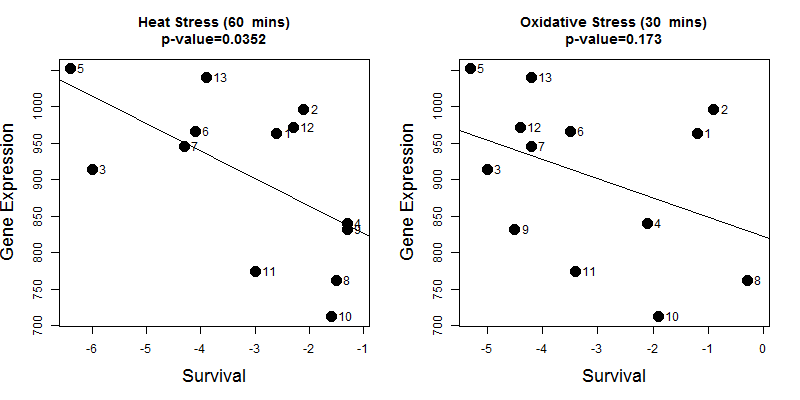

Supplement: S1 File — Expression levels of genes L0001 –L75633 plotted against survival after 60 minutes heat and 30 min oxidative stress. Survival is expressed as the difference of log CFU/ml after stress and before stress. Numbers indicate fermentations as presented in Table 1. P-values above the plots indicate significance of correlation (assessed by a linear model). (ZIP) [file pone.0167944.s006.zip › S1_File/L0125_real_dat.png]

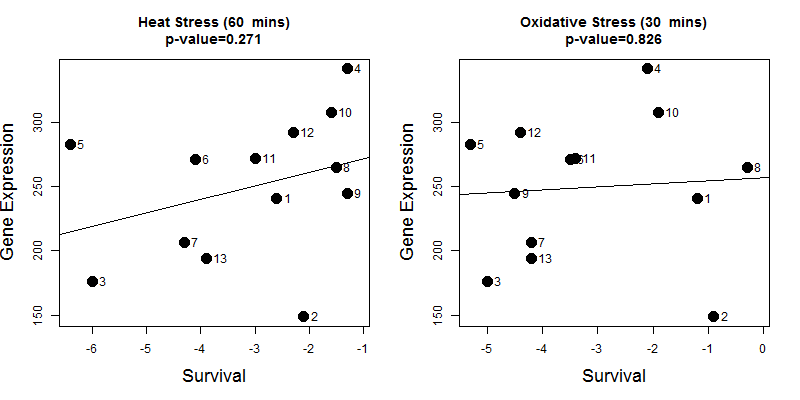

Supplement: S1 File — Expression levels of genes L0001 –L75633 plotted against survival after 60 minutes heat and 30 min oxidative stress. Survival is expressed as the difference of log CFU/ml after stress and before stress. Numbers indicate fermentations as presented in Table 1. P-values above the plots indicate significance of correlation (assessed by a linear model). (ZIP) [file pone.0167944.s006.zip › S1_File/L0126_real_dat.png]

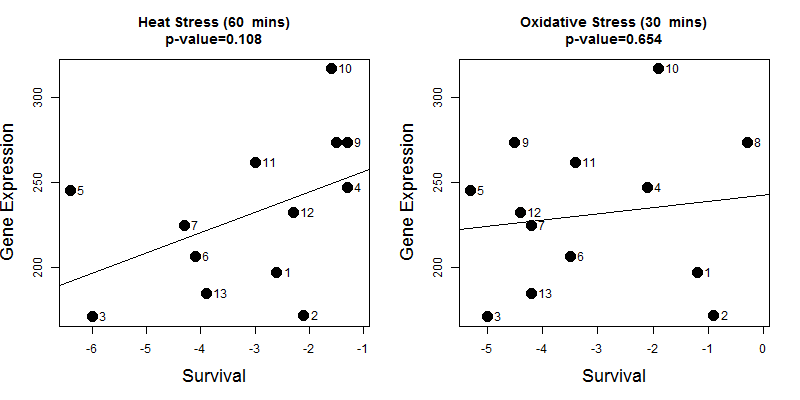

Supplement: S1 File — Expression levels of genes L0001 –L75633 plotted against survival after 60 minutes heat and 30 min oxidative stress. Survival is expressed as the difference of log CFU/ml after stress and before stress. Numbers indicate fermentations as presented in Table 1. P-values above the plots indicate significance of correlation (assessed by a linear model). (ZIP) [file pone.0167944.s006.zip › S1_File/L0127_real_dat.png]

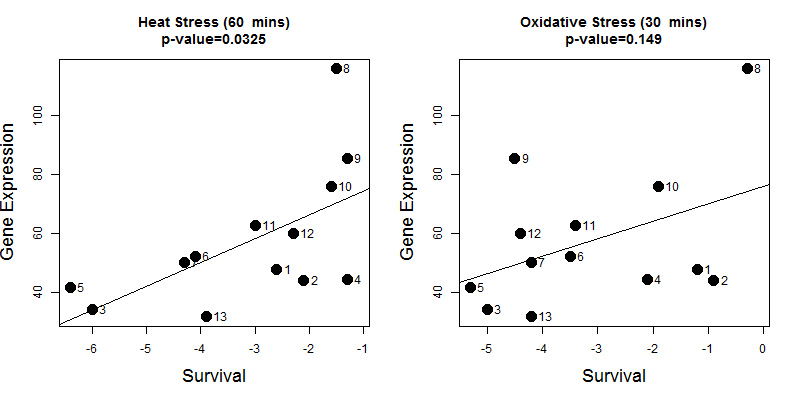

Supplement: S1 File — Expression levels of genes L0001 –L75633 plotted against survival after 60 minutes heat and 30 min oxidative stress. Survival is expressed as the difference of log CFU/ml after stress and before stress. Numbers indicate fermentations as presented in Table 1. P-values above the plots indicate significance of correlation (assessed by a linear model). (ZIP) [file pone.0167944.s006.zip › S1_File/L0128_real_dat.png]

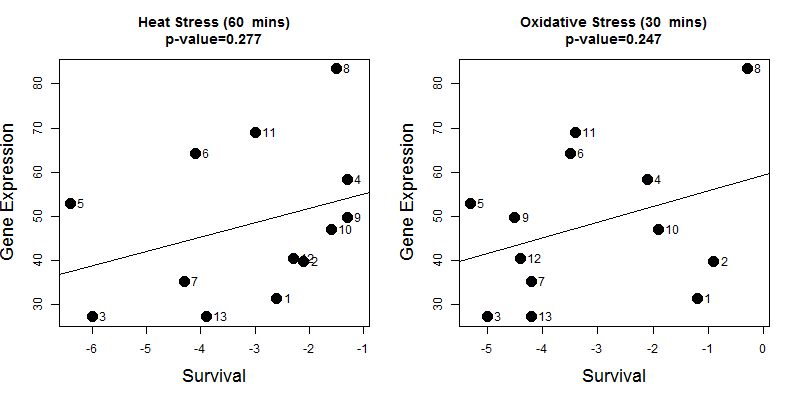

Supplement: S1 File — Expression levels of genes L0001 –L75633 plotted against survival after 60 minutes heat and 30 min oxidative stress. Survival is expressed as the difference of log CFU/ml after stress and before stress. Numbers indicate fermentations as presented in Table 1. P-values above the plots indicate significance of correlation (assessed by a linear model). (ZIP) [file pone.0167944.s006.zip › S1_File/L0129_real_dat.png]

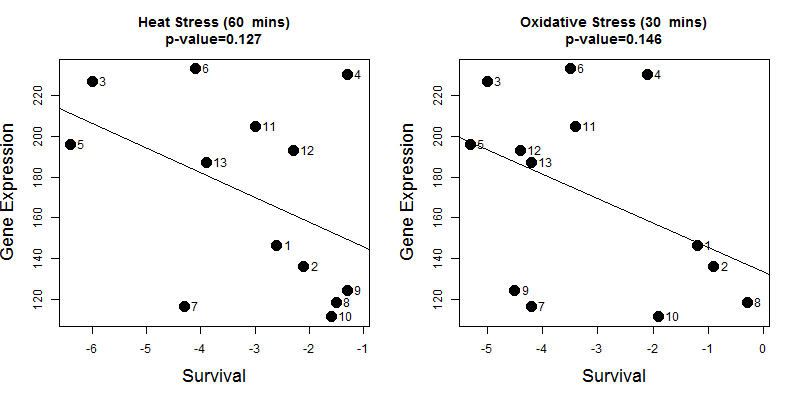

Supplement: S1 File — Expression levels of genes L0001 –L75633 plotted against survival after 60 minutes heat and 30 min oxidative stress. Survival is expressed as the difference of log CFU/ml after stress and before stress. Numbers indicate fermentations as presented in Table 1. P-values above the plots indicate significance of correlation (assessed by a linear model). (ZIP) [file pone.0167944.s006.zip › S1_File/L0130_real_dat.png]

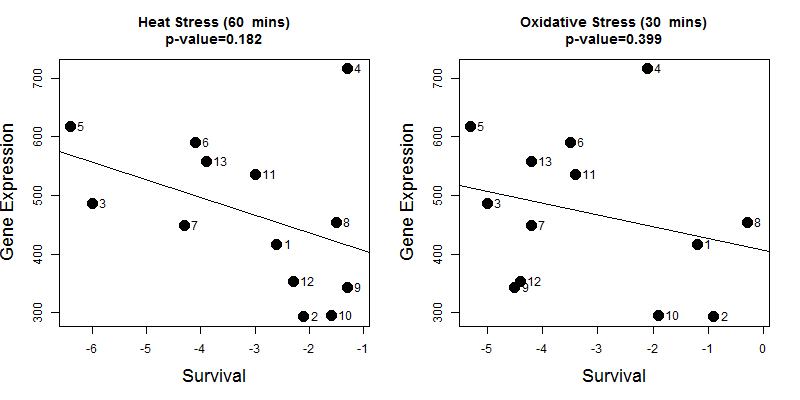

Supplement: S1 File — Expression levels of genes L0001 –L75633 plotted against survival after 60 minutes heat and 30 min oxidative stress. Survival is expressed as the difference of log CFU/ml after stress and before stress. Numbers indicate fermentations as presented in Table 1. P-values above the plots indicate significance of correlation (assessed by a linear model). (ZIP) [file pone.0167944.s006.zip › S1_File/L0131_real_dat.png]

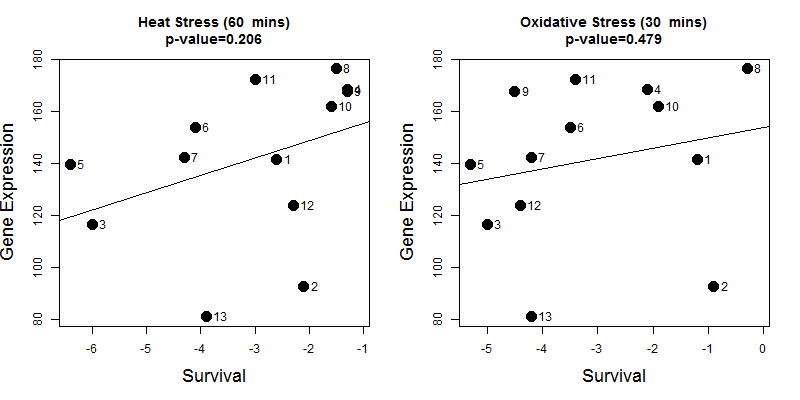

Supplement: S1 File — Expression levels of genes L0001 –L75633 plotted against survival after 60 minutes heat and 30 min oxidative stress. Survival is expressed as the difference of log CFU/ml after stress and before stress. Numbers indicate fermentations as presented in Table 1. P-values above the plots indicate significance of correlation (assessed by a linear model). (ZIP) [file pone.0167944.s006.zip › S1_File/L0132_real_dat.png]

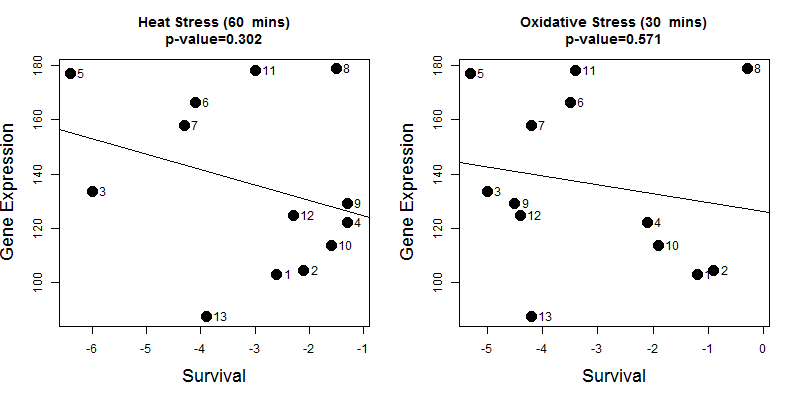

Supplement: S1 File — Expression levels of genes L0001 –L75633 plotted against survival after 60 minutes heat and 30 min oxidative stress. Survival is expressed as the difference of log CFU/ml after stress and before stress. Numbers indicate fermentations as presented in Table 1. P-values above the plots indicate significance of correlation (assessed by a linear model). (ZIP) [file pone.0167944.s006.zip › S1_File/L0133_real_dat.png]

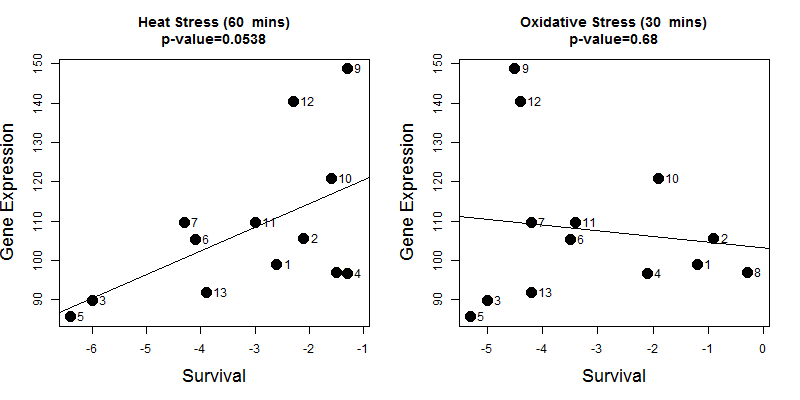

Supplement: S1 File — Expression levels of genes L0001 –L75633 plotted against survival after 60 minutes heat and 30 min oxidative stress. Survival is expressed as the difference of log CFU/ml after stress and before stress. Numbers indicate fermentations as presented in Table 1. P-values above the plots indicate significance of correlation (assessed by a linear model). (ZIP) [file pone.0167944.s006.zip › S1_File/L0134_real_dat.png]

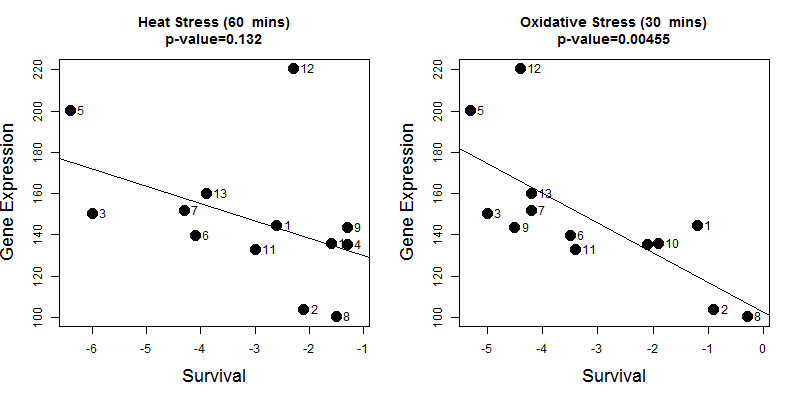

Supplement: S1 File — Expression levels of genes L0001 –L75633 plotted against survival after 60 minutes heat and 30 min oxidative stress. Survival is expressed as the difference of log CFU/ml after stress and before stress. Numbers indicate fermentations as presented in Table 1. P-values above the plots indicate significance of correlation (assessed by a linear model). (ZIP) [file pone.0167944.s006.zip › S1_File/L0135_real_dat.png]

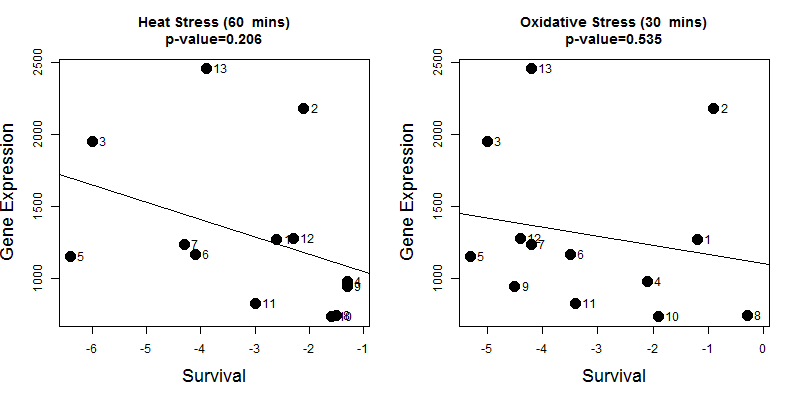

Supplement: S1 File — Expression levels of genes L0001 –L75633 plotted against survival after 60 minutes heat and 30 min oxidative stress. Survival is expressed as the difference of log CFU/ml after stress and before stress. Numbers indicate fermentations as presented in Table 1. P-values above the plots indicate significance of correlation (assessed by a linear model). (ZIP) [file pone.0167944.s006.zip › S1_File/L0136_real_dat.png]

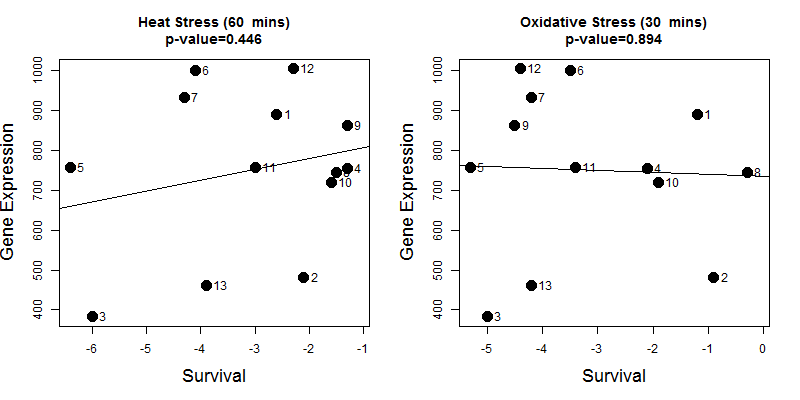

Supplement: S1 File — Expression levels of genes L0001 –L75633 plotted against survival after 60 minutes heat and 30 min oxidative stress. Survival is expressed as the difference of log CFU/ml after stress and before stress. Numbers indicate fermentations as presented in Table 1. P-values above the plots indicate significance of correlation (assessed by a linear model). (ZIP) [file pone.0167944.s006.zip › S1_File/L0137_real_dat.png]

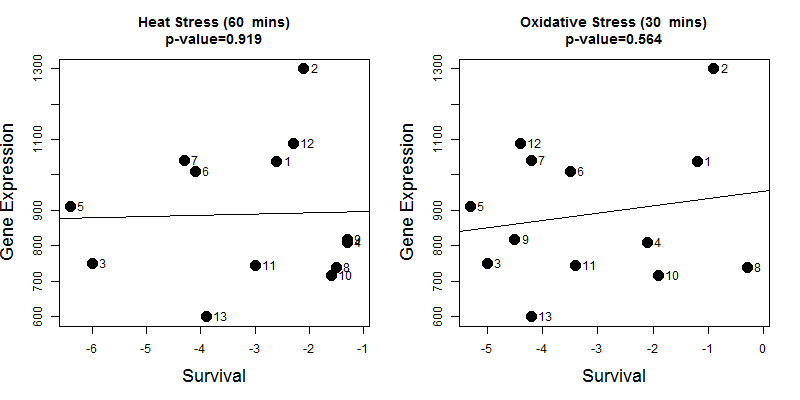

Supplement: S1 File — Expression levels of genes L0001 –L75633 plotted against survival after 60 minutes heat and 30 min oxidative stress. Survival is expressed as the difference of log CFU/ml after stress and before stress. Numbers indicate fermentations as presented in Table 1. P-values above the plots indicate significance of correlation (assessed by a linear model). (ZIP) [file pone.0167944.s006.zip › S1_File/L0138_real_dat.png]

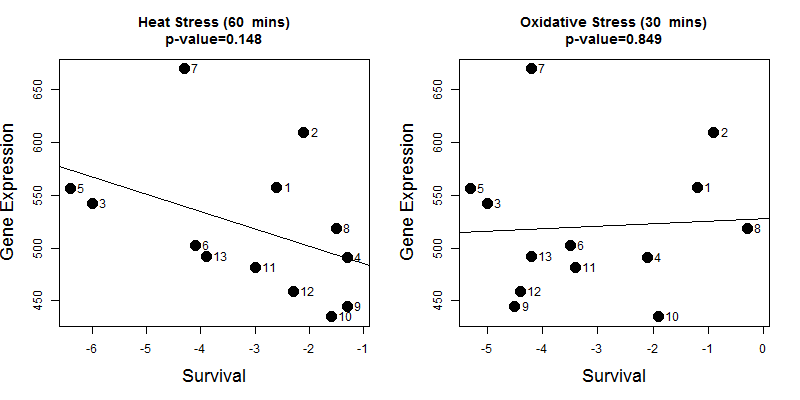

Supplement: S1 File — Expression levels of genes L0001 –L75633 plotted against survival after 60 minutes heat and 30 min oxidative stress. Survival is expressed as the difference of log CFU/ml after stress and before stress. Numbers indicate fermentations as presented in Table 1. P-values above the plots indicate significance of correlation (assessed by a linear model). (ZIP) [file pone.0167944.s006.zip › S1_File/L0139_real_dat.png]

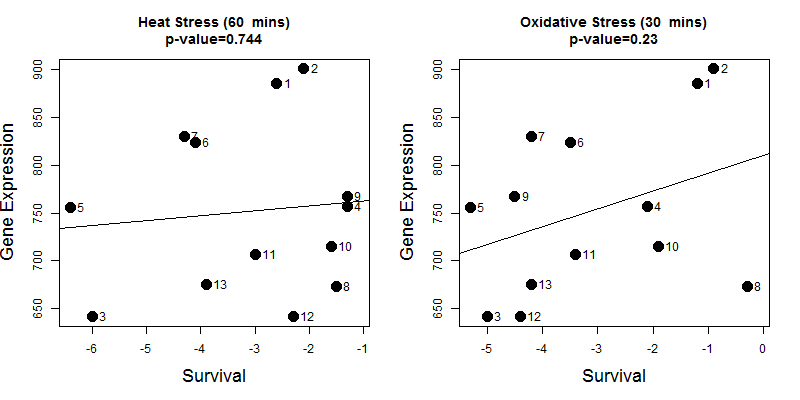

Supplement: S1 File — Expression levels of genes L0001 –L75633 plotted against survival after 60 minutes heat and 30 min oxidative stress. Survival is expressed as the difference of log CFU/ml after stress and before stress. Numbers indicate fermentations as presented in Table 1. P-values above the plots indicate significance of correlation (assessed by a linear model). (ZIP) [file pone.0167944.s006.zip › S1_File/L0140_real_dat.png]

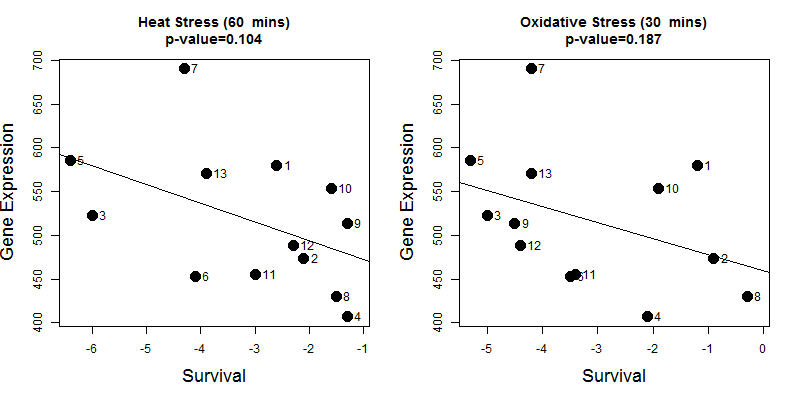

Supplement: S1 File — Expression levels of genes L0001 –L75633 plotted against survival after 60 minutes heat and 30 min oxidative stress. Survival is expressed as the difference of log CFU/ml after stress and before stress. Numbers indicate fermentations as presented in Table 1. P-values above the plots indicate significance of correlation (assessed by a linear model). (ZIP) [file pone.0167944.s006.zip › S1_File/L0141_real_dat.png]

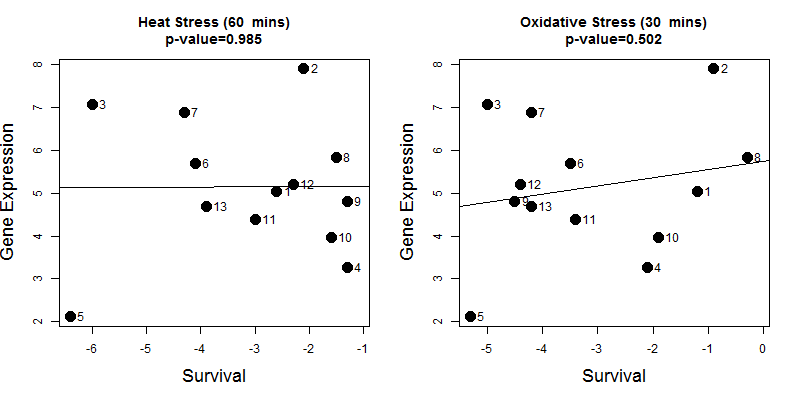

Supplement: S1 File — Expression levels of genes L0001 –L75633 plotted against survival after 60 minutes heat and 30 min oxidative stress. Survival is expressed as the difference of log CFU/ml after stress and before stress. Numbers indicate fermentations as presented in Table 1. P-values above the plots indicate significance of correlation (assessed by a linear model). (ZIP) [file pone.0167944.s006.zip › S1_File/L0142_real_dat.png]

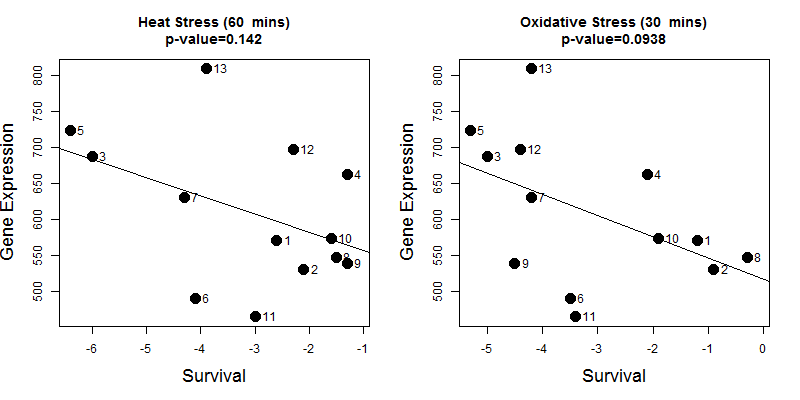

Supplement: S1 File — Expression levels of genes L0001 –L75633 plotted against survival after 60 minutes heat and 30 min oxidative stress. Survival is expressed as the difference of log CFU/ml after stress and before stress. Numbers indicate fermentations as presented in Table 1. P-values above the plots indicate significance of correlation (assessed by a linear model). (ZIP) [file pone.0167944.s006.zip › S1_File/L0143_real_dat.png]

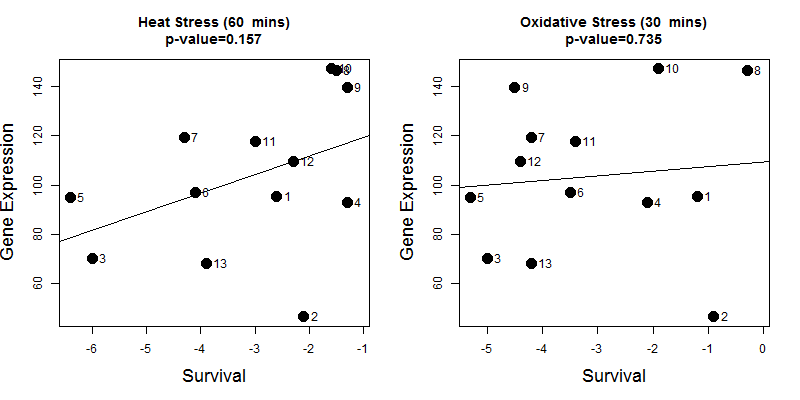

Supplement: S1 File — Expression levels of genes L0001 –L75633 plotted against survival after 60 minutes heat and 30 min oxidative stress. Survival is expressed as the difference of log CFU/ml after stress and before stress. Numbers indicate fermentations as presented in Table 1. P-values above the plots indicate significance of correlation (assessed by a linear model). (ZIP) [file pone.0167944.s006.zip › S1_File/L0144_real_dat.png]

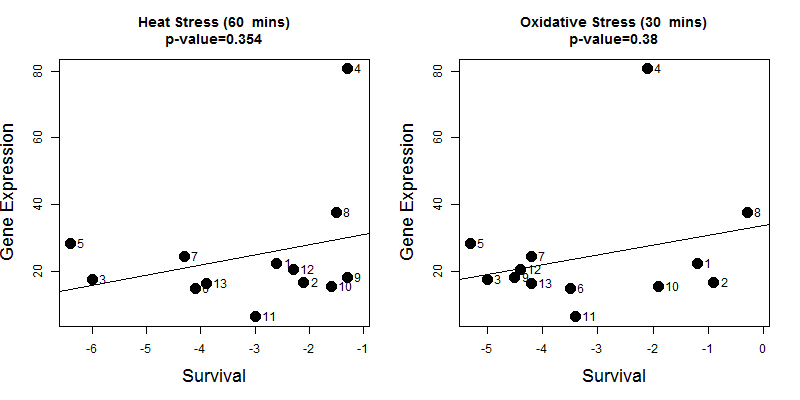

Supplement: S1 File — Expression levels of genes L0001 –L75633 plotted against survival after 60 minutes heat and 30 min oxidative stress. Survival is expressed as the difference of log CFU/ml after stress and before stress. Numbers indicate fermentations as presented in Table 1. P-values above the plots indicate significance of correlation (assessed by a linear model). (ZIP) [file pone.0167944.s006.zip › S1_File/L0145_real_dat.png]

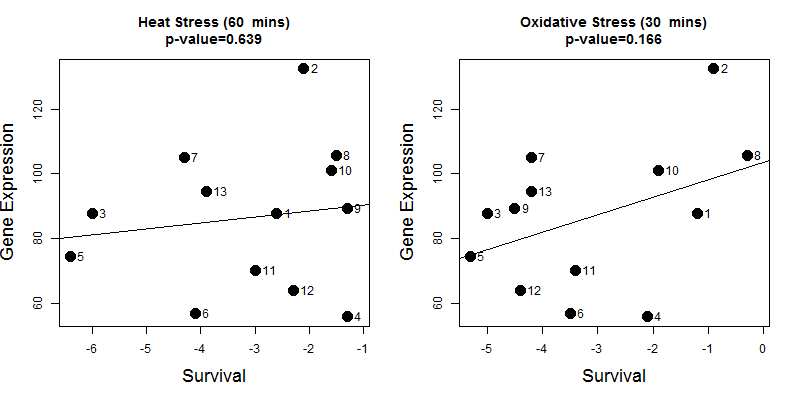

Supplement: S1 File — Expression levels of genes L0001 –L75633 plotted against survival after 60 minutes heat and 30 min oxidative stress. Survival is expressed as the difference of log CFU/ml after stress and before stress. Numbers indicate fermentations as presented in Table 1. P-values above the plots indicate significance of correlation (assessed by a linear model). (ZIP) [file pone.0167944.s006.zip › S1_File/L0146_real_dat.png]

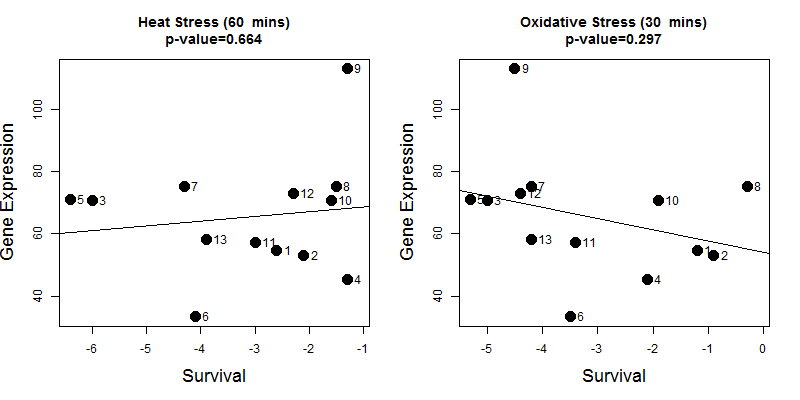

Supplement: S1 File — Expression levels of genes L0001 –L75633 plotted against survival after 60 minutes heat and 30 min oxidative stress. Survival is expressed as the difference of log CFU/ml after stress and before stress. Numbers indicate fermentations as presented in Table 1. P-values above the plots indicate significance of correlation (assessed by a linear model). (ZIP) [file pone.0167944.s006.zip › S1_File/L0147_real_dat.png]

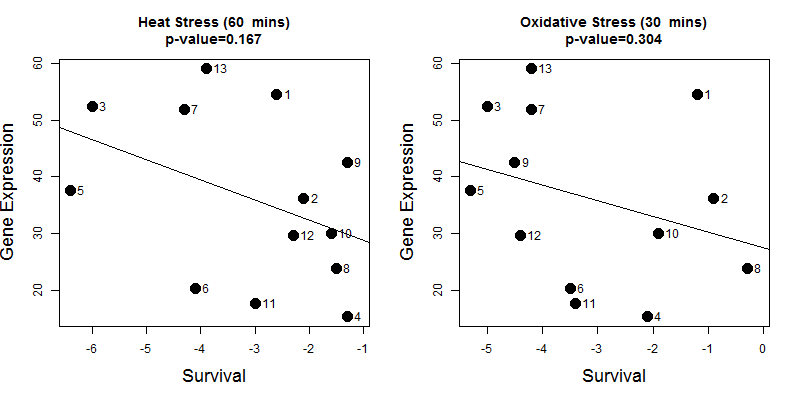

Supplement: S1 File — Expression levels of genes L0001 –L75633 plotted against survival after 60 minutes heat and 30 min oxidative stress. Survival is expressed as the difference of log CFU/ml after stress and before stress. Numbers indicate fermentations as presented in Table 1. P-values above the plots indicate significance of correlation (assessed by a linear model). (ZIP) [file pone.0167944.s006.zip › S1_File/L0148_real_dat.png]

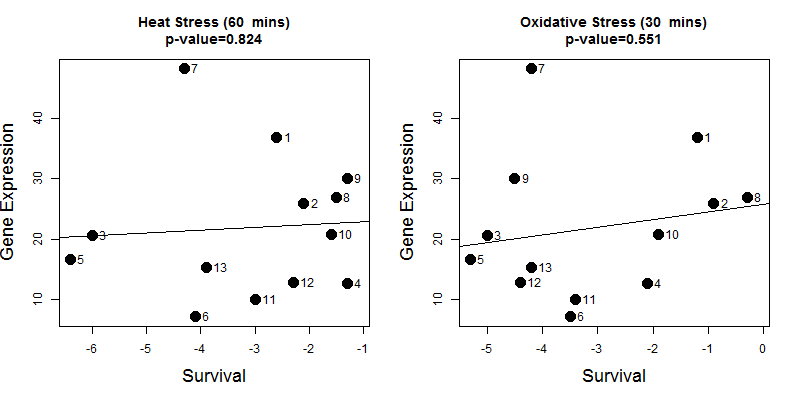

Supplement: S1 File — Expression levels of genes L0001 –L75633 plotted against survival after 60 minutes heat and 30 min oxidative stress. Survival is expressed as the difference of log CFU/ml after stress and before stress. Numbers indicate fermentations as presented in Table 1. P-values above the plots indicate significance of correlation (assessed by a linear model). (ZIP) [file pone.0167944.s006.zip › S1_File/L0149_real_dat.png]

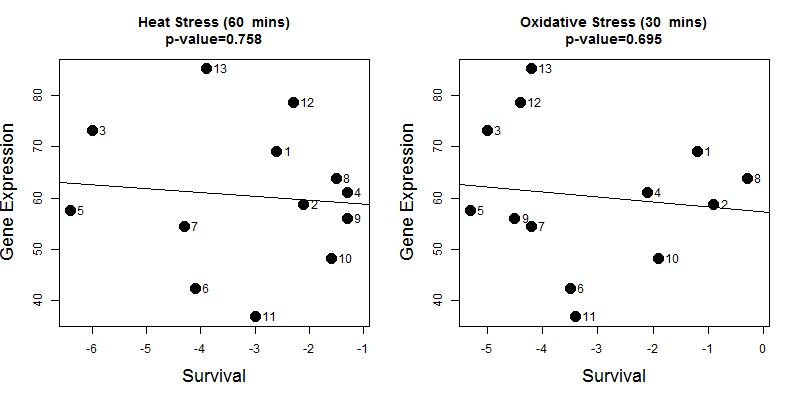

Supplement: S1 File — Expression levels of genes L0001 –L75633 plotted against survival after 60 minutes heat and 30 min oxidative stress. Survival is expressed as the difference of log CFU/ml after stress and before stress. Numbers indicate fermentations as presented in Table 1. P-values above the plots indicate significance of correlation (assessed by a linear model). (ZIP) [file pone.0167944.s006.zip › S1_File/L0150_real_dat.png]

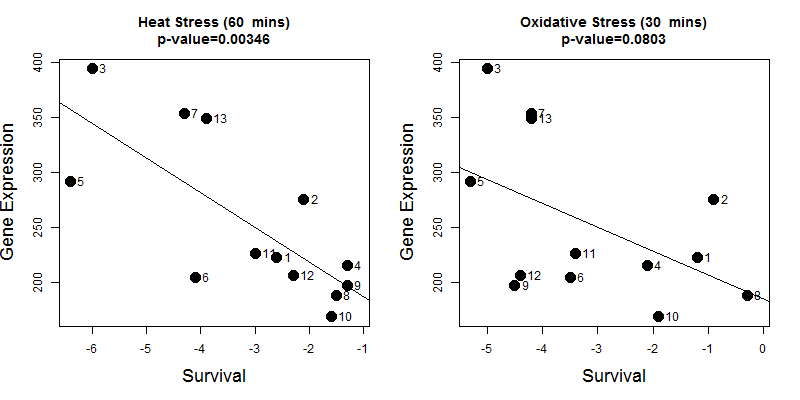

Supplement: S1 File — Expression levels of genes L0001 –L75633 plotted against survival after 60 minutes heat and 30 min oxidative stress. Survival is expressed as the difference of log CFU/ml after stress and before stress. Numbers indicate fermentations as presented in Table 1. P-values above the plots indicate significance of correlation (assessed by a linear model). (ZIP) [file pone.0167944.s006.zip › S1_File/L0151_real_dat.png]

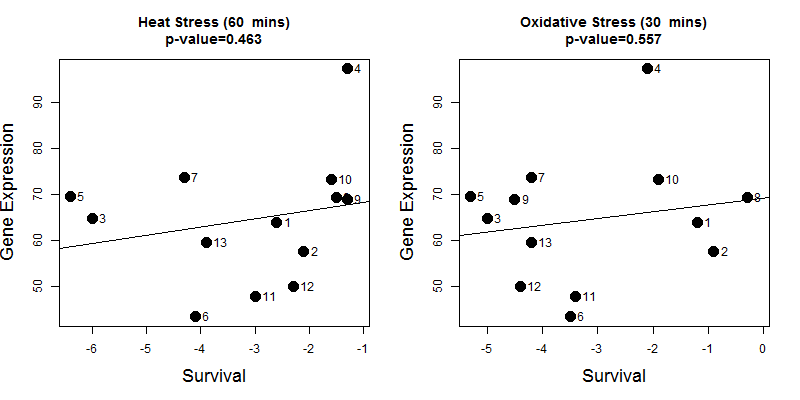

Supplement: S1 File — Expression levels of genes L0001 –L75633 plotted against survival after 60 minutes heat and 30 min oxidative stress. Survival is expressed as the difference of log CFU/ml after stress and before stress. Numbers indicate fermentations as presented in Table 1. P-values above the plots indicate significance of correlation (assessed by a linear model). (ZIP) [file pone.0167944.s006.zip › S1_File/L0152_real_dat.png]

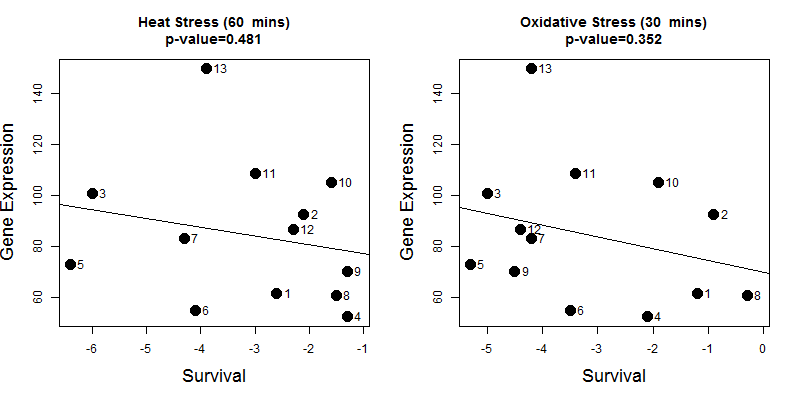

Supplement: S1 File — Expression levels of genes L0001 –L75633 plotted against survival after 60 minutes heat and 30 min oxidative stress. Survival is expressed as the difference of log CFU/ml after stress and before stress. Numbers indicate fermentations as presented in Table 1. P-values above the plots indicate significance of correlation (assessed by a linear model). (ZIP) [file pone.0167944.s006.zip › S1_File/L0153_real_dat.png]

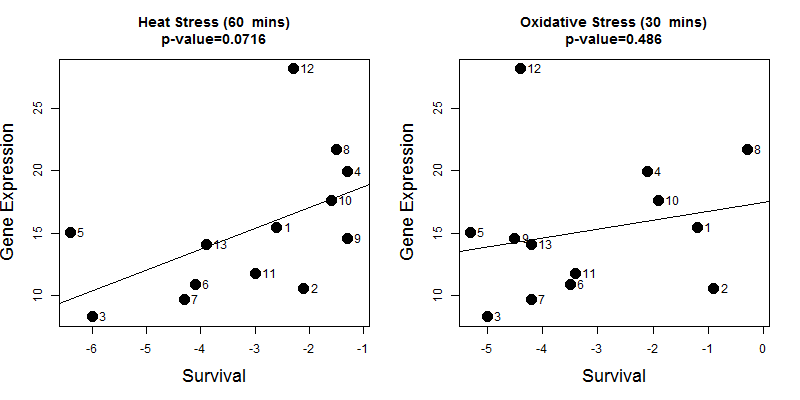

Supplement: S1 File — Expression levels of genes L0001 –L75633 plotted against survival after 60 minutes heat and 30 min oxidative stress. Survival is expressed as the difference of log CFU/ml after stress and before stress. Numbers indicate fermentations as presented in Table 1. P-values above the plots indicate significance of correlation (assessed by a linear model). (ZIP) [file pone.0167944.s006.zip › S1_File/L0154_real_dat.png]

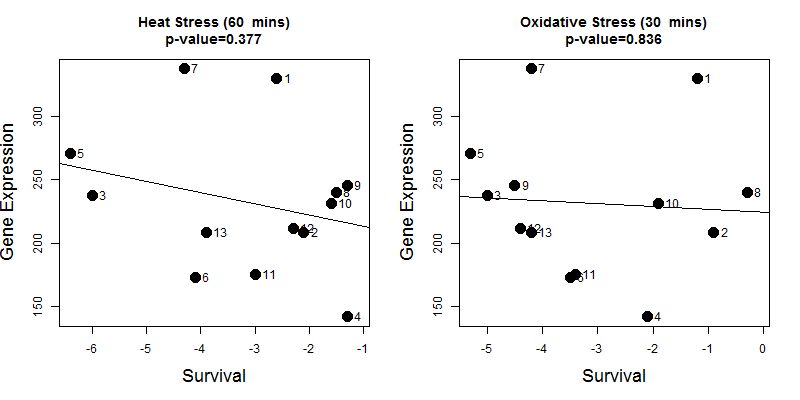

Supplement: S1 File — Expression levels of genes L0001 –L75633 plotted against survival after 60 minutes heat and 30 min oxidative stress. Survival is expressed as the difference of log CFU/ml after stress and before stress. Numbers indicate fermentations as presented in Table 1. P-values above the plots indicate significance of correlation (assessed by a linear model). (ZIP) [file pone.0167944.s006.zip › S1_File/L0155_real_dat.png]

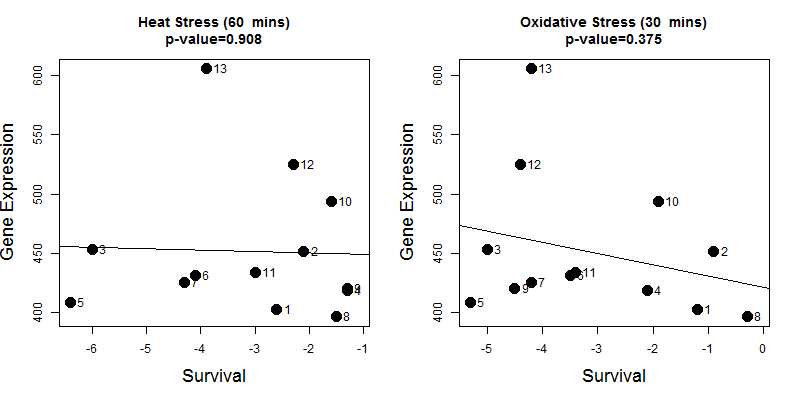

Supplement: S1 File — Expression levels of genes L0001 –L75633 plotted against survival after 60 minutes heat and 30 min oxidative stress. Survival is expressed as the difference of log CFU/ml after stress and before stress. Numbers indicate fermentations as presented in Table 1. P-values above the plots indicate significance of correlation (assessed by a linear model). (ZIP) [file pone.0167944.s006.zip › S1_File/L0156_real_dat.png]

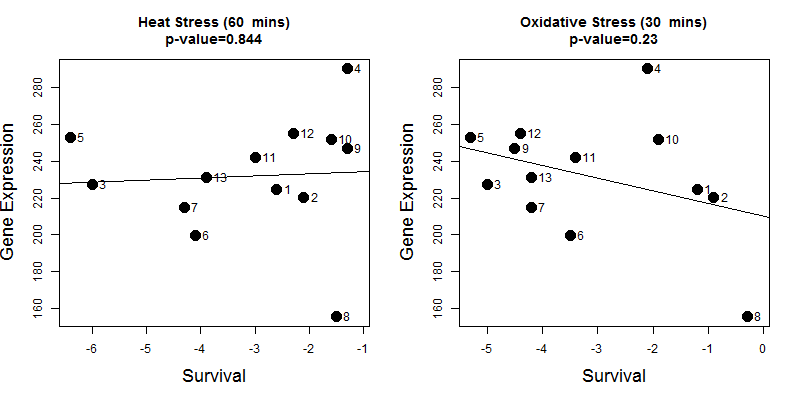

Supplement: S1 File — Expression levels of genes L0001 –L75633 plotted against survival after 60 minutes heat and 30 min oxidative stress. Survival is expressed as the difference of log CFU/ml after stress and before stress. Numbers indicate fermentations as presented in Table 1. P-values above the plots indicate significance of correlation (assessed by a linear model). (ZIP) [file pone.0167944.s006.zip › S1_File/L0157_real_dat.png]

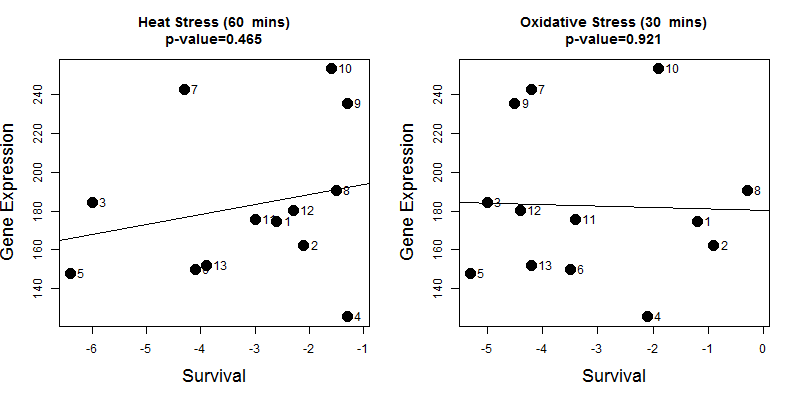

Supplement: S1 File — Expression levels of genes L0001 –L75633 plotted against survival after 60 minutes heat and 30 min oxidative stress. Survival is expressed as the difference of log CFU/ml after stress and before stress. Numbers indicate fermentations as presented in Table 1. P-values above the plots indicate significance of correlation (assessed by a linear model). (ZIP) [file pone.0167944.s006.zip › S1_File/L0158_real_dat.png]

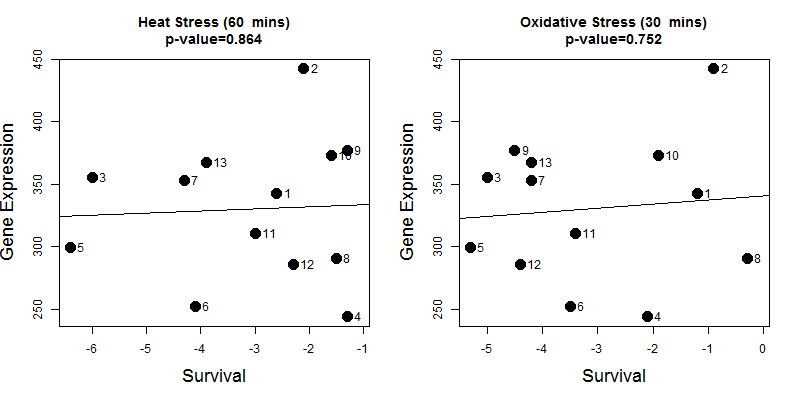

Supplement: S1 File — Expression levels of genes L0001 –L75633 plotted against survival after 60 minutes heat and 30 min oxidative stress. Survival is expressed as the difference of log CFU/ml after stress and before stress. Numbers indicate fermentations as presented in Table 1. P-values above the plots indicate significance of correlation (assessed by a linear model). (ZIP) [file pone.0167944.s006.zip › S1_File/L0159_real_dat.png]

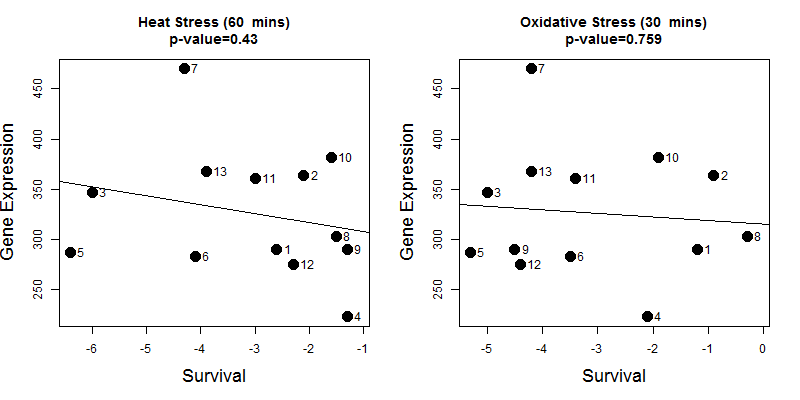

Supplement: S1 File — Expression levels of genes L0001 –L75633 plotted against survival after 60 minutes heat and 30 min oxidative stress. Survival is expressed as the difference of log CFU/ml after stress and before stress. Numbers indicate fermentations as presented in Table 1. P-values above the plots indicate significance of correlation (assessed by a linear model). (ZIP) [file pone.0167944.s006.zip › S1_File/L0160_real_dat.png]

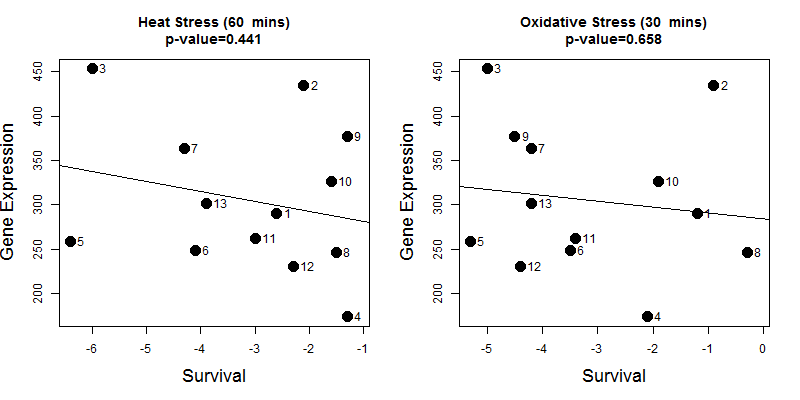

Supplement: S1 File — Expression levels of genes L0001 –L75633 plotted against survival after 60 minutes heat and 30 min oxidative stress. Survival is expressed as the difference of log CFU/ml after stress and before stress. Numbers indicate fermentations as presented in Table 1. P-values above the plots indicate significance of correlation (assessed by a linear model). (ZIP) [file pone.0167944.s006.zip › S1_File/L0161_real_dat.png]

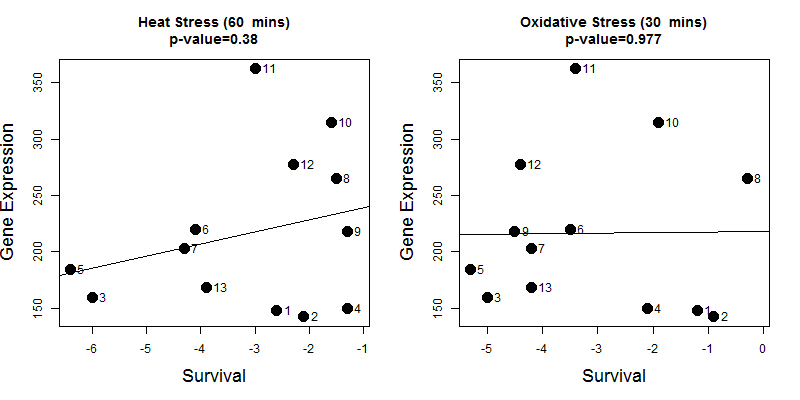

Supplement: S1 File — Expression levels of genes L0001 –L75633 plotted against survival after 60 minutes heat and 30 min oxidative stress. Survival is expressed as the difference of log CFU/ml after stress and before stress. Numbers indicate fermentations as presented in Table 1. P-values above the plots indicate significance of correlation (assessed by a linear model). (ZIP) [file pone.0167944.s006.zip › S1_File/L0162_real_dat.png]

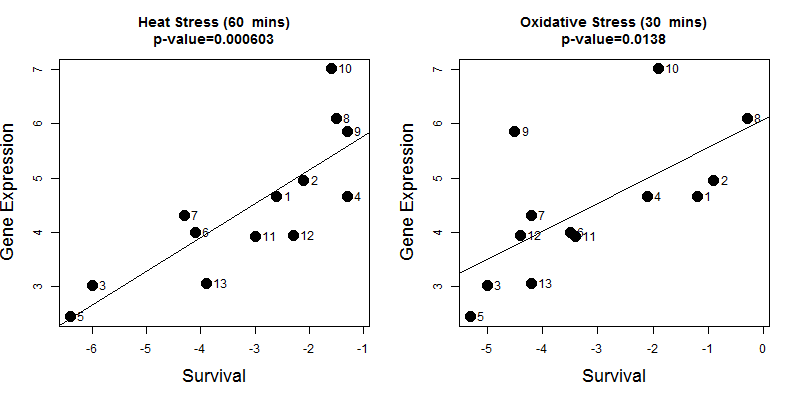

Supplement: S1 File — Expression levels of genes L0001 –L75633 plotted against survival after 60 minutes heat and 30 min oxidative stress. Survival is expressed as the difference of log CFU/ml after stress and before stress. Numbers indicate fermentations as presented in Table 1. P-values above the plots indicate significance of correlation (assessed by a linear model). (ZIP) [file pone.0167944.s006.zip › S1_File/L0163_real_dat.png]

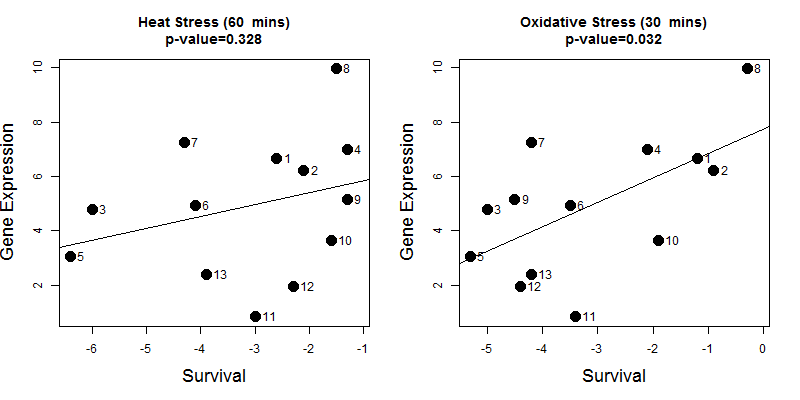

Supplement: S1 File — Expression levels of genes L0001 –L75633 plotted against survival after 60 minutes heat and 30 min oxidative stress. Survival is expressed as the difference of log CFU/ml after stress and before stress. Numbers indicate fermentations as presented in Table 1. P-values above the plots indicate significance of correlation (assessed by a linear model). (ZIP) [file pone.0167944.s006.zip › S1_File/L0164_real_dat.png]

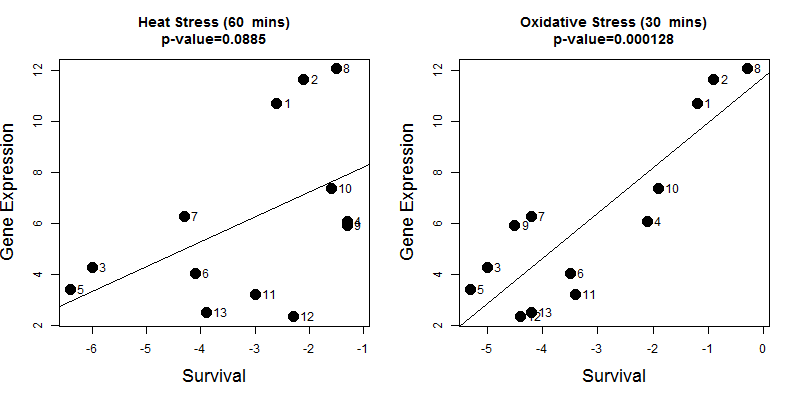

Supplement: S1 File — Expression levels of genes L0001 –L75633 plotted against survival after 60 minutes heat and 30 min oxidative stress. Survival is expressed as the difference of log CFU/ml after stress and before stress. Numbers indicate fermentations as presented in Table 1. P-values above the plots indicate significance of correlation (assessed by a linear model). (ZIP) [file pone.0167944.s006.zip › S1_File/L0165_real_dat.png]

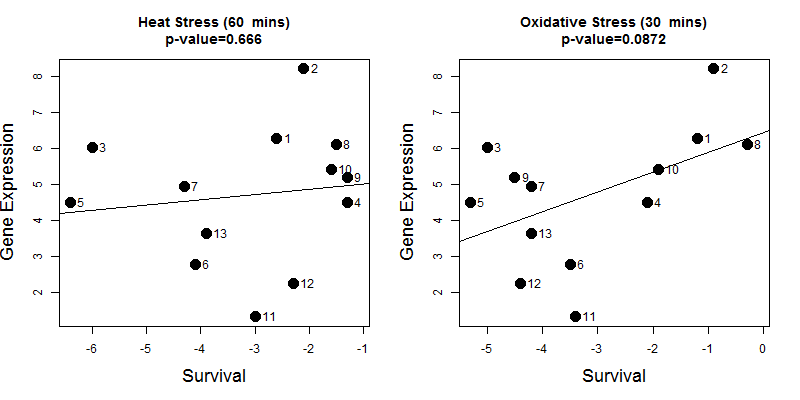

Supplement: S1 File — Expression levels of genes L0001 –L75633 plotted against survival after 60 minutes heat and 30 min oxidative stress. Survival is expressed as the difference of log CFU/ml after stress and before stress. Numbers indicate fermentations as presented in Table 1. P-values above the plots indicate significance of correlation (assessed by a linear model). (ZIP) [file pone.0167944.s006.zip › S1_File/L0166_real_dat.png]

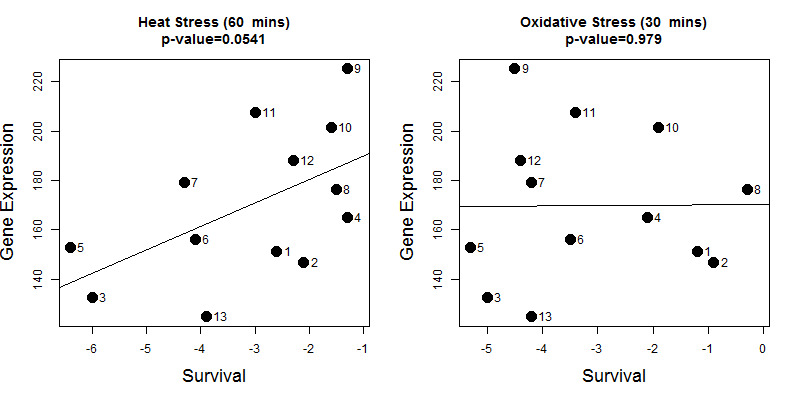

Supplement: S1 File — Expression levels of genes L0001 –L75633 plotted against survival after 60 minutes heat and 30 min oxidative stress. Survival is expressed as the difference of log CFU/ml after stress and before stress. Numbers indicate fermentations as presented in Table 1. P-values above the plots indicate significance of correlation (assessed by a linear model). (ZIP) [file pone.0167944.s006.zip › S1_File/L0167_real_dat.png]

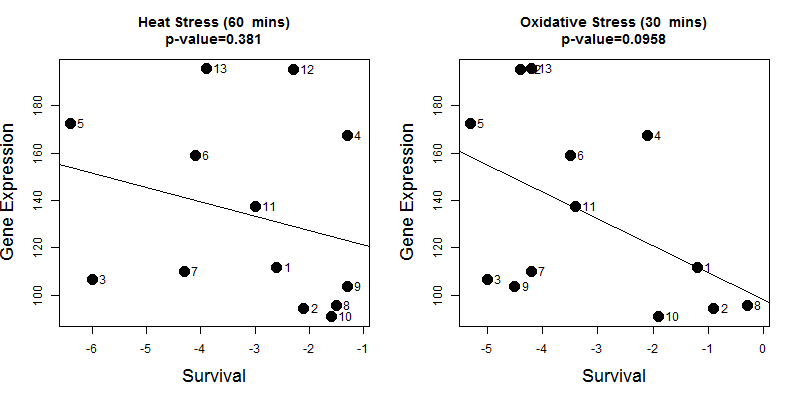

Supplement: S1 File — Expression levels of genes L0001 –L75633 plotted against survival after 60 minutes heat and 30 min oxidative stress. Survival is expressed as the difference of log CFU/ml after stress and before stress. Numbers indicate fermentations as presented in Table 1. P-values above the plots indicate significance of correlation (assessed by a linear model). (ZIP) [file pone.0167944.s006.zip › S1_File/L0168_real_dat.png]

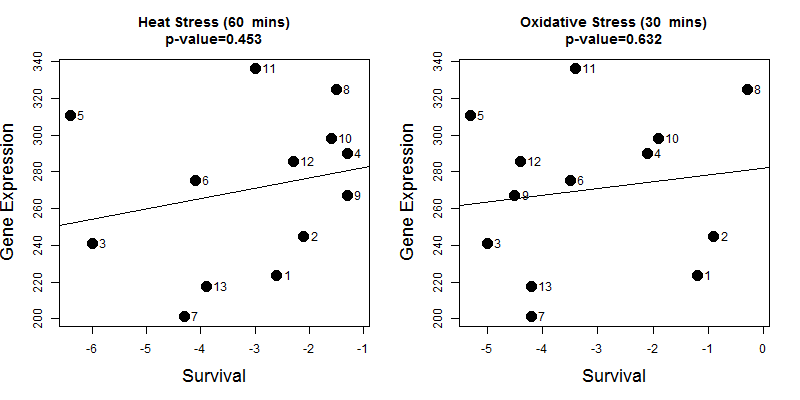

Supplement: S1 File — Expression levels of genes L0001 –L75633 plotted against survival after 60 minutes heat and 30 min oxidative stress. Survival is expressed as the difference of log CFU/ml after stress and before stress. Numbers indicate fermentations as presented in Table 1. P-values above the plots indicate significance of correlation (assessed by a linear model). (ZIP) [file pone.0167944.s006.zip › S1_File/L0169_real_dat.png]

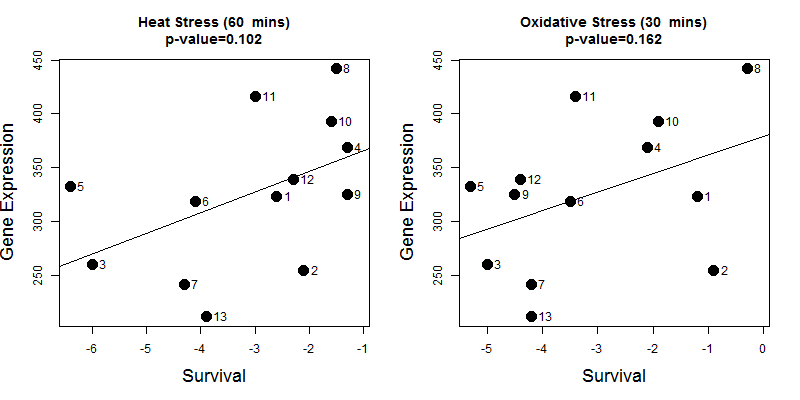

Supplement: S1 File — Expression levels of genes L0001 –L75633 plotted against survival after 60 minutes heat and 30 min oxidative stress. Survival is expressed as the difference of log CFU/ml after stress and before stress. Numbers indicate fermentations as presented in Table 1. P-values above the plots indicate significance of correlation (assessed by a linear model). (ZIP) [file pone.0167944.s006.zip › S1_File/L0170_real_dat.png]

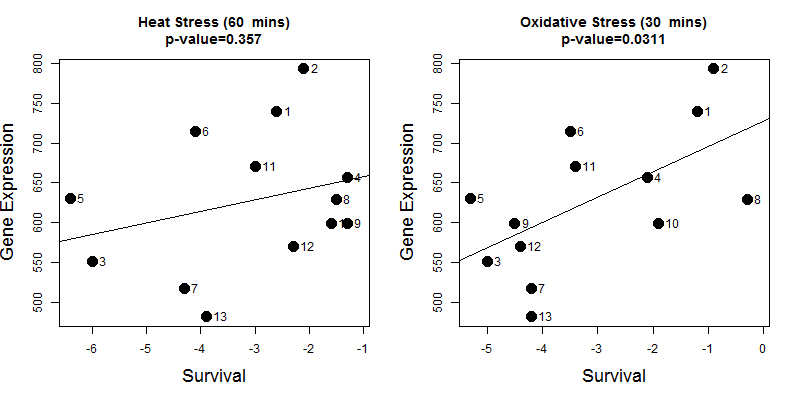

Supplement: S1 File — Expression levels of genes L0001 –L75633 plotted against survival after 60 minutes heat and 30 min oxidative stress. Survival is expressed as the difference of log CFU/ml after stress and before stress. Numbers indicate fermentations as presented in Table 1. P-values above the plots indicate significance of correlation (assessed by a linear model). (ZIP) [file pone.0167944.s006.zip › S1_File/L0171_real_dat.png]

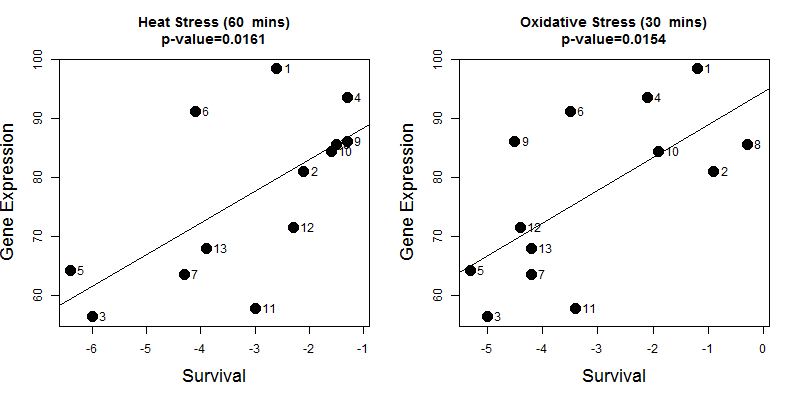

Supplement: S1 File — Expression levels of genes L0001 –L75633 plotted against survival after 60 minutes heat and 30 min oxidative stress. Survival is expressed as the difference of log CFU/ml after stress and before stress. Numbers indicate fermentations as presented in Table 1. P-values above the plots indicate significance of correlation (assessed by a linear model). (ZIP) [file pone.0167944.s006.zip › S1_File/L0172_real_dat.png]

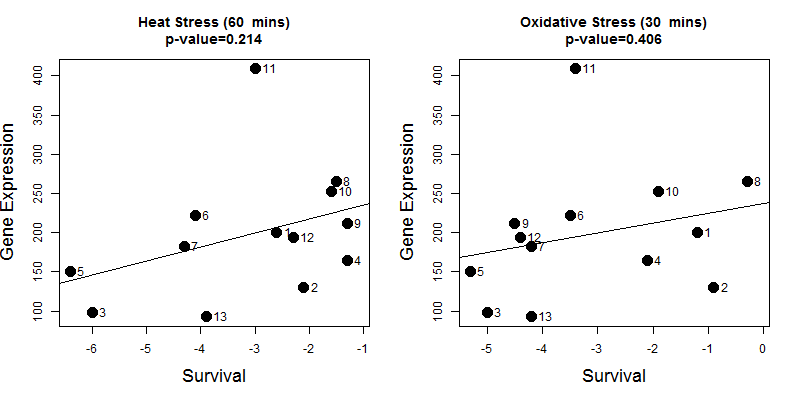

Supplement: S1 File — Expression levels of genes L0001 –L75633 plotted against survival after 60 minutes heat and 30 min oxidative stress. Survival is expressed as the difference of log CFU/ml after stress and before stress. Numbers indicate fermentations as presented in Table 1. P-values above the plots indicate significance of correlation (assessed by a linear model). (ZIP) [file pone.0167944.s006.zip › S1_File/L0175_real_dat.png]

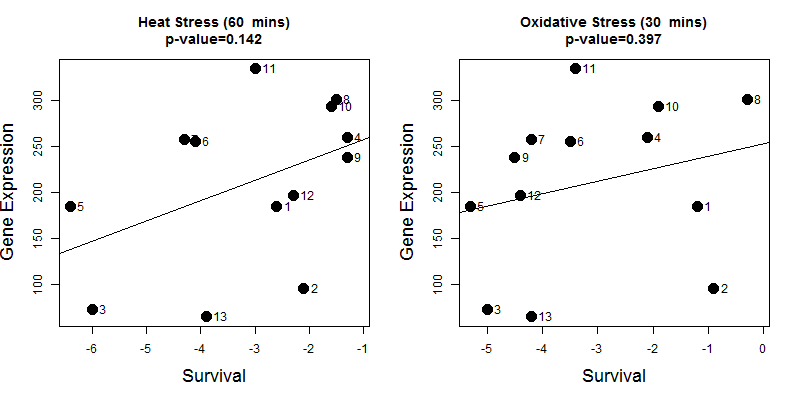

Supplement: S1 File — Expression levels of genes L0001 –L75633 plotted against survival after 60 minutes heat and 30 min oxidative stress. Survival is expressed as the difference of log CFU/ml after stress and before stress. Numbers indicate fermentations as presented in Table 1. P-values above the plots indicate significance of correlation (assessed by a linear model). (ZIP) [file pone.0167944.s006.zip › S1_File/L0176_real_dat.png]

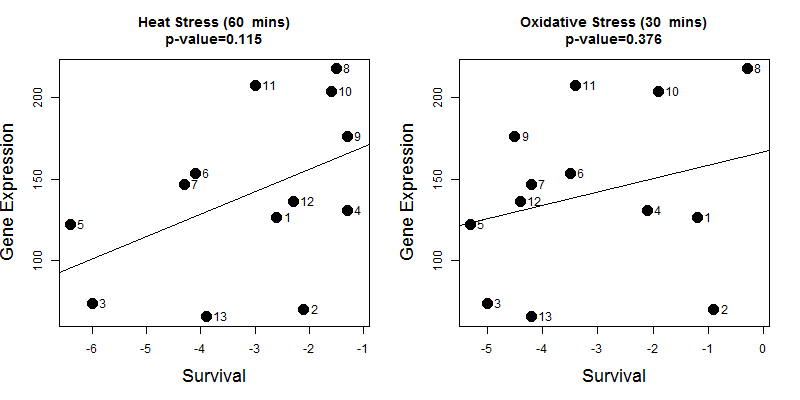

Supplement: S1 File — Expression levels of genes L0001 –L75633 plotted against survival after 60 minutes heat and 30 min oxidative stress. Survival is expressed as the difference of log CFU/ml after stress and before stress. Numbers indicate fermentations as presented in Table 1. P-values above the plots indicate significance of correlation (assessed by a linear model). (ZIP) [file pone.0167944.s006.zip › S1_File/L0177_real_dat.png]

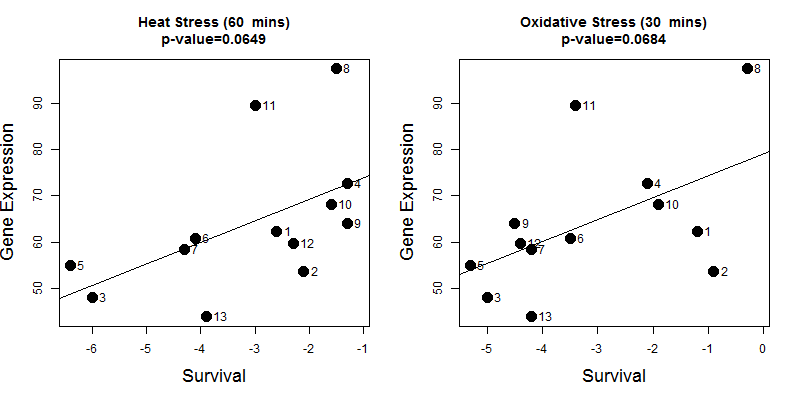

Supplement: S1 File — Expression levels of genes L0001 –L75633 plotted against survival after 60 minutes heat and 30 min oxidative stress. Survival is expressed as the difference of log CFU/ml after stress and before stress. Numbers indicate fermentations as presented in Table 1. P-values above the plots indicate significance of correlation (assessed by a linear model). (ZIP) [file pone.0167944.s006.zip › S1_File/L0178_real_dat.png]

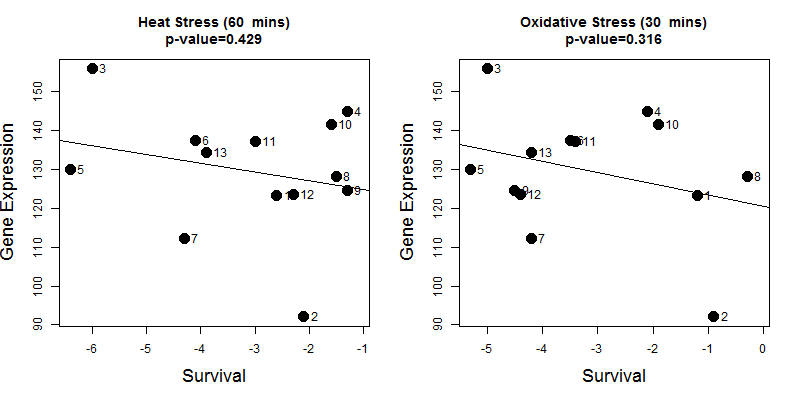

Supplement: S1 File — Expression levels of genes L0001 –L75633 plotted against survival after 60 minutes heat and 30 min oxidative stress. Survival is expressed as the difference of log CFU/ml after stress and before stress. Numbers indicate fermentations as presented in Table 1. P-values above the plots indicate significance of correlation (assessed by a linear model). (ZIP) [file pone.0167944.s006.zip › S1_File/L0179_real_dat.png]

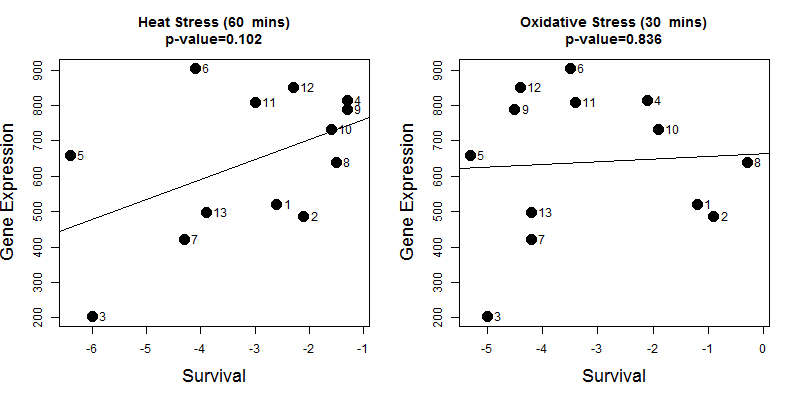

Supplement: S1 File — Expression levels of genes L0001 –L75633 plotted against survival after 60 minutes heat and 30 min oxidative stress. Survival is expressed as the difference of log CFU/ml after stress and before stress. Numbers indicate fermentations as presented in Table 1. P-values above the plots indicate significance of correlation (assessed by a linear model). (ZIP) [file pone.0167944.s006.zip › S1_File/L0180_real_dat.png]

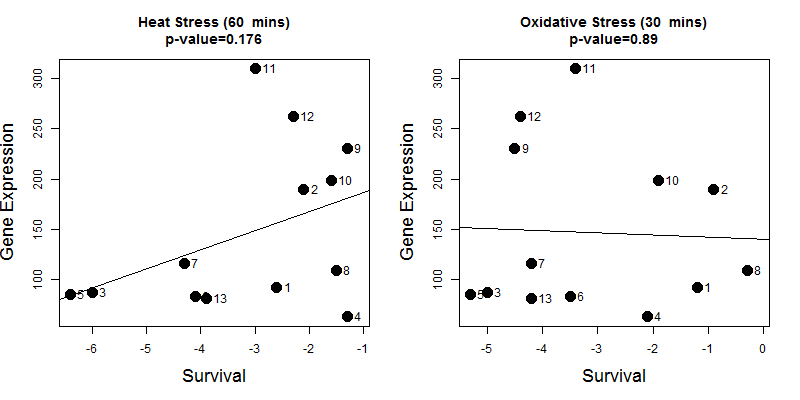

Supplement: S1 File — Expression levels of genes L0001 –L75633 plotted against survival after 60 minutes heat and 30 min oxidative stress. Survival is expressed as the difference of log CFU/ml after stress and before stress. Numbers indicate fermentations as presented in Table 1. P-values above the plots indicate significance of correlation (assessed by a linear model). (ZIP) [file pone.0167944.s006.zip › S1_File/L0181_real_dat.png]

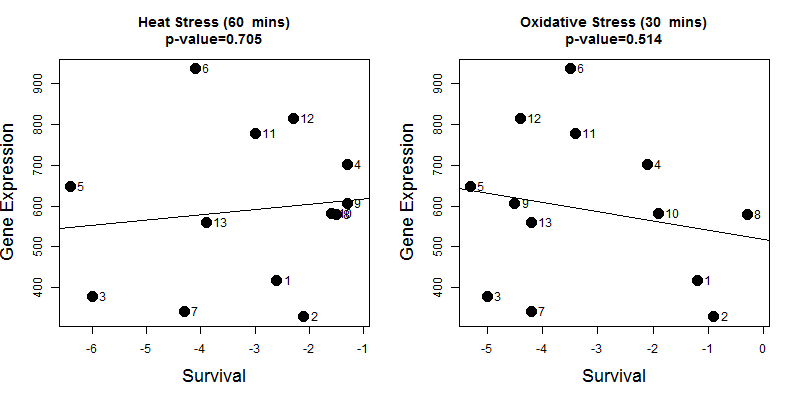

Supplement: S1 File — Expression levels of genes L0001 –L75633 plotted against survival after 60 minutes heat and 30 min oxidative stress. Survival is expressed as the difference of log CFU/ml after stress and before stress. Numbers indicate fermentations as presented in Table 1. P-values above the plots indicate significance of correlation (assessed by a linear model). (ZIP) [file pone.0167944.s006.zip › S1_File/L0182_real_dat.png]

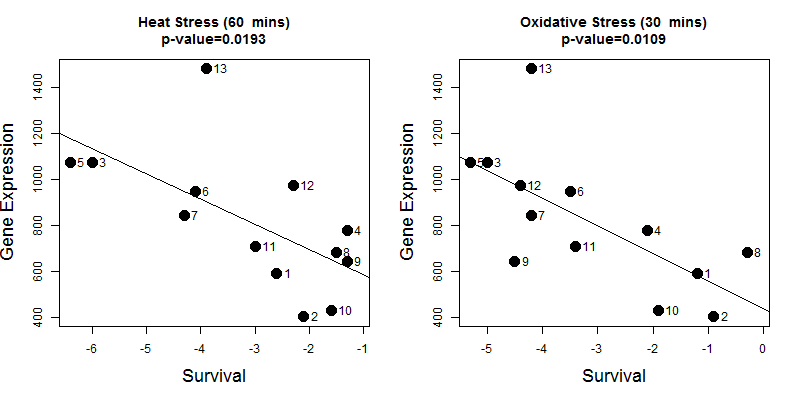

Supplement: S1 File — Expression levels of genes L0001 –L75633 plotted against survival after 60 minutes heat and 30 min oxidative stress. Survival is expressed as the difference of log CFU/ml after stress and before stress. Numbers indicate fermentations as presented in Table 1. P-values above the plots indicate significance of correlation (assessed by a linear model). (ZIP) [file pone.0167944.s006.zip › S1_File/L0183_real_dat.png]

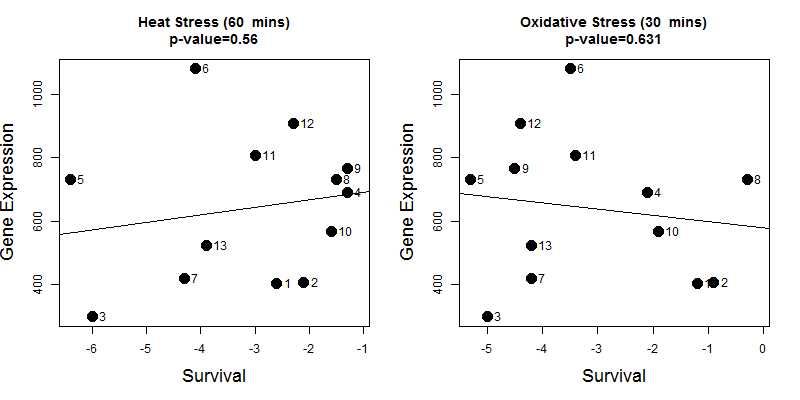

Supplement: S1 File — Expression levels of genes L0001 –L75633 plotted against survival after 60 minutes heat and 30 min oxidative stress. Survival is expressed as the difference of log CFU/ml after stress and before stress. Numbers indicate fermentations as presented in Table 1. P-values above the plots indicate significance of correlation (assessed by a linear model). (ZIP) [file pone.0167944.s006.zip › S1_File/L0184_real_dat.png]

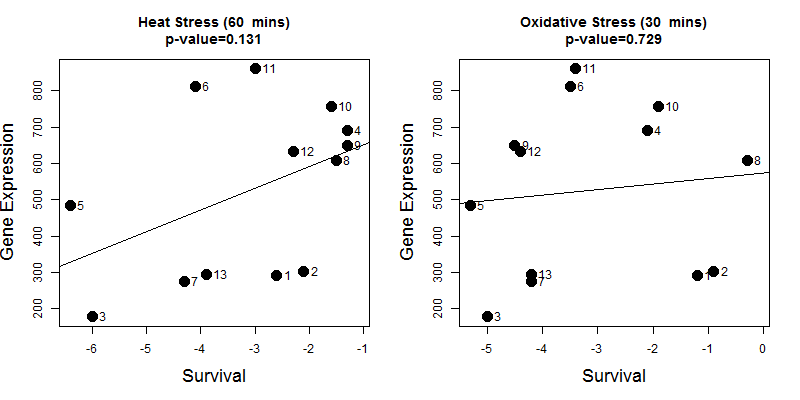

Supplement: S1 File — Expression levels of genes L0001 –L75633 plotted against survival after 60 minutes heat and 30 min oxidative stress. Survival is expressed as the difference of log CFU/ml after stress and before stress. Numbers indicate fermentations as presented in Table 1. P-values above the plots indicate significance of correlation (assessed by a linear model). (ZIP) [file pone.0167944.s006.zip › S1_File/L0185_real_dat.png]

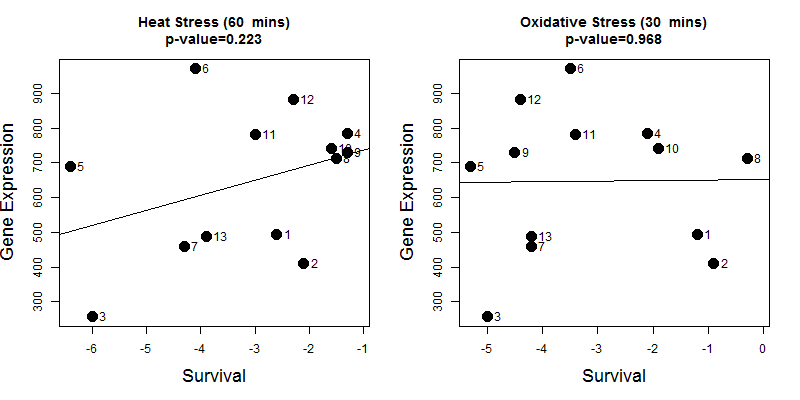

Supplement: S1 File — Expression levels of genes L0001 –L75633 plotted against survival after 60 minutes heat and 30 min oxidative stress. Survival is expressed as the difference of log CFU/ml after stress and before stress. Numbers indicate fermentations as presented in Table 1. P-values above the plots indicate significance of correlation (assessed by a linear model). (ZIP) [file pone.0167944.s006.zip › S1_File/L0186_real_dat.png]

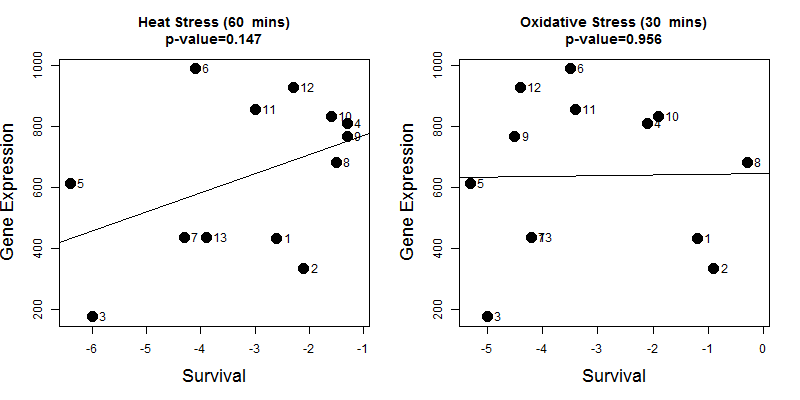

Supplement: S1 File — Expression levels of genes L0001 –L75633 plotted against survival after 60 minutes heat and 30 min oxidative stress. Survival is expressed as the difference of log CFU/ml after stress and before stress. Numbers indicate fermentations as presented in Table 1. P-values above the plots indicate significance of correlation (assessed by a linear model). (ZIP) [file pone.0167944.s006.zip › S1_File/L0187_real_dat.png]

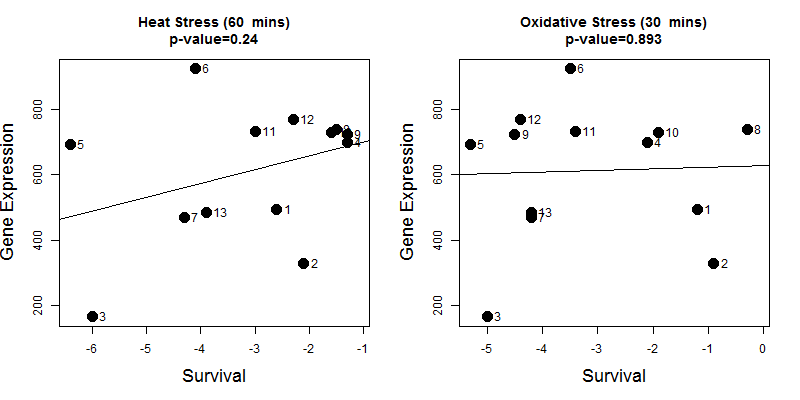

Supplement: S1 File — Expression levels of genes L0001 –L75633 plotted against survival after 60 minutes heat and 30 min oxidative stress. Survival is expressed as the difference of log CFU/ml after stress and before stress. Numbers indicate fermentations as presented in Table 1. P-values above the plots indicate significance of correlation (assessed by a linear model). (ZIP) [file pone.0167944.s006.zip › S1_File/L0188_real_dat.png]

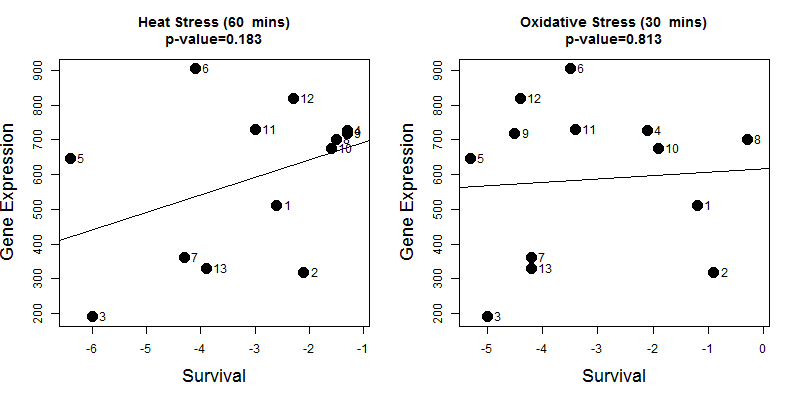

Supplement: S1 File — Expression levels of genes L0001 –L75633 plotted against survival after 60 minutes heat and 30 min oxidative stress. Survival is expressed as the difference of log CFU/ml after stress and before stress. Numbers indicate fermentations as presented in Table 1. P-values above the plots indicate significance of correlation (assessed by a linear model). (ZIP) [file pone.0167944.s006.zip › S1_File/L0189_real_dat.png]

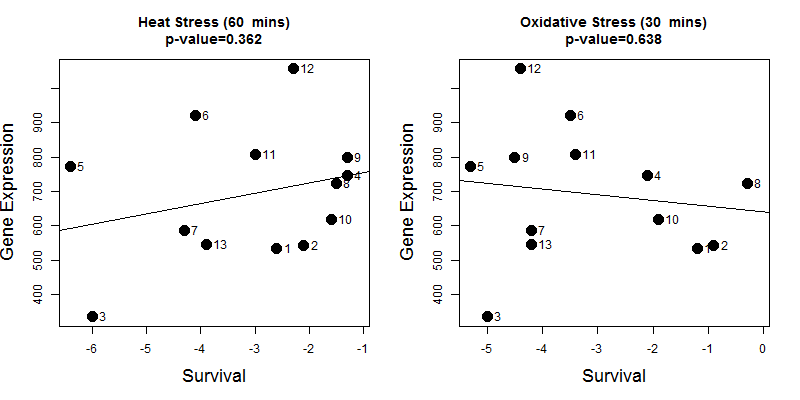

Supplement: S1 File — Expression levels of genes L0001 –L75633 plotted against survival after 60 minutes heat and 30 min oxidative stress. Survival is expressed as the difference of log CFU/ml after stress and before stress. Numbers indicate fermentations as presented in Table 1. P-values above the plots indicate significance of correlation (assessed by a linear model). (ZIP) [file pone.0167944.s006.zip › S1_File/L0190_real_dat.png]

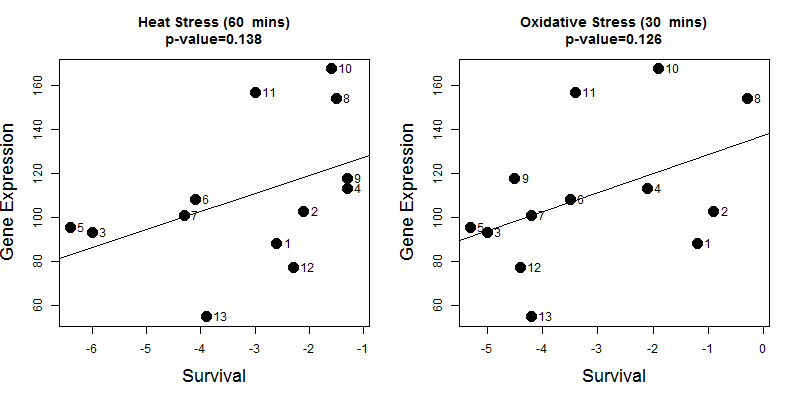

Supplement: S1 File — Expression levels of genes L0001 –L75633 plotted against survival after 60 minutes heat and 30 min oxidative stress. Survival is expressed as the difference of log CFU/ml after stress and before stress. Numbers indicate fermentations as presented in Table 1. P-values above the plots indicate significance of correlation (assessed by a linear model). (ZIP) [file pone.0167944.s006.zip › S1_File/L0191_real_dat.png]

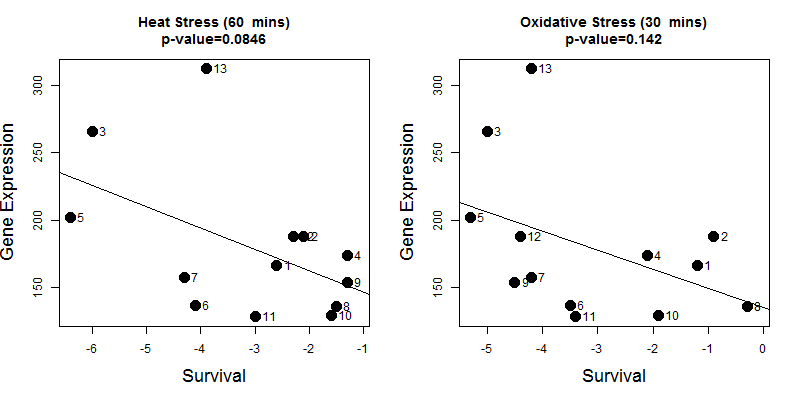

Supplement: S1 File — Expression levels of genes L0001 –L75633 plotted against survival after 60 minutes heat and 30 min oxidative stress. Survival is expressed as the difference of log CFU/ml after stress and before stress. Numbers indicate fermentations as presented in Table 1. P-values above the plots indicate significance of correlation (assessed by a linear model). (ZIP) [file pone.0167944.s006.zip › S1_File/L0192_real_dat.png]

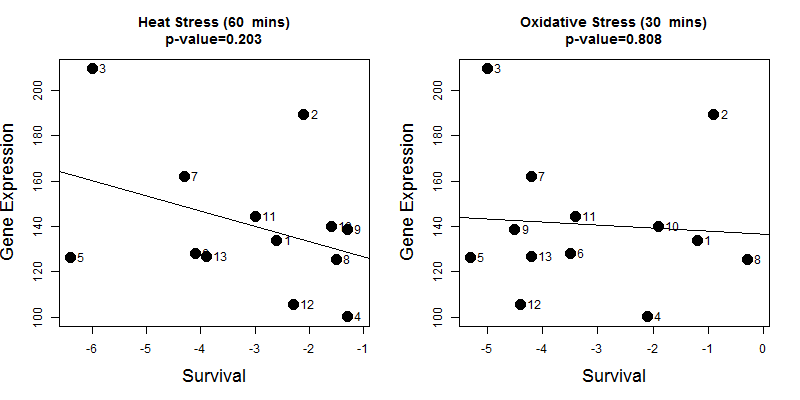

Supplement: S1 File — Expression levels of genes L0001 –L75633 plotted against survival after 60 minutes heat and 30 min oxidative stress. Survival is expressed as the difference of log CFU/ml after stress and before stress. Numbers indicate fermentations as presented in Table 1. P-values above the plots indicate significance of correlation (assessed by a linear model). (ZIP) [file pone.0167944.s006.zip › S1_File/L0193_real_dat.png]

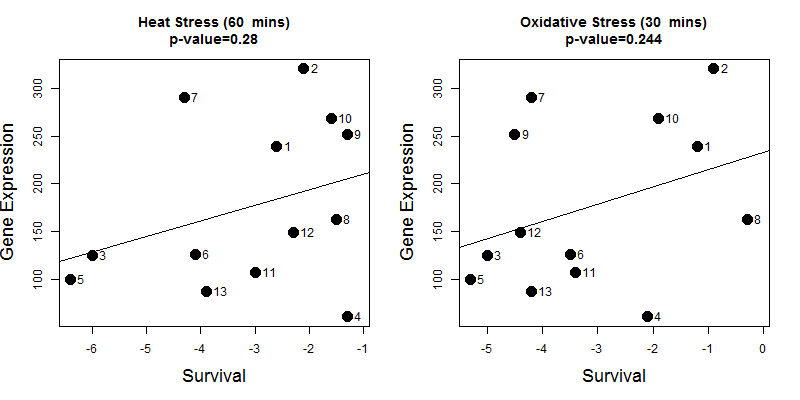

Supplement: S1 File — Expression levels of genes L0001 –L75633 plotted against survival after 60 minutes heat and 30 min oxidative stress. Survival is expressed as the difference of log CFU/ml after stress and before stress. Numbers indicate fermentations as presented in Table 1. P-values above the plots indicate significance of correlation (assessed by a linear model). (ZIP) [file pone.0167944.s006.zip › S1_File/L0194_real_dat.png]

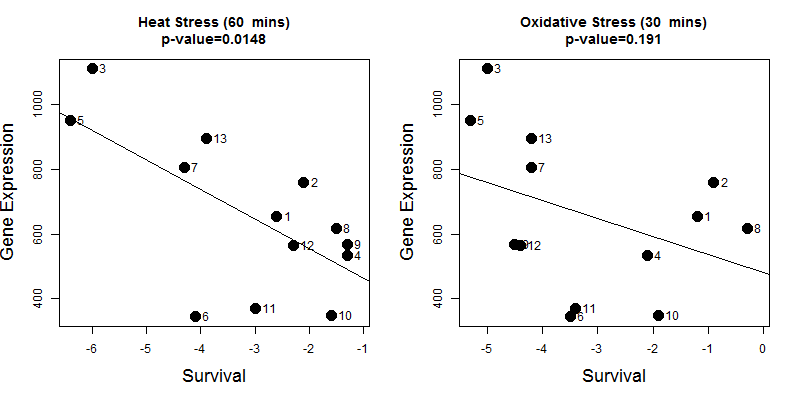

Supplement: S1 File — Expression levels of genes L0001 –L75633 plotted against survival after 60 minutes heat and 30 min oxidative stress. Survival is expressed as the difference of log CFU/ml after stress and before stress. Numbers indicate fermentations as presented in Table 1. P-values above the plots indicate significance of correlation (assessed by a linear model). (ZIP) [file pone.0167944.s006.zip › S1_File/L0195_real_dat.png]

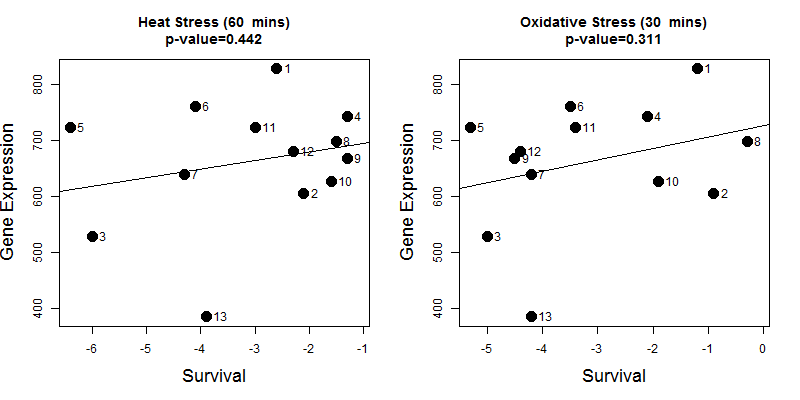

Supplement: S1 File — Expression levels of genes L0001 –L75633 plotted against survival after 60 minutes heat and 30 min oxidative stress. Survival is expressed as the difference of log CFU/ml after stress and before stress. Numbers indicate fermentations as presented in Table 1. P-values above the plots indicate significance of correlation (assessed by a linear model). (ZIP) [file pone.0167944.s006.zip › S1_File/L0196_real_dat.png]
