# Supplementary figures and images for: Strain-Dependent Transcriptome Signatures for Robustness in Lactococcus lactis (part 5 of 13)
Source: PLoS One. 2016 Dec 14;11(12):e0167944. doi: 10.1371/journal.pone.0167944 (PMC5156439; doi:10.1371/journal.pone.0167944)

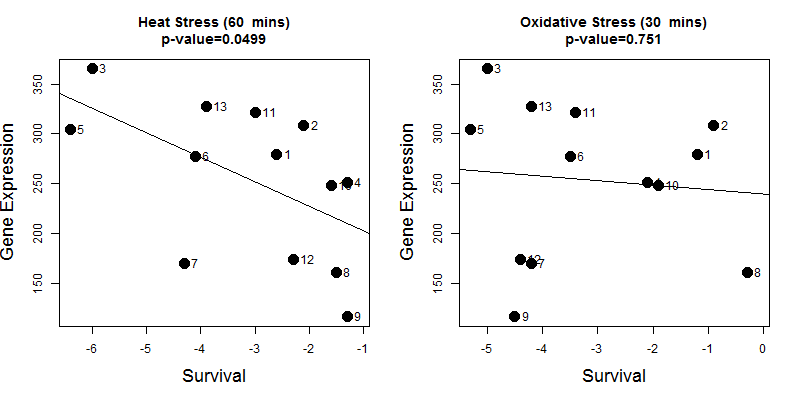

Supplement: S2 File — Expression levels of genes L75676 –L1889726 plotted against survival after 60 minutes heat and 30 min oxidative stress. Survival is expressed as the difference of log CFU/ml after stress and before stress. Numbers indicate fermentations as presented in Table 1. P-values above the plots indicate significance of correlation (assessed by a linear model). (ZIP) [file pone.0167944.s007.zip › S2_File/L119891_real_dat.png]

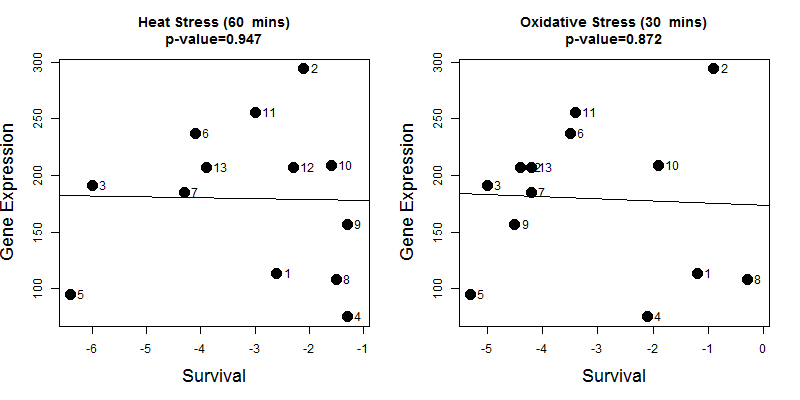

Supplement: S2 File — Expression levels of genes L75676 –L1889726 plotted against survival after 60 minutes heat and 30 min oxidative stress. Survival is expressed as the difference of log CFU/ml after stress and before stress. Numbers indicate fermentations as presented in Table 1. P-values above the plots indicate significance of correlation (assessed by a linear model). (ZIP) [file pone.0167944.s007.zip › S2_File/L120334_real_dat.png]

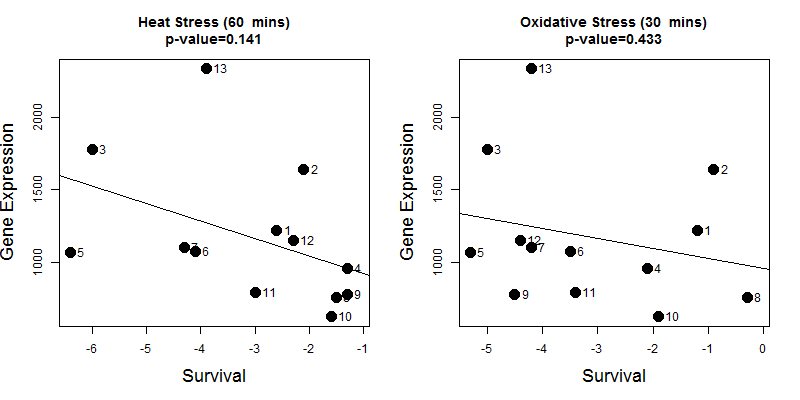

Supplement: S2 File — Expression levels of genes L75676 –L1889726 plotted against survival after 60 minutes heat and 30 min oxidative stress. Survival is expressed as the difference of log CFU/ml after stress and before stress. Numbers indicate fermentations as presented in Table 1. P-values above the plots indicate significance of correlation (assessed by a linear model). (ZIP) [file pone.0167944.s007.zip › S2_File/L120335_real_dat.png]

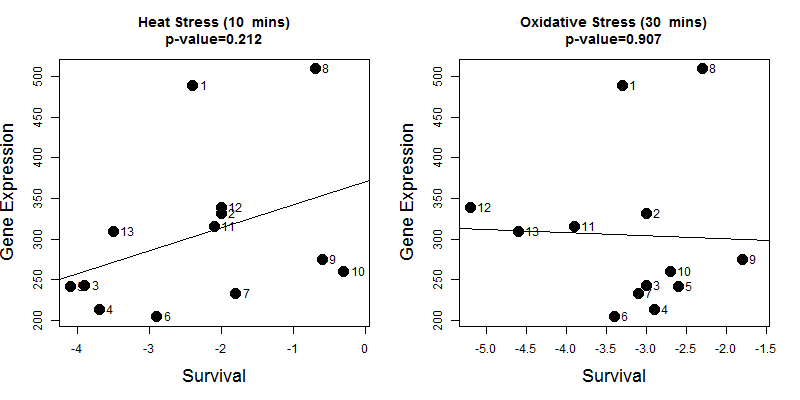

Supplement: S3 File — Expression levels of genes LLKF_0001 –LLKF_1273 plotted against survival after 10 minutes heat and 30 minutes oxidative stress. Survival is expressed as the difference of log CFU/ml after stress and before stress. Numbers indicate fermentations as presented in Table 1. P-values above the plots indicate significance of correlation (assessed by a linear model). (ZIP) [file pone.0167944.s008.zip › S3_File/LLKF_0001_real_dat.png]

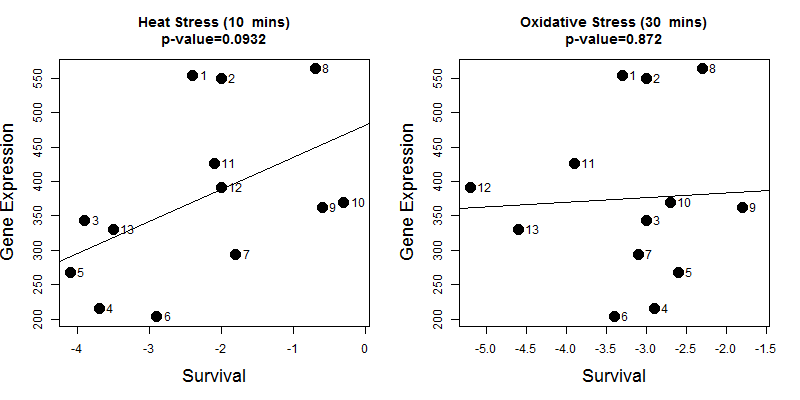

Supplement: S3 File — Expression levels of genes LLKF_0001 –LLKF_1273 plotted against survival after 10 minutes heat and 30 minutes oxidative stress. Survival is expressed as the difference of log CFU/ml after stress and before stress. Numbers indicate fermentations as presented in Table 1. P-values above the plots indicate significance of correlation (assessed by a linear model). (ZIP) [file pone.0167944.s008.zip › S3_File/LLKF_0002_real_dat.png]

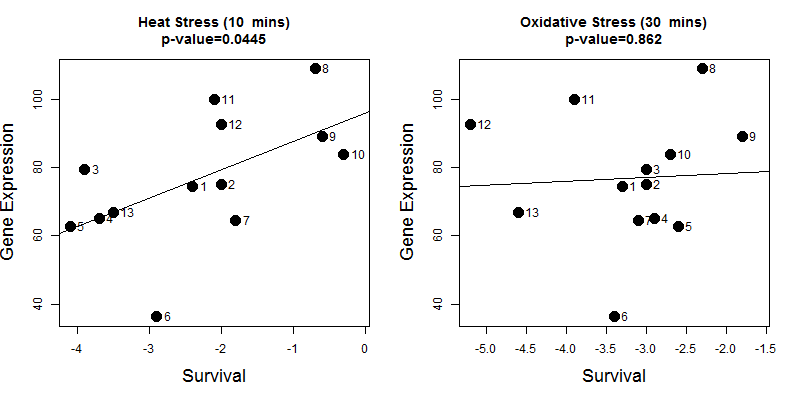

Supplement: S3 File — Expression levels of genes LLKF_0001 –LLKF_1273 plotted against survival after 10 minutes heat and 30 minutes oxidative stress. Survival is expressed as the difference of log CFU/ml after stress and before stress. Numbers indicate fermentations as presented in Table 1. P-values above the plots indicate significance of correlation (assessed by a linear model). (ZIP) [file pone.0167944.s008.zip › S3_File/LLKF_0003_real_dat.png]

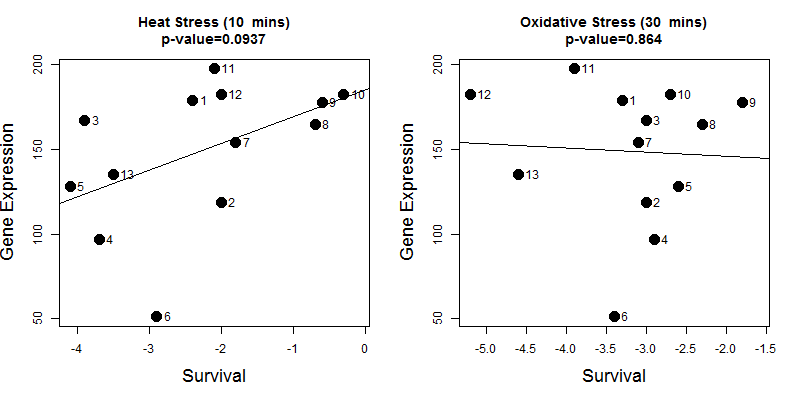

Supplement: S3 File — Expression levels of genes LLKF_0001 –LLKF_1273 plotted against survival after 10 minutes heat and 30 minutes oxidative stress. Survival is expressed as the difference of log CFU/ml after stress and before stress. Numbers indicate fermentations as presented in Table 1. P-values above the plots indicate significance of correlation (assessed by a linear model). (ZIP) [file pone.0167944.s008.zip › S3_File/LLKF_0004_real_dat.png]

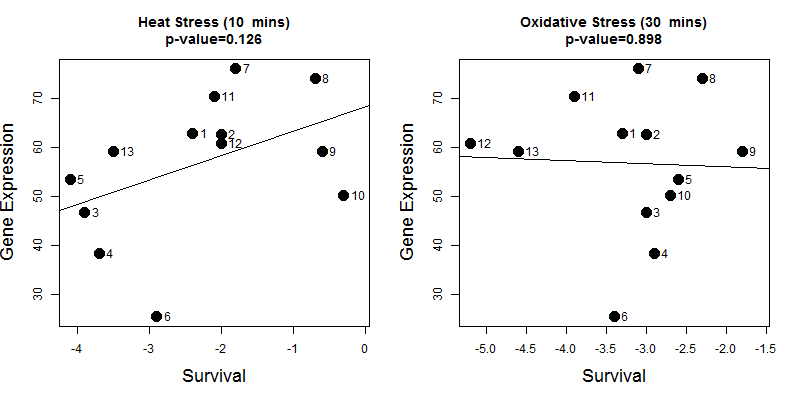

Supplement: S3 File — Expression levels of genes LLKF_0001 –LLKF_1273 plotted against survival after 10 minutes heat and 30 minutes oxidative stress. Survival is expressed as the difference of log CFU/ml after stress and before stress. Numbers indicate fermentations as presented in Table 1. P-values above the plots indicate significance of correlation (assessed by a linear model). (ZIP) [file pone.0167944.s008.zip › S3_File/LLKF_0005_real_dat.png]

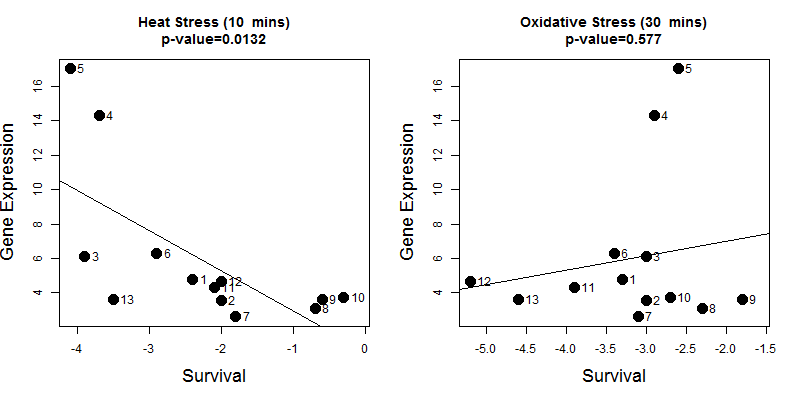

Supplement: S3 File — Expression levels of genes LLKF_0001 –LLKF_1273 plotted against survival after 10 minutes heat and 30 minutes oxidative stress. Survival is expressed as the difference of log CFU/ml after stress and before stress. Numbers indicate fermentations as presented in Table 1. P-values above the plots indicate significance of correlation (assessed by a linear model). (ZIP) [file pone.0167944.s008.zip › S3_File/LLKF_0006_real_dat.png]

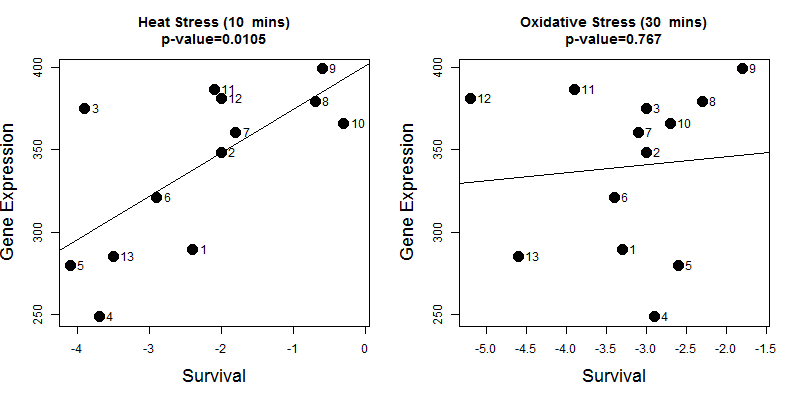

Supplement: S3 File — Expression levels of genes LLKF_0001 –LLKF_1273 plotted against survival after 10 minutes heat and 30 minutes oxidative stress. Survival is expressed as the difference of log CFU/ml after stress and before stress. Numbers indicate fermentations as presented in Table 1. P-values above the plots indicate significance of correlation (assessed by a linear model). (ZIP) [file pone.0167944.s008.zip › S3_File/LLKF_0007_real_dat.png]

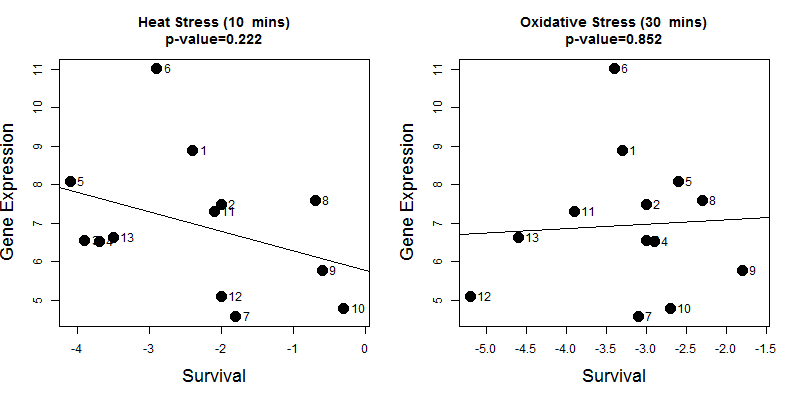

Supplement: S3 File — Expression levels of genes LLKF_0001 –LLKF_1273 plotted against survival after 10 minutes heat and 30 minutes oxidative stress. Survival is expressed as the difference of log CFU/ml after stress and before stress. Numbers indicate fermentations as presented in Table 1. P-values above the plots indicate significance of correlation (assessed by a linear model). (ZIP) [file pone.0167944.s008.zip › S3_File/LLKF_0008_real_dat.png]

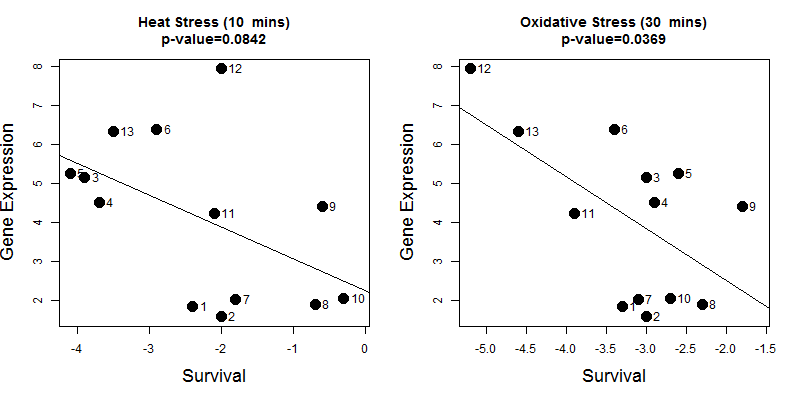

Supplement: S3 File — Expression levels of genes LLKF_0001 –LLKF_1273 plotted against survival after 10 minutes heat and 30 minutes oxidative stress. Survival is expressed as the difference of log CFU/ml after stress and before stress. Numbers indicate fermentations as presented in Table 1. P-values above the plots indicate significance of correlation (assessed by a linear model). (ZIP) [file pone.0167944.s008.zip › S3_File/LLKF_0009_real_dat.png]

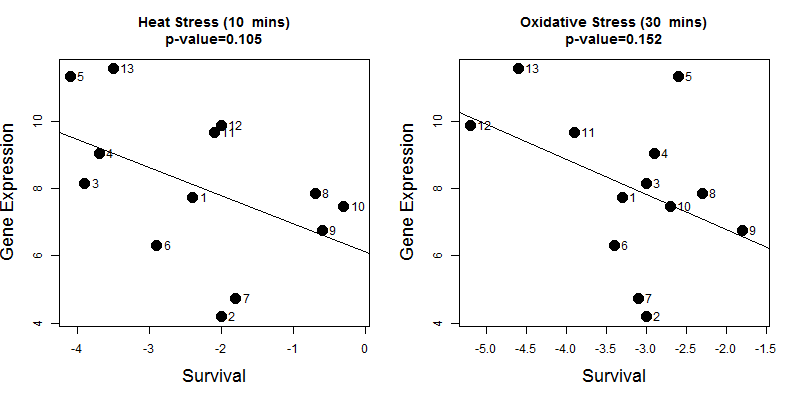

Supplement: S3 File — Expression levels of genes LLKF_0001 –LLKF_1273 plotted against survival after 10 minutes heat and 30 minutes oxidative stress. Survival is expressed as the difference of log CFU/ml after stress and before stress. Numbers indicate fermentations as presented in Table 1. P-values above the plots indicate significance of correlation (assessed by a linear model). (ZIP) [file pone.0167944.s008.zip › S3_File/LLKF_0010_real_dat.png]

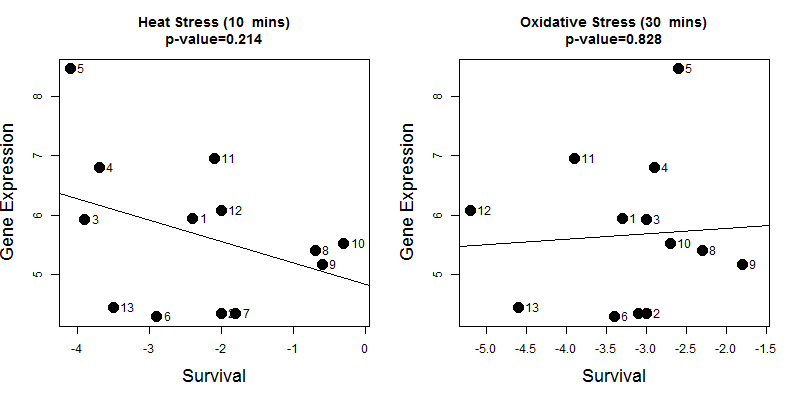

Supplement: S3 File — Expression levels of genes LLKF_0001 –LLKF_1273 plotted against survival after 10 minutes heat and 30 minutes oxidative stress. Survival is expressed as the difference of log CFU/ml after stress and before stress. Numbers indicate fermentations as presented in Table 1. P-values above the plots indicate significance of correlation (assessed by a linear model). (ZIP) [file pone.0167944.s008.zip › S3_File/LLKF_0011_real_dat.png]

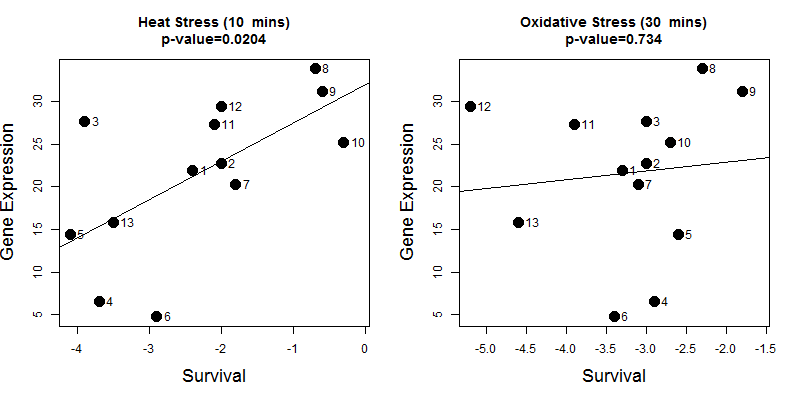

Supplement: S3 File — Expression levels of genes LLKF_0001 –LLKF_1273 plotted against survival after 10 minutes heat and 30 minutes oxidative stress. Survival is expressed as the difference of log CFU/ml after stress and before stress. Numbers indicate fermentations as presented in Table 1. P-values above the plots indicate significance of correlation (assessed by a linear model). (ZIP) [file pone.0167944.s008.zip › S3_File/LLKF_0012_real_dat.png]

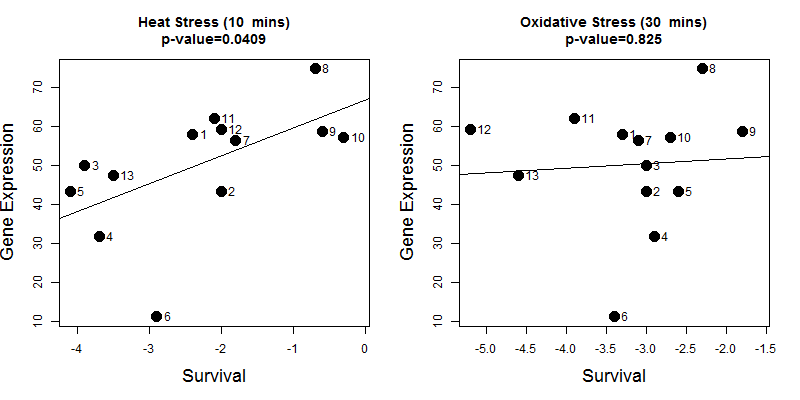

Supplement: S3 File — Expression levels of genes LLKF_0001 –LLKF_1273 plotted against survival after 10 minutes heat and 30 minutes oxidative stress. Survival is expressed as the difference of log CFU/ml after stress and before stress. Numbers indicate fermentations as presented in Table 1. P-values above the plots indicate significance of correlation (assessed by a linear model). (ZIP) [file pone.0167944.s008.zip › S3_File/LLKF_0013_real_dat.png]

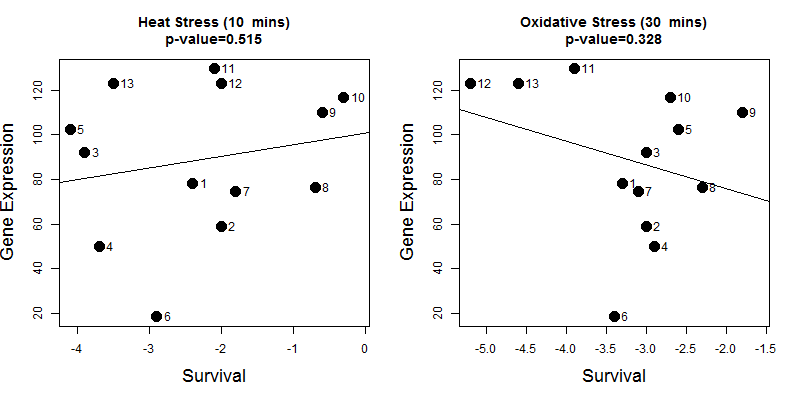

Supplement: S3 File — Expression levels of genes LLKF_0001 –LLKF_1273 plotted against survival after 10 minutes heat and 30 minutes oxidative stress. Survival is expressed as the difference of log CFU/ml after stress and before stress. Numbers indicate fermentations as presented in Table 1. P-values above the plots indicate significance of correlation (assessed by a linear model). (ZIP) [file pone.0167944.s008.zip › S3_File/LLKF_0014_real_dat.png]

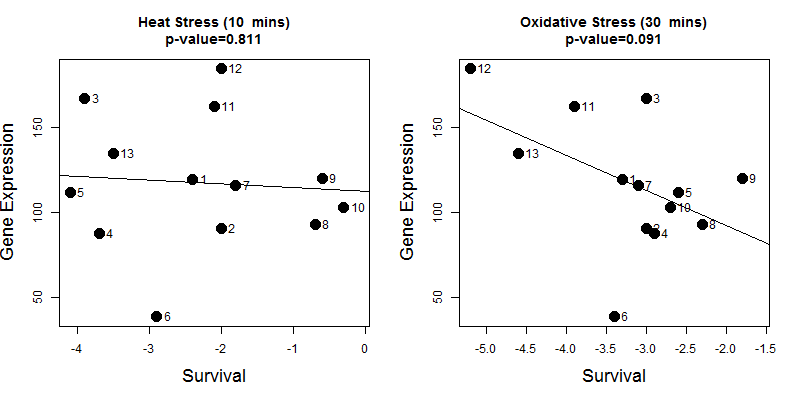

Supplement: S3 File — Expression levels of genes LLKF_0001 –LLKF_1273 plotted against survival after 10 minutes heat and 30 minutes oxidative stress. Survival is expressed as the difference of log CFU/ml after stress and before stress. Numbers indicate fermentations as presented in Table 1. P-values above the plots indicate significance of correlation (assessed by a linear model). (ZIP) [file pone.0167944.s008.zip › S3_File/LLKF_0015_real_dat.png]

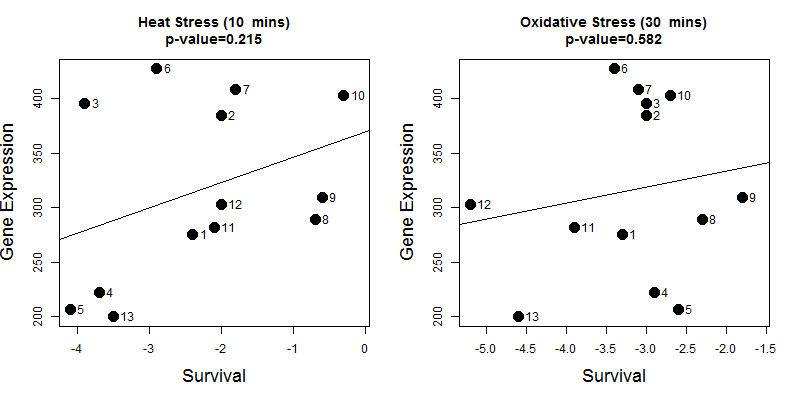

Supplement: S3 File — Expression levels of genes LLKF_0001 –LLKF_1273 plotted against survival after 10 minutes heat and 30 minutes oxidative stress. Survival is expressed as the difference of log CFU/ml after stress and before stress. Numbers indicate fermentations as presented in Table 1. P-values above the plots indicate significance of correlation (assessed by a linear model). (ZIP) [file pone.0167944.s008.zip › S3_File/LLKF_0016_real_dat.png]

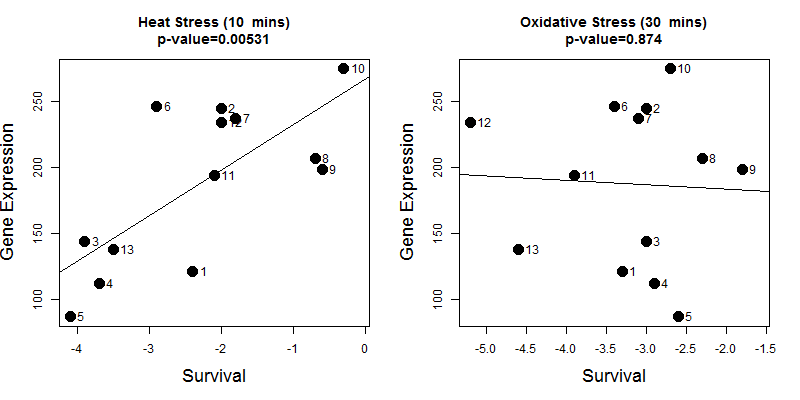

Supplement: S3 File — Expression levels of genes LLKF_0001 –LLKF_1273 plotted against survival after 10 minutes heat and 30 minutes oxidative stress. Survival is expressed as the difference of log CFU/ml after stress and before stress. Numbers indicate fermentations as presented in Table 1. P-values above the plots indicate significance of correlation (assessed by a linear model). (ZIP) [file pone.0167944.s008.zip › S3_File/LLKF_0017_real_dat.png]

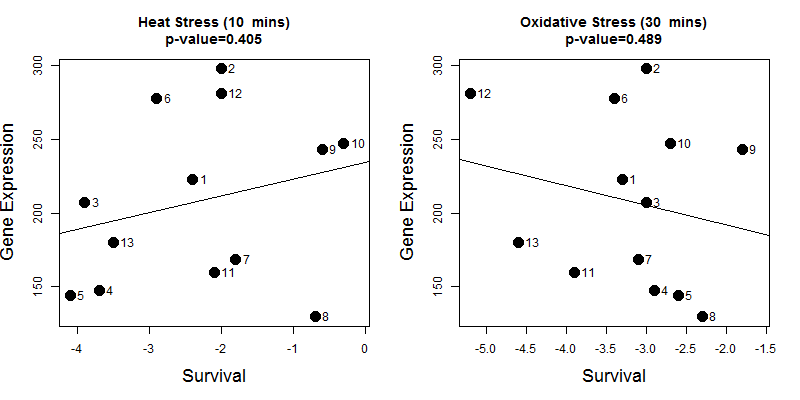

Supplement: S3 File — Expression levels of genes LLKF_0001 –LLKF_1273 plotted against survival after 10 minutes heat and 30 minutes oxidative stress. Survival is expressed as the difference of log CFU/ml after stress and before stress. Numbers indicate fermentations as presented in Table 1. P-values above the plots indicate significance of correlation (assessed by a linear model). (ZIP) [file pone.0167944.s008.zip › S3_File/LLKF_0018_real_dat.png]

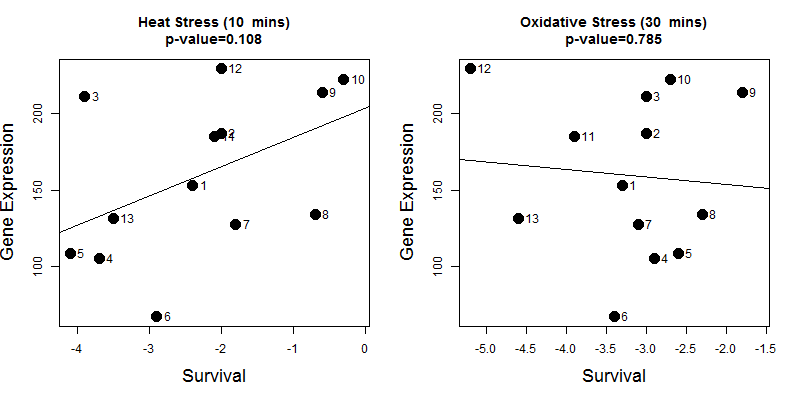

Supplement: S3 File — Expression levels of genes LLKF_0001 –LLKF_1273 plotted against survival after 10 minutes heat and 30 minutes oxidative stress. Survival is expressed as the difference of log CFU/ml after stress and before stress. Numbers indicate fermentations as presented in Table 1. P-values above the plots indicate significance of correlation (assessed by a linear model). (ZIP) [file pone.0167944.s008.zip › S3_File/LLKF_0019_real_dat.png]

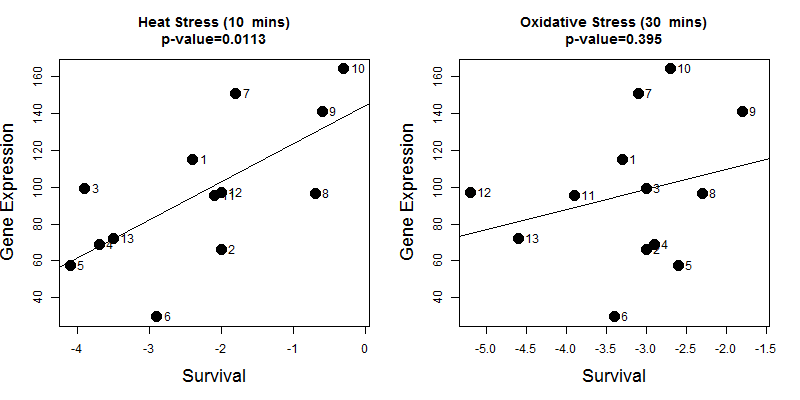

Supplement: S3 File — Expression levels of genes LLKF_0001 –LLKF_1273 plotted against survival after 10 minutes heat and 30 minutes oxidative stress. Survival is expressed as the difference of log CFU/ml after stress and before stress. Numbers indicate fermentations as presented in Table 1. P-values above the plots indicate significance of correlation (assessed by a linear model). (ZIP) [file pone.0167944.s008.zip › S3_File/LLKF_0020_real_dat.png]

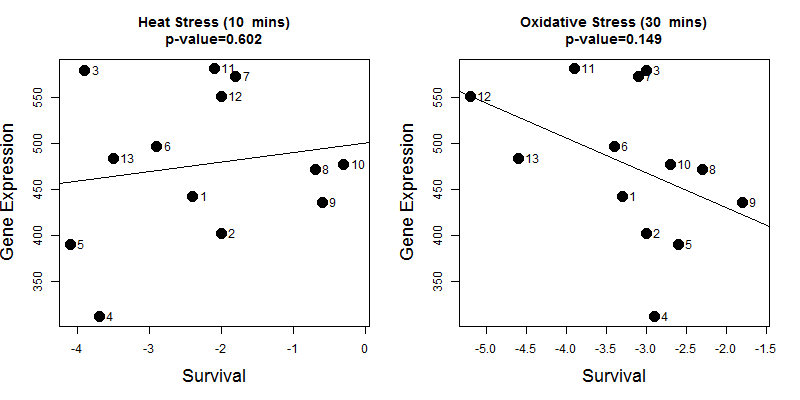

Supplement: S3 File — Expression levels of genes LLKF_0001 –LLKF_1273 plotted against survival after 10 minutes heat and 30 minutes oxidative stress. Survival is expressed as the difference of log CFU/ml after stress and before stress. Numbers indicate fermentations as presented in Table 1. P-values above the plots indicate significance of correlation (assessed by a linear model). (ZIP) [file pone.0167944.s008.zip › S3_File/LLKF_0021_real_dat.png]

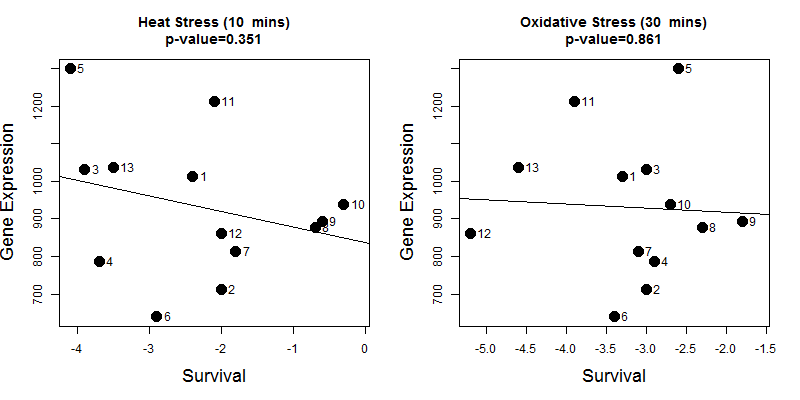

Supplement: S3 File — Expression levels of genes LLKF_0001 –LLKF_1273 plotted against survival after 10 minutes heat and 30 minutes oxidative stress. Survival is expressed as the difference of log CFU/ml after stress and before stress. Numbers indicate fermentations as presented in Table 1. P-values above the plots indicate significance of correlation (assessed by a linear model). (ZIP) [file pone.0167944.s008.zip › S3_File/LLKF_0022_real_dat.png]

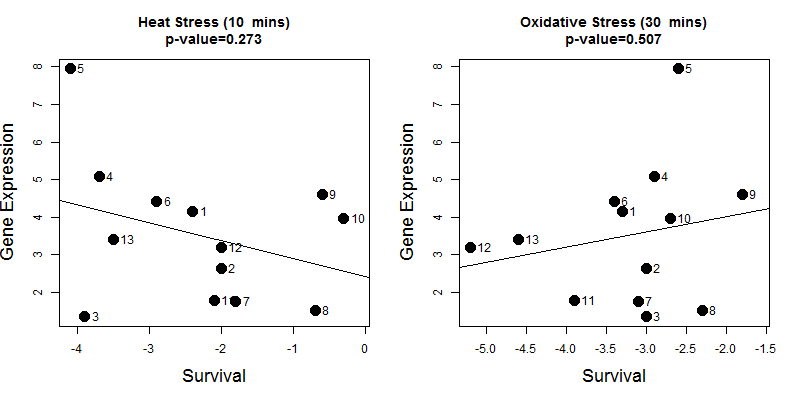

Supplement: S3 File — Expression levels of genes LLKF_0001 –LLKF_1273 plotted against survival after 10 minutes heat and 30 minutes oxidative stress. Survival is expressed as the difference of log CFU/ml after stress and before stress. Numbers indicate fermentations as presented in Table 1. P-values above the plots indicate significance of correlation (assessed by a linear model). (ZIP) [file pone.0167944.s008.zip › S3_File/LLKF_0023_real_dat.png]

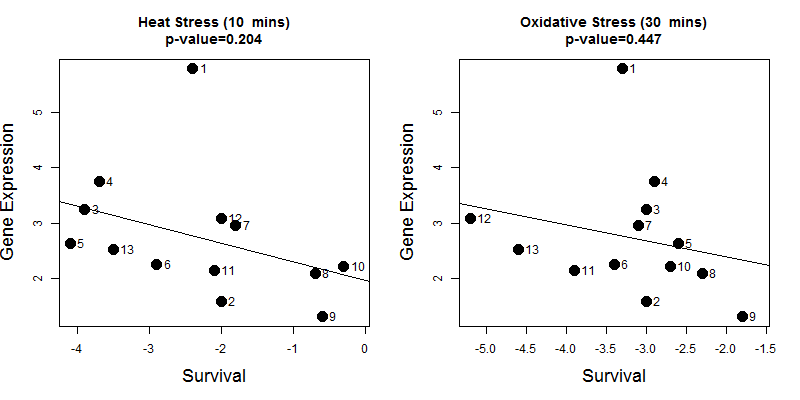

Supplement: S3 File — Expression levels of genes LLKF_0001 –LLKF_1273 plotted against survival after 10 minutes heat and 30 minutes oxidative stress. Survival is expressed as the difference of log CFU/ml after stress and before stress. Numbers indicate fermentations as presented in Table 1. P-values above the plots indicate significance of correlation (assessed by a linear model). (ZIP) [file pone.0167944.s008.zip › S3_File/LLKF_0024_real_dat.png]

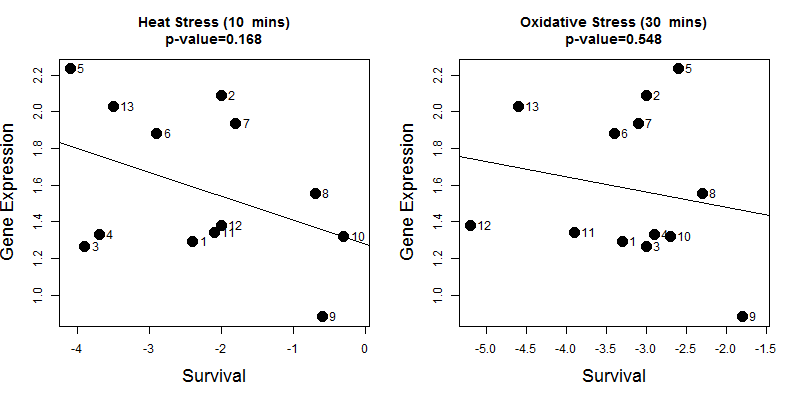

Supplement: S3 File — Expression levels of genes LLKF_0001 –LLKF_1273 plotted against survival after 10 minutes heat and 30 minutes oxidative stress. Survival is expressed as the difference of log CFU/ml after stress and before stress. Numbers indicate fermentations as presented in Table 1. P-values above the plots indicate significance of correlation (assessed by a linear model). (ZIP) [file pone.0167944.s008.zip › S3_File/LLKF_0025_real_dat.png]

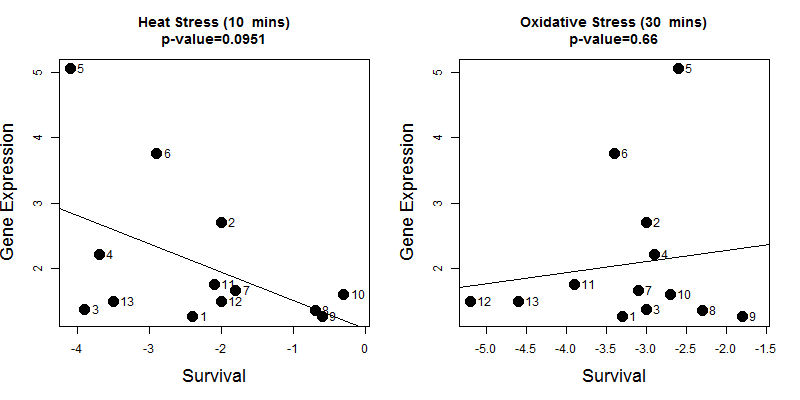

Supplement: S3 File — Expression levels of genes LLKF_0001 –LLKF_1273 plotted against survival after 10 minutes heat and 30 minutes oxidative stress. Survival is expressed as the difference of log CFU/ml after stress and before stress. Numbers indicate fermentations as presented in Table 1. P-values above the plots indicate significance of correlation (assessed by a linear model). (ZIP) [file pone.0167944.s008.zip › S3_File/LLKF_0026_real_dat.png]

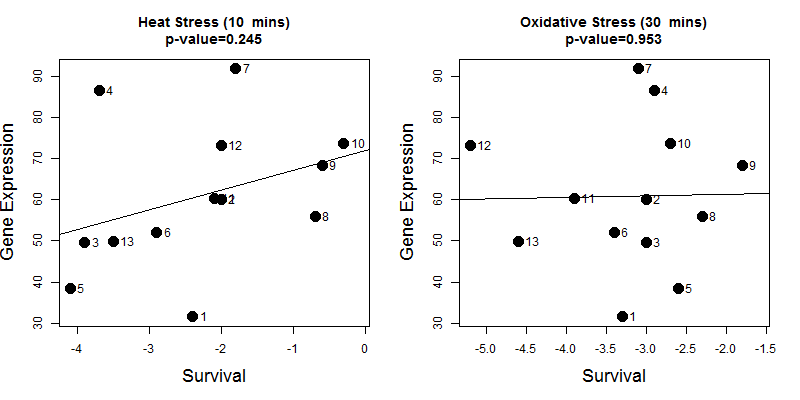

Supplement: S3 File — Expression levels of genes LLKF_0001 –LLKF_1273 plotted against survival after 10 minutes heat and 30 minutes oxidative stress. Survival is expressed as the difference of log CFU/ml after stress and before stress. Numbers indicate fermentations as presented in Table 1. P-values above the plots indicate significance of correlation (assessed by a linear model). (ZIP) [file pone.0167944.s008.zip › S3_File/LLKF_0027_real_dat.png]

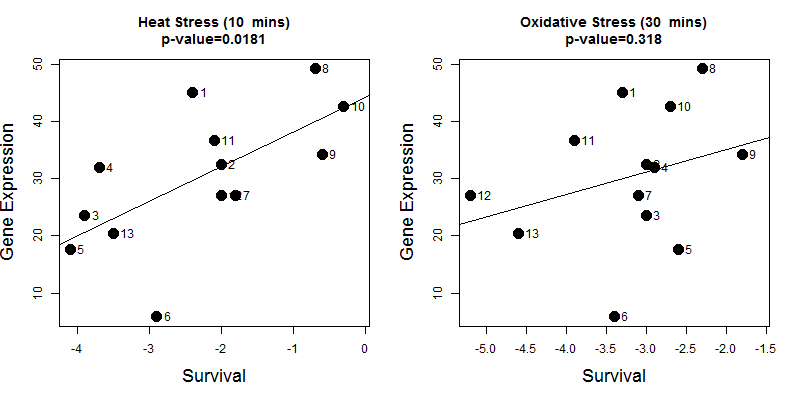

Supplement: S3 File — Expression levels of genes LLKF_0001 –LLKF_1273 plotted against survival after 10 minutes heat and 30 minutes oxidative stress. Survival is expressed as the difference of log CFU/ml after stress and before stress. Numbers indicate fermentations as presented in Table 1. P-values above the plots indicate significance of correlation (assessed by a linear model). (ZIP) [file pone.0167944.s008.zip › S3_File/LLKF_0028_real_dat.png]

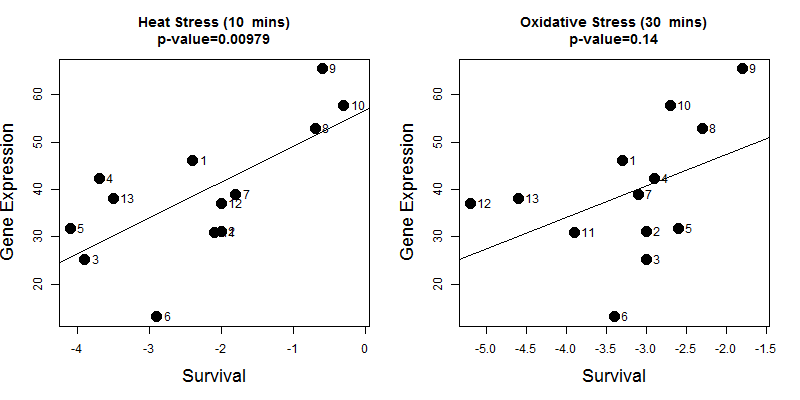

Supplement: S3 File — Expression levels of genes LLKF_0001 –LLKF_1273 plotted against survival after 10 minutes heat and 30 minutes oxidative stress. Survival is expressed as the difference of log CFU/ml after stress and before stress. Numbers indicate fermentations as presented in Table 1. P-values above the plots indicate significance of correlation (assessed by a linear model). (ZIP) [file pone.0167944.s008.zip › S3_File/LLKF_0029_real_dat.png]

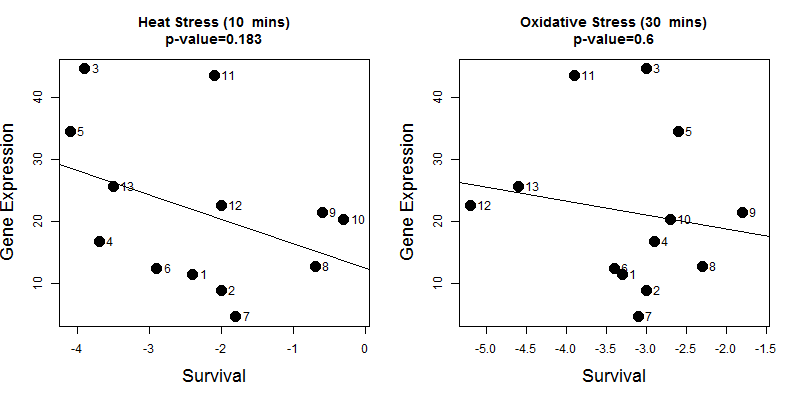

Supplement: S3 File — Expression levels of genes LLKF_0001 –LLKF_1273 plotted against survival after 10 minutes heat and 30 minutes oxidative stress. Survival is expressed as the difference of log CFU/ml after stress and before stress. Numbers indicate fermentations as presented in Table 1. P-values above the plots indicate significance of correlation (assessed by a linear model). (ZIP) [file pone.0167944.s008.zip › S3_File/LLKF_0030_real_dat.png]

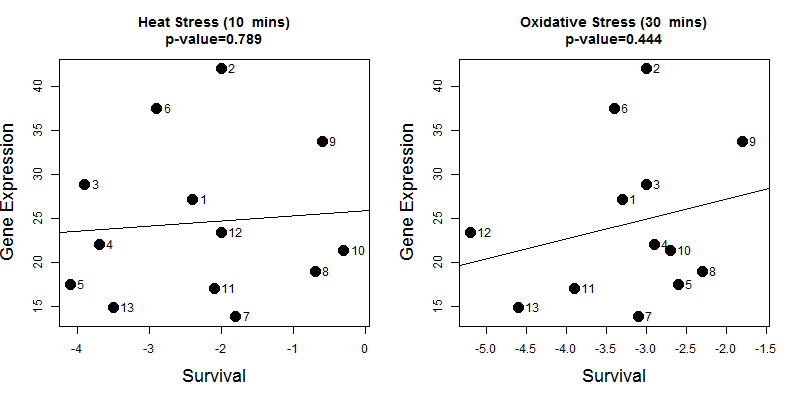

Supplement: S3 File — Expression levels of genes LLKF_0001 –LLKF_1273 plotted against survival after 10 minutes heat and 30 minutes oxidative stress. Survival is expressed as the difference of log CFU/ml after stress and before stress. Numbers indicate fermentations as presented in Table 1. P-values above the plots indicate significance of correlation (assessed by a linear model). (ZIP) [file pone.0167944.s008.zip › S3_File/LLKF_0031_real_dat.png]

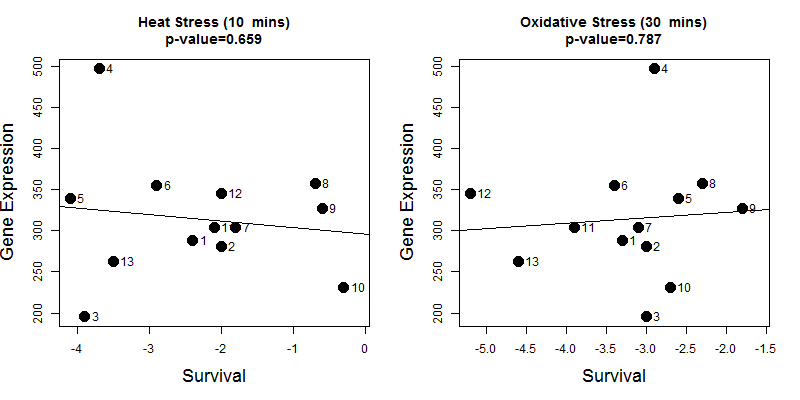

Supplement: S3 File — Expression levels of genes LLKF_0001 –LLKF_1273 plotted against survival after 10 minutes heat and 30 minutes oxidative stress. Survival is expressed as the difference of log CFU/ml after stress and before stress. Numbers indicate fermentations as presented in Table 1. P-values above the plots indicate significance of correlation (assessed by a linear model). (ZIP) [file pone.0167944.s008.zip › S3_File/LLKF_0032_real_dat.png]

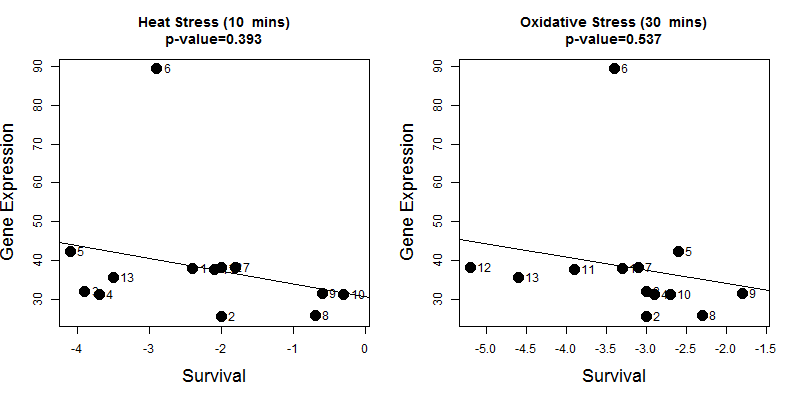

Supplement: S3 File — Expression levels of genes LLKF_0001 –LLKF_1273 plotted against survival after 10 minutes heat and 30 minutes oxidative stress. Survival is expressed as the difference of log CFU/ml after stress and before stress. Numbers indicate fermentations as presented in Table 1. P-values above the plots indicate significance of correlation (assessed by a linear model). (ZIP) [file pone.0167944.s008.zip › S3_File/LLKF_0033_real_dat.png]

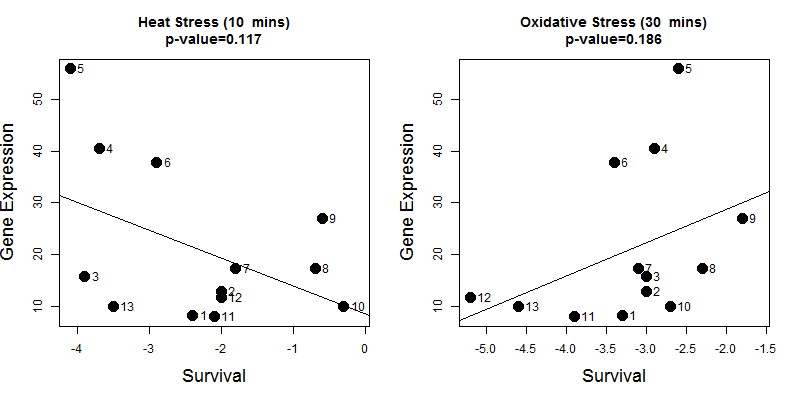

Supplement: S3 File — Expression levels of genes LLKF_0001 –LLKF_1273 plotted against survival after 10 minutes heat and 30 minutes oxidative stress. Survival is expressed as the difference of log CFU/ml after stress and before stress. Numbers indicate fermentations as presented in Table 1. P-values above the plots indicate significance of correlation (assessed by a linear model). (ZIP) [file pone.0167944.s008.zip › S3_File/LLKF_0034_real_dat.png]

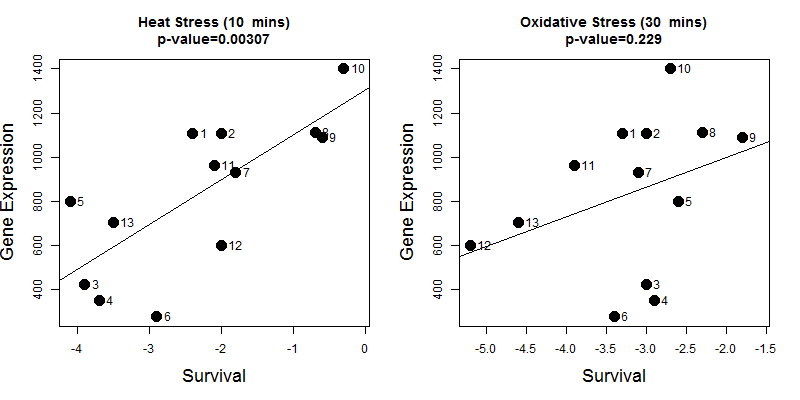

Supplement: S3 File — Expression levels of genes LLKF_0001 –LLKF_1273 plotted against survival after 10 minutes heat and 30 minutes oxidative stress. Survival is expressed as the difference of log CFU/ml after stress and before stress. Numbers indicate fermentations as presented in Table 1. P-values above the plots indicate significance of correlation (assessed by a linear model). (ZIP) [file pone.0167944.s008.zip › S3_File/LLKF_0035_real_dat.png]

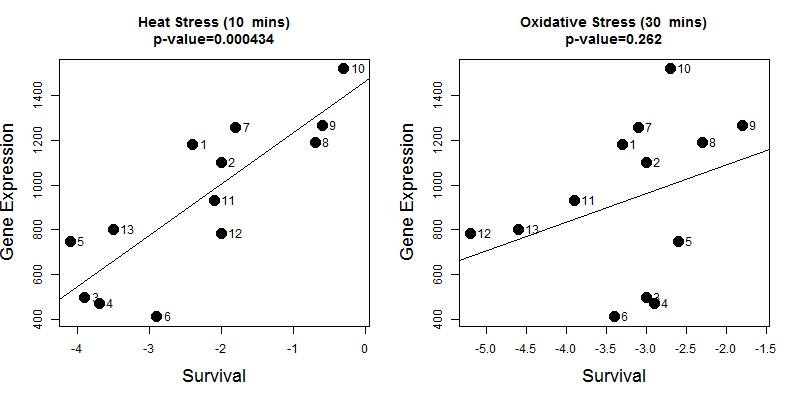

Supplement: S3 File — Expression levels of genes LLKF_0001 –LLKF_1273 plotted against survival after 10 minutes heat and 30 minutes oxidative stress. Survival is expressed as the difference of log CFU/ml after stress and before stress. Numbers indicate fermentations as presented in Table 1. P-values above the plots indicate significance of correlation (assessed by a linear model). (ZIP) [file pone.0167944.s008.zip › S3_File/LLKF_0036_real_dat.png]

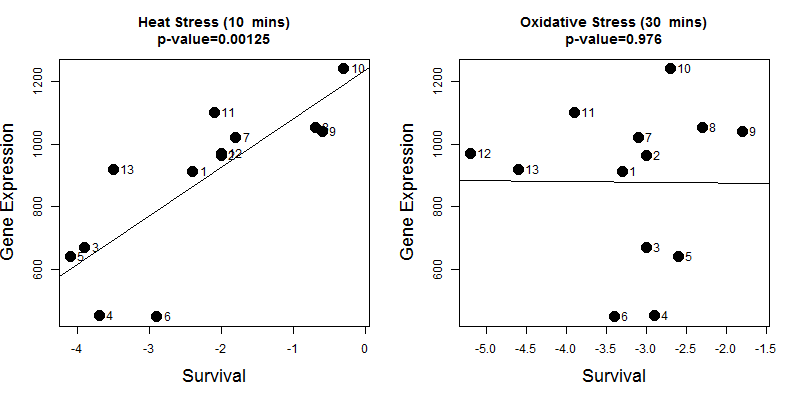

Supplement: S3 File — Expression levels of genes LLKF_0001 –LLKF_1273 plotted against survival after 10 minutes heat and 30 minutes oxidative stress. Survival is expressed as the difference of log CFU/ml after stress and before stress. Numbers indicate fermentations as presented in Table 1. P-values above the plots indicate significance of correlation (assessed by a linear model). (ZIP) [file pone.0167944.s008.zip › S3_File/LLKF_0037_real_dat.png]

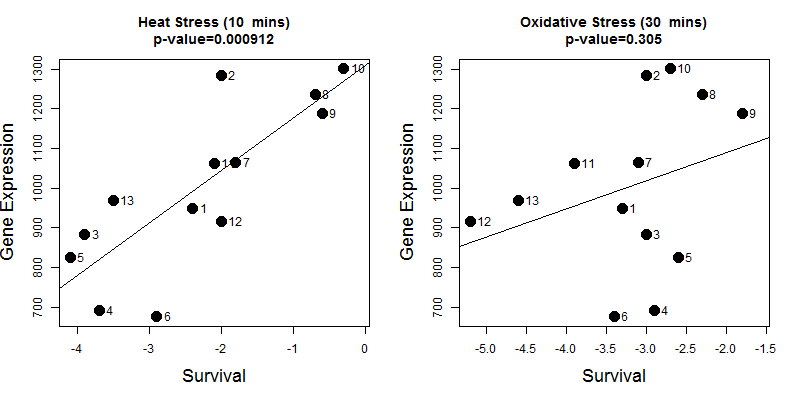

Supplement: S3 File — Expression levels of genes LLKF_0001 –LLKF_1273 plotted against survival after 10 minutes heat and 30 minutes oxidative stress. Survival is expressed as the difference of log CFU/ml after stress and before stress. Numbers indicate fermentations as presented in Table 1. P-values above the plots indicate significance of correlation (assessed by a linear model). (ZIP) [file pone.0167944.s008.zip › S3_File/LLKF_0038_real_dat.png]

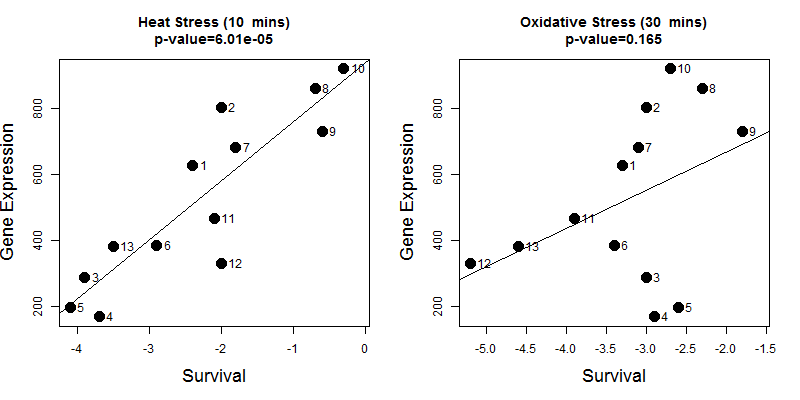

Supplement: S3 File — Expression levels of genes LLKF_0001 –LLKF_1273 plotted against survival after 10 minutes heat and 30 minutes oxidative stress. Survival is expressed as the difference of log CFU/ml after stress and before stress. Numbers indicate fermentations as presented in Table 1. P-values above the plots indicate significance of correlation (assessed by a linear model). (ZIP) [file pone.0167944.s008.zip › S3_File/LLKF_0039_real_dat.png]

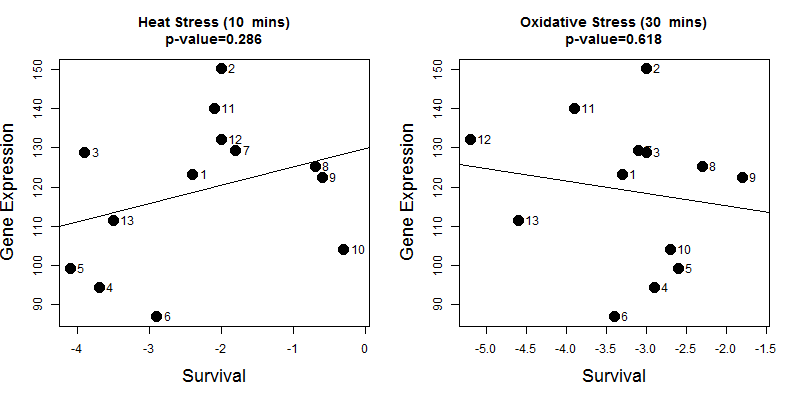

Supplement: S3 File — Expression levels of genes LLKF_0001 –LLKF_1273 plotted against survival after 10 minutes heat and 30 minutes oxidative stress. Survival is expressed as the difference of log CFU/ml after stress and before stress. Numbers indicate fermentations as presented in Table 1. P-values above the plots indicate significance of correlation (assessed by a linear model). (ZIP) [file pone.0167944.s008.zip › S3_File/LLKF_0040_real_dat.png]

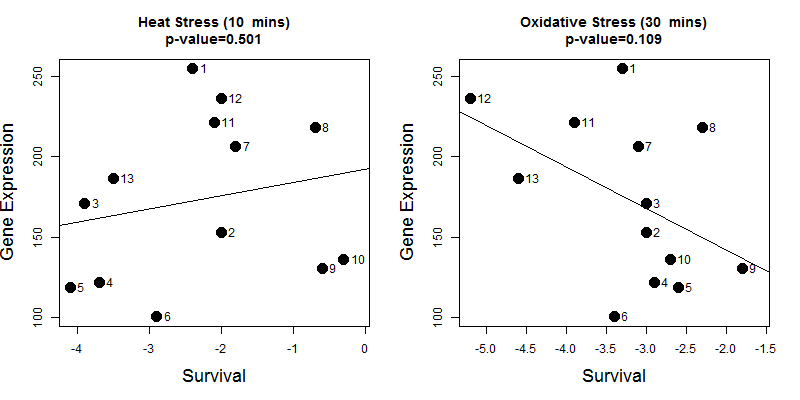

Supplement: S3 File — Expression levels of genes LLKF_0001 –LLKF_1273 plotted against survival after 10 minutes heat and 30 minutes oxidative stress. Survival is expressed as the difference of log CFU/ml after stress and before stress. Numbers indicate fermentations as presented in Table 1. P-values above the plots indicate significance of correlation (assessed by a linear model). (ZIP) [file pone.0167944.s008.zip › S3_File/LLKF_0041_real_dat.png]

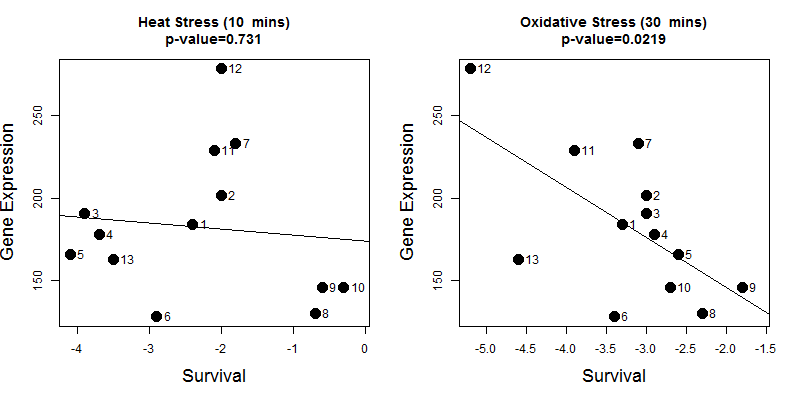

Supplement: S3 File — Expression levels of genes LLKF_0001 –LLKF_1273 plotted against survival after 10 minutes heat and 30 minutes oxidative stress. Survival is expressed as the difference of log CFU/ml after stress and before stress. Numbers indicate fermentations as presented in Table 1. P-values above the plots indicate significance of correlation (assessed by a linear model). (ZIP) [file pone.0167944.s008.zip › S3_File/LLKF_0042_real_dat.png]

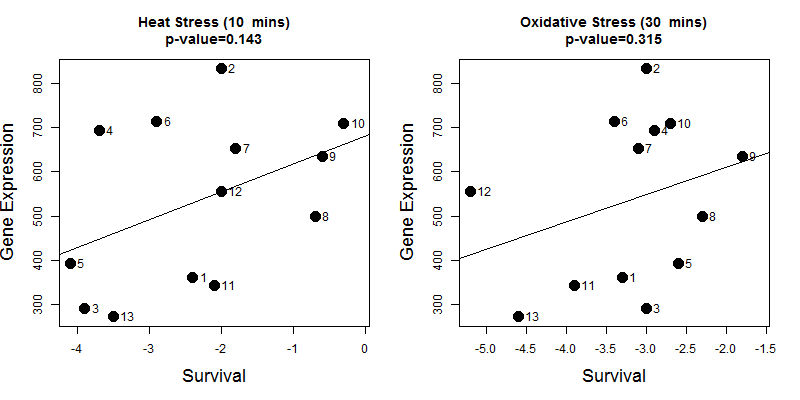

Supplement: S3 File — Expression levels of genes LLKF_0001 –LLKF_1273 plotted against survival after 10 minutes heat and 30 minutes oxidative stress. Survival is expressed as the difference of log CFU/ml after stress and before stress. Numbers indicate fermentations as presented in Table 1. P-values above the plots indicate significance of correlation (assessed by a linear model). (ZIP) [file pone.0167944.s008.zip › S3_File/LLKF_0043_real_dat.png]

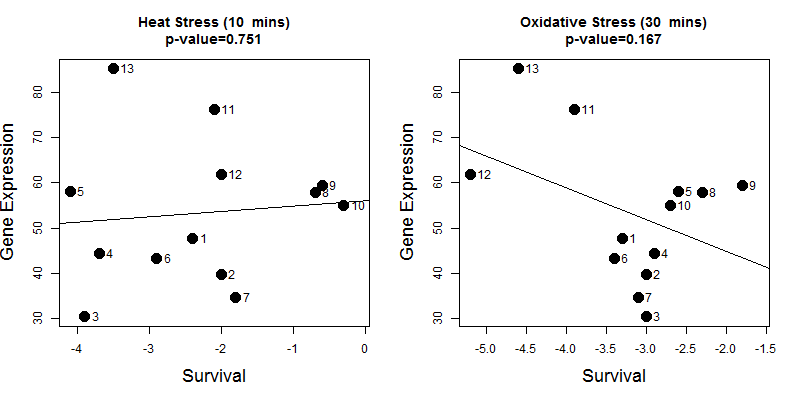

Supplement: S3 File — Expression levels of genes LLKF_0001 –LLKF_1273 plotted against survival after 10 minutes heat and 30 minutes oxidative stress. Survival is expressed as the difference of log CFU/ml after stress and before stress. Numbers indicate fermentations as presented in Table 1. P-values above the plots indicate significance of correlation (assessed by a linear model). (ZIP) [file pone.0167944.s008.zip › S3_File/LLKF_0044_real_dat.png]

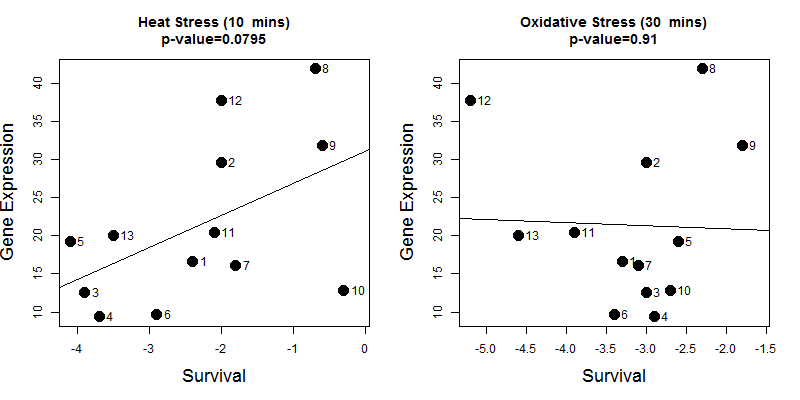

Supplement: S3 File — Expression levels of genes LLKF_0001 –LLKF_1273 plotted against survival after 10 minutes heat and 30 minutes oxidative stress. Survival is expressed as the difference of log CFU/ml after stress and before stress. Numbers indicate fermentations as presented in Table 1. P-values above the plots indicate significance of correlation (assessed by a linear model). (ZIP) [file pone.0167944.s008.zip › S3_File/LLKF_0045_real_dat.png]

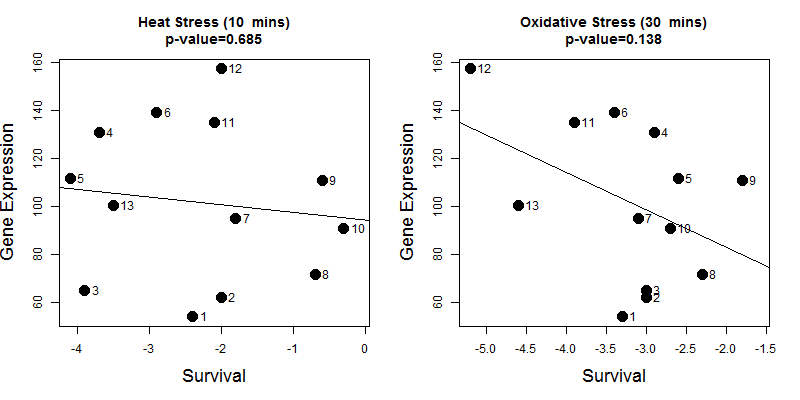

Supplement: S3 File — Expression levels of genes LLKF_0001 –LLKF_1273 plotted against survival after 10 minutes heat and 30 minutes oxidative stress. Survival is expressed as the difference of log CFU/ml after stress and before stress. Numbers indicate fermentations as presented in Table 1. P-values above the plots indicate significance of correlation (assessed by a linear model). (ZIP) [file pone.0167944.s008.zip › S3_File/LLKF_0046_real_dat.png]

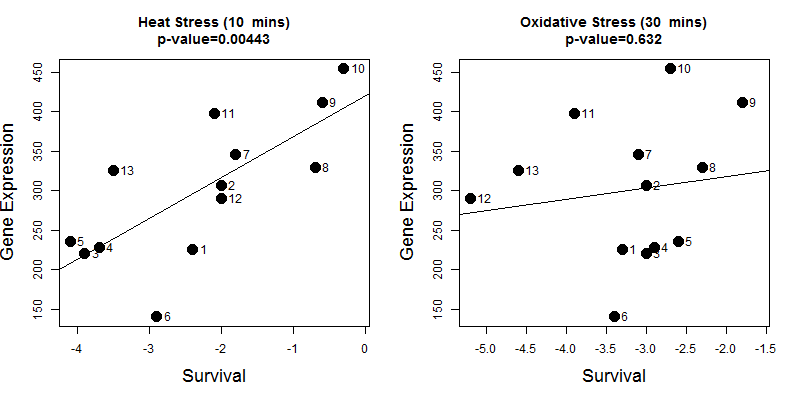

Supplement: S3 File — Expression levels of genes LLKF_0001 –LLKF_1273 plotted against survival after 10 minutes heat and 30 minutes oxidative stress. Survival is expressed as the difference of log CFU/ml after stress and before stress. Numbers indicate fermentations as presented in Table 1. P-values above the plots indicate significance of correlation (assessed by a linear model). (ZIP) [file pone.0167944.s008.zip › S3_File/LLKF_0047_real_dat.png]

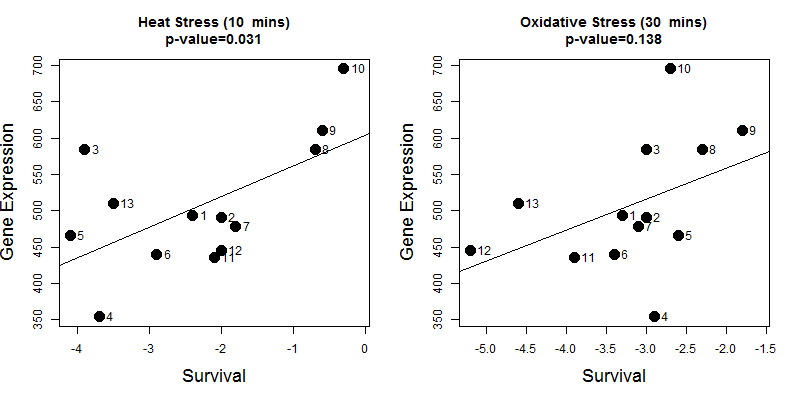

Supplement: S3 File — Expression levels of genes LLKF_0001 –LLKF_1273 plotted against survival after 10 minutes heat and 30 minutes oxidative stress. Survival is expressed as the difference of log CFU/ml after stress and before stress. Numbers indicate fermentations as presented in Table 1. P-values above the plots indicate significance of correlation (assessed by a linear model). (ZIP) [file pone.0167944.s008.zip › S3_File/LLKF_0048_real_dat.png]

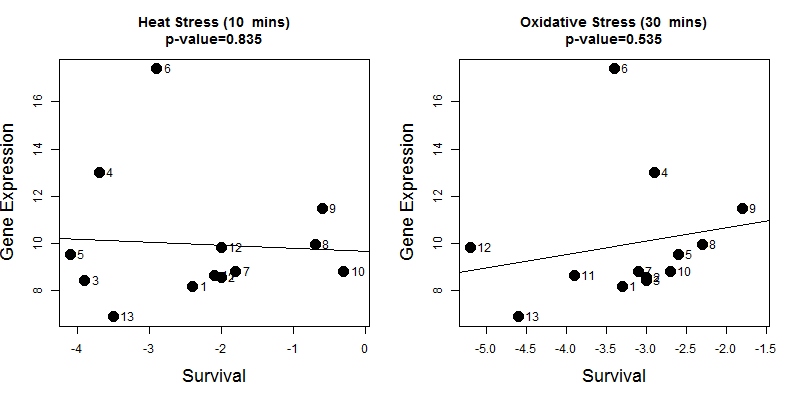

Supplement: S3 File — Expression levels of genes LLKF_0001 –LLKF_1273 plotted against survival after 10 minutes heat and 30 minutes oxidative stress. Survival is expressed as the difference of log CFU/ml after stress and before stress. Numbers indicate fermentations as presented in Table 1. P-values above the plots indicate significance of correlation (assessed by a linear model). (ZIP) [file pone.0167944.s008.zip › S3_File/LLKF_0049_real_dat.png]

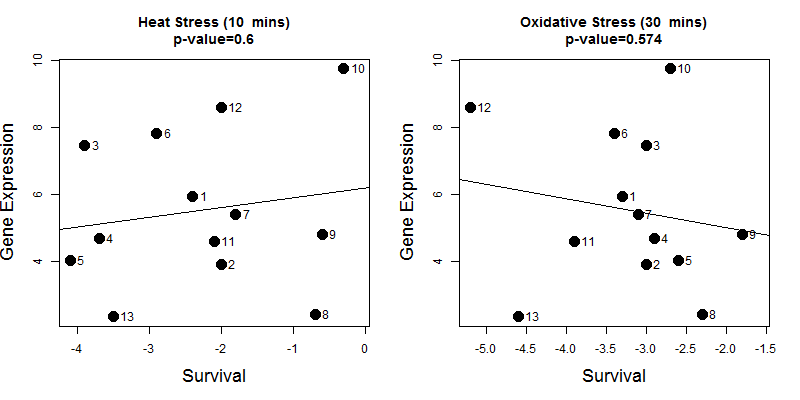

Supplement: S3 File — Expression levels of genes LLKF_0001 –LLKF_1273 plotted against survival after 10 minutes heat and 30 minutes oxidative stress. Survival is expressed as the difference of log CFU/ml after stress and before stress. Numbers indicate fermentations as presented in Table 1. P-values above the plots indicate significance of correlation (assessed by a linear model). (ZIP) [file pone.0167944.s008.zip › S3_File/LLKF_0050_real_dat.png]

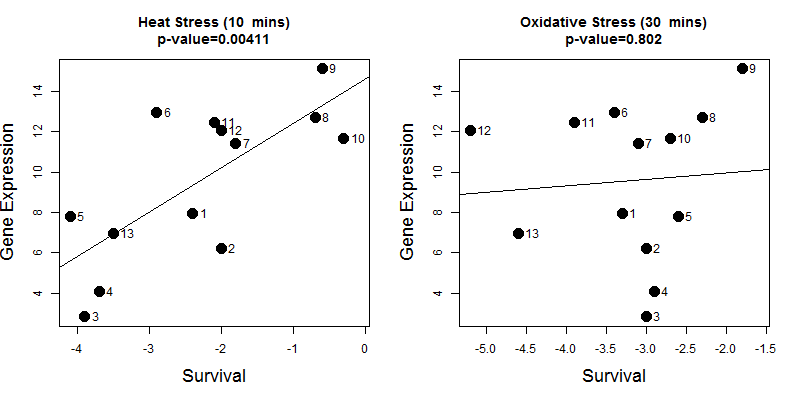

Supplement: S3 File — Expression levels of genes LLKF_0001 –LLKF_1273 plotted against survival after 10 minutes heat and 30 minutes oxidative stress. Survival is expressed as the difference of log CFU/ml after stress and before stress. Numbers indicate fermentations as presented in Table 1. P-values above the plots indicate significance of correlation (assessed by a linear model). (ZIP) [file pone.0167944.s008.zip › S3_File/LLKF_0051_real_dat.png]

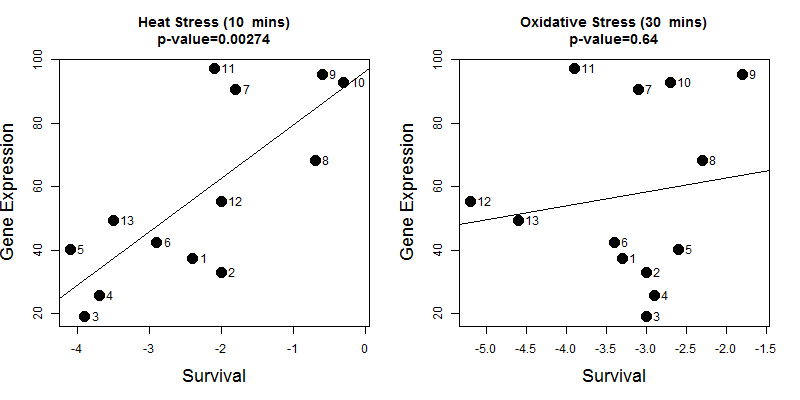

Supplement: S3 File — Expression levels of genes LLKF_0001 –LLKF_1273 plotted against survival after 10 minutes heat and 30 minutes oxidative stress. Survival is expressed as the difference of log CFU/ml after stress and before stress. Numbers indicate fermentations as presented in Table 1. P-values above the plots indicate significance of correlation (assessed by a linear model). (ZIP) [file pone.0167944.s008.zip › S3_File/LLKF_0052_real_dat.png]

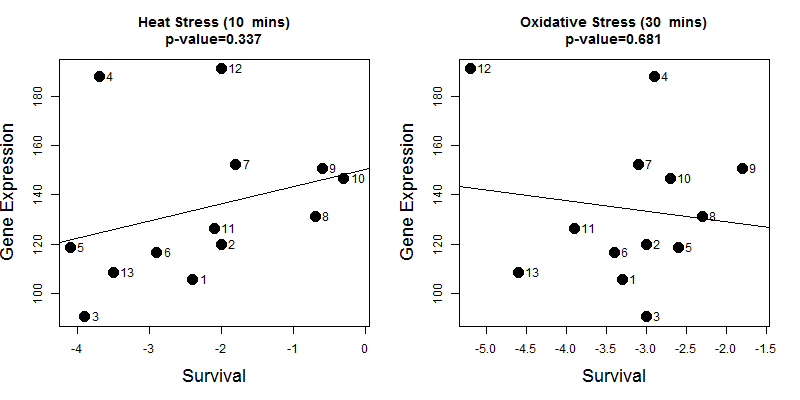

Supplement: S3 File — Expression levels of genes LLKF_0001 –LLKF_1273 plotted against survival after 10 minutes heat and 30 minutes oxidative stress. Survival is expressed as the difference of log CFU/ml after stress and before stress. Numbers indicate fermentations as presented in Table 1. P-values above the plots indicate significance of correlation (assessed by a linear model). (ZIP) [file pone.0167944.s008.zip › S3_File/LLKF_0053_real_dat.png]

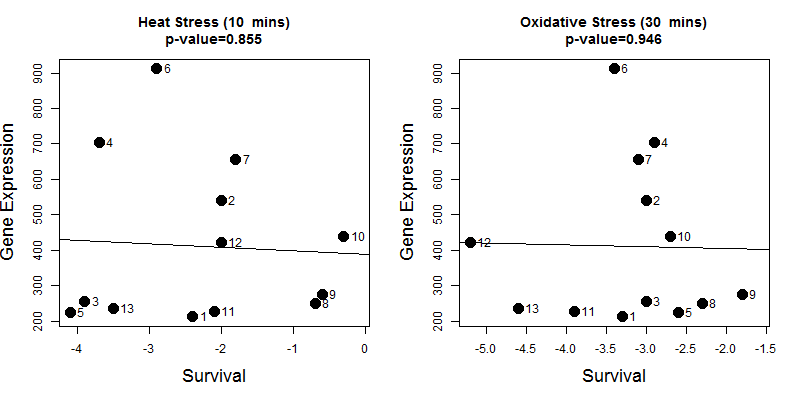

Supplement: S3 File — Expression levels of genes LLKF_0001 –LLKF_1273 plotted against survival after 10 minutes heat and 30 minutes oxidative stress. Survival is expressed as the difference of log CFU/ml after stress and before stress. Numbers indicate fermentations as presented in Table 1. P-values above the plots indicate significance of correlation (assessed by a linear model). (ZIP) [file pone.0167944.s008.zip › S3_File/LLKF_0054_real_dat.png]

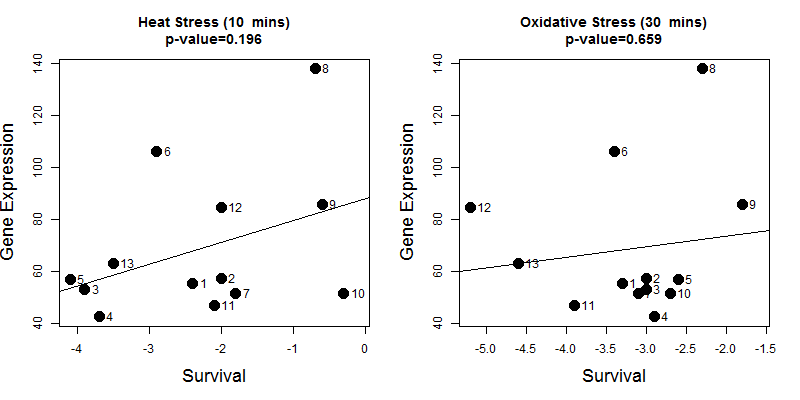

Supplement: S3 File — Expression levels of genes LLKF_0001 –LLKF_1273 plotted against survival after 10 minutes heat and 30 minutes oxidative stress. Survival is expressed as the difference of log CFU/ml after stress and before stress. Numbers indicate fermentations as presented in Table 1. P-values above the plots indicate significance of correlation (assessed by a linear model). (ZIP) [file pone.0167944.s008.zip › S3_File/LLKF_0055_real_dat.png]

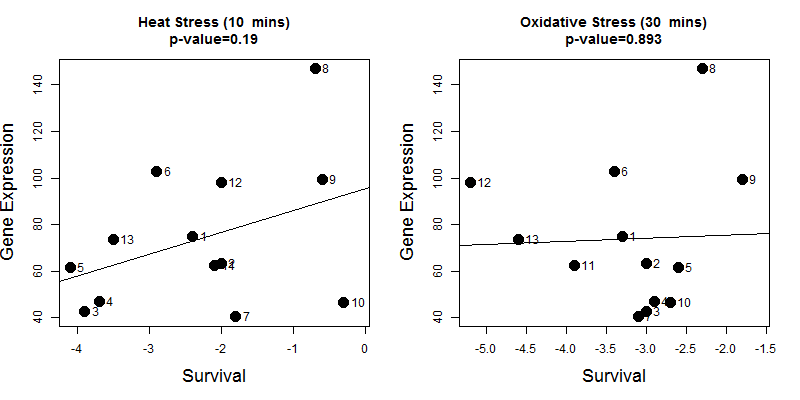

Supplement: S3 File — Expression levels of genes LLKF_0001 –LLKF_1273 plotted against survival after 10 minutes heat and 30 minutes oxidative stress. Survival is expressed as the difference of log CFU/ml after stress and before stress. Numbers indicate fermentations as presented in Table 1. P-values above the plots indicate significance of correlation (assessed by a linear model). (ZIP) [file pone.0167944.s008.zip › S3_File/LLKF_0056_real_dat.png]

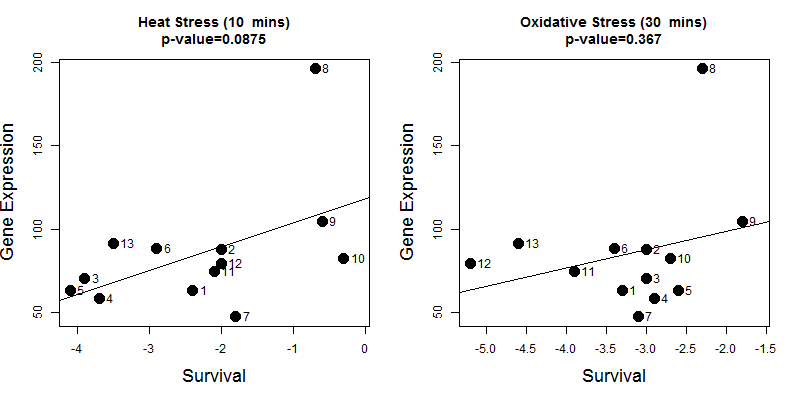

Supplement: S3 File — Expression levels of genes LLKF_0001 –LLKF_1273 plotted against survival after 10 minutes heat and 30 minutes oxidative stress. Survival is expressed as the difference of log CFU/ml after stress and before stress. Numbers indicate fermentations as presented in Table 1. P-values above the plots indicate significance of correlation (assessed by a linear model). (ZIP) [file pone.0167944.s008.zip › S3_File/LLKF_0057_real_dat.png]

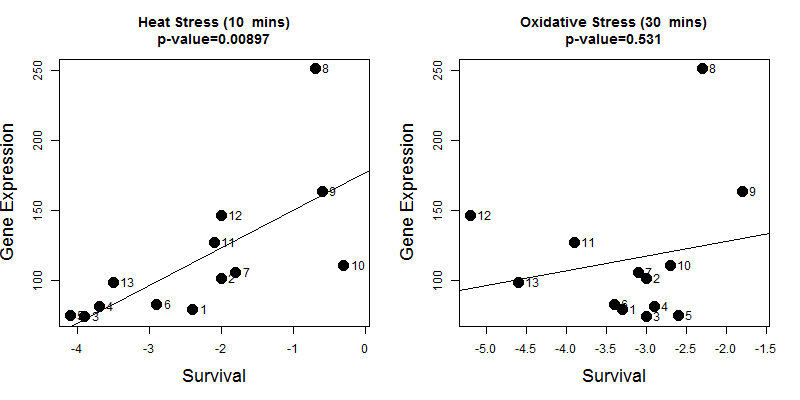

Supplement: S3 File — Expression levels of genes LLKF_0001 –LLKF_1273 plotted against survival after 10 minutes heat and 30 minutes oxidative stress. Survival is expressed as the difference of log CFU/ml after stress and before stress. Numbers indicate fermentations as presented in Table 1. P-values above the plots indicate significance of correlation (assessed by a linear model). (ZIP) [file pone.0167944.s008.zip › S3_File/LLKF_0058_real_dat.png]

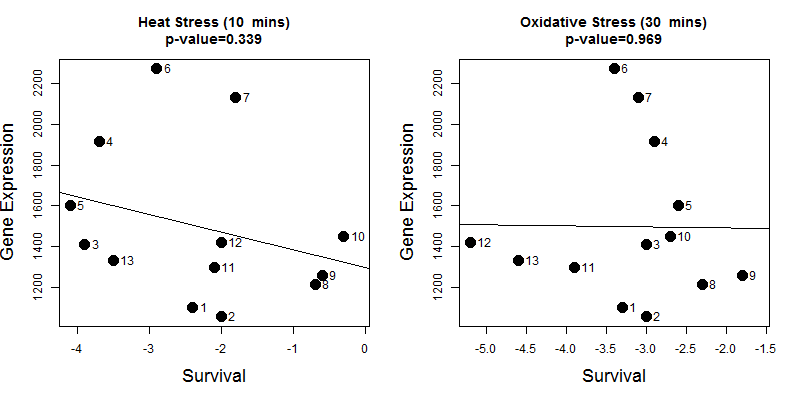

Supplement: S3 File — Expression levels of genes LLKF_0001 –LLKF_1273 plotted against survival after 10 minutes heat and 30 minutes oxidative stress. Survival is expressed as the difference of log CFU/ml after stress and before stress. Numbers indicate fermentations as presented in Table 1. P-values above the plots indicate significance of correlation (assessed by a linear model). (ZIP) [file pone.0167944.s008.zip › S3_File/LLKF_0059_real_dat.png]

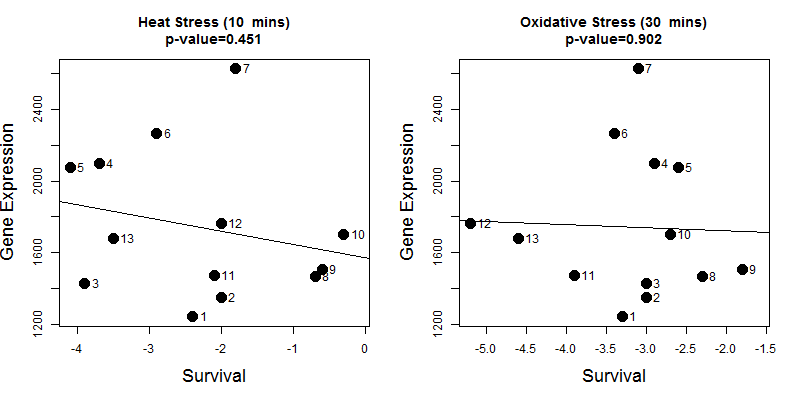

Supplement: S3 File — Expression levels of genes LLKF_0001 –LLKF_1273 plotted against survival after 10 minutes heat and 30 minutes oxidative stress. Survival is expressed as the difference of log CFU/ml after stress and before stress. Numbers indicate fermentations as presented in Table 1. P-values above the plots indicate significance of correlation (assessed by a linear model). (ZIP) [file pone.0167944.s008.zip › S3_File/LLKF_0060_real_dat.png]

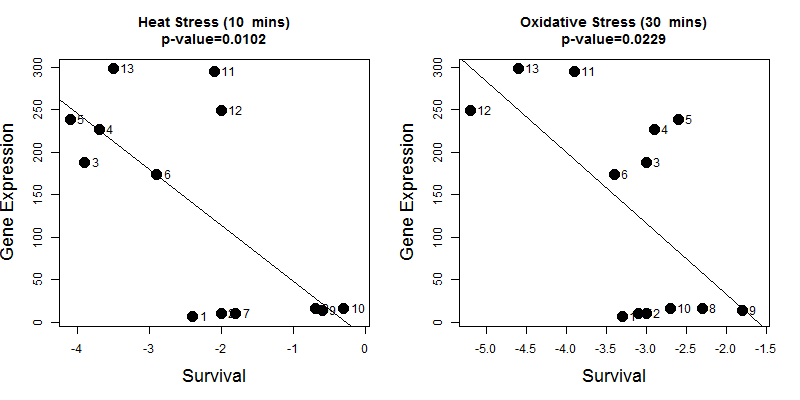

Supplement: S3 File — Expression levels of genes LLKF_0001 –LLKF_1273 plotted against survival after 10 minutes heat and 30 minutes oxidative stress. Survival is expressed as the difference of log CFU/ml after stress and before stress. Numbers indicate fermentations as presented in Table 1. P-values above the plots indicate significance of correlation (assessed by a linear model). (ZIP) [file pone.0167944.s008.zip › S3_File/LLKF_0061_real_dat.png]

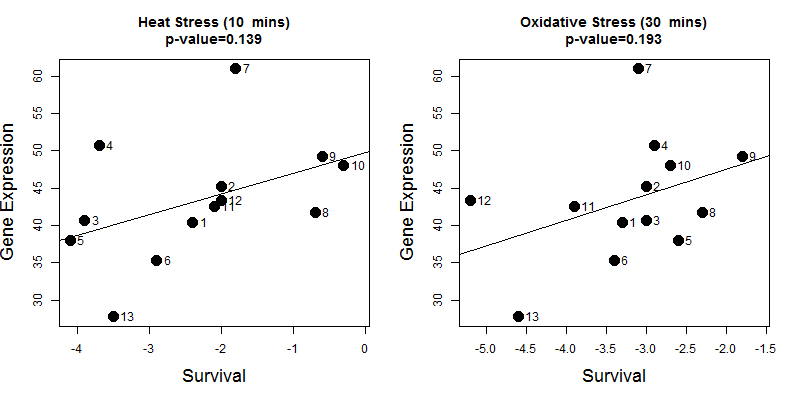

Supplement: S3 File — Expression levels of genes LLKF_0001 –LLKF_1273 plotted against survival after 10 minutes heat and 30 minutes oxidative stress. Survival is expressed as the difference of log CFU/ml after stress and before stress. Numbers indicate fermentations as presented in Table 1. P-values above the plots indicate significance of correlation (assessed by a linear model). (ZIP) [file pone.0167944.s008.zip › S3_File/LLKF_0062_real_dat.png]

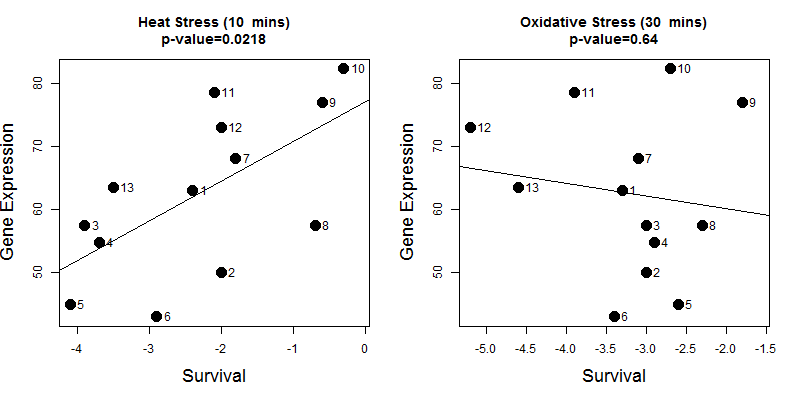

Supplement: S3 File — Expression levels of genes LLKF_0001 –LLKF_1273 plotted against survival after 10 minutes heat and 30 minutes oxidative stress. Survival is expressed as the difference of log CFU/ml after stress and before stress. Numbers indicate fermentations as presented in Table 1. P-values above the plots indicate significance of correlation (assessed by a linear model). (ZIP) [file pone.0167944.s008.zip › S3_File/LLKF_0063_real_dat.png]

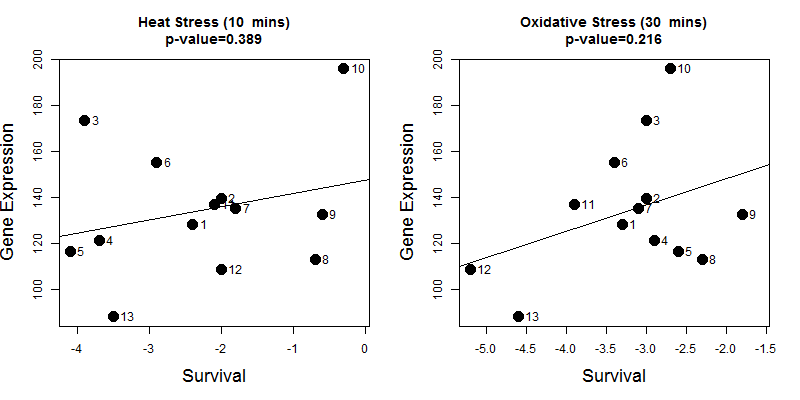

Supplement: S3 File — Expression levels of genes LLKF_0001 –LLKF_1273 plotted against survival after 10 minutes heat and 30 minutes oxidative stress. Survival is expressed as the difference of log CFU/ml after stress and before stress. Numbers indicate fermentations as presented in Table 1. P-values above the plots indicate significance of correlation (assessed by a linear model). (ZIP) [file pone.0167944.s008.zip › S3_File/LLKF_0064_real_dat.png]

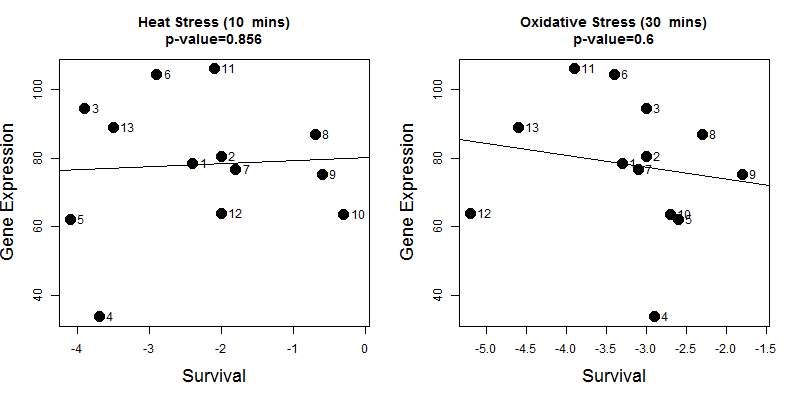

Supplement: S3 File — Expression levels of genes LLKF_0001 –LLKF_1273 plotted against survival after 10 minutes heat and 30 minutes oxidative stress. Survival is expressed as the difference of log CFU/ml after stress and before stress. Numbers indicate fermentations as presented in Table 1. P-values above the plots indicate significance of correlation (assessed by a linear model). (ZIP) [file pone.0167944.s008.zip › S3_File/LLKF_0065_real_dat.png]

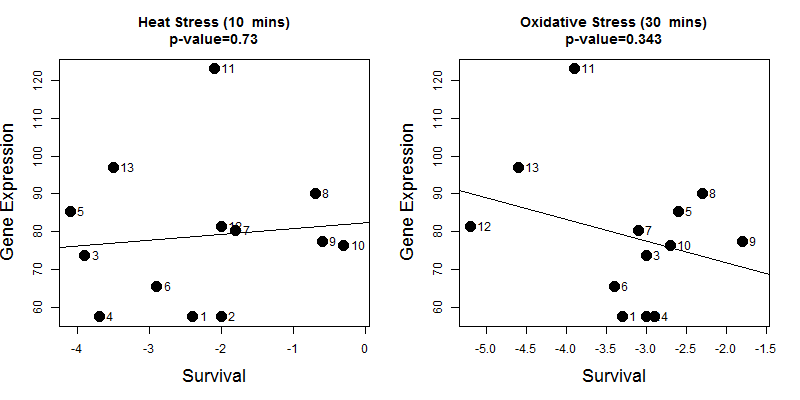

Supplement: S3 File — Expression levels of genes LLKF_0001 –LLKF_1273 plotted against survival after 10 minutes heat and 30 minutes oxidative stress. Survival is expressed as the difference of log CFU/ml after stress and before stress. Numbers indicate fermentations as presented in Table 1. P-values above the plots indicate significance of correlation (assessed by a linear model). (ZIP) [file pone.0167944.s008.zip › S3_File/LLKF_0066_real_dat.png]

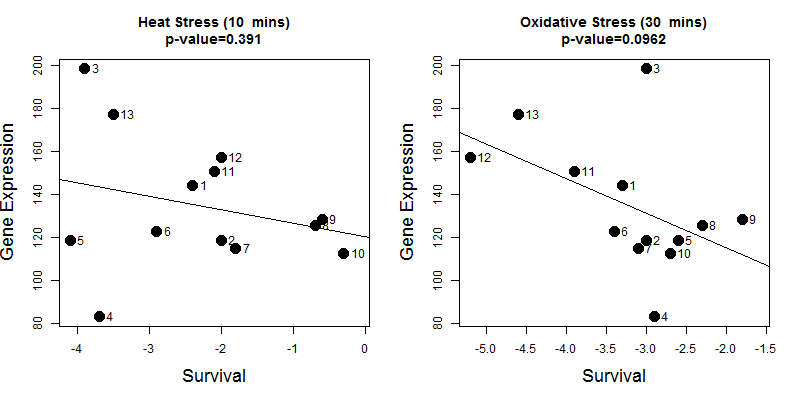

Supplement: S3 File — Expression levels of genes LLKF_0001 –LLKF_1273 plotted against survival after 10 minutes heat and 30 minutes oxidative stress. Survival is expressed as the difference of log CFU/ml after stress and before stress. Numbers indicate fermentations as presented in Table 1. P-values above the plots indicate significance of correlation (assessed by a linear model). (ZIP) [file pone.0167944.s008.zip › S3_File/LLKF_0069_real_dat.png]

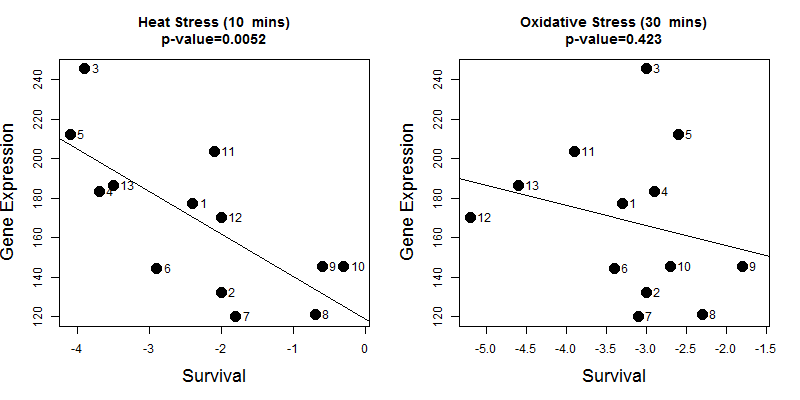

Supplement: S3 File — Expression levels of genes LLKF_0001 –LLKF_1273 plotted against survival after 10 minutes heat and 30 minutes oxidative stress. Survival is expressed as the difference of log CFU/ml after stress and before stress. Numbers indicate fermentations as presented in Table 1. P-values above the plots indicate significance of correlation (assessed by a linear model). (ZIP) [file pone.0167944.s008.zip › S3_File/LLKF_0070_real_dat.png]

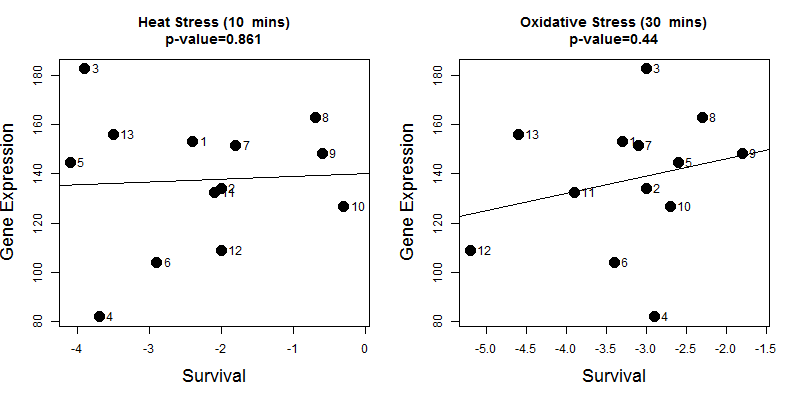

Supplement: S3 File — Expression levels of genes LLKF_0001 –LLKF_1273 plotted against survival after 10 minutes heat and 30 minutes oxidative stress. Survival is expressed as the difference of log CFU/ml after stress and before stress. Numbers indicate fermentations as presented in Table 1. P-values above the plots indicate significance of correlation (assessed by a linear model). (ZIP) [file pone.0167944.s008.zip › S3_File/LLKF_0071_real_dat.png]

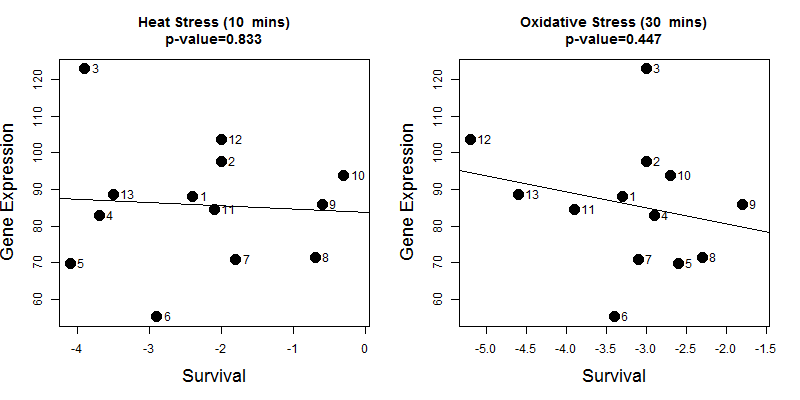

Supplement: S3 File — Expression levels of genes LLKF_0001 –LLKF_1273 plotted against survival after 10 minutes heat and 30 minutes oxidative stress. Survival is expressed as the difference of log CFU/ml after stress and before stress. Numbers indicate fermentations as presented in Table 1. P-values above the plots indicate significance of correlation (assessed by a linear model). (ZIP) [file pone.0167944.s008.zip › S3_File/LLKF_0072_real_dat.png]

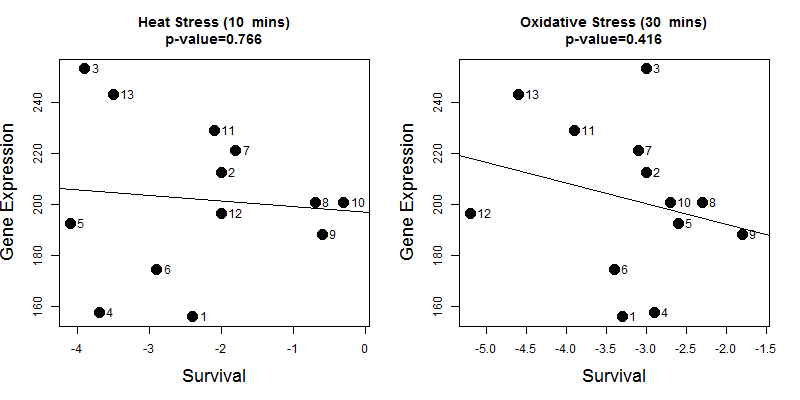

Supplement: S3 File — Expression levels of genes LLKF_0001 –LLKF_1273 plotted against survival after 10 minutes heat and 30 minutes oxidative stress. Survival is expressed as the difference of log CFU/ml after stress and before stress. Numbers indicate fermentations as presented in Table 1. P-values above the plots indicate significance of correlation (assessed by a linear model). (ZIP) [file pone.0167944.s008.zip › S3_File/LLKF_0073_real_dat.png]

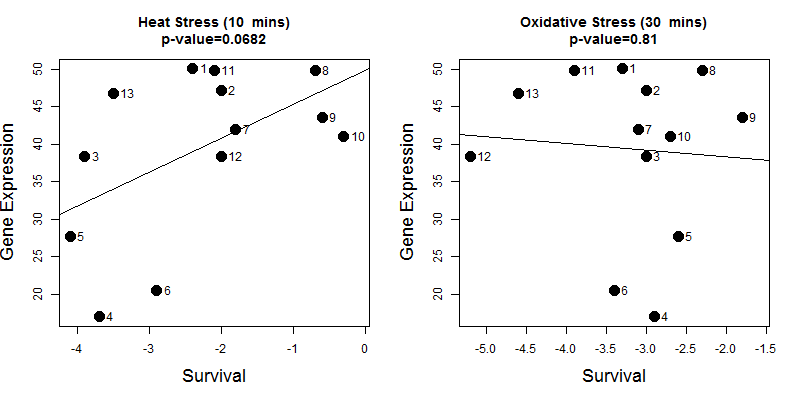

Supplement: S3 File — Expression levels of genes LLKF_0001 –LLKF_1273 plotted against survival after 10 minutes heat and 30 minutes oxidative stress. Survival is expressed as the difference of log CFU/ml after stress and before stress. Numbers indicate fermentations as presented in Table 1. P-values above the plots indicate significance of correlation (assessed by a linear model). (ZIP) [file pone.0167944.s008.zip › S3_File/LLKF_0074_real_dat.png]

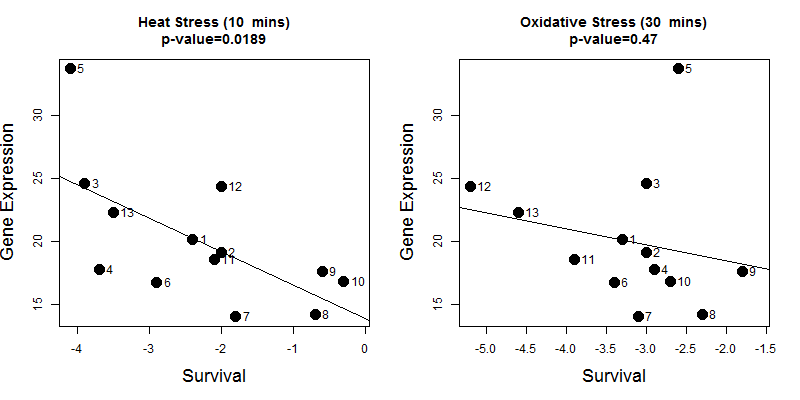

Supplement: S3 File — Expression levels of genes LLKF_0001 –LLKF_1273 plotted against survival after 10 minutes heat and 30 minutes oxidative stress. Survival is expressed as the difference of log CFU/ml after stress and before stress. Numbers indicate fermentations as presented in Table 1. P-values above the plots indicate significance of correlation (assessed by a linear model). (ZIP) [file pone.0167944.s008.zip › S3_File/LLKF_0075_real_dat.png]

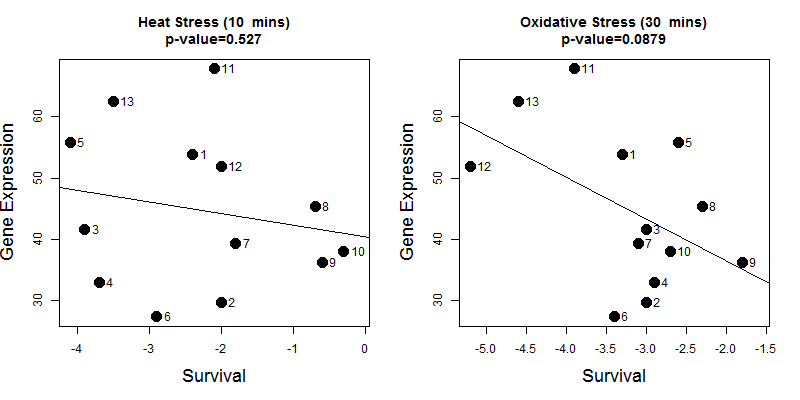

Supplement: S3 File — Expression levels of genes LLKF_0001 –LLKF_1273 plotted against survival after 10 minutes heat and 30 minutes oxidative stress. Survival is expressed as the difference of log CFU/ml after stress and before stress. Numbers indicate fermentations as presented in Table 1. P-values above the plots indicate significance of correlation (assessed by a linear model). (ZIP) [file pone.0167944.s008.zip › S3_File/LLKF_0076_real_dat.png]

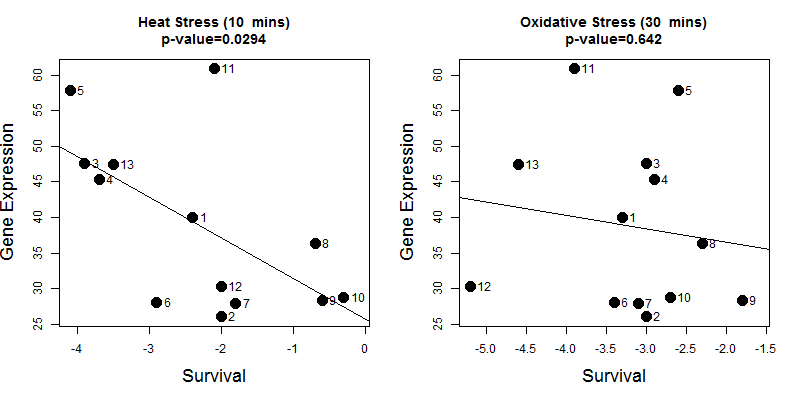

Supplement: S3 File — Expression levels of genes LLKF_0001 –LLKF_1273 plotted against survival after 10 minutes heat and 30 minutes oxidative stress. Survival is expressed as the difference of log CFU/ml after stress and before stress. Numbers indicate fermentations as presented in Table 1. P-values above the plots indicate significance of correlation (assessed by a linear model). (ZIP) [file pone.0167944.s008.zip › S3_File/LLKF_0077_real_dat.png]

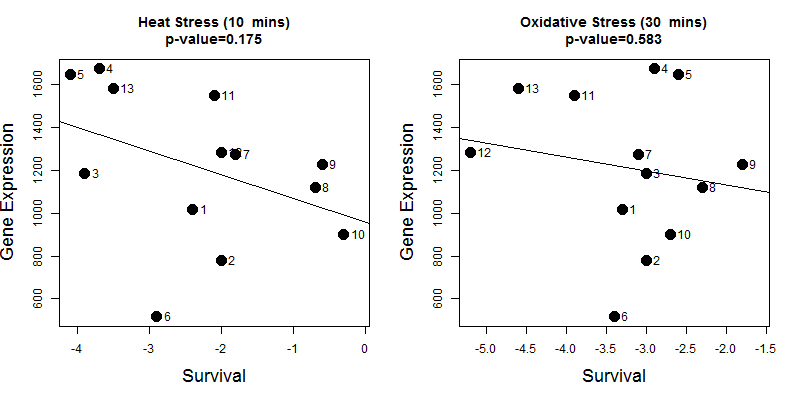

Supplement: S3 File — Expression levels of genes LLKF_0001 –LLKF_1273 plotted against survival after 10 minutes heat and 30 minutes oxidative stress. Survival is expressed as the difference of log CFU/ml after stress and before stress. Numbers indicate fermentations as presented in Table 1. P-values above the plots indicate significance of correlation (assessed by a linear model). (ZIP) [file pone.0167944.s008.zip › S3_File/LLKF_0078_real_dat.png]

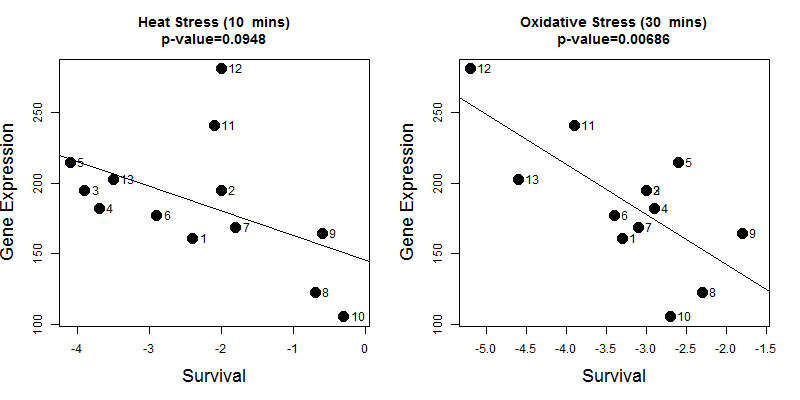

Supplement: S3 File — Expression levels of genes LLKF_0001 –LLKF_1273 plotted against survival after 10 minutes heat and 30 minutes oxidative stress. Survival is expressed as the difference of log CFU/ml after stress and before stress. Numbers indicate fermentations as presented in Table 1. P-values above the plots indicate significance of correlation (assessed by a linear model). (ZIP) [file pone.0167944.s008.zip › S3_File/LLKF_0079_real_dat.png]

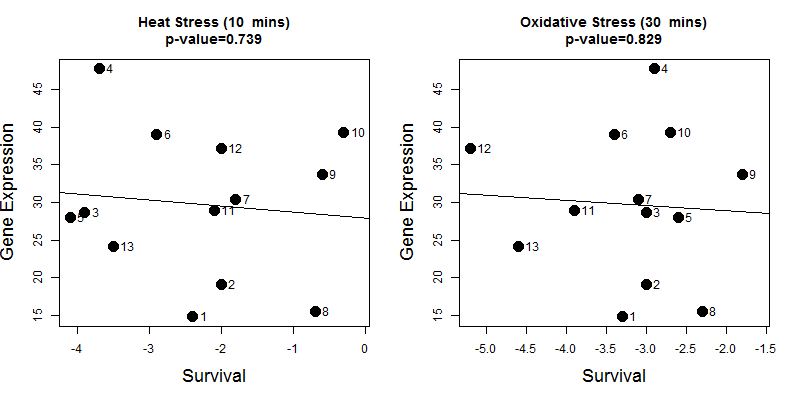

Supplement: S3 File — Expression levels of genes LLKF_0001 –LLKF_1273 plotted against survival after 10 minutes heat and 30 minutes oxidative stress. Survival is expressed as the difference of log CFU/ml after stress and before stress. Numbers indicate fermentations as presented in Table 1. P-values above the plots indicate significance of correlation (assessed by a linear model). (ZIP) [file pone.0167944.s008.zip › S3_File/LLKF_0080_real_dat.png]

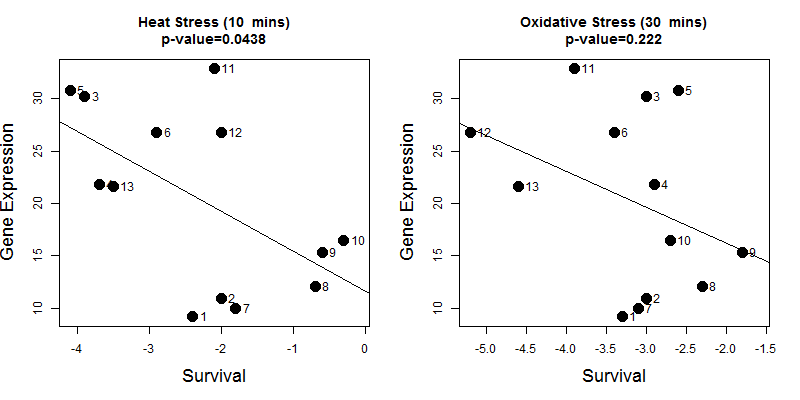

Supplement: S3 File — Expression levels of genes LLKF_0001 –LLKF_1273 plotted against survival after 10 minutes heat and 30 minutes oxidative stress. Survival is expressed as the difference of log CFU/ml after stress and before stress. Numbers indicate fermentations as presented in Table 1. P-values above the plots indicate significance of correlation (assessed by a linear model). (ZIP) [file pone.0167944.s008.zip › S3_File/LLKF_0081_real_dat.png]

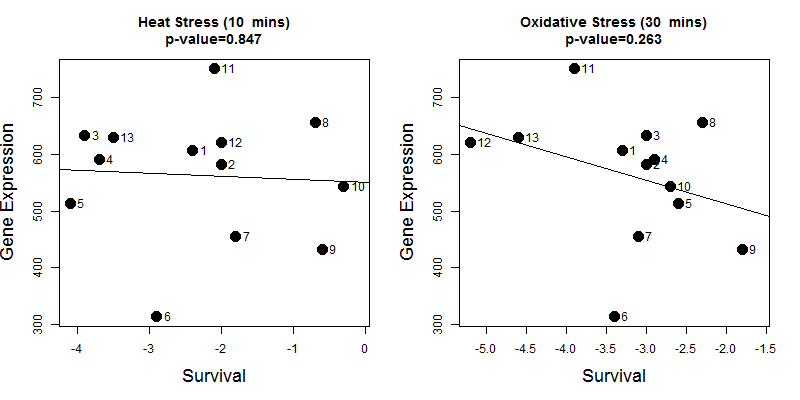

Supplement: S3 File — Expression levels of genes LLKF_0001 –LLKF_1273 plotted against survival after 10 minutes heat and 30 minutes oxidative stress. Survival is expressed as the difference of log CFU/ml after stress and before stress. Numbers indicate fermentations as presented in Table 1. P-values above the plots indicate significance of correlation (assessed by a linear model). (ZIP) [file pone.0167944.s008.zip › S3_File/LLKF_0082_real_dat.png]

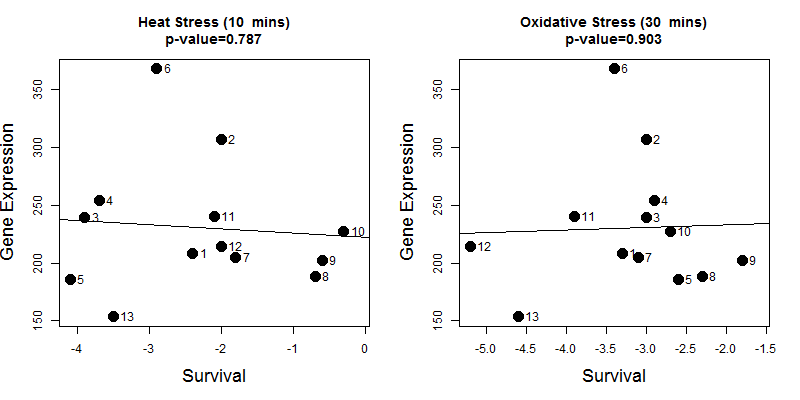

Supplement: S3 File — Expression levels of genes LLKF_0001 –LLKF_1273 plotted against survival after 10 minutes heat and 30 minutes oxidative stress. Survival is expressed as the difference of log CFU/ml after stress and before stress. Numbers indicate fermentations as presented in Table 1. P-values above the plots indicate significance of correlation (assessed by a linear model). (ZIP) [file pone.0167944.s008.zip › S3_File/LLKF_0083_real_dat.png]

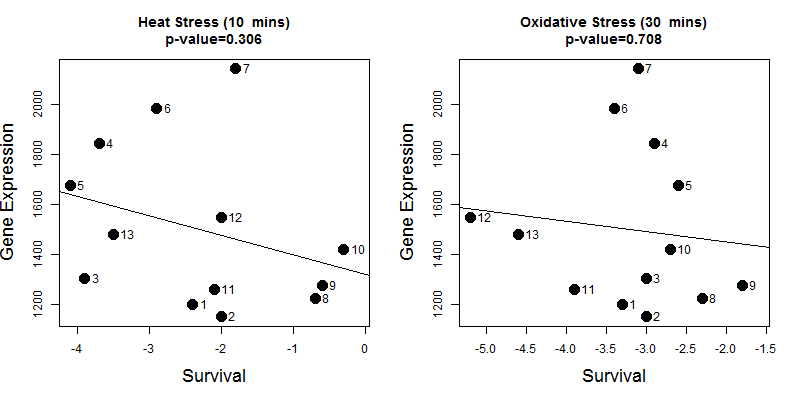

Supplement: S3 File — Expression levels of genes LLKF_0001 –LLKF_1273 plotted against survival after 10 minutes heat and 30 minutes oxidative stress. Survival is expressed as the difference of log CFU/ml after stress and before stress. Numbers indicate fermentations as presented in Table 1. P-values above the plots indicate significance of correlation (assessed by a linear model). (ZIP) [file pone.0167944.s008.zip › S3_File/LLKF_0084_real_dat.png]

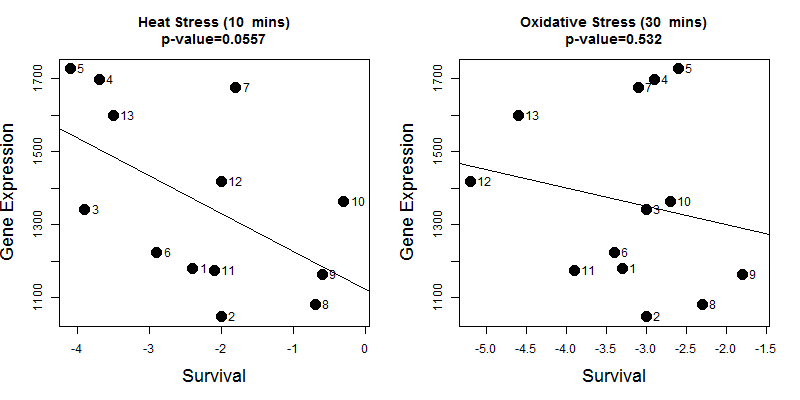

Supplement: S3 File — Expression levels of genes LLKF_0001 –LLKF_1273 plotted against survival after 10 minutes heat and 30 minutes oxidative stress. Survival is expressed as the difference of log CFU/ml after stress and before stress. Numbers indicate fermentations as presented in Table 1. P-values above the plots indicate significance of correlation (assessed by a linear model). (ZIP) [file pone.0167944.s008.zip › S3_File/LLKF_0085_real_dat.png]

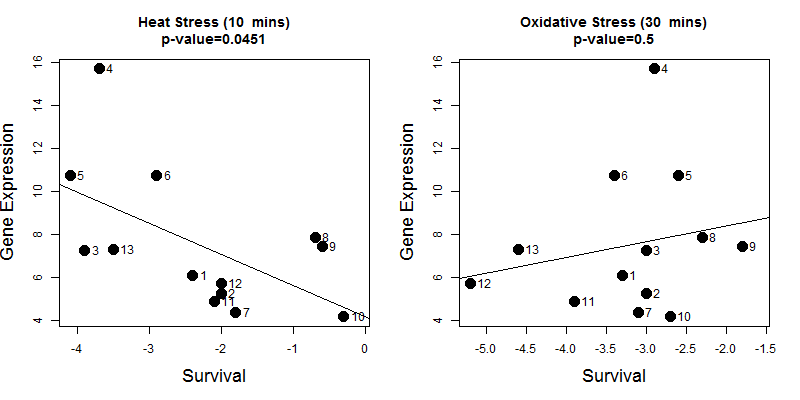

Supplement: S3 File — Expression levels of genes LLKF_0001 –LLKF_1273 plotted against survival after 10 minutes heat and 30 minutes oxidative stress. Survival is expressed as the difference of log CFU/ml after stress and before stress. Numbers indicate fermentations as presented in Table 1. P-values above the plots indicate significance of correlation (assessed by a linear model). (ZIP) [file pone.0167944.s008.zip › S3_File/LLKF_0086_real_dat.png]

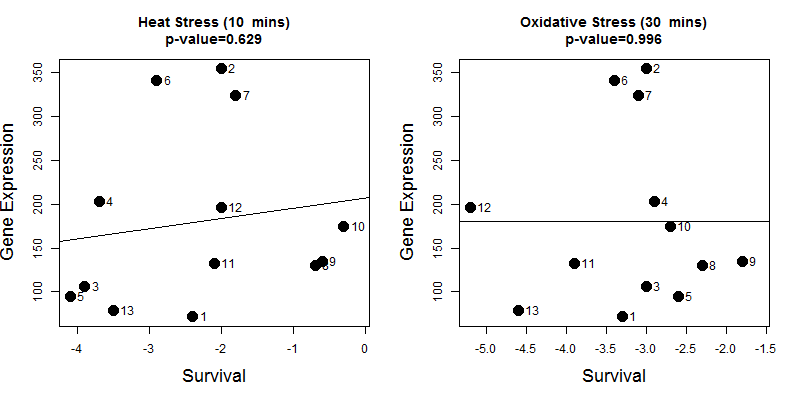

Supplement: S3 File — Expression levels of genes LLKF_0001 –LLKF_1273 plotted against survival after 10 minutes heat and 30 minutes oxidative stress. Survival is expressed as the difference of log CFU/ml after stress and before stress. Numbers indicate fermentations as presented in Table 1. P-values above the plots indicate significance of correlation (assessed by a linear model). (ZIP) [file pone.0167944.s008.zip › S3_File/LLKF_0087_real_dat.png]

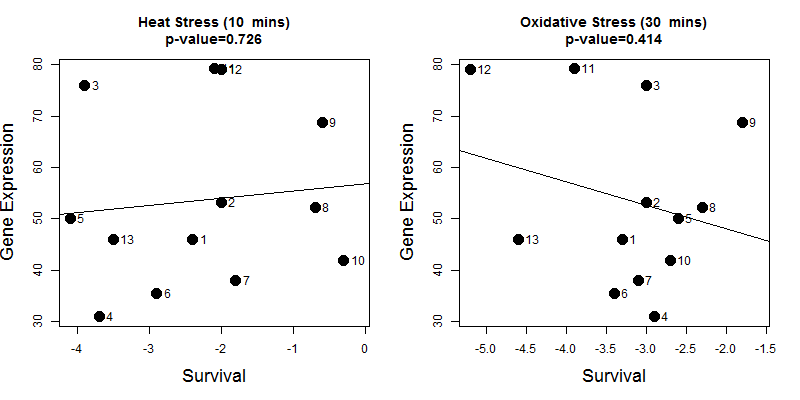

Supplement: S3 File — Expression levels of genes LLKF_0001 –LLKF_1273 plotted against survival after 10 minutes heat and 30 minutes oxidative stress. Survival is expressed as the difference of log CFU/ml after stress and before stress. Numbers indicate fermentations as presented in Table 1. P-values above the plots indicate significance of correlation (assessed by a linear model). (ZIP) [file pone.0167944.s008.zip › S3_File/LLKF_0088_real_dat.png]

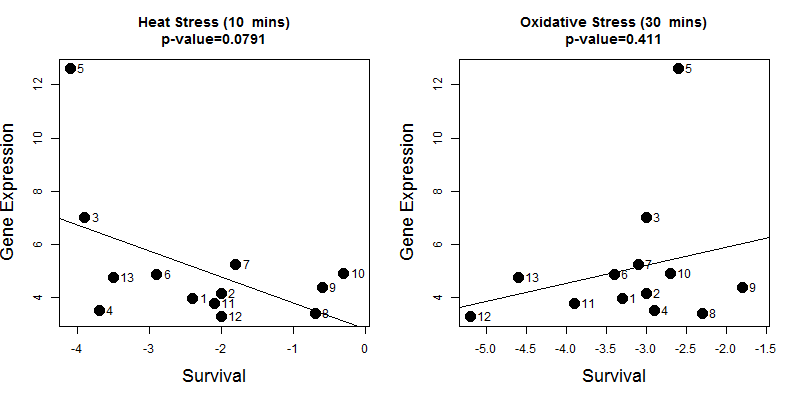

Supplement: S3 File — Expression levels of genes LLKF_0001 –LLKF_1273 plotted against survival after 10 minutes heat and 30 minutes oxidative stress. Survival is expressed as the difference of log CFU/ml after stress and before stress. Numbers indicate fermentations as presented in Table 1. P-values above the plots indicate significance of correlation (assessed by a linear model). (ZIP) [file pone.0167944.s008.zip › S3_File/LLKF_0089_real_dat.png]

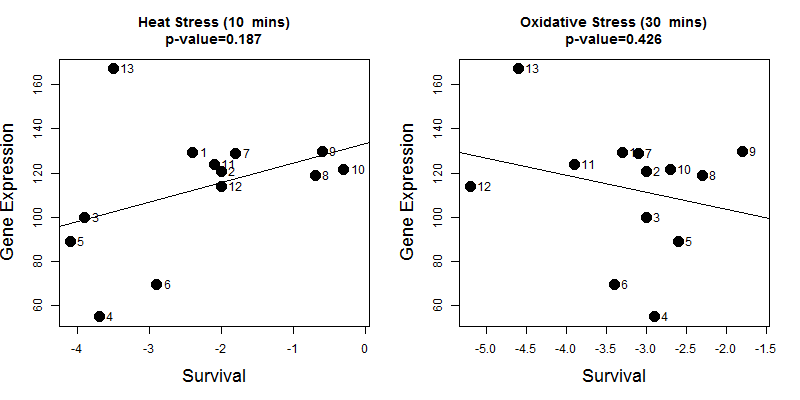

Supplement: S3 File — Expression levels of genes LLKF_0001 –LLKF_1273 plotted against survival after 10 minutes heat and 30 minutes oxidative stress. Survival is expressed as the difference of log CFU/ml after stress and before stress. Numbers indicate fermentations as presented in Table 1. P-values above the plots indicate significance of correlation (assessed by a linear model). (ZIP) [file pone.0167944.s008.zip › S3_File/LLKF_0090_real_dat.png]

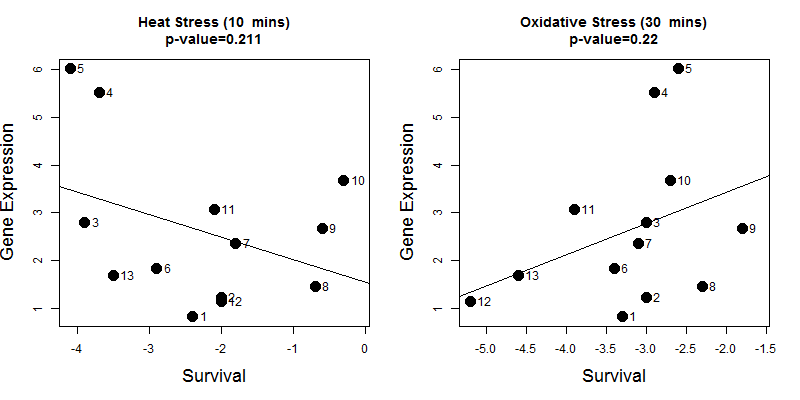

Supplement: S3 File — Expression levels of genes LLKF_0001 –LLKF_1273 plotted against survival after 10 minutes heat and 30 minutes oxidative stress. Survival is expressed as the difference of log CFU/ml after stress and before stress. Numbers indicate fermentations as presented in Table 1. P-values above the plots indicate significance of correlation (assessed by a linear model). (ZIP) [file pone.0167944.s008.zip › S3_File/LLKF_0091_real_dat.png]

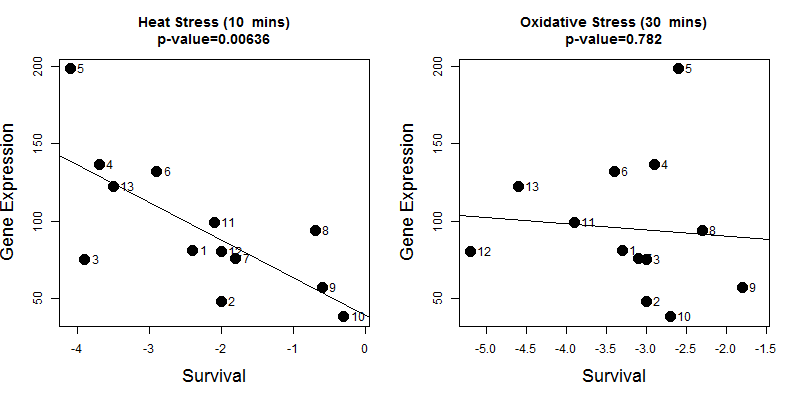

Supplement: S3 File — Expression levels of genes LLKF_0001 –LLKF_1273 plotted against survival after 10 minutes heat and 30 minutes oxidative stress. Survival is expressed as the difference of log CFU/ml after stress and before stress. Numbers indicate fermentations as presented in Table 1. P-values above the plots indicate significance of correlation (assessed by a linear model). (ZIP) [file pone.0167944.s008.zip › S3_File/LLKF_0092_real_dat.png]

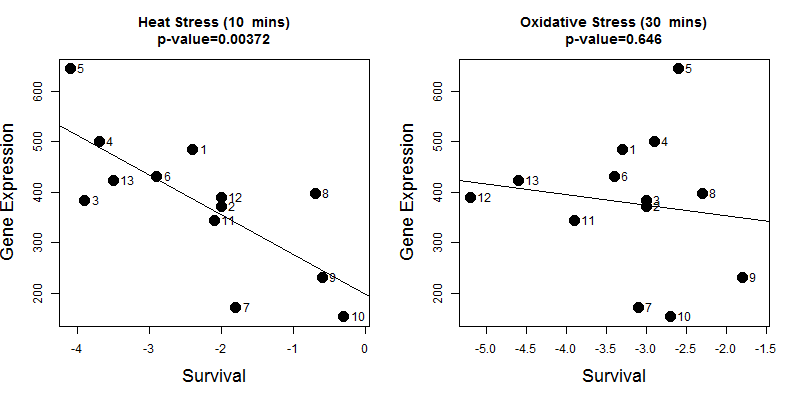

Supplement: S3 File — Expression levels of genes LLKF_0001 –LLKF_1273 plotted against survival after 10 minutes heat and 30 minutes oxidative stress. Survival is expressed as the difference of log CFU/ml after stress and before stress. Numbers indicate fermentations as presented in Table 1. P-values above the plots indicate significance of correlation (assessed by a linear model). (ZIP) [file pone.0167944.s008.zip › S3_File/LLKF_0093_real_dat.png]

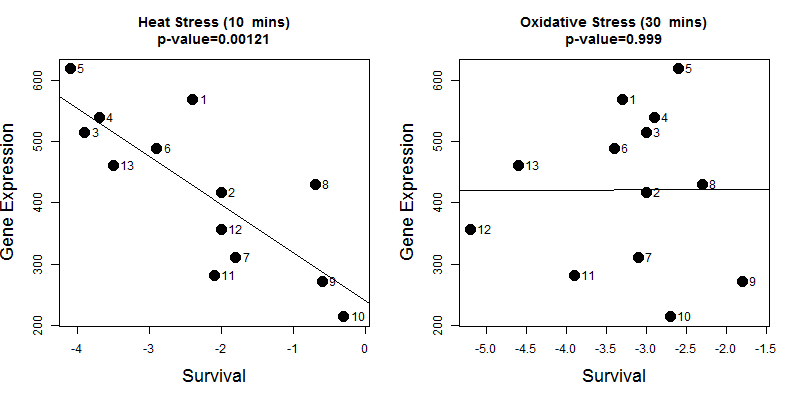

Supplement: S3 File — Expression levels of genes LLKF_0001 –LLKF_1273 plotted against survival after 10 minutes heat and 30 minutes oxidative stress. Survival is expressed as the difference of log CFU/ml after stress and before stress. Numbers indicate fermentations as presented in Table 1. P-values above the plots indicate significance of correlation (assessed by a linear model). (ZIP) [file pone.0167944.s008.zip › S3_File/LLKF_0094_real_dat.png]

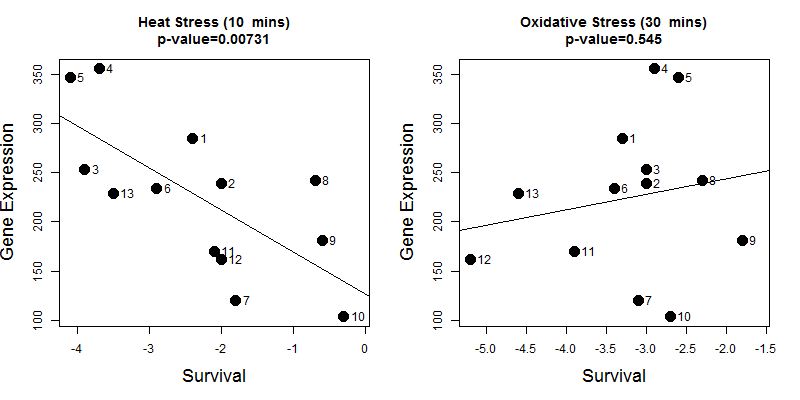

Supplement: S3 File — Expression levels of genes LLKF_0001 –LLKF_1273 plotted against survival after 10 minutes heat and 30 minutes oxidative stress. Survival is expressed as the difference of log CFU/ml after stress and before stress. Numbers indicate fermentations as presented in Table 1. P-values above the plots indicate significance of correlation (assessed by a linear model). (ZIP) [file pone.0167944.s008.zip › S3_File/LLKF_0095_real_dat.png]

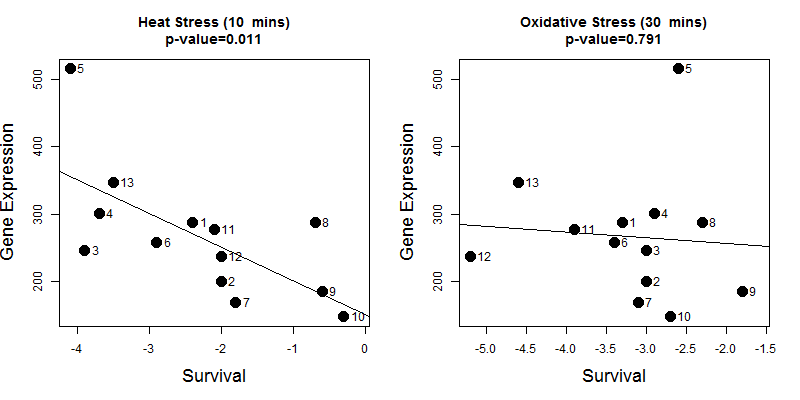

Supplement: S3 File — Expression levels of genes LLKF_0001 –LLKF_1273 plotted against survival after 10 minutes heat and 30 minutes oxidative stress. Survival is expressed as the difference of log CFU/ml after stress and before stress. Numbers indicate fermentations as presented in Table 1. P-values above the plots indicate significance of correlation (assessed by a linear model). (ZIP) [file pone.0167944.s008.zip › S3_File/LLKF_0096_real_dat.png]

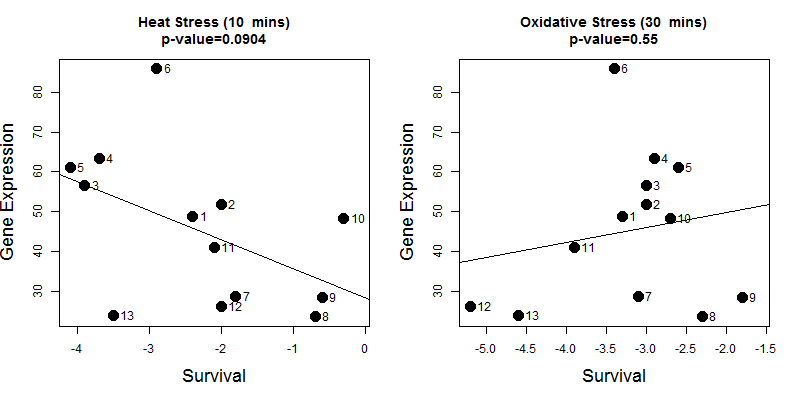

Supplement: S3 File — Expression levels of genes LLKF_0001 –LLKF_1273 plotted against survival after 10 minutes heat and 30 minutes oxidative stress. Survival is expressed as the difference of log CFU/ml after stress and before stress. Numbers indicate fermentations as presented in Table 1. P-values above the plots indicate significance of correlation (assessed by a linear model). (ZIP) [file pone.0167944.s008.zip › S3_File/LLKF_0097_real_dat.png]

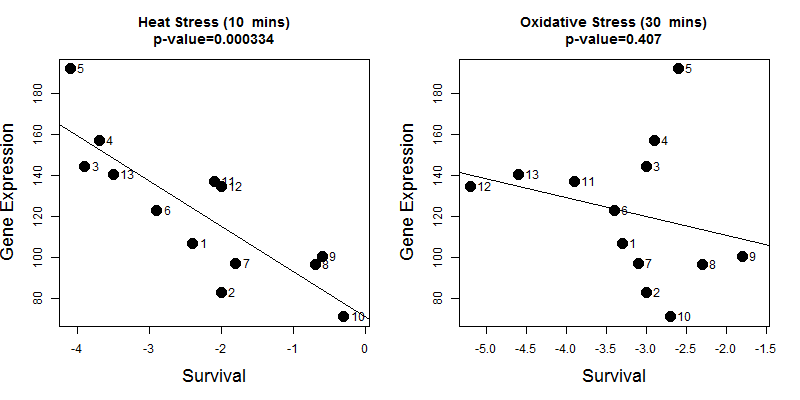

Supplement: S3 File — Expression levels of genes LLKF_0001 –LLKF_1273 plotted against survival after 10 minutes heat and 30 minutes oxidative stress. Survival is expressed as the difference of log CFU/ml after stress and before stress. Numbers indicate fermentations as presented in Table 1. P-values above the plots indicate significance of correlation (assessed by a linear model). (ZIP) [file pone.0167944.s008.zip › S3_File/LLKF_0098_real_dat.png]

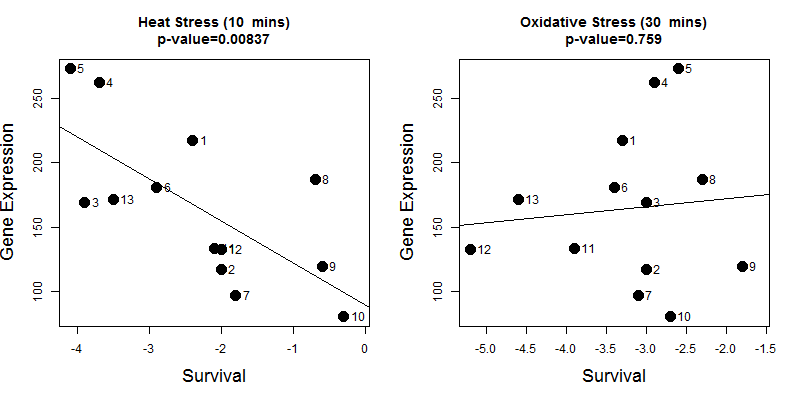

Supplement: S3 File — Expression levels of genes LLKF_0001 –LLKF_1273 plotted against survival after 10 minutes heat and 30 minutes oxidative stress. Survival is expressed as the difference of log CFU/ml after stress and before stress. Numbers indicate fermentations as presented in Table 1. P-values above the plots indicate significance of correlation (assessed by a linear model). (ZIP) [file pone.0167944.s008.zip › S3_File/LLKF_0099_real_dat.png]
