# Supplementary figures and images for: Strain-Dependent Transcriptome Signatures for Robustness in Lactococcus lactis (part 4 of 13)
Source: PLoS One. 2016 Dec 14;11(12):e0167944. doi: 10.1371/journal.pone.0167944 (PMC5156439; doi:10.1371/journal.pone.0167944)

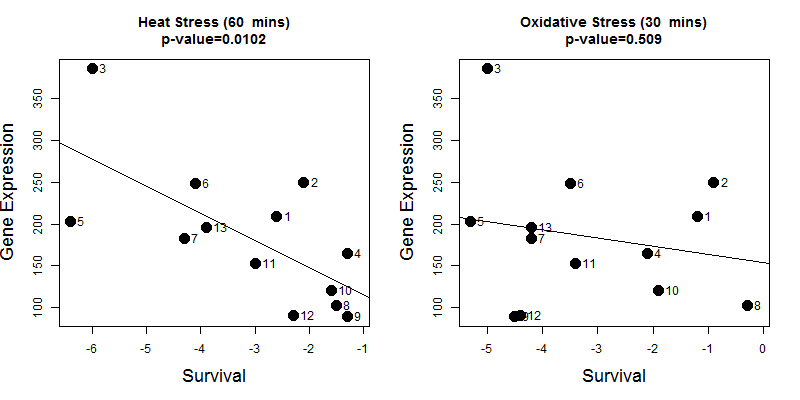

Supplement: S2 File — Expression levels of genes L75676 –L1889726 plotted against survival after 60 minutes heat and 30 min oxidative stress. Survival is expressed as the difference of log CFU/ml after stress and before stress. Numbers indicate fermentations as presented in Table 1. P-values above the plots indicate significance of correlation (assessed by a linear model). (ZIP) [file pone.0167944.s007.zip › S2_File/L109379_real_dat.png]

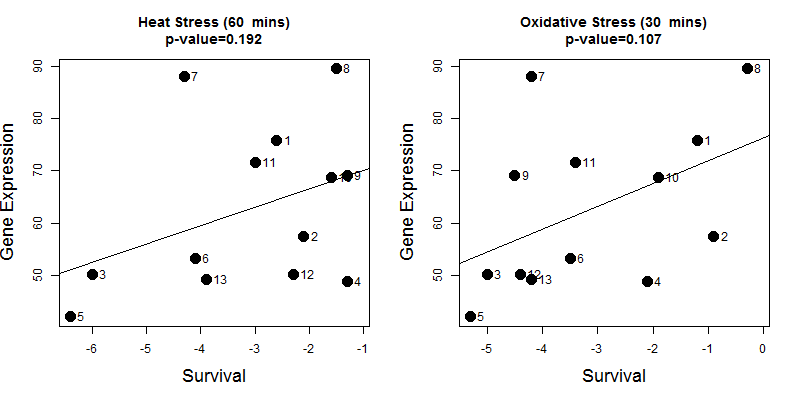

Supplement: S2 File — Expression levels of genes L75676 –L1889726 plotted against survival after 60 minutes heat and 30 min oxidative stress. Survival is expressed as the difference of log CFU/ml after stress and before stress. Numbers indicate fermentations as presented in Table 1. P-values above the plots indicate significance of correlation (assessed by a linear model). (ZIP) [file pone.0167944.s007.zip › S2_File/L109453_real_dat.png]

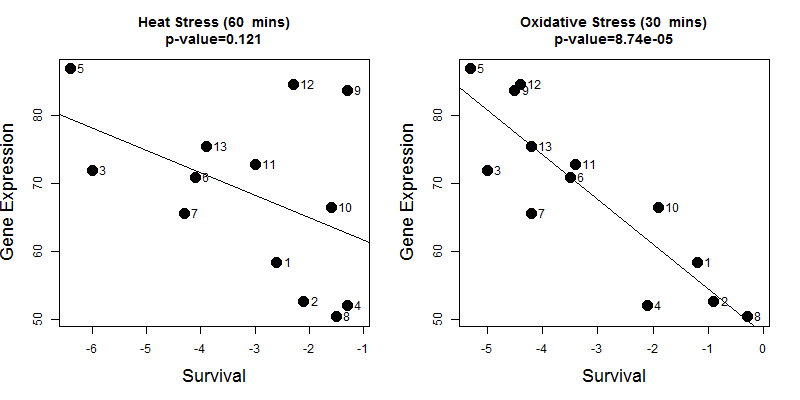

Supplement: S2 File — Expression levels of genes L75676 –L1889726 plotted against survival after 60 minutes heat and 30 min oxidative stress. Survival is expressed as the difference of log CFU/ml after stress and before stress. Numbers indicate fermentations as presented in Table 1. P-values above the plots indicate significance of correlation (assessed by a linear model). (ZIP) [file pone.0167944.s007.zip › S2_File/L109527_real_dat.png]

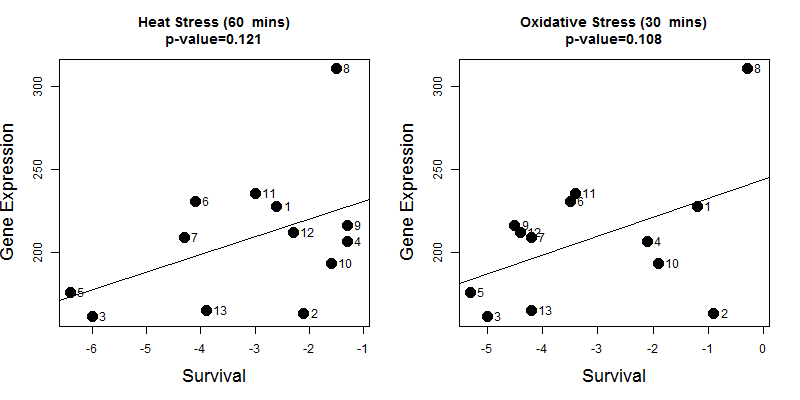

Supplement: S2 File — Expression levels of genes L75676 –L1889726 plotted against survival after 60 minutes heat and 30 min oxidative stress. Survival is expressed as the difference of log CFU/ml after stress and before stress. Numbers indicate fermentations as presented in Table 1. P-values above the plots indicate significance of correlation (assessed by a linear model). (ZIP) [file pone.0167944.s007.zip › S2_File/L109747_real_dat.png]

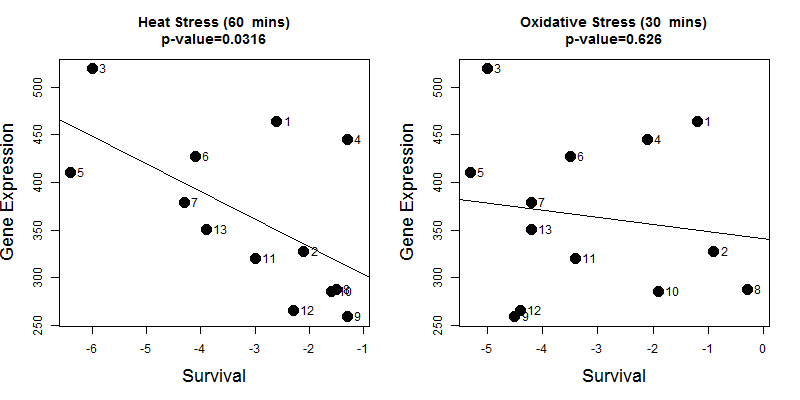

Supplement: S2 File — Expression levels of genes L75676 –L1889726 plotted against survival after 60 minutes heat and 30 min oxidative stress. Survival is expressed as the difference of log CFU/ml after stress and before stress. Numbers indicate fermentations as presented in Table 1. P-values above the plots indicate significance of correlation (assessed by a linear model). (ZIP) [file pone.0167944.s007.zip › S2_File/L109882_real_dat.png]

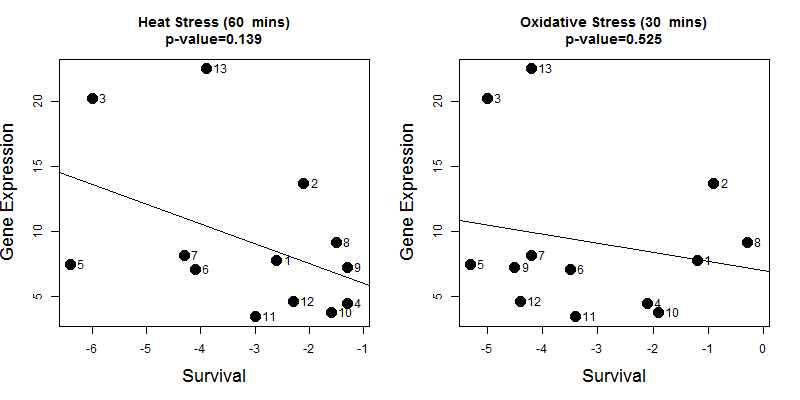

Supplement: S2 File — Expression levels of genes L75676 –L1889726 plotted against survival after 60 minutes heat and 30 min oxidative stress. Survival is expressed as the difference of log CFU/ml after stress and before stress. Numbers indicate fermentations as presented in Table 1. P-values above the plots indicate significance of correlation (assessed by a linear model). (ZIP) [file pone.0167944.s007.zip › S2_File/L110165_real_dat.png]

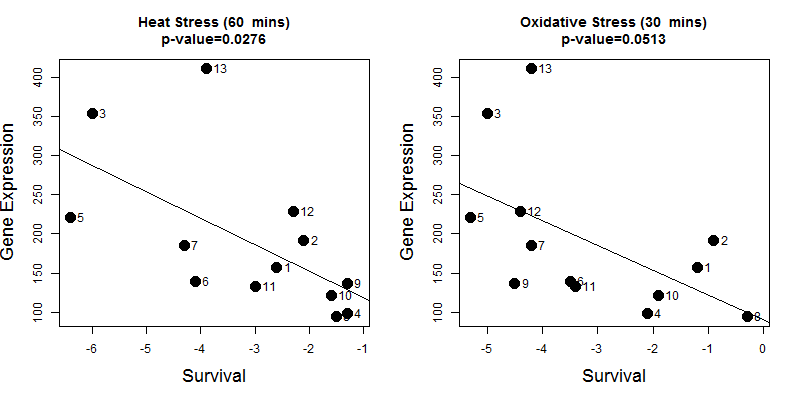

Supplement: S2 File — Expression levels of genes L75676 –L1889726 plotted against survival after 60 minutes heat and 30 min oxidative stress. Survival is expressed as the difference of log CFU/ml after stress and before stress. Numbers indicate fermentations as presented in Table 1. P-values above the plots indicate significance of correlation (assessed by a linear model). (ZIP) [file pone.0167944.s007.zip › S2_File/L110351_real_dat.png]

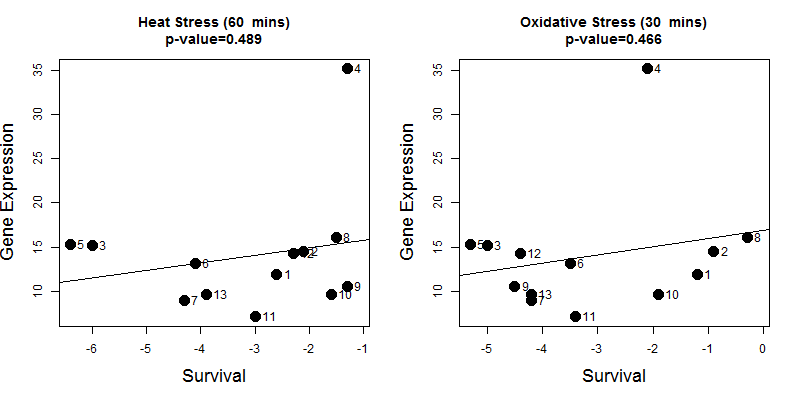

Supplement: S2 File — Expression levels of genes L75676 –L1889726 plotted against survival after 60 minutes heat and 30 min oxidative stress. Survival is expressed as the difference of log CFU/ml after stress and before stress. Numbers indicate fermentations as presented in Table 1. P-values above the plots indicate significance of correlation (assessed by a linear model). (ZIP) [file pone.0167944.s007.zip › S2_File/L110431_real_dat.png]

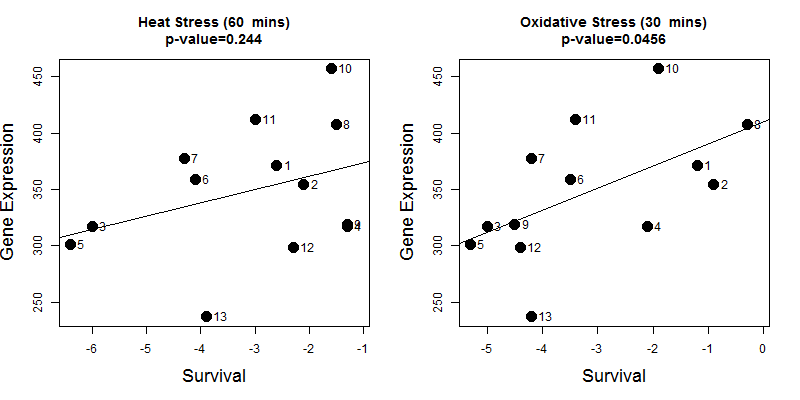

Supplement: S2 File — Expression levels of genes L75676 –L1889726 plotted against survival after 60 minutes heat and 30 min oxidative stress. Survival is expressed as the difference of log CFU/ml after stress and before stress. Numbers indicate fermentations as presented in Table 1. P-values above the plots indicate significance of correlation (assessed by a linear model). (ZIP) [file pone.0167944.s007.zip › S2_File/L110441_real_dat.png]

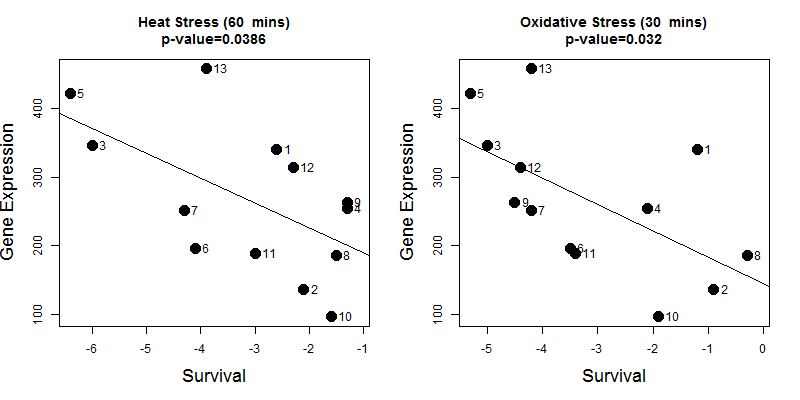

Supplement: S2 File — Expression levels of genes L75676 –L1889726 plotted against survival after 60 minutes heat and 30 min oxidative stress. Survival is expressed as the difference of log CFU/ml after stress and before stress. Numbers indicate fermentations as presented in Table 1. P-values above the plots indicate significance of correlation (assessed by a linear model). (ZIP) [file pone.0167944.s007.zip › S2_File/L110467_real_dat.png]

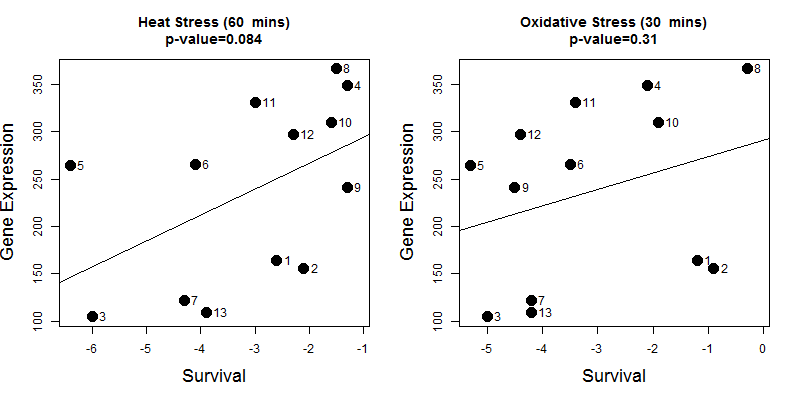

Supplement: S2 File — Expression levels of genes L75676 –L1889726 plotted against survival after 60 minutes heat and 30 min oxidative stress. Survival is expressed as the difference of log CFU/ml after stress and before stress. Numbers indicate fermentations as presented in Table 1. P-values above the plots indicate significance of correlation (assessed by a linear model). (ZIP) [file pone.0167944.s007.zip › S2_File/L110479_real_dat.png]

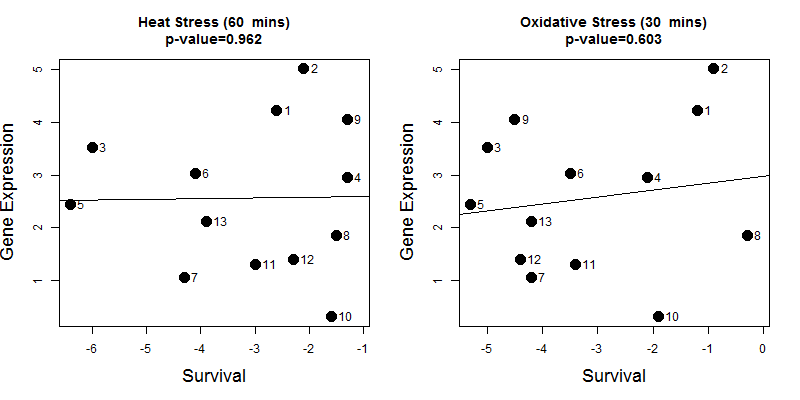

Supplement: S2 File — Expression levels of genes L75676 –L1889726 plotted against survival after 60 minutes heat and 30 min oxidative stress. Survival is expressed as the difference of log CFU/ml after stress and before stress. Numbers indicate fermentations as presented in Table 1. P-values above the plots indicate significance of correlation (assessed by a linear model). (ZIP) [file pone.0167944.s007.zip › S2_File/L110560_real_dat.png]

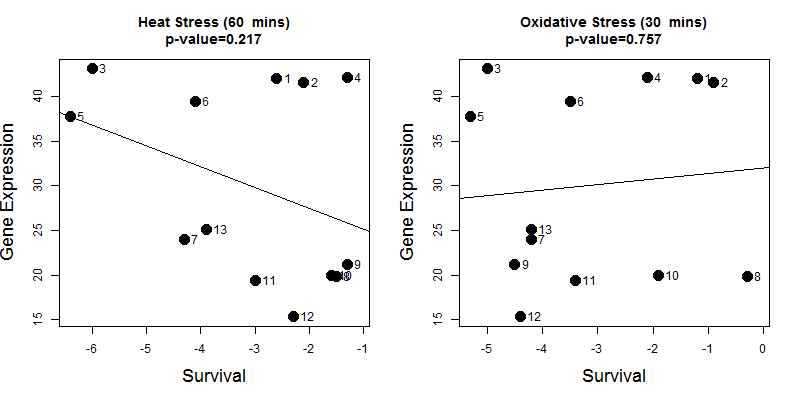

Supplement: S2 File — Expression levels of genes L75676 –L1889726 plotted against survival after 60 minutes heat and 30 min oxidative stress. Survival is expressed as the difference of log CFU/ml after stress and before stress. Numbers indicate fermentations as presented in Table 1. P-values above the plots indicate significance of correlation (assessed by a linear model). (ZIP) [file pone.0167944.s007.zip › S2_File/L110564_real_dat.png]

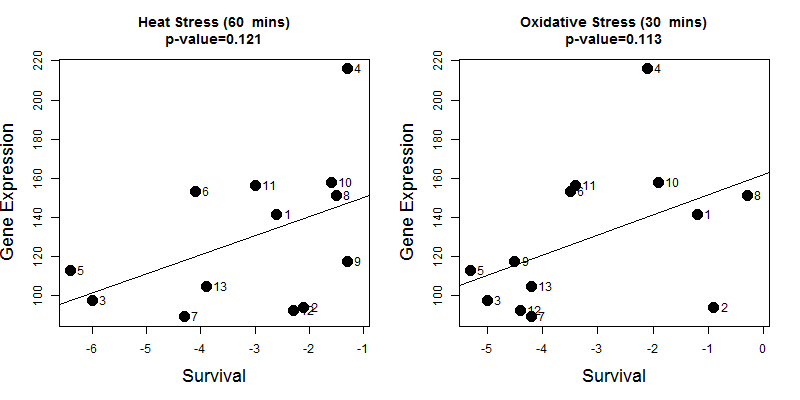

Supplement: S2 File — Expression levels of genes L75676 –L1889726 plotted against survival after 60 minutes heat and 30 min oxidative stress. Survival is expressed as the difference of log CFU/ml after stress and before stress. Numbers indicate fermentations as presented in Table 1. P-values above the plots indicate significance of correlation (assessed by a linear model). (ZIP) [file pone.0167944.s007.zip › S2_File/L110588_real_dat.png]

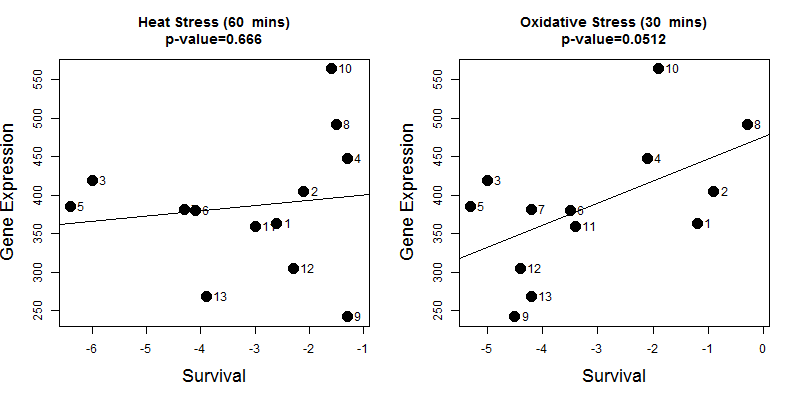

Supplement: S2 File — Expression levels of genes L75676 –L1889726 plotted against survival after 60 minutes heat and 30 min oxidative stress. Survival is expressed as the difference of log CFU/ml after stress and before stress. Numbers indicate fermentations as presented in Table 1. P-values above the plots indicate significance of correlation (assessed by a linear model). (ZIP) [file pone.0167944.s007.zip › S2_File/L110899_real_dat.png]

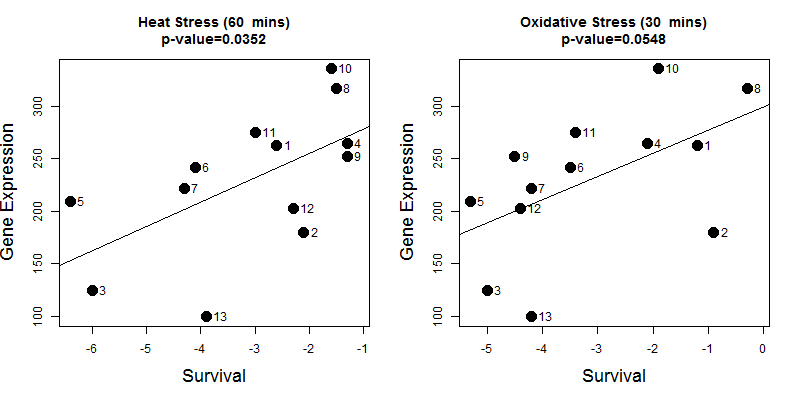

Supplement: S2 File — Expression levels of genes L75676 –L1889726 plotted against survival after 60 minutes heat and 30 min oxidative stress. Survival is expressed as the difference of log CFU/ml after stress and before stress. Numbers indicate fermentations as presented in Table 1. P-values above the plots indicate significance of correlation (assessed by a linear model). (ZIP) [file pone.0167944.s007.zip › S2_File/L110933_real_dat.png]

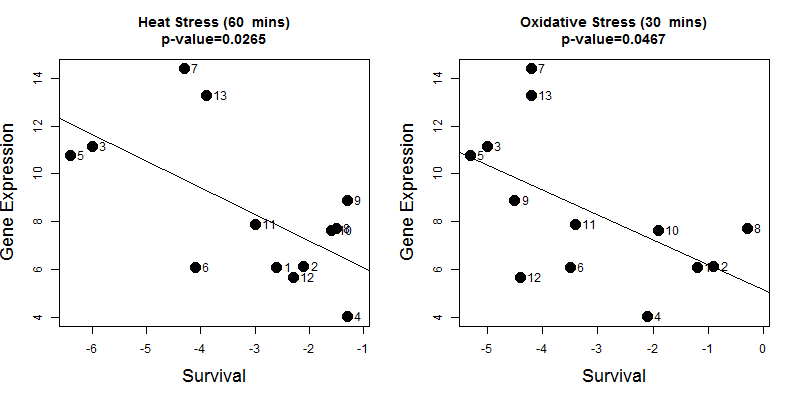

Supplement: S2 File — Expression levels of genes L75676 –L1889726 plotted against survival after 60 minutes heat and 30 min oxidative stress. Survival is expressed as the difference of log CFU/ml after stress and before stress. Numbers indicate fermentations as presented in Table 1. P-values above the plots indicate significance of correlation (assessed by a linear model). (ZIP) [file pone.0167944.s007.zip › S2_File/L110960_real_dat.png]

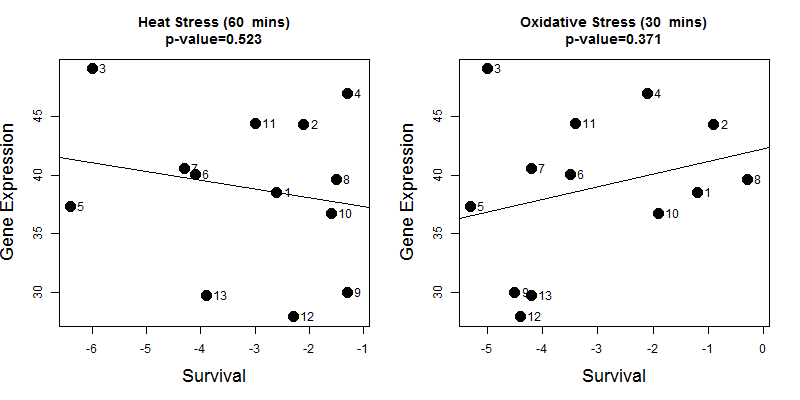

Supplement: S2 File — Expression levels of genes L75676 –L1889726 plotted against survival after 60 minutes heat and 30 min oxidative stress. Survival is expressed as the difference of log CFU/ml after stress and before stress. Numbers indicate fermentations as presented in Table 1. P-values above the plots indicate significance of correlation (assessed by a linear model). (ZIP) [file pone.0167944.s007.zip › S2_File/L111003_real_dat.png]

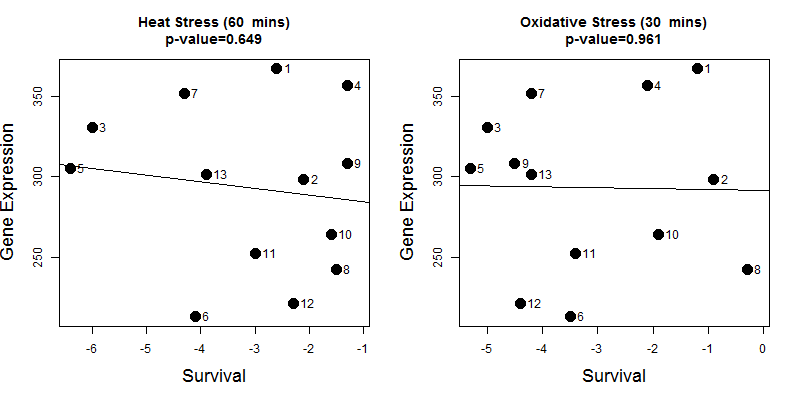

Supplement: S2 File — Expression levels of genes L75676 –L1889726 plotted against survival after 60 minutes heat and 30 min oxidative stress. Survival is expressed as the difference of log CFU/ml after stress and before stress. Numbers indicate fermentations as presented in Table 1. P-values above the plots indicate significance of correlation (assessed by a linear model). (ZIP) [file pone.0167944.s007.zip › S2_File/L111126_real_dat.png]

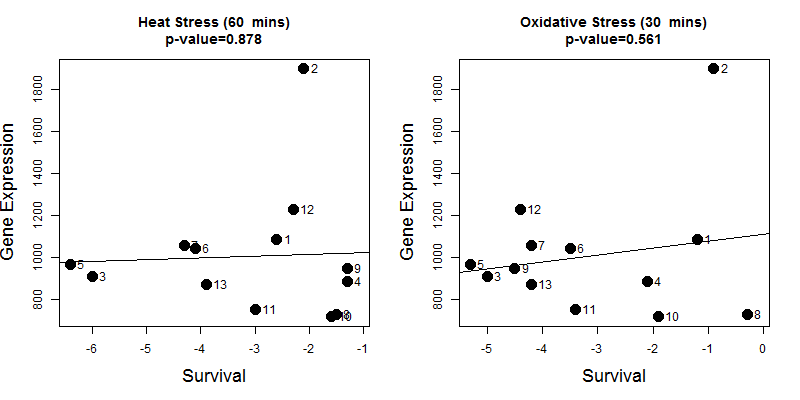

Supplement: S2 File — Expression levels of genes L75676 –L1889726 plotted against survival after 60 minutes heat and 30 min oxidative stress. Survival is expressed as the difference of log CFU/ml after stress and before stress. Numbers indicate fermentations as presented in Table 1. P-values above the plots indicate significance of correlation (assessed by a linear model). (ZIP) [file pone.0167944.s007.zip › S2_File/L111159_real_dat.png]

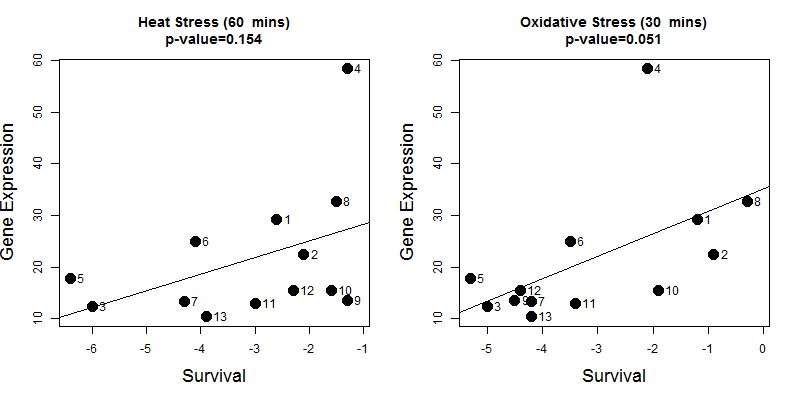

Supplement: S2 File — Expression levels of genes L75676 –L1889726 plotted against survival after 60 minutes heat and 30 min oxidative stress. Survival is expressed as the difference of log CFU/ml after stress and before stress. Numbers indicate fermentations as presented in Table 1. P-values above the plots indicate significance of correlation (assessed by a linear model). (ZIP) [file pone.0167944.s007.zip › S2_File/L111162_real_dat.png]

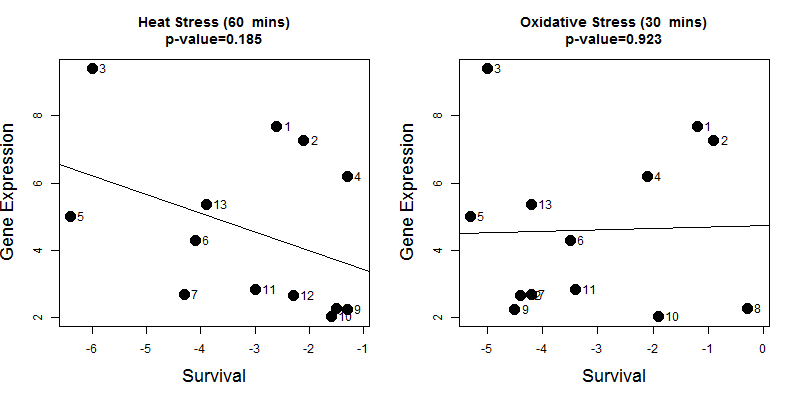

Supplement: S2 File — Expression levels of genes L75676 –L1889726 plotted against survival after 60 minutes heat and 30 min oxidative stress. Survival is expressed as the difference of log CFU/ml after stress and before stress. Numbers indicate fermentations as presented in Table 1. P-values above the plots indicate significance of correlation (assessed by a linear model). (ZIP) [file pone.0167944.s007.zip › S2_File/L111351_real_dat.png]

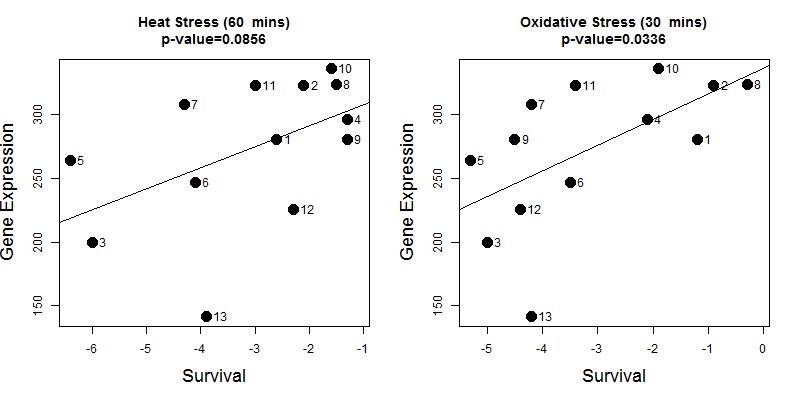

Supplement: S2 File — Expression levels of genes L75676 –L1889726 plotted against survival after 60 minutes heat and 30 min oxidative stress. Survival is expressed as the difference of log CFU/ml after stress and before stress. Numbers indicate fermentations as presented in Table 1. P-values above the plots indicate significance of correlation (assessed by a linear model). (ZIP) [file pone.0167944.s007.zip › S2_File/L111484_real_dat.png]

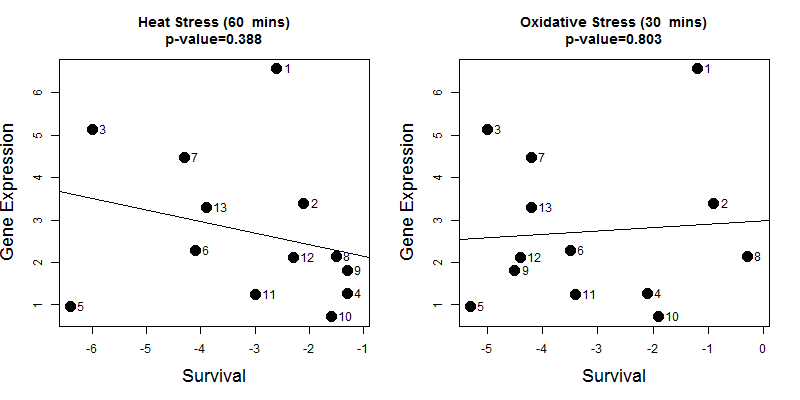

Supplement: S2 File — Expression levels of genes L75676 –L1889726 plotted against survival after 60 minutes heat and 30 min oxidative stress. Survival is expressed as the difference of log CFU/ml after stress and before stress. Numbers indicate fermentations as presented in Table 1. P-values above the plots indicate significance of correlation (assessed by a linear model). (ZIP) [file pone.0167944.s007.zip › S2_File/L111521_real_dat.png]

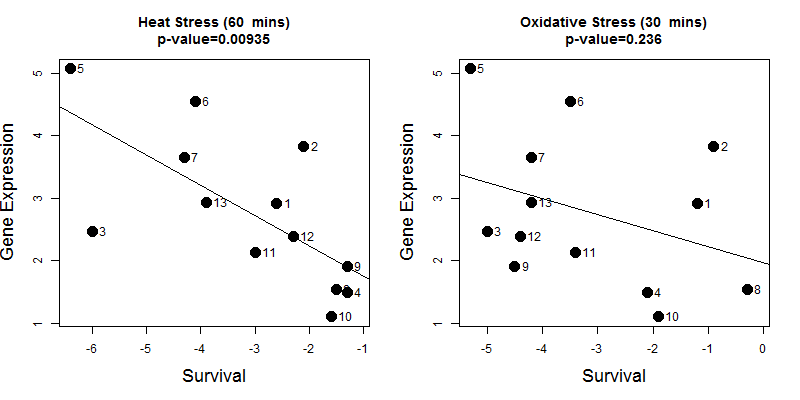

Supplement: S2 File — Expression levels of genes L75676 –L1889726 plotted against survival after 60 minutes heat and 30 min oxidative stress. Survival is expressed as the difference of log CFU/ml after stress and before stress. Numbers indicate fermentations as presented in Table 1. P-values above the plots indicate significance of correlation (assessed by a linear model). (ZIP) [file pone.0167944.s007.zip › S2_File/L111525_real_dat.png]

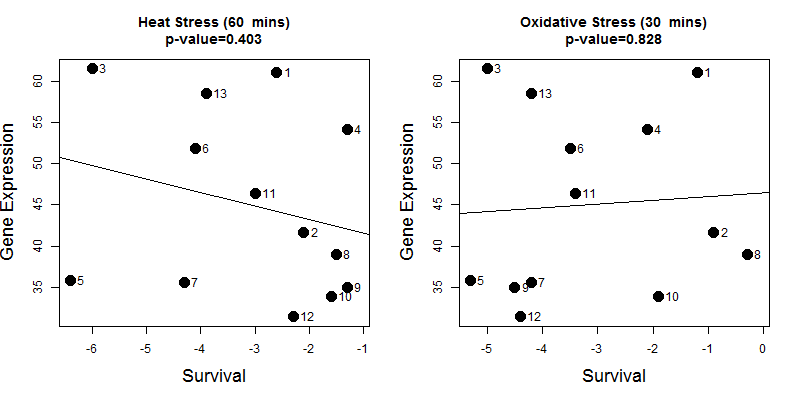

Supplement: S2 File — Expression levels of genes L75676 –L1889726 plotted against survival after 60 minutes heat and 30 min oxidative stress. Survival is expressed as the difference of log CFU/ml after stress and before stress. Numbers indicate fermentations as presented in Table 1. P-values above the plots indicate significance of correlation (assessed by a linear model). (ZIP) [file pone.0167944.s007.zip › S2_File/L111950_real_dat.png]

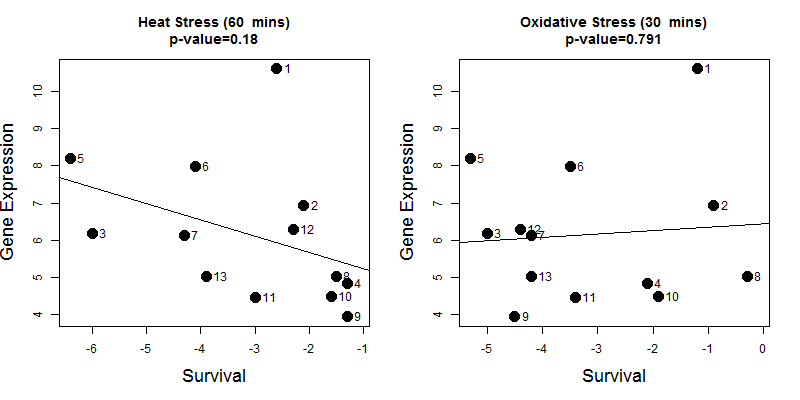

Supplement: S2 File — Expression levels of genes L75676 –L1889726 plotted against survival after 60 minutes heat and 30 min oxidative stress. Survival is expressed as the difference of log CFU/ml after stress and before stress. Numbers indicate fermentations as presented in Table 1. P-values above the plots indicate significance of correlation (assessed by a linear model). (ZIP) [file pone.0167944.s007.zip › S2_File/L112195_real_dat.png]

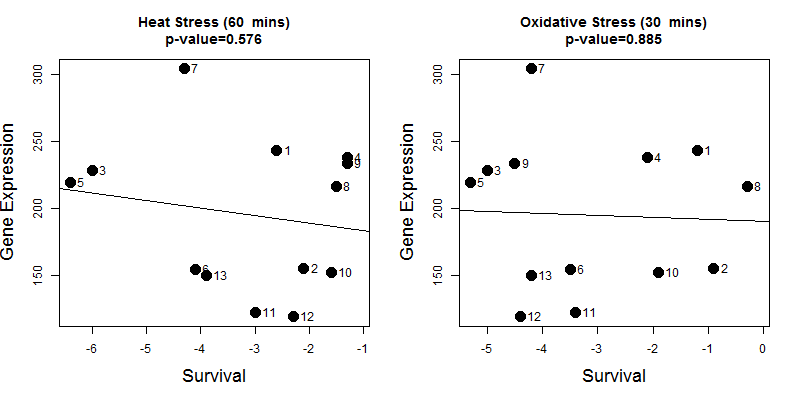

Supplement: S2 File — Expression levels of genes L75676 –L1889726 plotted against survival after 60 minutes heat and 30 min oxidative stress. Survival is expressed as the difference of log CFU/ml after stress and before stress. Numbers indicate fermentations as presented in Table 1. P-values above the plots indicate significance of correlation (assessed by a linear model). (ZIP) [file pone.0167944.s007.zip › S2_File/L112263_real_dat.png]

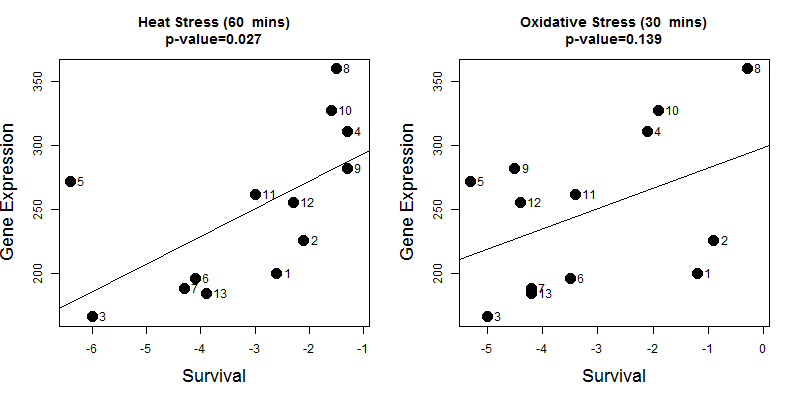

Supplement: S2 File — Expression levels of genes L75676 –L1889726 plotted against survival after 60 minutes heat and 30 min oxidative stress. Survival is expressed as the difference of log CFU/ml after stress and before stress. Numbers indicate fermentations as presented in Table 1. P-values above the plots indicate significance of correlation (assessed by a linear model). (ZIP) [file pone.0167944.s007.zip › S2_File/L112352_real_dat.png]

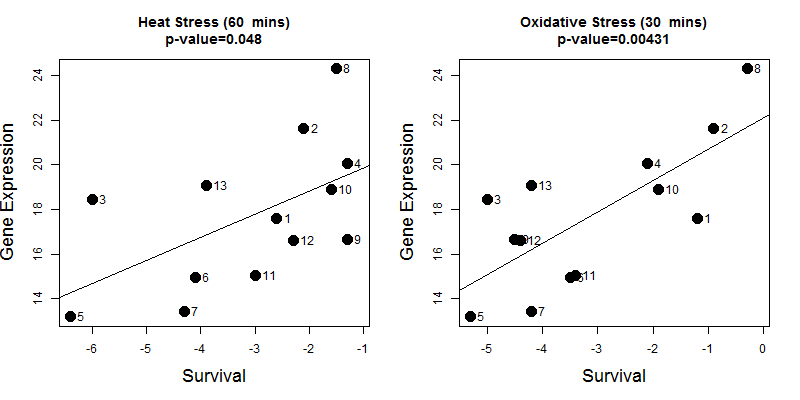

Supplement: S2 File — Expression levels of genes L75676 –L1889726 plotted against survival after 60 minutes heat and 30 min oxidative stress. Survival is expressed as the difference of log CFU/ml after stress and before stress. Numbers indicate fermentations as presented in Table 1. P-values above the plots indicate significance of correlation (assessed by a linear model). (ZIP) [file pone.0167944.s007.zip › S2_File/L112353_real_dat.png]

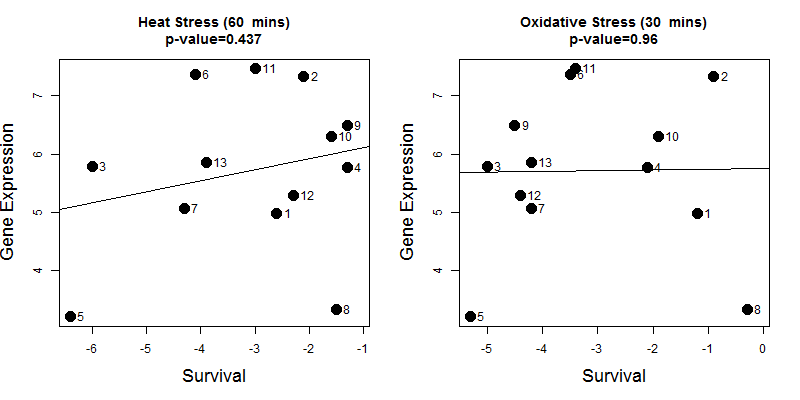

Supplement: S2 File — Expression levels of genes L75676 –L1889726 plotted against survival after 60 minutes heat and 30 min oxidative stress. Survival is expressed as the difference of log CFU/ml after stress and before stress. Numbers indicate fermentations as presented in Table 1. P-values above the plots indicate significance of correlation (assessed by a linear model). (ZIP) [file pone.0167944.s007.zip › S2_File/L112499_real_dat.png]

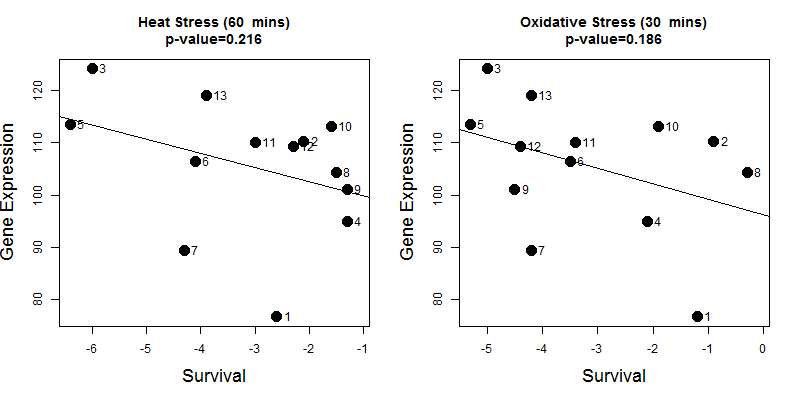

Supplement: S2 File — Expression levels of genes L75676 –L1889726 plotted against survival after 60 minutes heat and 30 min oxidative stress. Survival is expressed as the difference of log CFU/ml after stress and before stress. Numbers indicate fermentations as presented in Table 1. P-values above the plots indicate significance of correlation (assessed by a linear model). (ZIP) [file pone.0167944.s007.zip › S2_File/L112616_real_dat.png]

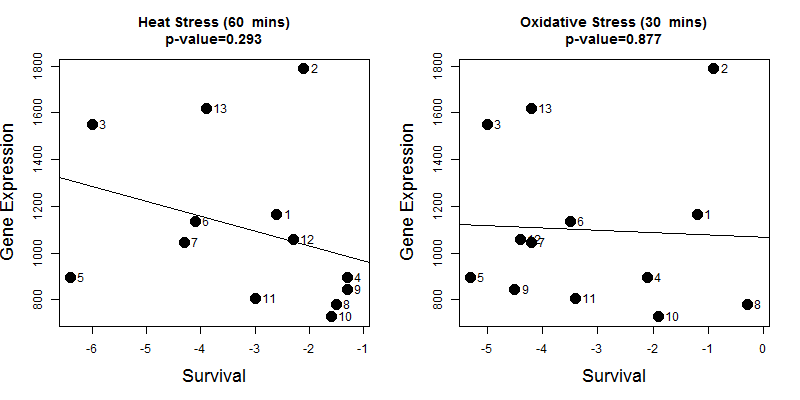

Supplement: S2 File — Expression levels of genes L75676 –L1889726 plotted against survival after 60 minutes heat and 30 min oxidative stress. Survival is expressed as the difference of log CFU/ml after stress and before stress. Numbers indicate fermentations as presented in Table 1. P-values above the plots indicate significance of correlation (assessed by a linear model). (ZIP) [file pone.0167944.s007.zip › S2_File/L112776_real_dat.png]

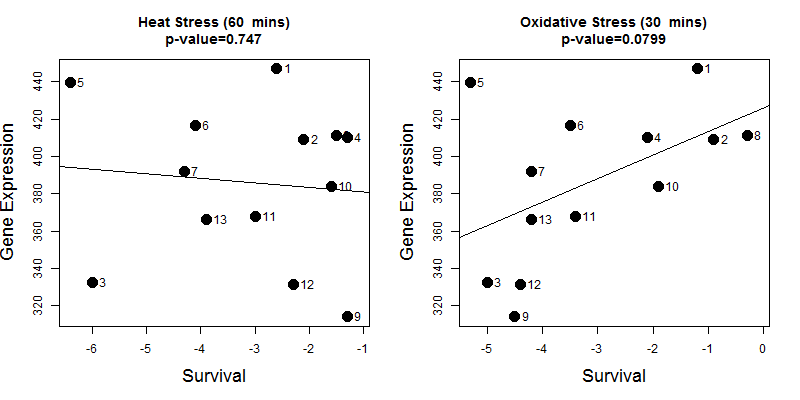

Supplement: S2 File — Expression levels of genes L75676 –L1889726 plotted against survival after 60 minutes heat and 30 min oxidative stress. Survival is expressed as the difference of log CFU/ml after stress and before stress. Numbers indicate fermentations as presented in Table 1. P-values above the plots indicate significance of correlation (assessed by a linear model). (ZIP) [file pone.0167944.s007.zip › S2_File/L112952_real_dat.png]

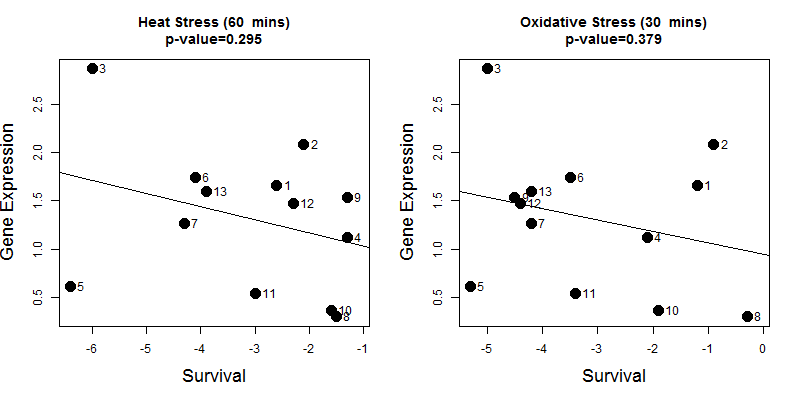

Supplement: S2 File — Expression levels of genes L75676 –L1889726 plotted against survival after 60 minutes heat and 30 min oxidative stress. Survival is expressed as the difference of log CFU/ml after stress and before stress. Numbers indicate fermentations as presented in Table 1. P-values above the plots indicate significance of correlation (assessed by a linear model). (ZIP) [file pone.0167944.s007.zip › S2_File/L113001_real_dat.png]

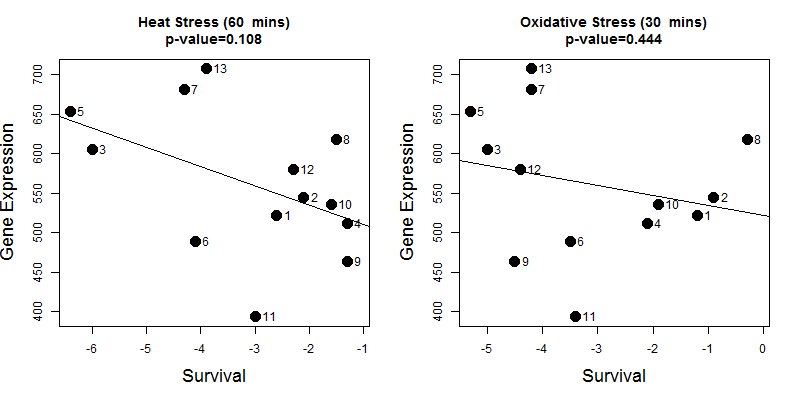

Supplement: S2 File — Expression levels of genes L75676 –L1889726 plotted against survival after 60 minutes heat and 30 min oxidative stress. Survival is expressed as the difference of log CFU/ml after stress and before stress. Numbers indicate fermentations as presented in Table 1. P-values above the plots indicate significance of correlation (assessed by a linear model). (ZIP) [file pone.0167944.s007.zip › S2_File/L113060_real_dat.png]

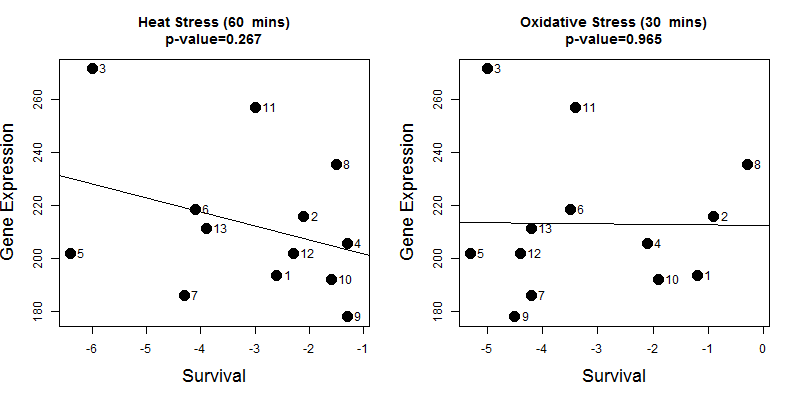

Supplement: S2 File — Expression levels of genes L75676 –L1889726 plotted against survival after 60 minutes heat and 30 min oxidative stress. Survival is expressed as the difference of log CFU/ml after stress and before stress. Numbers indicate fermentations as presented in Table 1. P-values above the plots indicate significance of correlation (assessed by a linear model). (ZIP) [file pone.0167944.s007.zip › S2_File/L113067_real_dat.png]

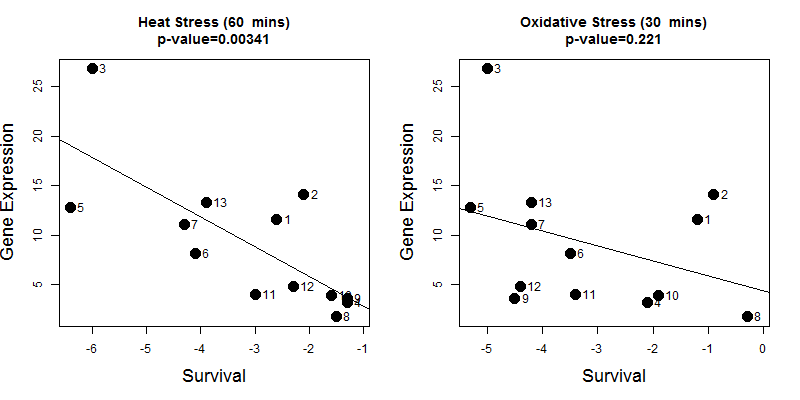

Supplement: S2 File — Expression levels of genes L75676 –L1889726 plotted against survival after 60 minutes heat and 30 min oxidative stress. Survival is expressed as the difference of log CFU/ml after stress and before stress. Numbers indicate fermentations as presented in Table 1. P-values above the plots indicate significance of correlation (assessed by a linear model). (ZIP) [file pone.0167944.s007.zip › S2_File/L113377_real_dat.png]

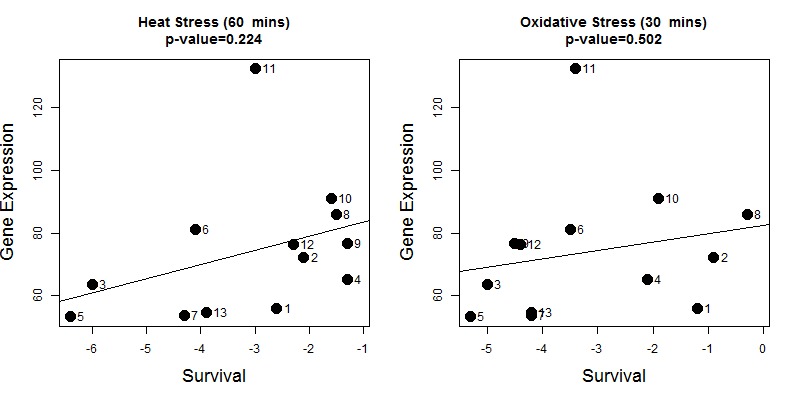

Supplement: S2 File — Expression levels of genes L75676 –L1889726 plotted against survival after 60 minutes heat and 30 min oxidative stress. Survival is expressed as the difference of log CFU/ml after stress and before stress. Numbers indicate fermentations as presented in Table 1. P-values above the plots indicate significance of correlation (assessed by a linear model). (ZIP) [file pone.0167944.s007.zip › S2_File/L113400_real_dat.png]

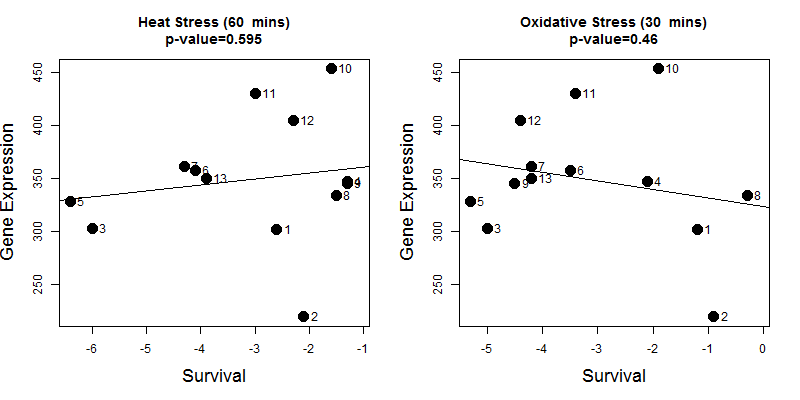

Supplement: S2 File — Expression levels of genes L75676 –L1889726 plotted against survival after 60 minutes heat and 30 min oxidative stress. Survival is expressed as the difference of log CFU/ml after stress and before stress. Numbers indicate fermentations as presented in Table 1. P-values above the plots indicate significance of correlation (assessed by a linear model). (ZIP) [file pone.0167944.s007.zip › S2_File/L113864_real_dat.png]

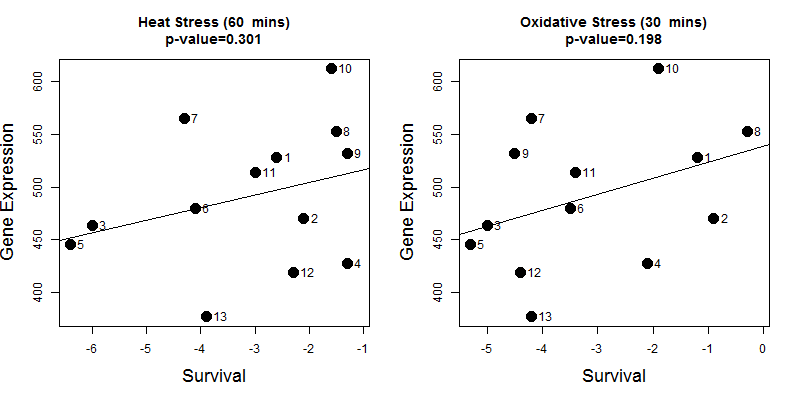

Supplement: S2 File — Expression levels of genes L75676 –L1889726 plotted against survival after 60 minutes heat and 30 min oxidative stress. Survival is expressed as the difference of log CFU/ml after stress and before stress. Numbers indicate fermentations as presented in Table 1. P-values above the plots indicate significance of correlation (assessed by a linear model). (ZIP) [file pone.0167944.s007.zip › S2_File/L113931_real_dat.png]

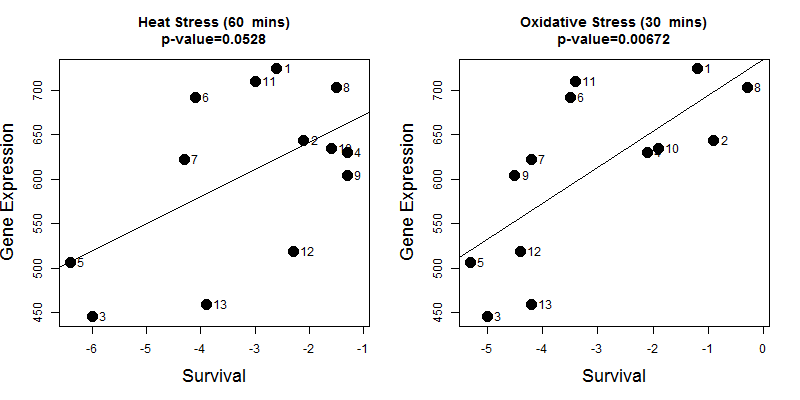

Supplement: S2 File — Expression levels of genes L75676 –L1889726 plotted against survival after 60 minutes heat and 30 min oxidative stress. Survival is expressed as the difference of log CFU/ml after stress and before stress. Numbers indicate fermentations as presented in Table 1. P-values above the plots indicate significance of correlation (assessed by a linear model). (ZIP) [file pone.0167944.s007.zip › S2_File/L113994_real_dat.png]

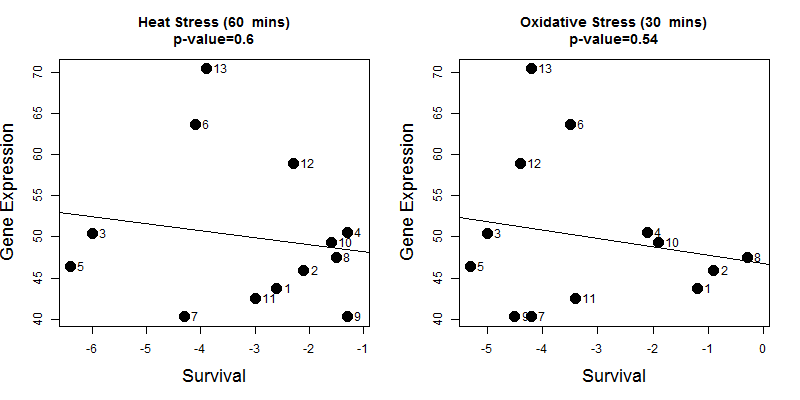

Supplement: S2 File — Expression levels of genes L75676 –L1889726 plotted against survival after 60 minutes heat and 30 min oxidative stress. Survival is expressed as the difference of log CFU/ml after stress and before stress. Numbers indicate fermentations as presented in Table 1. P-values above the plots indicate significance of correlation (assessed by a linear model). (ZIP) [file pone.0167944.s007.zip › S2_File/L114054_real_dat.png]

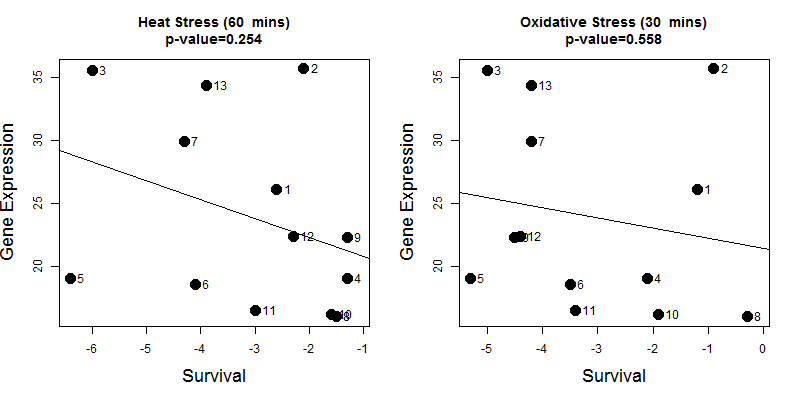

Supplement: S2 File — Expression levels of genes L75676 –L1889726 plotted against survival after 60 minutes heat and 30 min oxidative stress. Survival is expressed as the difference of log CFU/ml after stress and before stress. Numbers indicate fermentations as presented in Table 1. P-values above the plots indicate significance of correlation (assessed by a linear model). (ZIP) [file pone.0167944.s007.zip › S2_File/L114078_real_dat.png]

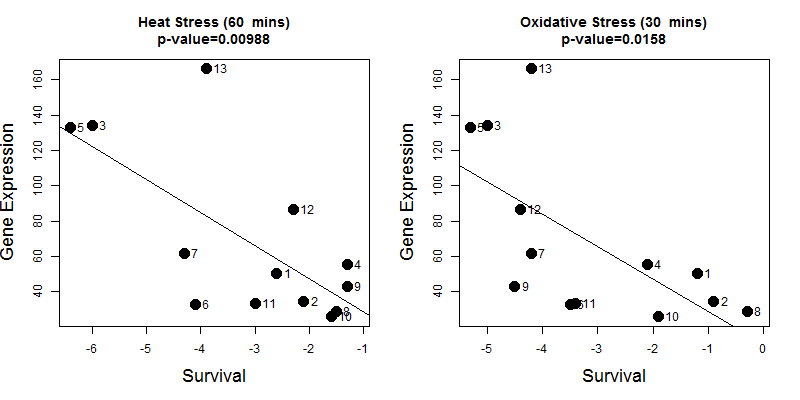

Supplement: S2 File — Expression levels of genes L75676 –L1889726 plotted against survival after 60 minutes heat and 30 min oxidative stress. Survival is expressed as the difference of log CFU/ml after stress and before stress. Numbers indicate fermentations as presented in Table 1. P-values above the plots indicate significance of correlation (assessed by a linear model). (ZIP) [file pone.0167944.s007.zip › S2_File/L114099_real_dat.png]

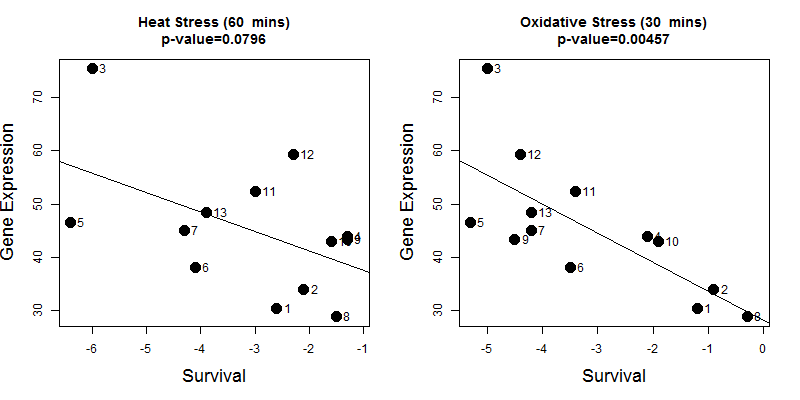

Supplement: S2 File — Expression levels of genes L75676 –L1889726 plotted against survival after 60 minutes heat and 30 min oxidative stress. Survival is expressed as the difference of log CFU/ml after stress and before stress. Numbers indicate fermentations as presented in Table 1. P-values above the plots indicate significance of correlation (assessed by a linear model). (ZIP) [file pone.0167944.s007.zip › S2_File/L114325_real_dat.png]

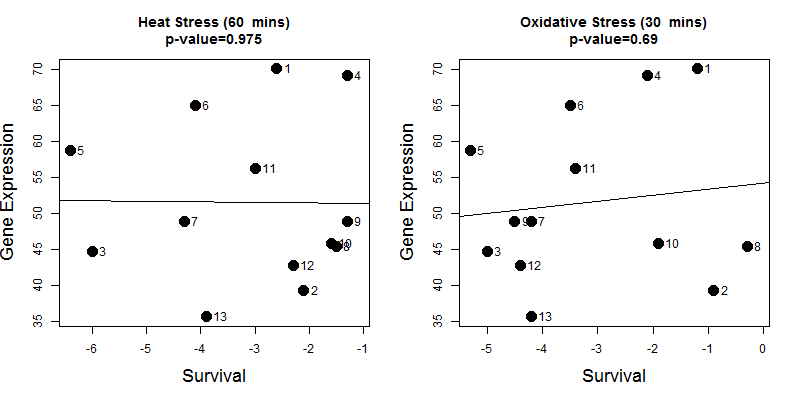

Supplement: S2 File — Expression levels of genes L75676 –L1889726 plotted against survival after 60 minutes heat and 30 min oxidative stress. Survival is expressed as the difference of log CFU/ml after stress and before stress. Numbers indicate fermentations as presented in Table 1. P-values above the plots indicate significance of correlation (assessed by a linear model). (ZIP) [file pone.0167944.s007.zip › S2_File/L114363_real_dat.png]

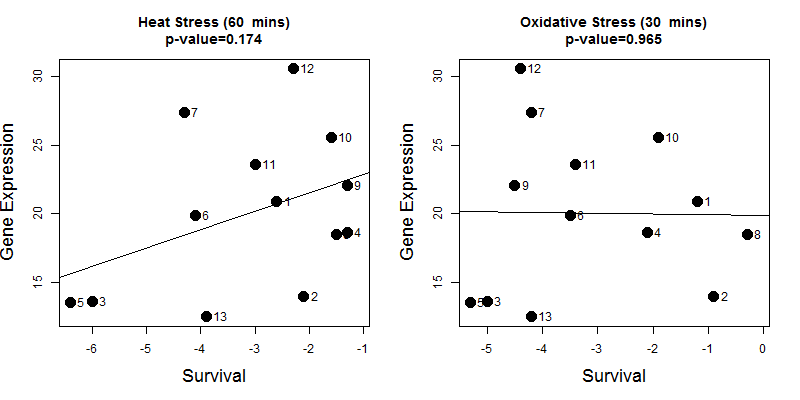

Supplement: S2 File — Expression levels of genes L75676 –L1889726 plotted against survival after 60 minutes heat and 30 min oxidative stress. Survival is expressed as the difference of log CFU/ml after stress and before stress. Numbers indicate fermentations as presented in Table 1. P-values above the plots indicate significance of correlation (assessed by a linear model). (ZIP) [file pone.0167944.s007.zip › S2_File/L114370_real_dat.png]

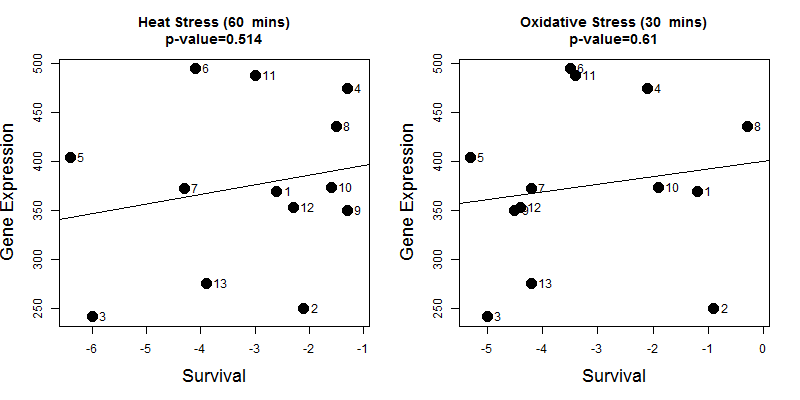

Supplement: S2 File — Expression levels of genes L75676 –L1889726 plotted against survival after 60 minutes heat and 30 min oxidative stress. Survival is expressed as the difference of log CFU/ml after stress and before stress. Numbers indicate fermentations as presented in Table 1. P-values above the plots indicate significance of correlation (assessed by a linear model). (ZIP) [file pone.0167944.s007.zip › S2_File/L114402_real_dat.png]

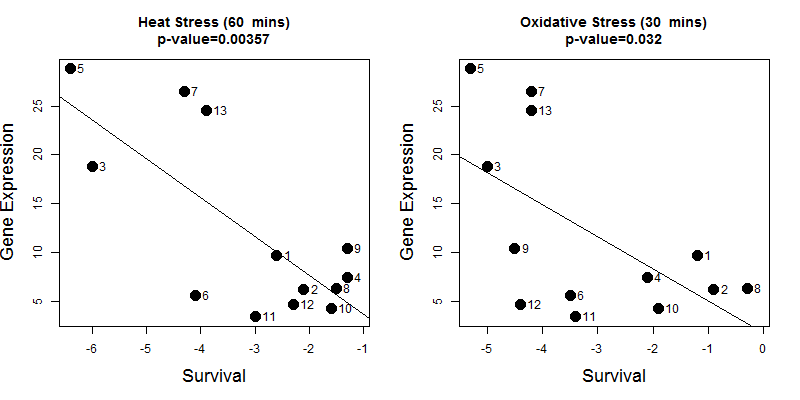

Supplement: S2 File — Expression levels of genes L75676 –L1889726 plotted against survival after 60 minutes heat and 30 min oxidative stress. Survival is expressed as the difference of log CFU/ml after stress and before stress. Numbers indicate fermentations as presented in Table 1. P-values above the plots indicate significance of correlation (assessed by a linear model). (ZIP) [file pone.0167944.s007.zip › S2_File/L114419_real_dat.png]

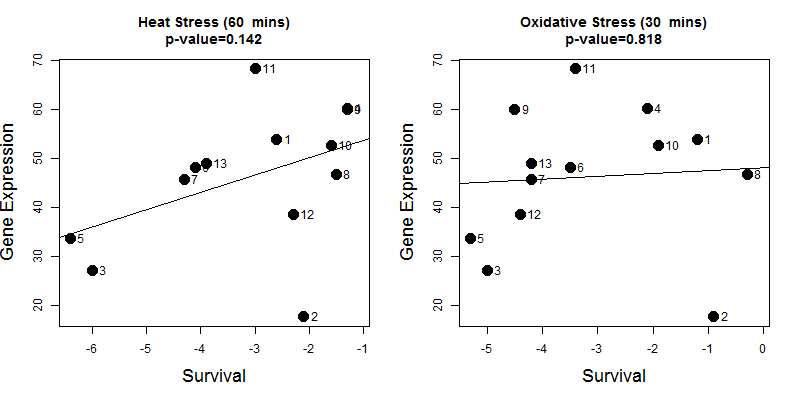

Supplement: S2 File — Expression levels of genes L75676 –L1889726 plotted against survival after 60 minutes heat and 30 min oxidative stress. Survival is expressed as the difference of log CFU/ml after stress and before stress. Numbers indicate fermentations as presented in Table 1. P-values above the plots indicate significance of correlation (assessed by a linear model). (ZIP) [file pone.0167944.s007.zip › S2_File/L114632_real_dat.png]

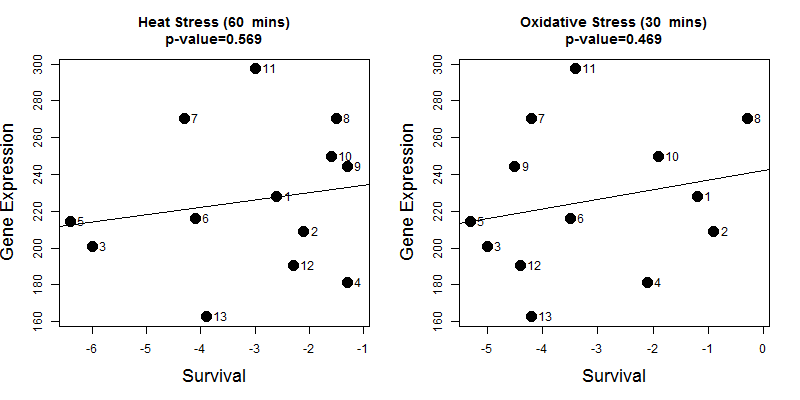

Supplement: S2 File — Expression levels of genes L75676 –L1889726 plotted against survival after 60 minutes heat and 30 min oxidative stress. Survival is expressed as the difference of log CFU/ml after stress and before stress. Numbers indicate fermentations as presented in Table 1. P-values above the plots indicate significance of correlation (assessed by a linear model). (ZIP) [file pone.0167944.s007.zip › S2_File/L114717_real_dat.png]

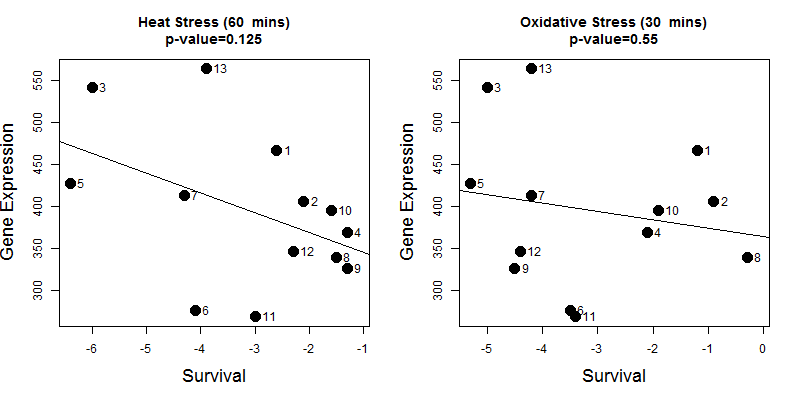

Supplement: S2 File — Expression levels of genes L75676 –L1889726 plotted against survival after 60 minutes heat and 30 min oxidative stress. Survival is expressed as the difference of log CFU/ml after stress and before stress. Numbers indicate fermentations as presented in Table 1. P-values above the plots indicate significance of correlation (assessed by a linear model). (ZIP) [file pone.0167944.s007.zip › S2_File/L114740_real_dat.png]

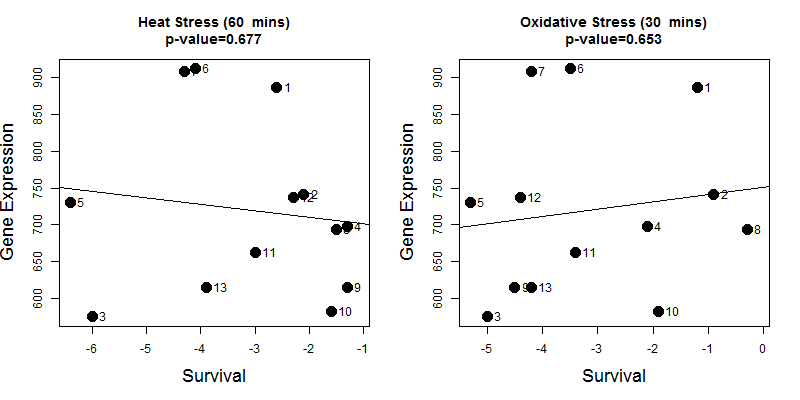

Supplement: S2 File — Expression levels of genes L75676 –L1889726 plotted against survival after 60 minutes heat and 30 min oxidative stress. Survival is expressed as the difference of log CFU/ml after stress and before stress. Numbers indicate fermentations as presented in Table 1. P-values above the plots indicate significance of correlation (assessed by a linear model). (ZIP) [file pone.0167944.s007.zip › S2_File/L114827_real_dat.png]

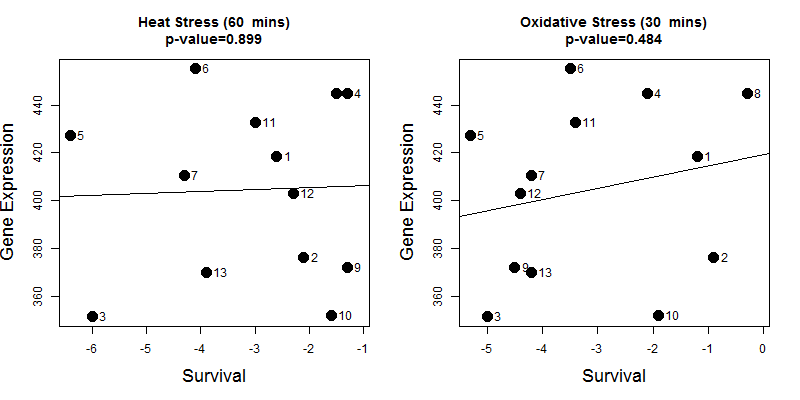

Supplement: S2 File — Expression levels of genes L75676 –L1889726 plotted against survival after 60 minutes heat and 30 min oxidative stress. Survival is expressed as the difference of log CFU/ml after stress and before stress. Numbers indicate fermentations as presented in Table 1. P-values above the plots indicate significance of correlation (assessed by a linear model). (ZIP) [file pone.0167944.s007.zip › S2_File/L114884_real_dat.png]

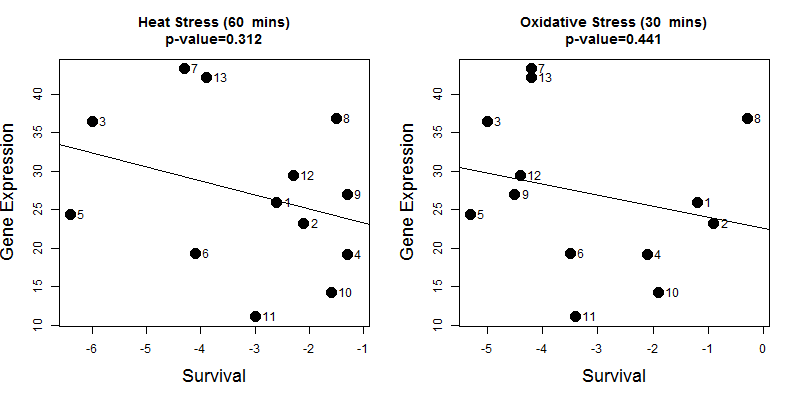

Supplement: S2 File — Expression levels of genes L75676 –L1889726 plotted against survival after 60 minutes heat and 30 min oxidative stress. Survival is expressed as the difference of log CFU/ml after stress and before stress. Numbers indicate fermentations as presented in Table 1. P-values above the plots indicate significance of correlation (assessed by a linear model). (ZIP) [file pone.0167944.s007.zip › S2_File/L115025_real_dat.png]

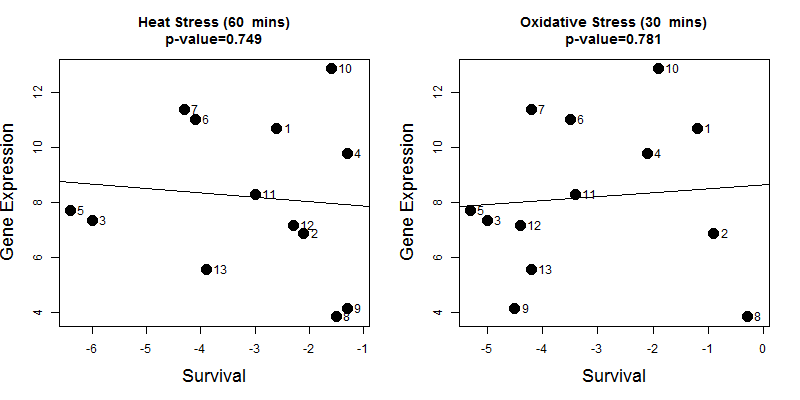

Supplement: S2 File — Expression levels of genes L75676 –L1889726 plotted against survival after 60 minutes heat and 30 min oxidative stress. Survival is expressed as the difference of log CFU/ml after stress and before stress. Numbers indicate fermentations as presented in Table 1. P-values above the plots indicate significance of correlation (assessed by a linear model). (ZIP) [file pone.0167944.s007.zip › S2_File/L115107_real_dat.png]

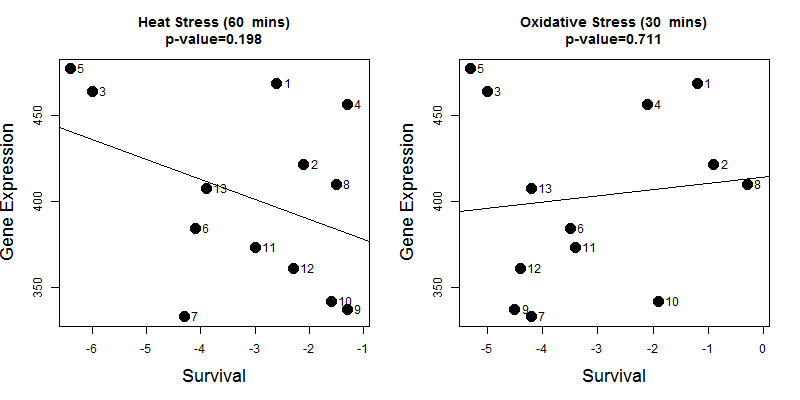

Supplement: S2 File — Expression levels of genes L75676 –L1889726 plotted against survival after 60 minutes heat and 30 min oxidative stress. Survival is expressed as the difference of log CFU/ml after stress and before stress. Numbers indicate fermentations as presented in Table 1. P-values above the plots indicate significance of correlation (assessed by a linear model). (ZIP) [file pone.0167944.s007.zip › S2_File/L115113_real_dat.png]

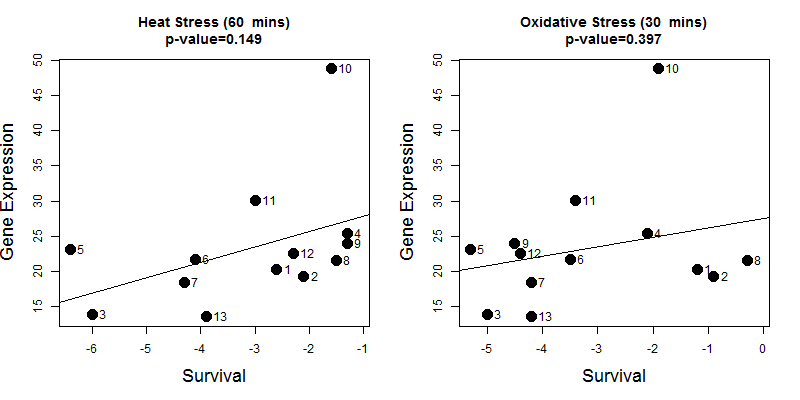

Supplement: S2 File — Expression levels of genes L75676 –L1889726 plotted against survival after 60 minutes heat and 30 min oxidative stress. Survival is expressed as the difference of log CFU/ml after stress and before stress. Numbers indicate fermentations as presented in Table 1. P-values above the plots indicate significance of correlation (assessed by a linear model). (ZIP) [file pone.0167944.s007.zip › S2_File/L115265_real_dat.png]

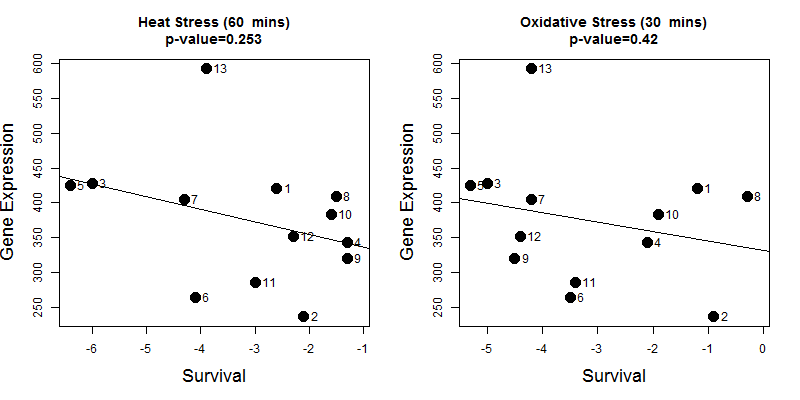

Supplement: S2 File — Expression levels of genes L75676 –L1889726 plotted against survival after 60 minutes heat and 30 min oxidative stress. Survival is expressed as the difference of log CFU/ml after stress and before stress. Numbers indicate fermentations as presented in Table 1. P-values above the plots indicate significance of correlation (assessed by a linear model). (ZIP) [file pone.0167944.s007.zip › S2_File/L115302_real_dat.png]

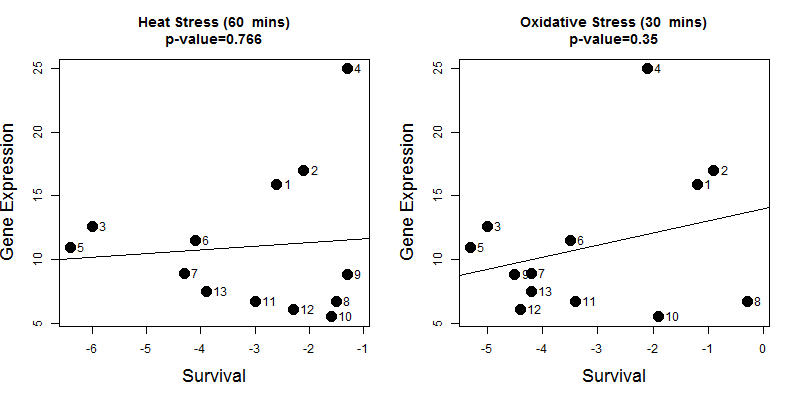

Supplement: S2 File — Expression levels of genes L75676 –L1889726 plotted against survival after 60 minutes heat and 30 min oxidative stress. Survival is expressed as the difference of log CFU/ml after stress and before stress. Numbers indicate fermentations as presented in Table 1. P-values above the plots indicate significance of correlation (assessed by a linear model). (ZIP) [file pone.0167944.s007.zip › S2_File/L115362_real_dat.png]

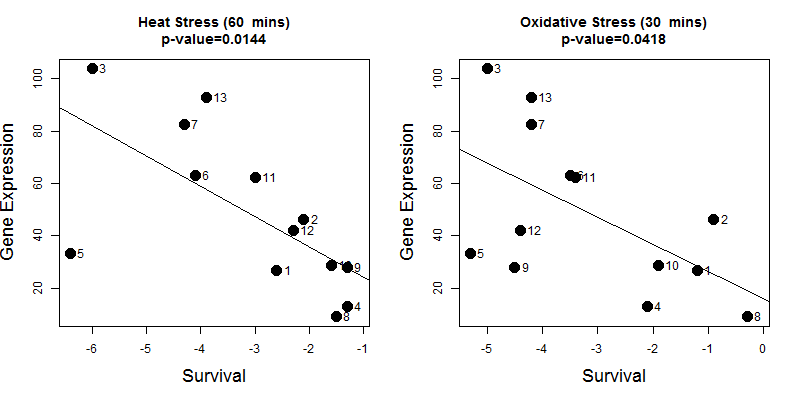

Supplement: S2 File — Expression levels of genes L75676 –L1889726 plotted against survival after 60 minutes heat and 30 min oxidative stress. Survival is expressed as the difference of log CFU/ml after stress and before stress. Numbers indicate fermentations as presented in Table 1. P-values above the plots indicate significance of correlation (assessed by a linear model). (ZIP) [file pone.0167944.s007.zip › S2_File/L115500_real_dat.png]

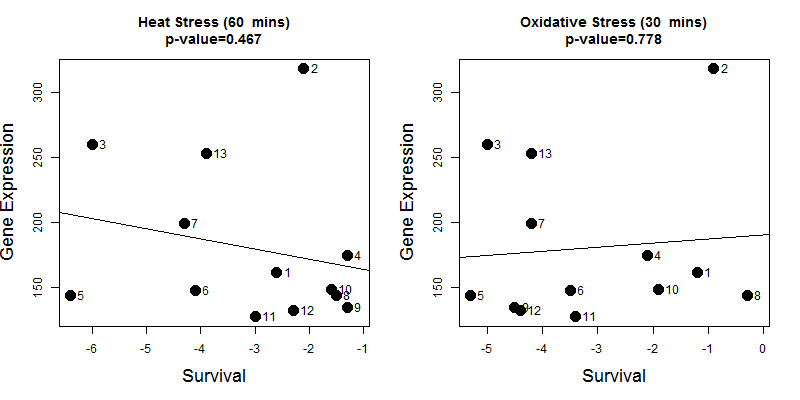

Supplement: S2 File — Expression levels of genes L75676 –L1889726 plotted against survival after 60 minutes heat and 30 min oxidative stress. Survival is expressed as the difference of log CFU/ml after stress and before stress. Numbers indicate fermentations as presented in Table 1. P-values above the plots indicate significance of correlation (assessed by a linear model). (ZIP) [file pone.0167944.s007.zip › S2_File/L115551_real_dat.png]

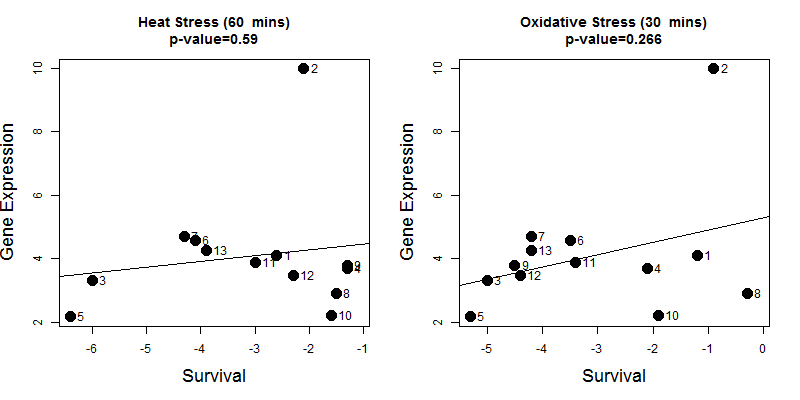

Supplement: S2 File — Expression levels of genes L75676 –L1889726 plotted against survival after 60 minutes heat and 30 min oxidative stress. Survival is expressed as the difference of log CFU/ml after stress and before stress. Numbers indicate fermentations as presented in Table 1. P-values above the plots indicate significance of correlation (assessed by a linear model). (ZIP) [file pone.0167944.s007.zip › S2_File/L115661_real_dat.png]

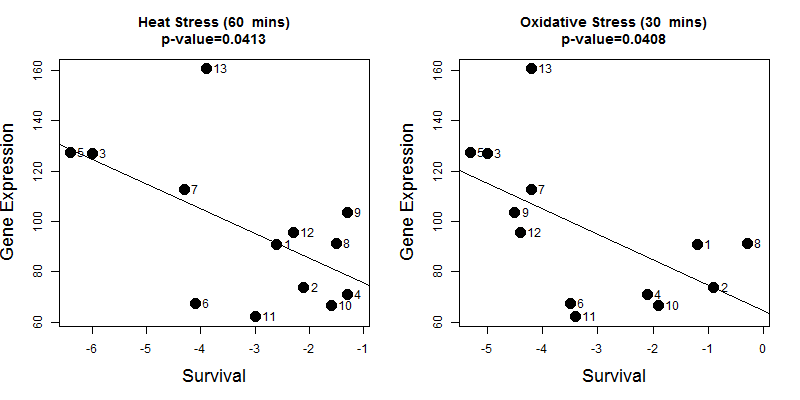

Supplement: S2 File — Expression levels of genes L75676 –L1889726 plotted against survival after 60 minutes heat and 30 min oxidative stress. Survival is expressed as the difference of log CFU/ml after stress and before stress. Numbers indicate fermentations as presented in Table 1. P-values above the plots indicate significance of correlation (assessed by a linear model). (ZIP) [file pone.0167944.s007.zip › S2_File/L115671_real_dat.png]

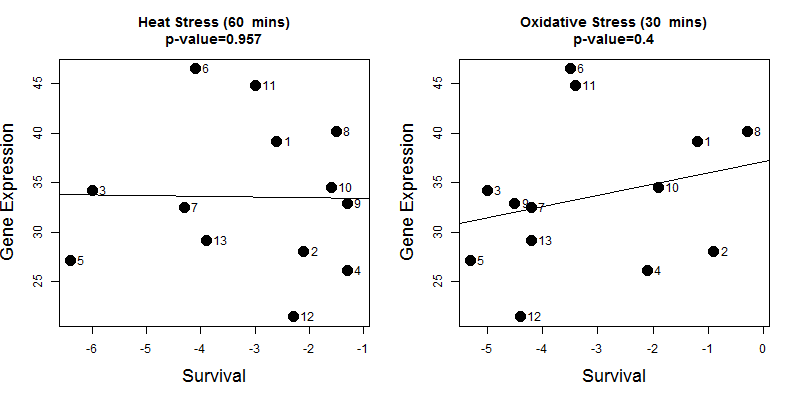

Supplement: S2 File — Expression levels of genes L75676 –L1889726 plotted against survival after 60 minutes heat and 30 min oxidative stress. Survival is expressed as the difference of log CFU/ml after stress and before stress. Numbers indicate fermentations as presented in Table 1. P-values above the plots indicate significance of correlation (assessed by a linear model). (ZIP) [file pone.0167944.s007.zip › S2_File/L115706_real_dat.png]

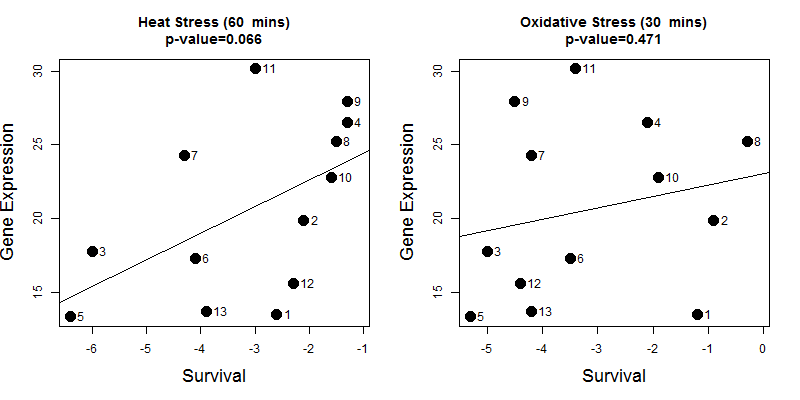

Supplement: S2 File — Expression levels of genes L75676 –L1889726 plotted against survival after 60 minutes heat and 30 min oxidative stress. Survival is expressed as the difference of log CFU/ml after stress and before stress. Numbers indicate fermentations as presented in Table 1. P-values above the plots indicate significance of correlation (assessed by a linear model). (ZIP) [file pone.0167944.s007.zip › S2_File/L115789_real_dat.png]

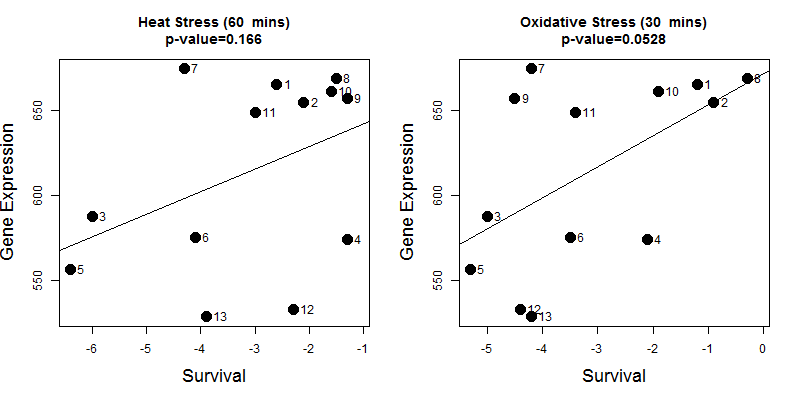

Supplement: S2 File — Expression levels of genes L75676 –L1889726 plotted against survival after 60 minutes heat and 30 min oxidative stress. Survival is expressed as the difference of log CFU/ml after stress and before stress. Numbers indicate fermentations as presented in Table 1. P-values above the plots indicate significance of correlation (assessed by a linear model). (ZIP) [file pone.0167944.s007.zip › S2_File/L115968_real_dat.png]

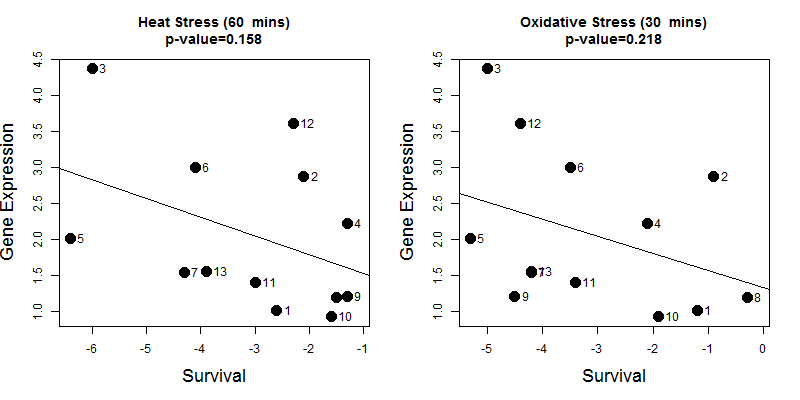

Supplement: S2 File — Expression levels of genes L75676 –L1889726 plotted against survival after 60 minutes heat and 30 min oxidative stress. Survival is expressed as the difference of log CFU/ml after stress and before stress. Numbers indicate fermentations as presented in Table 1. P-values above the plots indicate significance of correlation (assessed by a linear model). (ZIP) [file pone.0167944.s007.zip › S2_File/L116178_real_dat.png]

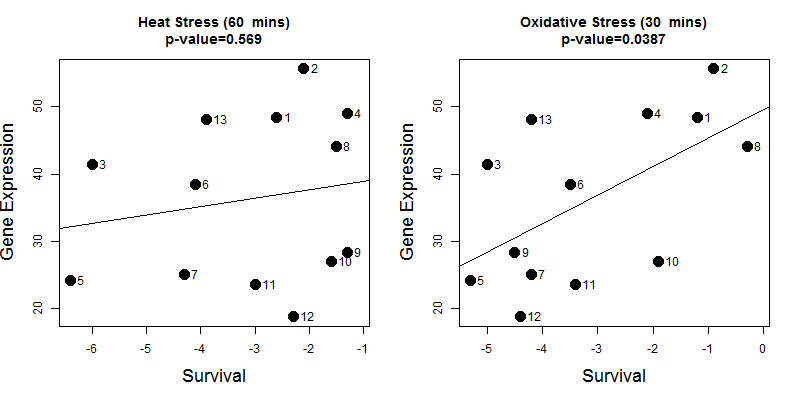

Supplement: S2 File — Expression levels of genes L75676 –L1889726 plotted against survival after 60 minutes heat and 30 min oxidative stress. Survival is expressed as the difference of log CFU/ml after stress and before stress. Numbers indicate fermentations as presented in Table 1. P-values above the plots indicate significance of correlation (assessed by a linear model). (ZIP) [file pone.0167944.s007.zip › S2_File/L116212_real_dat.png]

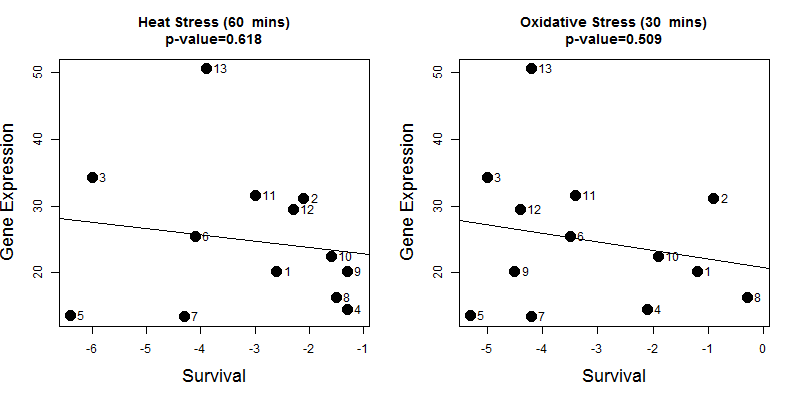

Supplement: S2 File — Expression levels of genes L75676 –L1889726 plotted against survival after 60 minutes heat and 30 min oxidative stress. Survival is expressed as the difference of log CFU/ml after stress and before stress. Numbers indicate fermentations as presented in Table 1. P-values above the plots indicate significance of correlation (assessed by a linear model). (ZIP) [file pone.0167944.s007.zip › S2_File/L116216_real_dat.png]

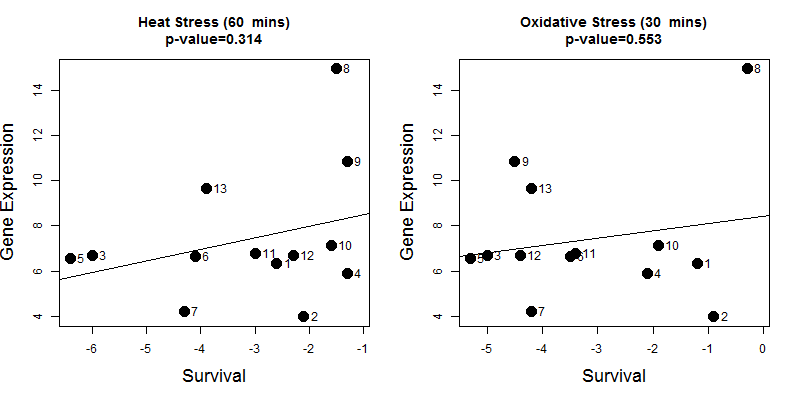

Supplement: S2 File — Expression levels of genes L75676 –L1889726 plotted against survival after 60 minutes heat and 30 min oxidative stress. Survival is expressed as the difference of log CFU/ml after stress and before stress. Numbers indicate fermentations as presented in Table 1. P-values above the plots indicate significance of correlation (assessed by a linear model). (ZIP) [file pone.0167944.s007.zip › S2_File/L116299_real_dat.png]

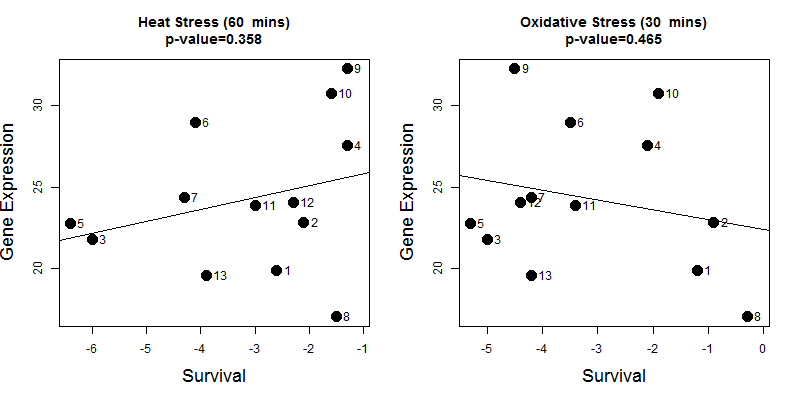

Supplement: S2 File — Expression levels of genes L75676 –L1889726 plotted against survival after 60 minutes heat and 30 min oxidative stress. Survival is expressed as the difference of log CFU/ml after stress and before stress. Numbers indicate fermentations as presented in Table 1. P-values above the plots indicate significance of correlation (assessed by a linear model). (ZIP) [file pone.0167944.s007.zip › S2_File/L116532_real_dat.png]

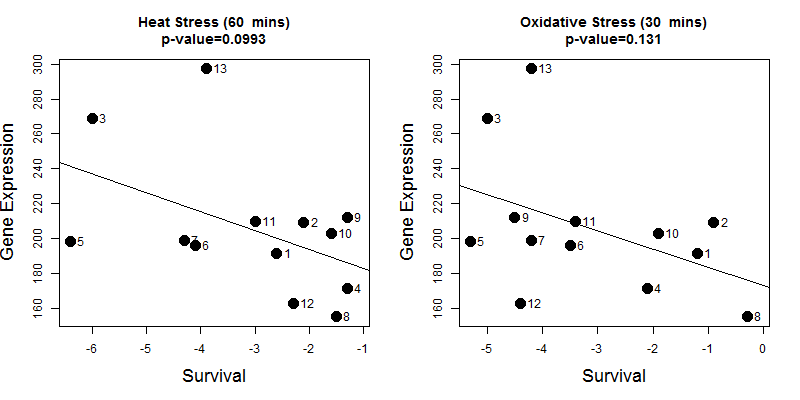

Supplement: S2 File — Expression levels of genes L75676 –L1889726 plotted against survival after 60 minutes heat and 30 min oxidative stress. Survival is expressed as the difference of log CFU/ml after stress and before stress. Numbers indicate fermentations as presented in Table 1. P-values above the plots indicate significance of correlation (assessed by a linear model). (ZIP) [file pone.0167944.s007.zip › S2_File/L116756_real_dat.png]

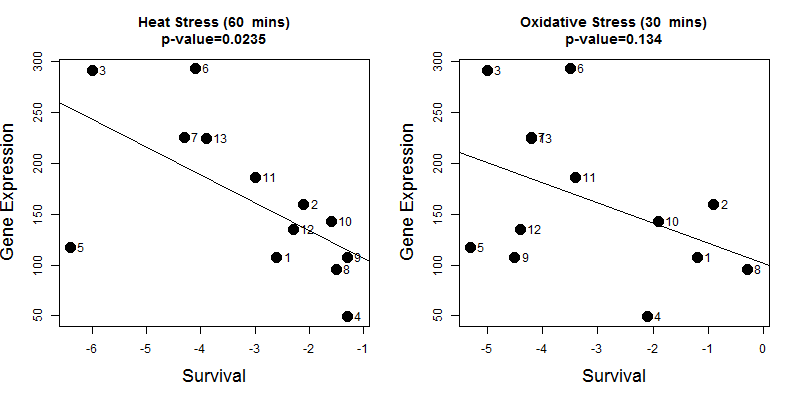

Supplement: S2 File — Expression levels of genes L75676 –L1889726 plotted against survival after 60 minutes heat and 30 min oxidative stress. Survival is expressed as the difference of log CFU/ml after stress and before stress. Numbers indicate fermentations as presented in Table 1. P-values above the plots indicate significance of correlation (assessed by a linear model). (ZIP) [file pone.0167944.s007.zip › S2_File/L117074_real_dat.png]

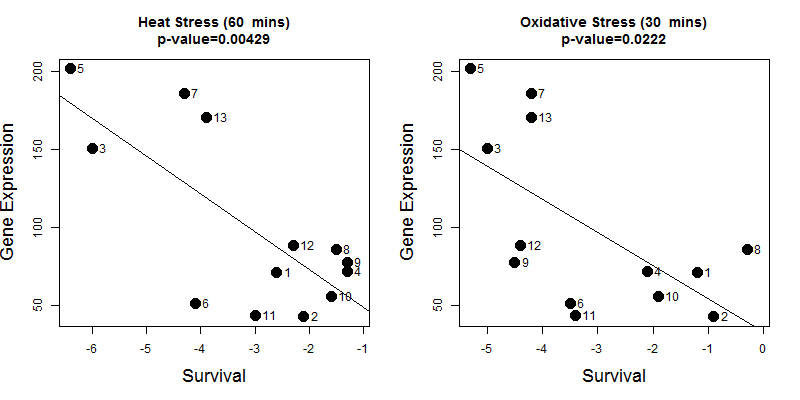

Supplement: S2 File — Expression levels of genes L75676 –L1889726 plotted against survival after 60 minutes heat and 30 min oxidative stress. Survival is expressed as the difference of log CFU/ml after stress and before stress. Numbers indicate fermentations as presented in Table 1. P-values above the plots indicate significance of correlation (assessed by a linear model). (ZIP) [file pone.0167944.s007.zip › S2_File/L117090_real_dat.png]

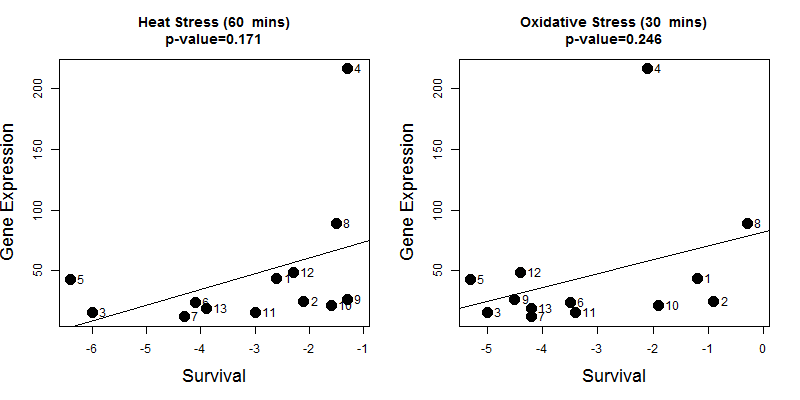

Supplement: S2 File — Expression levels of genes L75676 –L1889726 plotted against survival after 60 minutes heat and 30 min oxidative stress. Survival is expressed as the difference of log CFU/ml after stress and before stress. Numbers indicate fermentations as presented in Table 1. P-values above the plots indicate significance of correlation (assessed by a linear model). (ZIP) [file pone.0167944.s007.zip › S2_File/L117145_real_dat.png]

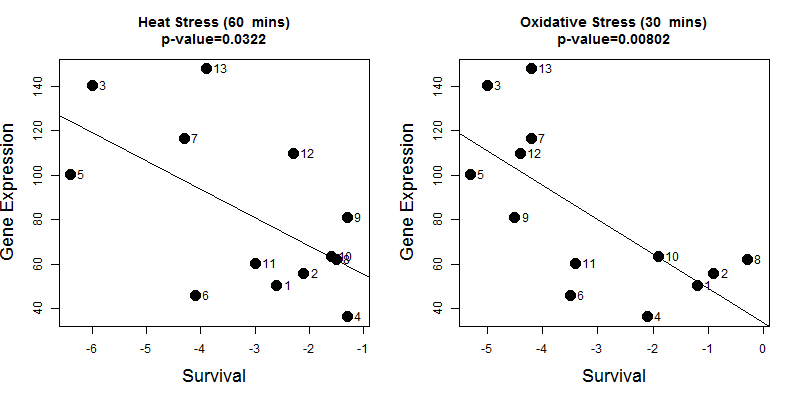

Supplement: S2 File — Expression levels of genes L75676 –L1889726 plotted against survival after 60 minutes heat and 30 min oxidative stress. Survival is expressed as the difference of log CFU/ml after stress and before stress. Numbers indicate fermentations as presented in Table 1. P-values above the plots indicate significance of correlation (assessed by a linear model). (ZIP) [file pone.0167944.s007.zip › S2_File/L117205_real_dat.png]

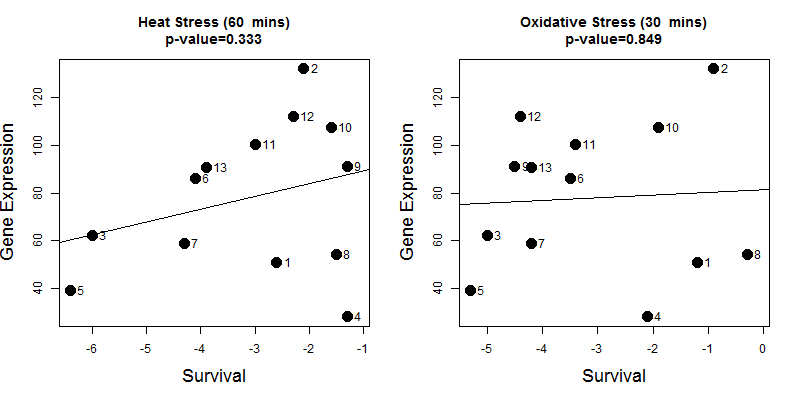

Supplement: S2 File — Expression levels of genes L75676 –L1889726 plotted against survival after 60 minutes heat and 30 min oxidative stress. Survival is expressed as the difference of log CFU/ml after stress and before stress. Numbers indicate fermentations as presented in Table 1. P-values above the plots indicate significance of correlation (assessed by a linear model). (ZIP) [file pone.0167944.s007.zip › S2_File/L117444_real_dat.png]

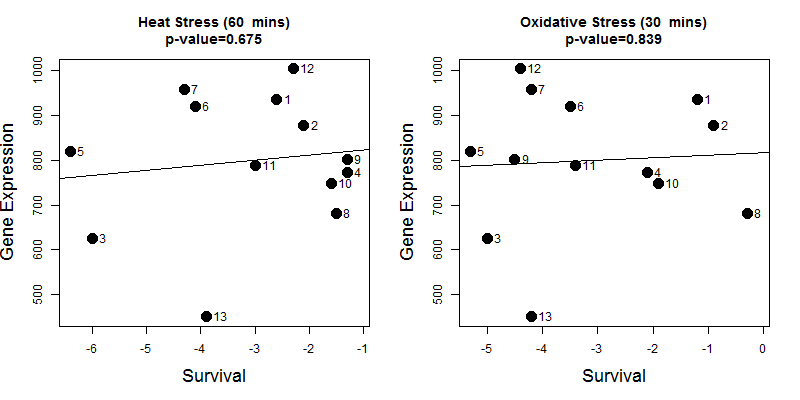

Supplement: S2 File — Expression levels of genes L75676 –L1889726 plotted against survival after 60 minutes heat and 30 min oxidative stress. Survival is expressed as the difference of log CFU/ml after stress and before stress. Numbers indicate fermentations as presented in Table 1. P-values above the plots indicate significance of correlation (assessed by a linear model). (ZIP) [file pone.0167944.s007.zip › S2_File/L117685_real_dat.png]

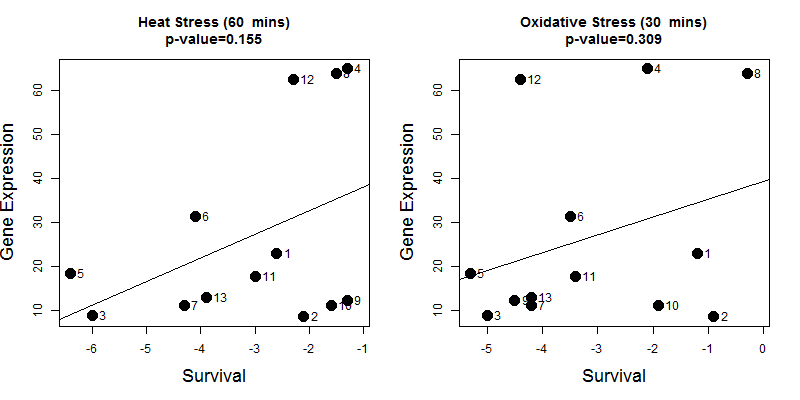

Supplement: S2 File — Expression levels of genes L75676 –L1889726 plotted against survival after 60 minutes heat and 30 min oxidative stress. Survival is expressed as the difference of log CFU/ml after stress and before stress. Numbers indicate fermentations as presented in Table 1. P-values above the plots indicate significance of correlation (assessed by a linear model). (ZIP) [file pone.0167944.s007.zip › S2_File/L117718_real_dat.png]

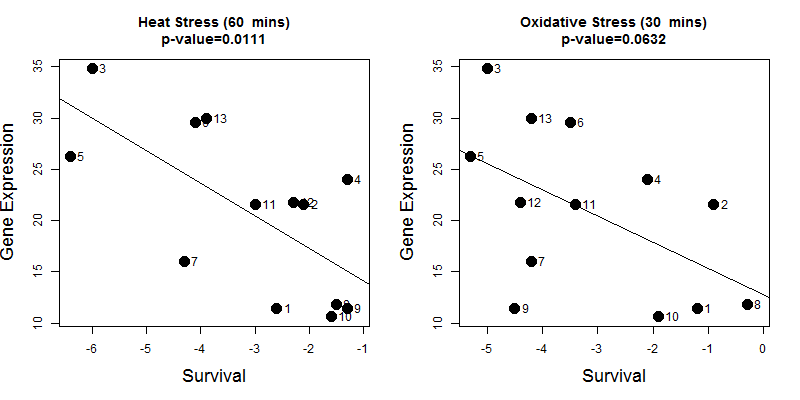

Supplement: S2 File — Expression levels of genes L75676 –L1889726 plotted against survival after 60 minutes heat and 30 min oxidative stress. Survival is expressed as the difference of log CFU/ml after stress and before stress. Numbers indicate fermentations as presented in Table 1. P-values above the plots indicate significance of correlation (assessed by a linear model). (ZIP) [file pone.0167944.s007.zip › S2_File/L117821_real_dat.png]

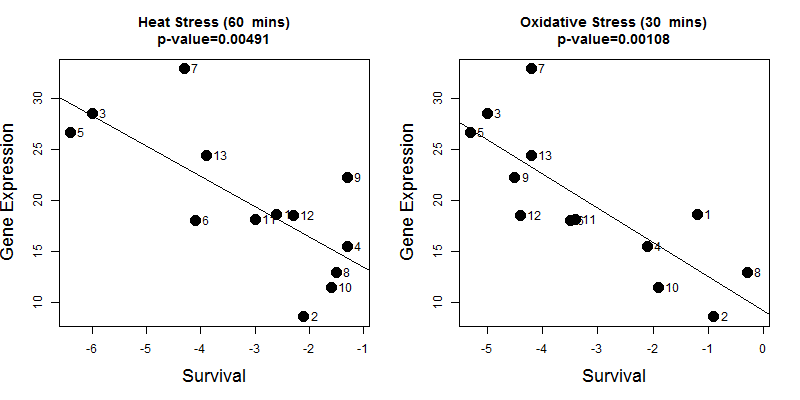

Supplement: S2 File — Expression levels of genes L75676 –L1889726 plotted against survival after 60 minutes heat and 30 min oxidative stress. Survival is expressed as the difference of log CFU/ml after stress and before stress. Numbers indicate fermentations as presented in Table 1. P-values above the plots indicate significance of correlation (assessed by a linear model). (ZIP) [file pone.0167944.s007.zip › S2_File/L117867_real_dat.png]

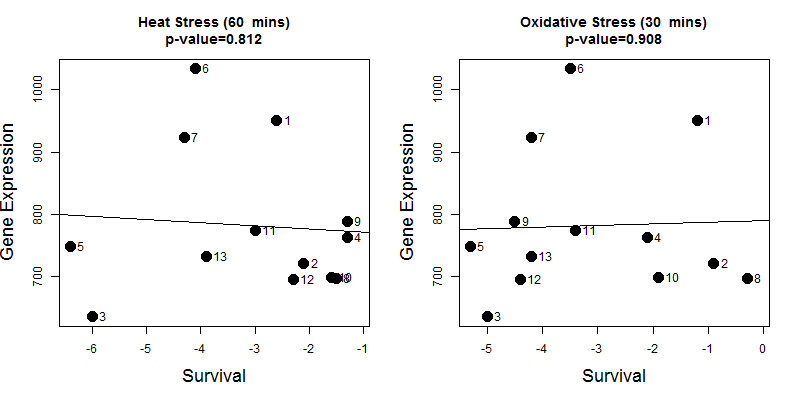

Supplement: S2 File — Expression levels of genes L75676 –L1889726 plotted against survival after 60 minutes heat and 30 min oxidative stress. Survival is expressed as the difference of log CFU/ml after stress and before stress. Numbers indicate fermentations as presented in Table 1. P-values above the plots indicate significance of correlation (assessed by a linear model). (ZIP) [file pone.0167944.s007.zip › S2_File/L118079_real_dat.png]

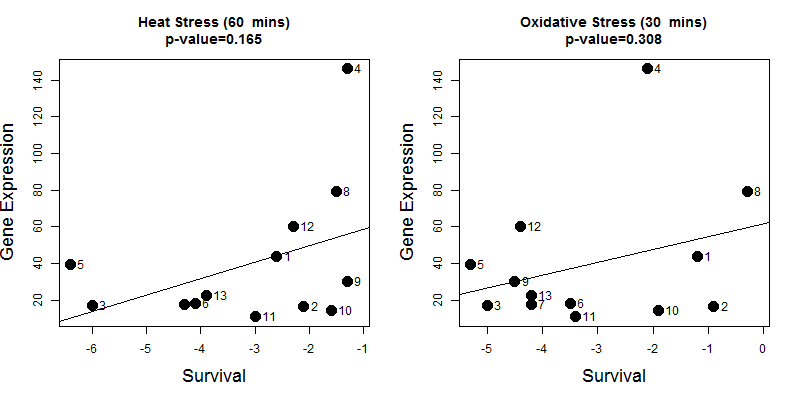

Supplement: S2 File — Expression levels of genes L75676 –L1889726 plotted against survival after 60 minutes heat and 30 min oxidative stress. Survival is expressed as the difference of log CFU/ml after stress and before stress. Numbers indicate fermentations as presented in Table 1. P-values above the plots indicate significance of correlation (assessed by a linear model). (ZIP) [file pone.0167944.s007.zip › S2_File/L118271_real_dat.png]

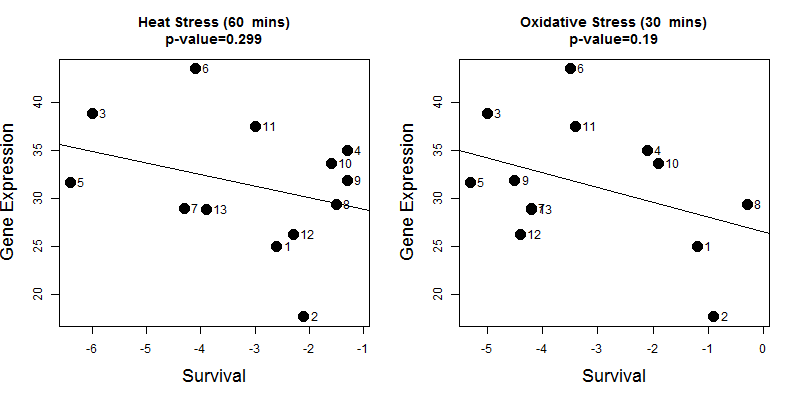

Supplement: S2 File — Expression levels of genes L75676 –L1889726 plotted against survival after 60 minutes heat and 30 min oxidative stress. Survival is expressed as the difference of log CFU/ml after stress and before stress. Numbers indicate fermentations as presented in Table 1. P-values above the plots indicate significance of correlation (assessed by a linear model). (ZIP) [file pone.0167944.s007.zip › S2_File/L118350_real_dat.png]

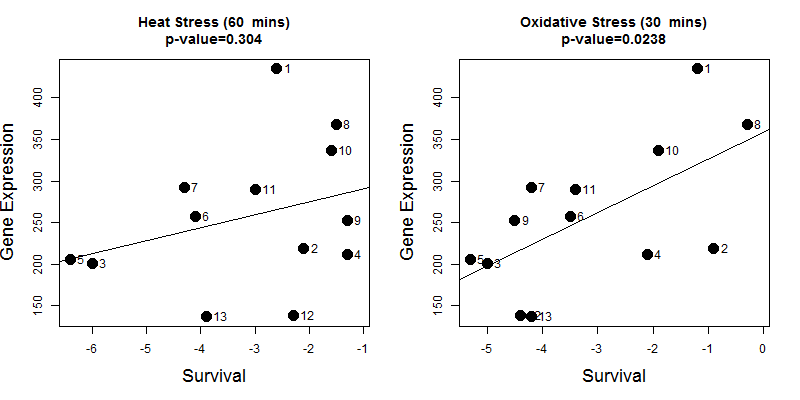

Supplement: S2 File — Expression levels of genes L75676 –L1889726 plotted against survival after 60 minutes heat and 30 min oxidative stress. Survival is expressed as the difference of log CFU/ml after stress and before stress. Numbers indicate fermentations as presented in Table 1. P-values above the plots indicate significance of correlation (assessed by a linear model). (ZIP) [file pone.0167944.s007.zip › S2_File/L118462_real_dat.png]

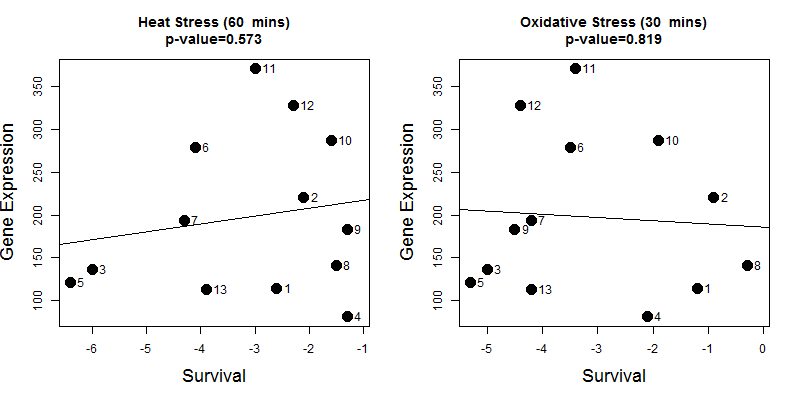

Supplement: S2 File — Expression levels of genes L75676 –L1889726 plotted against survival after 60 minutes heat and 30 min oxidative stress. Survival is expressed as the difference of log CFU/ml after stress and before stress. Numbers indicate fermentations as presented in Table 1. P-values above the plots indicate significance of correlation (assessed by a linear model). (ZIP) [file pone.0167944.s007.zip › S2_File/L118475_real_dat.png]

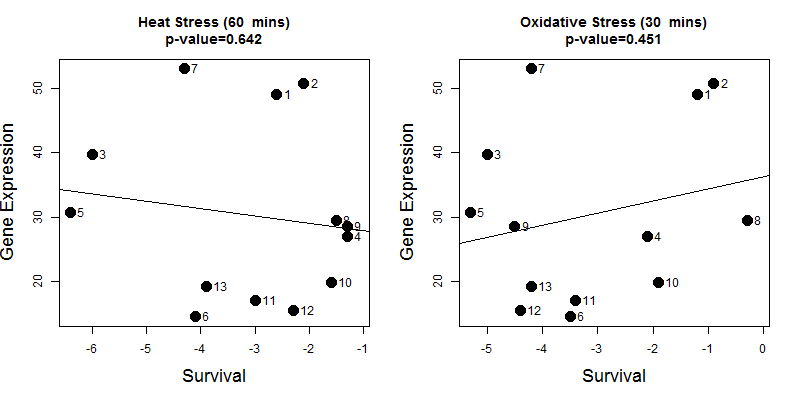

Supplement: S2 File — Expression levels of genes L75676 –L1889726 plotted against survival after 60 minutes heat and 30 min oxidative stress. Survival is expressed as the difference of log CFU/ml after stress and before stress. Numbers indicate fermentations as presented in Table 1. P-values above the plots indicate significance of correlation (assessed by a linear model). (ZIP) [file pone.0167944.s007.zip › S2_File/L118481_real_dat.png]

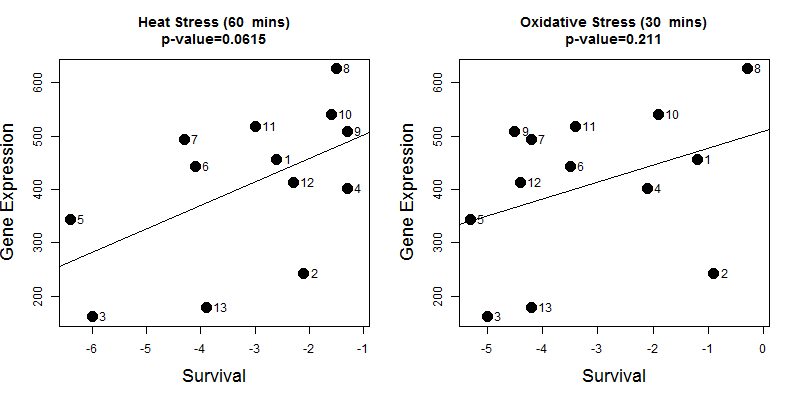

Supplement: S2 File — Expression levels of genes L75676 –L1889726 plotted against survival after 60 minutes heat and 30 min oxidative stress. Survival is expressed as the difference of log CFU/ml after stress and before stress. Numbers indicate fermentations as presented in Table 1. P-values above the plots indicate significance of correlation (assessed by a linear model). (ZIP) [file pone.0167944.s007.zip › S2_File/L118668_real_dat.png]

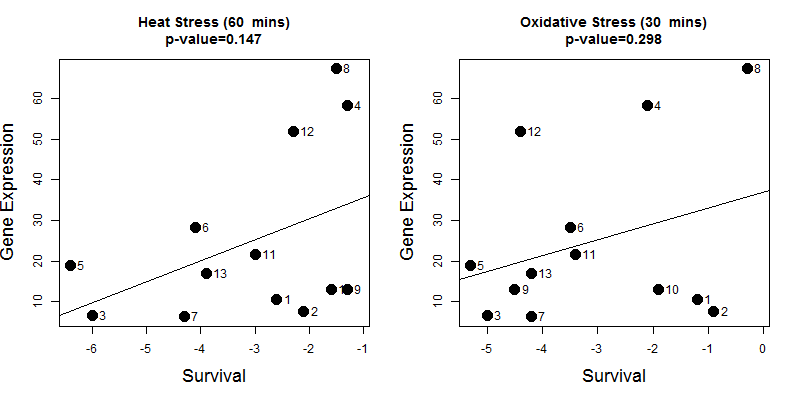

Supplement: S2 File — Expression levels of genes L75676 –L1889726 plotted against survival after 60 minutes heat and 30 min oxidative stress. Survival is expressed as the difference of log CFU/ml after stress and before stress. Numbers indicate fermentations as presented in Table 1. P-values above the plots indicate significance of correlation (assessed by a linear model). (ZIP) [file pone.0167944.s007.zip › S2_File/L118696_real_dat.png]

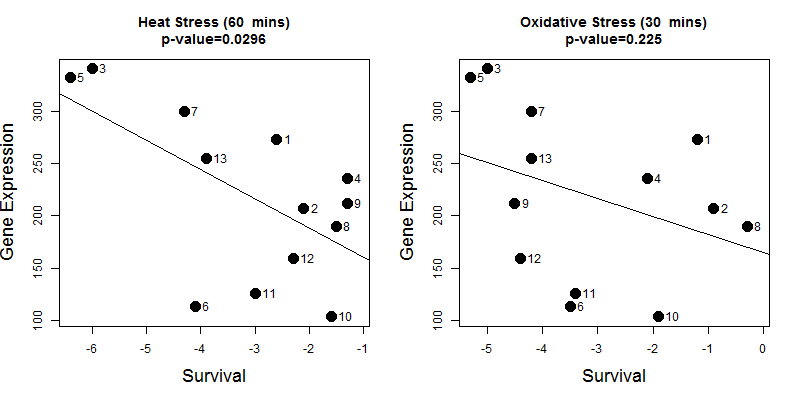

Supplement: S2 File — Expression levels of genes L75676 –L1889726 plotted against survival after 60 minutes heat and 30 min oxidative stress. Survival is expressed as the difference of log CFU/ml after stress and before stress. Numbers indicate fermentations as presented in Table 1. P-values above the plots indicate significance of correlation (assessed by a linear model). (ZIP) [file pone.0167944.s007.zip › S2_File/L119013_real_dat.png]

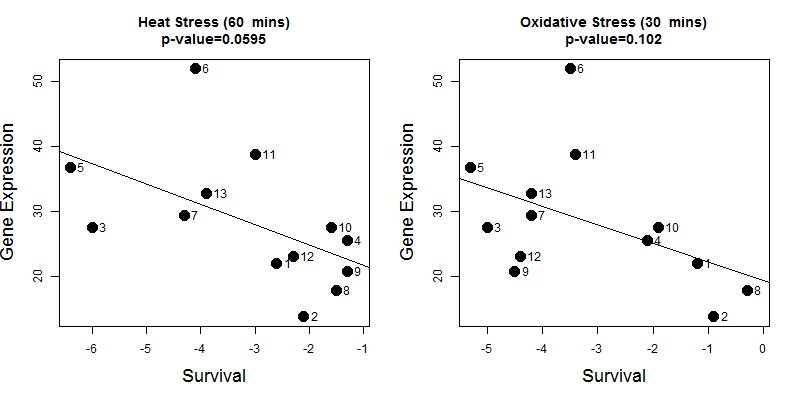

Supplement: S2 File — Expression levels of genes L75676 –L1889726 plotted against survival after 60 minutes heat and 30 min oxidative stress. Survival is expressed as the difference of log CFU/ml after stress and before stress. Numbers indicate fermentations as presented in Table 1. P-values above the plots indicate significance of correlation (assessed by a linear model). (ZIP) [file pone.0167944.s007.zip › S2_File/L119032_real_dat.png]

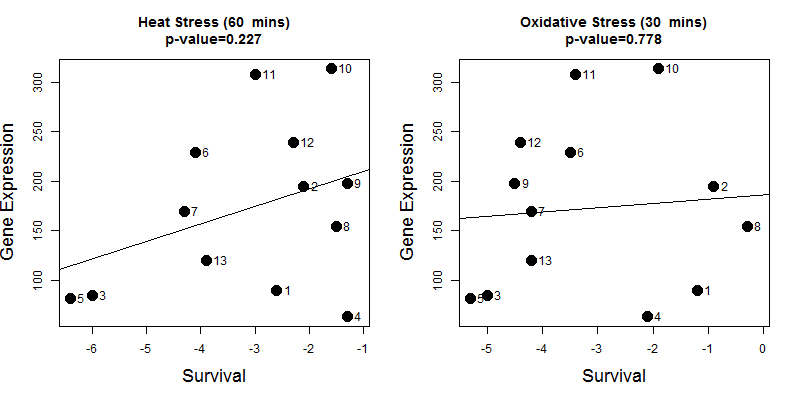

Supplement: S2 File — Expression levels of genes L75676 –L1889726 plotted against survival after 60 minutes heat and 30 min oxidative stress. Survival is expressed as the difference of log CFU/ml after stress and before stress. Numbers indicate fermentations as presented in Table 1. P-values above the plots indicate significance of correlation (assessed by a linear model). (ZIP) [file pone.0167944.s007.zip › S2_File/L119452_real_dat.png]

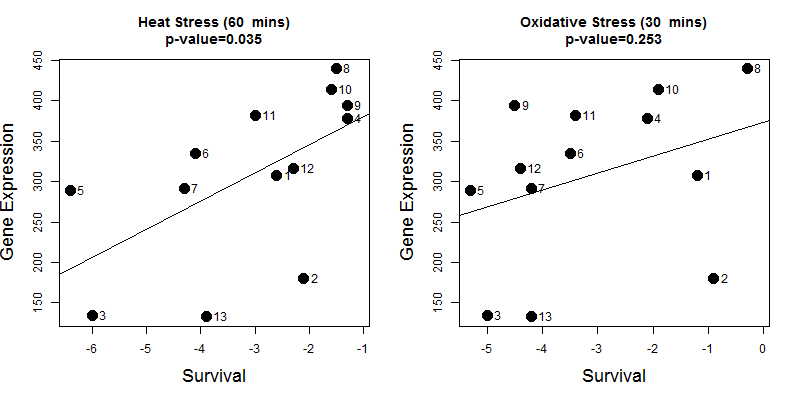

Supplement: S2 File — Expression levels of genes L75676 –L1889726 plotted against survival after 60 minutes heat and 30 min oxidative stress. Survival is expressed as the difference of log CFU/ml after stress and before stress. Numbers indicate fermentations as presented in Table 1. P-values above the plots indicate significance of correlation (assessed by a linear model). (ZIP) [file pone.0167944.s007.zip › S2_File/L119456_real_dat.png]

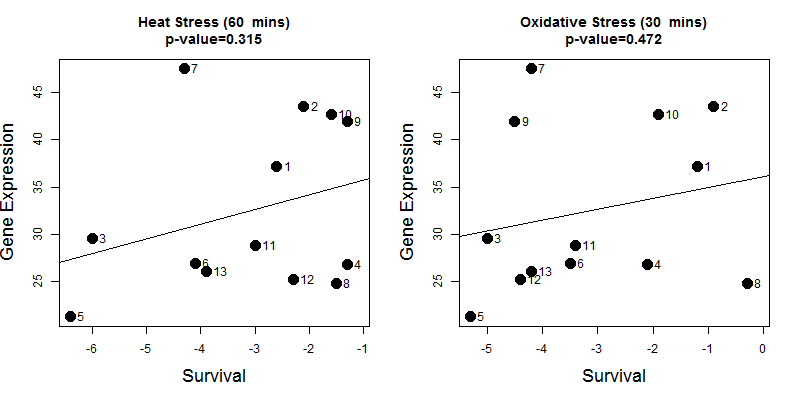

Supplement: S2 File — Expression levels of genes L75676 –L1889726 plotted against survival after 60 minutes heat and 30 min oxidative stress. Survival is expressed as the difference of log CFU/ml after stress and before stress. Numbers indicate fermentations as presented in Table 1. P-values above the plots indicate significance of correlation (assessed by a linear model). (ZIP) [file pone.0167944.s007.zip › S2_File/L119564_real_dat.png]

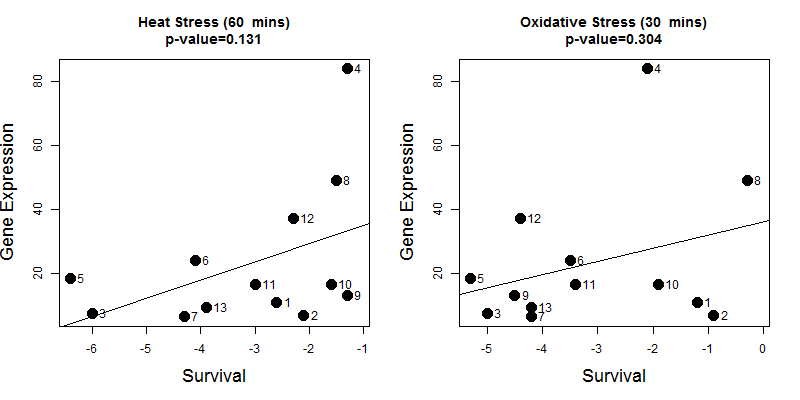

Supplement: S2 File — Expression levels of genes L75676 –L1889726 plotted against survival after 60 minutes heat and 30 min oxidative stress. Survival is expressed as the difference of log CFU/ml after stress and before stress. Numbers indicate fermentations as presented in Table 1. P-values above the plots indicate significance of correlation (assessed by a linear model). (ZIP) [file pone.0167944.s007.zip › S2_File/L119614_real_dat.png]

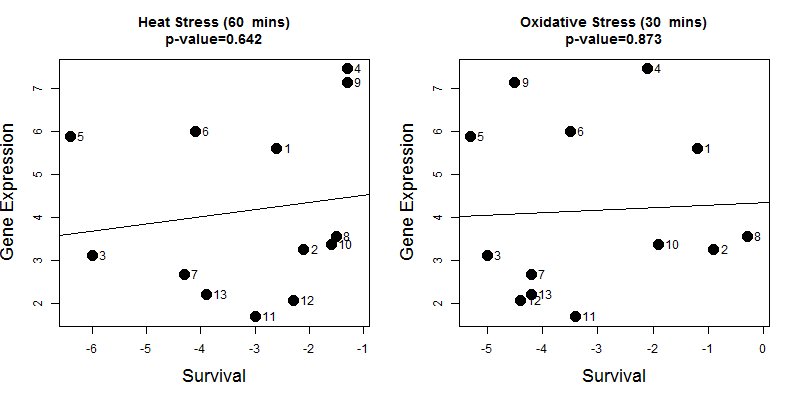

Supplement: S2 File — Expression levels of genes L75676 –L1889726 plotted against survival after 60 minutes heat and 30 min oxidative stress. Survival is expressed as the difference of log CFU/ml after stress and before stress. Numbers indicate fermentations as presented in Table 1. P-values above the plots indicate significance of correlation (assessed by a linear model). (ZIP) [file pone.0167944.s007.zip › S2_File/L119676_real_dat.png]

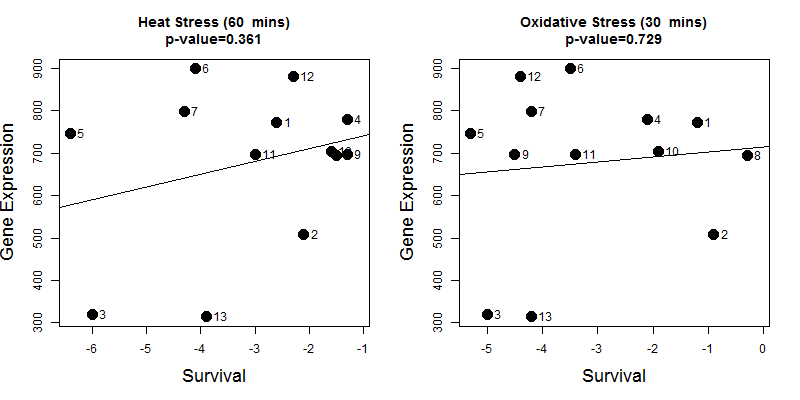

Supplement: S2 File — Expression levels of genes L75676 –L1889726 plotted against survival after 60 minutes heat and 30 min oxidative stress. Survival is expressed as the difference of log CFU/ml after stress and before stress. Numbers indicate fermentations as presented in Table 1. P-values above the plots indicate significance of correlation (assessed by a linear model). (ZIP) [file pone.0167944.s007.zip › S2_File/L119731_real_dat.png]

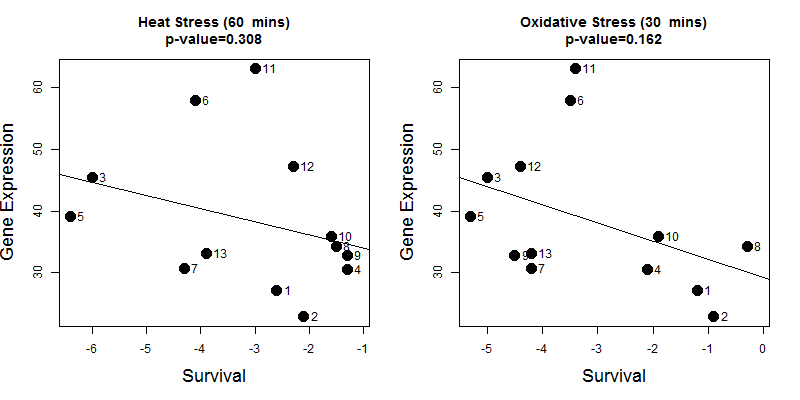

Supplement: S2 File — Expression levels of genes L75676 –L1889726 plotted against survival after 60 minutes heat and 30 min oxidative stress. Survival is expressed as the difference of log CFU/ml after stress and before stress. Numbers indicate fermentations as presented in Table 1. P-values above the plots indicate significance of correlation (assessed by a linear model). (ZIP) [file pone.0167944.s007.zip › S2_File/L119780_real_dat.png]
