# Supplementary figures and images for: Strain-Dependent Transcriptome Signatures for Robustness in Lactococcus lactis (part 7 of 13)
Source: PLoS One. 2016 Dec 14;11(12):e0167944. doi: 10.1371/journal.pone.0167944 (PMC5156439; doi:10.1371/journal.pone.0167944)

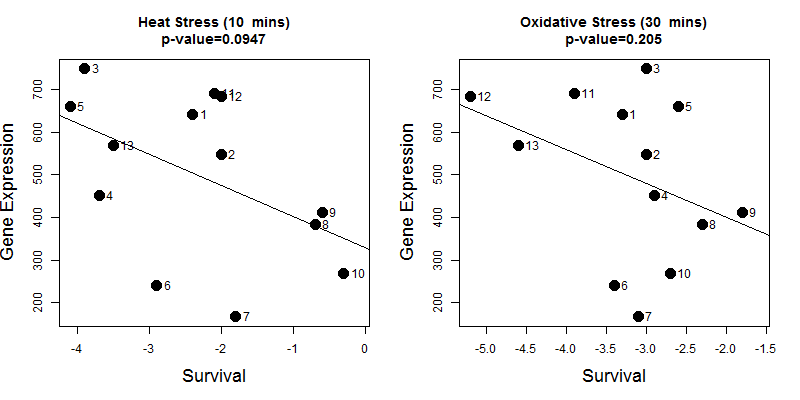

Supplement: S3 File — Expression levels of genes LLKF_0001 –LLKF_1273 plotted against survival after 10 minutes heat and 30 minutes oxidative stress. Survival is expressed as the difference of log CFU/ml after stress and before stress. Numbers indicate fermentations as presented in Table 1. P-values above the plots indicate significance of correlation (assessed by a linear model). (ZIP) [file pone.0167944.s008.zip › S3_File/LLKF_0204_real_dat.png]

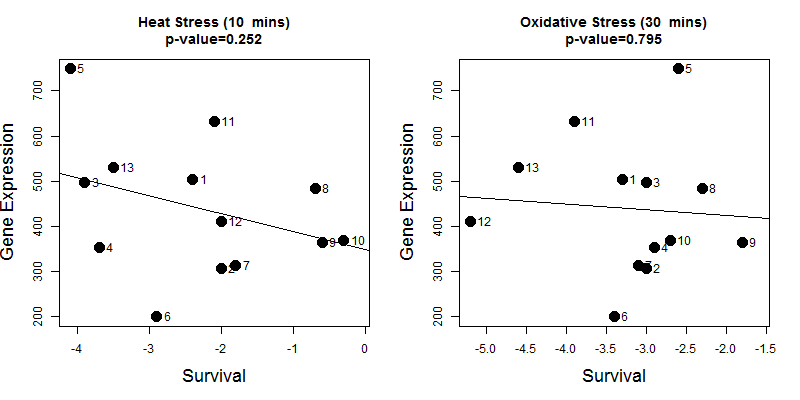

Supplement: S3 File — Expression levels of genes LLKF_0001 –LLKF_1273 plotted against survival after 10 minutes heat and 30 minutes oxidative stress. Survival is expressed as the difference of log CFU/ml after stress and before stress. Numbers indicate fermentations as presented in Table 1. P-values above the plots indicate significance of correlation (assessed by a linear model). (ZIP) [file pone.0167944.s008.zip › S3_File/LLKF_0205_real_dat.png]

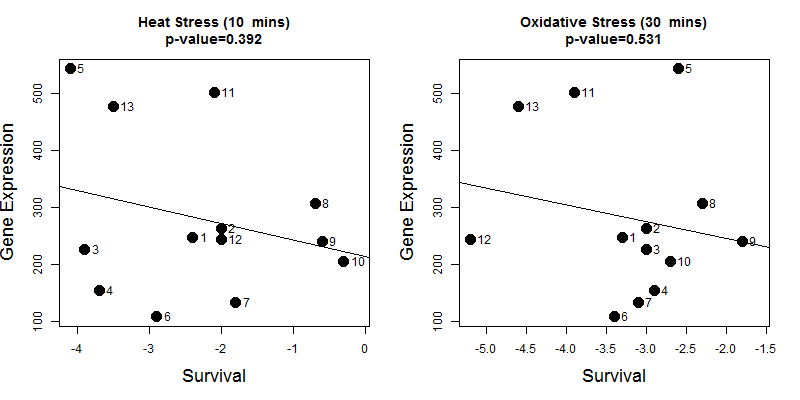

Supplement: S3 File — Expression levels of genes LLKF_0001 –LLKF_1273 plotted against survival after 10 minutes heat and 30 minutes oxidative stress. Survival is expressed as the difference of log CFU/ml after stress and before stress. Numbers indicate fermentations as presented in Table 1. P-values above the plots indicate significance of correlation (assessed by a linear model). (ZIP) [file pone.0167944.s008.zip › S3_File/LLKF_0206_real_dat.png]

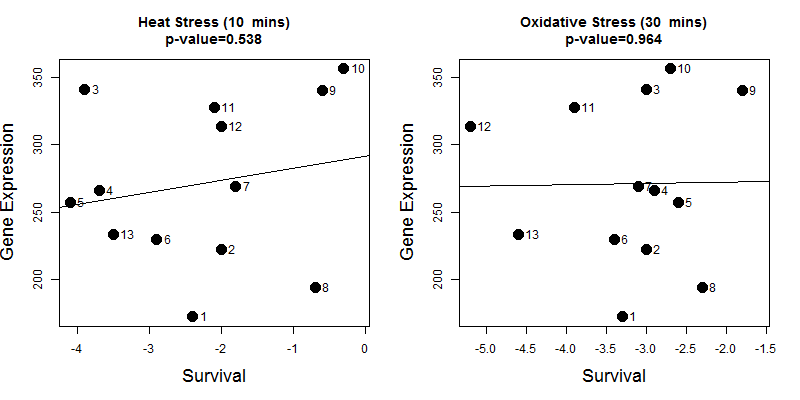

Supplement: S4 File — Expression levels of genes LLKF_1274 –LLKF_2533 and LLKF_p0001 –LLKF_p0036 plotted against survival after 10 minutes heat and 30 minutes oxidative stress. Survival is expressed as the difference of log CFU/ml after stress and before stress. Numbers indicate fermentations as presented in Table 1. P-values above the plots indicate significance of correlation (assessed by a linear model). (ZIP) [file pone.0167944.s009.zip › S4_File/LLKF_1274_real_dat.png]

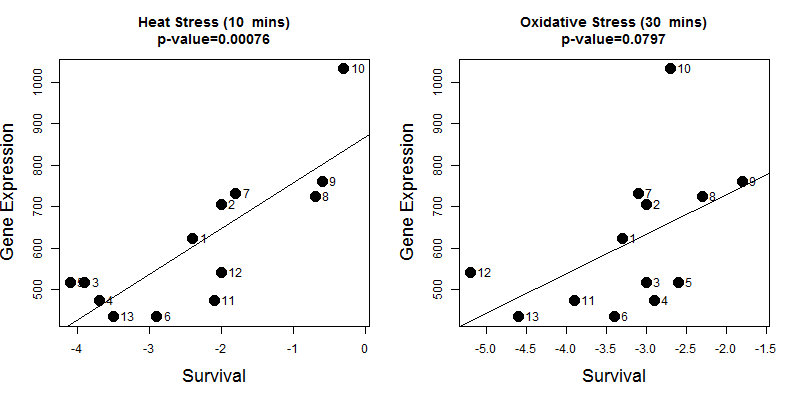

Supplement: S4 File — Expression levels of genes LLKF_1274 –LLKF_2533 and LLKF_p0001 –LLKF_p0036 plotted against survival after 10 minutes heat and 30 minutes oxidative stress. Survival is expressed as the difference of log CFU/ml after stress and before stress. Numbers indicate fermentations as presented in Table 1. P-values above the plots indicate significance of correlation (assessed by a linear model). (ZIP) [file pone.0167944.s009.zip › S4_File/LLKF_1275_real_dat.png]

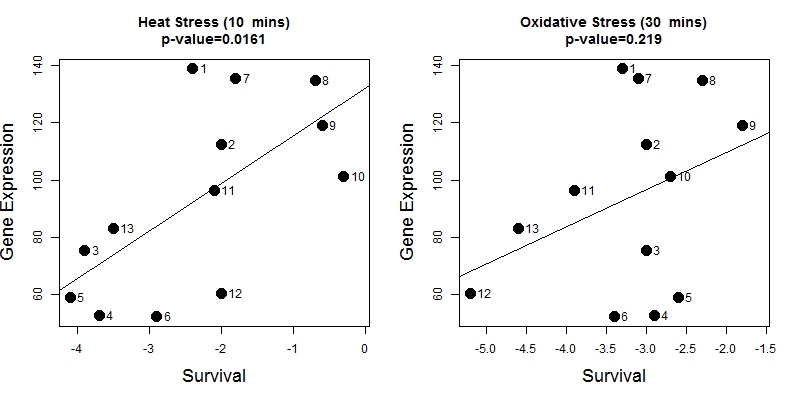

Supplement: S4 File — Expression levels of genes LLKF_1274 –LLKF_2533 and LLKF_p0001 –LLKF_p0036 plotted against survival after 10 minutes heat and 30 minutes oxidative stress. Survival is expressed as the difference of log CFU/ml after stress and before stress. Numbers indicate fermentations as presented in Table 1. P-values above the plots indicate significance of correlation (assessed by a linear model). (ZIP) [file pone.0167944.s009.zip › S4_File/LLKF_1276_real_dat.png]

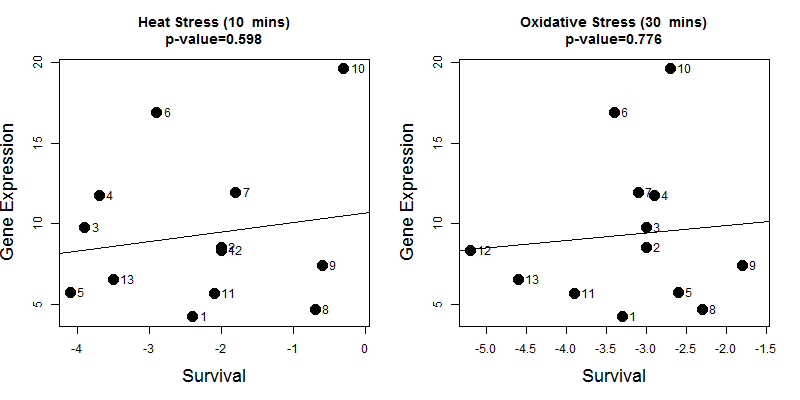

Supplement: S4 File — Expression levels of genes LLKF_1274 –LLKF_2533 and LLKF_p0001 –LLKF_p0036 plotted against survival after 10 minutes heat and 30 minutes oxidative stress. Survival is expressed as the difference of log CFU/ml after stress and before stress. Numbers indicate fermentations as presented in Table 1. P-values above the plots indicate significance of correlation (assessed by a linear model). (ZIP) [file pone.0167944.s009.zip › S4_File/LLKF_1277_real_dat.png]

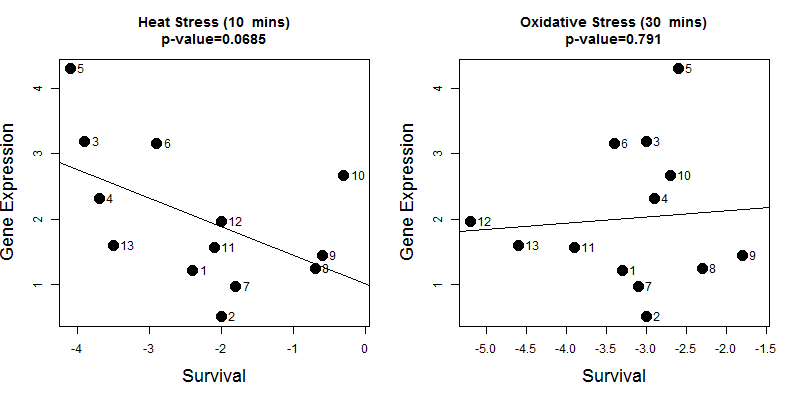

Supplement: S4 File — Expression levels of genes LLKF_1274 –LLKF_2533 and LLKF_p0001 –LLKF_p0036 plotted against survival after 10 minutes heat and 30 minutes oxidative stress. Survival is expressed as the difference of log CFU/ml after stress and before stress. Numbers indicate fermentations as presented in Table 1. P-values above the plots indicate significance of correlation (assessed by a linear model). (ZIP) [file pone.0167944.s009.zip › S4_File/LLKF_1278_real_dat.png]

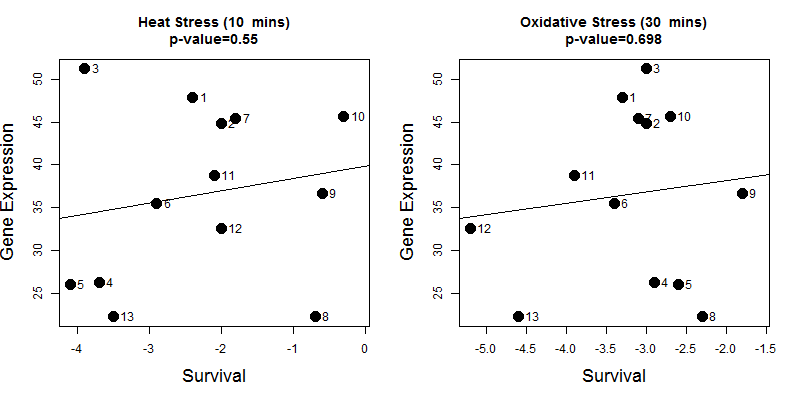

Supplement: S4 File — Expression levels of genes LLKF_1274 –LLKF_2533 and LLKF_p0001 –LLKF_p0036 plotted against survival after 10 minutes heat and 30 minutes oxidative stress. Survival is expressed as the difference of log CFU/ml after stress and before stress. Numbers indicate fermentations as presented in Table 1. P-values above the plots indicate significance of correlation (assessed by a linear model). (ZIP) [file pone.0167944.s009.zip › S4_File/LLKF_1279_real_dat.png]

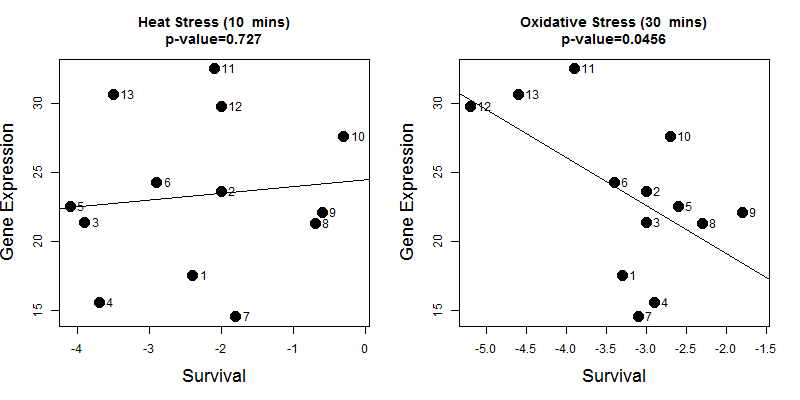

Supplement: S4 File — Expression levels of genes LLKF_1274 –LLKF_2533 and LLKF_p0001 –LLKF_p0036 plotted against survival after 10 minutes heat and 30 minutes oxidative stress. Survival is expressed as the difference of log CFU/ml after stress and before stress. Numbers indicate fermentations as presented in Table 1. P-values above the plots indicate significance of correlation (assessed by a linear model). (ZIP) [file pone.0167944.s009.zip › S4_File/LLKF_1280_real_dat.png]

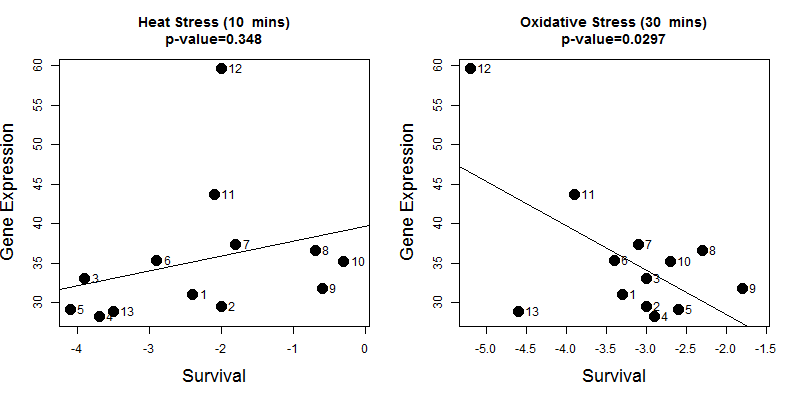

Supplement: S4 File — Expression levels of genes LLKF_1274 –LLKF_2533 and LLKF_p0001 –LLKF_p0036 plotted against survival after 10 minutes heat and 30 minutes oxidative stress. Survival is expressed as the difference of log CFU/ml after stress and before stress. Numbers indicate fermentations as presented in Table 1. P-values above the plots indicate significance of correlation (assessed by a linear model). (ZIP) [file pone.0167944.s009.zip › S4_File/LLKF_1281_real_dat.png]

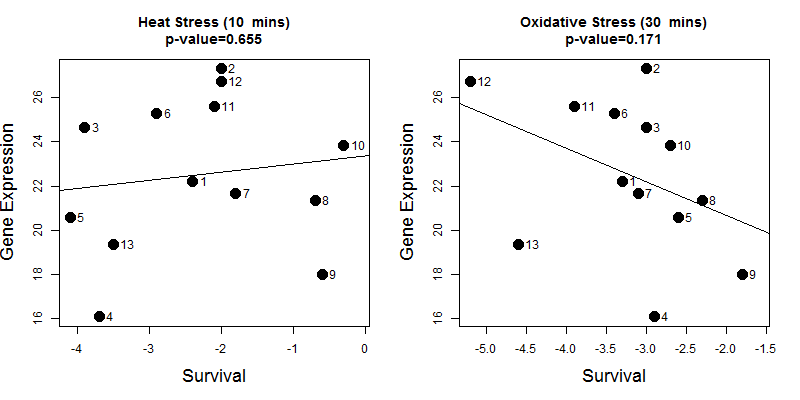

Supplement: S4 File — Expression levels of genes LLKF_1274 –LLKF_2533 and LLKF_p0001 –LLKF_p0036 plotted against survival after 10 minutes heat and 30 minutes oxidative stress. Survival is expressed as the difference of log CFU/ml after stress and before stress. Numbers indicate fermentations as presented in Table 1. P-values above the plots indicate significance of correlation (assessed by a linear model). (ZIP) [file pone.0167944.s009.zip › S4_File/LLKF_1282_real_dat.png]

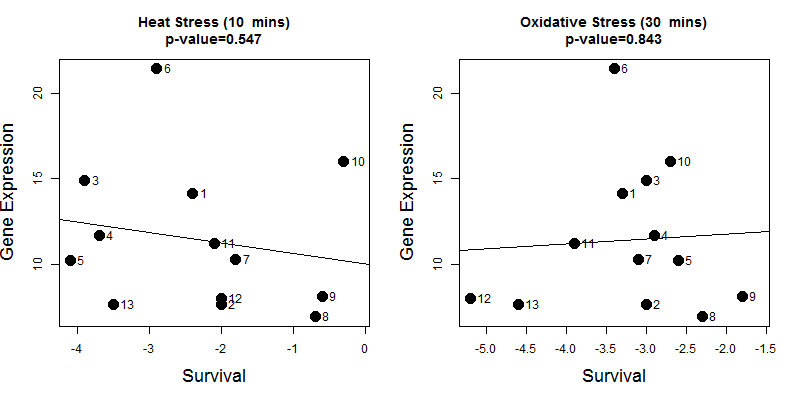

Supplement: S4 File — Expression levels of genes LLKF_1274 –LLKF_2533 and LLKF_p0001 –LLKF_p0036 plotted against survival after 10 minutes heat and 30 minutes oxidative stress. Survival is expressed as the difference of log CFU/ml after stress and before stress. Numbers indicate fermentations as presented in Table 1. P-values above the plots indicate significance of correlation (assessed by a linear model). (ZIP) [file pone.0167944.s009.zip › S4_File/LLKF_1283_real_dat.png]

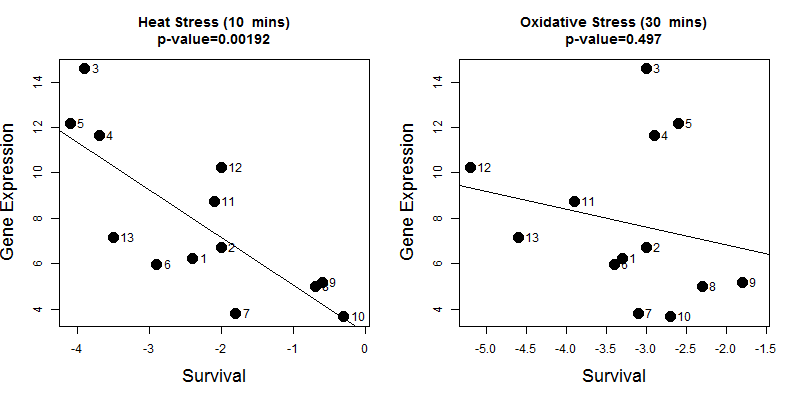

Supplement: S4 File — Expression levels of genes LLKF_1274 –LLKF_2533 and LLKF_p0001 –LLKF_p0036 plotted against survival after 10 minutes heat and 30 minutes oxidative stress. Survival is expressed as the difference of log CFU/ml after stress and before stress. Numbers indicate fermentations as presented in Table 1. P-values above the plots indicate significance of correlation (assessed by a linear model). (ZIP) [file pone.0167944.s009.zip › S4_File/LLKF_1284_real_dat.png]

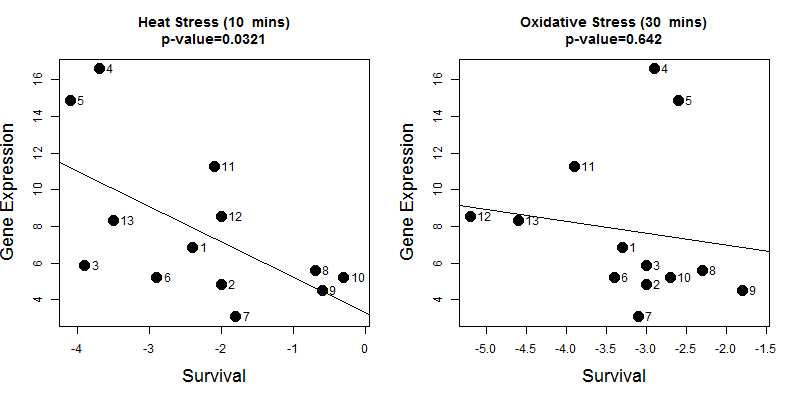

Supplement: S4 File — Expression levels of genes LLKF_1274 –LLKF_2533 and LLKF_p0001 –LLKF_p0036 plotted against survival after 10 minutes heat and 30 minutes oxidative stress. Survival is expressed as the difference of log CFU/ml after stress and before stress. Numbers indicate fermentations as presented in Table 1. P-values above the plots indicate significance of correlation (assessed by a linear model). (ZIP) [file pone.0167944.s009.zip › S4_File/LLKF_1287_real_dat.png]

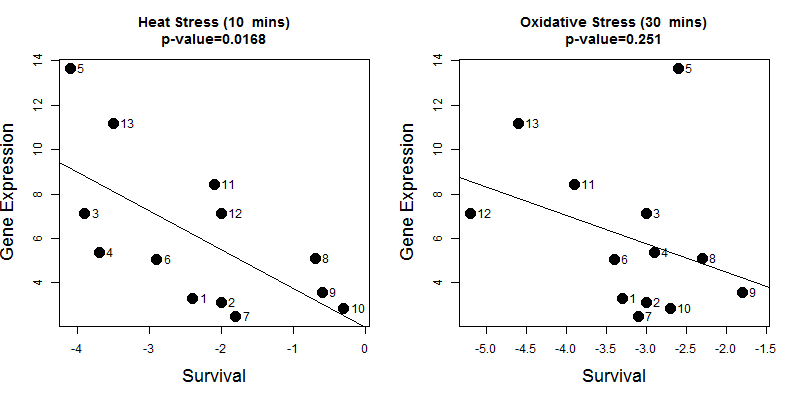

Supplement: S4 File — Expression levels of genes LLKF_1274 –LLKF_2533 and LLKF_p0001 –LLKF_p0036 plotted against survival after 10 minutes heat and 30 minutes oxidative stress. Survival is expressed as the difference of log CFU/ml after stress and before stress. Numbers indicate fermentations as presented in Table 1. P-values above the plots indicate significance of correlation (assessed by a linear model). (ZIP) [file pone.0167944.s009.zip › S4_File/LLKF_1288_real_dat.png]

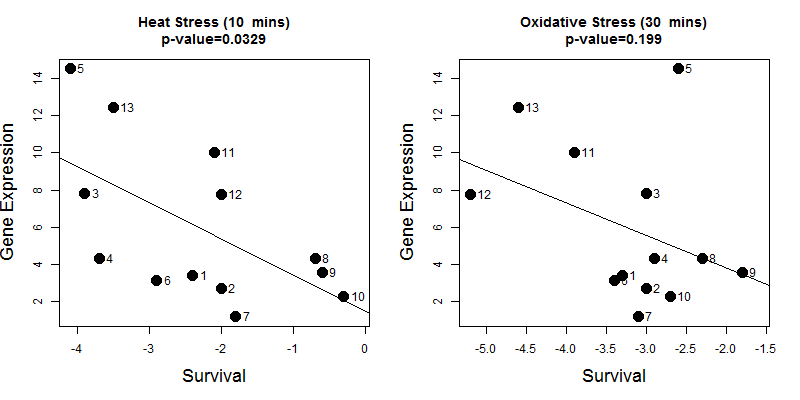

Supplement: S4 File — Expression levels of genes LLKF_1274 –LLKF_2533 and LLKF_p0001 –LLKF_p0036 plotted against survival after 10 minutes heat and 30 minutes oxidative stress. Survival is expressed as the difference of log CFU/ml after stress and before stress. Numbers indicate fermentations as presented in Table 1. P-values above the plots indicate significance of correlation (assessed by a linear model). (ZIP) [file pone.0167944.s009.zip › S4_File/LLKF_1289_real_dat.png]

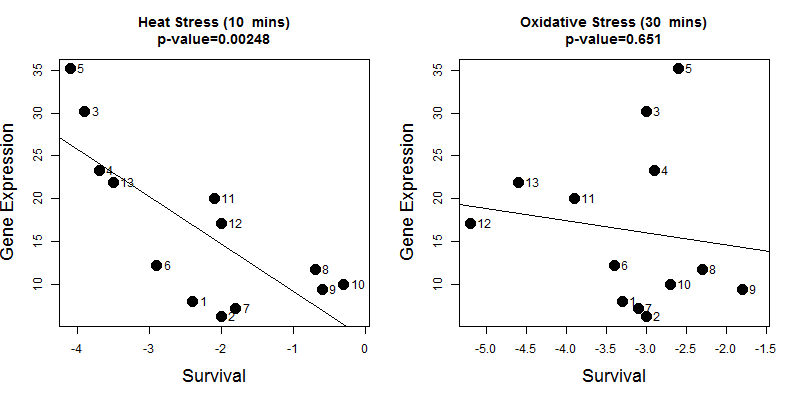

Supplement: S4 File — Expression levels of genes LLKF_1274 –LLKF_2533 and LLKF_p0001 –LLKF_p0036 plotted against survival after 10 minutes heat and 30 minutes oxidative stress. Survival is expressed as the difference of log CFU/ml after stress and before stress. Numbers indicate fermentations as presented in Table 1. P-values above the plots indicate significance of correlation (assessed by a linear model). (ZIP) [file pone.0167944.s009.zip › S4_File/LLKF_1290_real_dat.png]

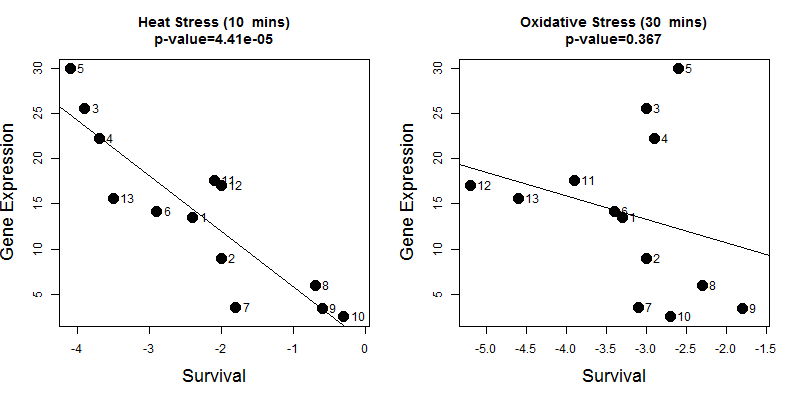

Supplement: S4 File — Expression levels of genes LLKF_1274 –LLKF_2533 and LLKF_p0001 –LLKF_p0036 plotted against survival after 10 minutes heat and 30 minutes oxidative stress. Survival is expressed as the difference of log CFU/ml after stress and before stress. Numbers indicate fermentations as presented in Table 1. P-values above the plots indicate significance of correlation (assessed by a linear model). (ZIP) [file pone.0167944.s009.zip › S4_File/LLKF_1293_real_dat.png]

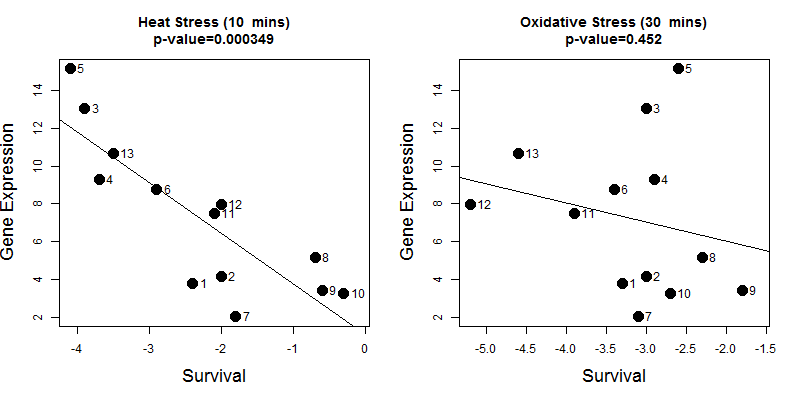

Supplement: S4 File — Expression levels of genes LLKF_1274 –LLKF_2533 and LLKF_p0001 –LLKF_p0036 plotted against survival after 10 minutes heat and 30 minutes oxidative stress. Survival is expressed as the difference of log CFU/ml after stress and before stress. Numbers indicate fermentations as presented in Table 1. P-values above the plots indicate significance of correlation (assessed by a linear model). (ZIP) [file pone.0167944.s009.zip › S4_File/LLKF_1294_real_dat.png]

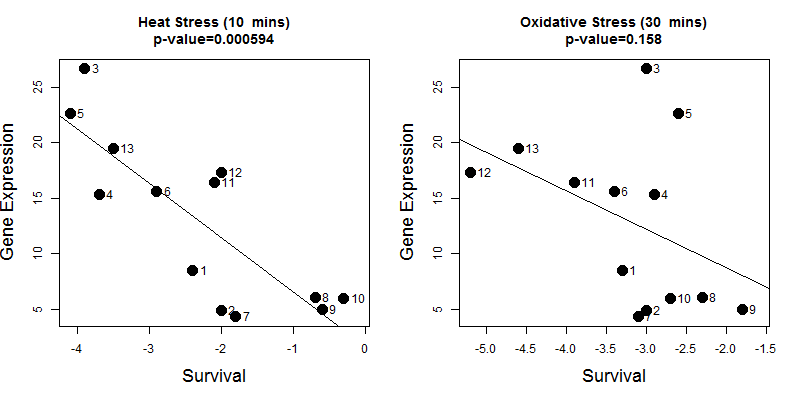

Supplement: S4 File — Expression levels of genes LLKF_1274 –LLKF_2533 and LLKF_p0001 –LLKF_p0036 plotted against survival after 10 minutes heat and 30 minutes oxidative stress. Survival is expressed as the difference of log CFU/ml after stress and before stress. Numbers indicate fermentations as presented in Table 1. P-values above the plots indicate significance of correlation (assessed by a linear model). (ZIP) [file pone.0167944.s009.zip › S4_File/LLKF_1295_real_dat.png]

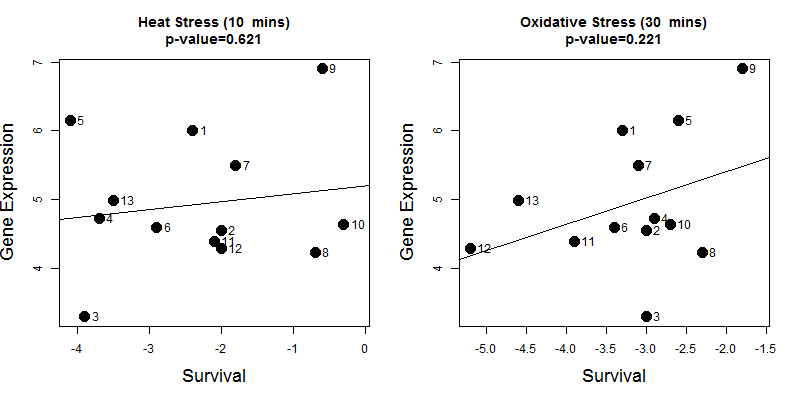

Supplement: S4 File — Expression levels of genes LLKF_1274 –LLKF_2533 and LLKF_p0001 –LLKF_p0036 plotted against survival after 10 minutes heat and 30 minutes oxidative stress. Survival is expressed as the difference of log CFU/ml after stress and before stress. Numbers indicate fermentations as presented in Table 1. P-values above the plots indicate significance of correlation (assessed by a linear model). (ZIP) [file pone.0167944.s009.zip › S4_File/LLKF_1296_real_dat.png]

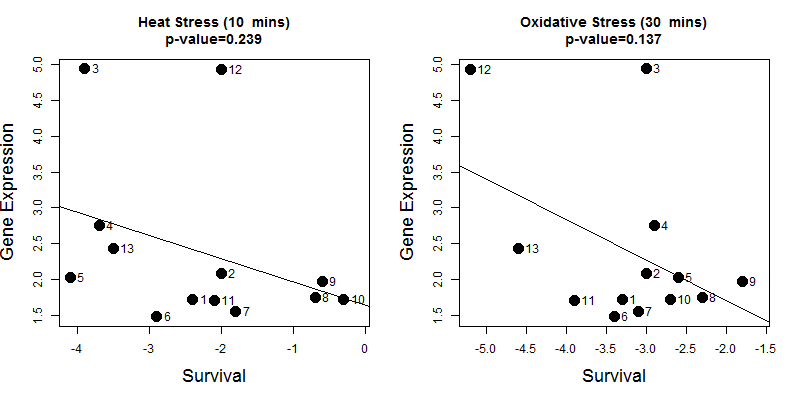

Supplement: S4 File — Expression levels of genes LLKF_1274 –LLKF_2533 and LLKF_p0001 –LLKF_p0036 plotted against survival after 10 minutes heat and 30 minutes oxidative stress. Survival is expressed as the difference of log CFU/ml after stress and before stress. Numbers indicate fermentations as presented in Table 1. P-values above the plots indicate significance of correlation (assessed by a linear model). (ZIP) [file pone.0167944.s009.zip › S4_File/LLKF_1297_real_dat.png]

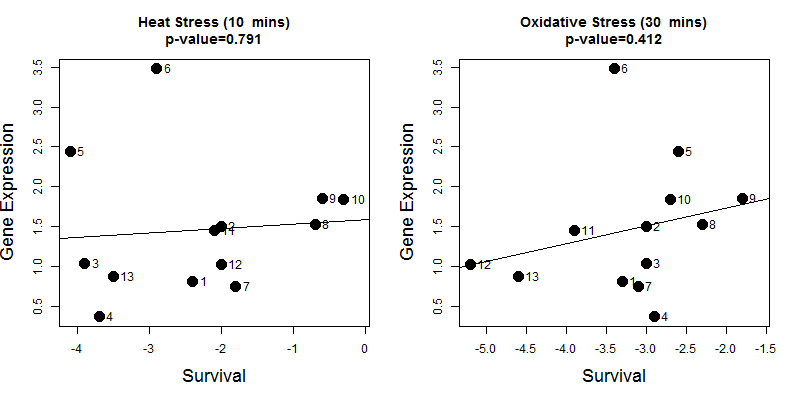

Supplement: S4 File — Expression levels of genes LLKF_1274 –LLKF_2533 and LLKF_p0001 –LLKF_p0036 plotted against survival after 10 minutes heat and 30 minutes oxidative stress. Survival is expressed as the difference of log CFU/ml after stress and before stress. Numbers indicate fermentations as presented in Table 1. P-values above the plots indicate significance of correlation (assessed by a linear model). (ZIP) [file pone.0167944.s009.zip › S4_File/LLKF_1298_real_dat.png]

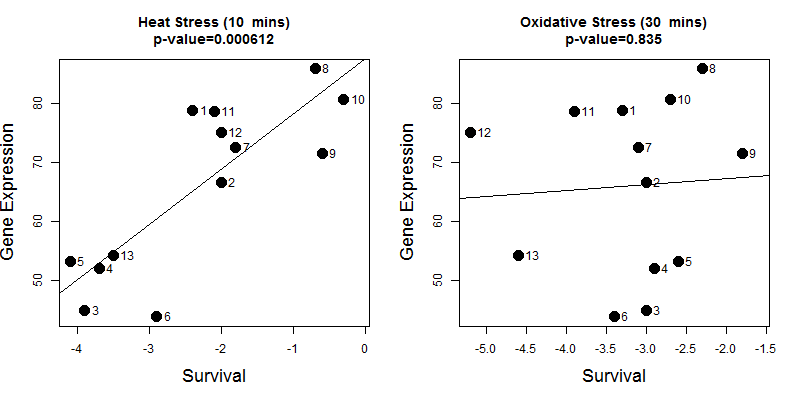

Supplement: S4 File — Expression levels of genes LLKF_1274 –LLKF_2533 and LLKF_p0001 –LLKF_p0036 plotted against survival after 10 minutes heat and 30 minutes oxidative stress. Survival is expressed as the difference of log CFU/ml after stress and before stress. Numbers indicate fermentations as presented in Table 1. P-values above the plots indicate significance of correlation (assessed by a linear model). (ZIP) [file pone.0167944.s009.zip › S4_File/LLKF_1299_real_dat.png]

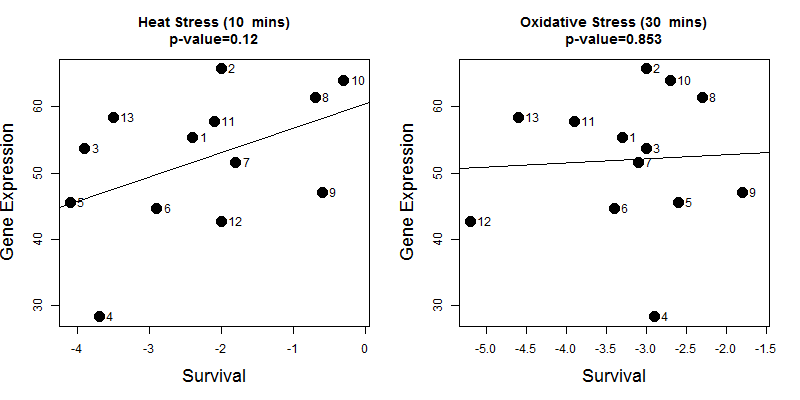

Supplement: S4 File — Expression levels of genes LLKF_1274 –LLKF_2533 and LLKF_p0001 –LLKF_p0036 plotted against survival after 10 minutes heat and 30 minutes oxidative stress. Survival is expressed as the difference of log CFU/ml after stress and before stress. Numbers indicate fermentations as presented in Table 1. P-values above the plots indicate significance of correlation (assessed by a linear model). (ZIP) [file pone.0167944.s009.zip › S4_File/LLKF_1300_real_dat.png]

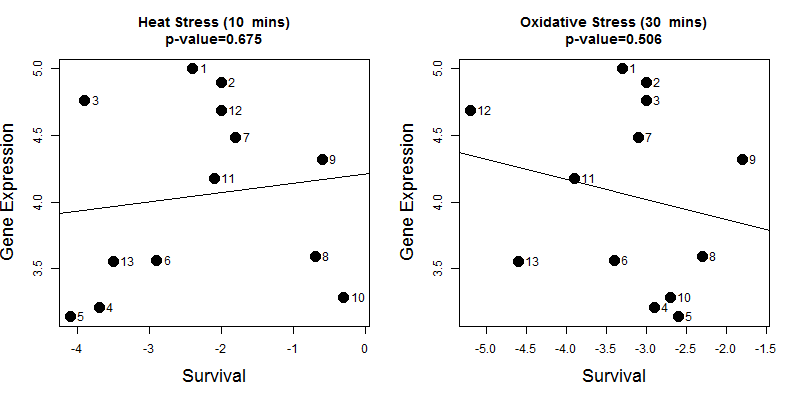

Supplement: S4 File — Expression levels of genes LLKF_1274 –LLKF_2533 and LLKF_p0001 –LLKF_p0036 plotted against survival after 10 minutes heat and 30 minutes oxidative stress. Survival is expressed as the difference of log CFU/ml after stress and before stress. Numbers indicate fermentations as presented in Table 1. P-values above the plots indicate significance of correlation (assessed by a linear model). (ZIP) [file pone.0167944.s009.zip › S4_File/LLKF_1304_real_dat.png]

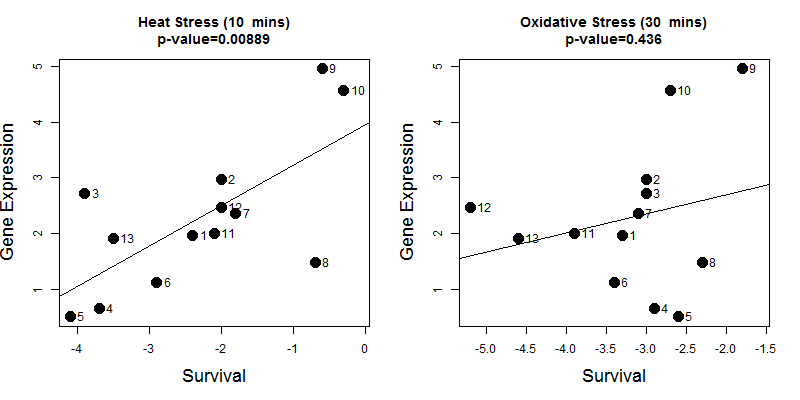

Supplement: S4 File — Expression levels of genes LLKF_1274 –LLKF_2533 and LLKF_p0001 –LLKF_p0036 plotted against survival after 10 minutes heat and 30 minutes oxidative stress. Survival is expressed as the difference of log CFU/ml after stress and before stress. Numbers indicate fermentations as presented in Table 1. P-values above the plots indicate significance of correlation (assessed by a linear model). (ZIP) [file pone.0167944.s009.zip › S4_File/LLKF_1307_real_dat.png]

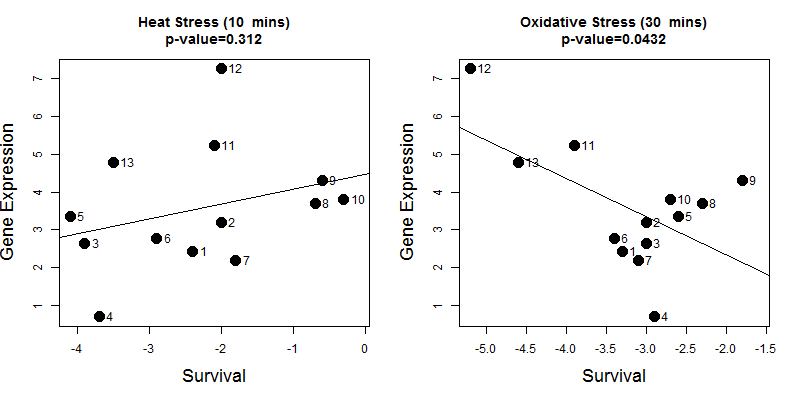

Supplement: S4 File — Expression levels of genes LLKF_1274 –LLKF_2533 and LLKF_p0001 –LLKF_p0036 plotted against survival after 10 minutes heat and 30 minutes oxidative stress. Survival is expressed as the difference of log CFU/ml after stress and before stress. Numbers indicate fermentations as presented in Table 1. P-values above the plots indicate significance of correlation (assessed by a linear model). (ZIP) [file pone.0167944.s009.zip › S4_File/LLKF_1308_real_dat.png]

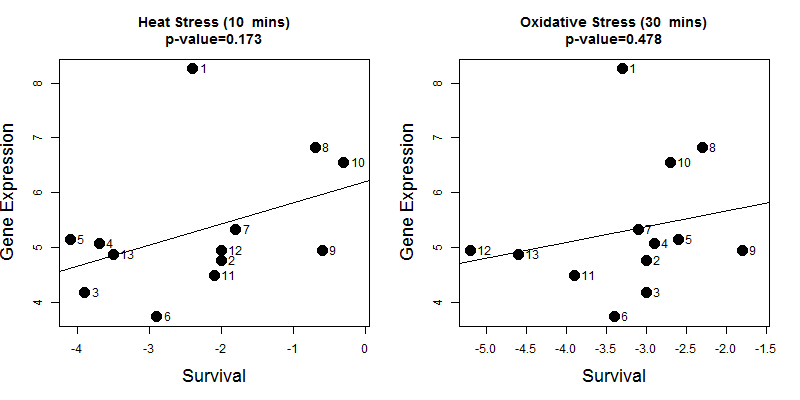

Supplement: S4 File — Expression levels of genes LLKF_1274 –LLKF_2533 and LLKF_p0001 –LLKF_p0036 plotted against survival after 10 minutes heat and 30 minutes oxidative stress. Survival is expressed as the difference of log CFU/ml after stress and before stress. Numbers indicate fermentations as presented in Table 1. P-values above the plots indicate significance of correlation (assessed by a linear model). (ZIP) [file pone.0167944.s009.zip › S4_File/LLKF_1309_real_dat.png]

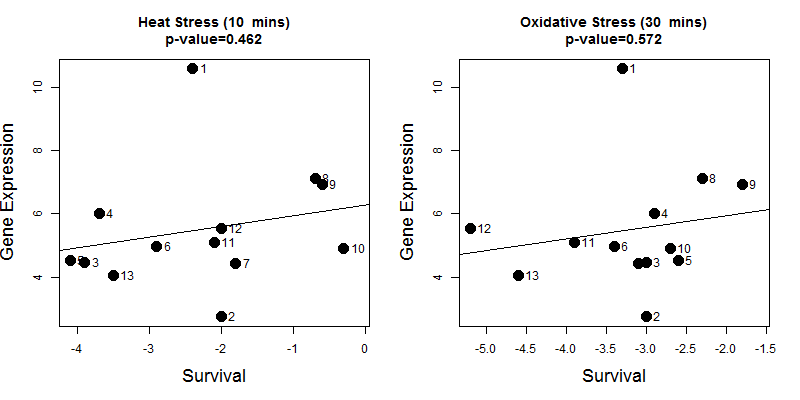

Supplement: S4 File — Expression levels of genes LLKF_1274 –LLKF_2533 and LLKF_p0001 –LLKF_p0036 plotted against survival after 10 minutes heat and 30 minutes oxidative stress. Survival is expressed as the difference of log CFU/ml after stress and before stress. Numbers indicate fermentations as presented in Table 1. P-values above the plots indicate significance of correlation (assessed by a linear model). (ZIP) [file pone.0167944.s009.zip › S4_File/LLKF_1310_real_dat.png]

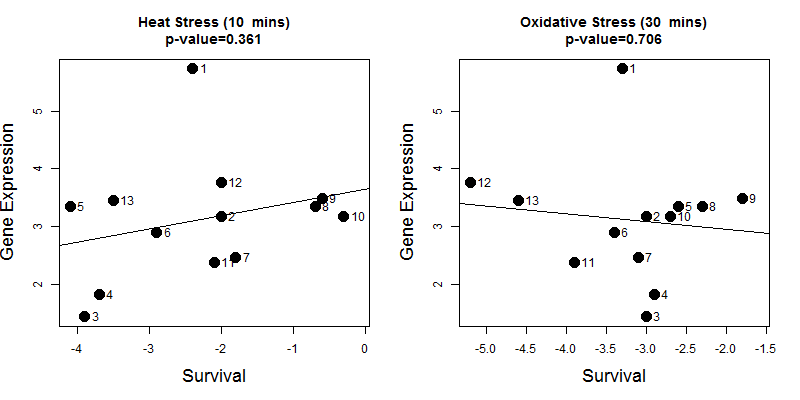

Supplement: S4 File — Expression levels of genes LLKF_1274 –LLKF_2533 and LLKF_p0001 –LLKF_p0036 plotted against survival after 10 minutes heat and 30 minutes oxidative stress. Survival is expressed as the difference of log CFU/ml after stress and before stress. Numbers indicate fermentations as presented in Table 1. P-values above the plots indicate significance of correlation (assessed by a linear model). (ZIP) [file pone.0167944.s009.zip › S4_File/LLKF_1311_real_dat.png]

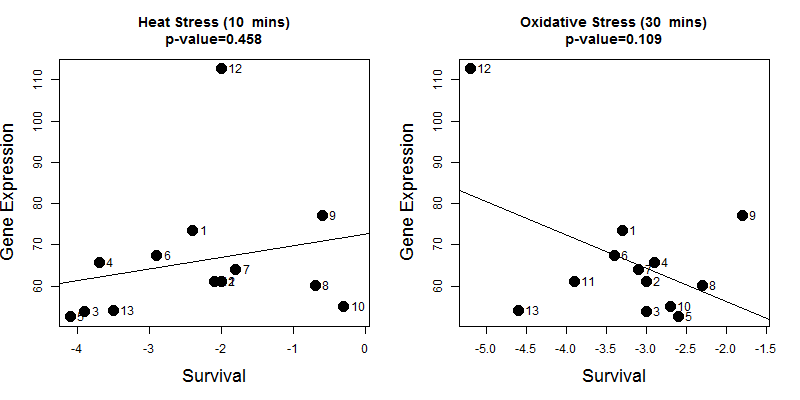

Supplement: S4 File — Expression levels of genes LLKF_1274 –LLKF_2533 and LLKF_p0001 –LLKF_p0036 plotted against survival after 10 minutes heat and 30 minutes oxidative stress. Survival is expressed as the difference of log CFU/ml after stress and before stress. Numbers indicate fermentations as presented in Table 1. P-values above the plots indicate significance of correlation (assessed by a linear model). (ZIP) [file pone.0167944.s009.zip › S4_File/LLKF_1312_real_dat.png]

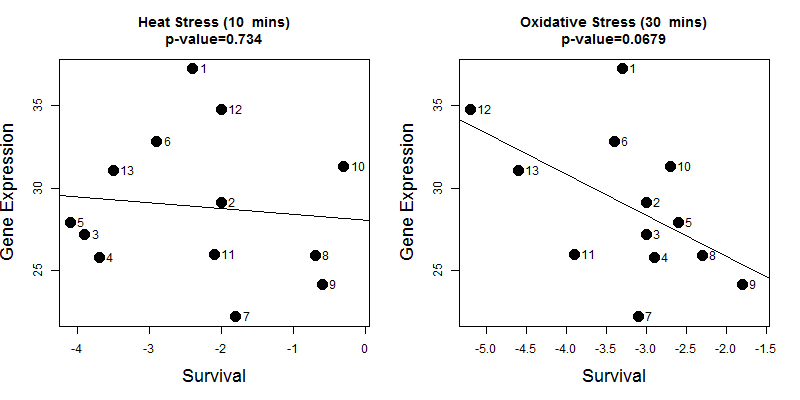

Supplement: S4 File — Expression levels of genes LLKF_1274 –LLKF_2533 and LLKF_p0001 –LLKF_p0036 plotted against survival after 10 minutes heat and 30 minutes oxidative stress. Survival is expressed as the difference of log CFU/ml after stress and before stress. Numbers indicate fermentations as presented in Table 1. P-values above the plots indicate significance of correlation (assessed by a linear model). (ZIP) [file pone.0167944.s009.zip › S4_File/LLKF_1313_real_dat.png]

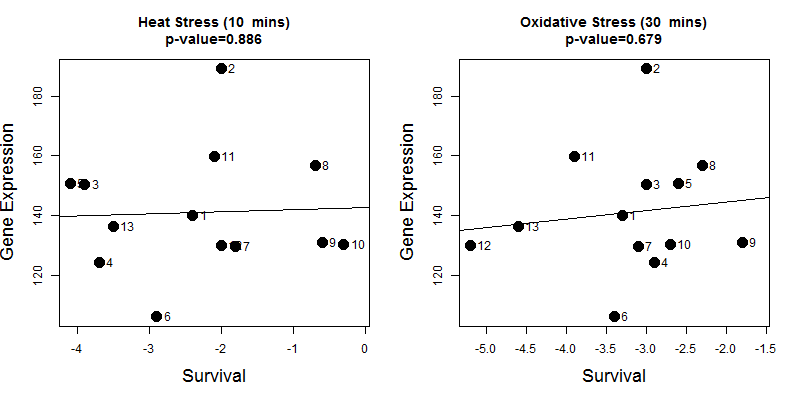

Supplement: S4 File — Expression levels of genes LLKF_1274 –LLKF_2533 and LLKF_p0001 –LLKF_p0036 plotted against survival after 10 minutes heat and 30 minutes oxidative stress. Survival is expressed as the difference of log CFU/ml after stress and before stress. Numbers indicate fermentations as presented in Table 1. P-values above the plots indicate significance of correlation (assessed by a linear model). (ZIP) [file pone.0167944.s009.zip › S4_File/LLKF_1314_real_dat.png]

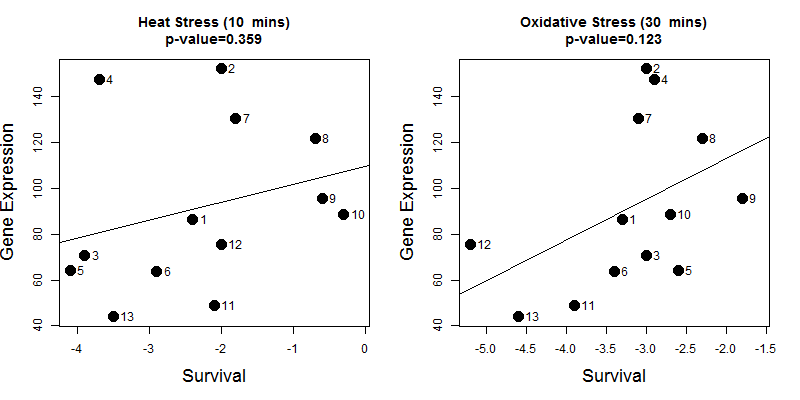

Supplement: S4 File — Expression levels of genes LLKF_1274 –LLKF_2533 and LLKF_p0001 –LLKF_p0036 plotted against survival after 10 minutes heat and 30 minutes oxidative stress. Survival is expressed as the difference of log CFU/ml after stress and before stress. Numbers indicate fermentations as presented in Table 1. P-values above the plots indicate significance of correlation (assessed by a linear model). (ZIP) [file pone.0167944.s009.zip › S4_File/LLKF_1315_real_dat.png]

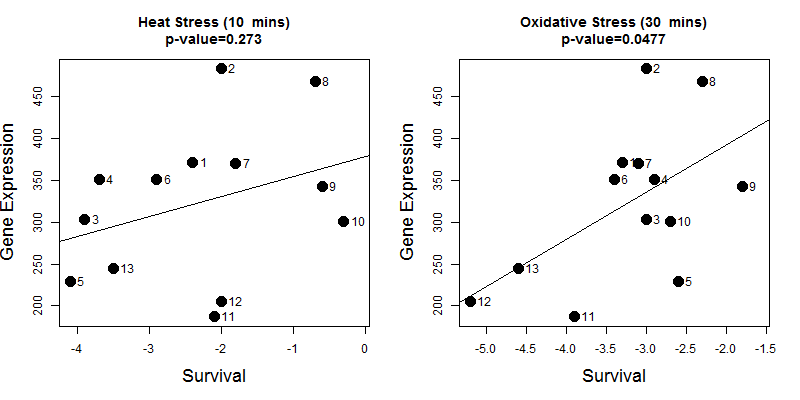

Supplement: S4 File — Expression levels of genes LLKF_1274 –LLKF_2533 and LLKF_p0001 –LLKF_p0036 plotted against survival after 10 minutes heat and 30 minutes oxidative stress. Survival is expressed as the difference of log CFU/ml after stress and before stress. Numbers indicate fermentations as presented in Table 1. P-values above the plots indicate significance of correlation (assessed by a linear model). (ZIP) [file pone.0167944.s009.zip › S4_File/LLKF_1316_real_dat.png]

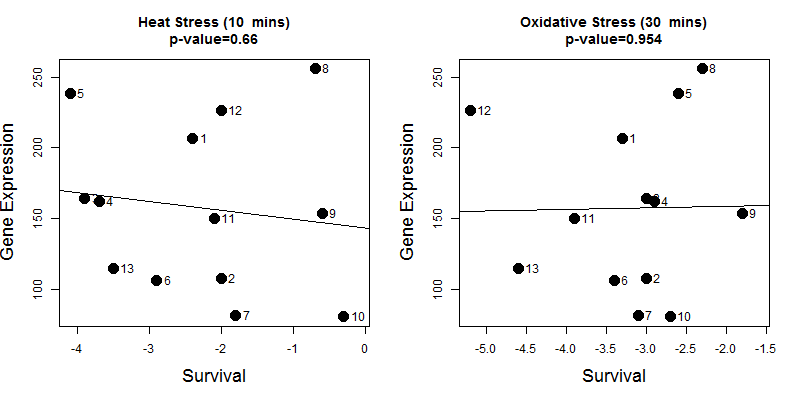

Supplement: S4 File — Expression levels of genes LLKF_1274 –LLKF_2533 and LLKF_p0001 –LLKF_p0036 plotted against survival after 10 minutes heat and 30 minutes oxidative stress. Survival is expressed as the difference of log CFU/ml after stress and before stress. Numbers indicate fermentations as presented in Table 1. P-values above the plots indicate significance of correlation (assessed by a linear model). (ZIP) [file pone.0167944.s009.zip › S4_File/LLKF_1317_real_dat.png]

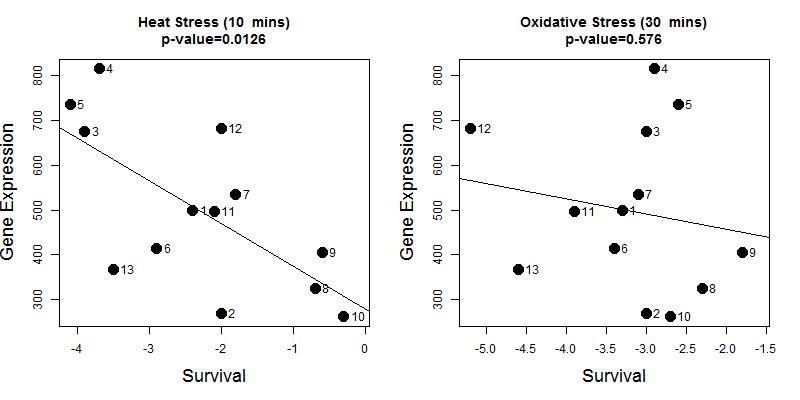

Supplement: S4 File — Expression levels of genes LLKF_1274 –LLKF_2533 and LLKF_p0001 –LLKF_p0036 plotted against survival after 10 minutes heat and 30 minutes oxidative stress. Survival is expressed as the difference of log CFU/ml after stress and before stress. Numbers indicate fermentations as presented in Table 1. P-values above the plots indicate significance of correlation (assessed by a linear model). (ZIP) [file pone.0167944.s009.zip › S4_File/LLKF_1318_real_dat.png]

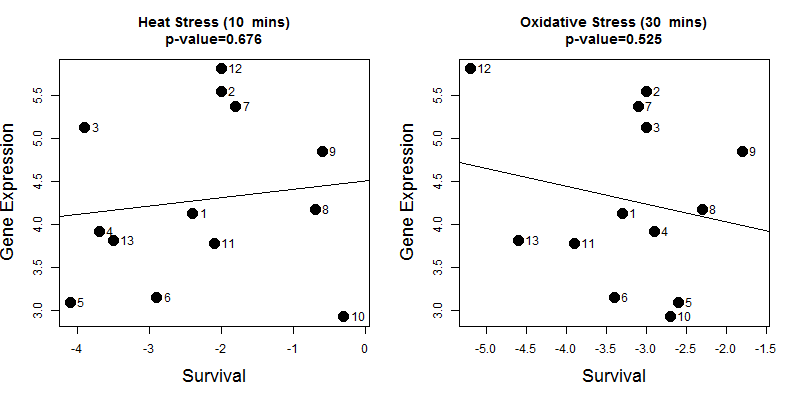

Supplement: S4 File — Expression levels of genes LLKF_1274 –LLKF_2533 and LLKF_p0001 –LLKF_p0036 plotted against survival after 10 minutes heat and 30 minutes oxidative stress. Survival is expressed as the difference of log CFU/ml after stress and before stress. Numbers indicate fermentations as presented in Table 1. P-values above the plots indicate significance of correlation (assessed by a linear model). (ZIP) [file pone.0167944.s009.zip › S4_File/LLKF_1319_real_dat.png]

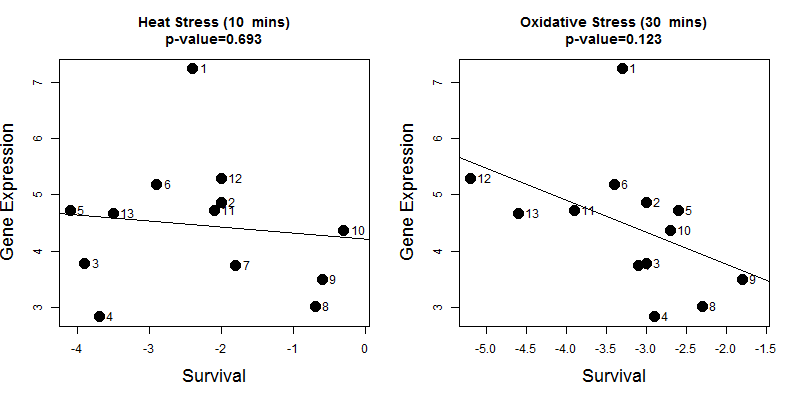

Supplement: S4 File — Expression levels of genes LLKF_1274 –LLKF_2533 and LLKF_p0001 –LLKF_p0036 plotted against survival after 10 minutes heat and 30 minutes oxidative stress. Survival is expressed as the difference of log CFU/ml after stress and before stress. Numbers indicate fermentations as presented in Table 1. P-values above the plots indicate significance of correlation (assessed by a linear model). (ZIP) [file pone.0167944.s009.zip › S4_File/LLKF_1320_real_dat.png]

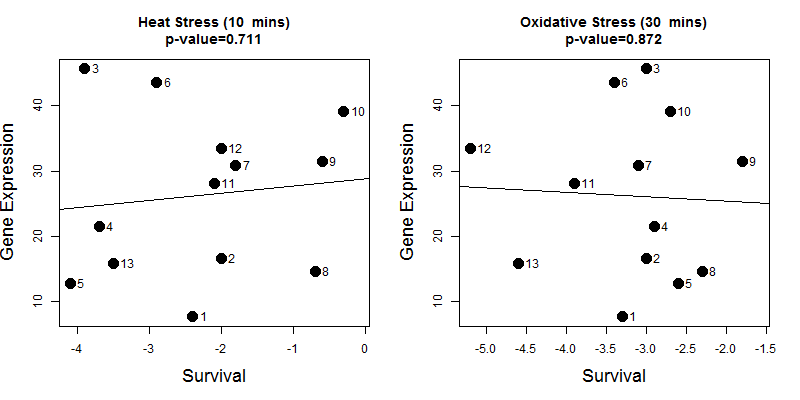

Supplement: S4 File — Expression levels of genes LLKF_1274 –LLKF_2533 and LLKF_p0001 –LLKF_p0036 plotted against survival after 10 minutes heat and 30 minutes oxidative stress. Survival is expressed as the difference of log CFU/ml after stress and before stress. Numbers indicate fermentations as presented in Table 1. P-values above the plots indicate significance of correlation (assessed by a linear model). (ZIP) [file pone.0167944.s009.zip › S4_File/LLKF_1321_real_dat.png]

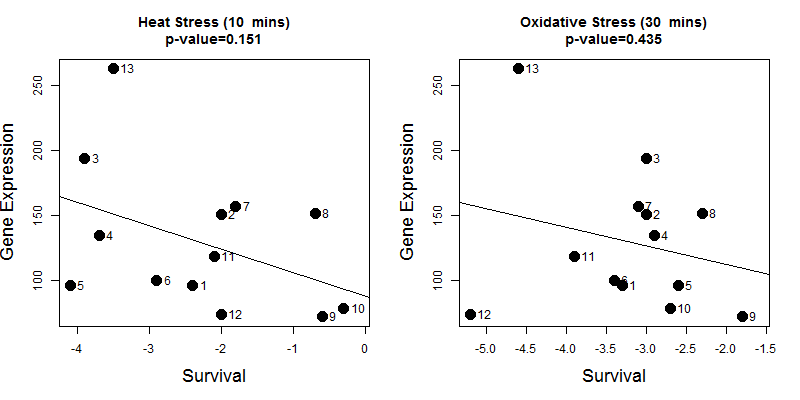

Supplement: S4 File — Expression levels of genes LLKF_1274 –LLKF_2533 and LLKF_p0001 –LLKF_p0036 plotted against survival after 10 minutes heat and 30 minutes oxidative stress. Survival is expressed as the difference of log CFU/ml after stress and before stress. Numbers indicate fermentations as presented in Table 1. P-values above the plots indicate significance of correlation (assessed by a linear model). (ZIP) [file pone.0167944.s009.zip › S4_File/LLKF_1322_real_dat.png]

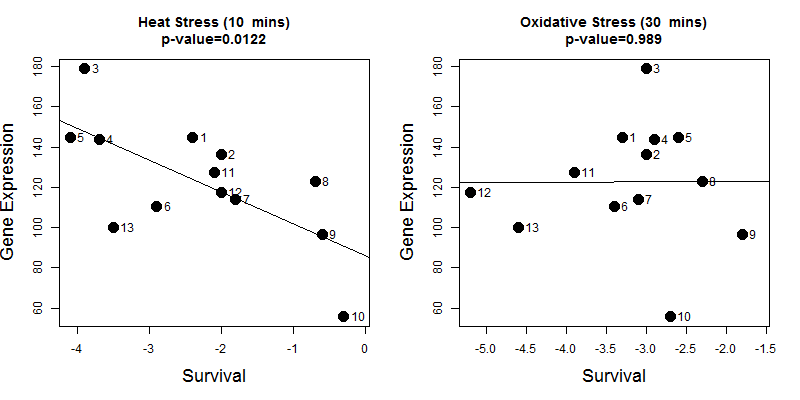

Supplement: S4 File — Expression levels of genes LLKF_1274 –LLKF_2533 and LLKF_p0001 –LLKF_p0036 plotted against survival after 10 minutes heat and 30 minutes oxidative stress. Survival is expressed as the difference of log CFU/ml after stress and before stress. Numbers indicate fermentations as presented in Table 1. P-values above the plots indicate significance of correlation (assessed by a linear model). (ZIP) [file pone.0167944.s009.zip › S4_File/LLKF_1323_real_dat.png]

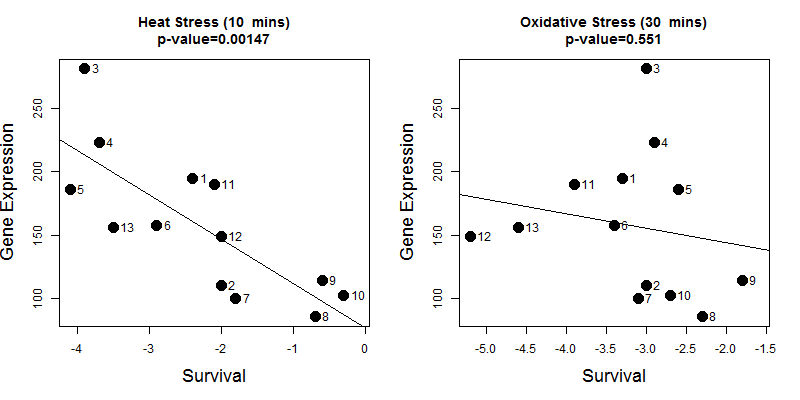

Supplement: S4 File — Expression levels of genes LLKF_1274 –LLKF_2533 and LLKF_p0001 –LLKF_p0036 plotted against survival after 10 minutes heat and 30 minutes oxidative stress. Survival is expressed as the difference of log CFU/ml after stress and before stress. Numbers indicate fermentations as presented in Table 1. P-values above the plots indicate significance of correlation (assessed by a linear model). (ZIP) [file pone.0167944.s009.zip › S4_File/LLKF_1324_real_dat.png]

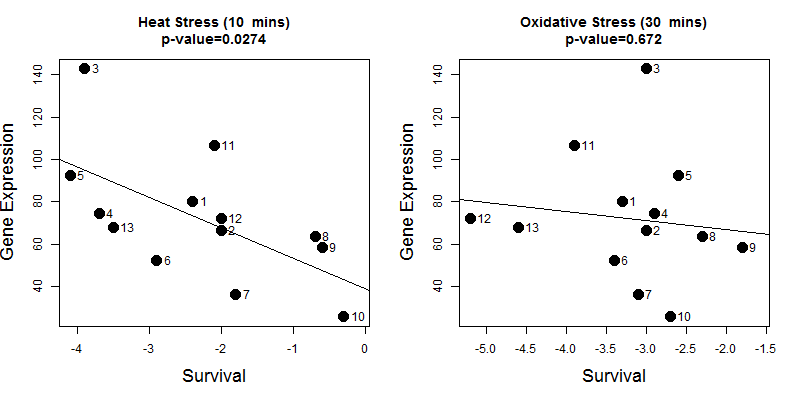

Supplement: S4 File — Expression levels of genes LLKF_1274 –LLKF_2533 and LLKF_p0001 –LLKF_p0036 plotted against survival after 10 minutes heat and 30 minutes oxidative stress. Survival is expressed as the difference of log CFU/ml after stress and before stress. Numbers indicate fermentations as presented in Table 1. P-values above the plots indicate significance of correlation (assessed by a linear model). (ZIP) [file pone.0167944.s009.zip › S4_File/LLKF_1325_real_dat.png]

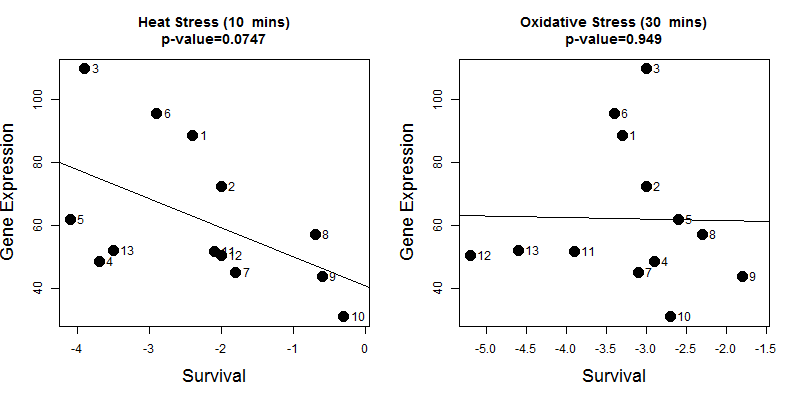

Supplement: S4 File — Expression levels of genes LLKF_1274 –LLKF_2533 and LLKF_p0001 –LLKF_p0036 plotted against survival after 10 minutes heat and 30 minutes oxidative stress. Survival is expressed as the difference of log CFU/ml after stress and before stress. Numbers indicate fermentations as presented in Table 1. P-values above the plots indicate significance of correlation (assessed by a linear model). (ZIP) [file pone.0167944.s009.zip › S4_File/LLKF_1326_real_dat.png]

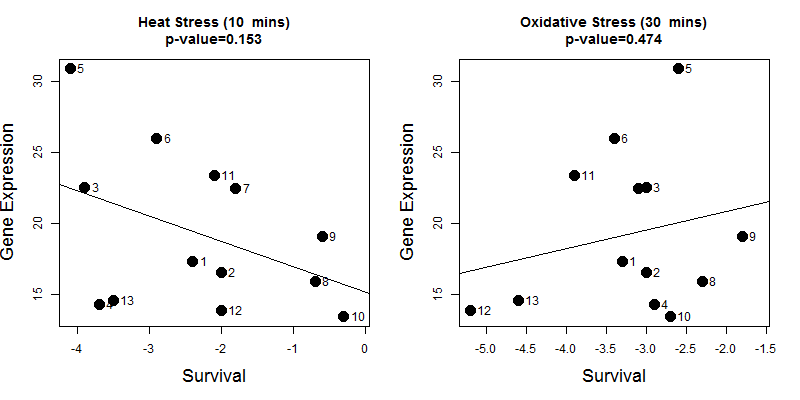

Supplement: S4 File — Expression levels of genes LLKF_1274 –LLKF_2533 and LLKF_p0001 –LLKF_p0036 plotted against survival after 10 minutes heat and 30 minutes oxidative stress. Survival is expressed as the difference of log CFU/ml after stress and before stress. Numbers indicate fermentations as presented in Table 1. P-values above the plots indicate significance of correlation (assessed by a linear model). (ZIP) [file pone.0167944.s009.zip › S4_File/LLKF_1327_real_dat.png]

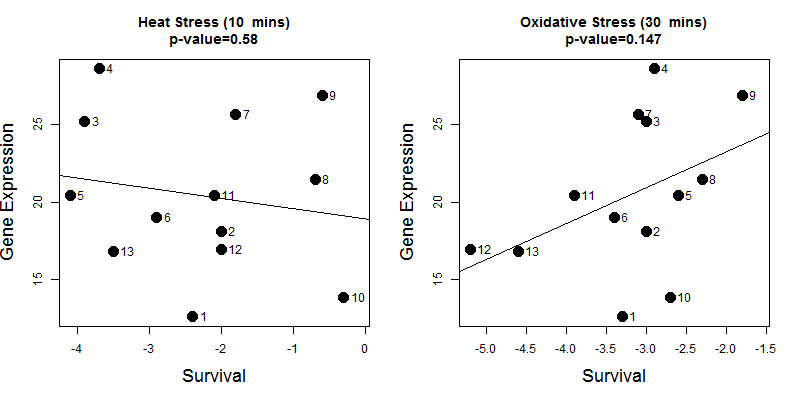

Supplement: S4 File — Expression levels of genes LLKF_1274 –LLKF_2533 and LLKF_p0001 –LLKF_p0036 plotted against survival after 10 minutes heat and 30 minutes oxidative stress. Survival is expressed as the difference of log CFU/ml after stress and before stress. Numbers indicate fermentations as presented in Table 1. P-values above the plots indicate significance of correlation (assessed by a linear model). (ZIP) [file pone.0167944.s009.zip › S4_File/LLKF_1328_real_dat.png]

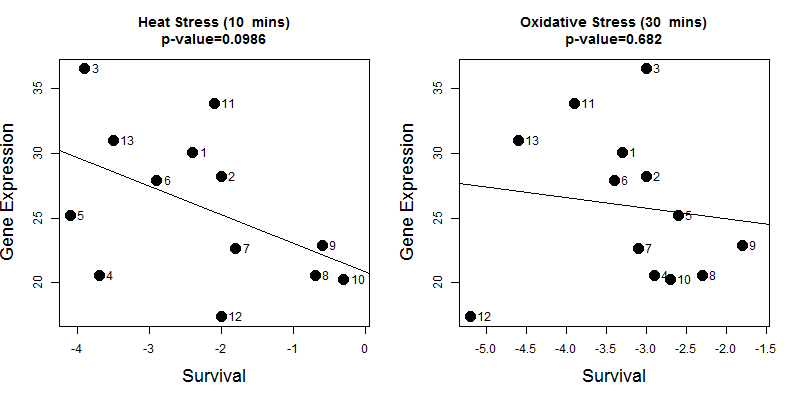

Supplement: S4 File — Expression levels of genes LLKF_1274 –LLKF_2533 and LLKF_p0001 –LLKF_p0036 plotted against survival after 10 minutes heat and 30 minutes oxidative stress. Survival is expressed as the difference of log CFU/ml after stress and before stress. Numbers indicate fermentations as presented in Table 1. P-values above the plots indicate significance of correlation (assessed by a linear model). (ZIP) [file pone.0167944.s009.zip › S4_File/LLKF_1329_real_dat.png]

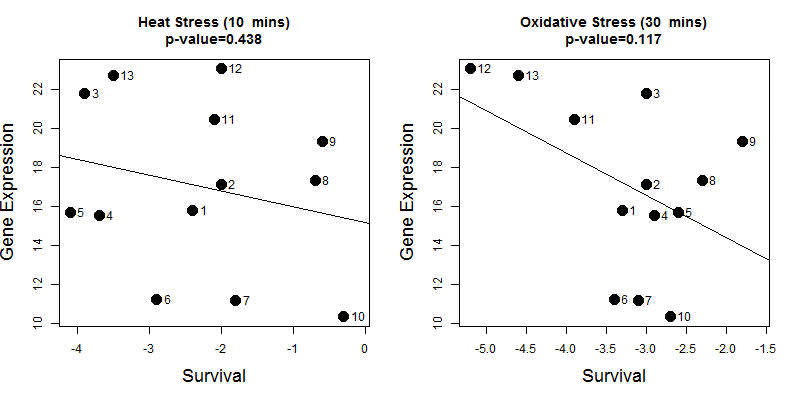

Supplement: S4 File — Expression levels of genes LLKF_1274 –LLKF_2533 and LLKF_p0001 –LLKF_p0036 plotted against survival after 10 minutes heat and 30 minutes oxidative stress. Survival is expressed as the difference of log CFU/ml after stress and before stress. Numbers indicate fermentations as presented in Table 1. P-values above the plots indicate significance of correlation (assessed by a linear model). (ZIP) [file pone.0167944.s009.zip › S4_File/LLKF_1330_real_dat.png]

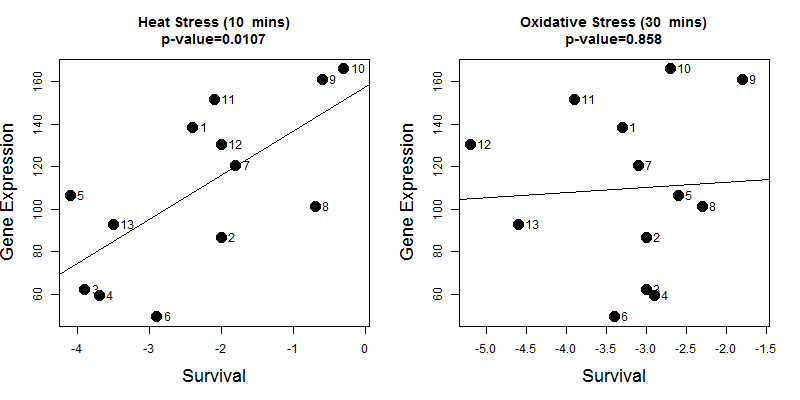

Supplement: S4 File — Expression levels of genes LLKF_1274 –LLKF_2533 and LLKF_p0001 –LLKF_p0036 plotted against survival after 10 minutes heat and 30 minutes oxidative stress. Survival is expressed as the difference of log CFU/ml after stress and before stress. Numbers indicate fermentations as presented in Table 1. P-values above the plots indicate significance of correlation (assessed by a linear model). (ZIP) [file pone.0167944.s009.zip › S4_File/LLKF_1331_real_dat.png]

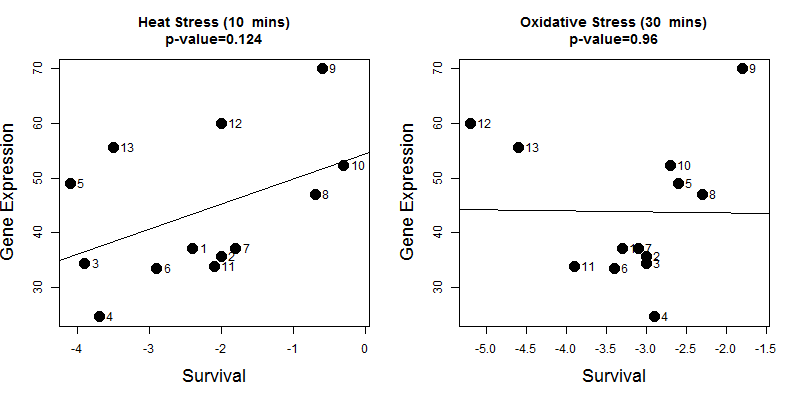

Supplement: S4 File — Expression levels of genes LLKF_1274 –LLKF_2533 and LLKF_p0001 –LLKF_p0036 plotted against survival after 10 minutes heat and 30 minutes oxidative stress. Survival is expressed as the difference of log CFU/ml after stress and before stress. Numbers indicate fermentations as presented in Table 1. P-values above the plots indicate significance of correlation (assessed by a linear model). (ZIP) [file pone.0167944.s009.zip › S4_File/LLKF_1332_real_dat.png]

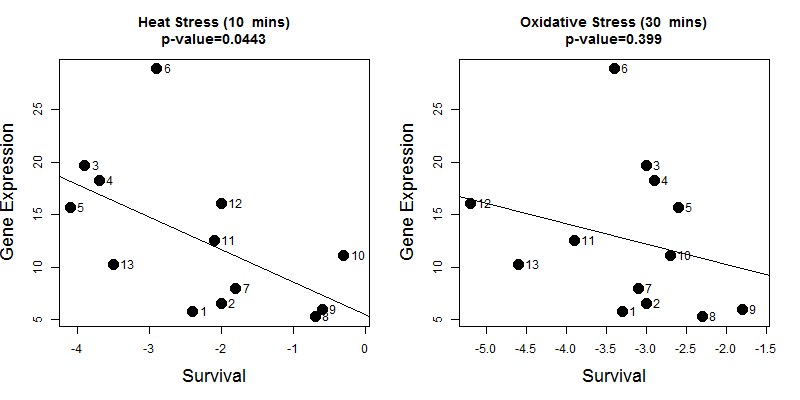

Supplement: S4 File — Expression levels of genes LLKF_1274 –LLKF_2533 and LLKF_p0001 –LLKF_p0036 plotted against survival after 10 minutes heat and 30 minutes oxidative stress. Survival is expressed as the difference of log CFU/ml after stress and before stress. Numbers indicate fermentations as presented in Table 1. P-values above the plots indicate significance of correlation (assessed by a linear model). (ZIP) [file pone.0167944.s009.zip › S4_File/LLKF_1333_real_dat.png]

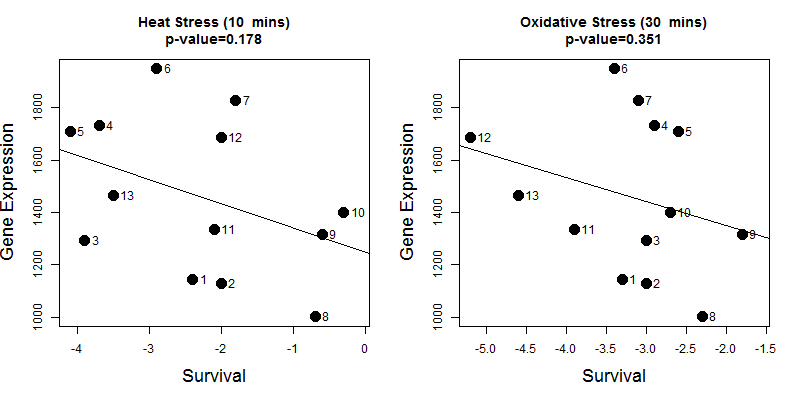

Supplement: S4 File — Expression levels of genes LLKF_1274 –LLKF_2533 and LLKF_p0001 –LLKF_p0036 plotted against survival after 10 minutes heat and 30 minutes oxidative stress. Survival is expressed as the difference of log CFU/ml after stress and before stress. Numbers indicate fermentations as presented in Table 1. P-values above the plots indicate significance of correlation (assessed by a linear model). (ZIP) [file pone.0167944.s009.zip › S4_File/LLKF_1334_real_dat.png]

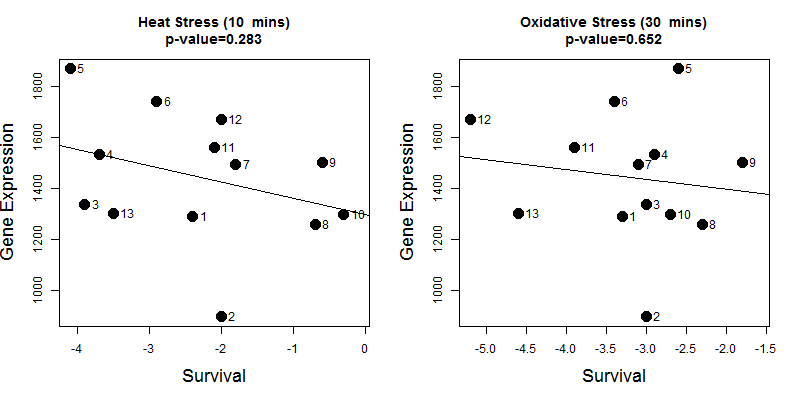

Supplement: S4 File — Expression levels of genes LLKF_1274 –LLKF_2533 and LLKF_p0001 –LLKF_p0036 plotted against survival after 10 minutes heat and 30 minutes oxidative stress. Survival is expressed as the difference of log CFU/ml after stress and before stress. Numbers indicate fermentations as presented in Table 1. P-values above the plots indicate significance of correlation (assessed by a linear model). (ZIP) [file pone.0167944.s009.zip › S4_File/LLKF_1335_real_dat.png]

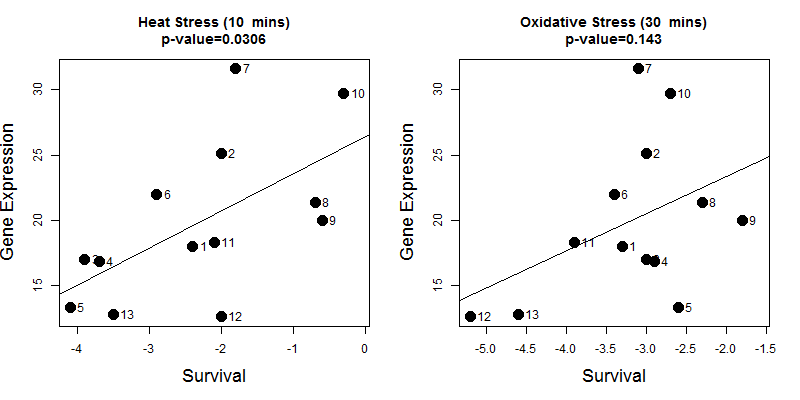

Supplement: S4 File — Expression levels of genes LLKF_1274 –LLKF_2533 and LLKF_p0001 –LLKF_p0036 plotted against survival after 10 minutes heat and 30 minutes oxidative stress. Survival is expressed as the difference of log CFU/ml after stress and before stress. Numbers indicate fermentations as presented in Table 1. P-values above the plots indicate significance of correlation (assessed by a linear model). (ZIP) [file pone.0167944.s009.zip › S4_File/LLKF_1336_real_dat.png]

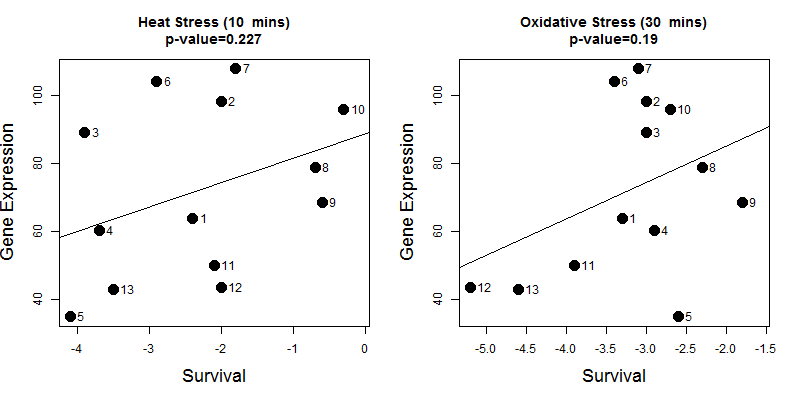

Supplement: S4 File — Expression levels of genes LLKF_1274 –LLKF_2533 and LLKF_p0001 –LLKF_p0036 plotted against survival after 10 minutes heat and 30 minutes oxidative stress. Survival is expressed as the difference of log CFU/ml after stress and before stress. Numbers indicate fermentations as presented in Table 1. P-values above the plots indicate significance of correlation (assessed by a linear model). (ZIP) [file pone.0167944.s009.zip › S4_File/LLKF_1337_real_dat.png]

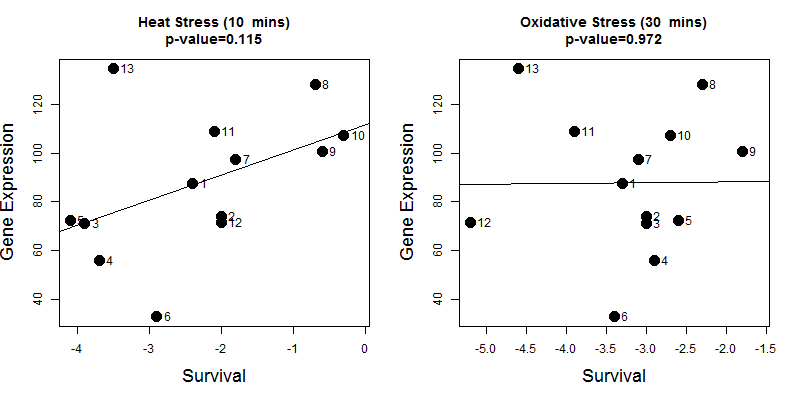

Supplement: S4 File — Expression levels of genes LLKF_1274 –LLKF_2533 and LLKF_p0001 –LLKF_p0036 plotted against survival after 10 minutes heat and 30 minutes oxidative stress. Survival is expressed as the difference of log CFU/ml after stress and before stress. Numbers indicate fermentations as presented in Table 1. P-values above the plots indicate significance of correlation (assessed by a linear model). (ZIP) [file pone.0167944.s009.zip › S4_File/LLKF_1338_real_dat.png]

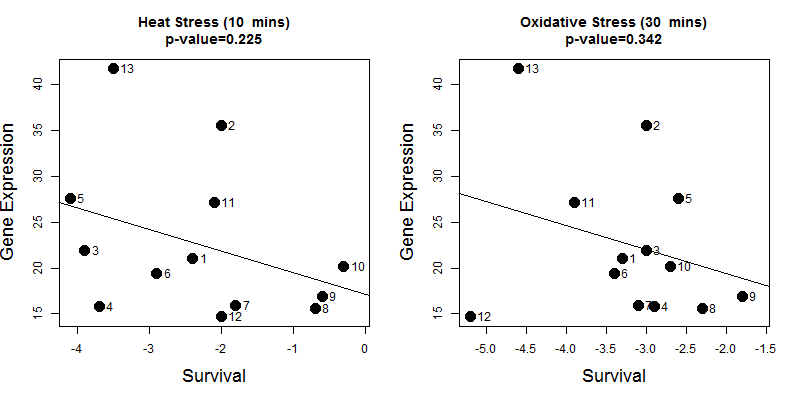

Supplement: S4 File — Expression levels of genes LLKF_1274 –LLKF_2533 and LLKF_p0001 –LLKF_p0036 plotted against survival after 10 minutes heat and 30 minutes oxidative stress. Survival is expressed as the difference of log CFU/ml after stress and before stress. Numbers indicate fermentations as presented in Table 1. P-values above the plots indicate significance of correlation (assessed by a linear model). (ZIP) [file pone.0167944.s009.zip › S4_File/LLKF_1339_real_dat.png]

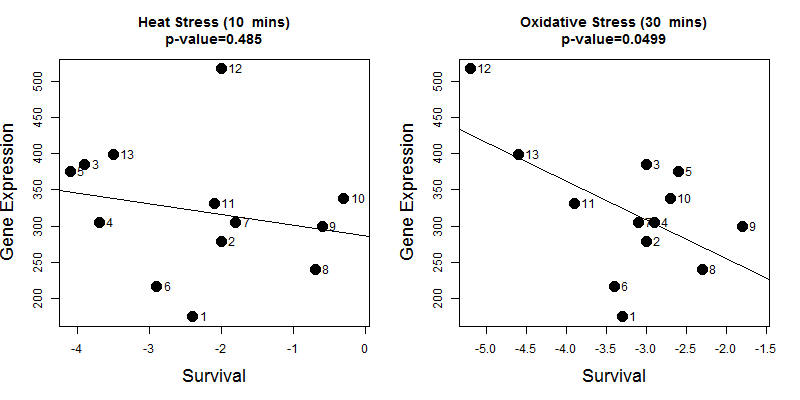

Supplement: S4 File — Expression levels of genes LLKF_1274 –LLKF_2533 and LLKF_p0001 –LLKF_p0036 plotted against survival after 10 minutes heat and 30 minutes oxidative stress. Survival is expressed as the difference of log CFU/ml after stress and before stress. Numbers indicate fermentations as presented in Table 1. P-values above the plots indicate significance of correlation (assessed by a linear model). (ZIP) [file pone.0167944.s009.zip › S4_File/LLKF_1340_real_dat.png]

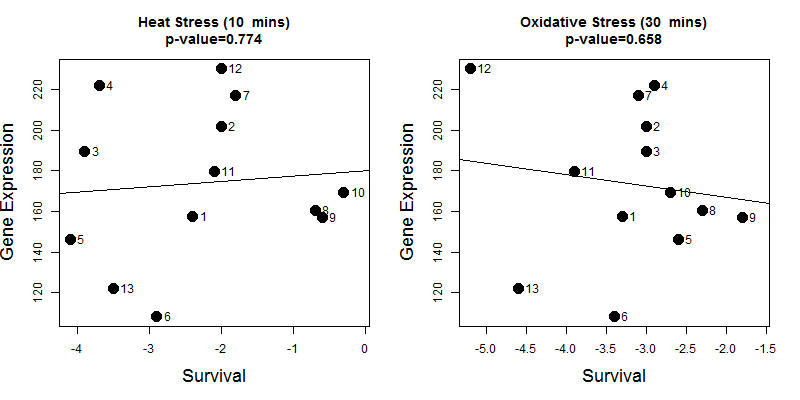

Supplement: S4 File — Expression levels of genes LLKF_1274 –LLKF_2533 and LLKF_p0001 –LLKF_p0036 plotted against survival after 10 minutes heat and 30 minutes oxidative stress. Survival is expressed as the difference of log CFU/ml after stress and before stress. Numbers indicate fermentations as presented in Table 1. P-values above the plots indicate significance of correlation (assessed by a linear model). (ZIP) [file pone.0167944.s009.zip › S4_File/LLKF_1341_real_dat.png]

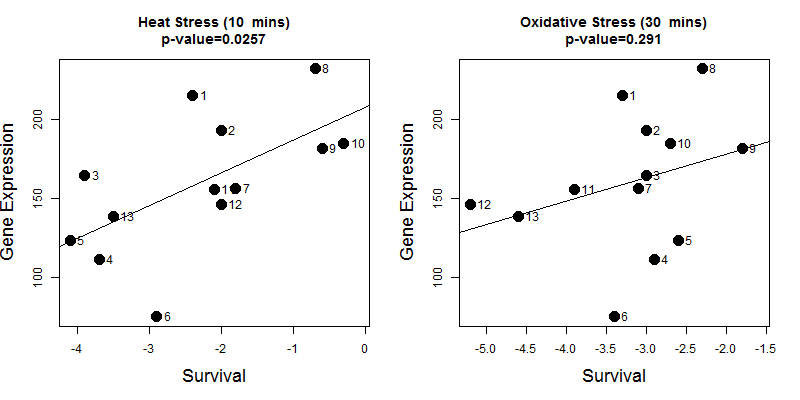

Supplement: S4 File — Expression levels of genes LLKF_1274 –LLKF_2533 and LLKF_p0001 –LLKF_p0036 plotted against survival after 10 minutes heat and 30 minutes oxidative stress. Survival is expressed as the difference of log CFU/ml after stress and before stress. Numbers indicate fermentations as presented in Table 1. P-values above the plots indicate significance of correlation (assessed by a linear model). (ZIP) [file pone.0167944.s009.zip › S4_File/LLKF_1342_real_dat.png]

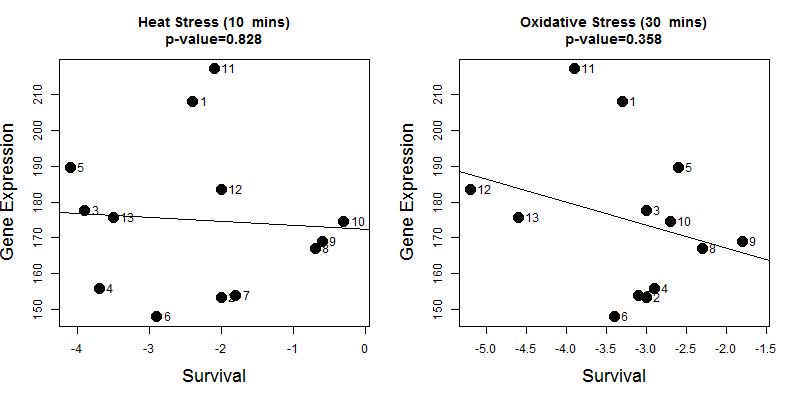

Supplement: S4 File — Expression levels of genes LLKF_1274 –LLKF_2533 and LLKF_p0001 –LLKF_p0036 plotted against survival after 10 minutes heat and 30 minutes oxidative stress. Survival is expressed as the difference of log CFU/ml after stress and before stress. Numbers indicate fermentations as presented in Table 1. P-values above the plots indicate significance of correlation (assessed by a linear model). (ZIP) [file pone.0167944.s009.zip › S4_File/LLKF_1343_real_dat.png]

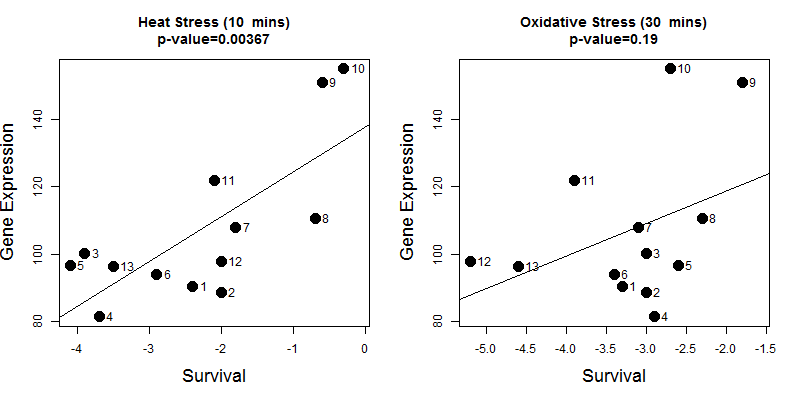

Supplement: S4 File — Expression levels of genes LLKF_1274 –LLKF_2533 and LLKF_p0001 –LLKF_p0036 plotted against survival after 10 minutes heat and 30 minutes oxidative stress. Survival is expressed as the difference of log CFU/ml after stress and before stress. Numbers indicate fermentations as presented in Table 1. P-values above the plots indicate significance of correlation (assessed by a linear model). (ZIP) [file pone.0167944.s009.zip › S4_File/LLKF_1344_real_dat.png]

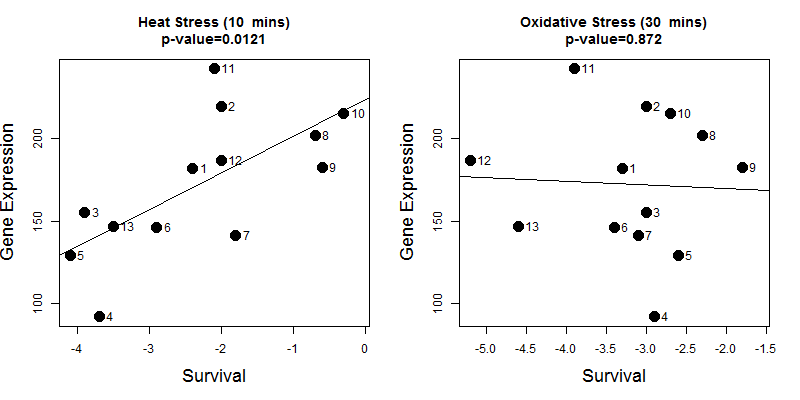

Supplement: S4 File — Expression levels of genes LLKF_1274 –LLKF_2533 and LLKF_p0001 –LLKF_p0036 plotted against survival after 10 minutes heat and 30 minutes oxidative stress. Survival is expressed as the difference of log CFU/ml after stress and before stress. Numbers indicate fermentations as presented in Table 1. P-values above the plots indicate significance of correlation (assessed by a linear model). (ZIP) [file pone.0167944.s009.zip › S4_File/LLKF_1345_real_dat.png]

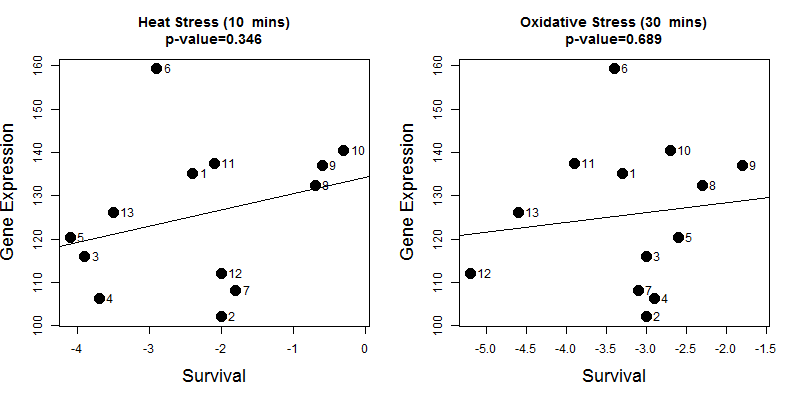

Supplement: S4 File — Expression levels of genes LLKF_1274 –LLKF_2533 and LLKF_p0001 –LLKF_p0036 plotted against survival after 10 minutes heat and 30 minutes oxidative stress. Survival is expressed as the difference of log CFU/ml after stress and before stress. Numbers indicate fermentations as presented in Table 1. P-values above the plots indicate significance of correlation (assessed by a linear model). (ZIP) [file pone.0167944.s009.zip › S4_File/LLKF_1346_real_dat.png]

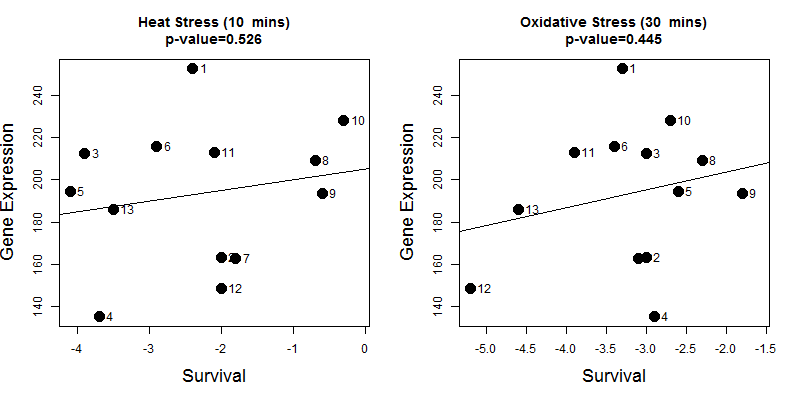

Supplement: S4 File — Expression levels of genes LLKF_1274 –LLKF_2533 and LLKF_p0001 –LLKF_p0036 plotted against survival after 10 minutes heat and 30 minutes oxidative stress. Survival is expressed as the difference of log CFU/ml after stress and before stress. Numbers indicate fermentations as presented in Table 1. P-values above the plots indicate significance of correlation (assessed by a linear model). (ZIP) [file pone.0167944.s009.zip › S4_File/LLKF_1347_real_dat.png]

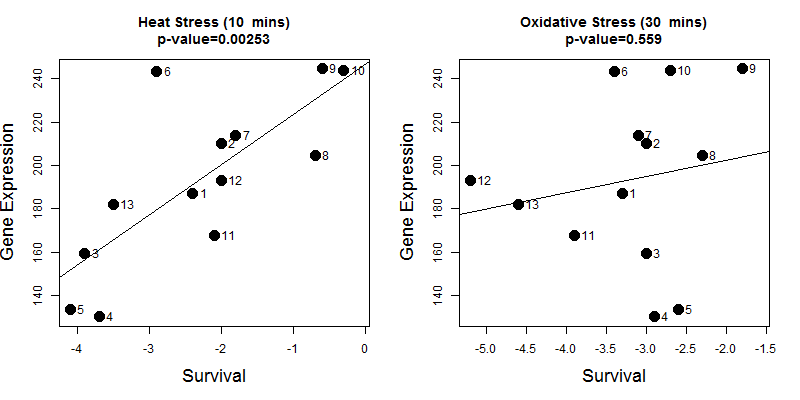

Supplement: S4 File — Expression levels of genes LLKF_1274 –LLKF_2533 and LLKF_p0001 –LLKF_p0036 plotted against survival after 10 minutes heat and 30 minutes oxidative stress. Survival is expressed as the difference of log CFU/ml after stress and before stress. Numbers indicate fermentations as presented in Table 1. P-values above the plots indicate significance of correlation (assessed by a linear model). (ZIP) [file pone.0167944.s009.zip › S4_File/LLKF_1348_real_dat.png]

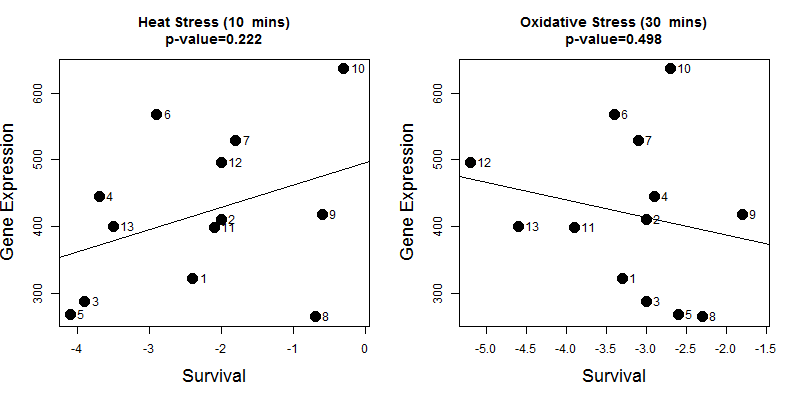

Supplement: S4 File — Expression levels of genes LLKF_1274 –LLKF_2533 and LLKF_p0001 –LLKF_p0036 plotted against survival after 10 minutes heat and 30 minutes oxidative stress. Survival is expressed as the difference of log CFU/ml after stress and before stress. Numbers indicate fermentations as presented in Table 1. P-values above the plots indicate significance of correlation (assessed by a linear model). (ZIP) [file pone.0167944.s009.zip › S4_File/LLKF_1349_real_dat.png]

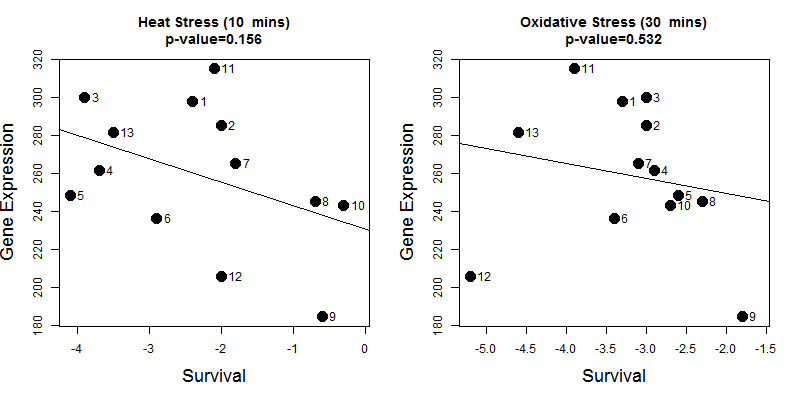

Supplement: S4 File — Expression levels of genes LLKF_1274 –LLKF_2533 and LLKF_p0001 –LLKF_p0036 plotted against survival after 10 minutes heat and 30 minutes oxidative stress. Survival is expressed as the difference of log CFU/ml after stress and before stress. Numbers indicate fermentations as presented in Table 1. P-values above the plots indicate significance of correlation (assessed by a linear model). (ZIP) [file pone.0167944.s009.zip › S4_File/LLKF_1350_real_dat.png]

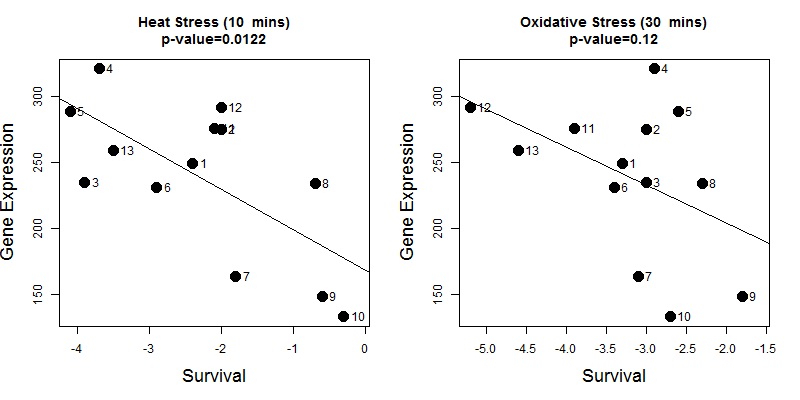

Supplement: S4 File — Expression levels of genes LLKF_1274 –LLKF_2533 and LLKF_p0001 –LLKF_p0036 plotted against survival after 10 minutes heat and 30 minutes oxidative stress. Survival is expressed as the difference of log CFU/ml after stress and before stress. Numbers indicate fermentations as presented in Table 1. P-values above the plots indicate significance of correlation (assessed by a linear model). (ZIP) [file pone.0167944.s009.zip › S4_File/LLKF_1351_real_dat.png]

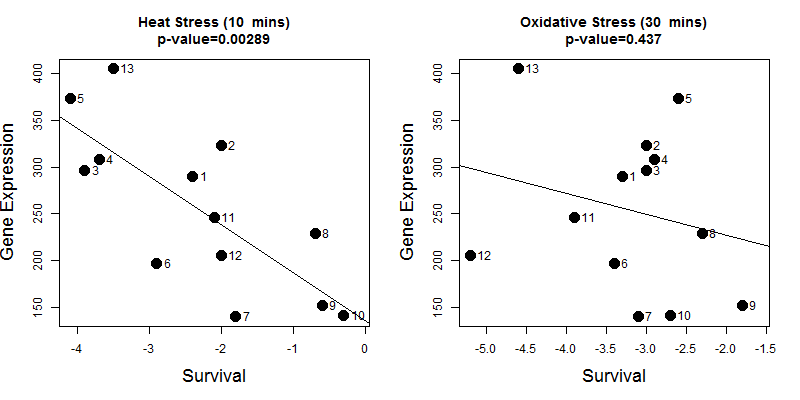

Supplement: S4 File — Expression levels of genes LLKF_1274 –LLKF_2533 and LLKF_p0001 –LLKF_p0036 plotted against survival after 10 minutes heat and 30 minutes oxidative stress. Survival is expressed as the difference of log CFU/ml after stress and before stress. Numbers indicate fermentations as presented in Table 1. P-values above the plots indicate significance of correlation (assessed by a linear model). (ZIP) [file pone.0167944.s009.zip › S4_File/LLKF_1352_real_dat.png]

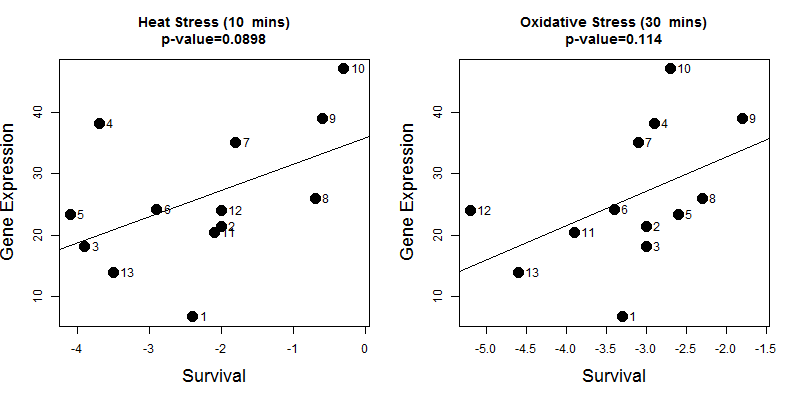

Supplement: S4 File — Expression levels of genes LLKF_1274 –LLKF_2533 and LLKF_p0001 –LLKF_p0036 plotted against survival after 10 minutes heat and 30 minutes oxidative stress. Survival is expressed as the difference of log CFU/ml after stress and before stress. Numbers indicate fermentations as presented in Table 1. P-values above the plots indicate significance of correlation (assessed by a linear model). (ZIP) [file pone.0167944.s009.zip › S4_File/LLKF_1353_real_dat.png]

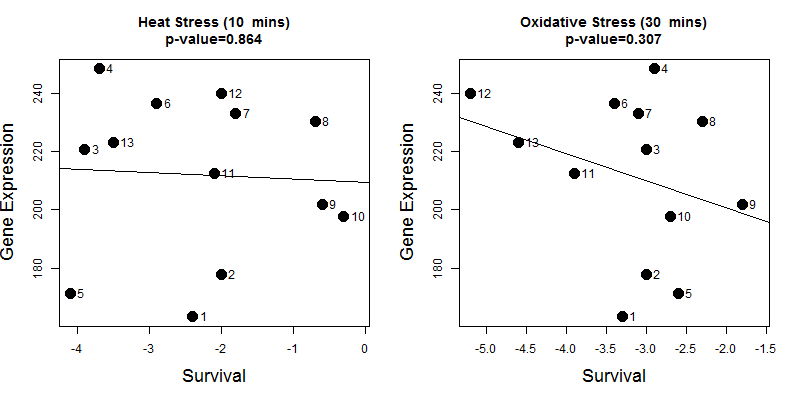

Supplement: S4 File — Expression levels of genes LLKF_1274 –LLKF_2533 and LLKF_p0001 –LLKF_p0036 plotted against survival after 10 minutes heat and 30 minutes oxidative stress. Survival is expressed as the difference of log CFU/ml after stress and before stress. Numbers indicate fermentations as presented in Table 1. P-values above the plots indicate significance of correlation (assessed by a linear model). (ZIP) [file pone.0167944.s009.zip › S4_File/LLKF_1354_real_dat.png]

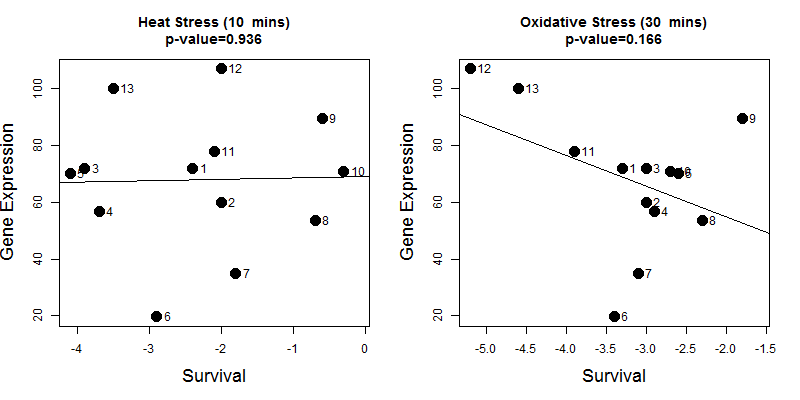

Supplement: S4 File — Expression levels of genes LLKF_1274 –LLKF_2533 and LLKF_p0001 –LLKF_p0036 plotted against survival after 10 minutes heat and 30 minutes oxidative stress. Survival is expressed as the difference of log CFU/ml after stress and before stress. Numbers indicate fermentations as presented in Table 1. P-values above the plots indicate significance of correlation (assessed by a linear model). (ZIP) [file pone.0167944.s009.zip › S4_File/LLKF_1355_real_dat.png]

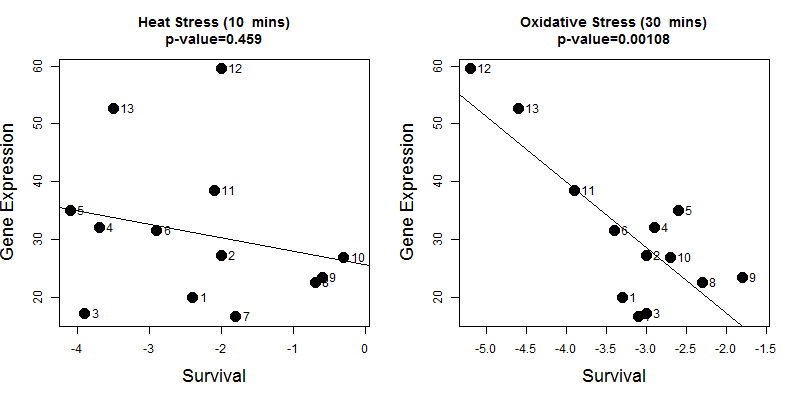

Supplement: S4 File — Expression levels of genes LLKF_1274 –LLKF_2533 and LLKF_p0001 –LLKF_p0036 plotted against survival after 10 minutes heat and 30 minutes oxidative stress. Survival is expressed as the difference of log CFU/ml after stress and before stress. Numbers indicate fermentations as presented in Table 1. P-values above the plots indicate significance of correlation (assessed by a linear model). (ZIP) [file pone.0167944.s009.zip › S4_File/LLKF_1356_real_dat.png]

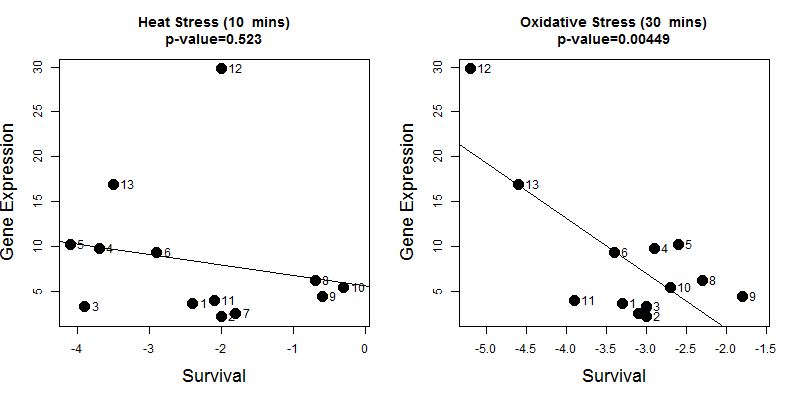

Supplement: S4 File — Expression levels of genes LLKF_1274 –LLKF_2533 and LLKF_p0001 –LLKF_p0036 plotted against survival after 10 minutes heat and 30 minutes oxidative stress. Survival is expressed as the difference of log CFU/ml after stress and before stress. Numbers indicate fermentations as presented in Table 1. P-values above the plots indicate significance of correlation (assessed by a linear model). (ZIP) [file pone.0167944.s009.zip › S4_File/LLKF_1357_real_dat.png]

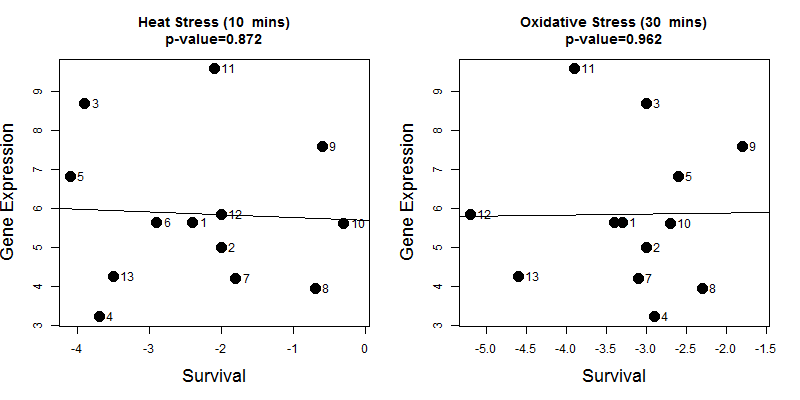

Supplement: S4 File — Expression levels of genes LLKF_1274 –LLKF_2533 and LLKF_p0001 –LLKF_p0036 plotted against survival after 10 minutes heat and 30 minutes oxidative stress. Survival is expressed as the difference of log CFU/ml after stress and before stress. Numbers indicate fermentations as presented in Table 1. P-values above the plots indicate significance of correlation (assessed by a linear model). (ZIP) [file pone.0167944.s009.zip › S4_File/LLKF_1358_real_dat.png]

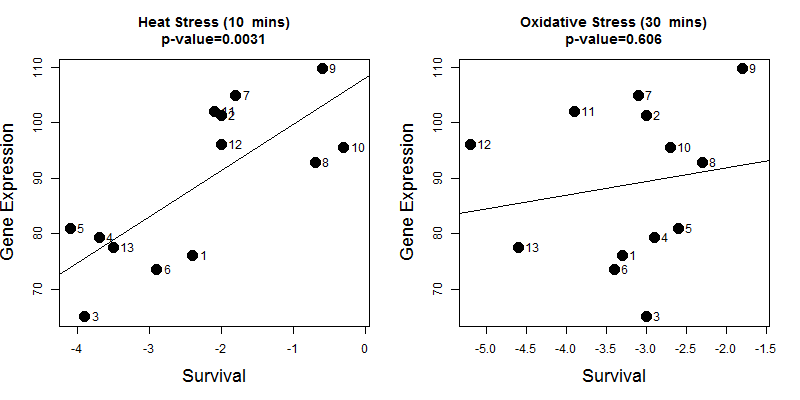

Supplement: S4 File — Expression levels of genes LLKF_1274 –LLKF_2533 and LLKF_p0001 –LLKF_p0036 plotted against survival after 10 minutes heat and 30 minutes oxidative stress. Survival is expressed as the difference of log CFU/ml after stress and before stress. Numbers indicate fermentations as presented in Table 1. P-values above the plots indicate significance of correlation (assessed by a linear model). (ZIP) [file pone.0167944.s009.zip › S4_File/LLKF_1359_real_dat.png]

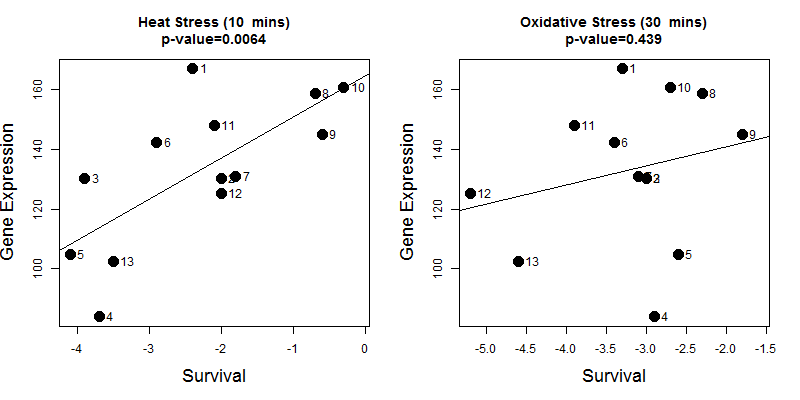

Supplement: S4 File — Expression levels of genes LLKF_1274 –LLKF_2533 and LLKF_p0001 –LLKF_p0036 plotted against survival after 10 minutes heat and 30 minutes oxidative stress. Survival is expressed as the difference of log CFU/ml after stress and before stress. Numbers indicate fermentations as presented in Table 1. P-values above the plots indicate significance of correlation (assessed by a linear model). (ZIP) [file pone.0167944.s009.zip › S4_File/LLKF_1360_real_dat.png]

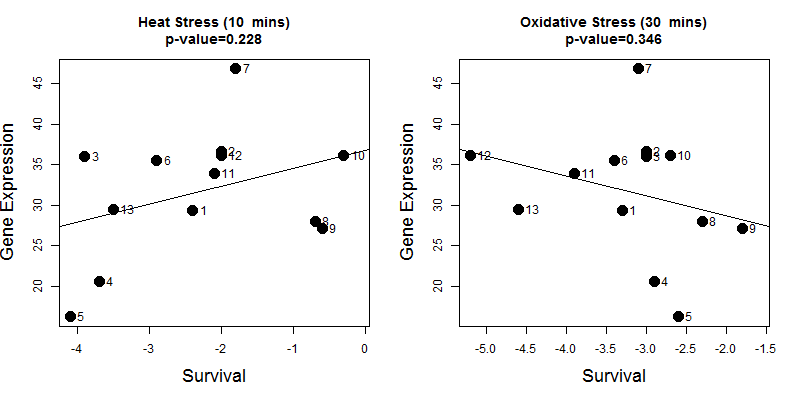

Supplement: S4 File — Expression levels of genes LLKF_1274 –LLKF_2533 and LLKF_p0001 –LLKF_p0036 plotted against survival after 10 minutes heat and 30 minutes oxidative stress. Survival is expressed as the difference of log CFU/ml after stress and before stress. Numbers indicate fermentations as presented in Table 1. P-values above the plots indicate significance of correlation (assessed by a linear model). (ZIP) [file pone.0167944.s009.zip › S4_File/LLKF_1361_real_dat.png]

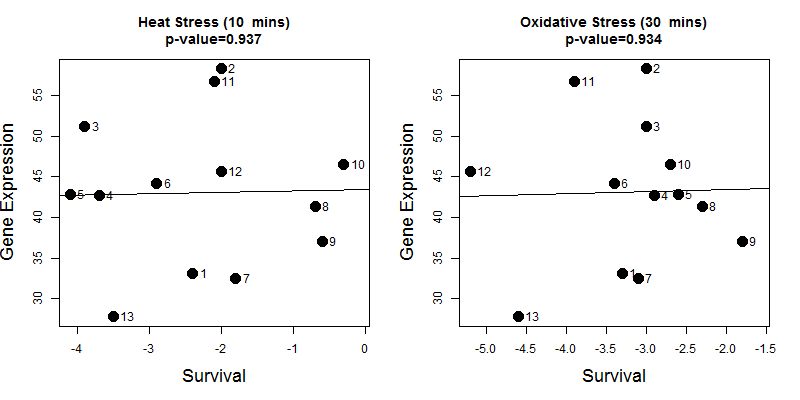

Supplement: S4 File — Expression levels of genes LLKF_1274 –LLKF_2533 and LLKF_p0001 –LLKF_p0036 plotted against survival after 10 minutes heat and 30 minutes oxidative stress. Survival is expressed as the difference of log CFU/ml after stress and before stress. Numbers indicate fermentations as presented in Table 1. P-values above the plots indicate significance of correlation (assessed by a linear model). (ZIP) [file pone.0167944.s009.zip › S4_File/LLKF_1362_real_dat.png]

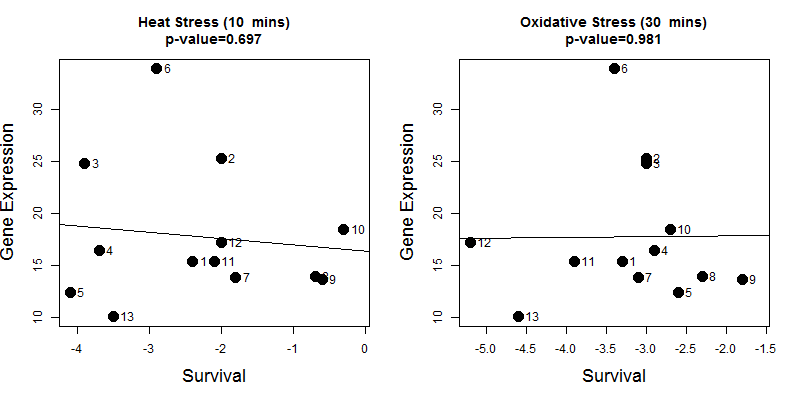

Supplement: S4 File — Expression levels of genes LLKF_1274 –LLKF_2533 and LLKF_p0001 –LLKF_p0036 plotted against survival after 10 minutes heat and 30 minutes oxidative stress. Survival is expressed as the difference of log CFU/ml after stress and before stress. Numbers indicate fermentations as presented in Table 1. P-values above the plots indicate significance of correlation (assessed by a linear model). (ZIP) [file pone.0167944.s009.zip › S4_File/LLKF_1363_real_dat.png]

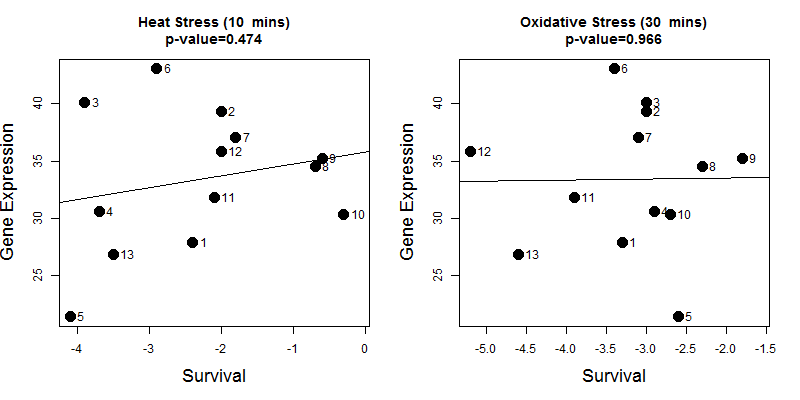

Supplement: S4 File — Expression levels of genes LLKF_1274 –LLKF_2533 and LLKF_p0001 –LLKF_p0036 plotted against survival after 10 minutes heat and 30 minutes oxidative stress. Survival is expressed as the difference of log CFU/ml after stress and before stress. Numbers indicate fermentations as presented in Table 1. P-values above the plots indicate significance of correlation (assessed by a linear model). (ZIP) [file pone.0167944.s009.zip › S4_File/LLKF_1364_real_dat.png]

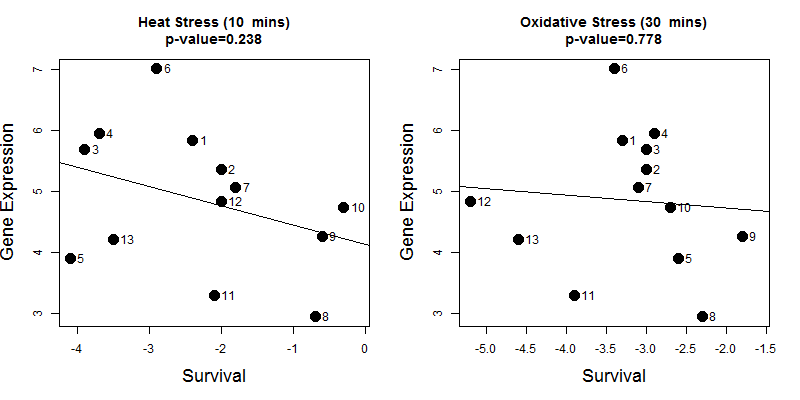

Supplement: S4 File — Expression levels of genes LLKF_1274 –LLKF_2533 and LLKF_p0001 –LLKF_p0036 plotted against survival after 10 minutes heat and 30 minutes oxidative stress. Survival is expressed as the difference of log CFU/ml after stress and before stress. Numbers indicate fermentations as presented in Table 1. P-values above the plots indicate significance of correlation (assessed by a linear model). (ZIP) [file pone.0167944.s009.zip › S4_File/LLKF_1365_real_dat.png]

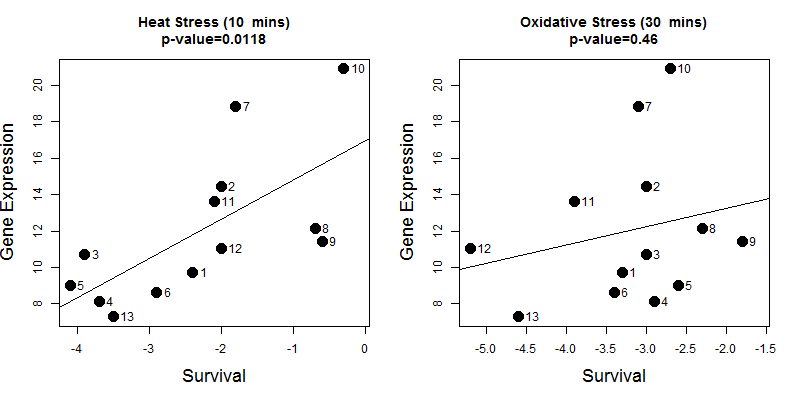

Supplement: S4 File — Expression levels of genes LLKF_1274 –LLKF_2533 and LLKF_p0001 –LLKF_p0036 plotted against survival after 10 minutes heat and 30 minutes oxidative stress. Survival is expressed as the difference of log CFU/ml after stress and before stress. Numbers indicate fermentations as presented in Table 1. P-values above the plots indicate significance of correlation (assessed by a linear model). (ZIP) [file pone.0167944.s009.zip › S4_File/LLKF_1366_real_dat.png]

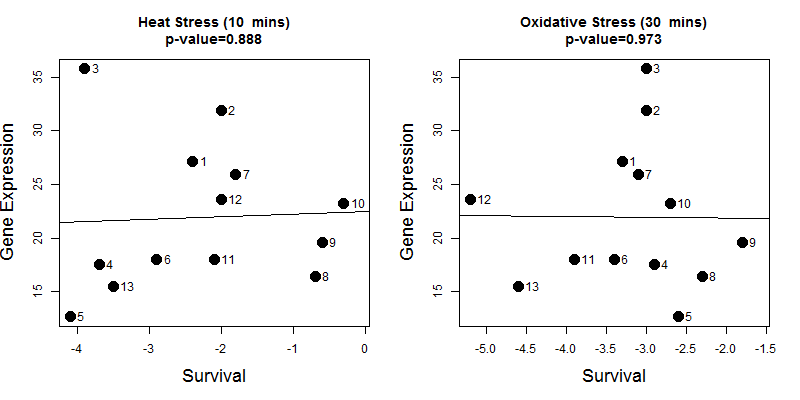

Supplement: S4 File — Expression levels of genes LLKF_1274 –LLKF_2533 and LLKF_p0001 –LLKF_p0036 plotted against survival after 10 minutes heat and 30 minutes oxidative stress. Survival is expressed as the difference of log CFU/ml after stress and before stress. Numbers indicate fermentations as presented in Table 1. P-values above the plots indicate significance of correlation (assessed by a linear model). (ZIP) [file pone.0167944.s009.zip › S4_File/LLKF_1367_real_dat.png]

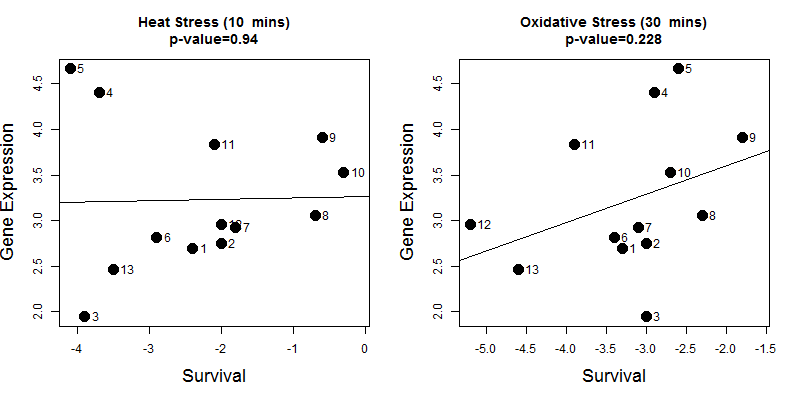

Supplement: S4 File — Expression levels of genes LLKF_1274 –LLKF_2533 and LLKF_p0001 –LLKF_p0036 plotted against survival after 10 minutes heat and 30 minutes oxidative stress. Survival is expressed as the difference of log CFU/ml after stress and before stress. Numbers indicate fermentations as presented in Table 1. P-values above the plots indicate significance of correlation (assessed by a linear model). (ZIP) [file pone.0167944.s009.zip › S4_File/LLKF_1368_real_dat.png]

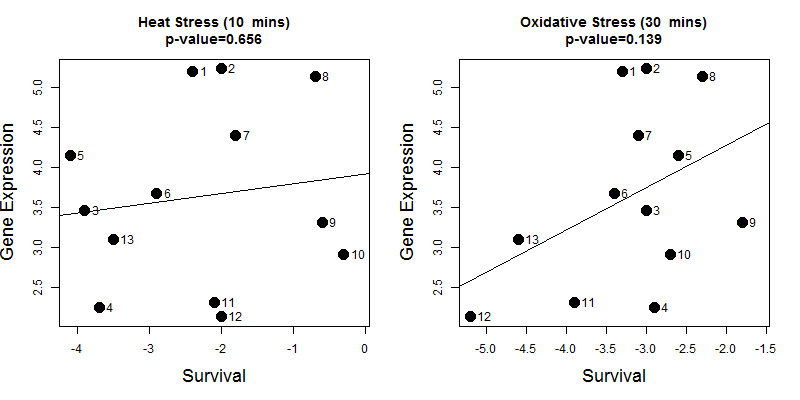

Supplement: S4 File — Expression levels of genes LLKF_1274 –LLKF_2533 and LLKF_p0001 –LLKF_p0036 plotted against survival after 10 minutes heat and 30 minutes oxidative stress. Survival is expressed as the difference of log CFU/ml after stress and before stress. Numbers indicate fermentations as presented in Table 1. P-values above the plots indicate significance of correlation (assessed by a linear model). (ZIP) [file pone.0167944.s009.zip › S4_File/LLKF_1369_real_dat.png]

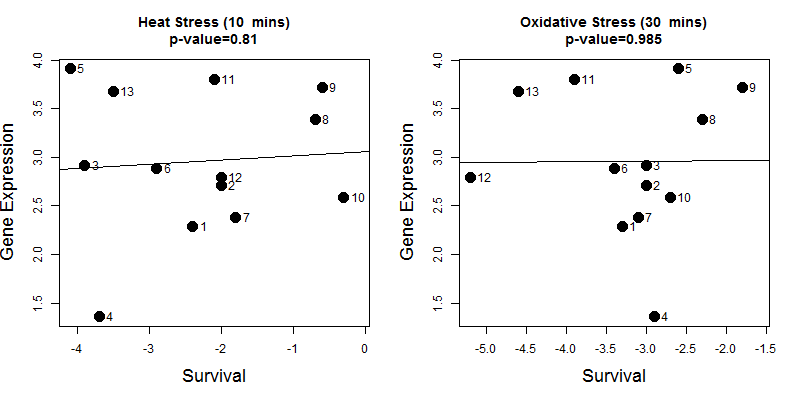

Supplement: S4 File — Expression levels of genes LLKF_1274 –LLKF_2533 and LLKF_p0001 –LLKF_p0036 plotted against survival after 10 minutes heat and 30 minutes oxidative stress. Survival is expressed as the difference of log CFU/ml after stress and before stress. Numbers indicate fermentations as presented in Table 1. P-values above the plots indicate significance of correlation (assessed by a linear model). (ZIP) [file pone.0167944.s009.zip › S4_File/LLKF_1370_real_dat.png]

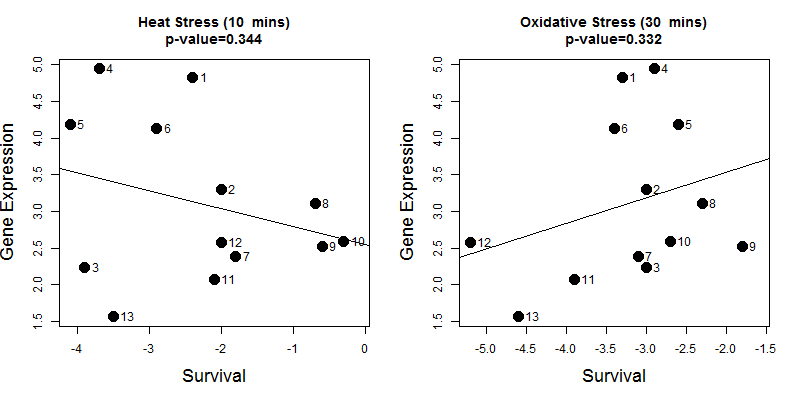

Supplement: S4 File — Expression levels of genes LLKF_1274 –LLKF_2533 and LLKF_p0001 –LLKF_p0036 plotted against survival after 10 minutes heat and 30 minutes oxidative stress. Survival is expressed as the difference of log CFU/ml after stress and before stress. Numbers indicate fermentations as presented in Table 1. P-values above the plots indicate significance of correlation (assessed by a linear model). (ZIP) [file pone.0167944.s009.zip › S4_File/LLKF_1371_real_dat.png]

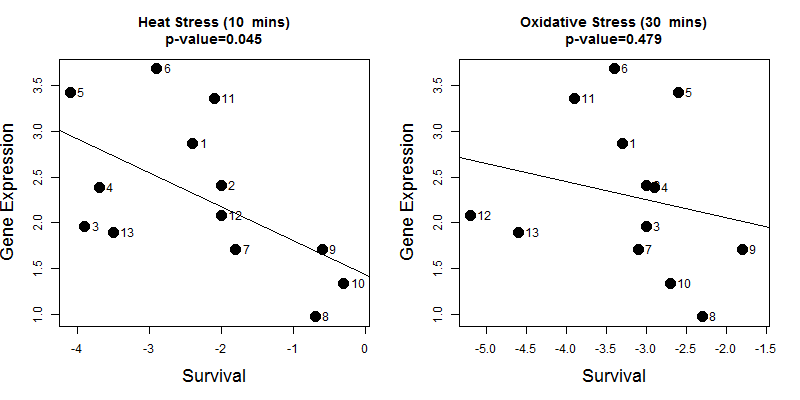

Supplement: S4 File — Expression levels of genes LLKF_1274 –LLKF_2533 and LLKF_p0001 –LLKF_p0036 plotted against survival after 10 minutes heat and 30 minutes oxidative stress. Survival is expressed as the difference of log CFU/ml after stress and before stress. Numbers indicate fermentations as presented in Table 1. P-values above the plots indicate significance of correlation (assessed by a linear model). (ZIP) [file pone.0167944.s009.zip › S4_File/LLKF_1372_real_dat.png]

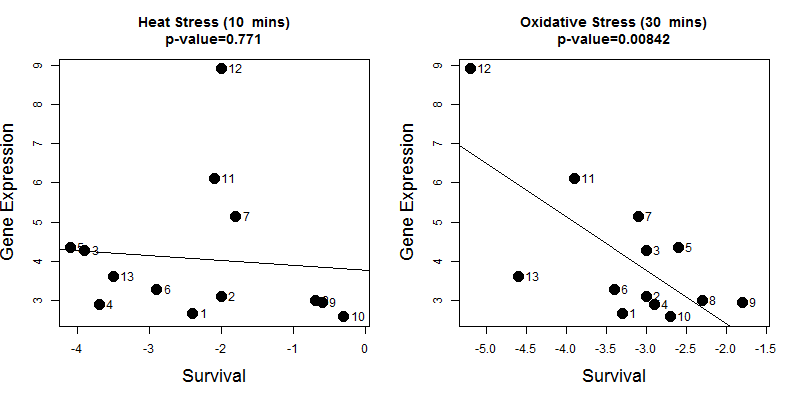

Supplement: S4 File — Expression levels of genes LLKF_1274 –LLKF_2533 and LLKF_p0001 –LLKF_p0036 plotted against survival after 10 minutes heat and 30 minutes oxidative stress. Survival is expressed as the difference of log CFU/ml after stress and before stress. Numbers indicate fermentations as presented in Table 1. P-values above the plots indicate significance of correlation (assessed by a linear model). (ZIP) [file pone.0167944.s009.zip › S4_File/LLKF_1373_real_dat.png]

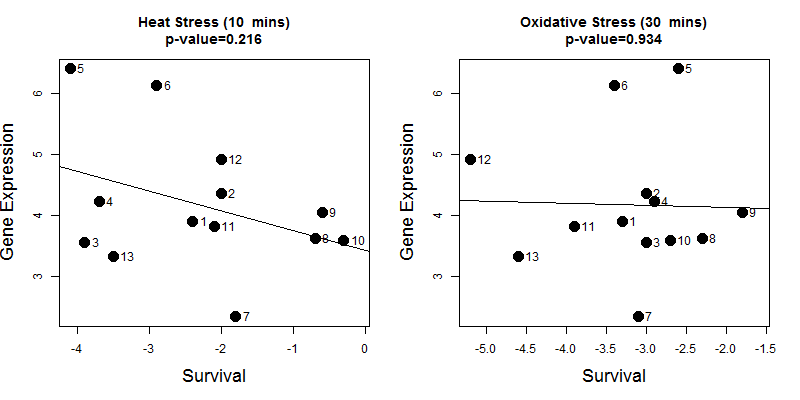

Supplement: S4 File — Expression levels of genes LLKF_1274 –LLKF_2533 and LLKF_p0001 –LLKF_p0036 plotted against survival after 10 minutes heat and 30 minutes oxidative stress. Survival is expressed as the difference of log CFU/ml after stress and before stress. Numbers indicate fermentations as presented in Table 1. P-values above the plots indicate significance of correlation (assessed by a linear model). (ZIP) [file pone.0167944.s009.zip › S4_File/LLKF_1374_real_dat.png]

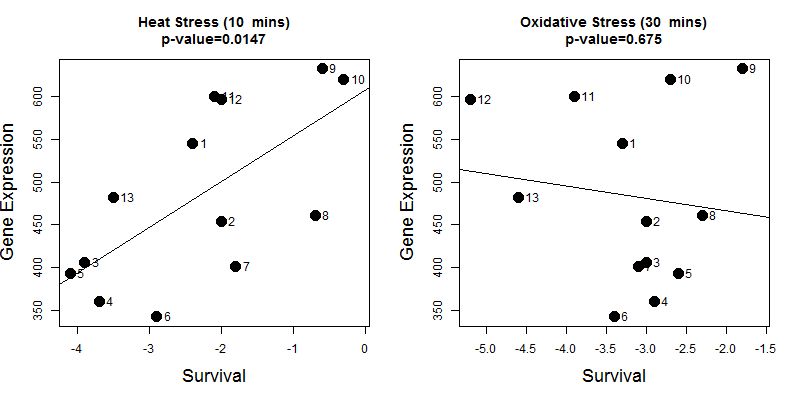

Supplement: S4 File — Expression levels of genes LLKF_1274 –LLKF_2533 and LLKF_p0001 –LLKF_p0036 plotted against survival after 10 minutes heat and 30 minutes oxidative stress. Survival is expressed as the difference of log CFU/ml after stress and before stress. Numbers indicate fermentations as presented in Table 1. P-values above the plots indicate significance of correlation (assessed by a linear model). (ZIP) [file pone.0167944.s009.zip › S4_File/LLKF_1375_real_dat.png]

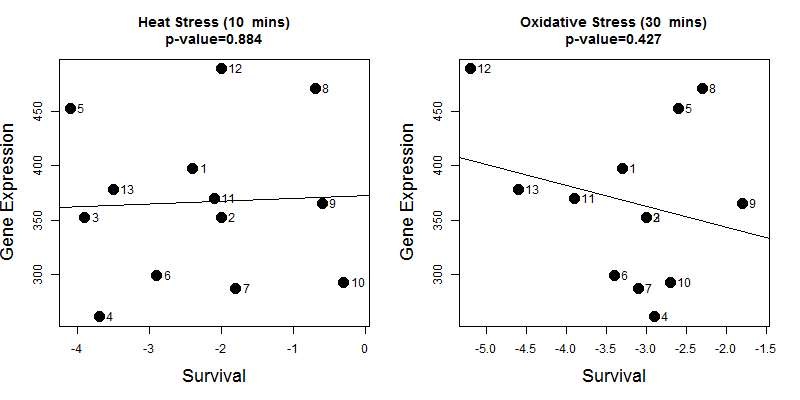

Supplement: S4 File — Expression levels of genes LLKF_1274 –LLKF_2533 and LLKF_p0001 –LLKF_p0036 plotted against survival after 10 minutes heat and 30 minutes oxidative stress. Survival is expressed as the difference of log CFU/ml after stress and before stress. Numbers indicate fermentations as presented in Table 1. P-values above the plots indicate significance of correlation (assessed by a linear model). (ZIP) [file pone.0167944.s009.zip › S4_File/LLKF_1376_real_dat.png]

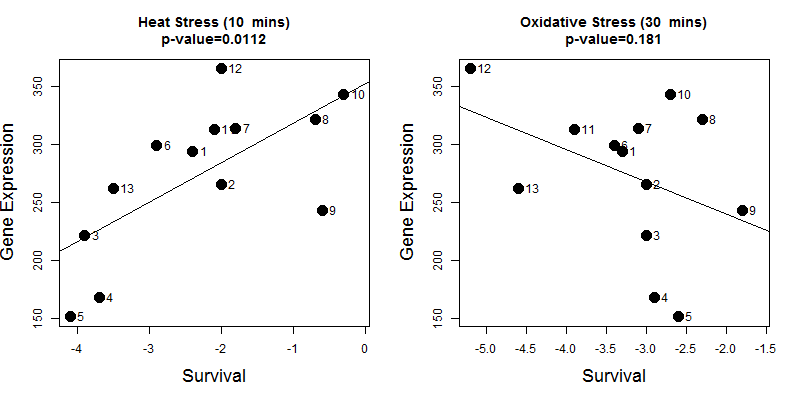

Supplement: S4 File — Expression levels of genes LLKF_1274 –LLKF_2533 and LLKF_p0001 –LLKF_p0036 plotted against survival after 10 minutes heat and 30 minutes oxidative stress. Survival is expressed as the difference of log CFU/ml after stress and before stress. Numbers indicate fermentations as presented in Table 1. P-values above the plots indicate significance of correlation (assessed by a linear model). (ZIP) [file pone.0167944.s009.zip › S4_File/LLKF_1377_real_dat.png]

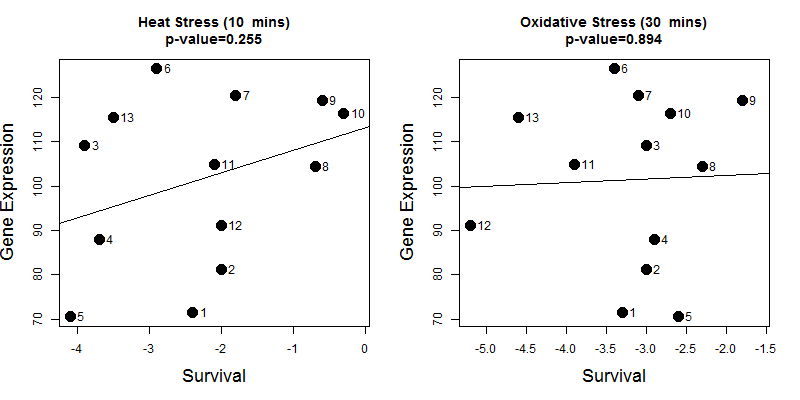

Supplement: S4 File — Expression levels of genes LLKF_1274 –LLKF_2533 and LLKF_p0001 –LLKF_p0036 plotted against survival after 10 minutes heat and 30 minutes oxidative stress. Survival is expressed as the difference of log CFU/ml after stress and before stress. Numbers indicate fermentations as presented in Table 1. P-values above the plots indicate significance of correlation (assessed by a linear model). (ZIP) [file pone.0167944.s009.zip › S4_File/LLKF_1378_real_dat.png]

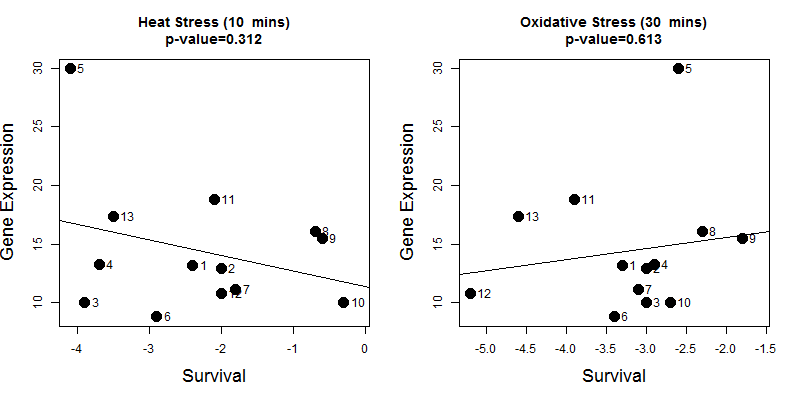

Supplement: S4 File — Expression levels of genes LLKF_1274 –LLKF_2533 and LLKF_p0001 –LLKF_p0036 plotted against survival after 10 minutes heat and 30 minutes oxidative stress. Survival is expressed as the difference of log CFU/ml after stress and before stress. Numbers indicate fermentations as presented in Table 1. P-values above the plots indicate significance of correlation (assessed by a linear model). (ZIP) [file pone.0167944.s009.zip › S4_File/LLKF_1379_real_dat.png]
