# Supplementary figures and images for: Strain-Dependent Transcriptome Signatures for Robustness in Lactococcus lactis (part 6 of 13)
Source: PLoS One. 2016 Dec 14;11(12):e0167944. doi: 10.1371/journal.pone.0167944 (PMC5156439; doi:10.1371/journal.pone.0167944)

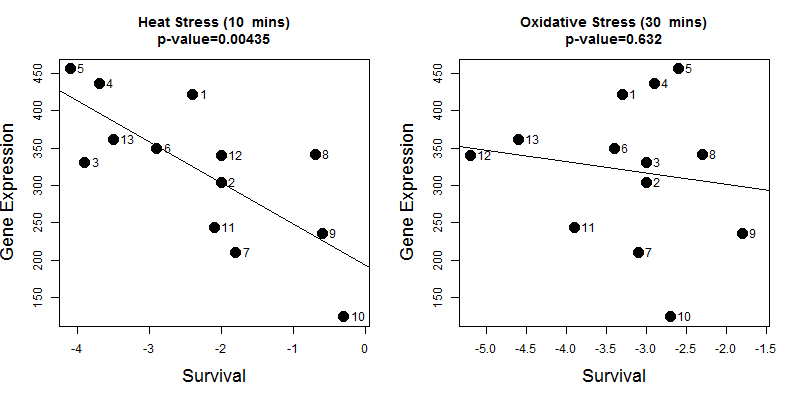

Supplement: S3 File — Expression levels of genes LLKF_0001 –LLKF_1273 plotted against survival after 10 minutes heat and 30 minutes oxidative stress. Survival is expressed as the difference of log CFU/ml after stress and before stress. Numbers indicate fermentations as presented in Table 1. P-values above the plots indicate significance of correlation (assessed by a linear model). (ZIP) [file pone.0167944.s008.zip › S3_File/LLKF_0100_real_dat.png]

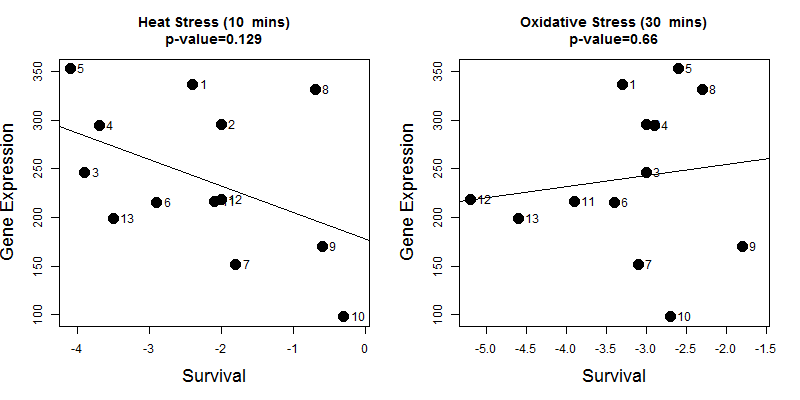

Supplement: S3 File — Expression levels of genes LLKF_0001 –LLKF_1273 plotted against survival after 10 minutes heat and 30 minutes oxidative stress. Survival is expressed as the difference of log CFU/ml after stress and before stress. Numbers indicate fermentations as presented in Table 1. P-values above the plots indicate significance of correlation (assessed by a linear model). (ZIP) [file pone.0167944.s008.zip › S3_File/LLKF_0101_real_dat.png]

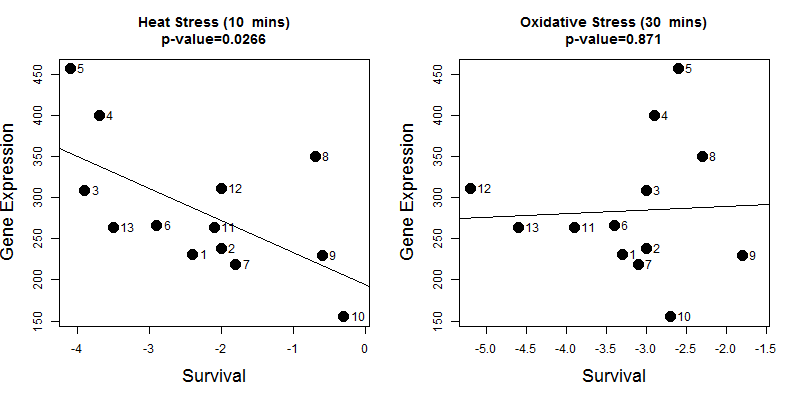

Supplement: S3 File — Expression levels of genes LLKF_0001 –LLKF_1273 plotted against survival after 10 minutes heat and 30 minutes oxidative stress. Survival is expressed as the difference of log CFU/ml after stress and before stress. Numbers indicate fermentations as presented in Table 1. P-values above the plots indicate significance of correlation (assessed by a linear model). (ZIP) [file pone.0167944.s008.zip › S3_File/LLKF_0102_real_dat.png]

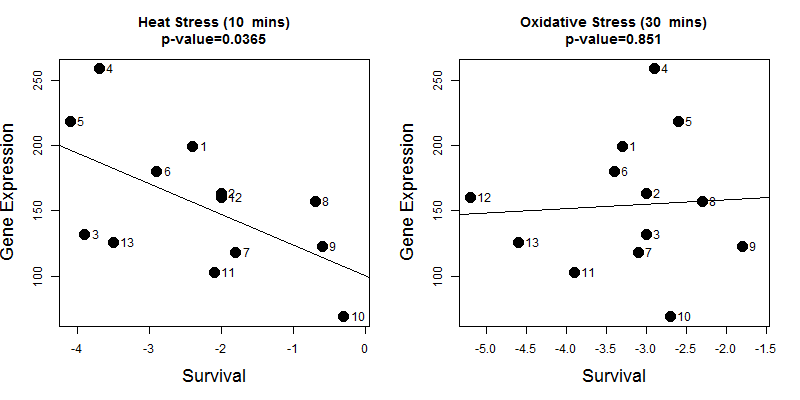

Supplement: S3 File — Expression levels of genes LLKF_0001 –LLKF_1273 plotted against survival after 10 minutes heat and 30 minutes oxidative stress. Survival is expressed as the difference of log CFU/ml after stress and before stress. Numbers indicate fermentations as presented in Table 1. P-values above the plots indicate significance of correlation (assessed by a linear model). (ZIP) [file pone.0167944.s008.zip › S3_File/LLKF_0103_real_dat.png]

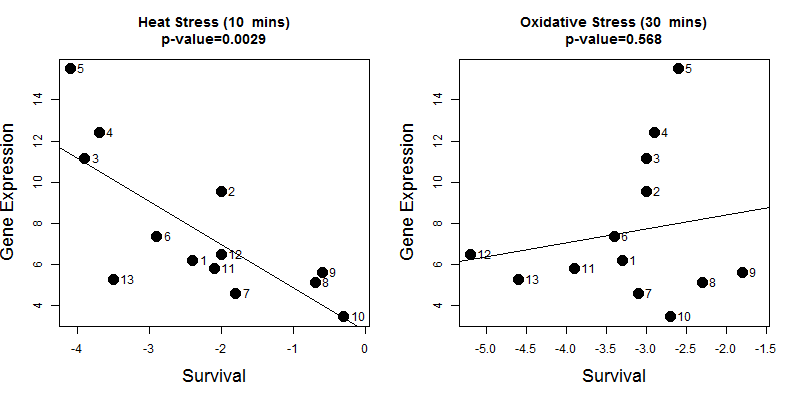

Supplement: S3 File — Expression levels of genes LLKF_0001 –LLKF_1273 plotted against survival after 10 minutes heat and 30 minutes oxidative stress. Survival is expressed as the difference of log CFU/ml after stress and before stress. Numbers indicate fermentations as presented in Table 1. P-values above the plots indicate significance of correlation (assessed by a linear model). (ZIP) [file pone.0167944.s008.zip › S3_File/LLKF_0104_real_dat.png]

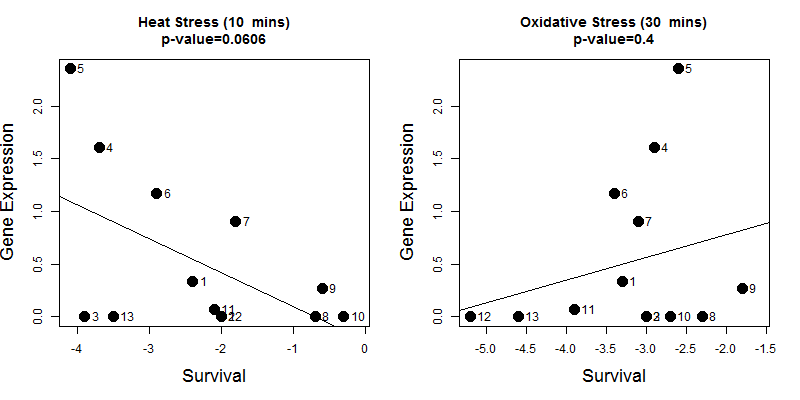

Supplement: S3 File — Expression levels of genes LLKF_0001 –LLKF_1273 plotted against survival after 10 minutes heat and 30 minutes oxidative stress. Survival is expressed as the difference of log CFU/ml after stress and before stress. Numbers indicate fermentations as presented in Table 1. P-values above the plots indicate significance of correlation (assessed by a linear model). (ZIP) [file pone.0167944.s008.zip › S3_File/LLKF_0107_real_dat.png]

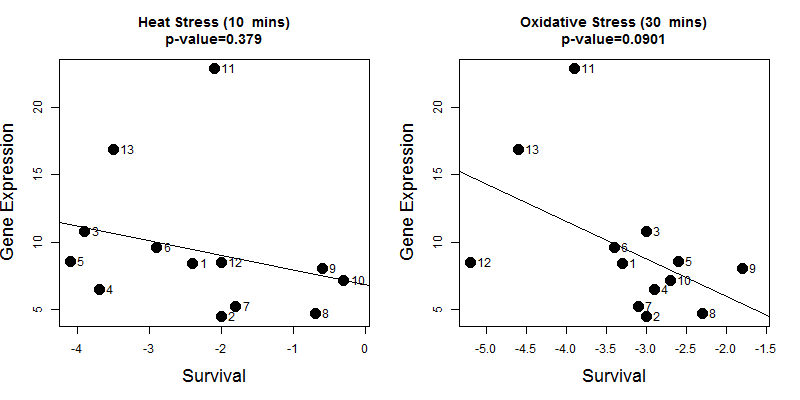

Supplement: S3 File — Expression levels of genes LLKF_0001 –LLKF_1273 plotted against survival after 10 minutes heat and 30 minutes oxidative stress. Survival is expressed as the difference of log CFU/ml after stress and before stress. Numbers indicate fermentations as presented in Table 1. P-values above the plots indicate significance of correlation (assessed by a linear model). (ZIP) [file pone.0167944.s008.zip › S3_File/LLKF_0108_real_dat.png]

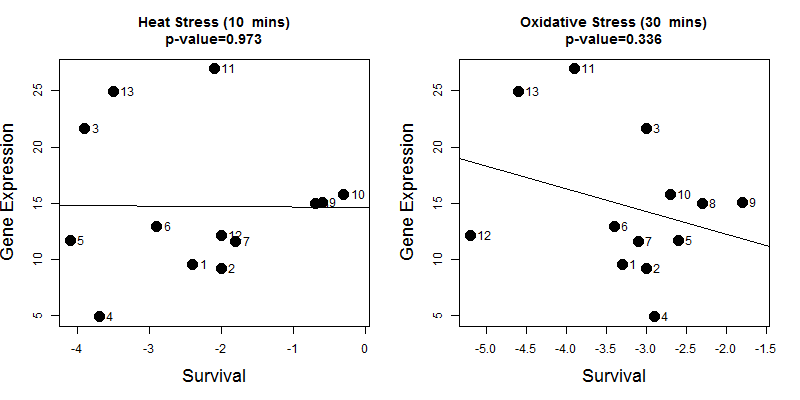

Supplement: S3 File — Expression levels of genes LLKF_0001 –LLKF_1273 plotted against survival after 10 minutes heat and 30 minutes oxidative stress. Survival is expressed as the difference of log CFU/ml after stress and before stress. Numbers indicate fermentations as presented in Table 1. P-values above the plots indicate significance of correlation (assessed by a linear model). (ZIP) [file pone.0167944.s008.zip › S3_File/LLKF_0109_real_dat.png]

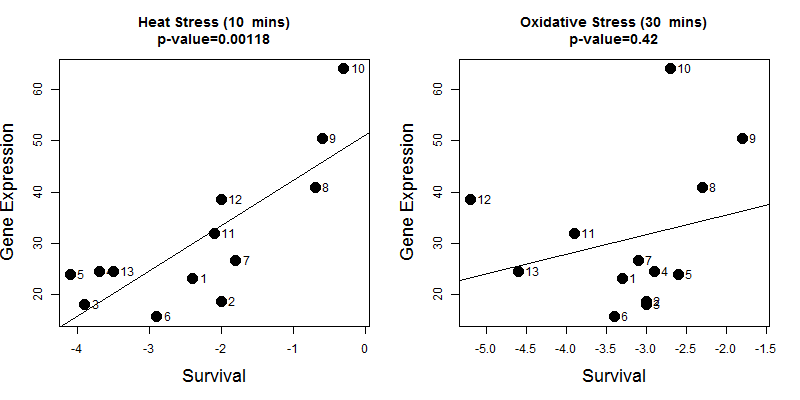

Supplement: S3 File — Expression levels of genes LLKF_0001 –LLKF_1273 plotted against survival after 10 minutes heat and 30 minutes oxidative stress. Survival is expressed as the difference of log CFU/ml after stress and before stress. Numbers indicate fermentations as presented in Table 1. P-values above the plots indicate significance of correlation (assessed by a linear model). (ZIP) [file pone.0167944.s008.zip › S3_File/LLKF_0110_real_dat.png]

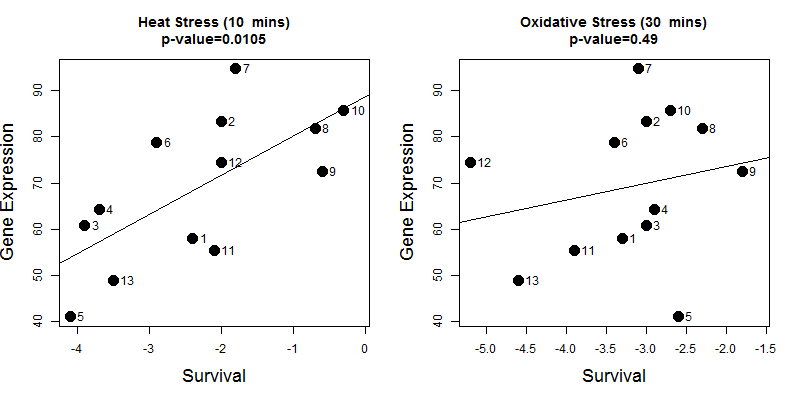

Supplement: S3 File — Expression levels of genes LLKF_0001 –LLKF_1273 plotted against survival after 10 minutes heat and 30 minutes oxidative stress. Survival is expressed as the difference of log CFU/ml after stress and before stress. Numbers indicate fermentations as presented in Table 1. P-values above the plots indicate significance of correlation (assessed by a linear model). (ZIP) [file pone.0167944.s008.zip › S3_File/LLKF_0111_real_dat.png]

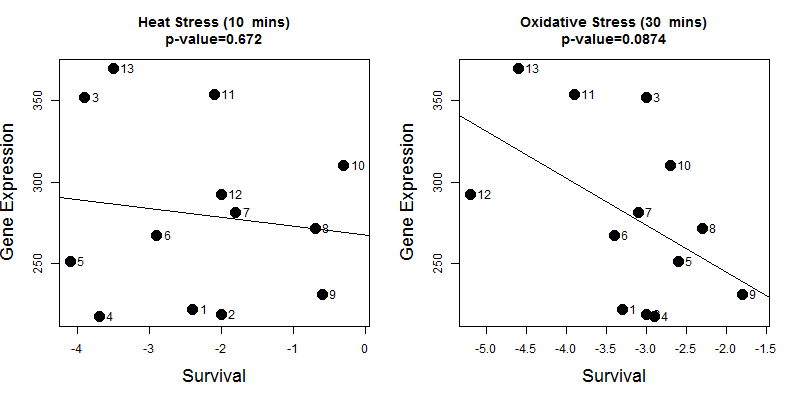

Supplement: S3 File — Expression levels of genes LLKF_0001 –LLKF_1273 plotted against survival after 10 minutes heat and 30 minutes oxidative stress. Survival is expressed as the difference of log CFU/ml after stress and before stress. Numbers indicate fermentations as presented in Table 1. P-values above the plots indicate significance of correlation (assessed by a linear model). (ZIP) [file pone.0167944.s008.zip › S3_File/LLKF_0112_real_dat.png]

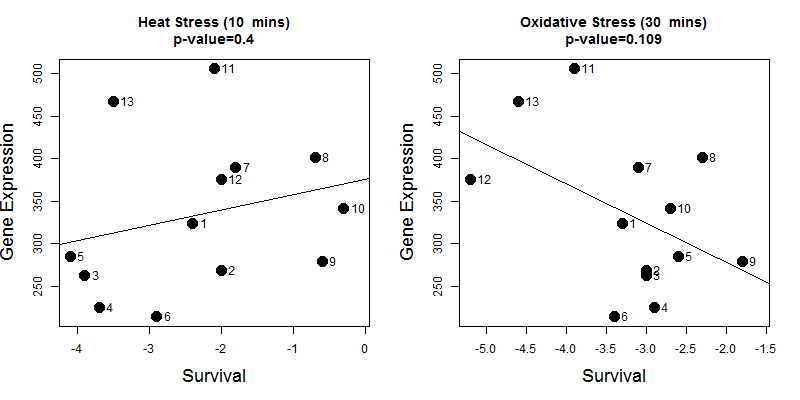

Supplement: S3 File — Expression levels of genes LLKF_0001 –LLKF_1273 plotted against survival after 10 minutes heat and 30 minutes oxidative stress. Survival is expressed as the difference of log CFU/ml after stress and before stress. Numbers indicate fermentations as presented in Table 1. P-values above the plots indicate significance of correlation (assessed by a linear model). (ZIP) [file pone.0167944.s008.zip › S3_File/LLKF_0113_real_dat.png]

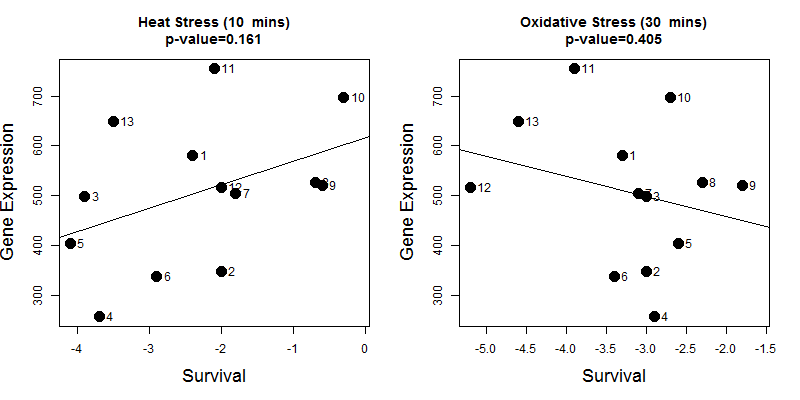

Supplement: S3 File — Expression levels of genes LLKF_0001 –LLKF_1273 plotted against survival after 10 minutes heat and 30 minutes oxidative stress. Survival is expressed as the difference of log CFU/ml after stress and before stress. Numbers indicate fermentations as presented in Table 1. P-values above the plots indicate significance of correlation (assessed by a linear model). (ZIP) [file pone.0167944.s008.zip › S3_File/LLKF_0114_real_dat.png]

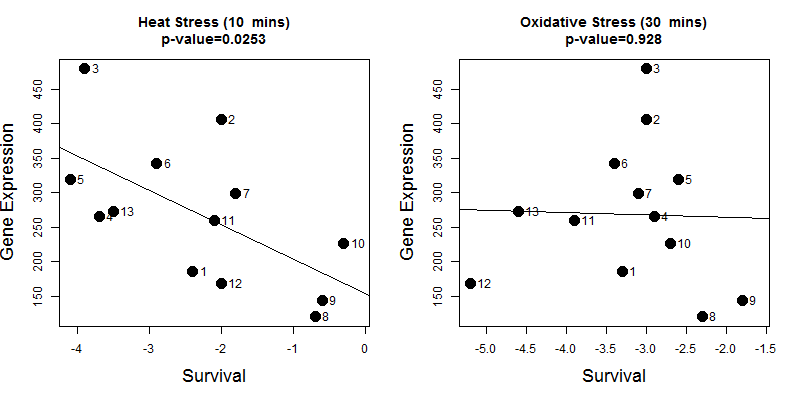

Supplement: S3 File — Expression levels of genes LLKF_0001 –LLKF_1273 plotted against survival after 10 minutes heat and 30 minutes oxidative stress. Survival is expressed as the difference of log CFU/ml after stress and before stress. Numbers indicate fermentations as presented in Table 1. P-values above the plots indicate significance of correlation (assessed by a linear model). (ZIP) [file pone.0167944.s008.zip › S3_File/LLKF_0115_real_dat.png]

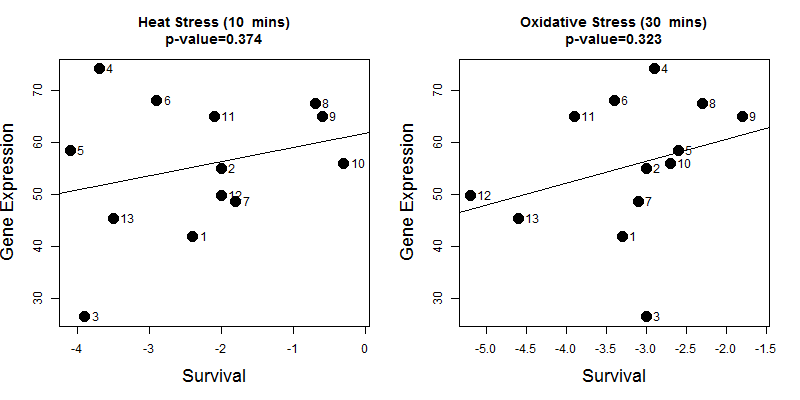

Supplement: S3 File — Expression levels of genes LLKF_0001 –LLKF_1273 plotted against survival after 10 minutes heat and 30 minutes oxidative stress. Survival is expressed as the difference of log CFU/ml after stress and before stress. Numbers indicate fermentations as presented in Table 1. P-values above the plots indicate significance of correlation (assessed by a linear model). (ZIP) [file pone.0167944.s008.zip › S3_File/LLKF_0116_real_dat.png]

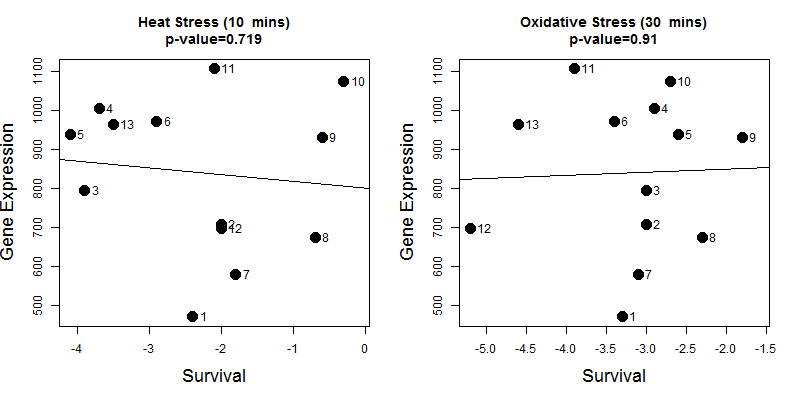

Supplement: S3 File — Expression levels of genes LLKF_0001 –LLKF_1273 plotted against survival after 10 minutes heat and 30 minutes oxidative stress. Survival is expressed as the difference of log CFU/ml after stress and before stress. Numbers indicate fermentations as presented in Table 1. P-values above the plots indicate significance of correlation (assessed by a linear model). (ZIP) [file pone.0167944.s008.zip › S3_File/LLKF_0117_real_dat.png]

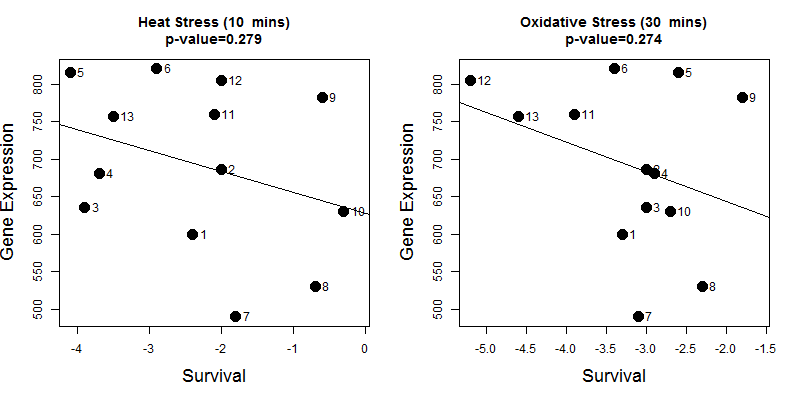

Supplement: S3 File — Expression levels of genes LLKF_0001 –LLKF_1273 plotted against survival after 10 minutes heat and 30 minutes oxidative stress. Survival is expressed as the difference of log CFU/ml after stress and before stress. Numbers indicate fermentations as presented in Table 1. P-values above the plots indicate significance of correlation (assessed by a linear model). (ZIP) [file pone.0167944.s008.zip › S3_File/LLKF_0118_real_dat.png]

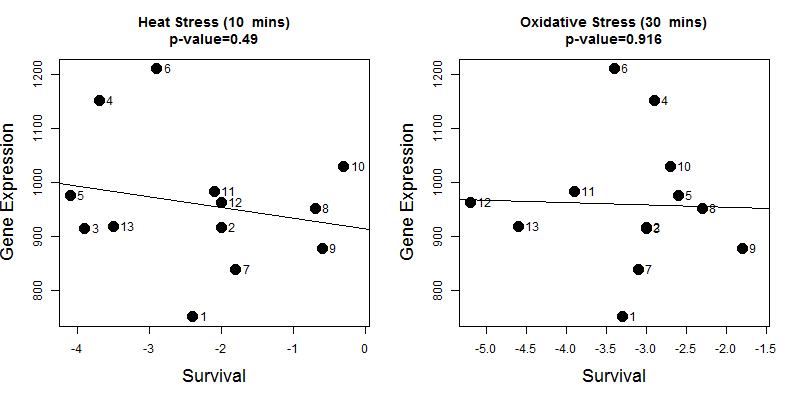

Supplement: S3 File — Expression levels of genes LLKF_0001 –LLKF_1273 plotted against survival after 10 minutes heat and 30 minutes oxidative stress. Survival is expressed as the difference of log CFU/ml after stress and before stress. Numbers indicate fermentations as presented in Table 1. P-values above the plots indicate significance of correlation (assessed by a linear model). (ZIP) [file pone.0167944.s008.zip › S3_File/LLKF_0119_real_dat.png]

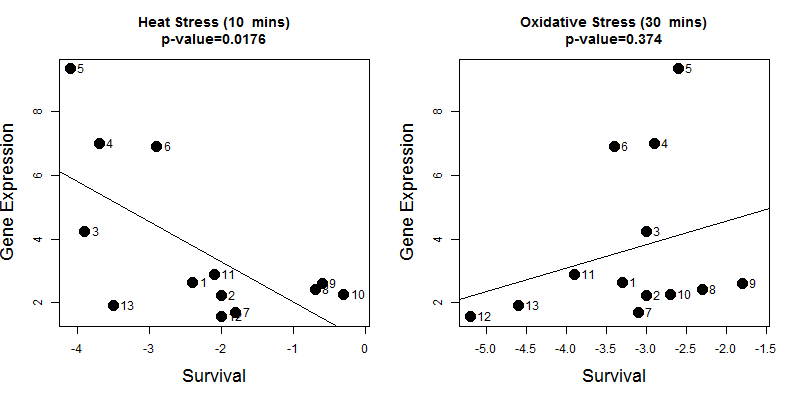

Supplement: S3 File — Expression levels of genes LLKF_0001 –LLKF_1273 plotted against survival after 10 minutes heat and 30 minutes oxidative stress. Survival is expressed as the difference of log CFU/ml after stress and before stress. Numbers indicate fermentations as presented in Table 1. P-values above the plots indicate significance of correlation (assessed by a linear model). (ZIP) [file pone.0167944.s008.zip › S3_File/LLKF_0120_real_dat.png]

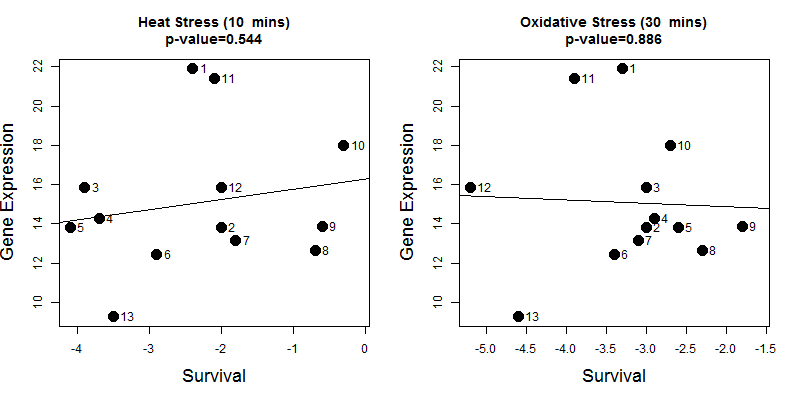

Supplement: S3 File — Expression levels of genes LLKF_0001 –LLKF_1273 plotted against survival after 10 minutes heat and 30 minutes oxidative stress. Survival is expressed as the difference of log CFU/ml after stress and before stress. Numbers indicate fermentations as presented in Table 1. P-values above the plots indicate significance of correlation (assessed by a linear model). (ZIP) [file pone.0167944.s008.zip › S3_File/LLKF_0121_real_dat.png]

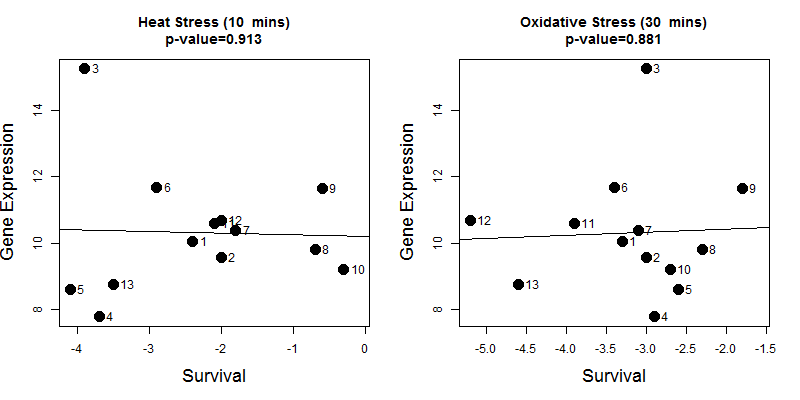

Supplement: S3 File — Expression levels of genes LLKF_0001 –LLKF_1273 plotted against survival after 10 minutes heat and 30 minutes oxidative stress. Survival is expressed as the difference of log CFU/ml after stress and before stress. Numbers indicate fermentations as presented in Table 1. P-values above the plots indicate significance of correlation (assessed by a linear model). (ZIP) [file pone.0167944.s008.zip › S3_File/LLKF_0122_real_dat.png]

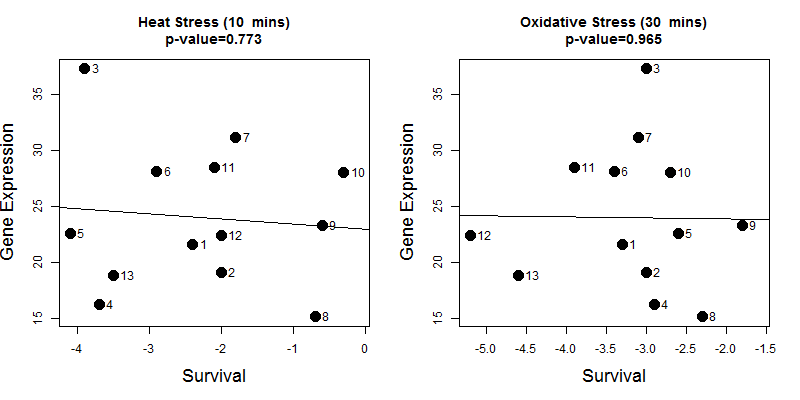

Supplement: S3 File — Expression levels of genes LLKF_0001 –LLKF_1273 plotted against survival after 10 minutes heat and 30 minutes oxidative stress. Survival is expressed as the difference of log CFU/ml after stress and before stress. Numbers indicate fermentations as presented in Table 1. P-values above the plots indicate significance of correlation (assessed by a linear model). (ZIP) [file pone.0167944.s008.zip › S3_File/LLKF_0123_real_dat.png]

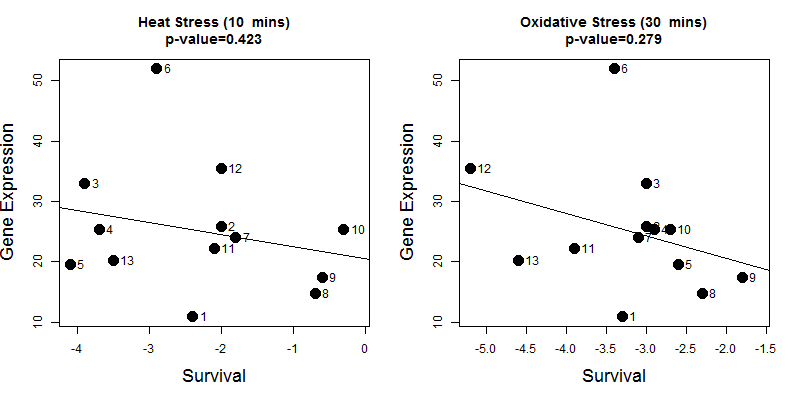

Supplement: S3 File — Expression levels of genes LLKF_0001 –LLKF_1273 plotted against survival after 10 minutes heat and 30 minutes oxidative stress. Survival is expressed as the difference of log CFU/ml after stress and before stress. Numbers indicate fermentations as presented in Table 1. P-values above the plots indicate significance of correlation (assessed by a linear model). (ZIP) [file pone.0167944.s008.zip › S3_File/LLKF_0124_real_dat.png]

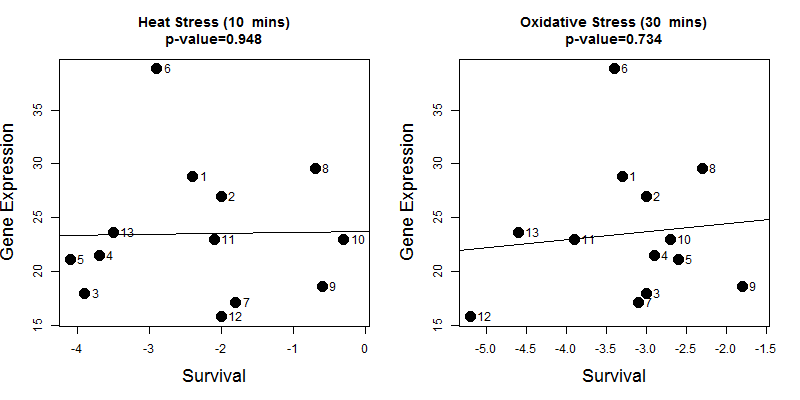

Supplement: S3 File — Expression levels of genes LLKF_0001 –LLKF_1273 plotted against survival after 10 minutes heat and 30 minutes oxidative stress. Survival is expressed as the difference of log CFU/ml after stress and before stress. Numbers indicate fermentations as presented in Table 1. P-values above the plots indicate significance of correlation (assessed by a linear model). (ZIP) [file pone.0167944.s008.zip › S3_File/LLKF_0125_real_dat.png]

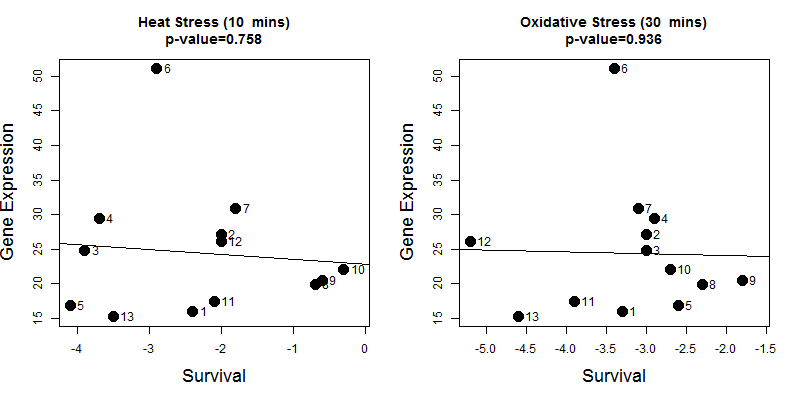

Supplement: S3 File — Expression levels of genes LLKF_0001 –LLKF_1273 plotted against survival after 10 minutes heat and 30 minutes oxidative stress. Survival is expressed as the difference of log CFU/ml after stress and before stress. Numbers indicate fermentations as presented in Table 1. P-values above the plots indicate significance of correlation (assessed by a linear model). (ZIP) [file pone.0167944.s008.zip › S3_File/LLKF_0126_real_dat.png]

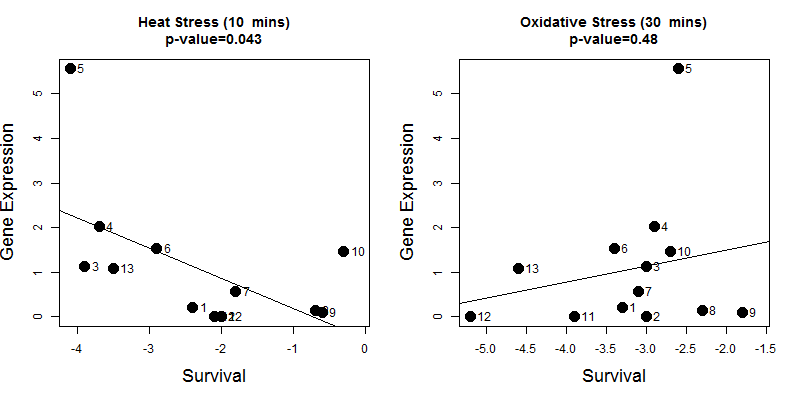

Supplement: S3 File — Expression levels of genes LLKF_0001 –LLKF_1273 plotted against survival after 10 minutes heat and 30 minutes oxidative stress. Survival is expressed as the difference of log CFU/ml after stress and before stress. Numbers indicate fermentations as presented in Table 1. P-values above the plots indicate significance of correlation (assessed by a linear model). (ZIP) [file pone.0167944.s008.zip › S3_File/LLKF_0129_real_dat.png]

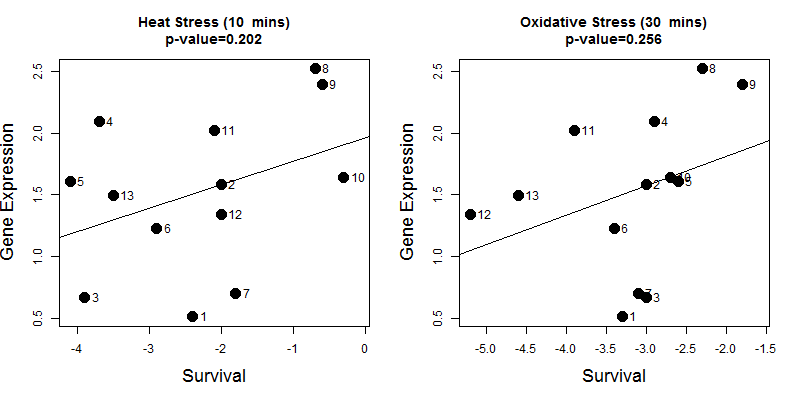

Supplement: S3 File — Expression levels of genes LLKF_0001 –LLKF_1273 plotted against survival after 10 minutes heat and 30 minutes oxidative stress. Survival is expressed as the difference of log CFU/ml after stress and before stress. Numbers indicate fermentations as presented in Table 1. P-values above the plots indicate significance of correlation (assessed by a linear model). (ZIP) [file pone.0167944.s008.zip › S3_File/LLKF_0130_real_dat.png]

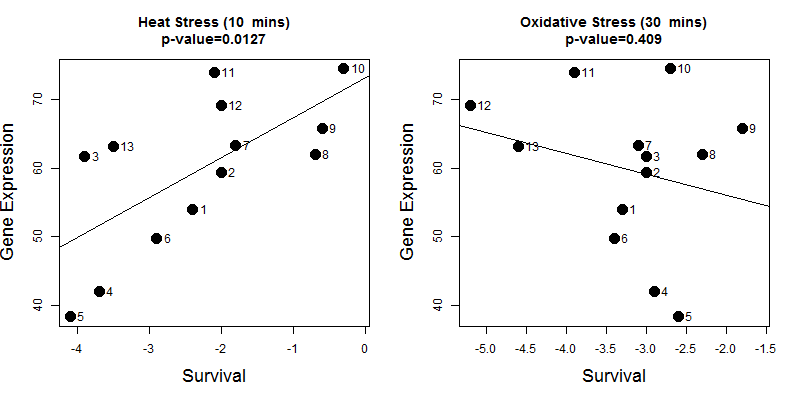

Supplement: S3 File — Expression levels of genes LLKF_0001 –LLKF_1273 plotted against survival after 10 minutes heat and 30 minutes oxidative stress. Survival is expressed as the difference of log CFU/ml after stress and before stress. Numbers indicate fermentations as presented in Table 1. P-values above the plots indicate significance of correlation (assessed by a linear model). (ZIP) [file pone.0167944.s008.zip › S3_File/LLKF_0131_real_dat.png]

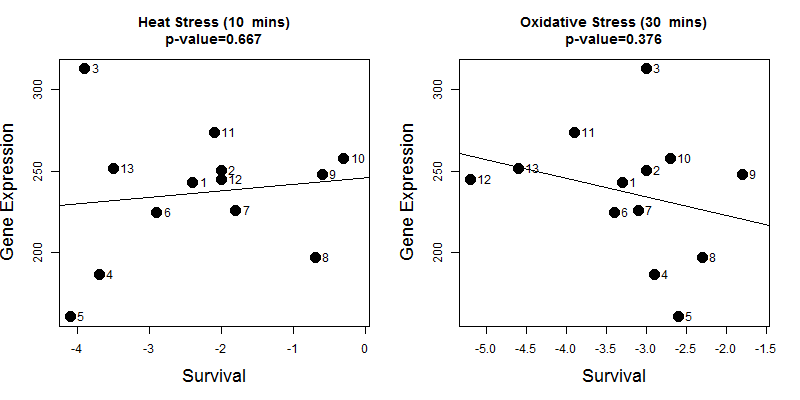

Supplement: S3 File — Expression levels of genes LLKF_0001 –LLKF_1273 plotted against survival after 10 minutes heat and 30 minutes oxidative stress. Survival is expressed as the difference of log CFU/ml after stress and before stress. Numbers indicate fermentations as presented in Table 1. P-values above the plots indicate significance of correlation (assessed by a linear model). (ZIP) [file pone.0167944.s008.zip › S3_File/LLKF_0132_real_dat.png]

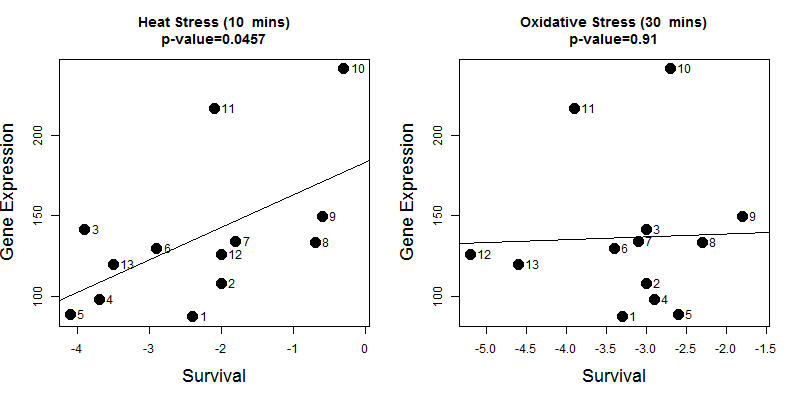

Supplement: S3 File — Expression levels of genes LLKF_0001 –LLKF_1273 plotted against survival after 10 minutes heat and 30 minutes oxidative stress. Survival is expressed as the difference of log CFU/ml after stress and before stress. Numbers indicate fermentations as presented in Table 1. P-values above the plots indicate significance of correlation (assessed by a linear model). (ZIP) [file pone.0167944.s008.zip › S3_File/LLKF_0133_real_dat.png]

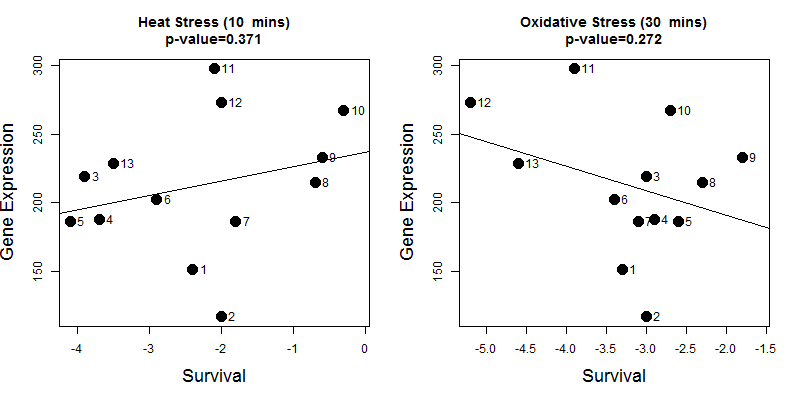

Supplement: S3 File — Expression levels of genes LLKF_0001 –LLKF_1273 plotted against survival after 10 minutes heat and 30 minutes oxidative stress. Survival is expressed as the difference of log CFU/ml after stress and before stress. Numbers indicate fermentations as presented in Table 1. P-values above the plots indicate significance of correlation (assessed by a linear model). (ZIP) [file pone.0167944.s008.zip › S3_File/LLKF_0134_real_dat.png]

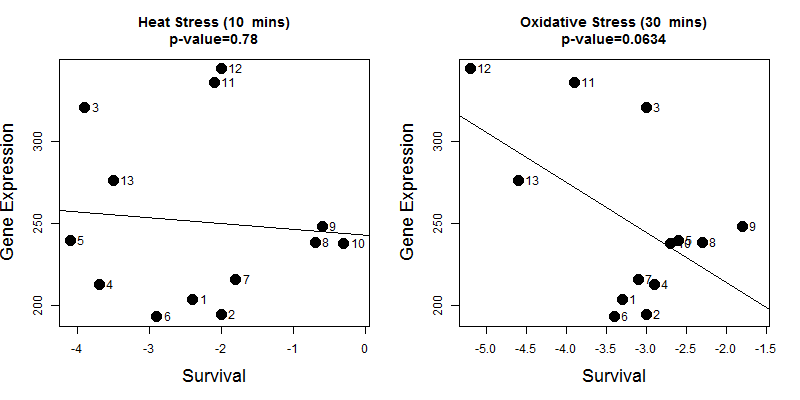

Supplement: S3 File — Expression levels of genes LLKF_0001 –LLKF_1273 plotted against survival after 10 minutes heat and 30 minutes oxidative stress. Survival is expressed as the difference of log CFU/ml after stress and before stress. Numbers indicate fermentations as presented in Table 1. P-values above the plots indicate significance of correlation (assessed by a linear model). (ZIP) [file pone.0167944.s008.zip › S3_File/LLKF_0135_real_dat.png]

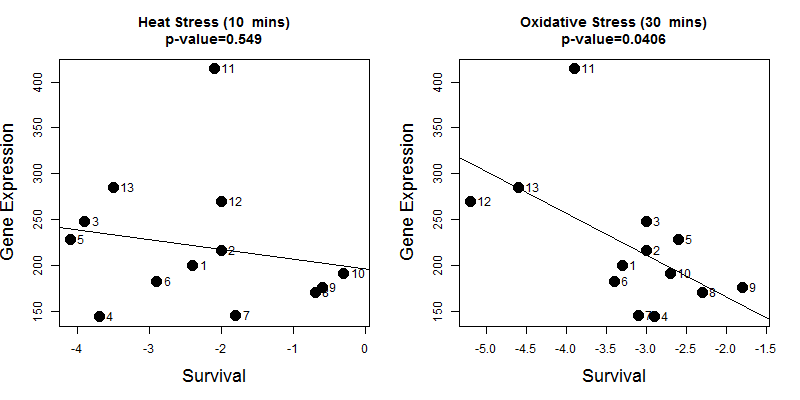

Supplement: S3 File — Expression levels of genes LLKF_0001 –LLKF_1273 plotted against survival after 10 minutes heat and 30 minutes oxidative stress. Survival is expressed as the difference of log CFU/ml after stress and before stress. Numbers indicate fermentations as presented in Table 1. P-values above the plots indicate significance of correlation (assessed by a linear model). (ZIP) [file pone.0167944.s008.zip › S3_File/LLKF_0136_real_dat.png]

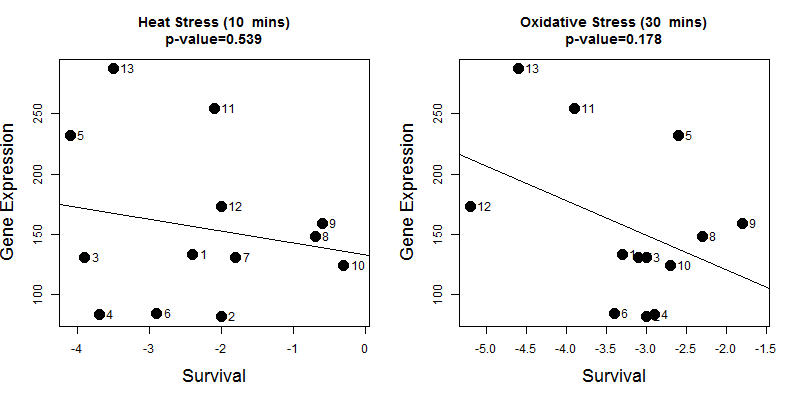

Supplement: S3 File — Expression levels of genes LLKF_0001 –LLKF_1273 plotted against survival after 10 minutes heat and 30 minutes oxidative stress. Survival is expressed as the difference of log CFU/ml after stress and before stress. Numbers indicate fermentations as presented in Table 1. P-values above the plots indicate significance of correlation (assessed by a linear model). (ZIP) [file pone.0167944.s008.zip › S3_File/LLKF_0137_real_dat.png]

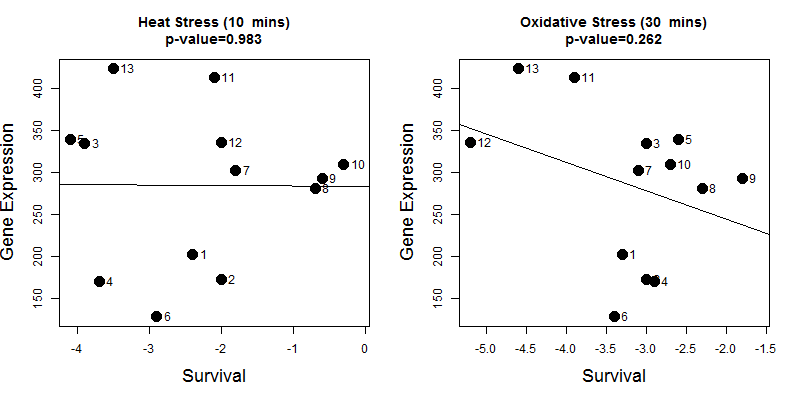

Supplement: S3 File — Expression levels of genes LLKF_0001 –LLKF_1273 plotted against survival after 10 minutes heat and 30 minutes oxidative stress. Survival is expressed as the difference of log CFU/ml after stress and before stress. Numbers indicate fermentations as presented in Table 1. P-values above the plots indicate significance of correlation (assessed by a linear model). (ZIP) [file pone.0167944.s008.zip › S3_File/LLKF_0138_real_dat.png]

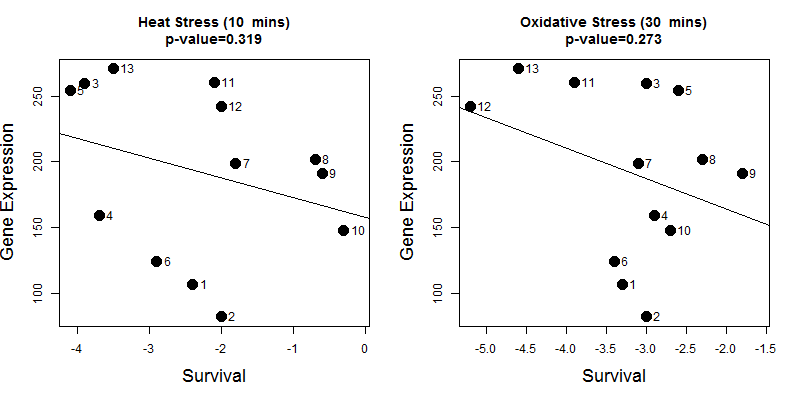

Supplement: S3 File — Expression levels of genes LLKF_0001 –LLKF_1273 plotted against survival after 10 minutes heat and 30 minutes oxidative stress. Survival is expressed as the difference of log CFU/ml after stress and before stress. Numbers indicate fermentations as presented in Table 1. P-values above the plots indicate significance of correlation (assessed by a linear model). (ZIP) [file pone.0167944.s008.zip › S3_File/LLKF_0139_real_dat.png]

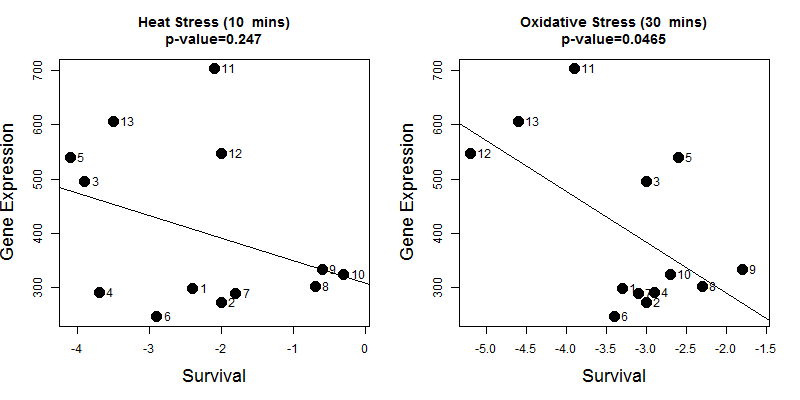

Supplement: S3 File — Expression levels of genes LLKF_0001 –LLKF_1273 plotted against survival after 10 minutes heat and 30 minutes oxidative stress. Survival is expressed as the difference of log CFU/ml after stress and before stress. Numbers indicate fermentations as presented in Table 1. P-values above the plots indicate significance of correlation (assessed by a linear model). (ZIP) [file pone.0167944.s008.zip › S3_File/LLKF_0140_real_dat.png]

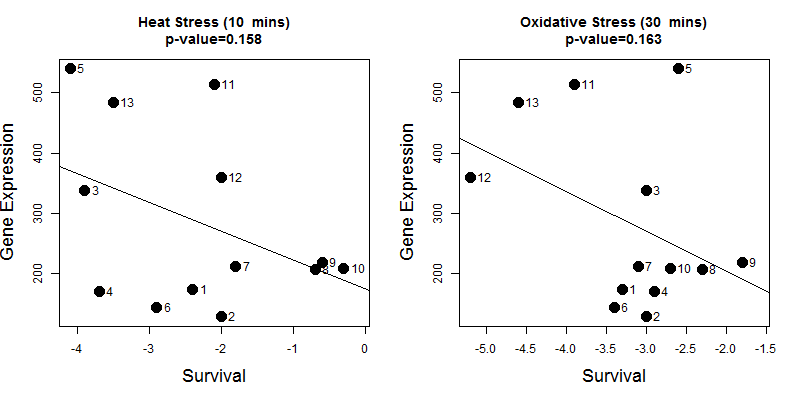

Supplement: S3 File — Expression levels of genes LLKF_0001 –LLKF_1273 plotted against survival after 10 minutes heat and 30 minutes oxidative stress. Survival is expressed as the difference of log CFU/ml after stress and before stress. Numbers indicate fermentations as presented in Table 1. P-values above the plots indicate significance of correlation (assessed by a linear model). (ZIP) [file pone.0167944.s008.zip › S3_File/LLKF_0141_real_dat.png]

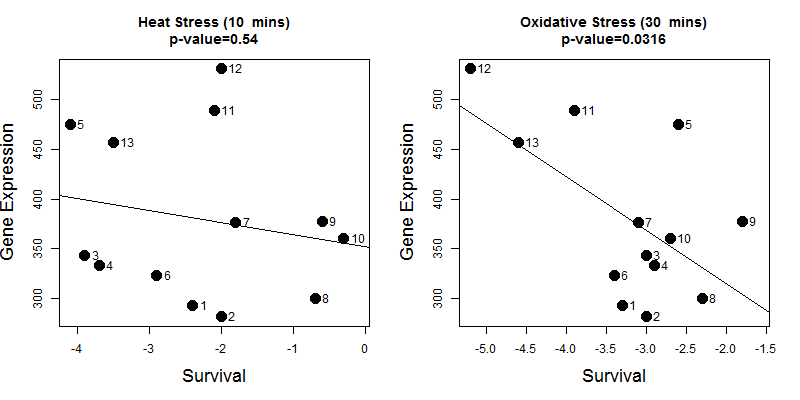

Supplement: S3 File — Expression levels of genes LLKF_0001 –LLKF_1273 plotted against survival after 10 minutes heat and 30 minutes oxidative stress. Survival is expressed as the difference of log CFU/ml after stress and before stress. Numbers indicate fermentations as presented in Table 1. P-values above the plots indicate significance of correlation (assessed by a linear model). (ZIP) [file pone.0167944.s008.zip › S3_File/LLKF_0142_real_dat.png]

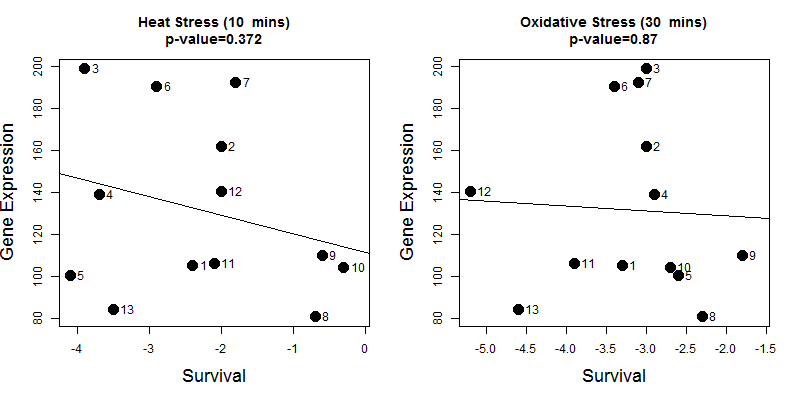

Supplement: S3 File — Expression levels of genes LLKF_0001 –LLKF_1273 plotted against survival after 10 minutes heat and 30 minutes oxidative stress. Survival is expressed as the difference of log CFU/ml after stress and before stress. Numbers indicate fermentations as presented in Table 1. P-values above the plots indicate significance of correlation (assessed by a linear model). (ZIP) [file pone.0167944.s008.zip › S3_File/LLKF_0143_real_dat.png]

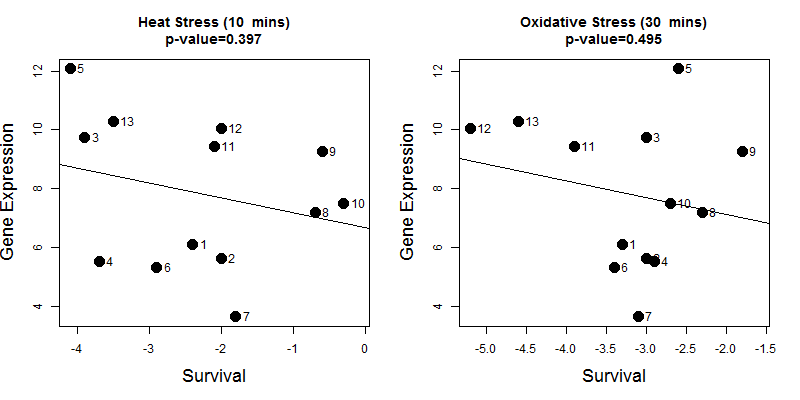

Supplement: S3 File — Expression levels of genes LLKF_0001 –LLKF_1273 plotted against survival after 10 minutes heat and 30 minutes oxidative stress. Survival is expressed as the difference of log CFU/ml after stress and before stress. Numbers indicate fermentations as presented in Table 1. P-values above the plots indicate significance of correlation (assessed by a linear model). (ZIP) [file pone.0167944.s008.zip › S3_File/LLKF_0144_real_dat.png]

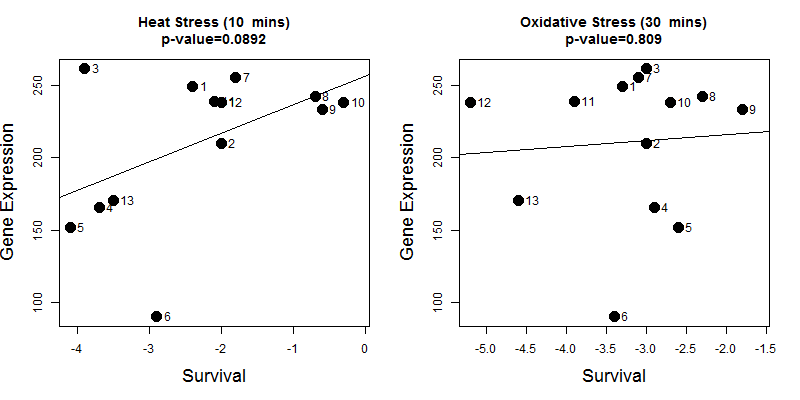

Supplement: S3 File — Expression levels of genes LLKF_0001 –LLKF_1273 plotted against survival after 10 minutes heat and 30 minutes oxidative stress. Survival is expressed as the difference of log CFU/ml after stress and before stress. Numbers indicate fermentations as presented in Table 1. P-values above the plots indicate significance of correlation (assessed by a linear model). (ZIP) [file pone.0167944.s008.zip › S3_File/LLKF_0145_real_dat.png]

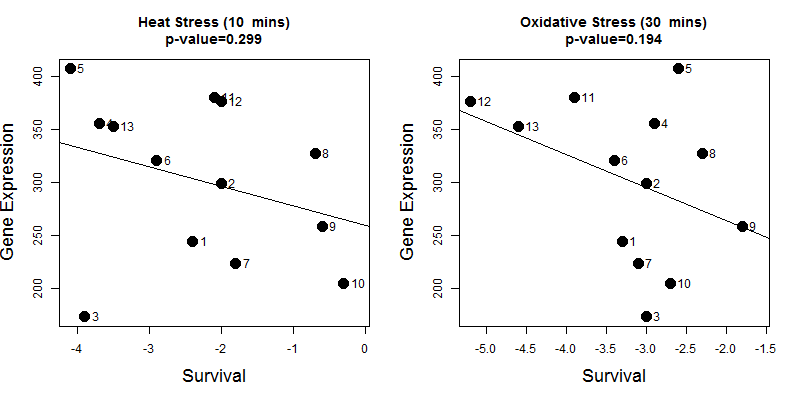

Supplement: S3 File — Expression levels of genes LLKF_0001 –LLKF_1273 plotted against survival after 10 minutes heat and 30 minutes oxidative stress. Survival is expressed as the difference of log CFU/ml after stress and before stress. Numbers indicate fermentations as presented in Table 1. P-values above the plots indicate significance of correlation (assessed by a linear model). (ZIP) [file pone.0167944.s008.zip › S3_File/LLKF_0146_real_dat.png]

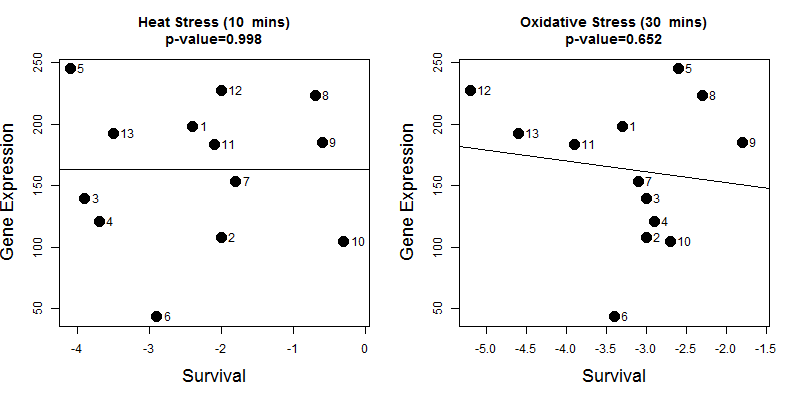

Supplement: S3 File — Expression levels of genes LLKF_0001 –LLKF_1273 plotted against survival after 10 minutes heat and 30 minutes oxidative stress. Survival is expressed as the difference of log CFU/ml after stress and before stress. Numbers indicate fermentations as presented in Table 1. P-values above the plots indicate significance of correlation (assessed by a linear model). (ZIP) [file pone.0167944.s008.zip › S3_File/LLKF_0147_real_dat.png]

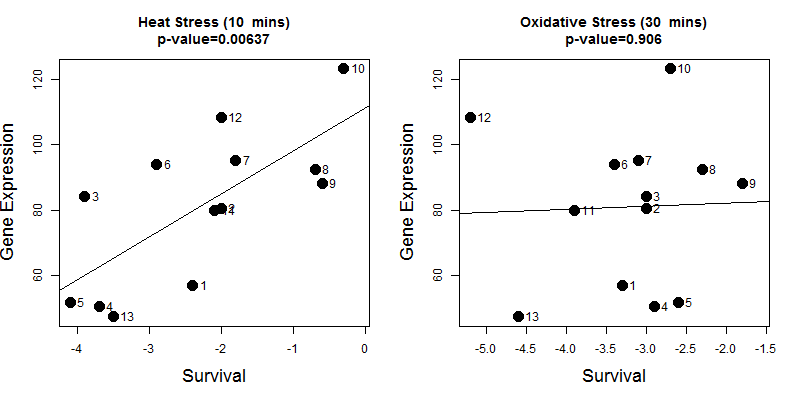

Supplement: S3 File — Expression levels of genes LLKF_0001 –LLKF_1273 plotted against survival after 10 minutes heat and 30 minutes oxidative stress. Survival is expressed as the difference of log CFU/ml after stress and before stress. Numbers indicate fermentations as presented in Table 1. P-values above the plots indicate significance of correlation (assessed by a linear model). (ZIP) [file pone.0167944.s008.zip › S3_File/LLKF_0148_real_dat.png]

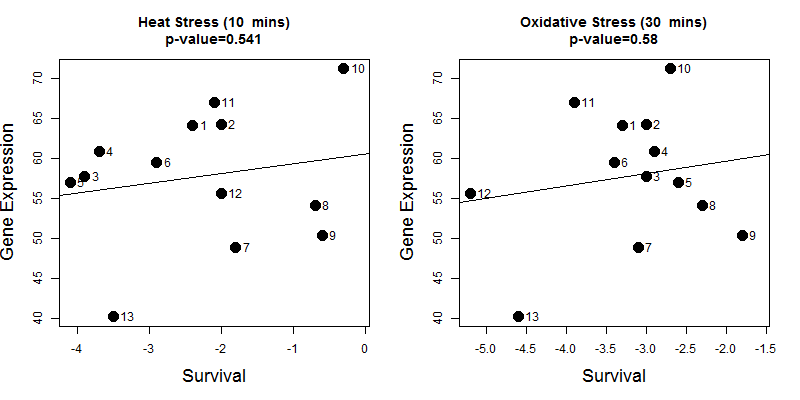

Supplement: S3 File — Expression levels of genes LLKF_0001 –LLKF_1273 plotted against survival after 10 minutes heat and 30 minutes oxidative stress. Survival is expressed as the difference of log CFU/ml after stress and before stress. Numbers indicate fermentations as presented in Table 1. P-values above the plots indicate significance of correlation (assessed by a linear model). (ZIP) [file pone.0167944.s008.zip › S3_File/LLKF_0149_real_dat.png]

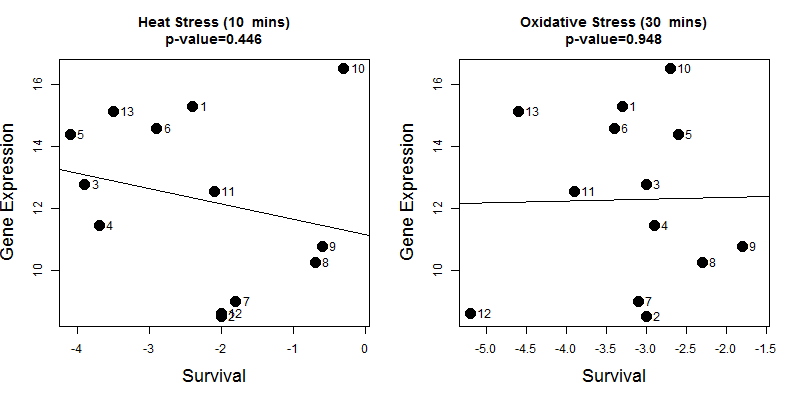

Supplement: S3 File — Expression levels of genes LLKF_0001 –LLKF_1273 plotted against survival after 10 minutes heat and 30 minutes oxidative stress. Survival is expressed as the difference of log CFU/ml after stress and before stress. Numbers indicate fermentations as presented in Table 1. P-values above the plots indicate significance of correlation (assessed by a linear model). (ZIP) [file pone.0167944.s008.zip › S3_File/LLKF_0150_real_dat.png]

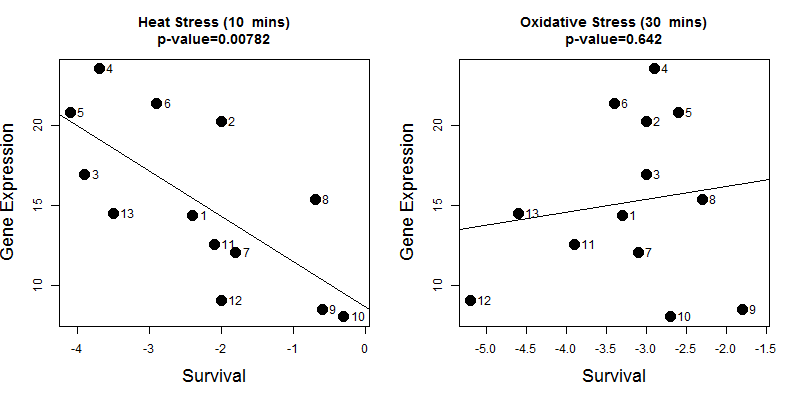

Supplement: S3 File — Expression levels of genes LLKF_0001 –LLKF_1273 plotted against survival after 10 minutes heat and 30 minutes oxidative stress. Survival is expressed as the difference of log CFU/ml after stress and before stress. Numbers indicate fermentations as presented in Table 1. P-values above the plots indicate significance of correlation (assessed by a linear model). (ZIP) [file pone.0167944.s008.zip › S3_File/LLKF_0151_real_dat.png]

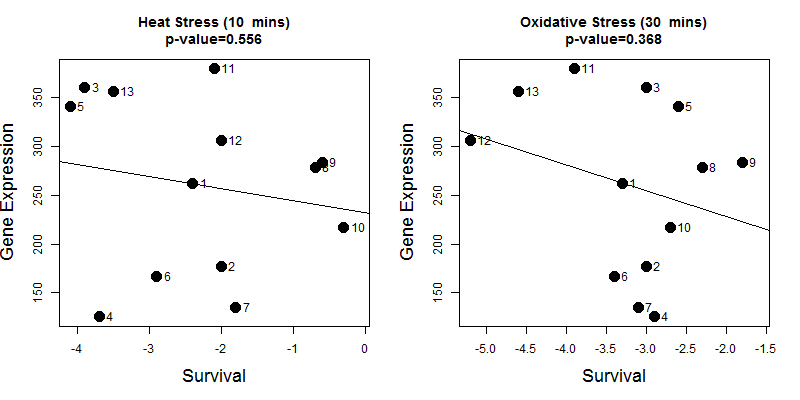

Supplement: S3 File — Expression levels of genes LLKF_0001 –LLKF_1273 plotted against survival after 10 minutes heat and 30 minutes oxidative stress. Survival is expressed as the difference of log CFU/ml after stress and before stress. Numbers indicate fermentations as presented in Table 1. P-values above the plots indicate significance of correlation (assessed by a linear model). (ZIP) [file pone.0167944.s008.zip › S3_File/LLKF_0152_real_dat.png]

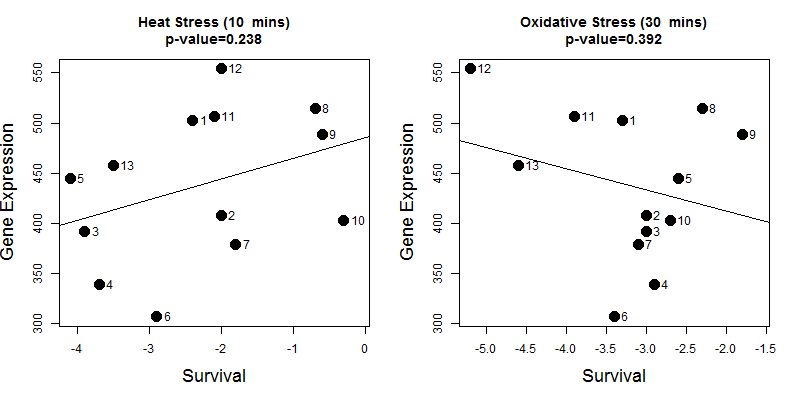

Supplement: S3 File — Expression levels of genes LLKF_0001 –LLKF_1273 plotted against survival after 10 minutes heat and 30 minutes oxidative stress. Survival is expressed as the difference of log CFU/ml after stress and before stress. Numbers indicate fermentations as presented in Table 1. P-values above the plots indicate significance of correlation (assessed by a linear model). (ZIP) [file pone.0167944.s008.zip › S3_File/LLKF_0153_real_dat.png]

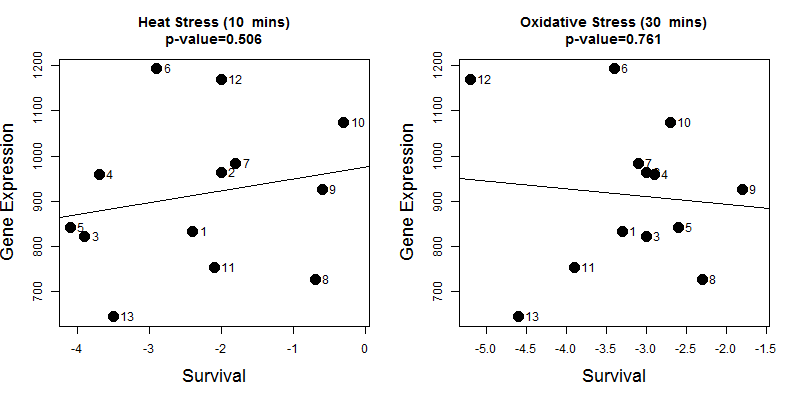

Supplement: S3 File — Expression levels of genes LLKF_0001 –LLKF_1273 plotted against survival after 10 minutes heat and 30 minutes oxidative stress. Survival is expressed as the difference of log CFU/ml after stress and before stress. Numbers indicate fermentations as presented in Table 1. P-values above the plots indicate significance of correlation (assessed by a linear model). (ZIP) [file pone.0167944.s008.zip › S3_File/LLKF_0154_real_dat.png]

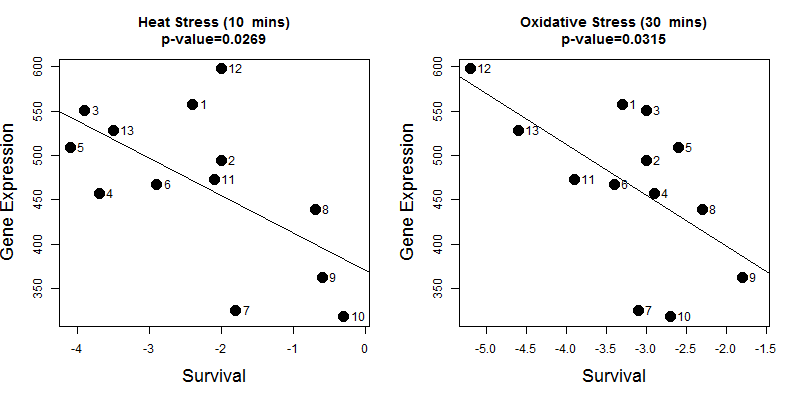

Supplement: S3 File — Expression levels of genes LLKF_0001 –LLKF_1273 plotted against survival after 10 minutes heat and 30 minutes oxidative stress. Survival is expressed as the difference of log CFU/ml after stress and before stress. Numbers indicate fermentations as presented in Table 1. P-values above the plots indicate significance of correlation (assessed by a linear model). (ZIP) [file pone.0167944.s008.zip › S3_File/LLKF_0155_real_dat.png]

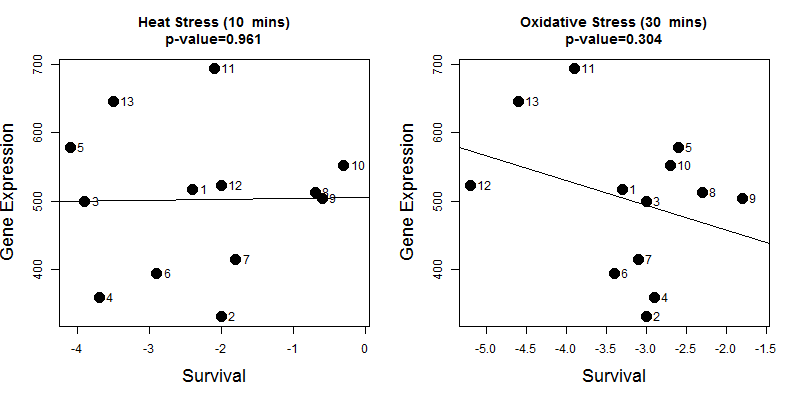

Supplement: S3 File — Expression levels of genes LLKF_0001 –LLKF_1273 plotted against survival after 10 minutes heat and 30 minutes oxidative stress. Survival is expressed as the difference of log CFU/ml after stress and before stress. Numbers indicate fermentations as presented in Table 1. P-values above the plots indicate significance of correlation (assessed by a linear model). (ZIP) [file pone.0167944.s008.zip › S3_File/LLKF_0156_real_dat.png]

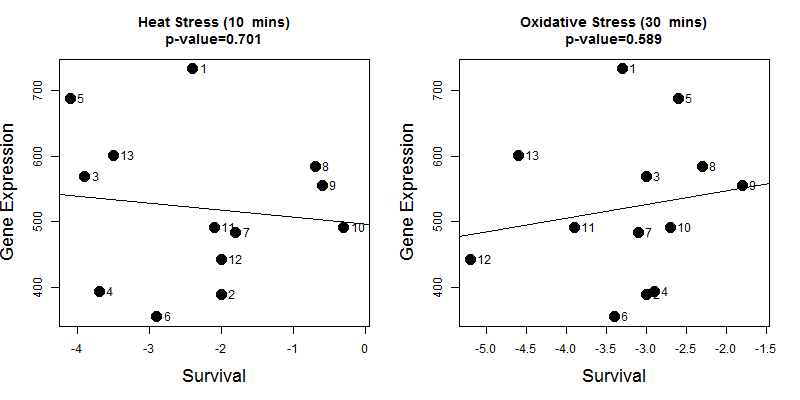

Supplement: S3 File — Expression levels of genes LLKF_0001 –LLKF_1273 plotted against survival after 10 minutes heat and 30 minutes oxidative stress. Survival is expressed as the difference of log CFU/ml after stress and before stress. Numbers indicate fermentations as presented in Table 1. P-values above the plots indicate significance of correlation (assessed by a linear model). (ZIP) [file pone.0167944.s008.zip › S3_File/LLKF_0157_real_dat.png]

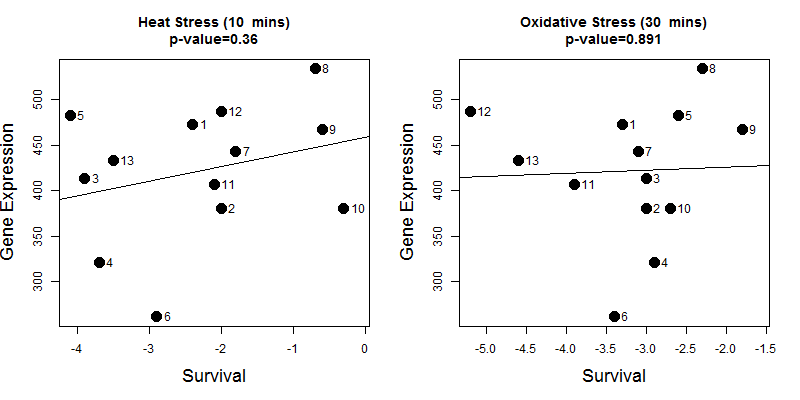

Supplement: S3 File — Expression levels of genes LLKF_0001 –LLKF_1273 plotted against survival after 10 minutes heat and 30 minutes oxidative stress. Survival is expressed as the difference of log CFU/ml after stress and before stress. Numbers indicate fermentations as presented in Table 1. P-values above the plots indicate significance of correlation (assessed by a linear model). (ZIP) [file pone.0167944.s008.zip › S3_File/LLKF_0158_real_dat.png]

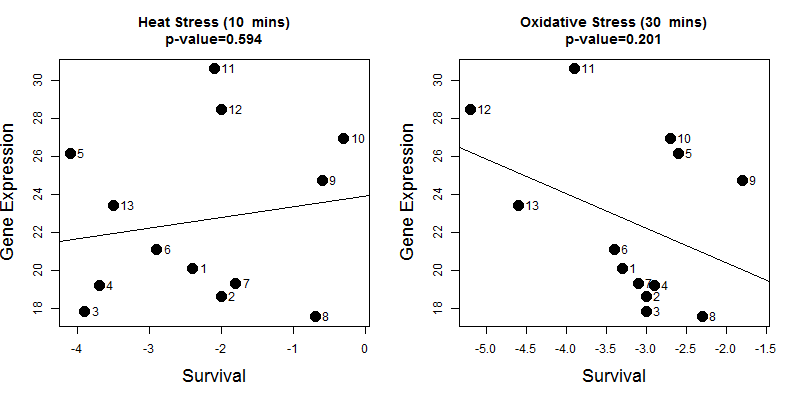

Supplement: S3 File — Expression levels of genes LLKF_0001 –LLKF_1273 plotted against survival after 10 minutes heat and 30 minutes oxidative stress. Survival is expressed as the difference of log CFU/ml after stress and before stress. Numbers indicate fermentations as presented in Table 1. P-values above the plots indicate significance of correlation (assessed by a linear model). (ZIP) [file pone.0167944.s008.zip › S3_File/LLKF_0159_real_dat.png]

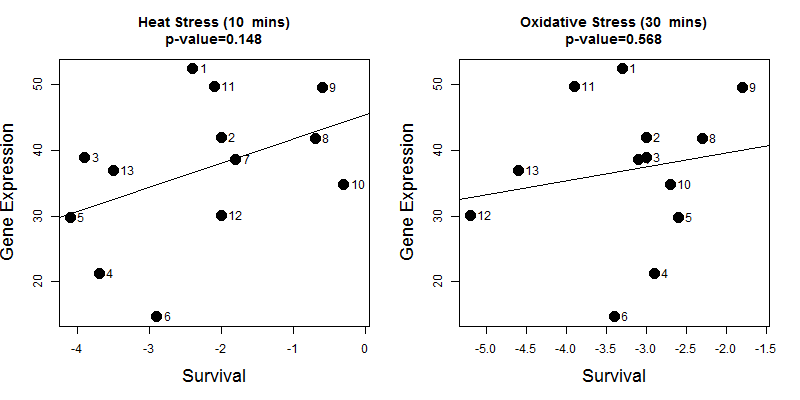

Supplement: S3 File — Expression levels of genes LLKF_0001 –LLKF_1273 plotted against survival after 10 minutes heat and 30 minutes oxidative stress. Survival is expressed as the difference of log CFU/ml after stress and before stress. Numbers indicate fermentations as presented in Table 1. P-values above the plots indicate significance of correlation (assessed by a linear model). (ZIP) [file pone.0167944.s008.zip › S3_File/LLKF_0160_real_dat.png]

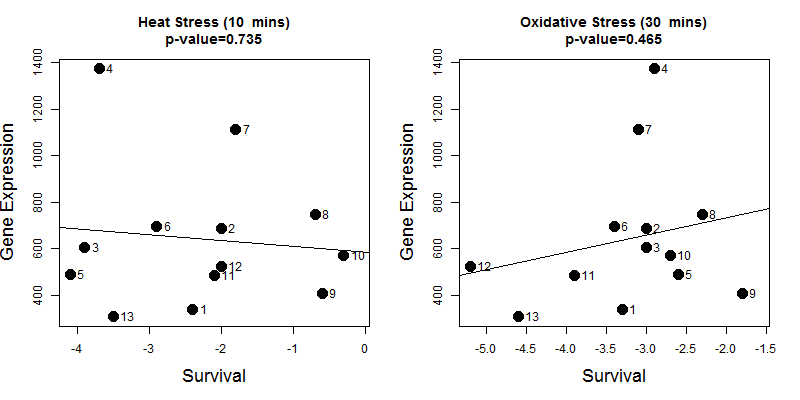

Supplement: S3 File — Expression levels of genes LLKF_0001 –LLKF_1273 plotted against survival after 10 minutes heat and 30 minutes oxidative stress. Survival is expressed as the difference of log CFU/ml after stress and before stress. Numbers indicate fermentations as presented in Table 1. P-values above the plots indicate significance of correlation (assessed by a linear model). (ZIP) [file pone.0167944.s008.zip › S3_File/LLKF_0161_real_dat.png]

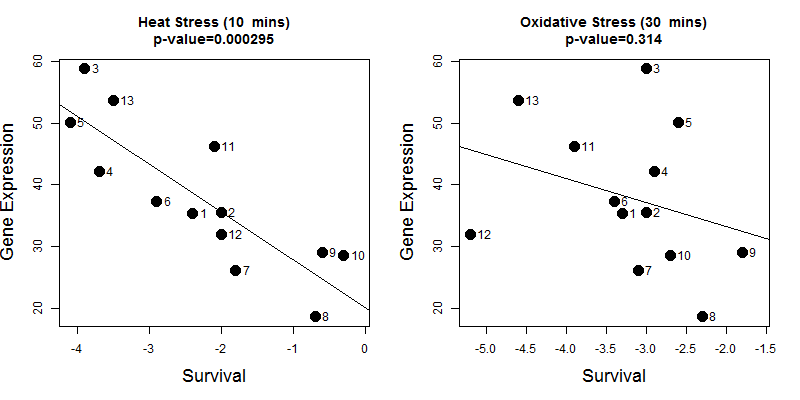

Supplement: S3 File — Expression levels of genes LLKF_0001 –LLKF_1273 plotted against survival after 10 minutes heat and 30 minutes oxidative stress. Survival is expressed as the difference of log CFU/ml after stress and before stress. Numbers indicate fermentations as presented in Table 1. P-values above the plots indicate significance of correlation (assessed by a linear model). (ZIP) [file pone.0167944.s008.zip › S3_File/LLKF_0162_real_dat.png]

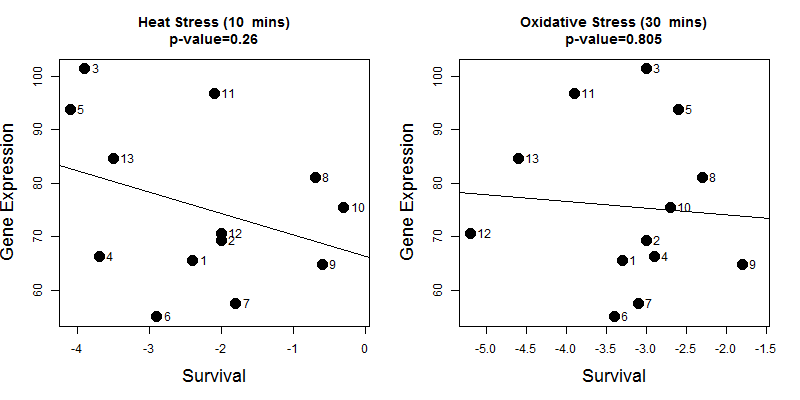

Supplement: S3 File — Expression levels of genes LLKF_0001 –LLKF_1273 plotted against survival after 10 minutes heat and 30 minutes oxidative stress. Survival is expressed as the difference of log CFU/ml after stress and before stress. Numbers indicate fermentations as presented in Table 1. P-values above the plots indicate significance of correlation (assessed by a linear model). (ZIP) [file pone.0167944.s008.zip › S3_File/LLKF_0163_real_dat.png]

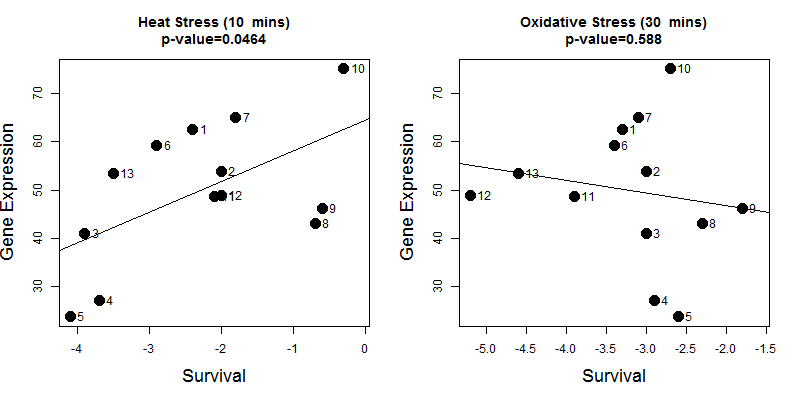

Supplement: S3 File — Expression levels of genes LLKF_0001 –LLKF_1273 plotted against survival after 10 minutes heat and 30 minutes oxidative stress. Survival is expressed as the difference of log CFU/ml after stress and before stress. Numbers indicate fermentations as presented in Table 1. P-values above the plots indicate significance of correlation (assessed by a linear model). (ZIP) [file pone.0167944.s008.zip › S3_File/LLKF_0164_real_dat.png]

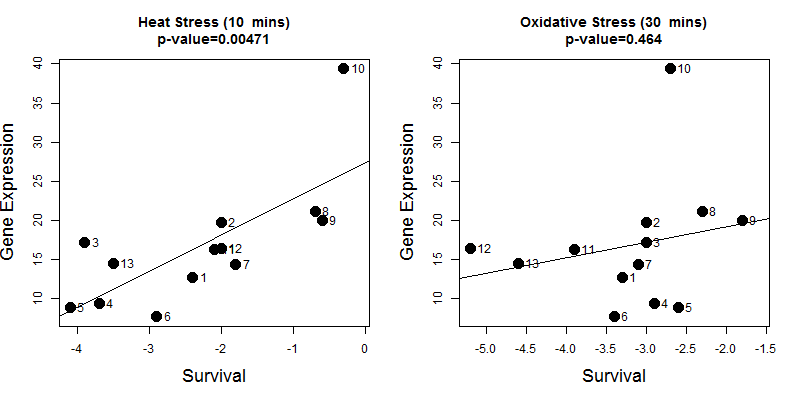

Supplement: S3 File — Expression levels of genes LLKF_0001 –LLKF_1273 plotted against survival after 10 minutes heat and 30 minutes oxidative stress. Survival is expressed as the difference of log CFU/ml after stress and before stress. Numbers indicate fermentations as presented in Table 1. P-values above the plots indicate significance of correlation (assessed by a linear model). (ZIP) [file pone.0167944.s008.zip › S3_File/LLKF_0165_real_dat.png]

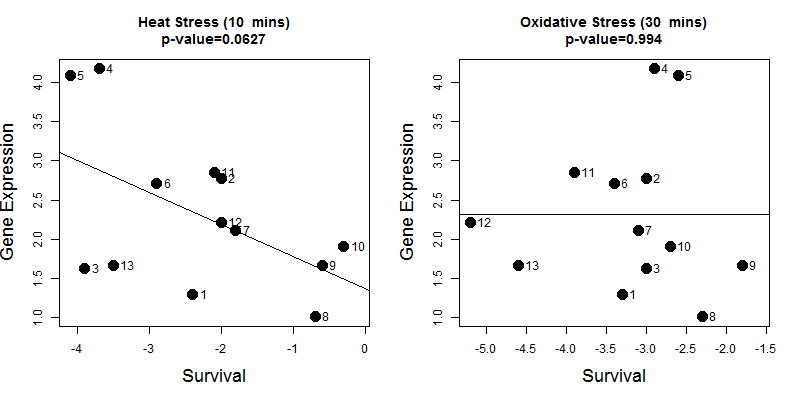

Supplement: S3 File — Expression levels of genes LLKF_0001 –LLKF_1273 plotted against survival after 10 minutes heat and 30 minutes oxidative stress. Survival is expressed as the difference of log CFU/ml after stress and before stress. Numbers indicate fermentations as presented in Table 1. P-values above the plots indicate significance of correlation (assessed by a linear model). (ZIP) [file pone.0167944.s008.zip › S3_File/LLKF_0166_real_dat.png]

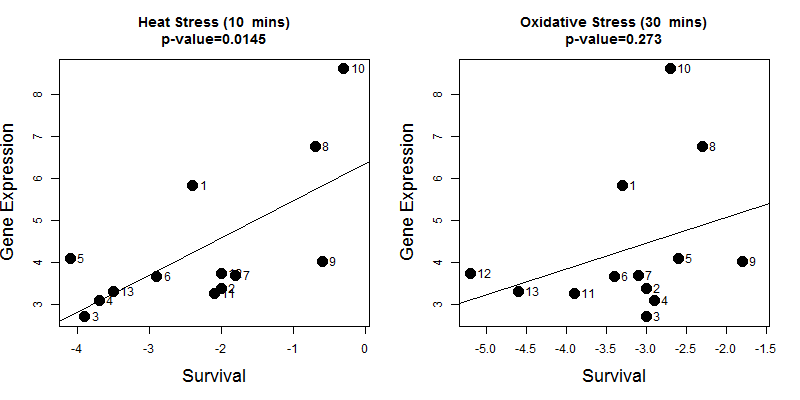

Supplement: S3 File — Expression levels of genes LLKF_0001 –LLKF_1273 plotted against survival after 10 minutes heat and 30 minutes oxidative stress. Survival is expressed as the difference of log CFU/ml after stress and before stress. Numbers indicate fermentations as presented in Table 1. P-values above the plots indicate significance of correlation (assessed by a linear model). (ZIP) [file pone.0167944.s008.zip › S3_File/LLKF_0167_real_dat.png]

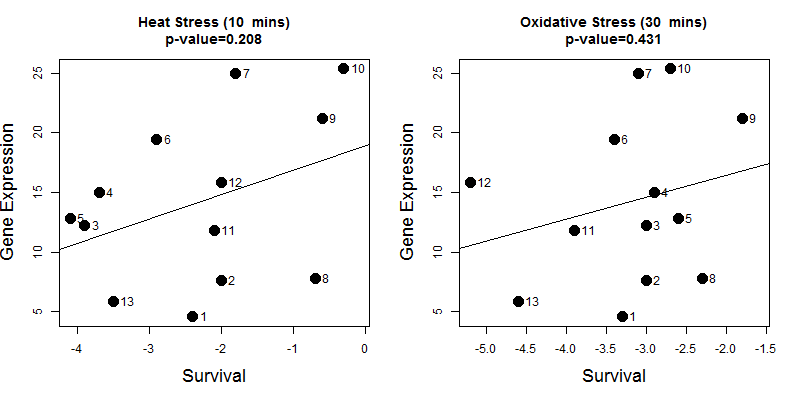

Supplement: S3 File — Expression levels of genes LLKF_0001 –LLKF_1273 plotted against survival after 10 minutes heat and 30 minutes oxidative stress. Survival is expressed as the difference of log CFU/ml after stress and before stress. Numbers indicate fermentations as presented in Table 1. P-values above the plots indicate significance of correlation (assessed by a linear model). (ZIP) [file pone.0167944.s008.zip › S3_File/LLKF_0168_real_dat.png]

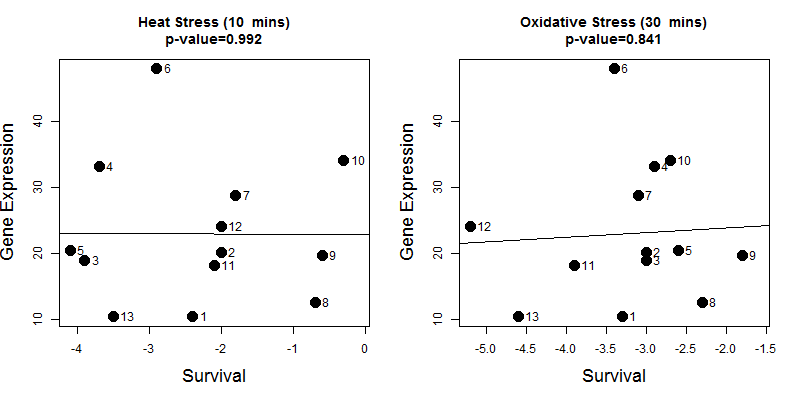

Supplement: S3 File — Expression levels of genes LLKF_0001 –LLKF_1273 plotted against survival after 10 minutes heat and 30 minutes oxidative stress. Survival is expressed as the difference of log CFU/ml after stress and before stress. Numbers indicate fermentations as presented in Table 1. P-values above the plots indicate significance of correlation (assessed by a linear model). (ZIP) [file pone.0167944.s008.zip › S3_File/LLKF_0169_real_dat.png]

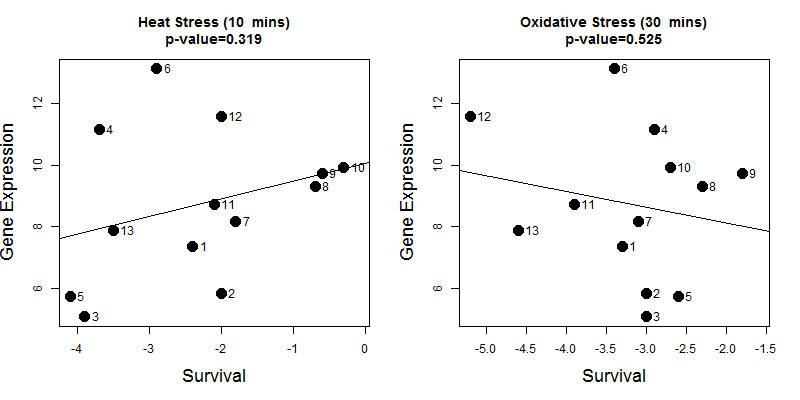

Supplement: S3 File — Expression levels of genes LLKF_0001 –LLKF_1273 plotted against survival after 10 minutes heat and 30 minutes oxidative stress. Survival is expressed as the difference of log CFU/ml after stress and before stress. Numbers indicate fermentations as presented in Table 1. P-values above the plots indicate significance of correlation (assessed by a linear model). (ZIP) [file pone.0167944.s008.zip › S3_File/LLKF_0170_real_dat.png]

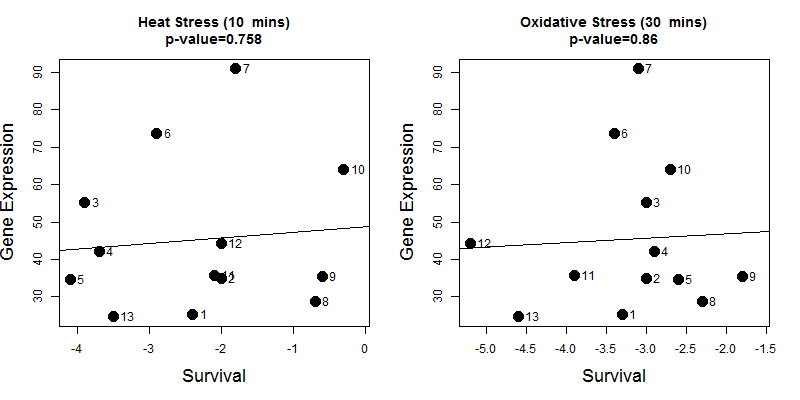

Supplement: S3 File — Expression levels of genes LLKF_0001 –LLKF_1273 plotted against survival after 10 minutes heat and 30 minutes oxidative stress. Survival is expressed as the difference of log CFU/ml after stress and before stress. Numbers indicate fermentations as presented in Table 1. P-values above the plots indicate significance of correlation (assessed by a linear model). (ZIP) [file pone.0167944.s008.zip › S3_File/LLKF_0171_real_dat.png]

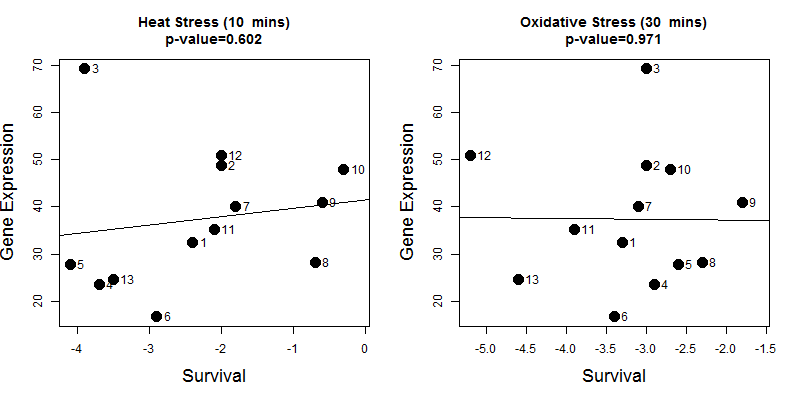

Supplement: S3 File — Expression levels of genes LLKF_0001 –LLKF_1273 plotted against survival after 10 minutes heat and 30 minutes oxidative stress. Survival is expressed as the difference of log CFU/ml after stress and before stress. Numbers indicate fermentations as presented in Table 1. P-values above the plots indicate significance of correlation (assessed by a linear model). (ZIP) [file pone.0167944.s008.zip › S3_File/LLKF_0172_real_dat.png]

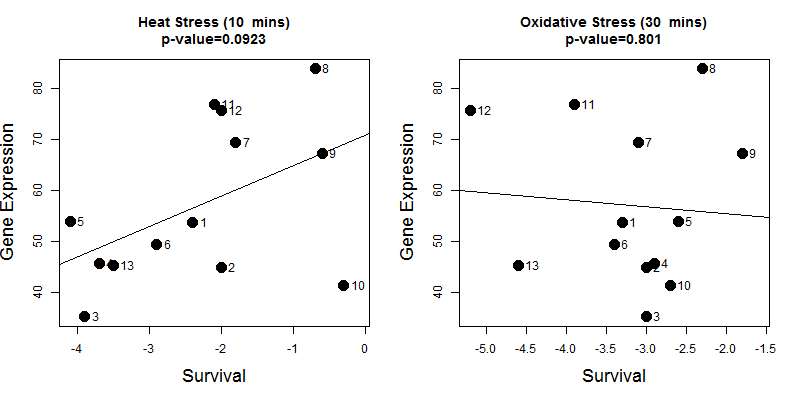

Supplement: S3 File — Expression levels of genes LLKF_0001 –LLKF_1273 plotted against survival after 10 minutes heat and 30 minutes oxidative stress. Survival is expressed as the difference of log CFU/ml after stress and before stress. Numbers indicate fermentations as presented in Table 1. P-values above the plots indicate significance of correlation (assessed by a linear model). (ZIP) [file pone.0167944.s008.zip › S3_File/LLKF_0173_real_dat.png]

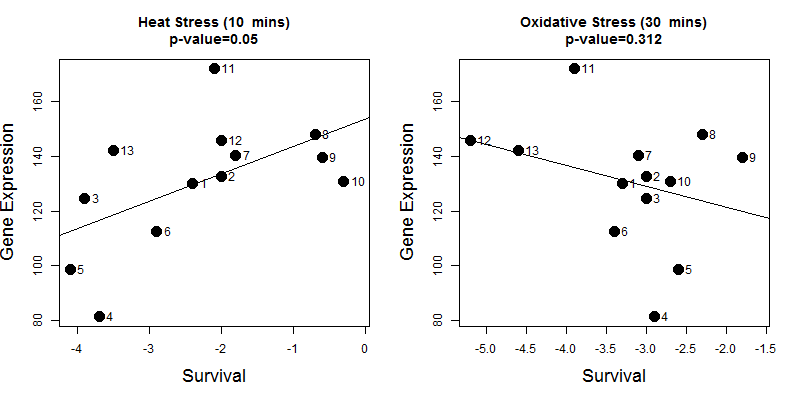

Supplement: S3 File — Expression levels of genes LLKF_0001 –LLKF_1273 plotted against survival after 10 minutes heat and 30 minutes oxidative stress. Survival is expressed as the difference of log CFU/ml after stress and before stress. Numbers indicate fermentations as presented in Table 1. P-values above the plots indicate significance of correlation (assessed by a linear model). (ZIP) [file pone.0167944.s008.zip › S3_File/LLKF_0174_real_dat.png]

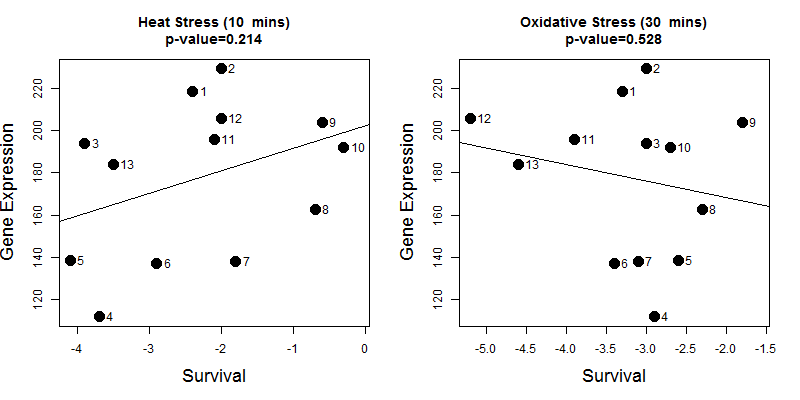

Supplement: S3 File — Expression levels of genes LLKF_0001 –LLKF_1273 plotted against survival after 10 minutes heat and 30 minutes oxidative stress. Survival is expressed as the difference of log CFU/ml after stress and before stress. Numbers indicate fermentations as presented in Table 1. P-values above the plots indicate significance of correlation (assessed by a linear model). (ZIP) [file pone.0167944.s008.zip › S3_File/LLKF_0175_real_dat.png]

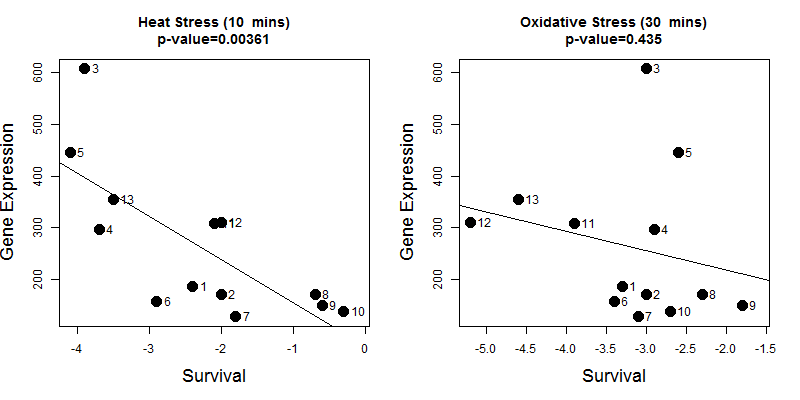

Supplement: S3 File — Expression levels of genes LLKF_0001 –LLKF_1273 plotted against survival after 10 minutes heat and 30 minutes oxidative stress. Survival is expressed as the difference of log CFU/ml after stress and before stress. Numbers indicate fermentations as presented in Table 1. P-values above the plots indicate significance of correlation (assessed by a linear model). (ZIP) [file pone.0167944.s008.zip › S3_File/LLKF_0176_real_dat.png]

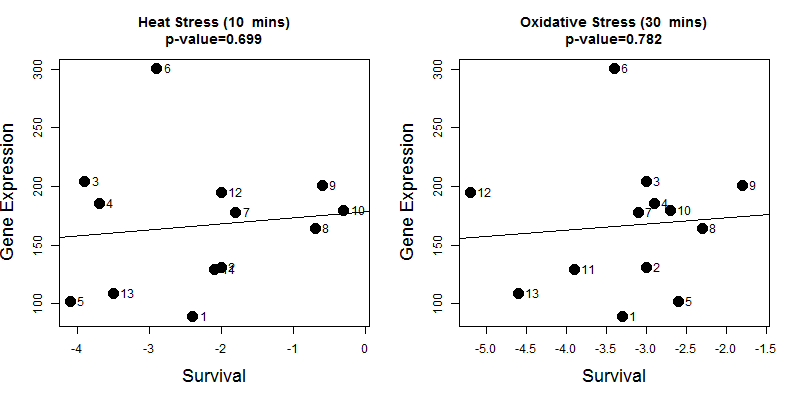

Supplement: S3 File — Expression levels of genes LLKF_0001 –LLKF_1273 plotted against survival after 10 minutes heat and 30 minutes oxidative stress. Survival is expressed as the difference of log CFU/ml after stress and before stress. Numbers indicate fermentations as presented in Table 1. P-values above the plots indicate significance of correlation (assessed by a linear model). (ZIP) [file pone.0167944.s008.zip › S3_File/LLKF_0177_real_dat.png]

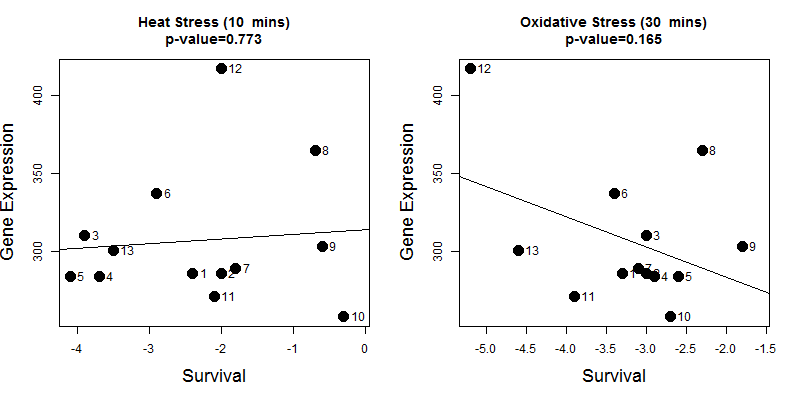

Supplement: S3 File — Expression levels of genes LLKF_0001 –LLKF_1273 plotted against survival after 10 minutes heat and 30 minutes oxidative stress. Survival is expressed as the difference of log CFU/ml after stress and before stress. Numbers indicate fermentations as presented in Table 1. P-values above the plots indicate significance of correlation (assessed by a linear model). (ZIP) [file pone.0167944.s008.zip › S3_File/LLKF_0178_real_dat.png]

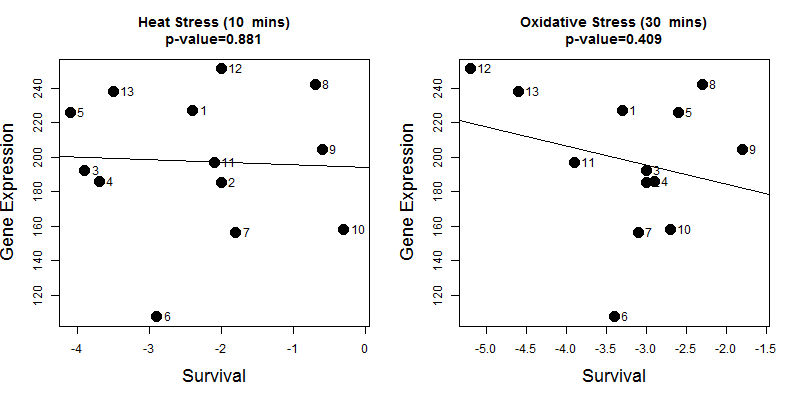

Supplement: S3 File — Expression levels of genes LLKF_0001 –LLKF_1273 plotted against survival after 10 minutes heat and 30 minutes oxidative stress. Survival is expressed as the difference of log CFU/ml after stress and before stress. Numbers indicate fermentations as presented in Table 1. P-values above the plots indicate significance of correlation (assessed by a linear model). (ZIP) [file pone.0167944.s008.zip › S3_File/LLKF_0179_real_dat.png]

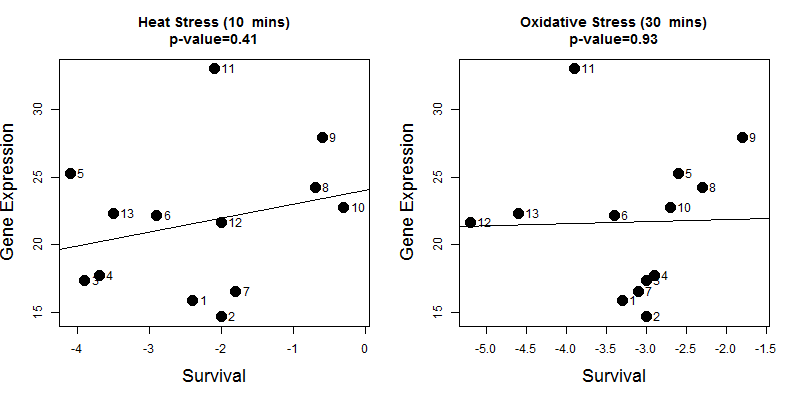

Supplement: S3 File — Expression levels of genes LLKF_0001 –LLKF_1273 plotted against survival after 10 minutes heat and 30 minutes oxidative stress. Survival is expressed as the difference of log CFU/ml after stress and before stress. Numbers indicate fermentations as presented in Table 1. P-values above the plots indicate significance of correlation (assessed by a linear model). (ZIP) [file pone.0167944.s008.zip › S3_File/LLKF_0180_real_dat.png]

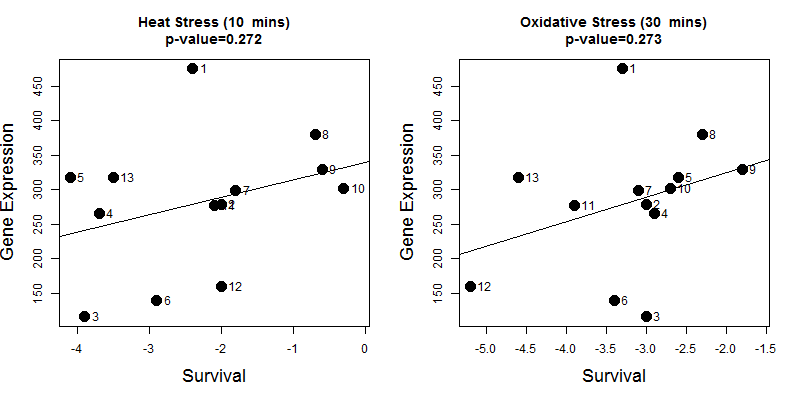

Supplement: S3 File — Expression levels of genes LLKF_0001 –LLKF_1273 plotted against survival after 10 minutes heat and 30 minutes oxidative stress. Survival is expressed as the difference of log CFU/ml after stress and before stress. Numbers indicate fermentations as presented in Table 1. P-values above the plots indicate significance of correlation (assessed by a linear model). (ZIP) [file pone.0167944.s008.zip › S3_File/LLKF_0181_real_dat.png]

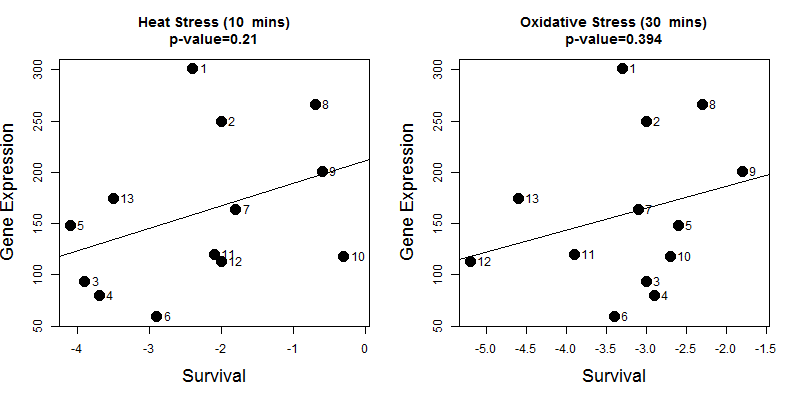

Supplement: S3 File — Expression levels of genes LLKF_0001 –LLKF_1273 plotted against survival after 10 minutes heat and 30 minutes oxidative stress. Survival is expressed as the difference of log CFU/ml after stress and before stress. Numbers indicate fermentations as presented in Table 1. P-values above the plots indicate significance of correlation (assessed by a linear model). (ZIP) [file pone.0167944.s008.zip › S3_File/LLKF_0182_real_dat.png]

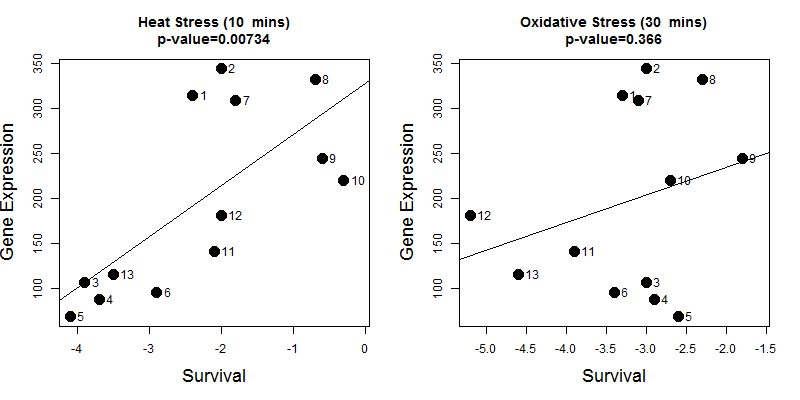

Supplement: S3 File — Expression levels of genes LLKF_0001 –LLKF_1273 plotted against survival after 10 minutes heat and 30 minutes oxidative stress. Survival is expressed as the difference of log CFU/ml after stress and before stress. Numbers indicate fermentations as presented in Table 1. P-values above the plots indicate significance of correlation (assessed by a linear model). (ZIP) [file pone.0167944.s008.zip › S3_File/LLKF_0183_real_dat.png]

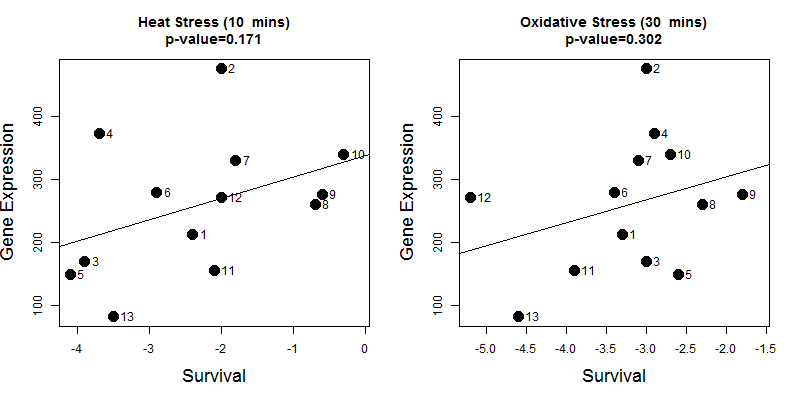

Supplement: S3 File — Expression levels of genes LLKF_0001 –LLKF_1273 plotted against survival after 10 minutes heat and 30 minutes oxidative stress. Survival is expressed as the difference of log CFU/ml after stress and before stress. Numbers indicate fermentations as presented in Table 1. P-values above the plots indicate significance of correlation (assessed by a linear model). (ZIP) [file pone.0167944.s008.zip › S3_File/LLKF_0184_real_dat.png]

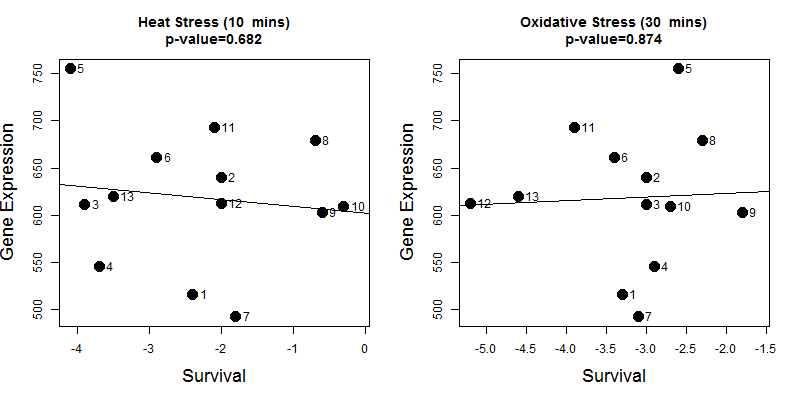

Supplement: S3 File — Expression levels of genes LLKF_0001 –LLKF_1273 plotted against survival after 10 minutes heat and 30 minutes oxidative stress. Survival is expressed as the difference of log CFU/ml after stress and before stress. Numbers indicate fermentations as presented in Table 1. P-values above the plots indicate significance of correlation (assessed by a linear model). (ZIP) [file pone.0167944.s008.zip › S3_File/LLKF_0185_real_dat.png]

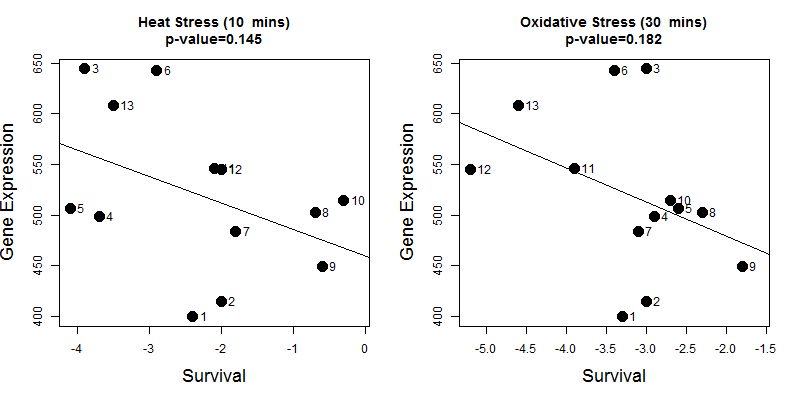

Supplement: S3 File — Expression levels of genes LLKF_0001 –LLKF_1273 plotted against survival after 10 minutes heat and 30 minutes oxidative stress. Survival is expressed as the difference of log CFU/ml after stress and before stress. Numbers indicate fermentations as presented in Table 1. P-values above the plots indicate significance of correlation (assessed by a linear model). (ZIP) [file pone.0167944.s008.zip › S3_File/LLKF_0186_real_dat.png]

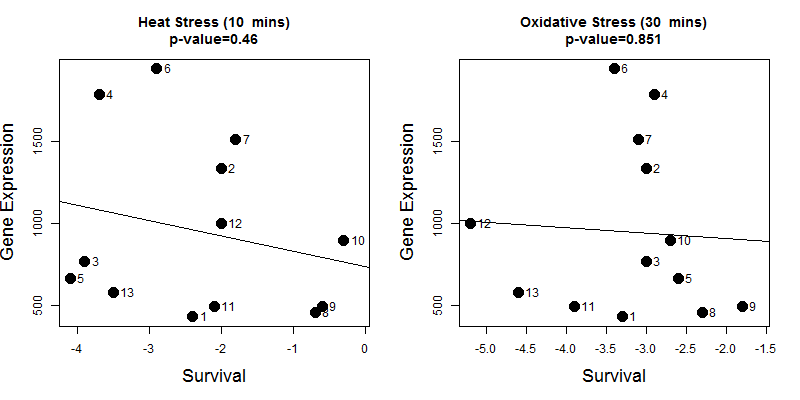

Supplement: S3 File — Expression levels of genes LLKF_0001 –LLKF_1273 plotted against survival after 10 minutes heat and 30 minutes oxidative stress. Survival is expressed as the difference of log CFU/ml after stress and before stress. Numbers indicate fermentations as presented in Table 1. P-values above the plots indicate significance of correlation (assessed by a linear model). (ZIP) [file pone.0167944.s008.zip › S3_File/LLKF_0187_real_dat.png]

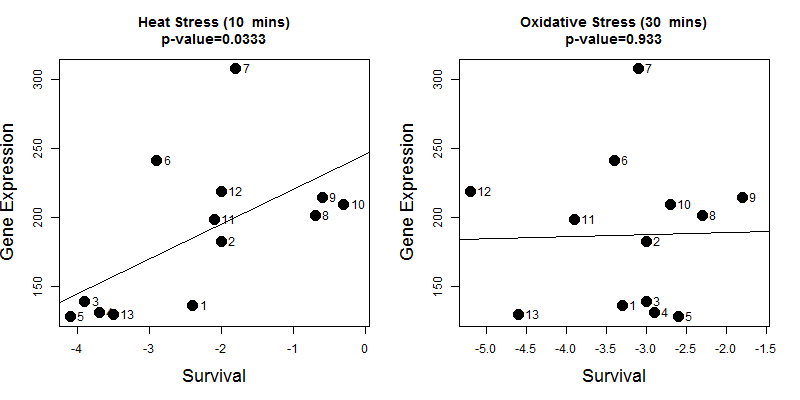

Supplement: S3 File — Expression levels of genes LLKF_0001 –LLKF_1273 plotted against survival after 10 minutes heat and 30 minutes oxidative stress. Survival is expressed as the difference of log CFU/ml after stress and before stress. Numbers indicate fermentations as presented in Table 1. P-values above the plots indicate significance of correlation (assessed by a linear model). (ZIP) [file pone.0167944.s008.zip › S3_File/LLKF_0188_real_dat.png]

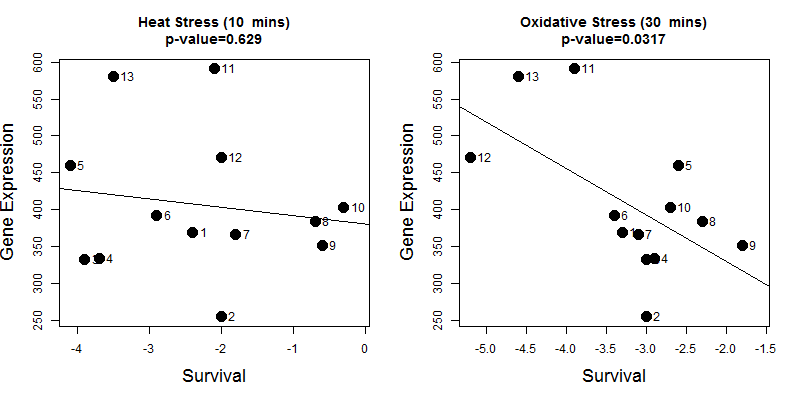

Supplement: S3 File — Expression levels of genes LLKF_0001 –LLKF_1273 plotted against survival after 10 minutes heat and 30 minutes oxidative stress. Survival is expressed as the difference of log CFU/ml after stress and before stress. Numbers indicate fermentations as presented in Table 1. P-values above the plots indicate significance of correlation (assessed by a linear model). (ZIP) [file pone.0167944.s008.zip › S3_File/LLKF_0189_real_dat.png]

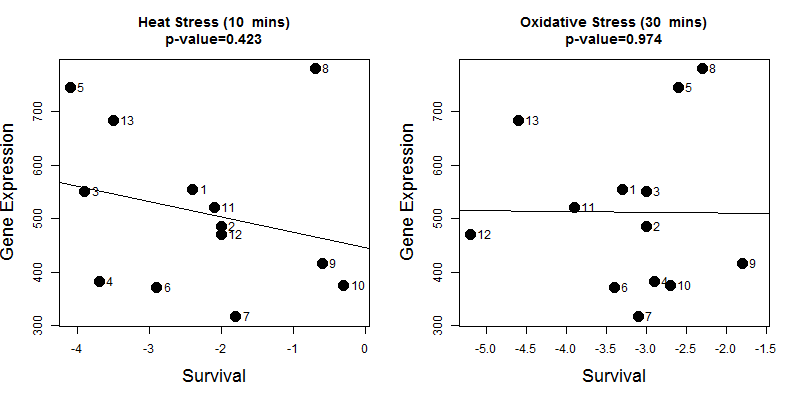

Supplement: S3 File — Expression levels of genes LLKF_0001 –LLKF_1273 plotted against survival after 10 minutes heat and 30 minutes oxidative stress. Survival is expressed as the difference of log CFU/ml after stress and before stress. Numbers indicate fermentations as presented in Table 1. P-values above the plots indicate significance of correlation (assessed by a linear model). (ZIP) [file pone.0167944.s008.zip › S3_File/LLKF_0190_real_dat.png]

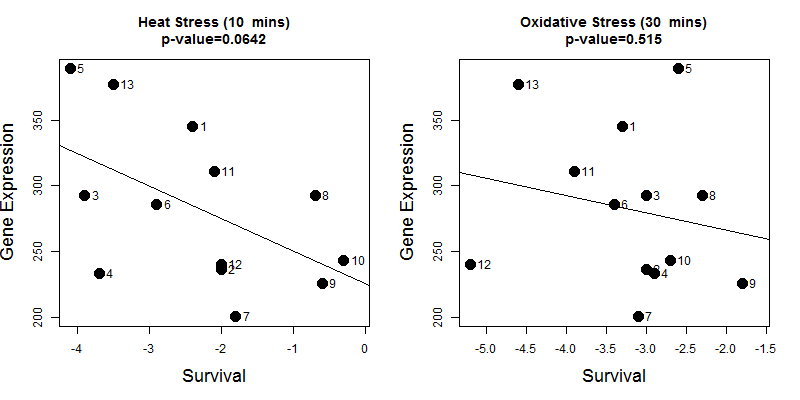

Supplement: S3 File — Expression levels of genes LLKF_0001 –LLKF_1273 plotted against survival after 10 minutes heat and 30 minutes oxidative stress. Survival is expressed as the difference of log CFU/ml after stress and before stress. Numbers indicate fermentations as presented in Table 1. P-values above the plots indicate significance of correlation (assessed by a linear model). (ZIP) [file pone.0167944.s008.zip › S3_File/LLKF_0191_real_dat.png]

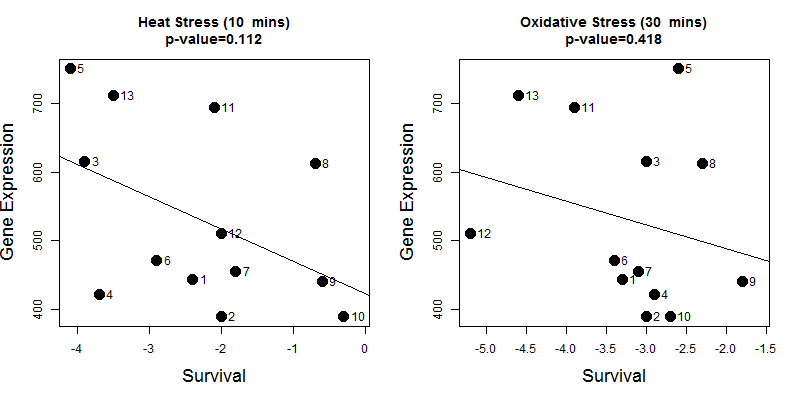

Supplement: S3 File — Expression levels of genes LLKF_0001 –LLKF_1273 plotted against survival after 10 minutes heat and 30 minutes oxidative stress. Survival is expressed as the difference of log CFU/ml after stress and before stress. Numbers indicate fermentations as presented in Table 1. P-values above the plots indicate significance of correlation (assessed by a linear model). (ZIP) [file pone.0167944.s008.zip › S3_File/LLKF_0192_real_dat.png]

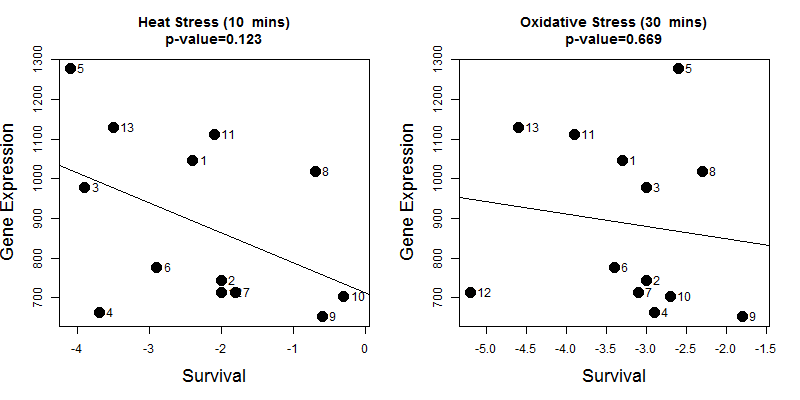

Supplement: S3 File — Expression levels of genes LLKF_0001 –LLKF_1273 plotted against survival after 10 minutes heat and 30 minutes oxidative stress. Survival is expressed as the difference of log CFU/ml after stress and before stress. Numbers indicate fermentations as presented in Table 1. P-values above the plots indicate significance of correlation (assessed by a linear model). (ZIP) [file pone.0167944.s008.zip › S3_File/LLKF_0193_real_dat.png]

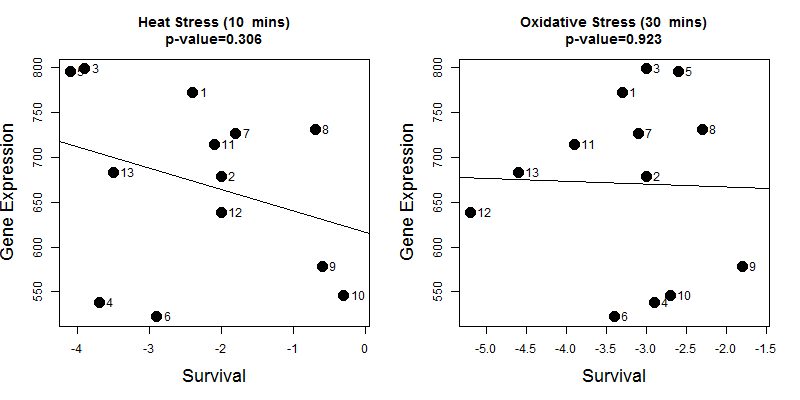

Supplement: S3 File — Expression levels of genes LLKF_0001 –LLKF_1273 plotted against survival after 10 minutes heat and 30 minutes oxidative stress. Survival is expressed as the difference of log CFU/ml after stress and before stress. Numbers indicate fermentations as presented in Table 1. P-values above the plots indicate significance of correlation (assessed by a linear model). (ZIP) [file pone.0167944.s008.zip › S3_File/LLKF_0194_real_dat.png]

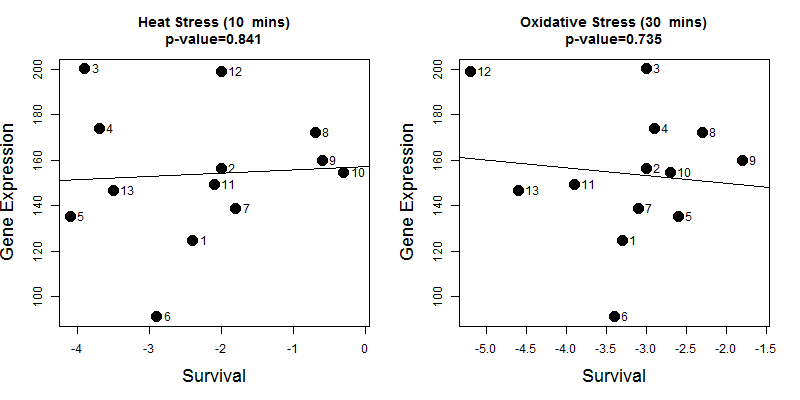

Supplement: S3 File — Expression levels of genes LLKF_0001 –LLKF_1273 plotted against survival after 10 minutes heat and 30 minutes oxidative stress. Survival is expressed as the difference of log CFU/ml after stress and before stress. Numbers indicate fermentations as presented in Table 1. P-values above the plots indicate significance of correlation (assessed by a linear model). (ZIP) [file pone.0167944.s008.zip › S3_File/LLKF_0195_real_dat.png]

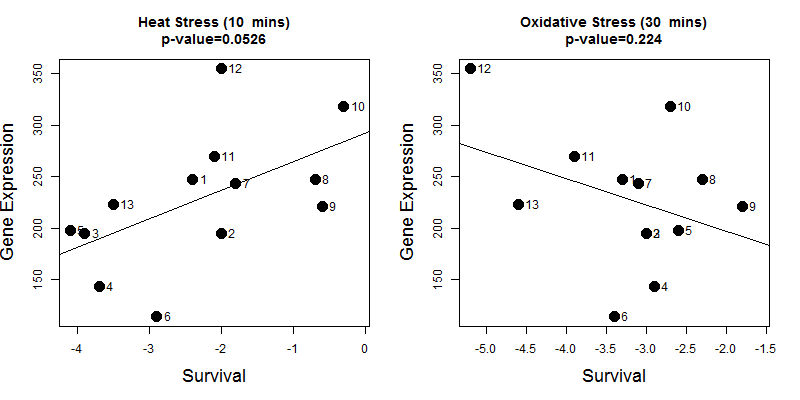

Supplement: S3 File — Expression levels of genes LLKF_0001 –LLKF_1273 plotted against survival after 10 minutes heat and 30 minutes oxidative stress. Survival is expressed as the difference of log CFU/ml after stress and before stress. Numbers indicate fermentations as presented in Table 1. P-values above the plots indicate significance of correlation (assessed by a linear model). (ZIP) [file pone.0167944.s008.zip › S3_File/LLKF_0196_real_dat.png]

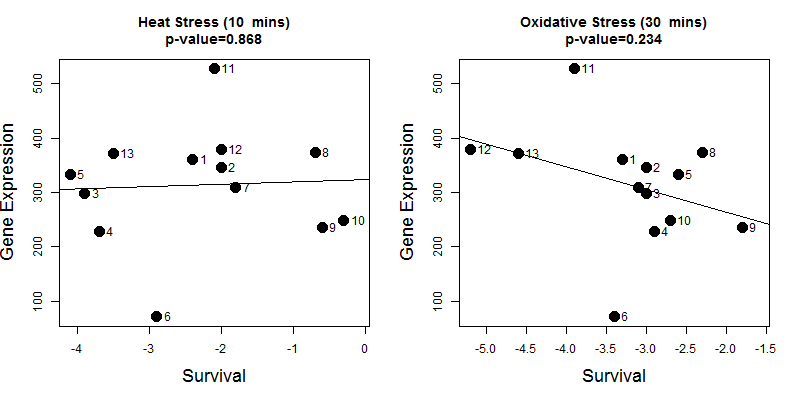

Supplement: S3 File — Expression levels of genes LLKF_0001 –LLKF_1273 plotted against survival after 10 minutes heat and 30 minutes oxidative stress. Survival is expressed as the difference of log CFU/ml after stress and before stress. Numbers indicate fermentations as presented in Table 1. P-values above the plots indicate significance of correlation (assessed by a linear model). (ZIP) [file pone.0167944.s008.zip › S3_File/LLKF_0197_real_dat.png]

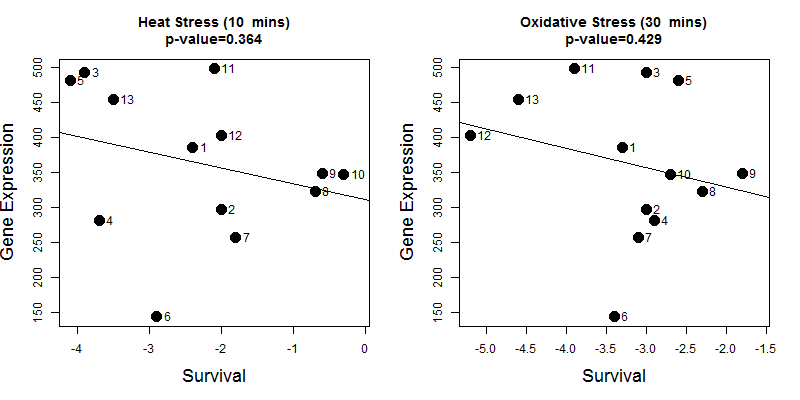

Supplement: S3 File — Expression levels of genes LLKF_0001 –LLKF_1273 plotted against survival after 10 minutes heat and 30 minutes oxidative stress. Survival is expressed as the difference of log CFU/ml after stress and before stress. Numbers indicate fermentations as presented in Table 1. P-values above the plots indicate significance of correlation (assessed by a linear model). (ZIP) [file pone.0167944.s008.zip › S3_File/LLKF_0198_real_dat.png]

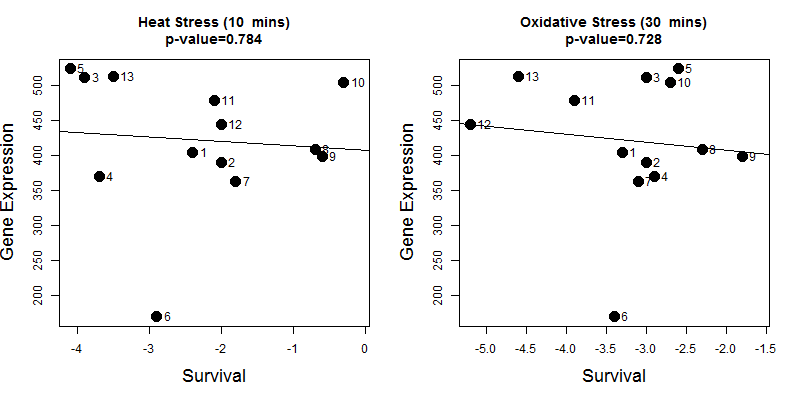

Supplement: S3 File — Expression levels of genes LLKF_0001 –LLKF_1273 plotted against survival after 10 minutes heat and 30 minutes oxidative stress. Survival is expressed as the difference of log CFU/ml after stress and before stress. Numbers indicate fermentations as presented in Table 1. P-values above the plots indicate significance of correlation (assessed by a linear model). (ZIP) [file pone.0167944.s008.zip › S3_File/LLKF_0199_real_dat.png]

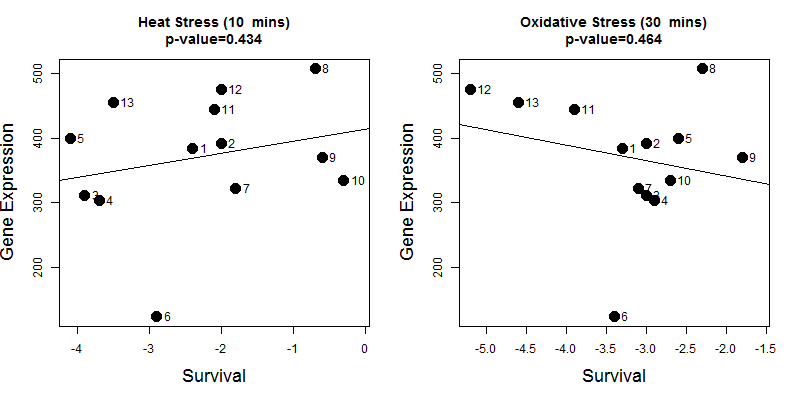

Supplement: S3 File — Expression levels of genes LLKF_0001 –LLKF_1273 plotted against survival after 10 minutes heat and 30 minutes oxidative stress. Survival is expressed as the difference of log CFU/ml after stress and before stress. Numbers indicate fermentations as presented in Table 1. P-values above the plots indicate significance of correlation (assessed by a linear model). (ZIP) [file pone.0167944.s008.zip › S3_File/LLKF_0200_real_dat.png]

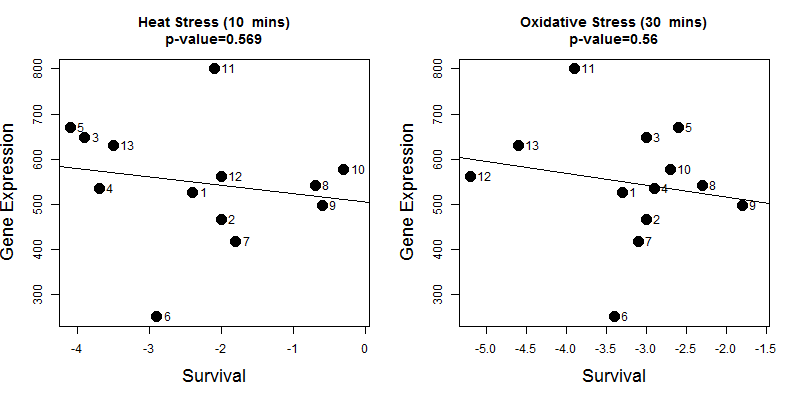

Supplement: S3 File — Expression levels of genes LLKF_0001 –LLKF_1273 plotted against survival after 10 minutes heat and 30 minutes oxidative stress. Survival is expressed as the difference of log CFU/ml after stress and before stress. Numbers indicate fermentations as presented in Table 1. P-values above the plots indicate significance of correlation (assessed by a linear model). (ZIP) [file pone.0167944.s008.zip › S3_File/LLKF_0201_real_dat.png]

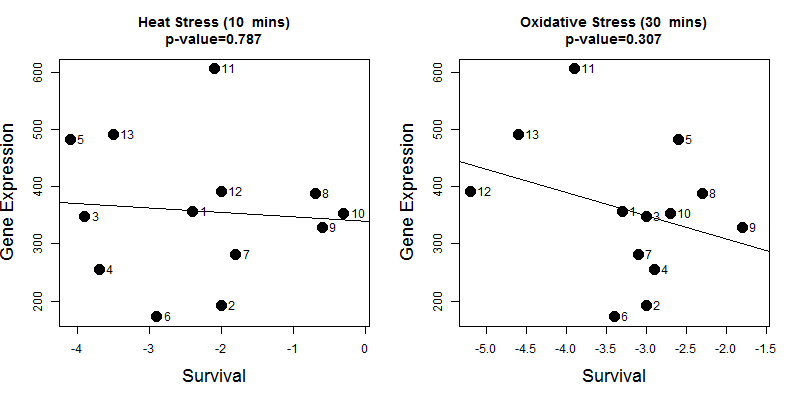

Supplement: S3 File — Expression levels of genes LLKF_0001 –LLKF_1273 plotted against survival after 10 minutes heat and 30 minutes oxidative stress. Survival is expressed as the difference of log CFU/ml after stress and before stress. Numbers indicate fermentations as presented in Table 1. P-values above the plots indicate significance of correlation (assessed by a linear model). (ZIP) [file pone.0167944.s008.zip › S3_File/LLKF_0202_real_dat.png]

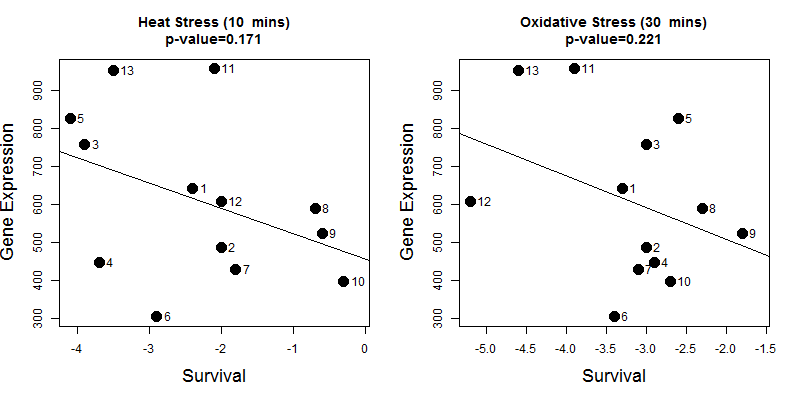

Supplement: S3 File — Expression levels of genes LLKF_0001 –LLKF_1273 plotted against survival after 10 minutes heat and 30 minutes oxidative stress. Survival is expressed as the difference of log CFU/ml after stress and before stress. Numbers indicate fermentations as presented in Table 1. P-values above the plots indicate significance of correlation (assessed by a linear model). (ZIP) [file pone.0167944.s008.zip › S3_File/LLKF_0203_real_dat.png]
