# Supplementary figures and images for: Strain-Dependent Transcriptome Signatures for Robustness in Lactococcus lactis (part 9 of 13)
Source: PLoS One. 2016 Dec 14;11(12):e0167944. doi: 10.1371/journal.pone.0167944 (PMC5156439; doi:10.1371/journal.pone.0167944)

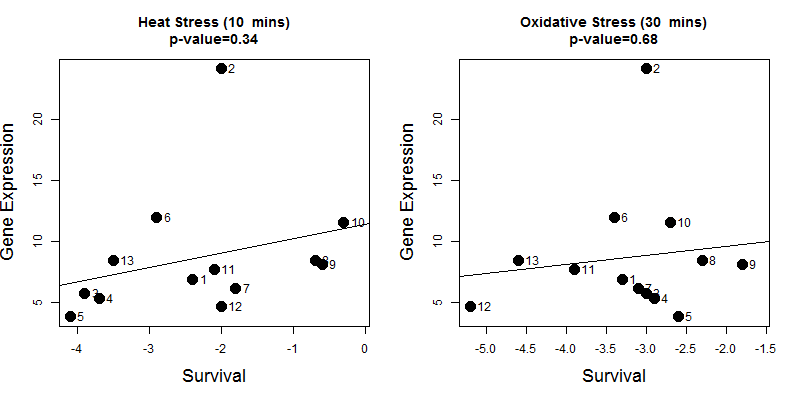

Supplement: S4 File — Expression levels of genes LLKF_1274 –LLKF_2533 and LLKF_p0001 –LLKF_p0036 plotted against survival after 10 minutes heat and 30 minutes oxidative stress. Survival is expressed as the difference of log CFU/ml after stress and before stress. Numbers indicate fermentations as presented in Table 1. P-values above the plots indicate significance of correlation (assessed by a linear model). (ZIP) [file pone.0167944.s009.zip › S4_File/LLKF_1484_real_dat.png]

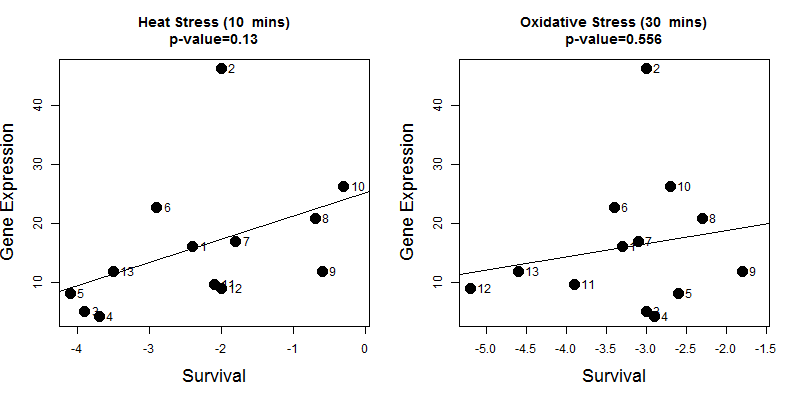

Supplement: S4 File — Expression levels of genes LLKF_1274 –LLKF_2533 and LLKF_p0001 –LLKF_p0036 plotted against survival after 10 minutes heat and 30 minutes oxidative stress. Survival is expressed as the difference of log CFU/ml after stress and before stress. Numbers indicate fermentations as presented in Table 1. P-values above the plots indicate significance of correlation (assessed by a linear model). (ZIP) [file pone.0167944.s009.zip › S4_File/LLKF_1485_real_dat.png]

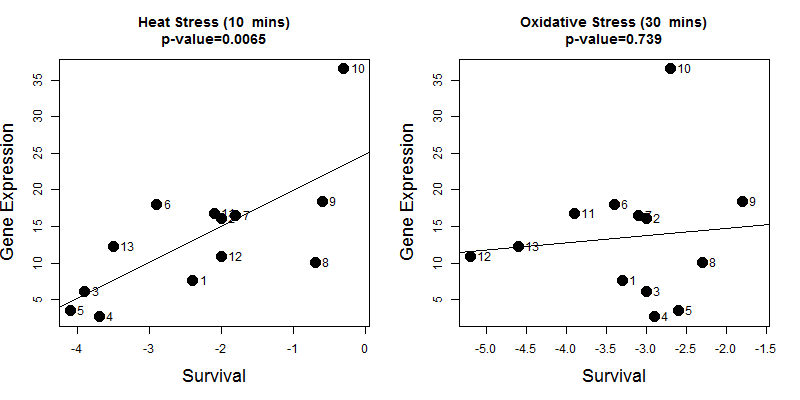

Supplement: S4 File — Expression levels of genes LLKF_1274 –LLKF_2533 and LLKF_p0001 –LLKF_p0036 plotted against survival after 10 minutes heat and 30 minutes oxidative stress. Survival is expressed as the difference of log CFU/ml after stress and before stress. Numbers indicate fermentations as presented in Table 1. P-values above the plots indicate significance of correlation (assessed by a linear model). (ZIP) [file pone.0167944.s009.zip › S4_File/LLKF_1486_real_dat.png]

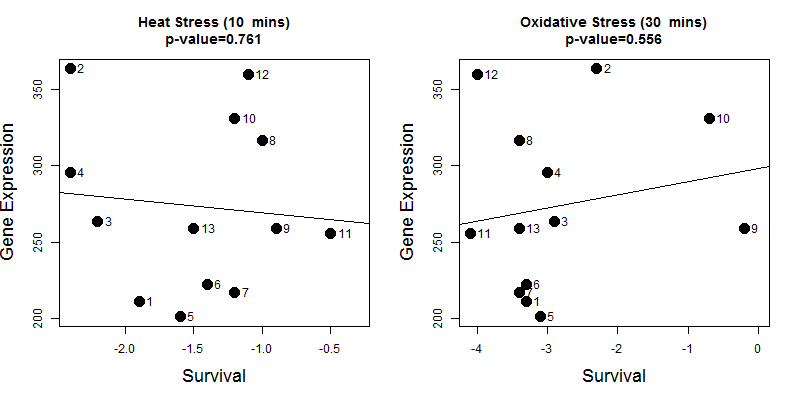

Supplement: S5 File — Expression levels of genes LACR_0001 –LACR_1382 plotted against survival after 10 minutes heat and 30 minutes oxidative stress. Survival is expressed as the difference of log CFU/ml after stress and before stress. Numbers indicate fermentations as presented in Table 1. P-values above the plots indicate significance of correlation (assessed by a linear model). (ZIP) [file pone.0167944.s010.zip › S5_File/LACR_0001_real_dat.png]

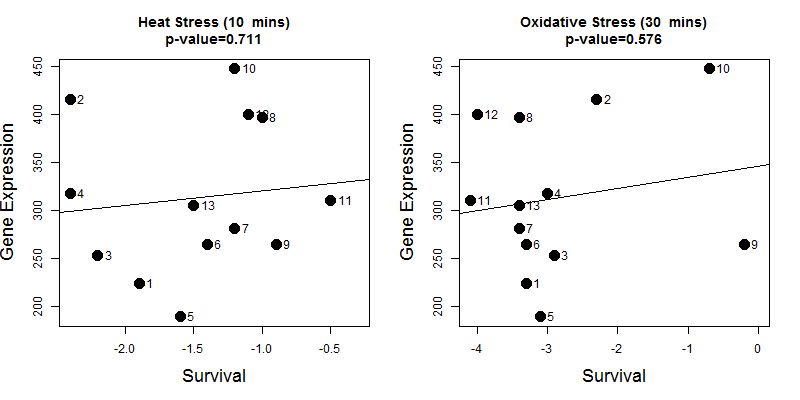

Supplement: S5 File — Expression levels of genes LACR_0001 –LACR_1382 plotted against survival after 10 minutes heat and 30 minutes oxidative stress. Survival is expressed as the difference of log CFU/ml after stress and before stress. Numbers indicate fermentations as presented in Table 1. P-values above the plots indicate significance of correlation (assessed by a linear model). (ZIP) [file pone.0167944.s010.zip › S5_File/LACR_0002_real_dat.png]

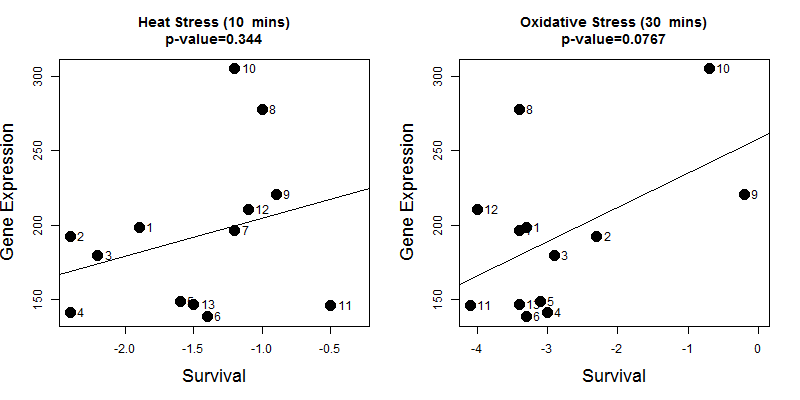

Supplement: S5 File — Expression levels of genes LACR_0001 –LACR_1382 plotted against survival after 10 minutes heat and 30 minutes oxidative stress. Survival is expressed as the difference of log CFU/ml after stress and before stress. Numbers indicate fermentations as presented in Table 1. P-values above the plots indicate significance of correlation (assessed by a linear model). (ZIP) [file pone.0167944.s010.zip › S5_File/LACR_0003_real_dat.png]

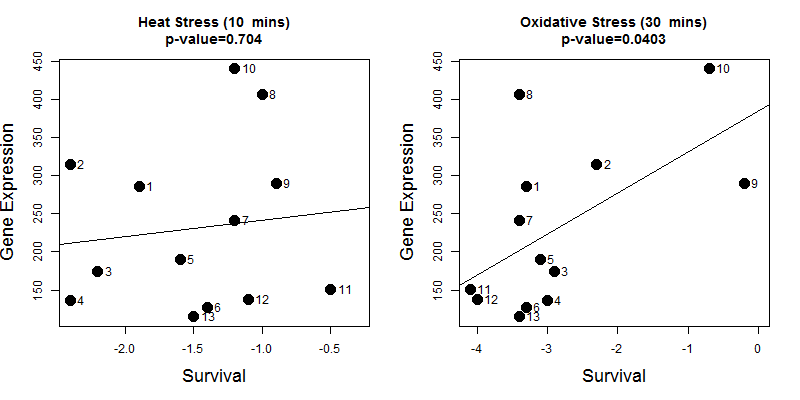

Supplement: S5 File — Expression levels of genes LACR_0001 –LACR_1382 plotted against survival after 10 minutes heat and 30 minutes oxidative stress. Survival is expressed as the difference of log CFU/ml after stress and before stress. Numbers indicate fermentations as presented in Table 1. P-values above the plots indicate significance of correlation (assessed by a linear model). (ZIP) [file pone.0167944.s010.zip › S5_File/LACR_0004_real_dat.png]

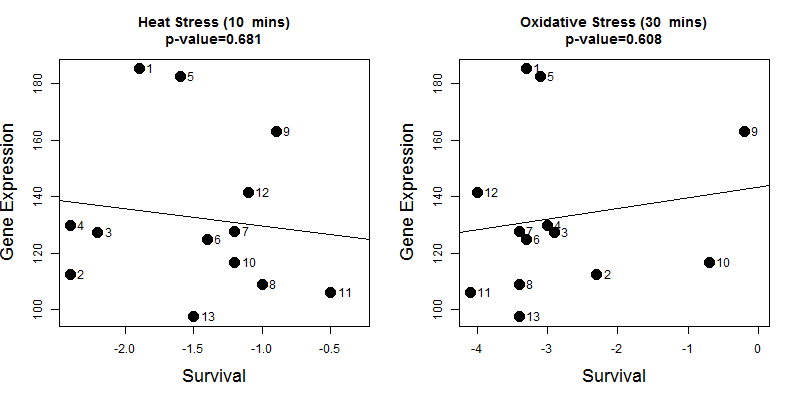

Supplement: S5 File — Expression levels of genes LACR_0001 –LACR_1382 plotted against survival after 10 minutes heat and 30 minutes oxidative stress. Survival is expressed as the difference of log CFU/ml after stress and before stress. Numbers indicate fermentations as presented in Table 1. P-values above the plots indicate significance of correlation (assessed by a linear model). (ZIP) [file pone.0167944.s010.zip › S5_File/LACR_0005_real_dat.png]

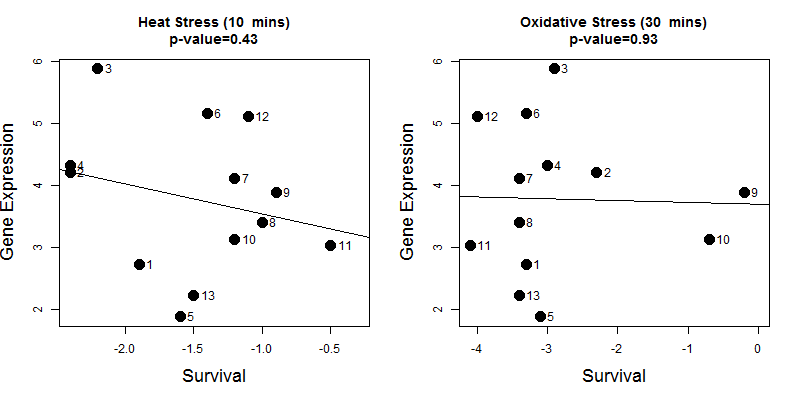

Supplement: S5 File — Expression levels of genes LACR_0001 –LACR_1382 plotted against survival after 10 minutes heat and 30 minutes oxidative stress. Survival is expressed as the difference of log CFU/ml after stress and before stress. Numbers indicate fermentations as presented in Table 1. P-values above the plots indicate significance of correlation (assessed by a linear model). (ZIP) [file pone.0167944.s010.zip › S5_File/LACR_0006_real_dat.png]

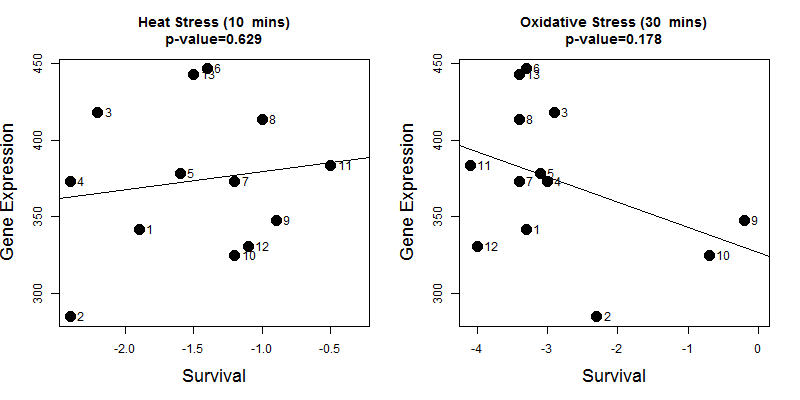

Supplement: S5 File — Expression levels of genes LACR_0001 –LACR_1382 plotted against survival after 10 minutes heat and 30 minutes oxidative stress. Survival is expressed as the difference of log CFU/ml after stress and before stress. Numbers indicate fermentations as presented in Table 1. P-values above the plots indicate significance of correlation (assessed by a linear model). (ZIP) [file pone.0167944.s010.zip › S5_File/LACR_0007_real_dat.png]

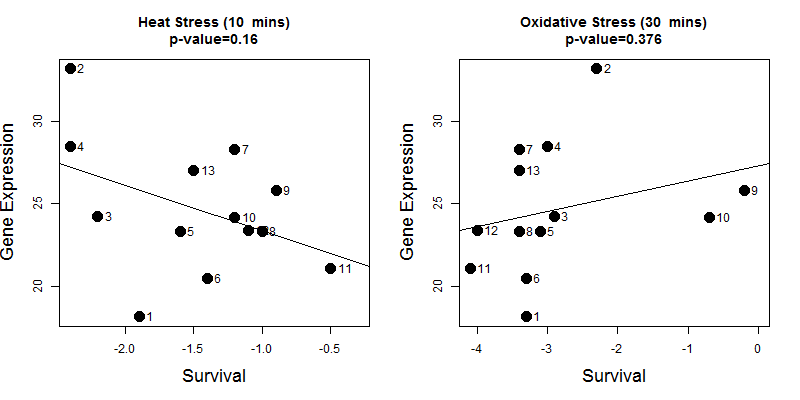

Supplement: S5 File — Expression levels of genes LACR_0001 –LACR_1382 plotted against survival after 10 minutes heat and 30 minutes oxidative stress. Survival is expressed as the difference of log CFU/ml after stress and before stress. Numbers indicate fermentations as presented in Table 1. P-values above the plots indicate significance of correlation (assessed by a linear model). (ZIP) [file pone.0167944.s010.zip › S5_File/LACR_0008_real_dat.png]

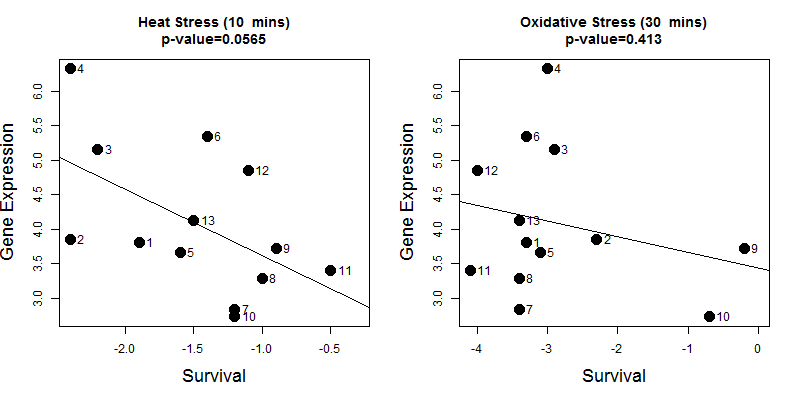

Supplement: S5 File — Expression levels of genes LACR_0001 –LACR_1382 plotted against survival after 10 minutes heat and 30 minutes oxidative stress. Survival is expressed as the difference of log CFU/ml after stress and before stress. Numbers indicate fermentations as presented in Table 1. P-values above the plots indicate significance of correlation (assessed by a linear model). (ZIP) [file pone.0167944.s010.zip › S5_File/LACR_0009_real_dat.png]

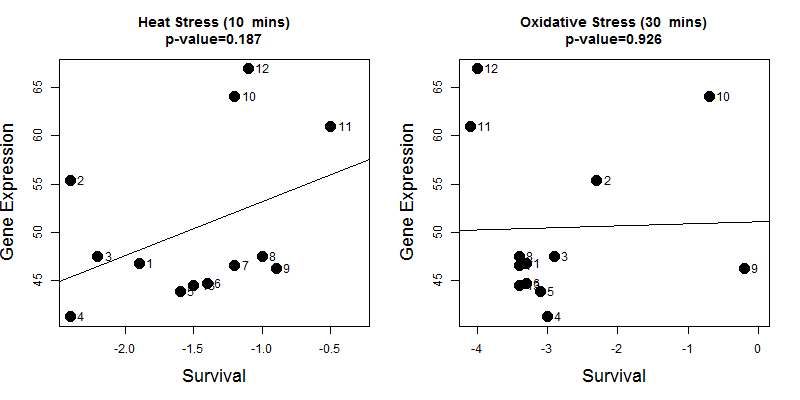

Supplement: S5 File — Expression levels of genes LACR_0001 –LACR_1382 plotted against survival after 10 minutes heat and 30 minutes oxidative stress. Survival is expressed as the difference of log CFU/ml after stress and before stress. Numbers indicate fermentations as presented in Table 1. P-values above the plots indicate significance of correlation (assessed by a linear model). (ZIP) [file pone.0167944.s010.zip › S5_File/LACR_0010_real_dat.png]

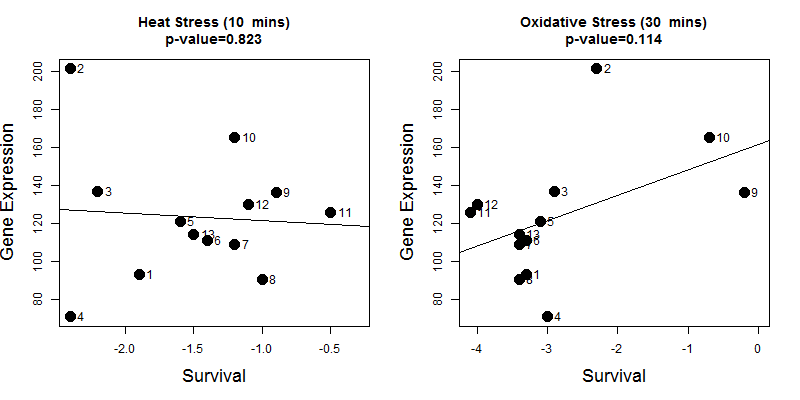

Supplement: S5 File — Expression levels of genes LACR_0001 –LACR_1382 plotted against survival after 10 minutes heat and 30 minutes oxidative stress. Survival is expressed as the difference of log CFU/ml after stress and before stress. Numbers indicate fermentations as presented in Table 1. P-values above the plots indicate significance of correlation (assessed by a linear model). (ZIP) [file pone.0167944.s010.zip › S5_File/LACR_0011_real_dat.png]

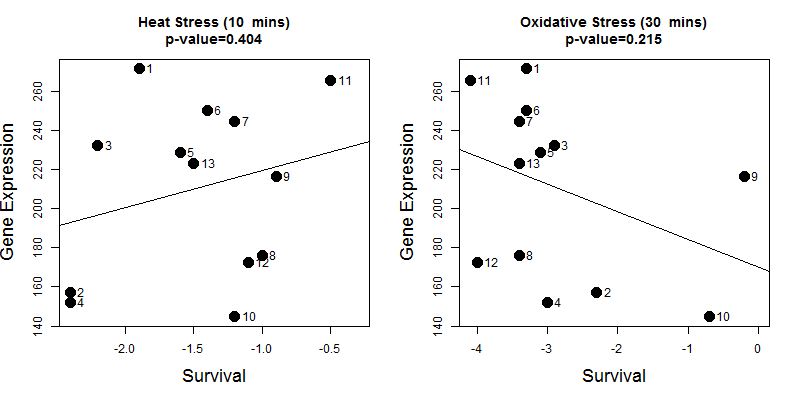

Supplement: S5 File — Expression levels of genes LACR_0001 –LACR_1382 plotted against survival after 10 minutes heat and 30 minutes oxidative stress. Survival is expressed as the difference of log CFU/ml after stress and before stress. Numbers indicate fermentations as presented in Table 1. P-values above the plots indicate significance of correlation (assessed by a linear model). (ZIP) [file pone.0167944.s010.zip › S5_File/LACR_0012_real_dat.png]

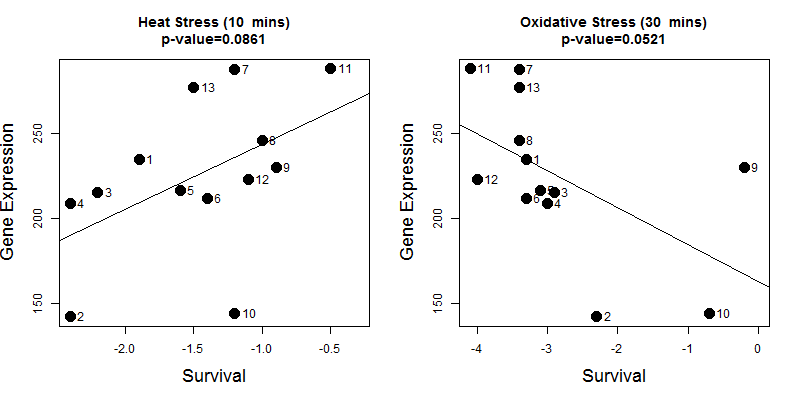

Supplement: S5 File — Expression levels of genes LACR_0001 –LACR_1382 plotted against survival after 10 minutes heat and 30 minutes oxidative stress. Survival is expressed as the difference of log CFU/ml after stress and before stress. Numbers indicate fermentations as presented in Table 1. P-values above the plots indicate significance of correlation (assessed by a linear model). (ZIP) [file pone.0167944.s010.zip › S5_File/LACR_0013_real_dat.png]

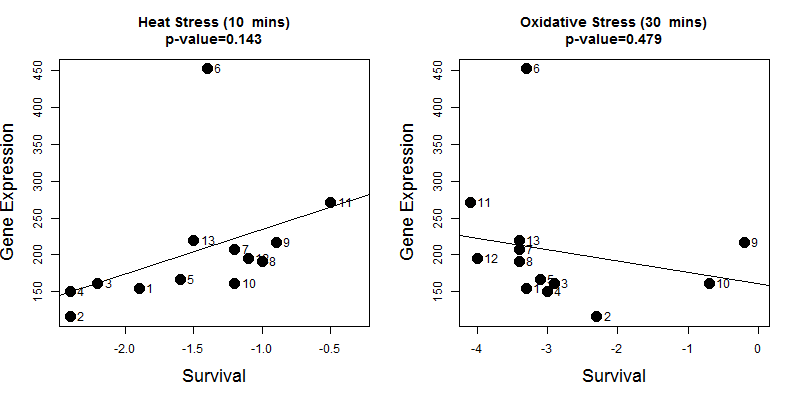

Supplement: S5 File — Expression levels of genes LACR_0001 –LACR_1382 plotted against survival after 10 minutes heat and 30 minutes oxidative stress. Survival is expressed as the difference of log CFU/ml after stress and before stress. Numbers indicate fermentations as presented in Table 1. P-values above the plots indicate significance of correlation (assessed by a linear model). (ZIP) [file pone.0167944.s010.zip › S5_File/LACR_0014_real_dat.png]

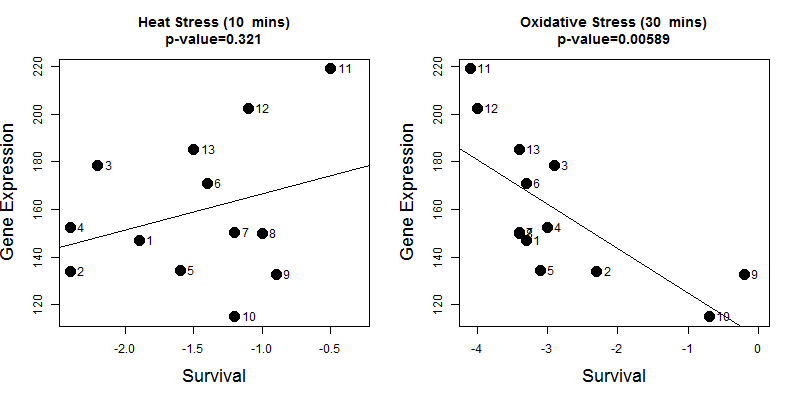

Supplement: S5 File — Expression levels of genes LACR_0001 –LACR_1382 plotted against survival after 10 minutes heat and 30 minutes oxidative stress. Survival is expressed as the difference of log CFU/ml after stress and before stress. Numbers indicate fermentations as presented in Table 1. P-values above the plots indicate significance of correlation (assessed by a linear model). (ZIP) [file pone.0167944.s010.zip › S5_File/LACR_0015_real_dat.png]

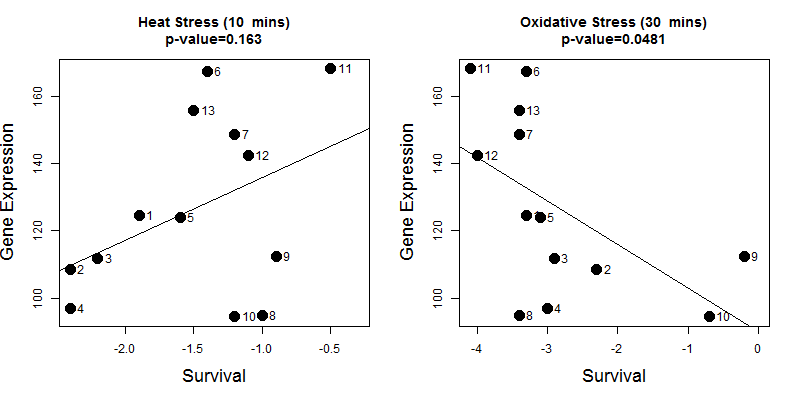

Supplement: S5 File — Expression levels of genes LACR_0001 –LACR_1382 plotted against survival after 10 minutes heat and 30 minutes oxidative stress. Survival is expressed as the difference of log CFU/ml after stress and before stress. Numbers indicate fermentations as presented in Table 1. P-values above the plots indicate significance of correlation (assessed by a linear model). (ZIP) [file pone.0167944.s010.zip › S5_File/LACR_0016_real_dat.png]

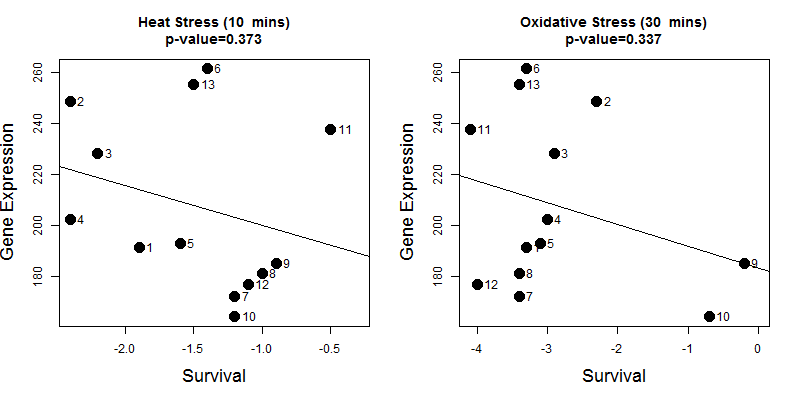

Supplement: S5 File — Expression levels of genes LACR_0001 –LACR_1382 plotted against survival after 10 minutes heat and 30 minutes oxidative stress. Survival is expressed as the difference of log CFU/ml after stress and before stress. Numbers indicate fermentations as presented in Table 1. P-values above the plots indicate significance of correlation (assessed by a linear model). (ZIP) [file pone.0167944.s010.zip › S5_File/LACR_0017_real_dat.png]

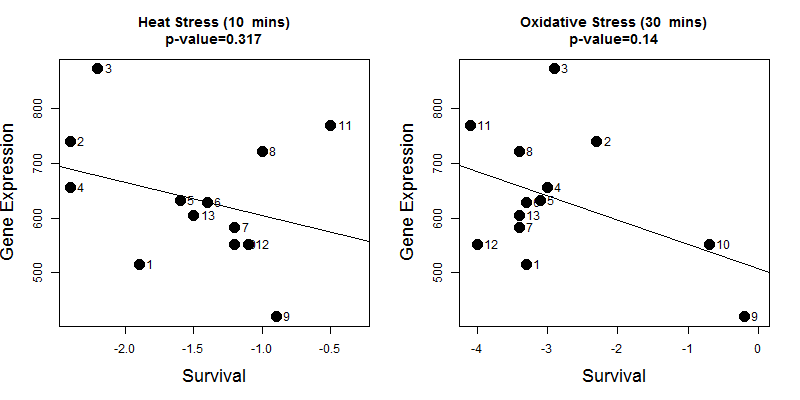

Supplement: S5 File — Expression levels of genes LACR_0001 –LACR_1382 plotted against survival after 10 minutes heat and 30 minutes oxidative stress. Survival is expressed as the difference of log CFU/ml after stress and before stress. Numbers indicate fermentations as presented in Table 1. P-values above the plots indicate significance of correlation (assessed by a linear model). (ZIP) [file pone.0167944.s010.zip › S5_File/LACR_0018_real_dat.png]

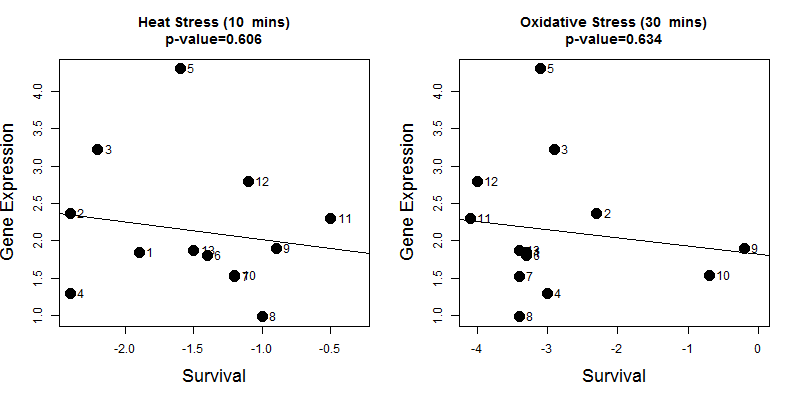

Supplement: S5 File — Expression levels of genes LACR_0001 –LACR_1382 plotted against survival after 10 minutes heat and 30 minutes oxidative stress. Survival is expressed as the difference of log CFU/ml after stress and before stress. Numbers indicate fermentations as presented in Table 1. P-values above the plots indicate significance of correlation (assessed by a linear model). (ZIP) [file pone.0167944.s010.zip › S5_File/LACR_0027_real_dat.png]

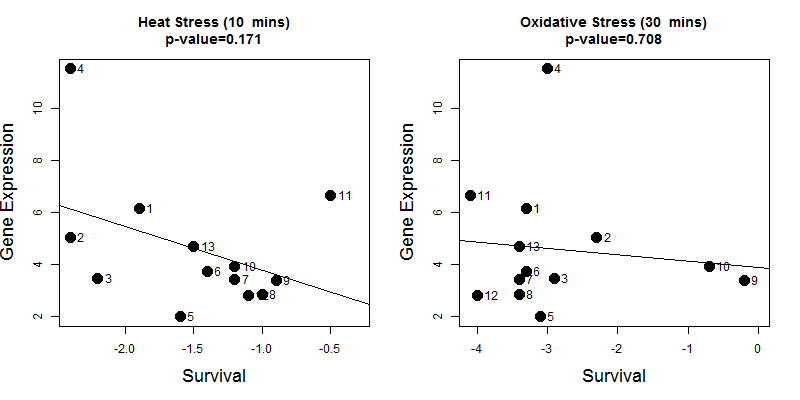

Supplement: S5 File — Expression levels of genes LACR_0001 –LACR_1382 plotted against survival after 10 minutes heat and 30 minutes oxidative stress. Survival is expressed as the difference of log CFU/ml after stress and before stress. Numbers indicate fermentations as presented in Table 1. P-values above the plots indicate significance of correlation (assessed by a linear model). (ZIP) [file pone.0167944.s010.zip › S5_File/LACR_0028_real_dat.png]

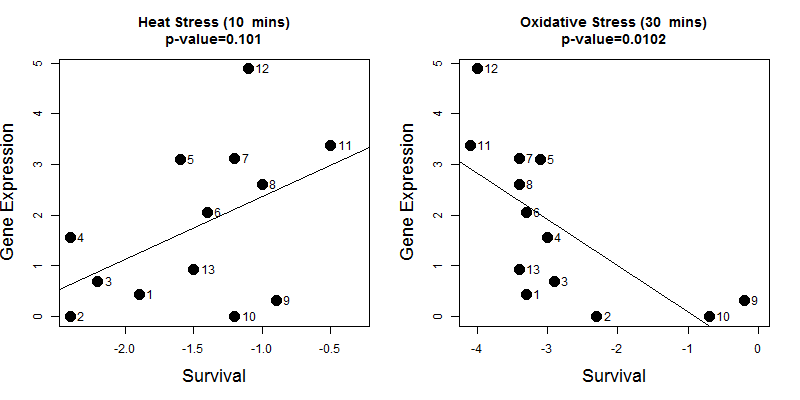

Supplement: S5 File — Expression levels of genes LACR_0001 –LACR_1382 plotted against survival after 10 minutes heat and 30 minutes oxidative stress. Survival is expressed as the difference of log CFU/ml after stress and before stress. Numbers indicate fermentations as presented in Table 1. P-values above the plots indicate significance of correlation (assessed by a linear model). (ZIP) [file pone.0167944.s010.zip › S5_File/LACR_0029_real_dat.png]

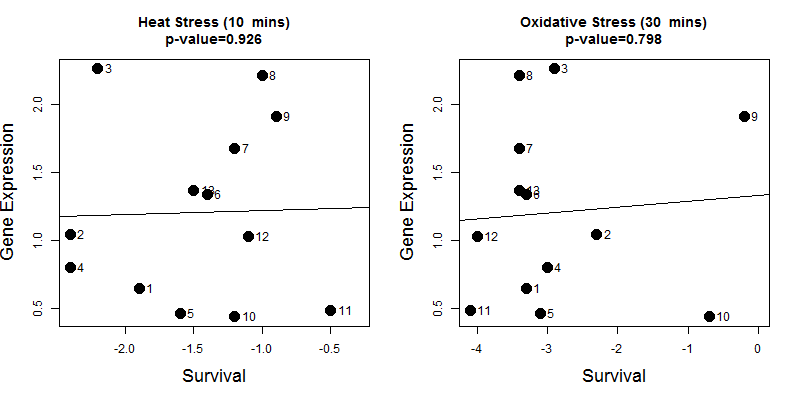

Supplement: S5 File — Expression levels of genes LACR_0001 –LACR_1382 plotted against survival after 10 minutes heat and 30 minutes oxidative stress. Survival is expressed as the difference of log CFU/ml after stress and before stress. Numbers indicate fermentations as presented in Table 1. P-values above the plots indicate significance of correlation (assessed by a linear model). (ZIP) [file pone.0167944.s010.zip › S5_File/LACR_0030_real_dat.png]

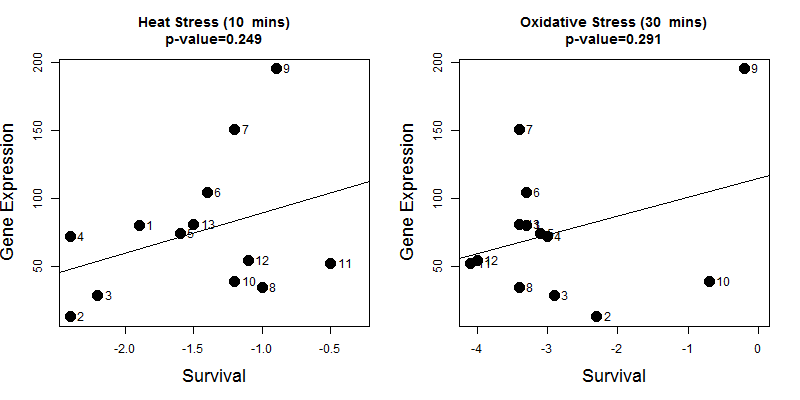

Supplement: S5 File — Expression levels of genes LACR_0001 –LACR_1382 plotted against survival after 10 minutes heat and 30 minutes oxidative stress. Survival is expressed as the difference of log CFU/ml after stress and before stress. Numbers indicate fermentations as presented in Table 1. P-values above the plots indicate significance of correlation (assessed by a linear model). (ZIP) [file pone.0167944.s010.zip › S5_File/LACR_0031_real_dat.png]

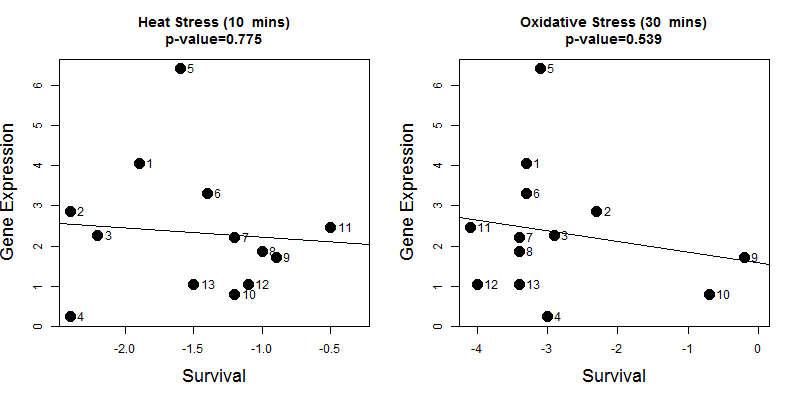

Supplement: S5 File — Expression levels of genes LACR_0001 –LACR_1382 plotted against survival after 10 minutes heat and 30 minutes oxidative stress. Survival is expressed as the difference of log CFU/ml after stress and before stress. Numbers indicate fermentations as presented in Table 1. P-values above the plots indicate significance of correlation (assessed by a linear model). (ZIP) [file pone.0167944.s010.zip › S5_File/LACR_0032_real_dat.png]

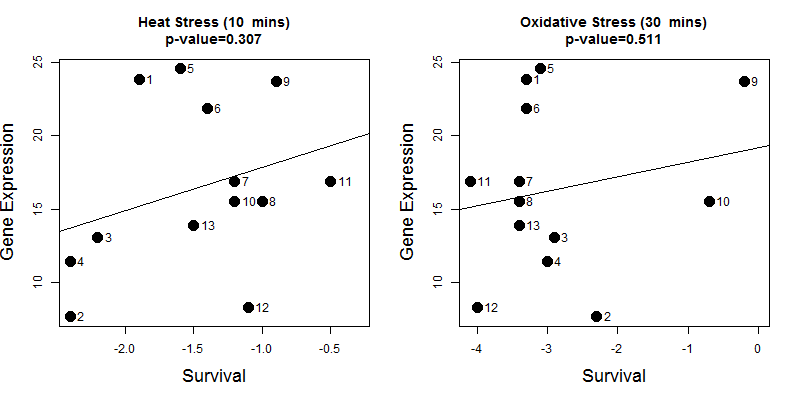

Supplement: S5 File — Expression levels of genes LACR_0001 –LACR_1382 plotted against survival after 10 minutes heat and 30 minutes oxidative stress. Survival is expressed as the difference of log CFU/ml after stress and before stress. Numbers indicate fermentations as presented in Table 1. P-values above the plots indicate significance of correlation (assessed by a linear model). (ZIP) [file pone.0167944.s010.zip › S5_File/LACR_0033_real_dat.png]

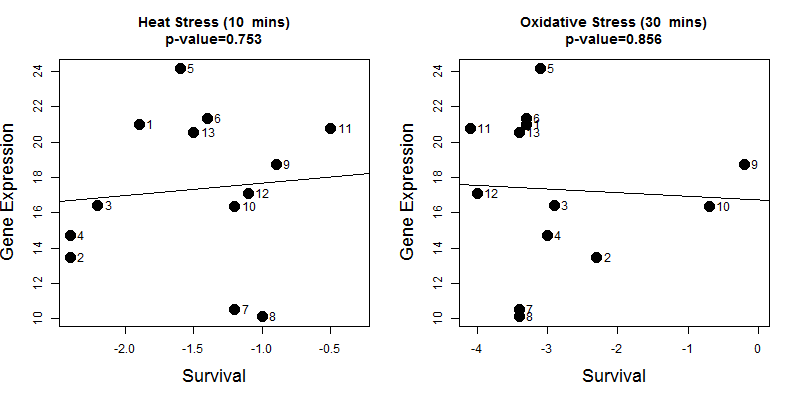

Supplement: S5 File — Expression levels of genes LACR_0001 –LACR_1382 plotted against survival after 10 minutes heat and 30 minutes oxidative stress. Survival is expressed as the difference of log CFU/ml after stress and before stress. Numbers indicate fermentations as presented in Table 1. P-values above the plots indicate significance of correlation (assessed by a linear model). (ZIP) [file pone.0167944.s010.zip › S5_File/LACR_0034_real_dat.png]

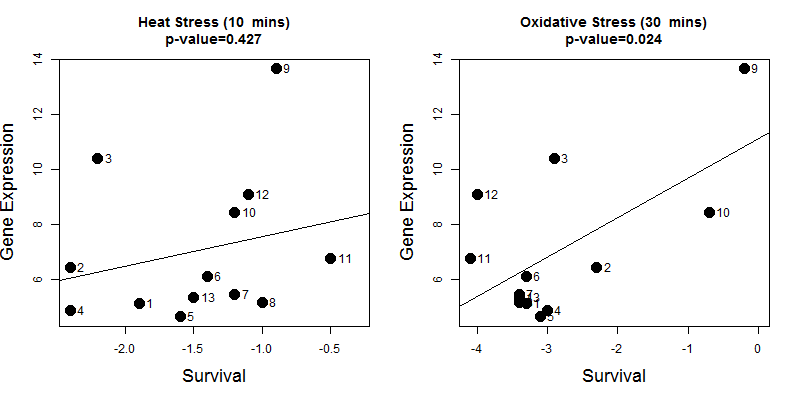

Supplement: S5 File — Expression levels of genes LACR_0001 –LACR_1382 plotted against survival after 10 minutes heat and 30 minutes oxidative stress. Survival is expressed as the difference of log CFU/ml after stress and before stress. Numbers indicate fermentations as presented in Table 1. P-values above the plots indicate significance of correlation (assessed by a linear model). (ZIP) [file pone.0167944.s010.zip › S5_File/LACR_0035_real_dat.png]

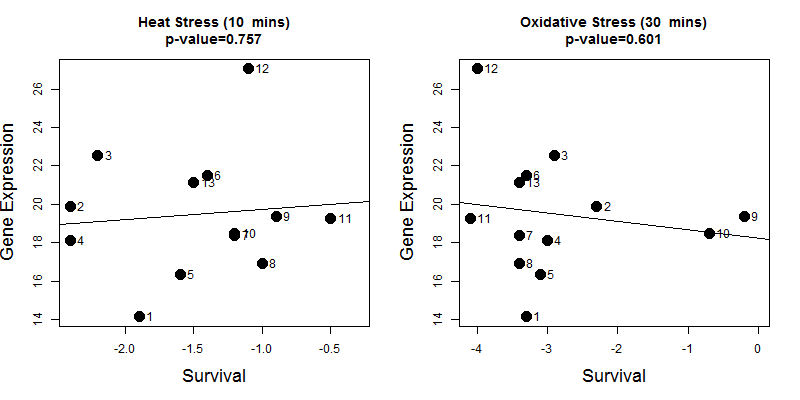

Supplement: S5 File — Expression levels of genes LACR_0001 –LACR_1382 plotted against survival after 10 minutes heat and 30 minutes oxidative stress. Survival is expressed as the difference of log CFU/ml after stress and before stress. Numbers indicate fermentations as presented in Table 1. P-values above the plots indicate significance of correlation (assessed by a linear model). (ZIP) [file pone.0167944.s010.zip › S5_File/LACR_0036_real_dat.png]

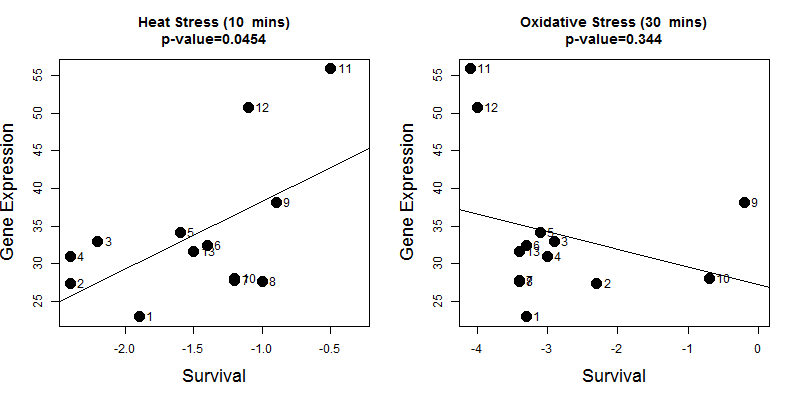

Supplement: S5 File — Expression levels of genes LACR_0001 –LACR_1382 plotted against survival after 10 minutes heat and 30 minutes oxidative stress. Survival is expressed as the difference of log CFU/ml after stress and before stress. Numbers indicate fermentations as presented in Table 1. P-values above the plots indicate significance of correlation (assessed by a linear model). (ZIP) [file pone.0167944.s010.zip › S5_File/LACR_0037_real_dat.png]

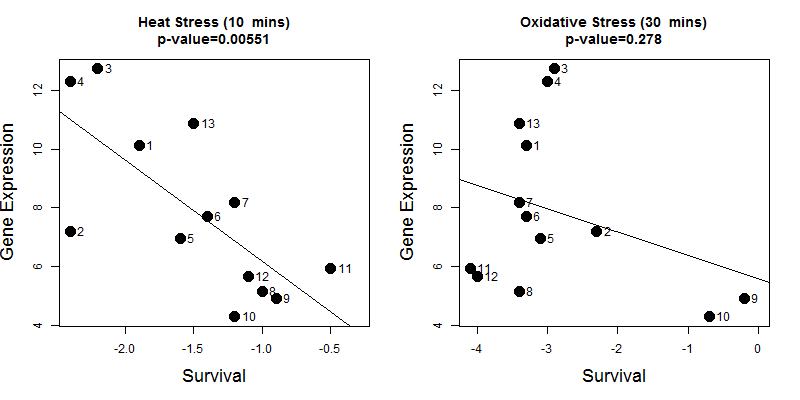

Supplement: S5 File — Expression levels of genes LACR_0001 –LACR_1382 plotted against survival after 10 minutes heat and 30 minutes oxidative stress. Survival is expressed as the difference of log CFU/ml after stress and before stress. Numbers indicate fermentations as presented in Table 1. P-values above the plots indicate significance of correlation (assessed by a linear model). (ZIP) [file pone.0167944.s010.zip › S5_File/LACR_0040_real_dat.png]

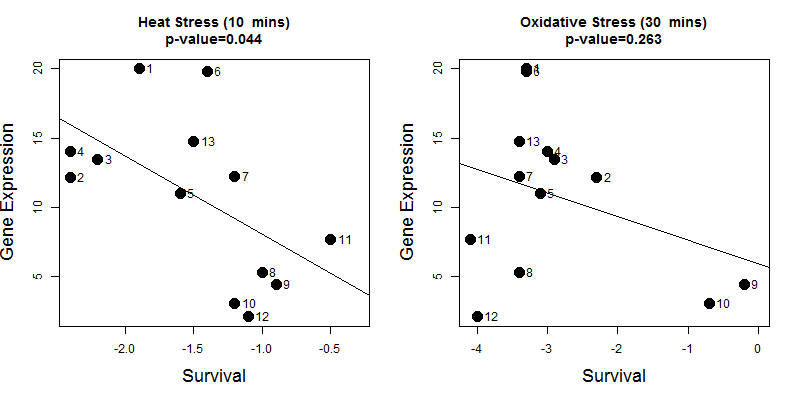

Supplement: S5 File — Expression levels of genes LACR_0001 –LACR_1382 plotted against survival after 10 minutes heat and 30 minutes oxidative stress. Survival is expressed as the difference of log CFU/ml after stress and before stress. Numbers indicate fermentations as presented in Table 1. P-values above the plots indicate significance of correlation (assessed by a linear model). (ZIP) [file pone.0167944.s010.zip › S5_File/LACR_0041_real_dat.png]

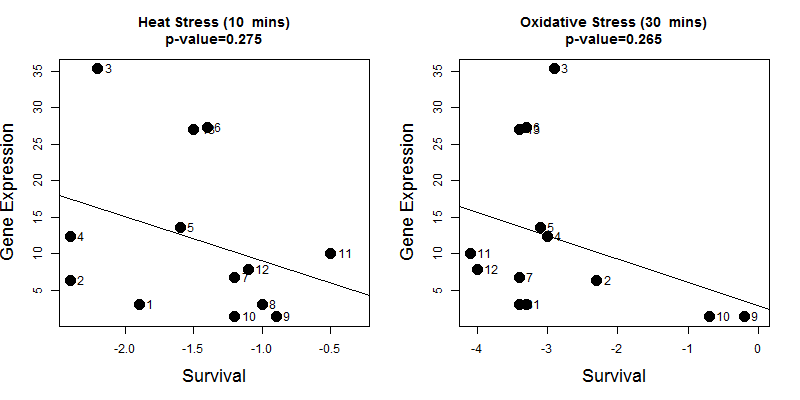

Supplement: S5 File — Expression levels of genes LACR_0001 –LACR_1382 plotted against survival after 10 minutes heat and 30 minutes oxidative stress. Survival is expressed as the difference of log CFU/ml after stress and before stress. Numbers indicate fermentations as presented in Table 1. P-values above the plots indicate significance of correlation (assessed by a linear model). (ZIP) [file pone.0167944.s010.zip › S5_File/LACR_0042_real_dat.png]

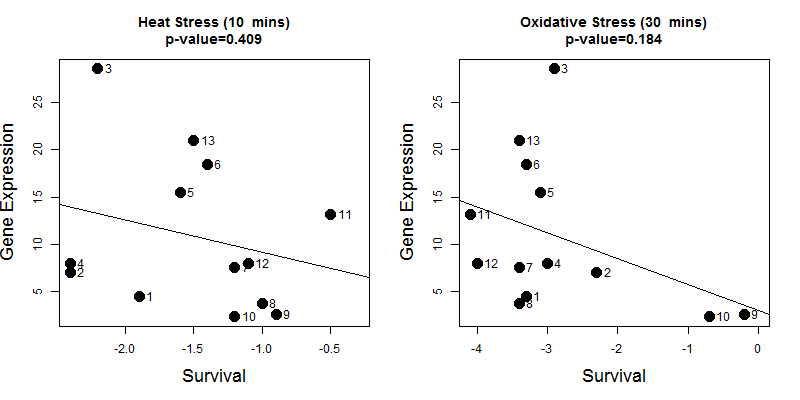

Supplement: S5 File — Expression levels of genes LACR_0001 –LACR_1382 plotted against survival after 10 minutes heat and 30 minutes oxidative stress. Survival is expressed as the difference of log CFU/ml after stress and before stress. Numbers indicate fermentations as presented in Table 1. P-values above the plots indicate significance of correlation (assessed by a linear model). (ZIP) [file pone.0167944.s010.zip › S5_File/LACR_0043_real_dat.png]

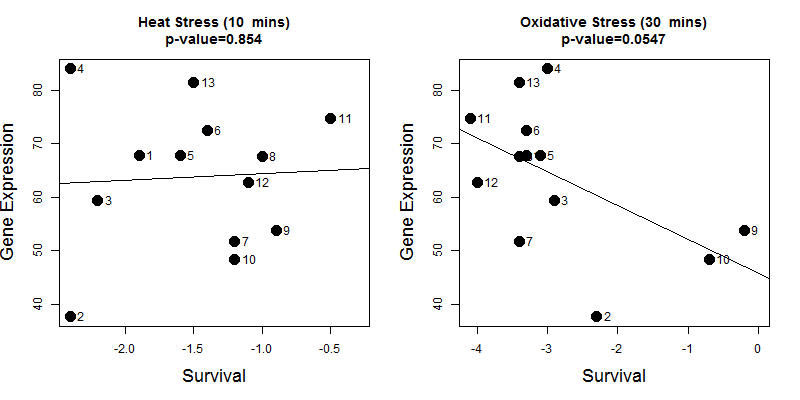

Supplement: S5 File — Expression levels of genes LACR_0001 –LACR_1382 plotted against survival after 10 minutes heat and 30 minutes oxidative stress. Survival is expressed as the difference of log CFU/ml after stress and before stress. Numbers indicate fermentations as presented in Table 1. P-values above the plots indicate significance of correlation (assessed by a linear model). (ZIP) [file pone.0167944.s010.zip › S5_File/LACR_0044_real_dat.png]

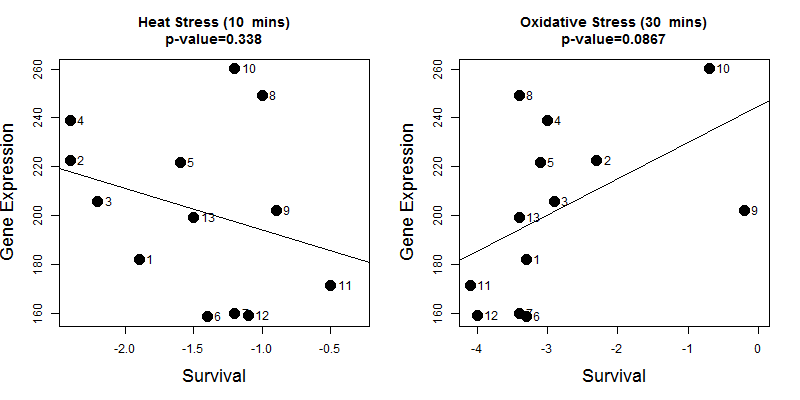

Supplement: S5 File — Expression levels of genes LACR_0001 –LACR_1382 plotted against survival after 10 minutes heat and 30 minutes oxidative stress. Survival is expressed as the difference of log CFU/ml after stress and before stress. Numbers indicate fermentations as presented in Table 1. P-values above the plots indicate significance of correlation (assessed by a linear model). (ZIP) [file pone.0167944.s010.zip › S5_File/LACR_0046_real_dat.png]

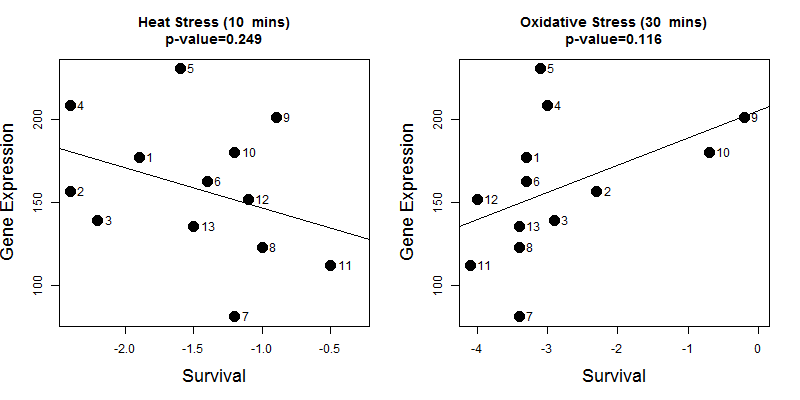

Supplement: S5 File — Expression levels of genes LACR_0001 –LACR_1382 plotted against survival after 10 minutes heat and 30 minutes oxidative stress. Survival is expressed as the difference of log CFU/ml after stress and before stress. Numbers indicate fermentations as presented in Table 1. P-values above the plots indicate significance of correlation (assessed by a linear model). (ZIP) [file pone.0167944.s010.zip › S5_File/LACR_0047_real_dat.png]

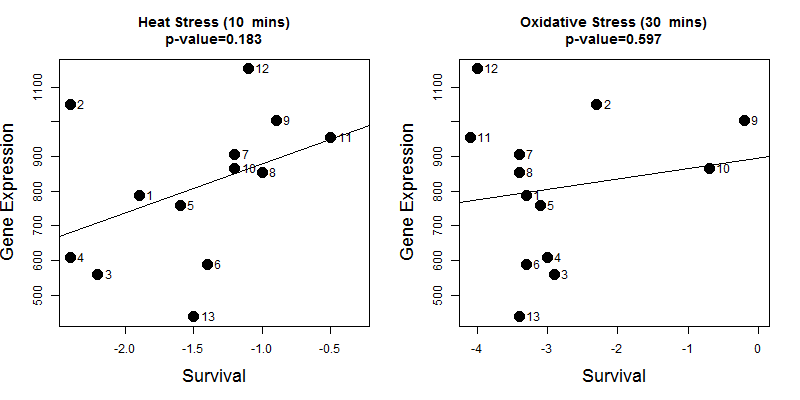

Supplement: S5 File — Expression levels of genes LACR_0001 –LACR_1382 plotted against survival after 10 minutes heat and 30 minutes oxidative stress. Survival is expressed as the difference of log CFU/ml after stress and before stress. Numbers indicate fermentations as presented in Table 1. P-values above the plots indicate significance of correlation (assessed by a linear model). (ZIP) [file pone.0167944.s010.zip › S5_File/LACR_0048_real_dat.png]

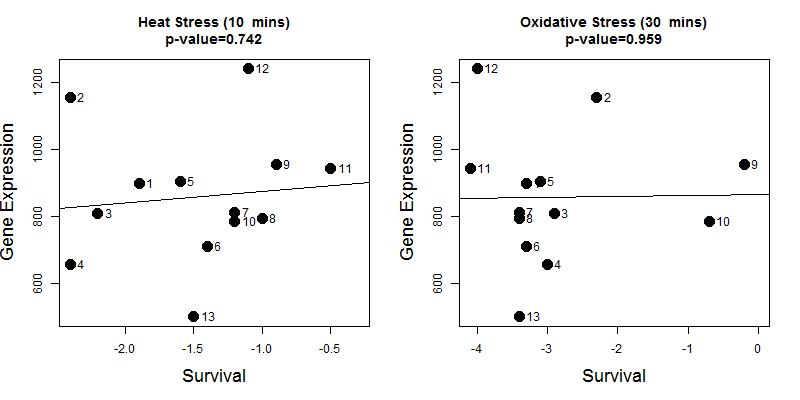

Supplement: S5 File — Expression levels of genes LACR_0001 –LACR_1382 plotted against survival after 10 minutes heat and 30 minutes oxidative stress. Survival is expressed as the difference of log CFU/ml after stress and before stress. Numbers indicate fermentations as presented in Table 1. P-values above the plots indicate significance of correlation (assessed by a linear model). (ZIP) [file pone.0167944.s010.zip › S5_File/LACR_0049_real_dat.png]

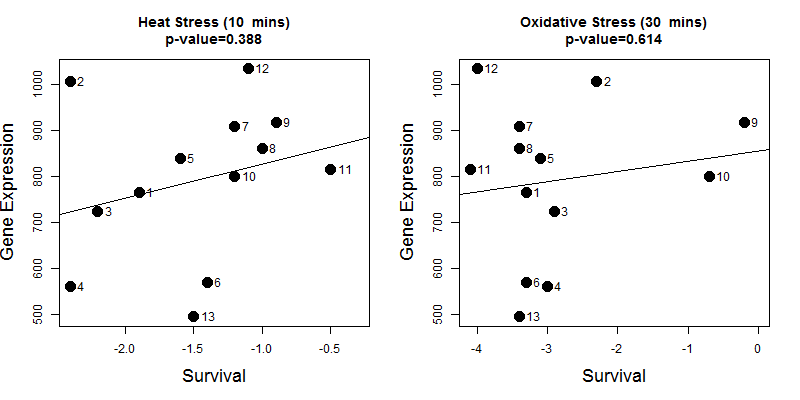

Supplement: S5 File — Expression levels of genes LACR_0001 –LACR_1382 plotted against survival after 10 minutes heat and 30 minutes oxidative stress. Survival is expressed as the difference of log CFU/ml after stress and before stress. Numbers indicate fermentations as presented in Table 1. P-values above the plots indicate significance of correlation (assessed by a linear model). (ZIP) [file pone.0167944.s010.zip › S5_File/LACR_0050_real_dat.png]

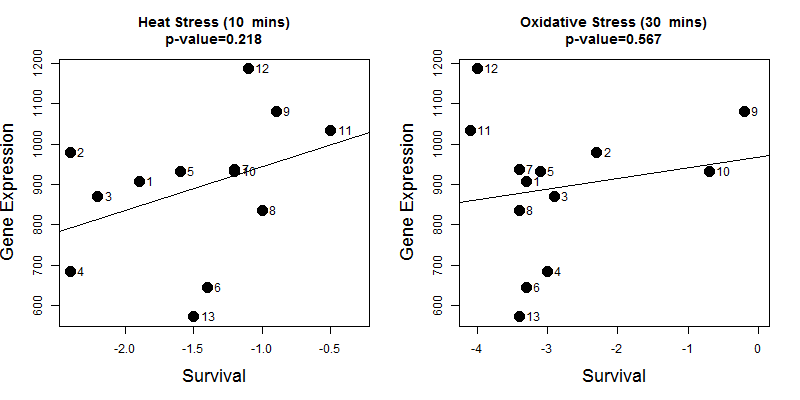

Supplement: S5 File — Expression levels of genes LACR_0001 –LACR_1382 plotted against survival after 10 minutes heat and 30 minutes oxidative stress. Survival is expressed as the difference of log CFU/ml after stress and before stress. Numbers indicate fermentations as presented in Table 1. P-values above the plots indicate significance of correlation (assessed by a linear model). (ZIP) [file pone.0167944.s010.zip › S5_File/LACR_0051_real_dat.png]

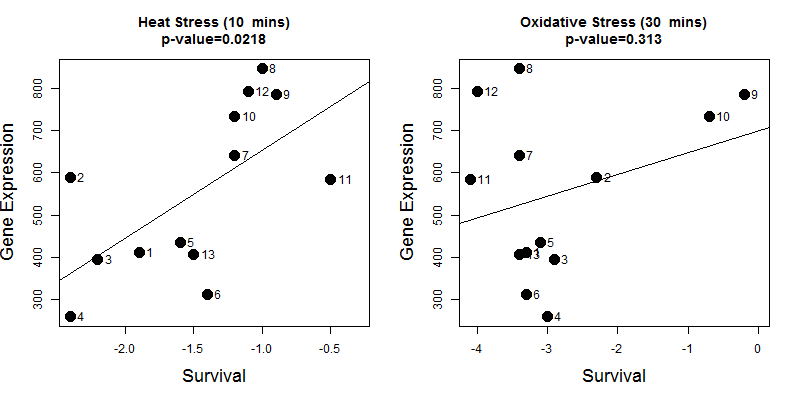

Supplement: S5 File — Expression levels of genes LACR_0001 –LACR_1382 plotted against survival after 10 minutes heat and 30 minutes oxidative stress. Survival is expressed as the difference of log CFU/ml after stress and before stress. Numbers indicate fermentations as presented in Table 1. P-values above the plots indicate significance of correlation (assessed by a linear model). (ZIP) [file pone.0167944.s010.zip › S5_File/LACR_0052_real_dat.png]

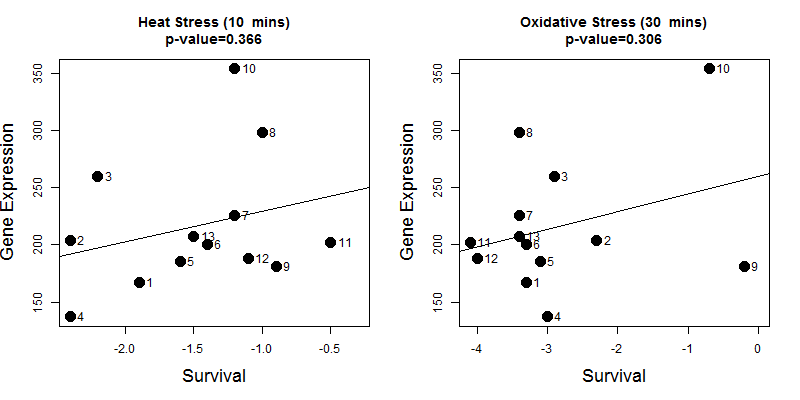

Supplement: S5 File — Expression levels of genes LACR_0001 –LACR_1382 plotted against survival after 10 minutes heat and 30 minutes oxidative stress. Survival is expressed as the difference of log CFU/ml after stress and before stress. Numbers indicate fermentations as presented in Table 1. P-values above the plots indicate significance of correlation (assessed by a linear model). (ZIP) [file pone.0167944.s010.zip › S5_File/LACR_0053_real_dat.png]

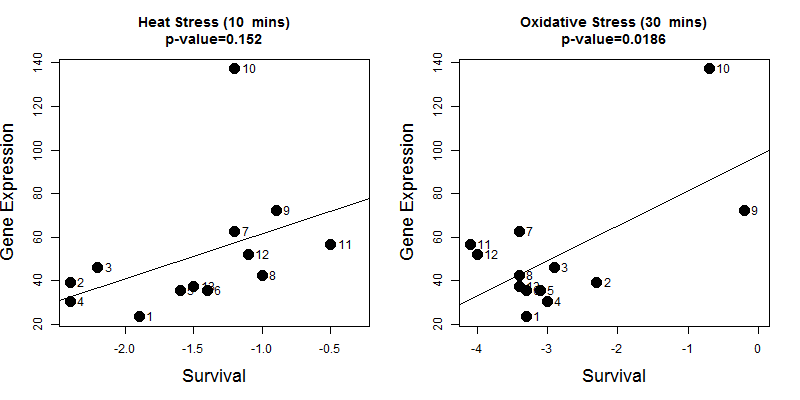

Supplement: S5 File — Expression levels of genes LACR_0001 –LACR_1382 plotted against survival after 10 minutes heat and 30 minutes oxidative stress. Survival is expressed as the difference of log CFU/ml after stress and before stress. Numbers indicate fermentations as presented in Table 1. P-values above the plots indicate significance of correlation (assessed by a linear model). (ZIP) [file pone.0167944.s010.zip › S5_File/LACR_0054_real_dat.png]

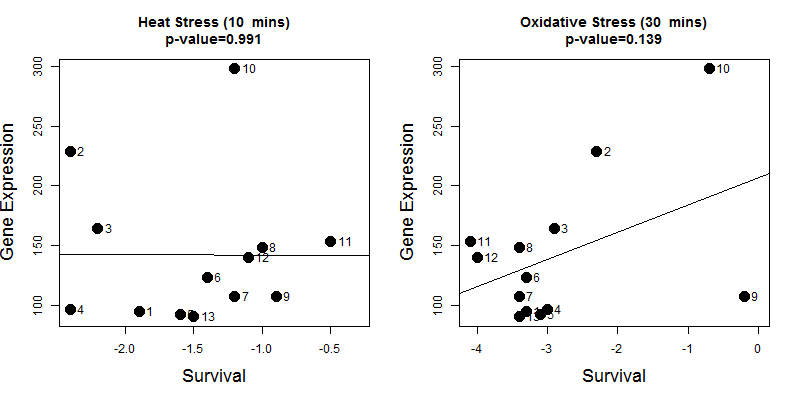

Supplement: S5 File — Expression levels of genes LACR_0001 –LACR_1382 plotted against survival after 10 minutes heat and 30 minutes oxidative stress. Survival is expressed as the difference of log CFU/ml after stress and before stress. Numbers indicate fermentations as presented in Table 1. P-values above the plots indicate significance of correlation (assessed by a linear model). (ZIP) [file pone.0167944.s010.zip › S5_File/LACR_0056_real_dat.png]

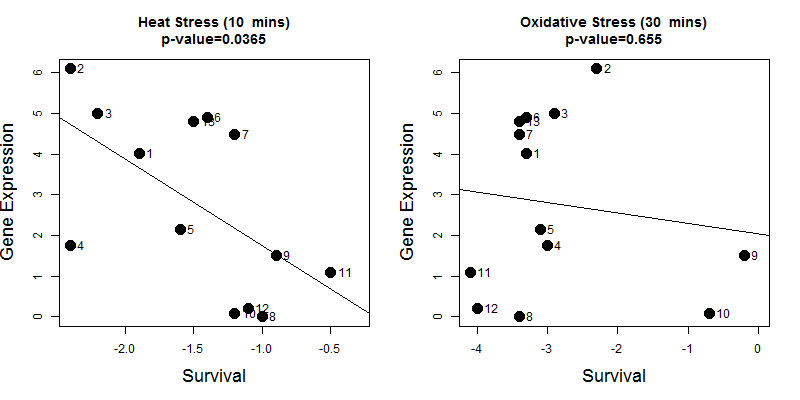

Supplement: S5 File — Expression levels of genes LACR_0001 –LACR_1382 plotted against survival after 10 minutes heat and 30 minutes oxidative stress. Survival is expressed as the difference of log CFU/ml after stress and before stress. Numbers indicate fermentations as presented in Table 1. P-values above the plots indicate significance of correlation (assessed by a linear model). (ZIP) [file pone.0167944.s010.zip › S5_File/LACR_0057_real_dat.png]

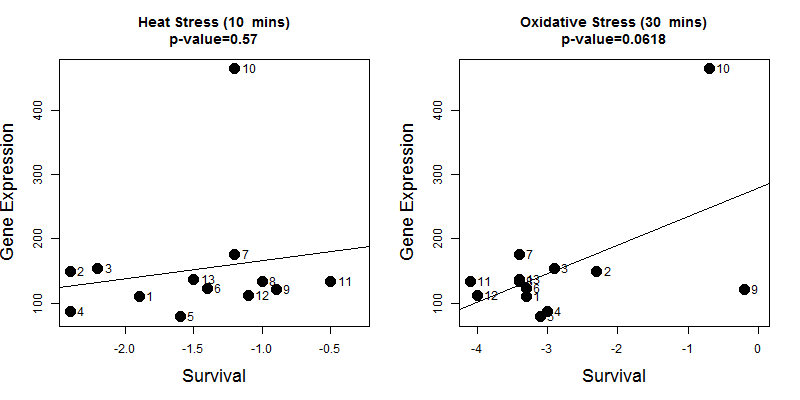

Supplement: S5 File — Expression levels of genes LACR_0001 –LACR_1382 plotted against survival after 10 minutes heat and 30 minutes oxidative stress. Survival is expressed as the difference of log CFU/ml after stress and before stress. Numbers indicate fermentations as presented in Table 1. P-values above the plots indicate significance of correlation (assessed by a linear model). (ZIP) [file pone.0167944.s010.zip › S5_File/LACR_0058_real_dat.png]

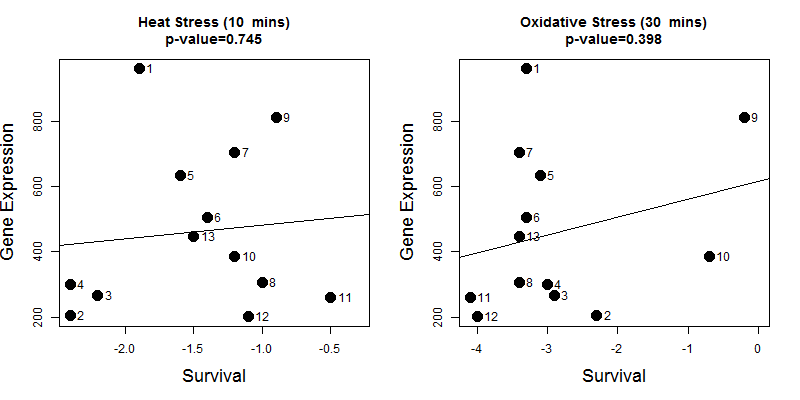

Supplement: S5 File — Expression levels of genes LACR_0001 –LACR_1382 plotted against survival after 10 minutes heat and 30 minutes oxidative stress. Survival is expressed as the difference of log CFU/ml after stress and before stress. Numbers indicate fermentations as presented in Table 1. P-values above the plots indicate significance of correlation (assessed by a linear model). (ZIP) [file pone.0167944.s010.zip › S5_File/LACR_0059_real_dat.png]

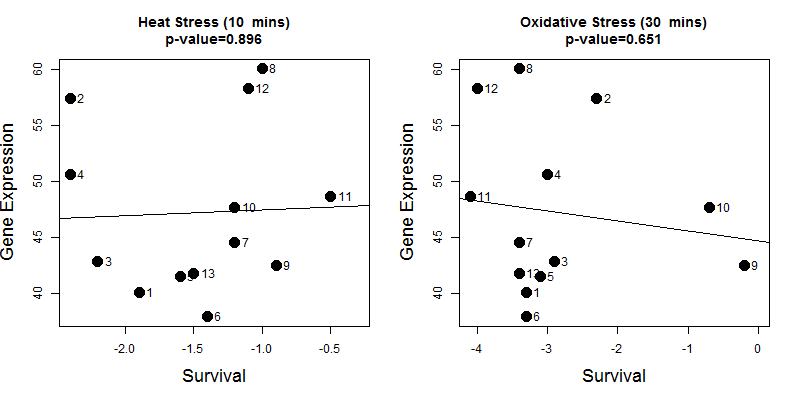

Supplement: S5 File — Expression levels of genes LACR_0001 –LACR_1382 plotted against survival after 10 minutes heat and 30 minutes oxidative stress. Survival is expressed as the difference of log CFU/ml after stress and before stress. Numbers indicate fermentations as presented in Table 1. P-values above the plots indicate significance of correlation (assessed by a linear model). (ZIP) [file pone.0167944.s010.zip › S5_File/LACR_0060_real_dat.png]

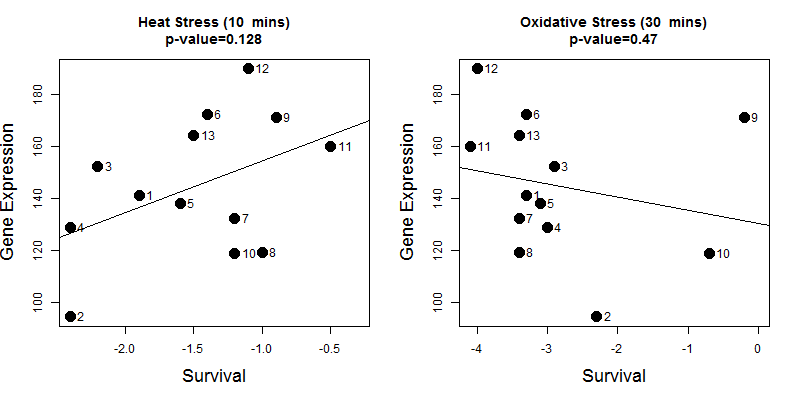

Supplement: S5 File — Expression levels of genes LACR_0001 –LACR_1382 plotted against survival after 10 minutes heat and 30 minutes oxidative stress. Survival is expressed as the difference of log CFU/ml after stress and before stress. Numbers indicate fermentations as presented in Table 1. P-values above the plots indicate significance of correlation (assessed by a linear model). (ZIP) [file pone.0167944.s010.zip › S5_File/LACR_0061_real_dat.png]

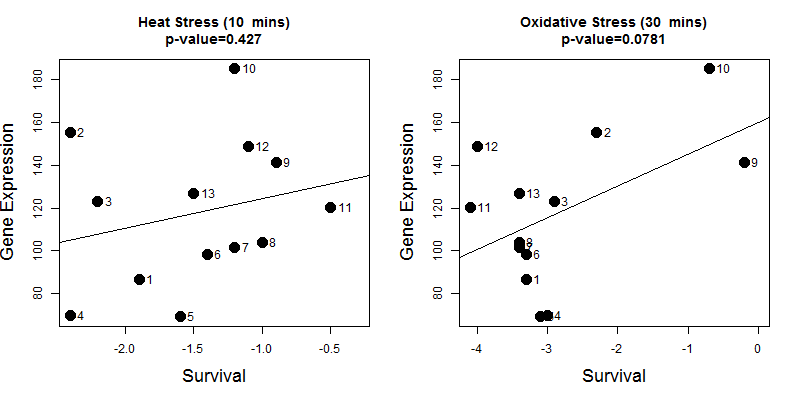

Supplement: S5 File — Expression levels of genes LACR_0001 –LACR_1382 plotted against survival after 10 minutes heat and 30 minutes oxidative stress. Survival is expressed as the difference of log CFU/ml after stress and before stress. Numbers indicate fermentations as presented in Table 1. P-values above the plots indicate significance of correlation (assessed by a linear model). (ZIP) [file pone.0167944.s010.zip › S5_File/LACR_0062_real_dat.png]

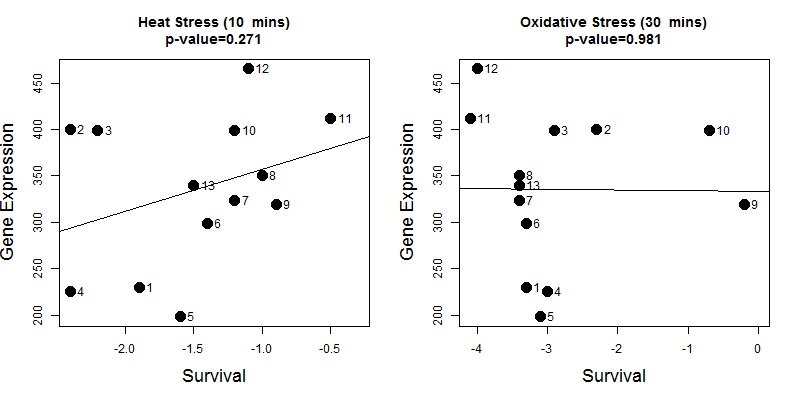

Supplement: S5 File — Expression levels of genes LACR_0001 –LACR_1382 plotted against survival after 10 minutes heat and 30 minutes oxidative stress. Survival is expressed as the difference of log CFU/ml after stress and before stress. Numbers indicate fermentations as presented in Table 1. P-values above the plots indicate significance of correlation (assessed by a linear model). (ZIP) [file pone.0167944.s010.zip › S5_File/LACR_0063_real_dat.png]

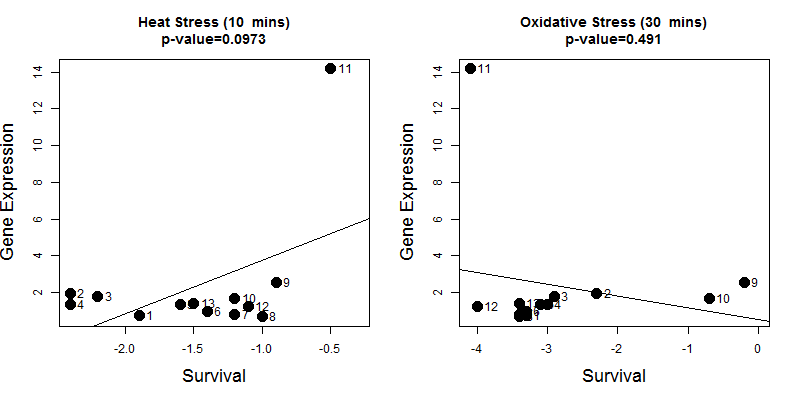

Supplement: S5 File — Expression levels of genes LACR_0001 –LACR_1382 plotted against survival after 10 minutes heat and 30 minutes oxidative stress. Survival is expressed as the difference of log CFU/ml after stress and before stress. Numbers indicate fermentations as presented in Table 1. P-values above the plots indicate significance of correlation (assessed by a linear model). (ZIP) [file pone.0167944.s010.zip › S5_File/LACR_0064_real_dat.png]

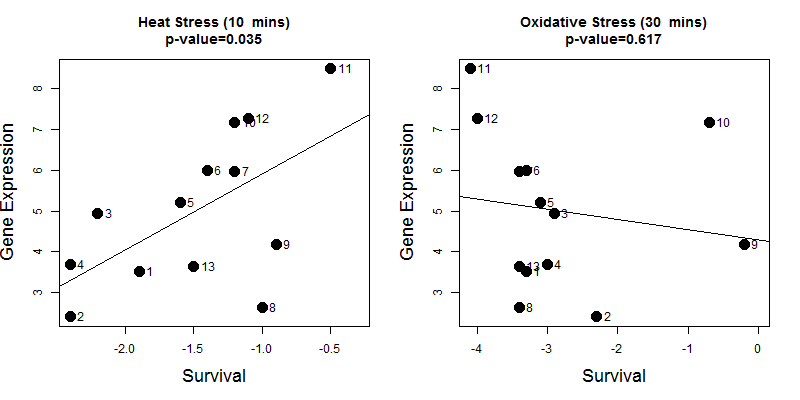

Supplement: S5 File — Expression levels of genes LACR_0001 –LACR_1382 plotted against survival after 10 minutes heat and 30 minutes oxidative stress. Survival is expressed as the difference of log CFU/ml after stress and before stress. Numbers indicate fermentations as presented in Table 1. P-values above the plots indicate significance of correlation (assessed by a linear model). (ZIP) [file pone.0167944.s010.zip › S5_File/LACR_0065_real_dat.png]

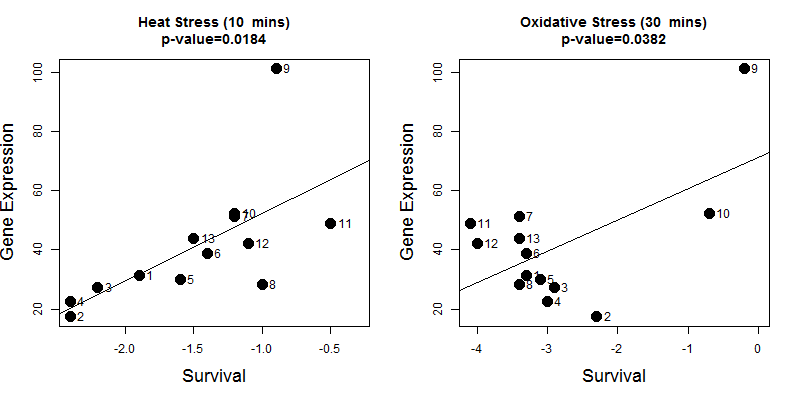

Supplement: S5 File — Expression levels of genes LACR_0001 –LACR_1382 plotted against survival after 10 minutes heat and 30 minutes oxidative stress. Survival is expressed as the difference of log CFU/ml after stress and before stress. Numbers indicate fermentations as presented in Table 1. P-values above the plots indicate significance of correlation (assessed by a linear model). (ZIP) [file pone.0167944.s010.zip › S5_File/LACR_0066_real_dat.png]

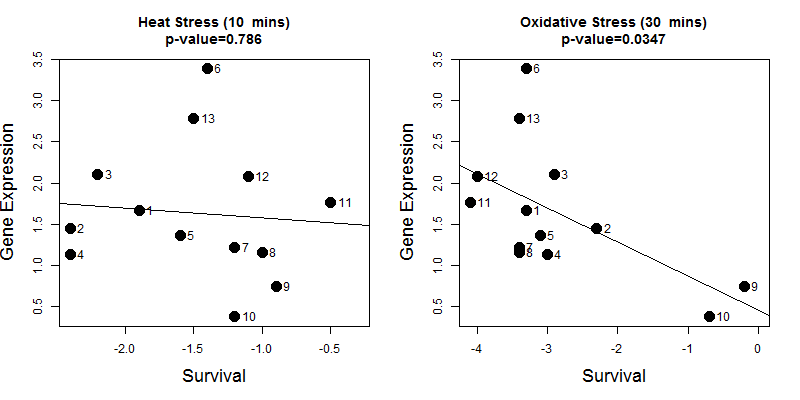

Supplement: S5 File — Expression levels of genes LACR_0001 –LACR_1382 plotted against survival after 10 minutes heat and 30 minutes oxidative stress. Survival is expressed as the difference of log CFU/ml after stress and before stress. Numbers indicate fermentations as presented in Table 1. P-values above the plots indicate significance of correlation (assessed by a linear model). (ZIP) [file pone.0167944.s010.zip › S5_File/LACR_0067_real_dat.png]

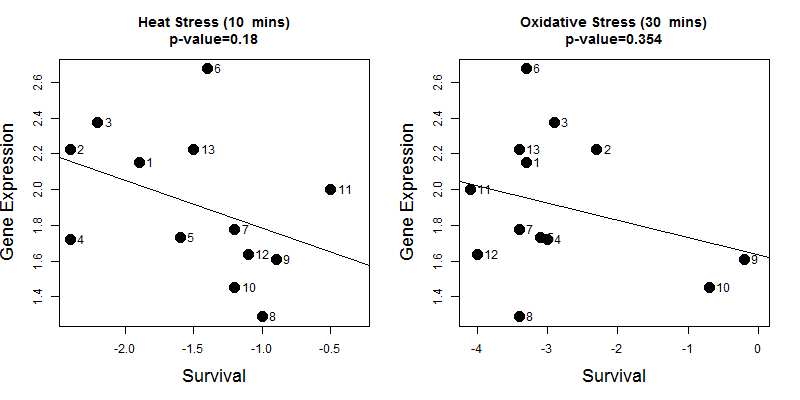

Supplement: S5 File — Expression levels of genes LACR_0001 –LACR_1382 plotted against survival after 10 minutes heat and 30 minutes oxidative stress. Survival is expressed as the difference of log CFU/ml after stress and before stress. Numbers indicate fermentations as presented in Table 1. P-values above the plots indicate significance of correlation (assessed by a linear model). (ZIP) [file pone.0167944.s010.zip › S5_File/LACR_0068_real_dat.png]

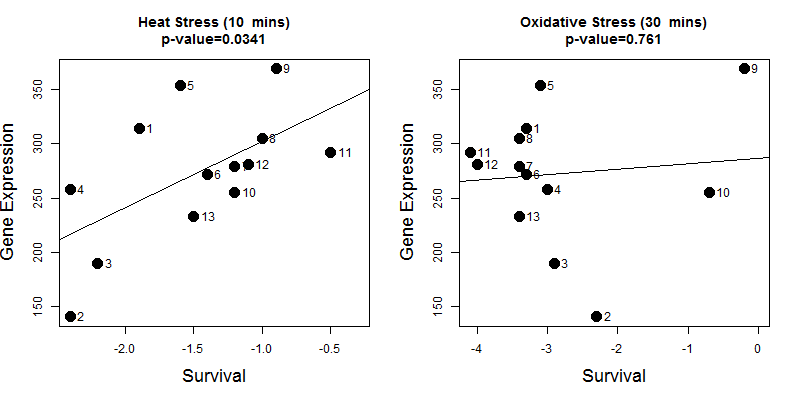

Supplement: S5 File — Expression levels of genes LACR_0001 –LACR_1382 plotted against survival after 10 minutes heat and 30 minutes oxidative stress. Survival is expressed as the difference of log CFU/ml after stress and before stress. Numbers indicate fermentations as presented in Table 1. P-values above the plots indicate significance of correlation (assessed by a linear model). (ZIP) [file pone.0167944.s010.zip › S5_File/LACR_0069_real_dat.png]

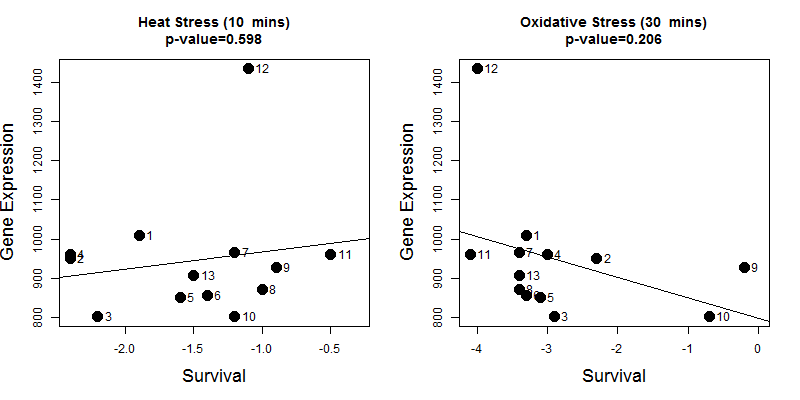

Supplement: S5 File — Expression levels of genes LACR_0001 –LACR_1382 plotted against survival after 10 minutes heat and 30 minutes oxidative stress. Survival is expressed as the difference of log CFU/ml after stress and before stress. Numbers indicate fermentations as presented in Table 1. P-values above the plots indicate significance of correlation (assessed by a linear model). (ZIP) [file pone.0167944.s010.zip › S5_File/LACR_0070_real_dat.png]

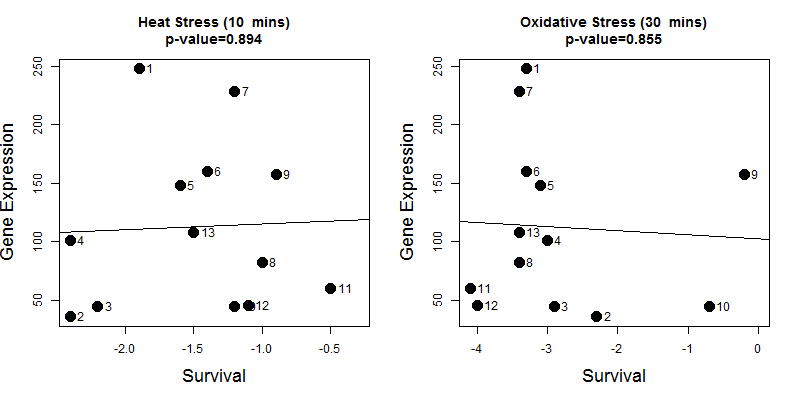

Supplement: S5 File — Expression levels of genes LACR_0001 –LACR_1382 plotted against survival after 10 minutes heat and 30 minutes oxidative stress. Survival is expressed as the difference of log CFU/ml after stress and before stress. Numbers indicate fermentations as presented in Table 1. P-values above the plots indicate significance of correlation (assessed by a linear model). (ZIP) [file pone.0167944.s010.zip › S5_File/LACR_0071_real_dat.png]

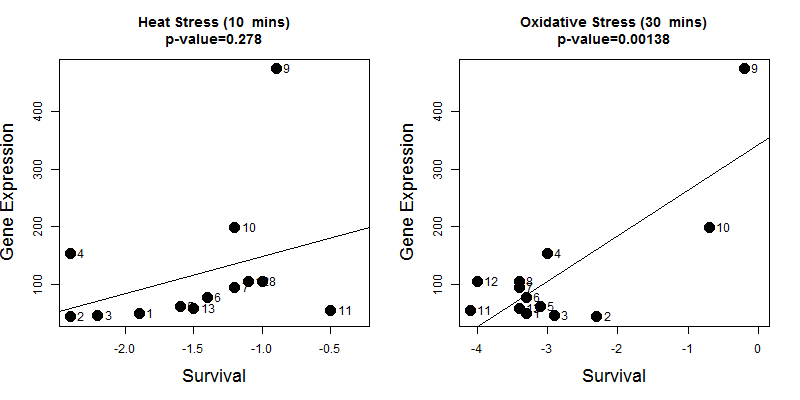

Supplement: S5 File — Expression levels of genes LACR_0001 –LACR_1382 plotted against survival after 10 minutes heat and 30 minutes oxidative stress. Survival is expressed as the difference of log CFU/ml after stress and before stress. Numbers indicate fermentations as presented in Table 1. P-values above the plots indicate significance of correlation (assessed by a linear model). (ZIP) [file pone.0167944.s010.zip › S5_File/LACR_0072_real_dat.png]

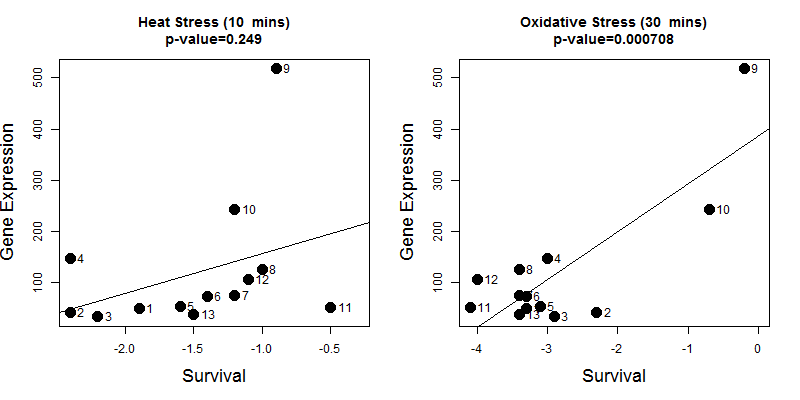

Supplement: S5 File — Expression levels of genes LACR_0001 –LACR_1382 plotted against survival after 10 minutes heat and 30 minutes oxidative stress. Survival is expressed as the difference of log CFU/ml after stress and before stress. Numbers indicate fermentations as presented in Table 1. P-values above the plots indicate significance of correlation (assessed by a linear model). (ZIP) [file pone.0167944.s010.zip › S5_File/LACR_0073_real_dat.png]

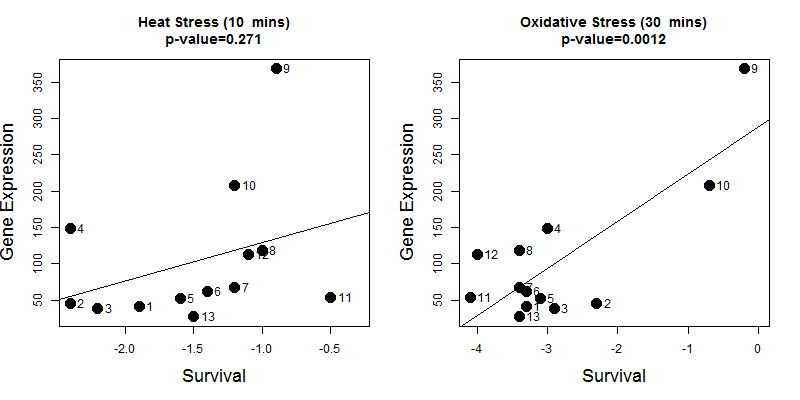

Supplement: S5 File — Expression levels of genes LACR_0001 –LACR_1382 plotted against survival after 10 minutes heat and 30 minutes oxidative stress. Survival is expressed as the difference of log CFU/ml after stress and before stress. Numbers indicate fermentations as presented in Table 1. P-values above the plots indicate significance of correlation (assessed by a linear model). (ZIP) [file pone.0167944.s010.zip › S5_File/LACR_0074_real_dat.png]

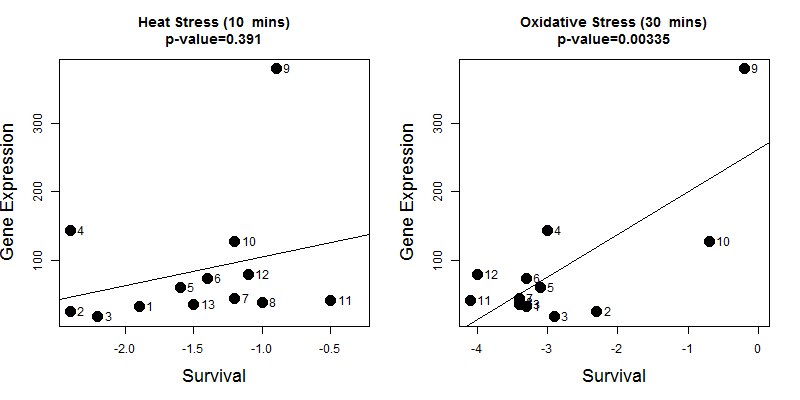

Supplement: S5 File — Expression levels of genes LACR_0001 –LACR_1382 plotted against survival after 10 minutes heat and 30 minutes oxidative stress. Survival is expressed as the difference of log CFU/ml after stress and before stress. Numbers indicate fermentations as presented in Table 1. P-values above the plots indicate significance of correlation (assessed by a linear model). (ZIP) [file pone.0167944.s010.zip › S5_File/LACR_0075_real_dat.png]

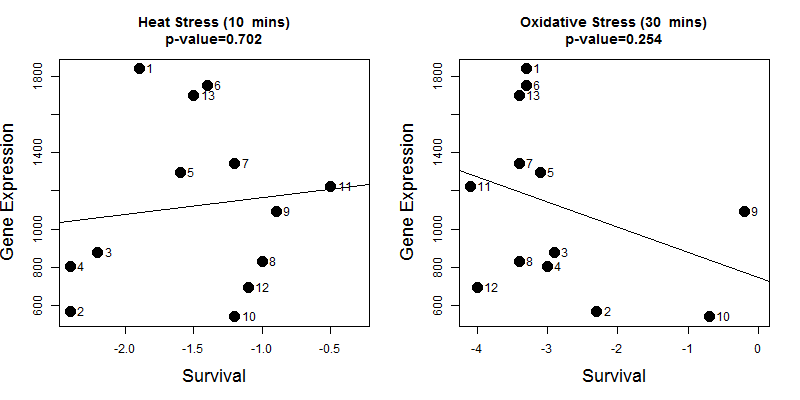

Supplement: S5 File — Expression levels of genes LACR_0001 –LACR_1382 plotted against survival after 10 minutes heat and 30 minutes oxidative stress. Survival is expressed as the difference of log CFU/ml after stress and before stress. Numbers indicate fermentations as presented in Table 1. P-values above the plots indicate significance of correlation (assessed by a linear model). (ZIP) [file pone.0167944.s010.zip › S5_File/LACR_0076_real_dat.png]

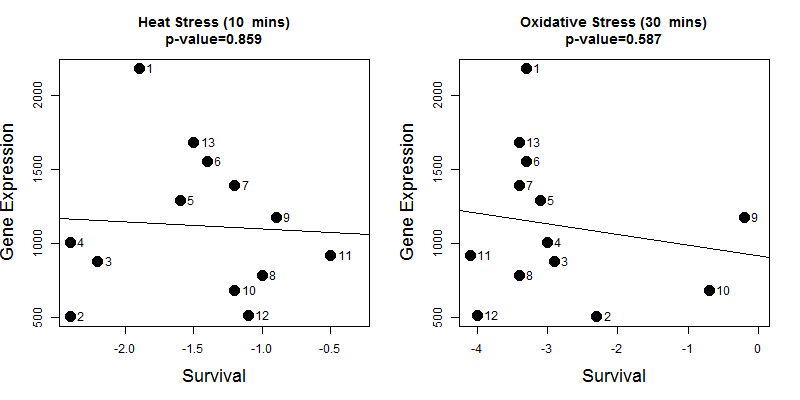

Supplement: S5 File — Expression levels of genes LACR_0001 –LACR_1382 plotted against survival after 10 minutes heat and 30 minutes oxidative stress. Survival is expressed as the difference of log CFU/ml after stress and before stress. Numbers indicate fermentations as presented in Table 1. P-values above the plots indicate significance of correlation (assessed by a linear model). (ZIP) [file pone.0167944.s010.zip › S5_File/LACR_0077_real_dat.png]

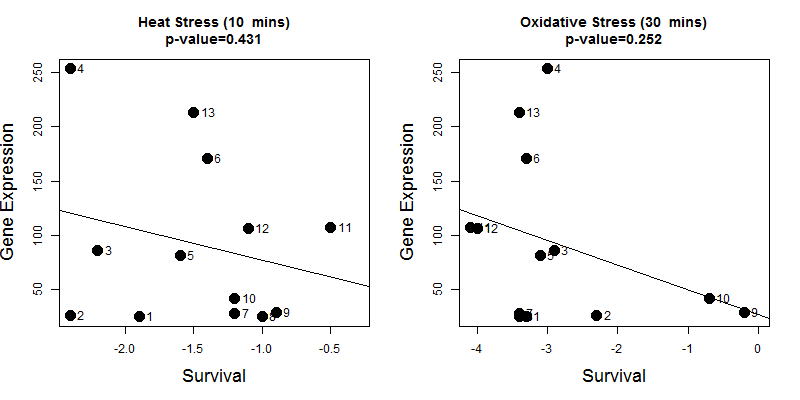

Supplement: S5 File — Expression levels of genes LACR_0001 –LACR_1382 plotted against survival after 10 minutes heat and 30 minutes oxidative stress. Survival is expressed as the difference of log CFU/ml after stress and before stress. Numbers indicate fermentations as presented in Table 1. P-values above the plots indicate significance of correlation (assessed by a linear model). (ZIP) [file pone.0167944.s010.zip › S5_File/LACR_0078_real_dat.png]

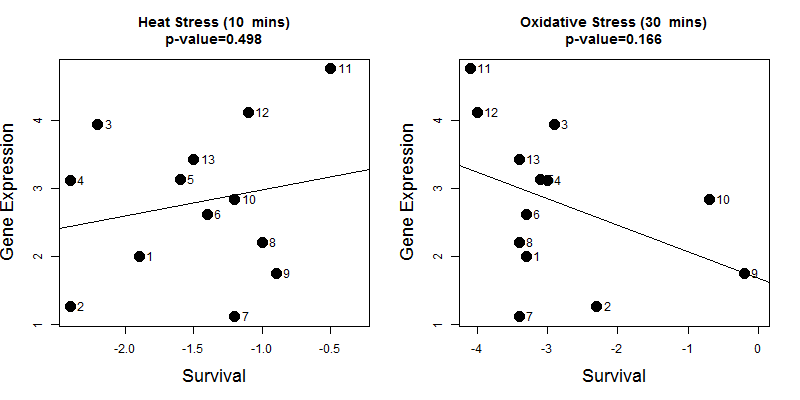

Supplement: S5 File — Expression levels of genes LACR_0001 –LACR_1382 plotted against survival after 10 minutes heat and 30 minutes oxidative stress. Survival is expressed as the difference of log CFU/ml after stress and before stress. Numbers indicate fermentations as presented in Table 1. P-values above the plots indicate significance of correlation (assessed by a linear model). (ZIP) [file pone.0167944.s010.zip › S5_File/LACR_0079_real_dat.png]

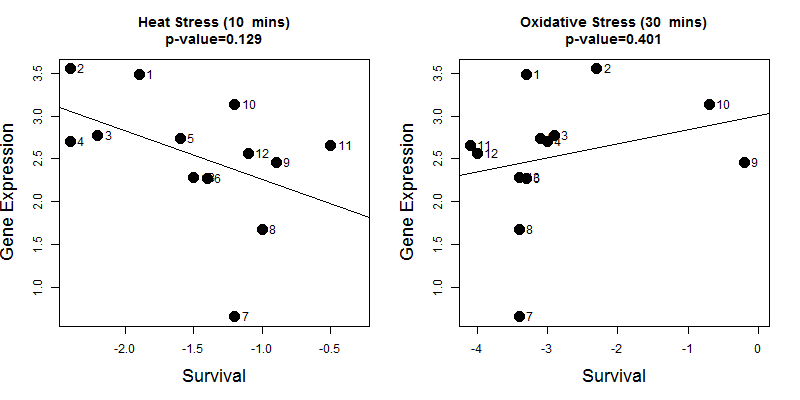

Supplement: S5 File — Expression levels of genes LACR_0001 –LACR_1382 plotted against survival after 10 minutes heat and 30 minutes oxidative stress. Survival is expressed as the difference of log CFU/ml after stress and before stress. Numbers indicate fermentations as presented in Table 1. P-values above the plots indicate significance of correlation (assessed by a linear model). (ZIP) [file pone.0167944.s010.zip › S5_File/LACR_0080_real_dat.png]

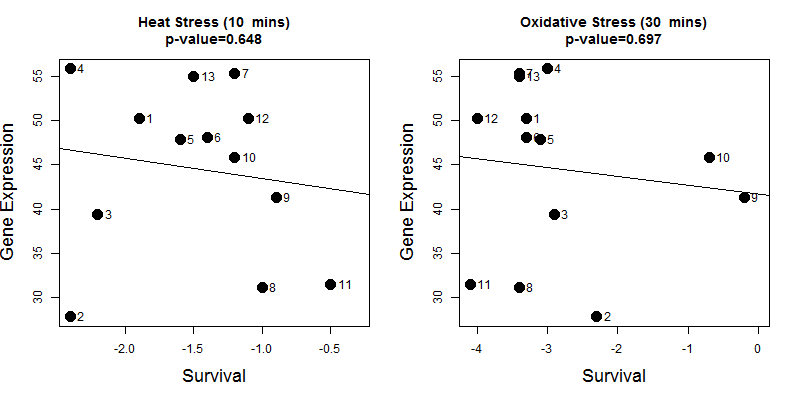

Supplement: S5 File — Expression levels of genes LACR_0001 –LACR_1382 plotted against survival after 10 minutes heat and 30 minutes oxidative stress. Survival is expressed as the difference of log CFU/ml after stress and before stress. Numbers indicate fermentations as presented in Table 1. P-values above the plots indicate significance of correlation (assessed by a linear model). (ZIP) [file pone.0167944.s010.zip › S5_File/LACR_0081_real_dat.png]

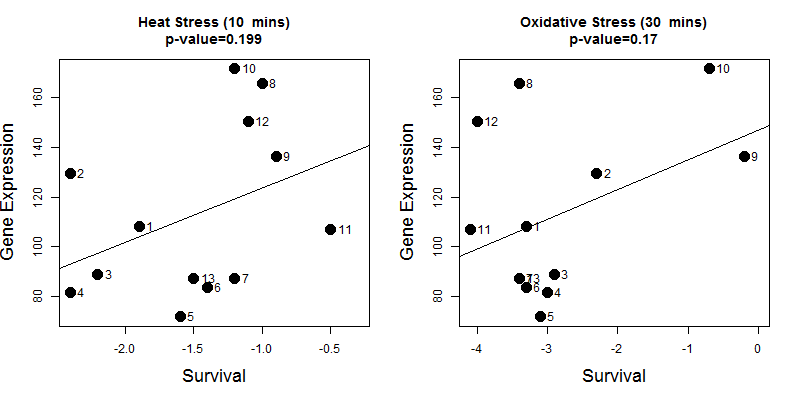

Supplement: S5 File — Expression levels of genes LACR_0001 –LACR_1382 plotted against survival after 10 minutes heat and 30 minutes oxidative stress. Survival is expressed as the difference of log CFU/ml after stress and before stress. Numbers indicate fermentations as presented in Table 1. P-values above the plots indicate significance of correlation (assessed by a linear model). (ZIP) [file pone.0167944.s010.zip › S5_File/LACR_0082_real_dat.png]

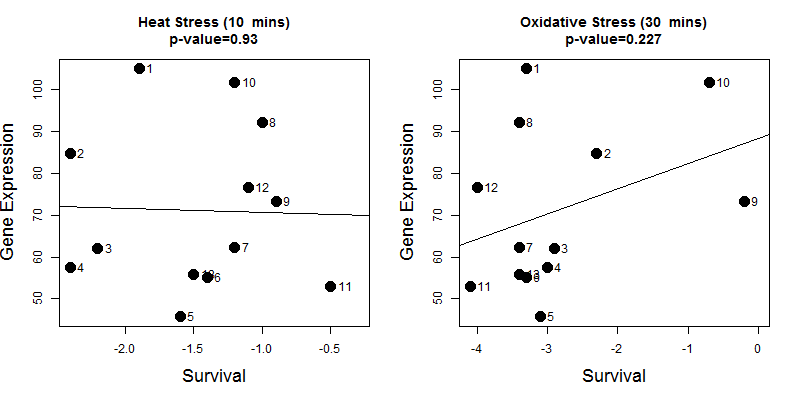

Supplement: S5 File — Expression levels of genes LACR_0001 –LACR_1382 plotted against survival after 10 minutes heat and 30 minutes oxidative stress. Survival is expressed as the difference of log CFU/ml after stress and before stress. Numbers indicate fermentations as presented in Table 1. P-values above the plots indicate significance of correlation (assessed by a linear model). (ZIP) [file pone.0167944.s010.zip › S5_File/LACR_0083_real_dat.png]

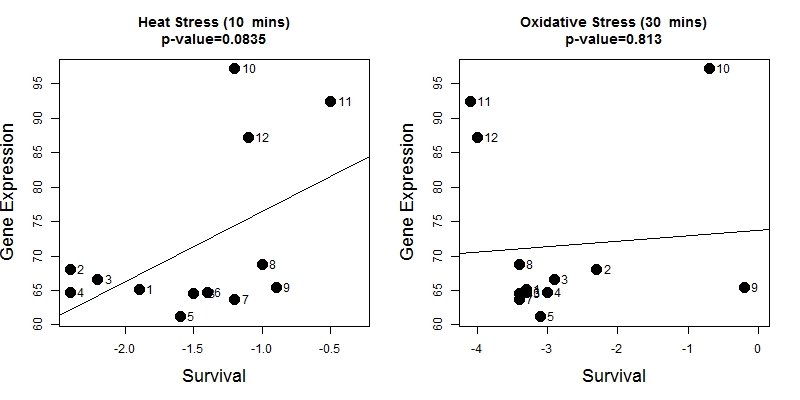

Supplement: S5 File — Expression levels of genes LACR_0001 –LACR_1382 plotted against survival after 10 minutes heat and 30 minutes oxidative stress. Survival is expressed as the difference of log CFU/ml after stress and before stress. Numbers indicate fermentations as presented in Table 1. P-values above the plots indicate significance of correlation (assessed by a linear model). (ZIP) [file pone.0167944.s010.zip › S5_File/LACR_0084_real_dat.png]

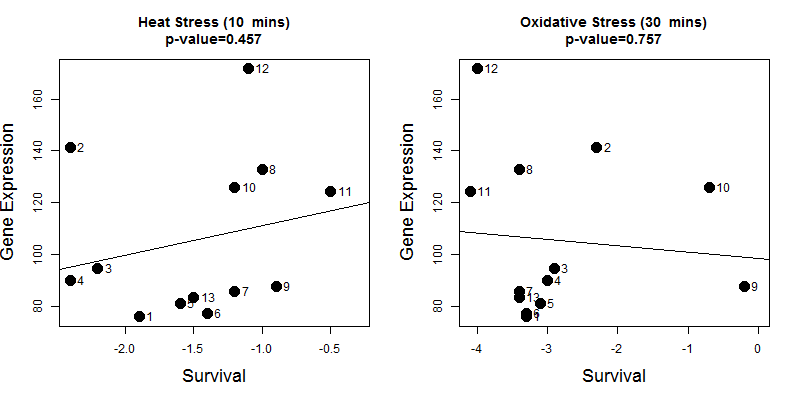

Supplement: S5 File — Expression levels of genes LACR_0001 –LACR_1382 plotted against survival after 10 minutes heat and 30 minutes oxidative stress. Survival is expressed as the difference of log CFU/ml after stress and before stress. Numbers indicate fermentations as presented in Table 1. P-values above the plots indicate significance of correlation (assessed by a linear model). (ZIP) [file pone.0167944.s010.zip › S5_File/LACR_0085_real_dat.png]

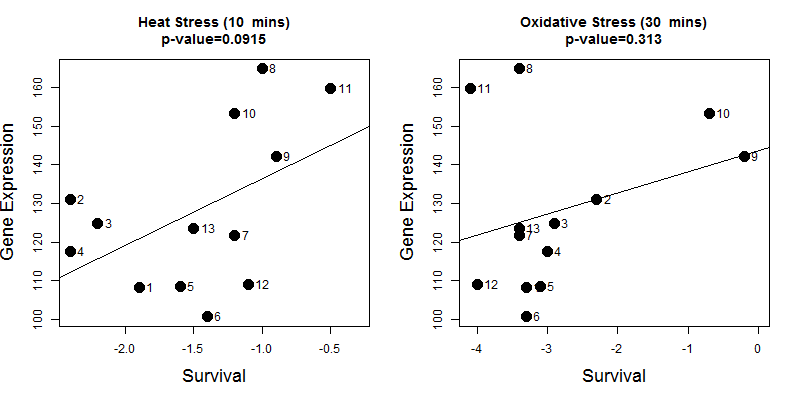

Supplement: S5 File — Expression levels of genes LACR_0001 –LACR_1382 plotted against survival after 10 minutes heat and 30 minutes oxidative stress. Survival is expressed as the difference of log CFU/ml after stress and before stress. Numbers indicate fermentations as presented in Table 1. P-values above the plots indicate significance of correlation (assessed by a linear model). (ZIP) [file pone.0167944.s010.zip › S5_File/LACR_0086_real_dat.png]

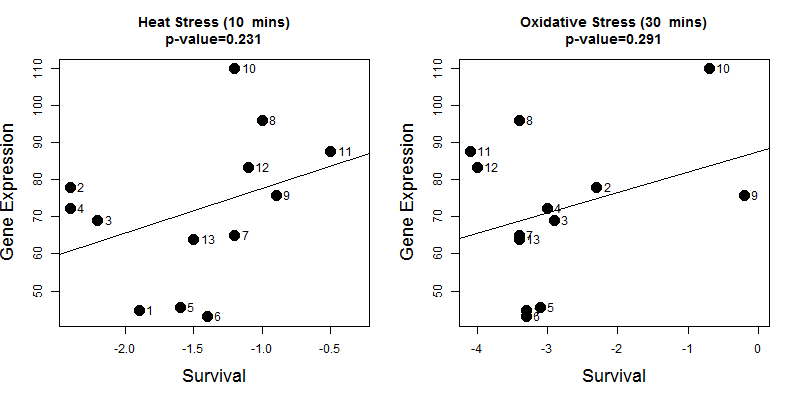

Supplement: S5 File — Expression levels of genes LACR_0001 –LACR_1382 plotted against survival after 10 minutes heat and 30 minutes oxidative stress. Survival is expressed as the difference of log CFU/ml after stress and before stress. Numbers indicate fermentations as presented in Table 1. P-values above the plots indicate significance of correlation (assessed by a linear model). (ZIP) [file pone.0167944.s010.zip › S5_File/LACR_0087_real_dat.png]

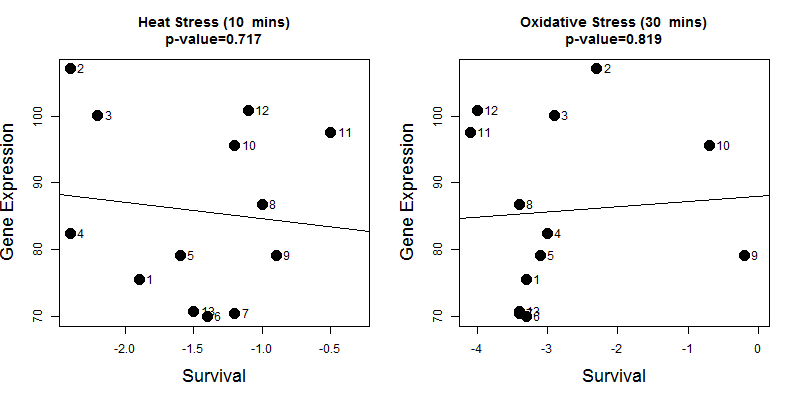

Supplement: S5 File — Expression levels of genes LACR_0001 –LACR_1382 plotted against survival after 10 minutes heat and 30 minutes oxidative stress. Survival is expressed as the difference of log CFU/ml after stress and before stress. Numbers indicate fermentations as presented in Table 1. P-values above the plots indicate significance of correlation (assessed by a linear model). (ZIP) [file pone.0167944.s010.zip › S5_File/LACR_0088_real_dat.png]

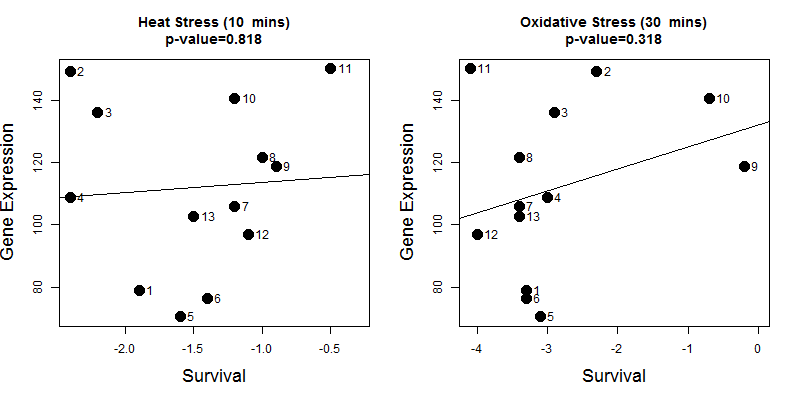

Supplement: S5 File — Expression levels of genes LACR_0001 –LACR_1382 plotted against survival after 10 minutes heat and 30 minutes oxidative stress. Survival is expressed as the difference of log CFU/ml after stress and before stress. Numbers indicate fermentations as presented in Table 1. P-values above the plots indicate significance of correlation (assessed by a linear model). (ZIP) [file pone.0167944.s010.zip › S5_File/LACR_0089_real_dat.png]

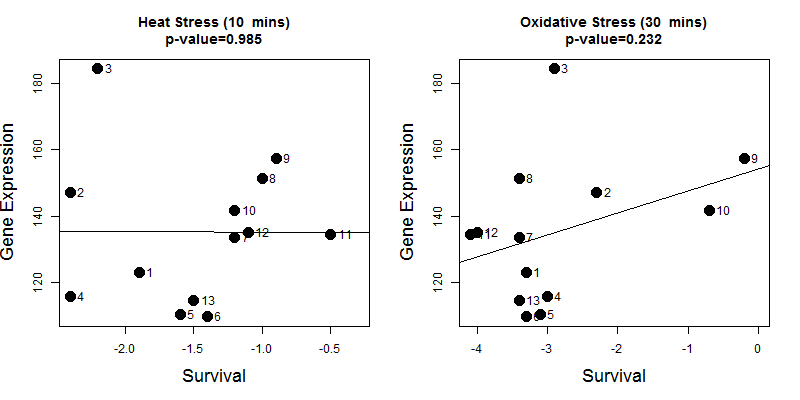

Supplement: S5 File — Expression levels of genes LACR_0001 –LACR_1382 plotted against survival after 10 minutes heat and 30 minutes oxidative stress. Survival is expressed as the difference of log CFU/ml after stress and before stress. Numbers indicate fermentations as presented in Table 1. P-values above the plots indicate significance of correlation (assessed by a linear model). (ZIP) [file pone.0167944.s010.zip › S5_File/LACR_0090_real_dat.png]

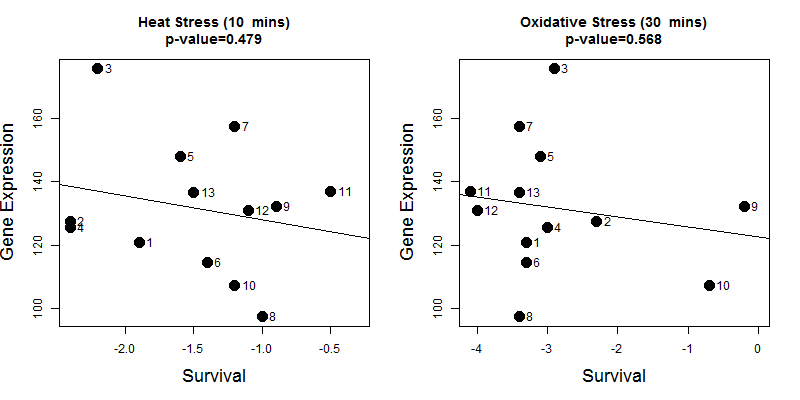

Supplement: S5 File — Expression levels of genes LACR_0001 –LACR_1382 plotted against survival after 10 minutes heat and 30 minutes oxidative stress. Survival is expressed as the difference of log CFU/ml after stress and before stress. Numbers indicate fermentations as presented in Table 1. P-values above the plots indicate significance of correlation (assessed by a linear model). (ZIP) [file pone.0167944.s010.zip › S5_File/LACR_0091_real_dat.png]

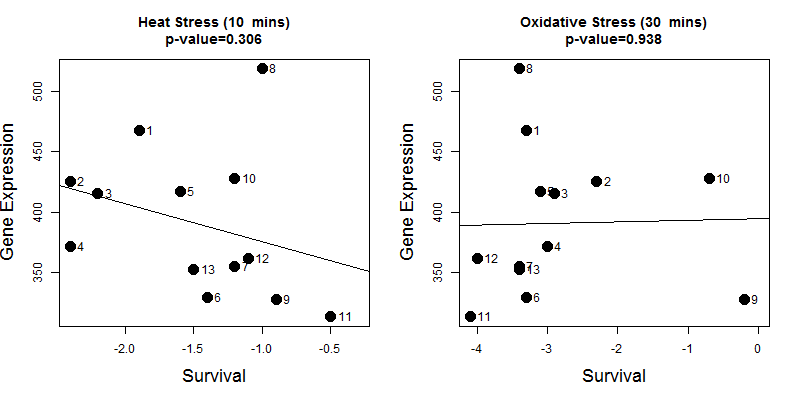

Supplement: S5 File — Expression levels of genes LACR_0001 –LACR_1382 plotted against survival after 10 minutes heat and 30 minutes oxidative stress. Survival is expressed as the difference of log CFU/ml after stress and before stress. Numbers indicate fermentations as presented in Table 1. P-values above the plots indicate significance of correlation (assessed by a linear model). (ZIP) [file pone.0167944.s010.zip › S5_File/LACR_0092_real_dat.png]

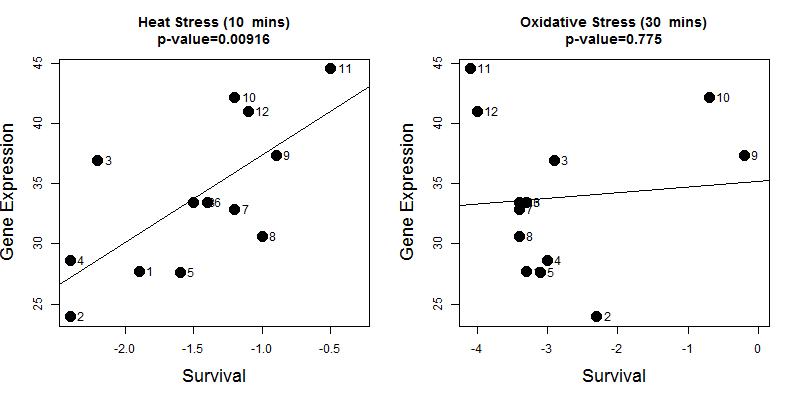

Supplement: S5 File — Expression levels of genes LACR_0001 –LACR_1382 plotted against survival after 10 minutes heat and 30 minutes oxidative stress. Survival is expressed as the difference of log CFU/ml after stress and before stress. Numbers indicate fermentations as presented in Table 1. P-values above the plots indicate significance of correlation (assessed by a linear model). (ZIP) [file pone.0167944.s010.zip › S5_File/LACR_0094_real_dat.png]

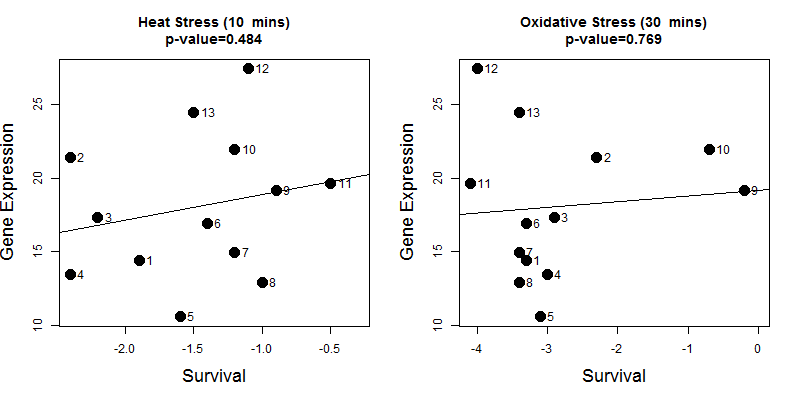

Supplement: S5 File — Expression levels of genes LACR_0001 –LACR_1382 plotted against survival after 10 minutes heat and 30 minutes oxidative stress. Survival is expressed as the difference of log CFU/ml after stress and before stress. Numbers indicate fermentations as presented in Table 1. P-values above the plots indicate significance of correlation (assessed by a linear model). (ZIP) [file pone.0167944.s010.zip › S5_File/LACR_0095_real_dat.png]

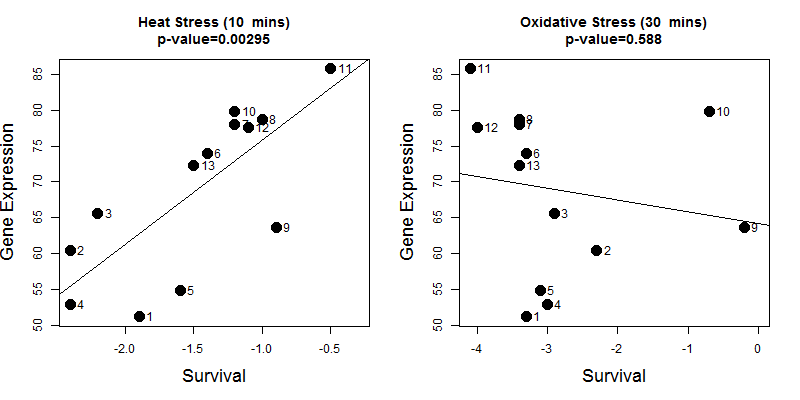

Supplement: S5 File — Expression levels of genes LACR_0001 –LACR_1382 plotted against survival after 10 minutes heat and 30 minutes oxidative stress. Survival is expressed as the difference of log CFU/ml after stress and before stress. Numbers indicate fermentations as presented in Table 1. P-values above the plots indicate significance of correlation (assessed by a linear model). (ZIP) [file pone.0167944.s010.zip › S5_File/LACR_0096_real_dat.png]

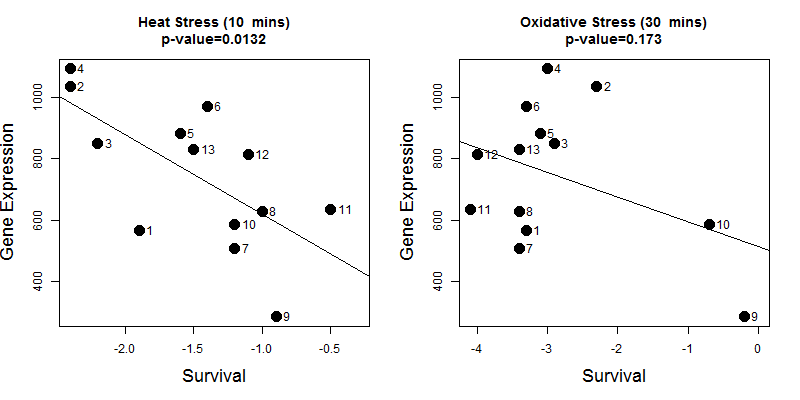

Supplement: S5 File — Expression levels of genes LACR_0001 –LACR_1382 plotted against survival after 10 minutes heat and 30 minutes oxidative stress. Survival is expressed as the difference of log CFU/ml after stress and before stress. Numbers indicate fermentations as presented in Table 1. P-values above the plots indicate significance of correlation (assessed by a linear model). (ZIP) [file pone.0167944.s010.zip › S5_File/LACR_0097_real_dat.png]

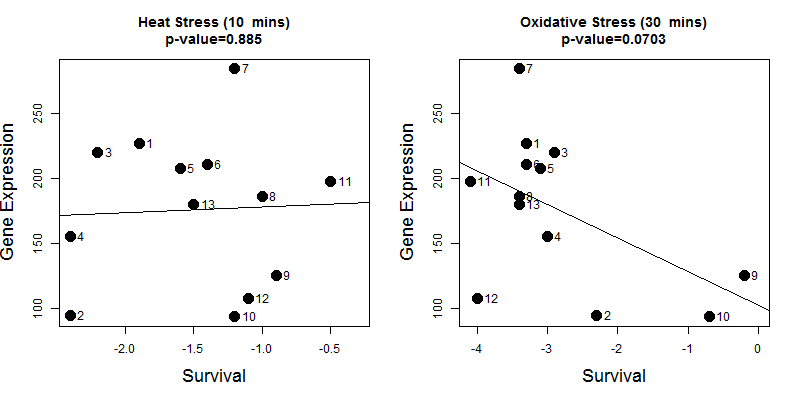

Supplement: S5 File — Expression levels of genes LACR_0001 –LACR_1382 plotted against survival after 10 minutes heat and 30 minutes oxidative stress. Survival is expressed as the difference of log CFU/ml after stress and before stress. Numbers indicate fermentations as presented in Table 1. P-values above the plots indicate significance of correlation (assessed by a linear model). (ZIP) [file pone.0167944.s010.zip › S5_File/LACR_0098_real_dat.png]

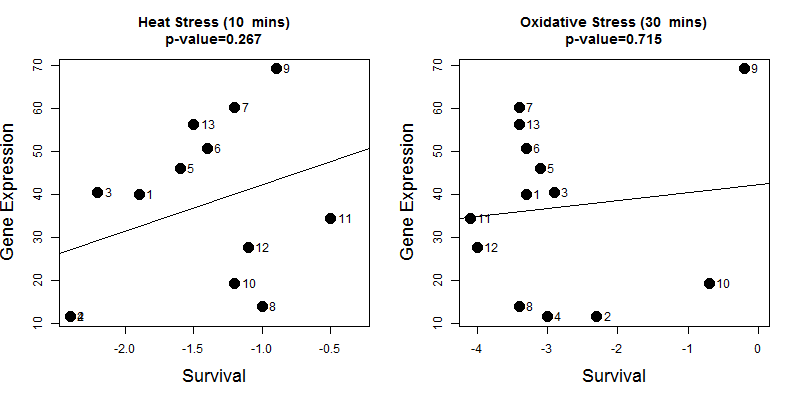

Supplement: S5 File — Expression levels of genes LACR_0001 –LACR_1382 plotted against survival after 10 minutes heat and 30 minutes oxidative stress. Survival is expressed as the difference of log CFU/ml after stress and before stress. Numbers indicate fermentations as presented in Table 1. P-values above the plots indicate significance of correlation (assessed by a linear model). (ZIP) [file pone.0167944.s010.zip › S5_File/LACR_0099_real_dat.png]

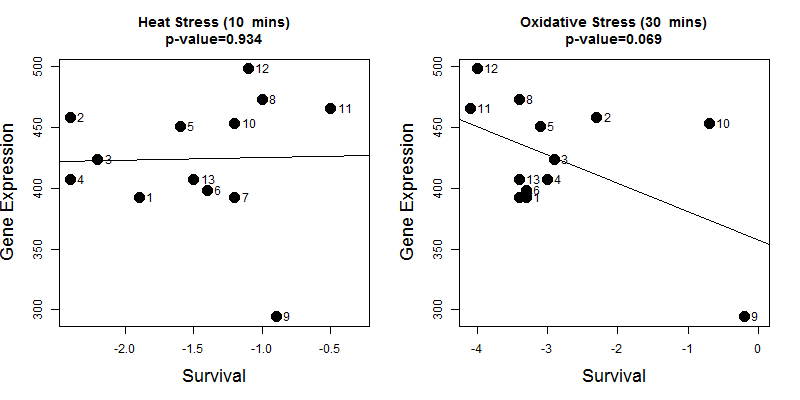

Supplement: S5 File — Expression levels of genes LACR_0001 –LACR_1382 plotted against survival after 10 minutes heat and 30 minutes oxidative stress. Survival is expressed as the difference of log CFU/ml after stress and before stress. Numbers indicate fermentations as presented in Table 1. P-values above the plots indicate significance of correlation (assessed by a linear model). (ZIP) [file pone.0167944.s010.zip › S5_File/LACR_0101_real_dat.png]

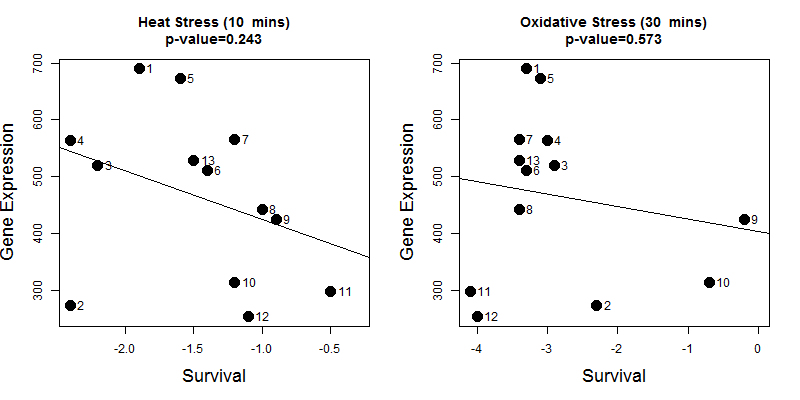

Supplement: S5 File — Expression levels of genes LACR_0001 –LACR_1382 plotted against survival after 10 minutes heat and 30 minutes oxidative stress. Survival is expressed as the difference of log CFU/ml after stress and before stress. Numbers indicate fermentations as presented in Table 1. P-values above the plots indicate significance of correlation (assessed by a linear model). (ZIP) [file pone.0167944.s010.zip › S5_File/LACR_0102_real_dat.png]

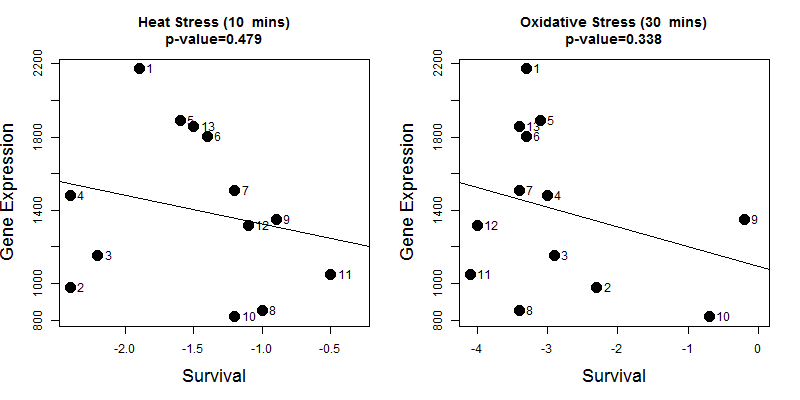

Supplement: S5 File — Expression levels of genes LACR_0001 –LACR_1382 plotted against survival after 10 minutes heat and 30 minutes oxidative stress. Survival is expressed as the difference of log CFU/ml after stress and before stress. Numbers indicate fermentations as presented in Table 1. P-values above the plots indicate significance of correlation (assessed by a linear model). (ZIP) [file pone.0167944.s010.zip › S5_File/LACR_0103_real_dat.png]

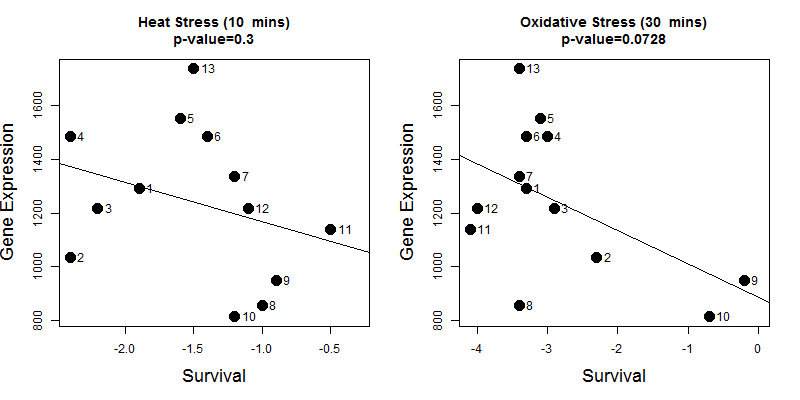

Supplement: S5 File — Expression levels of genes LACR_0001 –LACR_1382 plotted against survival after 10 minutes heat and 30 minutes oxidative stress. Survival is expressed as the difference of log CFU/ml after stress and before stress. Numbers indicate fermentations as presented in Table 1. P-values above the plots indicate significance of correlation (assessed by a linear model). (ZIP) [file pone.0167944.s010.zip › S5_File/LACR_0104_real_dat.png]

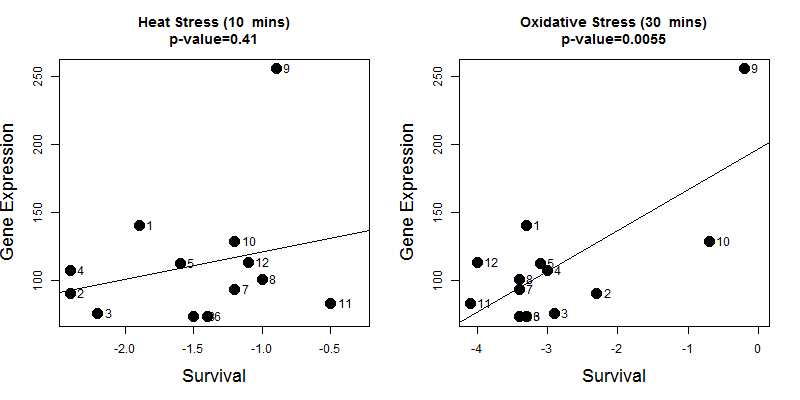

Supplement: S5 File — Expression levels of genes LACR_0001 –LACR_1382 plotted against survival after 10 minutes heat and 30 minutes oxidative stress. Survival is expressed as the difference of log CFU/ml after stress and before stress. Numbers indicate fermentations as presented in Table 1. P-values above the plots indicate significance of correlation (assessed by a linear model). (ZIP) [file pone.0167944.s010.zip › S5_File/LACR_0105_real_dat.png]

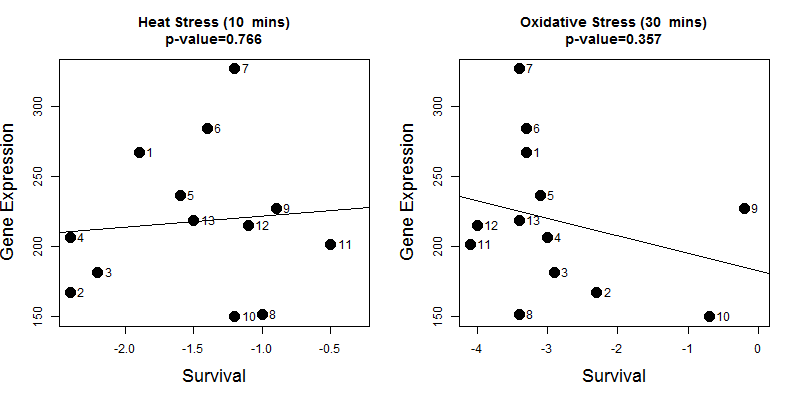

Supplement: S5 File — Expression levels of genes LACR_0001 –LACR_1382 plotted against survival after 10 minutes heat and 30 minutes oxidative stress. Survival is expressed as the difference of log CFU/ml after stress and before stress. Numbers indicate fermentations as presented in Table 1. P-values above the plots indicate significance of correlation (assessed by a linear model). (ZIP) [file pone.0167944.s010.zip › S5_File/LACR_0106_real_dat.png]

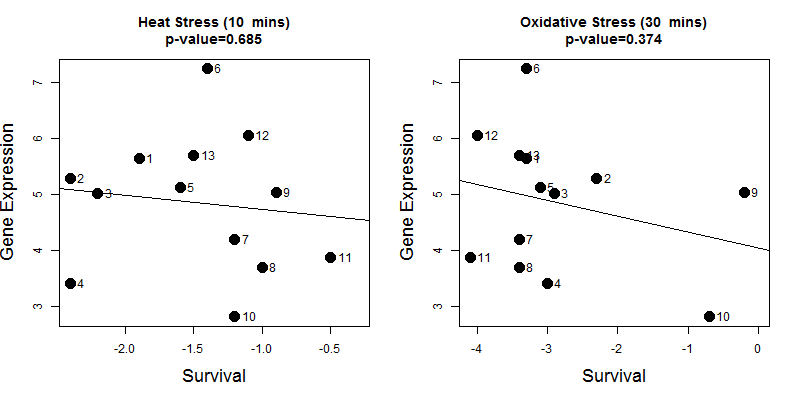

Supplement: S5 File — Expression levels of genes LACR_0001 –LACR_1382 plotted against survival after 10 minutes heat and 30 minutes oxidative stress. Survival is expressed as the difference of log CFU/ml after stress and before stress. Numbers indicate fermentations as presented in Table 1. P-values above the plots indicate significance of correlation (assessed by a linear model). (ZIP) [file pone.0167944.s010.zip › S5_File/LACR_0107_real_dat.png]

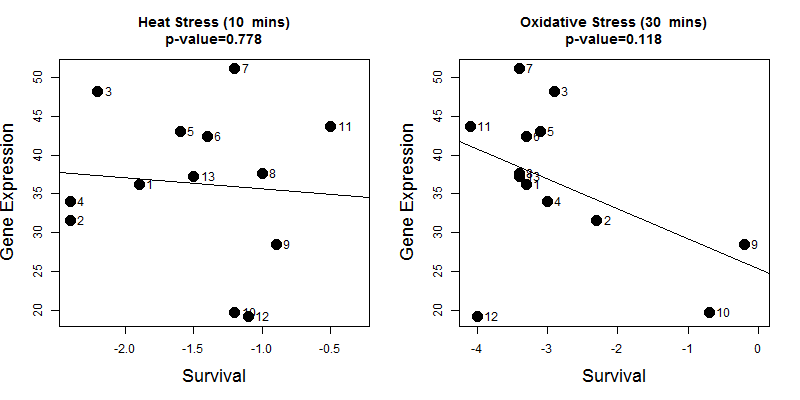

Supplement: S5 File — Expression levels of genes LACR_0001 –LACR_1382 plotted against survival after 10 minutes heat and 30 minutes oxidative stress. Survival is expressed as the difference of log CFU/ml after stress and before stress. Numbers indicate fermentations as presented in Table 1. P-values above the plots indicate significance of correlation (assessed by a linear model). (ZIP) [file pone.0167944.s010.zip › S5_File/LACR_0109_real_dat.png]

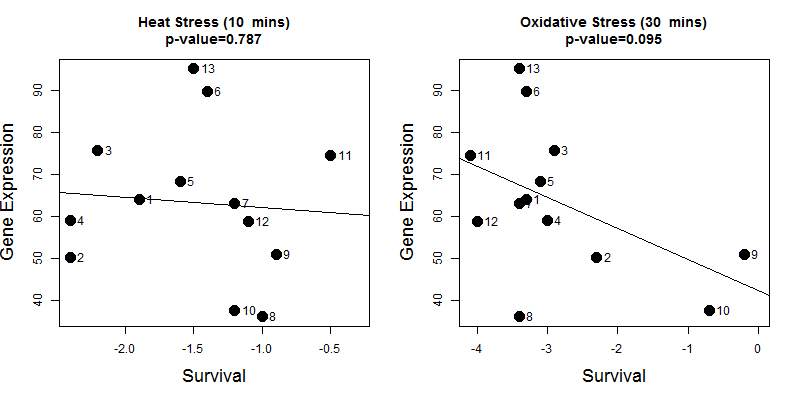

Supplement: S5 File — Expression levels of genes LACR_0001 –LACR_1382 plotted against survival after 10 minutes heat and 30 minutes oxidative stress. Survival is expressed as the difference of log CFU/ml after stress and before stress. Numbers indicate fermentations as presented in Table 1. P-values above the plots indicate significance of correlation (assessed by a linear model). (ZIP) [file pone.0167944.s010.zip › S5_File/LACR_0110_real_dat.png]

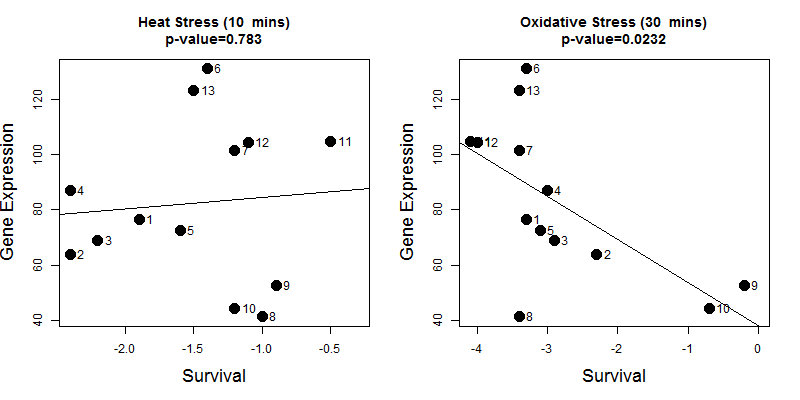

Supplement: S5 File — Expression levels of genes LACR_0001 –LACR_1382 plotted against survival after 10 minutes heat and 30 minutes oxidative stress. Survival is expressed as the difference of log CFU/ml after stress and before stress. Numbers indicate fermentations as presented in Table 1. P-values above the plots indicate significance of correlation (assessed by a linear model). (ZIP) [file pone.0167944.s010.zip › S5_File/LACR_0111_real_dat.png]

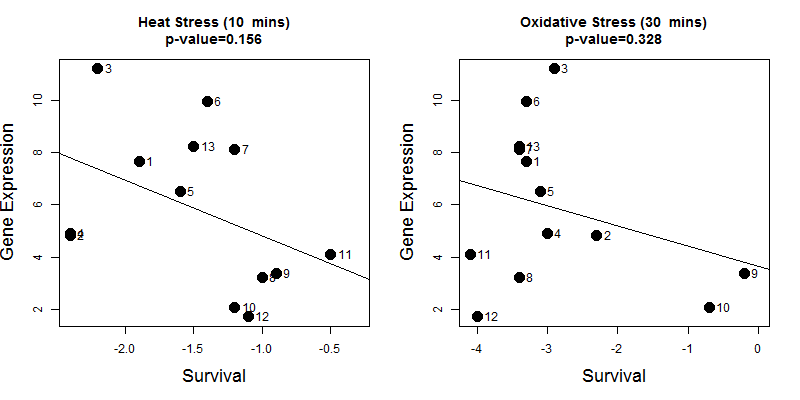

Supplement: S5 File — Expression levels of genes LACR_0001 –LACR_1382 plotted against survival after 10 minutes heat and 30 minutes oxidative stress. Survival is expressed as the difference of log CFU/ml after stress and before stress. Numbers indicate fermentations as presented in Table 1. P-values above the plots indicate significance of correlation (assessed by a linear model). (ZIP) [file pone.0167944.s010.zip › S5_File/LACR_0112_real_dat.png]
