# Supplementary figures and images for: Strain-Dependent Transcriptome Signatures for Robustness in Lactococcus lactis (part 8 of 13)
Source: PLoS One. 2016 Dec 14;11(12):e0167944. doi: 10.1371/journal.pone.0167944 (PMC5156439; doi:10.1371/journal.pone.0167944)

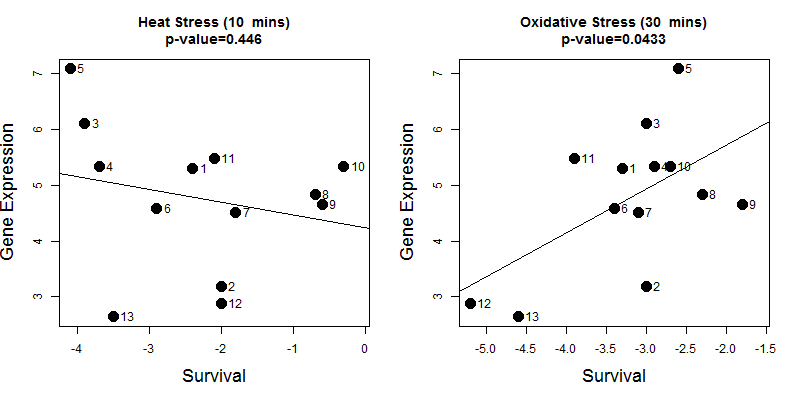

Supplement: S4 File — Expression levels of genes LLKF_1274 –LLKF_2533 and LLKF_p0001 –LLKF_p0036 plotted against survival after 10 minutes heat and 30 minutes oxidative stress. Survival is expressed as the difference of log CFU/ml after stress and before stress. Numbers indicate fermentations as presented in Table 1. P-values above the plots indicate significance of correlation (assessed by a linear model). (ZIP) [file pone.0167944.s009.zip › S4_File/LLKF_1380_real_dat.png]

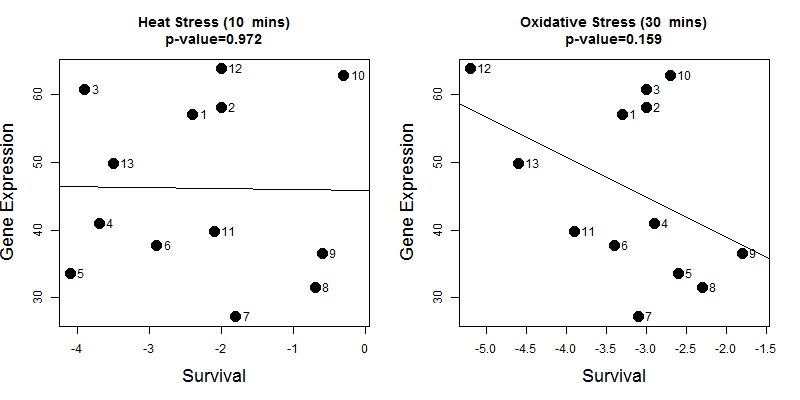

Supplement: S4 File — Expression levels of genes LLKF_1274 –LLKF_2533 and LLKF_p0001 –LLKF_p0036 plotted against survival after 10 minutes heat and 30 minutes oxidative stress. Survival is expressed as the difference of log CFU/ml after stress and before stress. Numbers indicate fermentations as presented in Table 1. P-values above the plots indicate significance of correlation (assessed by a linear model). (ZIP) [file pone.0167944.s009.zip › S4_File/LLKF_1381_real_dat.png]

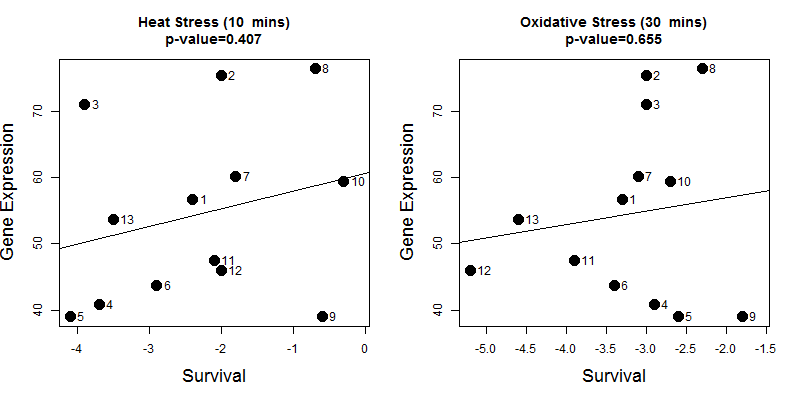

Supplement: S4 File — Expression levels of genes LLKF_1274 –LLKF_2533 and LLKF_p0001 –LLKF_p0036 plotted against survival after 10 minutes heat and 30 minutes oxidative stress. Survival is expressed as the difference of log CFU/ml after stress and before stress. Numbers indicate fermentations as presented in Table 1. P-values above the plots indicate significance of correlation (assessed by a linear model). (ZIP) [file pone.0167944.s009.zip › S4_File/LLKF_1382_real_dat.png]

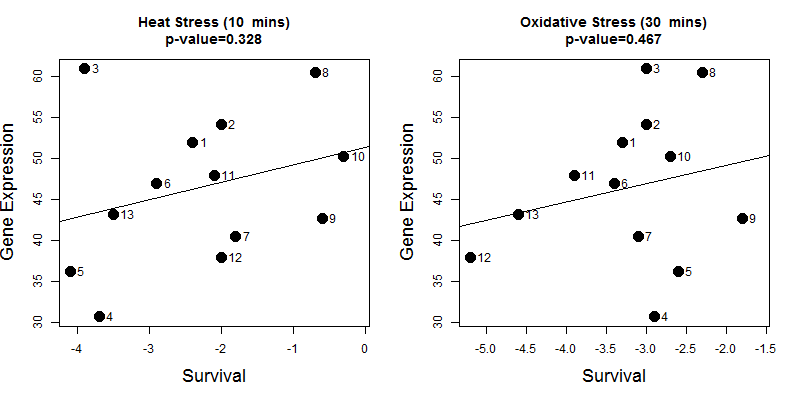

Supplement: S4 File — Expression levels of genes LLKF_1274 –LLKF_2533 and LLKF_p0001 –LLKF_p0036 plotted against survival after 10 minutes heat and 30 minutes oxidative stress. Survival is expressed as the difference of log CFU/ml after stress and before stress. Numbers indicate fermentations as presented in Table 1. P-values above the plots indicate significance of correlation (assessed by a linear model). (ZIP) [file pone.0167944.s009.zip › S4_File/LLKF_1383_real_dat.png]

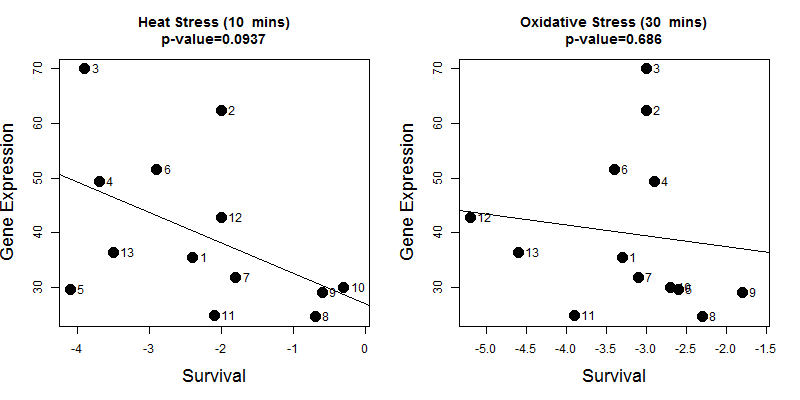

Supplement: S4 File — Expression levels of genes LLKF_1274 –LLKF_2533 and LLKF_p0001 –LLKF_p0036 plotted against survival after 10 minutes heat and 30 minutes oxidative stress. Survival is expressed as the difference of log CFU/ml after stress and before stress. Numbers indicate fermentations as presented in Table 1. P-values above the plots indicate significance of correlation (assessed by a linear model). (ZIP) [file pone.0167944.s009.zip › S4_File/LLKF_1384_real_dat.png]

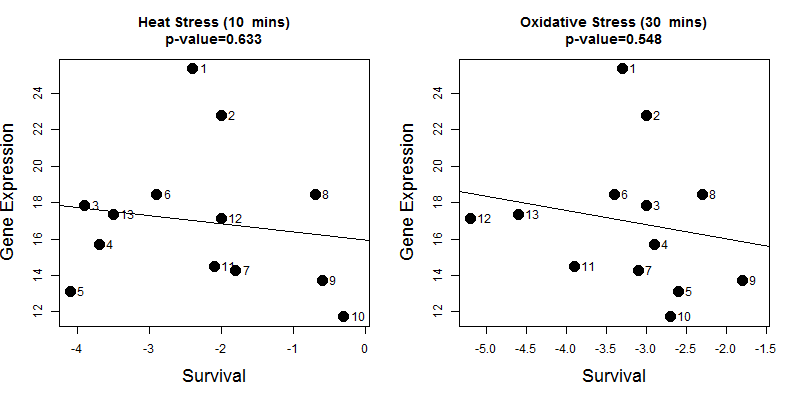

Supplement: S4 File — Expression levels of genes LLKF_1274 –LLKF_2533 and LLKF_p0001 –LLKF_p0036 plotted against survival after 10 minutes heat and 30 minutes oxidative stress. Survival is expressed as the difference of log CFU/ml after stress and before stress. Numbers indicate fermentations as presented in Table 1. P-values above the plots indicate significance of correlation (assessed by a linear model). (ZIP) [file pone.0167944.s009.zip › S4_File/LLKF_1385_real_dat.png]

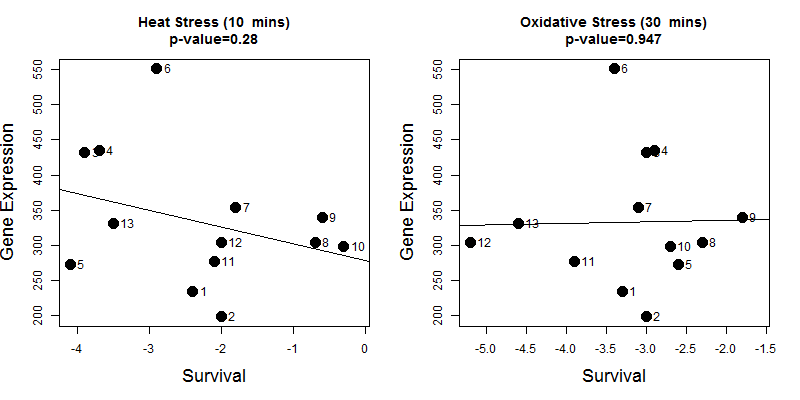

Supplement: S4 File — Expression levels of genes LLKF_1274 –LLKF_2533 and LLKF_p0001 –LLKF_p0036 plotted against survival after 10 minutes heat and 30 minutes oxidative stress. Survival is expressed as the difference of log CFU/ml after stress and before stress. Numbers indicate fermentations as presented in Table 1. P-values above the plots indicate significance of correlation (assessed by a linear model). (ZIP) [file pone.0167944.s009.zip › S4_File/LLKF_1386_real_dat.png]

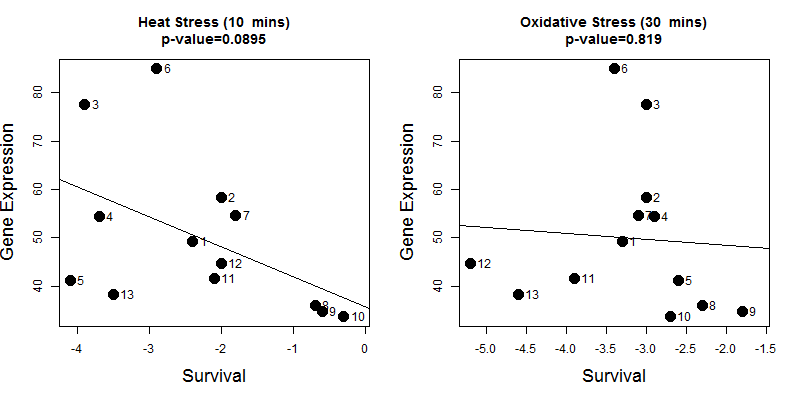

Supplement: S4 File — Expression levels of genes LLKF_1274 –LLKF_2533 and LLKF_p0001 –LLKF_p0036 plotted against survival after 10 minutes heat and 30 minutes oxidative stress. Survival is expressed as the difference of log CFU/ml after stress and before stress. Numbers indicate fermentations as presented in Table 1. P-values above the plots indicate significance of correlation (assessed by a linear model). (ZIP) [file pone.0167944.s009.zip › S4_File/LLKF_1387_real_dat.png]

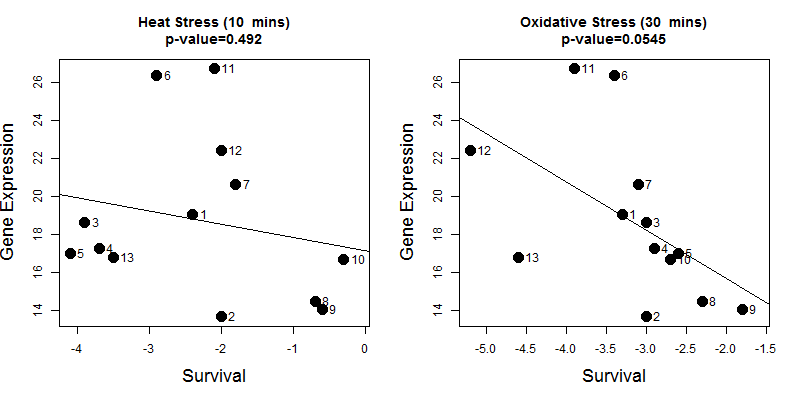

Supplement: S4 File — Expression levels of genes LLKF_1274 –LLKF_2533 and LLKF_p0001 –LLKF_p0036 plotted against survival after 10 minutes heat and 30 minutes oxidative stress. Survival is expressed as the difference of log CFU/ml after stress and before stress. Numbers indicate fermentations as presented in Table 1. P-values above the plots indicate significance of correlation (assessed by a linear model). (ZIP) [file pone.0167944.s009.zip › S4_File/LLKF_1388_real_dat.png]

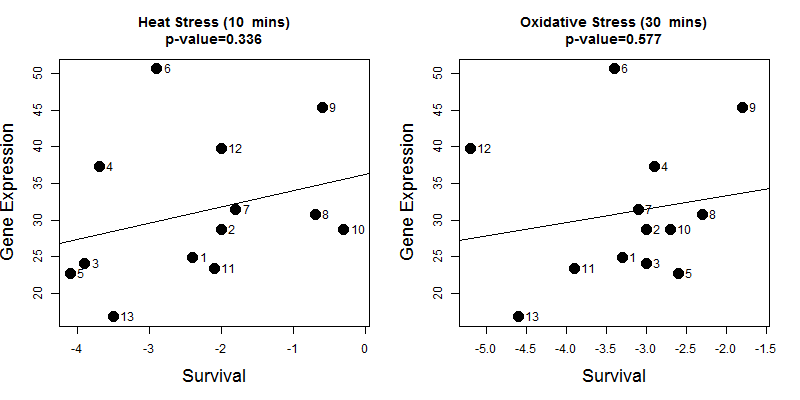

Supplement: S4 File — Expression levels of genes LLKF_1274 –LLKF_2533 and LLKF_p0001 –LLKF_p0036 plotted against survival after 10 minutes heat and 30 minutes oxidative stress. Survival is expressed as the difference of log CFU/ml after stress and before stress. Numbers indicate fermentations as presented in Table 1. P-values above the plots indicate significance of correlation (assessed by a linear model). (ZIP) [file pone.0167944.s009.zip › S4_File/LLKF_1389_real_dat.png]

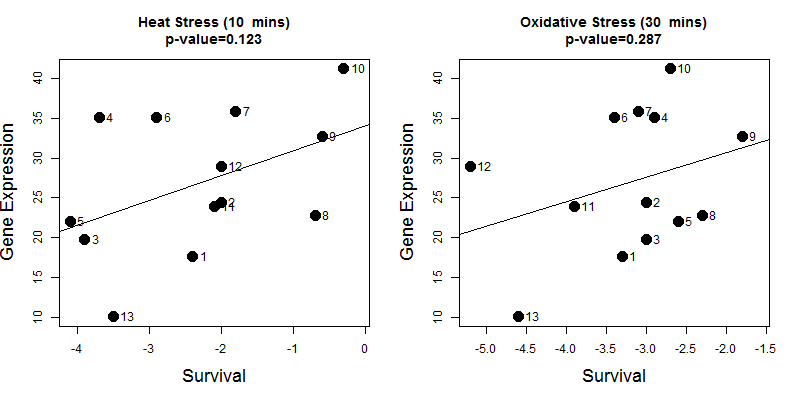

Supplement: S4 File — Expression levels of genes LLKF_1274 –LLKF_2533 and LLKF_p0001 –LLKF_p0036 plotted against survival after 10 minutes heat and 30 minutes oxidative stress. Survival is expressed as the difference of log CFU/ml after stress and before stress. Numbers indicate fermentations as presented in Table 1. P-values above the plots indicate significance of correlation (assessed by a linear model). (ZIP) [file pone.0167944.s009.zip › S4_File/LLKF_1390_real_dat.png]

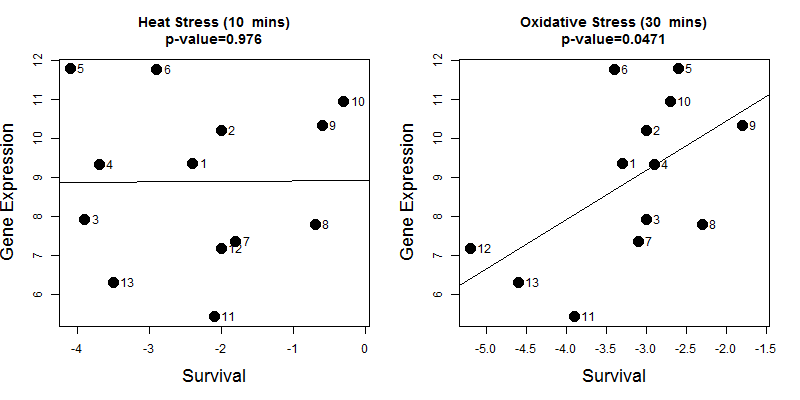

Supplement: S4 File — Expression levels of genes LLKF_1274 –LLKF_2533 and LLKF_p0001 –LLKF_p0036 plotted against survival after 10 minutes heat and 30 minutes oxidative stress. Survival is expressed as the difference of log CFU/ml after stress and before stress. Numbers indicate fermentations as presented in Table 1. P-values above the plots indicate significance of correlation (assessed by a linear model). (ZIP) [file pone.0167944.s009.zip › S4_File/LLKF_1391_real_dat.png]

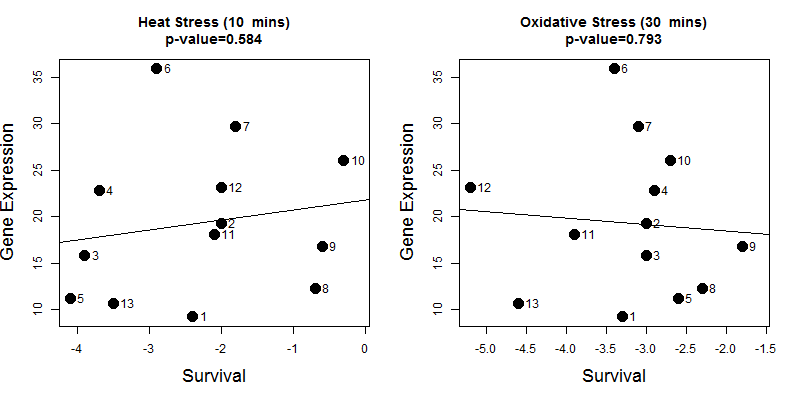

Supplement: S4 File — Expression levels of genes LLKF_1274 –LLKF_2533 and LLKF_p0001 –LLKF_p0036 plotted against survival after 10 minutes heat and 30 minutes oxidative stress. Survival is expressed as the difference of log CFU/ml after stress and before stress. Numbers indicate fermentations as presented in Table 1. P-values above the plots indicate significance of correlation (assessed by a linear model). (ZIP) [file pone.0167944.s009.zip › S4_File/LLKF_1392_real_dat.png]

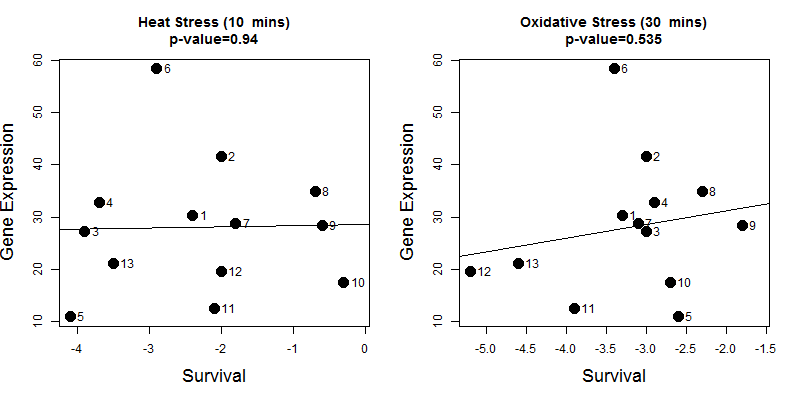

Supplement: S4 File — Expression levels of genes LLKF_1274 –LLKF_2533 and LLKF_p0001 –LLKF_p0036 plotted against survival after 10 minutes heat and 30 minutes oxidative stress. Survival is expressed as the difference of log CFU/ml after stress and before stress. Numbers indicate fermentations as presented in Table 1. P-values above the plots indicate significance of correlation (assessed by a linear model). (ZIP) [file pone.0167944.s009.zip › S4_File/LLKF_1393_real_dat.png]

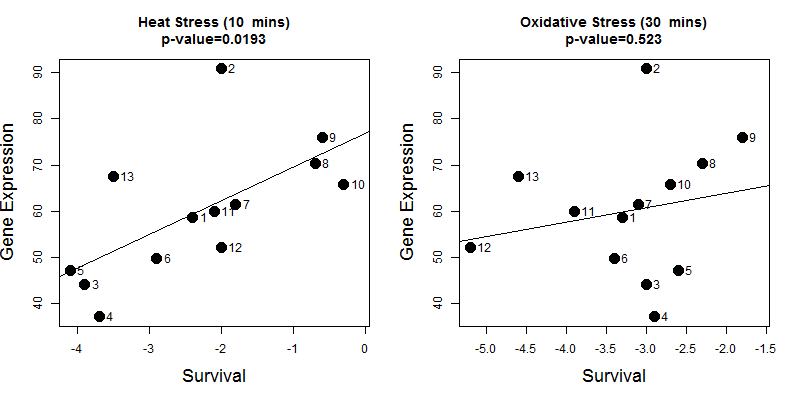

Supplement: S4 File — Expression levels of genes LLKF_1274 –LLKF_2533 and LLKF_p0001 –LLKF_p0036 plotted against survival after 10 minutes heat and 30 minutes oxidative stress. Survival is expressed as the difference of log CFU/ml after stress and before stress. Numbers indicate fermentations as presented in Table 1. P-values above the plots indicate significance of correlation (assessed by a linear model). (ZIP) [file pone.0167944.s009.zip › S4_File/LLKF_1394_real_dat.png]

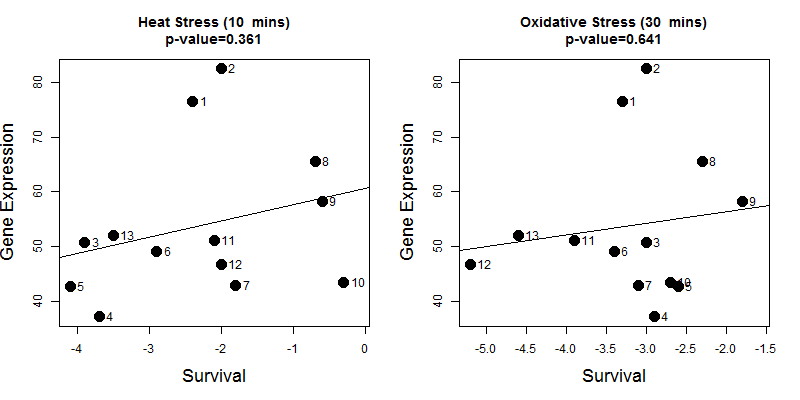

Supplement: S4 File — Expression levels of genes LLKF_1274 –LLKF_2533 and LLKF_p0001 –LLKF_p0036 plotted against survival after 10 minutes heat and 30 minutes oxidative stress. Survival is expressed as the difference of log CFU/ml after stress and before stress. Numbers indicate fermentations as presented in Table 1. P-values above the plots indicate significance of correlation (assessed by a linear model). (ZIP) [file pone.0167944.s009.zip › S4_File/LLKF_1395_real_dat.png]

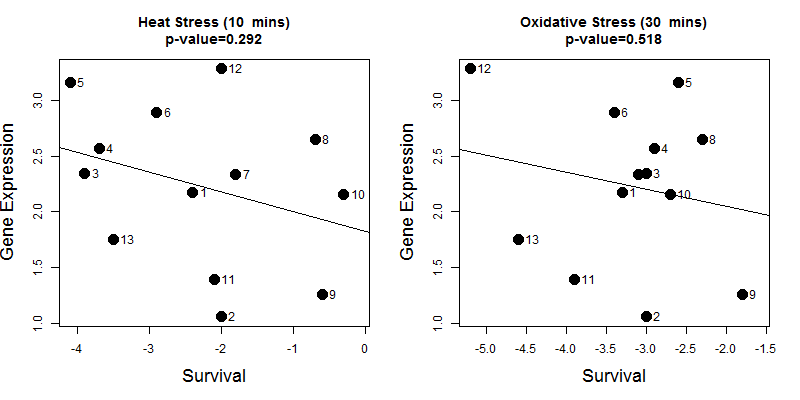

Supplement: S4 File — Expression levels of genes LLKF_1274 –LLKF_2533 and LLKF_p0001 –LLKF_p0036 plotted against survival after 10 minutes heat and 30 minutes oxidative stress. Survival is expressed as the difference of log CFU/ml after stress and before stress. Numbers indicate fermentations as presented in Table 1. P-values above the plots indicate significance of correlation (assessed by a linear model). (ZIP) [file pone.0167944.s009.zip › S4_File/LLKF_1396_real_dat.png]

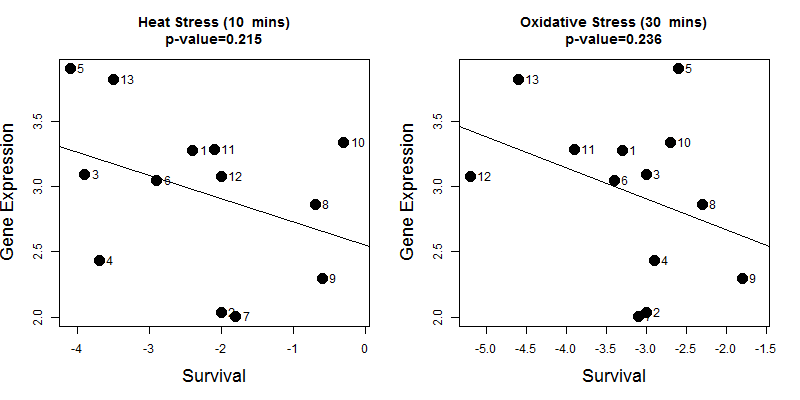

Supplement: S4 File — Expression levels of genes LLKF_1274 –LLKF_2533 and LLKF_p0001 –LLKF_p0036 plotted against survival after 10 minutes heat and 30 minutes oxidative stress. Survival is expressed as the difference of log CFU/ml after stress and before stress. Numbers indicate fermentations as presented in Table 1. P-values above the plots indicate significance of correlation (assessed by a linear model). (ZIP) [file pone.0167944.s009.zip › S4_File/LLKF_1397_real_dat.png]

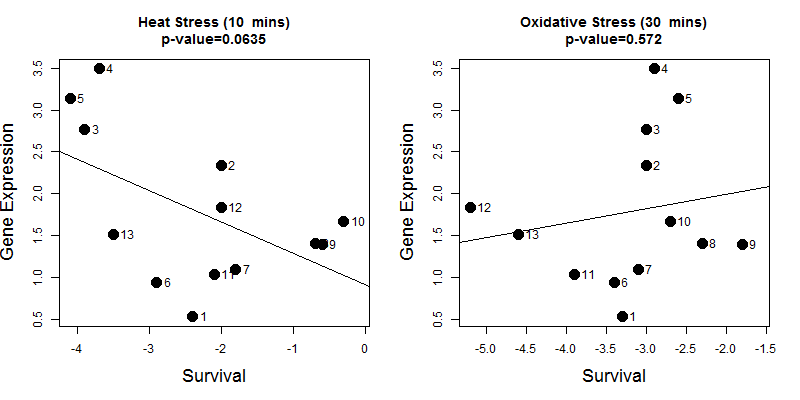

Supplement: S4 File — Expression levels of genes LLKF_1274 –LLKF_2533 and LLKF_p0001 –LLKF_p0036 plotted against survival after 10 minutes heat and 30 minutes oxidative stress. Survival is expressed as the difference of log CFU/ml after stress and before stress. Numbers indicate fermentations as presented in Table 1. P-values above the plots indicate significance of correlation (assessed by a linear model). (ZIP) [file pone.0167944.s009.zip › S4_File/LLKF_1398_real_dat.png]

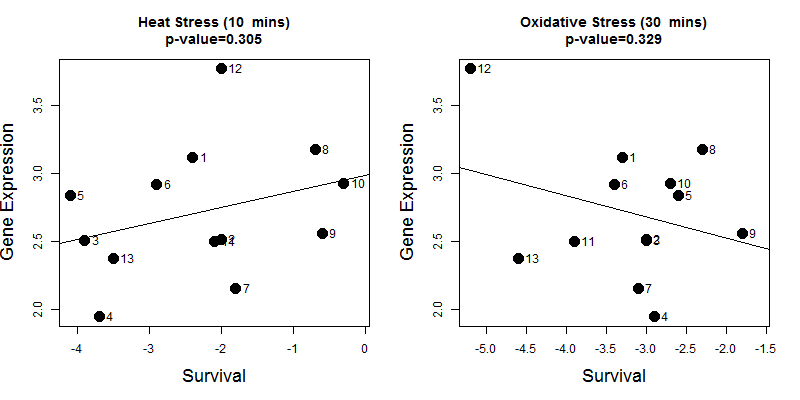

Supplement: S4 File — Expression levels of genes LLKF_1274 –LLKF_2533 and LLKF_p0001 –LLKF_p0036 plotted against survival after 10 minutes heat and 30 minutes oxidative stress. Survival is expressed as the difference of log CFU/ml after stress and before stress. Numbers indicate fermentations as presented in Table 1. P-values above the plots indicate significance of correlation (assessed by a linear model). (ZIP) [file pone.0167944.s009.zip › S4_File/LLKF_1399_real_dat.png]

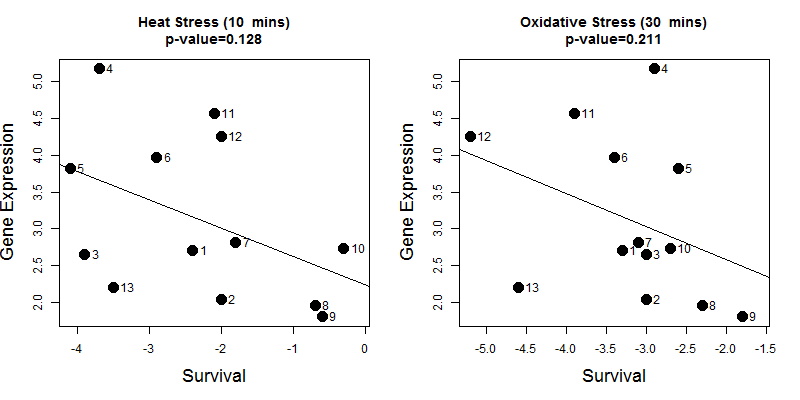

Supplement: S4 File — Expression levels of genes LLKF_1274 –LLKF_2533 and LLKF_p0001 –LLKF_p0036 plotted against survival after 10 minutes heat and 30 minutes oxidative stress. Survival is expressed as the difference of log CFU/ml after stress and before stress. Numbers indicate fermentations as presented in Table 1. P-values above the plots indicate significance of correlation (assessed by a linear model). (ZIP) [file pone.0167944.s009.zip › S4_File/LLKF_1400_real_dat.png]

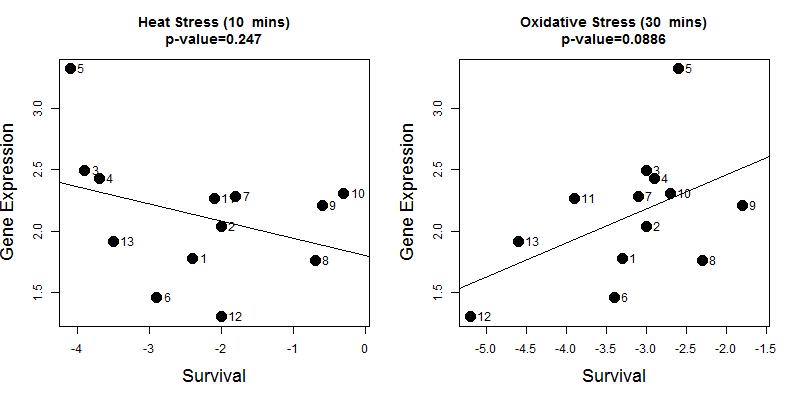

Supplement: S4 File — Expression levels of genes LLKF_1274 –LLKF_2533 and LLKF_p0001 –LLKF_p0036 plotted against survival after 10 minutes heat and 30 minutes oxidative stress. Survival is expressed as the difference of log CFU/ml after stress and before stress. Numbers indicate fermentations as presented in Table 1. P-values above the plots indicate significance of correlation (assessed by a linear model). (ZIP) [file pone.0167944.s009.zip › S4_File/LLKF_1401_real_dat.png]

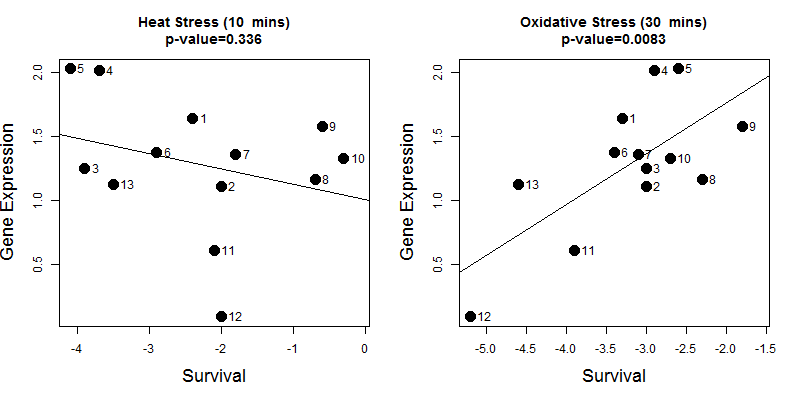

Supplement: S4 File — Expression levels of genes LLKF_1274 –LLKF_2533 and LLKF_p0001 –LLKF_p0036 plotted against survival after 10 minutes heat and 30 minutes oxidative stress. Survival is expressed as the difference of log CFU/ml after stress and before stress. Numbers indicate fermentations as presented in Table 1. P-values above the plots indicate significance of correlation (assessed by a linear model). (ZIP) [file pone.0167944.s009.zip › S4_File/LLKF_1402_real_dat.png]

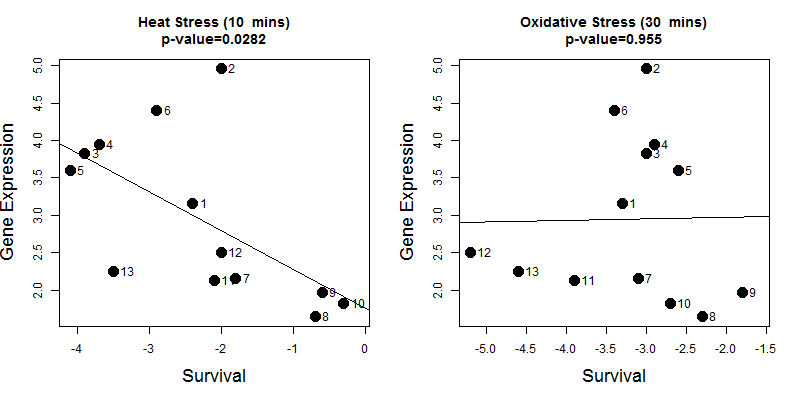

Supplement: S4 File — Expression levels of genes LLKF_1274 –LLKF_2533 and LLKF_p0001 –LLKF_p0036 plotted against survival after 10 minutes heat and 30 minutes oxidative stress. Survival is expressed as the difference of log CFU/ml after stress and before stress. Numbers indicate fermentations as presented in Table 1. P-values above the plots indicate significance of correlation (assessed by a linear model). (ZIP) [file pone.0167944.s009.zip › S4_File/LLKF_1403_real_dat.png]

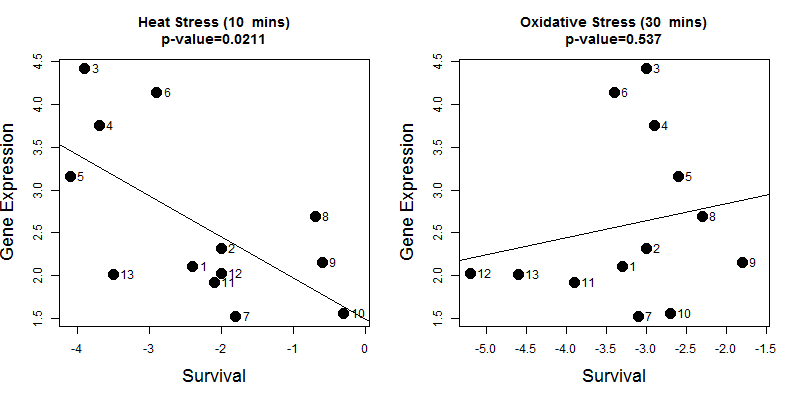

Supplement: S4 File — Expression levels of genes LLKF_1274 –LLKF_2533 and LLKF_p0001 –LLKF_p0036 plotted against survival after 10 minutes heat and 30 minutes oxidative stress. Survival is expressed as the difference of log CFU/ml after stress and before stress. Numbers indicate fermentations as presented in Table 1. P-values above the plots indicate significance of correlation (assessed by a linear model). (ZIP) [file pone.0167944.s009.zip › S4_File/LLKF_1404_real_dat.png]

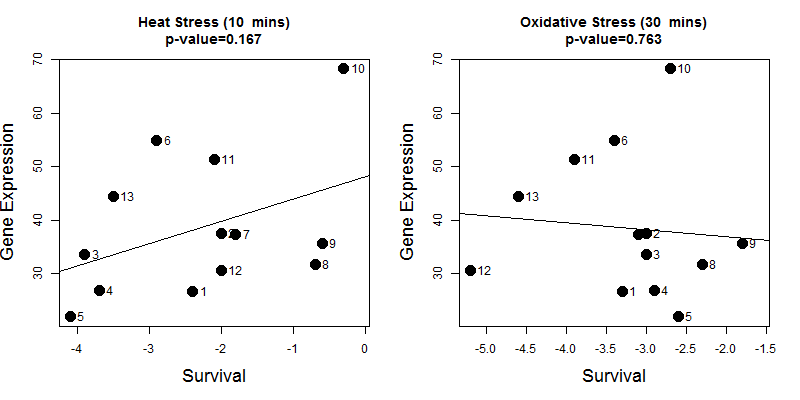

Supplement: S4 File — Expression levels of genes LLKF_1274 –LLKF_2533 and LLKF_p0001 –LLKF_p0036 plotted against survival after 10 minutes heat and 30 minutes oxidative stress. Survival is expressed as the difference of log CFU/ml after stress and before stress. Numbers indicate fermentations as presented in Table 1. P-values above the plots indicate significance of correlation (assessed by a linear model). (ZIP) [file pone.0167944.s009.zip › S4_File/LLKF_1405_real_dat.png]

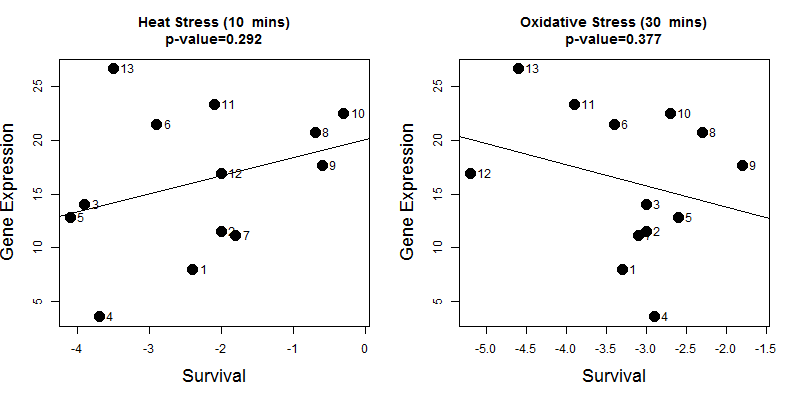

Supplement: S4 File — Expression levels of genes LLKF_1274 –LLKF_2533 and LLKF_p0001 –LLKF_p0036 plotted against survival after 10 minutes heat and 30 minutes oxidative stress. Survival is expressed as the difference of log CFU/ml after stress and before stress. Numbers indicate fermentations as presented in Table 1. P-values above the plots indicate significance of correlation (assessed by a linear model). (ZIP) [file pone.0167944.s009.zip › S4_File/LLKF_1406_real_dat.png]

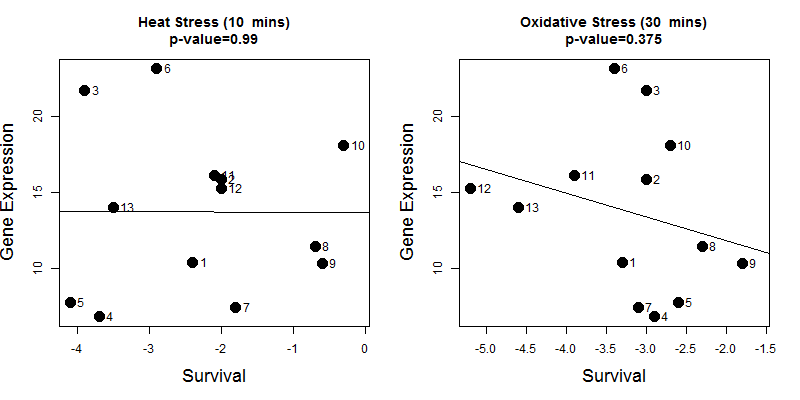

Supplement: S4 File — Expression levels of genes LLKF_1274 –LLKF_2533 and LLKF_p0001 –LLKF_p0036 plotted against survival after 10 minutes heat and 30 minutes oxidative stress. Survival is expressed as the difference of log CFU/ml after stress and before stress. Numbers indicate fermentations as presented in Table 1. P-values above the plots indicate significance of correlation (assessed by a linear model). (ZIP) [file pone.0167944.s009.zip › S4_File/LLKF_1407_real_dat.png]

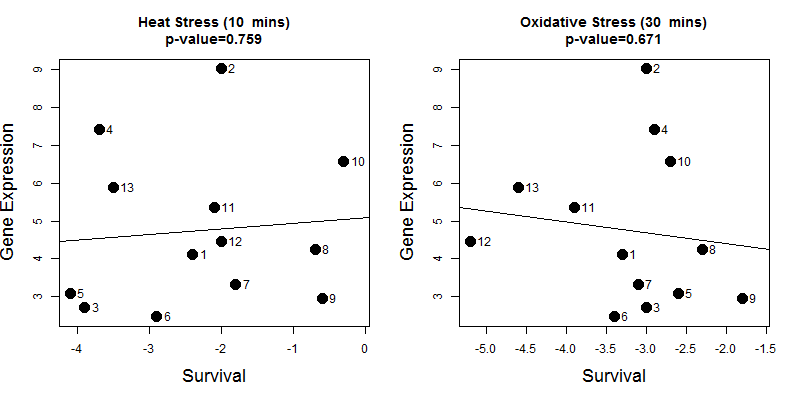

Supplement: S4 File — Expression levels of genes LLKF_1274 –LLKF_2533 and LLKF_p0001 –LLKF_p0036 plotted against survival after 10 minutes heat and 30 minutes oxidative stress. Survival is expressed as the difference of log CFU/ml after stress and before stress. Numbers indicate fermentations as presented in Table 1. P-values above the plots indicate significance of correlation (assessed by a linear model). (ZIP) [file pone.0167944.s009.zip › S4_File/LLKF_1408_real_dat.png]

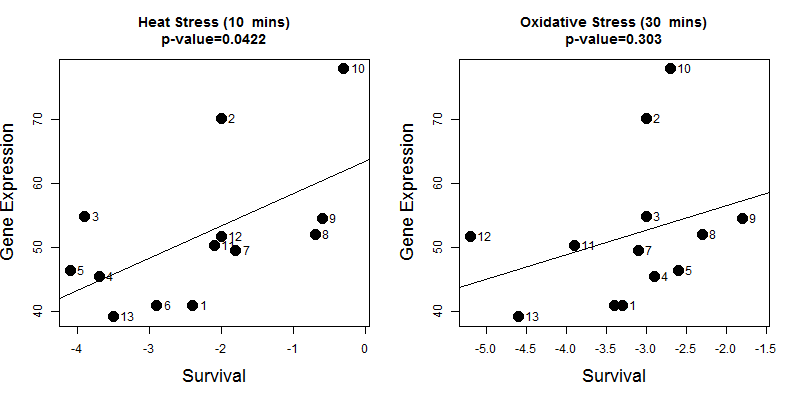

Supplement: S4 File — Expression levels of genes LLKF_1274 –LLKF_2533 and LLKF_p0001 –LLKF_p0036 plotted against survival after 10 minutes heat and 30 minutes oxidative stress. Survival is expressed as the difference of log CFU/ml after stress and before stress. Numbers indicate fermentations as presented in Table 1. P-values above the plots indicate significance of correlation (assessed by a linear model). (ZIP) [file pone.0167944.s009.zip › S4_File/LLKF_1409_real_dat.png]

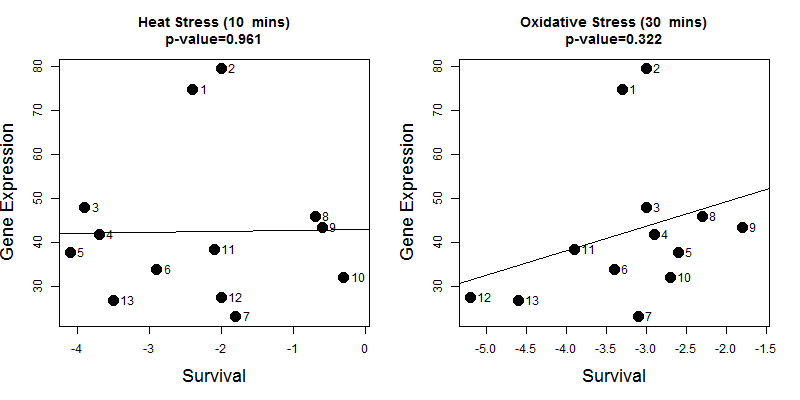

Supplement: S4 File — Expression levels of genes LLKF_1274 –LLKF_2533 and LLKF_p0001 –LLKF_p0036 plotted against survival after 10 minutes heat and 30 minutes oxidative stress. Survival is expressed as the difference of log CFU/ml after stress and before stress. Numbers indicate fermentations as presented in Table 1. P-values above the plots indicate significance of correlation (assessed by a linear model). (ZIP) [file pone.0167944.s009.zip › S4_File/LLKF_1410_real_dat.png]

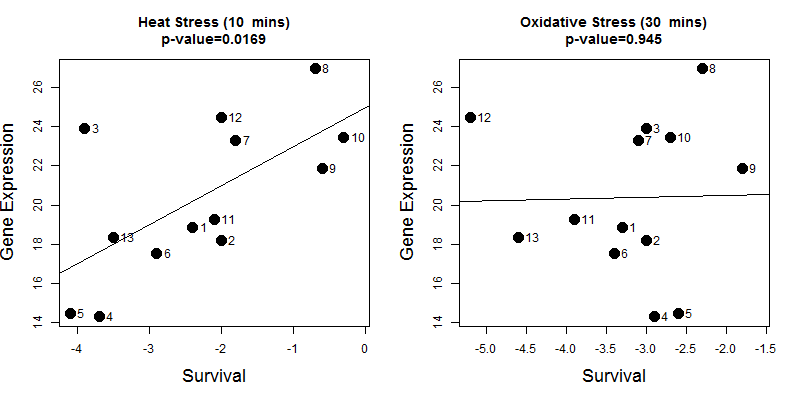

Supplement: S4 File — Expression levels of genes LLKF_1274 –LLKF_2533 and LLKF_p0001 –LLKF_p0036 plotted against survival after 10 minutes heat and 30 minutes oxidative stress. Survival is expressed as the difference of log CFU/ml after stress and before stress. Numbers indicate fermentations as presented in Table 1. P-values above the plots indicate significance of correlation (assessed by a linear model). (ZIP) [file pone.0167944.s009.zip › S4_File/LLKF_1411_real_dat.png]

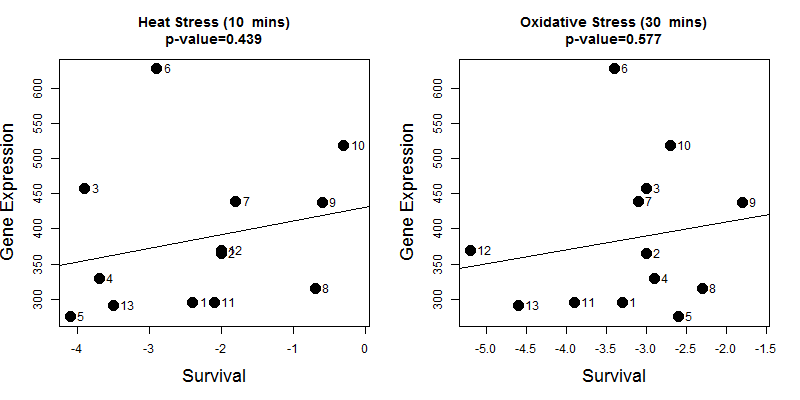

Supplement: S4 File — Expression levels of genes LLKF_1274 –LLKF_2533 and LLKF_p0001 –LLKF_p0036 plotted against survival after 10 minutes heat and 30 minutes oxidative stress. Survival is expressed as the difference of log CFU/ml after stress and before stress. Numbers indicate fermentations as presented in Table 1. P-values above the plots indicate significance of correlation (assessed by a linear model). (ZIP) [file pone.0167944.s009.zip › S4_File/LLKF_1412_real_dat.png]

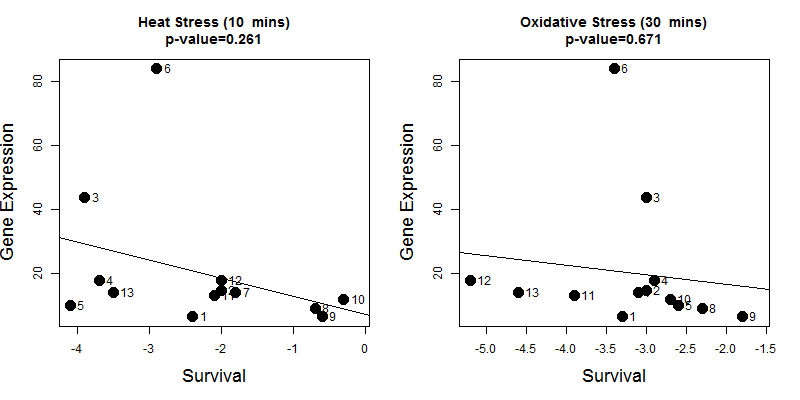

Supplement: S4 File — Expression levels of genes LLKF_1274 –LLKF_2533 and LLKF_p0001 –LLKF_p0036 plotted against survival after 10 minutes heat and 30 minutes oxidative stress. Survival is expressed as the difference of log CFU/ml after stress and before stress. Numbers indicate fermentations as presented in Table 1. P-values above the plots indicate significance of correlation (assessed by a linear model). (ZIP) [file pone.0167944.s009.zip › S4_File/LLKF_1413_real_dat.png]

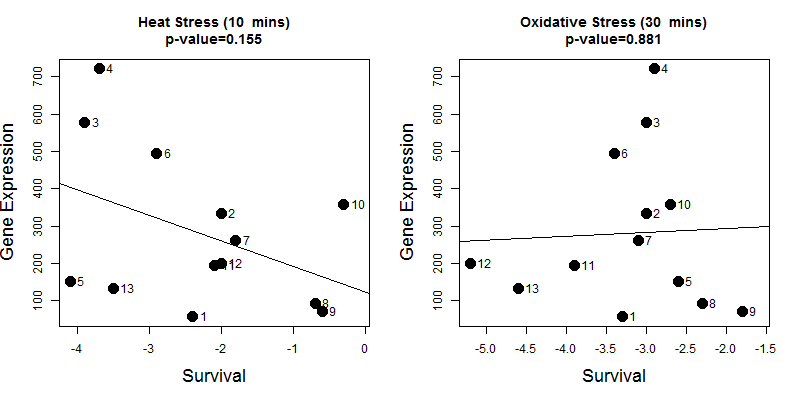

Supplement: S4 File — Expression levels of genes LLKF_1274 –LLKF_2533 and LLKF_p0001 –LLKF_p0036 plotted against survival after 10 minutes heat and 30 minutes oxidative stress. Survival is expressed as the difference of log CFU/ml after stress and before stress. Numbers indicate fermentations as presented in Table 1. P-values above the plots indicate significance of correlation (assessed by a linear model). (ZIP) [file pone.0167944.s009.zip › S4_File/LLKF_1414_real_dat.png]

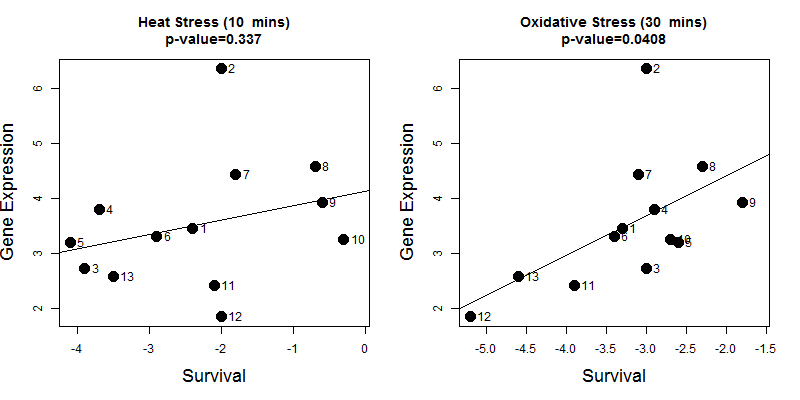

Supplement: S4 File — Expression levels of genes LLKF_1274 –LLKF_2533 and LLKF_p0001 –LLKF_p0036 plotted against survival after 10 minutes heat and 30 minutes oxidative stress. Survival is expressed as the difference of log CFU/ml after stress and before stress. Numbers indicate fermentations as presented in Table 1. P-values above the plots indicate significance of correlation (assessed by a linear model). (ZIP) [file pone.0167944.s009.zip › S4_File/LLKF_1415_real_dat.png]

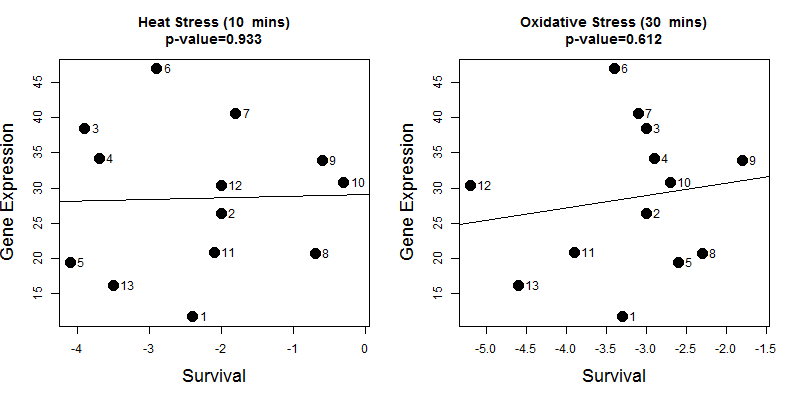

Supplement: S4 File — Expression levels of genes LLKF_1274 –LLKF_2533 and LLKF_p0001 –LLKF_p0036 plotted against survival after 10 minutes heat and 30 minutes oxidative stress. Survival is expressed as the difference of log CFU/ml after stress and before stress. Numbers indicate fermentations as presented in Table 1. P-values above the plots indicate significance of correlation (assessed by a linear model). (ZIP) [file pone.0167944.s009.zip › S4_File/LLKF_1416_real_dat.png]

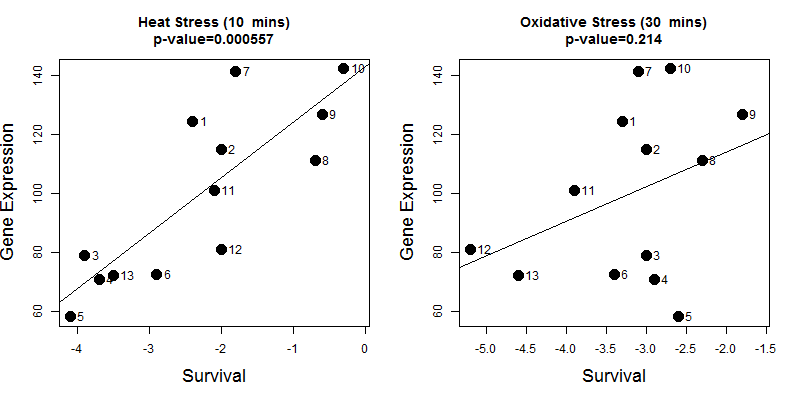

Supplement: S4 File — Expression levels of genes LLKF_1274 –LLKF_2533 and LLKF_p0001 –LLKF_p0036 plotted against survival after 10 minutes heat and 30 minutes oxidative stress. Survival is expressed as the difference of log CFU/ml after stress and before stress. Numbers indicate fermentations as presented in Table 1. P-values above the plots indicate significance of correlation (assessed by a linear model). (ZIP) [file pone.0167944.s009.zip › S4_File/LLKF_1417_real_dat.png]

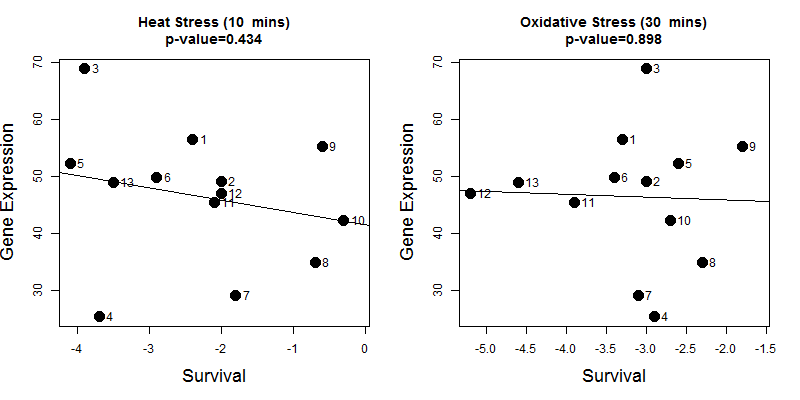

Supplement: S4 File — Expression levels of genes LLKF_1274 –LLKF_2533 and LLKF_p0001 –LLKF_p0036 plotted against survival after 10 minutes heat and 30 minutes oxidative stress. Survival is expressed as the difference of log CFU/ml after stress and before stress. Numbers indicate fermentations as presented in Table 1. P-values above the plots indicate significance of correlation (assessed by a linear model). (ZIP) [file pone.0167944.s009.zip › S4_File/LLKF_1418_real_dat.png]

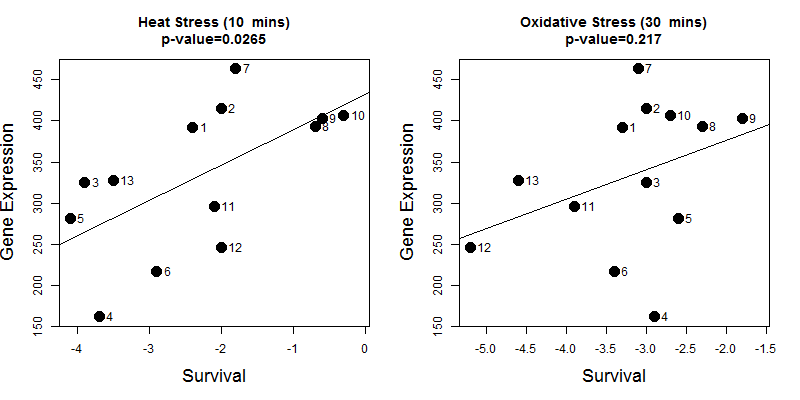

Supplement: S4 File — Expression levels of genes LLKF_1274 –LLKF_2533 and LLKF_p0001 –LLKF_p0036 plotted against survival after 10 minutes heat and 30 minutes oxidative stress. Survival is expressed as the difference of log CFU/ml after stress and before stress. Numbers indicate fermentations as presented in Table 1. P-values above the plots indicate significance of correlation (assessed by a linear model). (ZIP) [file pone.0167944.s009.zip › S4_File/LLKF_1419_real_dat.png]

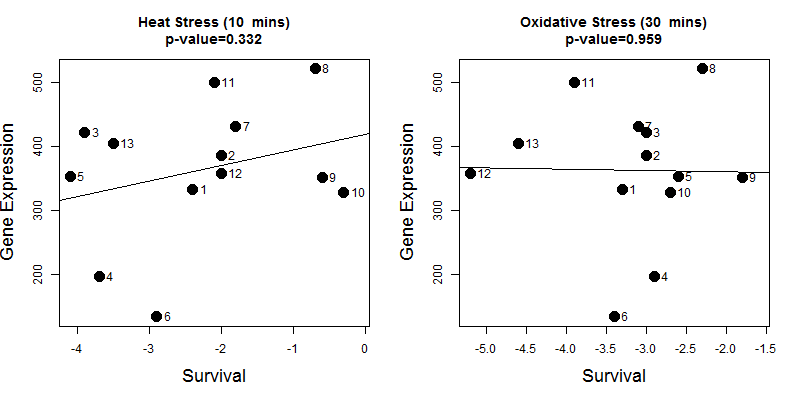

Supplement: S4 File — Expression levels of genes LLKF_1274 –LLKF_2533 and LLKF_p0001 –LLKF_p0036 plotted against survival after 10 minutes heat and 30 minutes oxidative stress. Survival is expressed as the difference of log CFU/ml after stress and before stress. Numbers indicate fermentations as presented in Table 1. P-values above the plots indicate significance of correlation (assessed by a linear model). (ZIP) [file pone.0167944.s009.zip › S4_File/LLKF_1420_real_dat.png]

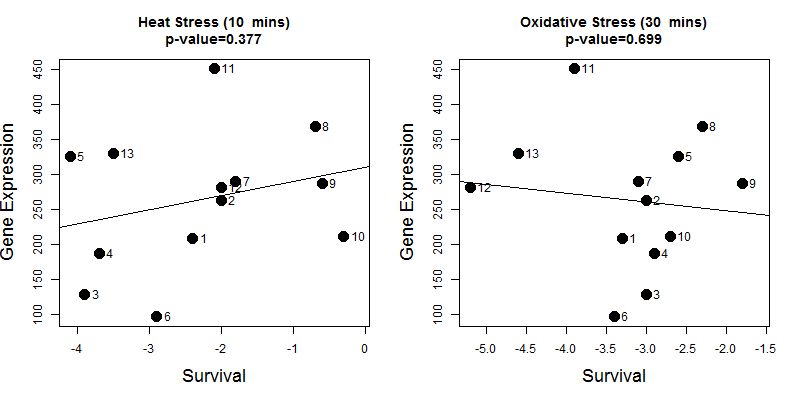

Supplement: S4 File — Expression levels of genes LLKF_1274 –LLKF_2533 and LLKF_p0001 –LLKF_p0036 plotted against survival after 10 minutes heat and 30 minutes oxidative stress. Survival is expressed as the difference of log CFU/ml after stress and before stress. Numbers indicate fermentations as presented in Table 1. P-values above the plots indicate significance of correlation (assessed by a linear model). (ZIP) [file pone.0167944.s009.zip › S4_File/LLKF_1421_real_dat.png]

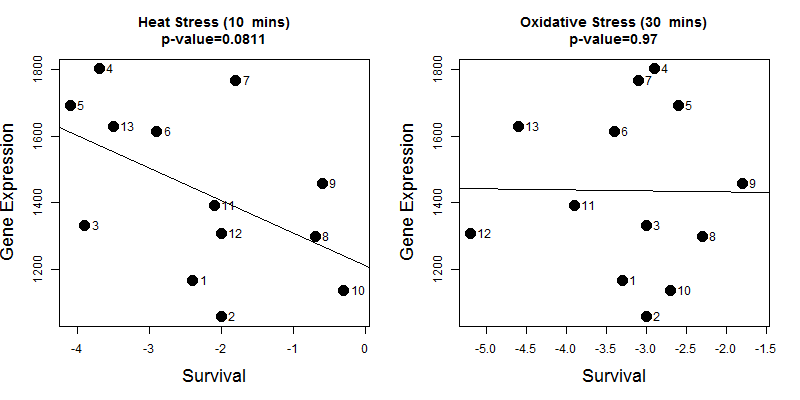

Supplement: S4 File — Expression levels of genes LLKF_1274 –LLKF_2533 and LLKF_p0001 –LLKF_p0036 plotted against survival after 10 minutes heat and 30 minutes oxidative stress. Survival is expressed as the difference of log CFU/ml after stress and before stress. Numbers indicate fermentations as presented in Table 1. P-values above the plots indicate significance of correlation (assessed by a linear model). (ZIP) [file pone.0167944.s009.zip › S4_File/LLKF_1422_real_dat.png]

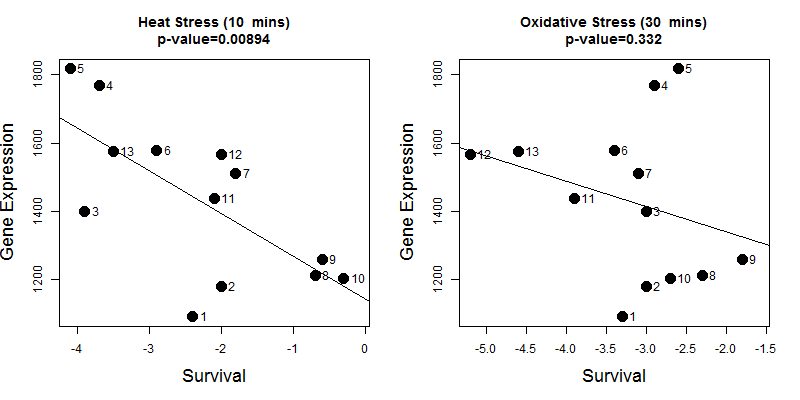

Supplement: S4 File — Expression levels of genes LLKF_1274 –LLKF_2533 and LLKF_p0001 –LLKF_p0036 plotted against survival after 10 minutes heat and 30 minutes oxidative stress. Survival is expressed as the difference of log CFU/ml after stress and before stress. Numbers indicate fermentations as presented in Table 1. P-values above the plots indicate significance of correlation (assessed by a linear model). (ZIP) [file pone.0167944.s009.zip › S4_File/LLKF_1423_real_dat.png]

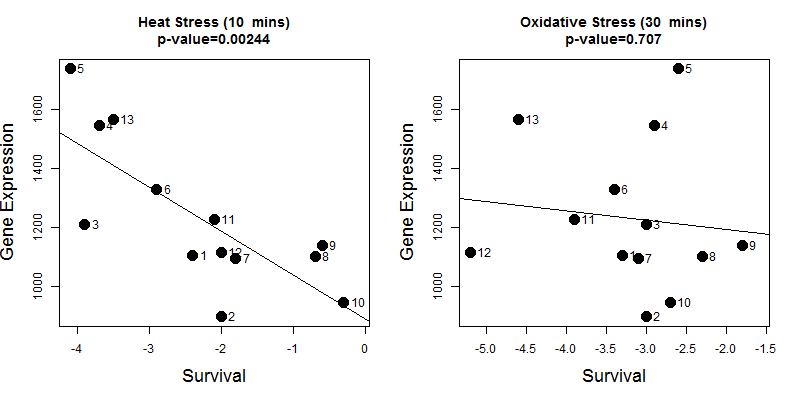

Supplement: S4 File — Expression levels of genes LLKF_1274 –LLKF_2533 and LLKF_p0001 –LLKF_p0036 plotted against survival after 10 minutes heat and 30 minutes oxidative stress. Survival is expressed as the difference of log CFU/ml after stress and before stress. Numbers indicate fermentations as presented in Table 1. P-values above the plots indicate significance of correlation (assessed by a linear model). (ZIP) [file pone.0167944.s009.zip › S4_File/LLKF_1424_real_dat.png]

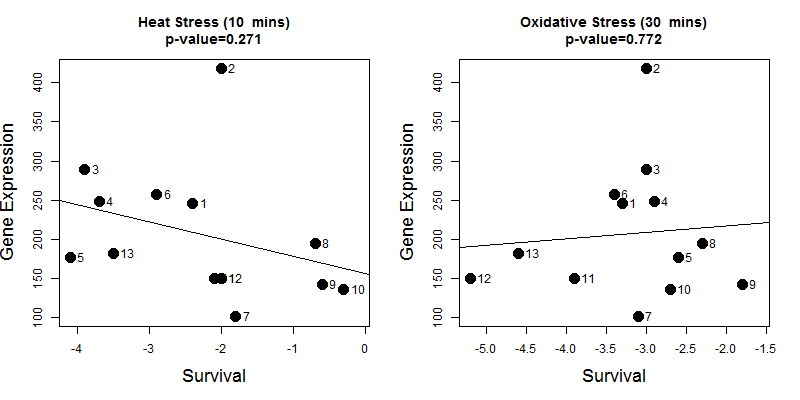

Supplement: S4 File — Expression levels of genes LLKF_1274 –LLKF_2533 and LLKF_p0001 –LLKF_p0036 plotted against survival after 10 minutes heat and 30 minutes oxidative stress. Survival is expressed as the difference of log CFU/ml after stress and before stress. Numbers indicate fermentations as presented in Table 1. P-values above the plots indicate significance of correlation (assessed by a linear model). (ZIP) [file pone.0167944.s009.zip › S4_File/LLKF_1425_real_dat.png]

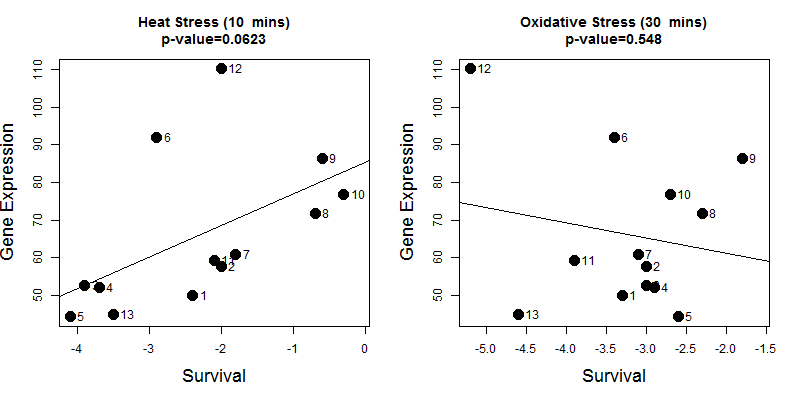

Supplement: S4 File — Expression levels of genes LLKF_1274 –LLKF_2533 and LLKF_p0001 –LLKF_p0036 plotted against survival after 10 minutes heat and 30 minutes oxidative stress. Survival is expressed as the difference of log CFU/ml after stress and before stress. Numbers indicate fermentations as presented in Table 1. P-values above the plots indicate significance of correlation (assessed by a linear model). (ZIP) [file pone.0167944.s009.zip › S4_File/LLKF_1426_real_dat.png]

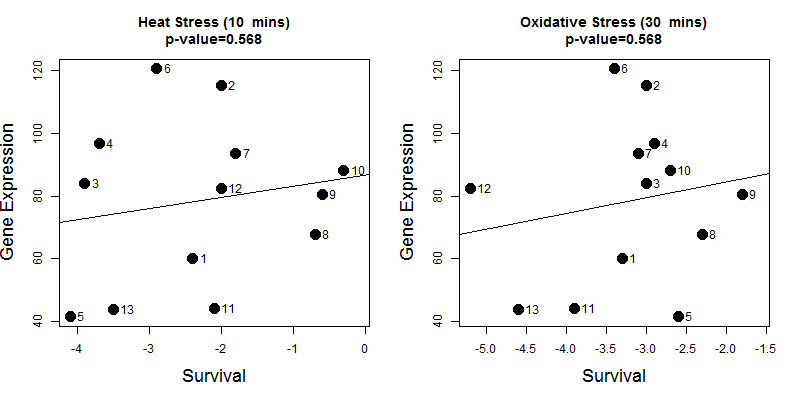

Supplement: S4 File — Expression levels of genes LLKF_1274 –LLKF_2533 and LLKF_p0001 –LLKF_p0036 plotted against survival after 10 minutes heat and 30 minutes oxidative stress. Survival is expressed as the difference of log CFU/ml after stress and before stress. Numbers indicate fermentations as presented in Table 1. P-values above the plots indicate significance of correlation (assessed by a linear model). (ZIP) [file pone.0167944.s009.zip › S4_File/LLKF_1427_real_dat.png]

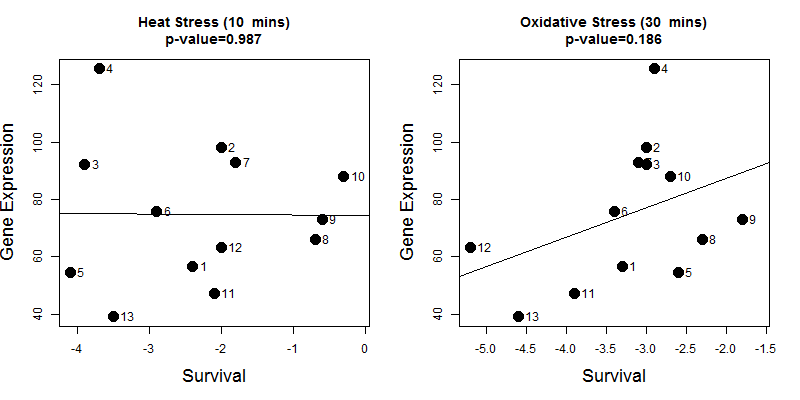

Supplement: S4 File — Expression levels of genes LLKF_1274 –LLKF_2533 and LLKF_p0001 –LLKF_p0036 plotted against survival after 10 minutes heat and 30 minutes oxidative stress. Survival is expressed as the difference of log CFU/ml after stress and before stress. Numbers indicate fermentations as presented in Table 1. P-values above the plots indicate significance of correlation (assessed by a linear model). (ZIP) [file pone.0167944.s009.zip › S4_File/LLKF_1428_real_dat.png]

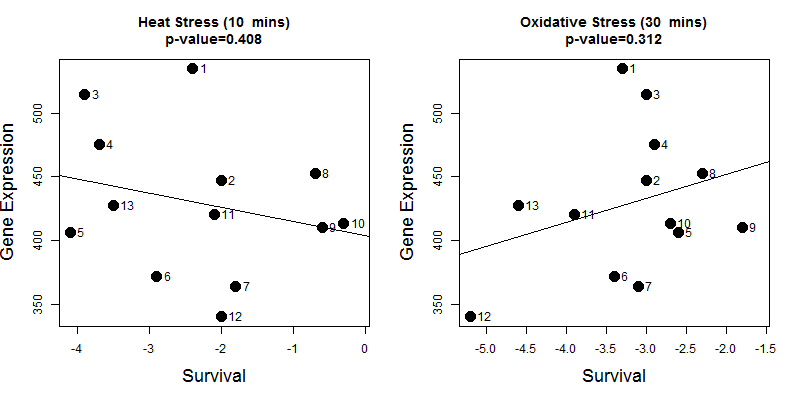

Supplement: S4 File — Expression levels of genes LLKF_1274 –LLKF_2533 and LLKF_p0001 –LLKF_p0036 plotted against survival after 10 minutes heat and 30 minutes oxidative stress. Survival is expressed as the difference of log CFU/ml after stress and before stress. Numbers indicate fermentations as presented in Table 1. P-values above the plots indicate significance of correlation (assessed by a linear model). (ZIP) [file pone.0167944.s009.zip › S4_File/LLKF_1429_real_dat.png]

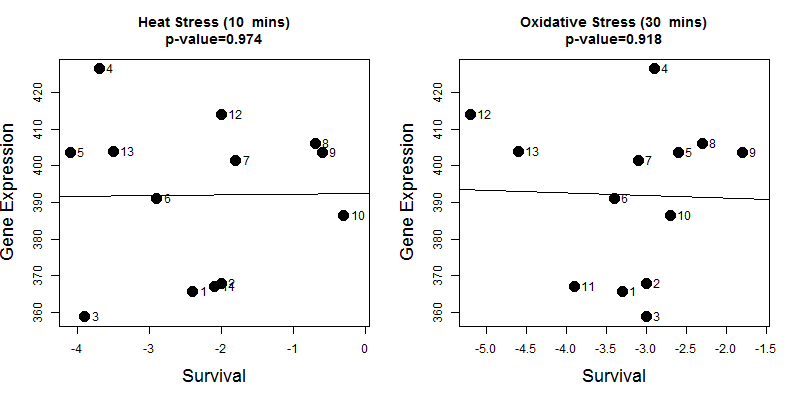

Supplement: S4 File — Expression levels of genes LLKF_1274 –LLKF_2533 and LLKF_p0001 –LLKF_p0036 plotted against survival after 10 minutes heat and 30 minutes oxidative stress. Survival is expressed as the difference of log CFU/ml after stress and before stress. Numbers indicate fermentations as presented in Table 1. P-values above the plots indicate significance of correlation (assessed by a linear model). (ZIP) [file pone.0167944.s009.zip › S4_File/LLKF_1430_real_dat.png]

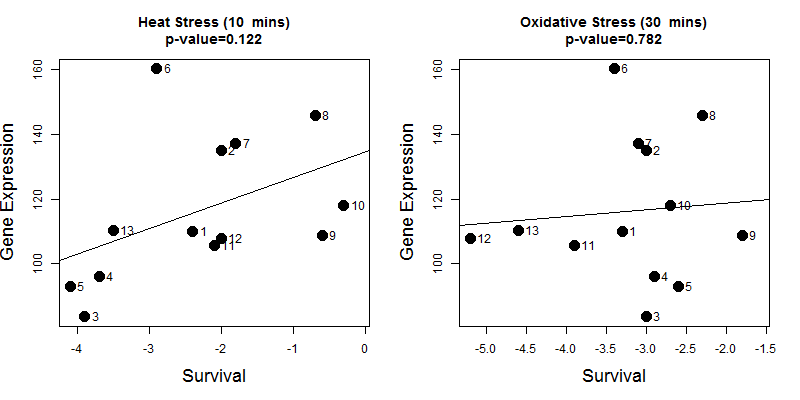

Supplement: S4 File — Expression levels of genes LLKF_1274 –LLKF_2533 and LLKF_p0001 –LLKF_p0036 plotted against survival after 10 minutes heat and 30 minutes oxidative stress. Survival is expressed as the difference of log CFU/ml after stress and before stress. Numbers indicate fermentations as presented in Table 1. P-values above the plots indicate significance of correlation (assessed by a linear model). (ZIP) [file pone.0167944.s009.zip › S4_File/LLKF_1431_real_dat.png]

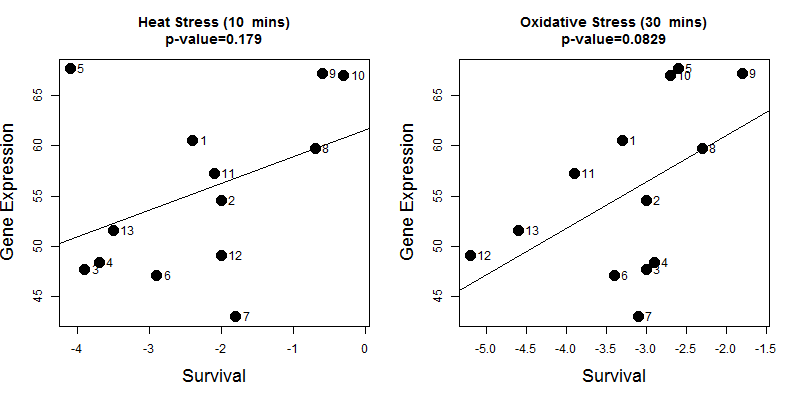

Supplement: S4 File — Expression levels of genes LLKF_1274 –LLKF_2533 and LLKF_p0001 –LLKF_p0036 plotted against survival after 10 minutes heat and 30 minutes oxidative stress. Survival is expressed as the difference of log CFU/ml after stress and before stress. Numbers indicate fermentations as presented in Table 1. P-values above the plots indicate significance of correlation (assessed by a linear model). (ZIP) [file pone.0167944.s009.zip › S4_File/LLKF_1432_real_dat.png]

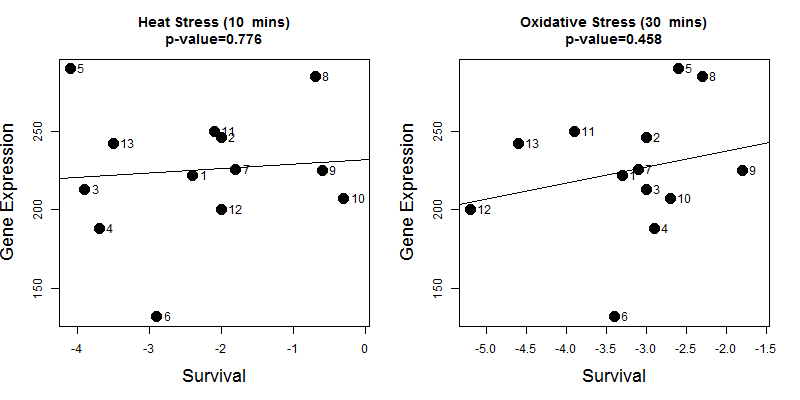

Supplement: S4 File — Expression levels of genes LLKF_1274 –LLKF_2533 and LLKF_p0001 –LLKF_p0036 plotted against survival after 10 minutes heat and 30 minutes oxidative stress. Survival is expressed as the difference of log CFU/ml after stress and before stress. Numbers indicate fermentations as presented in Table 1. P-values above the plots indicate significance of correlation (assessed by a linear model). (ZIP) [file pone.0167944.s009.zip › S4_File/LLKF_1433_real_dat.png]

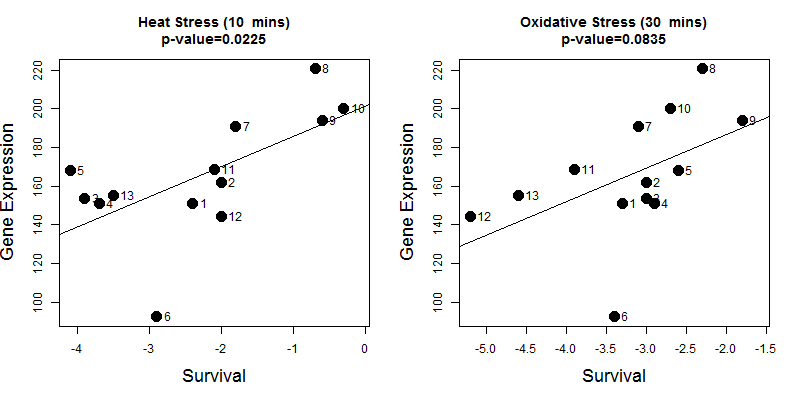

Supplement: S4 File — Expression levels of genes LLKF_1274 –LLKF_2533 and LLKF_p0001 –LLKF_p0036 plotted against survival after 10 minutes heat and 30 minutes oxidative stress. Survival is expressed as the difference of log CFU/ml after stress and before stress. Numbers indicate fermentations as presented in Table 1. P-values above the plots indicate significance of correlation (assessed by a linear model). (ZIP) [file pone.0167944.s009.zip › S4_File/LLKF_1434_real_dat.png]

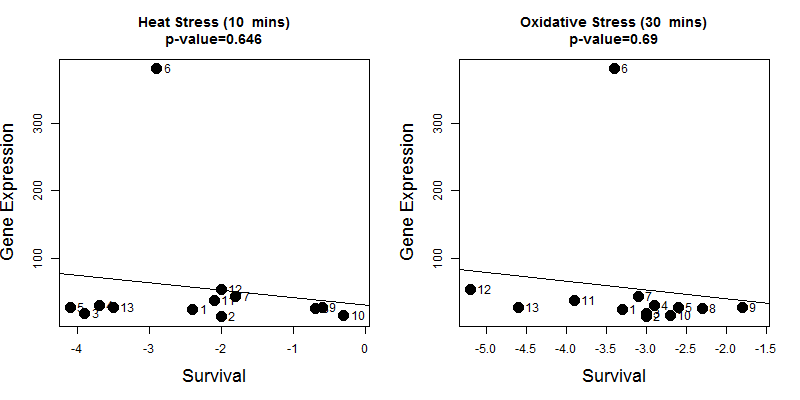

Supplement: S4 File — Expression levels of genes LLKF_1274 –LLKF_2533 and LLKF_p0001 –LLKF_p0036 plotted against survival after 10 minutes heat and 30 minutes oxidative stress. Survival is expressed as the difference of log CFU/ml after stress and before stress. Numbers indicate fermentations as presented in Table 1. P-values above the plots indicate significance of correlation (assessed by a linear model). (ZIP) [file pone.0167944.s009.zip › S4_File/LLKF_1435_real_dat.png]

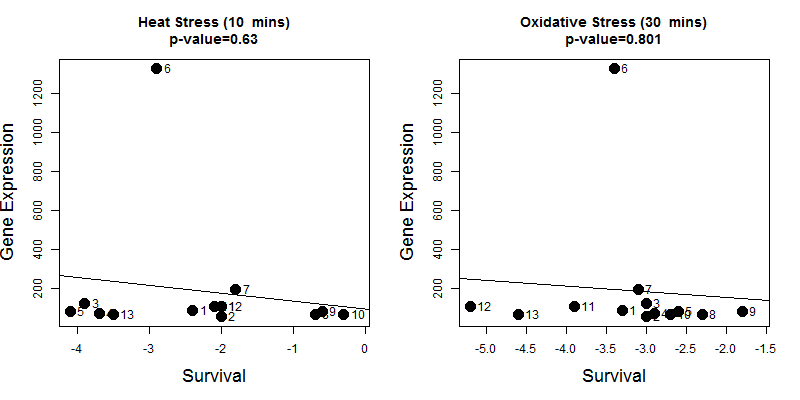

Supplement: S4 File — Expression levels of genes LLKF_1274 –LLKF_2533 and LLKF_p0001 –LLKF_p0036 plotted against survival after 10 minutes heat and 30 minutes oxidative stress. Survival is expressed as the difference of log CFU/ml after stress and before stress. Numbers indicate fermentations as presented in Table 1. P-values above the plots indicate significance of correlation (assessed by a linear model). (ZIP) [file pone.0167944.s009.zip › S4_File/LLKF_1436_real_dat.png]

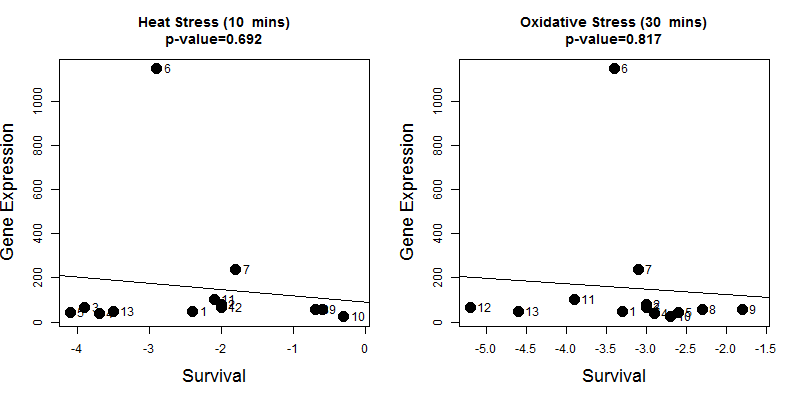

Supplement: S4 File — Expression levels of genes LLKF_1274 –LLKF_2533 and LLKF_p0001 –LLKF_p0036 plotted against survival after 10 minutes heat and 30 minutes oxidative stress. Survival is expressed as the difference of log CFU/ml after stress and before stress. Numbers indicate fermentations as presented in Table 1. P-values above the plots indicate significance of correlation (assessed by a linear model). (ZIP) [file pone.0167944.s009.zip › S4_File/LLKF_1437_real_dat.png]

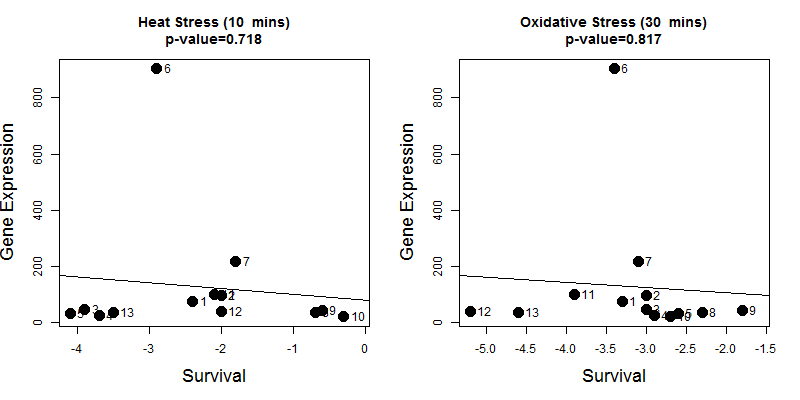

Supplement: S4 File — Expression levels of genes LLKF_1274 –LLKF_2533 and LLKF_p0001 –LLKF_p0036 plotted against survival after 10 minutes heat and 30 minutes oxidative stress. Survival is expressed as the difference of log CFU/ml after stress and before stress. Numbers indicate fermentations as presented in Table 1. P-values above the plots indicate significance of correlation (assessed by a linear model). (ZIP) [file pone.0167944.s009.zip › S4_File/LLKF_1438_real_dat.png]

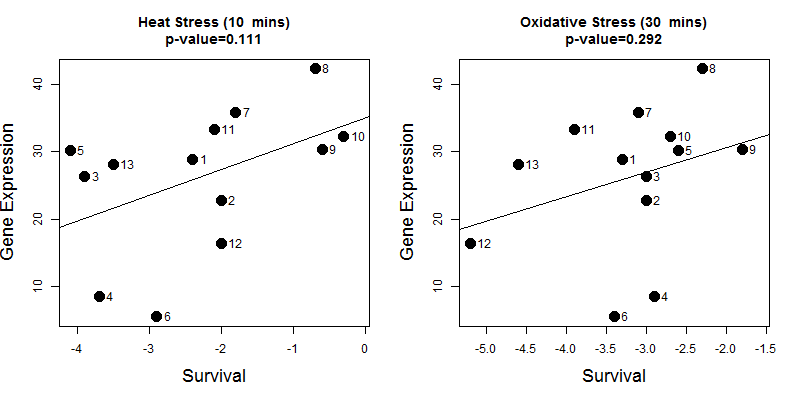

Supplement: S4 File — Expression levels of genes LLKF_1274 –LLKF_2533 and LLKF_p0001 –LLKF_p0036 plotted against survival after 10 minutes heat and 30 minutes oxidative stress. Survival is expressed as the difference of log CFU/ml after stress and before stress. Numbers indicate fermentations as presented in Table 1. P-values above the plots indicate significance of correlation (assessed by a linear model). (ZIP) [file pone.0167944.s009.zip › S4_File/LLKF_1439_real_dat.png]

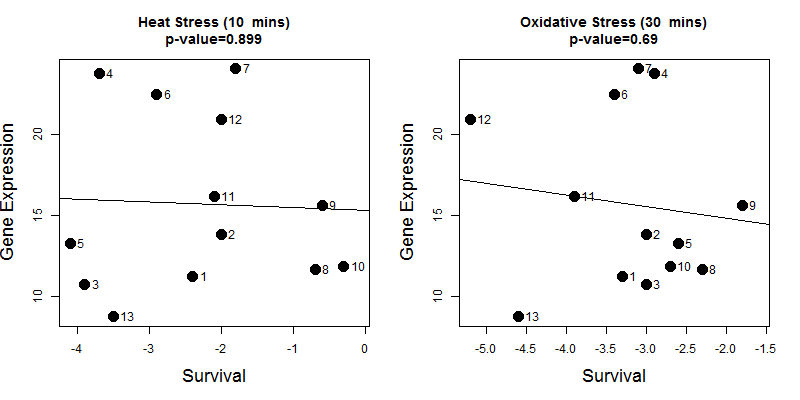

Supplement: S4 File — Expression levels of genes LLKF_1274 –LLKF_2533 and LLKF_p0001 –LLKF_p0036 plotted against survival after 10 minutes heat and 30 minutes oxidative stress. Survival is expressed as the difference of log CFU/ml after stress and before stress. Numbers indicate fermentations as presented in Table 1. P-values above the plots indicate significance of correlation (assessed by a linear model). (ZIP) [file pone.0167944.s009.zip › S4_File/LLKF_1440_real_dat.png]

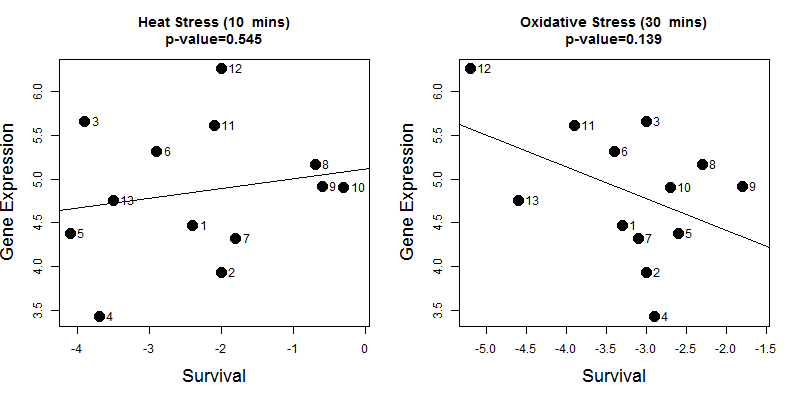

Supplement: S4 File — Expression levels of genes LLKF_1274 –LLKF_2533 and LLKF_p0001 –LLKF_p0036 plotted against survival after 10 minutes heat and 30 minutes oxidative stress. Survival is expressed as the difference of log CFU/ml after stress and before stress. Numbers indicate fermentations as presented in Table 1. P-values above the plots indicate significance of correlation (assessed by a linear model). (ZIP) [file pone.0167944.s009.zip › S4_File/LLKF_1441_real_dat.png]

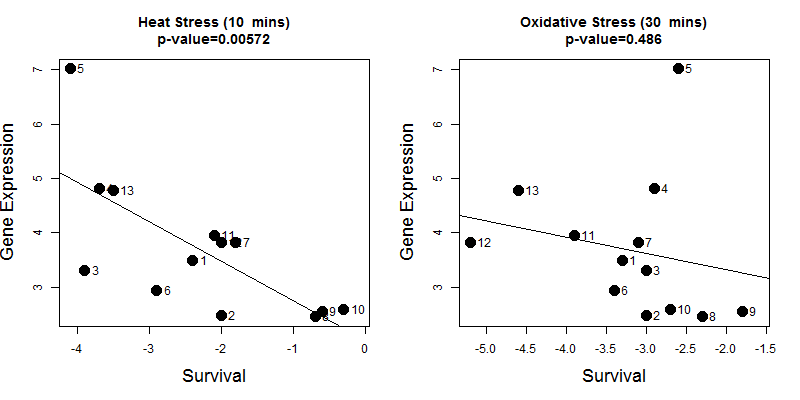

Supplement: S4 File — Expression levels of genes LLKF_1274 –LLKF_2533 and LLKF_p0001 –LLKF_p0036 plotted against survival after 10 minutes heat and 30 minutes oxidative stress. Survival is expressed as the difference of log CFU/ml after stress and before stress. Numbers indicate fermentations as presented in Table 1. P-values above the plots indicate significance of correlation (assessed by a linear model). (ZIP) [file pone.0167944.s009.zip › S4_File/LLKF_1442_real_dat.png]

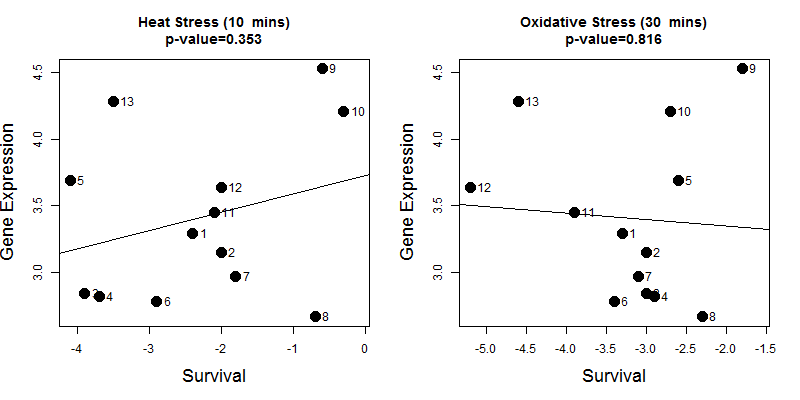

Supplement: S4 File — Expression levels of genes LLKF_1274 –LLKF_2533 and LLKF_p0001 –LLKF_p0036 plotted against survival after 10 minutes heat and 30 minutes oxidative stress. Survival is expressed as the difference of log CFU/ml after stress and before stress. Numbers indicate fermentations as presented in Table 1. P-values above the plots indicate significance of correlation (assessed by a linear model). (ZIP) [file pone.0167944.s009.zip › S4_File/LLKF_1443_real_dat.png]

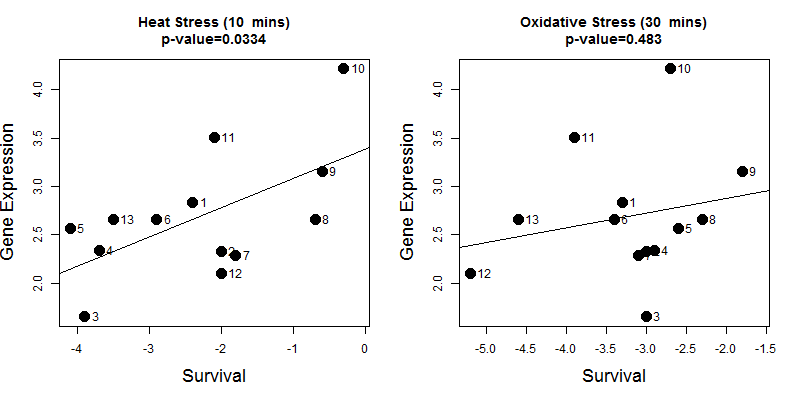

Supplement: S4 File — Expression levels of genes LLKF_1274 –LLKF_2533 and LLKF_p0001 –LLKF_p0036 plotted against survival after 10 minutes heat and 30 minutes oxidative stress. Survival is expressed as the difference of log CFU/ml after stress and before stress. Numbers indicate fermentations as presented in Table 1. P-values above the plots indicate significance of correlation (assessed by a linear model). (ZIP) [file pone.0167944.s009.zip › S4_File/LLKF_1444_real_dat.png]

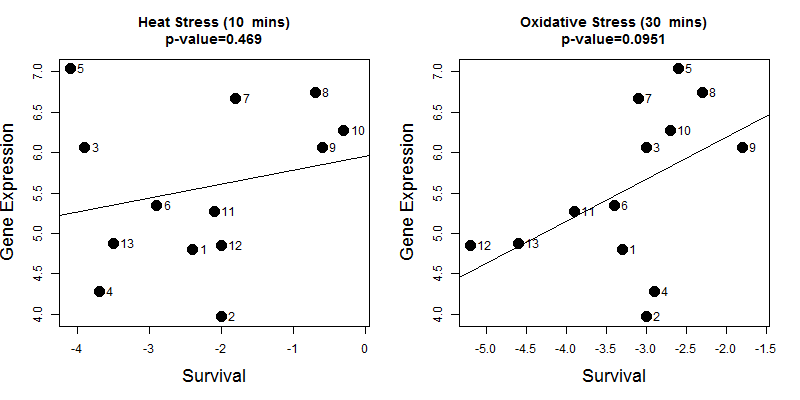

Supplement: S4 File — Expression levels of genes LLKF_1274 –LLKF_2533 and LLKF_p0001 –LLKF_p0036 plotted against survival after 10 minutes heat and 30 minutes oxidative stress. Survival is expressed as the difference of log CFU/ml after stress and before stress. Numbers indicate fermentations as presented in Table 1. P-values above the plots indicate significance of correlation (assessed by a linear model). (ZIP) [file pone.0167944.s009.zip › S4_File/LLKF_1445_real_dat.png]

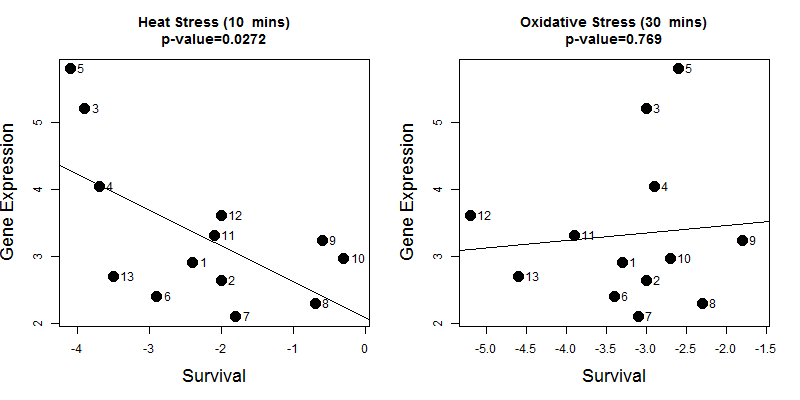

Supplement: S4 File — Expression levels of genes LLKF_1274 –LLKF_2533 and LLKF_p0001 –LLKF_p0036 plotted against survival after 10 minutes heat and 30 minutes oxidative stress. Survival is expressed as the difference of log CFU/ml after stress and before stress. Numbers indicate fermentations as presented in Table 1. P-values above the plots indicate significance of correlation (assessed by a linear model). (ZIP) [file pone.0167944.s009.zip › S4_File/LLKF_1446_real_dat.png]

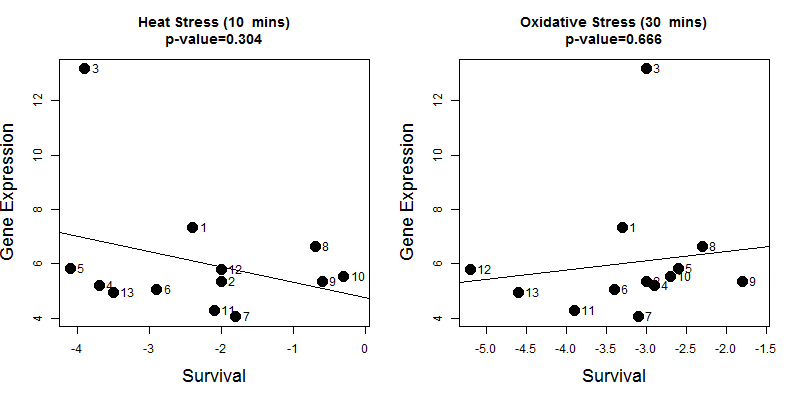

Supplement: S4 File — Expression levels of genes LLKF_1274 –LLKF_2533 and LLKF_p0001 –LLKF_p0036 plotted against survival after 10 minutes heat and 30 minutes oxidative stress. Survival is expressed as the difference of log CFU/ml after stress and before stress. Numbers indicate fermentations as presented in Table 1. P-values above the plots indicate significance of correlation (assessed by a linear model). (ZIP) [file pone.0167944.s009.zip › S4_File/LLKF_1447_real_dat.png]

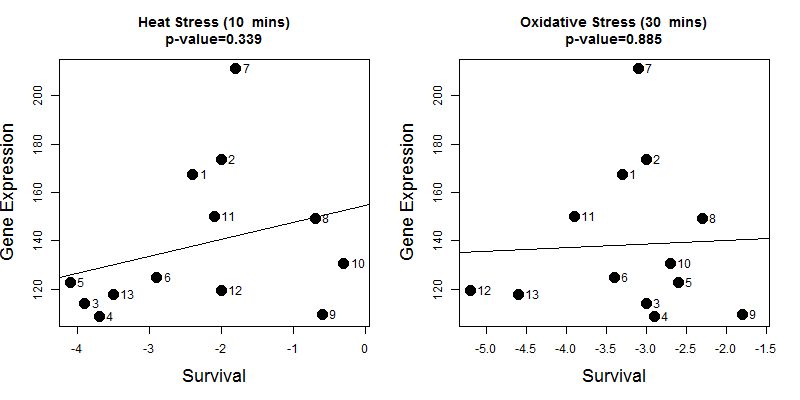

Supplement: S4 File — Expression levels of genes LLKF_1274 –LLKF_2533 and LLKF_p0001 –LLKF_p0036 plotted against survival after 10 minutes heat and 30 minutes oxidative stress. Survival is expressed as the difference of log CFU/ml after stress and before stress. Numbers indicate fermentations as presented in Table 1. P-values above the plots indicate significance of correlation (assessed by a linear model). (ZIP) [file pone.0167944.s009.zip › S4_File/LLKF_1448_real_dat.png]

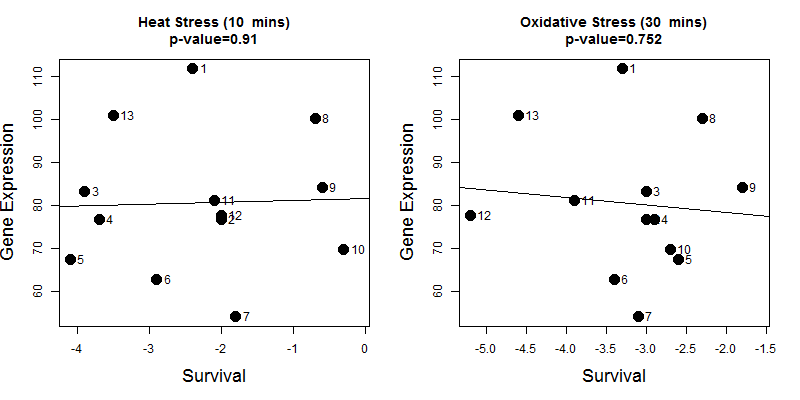

Supplement: S4 File — Expression levels of genes LLKF_1274 –LLKF_2533 and LLKF_p0001 –LLKF_p0036 plotted against survival after 10 minutes heat and 30 minutes oxidative stress. Survival is expressed as the difference of log CFU/ml after stress and before stress. Numbers indicate fermentations as presented in Table 1. P-values above the plots indicate significance of correlation (assessed by a linear model). (ZIP) [file pone.0167944.s009.zip › S4_File/LLKF_1449_real_dat.png]

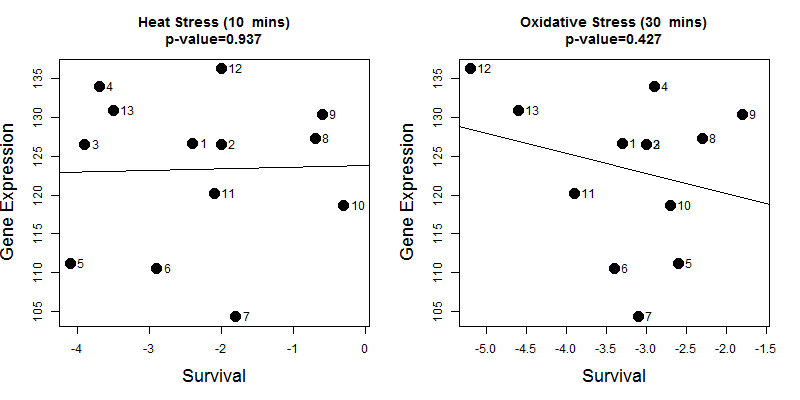

Supplement: S4 File — Expression levels of genes LLKF_1274 –LLKF_2533 and LLKF_p0001 –LLKF_p0036 plotted against survival after 10 minutes heat and 30 minutes oxidative stress. Survival is expressed as the difference of log CFU/ml after stress and before stress. Numbers indicate fermentations as presented in Table 1. P-values above the plots indicate significance of correlation (assessed by a linear model). (ZIP) [file pone.0167944.s009.zip › S4_File/LLKF_1450_real_dat.png]

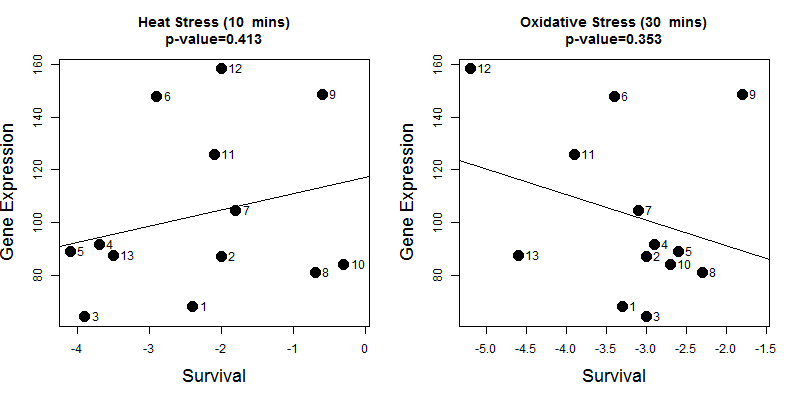

Supplement: S4 File — Expression levels of genes LLKF_1274 –LLKF_2533 and LLKF_p0001 –LLKF_p0036 plotted against survival after 10 minutes heat and 30 minutes oxidative stress. Survival is expressed as the difference of log CFU/ml after stress and before stress. Numbers indicate fermentations as presented in Table 1. P-values above the plots indicate significance of correlation (assessed by a linear model). (ZIP) [file pone.0167944.s009.zip › S4_File/LLKF_1451_real_dat.png]

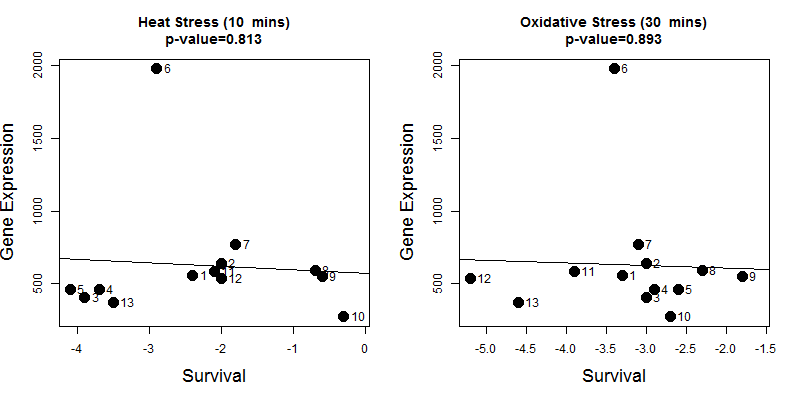

Supplement: S4 File — Expression levels of genes LLKF_1274 –LLKF_2533 and LLKF_p0001 –LLKF_p0036 plotted against survival after 10 minutes heat and 30 minutes oxidative stress. Survival is expressed as the difference of log CFU/ml after stress and before stress. Numbers indicate fermentations as presented in Table 1. P-values above the plots indicate significance of correlation (assessed by a linear model). (ZIP) [file pone.0167944.s009.zip › S4_File/LLKF_1452_real_dat.png]

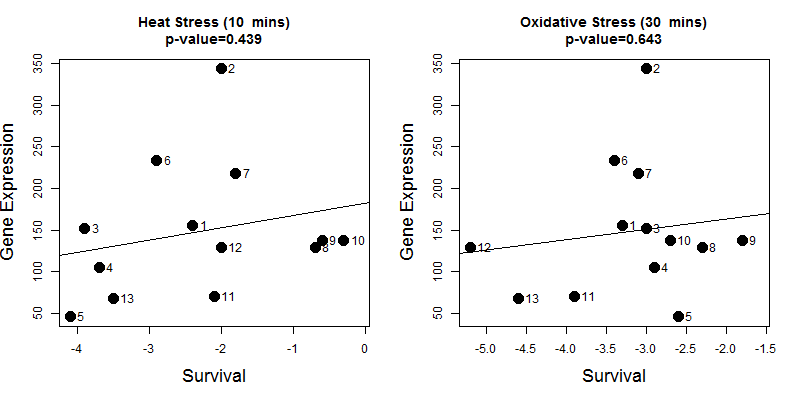

Supplement: S4 File — Expression levels of genes LLKF_1274 –LLKF_2533 and LLKF_p0001 –LLKF_p0036 plotted against survival after 10 minutes heat and 30 minutes oxidative stress. Survival is expressed as the difference of log CFU/ml after stress and before stress. Numbers indicate fermentations as presented in Table 1. P-values above the plots indicate significance of correlation (assessed by a linear model). (ZIP) [file pone.0167944.s009.zip › S4_File/LLKF_1453_real_dat.png]

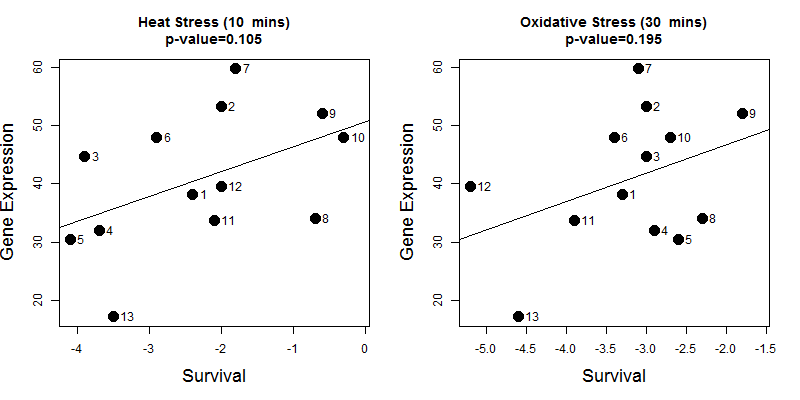

Supplement: S4 File — Expression levels of genes LLKF_1274 –LLKF_2533 and LLKF_p0001 –LLKF_p0036 plotted against survival after 10 minutes heat and 30 minutes oxidative stress. Survival is expressed as the difference of log CFU/ml after stress and before stress. Numbers indicate fermentations as presented in Table 1. P-values above the plots indicate significance of correlation (assessed by a linear model). (ZIP) [file pone.0167944.s009.zip › S4_File/LLKF_1454_real_dat.png]

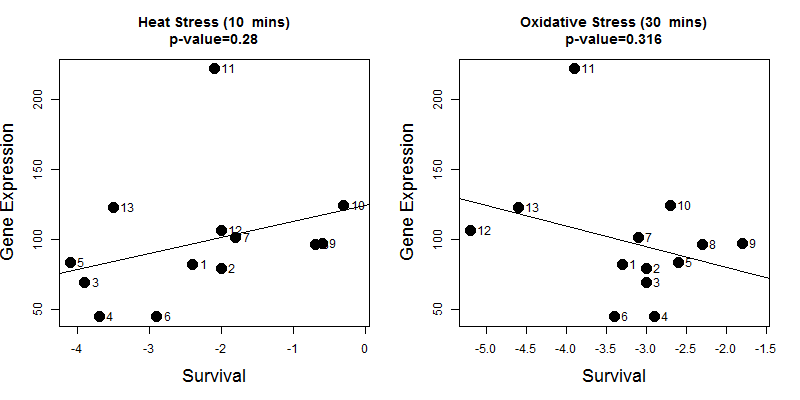

Supplement: S4 File — Expression levels of genes LLKF_1274 –LLKF_2533 and LLKF_p0001 –LLKF_p0036 plotted against survival after 10 minutes heat and 30 minutes oxidative stress. Survival is expressed as the difference of log CFU/ml after stress and before stress. Numbers indicate fermentations as presented in Table 1. P-values above the plots indicate significance of correlation (assessed by a linear model). (ZIP) [file pone.0167944.s009.zip › S4_File/LLKF_1455_real_dat.png]

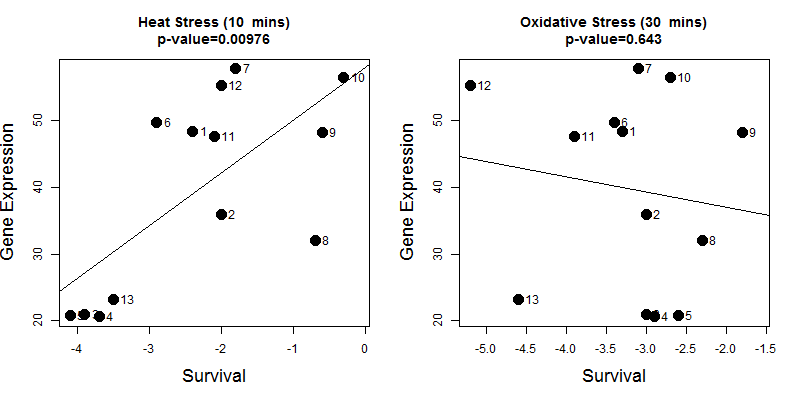

Supplement: S4 File — Expression levels of genes LLKF_1274 –LLKF_2533 and LLKF_p0001 –LLKF_p0036 plotted against survival after 10 minutes heat and 30 minutes oxidative stress. Survival is expressed as the difference of log CFU/ml after stress and before stress. Numbers indicate fermentations as presented in Table 1. P-values above the plots indicate significance of correlation (assessed by a linear model). (ZIP) [file pone.0167944.s009.zip › S4_File/LLKF_1456_real_dat.png]

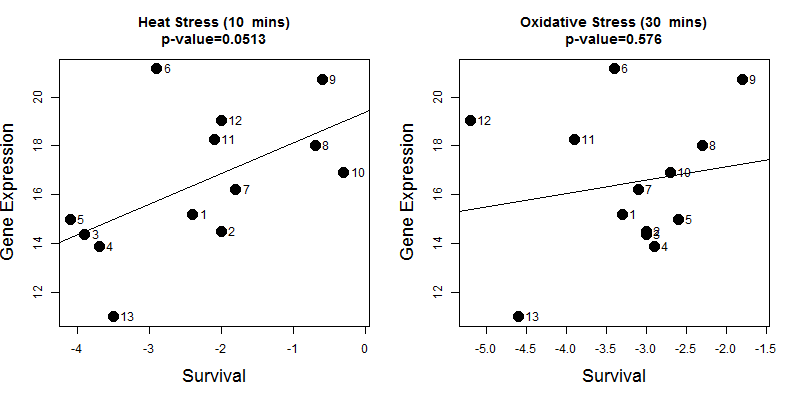

Supplement: S4 File — Expression levels of genes LLKF_1274 –LLKF_2533 and LLKF_p0001 –LLKF_p0036 plotted against survival after 10 minutes heat and 30 minutes oxidative stress. Survival is expressed as the difference of log CFU/ml after stress and before stress. Numbers indicate fermentations as presented in Table 1. P-values above the plots indicate significance of correlation (assessed by a linear model). (ZIP) [file pone.0167944.s009.zip › S4_File/LLKF_1457_real_dat.png]

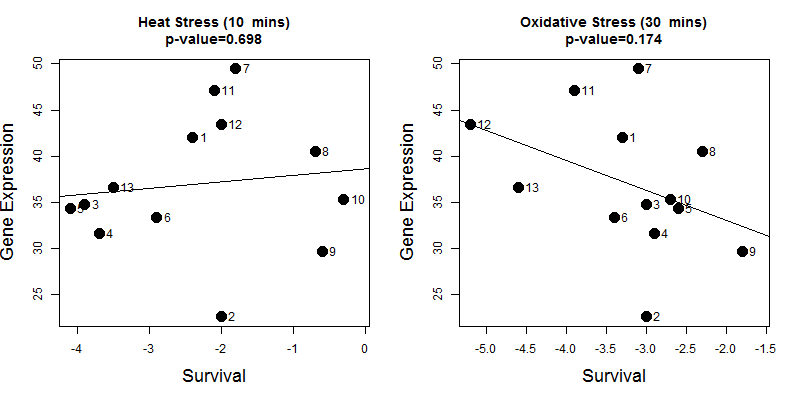

Supplement: S4 File — Expression levels of genes LLKF_1274 –LLKF_2533 and LLKF_p0001 –LLKF_p0036 plotted against survival after 10 minutes heat and 30 minutes oxidative stress. Survival is expressed as the difference of log CFU/ml after stress and before stress. Numbers indicate fermentations as presented in Table 1. P-values above the plots indicate significance of correlation (assessed by a linear model). (ZIP) [file pone.0167944.s009.zip › S4_File/LLKF_1460_real_dat.png]

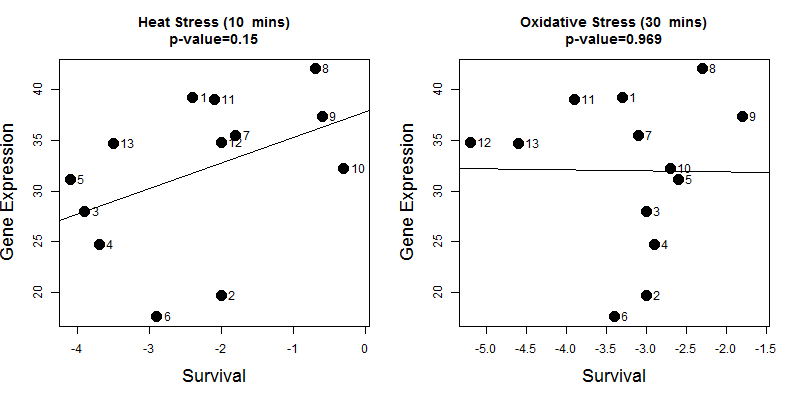

Supplement: S4 File — Expression levels of genes LLKF_1274 –LLKF_2533 and LLKF_p0001 –LLKF_p0036 plotted against survival after 10 minutes heat and 30 minutes oxidative stress. Survival is expressed as the difference of log CFU/ml after stress and before stress. Numbers indicate fermentations as presented in Table 1. P-values above the plots indicate significance of correlation (assessed by a linear model). (ZIP) [file pone.0167944.s009.zip › S4_File/LLKF_1461_real_dat.png]

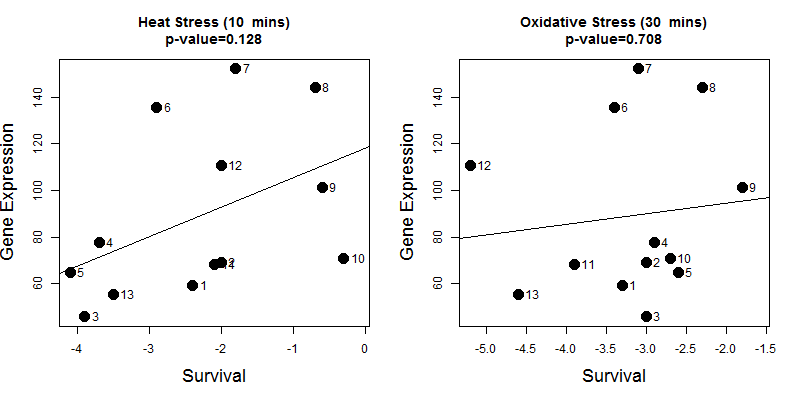

Supplement: S4 File — Expression levels of genes LLKF_1274 –LLKF_2533 and LLKF_p0001 –LLKF_p0036 plotted against survival after 10 minutes heat and 30 minutes oxidative stress. Survival is expressed as the difference of log CFU/ml after stress and before stress. Numbers indicate fermentations as presented in Table 1. P-values above the plots indicate significance of correlation (assessed by a linear model). (ZIP) [file pone.0167944.s009.zip › S4_File/LLKF_1462_real_dat.png]

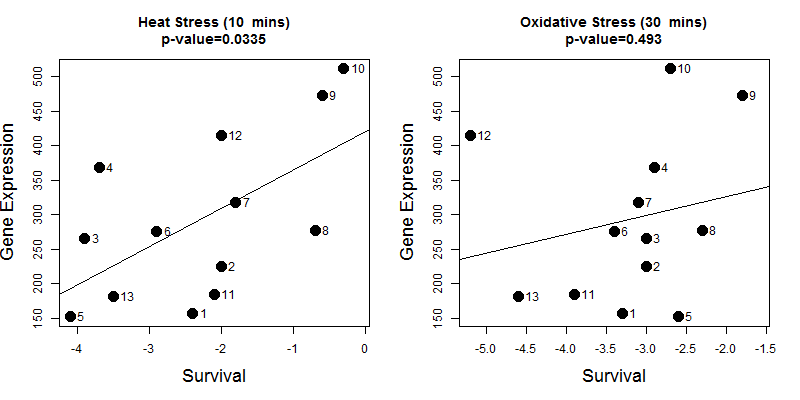

Supplement: S4 File — Expression levels of genes LLKF_1274 –LLKF_2533 and LLKF_p0001 –LLKF_p0036 plotted against survival after 10 minutes heat and 30 minutes oxidative stress. Survival is expressed as the difference of log CFU/ml after stress and before stress. Numbers indicate fermentations as presented in Table 1. P-values above the plots indicate significance of correlation (assessed by a linear model). (ZIP) [file pone.0167944.s009.zip › S4_File/LLKF_1463_real_dat.png]

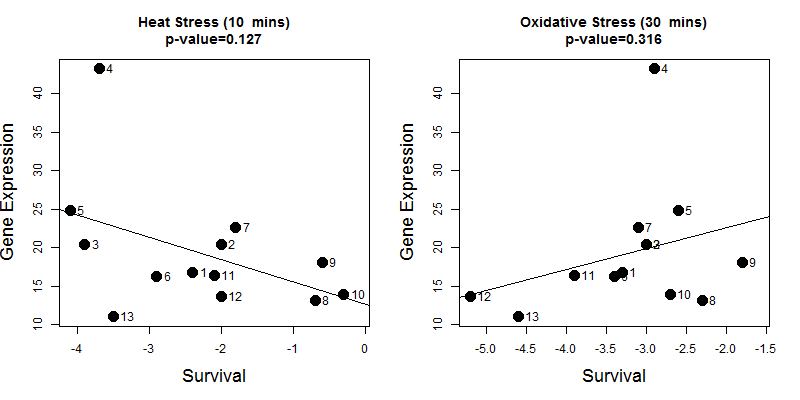

Supplement: S4 File — Expression levels of genes LLKF_1274 –LLKF_2533 and LLKF_p0001 –LLKF_p0036 plotted against survival after 10 minutes heat and 30 minutes oxidative stress. Survival is expressed as the difference of log CFU/ml after stress and before stress. Numbers indicate fermentations as presented in Table 1. P-values above the plots indicate significance of correlation (assessed by a linear model). (ZIP) [file pone.0167944.s009.zip › S4_File/LLKF_1464_real_dat.png]

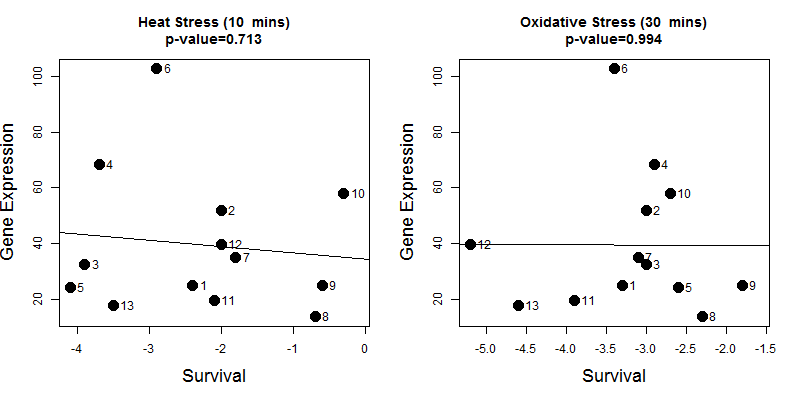

Supplement: S4 File — Expression levels of genes LLKF_1274 –LLKF_2533 and LLKF_p0001 –LLKF_p0036 plotted against survival after 10 minutes heat and 30 minutes oxidative stress. Survival is expressed as the difference of log CFU/ml after stress and before stress. Numbers indicate fermentations as presented in Table 1. P-values above the plots indicate significance of correlation (assessed by a linear model). (ZIP) [file pone.0167944.s009.zip › S4_File/LLKF_1467_real_dat.png]

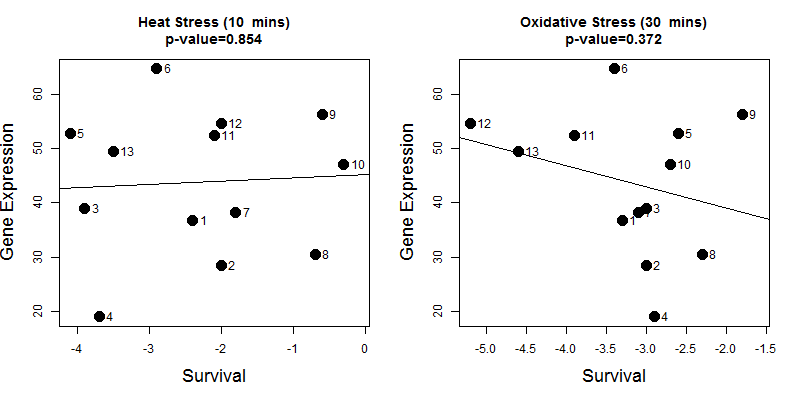

Supplement: S4 File — Expression levels of genes LLKF_1274 –LLKF_2533 and LLKF_p0001 –LLKF_p0036 plotted against survival after 10 minutes heat and 30 minutes oxidative stress. Survival is expressed as the difference of log CFU/ml after stress and before stress. Numbers indicate fermentations as presented in Table 1. P-values above the plots indicate significance of correlation (assessed by a linear model). (ZIP) [file pone.0167944.s009.zip › S4_File/LLKF_1468_real_dat.png]

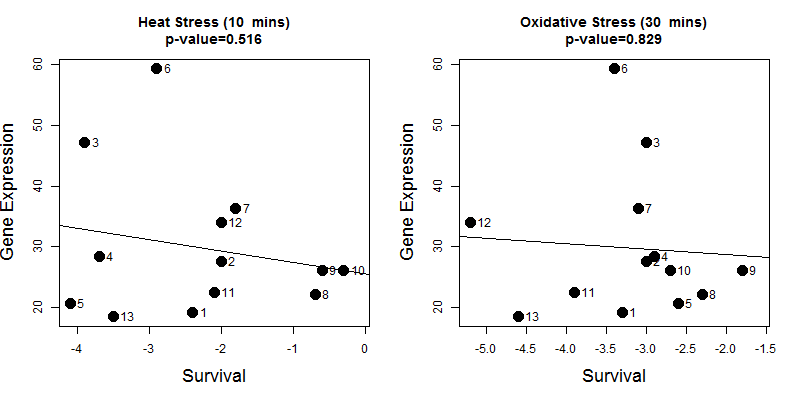

Supplement: S4 File — Expression levels of genes LLKF_1274 –LLKF_2533 and LLKF_p0001 –LLKF_p0036 plotted against survival after 10 minutes heat and 30 minutes oxidative stress. Survival is expressed as the difference of log CFU/ml after stress and before stress. Numbers indicate fermentations as presented in Table 1. P-values above the plots indicate significance of correlation (assessed by a linear model). (ZIP) [file pone.0167944.s009.zip › S4_File/LLKF_1469_real_dat.png]

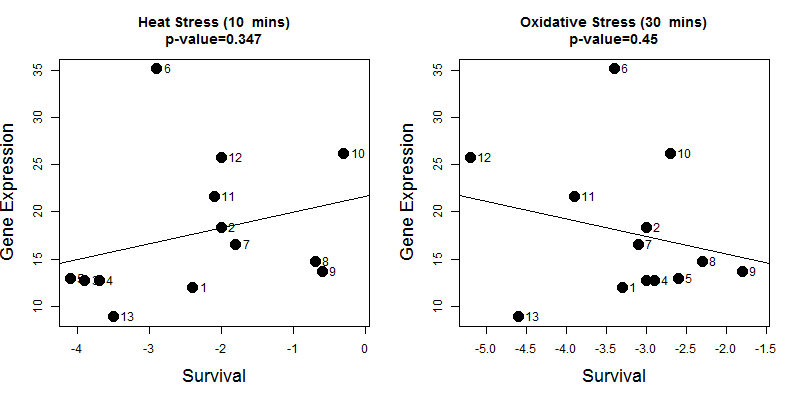

Supplement: S4 File — Expression levels of genes LLKF_1274 –LLKF_2533 and LLKF_p0001 –LLKF_p0036 plotted against survival after 10 minutes heat and 30 minutes oxidative stress. Survival is expressed as the difference of log CFU/ml after stress and before stress. Numbers indicate fermentations as presented in Table 1. P-values above the plots indicate significance of correlation (assessed by a linear model). (ZIP) [file pone.0167944.s009.zip › S4_File/LLKF_1470_real_dat.png]

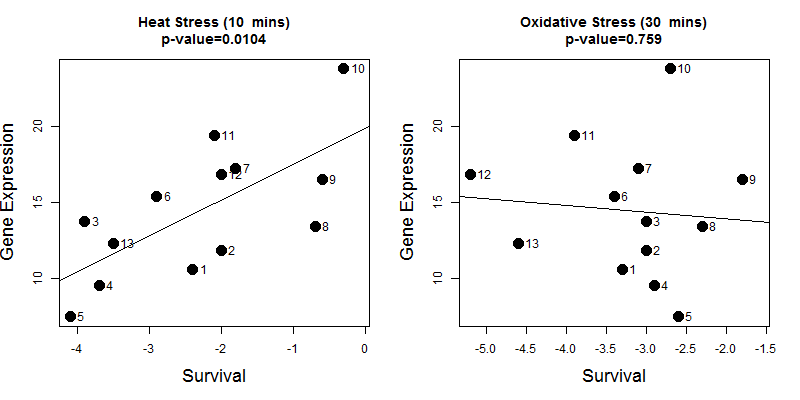

Supplement: S4 File — Expression levels of genes LLKF_1274 –LLKF_2533 and LLKF_p0001 –LLKF_p0036 plotted against survival after 10 minutes heat and 30 minutes oxidative stress. Survival is expressed as the difference of log CFU/ml after stress and before stress. Numbers indicate fermentations as presented in Table 1. P-values above the plots indicate significance of correlation (assessed by a linear model). (ZIP) [file pone.0167944.s009.zip › S4_File/LLKF_1471_real_dat.png]

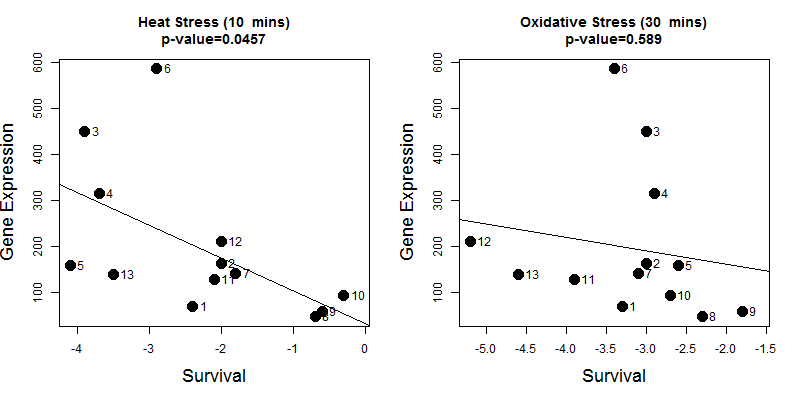

Supplement: S4 File — Expression levels of genes LLKF_1274 –LLKF_2533 and LLKF_p0001 –LLKF_p0036 plotted against survival after 10 minutes heat and 30 minutes oxidative stress. Survival is expressed as the difference of log CFU/ml after stress and before stress. Numbers indicate fermentations as presented in Table 1. P-values above the plots indicate significance of correlation (assessed by a linear model). (ZIP) [file pone.0167944.s009.zip › S4_File/LLKF_1472_real_dat.png]

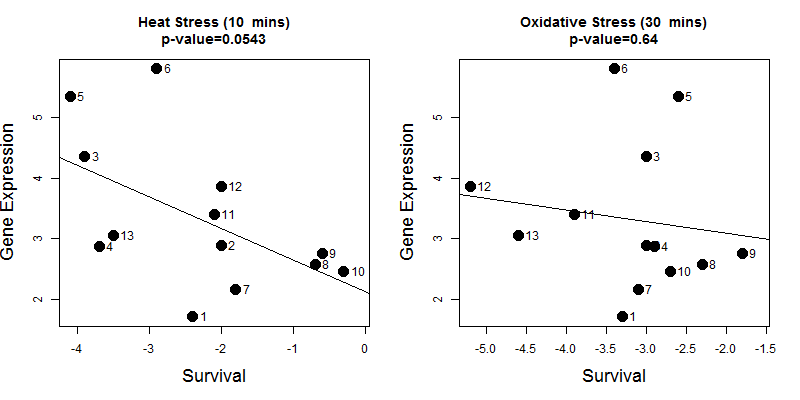

Supplement: S4 File — Expression levels of genes LLKF_1274 –LLKF_2533 and LLKF_p0001 –LLKF_p0036 plotted against survival after 10 minutes heat and 30 minutes oxidative stress. Survival is expressed as the difference of log CFU/ml after stress and before stress. Numbers indicate fermentations as presented in Table 1. P-values above the plots indicate significance of correlation (assessed by a linear model). (ZIP) [file pone.0167944.s009.zip › S4_File/LLKF_1473_real_dat.png]

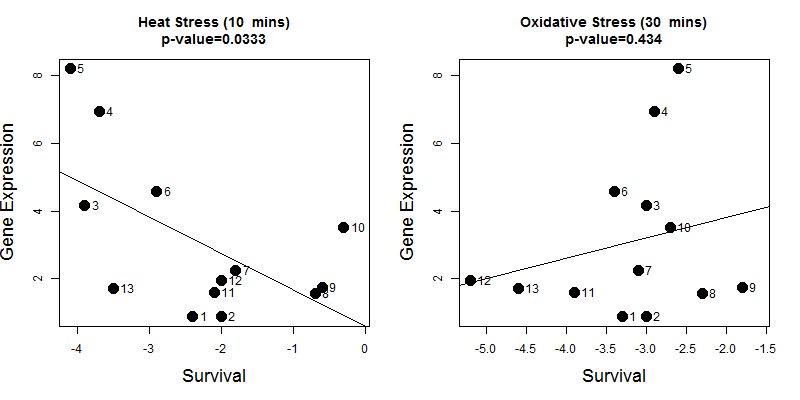

Supplement: S4 File — Expression levels of genes LLKF_1274 –LLKF_2533 and LLKF_p0001 –LLKF_p0036 plotted against survival after 10 minutes heat and 30 minutes oxidative stress. Survival is expressed as the difference of log CFU/ml after stress and before stress. Numbers indicate fermentations as presented in Table 1. P-values above the plots indicate significance of correlation (assessed by a linear model). (ZIP) [file pone.0167944.s009.zip › S4_File/LLKF_1474_real_dat.png]

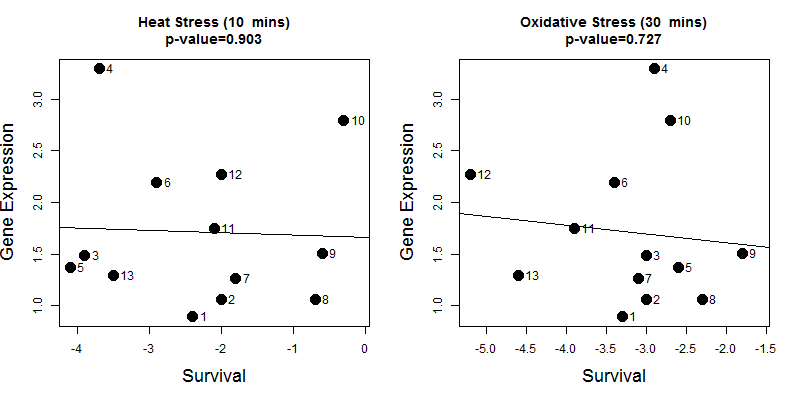

Supplement: S4 File — Expression levels of genes LLKF_1274 –LLKF_2533 and LLKF_p0001 –LLKF_p0036 plotted against survival after 10 minutes heat and 30 minutes oxidative stress. Survival is expressed as the difference of log CFU/ml after stress and before stress. Numbers indicate fermentations as presented in Table 1. P-values above the plots indicate significance of correlation (assessed by a linear model). (ZIP) [file pone.0167944.s009.zip › S4_File/LLKF_1475_real_dat.png]

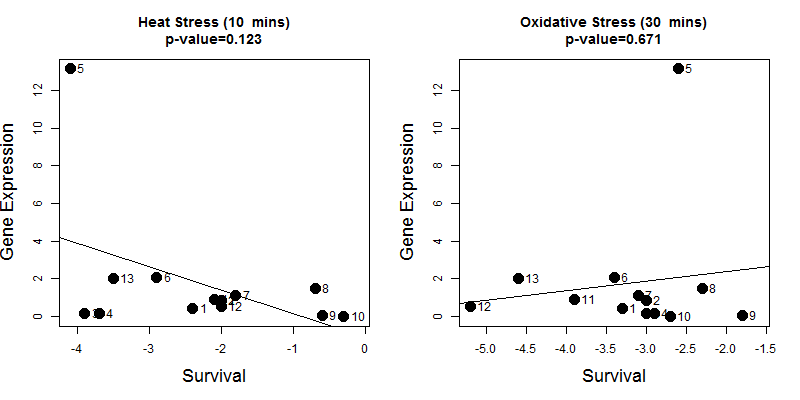

Supplement: S4 File — Expression levels of genes LLKF_1274 –LLKF_2533 and LLKF_p0001 –LLKF_p0036 plotted against survival after 10 minutes heat and 30 minutes oxidative stress. Survival is expressed as the difference of log CFU/ml after stress and before stress. Numbers indicate fermentations as presented in Table 1. P-values above the plots indicate significance of correlation (assessed by a linear model). (ZIP) [file pone.0167944.s009.zip › S4_File/LLKF_1476_real_dat.png]

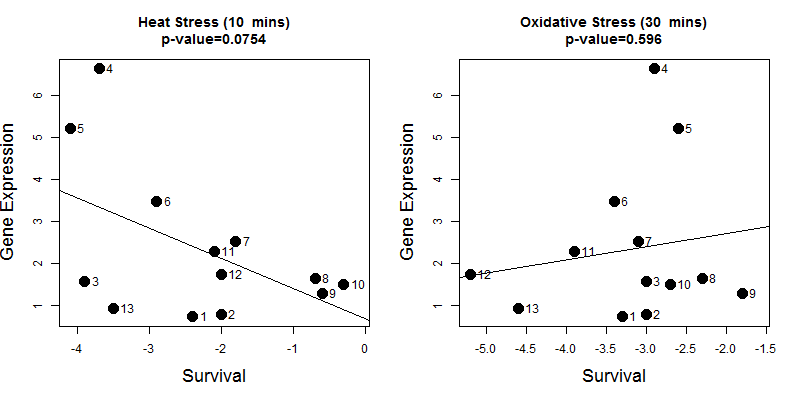

Supplement: S4 File — Expression levels of genes LLKF_1274 –LLKF_2533 and LLKF_p0001 –LLKF_p0036 plotted against survival after 10 minutes heat and 30 minutes oxidative stress. Survival is expressed as the difference of log CFU/ml after stress and before stress. Numbers indicate fermentations as presented in Table 1. P-values above the plots indicate significance of correlation (assessed by a linear model). (ZIP) [file pone.0167944.s009.zip › S4_File/LLKF_1477_real_dat.png]

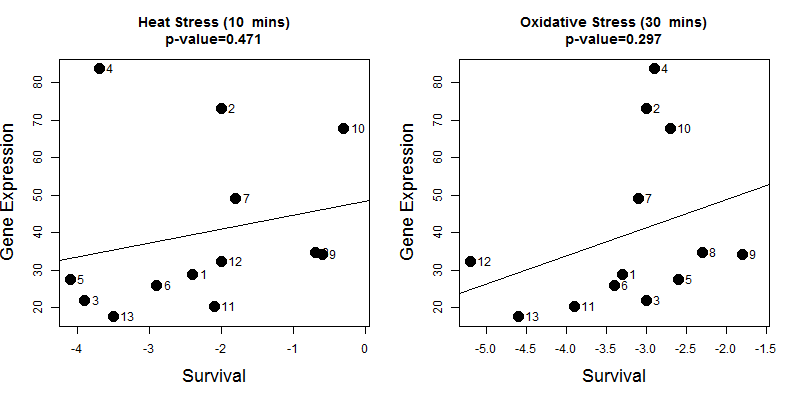

Supplement: S4 File — Expression levels of genes LLKF_1274 –LLKF_2533 and LLKF_p0001 –LLKF_p0036 plotted against survival after 10 minutes heat and 30 minutes oxidative stress. Survival is expressed as the difference of log CFU/ml after stress and before stress. Numbers indicate fermentations as presented in Table 1. P-values above the plots indicate significance of correlation (assessed by a linear model). (ZIP) [file pone.0167944.s009.zip › S4_File/LLKF_1478_real_dat.png]

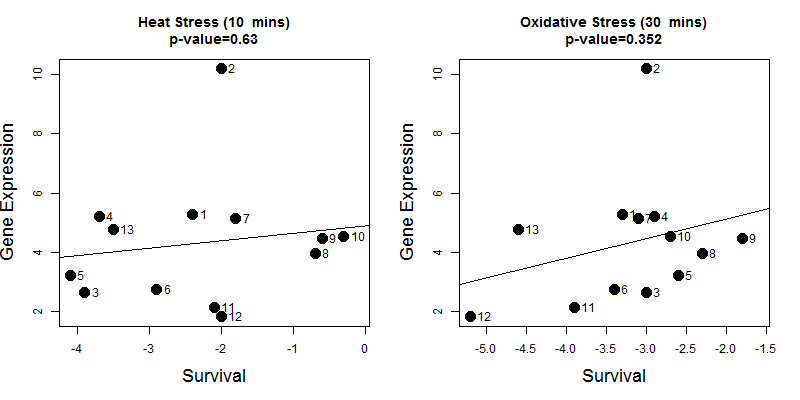

Supplement: S4 File — Expression levels of genes LLKF_1274 –LLKF_2533 and LLKF_p0001 –LLKF_p0036 plotted against survival after 10 minutes heat and 30 minutes oxidative stress. Survival is expressed as the difference of log CFU/ml after stress and before stress. Numbers indicate fermentations as presented in Table 1. P-values above the plots indicate significance of correlation (assessed by a linear model). (ZIP) [file pone.0167944.s009.zip › S4_File/LLKF_1479_real_dat.png]

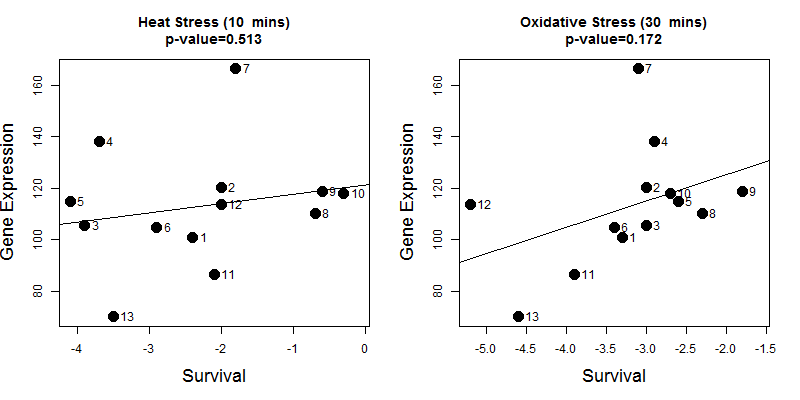

Supplement: S4 File — Expression levels of genes LLKF_1274 –LLKF_2533 and LLKF_p0001 –LLKF_p0036 plotted against survival after 10 minutes heat and 30 minutes oxidative stress. Survival is expressed as the difference of log CFU/ml after stress and before stress. Numbers indicate fermentations as presented in Table 1. P-values above the plots indicate significance of correlation (assessed by a linear model). (ZIP) [file pone.0167944.s009.zip › S4_File/LLKF_1480_real_dat.png]

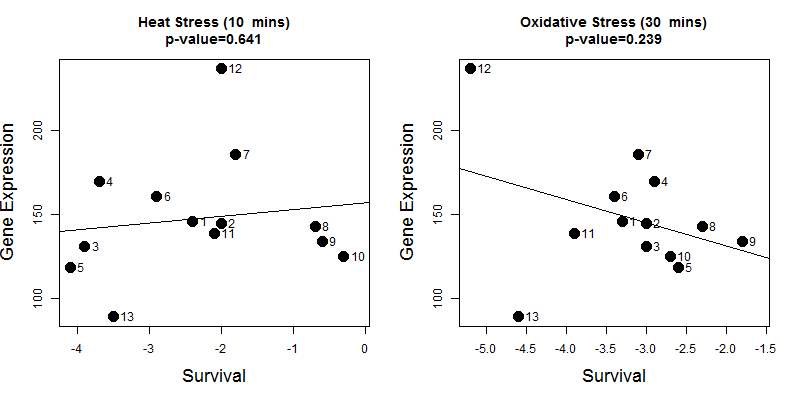

Supplement: S4 File — Expression levels of genes LLKF_1274 –LLKF_2533 and LLKF_p0001 –LLKF_p0036 plotted against survival after 10 minutes heat and 30 minutes oxidative stress. Survival is expressed as the difference of log CFU/ml after stress and before stress. Numbers indicate fermentations as presented in Table 1. P-values above the plots indicate significance of correlation (assessed by a linear model). (ZIP) [file pone.0167944.s009.zip › S4_File/LLKF_1481_real_dat.png]

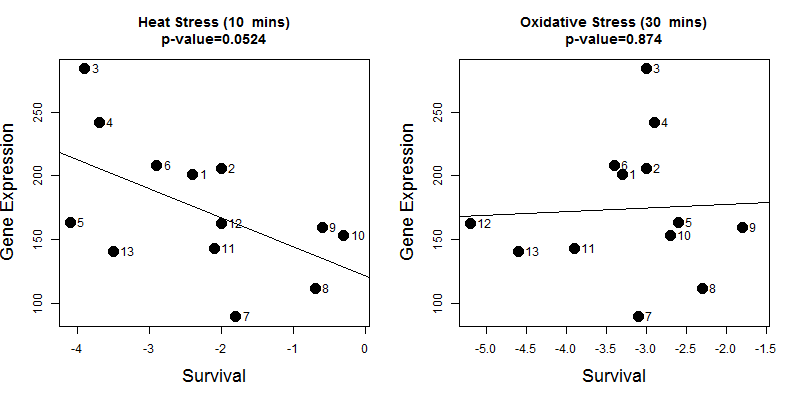

Supplement: S4 File — Expression levels of genes LLKF_1274 –LLKF_2533 and LLKF_p0001 –LLKF_p0036 plotted against survival after 10 minutes heat and 30 minutes oxidative stress. Survival is expressed as the difference of log CFU/ml after stress and before stress. Numbers indicate fermentations as presented in Table 1. P-values above the plots indicate significance of correlation (assessed by a linear model). (ZIP) [file pone.0167944.s009.zip › S4_File/LLKF_1482_real_dat.png]

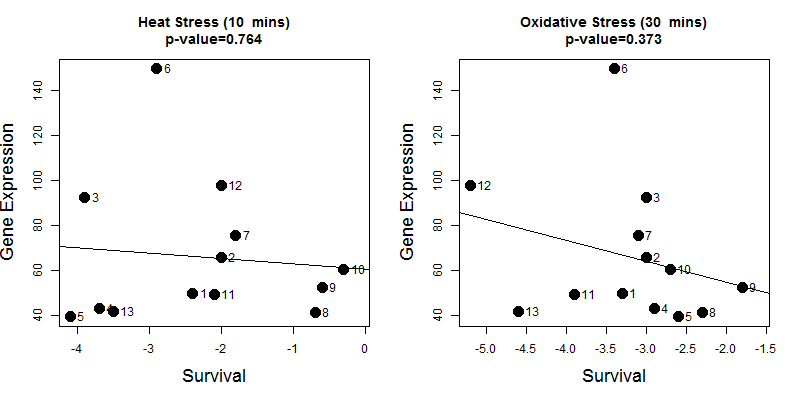

Supplement: S4 File — Expression levels of genes LLKF_1274 –LLKF_2533 and LLKF_p0001 –LLKF_p0036 plotted against survival after 10 minutes heat and 30 minutes oxidative stress. Survival is expressed as the difference of log CFU/ml after stress and before stress. Numbers indicate fermentations as presented in Table 1. P-values above the plots indicate significance of correlation (assessed by a linear model). (ZIP) [file pone.0167944.s009.zip › S4_File/LLKF_1483_real_dat.png]
