# Supplementary figures and images for: Strain-Dependent Transcriptome Signatures for Robustness in Lactococcus lactis (part 11 of 13)
Source: PLoS One. 2016 Dec 14;11(12):e0167944. doi: 10.1371/journal.pone.0167944 (PMC5156439; doi:10.1371/journal.pone.0167944)

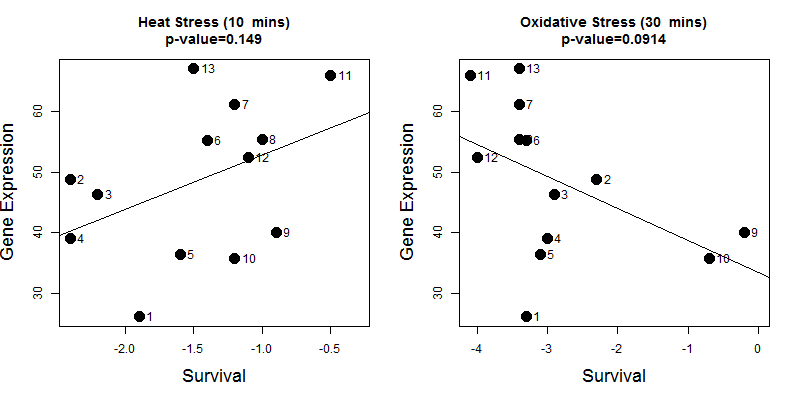

Supplement: S5 File — Expression levels of genes LACR_0001 –LACR_1382 plotted against survival after 10 minutes heat and 30 minutes oxidative stress. Survival is expressed as the difference of log CFU/ml after stress and before stress. Numbers indicate fermentations as presented in Table 1. P-values above the plots indicate significance of correlation (assessed by a linear model). (ZIP) [file pone.0167944.s010.zip › S5_File/LACR_0223_real_dat.png]

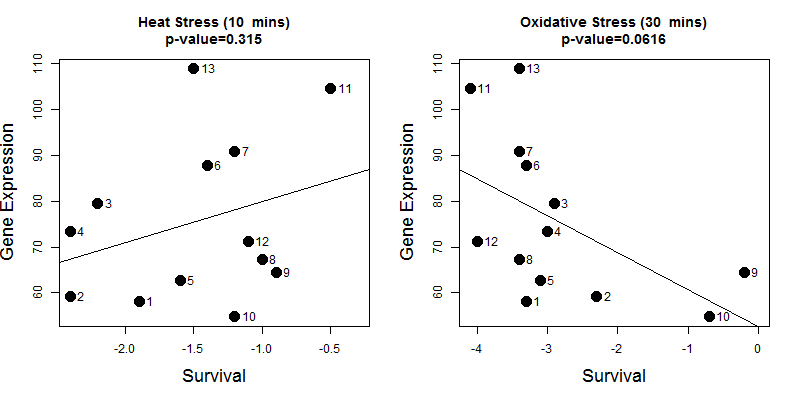

Supplement: S5 File — Expression levels of genes LACR_0001 –LACR_1382 plotted against survival after 10 minutes heat and 30 minutes oxidative stress. Survival is expressed as the difference of log CFU/ml after stress and before stress. Numbers indicate fermentations as presented in Table 1. P-values above the plots indicate significance of correlation (assessed by a linear model). (ZIP) [file pone.0167944.s010.zip › S5_File/LACR_0224_real_dat.png]

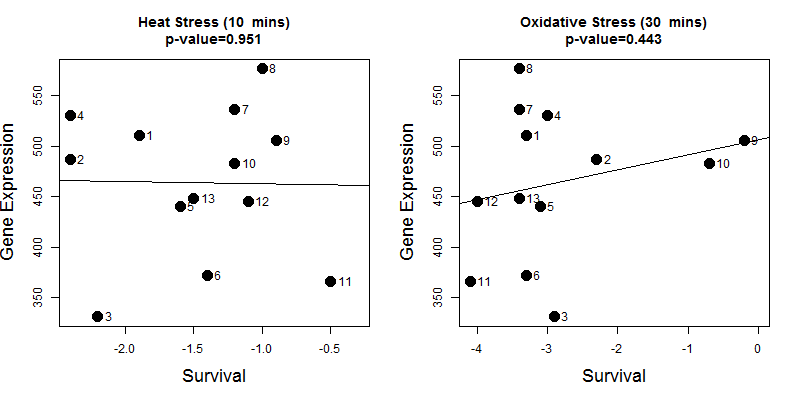

Supplement: S5 File — Expression levels of genes LACR_0001 –LACR_1382 plotted against survival after 10 minutes heat and 30 minutes oxidative stress. Survival is expressed as the difference of log CFU/ml after stress and before stress. Numbers indicate fermentations as presented in Table 1. P-values above the plots indicate significance of correlation (assessed by a linear model). (ZIP) [file pone.0167944.s010.zip › S5_File/LACR_0225_real_dat.png]

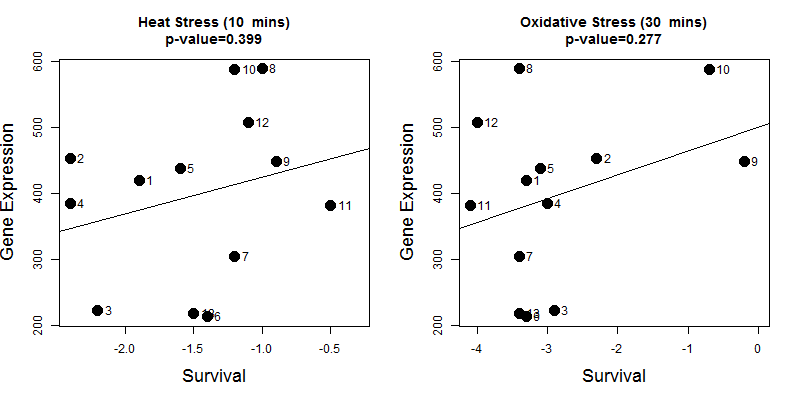

Supplement: S6 File — Expression levels of genes LACR_1383 –LACR_2610 and LACR_A01 –LACR_E8 plotted against survival after 10 minutes heat and 30 minutes oxidative stress. Survival is expressed as the difference of log CFU/ml after stress and before stress. Numbers indicate fermentations as presented in Table 1. P-values above the plots indicate significance of correlation (assessed by a linear model). (ZIP) [file pone.0167944.s011.zip › S6_File/LACR_1383_real_dat.png]

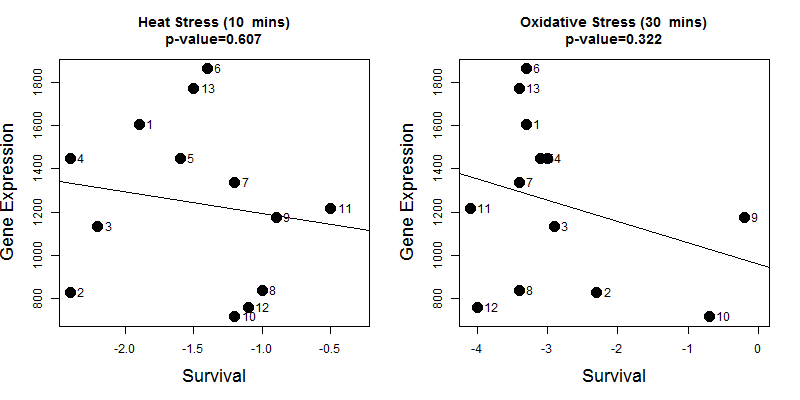

Supplement: S6 File — Expression levels of genes LACR_1383 –LACR_2610 and LACR_A01 –LACR_E8 plotted against survival after 10 minutes heat and 30 minutes oxidative stress. Survival is expressed as the difference of log CFU/ml after stress and before stress. Numbers indicate fermentations as presented in Table 1. P-values above the plots indicate significance of correlation (assessed by a linear model). (ZIP) [file pone.0167944.s011.zip › S6_File/LACR_1386_real_dat.png]

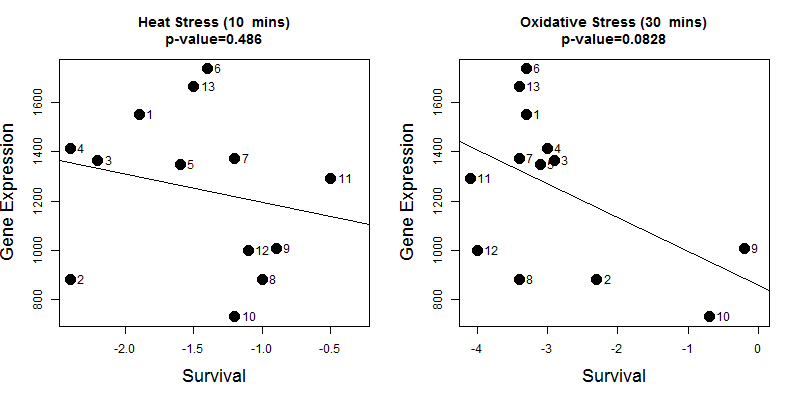

Supplement: S6 File — Expression levels of genes LACR_1383 –LACR_2610 and LACR_A01 –LACR_E8 plotted against survival after 10 minutes heat and 30 minutes oxidative stress. Survival is expressed as the difference of log CFU/ml after stress and before stress. Numbers indicate fermentations as presented in Table 1. P-values above the plots indicate significance of correlation (assessed by a linear model). (ZIP) [file pone.0167944.s011.zip › S6_File/LACR_1387_real_dat.png]

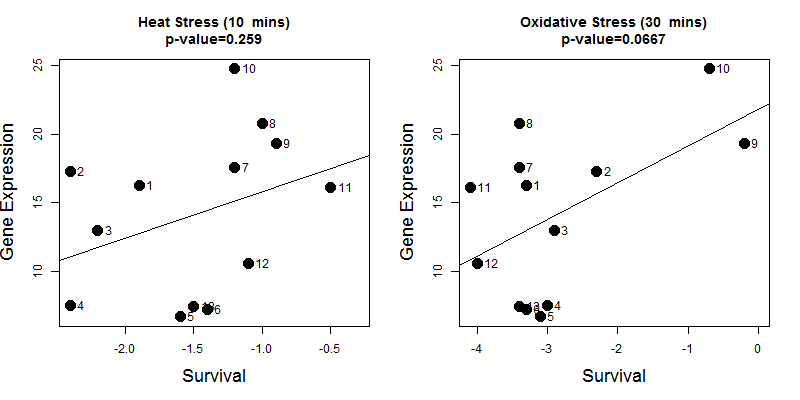

Supplement: S6 File — Expression levels of genes LACR_1383 –LACR_2610 and LACR_A01 –LACR_E8 plotted against survival after 10 minutes heat and 30 minutes oxidative stress. Survival is expressed as the difference of log CFU/ml after stress and before stress. Numbers indicate fermentations as presented in Table 1. P-values above the plots indicate significance of correlation (assessed by a linear model). (ZIP) [file pone.0167944.s011.zip › S6_File/LACR_1388_real_dat.png]

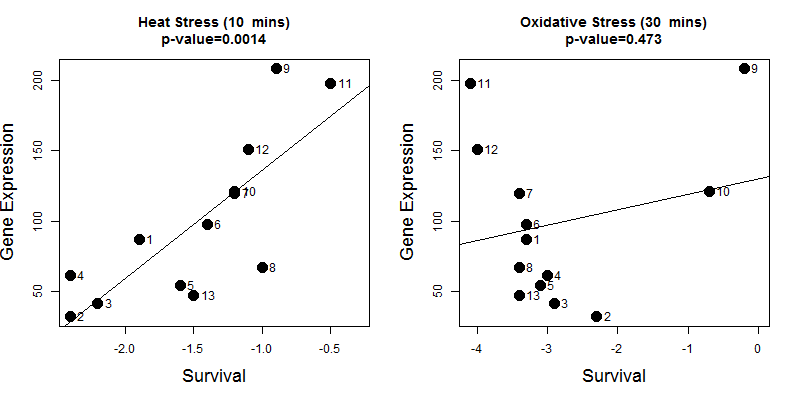

Supplement: S6 File — Expression levels of genes LACR_1383 –LACR_2610 and LACR_A01 –LACR_E8 plotted against survival after 10 minutes heat and 30 minutes oxidative stress. Survival is expressed as the difference of log CFU/ml after stress and before stress. Numbers indicate fermentations as presented in Table 1. P-values above the plots indicate significance of correlation (assessed by a linear model). (ZIP) [file pone.0167944.s011.zip › S6_File/LACR_1389_real_dat.png]

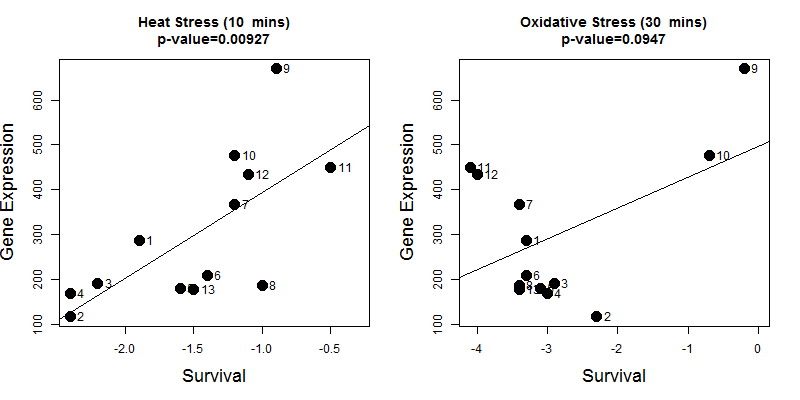

Supplement: S6 File — Expression levels of genes LACR_1383 –LACR_2610 and LACR_A01 –LACR_E8 plotted against survival after 10 minutes heat and 30 minutes oxidative stress. Survival is expressed as the difference of log CFU/ml after stress and before stress. Numbers indicate fermentations as presented in Table 1. P-values above the plots indicate significance of correlation (assessed by a linear model). (ZIP) [file pone.0167944.s011.zip › S6_File/LACR_1390_real_dat.png]

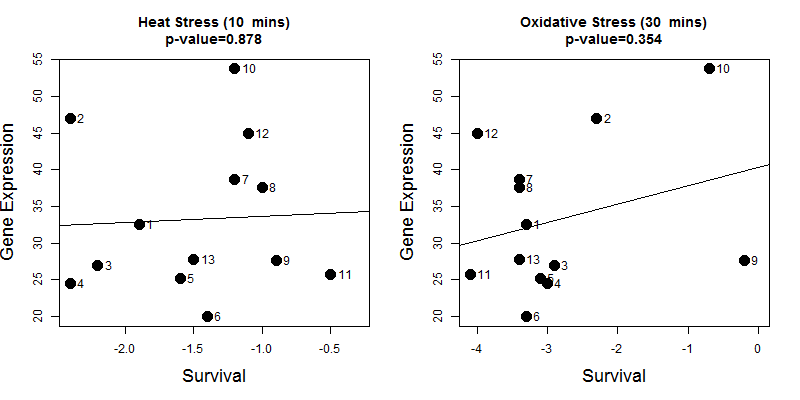

Supplement: S6 File — Expression levels of genes LACR_1383 –LACR_2610 and LACR_A01 –LACR_E8 plotted against survival after 10 minutes heat and 30 minutes oxidative stress. Survival is expressed as the difference of log CFU/ml after stress and before stress. Numbers indicate fermentations as presented in Table 1. P-values above the plots indicate significance of correlation (assessed by a linear model). (ZIP) [file pone.0167944.s011.zip › S6_File/LACR_1391_real_dat.png]

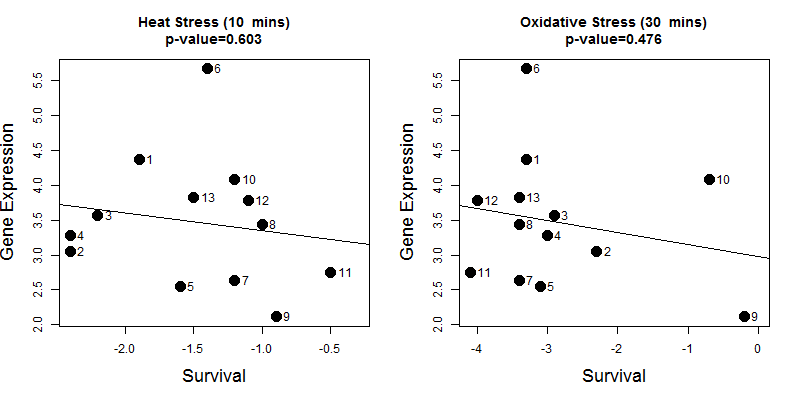

Supplement: S6 File — Expression levels of genes LACR_1383 –LACR_2610 and LACR_A01 –LACR_E8 plotted against survival after 10 minutes heat and 30 minutes oxidative stress. Survival is expressed as the difference of log CFU/ml after stress and before stress. Numbers indicate fermentations as presented in Table 1. P-values above the plots indicate significance of correlation (assessed by a linear model). (ZIP) [file pone.0167944.s011.zip › S6_File/LACR_1393_real_dat.png]

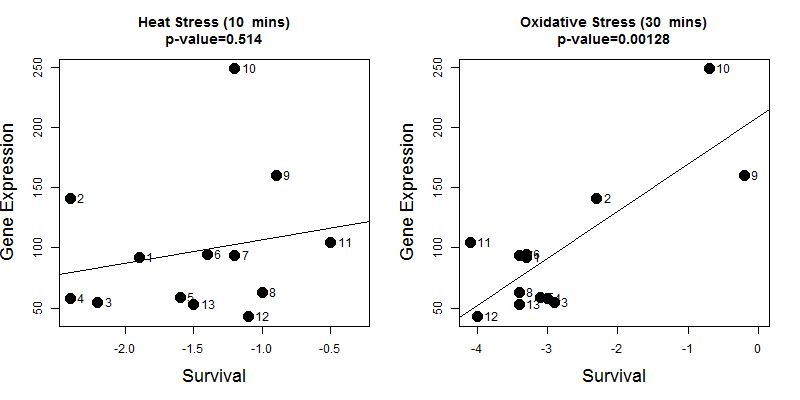

Supplement: S6 File — Expression levels of genes LACR_1383 –LACR_2610 and LACR_A01 –LACR_E8 plotted against survival after 10 minutes heat and 30 minutes oxidative stress. Survival is expressed as the difference of log CFU/ml after stress and before stress. Numbers indicate fermentations as presented in Table 1. P-values above the plots indicate significance of correlation (assessed by a linear model). (ZIP) [file pone.0167944.s011.zip › S6_File/LACR_1395_real_dat.png]

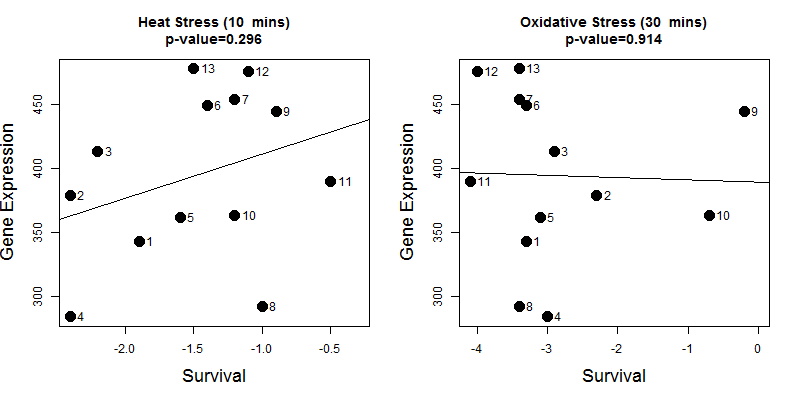

Supplement: S6 File — Expression levels of genes LACR_1383 –LACR_2610 and LACR_A01 –LACR_E8 plotted against survival after 10 minutes heat and 30 minutes oxidative stress. Survival is expressed as the difference of log CFU/ml after stress and before stress. Numbers indicate fermentations as presented in Table 1. P-values above the plots indicate significance of correlation (assessed by a linear model). (ZIP) [file pone.0167944.s011.zip › S6_File/LACR_1396_real_dat.png]

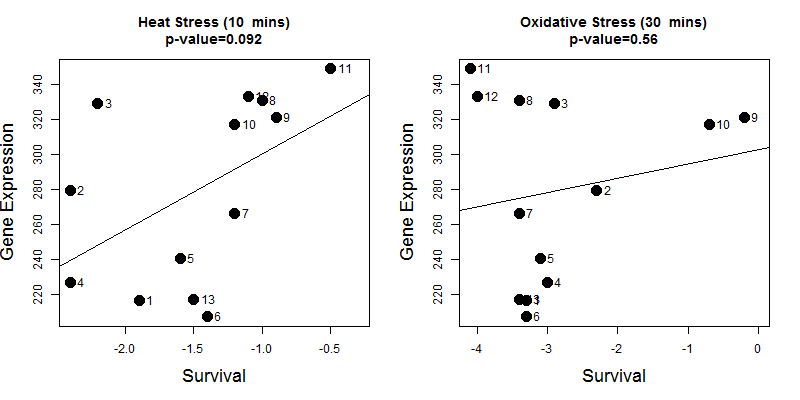

Supplement: S6 File — Expression levels of genes LACR_1383 –LACR_2610 and LACR_A01 –LACR_E8 plotted against survival after 10 minutes heat and 30 minutes oxidative stress. Survival is expressed as the difference of log CFU/ml after stress and before stress. Numbers indicate fermentations as presented in Table 1. P-values above the plots indicate significance of correlation (assessed by a linear model). (ZIP) [file pone.0167944.s011.zip › S6_File/LACR_1397_real_dat.png]

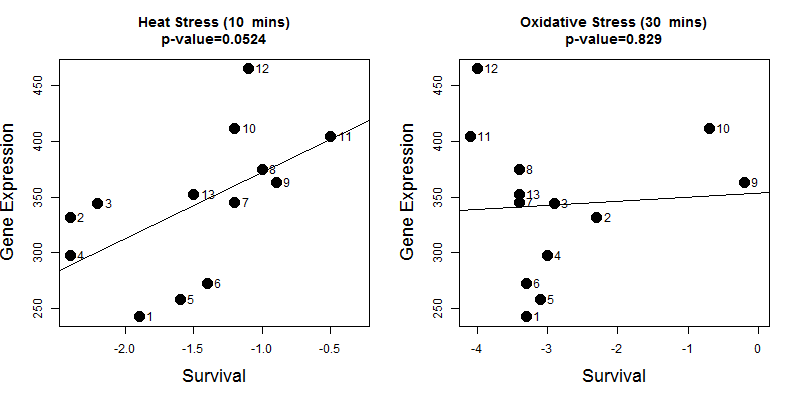

Supplement: S6 File — Expression levels of genes LACR_1383 –LACR_2610 and LACR_A01 –LACR_E8 plotted against survival after 10 minutes heat and 30 minutes oxidative stress. Survival is expressed as the difference of log CFU/ml after stress and before stress. Numbers indicate fermentations as presented in Table 1. P-values above the plots indicate significance of correlation (assessed by a linear model). (ZIP) [file pone.0167944.s011.zip › S6_File/LACR_1398_real_dat.png]

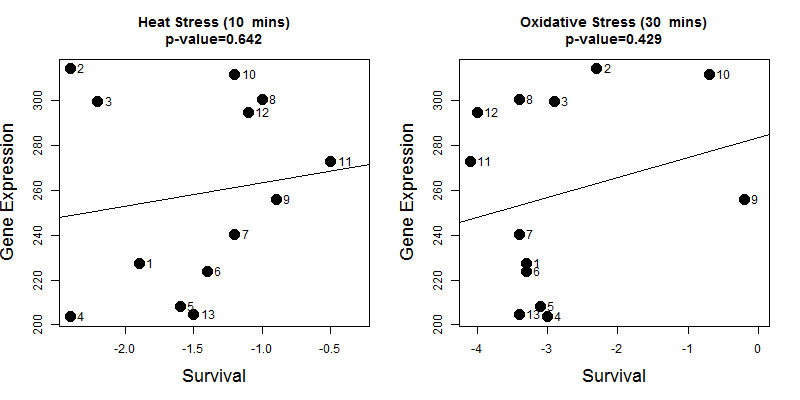

Supplement: S6 File — Expression levels of genes LACR_1383 –LACR_2610 and LACR_A01 –LACR_E8 plotted against survival after 10 minutes heat and 30 minutes oxidative stress. Survival is expressed as the difference of log CFU/ml after stress and before stress. Numbers indicate fermentations as presented in Table 1. P-values above the plots indicate significance of correlation (assessed by a linear model). (ZIP) [file pone.0167944.s011.zip › S6_File/LACR_1399_real_dat.png]

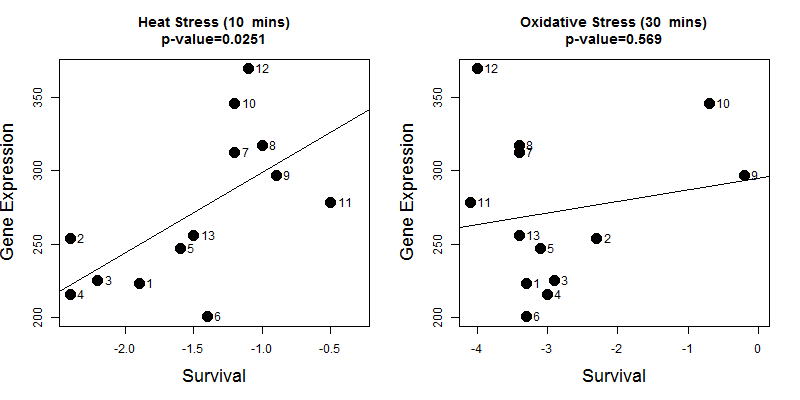

Supplement: S6 File — Expression levels of genes LACR_1383 –LACR_2610 and LACR_A01 –LACR_E8 plotted against survival after 10 minutes heat and 30 minutes oxidative stress. Survival is expressed as the difference of log CFU/ml after stress and before stress. Numbers indicate fermentations as presented in Table 1. P-values above the plots indicate significance of correlation (assessed by a linear model). (ZIP) [file pone.0167944.s011.zip › S6_File/LACR_1400_real_dat.png]

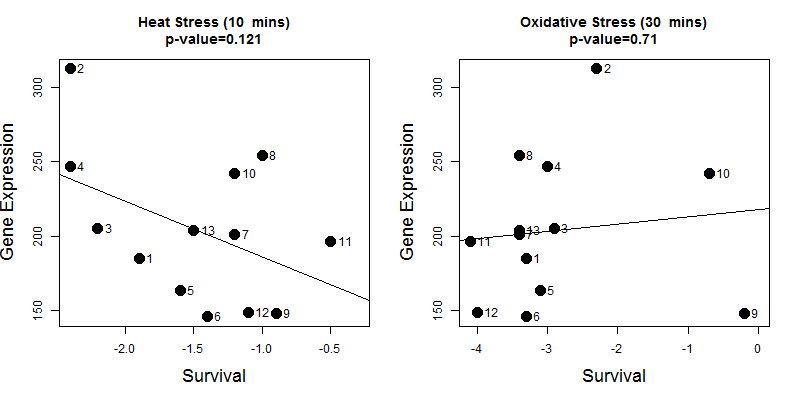

Supplement: S6 File — Expression levels of genes LACR_1383 –LACR_2610 and LACR_A01 –LACR_E8 plotted against survival after 10 minutes heat and 30 minutes oxidative stress. Survival is expressed as the difference of log CFU/ml after stress and before stress. Numbers indicate fermentations as presented in Table 1. P-values above the plots indicate significance of correlation (assessed by a linear model). (ZIP) [file pone.0167944.s011.zip › S6_File/LACR_1401_real_dat.png]

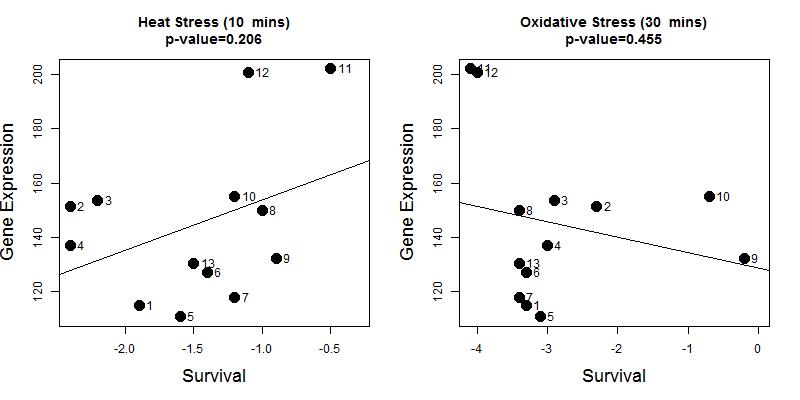

Supplement: S6 File — Expression levels of genes LACR_1383 –LACR_2610 and LACR_A01 –LACR_E8 plotted against survival after 10 minutes heat and 30 minutes oxidative stress. Survival is expressed as the difference of log CFU/ml after stress and before stress. Numbers indicate fermentations as presented in Table 1. P-values above the plots indicate significance of correlation (assessed by a linear model). (ZIP) [file pone.0167944.s011.zip › S6_File/LACR_1402_real_dat.png]

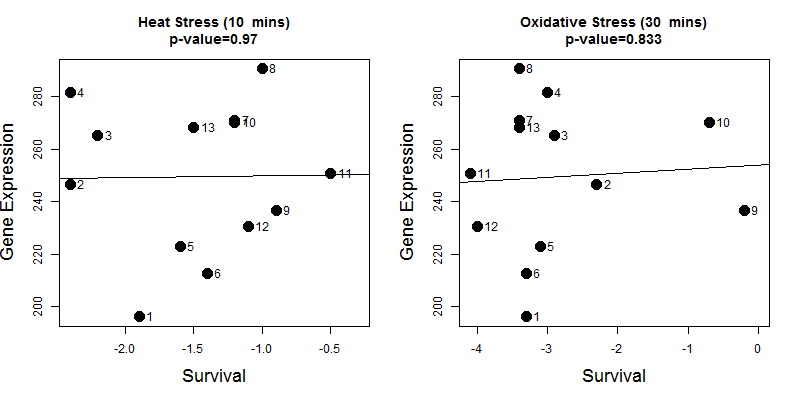

Supplement: S6 File — Expression levels of genes LACR_1383 –LACR_2610 and LACR_A01 –LACR_E8 plotted against survival after 10 minutes heat and 30 minutes oxidative stress. Survival is expressed as the difference of log CFU/ml after stress and before stress. Numbers indicate fermentations as presented in Table 1. P-values above the plots indicate significance of correlation (assessed by a linear model). (ZIP) [file pone.0167944.s011.zip › S6_File/LACR_1403_real_dat.png]

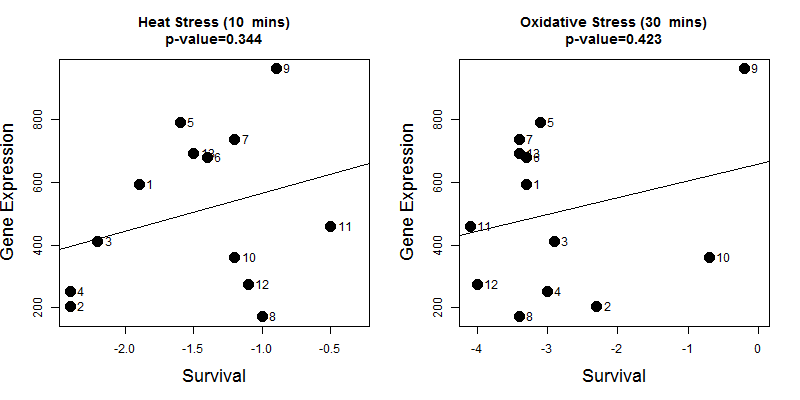

Supplement: S6 File — Expression levels of genes LACR_1383 –LACR_2610 and LACR_A01 –LACR_E8 plotted against survival after 10 minutes heat and 30 minutes oxidative stress. Survival is expressed as the difference of log CFU/ml after stress and before stress. Numbers indicate fermentations as presented in Table 1. P-values above the plots indicate significance of correlation (assessed by a linear model). (ZIP) [file pone.0167944.s011.zip › S6_File/LACR_1404_real_dat.png]

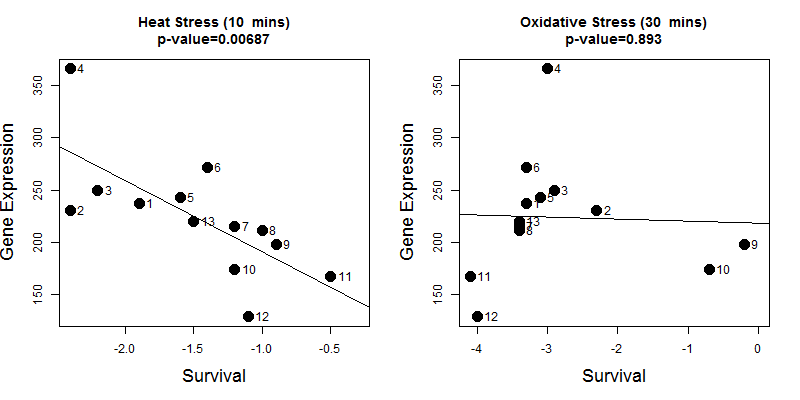

Supplement: S6 File — Expression levels of genes LACR_1383 –LACR_2610 and LACR_A01 –LACR_E8 plotted against survival after 10 minutes heat and 30 minutes oxidative stress. Survival is expressed as the difference of log CFU/ml after stress and before stress. Numbers indicate fermentations as presented in Table 1. P-values above the plots indicate significance of correlation (assessed by a linear model). (ZIP) [file pone.0167944.s011.zip › S6_File/LACR_1405_real_dat.png]

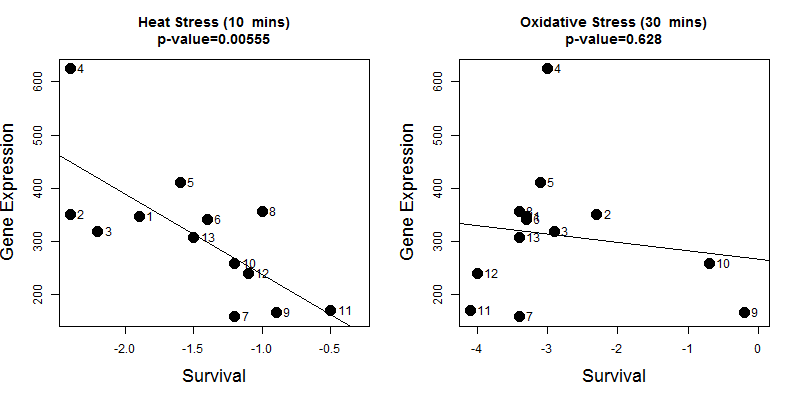

Supplement: S6 File — Expression levels of genes LACR_1383 –LACR_2610 and LACR_A01 –LACR_E8 plotted against survival after 10 minutes heat and 30 minutes oxidative stress. Survival is expressed as the difference of log CFU/ml after stress and before stress. Numbers indicate fermentations as presented in Table 1. P-values above the plots indicate significance of correlation (assessed by a linear model). (ZIP) [file pone.0167944.s011.zip › S6_File/LACR_1406_real_dat.png]

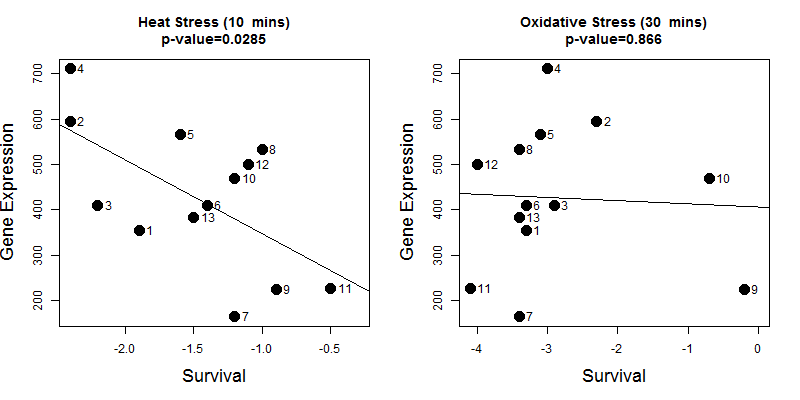

Supplement: S6 File — Expression levels of genes LACR_1383 –LACR_2610 and LACR_A01 –LACR_E8 plotted against survival after 10 minutes heat and 30 minutes oxidative stress. Survival is expressed as the difference of log CFU/ml after stress and before stress. Numbers indicate fermentations as presented in Table 1. P-values above the plots indicate significance of correlation (assessed by a linear model). (ZIP) [file pone.0167944.s011.zip › S6_File/LACR_1407_real_dat.png]

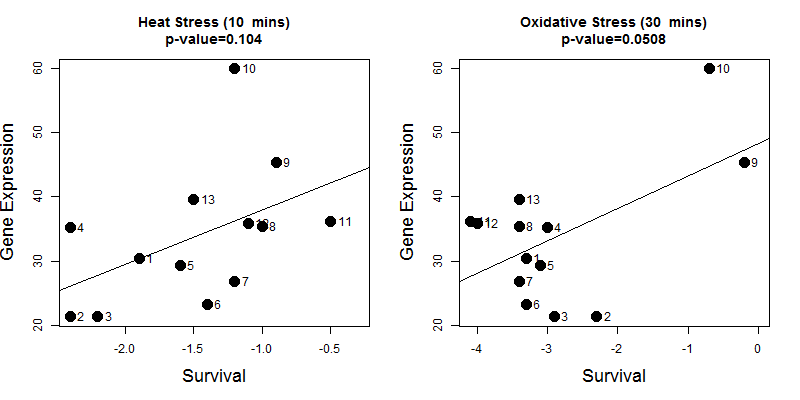

Supplement: S6 File — Expression levels of genes LACR_1383 –LACR_2610 and LACR_A01 –LACR_E8 plotted against survival after 10 minutes heat and 30 minutes oxidative stress. Survival is expressed as the difference of log CFU/ml after stress and before stress. Numbers indicate fermentations as presented in Table 1. P-values above the plots indicate significance of correlation (assessed by a linear model). (ZIP) [file pone.0167944.s011.zip › S6_File/LACR_1408_real_dat.png]

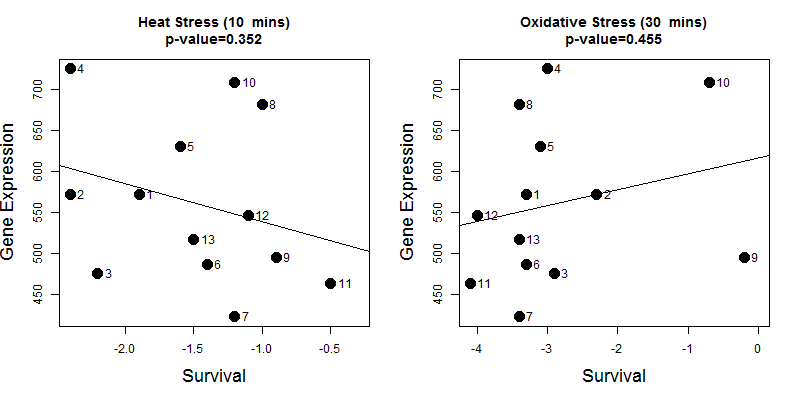

Supplement: S6 File — Expression levels of genes LACR_1383 –LACR_2610 and LACR_A01 –LACR_E8 plotted against survival after 10 minutes heat and 30 minutes oxidative stress. Survival is expressed as the difference of log CFU/ml after stress and before stress. Numbers indicate fermentations as presented in Table 1. P-values above the plots indicate significance of correlation (assessed by a linear model). (ZIP) [file pone.0167944.s011.zip › S6_File/LACR_1409_real_dat.png]

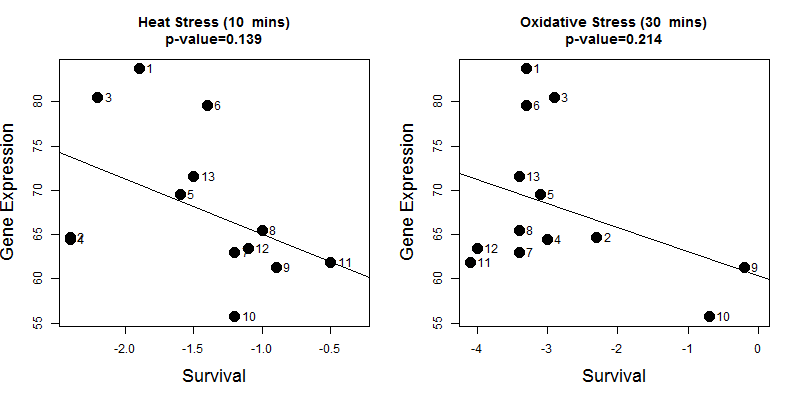

Supplement: S6 File — Expression levels of genes LACR_1383 –LACR_2610 and LACR_A01 –LACR_E8 plotted against survival after 10 minutes heat and 30 minutes oxidative stress. Survival is expressed as the difference of log CFU/ml after stress and before stress. Numbers indicate fermentations as presented in Table 1. P-values above the plots indicate significance of correlation (assessed by a linear model). (ZIP) [file pone.0167944.s011.zip › S6_File/LACR_1410_real_dat.png]

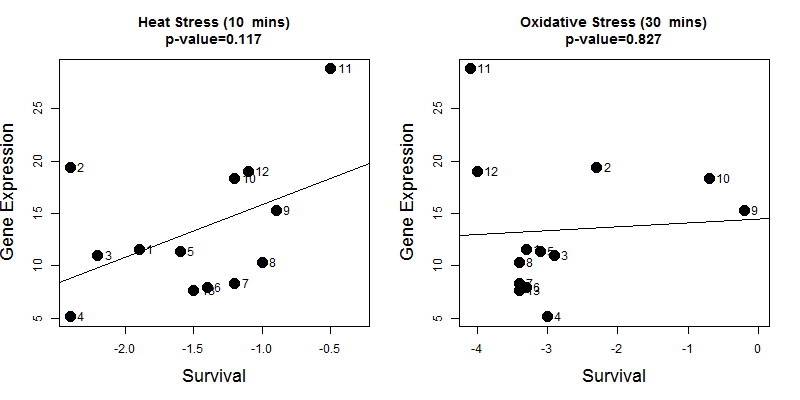

Supplement: S6 File — Expression levels of genes LACR_1383 –LACR_2610 and LACR_A01 –LACR_E8 plotted against survival after 10 minutes heat and 30 minutes oxidative stress. Survival is expressed as the difference of log CFU/ml after stress and before stress. Numbers indicate fermentations as presented in Table 1. P-values above the plots indicate significance of correlation (assessed by a linear model). (ZIP) [file pone.0167944.s011.zip › S6_File/LACR_1412_real_dat.png]

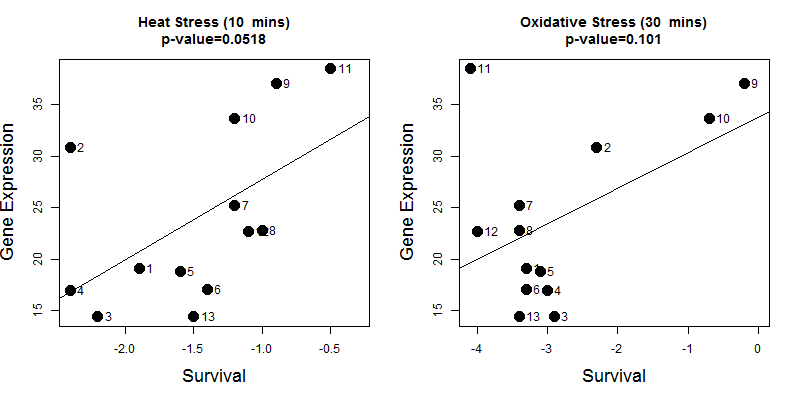

Supplement: S6 File — Expression levels of genes LACR_1383 –LACR_2610 and LACR_A01 –LACR_E8 plotted against survival after 10 minutes heat and 30 minutes oxidative stress. Survival is expressed as the difference of log CFU/ml after stress and before stress. Numbers indicate fermentations as presented in Table 1. P-values above the plots indicate significance of correlation (assessed by a linear model). (ZIP) [file pone.0167944.s011.zip › S6_File/LACR_1413_real_dat.png]

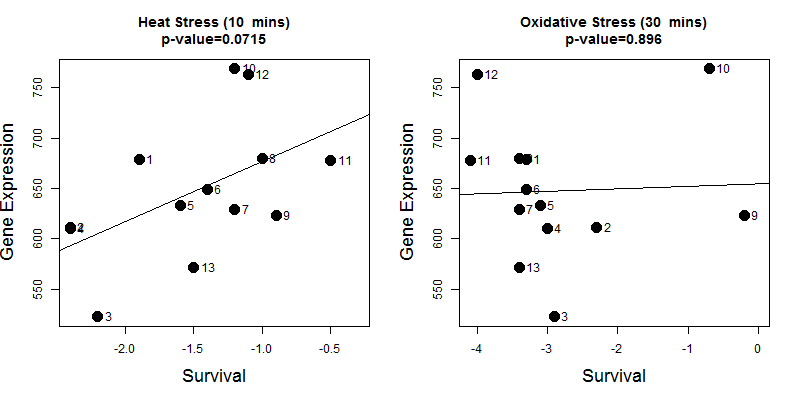

Supplement: S6 File — Expression levels of genes LACR_1383 –LACR_2610 and LACR_A01 –LACR_E8 plotted against survival after 10 minutes heat and 30 minutes oxidative stress. Survival is expressed as the difference of log CFU/ml after stress and before stress. Numbers indicate fermentations as presented in Table 1. P-values above the plots indicate significance of correlation (assessed by a linear model). (ZIP) [file pone.0167944.s011.zip › S6_File/LACR_1414_real_dat.png]

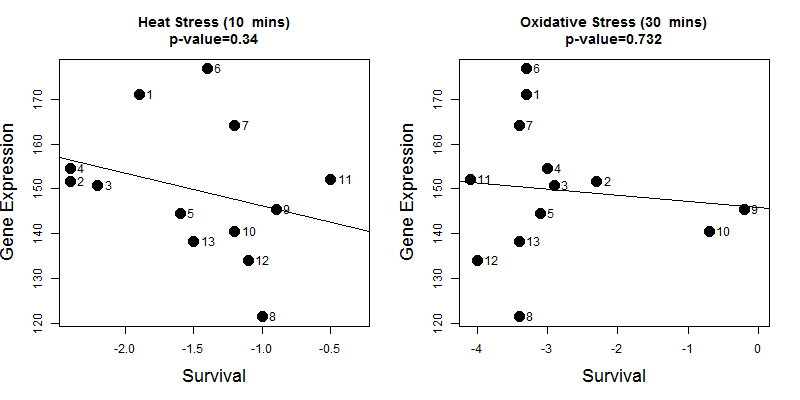

Supplement: S6 File — Expression levels of genes LACR_1383 –LACR_2610 and LACR_A01 –LACR_E8 plotted against survival after 10 minutes heat and 30 minutes oxidative stress. Survival is expressed as the difference of log CFU/ml after stress and before stress. Numbers indicate fermentations as presented in Table 1. P-values above the plots indicate significance of correlation (assessed by a linear model). (ZIP) [file pone.0167944.s011.zip › S6_File/LACR_1415_real_dat.png]

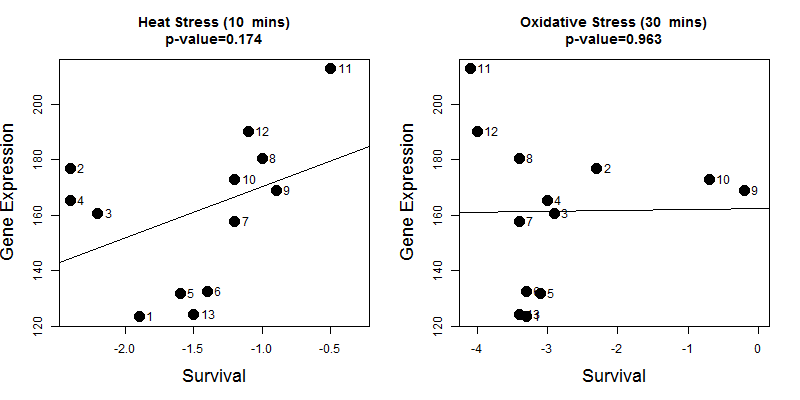

Supplement: S6 File — Expression levels of genes LACR_1383 –LACR_2610 and LACR_A01 –LACR_E8 plotted against survival after 10 minutes heat and 30 minutes oxidative stress. Survival is expressed as the difference of log CFU/ml after stress and before stress. Numbers indicate fermentations as presented in Table 1. P-values above the plots indicate significance of correlation (assessed by a linear model). (ZIP) [file pone.0167944.s011.zip › S6_File/LACR_1416_real_dat.png]

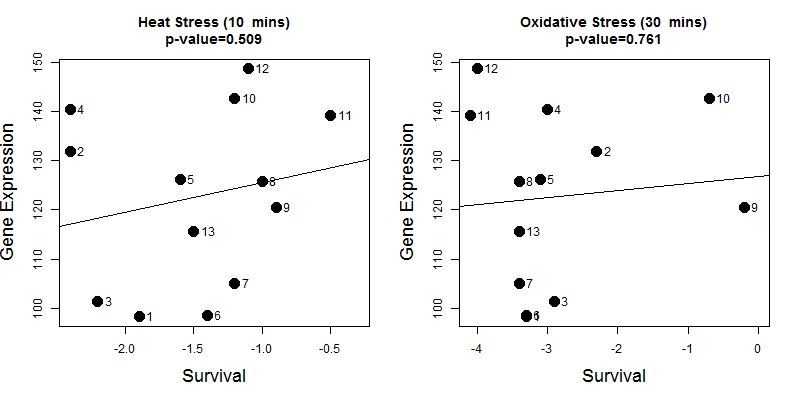

Supplement: S6 File — Expression levels of genes LACR_1383 –LACR_2610 and LACR_A01 –LACR_E8 plotted against survival after 10 minutes heat and 30 minutes oxidative stress. Survival is expressed as the difference of log CFU/ml after stress and before stress. Numbers indicate fermentations as presented in Table 1. P-values above the plots indicate significance of correlation (assessed by a linear model). (ZIP) [file pone.0167944.s011.zip › S6_File/LACR_1417_real_dat.png]

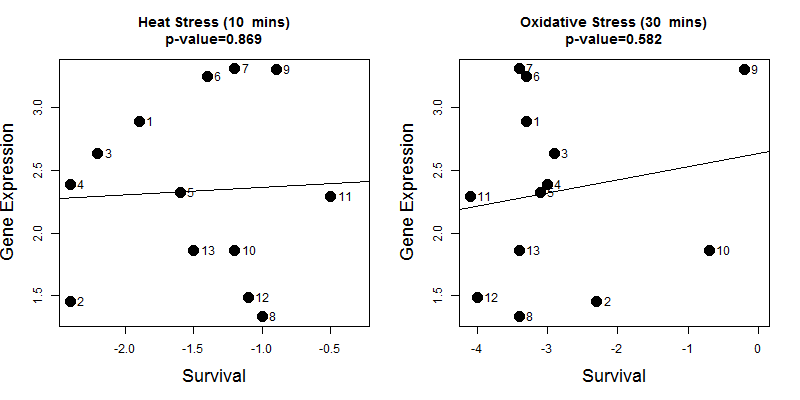

Supplement: S6 File — Expression levels of genes LACR_1383 –LACR_2610 and LACR_A01 –LACR_E8 plotted against survival after 10 minutes heat and 30 minutes oxidative stress. Survival is expressed as the difference of log CFU/ml after stress and before stress. Numbers indicate fermentations as presented in Table 1. P-values above the plots indicate significance of correlation (assessed by a linear model). (ZIP) [file pone.0167944.s011.zip › S6_File/LACR_1418_real_dat.png]

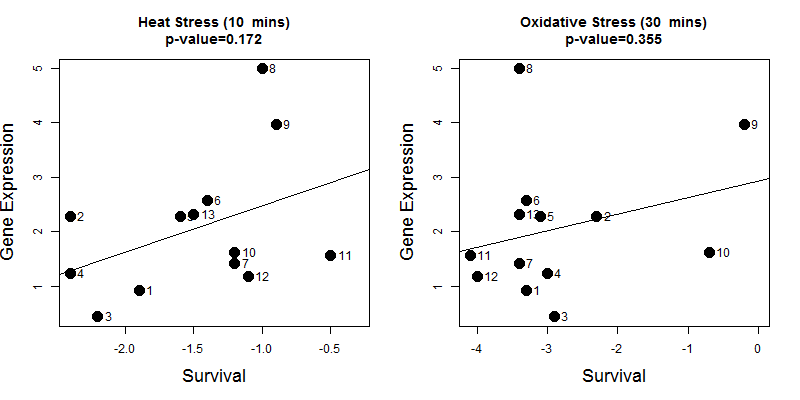

Supplement: S6 File — Expression levels of genes LACR_1383 –LACR_2610 and LACR_A01 –LACR_E8 plotted against survival after 10 minutes heat and 30 minutes oxidative stress. Survival is expressed as the difference of log CFU/ml after stress and before stress. Numbers indicate fermentations as presented in Table 1. P-values above the plots indicate significance of correlation (assessed by a linear model). (ZIP) [file pone.0167944.s011.zip › S6_File/LACR_1419_real_dat.png]

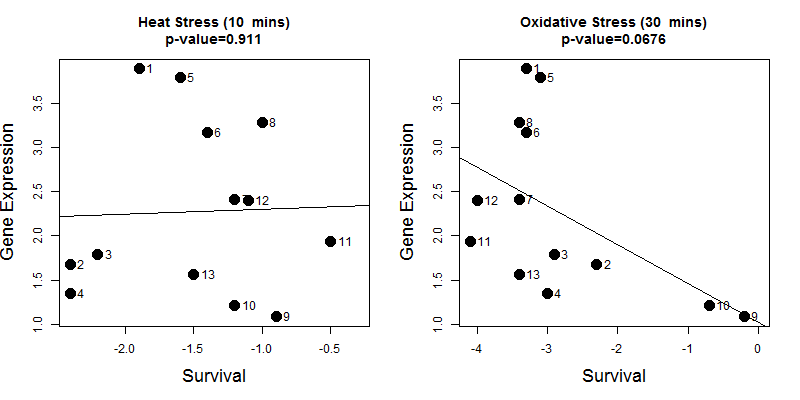

Supplement: S6 File — Expression levels of genes LACR_1383 –LACR_2610 and LACR_A01 –LACR_E8 plotted against survival after 10 minutes heat and 30 minutes oxidative stress. Survival is expressed as the difference of log CFU/ml after stress and before stress. Numbers indicate fermentations as presented in Table 1. P-values above the plots indicate significance of correlation (assessed by a linear model). (ZIP) [file pone.0167944.s011.zip › S6_File/LACR_1420_real_dat.png]

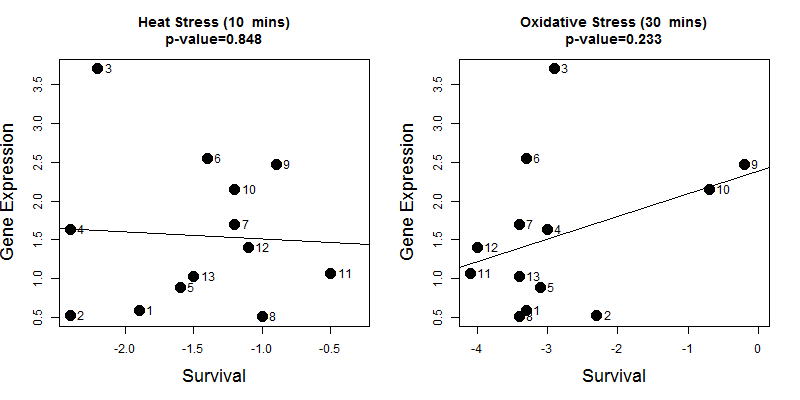

Supplement: S6 File — Expression levels of genes LACR_1383 –LACR_2610 and LACR_A01 –LACR_E8 plotted against survival after 10 minutes heat and 30 minutes oxidative stress. Survival is expressed as the difference of log CFU/ml after stress and before stress. Numbers indicate fermentations as presented in Table 1. P-values above the plots indicate significance of correlation (assessed by a linear model). (ZIP) [file pone.0167944.s011.zip › S6_File/LACR_1421_real_dat.png]

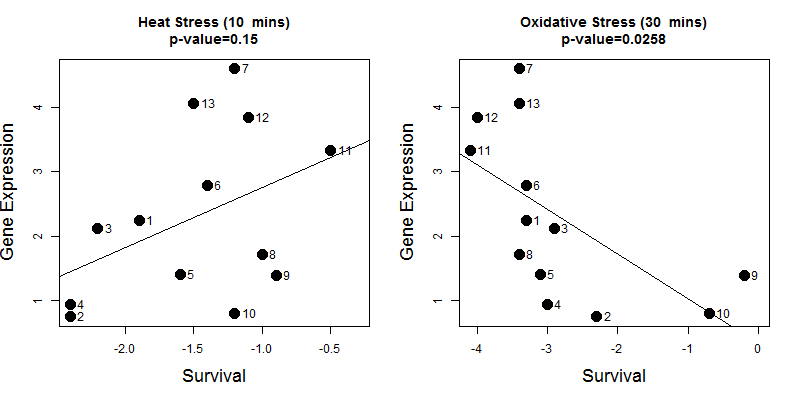

Supplement: S6 File — Expression levels of genes LACR_1383 –LACR_2610 and LACR_A01 –LACR_E8 plotted against survival after 10 minutes heat and 30 minutes oxidative stress. Survival is expressed as the difference of log CFU/ml after stress and before stress. Numbers indicate fermentations as presented in Table 1. P-values above the plots indicate significance of correlation (assessed by a linear model). (ZIP) [file pone.0167944.s011.zip › S6_File/LACR_1422_real_dat.png]

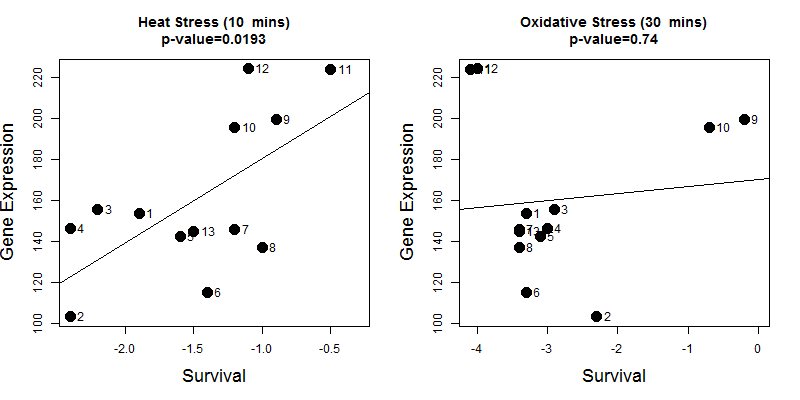

Supplement: S6 File — Expression levels of genes LACR_1383 –LACR_2610 and LACR_A01 –LACR_E8 plotted against survival after 10 minutes heat and 30 minutes oxidative stress. Survival is expressed as the difference of log CFU/ml after stress and before stress. Numbers indicate fermentations as presented in Table 1. P-values above the plots indicate significance of correlation (assessed by a linear model). (ZIP) [file pone.0167944.s011.zip › S6_File/LACR_1423_real_dat.png]

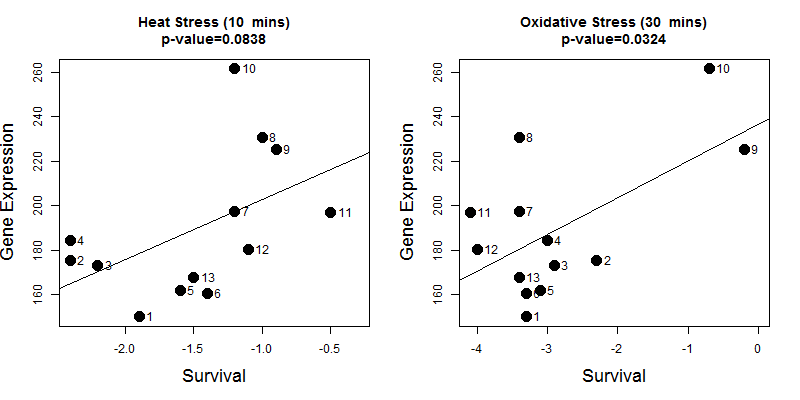

Supplement: S6 File — Expression levels of genes LACR_1383 –LACR_2610 and LACR_A01 –LACR_E8 plotted against survival after 10 minutes heat and 30 minutes oxidative stress. Survival is expressed as the difference of log CFU/ml after stress and before stress. Numbers indicate fermentations as presented in Table 1. P-values above the plots indicate significance of correlation (assessed by a linear model). (ZIP) [file pone.0167944.s011.zip › S6_File/LACR_1424_real_dat.png]

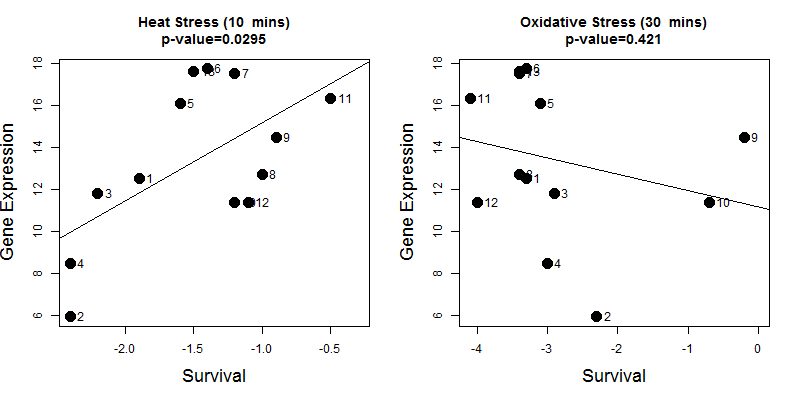

Supplement: S6 File — Expression levels of genes LACR_1383 –LACR_2610 and LACR_A01 –LACR_E8 plotted against survival after 10 minutes heat and 30 minutes oxidative stress. Survival is expressed as the difference of log CFU/ml after stress and before stress. Numbers indicate fermentations as presented in Table 1. P-values above the plots indicate significance of correlation (assessed by a linear model). (ZIP) [file pone.0167944.s011.zip › S6_File/LACR_1425_real_dat.png]

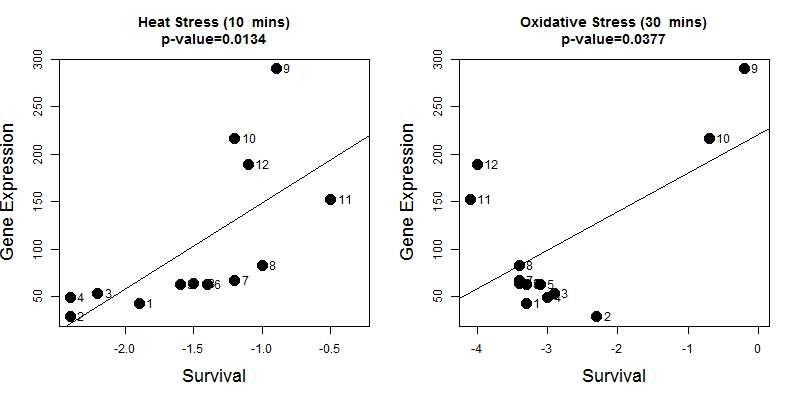

Supplement: S6 File — Expression levels of genes LACR_1383 –LACR_2610 and LACR_A01 –LACR_E8 plotted against survival after 10 minutes heat and 30 minutes oxidative stress. Survival is expressed as the difference of log CFU/ml after stress and before stress. Numbers indicate fermentations as presented in Table 1. P-values above the plots indicate significance of correlation (assessed by a linear model). (ZIP) [file pone.0167944.s011.zip › S6_File/LACR_1427_real_dat.png]

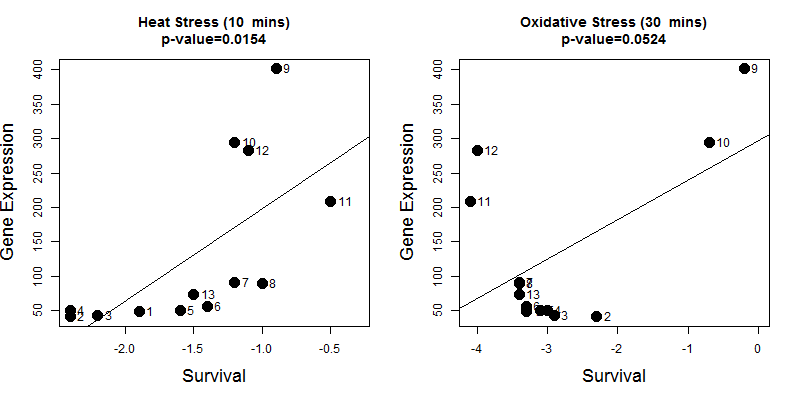

Supplement: S6 File — Expression levels of genes LACR_1383 –LACR_2610 and LACR_A01 –LACR_E8 plotted against survival after 10 minutes heat and 30 minutes oxidative stress. Survival is expressed as the difference of log CFU/ml after stress and before stress. Numbers indicate fermentations as presented in Table 1. P-values above the plots indicate significance of correlation (assessed by a linear model). (ZIP) [file pone.0167944.s011.zip › S6_File/LACR_1428_real_dat.png]

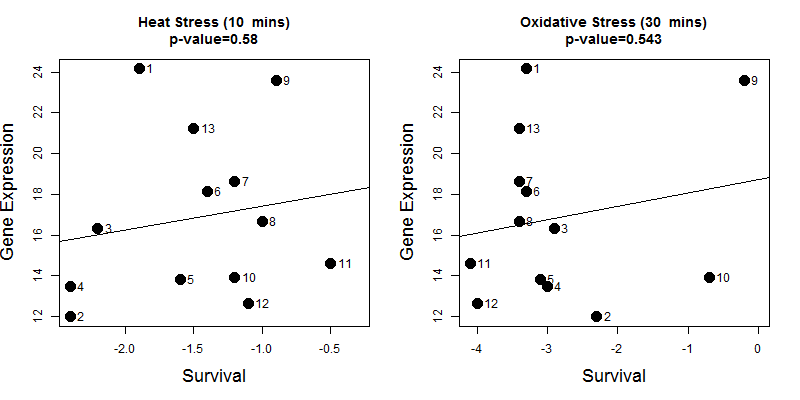

Supplement: S6 File — Expression levels of genes LACR_1383 –LACR_2610 and LACR_A01 –LACR_E8 plotted against survival after 10 minutes heat and 30 minutes oxidative stress. Survival is expressed as the difference of log CFU/ml after stress and before stress. Numbers indicate fermentations as presented in Table 1. P-values above the plots indicate significance of correlation (assessed by a linear model). (ZIP) [file pone.0167944.s011.zip › S6_File/LACR_1429_real_dat.png]

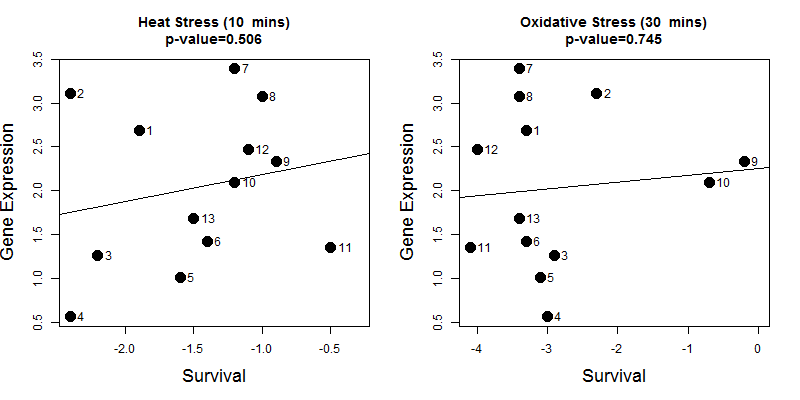

Supplement: S6 File — Expression levels of genes LACR_1383 –LACR_2610 and LACR_A01 –LACR_E8 plotted against survival after 10 minutes heat and 30 minutes oxidative stress. Survival is expressed as the difference of log CFU/ml after stress and before stress. Numbers indicate fermentations as presented in Table 1. P-values above the plots indicate significance of correlation (assessed by a linear model). (ZIP) [file pone.0167944.s011.zip › S6_File/LACR_1432_real_dat.png]

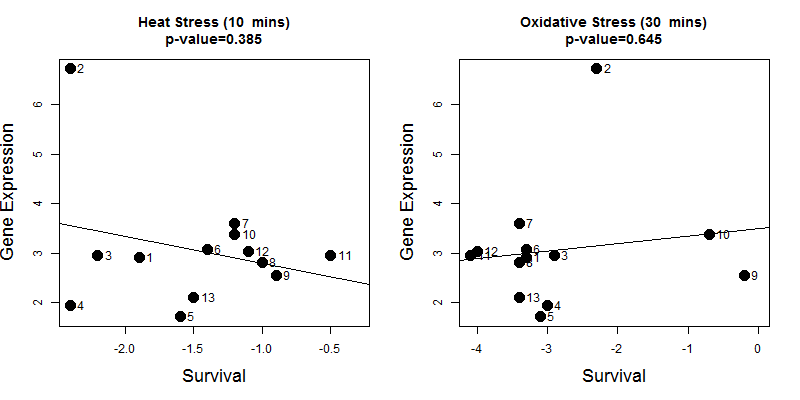

Supplement: S6 File — Expression levels of genes LACR_1383 –LACR_2610 and LACR_A01 –LACR_E8 plotted against survival after 10 minutes heat and 30 minutes oxidative stress. Survival is expressed as the difference of log CFU/ml after stress and before stress. Numbers indicate fermentations as presented in Table 1. P-values above the plots indicate significance of correlation (assessed by a linear model). (ZIP) [file pone.0167944.s011.zip › S6_File/LACR_1433_real_dat.png]

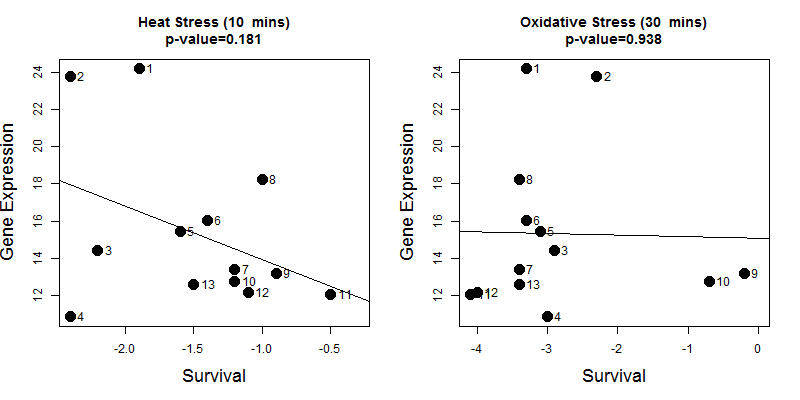

Supplement: S6 File — Expression levels of genes LACR_1383 –LACR_2610 and LACR_A01 –LACR_E8 plotted against survival after 10 minutes heat and 30 minutes oxidative stress. Survival is expressed as the difference of log CFU/ml after stress and before stress. Numbers indicate fermentations as presented in Table 1. P-values above the plots indicate significance of correlation (assessed by a linear model). (ZIP) [file pone.0167944.s011.zip › S6_File/LACR_1435_real_dat.png]

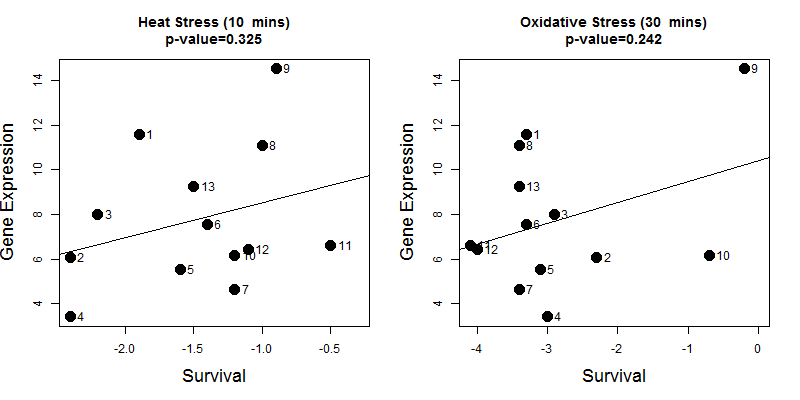

Supplement: S6 File — Expression levels of genes LACR_1383 –LACR_2610 and LACR_A01 –LACR_E8 plotted against survival after 10 minutes heat and 30 minutes oxidative stress. Survival is expressed as the difference of log CFU/ml after stress and before stress. Numbers indicate fermentations as presented in Table 1. P-values above the plots indicate significance of correlation (assessed by a linear model). (ZIP) [file pone.0167944.s011.zip › S6_File/LACR_1436_real_dat.png]

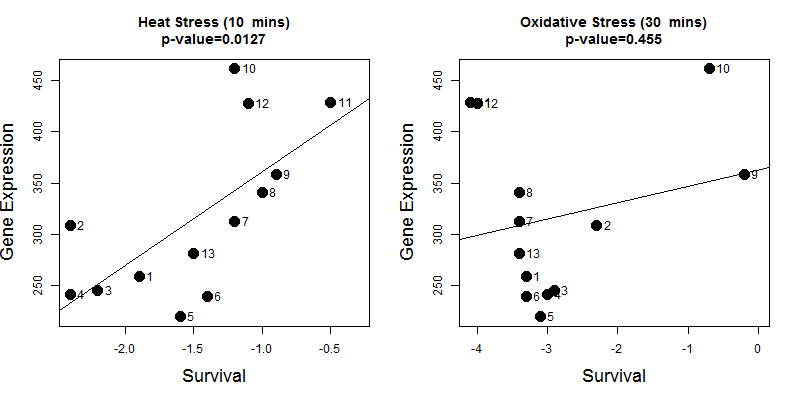

Supplement: S6 File — Expression levels of genes LACR_1383 –LACR_2610 and LACR_A01 –LACR_E8 plotted against survival after 10 minutes heat and 30 minutes oxidative stress. Survival is expressed as the difference of log CFU/ml after stress and before stress. Numbers indicate fermentations as presented in Table 1. P-values above the plots indicate significance of correlation (assessed by a linear model). (ZIP) [file pone.0167944.s011.zip › S6_File/LACR_1437_real_dat.png]

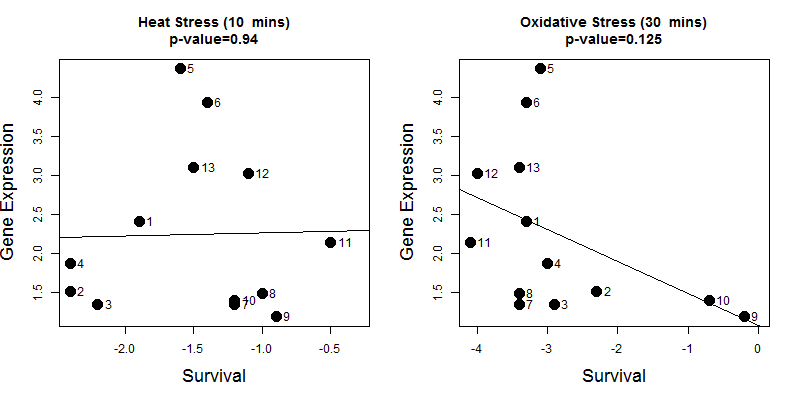

Supplement: S6 File — Expression levels of genes LACR_1383 –LACR_2610 and LACR_A01 –LACR_E8 plotted against survival after 10 minutes heat and 30 minutes oxidative stress. Survival is expressed as the difference of log CFU/ml after stress and before stress. Numbers indicate fermentations as presented in Table 1. P-values above the plots indicate significance of correlation (assessed by a linear model). (ZIP) [file pone.0167944.s011.zip › S6_File/LACR_1439_real_dat.png]

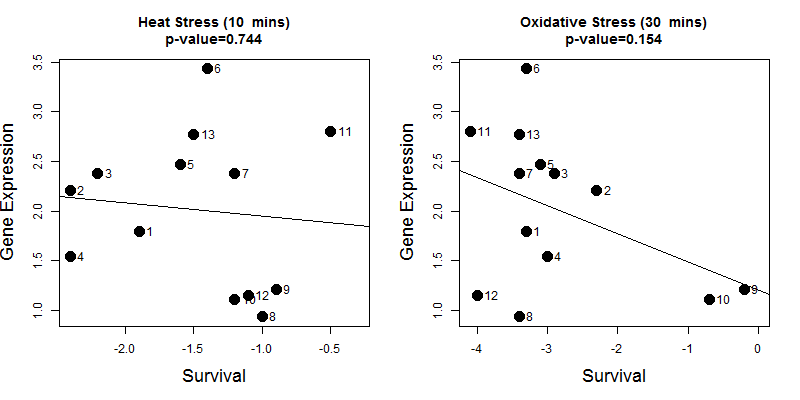

Supplement: S6 File — Expression levels of genes LACR_1383 –LACR_2610 and LACR_A01 –LACR_E8 plotted against survival after 10 minutes heat and 30 minutes oxidative stress. Survival is expressed as the difference of log CFU/ml after stress and before stress. Numbers indicate fermentations as presented in Table 1. P-values above the plots indicate significance of correlation (assessed by a linear model). (ZIP) [file pone.0167944.s011.zip › S6_File/LACR_1440_real_dat.png]

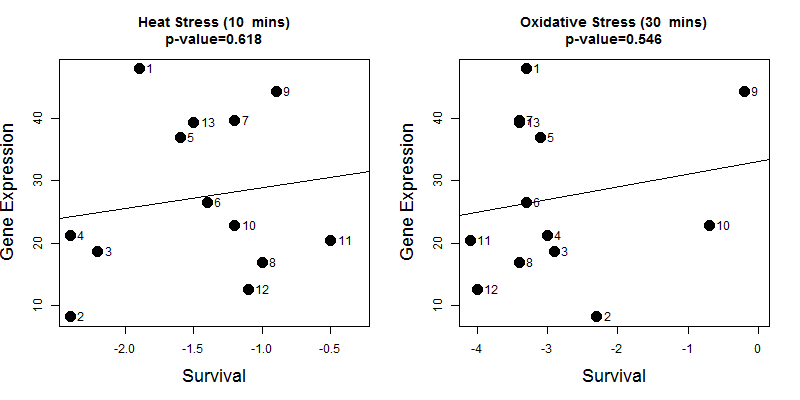

Supplement: S6 File — Expression levels of genes LACR_1383 –LACR_2610 and LACR_A01 –LACR_E8 plotted against survival after 10 minutes heat and 30 minutes oxidative stress. Survival is expressed as the difference of log CFU/ml after stress and before stress. Numbers indicate fermentations as presented in Table 1. P-values above the plots indicate significance of correlation (assessed by a linear model). (ZIP) [file pone.0167944.s011.zip › S6_File/LACR_1441_real_dat.png]

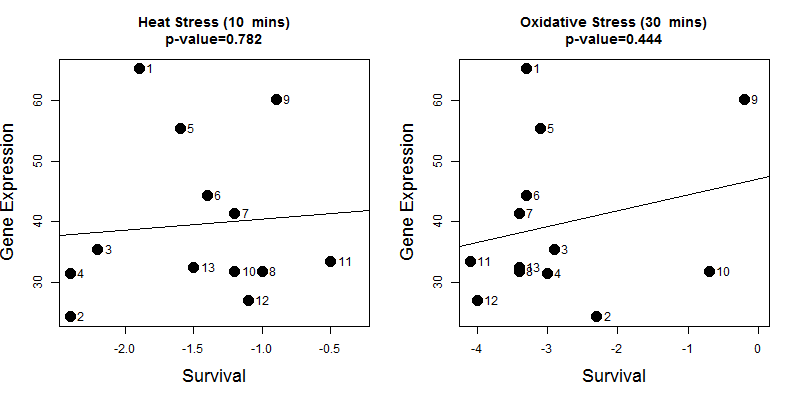

Supplement: S6 File — Expression levels of genes LACR_1383 –LACR_2610 and LACR_A01 –LACR_E8 plotted against survival after 10 minutes heat and 30 minutes oxidative stress. Survival is expressed as the difference of log CFU/ml after stress and before stress. Numbers indicate fermentations as presented in Table 1. P-values above the plots indicate significance of correlation (assessed by a linear model). (ZIP) [file pone.0167944.s011.zip › S6_File/LACR_1442_real_dat.png]

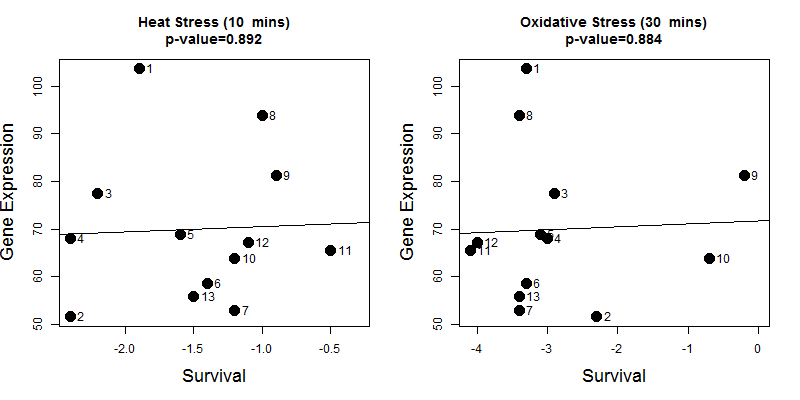

Supplement: S6 File — Expression levels of genes LACR_1383 –LACR_2610 and LACR_A01 –LACR_E8 plotted against survival after 10 minutes heat and 30 minutes oxidative stress. Survival is expressed as the difference of log CFU/ml after stress and before stress. Numbers indicate fermentations as presented in Table 1. P-values above the plots indicate significance of correlation (assessed by a linear model). (ZIP) [file pone.0167944.s011.zip › S6_File/LACR_1443_real_dat.png]

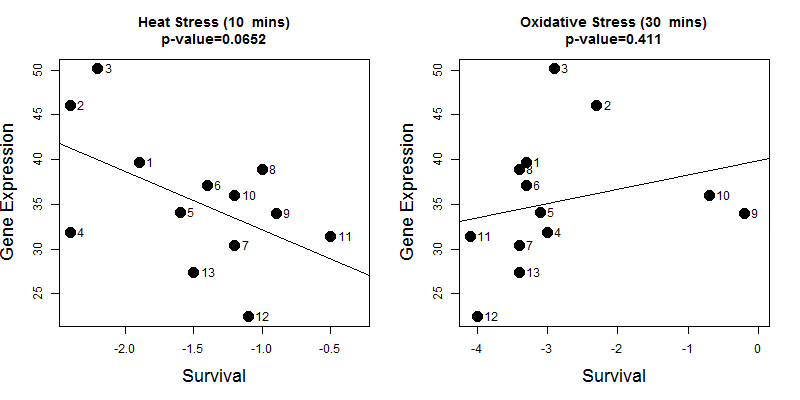

Supplement: S6 File — Expression levels of genes LACR_1383 –LACR_2610 and LACR_A01 –LACR_E8 plotted against survival after 10 minutes heat and 30 minutes oxidative stress. Survival is expressed as the difference of log CFU/ml after stress and before stress. Numbers indicate fermentations as presented in Table 1. P-values above the plots indicate significance of correlation (assessed by a linear model). (ZIP) [file pone.0167944.s011.zip › S6_File/LACR_1444_real_dat.png]

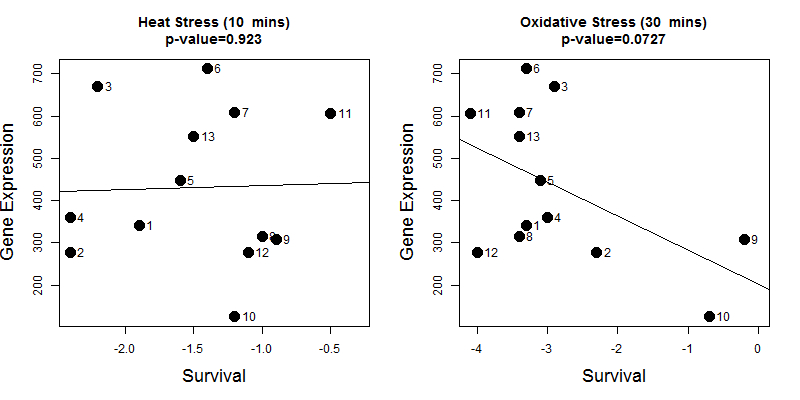

Supplement: S6 File — Expression levels of genes LACR_1383 –LACR_2610 and LACR_A01 –LACR_E8 plotted against survival after 10 minutes heat and 30 minutes oxidative stress. Survival is expressed as the difference of log CFU/ml after stress and before stress. Numbers indicate fermentations as presented in Table 1. P-values above the plots indicate significance of correlation (assessed by a linear model). (ZIP) [file pone.0167944.s011.zip › S6_File/LACR_1445_real_dat.png]

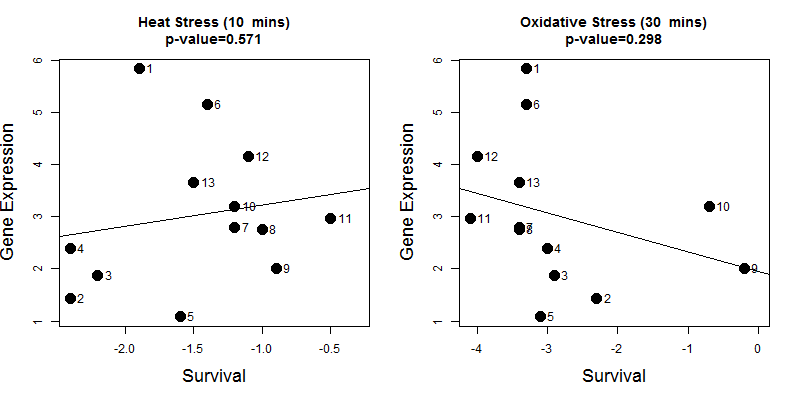

Supplement: S6 File — Expression levels of genes LACR_1383 –LACR_2610 and LACR_A01 –LACR_E8 plotted against survival after 10 minutes heat and 30 minutes oxidative stress. Survival is expressed as the difference of log CFU/ml after stress and before stress. Numbers indicate fermentations as presented in Table 1. P-values above the plots indicate significance of correlation (assessed by a linear model). (ZIP) [file pone.0167944.s011.zip › S6_File/LACR_1446_real_dat.png]

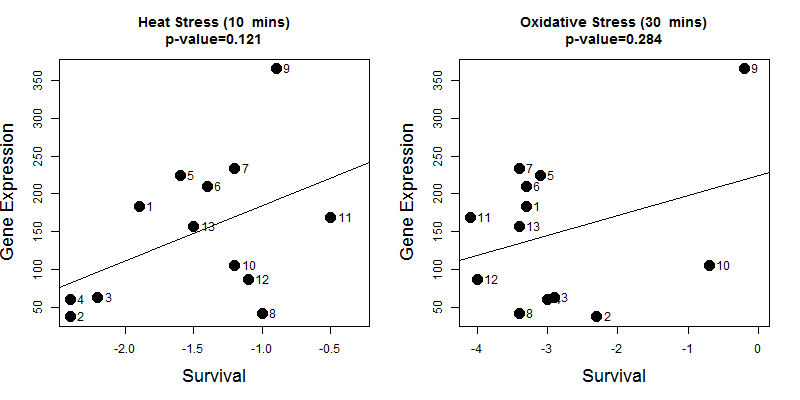

Supplement: S6 File — Expression levels of genes LACR_1383 –LACR_2610 and LACR_A01 –LACR_E8 plotted against survival after 10 minutes heat and 30 minutes oxidative stress. Survival is expressed as the difference of log CFU/ml after stress and before stress. Numbers indicate fermentations as presented in Table 1. P-values above the plots indicate significance of correlation (assessed by a linear model). (ZIP) [file pone.0167944.s011.zip › S6_File/LACR_1447_real_dat.png]

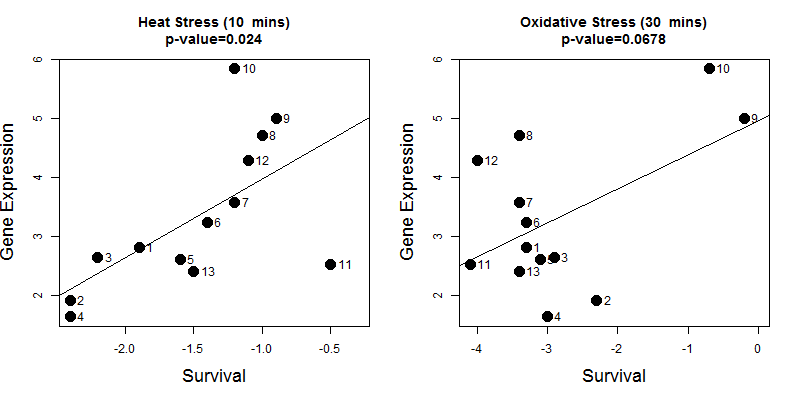

Supplement: S6 File — Expression levels of genes LACR_1383 –LACR_2610 and LACR_A01 –LACR_E8 plotted against survival after 10 minutes heat and 30 minutes oxidative stress. Survival is expressed as the difference of log CFU/ml after stress and before stress. Numbers indicate fermentations as presented in Table 1. P-values above the plots indicate significance of correlation (assessed by a linear model). (ZIP) [file pone.0167944.s011.zip › S6_File/LACR_1448_real_dat.png]

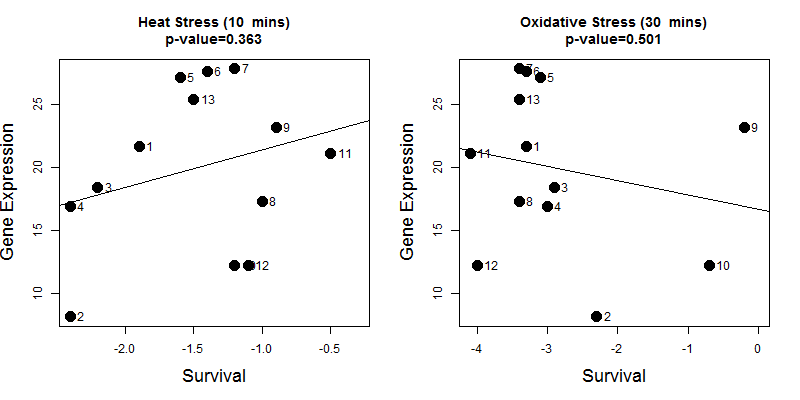

Supplement: S6 File — Expression levels of genes LACR_1383 –LACR_2610 and LACR_A01 –LACR_E8 plotted against survival after 10 minutes heat and 30 minutes oxidative stress. Survival is expressed as the difference of log CFU/ml after stress and before stress. Numbers indicate fermentations as presented in Table 1. P-values above the plots indicate significance of correlation (assessed by a linear model). (ZIP) [file pone.0167944.s011.zip › S6_File/LACR_1449_real_dat.png]

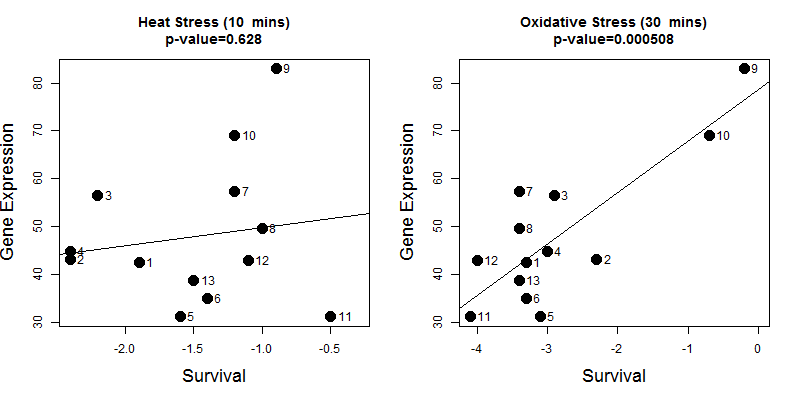

Supplement: S6 File — Expression levels of genes LACR_1383 –LACR_2610 and LACR_A01 –LACR_E8 plotted against survival after 10 minutes heat and 30 minutes oxidative stress. Survival is expressed as the difference of log CFU/ml after stress and before stress. Numbers indicate fermentations as presented in Table 1. P-values above the plots indicate significance of correlation (assessed by a linear model). (ZIP) [file pone.0167944.s011.zip › S6_File/LACR_1450_real_dat.png]

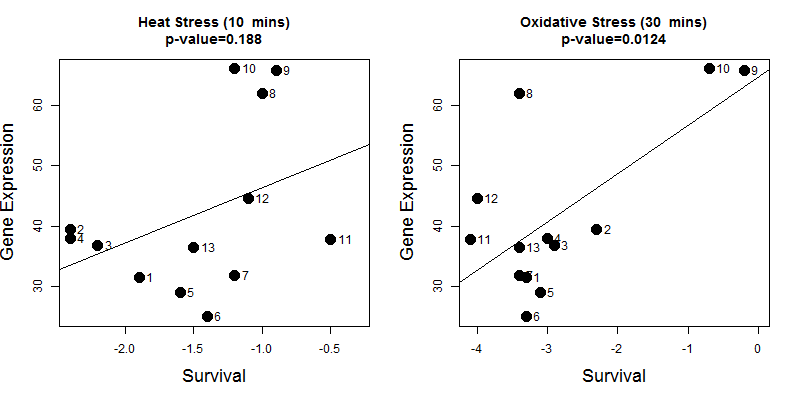

Supplement: S6 File — Expression levels of genes LACR_1383 –LACR_2610 and LACR_A01 –LACR_E8 plotted against survival after 10 minutes heat and 30 minutes oxidative stress. Survival is expressed as the difference of log CFU/ml after stress and before stress. Numbers indicate fermentations as presented in Table 1. P-values above the plots indicate significance of correlation (assessed by a linear model). (ZIP) [file pone.0167944.s011.zip › S6_File/LACR_1451_real_dat.png]

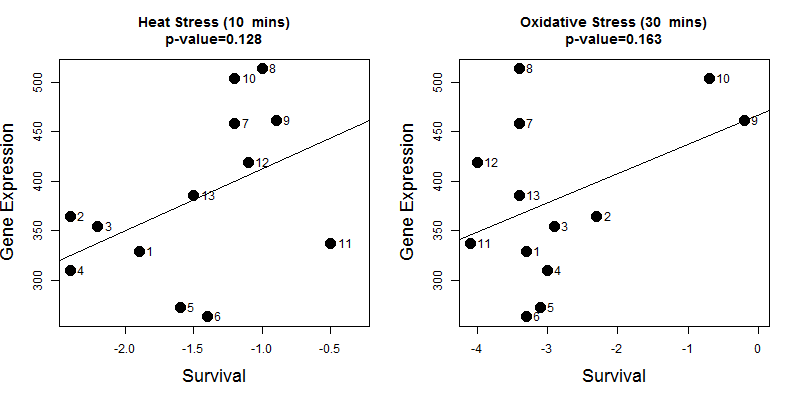

Supplement: S6 File — Expression levels of genes LACR_1383 –LACR_2610 and LACR_A01 –LACR_E8 plotted against survival after 10 minutes heat and 30 minutes oxidative stress. Survival is expressed as the difference of log CFU/ml after stress and before stress. Numbers indicate fermentations as presented in Table 1. P-values above the plots indicate significance of correlation (assessed by a linear model). (ZIP) [file pone.0167944.s011.zip › S6_File/LACR_1452_real_dat.png]

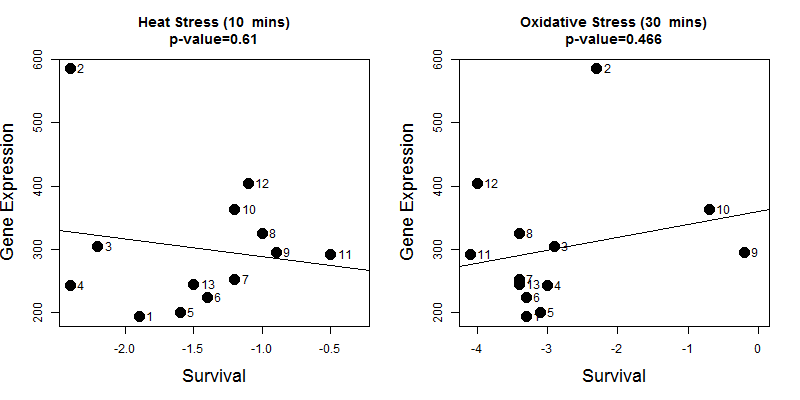

Supplement: S6 File — Expression levels of genes LACR_1383 –LACR_2610 and LACR_A01 –LACR_E8 plotted against survival after 10 minutes heat and 30 minutes oxidative stress. Survival is expressed as the difference of log CFU/ml after stress and before stress. Numbers indicate fermentations as presented in Table 1. P-values above the plots indicate significance of correlation (assessed by a linear model). (ZIP) [file pone.0167944.s011.zip › S6_File/LACR_1453_real_dat.png]

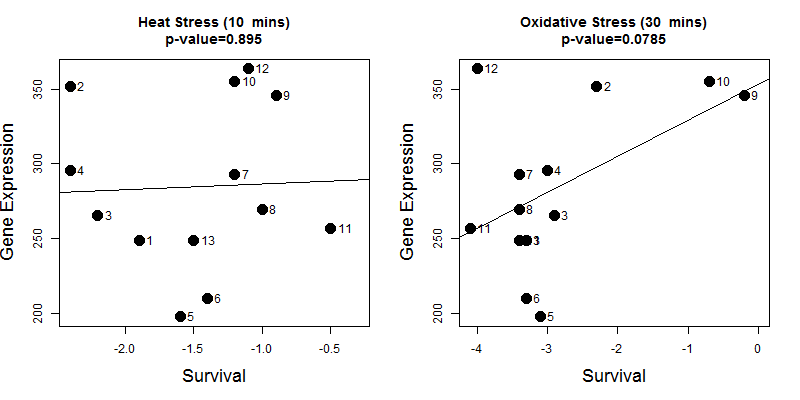

Supplement: S6 File — Expression levels of genes LACR_1383 –LACR_2610 and LACR_A01 –LACR_E8 plotted against survival after 10 minutes heat and 30 minutes oxidative stress. Survival is expressed as the difference of log CFU/ml after stress and before stress. Numbers indicate fermentations as presented in Table 1. P-values above the plots indicate significance of correlation (assessed by a linear model). (ZIP) [file pone.0167944.s011.zip › S6_File/LACR_1454_real_dat.png]

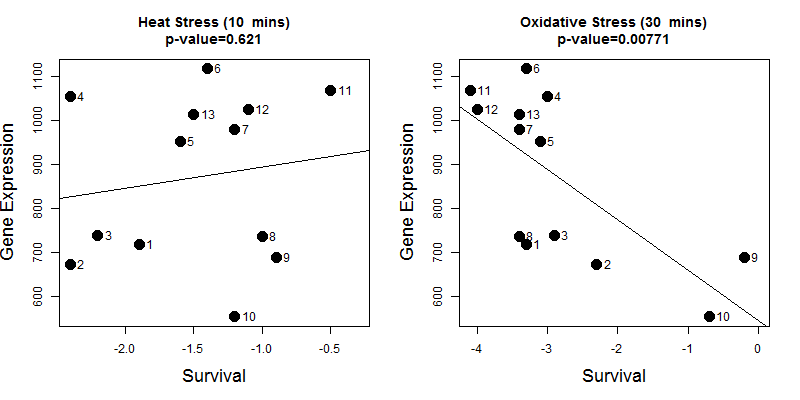

Supplement: S6 File — Expression levels of genes LACR_1383 –LACR_2610 and LACR_A01 –LACR_E8 plotted against survival after 10 minutes heat and 30 minutes oxidative stress. Survival is expressed as the difference of log CFU/ml after stress and before stress. Numbers indicate fermentations as presented in Table 1. P-values above the plots indicate significance of correlation (assessed by a linear model). (ZIP) [file pone.0167944.s011.zip › S6_File/LACR_1455_real_dat.png]

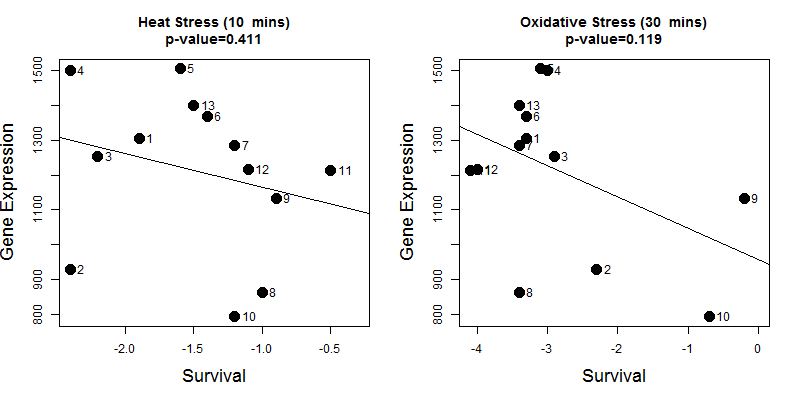

Supplement: S6 File — Expression levels of genes LACR_1383 –LACR_2610 and LACR_A01 –LACR_E8 plotted against survival after 10 minutes heat and 30 minutes oxidative stress. Survival is expressed as the difference of log CFU/ml after stress and before stress. Numbers indicate fermentations as presented in Table 1. P-values above the plots indicate significance of correlation (assessed by a linear model). (ZIP) [file pone.0167944.s011.zip › S6_File/LACR_1456_real_dat.png]

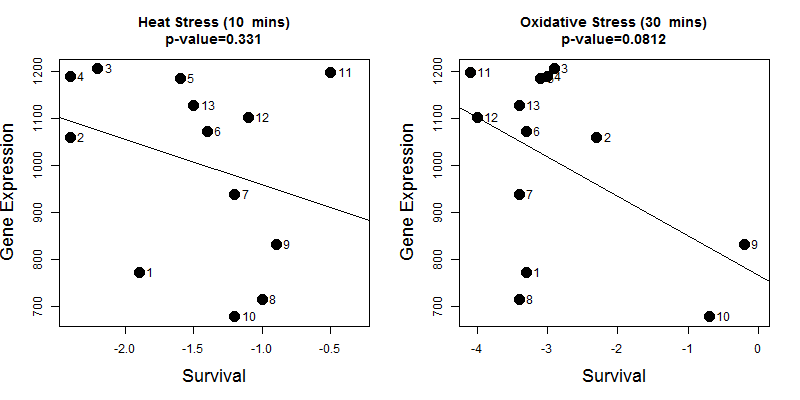

Supplement: S6 File — Expression levels of genes LACR_1383 –LACR_2610 and LACR_A01 –LACR_E8 plotted against survival after 10 minutes heat and 30 minutes oxidative stress. Survival is expressed as the difference of log CFU/ml after stress and before stress. Numbers indicate fermentations as presented in Table 1. P-values above the plots indicate significance of correlation (assessed by a linear model). (ZIP) [file pone.0167944.s011.zip › S6_File/LACR_1457_real_dat.png]

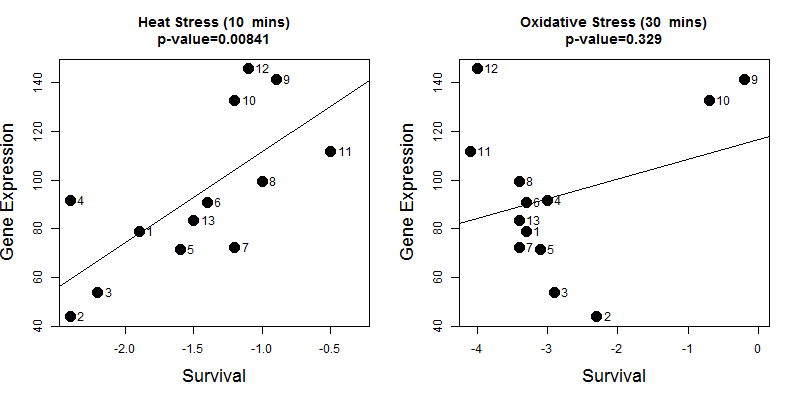

Supplement: S6 File — Expression levels of genes LACR_1383 –LACR_2610 and LACR_A01 –LACR_E8 plotted against survival after 10 minutes heat and 30 minutes oxidative stress. Survival is expressed as the difference of log CFU/ml after stress and before stress. Numbers indicate fermentations as presented in Table 1. P-values above the plots indicate significance of correlation (assessed by a linear model). (ZIP) [file pone.0167944.s011.zip › S6_File/LACR_1458_real_dat.png]

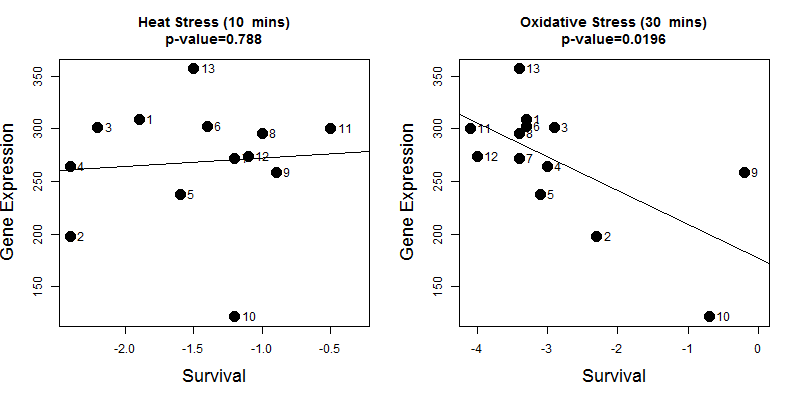

Supplement: S6 File — Expression levels of genes LACR_1383 –LACR_2610 and LACR_A01 –LACR_E8 plotted against survival after 10 minutes heat and 30 minutes oxidative stress. Survival is expressed as the difference of log CFU/ml after stress and before stress. Numbers indicate fermentations as presented in Table 1. P-values above the plots indicate significance of correlation (assessed by a linear model). (ZIP) [file pone.0167944.s011.zip › S6_File/LACR_1459_real_dat.png]

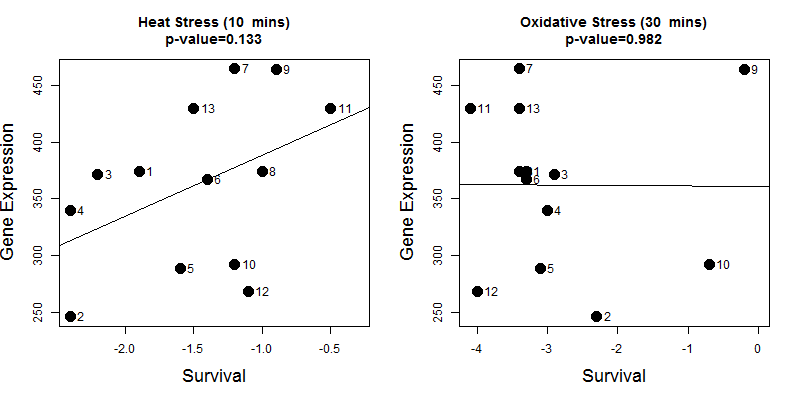

Supplement: S6 File — Expression levels of genes LACR_1383 –LACR_2610 and LACR_A01 –LACR_E8 plotted against survival after 10 minutes heat and 30 minutes oxidative stress. Survival is expressed as the difference of log CFU/ml after stress and before stress. Numbers indicate fermentations as presented in Table 1. P-values above the plots indicate significance of correlation (assessed by a linear model). (ZIP) [file pone.0167944.s011.zip › S6_File/LACR_1460_real_dat.png]

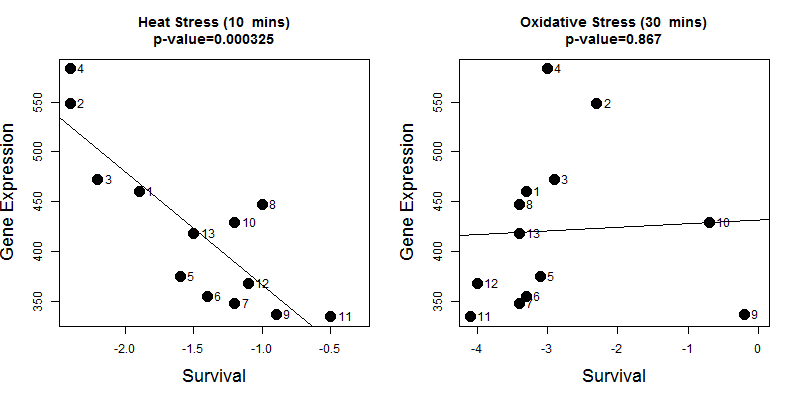

Supplement: S6 File — Expression levels of genes LACR_1383 –LACR_2610 and LACR_A01 –LACR_E8 plotted against survival after 10 minutes heat and 30 minutes oxidative stress. Survival is expressed as the difference of log CFU/ml after stress and before stress. Numbers indicate fermentations as presented in Table 1. P-values above the plots indicate significance of correlation (assessed by a linear model). (ZIP) [file pone.0167944.s011.zip › S6_File/LACR_1461_real_dat.png]

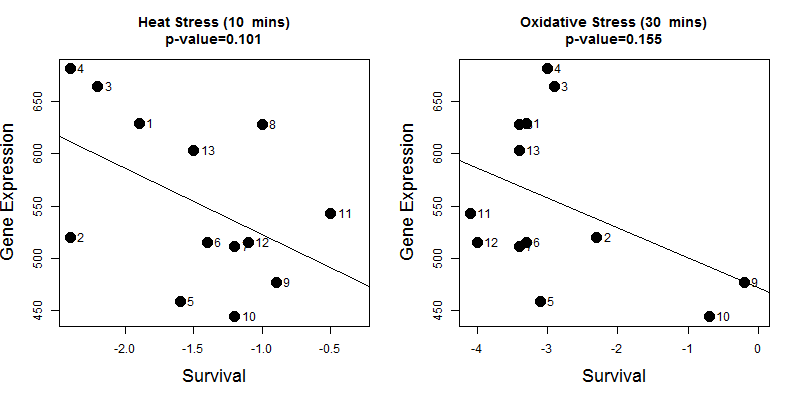

Supplement: S6 File — Expression levels of genes LACR_1383 –LACR_2610 and LACR_A01 –LACR_E8 plotted against survival after 10 minutes heat and 30 minutes oxidative stress. Survival is expressed as the difference of log CFU/ml after stress and before stress. Numbers indicate fermentations as presented in Table 1. P-values above the plots indicate significance of correlation (assessed by a linear model). (ZIP) [file pone.0167944.s011.zip › S6_File/LACR_1462_real_dat.png]

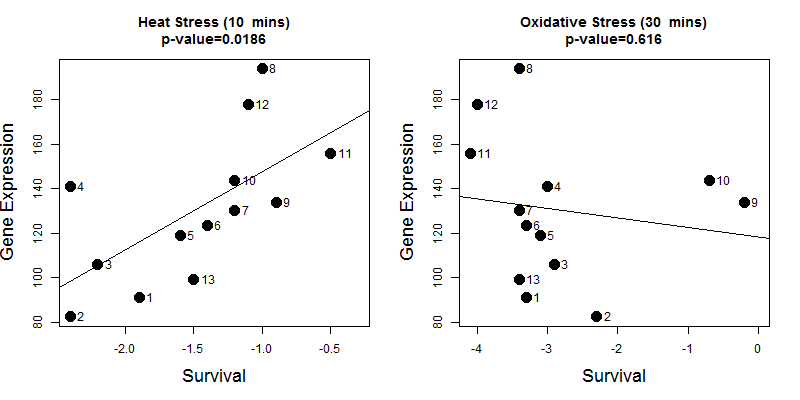

Supplement: S6 File — Expression levels of genes LACR_1383 –LACR_2610 and LACR_A01 –LACR_E8 plotted against survival after 10 minutes heat and 30 minutes oxidative stress. Survival is expressed as the difference of log CFU/ml after stress and before stress. Numbers indicate fermentations as presented in Table 1. P-values above the plots indicate significance of correlation (assessed by a linear model). (ZIP) [file pone.0167944.s011.zip › S6_File/LACR_1463_real_dat.png]

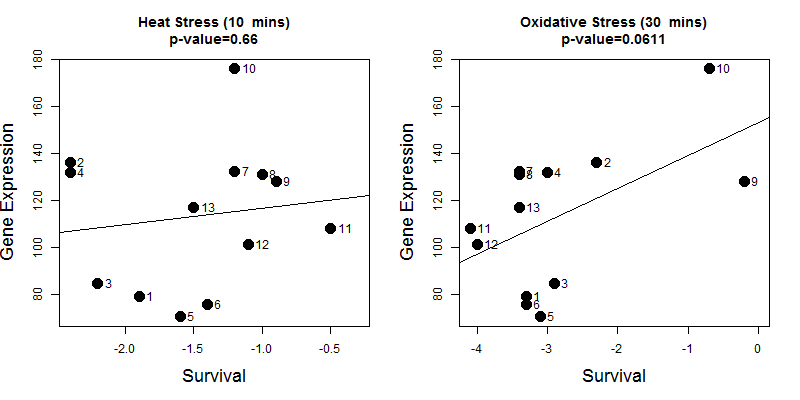

Supplement: S6 File — Expression levels of genes LACR_1383 –LACR_2610 and LACR_A01 –LACR_E8 plotted against survival after 10 minutes heat and 30 minutes oxidative stress. Survival is expressed as the difference of log CFU/ml after stress and before stress. Numbers indicate fermentations as presented in Table 1. P-values above the plots indicate significance of correlation (assessed by a linear model). (ZIP) [file pone.0167944.s011.zip › S6_File/LACR_1464_real_dat.png]

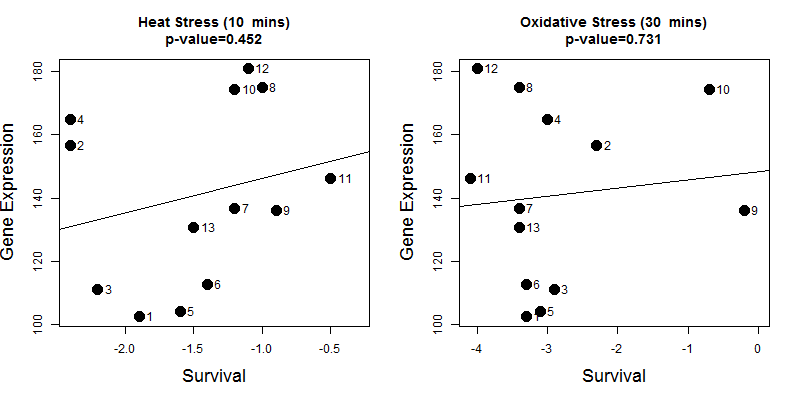

Supplement: S6 File — Expression levels of genes LACR_1383 –LACR_2610 and LACR_A01 –LACR_E8 plotted against survival after 10 minutes heat and 30 minutes oxidative stress. Survival is expressed as the difference of log CFU/ml after stress and before stress. Numbers indicate fermentations as presented in Table 1. P-values above the plots indicate significance of correlation (assessed by a linear model). (ZIP) [file pone.0167944.s011.zip › S6_File/LACR_1465_real_dat.png]

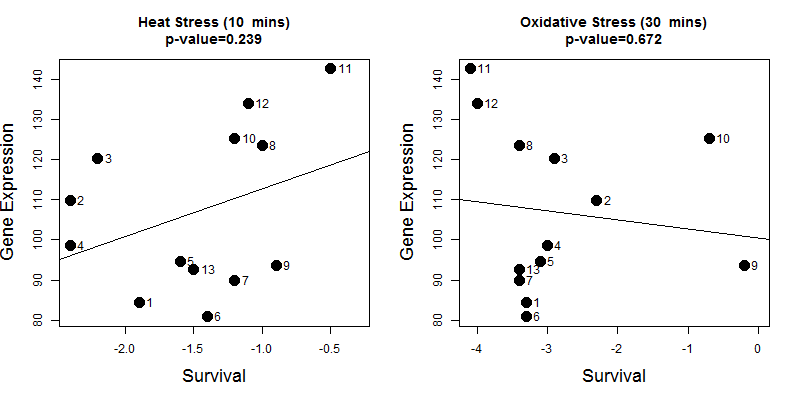

Supplement: S6 File — Expression levels of genes LACR_1383 –LACR_2610 and LACR_A01 –LACR_E8 plotted against survival after 10 minutes heat and 30 minutes oxidative stress. Survival is expressed as the difference of log CFU/ml after stress and before stress. Numbers indicate fermentations as presented in Table 1. P-values above the plots indicate significance of correlation (assessed by a linear model). (ZIP) [file pone.0167944.s011.zip › S6_File/LACR_1466_real_dat.png]

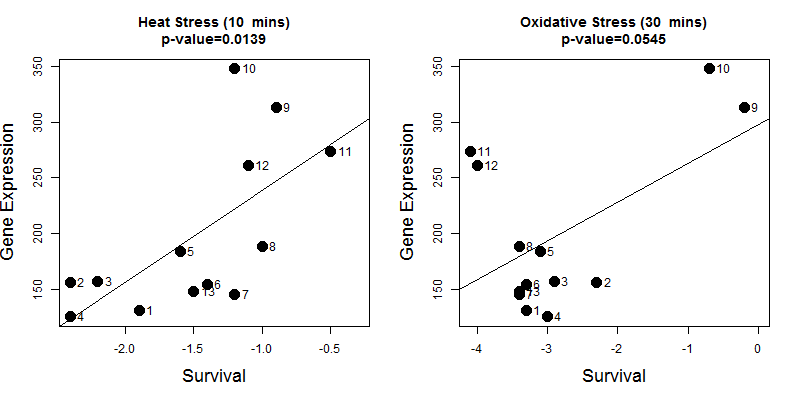

Supplement: S6 File — Expression levels of genes LACR_1383 –LACR_2610 and LACR_A01 –LACR_E8 plotted against survival after 10 minutes heat and 30 minutes oxidative stress. Survival is expressed as the difference of log CFU/ml after stress and before stress. Numbers indicate fermentations as presented in Table 1. P-values above the plots indicate significance of correlation (assessed by a linear model). (ZIP) [file pone.0167944.s011.zip › S6_File/LACR_1467_real_dat.png]

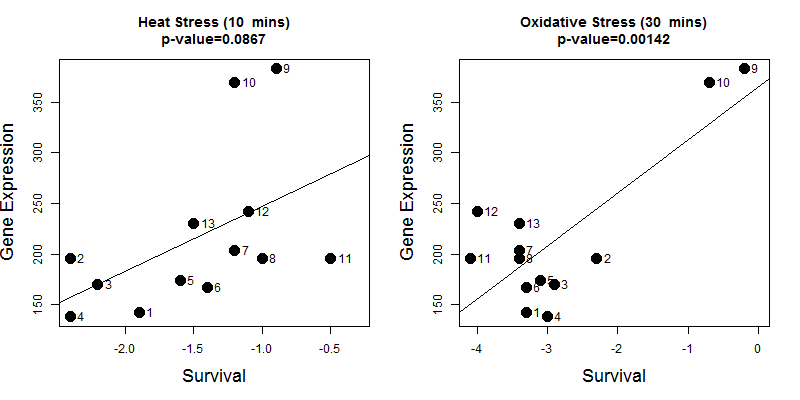

Supplement: S6 File — Expression levels of genes LACR_1383 –LACR_2610 and LACR_A01 –LACR_E8 plotted against survival after 10 minutes heat and 30 minutes oxidative stress. Survival is expressed as the difference of log CFU/ml after stress and before stress. Numbers indicate fermentations as presented in Table 1. P-values above the plots indicate significance of correlation (assessed by a linear model). (ZIP) [file pone.0167944.s011.zip › S6_File/LACR_1468_real_dat.png]

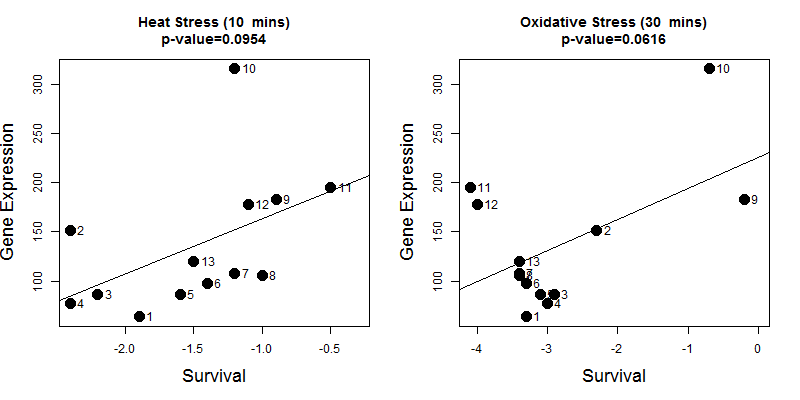

Supplement: S6 File — Expression levels of genes LACR_1383 –LACR_2610 and LACR_A01 –LACR_E8 plotted against survival after 10 minutes heat and 30 minutes oxidative stress. Survival is expressed as the difference of log CFU/ml after stress and before stress. Numbers indicate fermentations as presented in Table 1. P-values above the plots indicate significance of correlation (assessed by a linear model). (ZIP) [file pone.0167944.s011.zip › S6_File/LACR_1469_real_dat.png]

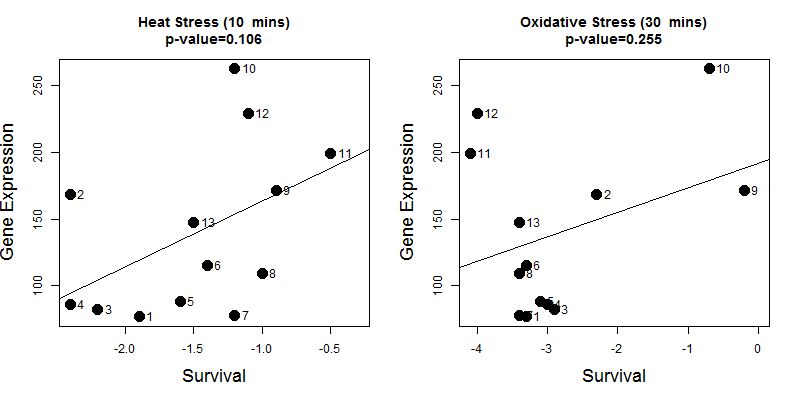

Supplement: S6 File — Expression levels of genes LACR_1383 –LACR_2610 and LACR_A01 –LACR_E8 plotted against survival after 10 minutes heat and 30 minutes oxidative stress. Survival is expressed as the difference of log CFU/ml after stress and before stress. Numbers indicate fermentations as presented in Table 1. P-values above the plots indicate significance of correlation (assessed by a linear model). (ZIP) [file pone.0167944.s011.zip › S6_File/LACR_1470_real_dat.png]

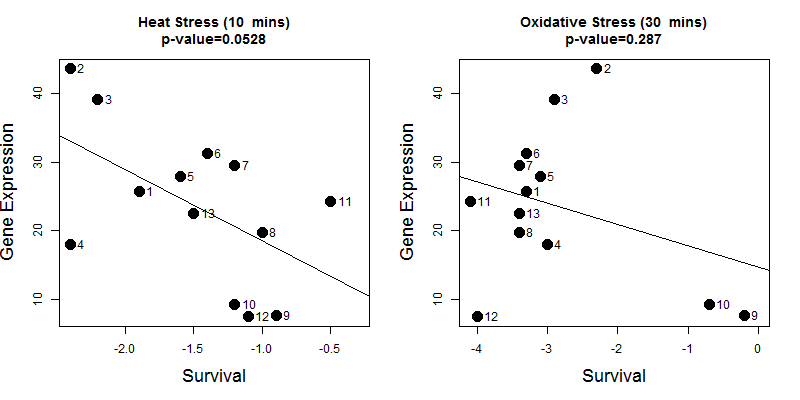

Supplement: S6 File — Expression levels of genes LACR_1383 –LACR_2610 and LACR_A01 –LACR_E8 plotted against survival after 10 minutes heat and 30 minutes oxidative stress. Survival is expressed as the difference of log CFU/ml after stress and before stress. Numbers indicate fermentations as presented in Table 1. P-values above the plots indicate significance of correlation (assessed by a linear model). (ZIP) [file pone.0167944.s011.zip › S6_File/LACR_1471_real_dat.png]

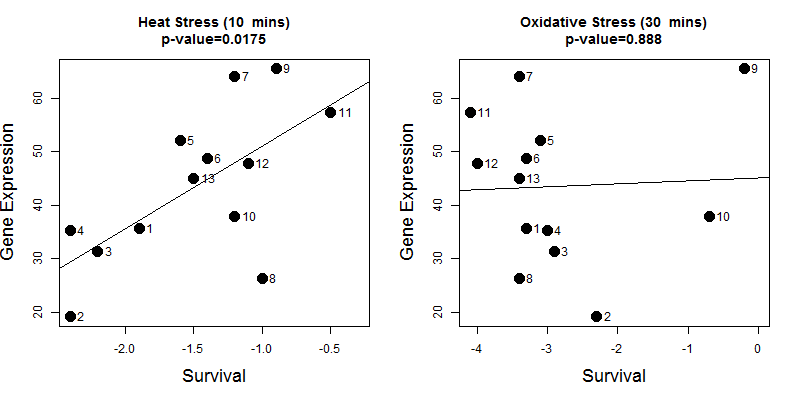

Supplement: S6 File — Expression levels of genes LACR_1383 –LACR_2610 and LACR_A01 –LACR_E8 plotted against survival after 10 minutes heat and 30 minutes oxidative stress. Survival is expressed as the difference of log CFU/ml after stress and before stress. Numbers indicate fermentations as presented in Table 1. P-values above the plots indicate significance of correlation (assessed by a linear model). (ZIP) [file pone.0167944.s011.zip › S6_File/LACR_1472_real_dat.png]

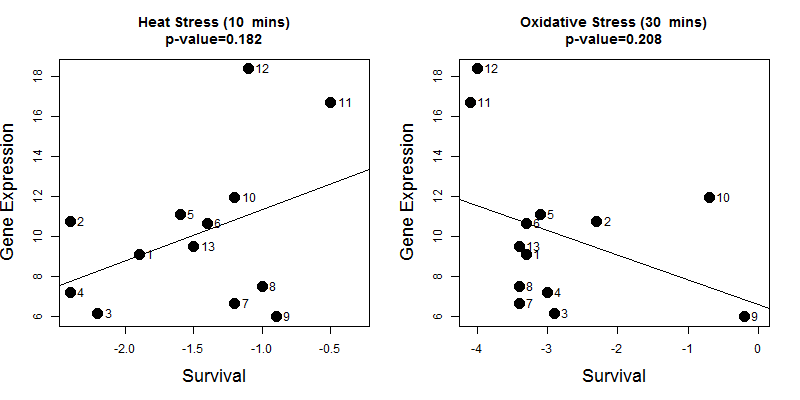

Supplement: S6 File — Expression levels of genes LACR_1383 –LACR_2610 and LACR_A01 –LACR_E8 plotted against survival after 10 minutes heat and 30 minutes oxidative stress. Survival is expressed as the difference of log CFU/ml after stress and before stress. Numbers indicate fermentations as presented in Table 1. P-values above the plots indicate significance of correlation (assessed by a linear model). (ZIP) [file pone.0167944.s011.zip › S6_File/LACR_1473_real_dat.png]

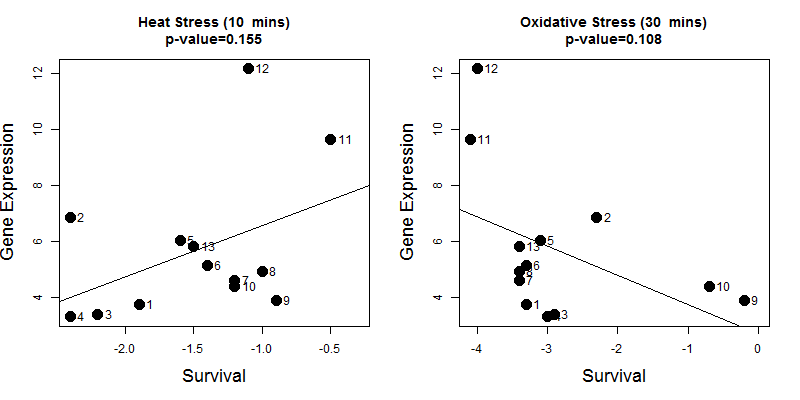

Supplement: S6 File — Expression levels of genes LACR_1383 –LACR_2610 and LACR_A01 –LACR_E8 plotted against survival after 10 minutes heat and 30 minutes oxidative stress. Survival is expressed as the difference of log CFU/ml after stress and before stress. Numbers indicate fermentations as presented in Table 1. P-values above the plots indicate significance of correlation (assessed by a linear model). (ZIP) [file pone.0167944.s011.zip › S6_File/LACR_1474_real_dat.png]

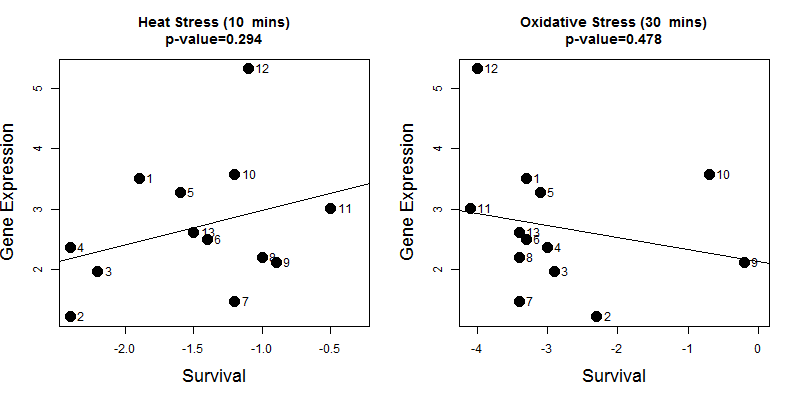

Supplement: S6 File — Expression levels of genes LACR_1383 –LACR_2610 and LACR_A01 –LACR_E8 plotted against survival after 10 minutes heat and 30 minutes oxidative stress. Survival is expressed as the difference of log CFU/ml after stress and before stress. Numbers indicate fermentations as presented in Table 1. P-values above the plots indicate significance of correlation (assessed by a linear model). (ZIP) [file pone.0167944.s011.zip › S6_File/LACR_1475_real_dat.png]

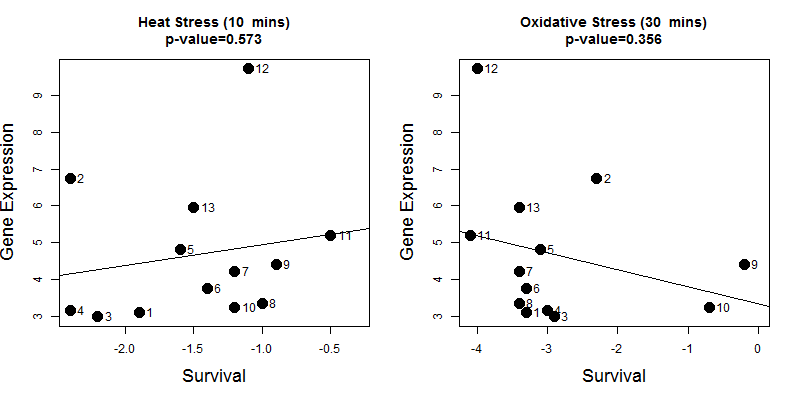

Supplement: S6 File — Expression levels of genes LACR_1383 –LACR_2610 and LACR_A01 –LACR_E8 plotted against survival after 10 minutes heat and 30 minutes oxidative stress. Survival is expressed as the difference of log CFU/ml after stress and before stress. Numbers indicate fermentations as presented in Table 1. P-values above the plots indicate significance of correlation (assessed by a linear model). (ZIP) [file pone.0167944.s011.zip › S6_File/LACR_1476_real_dat.png]

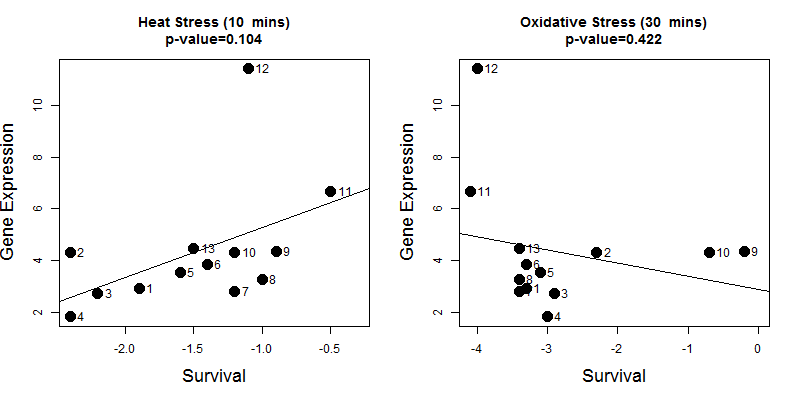

Supplement: S6 File — Expression levels of genes LACR_1383 –LACR_2610 and LACR_A01 –LACR_E8 plotted against survival after 10 minutes heat and 30 minutes oxidative stress. Survival is expressed as the difference of log CFU/ml after stress and before stress. Numbers indicate fermentations as presented in Table 1. P-values above the plots indicate significance of correlation (assessed by a linear model). (ZIP) [file pone.0167944.s011.zip › S6_File/LACR_1477_real_dat.png]

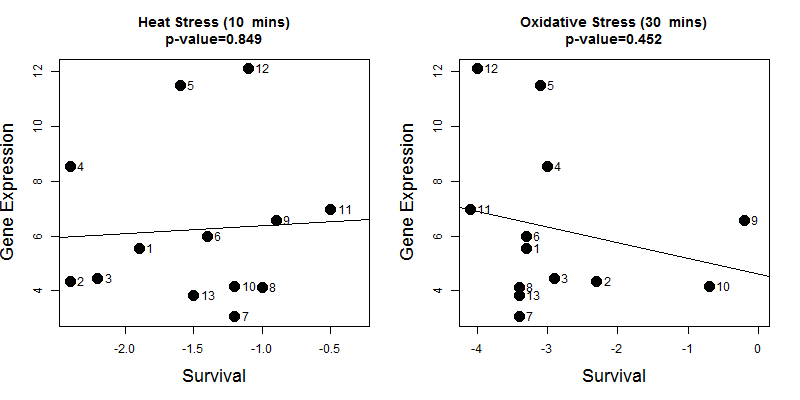

Supplement: S6 File — Expression levels of genes LACR_1383 –LACR_2610 and LACR_A01 –LACR_E8 plotted against survival after 10 minutes heat and 30 minutes oxidative stress. Survival is expressed as the difference of log CFU/ml after stress and before stress. Numbers indicate fermentations as presented in Table 1. P-values above the plots indicate significance of correlation (assessed by a linear model). (ZIP) [file pone.0167944.s011.zip › S6_File/LACR_1478_real_dat.png]

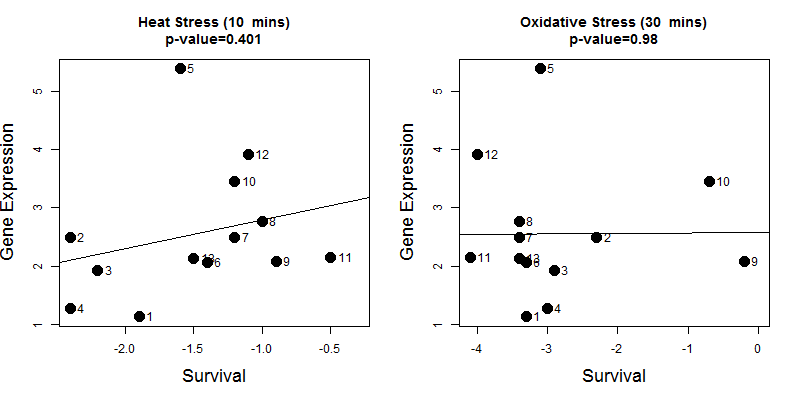

Supplement: S6 File — Expression levels of genes LACR_1383 –LACR_2610 and LACR_A01 –LACR_E8 plotted against survival after 10 minutes heat and 30 minutes oxidative stress. Survival is expressed as the difference of log CFU/ml after stress and before stress. Numbers indicate fermentations as presented in Table 1. P-values above the plots indicate significance of correlation (assessed by a linear model). (ZIP) [file pone.0167944.s011.zip › S6_File/LACR_1479_real_dat.png]

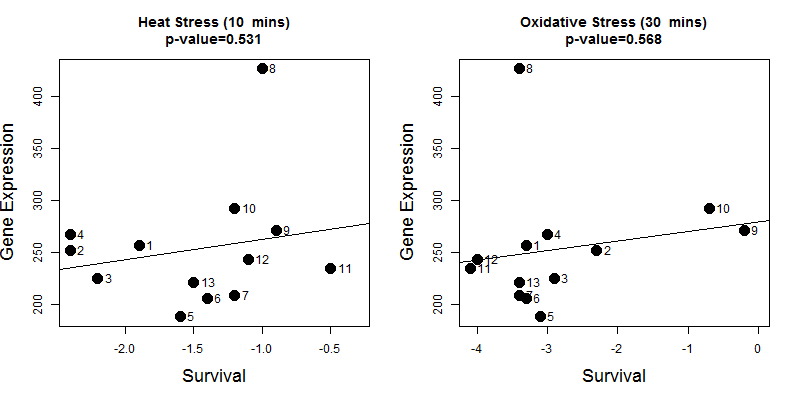

Supplement: S6 File — Expression levels of genes LACR_1383 –LACR_2610 and LACR_A01 –LACR_E8 plotted against survival after 10 minutes heat and 30 minutes oxidative stress. Survival is expressed as the difference of log CFU/ml after stress and before stress. Numbers indicate fermentations as presented in Table 1. P-values above the plots indicate significance of correlation (assessed by a linear model). (ZIP) [file pone.0167944.s011.zip › S6_File/LACR_1481_real_dat.png]

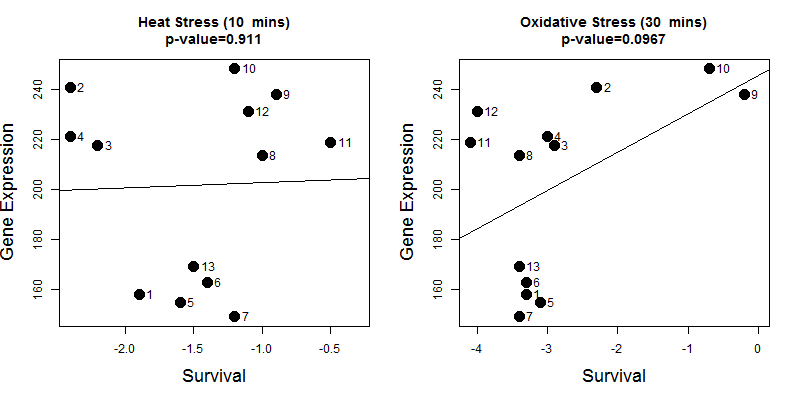

Supplement: S6 File — Expression levels of genes LACR_1383 –LACR_2610 and LACR_A01 –LACR_E8 plotted against survival after 10 minutes heat and 30 minutes oxidative stress. Survival is expressed as the difference of log CFU/ml after stress and before stress. Numbers indicate fermentations as presented in Table 1. P-values above the plots indicate significance of correlation (assessed by a linear model). (ZIP) [file pone.0167944.s011.zip › S6_File/LACR_1482_real_dat.png]

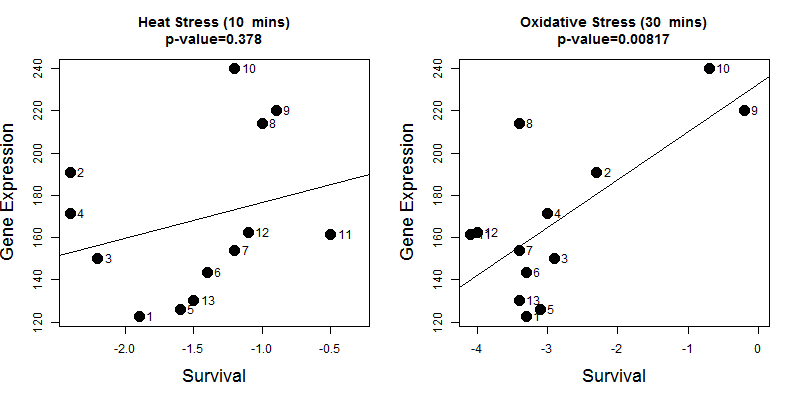

Supplement: S6 File — Expression levels of genes LACR_1383 –LACR_2610 and LACR_A01 –LACR_E8 plotted against survival after 10 minutes heat and 30 minutes oxidative stress. Survival is expressed as the difference of log CFU/ml after stress and before stress. Numbers indicate fermentations as presented in Table 1. P-values above the plots indicate significance of correlation (assessed by a linear model). (ZIP) [file pone.0167944.s011.zip › S6_File/LACR_1483_real_dat.png]

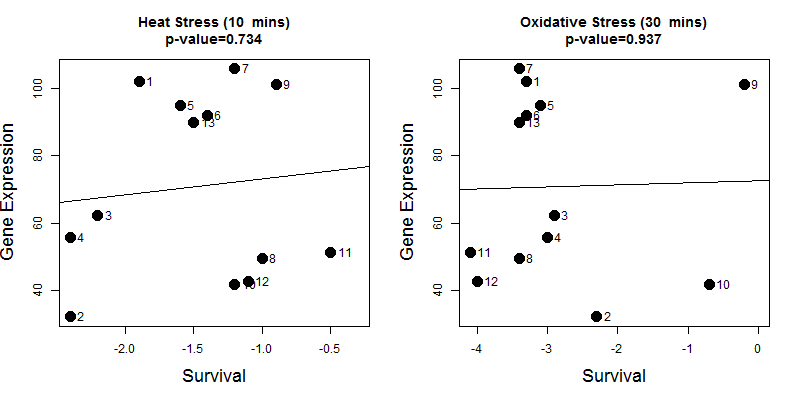

Supplement: S6 File — Expression levels of genes LACR_1383 –LACR_2610 and LACR_A01 –LACR_E8 plotted against survival after 10 minutes heat and 30 minutes oxidative stress. Survival is expressed as the difference of log CFU/ml after stress and before stress. Numbers indicate fermentations as presented in Table 1. P-values above the plots indicate significance of correlation (assessed by a linear model). (ZIP) [file pone.0167944.s011.zip › S6_File/LACR_1484_real_dat.png]

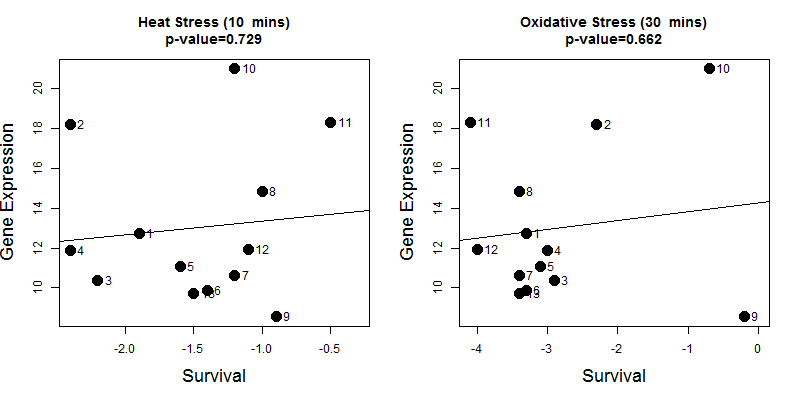

Supplement: S6 File — Expression levels of genes LACR_1383 –LACR_2610 and LACR_A01 –LACR_E8 plotted against survival after 10 minutes heat and 30 minutes oxidative stress. Survival is expressed as the difference of log CFU/ml after stress and before stress. Numbers indicate fermentations as presented in Table 1. P-values above the plots indicate significance of correlation (assessed by a linear model). (ZIP) [file pone.0167944.s011.zip › S6_File/LACR_1485_real_dat.png]

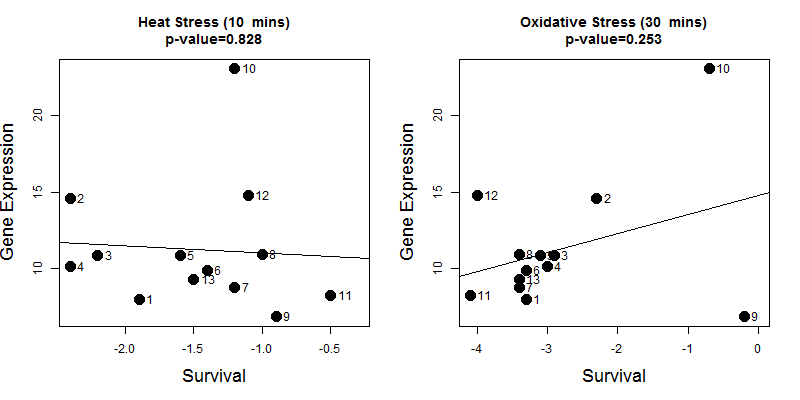

Supplement: S6 File — Expression levels of genes LACR_1383 –LACR_2610 and LACR_A01 –LACR_E8 plotted against survival after 10 minutes heat and 30 minutes oxidative stress. Survival is expressed as the difference of log CFU/ml after stress and before stress. Numbers indicate fermentations as presented in Table 1. P-values above the plots indicate significance of correlation (assessed by a linear model). (ZIP) [file pone.0167944.s011.zip › S6_File/LACR_1486_real_dat.png]

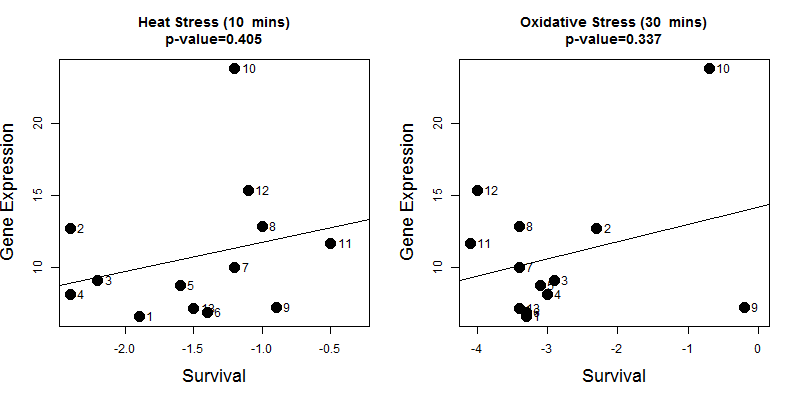

Supplement: S6 File — Expression levels of genes LACR_1383 –LACR_2610 and LACR_A01 –LACR_E8 plotted against survival after 10 minutes heat and 30 minutes oxidative stress. Survival is expressed as the difference of log CFU/ml after stress and before stress. Numbers indicate fermentations as presented in Table 1. P-values above the plots indicate significance of correlation (assessed by a linear model). (ZIP) [file pone.0167944.s011.zip › S6_File/LACR_1487_real_dat.png]

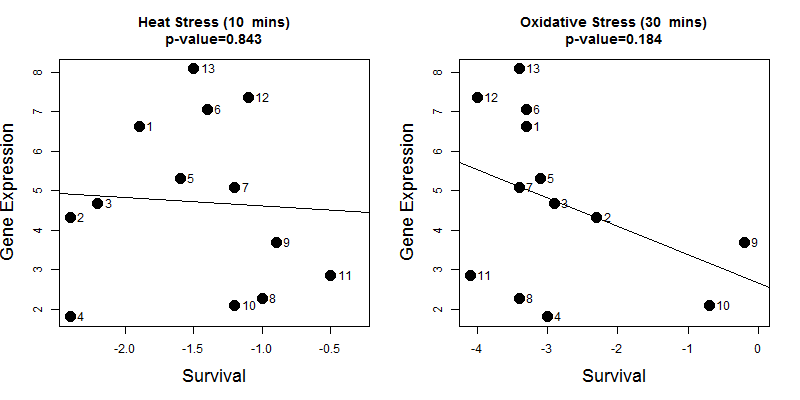

Supplement: S6 File — Expression levels of genes LACR_1383 –LACR_2610 and LACR_A01 –LACR_E8 plotted against survival after 10 minutes heat and 30 minutes oxidative stress. Survival is expressed as the difference of log CFU/ml after stress and before stress. Numbers indicate fermentations as presented in Table 1. P-values above the plots indicate significance of correlation (assessed by a linear model). (ZIP) [file pone.0167944.s011.zip › S6_File/LACR_1488_real_dat.png]

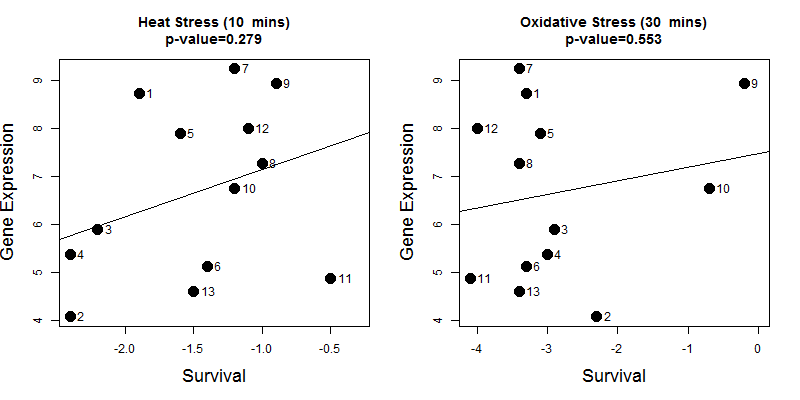

Supplement: S6 File — Expression levels of genes LACR_1383 –LACR_2610 and LACR_A01 –LACR_E8 plotted against survival after 10 minutes heat and 30 minutes oxidative stress. Survival is expressed as the difference of log CFU/ml after stress and before stress. Numbers indicate fermentations as presented in Table 1. P-values above the plots indicate significance of correlation (assessed by a linear model). (ZIP) [file pone.0167944.s011.zip › S6_File/LACR_1489_real_dat.png]

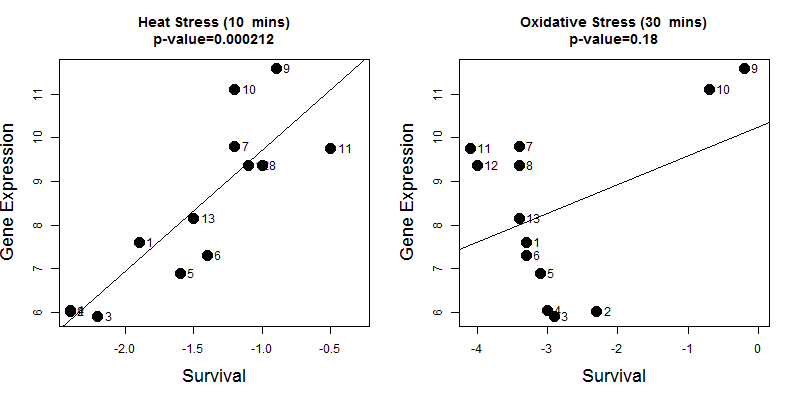

Supplement: S6 File — Expression levels of genes LACR_1383 –LACR_2610 and LACR_A01 –LACR_E8 plotted against survival after 10 minutes heat and 30 minutes oxidative stress. Survival is expressed as the difference of log CFU/ml after stress and before stress. Numbers indicate fermentations as presented in Table 1. P-values above the plots indicate significance of correlation (assessed by a linear model). (ZIP) [file pone.0167944.s011.zip › S6_File/LACR_1490_real_dat.png]
