# Supplementary figures and images for: Strain-Dependent Transcriptome Signatures for Robustness in Lactococcus lactis (part 10 of 13)
Source: PLoS One. 2016 Dec 14;11(12):e0167944. doi: 10.1371/journal.pone.0167944 (PMC5156439; doi:10.1371/journal.pone.0167944)

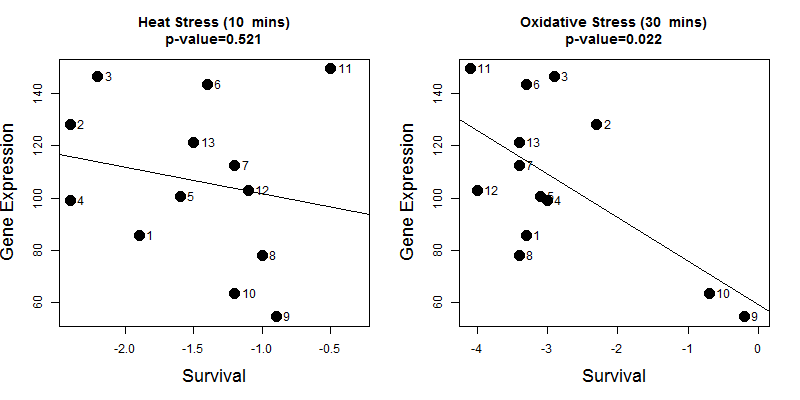

Supplement: S5 File — Expression levels of genes LACR_0001 –LACR_1382 plotted against survival after 10 minutes heat and 30 minutes oxidative stress. Survival is expressed as the difference of log CFU/ml after stress and before stress. Numbers indicate fermentations as presented in Table 1. P-values above the plots indicate significance of correlation (assessed by a linear model). (ZIP) [file pone.0167944.s010.zip › S5_File/LACR_0113_real_dat.png]

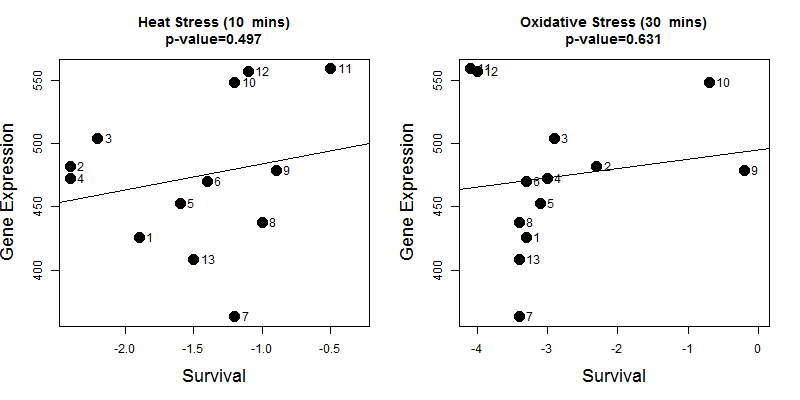

Supplement: S5 File — Expression levels of genes LACR_0001 –LACR_1382 plotted against survival after 10 minutes heat and 30 minutes oxidative stress. Survival is expressed as the difference of log CFU/ml after stress and before stress. Numbers indicate fermentations as presented in Table 1. P-values above the plots indicate significance of correlation (assessed by a linear model). (ZIP) [file pone.0167944.s010.zip › S5_File/LACR_0115_real_dat.png]

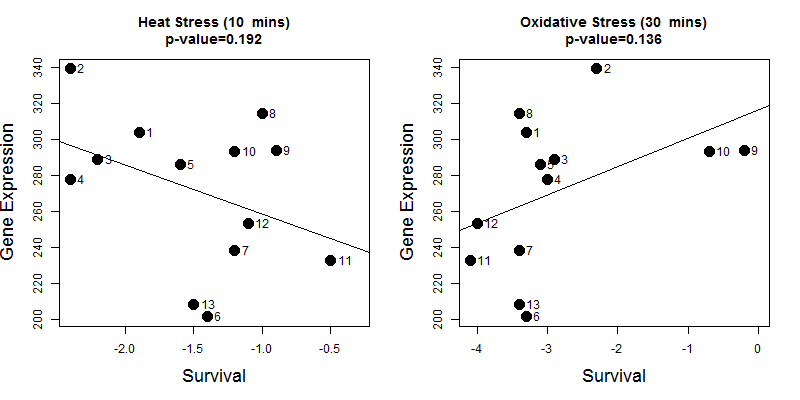

Supplement: S5 File — Expression levels of genes LACR_0001 –LACR_1382 plotted against survival after 10 minutes heat and 30 minutes oxidative stress. Survival is expressed as the difference of log CFU/ml after stress and before stress. Numbers indicate fermentations as presented in Table 1. P-values above the plots indicate significance of correlation (assessed by a linear model). (ZIP) [file pone.0167944.s010.zip › S5_File/LACR_0118_real_dat.png]

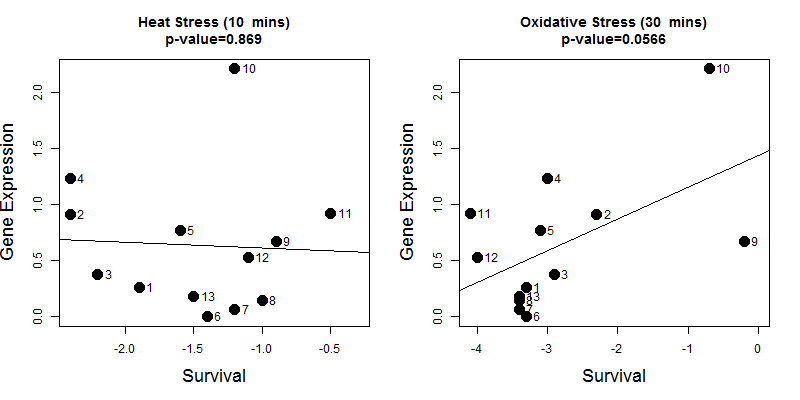

Supplement: S5 File — Expression levels of genes LACR_0001 –LACR_1382 plotted against survival after 10 minutes heat and 30 minutes oxidative stress. Survival is expressed as the difference of log CFU/ml after stress and before stress. Numbers indicate fermentations as presented in Table 1. P-values above the plots indicate significance of correlation (assessed by a linear model). (ZIP) [file pone.0167944.s010.zip › S5_File/LACR_0119_real_dat.png]

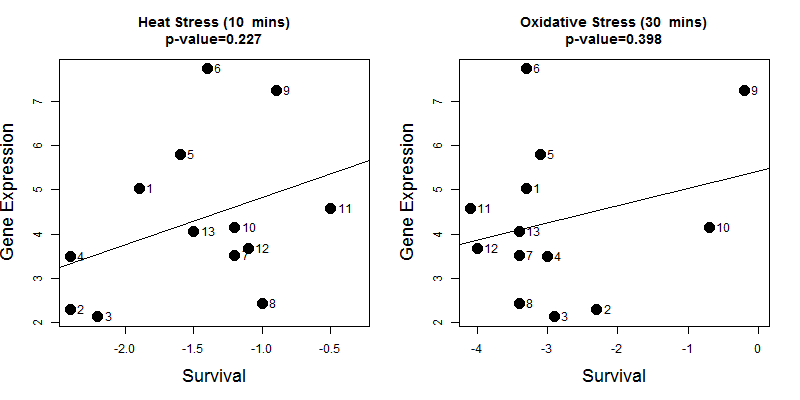

Supplement: S5 File — Expression levels of genes LACR_0001 –LACR_1382 plotted against survival after 10 minutes heat and 30 minutes oxidative stress. Survival is expressed as the difference of log CFU/ml after stress and before stress. Numbers indicate fermentations as presented in Table 1. P-values above the plots indicate significance of correlation (assessed by a linear model). (ZIP) [file pone.0167944.s010.zip › S5_File/LACR_0120_real_dat.png]

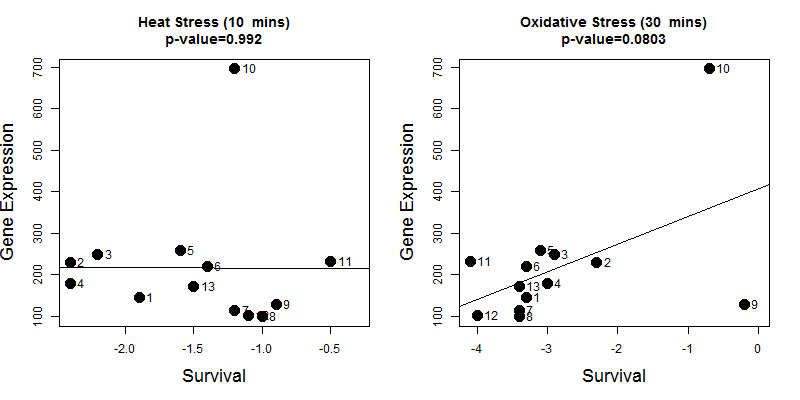

Supplement: S5 File — Expression levels of genes LACR_0001 –LACR_1382 plotted against survival after 10 minutes heat and 30 minutes oxidative stress. Survival is expressed as the difference of log CFU/ml after stress and before stress. Numbers indicate fermentations as presented in Table 1. P-values above the plots indicate significance of correlation (assessed by a linear model). (ZIP) [file pone.0167944.s010.zip › S5_File/LACR_0125_real_dat.png]

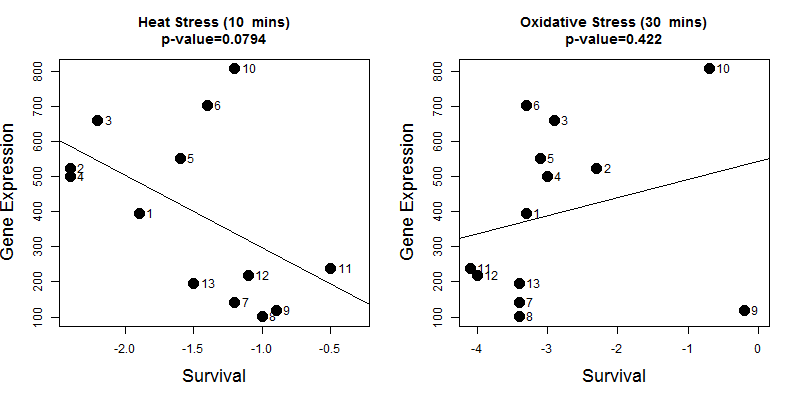

Supplement: S5 File — Expression levels of genes LACR_0001 –LACR_1382 plotted against survival after 10 minutes heat and 30 minutes oxidative stress. Survival is expressed as the difference of log CFU/ml after stress and before stress. Numbers indicate fermentations as presented in Table 1. P-values above the plots indicate significance of correlation (assessed by a linear model). (ZIP) [file pone.0167944.s010.zip › S5_File/LACR_0126_real_dat.png]

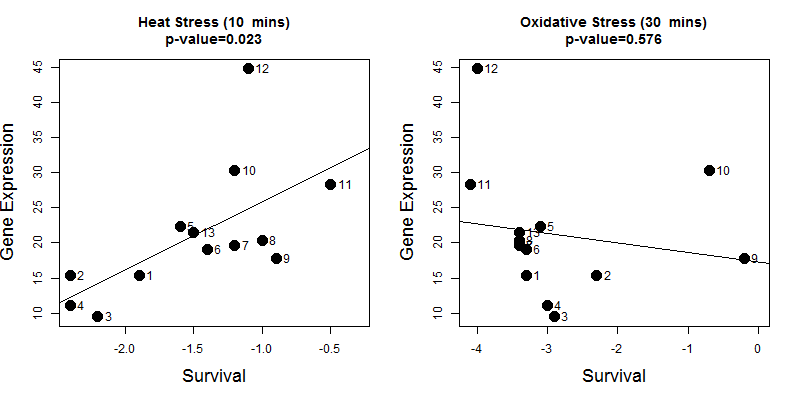

Supplement: S5 File — Expression levels of genes LACR_0001 –LACR_1382 plotted against survival after 10 minutes heat and 30 minutes oxidative stress. Survival is expressed as the difference of log CFU/ml after stress and before stress. Numbers indicate fermentations as presented in Table 1. P-values above the plots indicate significance of correlation (assessed by a linear model). (ZIP) [file pone.0167944.s010.zip › S5_File/LACR_0127_real_dat.png]

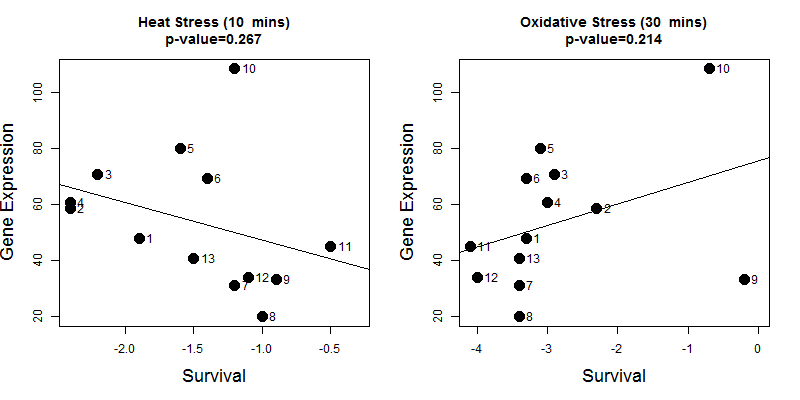

Supplement: S5 File — Expression levels of genes LACR_0001 –LACR_1382 plotted against survival after 10 minutes heat and 30 minutes oxidative stress. Survival is expressed as the difference of log CFU/ml after stress and before stress. Numbers indicate fermentations as presented in Table 1. P-values above the plots indicate significance of correlation (assessed by a linear model). (ZIP) [file pone.0167944.s010.zip › S5_File/LACR_0128_real_dat.png]

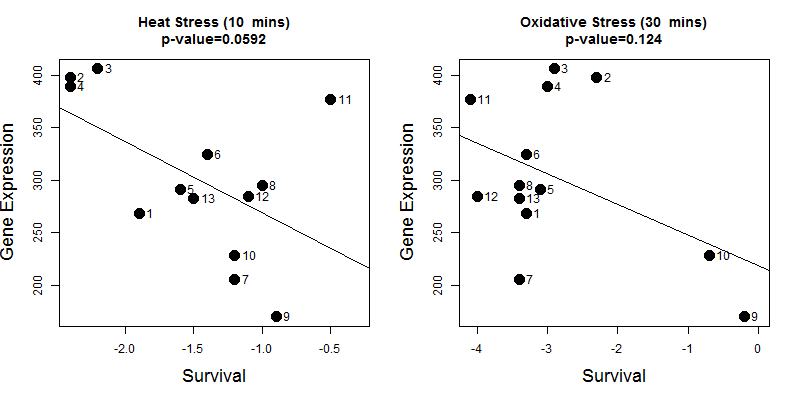

Supplement: S5 File — Expression levels of genes LACR_0001 –LACR_1382 plotted against survival after 10 minutes heat and 30 minutes oxidative stress. Survival is expressed as the difference of log CFU/ml after stress and before stress. Numbers indicate fermentations as presented in Table 1. P-values above the plots indicate significance of correlation (assessed by a linear model). (ZIP) [file pone.0167944.s010.zip › S5_File/LACR_0129_real_dat.png]

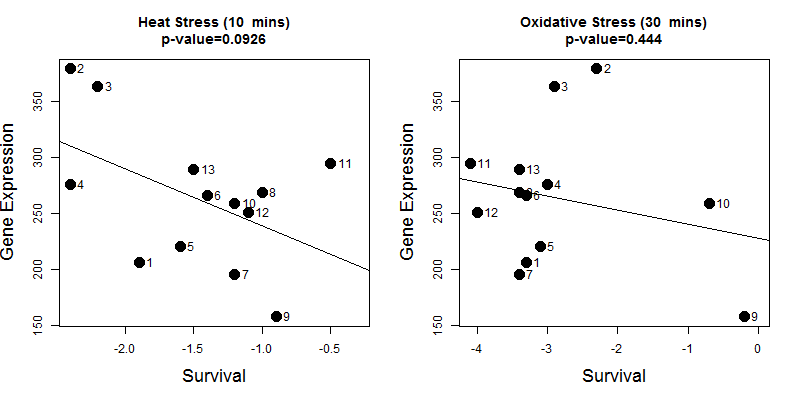

Supplement: S5 File — Expression levels of genes LACR_0001 –LACR_1382 plotted against survival after 10 minutes heat and 30 minutes oxidative stress. Survival is expressed as the difference of log CFU/ml after stress and before stress. Numbers indicate fermentations as presented in Table 1. P-values above the plots indicate significance of correlation (assessed by a linear model). (ZIP) [file pone.0167944.s010.zip › S5_File/LACR_0130_real_dat.png]

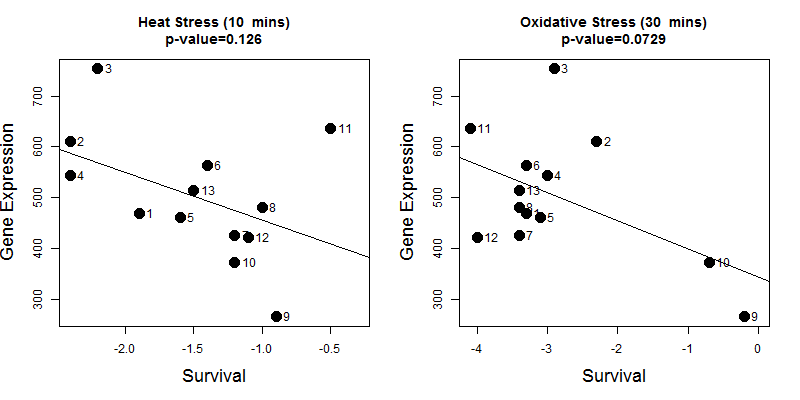

Supplement: S5 File — Expression levels of genes LACR_0001 –LACR_1382 plotted against survival after 10 minutes heat and 30 minutes oxidative stress. Survival is expressed as the difference of log CFU/ml after stress and before stress. Numbers indicate fermentations as presented in Table 1. P-values above the plots indicate significance of correlation (assessed by a linear model). (ZIP) [file pone.0167944.s010.zip › S5_File/LACR_0131_real_dat.png]

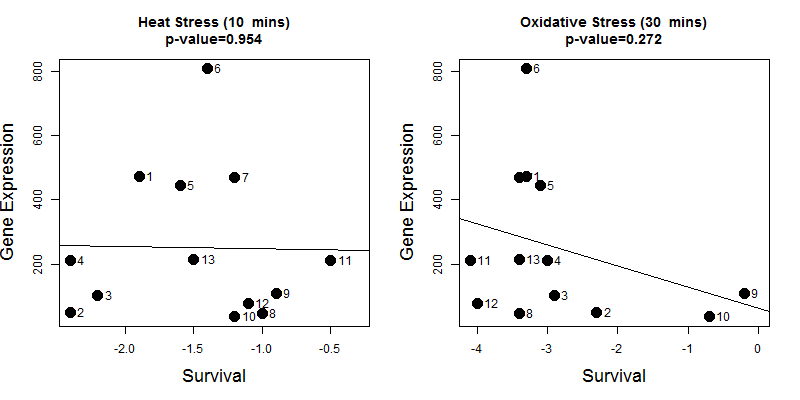

Supplement: S5 File — Expression levels of genes LACR_0001 –LACR_1382 plotted against survival after 10 minutes heat and 30 minutes oxidative stress. Survival is expressed as the difference of log CFU/ml after stress and before stress. Numbers indicate fermentations as presented in Table 1. P-values above the plots indicate significance of correlation (assessed by a linear model). (ZIP) [file pone.0167944.s010.zip › S5_File/LACR_0132_real_dat.png]

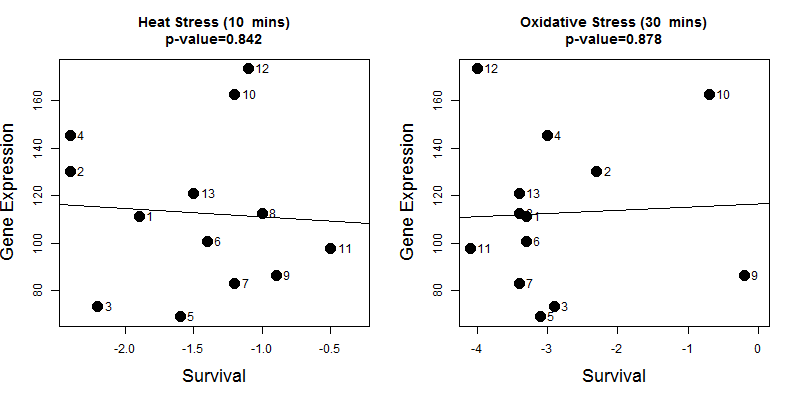

Supplement: S5 File — Expression levels of genes LACR_0001 –LACR_1382 plotted against survival after 10 minutes heat and 30 minutes oxidative stress. Survival is expressed as the difference of log CFU/ml after stress and before stress. Numbers indicate fermentations as presented in Table 1. P-values above the plots indicate significance of correlation (assessed by a linear model). (ZIP) [file pone.0167944.s010.zip › S5_File/LACR_0133_real_dat.png]

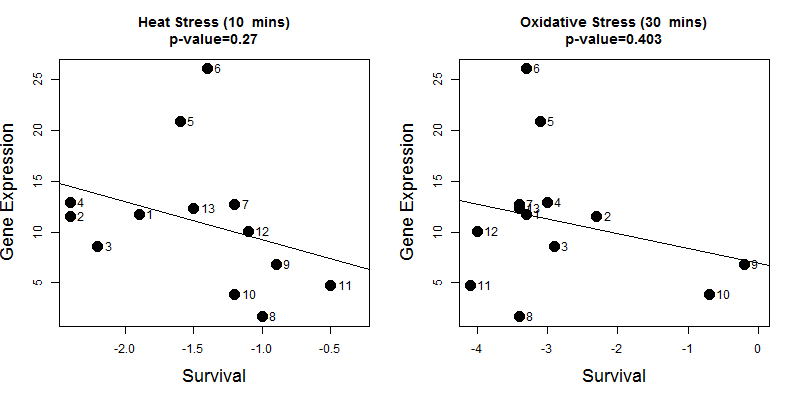

Supplement: S5 File — Expression levels of genes LACR_0001 –LACR_1382 plotted against survival after 10 minutes heat and 30 minutes oxidative stress. Survival is expressed as the difference of log CFU/ml after stress and before stress. Numbers indicate fermentations as presented in Table 1. P-values above the plots indicate significance of correlation (assessed by a linear model). (ZIP) [file pone.0167944.s010.zip › S5_File/LACR_0134_real_dat.png]

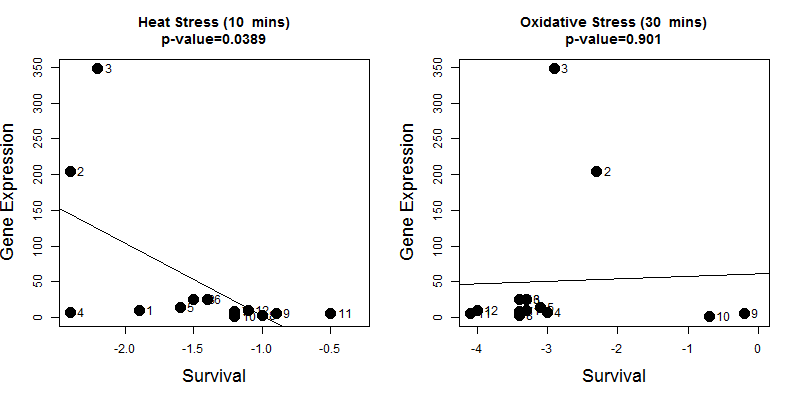

Supplement: S5 File — Expression levels of genes LACR_0001 –LACR_1382 plotted against survival after 10 minutes heat and 30 minutes oxidative stress. Survival is expressed as the difference of log CFU/ml after stress and before stress. Numbers indicate fermentations as presented in Table 1. P-values above the plots indicate significance of correlation (assessed by a linear model). (ZIP) [file pone.0167944.s010.zip › S5_File/LACR_0135_real_dat.png]

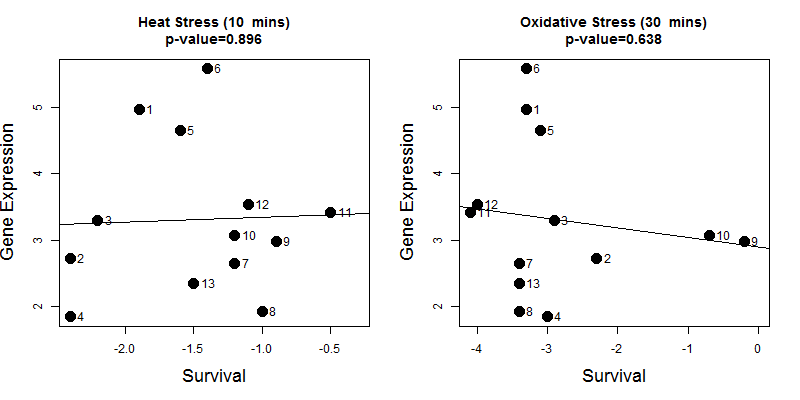

Supplement: S5 File — Expression levels of genes LACR_0001 –LACR_1382 plotted against survival after 10 minutes heat and 30 minutes oxidative stress. Survival is expressed as the difference of log CFU/ml after stress and before stress. Numbers indicate fermentations as presented in Table 1. P-values above the plots indicate significance of correlation (assessed by a linear model). (ZIP) [file pone.0167944.s010.zip › S5_File/LACR_0136_real_dat.png]

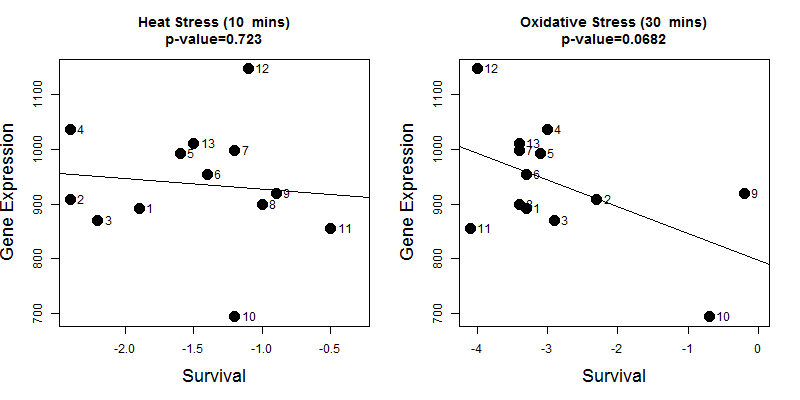

Supplement: S5 File — Expression levels of genes LACR_0001 –LACR_1382 plotted against survival after 10 minutes heat and 30 minutes oxidative stress. Survival is expressed as the difference of log CFU/ml after stress and before stress. Numbers indicate fermentations as presented in Table 1. P-values above the plots indicate significance of correlation (assessed by a linear model). (ZIP) [file pone.0167944.s010.zip › S5_File/LACR_0137_real_dat.png]

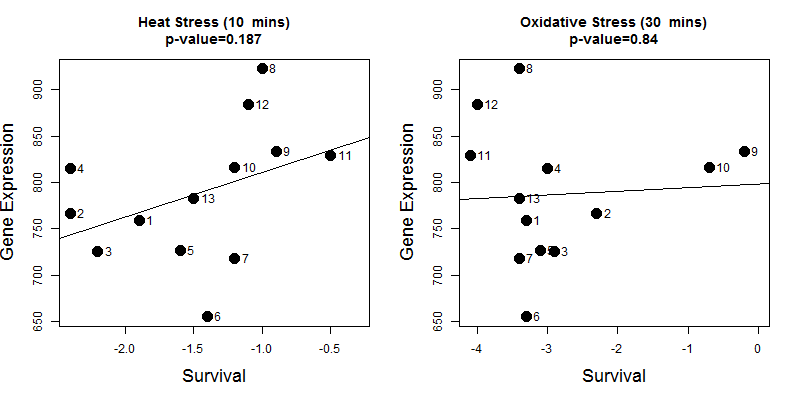

Supplement: S5 File — Expression levels of genes LACR_0001 –LACR_1382 plotted against survival after 10 minutes heat and 30 minutes oxidative stress. Survival is expressed as the difference of log CFU/ml after stress and before stress. Numbers indicate fermentations as presented in Table 1. P-values above the plots indicate significance of correlation (assessed by a linear model). (ZIP) [file pone.0167944.s010.zip › S5_File/LACR_0138_real_dat.png]

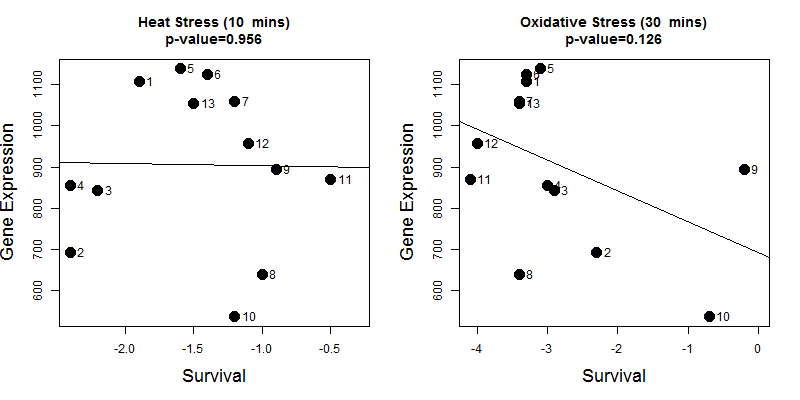

Supplement: S5 File — Expression levels of genes LACR_0001 –LACR_1382 plotted against survival after 10 minutes heat and 30 minutes oxidative stress. Survival is expressed as the difference of log CFU/ml after stress and before stress. Numbers indicate fermentations as presented in Table 1. P-values above the plots indicate significance of correlation (assessed by a linear model). (ZIP) [file pone.0167944.s010.zip › S5_File/LACR_0139_real_dat.png]

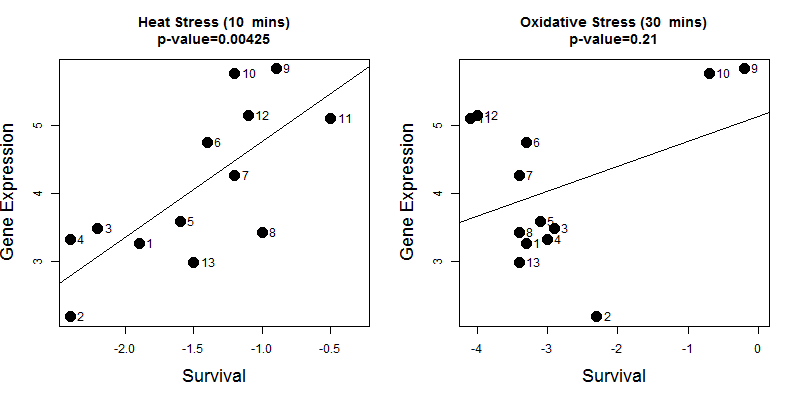

Supplement: S5 File — Expression levels of genes LACR_0001 –LACR_1382 plotted against survival after 10 minutes heat and 30 minutes oxidative stress. Survival is expressed as the difference of log CFU/ml after stress and before stress. Numbers indicate fermentations as presented in Table 1. P-values above the plots indicate significance of correlation (assessed by a linear model). (ZIP) [file pone.0167944.s010.zip › S5_File/LACR_0140_real_dat.png]

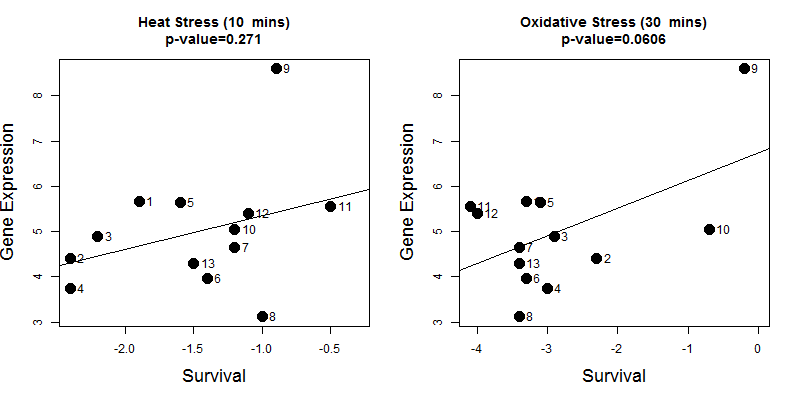

Supplement: S5 File — Expression levels of genes LACR_0001 –LACR_1382 plotted against survival after 10 minutes heat and 30 minutes oxidative stress. Survival is expressed as the difference of log CFU/ml after stress and before stress. Numbers indicate fermentations as presented in Table 1. P-values above the plots indicate significance of correlation (assessed by a linear model). (ZIP) [file pone.0167944.s010.zip › S5_File/LACR_0141_real_dat.png]

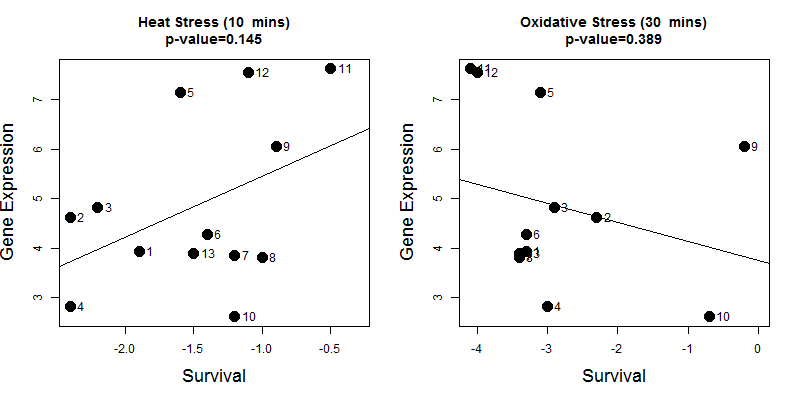

Supplement: S5 File — Expression levels of genes LACR_0001 –LACR_1382 plotted against survival after 10 minutes heat and 30 minutes oxidative stress. Survival is expressed as the difference of log CFU/ml after stress and before stress. Numbers indicate fermentations as presented in Table 1. P-values above the plots indicate significance of correlation (assessed by a linear model). (ZIP) [file pone.0167944.s010.zip › S5_File/LACR_0142_real_dat.png]

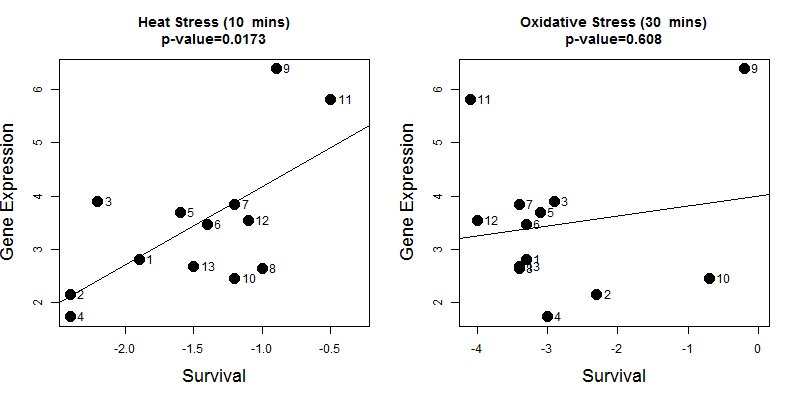

Supplement: S5 File — Expression levels of genes LACR_0001 –LACR_1382 plotted against survival after 10 minutes heat and 30 minutes oxidative stress. Survival is expressed as the difference of log CFU/ml after stress and before stress. Numbers indicate fermentations as presented in Table 1. P-values above the plots indicate significance of correlation (assessed by a linear model). (ZIP) [file pone.0167944.s010.zip › S5_File/LACR_0143_real_dat.png]

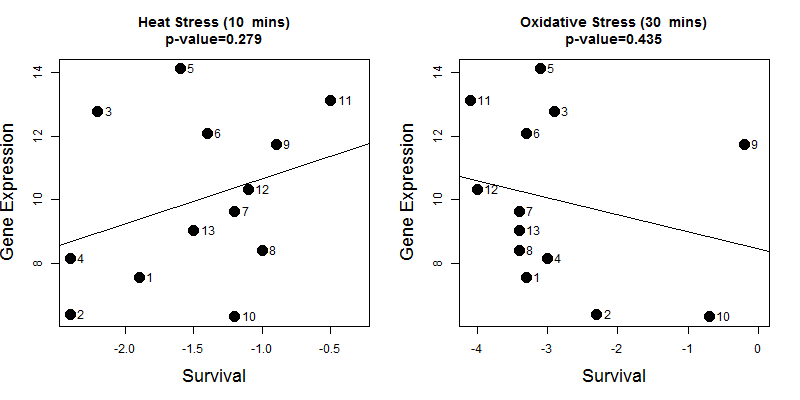

Supplement: S5 File — Expression levels of genes LACR_0001 –LACR_1382 plotted against survival after 10 minutes heat and 30 minutes oxidative stress. Survival is expressed as the difference of log CFU/ml after stress and before stress. Numbers indicate fermentations as presented in Table 1. P-values above the plots indicate significance of correlation (assessed by a linear model). (ZIP) [file pone.0167944.s010.zip › S5_File/LACR_0144_real_dat.png]

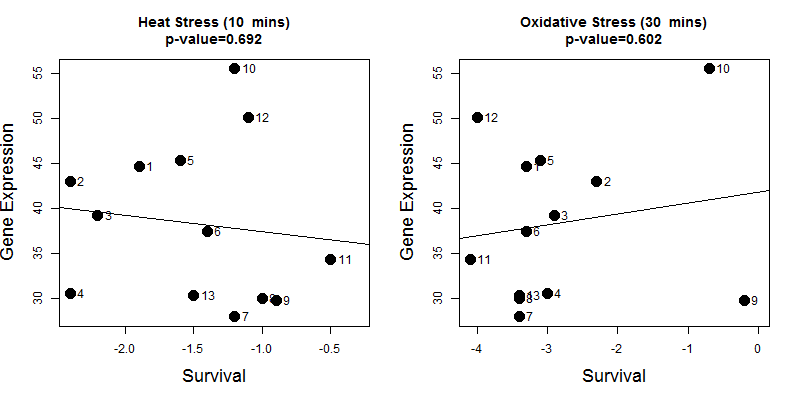

Supplement: S5 File — Expression levels of genes LACR_0001 –LACR_1382 plotted against survival after 10 minutes heat and 30 minutes oxidative stress. Survival is expressed as the difference of log CFU/ml after stress and before stress. Numbers indicate fermentations as presented in Table 1. P-values above the plots indicate significance of correlation (assessed by a linear model). (ZIP) [file pone.0167944.s010.zip › S5_File/LACR_0145_real_dat.png]

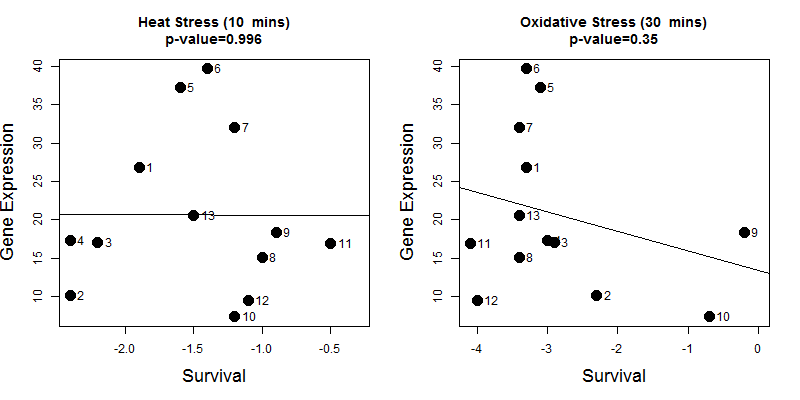

Supplement: S5 File — Expression levels of genes LACR_0001 –LACR_1382 plotted against survival after 10 minutes heat and 30 minutes oxidative stress. Survival is expressed as the difference of log CFU/ml after stress and before stress. Numbers indicate fermentations as presented in Table 1. P-values above the plots indicate significance of correlation (assessed by a linear model). (ZIP) [file pone.0167944.s010.zip › S5_File/LACR_0146_real_dat.png]

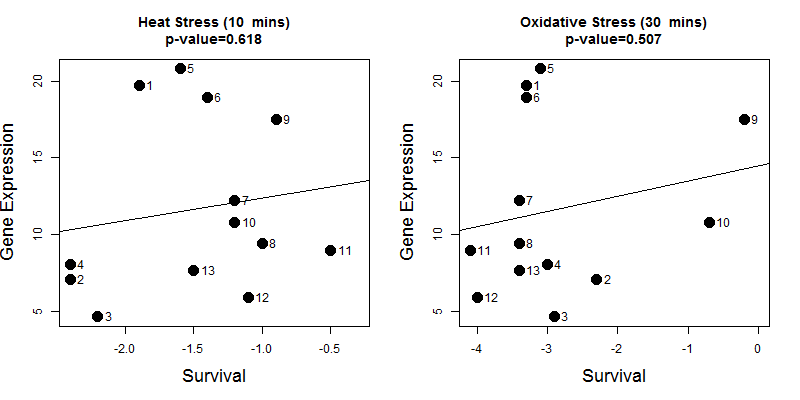

Supplement: S5 File — Expression levels of genes LACR_0001 –LACR_1382 plotted against survival after 10 minutes heat and 30 minutes oxidative stress. Survival is expressed as the difference of log CFU/ml after stress and before stress. Numbers indicate fermentations as presented in Table 1. P-values above the plots indicate significance of correlation (assessed by a linear model). (ZIP) [file pone.0167944.s010.zip › S5_File/LACR_0148_real_dat.png]

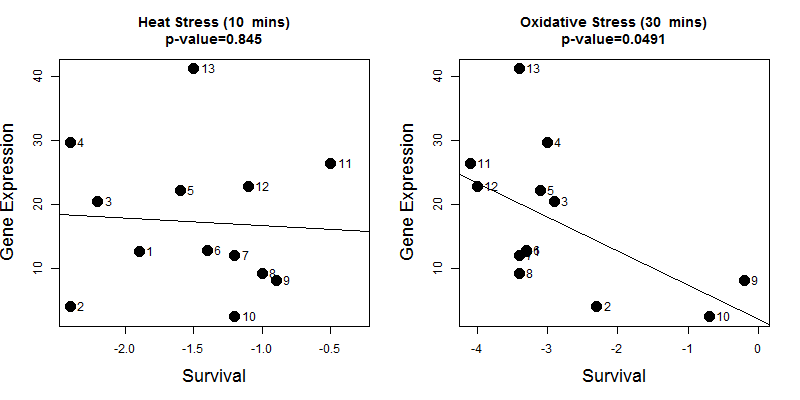

Supplement: S5 File — Expression levels of genes LACR_0001 –LACR_1382 plotted against survival after 10 minutes heat and 30 minutes oxidative stress. Survival is expressed as the difference of log CFU/ml after stress and before stress. Numbers indicate fermentations as presented in Table 1. P-values above the plots indicate significance of correlation (assessed by a linear model). (ZIP) [file pone.0167944.s010.zip › S5_File/LACR_0149_real_dat.png]

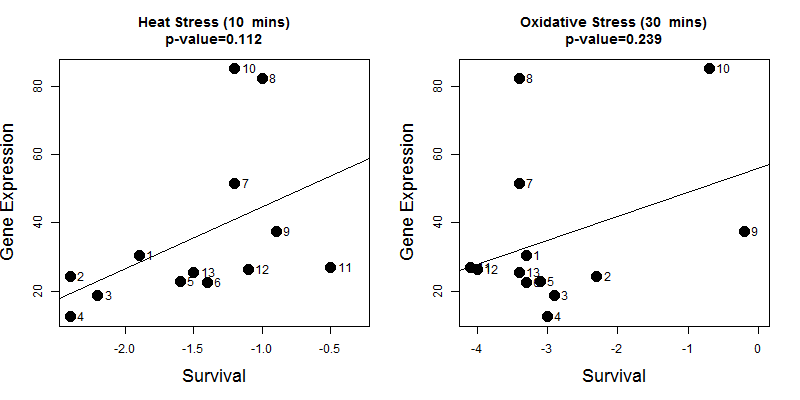

Supplement: S5 File — Expression levels of genes LACR_0001 –LACR_1382 plotted against survival after 10 minutes heat and 30 minutes oxidative stress. Survival is expressed as the difference of log CFU/ml after stress and before stress. Numbers indicate fermentations as presented in Table 1. P-values above the plots indicate significance of correlation (assessed by a linear model). (ZIP) [file pone.0167944.s010.zip › S5_File/LACR_0150_real_dat.png]

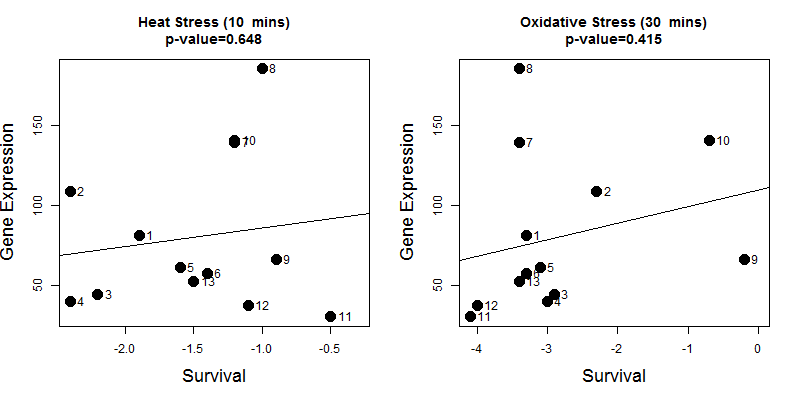

Supplement: S5 File — Expression levels of genes LACR_0001 –LACR_1382 plotted against survival after 10 minutes heat and 30 minutes oxidative stress. Survival is expressed as the difference of log CFU/ml after stress and before stress. Numbers indicate fermentations as presented in Table 1. P-values above the plots indicate significance of correlation (assessed by a linear model). (ZIP) [file pone.0167944.s010.zip › S5_File/LACR_0151_real_dat.png]

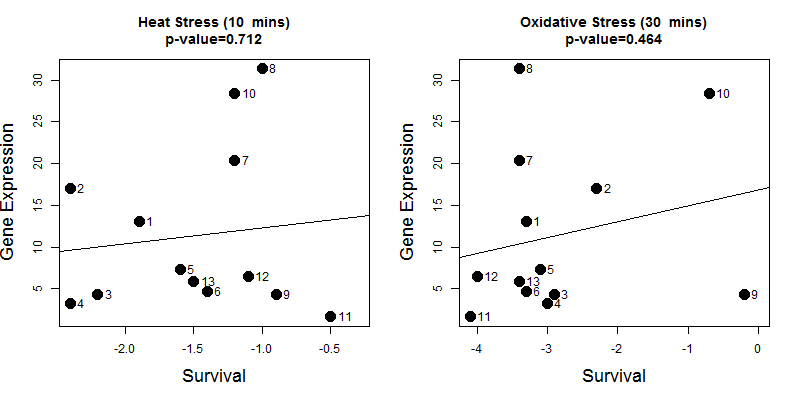

Supplement: S5 File — Expression levels of genes LACR_0001 –LACR_1382 plotted against survival after 10 minutes heat and 30 minutes oxidative stress. Survival is expressed as the difference of log CFU/ml after stress and before stress. Numbers indicate fermentations as presented in Table 1. P-values above the plots indicate significance of correlation (assessed by a linear model). (ZIP) [file pone.0167944.s010.zip › S5_File/LACR_0152_real_dat.png]

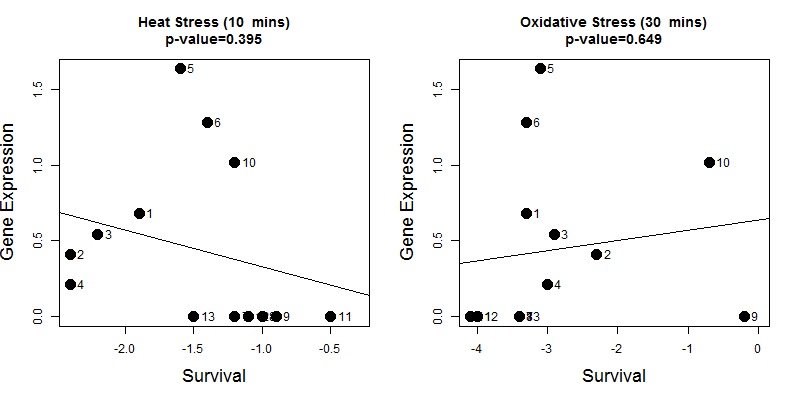

Supplement: S5 File — Expression levels of genes LACR_0001 –LACR_1382 plotted against survival after 10 minutes heat and 30 minutes oxidative stress. Survival is expressed as the difference of log CFU/ml after stress and before stress. Numbers indicate fermentations as presented in Table 1. P-values above the plots indicate significance of correlation (assessed by a linear model). (ZIP) [file pone.0167944.s010.zip › S5_File/LACR_0153_real_dat.png]

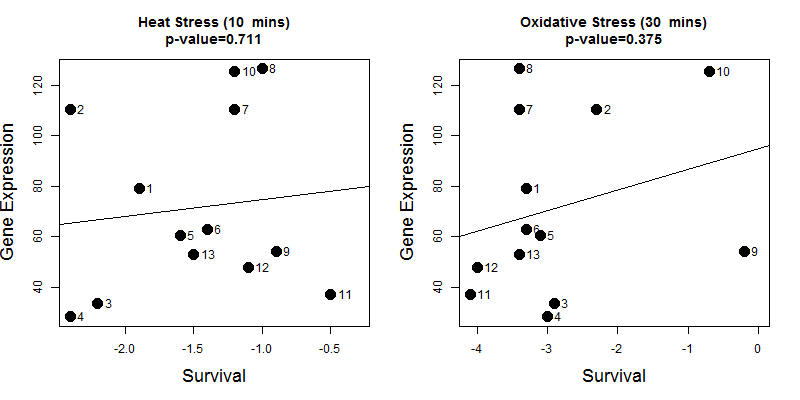

Supplement: S5 File — Expression levels of genes LACR_0001 –LACR_1382 plotted against survival after 10 minutes heat and 30 minutes oxidative stress. Survival is expressed as the difference of log CFU/ml after stress and before stress. Numbers indicate fermentations as presented in Table 1. P-values above the plots indicate significance of correlation (assessed by a linear model). (ZIP) [file pone.0167944.s010.zip › S5_File/LACR_0154_real_dat.png]

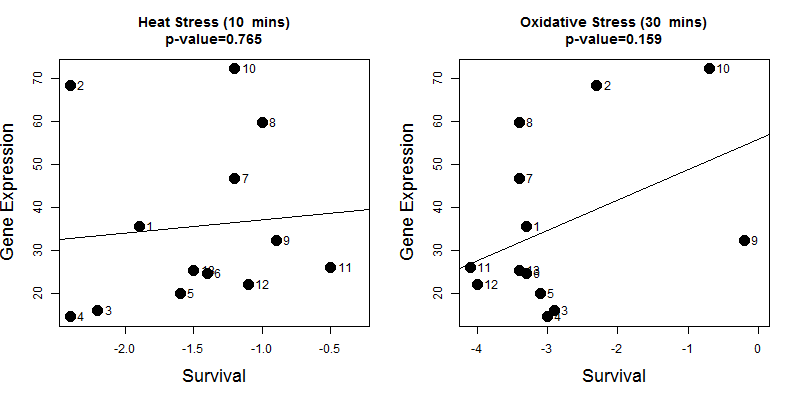

Supplement: S5 File — Expression levels of genes LACR_0001 –LACR_1382 plotted against survival after 10 minutes heat and 30 minutes oxidative stress. Survival is expressed as the difference of log CFU/ml after stress and before stress. Numbers indicate fermentations as presented in Table 1. P-values above the plots indicate significance of correlation (assessed by a linear model). (ZIP) [file pone.0167944.s010.zip › S5_File/LACR_0155_real_dat.png]

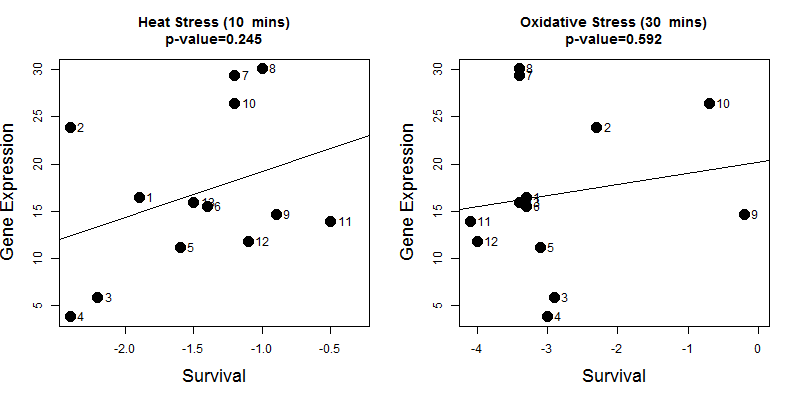

Supplement: S5 File — Expression levels of genes LACR_0001 –LACR_1382 plotted against survival after 10 minutes heat and 30 minutes oxidative stress. Survival is expressed as the difference of log CFU/ml after stress and before stress. Numbers indicate fermentations as presented in Table 1. P-values above the plots indicate significance of correlation (assessed by a linear model). (ZIP) [file pone.0167944.s010.zip › S5_File/LACR_0156_real_dat.png]

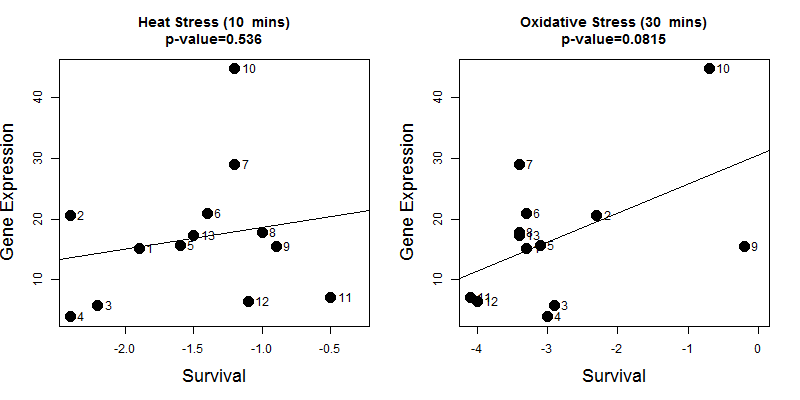

Supplement: S5 File — Expression levels of genes LACR_0001 –LACR_1382 plotted against survival after 10 minutes heat and 30 minutes oxidative stress. Survival is expressed as the difference of log CFU/ml after stress and before stress. Numbers indicate fermentations as presented in Table 1. P-values above the plots indicate significance of correlation (assessed by a linear model). (ZIP) [file pone.0167944.s010.zip › S5_File/LACR_0157_real_dat.png]

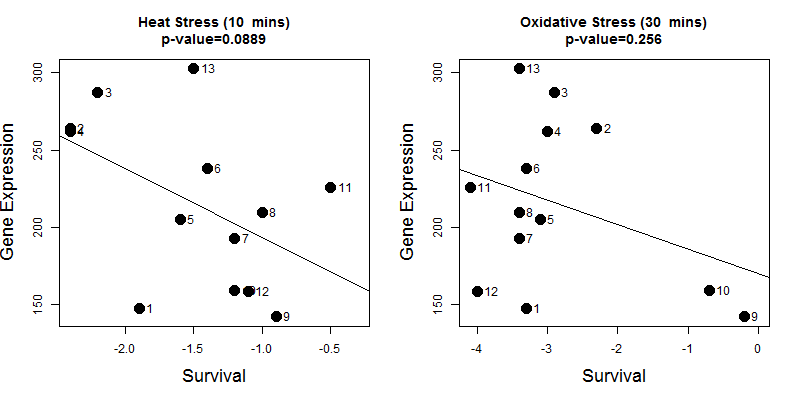

Supplement: S5 File — Expression levels of genes LACR_0001 –LACR_1382 plotted against survival after 10 minutes heat and 30 minutes oxidative stress. Survival is expressed as the difference of log CFU/ml after stress and before stress. Numbers indicate fermentations as presented in Table 1. P-values above the plots indicate significance of correlation (assessed by a linear model). (ZIP) [file pone.0167944.s010.zip › S5_File/LACR_0159_real_dat.png]

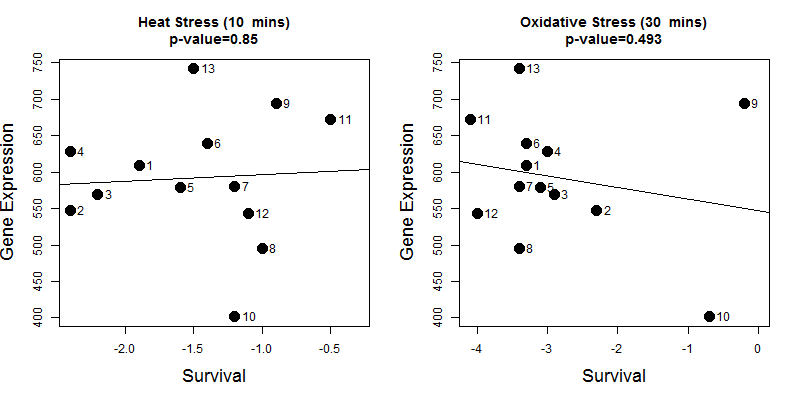

Supplement: S5 File — Expression levels of genes LACR_0001 –LACR_1382 plotted against survival after 10 minutes heat and 30 minutes oxidative stress. Survival is expressed as the difference of log CFU/ml after stress and before stress. Numbers indicate fermentations as presented in Table 1. P-values above the plots indicate significance of correlation (assessed by a linear model). (ZIP) [file pone.0167944.s010.zip › S5_File/LACR_0160_real_dat.png]

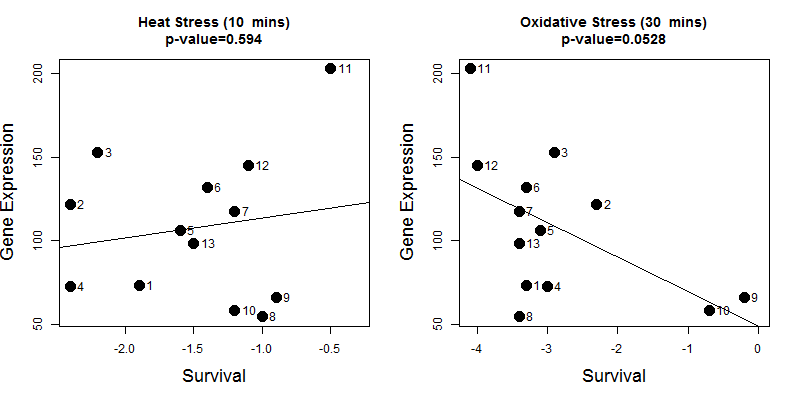

Supplement: S5 File — Expression levels of genes LACR_0001 –LACR_1382 plotted against survival after 10 minutes heat and 30 minutes oxidative stress. Survival is expressed as the difference of log CFU/ml after stress and before stress. Numbers indicate fermentations as presented in Table 1. P-values above the plots indicate significance of correlation (assessed by a linear model). (ZIP) [file pone.0167944.s010.zip › S5_File/LACR_0161_real_dat.png]

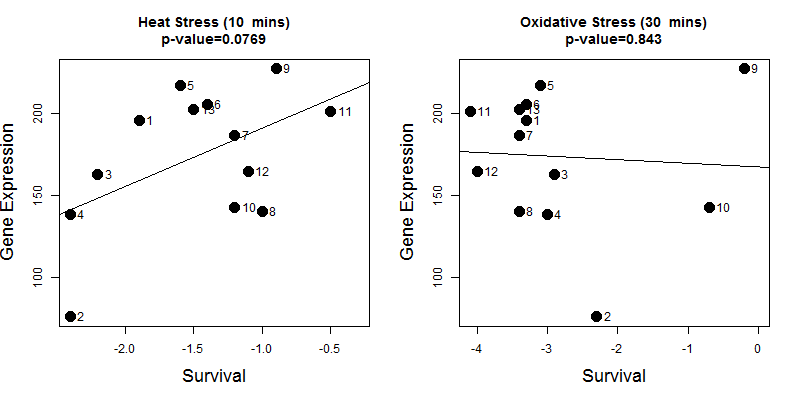

Supplement: S5 File — Expression levels of genes LACR_0001 –LACR_1382 plotted against survival after 10 minutes heat and 30 minutes oxidative stress. Survival is expressed as the difference of log CFU/ml after stress and before stress. Numbers indicate fermentations as presented in Table 1. P-values above the plots indicate significance of correlation (assessed by a linear model). (ZIP) [file pone.0167944.s010.zip › S5_File/LACR_0162_real_dat.png]

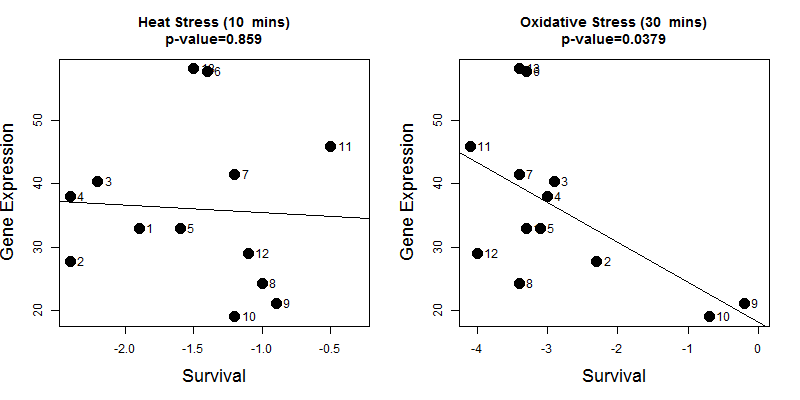

Supplement: S5 File — Expression levels of genes LACR_0001 –LACR_1382 plotted against survival after 10 minutes heat and 30 minutes oxidative stress. Survival is expressed as the difference of log CFU/ml after stress and before stress. Numbers indicate fermentations as presented in Table 1. P-values above the plots indicate significance of correlation (assessed by a linear model). (ZIP) [file pone.0167944.s010.zip › S5_File/LACR_0163_real_dat.png]

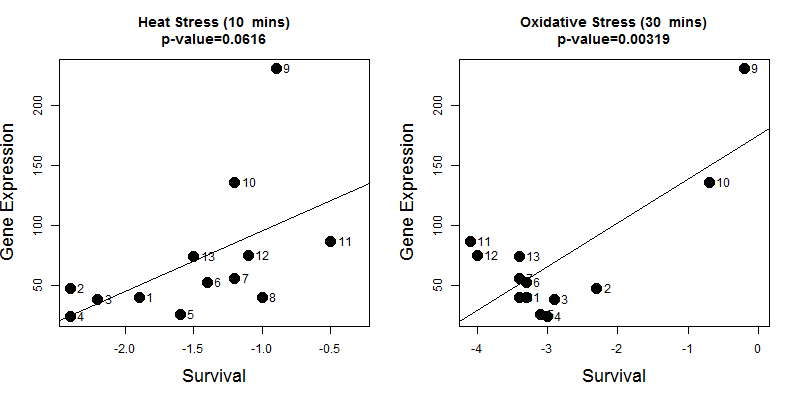

Supplement: S5 File — Expression levels of genes LACR_0001 –LACR_1382 plotted against survival after 10 minutes heat and 30 minutes oxidative stress. Survival is expressed as the difference of log CFU/ml after stress and before stress. Numbers indicate fermentations as presented in Table 1. P-values above the plots indicate significance of correlation (assessed by a linear model). (ZIP) [file pone.0167944.s010.zip › S5_File/LACR_0164_real_dat.png]

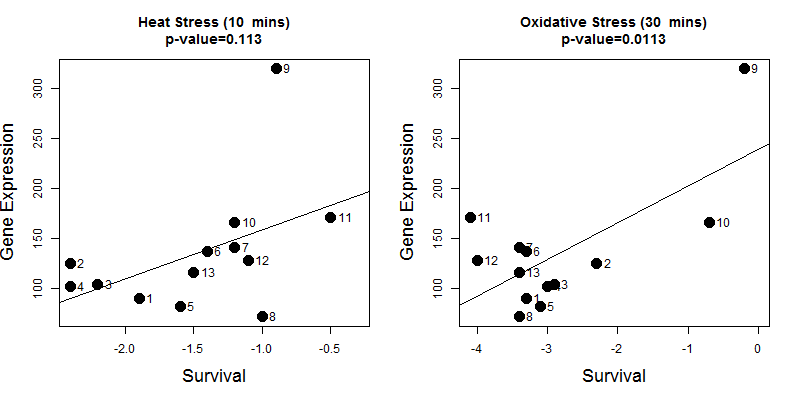

Supplement: S5 File — Expression levels of genes LACR_0001 –LACR_1382 plotted against survival after 10 minutes heat and 30 minutes oxidative stress. Survival is expressed as the difference of log CFU/ml after stress and before stress. Numbers indicate fermentations as presented in Table 1. P-values above the plots indicate significance of correlation (assessed by a linear model). (ZIP) [file pone.0167944.s010.zip › S5_File/LACR_0165_real_dat.png]

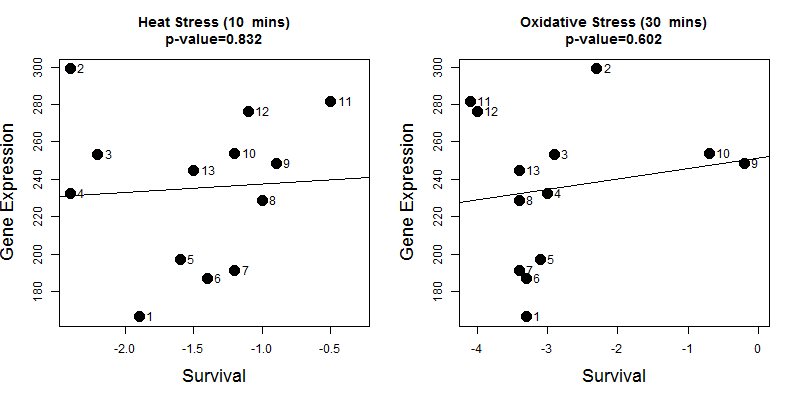

Supplement: S5 File — Expression levels of genes LACR_0001 –LACR_1382 plotted against survival after 10 minutes heat and 30 minutes oxidative stress. Survival is expressed as the difference of log CFU/ml after stress and before stress. Numbers indicate fermentations as presented in Table 1. P-values above the plots indicate significance of correlation (assessed by a linear model). (ZIP) [file pone.0167944.s010.zip › S5_File/LACR_0166_real_dat.png]

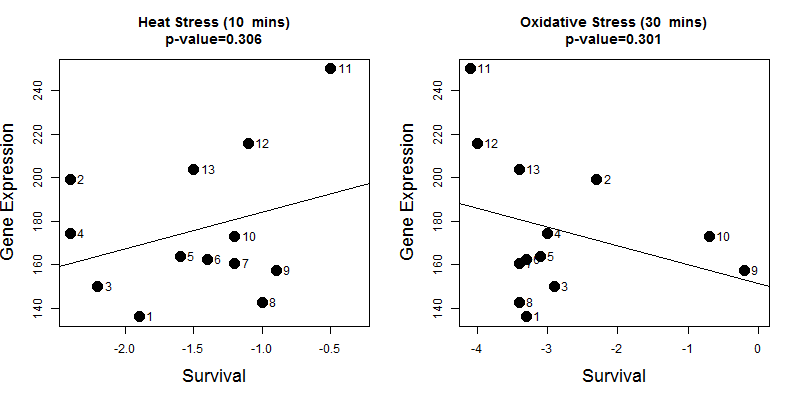

Supplement: S5 File — Expression levels of genes LACR_0001 –LACR_1382 plotted against survival after 10 minutes heat and 30 minutes oxidative stress. Survival is expressed as the difference of log CFU/ml after stress and before stress. Numbers indicate fermentations as presented in Table 1. P-values above the plots indicate significance of correlation (assessed by a linear model). (ZIP) [file pone.0167944.s010.zip › S5_File/LACR_0167_real_dat.png]

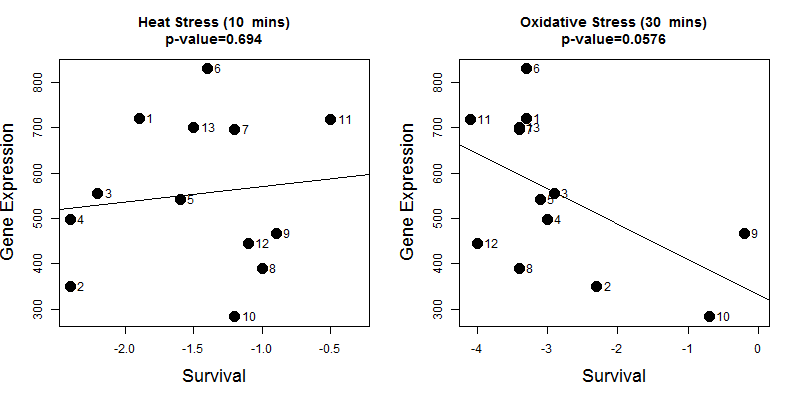

Supplement: S5 File — Expression levels of genes LACR_0001 –LACR_1382 plotted against survival after 10 minutes heat and 30 minutes oxidative stress. Survival is expressed as the difference of log CFU/ml after stress and before stress. Numbers indicate fermentations as presented in Table 1. P-values above the plots indicate significance of correlation (assessed by a linear model). (ZIP) [file pone.0167944.s010.zip › S5_File/LACR_0168_real_dat.png]

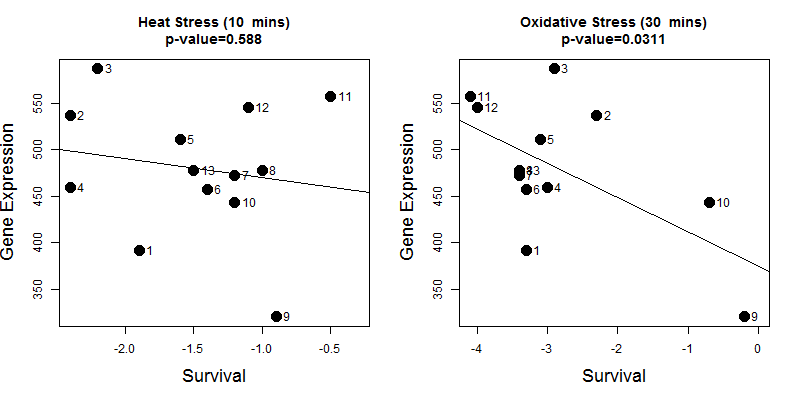

Supplement: S5 File — Expression levels of genes LACR_0001 –LACR_1382 plotted against survival after 10 minutes heat and 30 minutes oxidative stress. Survival is expressed as the difference of log CFU/ml after stress and before stress. Numbers indicate fermentations as presented in Table 1. P-values above the plots indicate significance of correlation (assessed by a linear model). (ZIP) [file pone.0167944.s010.zip › S5_File/LACR_0169_real_dat.png]

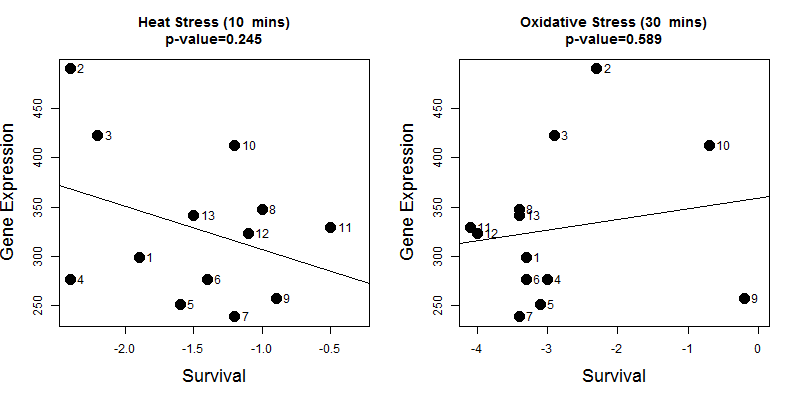

Supplement: S5 File — Expression levels of genes LACR_0001 –LACR_1382 plotted against survival after 10 minutes heat and 30 minutes oxidative stress. Survival is expressed as the difference of log CFU/ml after stress and before stress. Numbers indicate fermentations as presented in Table 1. P-values above the plots indicate significance of correlation (assessed by a linear model). (ZIP) [file pone.0167944.s010.zip › S5_File/LACR_0170_real_dat.png]

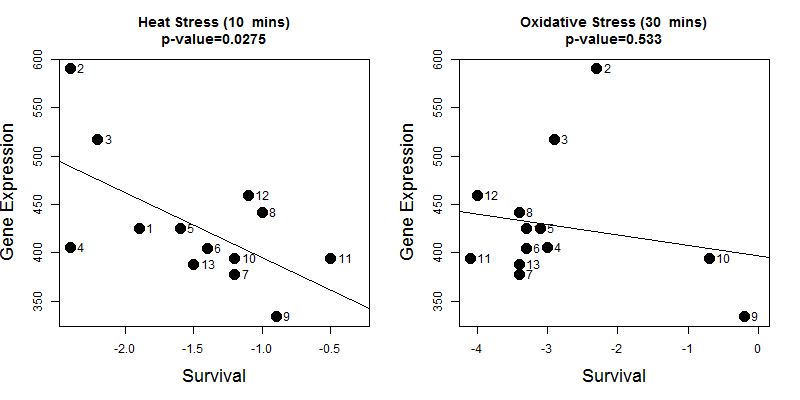

Supplement: S5 File — Expression levels of genes LACR_0001 –LACR_1382 plotted against survival after 10 minutes heat and 30 minutes oxidative stress. Survival is expressed as the difference of log CFU/ml after stress and before stress. Numbers indicate fermentations as presented in Table 1. P-values above the plots indicate significance of correlation (assessed by a linear model). (ZIP) [file pone.0167944.s010.zip › S5_File/LACR_0171_real_dat.png]

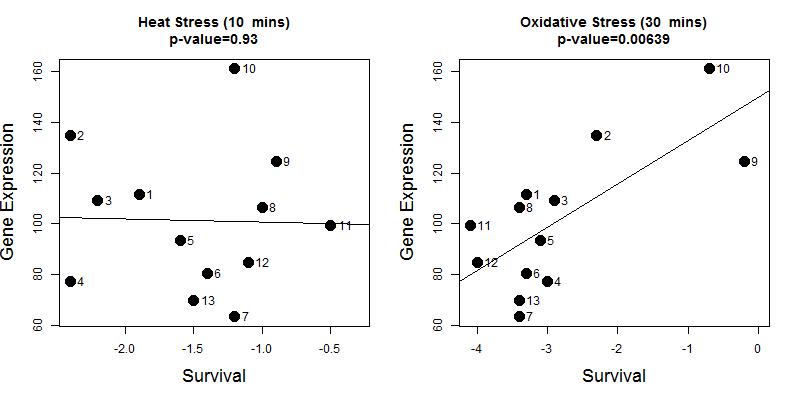

Supplement: S5 File — Expression levels of genes LACR_0001 –LACR_1382 plotted against survival after 10 minutes heat and 30 minutes oxidative stress. Survival is expressed as the difference of log CFU/ml after stress and before stress. Numbers indicate fermentations as presented in Table 1. P-values above the plots indicate significance of correlation (assessed by a linear model). (ZIP) [file pone.0167944.s010.zip › S5_File/LACR_0172_real_dat.png]

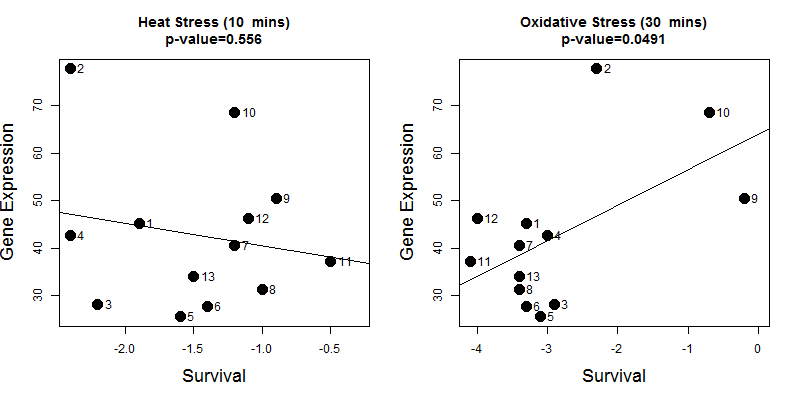

Supplement: S5 File — Expression levels of genes LACR_0001 –LACR_1382 plotted against survival after 10 minutes heat and 30 minutes oxidative stress. Survival is expressed as the difference of log CFU/ml after stress and before stress. Numbers indicate fermentations as presented in Table 1. P-values above the plots indicate significance of correlation (assessed by a linear model). (ZIP) [file pone.0167944.s010.zip › S5_File/LACR_0173_real_dat.png]

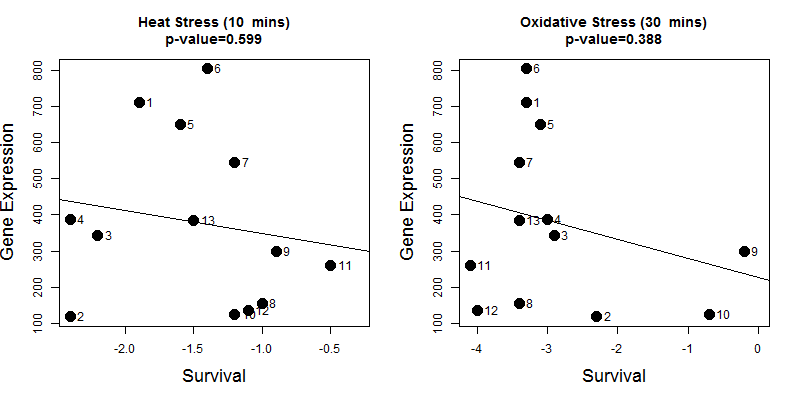

Supplement: S5 File — Expression levels of genes LACR_0001 –LACR_1382 plotted against survival after 10 minutes heat and 30 minutes oxidative stress. Survival is expressed as the difference of log CFU/ml after stress and before stress. Numbers indicate fermentations as presented in Table 1. P-values above the plots indicate significance of correlation (assessed by a linear model). (ZIP) [file pone.0167944.s010.zip › S5_File/LACR_0174_real_dat.png]

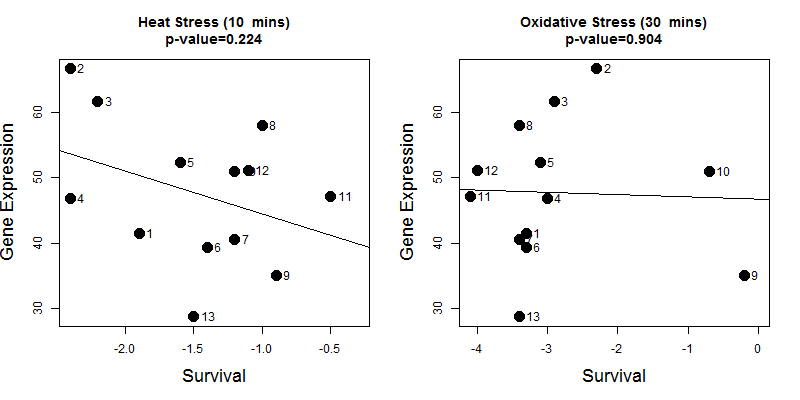

Supplement: S5 File — Expression levels of genes LACR_0001 –LACR_1382 plotted against survival after 10 minutes heat and 30 minutes oxidative stress. Survival is expressed as the difference of log CFU/ml after stress and before stress. Numbers indicate fermentations as presented in Table 1. P-values above the plots indicate significance of correlation (assessed by a linear model). (ZIP) [file pone.0167944.s010.zip › S5_File/LACR_0176_real_dat.png]

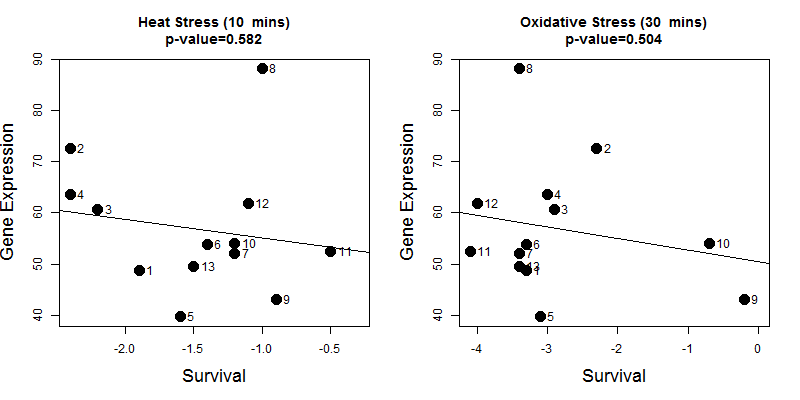

Supplement: S5 File — Expression levels of genes LACR_0001 –LACR_1382 plotted against survival after 10 minutes heat and 30 minutes oxidative stress. Survival is expressed as the difference of log CFU/ml after stress and before stress. Numbers indicate fermentations as presented in Table 1. P-values above the plots indicate significance of correlation (assessed by a linear model). (ZIP) [file pone.0167944.s010.zip › S5_File/LACR_0177_real_dat.png]

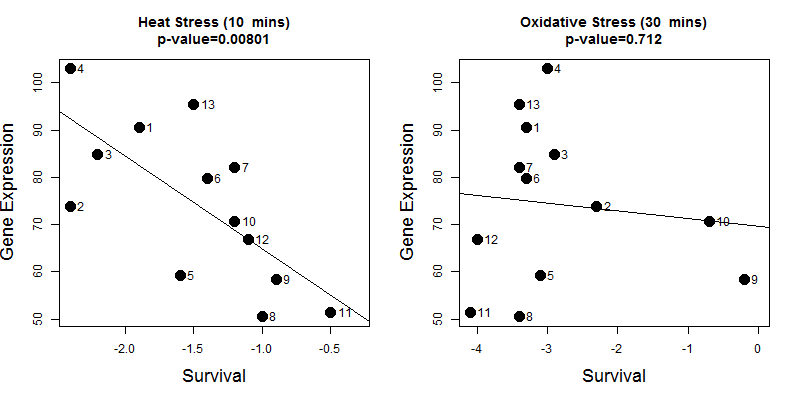

Supplement: S5 File — Expression levels of genes LACR_0001 –LACR_1382 plotted against survival after 10 minutes heat and 30 minutes oxidative stress. Survival is expressed as the difference of log CFU/ml after stress and before stress. Numbers indicate fermentations as presented in Table 1. P-values above the plots indicate significance of correlation (assessed by a linear model). (ZIP) [file pone.0167944.s010.zip › S5_File/LACR_0178_real_dat.png]

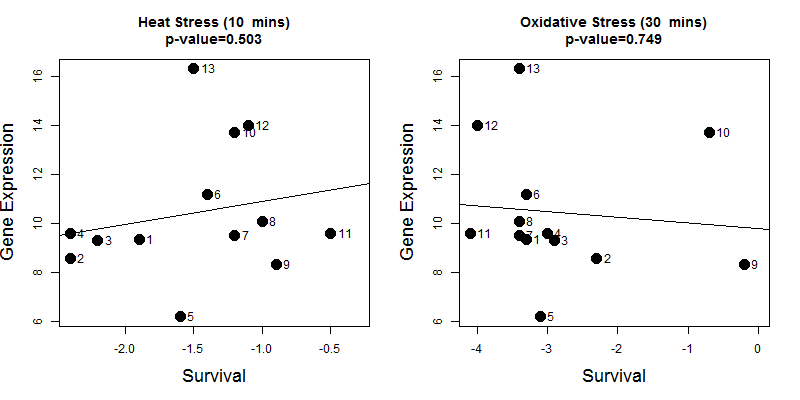

Supplement: S5 File — Expression levels of genes LACR_0001 –LACR_1382 plotted against survival after 10 minutes heat and 30 minutes oxidative stress. Survival is expressed as the difference of log CFU/ml after stress and before stress. Numbers indicate fermentations as presented in Table 1. P-values above the plots indicate significance of correlation (assessed by a linear model). (ZIP) [file pone.0167944.s010.zip › S5_File/LACR_0179_real_dat.png]

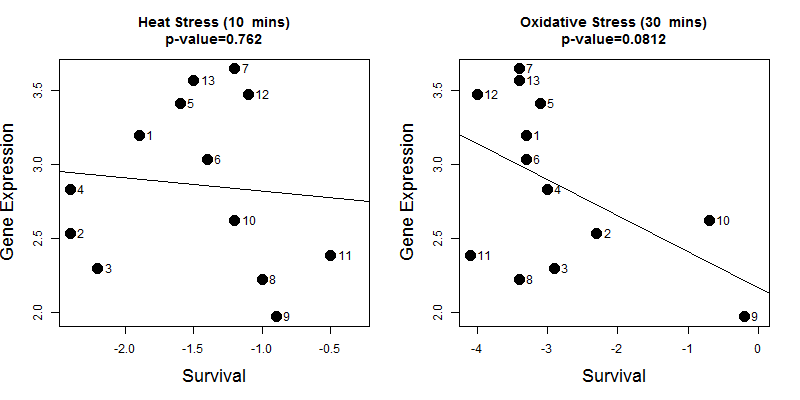

Supplement: S5 File — Expression levels of genes LACR_0001 –LACR_1382 plotted against survival after 10 minutes heat and 30 minutes oxidative stress. Survival is expressed as the difference of log CFU/ml after stress and before stress. Numbers indicate fermentations as presented in Table 1. P-values above the plots indicate significance of correlation (assessed by a linear model). (ZIP) [file pone.0167944.s010.zip › S5_File/LACR_0180_real_dat.png]

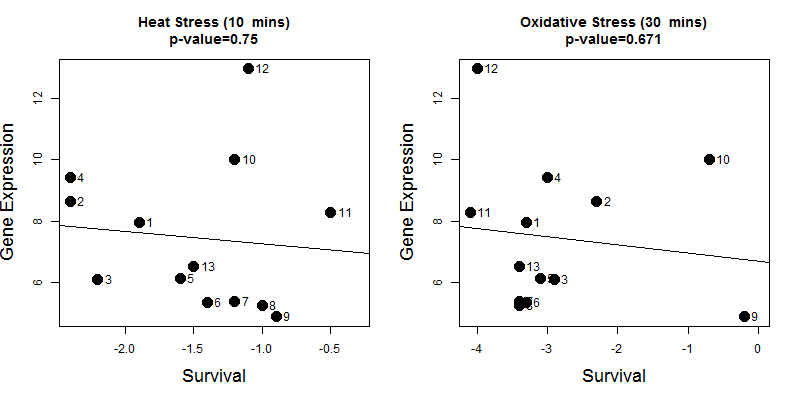

Supplement: S5 File — Expression levels of genes LACR_0001 –LACR_1382 plotted against survival after 10 minutes heat and 30 minutes oxidative stress. Survival is expressed as the difference of log CFU/ml after stress and before stress. Numbers indicate fermentations as presented in Table 1. P-values above the plots indicate significance of correlation (assessed by a linear model). (ZIP) [file pone.0167944.s010.zip › S5_File/LACR_0181_real_dat.png]

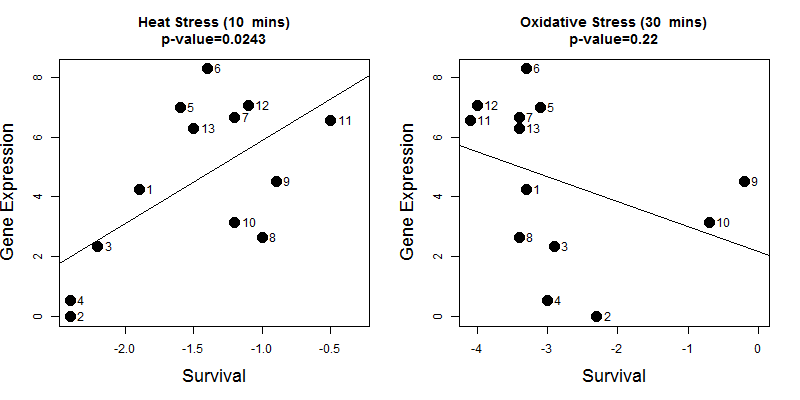

Supplement: S5 File — Expression levels of genes LACR_0001 –LACR_1382 plotted against survival after 10 minutes heat and 30 minutes oxidative stress. Survival is expressed as the difference of log CFU/ml after stress and before stress. Numbers indicate fermentations as presented in Table 1. P-values above the plots indicate significance of correlation (assessed by a linear model). (ZIP) [file pone.0167944.s010.zip › S5_File/LACR_0182_real_dat.png]

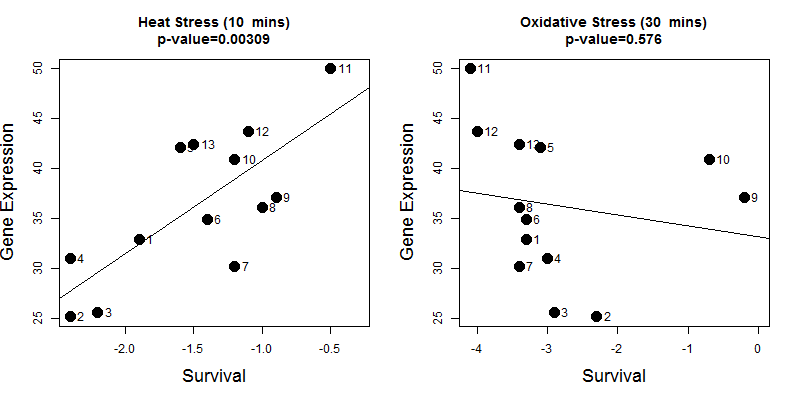

Supplement: S5 File — Expression levels of genes LACR_0001 –LACR_1382 plotted against survival after 10 minutes heat and 30 minutes oxidative stress. Survival is expressed as the difference of log CFU/ml after stress and before stress. Numbers indicate fermentations as presented in Table 1. P-values above the plots indicate significance of correlation (assessed by a linear model). (ZIP) [file pone.0167944.s010.zip › S5_File/LACR_0183_real_dat.png]

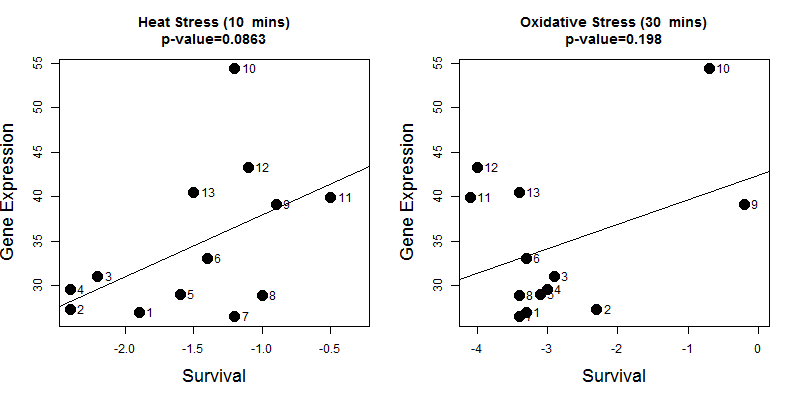

Supplement: S5 File — Expression levels of genes LACR_0001 –LACR_1382 plotted against survival after 10 minutes heat and 30 minutes oxidative stress. Survival is expressed as the difference of log CFU/ml after stress and before stress. Numbers indicate fermentations as presented in Table 1. P-values above the plots indicate significance of correlation (assessed by a linear model). (ZIP) [file pone.0167944.s010.zip › S5_File/LACR_0184_real_dat.png]

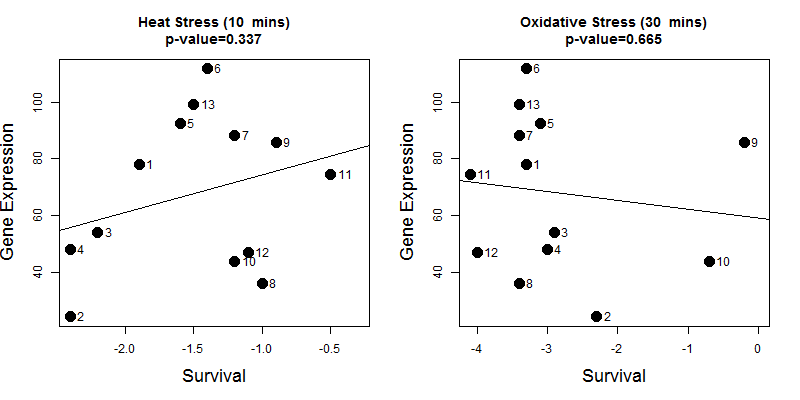

Supplement: S5 File — Expression levels of genes LACR_0001 –LACR_1382 plotted against survival after 10 minutes heat and 30 minutes oxidative stress. Survival is expressed as the difference of log CFU/ml after stress and before stress. Numbers indicate fermentations as presented in Table 1. P-values above the plots indicate significance of correlation (assessed by a linear model). (ZIP) [file pone.0167944.s010.zip › S5_File/LACR_0185_real_dat.png]

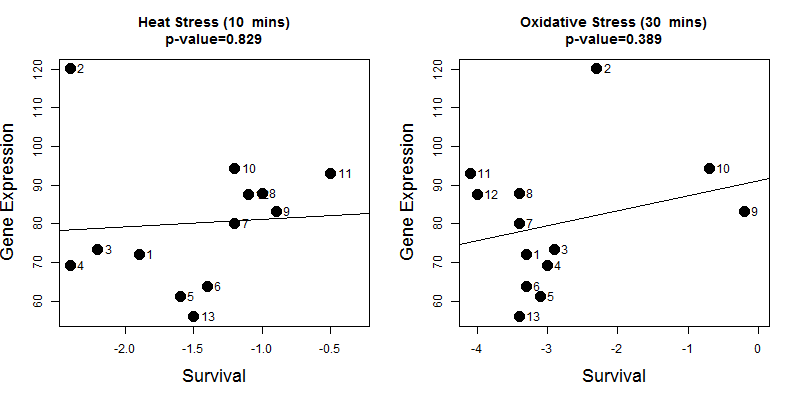

Supplement: S5 File — Expression levels of genes LACR_0001 –LACR_1382 plotted against survival after 10 minutes heat and 30 minutes oxidative stress. Survival is expressed as the difference of log CFU/ml after stress and before stress. Numbers indicate fermentations as presented in Table 1. P-values above the plots indicate significance of correlation (assessed by a linear model). (ZIP) [file pone.0167944.s010.zip › S5_File/LACR_0186_real_dat.png]

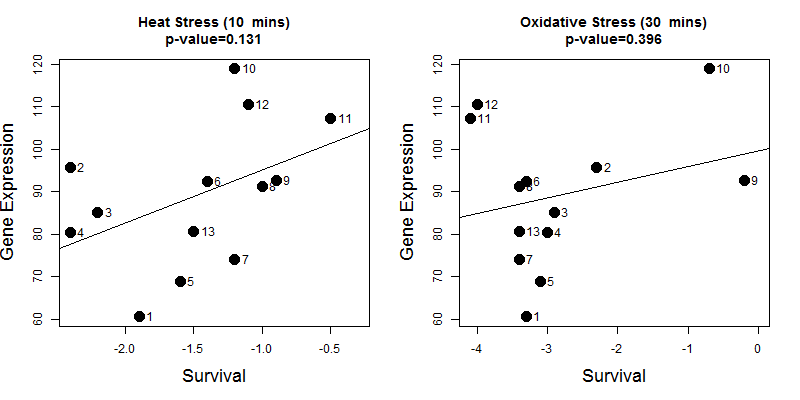

Supplement: S5 File — Expression levels of genes LACR_0001 –LACR_1382 plotted against survival after 10 minutes heat and 30 minutes oxidative stress. Survival is expressed as the difference of log CFU/ml after stress and before stress. Numbers indicate fermentations as presented in Table 1. P-values above the plots indicate significance of correlation (assessed by a linear model). (ZIP) [file pone.0167944.s010.zip › S5_File/LACR_0187_real_dat.png]

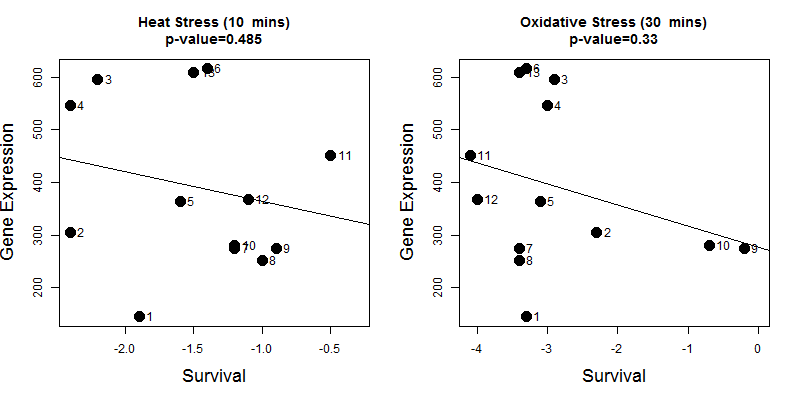

Supplement: S5 File — Expression levels of genes LACR_0001 –LACR_1382 plotted against survival after 10 minutes heat and 30 minutes oxidative stress. Survival is expressed as the difference of log CFU/ml after stress and before stress. Numbers indicate fermentations as presented in Table 1. P-values above the plots indicate significance of correlation (assessed by a linear model). (ZIP) [file pone.0167944.s010.zip › S5_File/LACR_0188_real_dat.png]

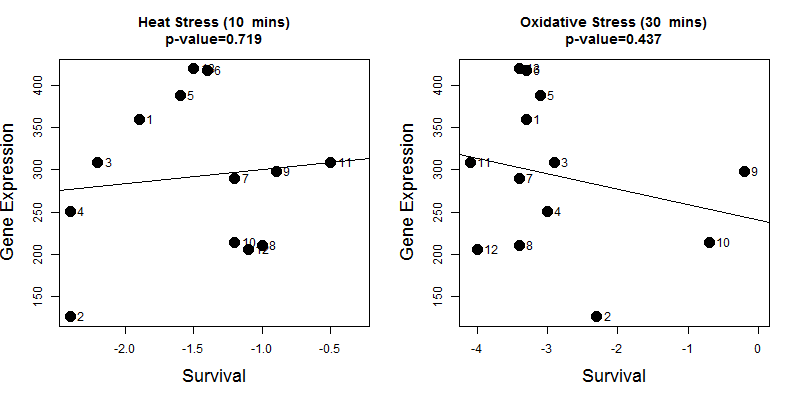

Supplement: S5 File — Expression levels of genes LACR_0001 –LACR_1382 plotted against survival after 10 minutes heat and 30 minutes oxidative stress. Survival is expressed as the difference of log CFU/ml after stress and before stress. Numbers indicate fermentations as presented in Table 1. P-values above the plots indicate significance of correlation (assessed by a linear model). (ZIP) [file pone.0167944.s010.zip › S5_File/LACR_0189_real_dat.png]

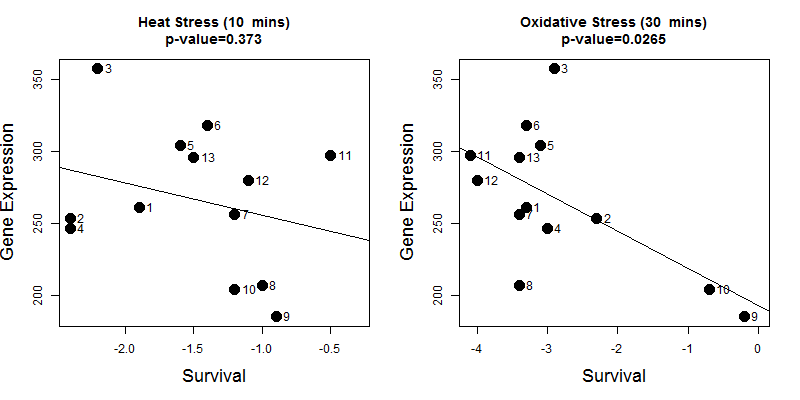

Supplement: S5 File — Expression levels of genes LACR_0001 –LACR_1382 plotted against survival after 10 minutes heat and 30 minutes oxidative stress. Survival is expressed as the difference of log CFU/ml after stress and before stress. Numbers indicate fermentations as presented in Table 1. P-values above the plots indicate significance of correlation (assessed by a linear model). (ZIP) [file pone.0167944.s010.zip › S5_File/LACR_0190_real_dat.png]

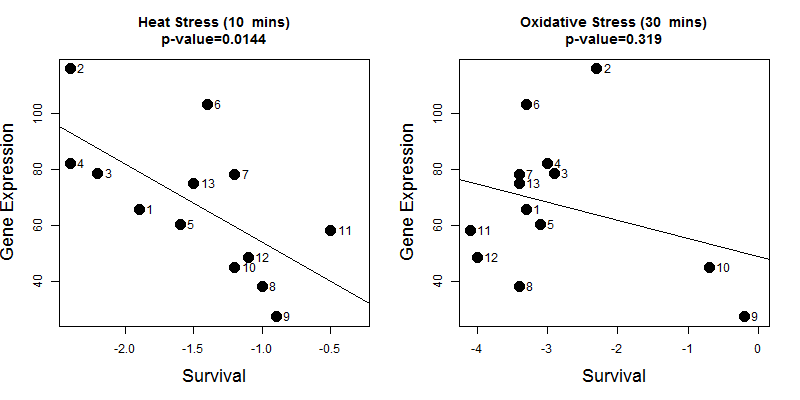

Supplement: S5 File — Expression levels of genes LACR_0001 –LACR_1382 plotted against survival after 10 minutes heat and 30 minutes oxidative stress. Survival is expressed as the difference of log CFU/ml after stress and before stress. Numbers indicate fermentations as presented in Table 1. P-values above the plots indicate significance of correlation (assessed by a linear model). (ZIP) [file pone.0167944.s010.zip › S5_File/LACR_0191_real_dat.png]

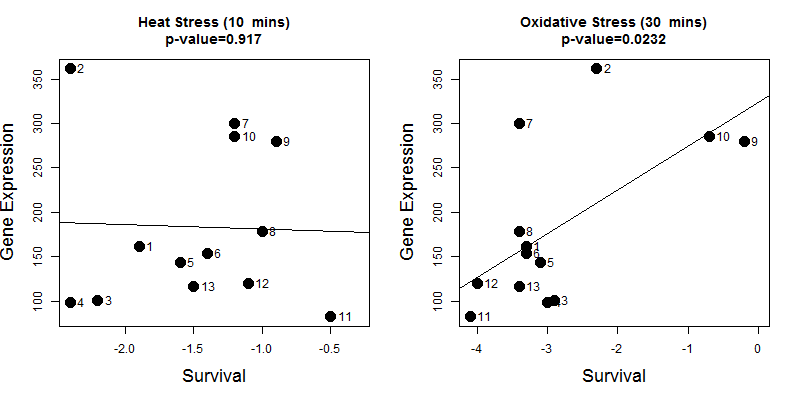

Supplement: S5 File — Expression levels of genes LACR_0001 –LACR_1382 plotted against survival after 10 minutes heat and 30 minutes oxidative stress. Survival is expressed as the difference of log CFU/ml after stress and before stress. Numbers indicate fermentations as presented in Table 1. P-values above the plots indicate significance of correlation (assessed by a linear model). (ZIP) [file pone.0167944.s010.zip › S5_File/LACR_0192_real_dat.png]

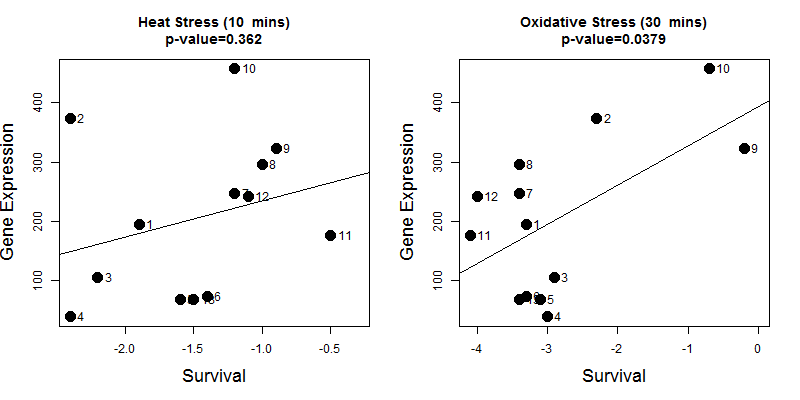

Supplement: S5 File — Expression levels of genes LACR_0001 –LACR_1382 plotted against survival after 10 minutes heat and 30 minutes oxidative stress. Survival is expressed as the difference of log CFU/ml after stress and before stress. Numbers indicate fermentations as presented in Table 1. P-values above the plots indicate significance of correlation (assessed by a linear model). (ZIP) [file pone.0167944.s010.zip › S5_File/LACR_0193_real_dat.png]

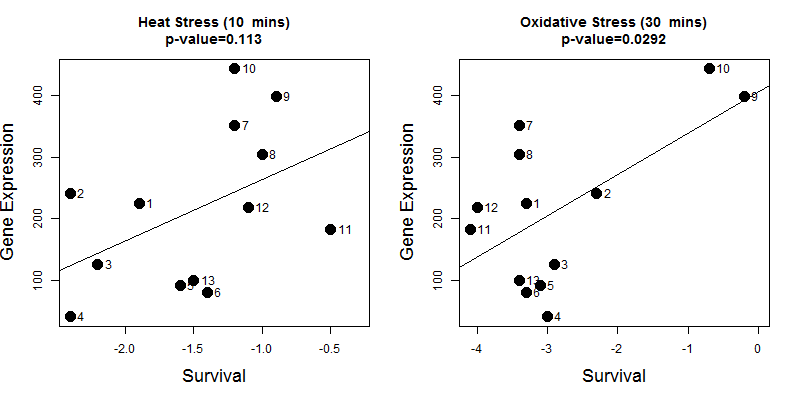

Supplement: S5 File — Expression levels of genes LACR_0001 –LACR_1382 plotted against survival after 10 minutes heat and 30 minutes oxidative stress. Survival is expressed as the difference of log CFU/ml after stress and before stress. Numbers indicate fermentations as presented in Table 1. P-values above the plots indicate significance of correlation (assessed by a linear model). (ZIP) [file pone.0167944.s010.zip › S5_File/LACR_0194_real_dat.png]

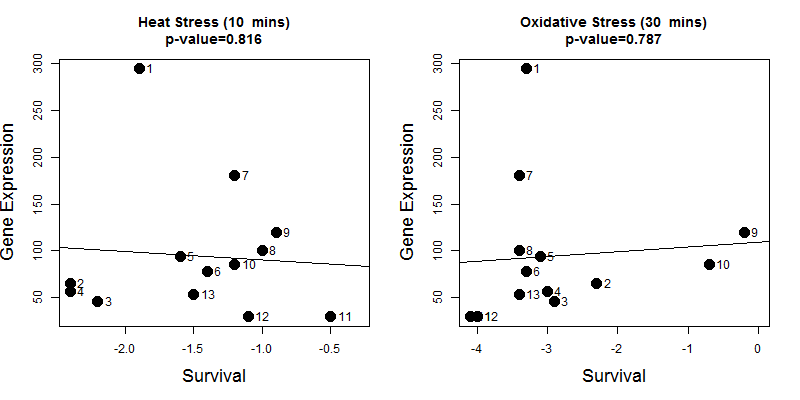

Supplement: S5 File — Expression levels of genes LACR_0001 –LACR_1382 plotted against survival after 10 minutes heat and 30 minutes oxidative stress. Survival is expressed as the difference of log CFU/ml after stress and before stress. Numbers indicate fermentations as presented in Table 1. P-values above the plots indicate significance of correlation (assessed by a linear model). (ZIP) [file pone.0167944.s010.zip › S5_File/LACR_0195_real_dat.png]

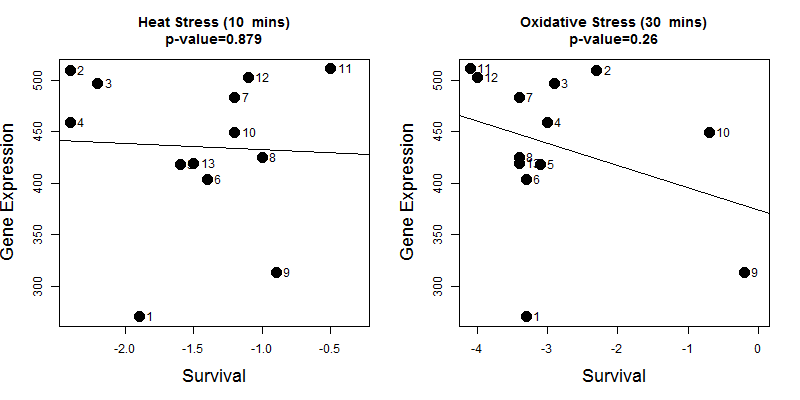

Supplement: S5 File — Expression levels of genes LACR_0001 –LACR_1382 plotted against survival after 10 minutes heat and 30 minutes oxidative stress. Survival is expressed as the difference of log CFU/ml after stress and before stress. Numbers indicate fermentations as presented in Table 1. P-values above the plots indicate significance of correlation (assessed by a linear model). (ZIP) [file pone.0167944.s010.zip › S5_File/LACR_0196_real_dat.png]

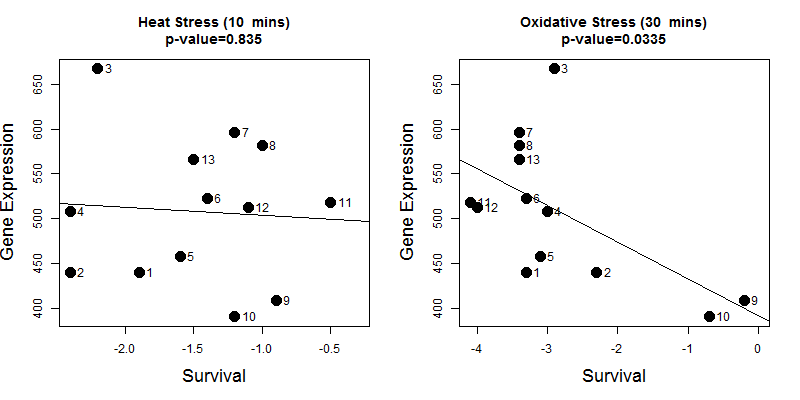

Supplement: S5 File — Expression levels of genes LACR_0001 –LACR_1382 plotted against survival after 10 minutes heat and 30 minutes oxidative stress. Survival is expressed as the difference of log CFU/ml after stress and before stress. Numbers indicate fermentations as presented in Table 1. P-values above the plots indicate significance of correlation (assessed by a linear model). (ZIP) [file pone.0167944.s010.zip › S5_File/LACR_0197_real_dat.png]

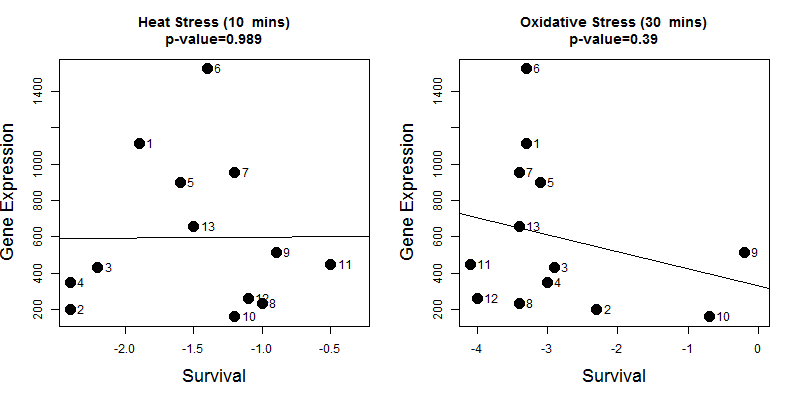

Supplement: S5 File — Expression levels of genes LACR_0001 –LACR_1382 plotted against survival after 10 minutes heat and 30 minutes oxidative stress. Survival is expressed as the difference of log CFU/ml after stress and before stress. Numbers indicate fermentations as presented in Table 1. P-values above the plots indicate significance of correlation (assessed by a linear model). (ZIP) [file pone.0167944.s010.zip › S5_File/LACR_0198_real_dat.png]

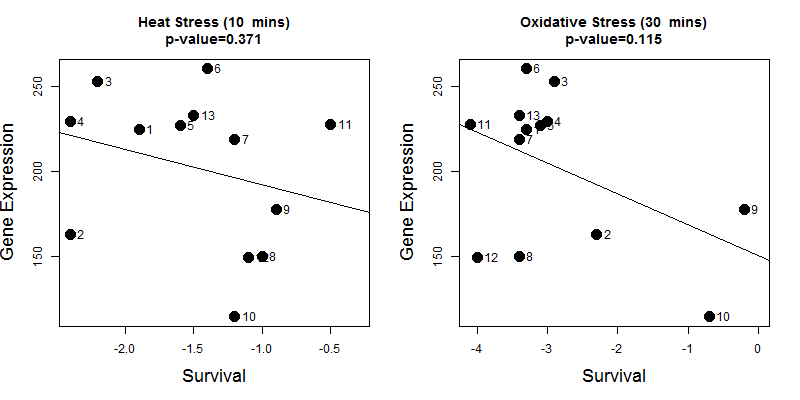

Supplement: S5 File — Expression levels of genes LACR_0001 –LACR_1382 plotted against survival after 10 minutes heat and 30 minutes oxidative stress. Survival is expressed as the difference of log CFU/ml after stress and before stress. Numbers indicate fermentations as presented in Table 1. P-values above the plots indicate significance of correlation (assessed by a linear model). (ZIP) [file pone.0167944.s010.zip › S5_File/LACR_0199_real_dat.png]

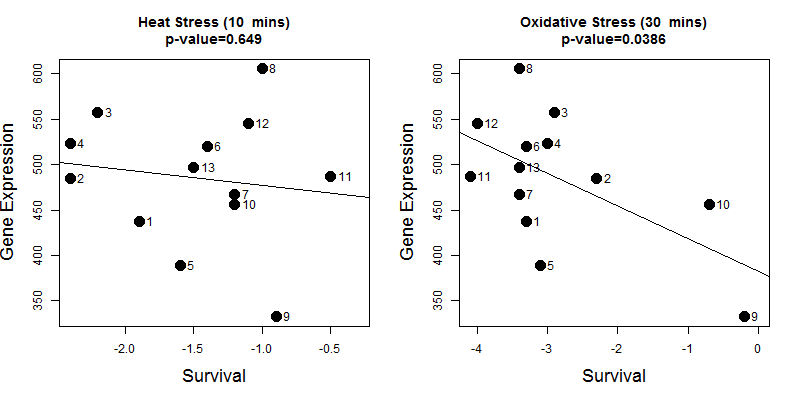

Supplement: S5 File — Expression levels of genes LACR_0001 –LACR_1382 plotted against survival after 10 minutes heat and 30 minutes oxidative stress. Survival is expressed as the difference of log CFU/ml after stress and before stress. Numbers indicate fermentations as presented in Table 1. P-values above the plots indicate significance of correlation (assessed by a linear model). (ZIP) [file pone.0167944.s010.zip › S5_File/LACR_0200_real_dat.png]

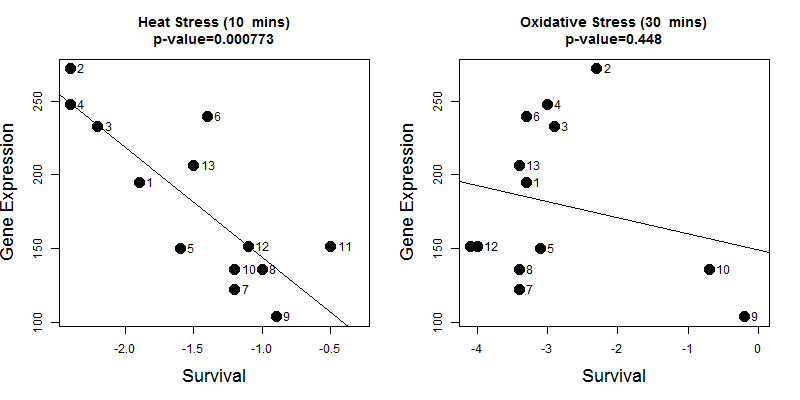

Supplement: S5 File — Expression levels of genes LACR_0001 –LACR_1382 plotted against survival after 10 minutes heat and 30 minutes oxidative stress. Survival is expressed as the difference of log CFU/ml after stress and before stress. Numbers indicate fermentations as presented in Table 1. P-values above the plots indicate significance of correlation (assessed by a linear model). (ZIP) [file pone.0167944.s010.zip › S5_File/LACR_0201_real_dat.png]

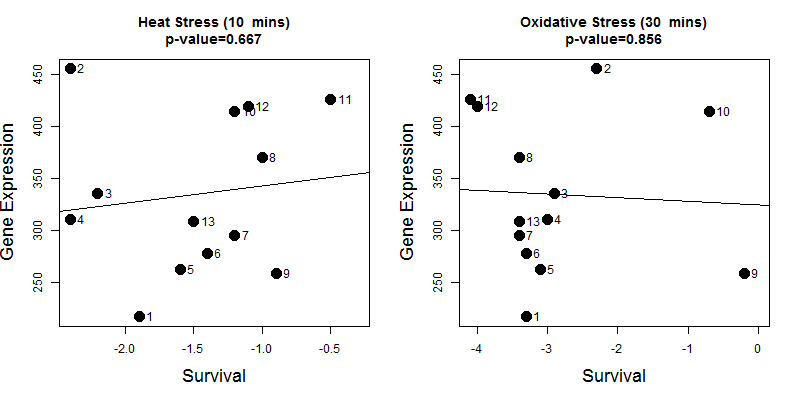

Supplement: S5 File — Expression levels of genes LACR_0001 –LACR_1382 plotted against survival after 10 minutes heat and 30 minutes oxidative stress. Survival is expressed as the difference of log CFU/ml after stress and before stress. Numbers indicate fermentations as presented in Table 1. P-values above the plots indicate significance of correlation (assessed by a linear model). (ZIP) [file pone.0167944.s010.zip › S5_File/LACR_0202_real_dat.png]

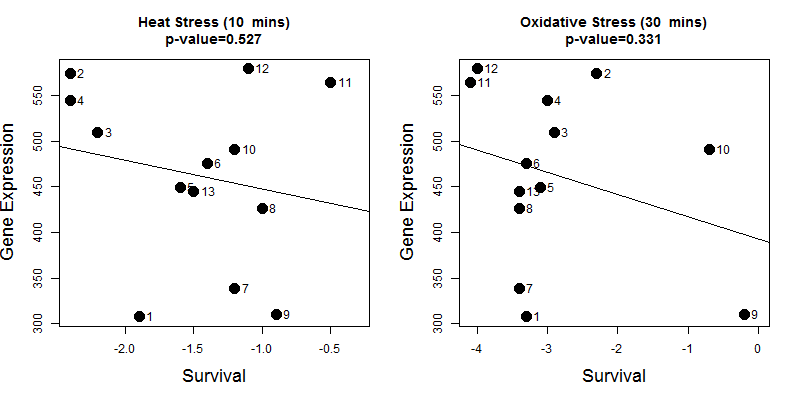

Supplement: S5 File — Expression levels of genes LACR_0001 –LACR_1382 plotted against survival after 10 minutes heat and 30 minutes oxidative stress. Survival is expressed as the difference of log CFU/ml after stress and before stress. Numbers indicate fermentations as presented in Table 1. P-values above the plots indicate significance of correlation (assessed by a linear model). (ZIP) [file pone.0167944.s010.zip › S5_File/LACR_0203_real_dat.png]

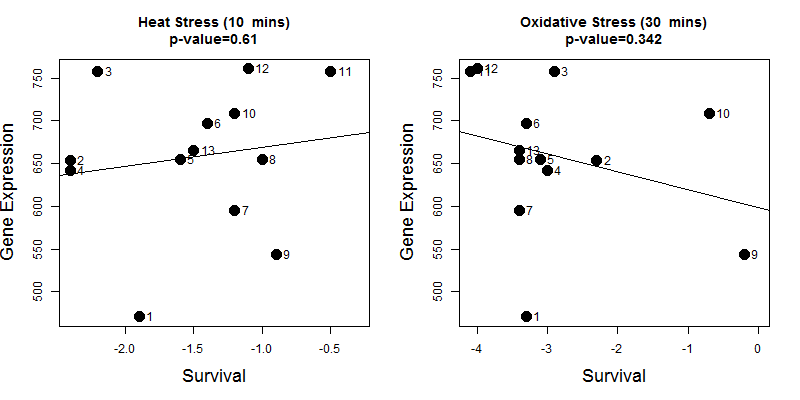

Supplement: S5 File — Expression levels of genes LACR_0001 –LACR_1382 plotted against survival after 10 minutes heat and 30 minutes oxidative stress. Survival is expressed as the difference of log CFU/ml after stress and before stress. Numbers indicate fermentations as presented in Table 1. P-values above the plots indicate significance of correlation (assessed by a linear model). (ZIP) [file pone.0167944.s010.zip › S5_File/LACR_0204_real_dat.png]

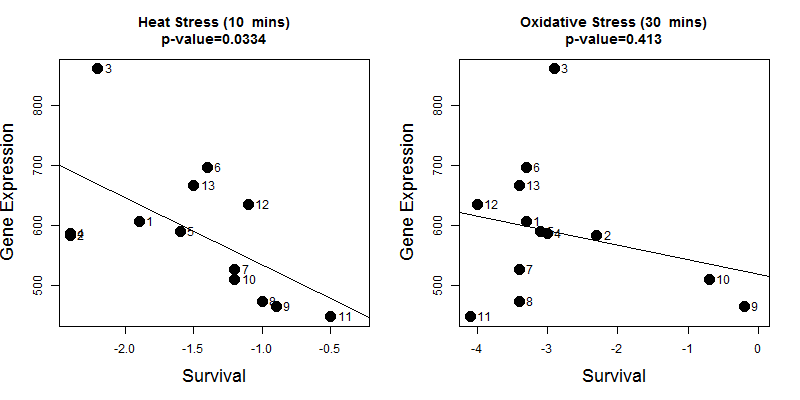

Supplement: S5 File — Expression levels of genes LACR_0001 –LACR_1382 plotted against survival after 10 minutes heat and 30 minutes oxidative stress. Survival is expressed as the difference of log CFU/ml after stress and before stress. Numbers indicate fermentations as presented in Table 1. P-values above the plots indicate significance of correlation (assessed by a linear model). (ZIP) [file pone.0167944.s010.zip › S5_File/LACR_0205_real_dat.png]

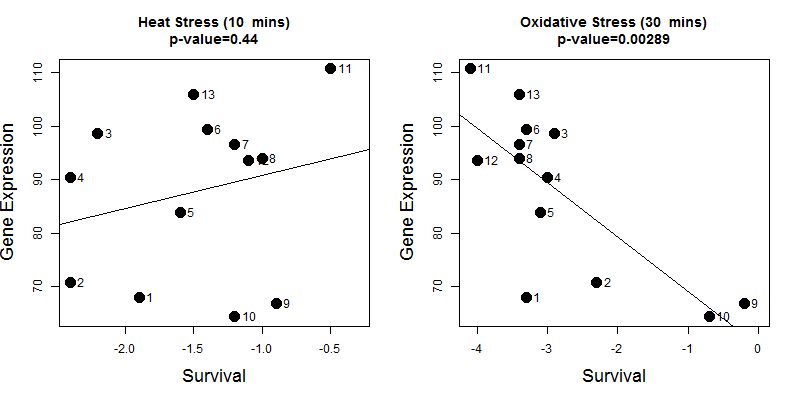

Supplement: S5 File — Expression levels of genes LACR_0001 –LACR_1382 plotted against survival after 10 minutes heat and 30 minutes oxidative stress. Survival is expressed as the difference of log CFU/ml after stress and before stress. Numbers indicate fermentations as presented in Table 1. P-values above the plots indicate significance of correlation (assessed by a linear model). (ZIP) [file pone.0167944.s010.zip › S5_File/LACR_0206_real_dat.png]

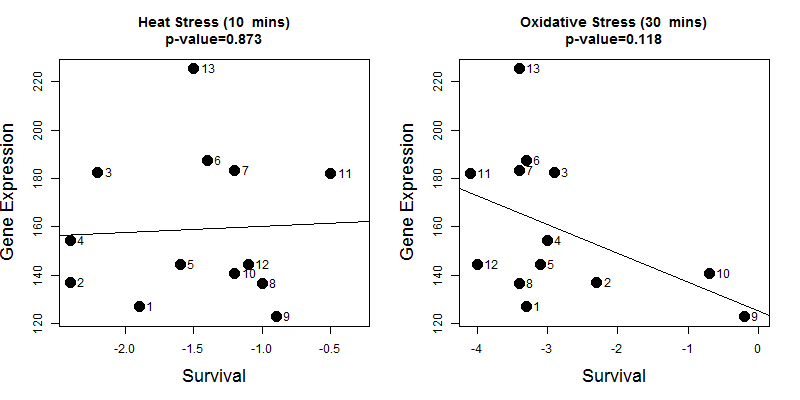

Supplement: S5 File — Expression levels of genes LACR_0001 –LACR_1382 plotted against survival after 10 minutes heat and 30 minutes oxidative stress. Survival is expressed as the difference of log CFU/ml after stress and before stress. Numbers indicate fermentations as presented in Table 1. P-values above the plots indicate significance of correlation (assessed by a linear model). (ZIP) [file pone.0167944.s010.zip › S5_File/LACR_0207_real_dat.png]

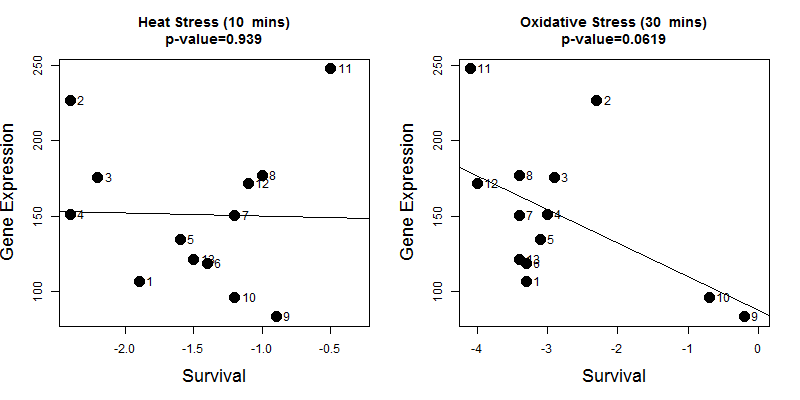

Supplement: S5 File — Expression levels of genes LACR_0001 –LACR_1382 plotted against survival after 10 minutes heat and 30 minutes oxidative stress. Survival is expressed as the difference of log CFU/ml after stress and before stress. Numbers indicate fermentations as presented in Table 1. P-values above the plots indicate significance of correlation (assessed by a linear model). (ZIP) [file pone.0167944.s010.zip › S5_File/LACR_0208_real_dat.png]

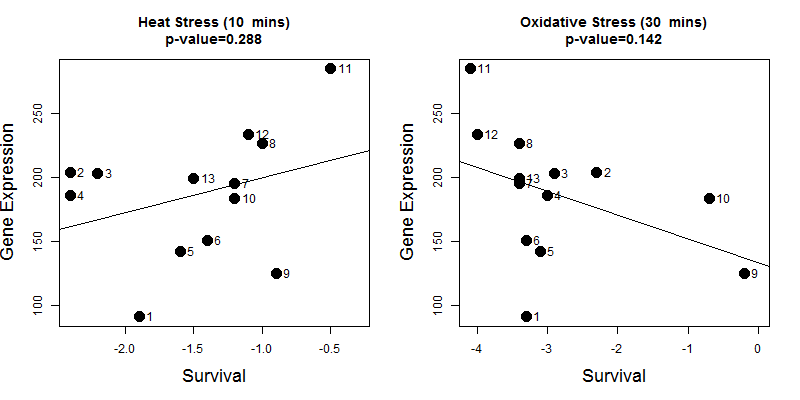

Supplement: S5 File — Expression levels of genes LACR_0001 –LACR_1382 plotted against survival after 10 minutes heat and 30 minutes oxidative stress. Survival is expressed as the difference of log CFU/ml after stress and before stress. Numbers indicate fermentations as presented in Table 1. P-values above the plots indicate significance of correlation (assessed by a linear model). (ZIP) [file pone.0167944.s010.zip › S5_File/LACR_0209_real_dat.png]

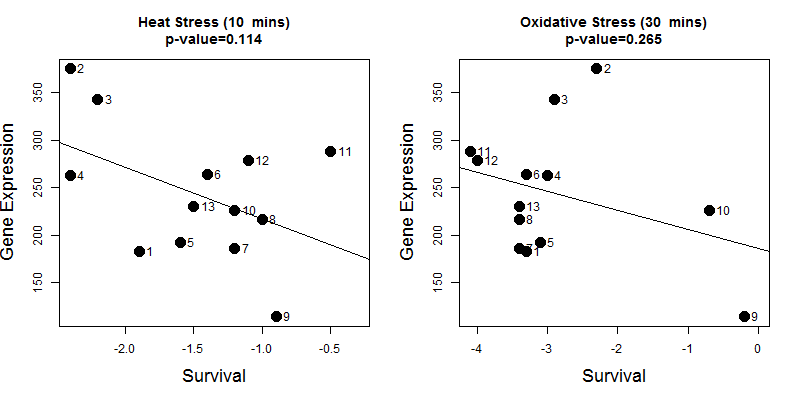

Supplement: S5 File — Expression levels of genes LACR_0001 –LACR_1382 plotted against survival after 10 minutes heat and 30 minutes oxidative stress. Survival is expressed as the difference of log CFU/ml after stress and before stress. Numbers indicate fermentations as presented in Table 1. P-values above the plots indicate significance of correlation (assessed by a linear model). (ZIP) [file pone.0167944.s010.zip › S5_File/LACR_0210_real_dat.png]

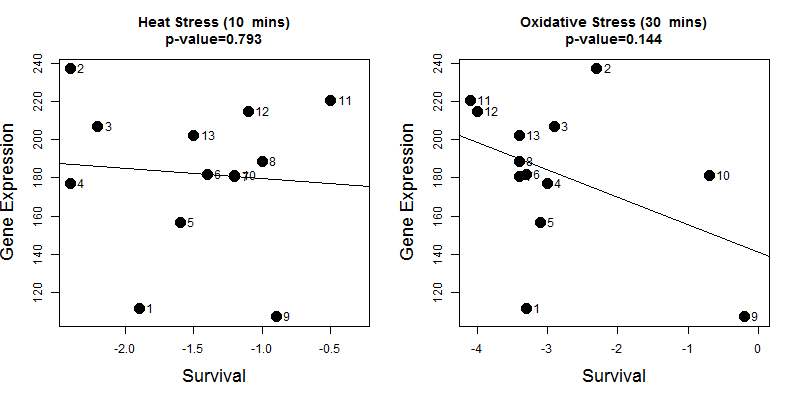

Supplement: S5 File — Expression levels of genes LACR_0001 –LACR_1382 plotted against survival after 10 minutes heat and 30 minutes oxidative stress. Survival is expressed as the difference of log CFU/ml after stress and before stress. Numbers indicate fermentations as presented in Table 1. P-values above the plots indicate significance of correlation (assessed by a linear model). (ZIP) [file pone.0167944.s010.zip › S5_File/LACR_0211_real_dat.png]

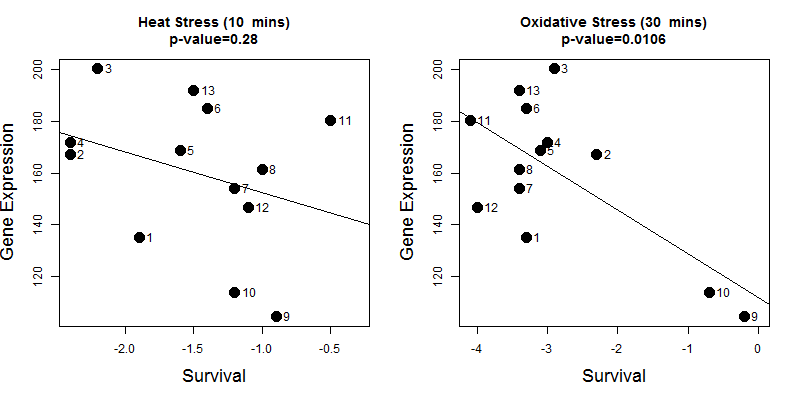

Supplement: S5 File — Expression levels of genes LACR_0001 –LACR_1382 plotted against survival after 10 minutes heat and 30 minutes oxidative stress. Survival is expressed as the difference of log CFU/ml after stress and before stress. Numbers indicate fermentations as presented in Table 1. P-values above the plots indicate significance of correlation (assessed by a linear model). (ZIP) [file pone.0167944.s010.zip › S5_File/LACR_0212_real_dat.png]

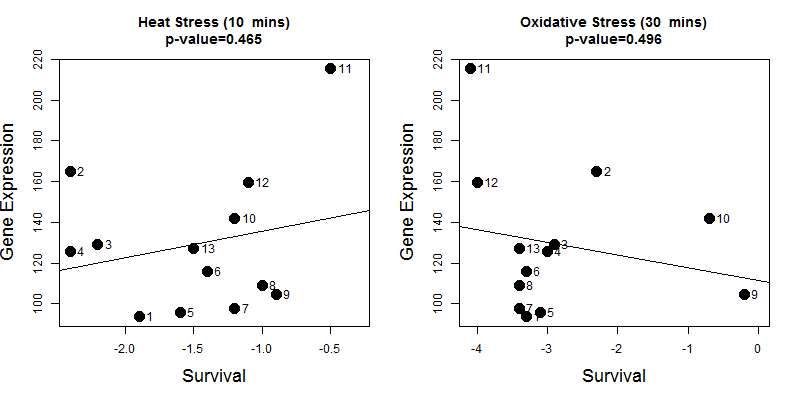

Supplement: S5 File — Expression levels of genes LACR_0001 –LACR_1382 plotted against survival after 10 minutes heat and 30 minutes oxidative stress. Survival is expressed as the difference of log CFU/ml after stress and before stress. Numbers indicate fermentations as presented in Table 1. P-values above the plots indicate significance of correlation (assessed by a linear model). (ZIP) [file pone.0167944.s010.zip › S5_File/LACR_0213_real_dat.png]

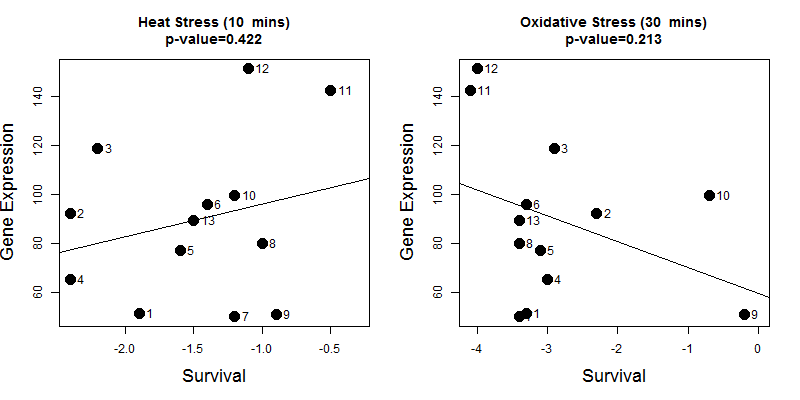

Supplement: S5 File — Expression levels of genes LACR_0001 –LACR_1382 plotted against survival after 10 minutes heat and 30 minutes oxidative stress. Survival is expressed as the difference of log CFU/ml after stress and before stress. Numbers indicate fermentations as presented in Table 1. P-values above the plots indicate significance of correlation (assessed by a linear model). (ZIP) [file pone.0167944.s010.zip › S5_File/LACR_0214_real_dat.png]

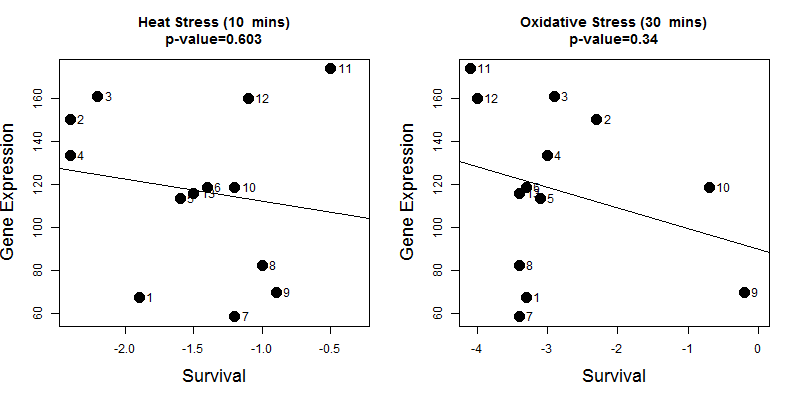

Supplement: S5 File — Expression levels of genes LACR_0001 –LACR_1382 plotted against survival after 10 minutes heat and 30 minutes oxidative stress. Survival is expressed as the difference of log CFU/ml after stress and before stress. Numbers indicate fermentations as presented in Table 1. P-values above the plots indicate significance of correlation (assessed by a linear model). (ZIP) [file pone.0167944.s010.zip › S5_File/LACR_0215_real_dat.png]

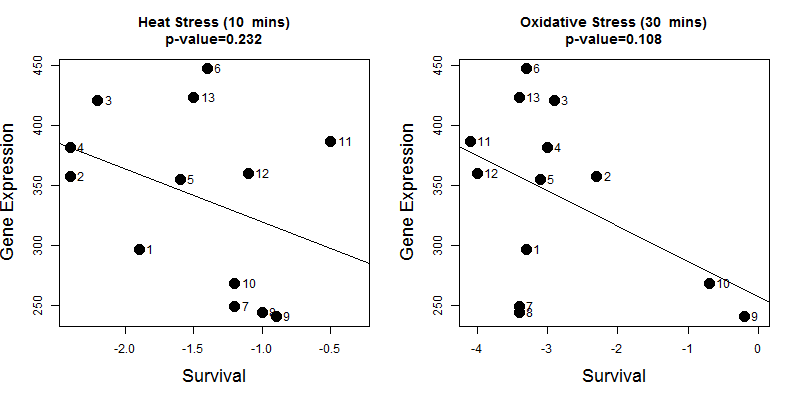

Supplement: S5 File — Expression levels of genes LACR_0001 –LACR_1382 plotted against survival after 10 minutes heat and 30 minutes oxidative stress. Survival is expressed as the difference of log CFU/ml after stress and before stress. Numbers indicate fermentations as presented in Table 1. P-values above the plots indicate significance of correlation (assessed by a linear model). (ZIP) [file pone.0167944.s010.zip › S5_File/LACR_0216_real_dat.png]

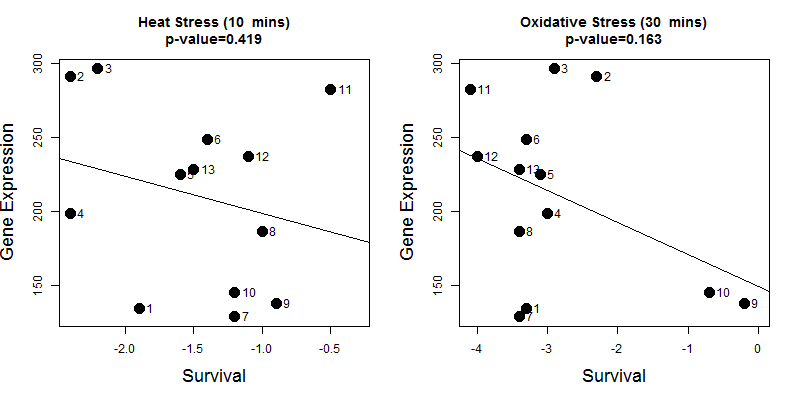

Supplement: S5 File — Expression levels of genes LACR_0001 –LACR_1382 plotted against survival after 10 minutes heat and 30 minutes oxidative stress. Survival is expressed as the difference of log CFU/ml after stress and before stress. Numbers indicate fermentations as presented in Table 1. P-values above the plots indicate significance of correlation (assessed by a linear model). (ZIP) [file pone.0167944.s010.zip › S5_File/LACR_0217_real_dat.png]

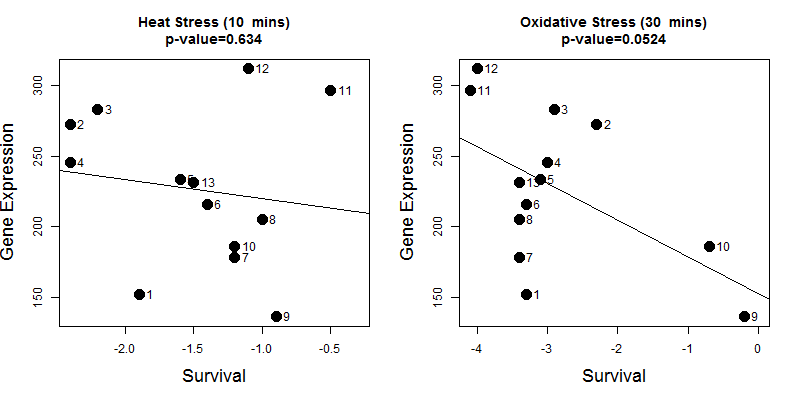

Supplement: S5 File — Expression levels of genes LACR_0001 –LACR_1382 plotted against survival after 10 minutes heat and 30 minutes oxidative stress. Survival is expressed as the difference of log CFU/ml after stress and before stress. Numbers indicate fermentations as presented in Table 1. P-values above the plots indicate significance of correlation (assessed by a linear model). (ZIP) [file pone.0167944.s010.zip › S5_File/LACR_0218_real_dat.png]

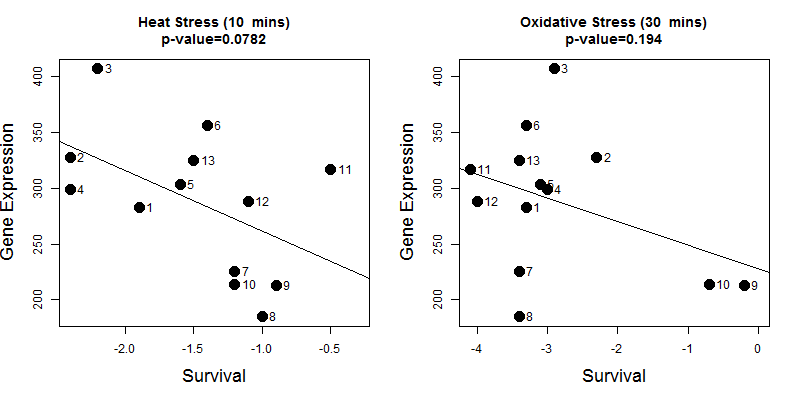

Supplement: S5 File — Expression levels of genes LACR_0001 –LACR_1382 plotted against survival after 10 minutes heat and 30 minutes oxidative stress. Survival is expressed as the difference of log CFU/ml after stress and before stress. Numbers indicate fermentations as presented in Table 1. P-values above the plots indicate significance of correlation (assessed by a linear model). (ZIP) [file pone.0167944.s010.zip › S5_File/LACR_0219_real_dat.png]

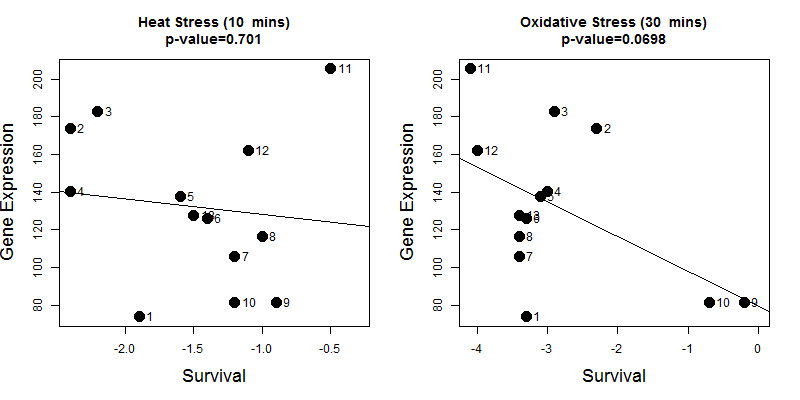

Supplement: S5 File — Expression levels of genes LACR_0001 –LACR_1382 plotted against survival after 10 minutes heat and 30 minutes oxidative stress. Survival is expressed as the difference of log CFU/ml after stress and before stress. Numbers indicate fermentations as presented in Table 1. P-values above the plots indicate significance of correlation (assessed by a linear model). (ZIP) [file pone.0167944.s010.zip › S5_File/LACR_0220_real_dat.png]

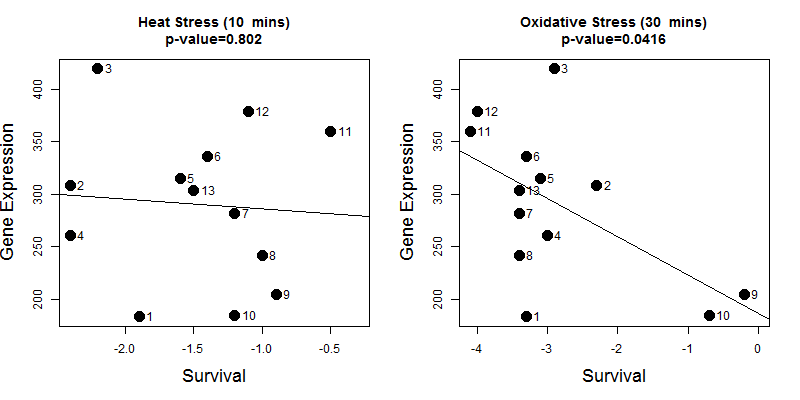

Supplement: S5 File — Expression levels of genes LACR_0001 –LACR_1382 plotted against survival after 10 minutes heat and 30 minutes oxidative stress. Survival is expressed as the difference of log CFU/ml after stress and before stress. Numbers indicate fermentations as presented in Table 1. P-values above the plots indicate significance of correlation (assessed by a linear model). (ZIP) [file pone.0167944.s010.zip › S5_File/LACR_0221_real_dat.png]

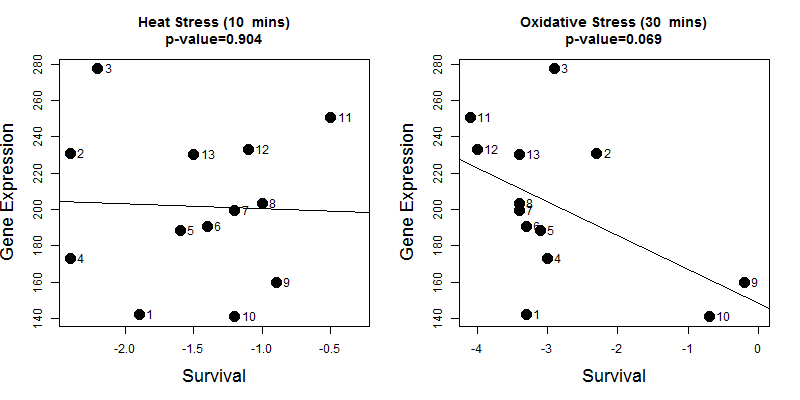

Supplement: S5 File — Expression levels of genes LACR_0001 –LACR_1382 plotted against survival after 10 minutes heat and 30 minutes oxidative stress. Survival is expressed as the difference of log CFU/ml after stress and before stress. Numbers indicate fermentations as presented in Table 1. P-values above the plots indicate significance of correlation (assessed by a linear model). (ZIP) [file pone.0167944.s010.zip › S5_File/LACR_0222_real_dat.png]
