# Supplementary figures and images for: Strain-Dependent Transcriptome Signatures for Robustness in Lactococcus lactis (part 13 of 13)
Source: PLoS One. 2016 Dec 14;11(12):e0167944. doi: 10.1371/journal.pone.0167944 (PMC5156439; doi:10.1371/journal.pone.0167944)

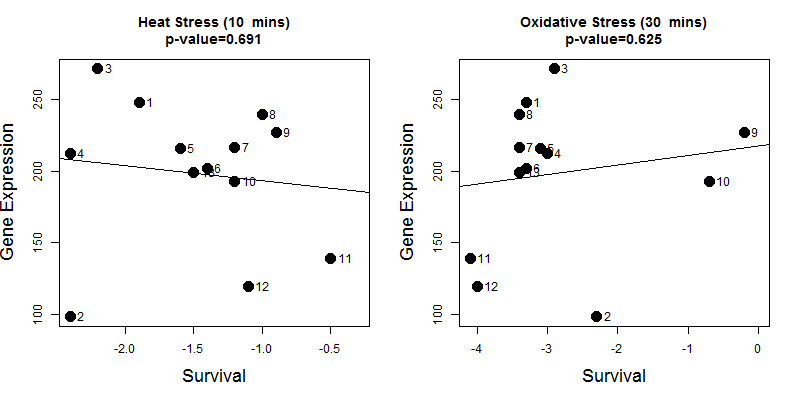

Supplement: S6 File — Expression levels of genes LACR_1383 –LACR_2610 and LACR_A01 –LACR_E8 plotted against survival after 10 minutes heat and 30 minutes oxidative stress. Survival is expressed as the difference of log CFU/ml after stress and before stress. Numbers indicate fermentations as presented in Table 1. P-values above the plots indicate significance of correlation (assessed by a linear model). (ZIP) [file pone.0167944.s011.zip › S6_File/LACR_1604_real_dat.png]

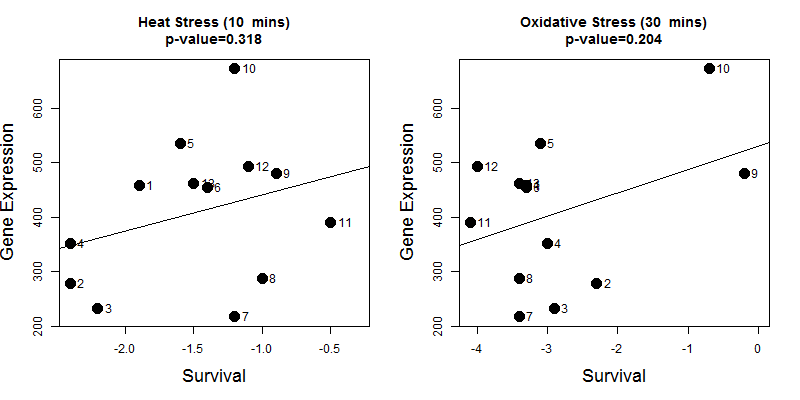

Supplement: S6 File — Expression levels of genes LACR_1383 –LACR_2610 and LACR_A01 –LACR_E8 plotted against survival after 10 minutes heat and 30 minutes oxidative stress. Survival is expressed as the difference of log CFU/ml after stress and before stress. Numbers indicate fermentations as presented in Table 1. P-values above the plots indicate significance of correlation (assessed by a linear model). (ZIP) [file pone.0167944.s011.zip › S6_File/LACR_1605_real_dat.png]

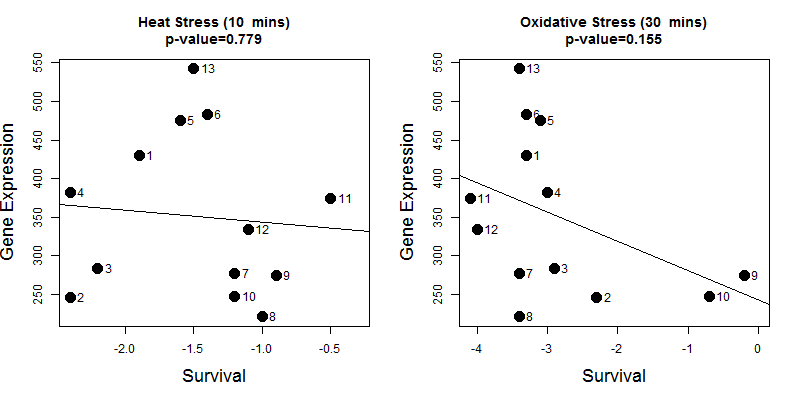

Supplement: S6 File — Expression levels of genes LACR_1383 –LACR_2610 and LACR_A01 –LACR_E8 plotted against survival after 10 minutes heat and 30 minutes oxidative stress. Survival is expressed as the difference of log CFU/ml after stress and before stress. Numbers indicate fermentations as presented in Table 1. P-values above the plots indicate significance of correlation (assessed by a linear model). (ZIP) [file pone.0167944.s011.zip › S6_File/LACR_1606_real_dat.png]
