# Supplementary figures and images for: Strain-Dependent Transcriptome Signatures for Robustness in Lactococcus lactis (part 12 of 13)
Source: PLoS One. 2016 Dec 14;11(12):e0167944. doi: 10.1371/journal.pone.0167944 (PMC5156439; doi:10.1371/journal.pone.0167944)

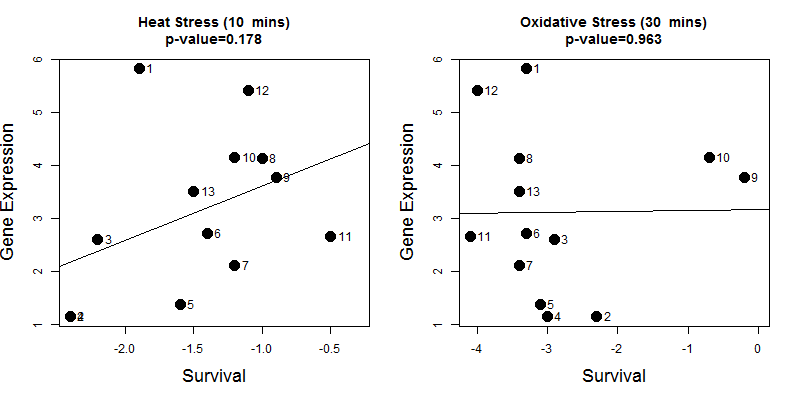

Supplement: S6 File — Expression levels of genes LACR_1383 –LACR_2610 and LACR_A01 –LACR_E8 plotted against survival after 10 minutes heat and 30 minutes oxidative stress. Survival is expressed as the difference of log CFU/ml after stress and before stress. Numbers indicate fermentations as presented in Table 1. P-values above the plots indicate significance of correlation (assessed by a linear model). (ZIP) [file pone.0167944.s011.zip › S6_File/LACR_1491_real_dat.png]

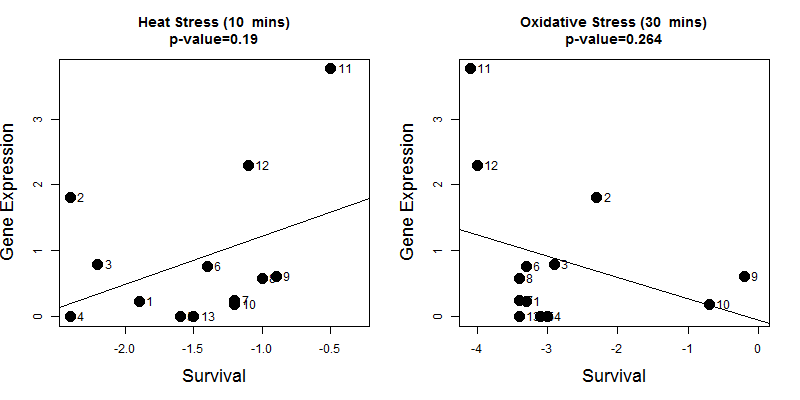

Supplement: S6 File — Expression levels of genes LACR_1383 –LACR_2610 and LACR_A01 –LACR_E8 plotted against survival after 10 minutes heat and 30 minutes oxidative stress. Survival is expressed as the difference of log CFU/ml after stress and before stress. Numbers indicate fermentations as presented in Table 1. P-values above the plots indicate significance of correlation (assessed by a linear model). (ZIP) [file pone.0167944.s011.zip › S6_File/LACR_1492_real_dat.png]

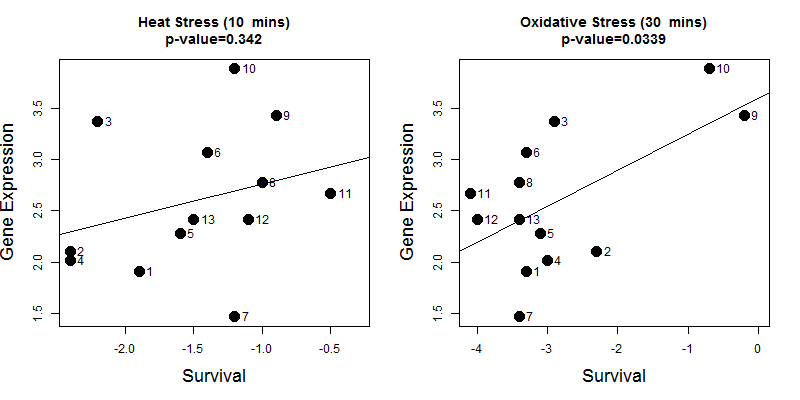

Supplement: S6 File — Expression levels of genes LACR_1383 –LACR_2610 and LACR_A01 –LACR_E8 plotted against survival after 10 minutes heat and 30 minutes oxidative stress. Survival is expressed as the difference of log CFU/ml after stress and before stress. Numbers indicate fermentations as presented in Table 1. P-values above the plots indicate significance of correlation (assessed by a linear model). (ZIP) [file pone.0167944.s011.zip › S6_File/LACR_1495_real_dat.png]

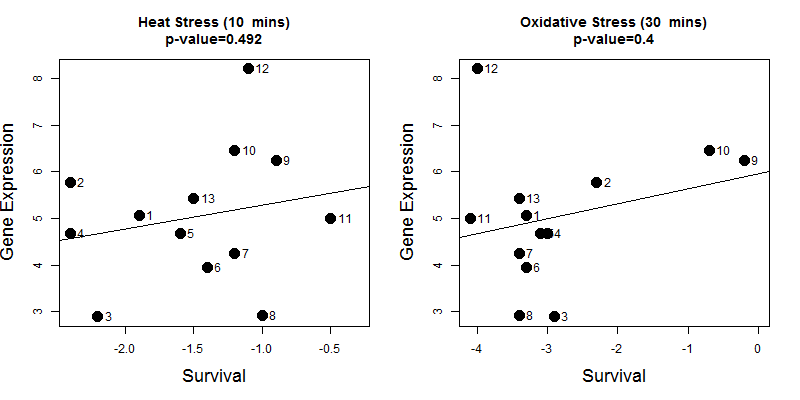

Supplement: S6 File — Expression levels of genes LACR_1383 –LACR_2610 and LACR_A01 –LACR_E8 plotted against survival after 10 minutes heat and 30 minutes oxidative stress. Survival is expressed as the difference of log CFU/ml after stress and before stress. Numbers indicate fermentations as presented in Table 1. P-values above the plots indicate significance of correlation (assessed by a linear model). (ZIP) [file pone.0167944.s011.zip › S6_File/LACR_1496_real_dat.png]

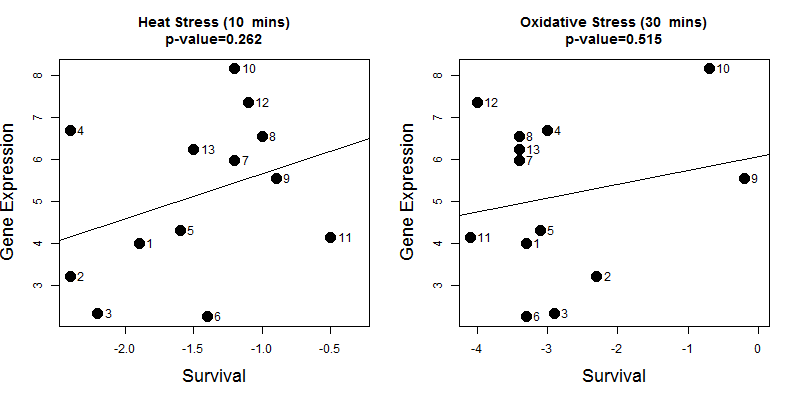

Supplement: S6 File — Expression levels of genes LACR_1383 –LACR_2610 and LACR_A01 –LACR_E8 plotted against survival after 10 minutes heat and 30 minutes oxidative stress. Survival is expressed as the difference of log CFU/ml after stress and before stress. Numbers indicate fermentations as presented in Table 1. P-values above the plots indicate significance of correlation (assessed by a linear model). (ZIP) [file pone.0167944.s011.zip › S6_File/LACR_1497_real_dat.png]

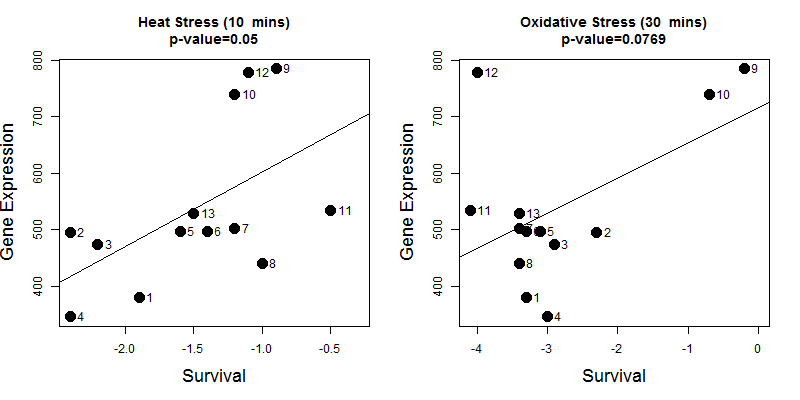

Supplement: S6 File — Expression levels of genes LACR_1383 –LACR_2610 and LACR_A01 –LACR_E8 plotted against survival after 10 minutes heat and 30 minutes oxidative stress. Survival is expressed as the difference of log CFU/ml after stress and before stress. Numbers indicate fermentations as presented in Table 1. P-values above the plots indicate significance of correlation (assessed by a linear model). (ZIP) [file pone.0167944.s011.zip › S6_File/LACR_1498_real_dat.png]

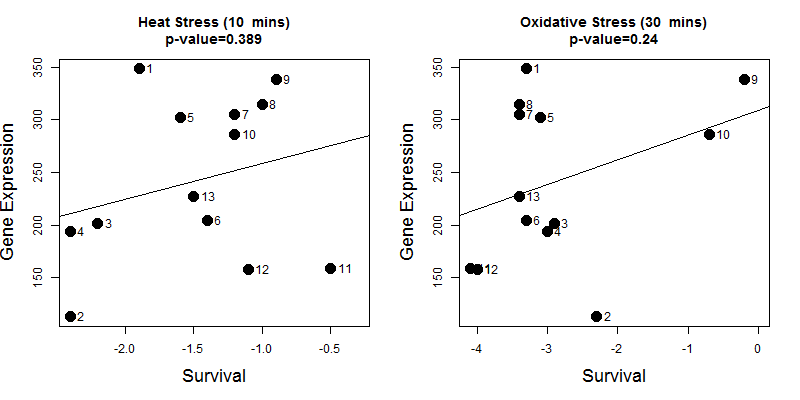

Supplement: S6 File — Expression levels of genes LACR_1383 –LACR_2610 and LACR_A01 –LACR_E8 plotted against survival after 10 minutes heat and 30 minutes oxidative stress. Survival is expressed as the difference of log CFU/ml after stress and before stress. Numbers indicate fermentations as presented in Table 1. P-values above the plots indicate significance of correlation (assessed by a linear model). (ZIP) [file pone.0167944.s011.zip › S6_File/LACR_1499_real_dat.png]

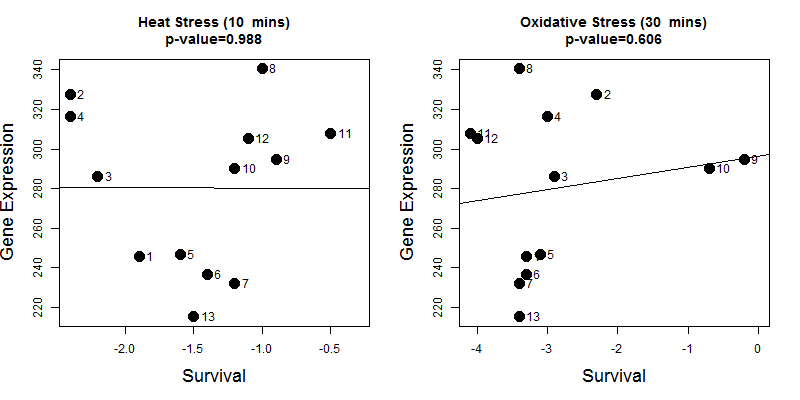

Supplement: S6 File — Expression levels of genes LACR_1383 –LACR_2610 and LACR_A01 –LACR_E8 plotted against survival after 10 minutes heat and 30 minutes oxidative stress. Survival is expressed as the difference of log CFU/ml after stress and before stress. Numbers indicate fermentations as presented in Table 1. P-values above the plots indicate significance of correlation (assessed by a linear model). (ZIP) [file pone.0167944.s011.zip › S6_File/LACR_1501_real_dat.png]

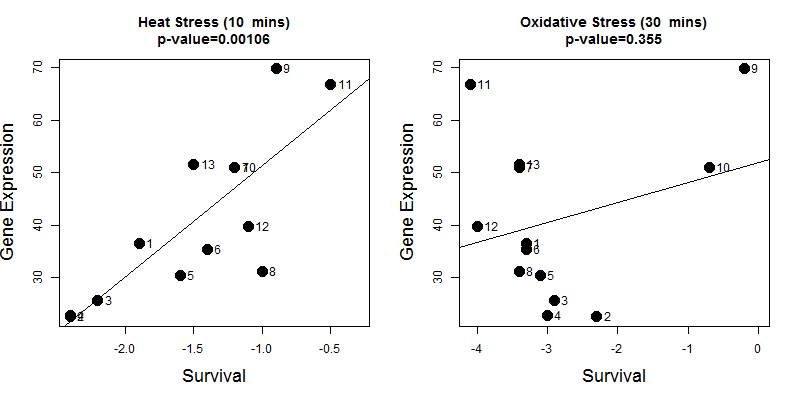

Supplement: S6 File — Expression levels of genes LACR_1383 –LACR_2610 and LACR_A01 –LACR_E8 plotted against survival after 10 minutes heat and 30 minutes oxidative stress. Survival is expressed as the difference of log CFU/ml after stress and before stress. Numbers indicate fermentations as presented in Table 1. P-values above the plots indicate significance of correlation (assessed by a linear model). (ZIP) [file pone.0167944.s011.zip › S6_File/LACR_1502_real_dat.png]

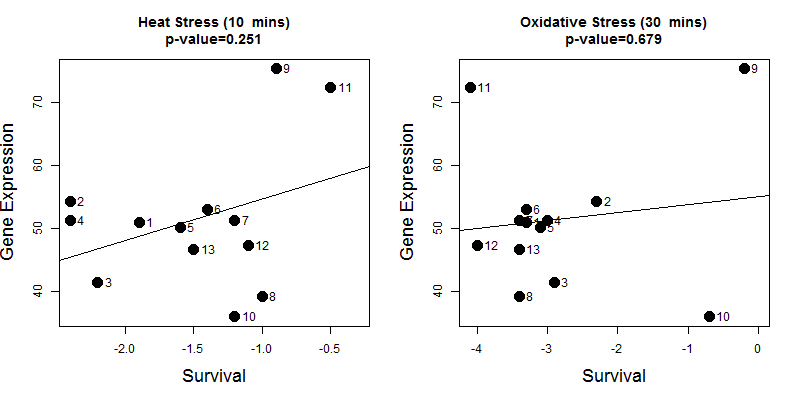

Supplement: S6 File — Expression levels of genes LACR_1383 –LACR_2610 and LACR_A01 –LACR_E8 plotted against survival after 10 minutes heat and 30 minutes oxidative stress. Survival is expressed as the difference of log CFU/ml after stress and before stress. Numbers indicate fermentations as presented in Table 1. P-values above the plots indicate significance of correlation (assessed by a linear model). (ZIP) [file pone.0167944.s011.zip › S6_File/LACR_1503_real_dat.png]

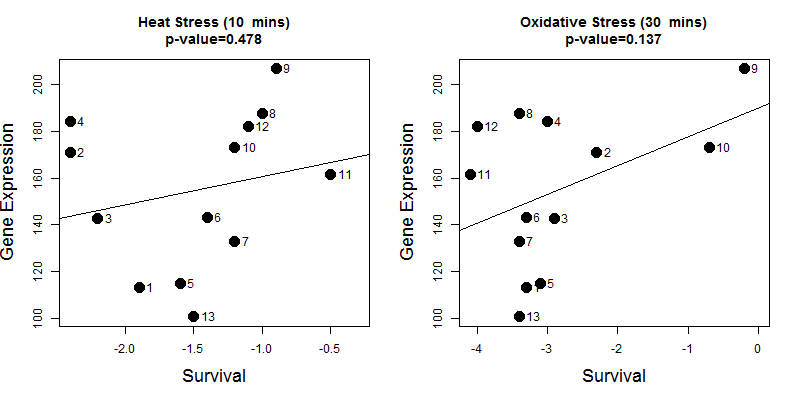

Supplement: S6 File — Expression levels of genes LACR_1383 –LACR_2610 and LACR_A01 –LACR_E8 plotted against survival after 10 minutes heat and 30 minutes oxidative stress. Survival is expressed as the difference of log CFU/ml after stress and before stress. Numbers indicate fermentations as presented in Table 1. P-values above the plots indicate significance of correlation (assessed by a linear model). (ZIP) [file pone.0167944.s011.zip › S6_File/LACR_1504_real_dat.png]

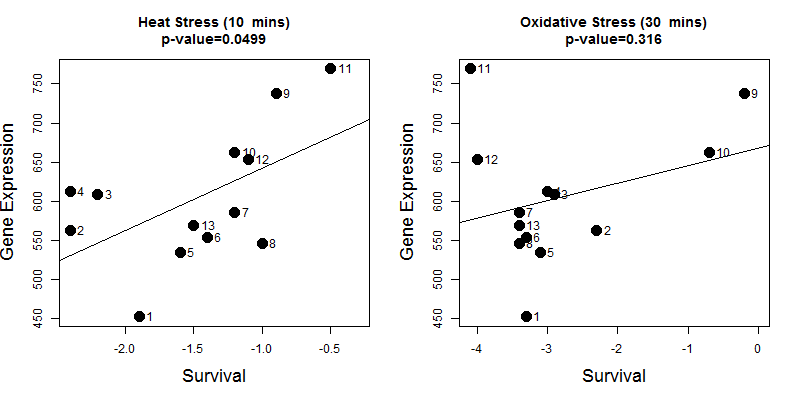

Supplement: S6 File — Expression levels of genes LACR_1383 –LACR_2610 and LACR_A01 –LACR_E8 plotted against survival after 10 minutes heat and 30 minutes oxidative stress. Survival is expressed as the difference of log CFU/ml after stress and before stress. Numbers indicate fermentations as presented in Table 1. P-values above the plots indicate significance of correlation (assessed by a linear model). (ZIP) [file pone.0167944.s011.zip › S6_File/LACR_1505_real_dat.png]

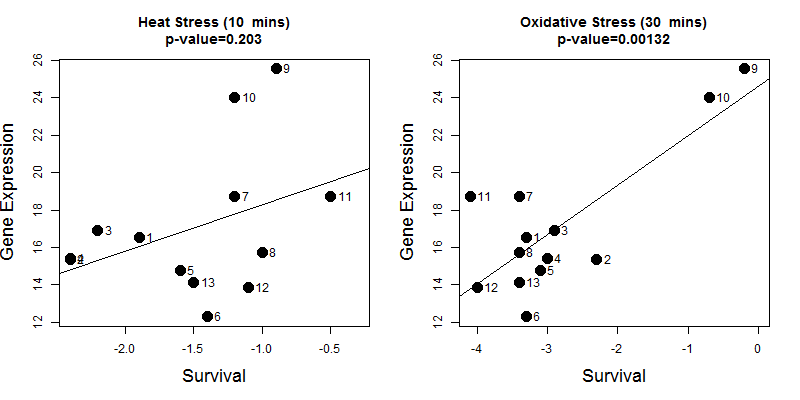

Supplement: S6 File — Expression levels of genes LACR_1383 –LACR_2610 and LACR_A01 –LACR_E8 plotted against survival after 10 minutes heat and 30 minutes oxidative stress. Survival is expressed as the difference of log CFU/ml after stress and before stress. Numbers indicate fermentations as presented in Table 1. P-values above the plots indicate significance of correlation (assessed by a linear model). (ZIP) [file pone.0167944.s011.zip › S6_File/LACR_1506_real_dat.png]

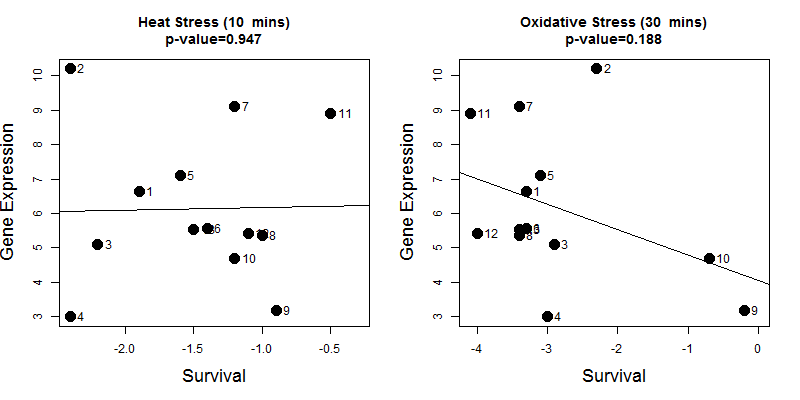

Supplement: S6 File — Expression levels of genes LACR_1383 –LACR_2610 and LACR_A01 –LACR_E8 plotted against survival after 10 minutes heat and 30 minutes oxidative stress. Survival is expressed as the difference of log CFU/ml after stress and before stress. Numbers indicate fermentations as presented in Table 1. P-values above the plots indicate significance of correlation (assessed by a linear model). (ZIP) [file pone.0167944.s011.zip › S6_File/LACR_1507_real_dat.png]

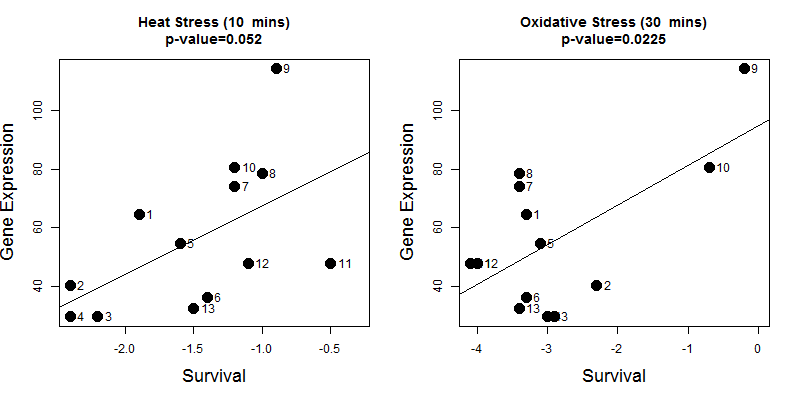

Supplement: S6 File — Expression levels of genes LACR_1383 –LACR_2610 and LACR_A01 –LACR_E8 plotted against survival after 10 minutes heat and 30 minutes oxidative stress. Survival is expressed as the difference of log CFU/ml after stress and before stress. Numbers indicate fermentations as presented in Table 1. P-values above the plots indicate significance of correlation (assessed by a linear model). (ZIP) [file pone.0167944.s011.zip › S6_File/LACR_1508_real_dat.png]

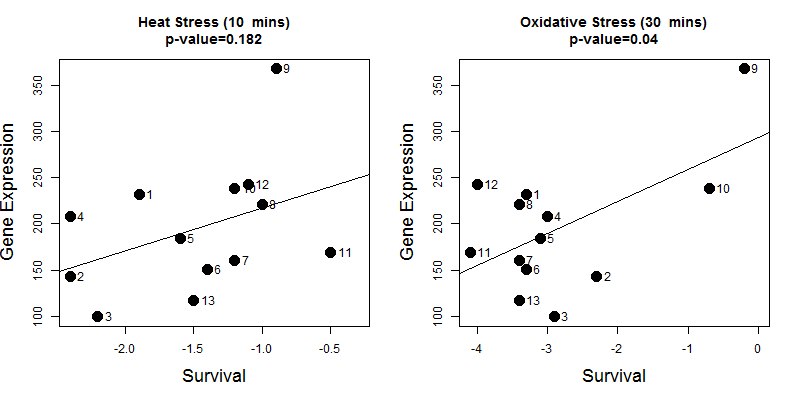

Supplement: S6 File — Expression levels of genes LACR_1383 –LACR_2610 and LACR_A01 –LACR_E8 plotted against survival after 10 minutes heat and 30 minutes oxidative stress. Survival is expressed as the difference of log CFU/ml after stress and before stress. Numbers indicate fermentations as presented in Table 1. P-values above the plots indicate significance of correlation (assessed by a linear model). (ZIP) [file pone.0167944.s011.zip › S6_File/LACR_1509_real_dat.png]

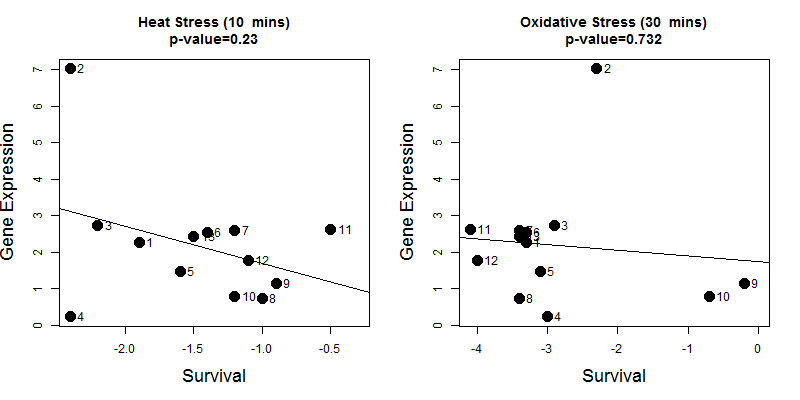

Supplement: S6 File — Expression levels of genes LACR_1383 –LACR_2610 and LACR_A01 –LACR_E8 plotted against survival after 10 minutes heat and 30 minutes oxidative stress. Survival is expressed as the difference of log CFU/ml after stress and before stress. Numbers indicate fermentations as presented in Table 1. P-values above the plots indicate significance of correlation (assessed by a linear model). (ZIP) [file pone.0167944.s011.zip › S6_File/LACR_1510_real_dat.png]

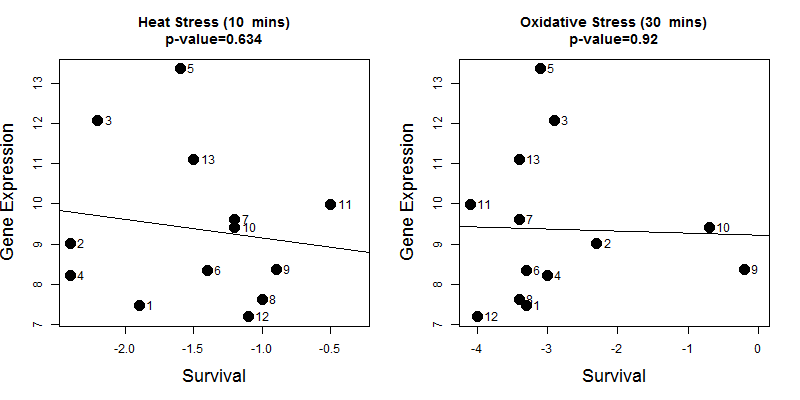

Supplement: S6 File — Expression levels of genes LACR_1383 –LACR_2610 and LACR_A01 –LACR_E8 plotted against survival after 10 minutes heat and 30 minutes oxidative stress. Survival is expressed as the difference of log CFU/ml after stress and before stress. Numbers indicate fermentations as presented in Table 1. P-values above the plots indicate significance of correlation (assessed by a linear model). (ZIP) [file pone.0167944.s011.zip › S6_File/LACR_1511_real_dat.png]

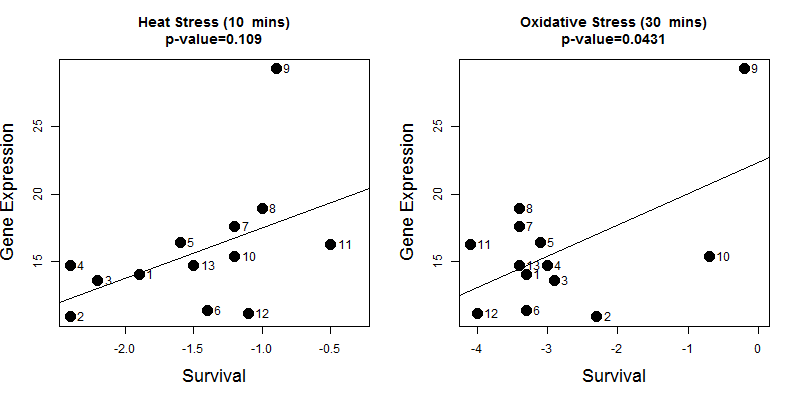

Supplement: S6 File — Expression levels of genes LACR_1383 –LACR_2610 and LACR_A01 –LACR_E8 plotted against survival after 10 minutes heat and 30 minutes oxidative stress. Survival is expressed as the difference of log CFU/ml after stress and before stress. Numbers indicate fermentations as presented in Table 1. P-values above the plots indicate significance of correlation (assessed by a linear model). (ZIP) [file pone.0167944.s011.zip › S6_File/LACR_1512_real_dat.png]

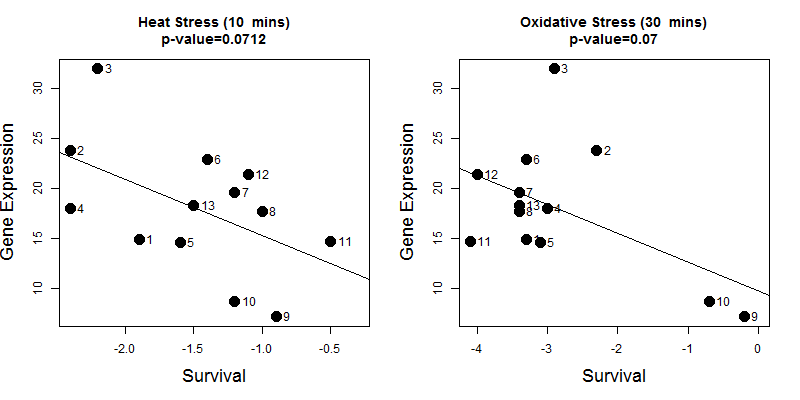

Supplement: S6 File — Expression levels of genes LACR_1383 –LACR_2610 and LACR_A01 –LACR_E8 plotted against survival after 10 minutes heat and 30 minutes oxidative stress. Survival is expressed as the difference of log CFU/ml after stress and before stress. Numbers indicate fermentations as presented in Table 1. P-values above the plots indicate significance of correlation (assessed by a linear model). (ZIP) [file pone.0167944.s011.zip › S6_File/LACR_1513_real_dat.png]

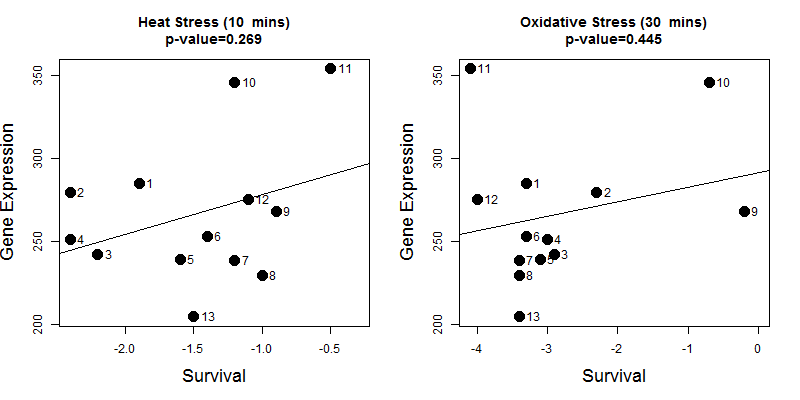

Supplement: S6 File — Expression levels of genes LACR_1383 –LACR_2610 and LACR_A01 –LACR_E8 plotted against survival after 10 minutes heat and 30 minutes oxidative stress. Survival is expressed as the difference of log CFU/ml after stress and before stress. Numbers indicate fermentations as presented in Table 1. P-values above the plots indicate significance of correlation (assessed by a linear model). (ZIP) [file pone.0167944.s011.zip › S6_File/LACR_1514_real_dat.png]

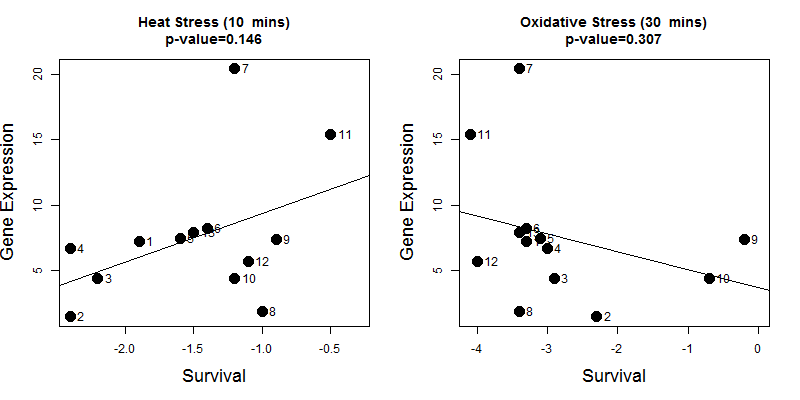

Supplement: S6 File — Expression levels of genes LACR_1383 –LACR_2610 and LACR_A01 –LACR_E8 plotted against survival after 10 minutes heat and 30 minutes oxidative stress. Survival is expressed as the difference of log CFU/ml after stress and before stress. Numbers indicate fermentations as presented in Table 1. P-values above the plots indicate significance of correlation (assessed by a linear model). (ZIP) [file pone.0167944.s011.zip › S6_File/LACR_1515_real_dat.png]

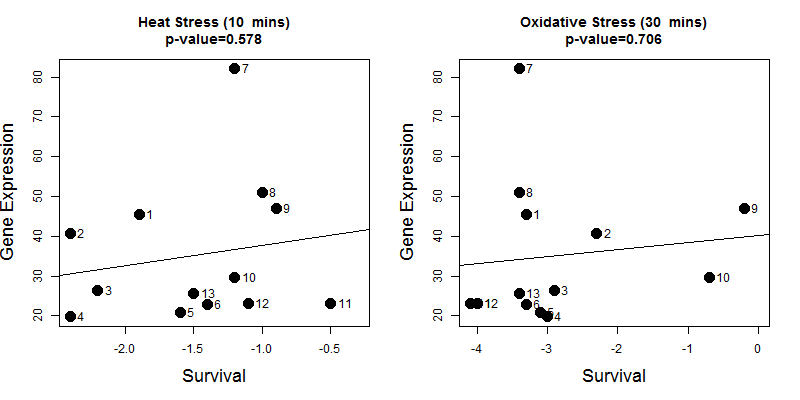

Supplement: S6 File — Expression levels of genes LACR_1383 –LACR_2610 and LACR_A01 –LACR_E8 plotted against survival after 10 minutes heat and 30 minutes oxidative stress. Survival is expressed as the difference of log CFU/ml after stress and before stress. Numbers indicate fermentations as presented in Table 1. P-values above the plots indicate significance of correlation (assessed by a linear model). (ZIP) [file pone.0167944.s011.zip › S6_File/LACR_1517_real_dat.png]

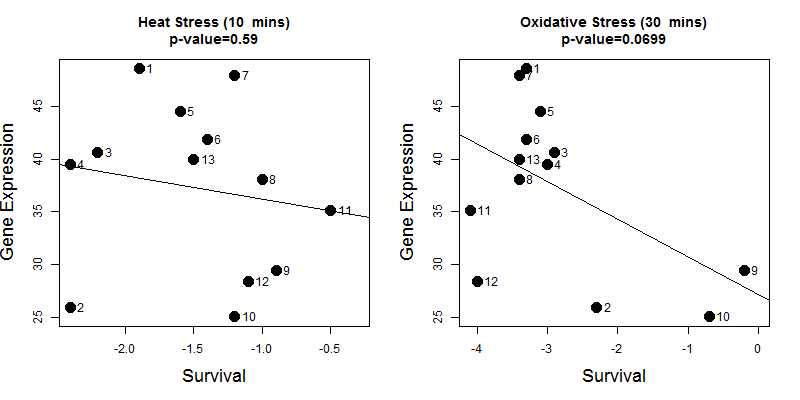

Supplement: S6 File — Expression levels of genes LACR_1383 –LACR_2610 and LACR_A01 –LACR_E8 plotted against survival after 10 minutes heat and 30 minutes oxidative stress. Survival is expressed as the difference of log CFU/ml after stress and before stress. Numbers indicate fermentations as presented in Table 1. P-values above the plots indicate significance of correlation (assessed by a linear model). (ZIP) [file pone.0167944.s011.zip › S6_File/LACR_1518_real_dat.png]

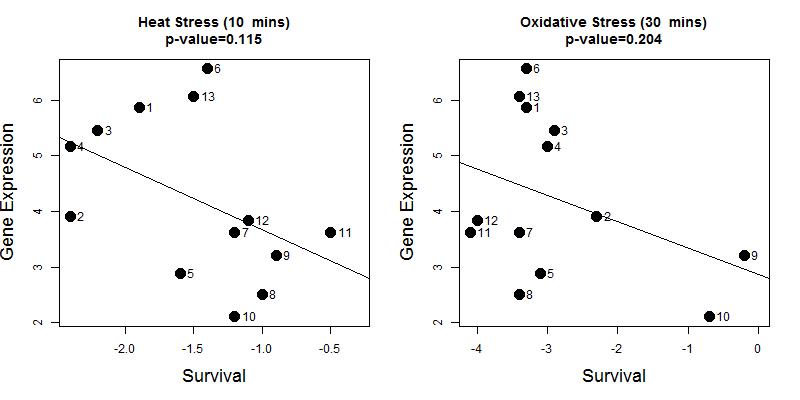

Supplement: S6 File — Expression levels of genes LACR_1383 –LACR_2610 and LACR_A01 –LACR_E8 plotted against survival after 10 minutes heat and 30 minutes oxidative stress. Survival is expressed as the difference of log CFU/ml after stress and before stress. Numbers indicate fermentations as presented in Table 1. P-values above the plots indicate significance of correlation (assessed by a linear model). (ZIP) [file pone.0167944.s011.zip › S6_File/LACR_1519_real_dat.png]

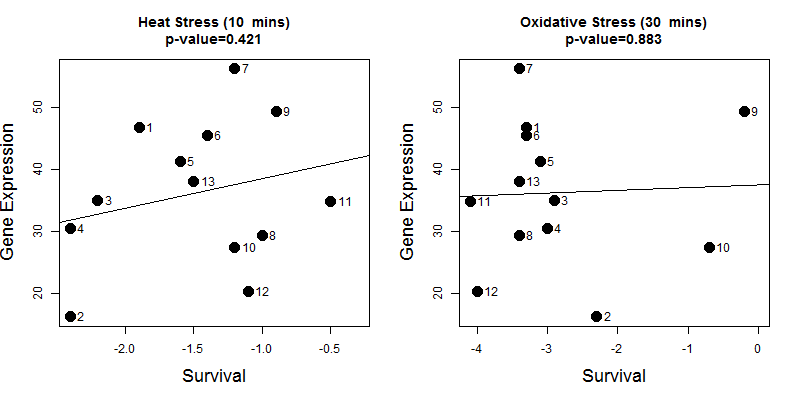

Supplement: S6 File — Expression levels of genes LACR_1383 –LACR_2610 and LACR_A01 –LACR_E8 plotted against survival after 10 minutes heat and 30 minutes oxidative stress. Survival is expressed as the difference of log CFU/ml after stress and before stress. Numbers indicate fermentations as presented in Table 1. P-values above the plots indicate significance of correlation (assessed by a linear model). (ZIP) [file pone.0167944.s011.zip › S6_File/LACR_1522_real_dat.png]

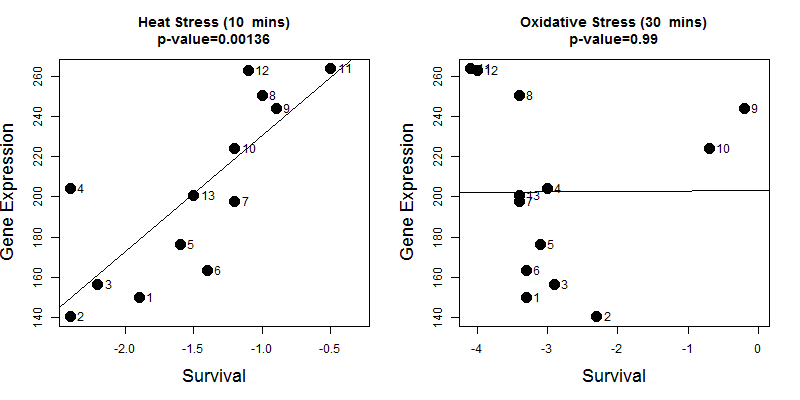

Supplement: S6 File — Expression levels of genes LACR_1383 –LACR_2610 and LACR_A01 –LACR_E8 plotted against survival after 10 minutes heat and 30 minutes oxidative stress. Survival is expressed as the difference of log CFU/ml after stress and before stress. Numbers indicate fermentations as presented in Table 1. P-values above the plots indicate significance of correlation (assessed by a linear model). (ZIP) [file pone.0167944.s011.zip › S6_File/LACR_1523_real_dat.png]

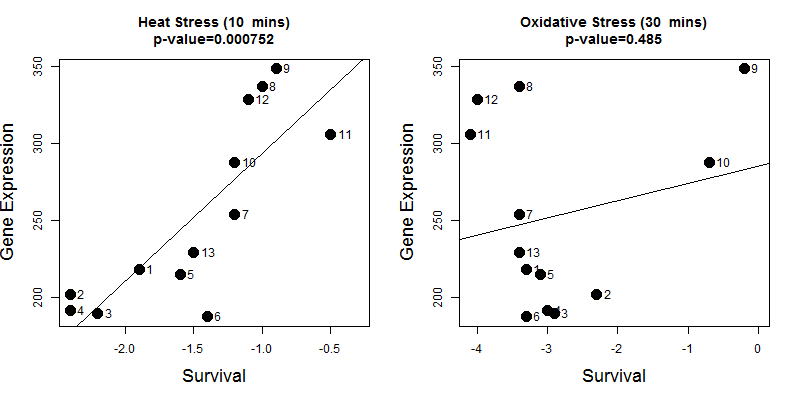

Supplement: S6 File — Expression levels of genes LACR_1383 –LACR_2610 and LACR_A01 –LACR_E8 plotted against survival after 10 minutes heat and 30 minutes oxidative stress. Survival is expressed as the difference of log CFU/ml after stress and before stress. Numbers indicate fermentations as presented in Table 1. P-values above the plots indicate significance of correlation (assessed by a linear model). (ZIP) [file pone.0167944.s011.zip › S6_File/LACR_1524_real_dat.png]

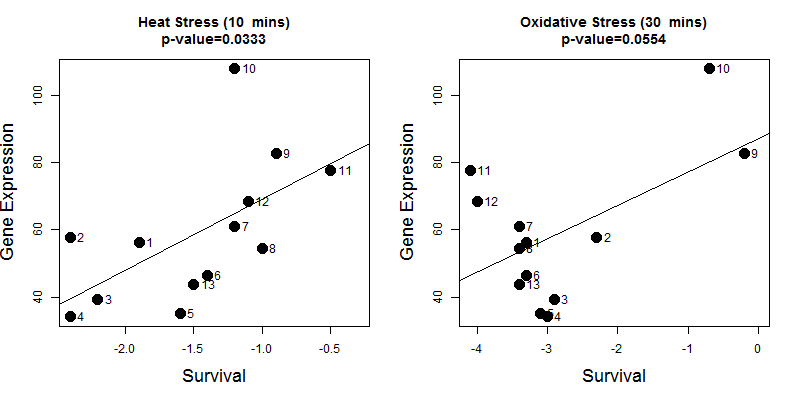

Supplement: S6 File — Expression levels of genes LACR_1383 –LACR_2610 and LACR_A01 –LACR_E8 plotted against survival after 10 minutes heat and 30 minutes oxidative stress. Survival is expressed as the difference of log CFU/ml after stress and before stress. Numbers indicate fermentations as presented in Table 1. P-values above the plots indicate significance of correlation (assessed by a linear model). (ZIP) [file pone.0167944.s011.zip › S6_File/LACR_1525_real_dat.png]

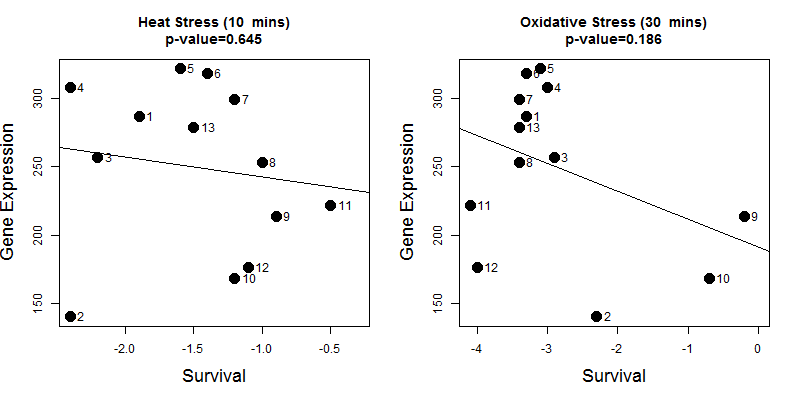

Supplement: S6 File — Expression levels of genes LACR_1383 –LACR_2610 and LACR_A01 –LACR_E8 plotted against survival after 10 minutes heat and 30 minutes oxidative stress. Survival is expressed as the difference of log CFU/ml after stress and before stress. Numbers indicate fermentations as presented in Table 1. P-values above the plots indicate significance of correlation (assessed by a linear model). (ZIP) [file pone.0167944.s011.zip › S6_File/LACR_1526_real_dat.png]

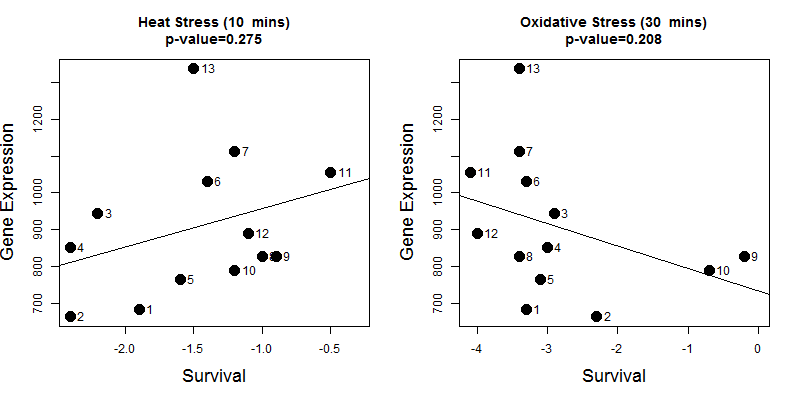

Supplement: S6 File — Expression levels of genes LACR_1383 –LACR_2610 and LACR_A01 –LACR_E8 plotted against survival after 10 minutes heat and 30 minutes oxidative stress. Survival is expressed as the difference of log CFU/ml after stress and before stress. Numbers indicate fermentations as presented in Table 1. P-values above the plots indicate significance of correlation (assessed by a linear model). (ZIP) [file pone.0167944.s011.zip › S6_File/LACR_1527_real_dat.png]

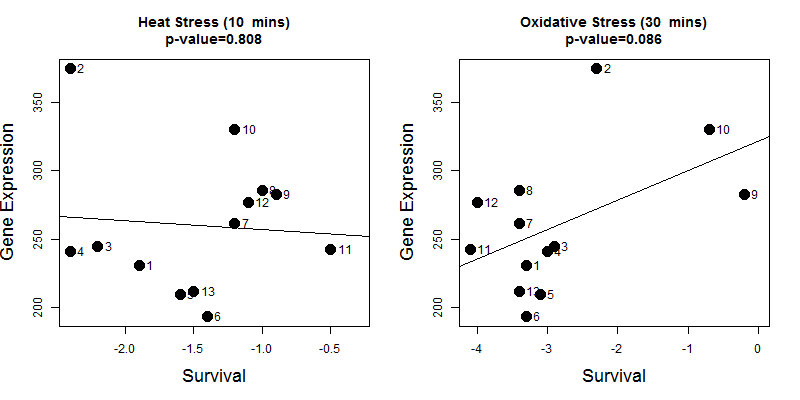

Supplement: S6 File — Expression levels of genes LACR_1383 –LACR_2610 and LACR_A01 –LACR_E8 plotted against survival after 10 minutes heat and 30 minutes oxidative stress. Survival is expressed as the difference of log CFU/ml after stress and before stress. Numbers indicate fermentations as presented in Table 1. P-values above the plots indicate significance of correlation (assessed by a linear model). (ZIP) [file pone.0167944.s011.zip › S6_File/LACR_1528_real_dat.png]

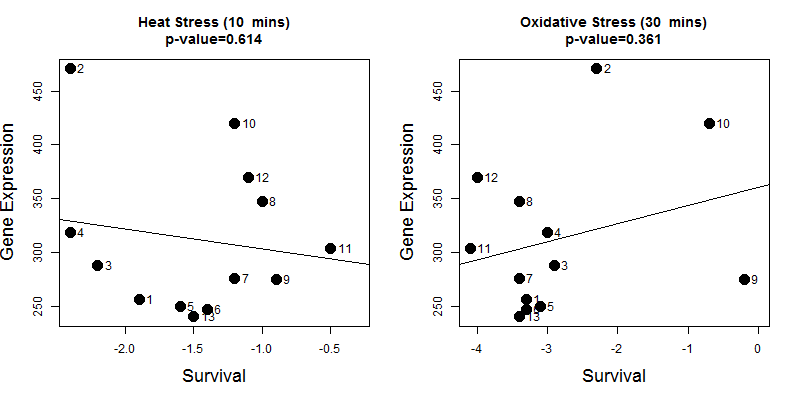

Supplement: S6 File — Expression levels of genes LACR_1383 –LACR_2610 and LACR_A01 –LACR_E8 plotted against survival after 10 minutes heat and 30 minutes oxidative stress. Survival is expressed as the difference of log CFU/ml after stress and before stress. Numbers indicate fermentations as presented in Table 1. P-values above the plots indicate significance of correlation (assessed by a linear model). (ZIP) [file pone.0167944.s011.zip › S6_File/LACR_1529_real_dat.png]

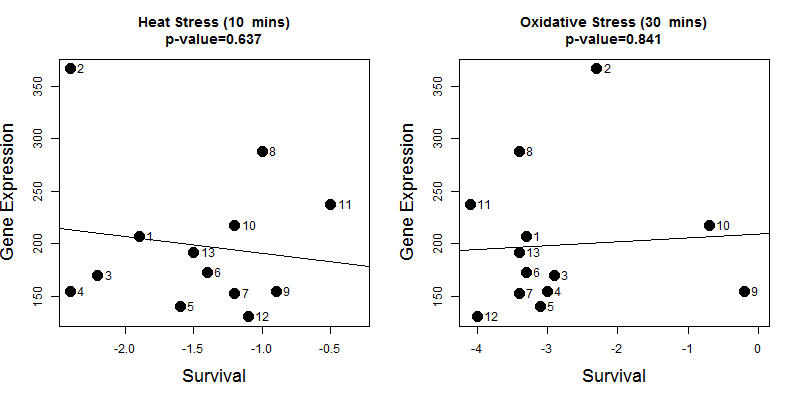

Supplement: S6 File — Expression levels of genes LACR_1383 –LACR_2610 and LACR_A01 –LACR_E8 plotted against survival after 10 minutes heat and 30 minutes oxidative stress. Survival is expressed as the difference of log CFU/ml after stress and before stress. Numbers indicate fermentations as presented in Table 1. P-values above the plots indicate significance of correlation (assessed by a linear model). (ZIP) [file pone.0167944.s011.zip › S6_File/LACR_1530_real_dat.png]

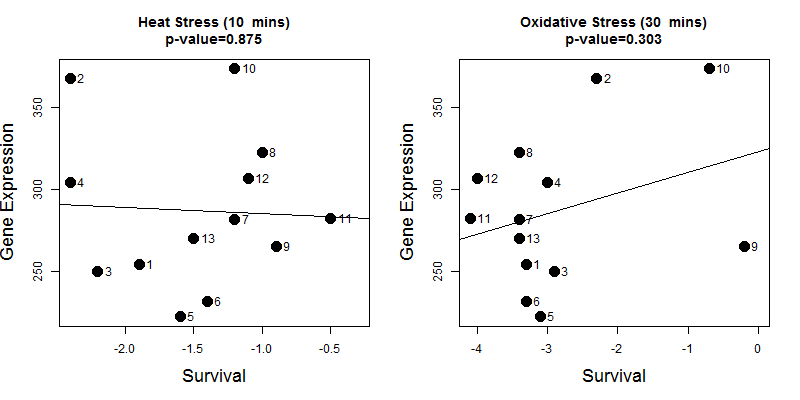

Supplement: S6 File — Expression levels of genes LACR_1383 –LACR_2610 and LACR_A01 –LACR_E8 plotted against survival after 10 minutes heat and 30 minutes oxidative stress. Survival is expressed as the difference of log CFU/ml after stress and before stress. Numbers indicate fermentations as presented in Table 1. P-values above the plots indicate significance of correlation (assessed by a linear model). (ZIP) [file pone.0167944.s011.zip › S6_File/LACR_1531_real_dat.png]

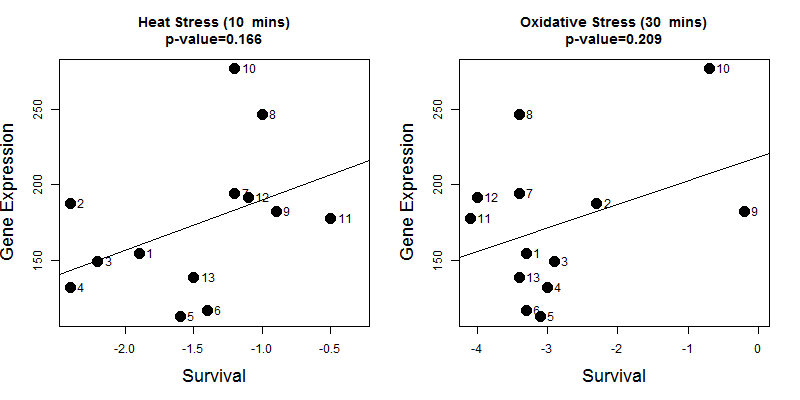

Supplement: S6 File — Expression levels of genes LACR_1383 –LACR_2610 and LACR_A01 –LACR_E8 plotted against survival after 10 minutes heat and 30 minutes oxidative stress. Survival is expressed as the difference of log CFU/ml after stress and before stress. Numbers indicate fermentations as presented in Table 1. P-values above the plots indicate significance of correlation (assessed by a linear model). (ZIP) [file pone.0167944.s011.zip › S6_File/LACR_1532_real_dat.png]

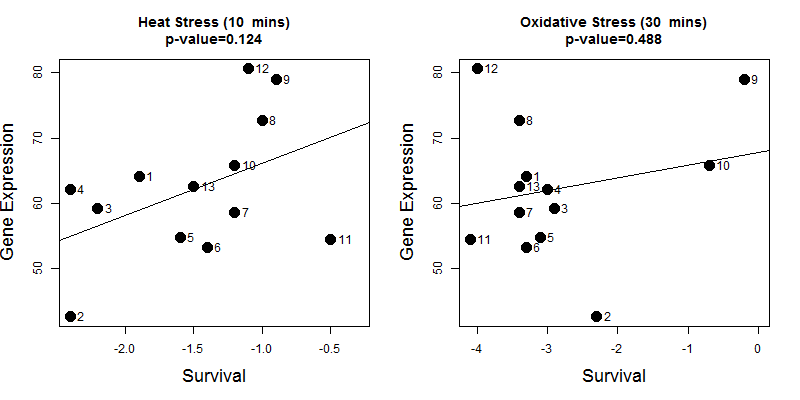

Supplement: S6 File — Expression levels of genes LACR_1383 –LACR_2610 and LACR_A01 –LACR_E8 plotted against survival after 10 minutes heat and 30 minutes oxidative stress. Survival is expressed as the difference of log CFU/ml after stress and before stress. Numbers indicate fermentations as presented in Table 1. P-values above the plots indicate significance of correlation (assessed by a linear model). (ZIP) [file pone.0167944.s011.zip › S6_File/LACR_1533_real_dat.png]

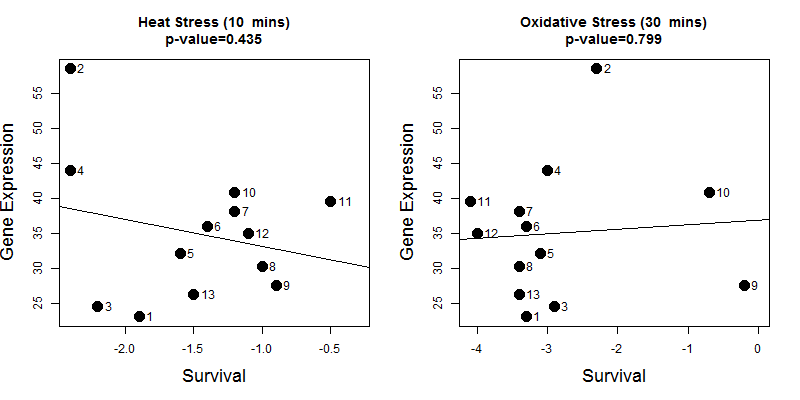

Supplement: S6 File — Expression levels of genes LACR_1383 –LACR_2610 and LACR_A01 –LACR_E8 plotted against survival after 10 minutes heat and 30 minutes oxidative stress. Survival is expressed as the difference of log CFU/ml after stress and before stress. Numbers indicate fermentations as presented in Table 1. P-values above the plots indicate significance of correlation (assessed by a linear model). (ZIP) [file pone.0167944.s011.zip › S6_File/LACR_1534_real_dat.png]

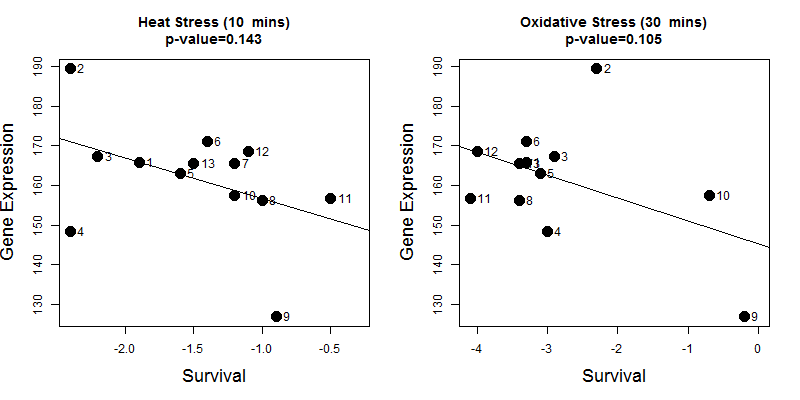

Supplement: S6 File — Expression levels of genes LACR_1383 –LACR_2610 and LACR_A01 –LACR_E8 plotted against survival after 10 minutes heat and 30 minutes oxidative stress. Survival is expressed as the difference of log CFU/ml after stress and before stress. Numbers indicate fermentations as presented in Table 1. P-values above the plots indicate significance of correlation (assessed by a linear model). (ZIP) [file pone.0167944.s011.zip › S6_File/LACR_1535_real_dat.png]

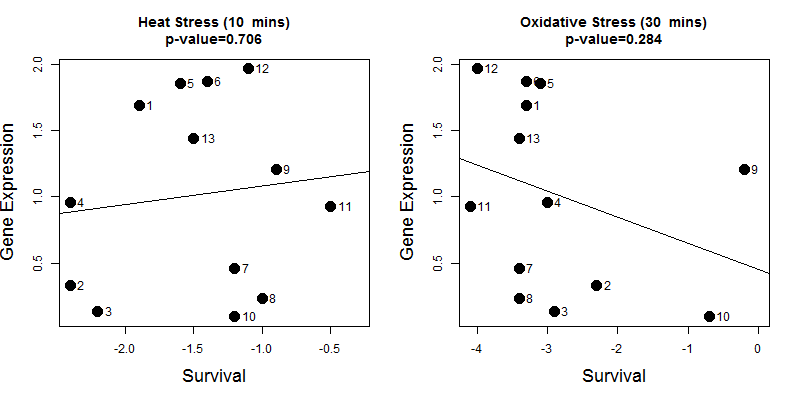

Supplement: S6 File — Expression levels of genes LACR_1383 –LACR_2610 and LACR_A01 –LACR_E8 plotted against survival after 10 minutes heat and 30 minutes oxidative stress. Survival is expressed as the difference of log CFU/ml after stress and before stress. Numbers indicate fermentations as presented in Table 1. P-values above the plots indicate significance of correlation (assessed by a linear model). (ZIP) [file pone.0167944.s011.zip › S6_File/LACR_1536_real_dat.png]

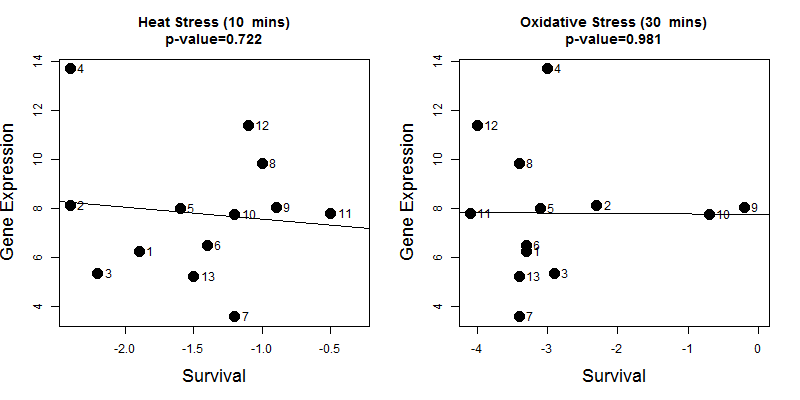

Supplement: S6 File — Expression levels of genes LACR_1383 –LACR_2610 and LACR_A01 –LACR_E8 plotted against survival after 10 minutes heat and 30 minutes oxidative stress. Survival is expressed as the difference of log CFU/ml after stress and before stress. Numbers indicate fermentations as presented in Table 1. P-values above the plots indicate significance of correlation (assessed by a linear model). (ZIP) [file pone.0167944.s011.zip › S6_File/LACR_1537_real_dat.png]

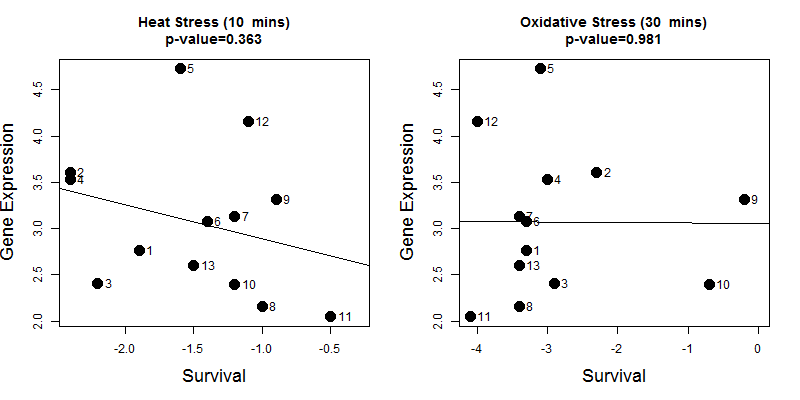

Supplement: S6 File — Expression levels of genes LACR_1383 –LACR_2610 and LACR_A01 –LACR_E8 plotted against survival after 10 minutes heat and 30 minutes oxidative stress. Survival is expressed as the difference of log CFU/ml after stress and before stress. Numbers indicate fermentations as presented in Table 1. P-values above the plots indicate significance of correlation (assessed by a linear model). (ZIP) [file pone.0167944.s011.zip › S6_File/LACR_1538_real_dat.png]

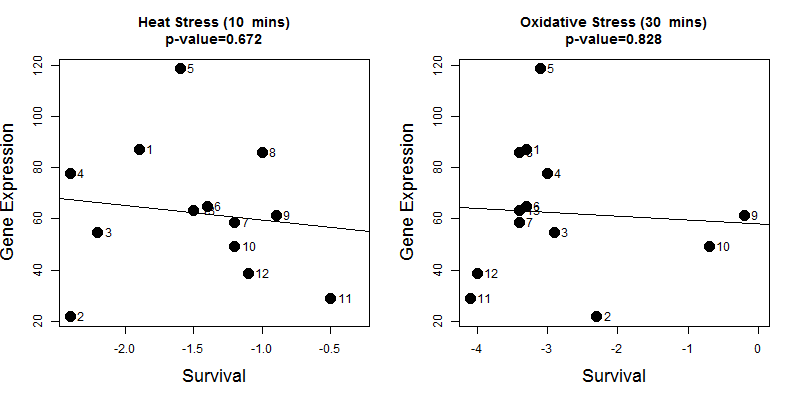

Supplement: S6 File — Expression levels of genes LACR_1383 –LACR_2610 and LACR_A01 –LACR_E8 plotted against survival after 10 minutes heat and 30 minutes oxidative stress. Survival is expressed as the difference of log CFU/ml after stress and before stress. Numbers indicate fermentations as presented in Table 1. P-values above the plots indicate significance of correlation (assessed by a linear model). (ZIP) [file pone.0167944.s011.zip › S6_File/LACR_1539_real_dat.png]

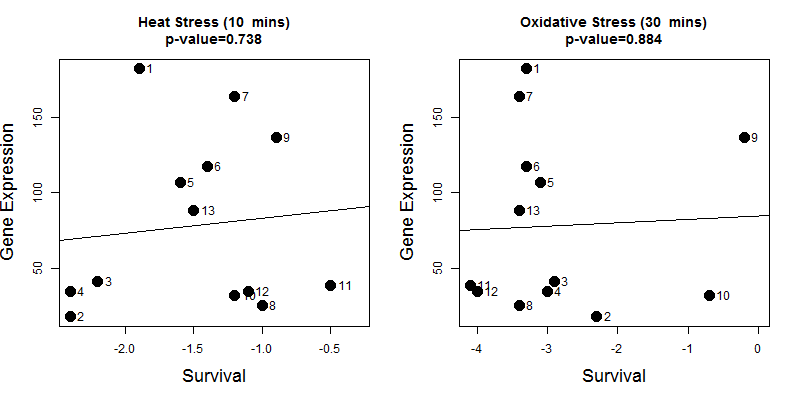

Supplement: S6 File — Expression levels of genes LACR_1383 –LACR_2610 and LACR_A01 –LACR_E8 plotted against survival after 10 minutes heat and 30 minutes oxidative stress. Survival is expressed as the difference of log CFU/ml after stress and before stress. Numbers indicate fermentations as presented in Table 1. P-values above the plots indicate significance of correlation (assessed by a linear model). (ZIP) [file pone.0167944.s011.zip › S6_File/LACR_1540_real_dat.png]

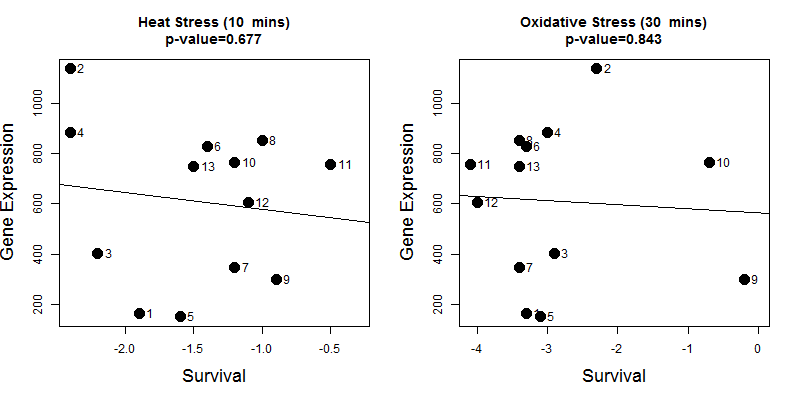

Supplement: S6 File — Expression levels of genes LACR_1383 –LACR_2610 and LACR_A01 –LACR_E8 plotted against survival after 10 minutes heat and 30 minutes oxidative stress. Survival is expressed as the difference of log CFU/ml after stress and before stress. Numbers indicate fermentations as presented in Table 1. P-values above the plots indicate significance of correlation (assessed by a linear model). (ZIP) [file pone.0167944.s011.zip › S6_File/LACR_1541_real_dat.png]

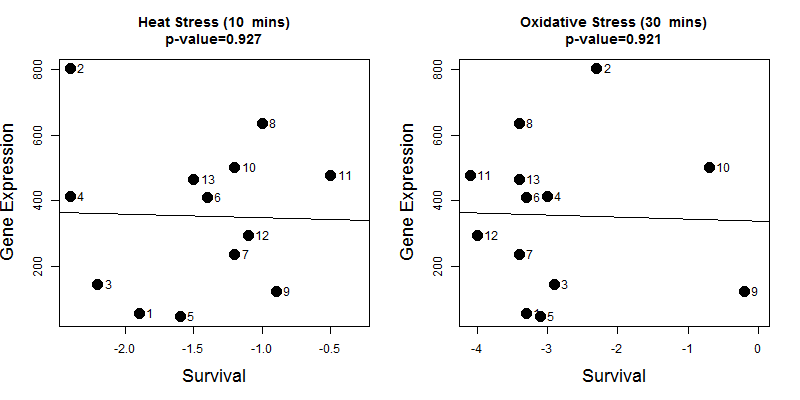

Supplement: S6 File — Expression levels of genes LACR_1383 –LACR_2610 and LACR_A01 –LACR_E8 plotted against survival after 10 minutes heat and 30 minutes oxidative stress. Survival is expressed as the difference of log CFU/ml after stress and before stress. Numbers indicate fermentations as presented in Table 1. P-values above the plots indicate significance of correlation (assessed by a linear model). (ZIP) [file pone.0167944.s011.zip › S6_File/LACR_1542_real_dat.png]

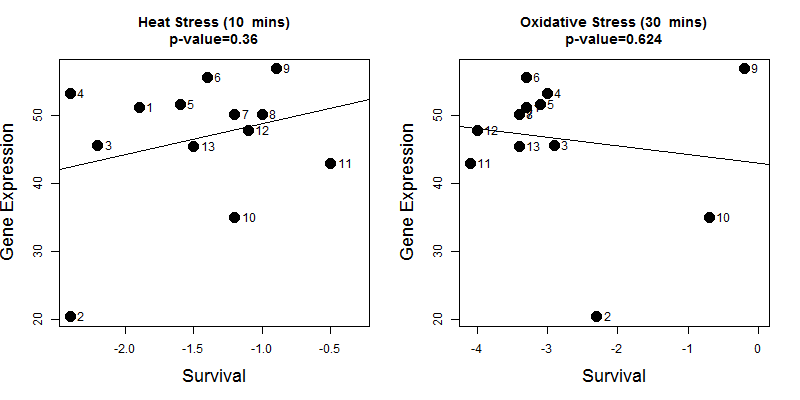

Supplement: S6 File — Expression levels of genes LACR_1383 –LACR_2610 and LACR_A01 –LACR_E8 plotted against survival after 10 minutes heat and 30 minutes oxidative stress. Survival is expressed as the difference of log CFU/ml after stress and before stress. Numbers indicate fermentations as presented in Table 1. P-values above the plots indicate significance of correlation (assessed by a linear model). (ZIP) [file pone.0167944.s011.zip › S6_File/LACR_1543_real_dat.png]

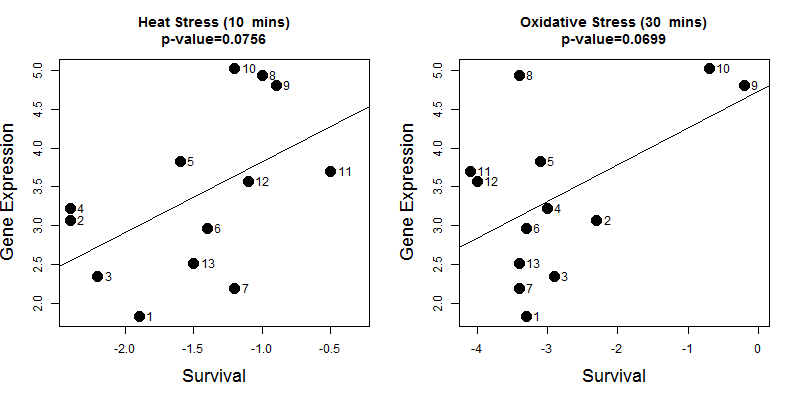

Supplement: S6 File — Expression levels of genes LACR_1383 –LACR_2610 and LACR_A01 –LACR_E8 plotted against survival after 10 minutes heat and 30 minutes oxidative stress. Survival is expressed as the difference of log CFU/ml after stress and before stress. Numbers indicate fermentations as presented in Table 1. P-values above the plots indicate significance of correlation (assessed by a linear model). (ZIP) [file pone.0167944.s011.zip › S6_File/LACR_1544_real_dat.png]

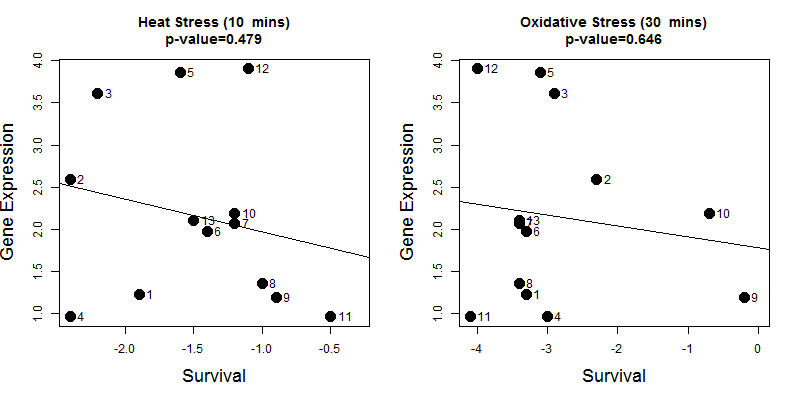

Supplement: S6 File — Expression levels of genes LACR_1383 –LACR_2610 and LACR_A01 –LACR_E8 plotted against survival after 10 minutes heat and 30 minutes oxidative stress. Survival is expressed as the difference of log CFU/ml after stress and before stress. Numbers indicate fermentations as presented in Table 1. P-values above the plots indicate significance of correlation (assessed by a linear model). (ZIP) [file pone.0167944.s011.zip › S6_File/LACR_1546_real_dat.png]

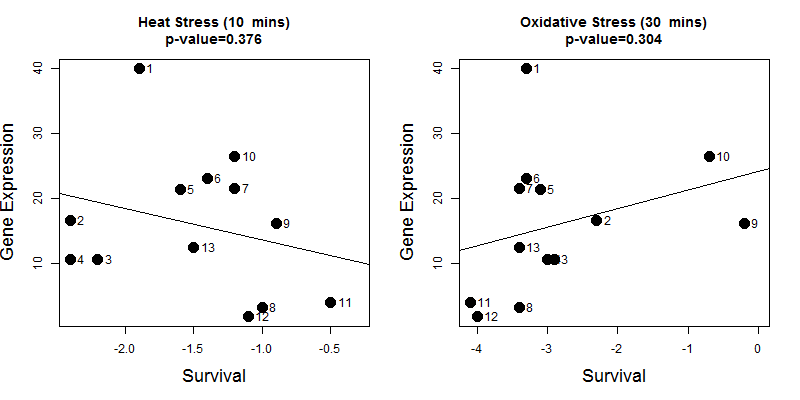

Supplement: S6 File — Expression levels of genes LACR_1383 –LACR_2610 and LACR_A01 –LACR_E8 plotted against survival after 10 minutes heat and 30 minutes oxidative stress. Survival is expressed as the difference of log CFU/ml after stress and before stress. Numbers indicate fermentations as presented in Table 1. P-values above the plots indicate significance of correlation (assessed by a linear model). (ZIP) [file pone.0167944.s011.zip › S6_File/LACR_1547_real_dat.png]

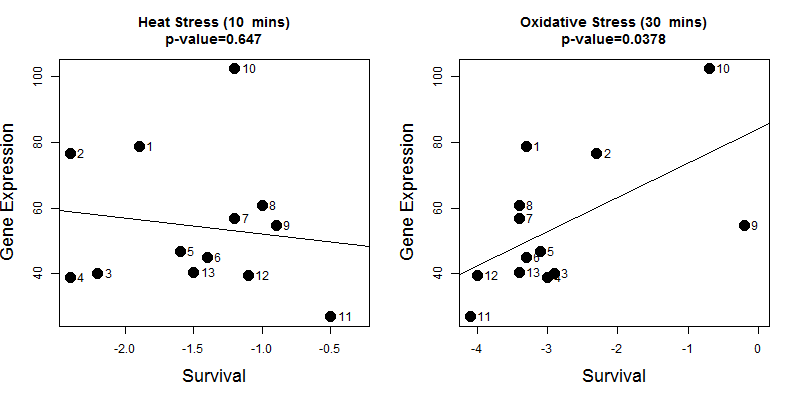

Supplement: S6 File — Expression levels of genes LACR_1383 –LACR_2610 and LACR_A01 –LACR_E8 plotted against survival after 10 minutes heat and 30 minutes oxidative stress. Survival is expressed as the difference of log CFU/ml after stress and before stress. Numbers indicate fermentations as presented in Table 1. P-values above the plots indicate significance of correlation (assessed by a linear model). (ZIP) [file pone.0167944.s011.zip › S6_File/LACR_1548_real_dat.png]

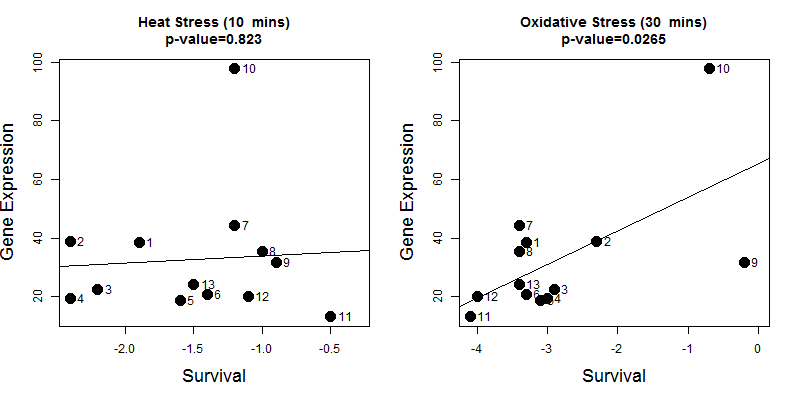

Supplement: S6 File — Expression levels of genes LACR_1383 –LACR_2610 and LACR_A01 –LACR_E8 plotted against survival after 10 minutes heat and 30 minutes oxidative stress. Survival is expressed as the difference of log CFU/ml after stress and before stress. Numbers indicate fermentations as presented in Table 1. P-values above the plots indicate significance of correlation (assessed by a linear model). (ZIP) [file pone.0167944.s011.zip › S6_File/LACR_1549_real_dat.png]

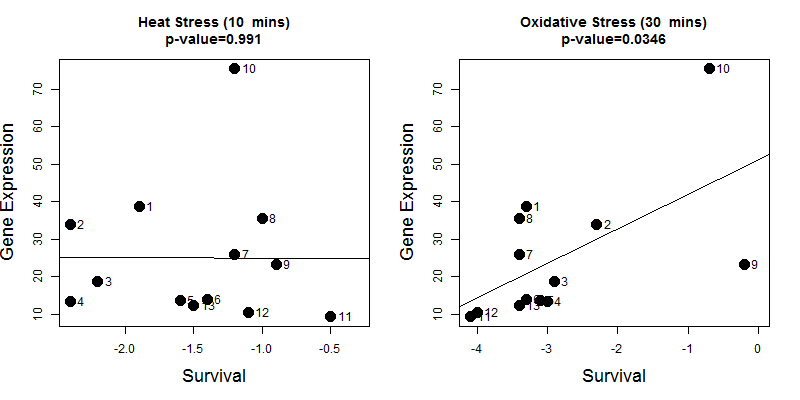

Supplement: S6 File — Expression levels of genes LACR_1383 –LACR_2610 and LACR_A01 –LACR_E8 plotted against survival after 10 minutes heat and 30 minutes oxidative stress. Survival is expressed as the difference of log CFU/ml after stress and before stress. Numbers indicate fermentations as presented in Table 1. P-values above the plots indicate significance of correlation (assessed by a linear model). (ZIP) [file pone.0167944.s011.zip › S6_File/LACR_1550_real_dat.png]

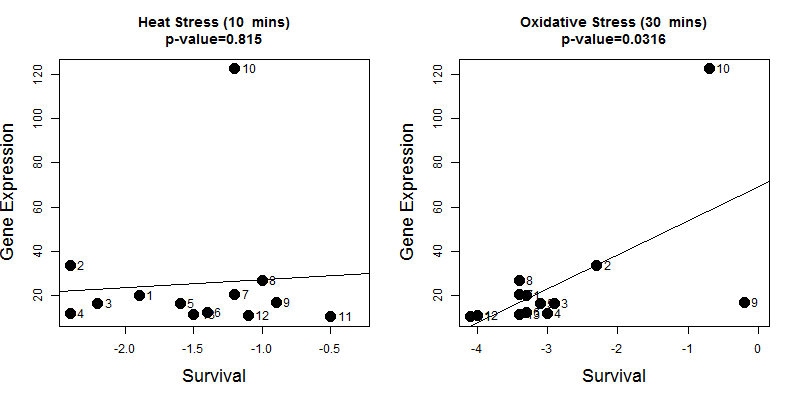

Supplement: S6 File — Expression levels of genes LACR_1383 –LACR_2610 and LACR_A01 –LACR_E8 plotted against survival after 10 minutes heat and 30 minutes oxidative stress. Survival is expressed as the difference of log CFU/ml after stress and before stress. Numbers indicate fermentations as presented in Table 1. P-values above the plots indicate significance of correlation (assessed by a linear model). (ZIP) [file pone.0167944.s011.zip › S6_File/LACR_1551_real_dat.png]

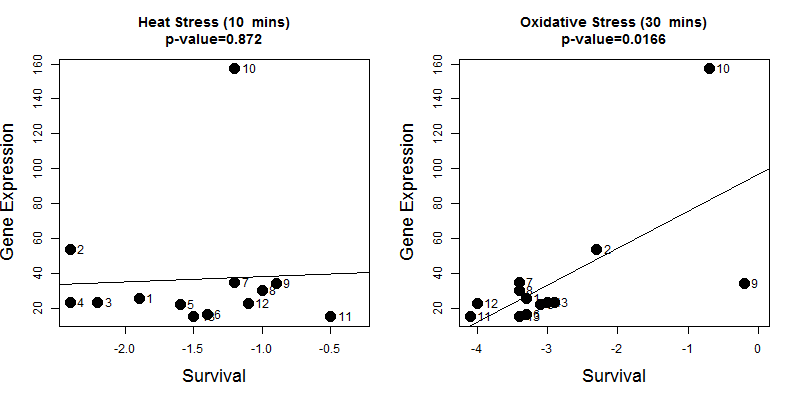

Supplement: S6 File — Expression levels of genes LACR_1383 –LACR_2610 and LACR_A01 –LACR_E8 plotted against survival after 10 minutes heat and 30 minutes oxidative stress. Survival is expressed as the difference of log CFU/ml after stress and before stress. Numbers indicate fermentations as presented in Table 1. P-values above the plots indicate significance of correlation (assessed by a linear model). (ZIP) [file pone.0167944.s011.zip › S6_File/LACR_1552_real_dat.png]

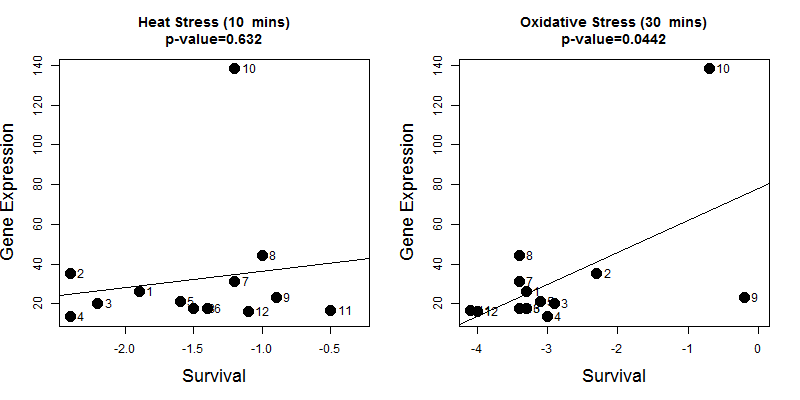

Supplement: S6 File — Expression levels of genes LACR_1383 –LACR_2610 and LACR_A01 –LACR_E8 plotted against survival after 10 minutes heat and 30 minutes oxidative stress. Survival is expressed as the difference of log CFU/ml after stress and before stress. Numbers indicate fermentations as presented in Table 1. P-values above the plots indicate significance of correlation (assessed by a linear model). (ZIP) [file pone.0167944.s011.zip › S6_File/LACR_1553_real_dat.png]

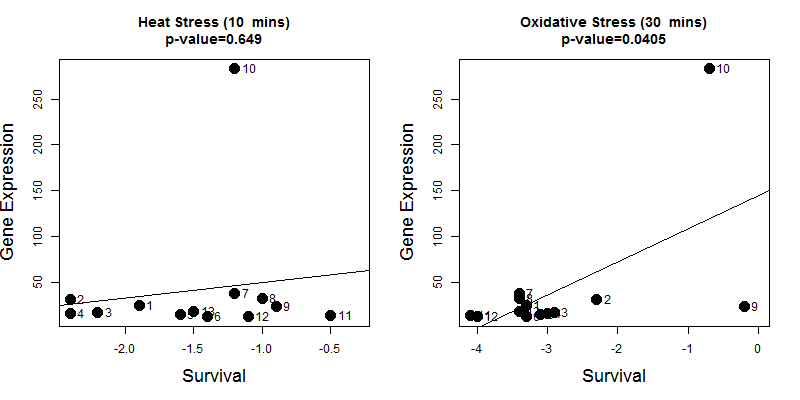

Supplement: S6 File — Expression levels of genes LACR_1383 –LACR_2610 and LACR_A01 –LACR_E8 plotted against survival after 10 minutes heat and 30 minutes oxidative stress. Survival is expressed as the difference of log CFU/ml after stress and before stress. Numbers indicate fermentations as presented in Table 1. P-values above the plots indicate significance of correlation (assessed by a linear model). (ZIP) [file pone.0167944.s011.zip › S6_File/LACR_1554_real_dat.png]

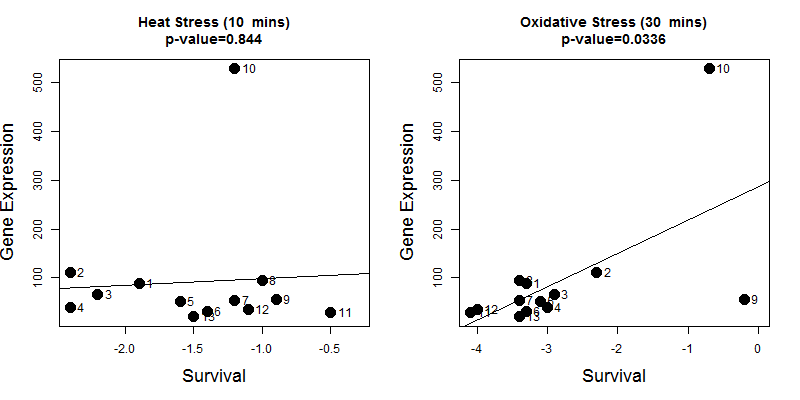

Supplement: S6 File — Expression levels of genes LACR_1383 –LACR_2610 and LACR_A01 –LACR_E8 plotted against survival after 10 minutes heat and 30 minutes oxidative stress. Survival is expressed as the difference of log CFU/ml after stress and before stress. Numbers indicate fermentations as presented in Table 1. P-values above the plots indicate significance of correlation (assessed by a linear model). (ZIP) [file pone.0167944.s011.zip › S6_File/LACR_1555_real_dat.png]

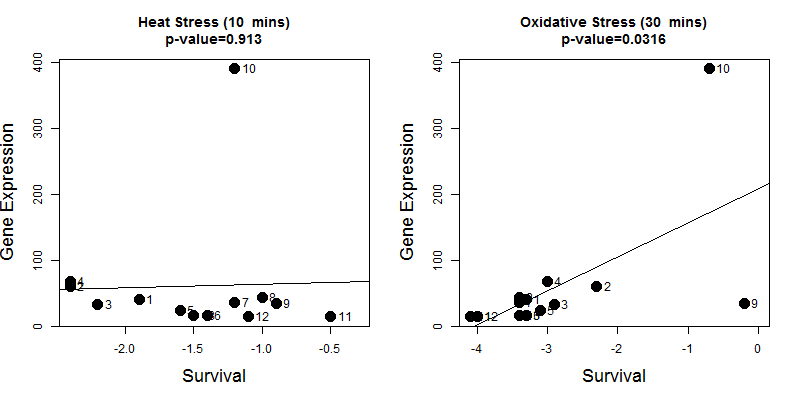

Supplement: S6 File — Expression levels of genes LACR_1383 –LACR_2610 and LACR_A01 –LACR_E8 plotted against survival after 10 minutes heat and 30 minutes oxidative stress. Survival is expressed as the difference of log CFU/ml after stress and before stress. Numbers indicate fermentations as presented in Table 1. P-values above the plots indicate significance of correlation (assessed by a linear model). (ZIP) [file pone.0167944.s011.zip › S6_File/LACR_1556_real_dat.png]

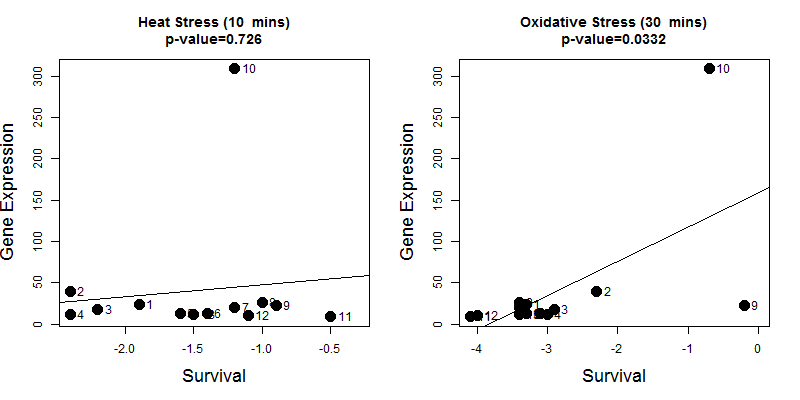

Supplement: S6 File — Expression levels of genes LACR_1383 –LACR_2610 and LACR_A01 –LACR_E8 plotted against survival after 10 minutes heat and 30 minutes oxidative stress. Survival is expressed as the difference of log CFU/ml after stress and before stress. Numbers indicate fermentations as presented in Table 1. P-values above the plots indicate significance of correlation (assessed by a linear model). (ZIP) [file pone.0167944.s011.zip › S6_File/LACR_1557_real_dat.png]

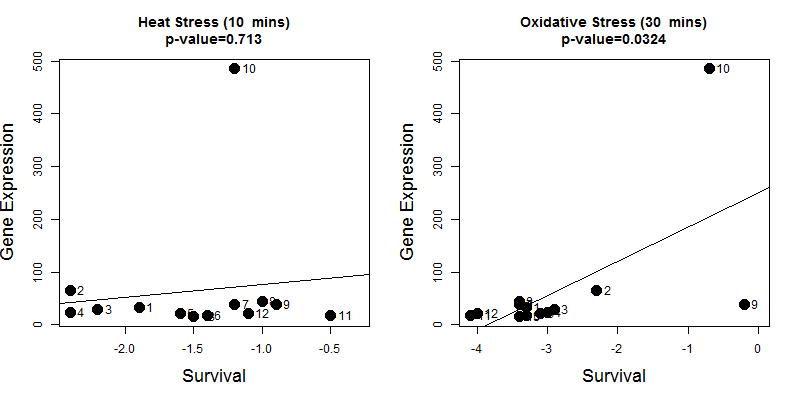

Supplement: S6 File — Expression levels of genes LACR_1383 –LACR_2610 and LACR_A01 –LACR_E8 plotted against survival after 10 minutes heat and 30 minutes oxidative stress. Survival is expressed as the difference of log CFU/ml after stress and before stress. Numbers indicate fermentations as presented in Table 1. P-values above the plots indicate significance of correlation (assessed by a linear model). (ZIP) [file pone.0167944.s011.zip › S6_File/LACR_1558_real_dat.png]

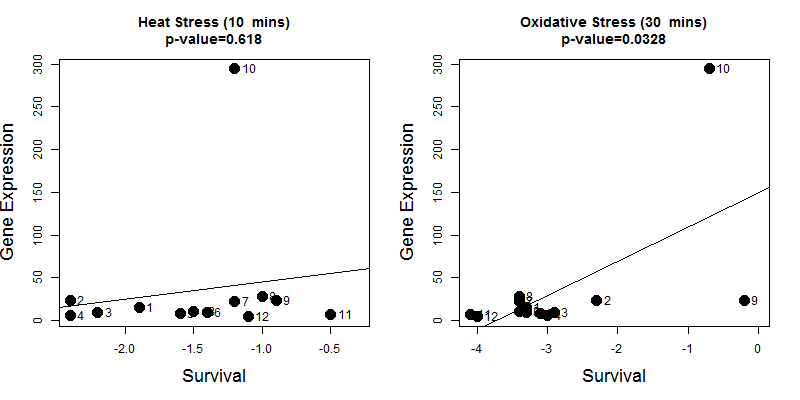

Supplement: S6 File — Expression levels of genes LACR_1383 –LACR_2610 and LACR_A01 –LACR_E8 plotted against survival after 10 minutes heat and 30 minutes oxidative stress. Survival is expressed as the difference of log CFU/ml after stress and before stress. Numbers indicate fermentations as presented in Table 1. P-values above the plots indicate significance of correlation (assessed by a linear model). (ZIP) [file pone.0167944.s011.zip › S6_File/LACR_1559_real_dat.png]

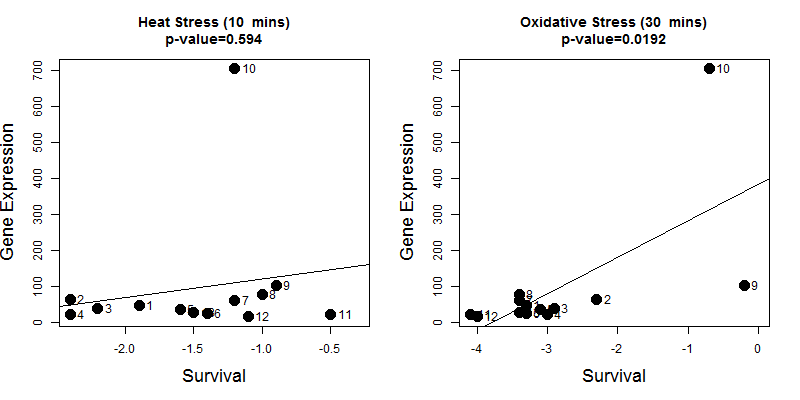

Supplement: S6 File — Expression levels of genes LACR_1383 –LACR_2610 and LACR_A01 –LACR_E8 plotted against survival after 10 minutes heat and 30 minutes oxidative stress. Survival is expressed as the difference of log CFU/ml after stress and before stress. Numbers indicate fermentations as presented in Table 1. P-values above the plots indicate significance of correlation (assessed by a linear model). (ZIP) [file pone.0167944.s011.zip › S6_File/LACR_1560_real_dat.png]

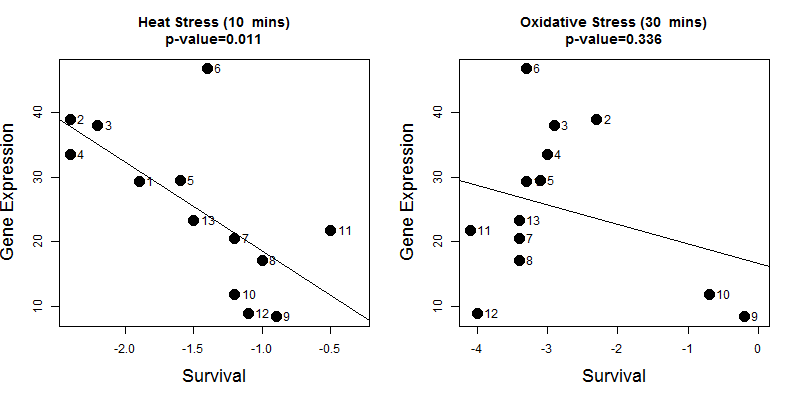

Supplement: S6 File — Expression levels of genes LACR_1383 –LACR_2610 and LACR_A01 –LACR_E8 plotted against survival after 10 minutes heat and 30 minutes oxidative stress. Survival is expressed as the difference of log CFU/ml after stress and before stress. Numbers indicate fermentations as presented in Table 1. P-values above the plots indicate significance of correlation (assessed by a linear model). (ZIP) [file pone.0167944.s011.zip › S6_File/LACR_1561_real_dat.png]

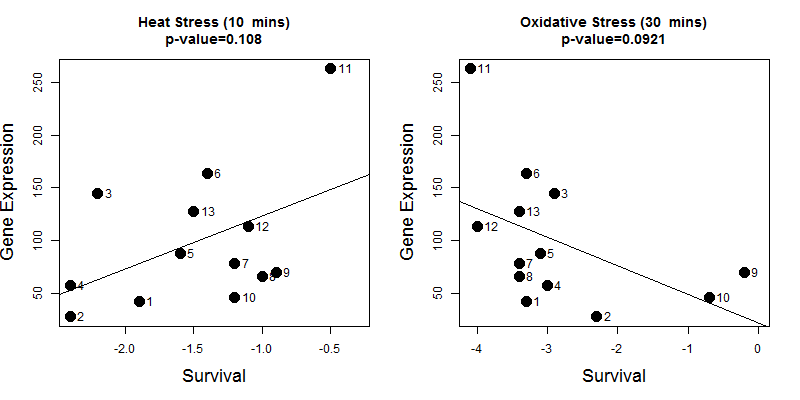

Supplement: S6 File — Expression levels of genes LACR_1383 –LACR_2610 and LACR_A01 –LACR_E8 plotted against survival after 10 minutes heat and 30 minutes oxidative stress. Survival is expressed as the difference of log CFU/ml after stress and before stress. Numbers indicate fermentations as presented in Table 1. P-values above the plots indicate significance of correlation (assessed by a linear model). (ZIP) [file pone.0167944.s011.zip › S6_File/LACR_1565_real_dat.png]

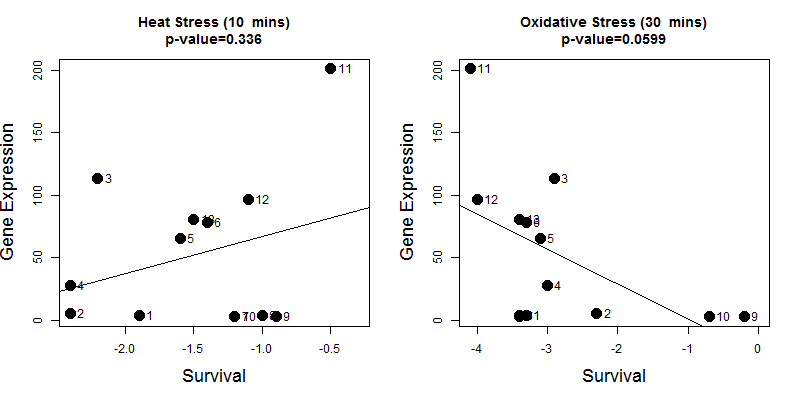

Supplement: S6 File — Expression levels of genes LACR_1383 –LACR_2610 and LACR_A01 –LACR_E8 plotted against survival after 10 minutes heat and 30 minutes oxidative stress. Survival is expressed as the difference of log CFU/ml after stress and before stress. Numbers indicate fermentations as presented in Table 1. P-values above the plots indicate significance of correlation (assessed by a linear model). (ZIP) [file pone.0167944.s011.zip › S6_File/LACR_1566_real_dat.png]

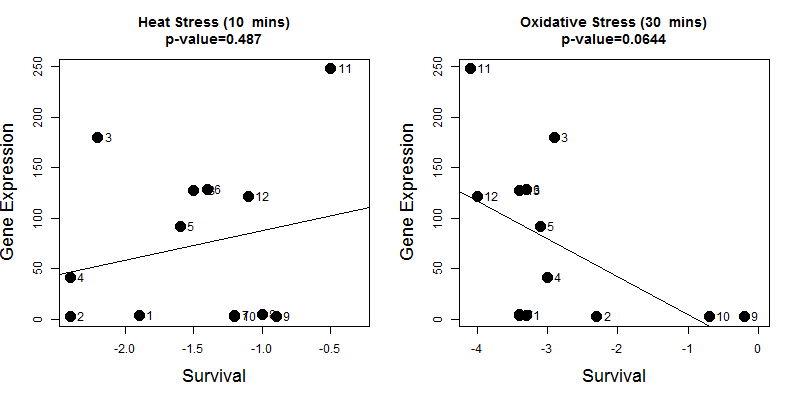

Supplement: S6 File — Expression levels of genes LACR_1383 –LACR_2610 and LACR_A01 –LACR_E8 plotted against survival after 10 minutes heat and 30 minutes oxidative stress. Survival is expressed as the difference of log CFU/ml after stress and before stress. Numbers indicate fermentations as presented in Table 1. P-values above the plots indicate significance of correlation (assessed by a linear model). (ZIP) [file pone.0167944.s011.zip › S6_File/LACR_1567_real_dat.png]

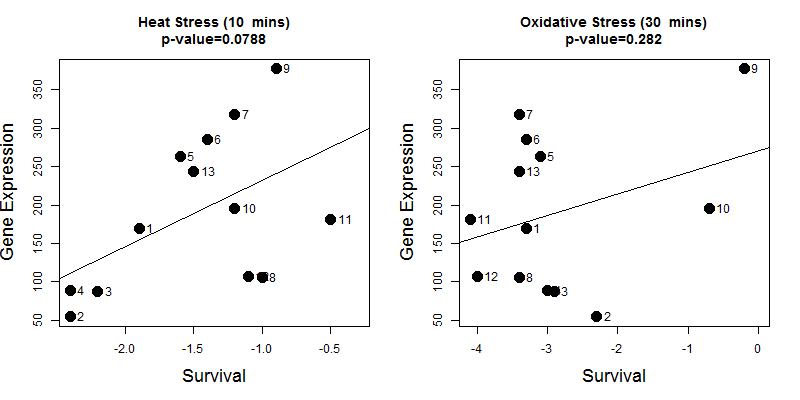

Supplement: S6 File — Expression levels of genes LACR_1383 –LACR_2610 and LACR_A01 –LACR_E8 plotted against survival after 10 minutes heat and 30 minutes oxidative stress. Survival is expressed as the difference of log CFU/ml after stress and before stress. Numbers indicate fermentations as presented in Table 1. P-values above the plots indicate significance of correlation (assessed by a linear model). (ZIP) [file pone.0167944.s011.zip › S6_File/LACR_1568_real_dat.png]

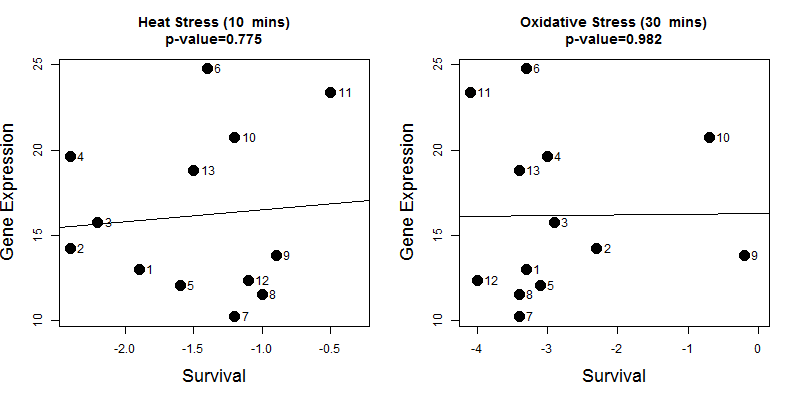

Supplement: S6 File — Expression levels of genes LACR_1383 –LACR_2610 and LACR_A01 –LACR_E8 plotted against survival after 10 minutes heat and 30 minutes oxidative stress. Survival is expressed as the difference of log CFU/ml after stress and before stress. Numbers indicate fermentations as presented in Table 1. P-values above the plots indicate significance of correlation (assessed by a linear model). (ZIP) [file pone.0167944.s011.zip › S6_File/LACR_1569_real_dat.png]

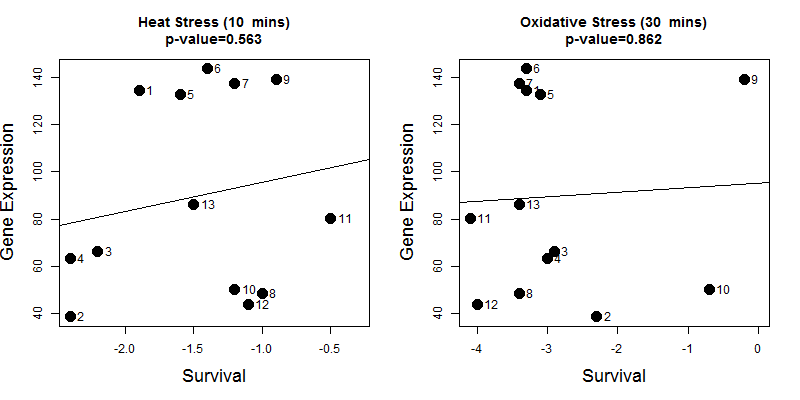

Supplement: S6 File — Expression levels of genes LACR_1383 –LACR_2610 and LACR_A01 –LACR_E8 plotted against survival after 10 minutes heat and 30 minutes oxidative stress. Survival is expressed as the difference of log CFU/ml after stress and before stress. Numbers indicate fermentations as presented in Table 1. P-values above the plots indicate significance of correlation (assessed by a linear model). (ZIP) [file pone.0167944.s011.zip › S6_File/LACR_1570_real_dat.png]

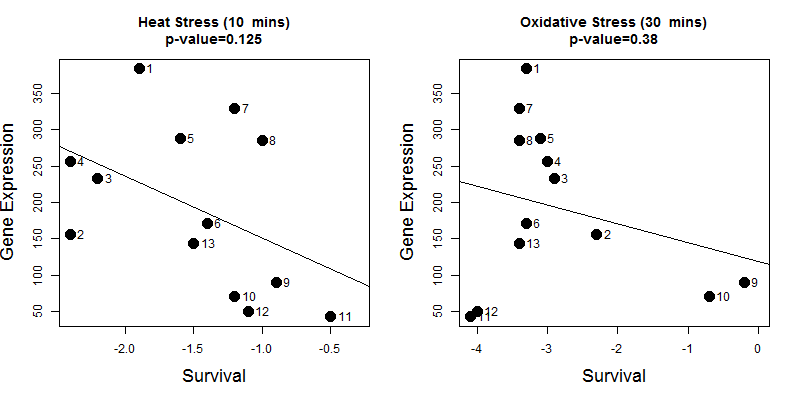

Supplement: S6 File — Expression levels of genes LACR_1383 –LACR_2610 and LACR_A01 –LACR_E8 plotted against survival after 10 minutes heat and 30 minutes oxidative stress. Survival is expressed as the difference of log CFU/ml after stress and before stress. Numbers indicate fermentations as presented in Table 1. P-values above the plots indicate significance of correlation (assessed by a linear model). (ZIP) [file pone.0167944.s011.zip › S6_File/LACR_1571_real_dat.png]

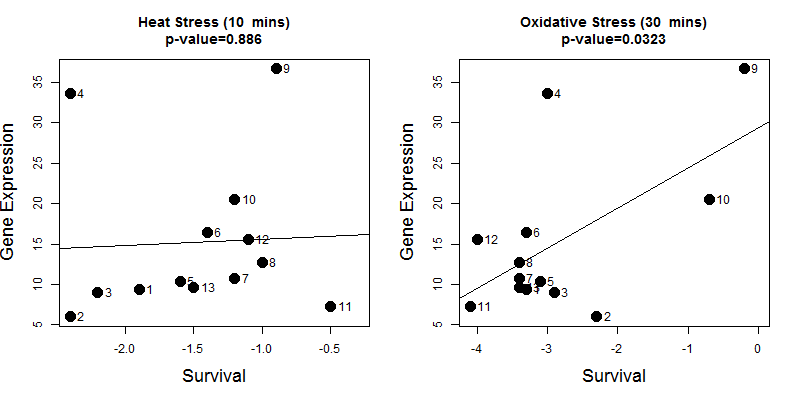

Supplement: S6 File — Expression levels of genes LACR_1383 –LACR_2610 and LACR_A01 –LACR_E8 plotted against survival after 10 minutes heat and 30 minutes oxidative stress. Survival is expressed as the difference of log CFU/ml after stress and before stress. Numbers indicate fermentations as presented in Table 1. P-values above the plots indicate significance of correlation (assessed by a linear model). (ZIP) [file pone.0167944.s011.zip › S6_File/LACR_1572_real_dat.png]

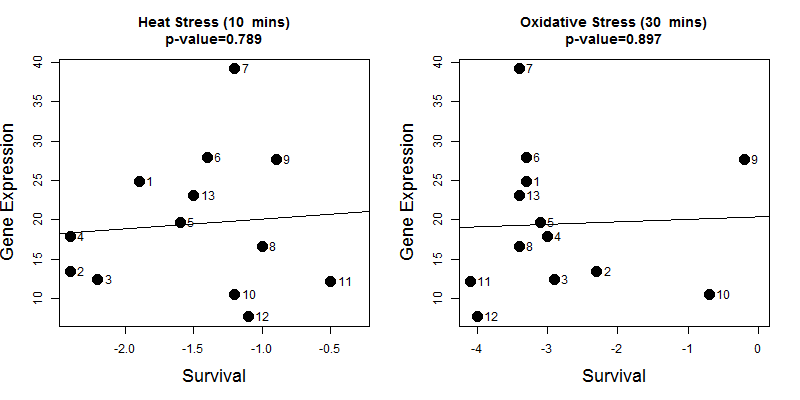

Supplement: S6 File — Expression levels of genes LACR_1383 –LACR_2610 and LACR_A01 –LACR_E8 plotted against survival after 10 minutes heat and 30 minutes oxidative stress. Survival is expressed as the difference of log CFU/ml after stress and before stress. Numbers indicate fermentations as presented in Table 1. P-values above the plots indicate significance of correlation (assessed by a linear model). (ZIP) [file pone.0167944.s011.zip › S6_File/LACR_1573_real_dat.png]

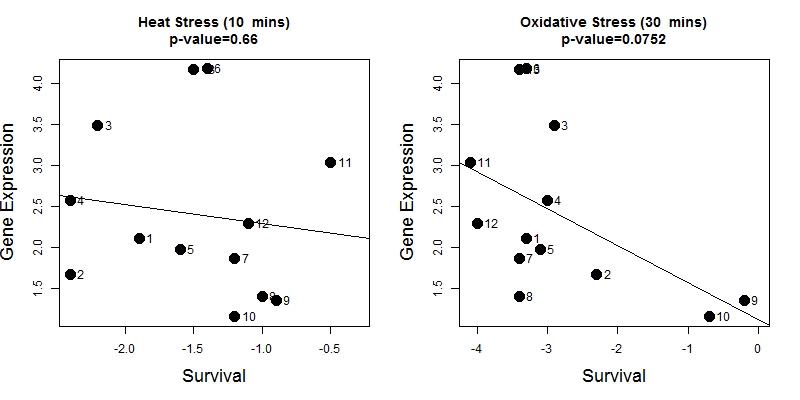

Supplement: S6 File — Expression levels of genes LACR_1383 –LACR_2610 and LACR_A01 –LACR_E8 plotted against survival after 10 minutes heat and 30 minutes oxidative stress. Survival is expressed as the difference of log CFU/ml after stress and before stress. Numbers indicate fermentations as presented in Table 1. P-values above the plots indicate significance of correlation (assessed by a linear model). (ZIP) [file pone.0167944.s011.zip › S6_File/LACR_1574_real_dat.png]

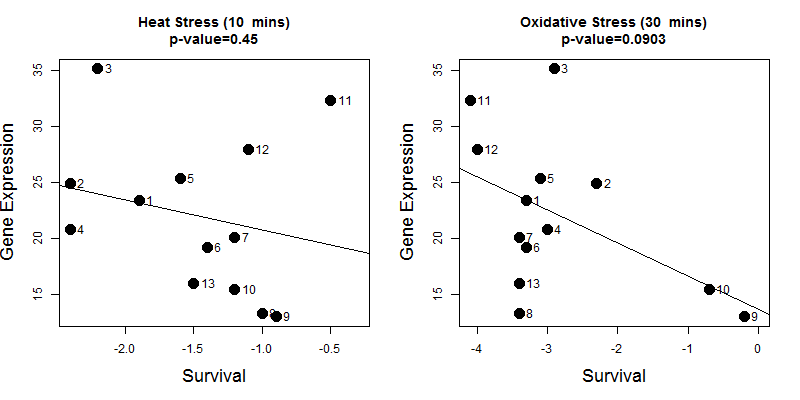

Supplement: S6 File — Expression levels of genes LACR_1383 –LACR_2610 and LACR_A01 –LACR_E8 plotted against survival after 10 minutes heat and 30 minutes oxidative stress. Survival is expressed as the difference of log CFU/ml after stress and before stress. Numbers indicate fermentations as presented in Table 1. P-values above the plots indicate significance of correlation (assessed by a linear model). (ZIP) [file pone.0167944.s011.zip › S6_File/LACR_1575_real_dat.png]

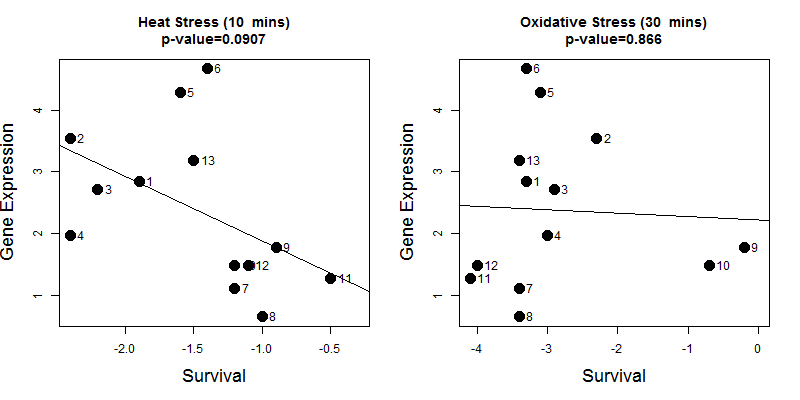

Supplement: S6 File — Expression levels of genes LACR_1383 –LACR_2610 and LACR_A01 –LACR_E8 plotted against survival after 10 minutes heat and 30 minutes oxidative stress. Survival is expressed as the difference of log CFU/ml after stress and before stress. Numbers indicate fermentations as presented in Table 1. P-values above the plots indicate significance of correlation (assessed by a linear model). (ZIP) [file pone.0167944.s011.zip › S6_File/LACR_1576_real_dat.png]

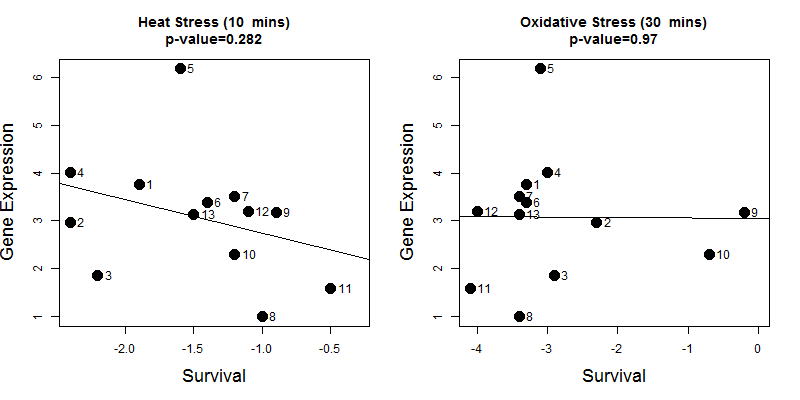

Supplement: S6 File — Expression levels of genes LACR_1383 –LACR_2610 and LACR_A01 –LACR_E8 plotted against survival after 10 minutes heat and 30 minutes oxidative stress. Survival is expressed as the difference of log CFU/ml after stress and before stress. Numbers indicate fermentations as presented in Table 1. P-values above the plots indicate significance of correlation (assessed by a linear model). (ZIP) [file pone.0167944.s011.zip › S6_File/LACR_1577_real_dat.png]

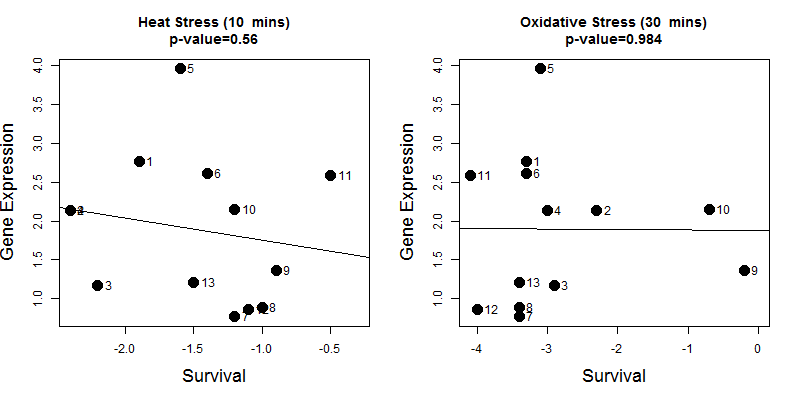

Supplement: S6 File — Expression levels of genes LACR_1383 –LACR_2610 and LACR_A01 –LACR_E8 plotted against survival after 10 minutes heat and 30 minutes oxidative stress. Survival is expressed as the difference of log CFU/ml after stress and before stress. Numbers indicate fermentations as presented in Table 1. P-values above the plots indicate significance of correlation (assessed by a linear model). (ZIP) [file pone.0167944.s011.zip › S6_File/LACR_1578_real_dat.png]

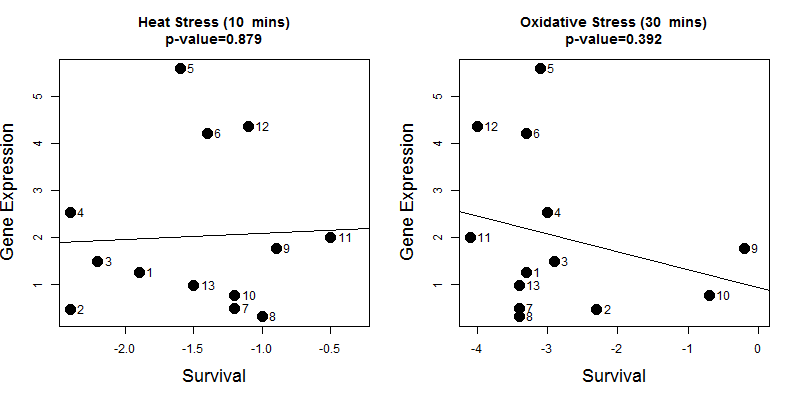

Supplement: S6 File — Expression levels of genes LACR_1383 –LACR_2610 and LACR_A01 –LACR_E8 plotted against survival after 10 minutes heat and 30 minutes oxidative stress. Survival is expressed as the difference of log CFU/ml after stress and before stress. Numbers indicate fermentations as presented in Table 1. P-values above the plots indicate significance of correlation (assessed by a linear model). (ZIP) [file pone.0167944.s011.zip › S6_File/LACR_1579_real_dat.png]

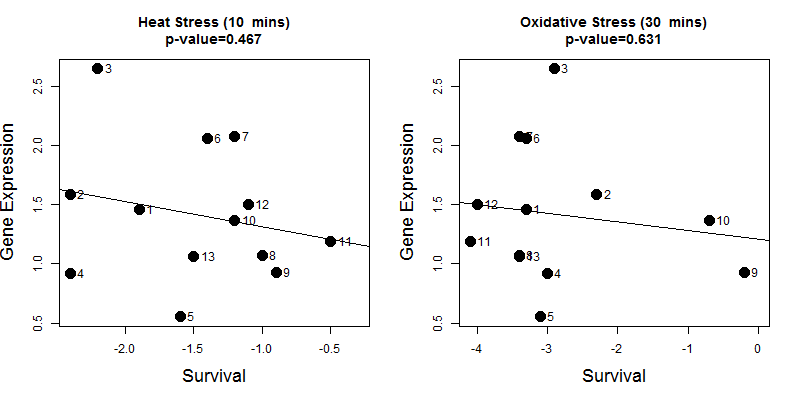

Supplement: S6 File — Expression levels of genes LACR_1383 –LACR_2610 and LACR_A01 –LACR_E8 plotted against survival after 10 minutes heat and 30 minutes oxidative stress. Survival is expressed as the difference of log CFU/ml after stress and before stress. Numbers indicate fermentations as presented in Table 1. P-values above the plots indicate significance of correlation (assessed by a linear model). (ZIP) [file pone.0167944.s011.zip › S6_File/LACR_1580_real_dat.png]

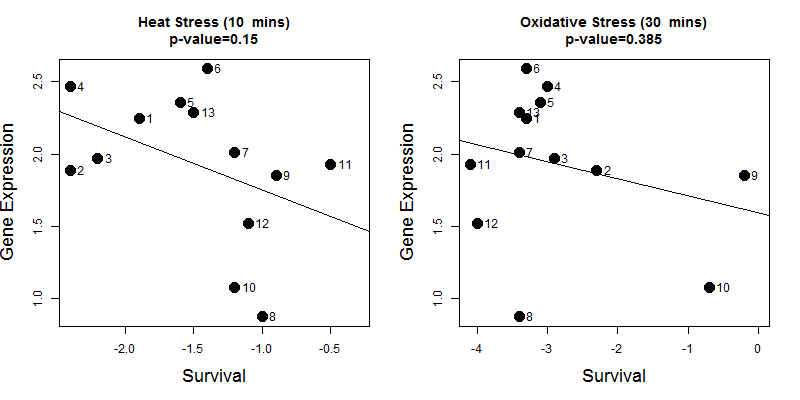

Supplement: S6 File — Expression levels of genes LACR_1383 –LACR_2610 and LACR_A01 –LACR_E8 plotted against survival after 10 minutes heat and 30 minutes oxidative stress. Survival is expressed as the difference of log CFU/ml after stress and before stress. Numbers indicate fermentations as presented in Table 1. P-values above the plots indicate significance of correlation (assessed by a linear model). (ZIP) [file pone.0167944.s011.zip › S6_File/LACR_1581_real_dat.png]

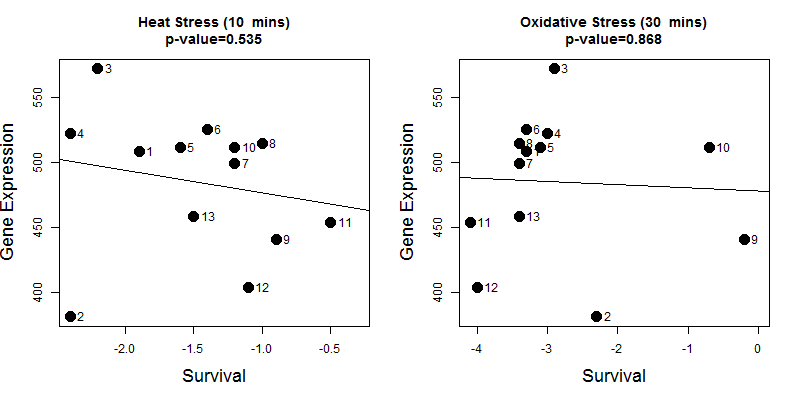

Supplement: S6 File — Expression levels of genes LACR_1383 –LACR_2610 and LACR_A01 –LACR_E8 plotted against survival after 10 minutes heat and 30 minutes oxidative stress. Survival is expressed as the difference of log CFU/ml after stress and before stress. Numbers indicate fermentations as presented in Table 1. P-values above the plots indicate significance of correlation (assessed by a linear model). (ZIP) [file pone.0167944.s011.zip › S6_File/LACR_1583_real_dat.png]

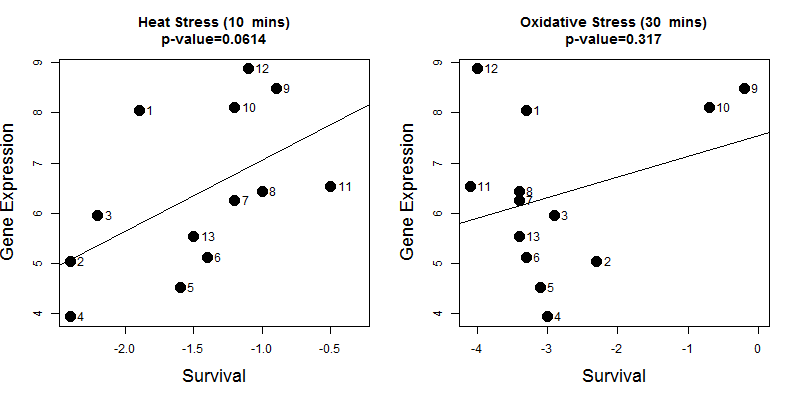

Supplement: S6 File — Expression levels of genes LACR_1383 –LACR_2610 and LACR_A01 –LACR_E8 plotted against survival after 10 minutes heat and 30 minutes oxidative stress. Survival is expressed as the difference of log CFU/ml after stress and before stress. Numbers indicate fermentations as presented in Table 1. P-values above the plots indicate significance of correlation (assessed by a linear model). (ZIP) [file pone.0167944.s011.zip › S6_File/LACR_1584_real_dat.png]

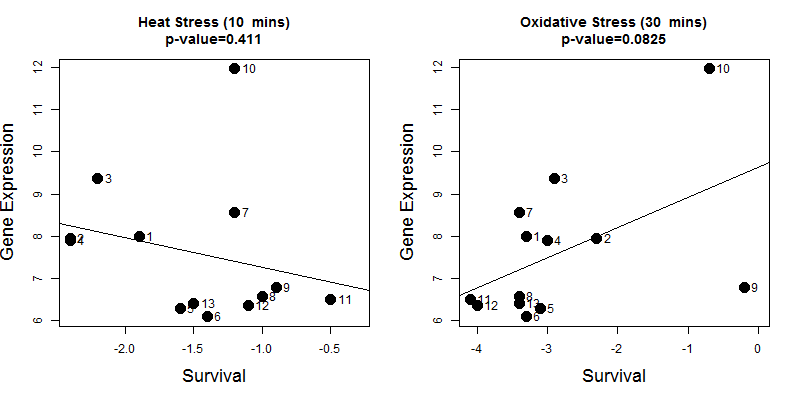

Supplement: S6 File — Expression levels of genes LACR_1383 –LACR_2610 and LACR_A01 –LACR_E8 plotted against survival after 10 minutes heat and 30 minutes oxidative stress. Survival is expressed as the difference of log CFU/ml after stress and before stress. Numbers indicate fermentations as presented in Table 1. P-values above the plots indicate significance of correlation (assessed by a linear model). (ZIP) [file pone.0167944.s011.zip › S6_File/LACR_1585_real_dat.png]

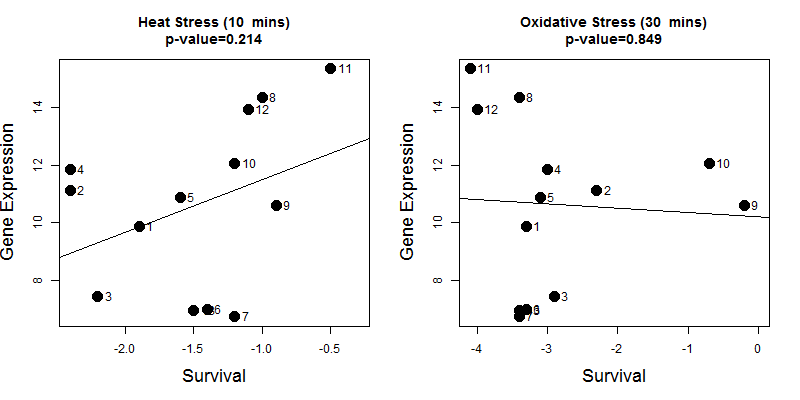

Supplement: S6 File — Expression levels of genes LACR_1383 –LACR_2610 and LACR_A01 –LACR_E8 plotted against survival after 10 minutes heat and 30 minutes oxidative stress. Survival is expressed as the difference of log CFU/ml after stress and before stress. Numbers indicate fermentations as presented in Table 1. P-values above the plots indicate significance of correlation (assessed by a linear model). (ZIP) [file pone.0167944.s011.zip › S6_File/LACR_1586_real_dat.png]

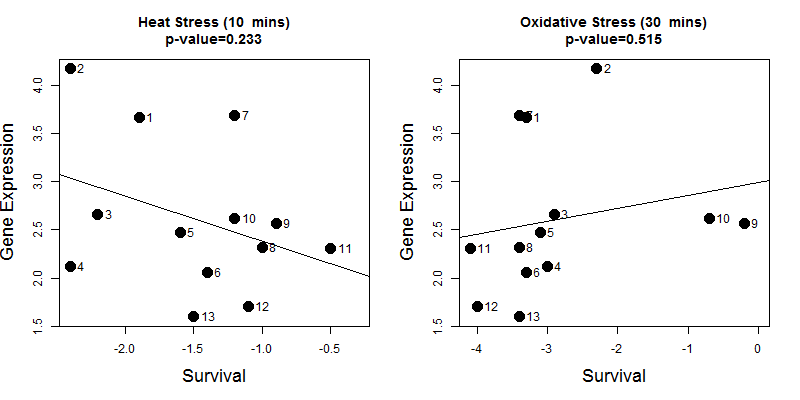

Supplement: S6 File — Expression levels of genes LACR_1383 –LACR_2610 and LACR_A01 –LACR_E8 plotted against survival after 10 minutes heat and 30 minutes oxidative stress. Survival is expressed as the difference of log CFU/ml after stress and before stress. Numbers indicate fermentations as presented in Table 1. P-values above the plots indicate significance of correlation (assessed by a linear model). (ZIP) [file pone.0167944.s011.zip › S6_File/LACR_1587_real_dat.png]

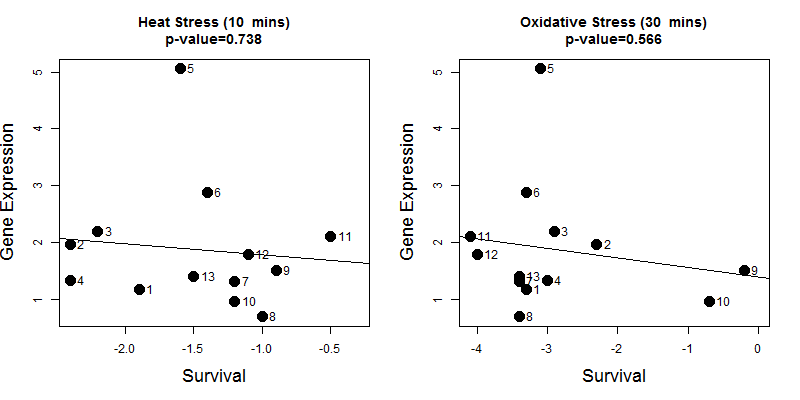

Supplement: S6 File — Expression levels of genes LACR_1383 –LACR_2610 and LACR_A01 –LACR_E8 plotted against survival after 10 minutes heat and 30 minutes oxidative stress. Survival is expressed as the difference of log CFU/ml after stress and before stress. Numbers indicate fermentations as presented in Table 1. P-values above the plots indicate significance of correlation (assessed by a linear model). (ZIP) [file pone.0167944.s011.zip › S6_File/LACR_1588_real_dat.png]

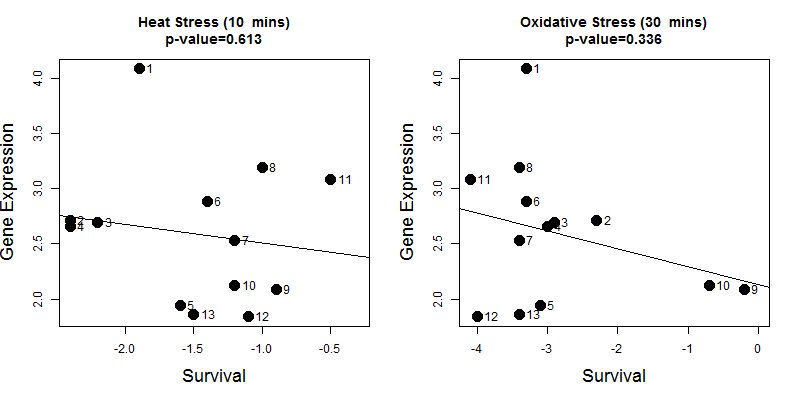

Supplement: S6 File — Expression levels of genes LACR_1383 –LACR_2610 and LACR_A01 –LACR_E8 plotted against survival after 10 minutes heat and 30 minutes oxidative stress. Survival is expressed as the difference of log CFU/ml after stress and before stress. Numbers indicate fermentations as presented in Table 1. P-values above the plots indicate significance of correlation (assessed by a linear model). (ZIP) [file pone.0167944.s011.zip › S6_File/LACR_1589_real_dat.png]

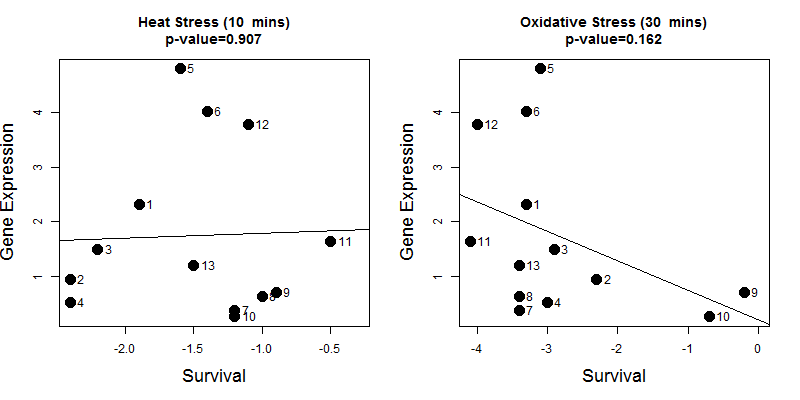

Supplement: S6 File — Expression levels of genes LACR_1383 –LACR_2610 and LACR_A01 –LACR_E8 plotted against survival after 10 minutes heat and 30 minutes oxidative stress. Survival is expressed as the difference of log CFU/ml after stress and before stress. Numbers indicate fermentations as presented in Table 1. P-values above the plots indicate significance of correlation (assessed by a linear model). (ZIP) [file pone.0167944.s011.zip › S6_File/LACR_1590_real_dat.png]

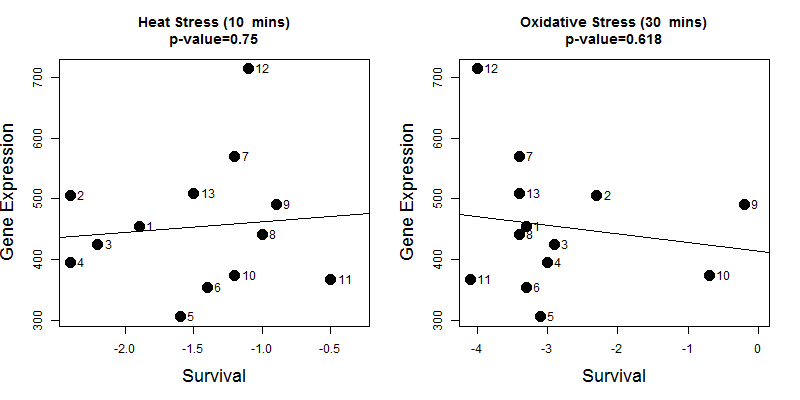

Supplement: S6 File — Expression levels of genes LACR_1383 –LACR_2610 and LACR_A01 –LACR_E8 plotted against survival after 10 minutes heat and 30 minutes oxidative stress. Survival is expressed as the difference of log CFU/ml after stress and before stress. Numbers indicate fermentations as presented in Table 1. P-values above the plots indicate significance of correlation (assessed by a linear model). (ZIP) [file pone.0167944.s011.zip › S6_File/LACR_1591_real_dat.png]

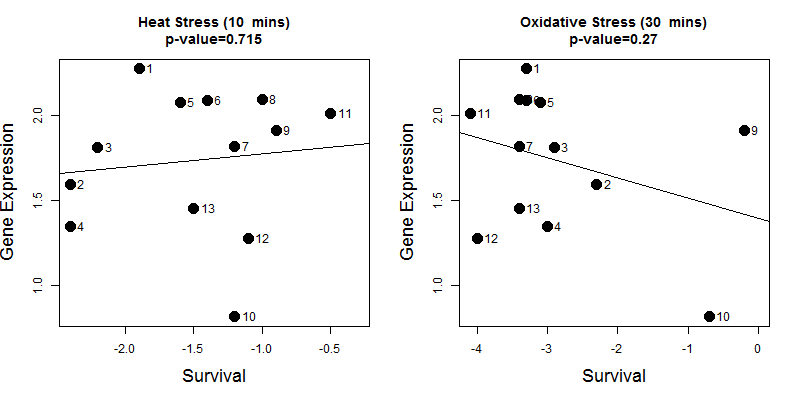

Supplement: S6 File — Expression levels of genes LACR_1383 –LACR_2610 and LACR_A01 –LACR_E8 plotted against survival after 10 minutes heat and 30 minutes oxidative stress. Survival is expressed as the difference of log CFU/ml after stress and before stress. Numbers indicate fermentations as presented in Table 1. P-values above the plots indicate significance of correlation (assessed by a linear model). (ZIP) [file pone.0167944.s011.zip › S6_File/LACR_1593_real_dat.png]

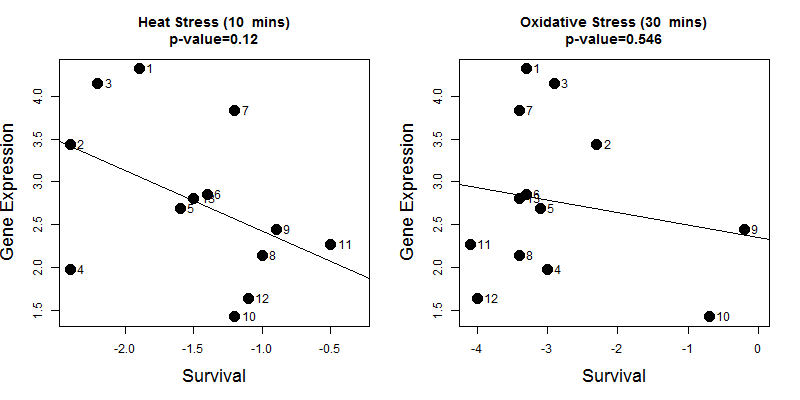

Supplement: S6 File — Expression levels of genes LACR_1383 –LACR_2610 and LACR_A01 –LACR_E8 plotted against survival after 10 minutes heat and 30 minutes oxidative stress. Survival is expressed as the difference of log CFU/ml after stress and before stress. Numbers indicate fermentations as presented in Table 1. P-values above the plots indicate significance of correlation (assessed by a linear model). (ZIP) [file pone.0167944.s011.zip › S6_File/LACR_1595_real_dat.png]

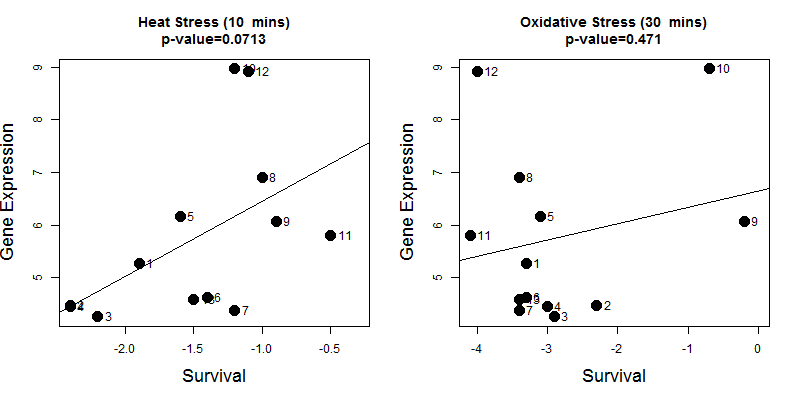

Supplement: S6 File — Expression levels of genes LACR_1383 –LACR_2610 and LACR_A01 –LACR_E8 plotted against survival after 10 minutes heat and 30 minutes oxidative stress. Survival is expressed as the difference of log CFU/ml after stress and before stress. Numbers indicate fermentations as presented in Table 1. P-values above the plots indicate significance of correlation (assessed by a linear model). (ZIP) [file pone.0167944.s011.zip › S6_File/LACR_1596_real_dat.png]

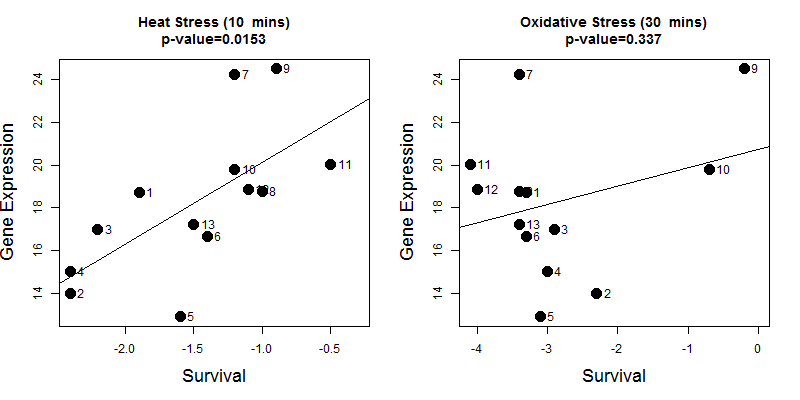

Supplement: S6 File — Expression levels of genes LACR_1383 –LACR_2610 and LACR_A01 –LACR_E8 plotted against survival after 10 minutes heat and 30 minutes oxidative stress. Survival is expressed as the difference of log CFU/ml after stress and before stress. Numbers indicate fermentations as presented in Table 1. P-values above the plots indicate significance of correlation (assessed by a linear model). (ZIP) [file pone.0167944.s011.zip › S6_File/LACR_1597_real_dat.png]

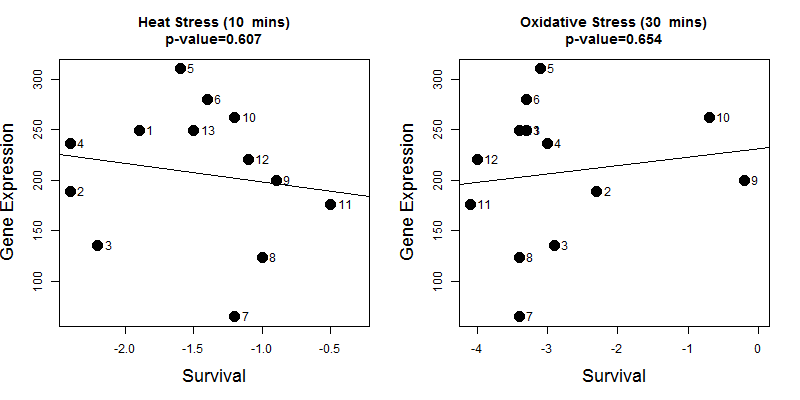

Supplement: S6 File — Expression levels of genes LACR_1383 –LACR_2610 and LACR_A01 –LACR_E8 plotted against survival after 10 minutes heat and 30 minutes oxidative stress. Survival is expressed as the difference of log CFU/ml after stress and before stress. Numbers indicate fermentations as presented in Table 1. P-values above the plots indicate significance of correlation (assessed by a linear model). (ZIP) [file pone.0167944.s011.zip › S6_File/LACR_1598_real_dat.png]

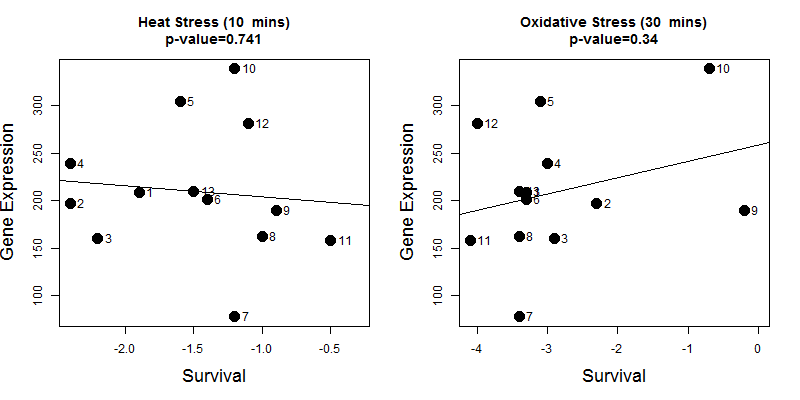

Supplement: S6 File — Expression levels of genes LACR_1383 –LACR_2610 and LACR_A01 –LACR_E8 plotted against survival after 10 minutes heat and 30 minutes oxidative stress. Survival is expressed as the difference of log CFU/ml after stress and before stress. Numbers indicate fermentations as presented in Table 1. P-values above the plots indicate significance of correlation (assessed by a linear model). (ZIP) [file pone.0167944.s011.zip › S6_File/LACR_1599_real_dat.png]

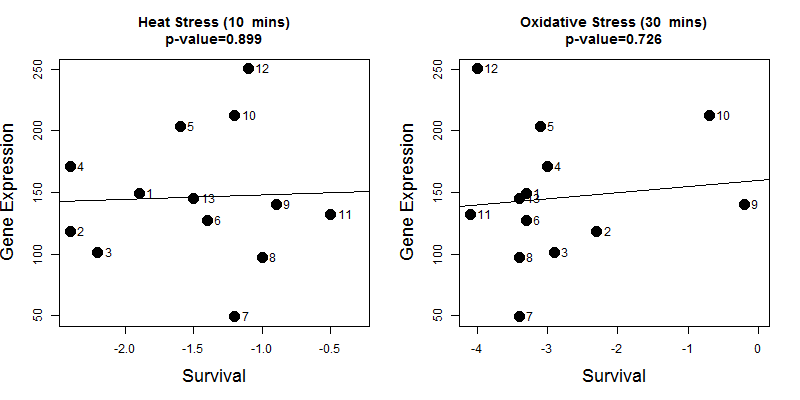

Supplement: S6 File — Expression levels of genes LACR_1383 –LACR_2610 and LACR_A01 –LACR_E8 plotted against survival after 10 minutes heat and 30 minutes oxidative stress. Survival is expressed as the difference of log CFU/ml after stress and before stress. Numbers indicate fermentations as presented in Table 1. P-values above the plots indicate significance of correlation (assessed by a linear model). (ZIP) [file pone.0167944.s011.zip › S6_File/LACR_1600_real_dat.png]

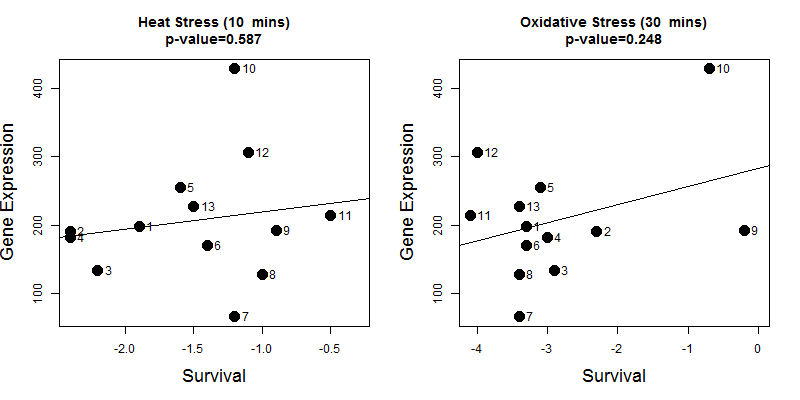

Supplement: S6 File — Expression levels of genes LACR_1383 –LACR_2610 and LACR_A01 –LACR_E8 plotted against survival after 10 minutes heat and 30 minutes oxidative stress. Survival is expressed as the difference of log CFU/ml after stress and before stress. Numbers indicate fermentations as presented in Table 1. P-values above the plots indicate significance of correlation (assessed by a linear model). (ZIP) [file pone.0167944.s011.zip › S6_File/LACR_1601_real_dat.png]

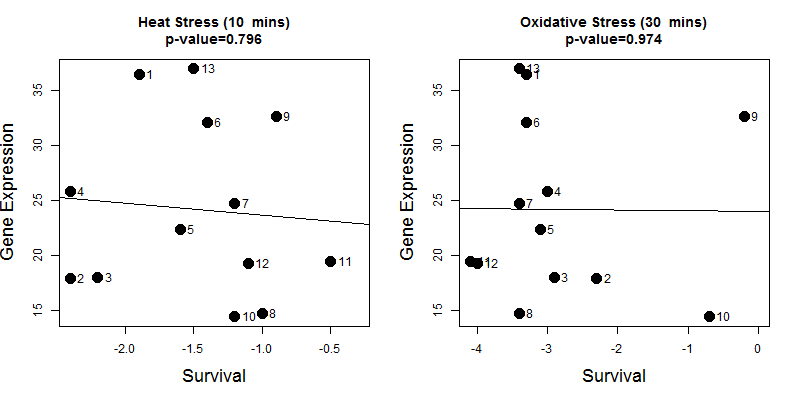

Supplement: S6 File — Expression levels of genes LACR_1383 –LACR_2610 and LACR_A01 –LACR_E8 plotted against survival after 10 minutes heat and 30 minutes oxidative stress. Survival is expressed as the difference of log CFU/ml after stress and before stress. Numbers indicate fermentations as presented in Table 1. P-values above the plots indicate significance of correlation (assessed by a linear model). (ZIP) [file pone.0167944.s011.zip › S6_File/LACR_1602_real_dat.png]

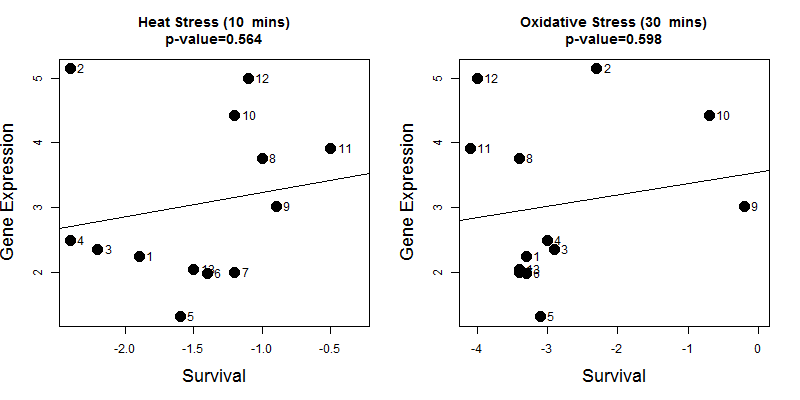

Supplement: S6 File — Expression levels of genes LACR_1383 –LACR_2610 and LACR_A01 –LACR_E8 plotted against survival after 10 minutes heat and 30 minutes oxidative stress. Survival is expressed as the difference of log CFU/ml after stress and before stress. Numbers indicate fermentations as presented in Table 1. P-values above the plots indicate significance of correlation (assessed by a linear model). (ZIP) [file pone.0167944.s011.zip › S6_File/LACR_1603_real_dat.png]
